# A Phase I, randomized, double-blind, placebo-controlled, dose escalation study to evaluate the safety, tolerability, and pharmacokinetics of intravenous ertapenem and zidebactam in combination (WCK 6777) in healthy adult subjects

**DOI:** 10.1128/aac.00390-26

**Published:** 2026-09-29

**Authors:** T. P. Lodise, R. Chavan, A. Patel, R. Yeole, S. Bhagwat, M. Patel, G. A. Saviolakis, M. Kankam, K. Gu, V. Ghazaryan, A. Jaunarajs, S. R. Fairfax, H. Howell, R. A. Bonomo

**Affiliations:** 1 Albany College of Pharmacy and Health Sciences 1091, Albany, New York, USA; 2 Wockhardt Research Centre 150815 , Chhatrapati Sambhaji Nagar, Maharashtra, India; 3 DynPort Vaccine Company 839133 , Frederick, Maryland, USA; 4 Altasciences Clinical Kansas, Inc., Overland Park, Kansas, USA; 5 Division of Microbiology and Infectious Diseases (DMID), National Institute of Allergy and Infectious Diseases (NIAID), National Institutes of Health (NIH) 2511, Bethesda, Maryland, USA; 6 The Emmes Company, LLC 6947 , Rockville, Maryland, USA; 7 Infectious Diseases Section, Medical and Research Service, Louis Stokes Cleveland Department of Veterans Affairs Medical Center, Geriatric Research Education and Clinical Center (GRECC), VA Northeast Ohio Healthcare System 465630, Cleveland, Ohio, USA; 8 Department of Medicine, Case Western Reserve University 2546, Cleveland, Ohio, USA; 9 Department of Molecular Biology and Microbiology, Case Western Reserve University 2546, Cleveland, Ohio, USA; 10 Department of Biochemistry, Case Western Reserve University 2546, Cleveland, Ohio, USA; 11 Department of Pharmacology, Case Western Reserve University 2546, Cleveland, Ohio, USA; 12 Department of Proteomics and Bioinformatics, Case Western Reserve University 2546, Cleveland, Ohio, USA; 13 Case VA CARES, Louis Stokes Cleveland Department of Veterans Affairs Medical Center, Cleveland, Ohio, USA; Providence Portland Medical Center, Portland, Oregon, USA

**Keywords:** safety, Phase 1, zidebactam, ertapenem, pharmacokinetics

## Abstract

WCK 6777 is a novel once-daily β-lactam enhancer combination comprising ertapenem (ERT) and zidebactam (ZID), designed to expand ERT activity against multidrug-resistant gram-negative Enterobacterales, including serine- and metallo-β-lactamase producers. This first-in-human, randomized, double-blind, placebo-controlled Phase 1 study evaluated the safety, tolerability, and pharmacokinetics (PK) of WCK 6777 and its individual components following once-daily intravenous administration for 7 days in healthy adults. In total, 54 participants were enrolled, of whom 52 received ERT alone (2 or 3 g), ZID alone (2 or 3 g), WCK 6777 (ERT + ZID at 1 + 1, 2 + 2, or 3 + 3 g), or placebo. Safety assessments included adverse events, clinical laboratory testing, vital signs, and electrocardiography. Plasma and urine PK of total and unbound ERT and ZID were characterized using noncompartmental methods. WCK 6777 was safe and well tolerated at all dose levels, with no deaths, serious adverse events, or treatment discontinuations. Adverse events were predominantly mild or moderate and were most commonly infusion-site reactions. Both ERT and ZID exhibited predictable, time-independent PK. Ertapenem exhibited a biphasic disposition with a terminal half-life supportive of once-daily dosing, whereas ZID displayed linear PK across the evaluated dose range. Plasma exposure, clearance, protein binding, and renal excretion were comparable between monotherapy and combination regimens, indicating no clinically meaningful PK interaction. Both drugs were eliminated predominantly unchanged in urine. In summary, WCK 6777 demonstrated a favorable safety profile and predictable PK following once-daily administration, supporting further clinical development, including evaluation in Phase II/III efficacy studies and potential application in outpatient parenteral antimicrobial therapy.

**CLINICAL TRIALS:**

This study is registered with ClinicalTrials.gov as NCT05645757.

## INTRODUCTION

Antimicrobial resistance (AMR) continues to erode the effectiveness of existing antibiotics and remains a major global health threat (1). Among gram-negative pathogens, carbapenem-resistant Enterobacterales (CRE) are of particular concern due to their ability to hydrolyze nearly all β-lactams and disseminate across both healthcare and community settings (2, 3). Carbapenem resistance is most commonly mediated by Ambler class A, B, and D β-lactamases, including *Klebsiella pneumoniae* carbapenemase, metallo-β-lactamases (MBLs) like NDM, VIM, IMP, and OXA-48-like enzymes, often accompanied by porin loss and efflux upregulation (4–7). Once considered predominantly hospital acquired, CRE are increasingly identified in community-onset Enterobacterales infections, with reported prevalence ranging from 8% to 30% globally and 5% to 11% in the United States (8).

Several intravenous (IV) agents active against CRE have been approved by the U.S. Food and Drug Administration and the European Medicines Agency, including β-lactam/β-lactamase inhibitor (BL/BLI) combinations such as ceftazidime–avibactam, meropenem–vaborbactam, and imipenem–relebactam (9–12). While these agents effectively inhibit serine carbapenemases, they lack activity against MBL-producing organisms and require every 8-h infusion schedules, limiting feasibility for outpatient parenteral antimicrobial therapy (OPAT) (9). Agents with activity against MBL-producing Enterobacterales, including aztreonam–avibactam and cefiderocol, are also available; however, these agents likewise require multiple daily doses, constraining their suitability for once-daily outpatient regimens (13–15). As community-onset CRE infections increase (8), the absence of broadly active, once-daily IV regimens constrains both early discharge and hospitalization avoidance strategies (16–18). Together, these considerations highlight the need for a broad-spectrum, once-daily IV regimen well suited for OPAT delivery.

Ertapenem–zidebactam (ERT–ZID; WCK 6777) is a novel β-lactam enhancer combination developed to address these limitations (19, 20). Ertapenem is a long-acting Group 1 carbapenem with favorable once-daily pharmacokinetics (PK) but is readily hydrolyzed by carbapenemases (21, 22). Zidebactam is a bicyclo-acyl hydrazide β-lactam enhancer that restores and extends β-lactam activity through high-affinity binding to penicillin-binding protein 2 (PBP2), conferring intrinsic antibacterial activity and potent inhibition of class A and C β-lactamases (23). This β-lactam enhancer mechanism is optimized when ZID is combined with β-lactams such as ERT that also bind other PBPs apart from PBP2, resulting in enhanced microbiologic activity against resistant gram-negative pathogens, including MBL producers, despite the absence of direct MBL inhibition (23). In addition, ZID markedly reduces the free-drug time above the MIC (%fT > MIC), required by the partner β-lactam, improving robust bactericidal activity (24–27). The two agents exhibit complementary PK: ERT’s prolonged half-life sustains target β-lactam exposure, while ZID’s rapid distribution and renal elimination maintain concentrations above its PBP2 engagement threshold (28), enabling once-daily dosing.

**Fig 1:**
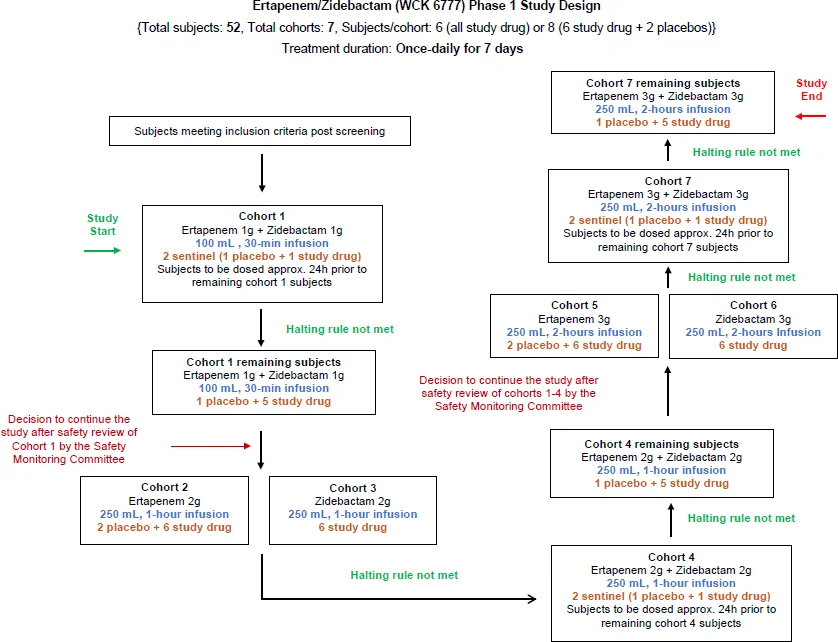
Schematic of study design.

This first-in-human, randomized, double-blind, placebo-controlled Phase I study (NCT05645757) was designed to primarily evaluate the safety and tolerability of ERT and ZID administered alone and in combination as WCK 6777 following once-daily intravenous dosing for 7 days in healthy adults. Three dose-escalating regimens of WCK 6777 (ERT + ZID) and two dose-escalating regimens of standalone ERT or ZID were evaluated, with total daily doses up to 6 g (ERT 3 g + ZID 3 g), to define safety margins across the intended clinical dose range (Table 1; Fig. 1). Secondary objectives were to characterize the PK profiles of ERT and ZID in plasma (total and unbound) and urine following repeated once-daily dosing, including assessment of systemic exposure, dose-dependent PK, renal excretion, plasma protein binding, and the potential for PK interactions between the two components. These data were intended to inform dose selection and regimen optimization for subsequent clinical development of WCK 6777.

**TABLE 1:** Study design^*a*^

Cohort no.	Study drug-dose	Infusion time—frequency	Participants
Cohort 1	WCK 6777 2 g (ERT 1 g + ZID 1 g) or placebo	30 (±5) min—once daily for 7 days	8 (6 drug combination, 2 placebo)
Cohort 2	ERT 2 g or placebo	60 (±10) min—once daily for 7 days	8 (6 drug, 2 placebo)
Cohort 3	ZID 2 g	60 (±10) min—once daily for 7 days	6 (all drug)
Cohort 4	WCK 6777 4 g (ERT 2 g + ZID 2 g) or placebo	60 (±10) min—once daily for 7 days	8 (6 drug combination, 2 placebo)
Cohort 5	ERT 3 g or placebo	120 (±10) min—once daily for 7 days	8 (6 drug, 2 placebo)
Cohort 6	ZID 3 g	120 (±10) min—once daily for 7 days	6 (all drug)
Cohort 7	WCK 6777 6 g (ERT 3 g + ZID 3 g) or placebo	120 (±10) min—once daily for 7 days	8 (6 drug combination, 2 placebo)

^*a*^ ERT, ertapenem; ZID, zidebactam. WCK 6777 consists of ertapenem co-administered with zidebactam.

## RESULTS

### Study population

Fifty-four healthy volunteers were enrolled between March and June 2023, of whom 52 received at least one dose of study product and were included in the safety population; 42 participants receiving study drug completed the study and comprised the PK analysis set (10 subjects who received placebo were not included in the PK analyses). Two participants withdrew prior to the first dose due to personal reasons. One participant in the WCK 6777 4 g cohort completed all doses and study visits but had a moderate, treatment-related QTcF prolongation that remained unresolved at the final visit; this participant was subsequently lost to follow-up for further assessment of the event (see Safety and tolerability). Baseline demographics were comparable across treatment groups (Table 2). The mean age was 33 years (SD, 7.6), mean body weight was 79.1 kg (SD, 9.0), and 63% were male. All participants were non-smokers and met eligibility criteria for normal hepatic and renal function. There were no clinically meaningful differences in age, sex, race, or BMI between cohorts. Overall, 27 participants (52%) reported pre-existing medical conditions (Table S1), which were minor, clinically insignificant, and consistent with protocol-defined eligibility for healthy adult volunteers. Overall, six participants (12%) reported the use of concomitant medications: three participants (6%) used analgesics, two participants (4%) used sex hormones and modulators of the genital system, and two participants (4%) used other gynecologicals. All concomitant medications were permitted per protocol, and none were known to affect renal transporters or interact with ERT or ZID.

**TABLE 2:** Baseline demographic and anthropometric characteristics of the study population^*a*^^,^^*b*^

Characteristic	WCK 6777 2 g (*N* = 6)	ERT 2 g (*N* = 6)	ZID 2 g (*N* = 6)	WCK 6777 4 g (*N* = 6)	ERT 3 g (*N* = 7)	ZID 3 g (*N* = 7)	WCK 6777 6 g (*N* = 6)	Placebo (*N* = 10)	All participants (*N* = 54)
Sex, *n* (%)									
Male	4 (67)	3 (50)	4 (67)	3 (50)	5 (71)	5 (71)	4 (67)	6 (60)	34 (63)
Female	2 (33)	3 (50)	2 (33)	3 (50)	2 (29)	2 (29)	2 (33)	4 (40)	20 (37)
Ethnicity, *n* (%)									
Not Hispanic or Latino	4 (67)	5 (83)	6 (100)	5 (83)	5 (71)	6 (86)	6 (100)	9 (90)	46 (85)
Hispanic or Latino	2 (33)	1 (17)	0	1 (17)	2 (29)	1 (14)	0	1 (10)	8 (15)
Race, *n* (%)									
Black or African American	5 (83)	4 (67)	2 (33)	1 (17)	2 (29)	2 (29)	5 (83)	7 (70)	28 (52)
White	0	2 (33)	4 (67)	5 (83)	4 (57)	5 (71)	1 (17)	2 (20)	23 (43)
Other/multiracial/unknown	1 (17)	0	0	0	1 (14)	0	0	1 (10)	3 (6)
Age (years)									
Mean ± SD	33.7 ± 7.3	35.2 ± 9.2	32.3 ± 9.5	33.2 ± 7.0	33.6 ± 7.9	37.3 ± 5.6	29.2 ± 9.0	30.7 ± 6.7	33.0 ± 7.6
Height (cm)									
Mean ± SD	177.0 ± 14.0	168.3 ± 9.0	176.5 ± 10.1	169.8 ± 9.0	175.3 ± 3.6	175.9 ± 7.3	177.5 ± 7.8	170.7 ± 12.2	173.7 ± 9.7
Weight (kg)									
Mean ± SD	84.5 ± 9.8	81.0 ± 8.8	77.7 ± 3.4	79.9 ± 12.1	73.3 ± 5.4	84.5 ± 10.9	75.1 ± 8.7	77.9 ± 8.4	79.1 ± 9.0
BMI (kg/m^2^)									
Mean ± SD	27.1 ± 2.9	28.6 ± 2.1	25.2 ± 3.4	27.6 ± 2.9	23.8 ± 1.6	27.3 ± 2.8	24.1 ± 4.4	26.9 ± 2.8	26.3 ± 3.2

^*a*^ BMI, body mass index; ERT, ertapenem; ZID, zidebactam; and WCK 6777, ertapenem/zidebactam.

^*b*^ Percentages are based on the number of participants randomized per cohort.

### Safety and tolerability

**Fig 2:**
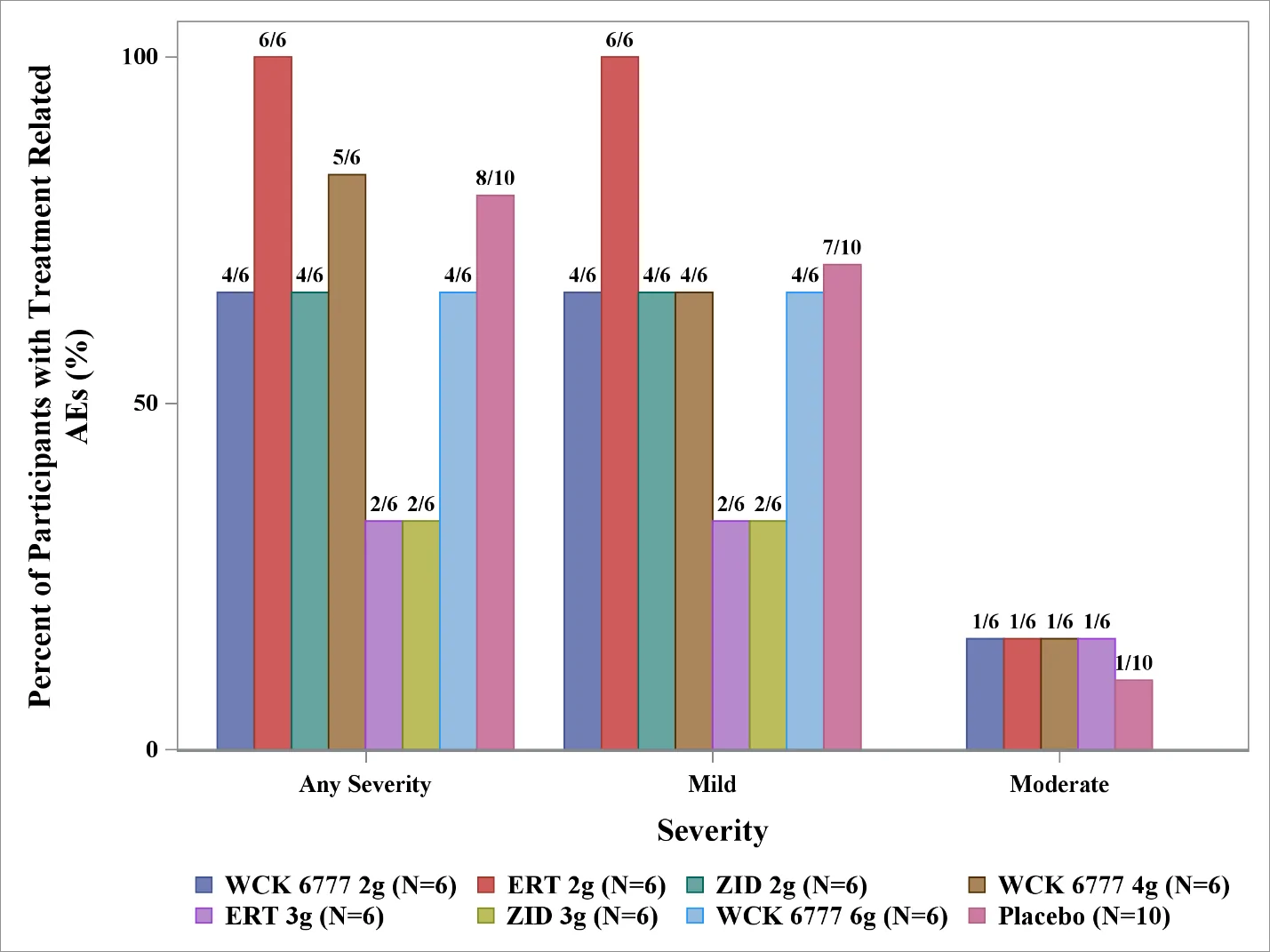
Overall summary of treatment-related adverse events. Data are presented as the percentage of participants within each treatment group experiencing at least one treatment-related adverse event, with counts shown above bars as *n*/*N* (number of participants with event/number of participants in the safety population for that cohort). Treatment-related AEs were defined as adverse events assessed by the investigator as related to study treatment. Severity categories were assigned by the investigator according to the protocol-defined grading criteria (mild, moderate, and severe). No severe treatment-related AEs were reported. Each participant was counted once within the highest severity category experienced for treatment-related AEs. The safety population included all participants who received at least one dose of study drug or placebo. WCK 6777 consists of ertapenem co-administered with zidebactam.

All 52 participants who received at least one dose of study drug or placebo were included in the safety analysis. No deaths, serious adverse events (SAEs), or study discontinuations occurred (Table 3). Overall, 41 participants (79%) experienced at least one adverse event (AE), and 35 participants (67%) experienced at least one treatment-related AE (Fig. 2). Of the 35 participants who reported a treatment-related adverse event, 30 (58%) experienced events whose maximum severity was mild. Of 52 participants in the safety population, 5 participants (10%) experienced at least one treatment-related AE of moderate severity, and none of the treatment-related AEs were of severe severity.

**TABLE 3:** Summary of adverse events (safety population)^*a*^^,^^*b*^

Participants with ≥1 event	WCK 6777 2 g (*N* = 6)	ERT 2 g (*N* = 6)	ZID 2 g (*N* = 6)	WCK 6777 4 g (*N* = 6)	ERT 3 g (*N* = 6)	ZID 3 g (*N* = 6)	WCK 6777 6 g (*N* = 6)	Placebo (*N* = 10)	All participants (*N* = 52)
At least one adverse event	6 (100)	6 (100)	5 (83)	5 (83)	4 (67)	2 (33)	4 (67)	9 (90)	41 (79)
At least one related adverse event	4 (67)	6 (100)	4 (67)	5 (83)	2 (33)	2 (33)	4 (67)	8 (80)	35 (67)
Mild (Grade 1)	3 (50)	5 (83)	4 (67)	4 (67)	1 (17)	2 (33)	4 (67)	7 (70)	30 (58)
Moderate (Grade 2)	1 (17)	1 (17)	0	1 (17)	1 (17)	0	0	1 (10)	5 (10)
Severe (Grade 3)	0	0	0	0	0	0	0	0	0
At least one severe (Grade 3) unsolicited adverse event	0	0	0	0	0	0	0	0	0
Related	0	0	0	0	0	0	0	0	0
Unrelated	0	0	0	0	0	0	0	0	0
At least one serious adverse event	0	0	0	0	0	0	0	0	0
At least one related serious adverse event	0	0	0	0	0	0	0	0	0
Adverse event leading to early termination^*c*^	0	0	0	0	0	0	0	0	0

^*a*^ ERT, ertapenem; ZID, zidebactam; and WCK 6777, ertapenem/zidebactam.

^*b*^ Values are *n *(%). *N*, number of participants in the safety population.

^*c*^ As reported on the Adverse Event electronic case report form. Participants are counted once per category regardless of the number of events.

Adverse events occurring in ≥5% of participants by MedDRA system organ class and preferred term are summarized in Table S2. The most frequently reported non-serious AEs were administration-site reactions, most commonly infusion-site erythema (14 events in 11/52 participants, 21%) and infusion-site extravasation (10 events in 9/52 participants, 17%). Infusion-site swelling was also reported in five participants (10%). These events were transient, predominantly mild, and did not result in treatment discontinuation. The most common systemic AEs were headache (12%), diarrhea (8%), and nausea (8%).

Moderate adverse events were uncommon and occurred in both active-treatment and placebo groups (Table S3). Seven moderate adverse events were reported in six participants. Six events were considered treatment related: diarrhea and infusion-site swelling in one participant in the ERT 2 g cohort; decreased heart rate in one participant in the WCK 6777 2 g cohort; QTcF prolongation in one participant each in the ERT 3 g cohort (Day 8; QTcF 418 ms, +33 ms from baseline) and the WCK 6777 4 g cohort (Day 11; QTcF 425 ms, +47 ms from baseline); and headache in one placebo recipient on Day 2. Both QTcF prolongation events met the protocol-defined Grade 2 threshold based on an increase of 30–59 ms from baseline rather than absolute QTcF prolongation; absolute QTcF values remained below 480 ms. One moderate event, vessel puncture site pain in one participant in the ERT 3 g cohort on Day 4, was considered unrelated to study treatment.

All moderate treatment-emergent adverse events were asymptomatic or minimally symptomatic, transient, and resolved by study completion, with one exception: the treatment-related QTcF prolongation in a participant in the WCK 6777 4 g cohort remained unresolved at the final study visit because the participant was subsequently lost to follow-up. No concomitant medication was required for any moderate adverse event, except acetaminophen administered to the placebo participant with headache. No clinically significant changes in electrocardiogram morphology or intervals were observed. The two QTcF prolongation events were asymptomatic and did not require intervention. The event in the ERT 3 g cohort resolved during follow-up, whereas the event in the WCK 6777 4 g cohort remained unresolved at the participant’s final study visit.

Laboratory TEAEs were predominantly mild, generally assessed as related to study drug and not clinically significant. Abnormal laboratory values were typically asymptomatic, transient, and resolved without sequelae. No clinically meaningful shifts in laboratory parameters were observed overall, except for modest increases in alanine aminotransferase (ALT) and aspartate aminotransferase (AST) between Days 8 and 11 in the ERT 2 g, ERT 3 g, and WCK 6777 4 g groups but not in the WCK 6777 6 g group. Moderate chemistry abnormalities occurred in two participants, consisting of ALT elevations in one participant in the ERT 2 g group (Day 11; maximum value, 118 U/L) and one participant in the ERT 3 g group (Days 8 and 11; maximum value, 137 U/L). Severe laboratory abnormalities were reported in two participants in the WCK 6777 4 g group, including one ALT elevation (Day 11; maximum value, 167 U/L) and one decrease in serum calcium (Day 2; lowest observed value, 7 mg/dL). All moderate and severe chemistry abnormalities were assessed as related to treatment. Relative to the upper limit of normal for ALT (approximately 46 U/L in males), these peak values corresponded to approximately 2.6-fold (118 U/L), 3.0-fold (137 U/L), and 3.6-fold (167 U/L) the ULN, and none was accompanied by an increase in total bilirubin.

Hematologic and coagulation abnormalities were reported across treatment groups and were predominantly mild and not related to study treatment. Moderate hematologic abnormalities were uncommon and not attributed to study drug. In hematology, two participants experienced moderate decreases: one participant in the WCK 6777 2 g cohort had pre-existing mild neutropenia at screening and at the pre-dose baseline visit (absolute neutrophil count 1,473 and 1,421 cells/µL, respectively), consistent with benign ethnic neutropenia, which transiently declined to moderate severity (nadir of 862 cells/µL on Day 8) before returning toward baseline by Day 15 (1,224 cells/µL), and one participant in the ERT 2 g cohort had moderate decreases in hemoglobin initially noted during the study and persisting beyond the last scheduled visit on Day 11. This participant underwent unscheduled follow-up assessments, with moderate hemoglobin decreases observed on Days 60 and 67 and a subsequent severe decrease on Day 93, all attributed to increased menstrual flow and assessed as not related to study treatment. No moderate coagulation abnormalities were observed. Coagulation findings were all mild and sporadic, predominantly involving activated partial thromboplastin time (aPTT). Isolated mild abnormalities in international normalized ratio and prothrombin time were observed in a small number of participants: one WCK 6777 6 g participant had mild elevations at all time points, including screening and baseline, and other participants with mild prothrombin time or international normalized ratio abnormalities had mild findings present at screening and/or baseline before study product administration. No consistent treatment-emergent prolongation pattern was observed, and the only coagulation finding attributed to study treatment was a transiently low, not prolonged, aPTT on Day 6 in the WCK 6777 2 g group, without associated bleeding. No study treatment modifications were required, and no participants discontinued due to hematologic or coagulation abnormalities.

Urinalysis abnormalities were generally trace or mild. Two severe urinalysis abnormalities were reported: in the ERT 2 g group, one participant had severe occult blood (3+) on Day 11, assessed as not related to study treatment, and in the placebo group, one participant had severe leukocyte esterase (3+) on Day 8, assessed as related to study treatment. Several moderate urinalysis abnormalities were also observed. In the WCK 6777 2 g cohort, one participant experienced moderate leukocyturia and leukocyte esterase abnormalities assessed as related to treatment; these findings were asymptomatic and resolved without intervention, and an additional moderate occult blood finding was attributed to menses. In the ERT 2 g cohort, two participants had moderate urinalysis findings (leukocyturia or leukocyte esterase) assessed as related to treatment, while moderate or severe occult blood findings in two female participants were assessed as not related and attributed to menstruation. One participant in the ZID 2 g cohort had moderate bacteriuria assessed as related to treatment without clinical sequelae. Moderate urinalysis abnormalities were also observed in placebo recipients, including leukocyte esterase positivity and occult blood. Across all cohorts, urinalysis abnormalities were asymptomatic, self-limited, and showed no dose-related trend or evidence of clinically meaningful urinary or renal toxicity.

### Pharmacokinetics

#### Plasma pharmacokinetics of ERT

**Fig 3:**
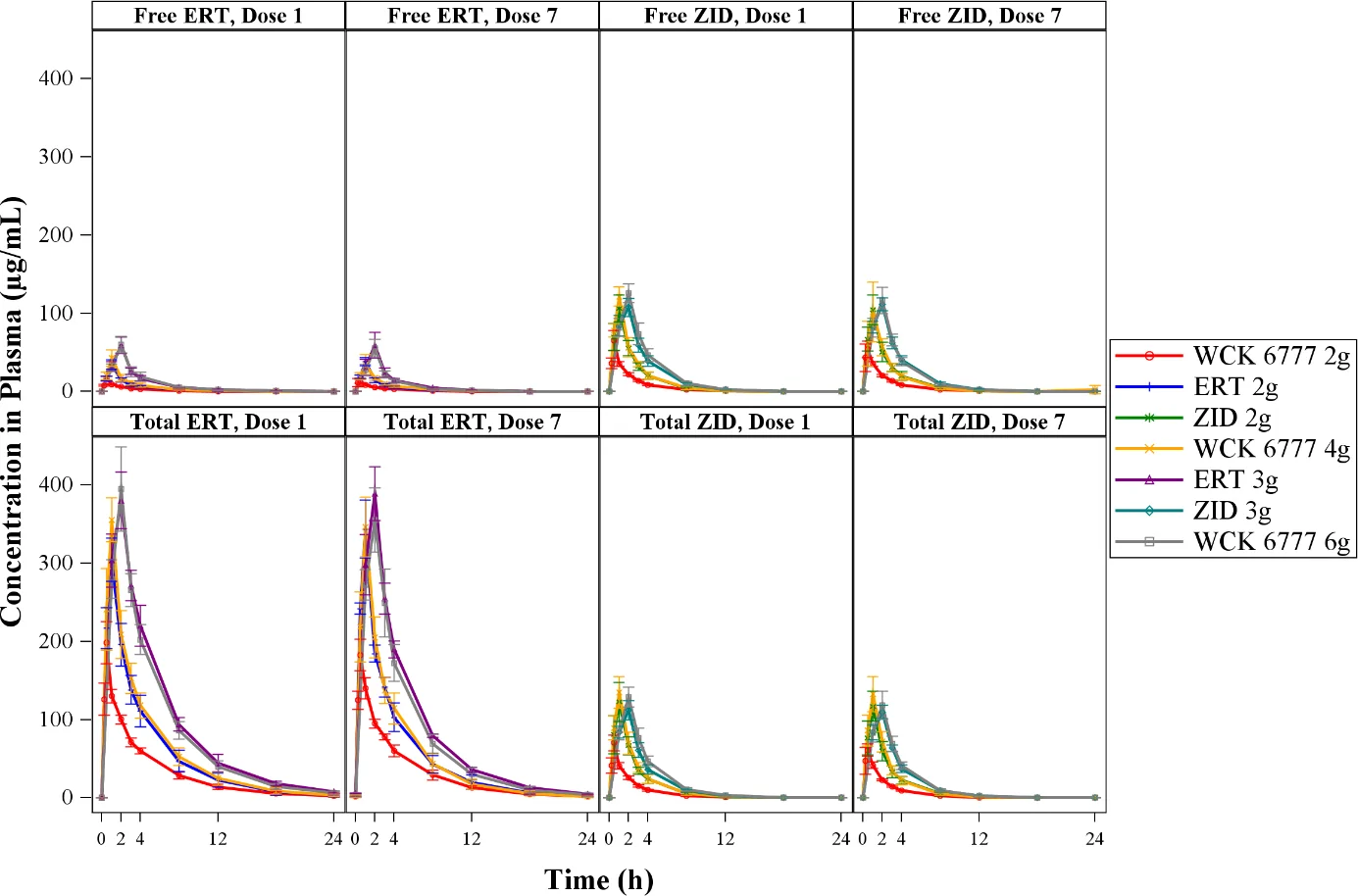
Mean total and free ertapenem and zidebactam plasma concentration–time profiles by treatment group, Doses 1 and 7. Data are presented as mean ± standard deviation plasma concentrations for each treatment group. Dose 1 represents concentrations following the first dose on Day 1; Dose 7 represents concentrations following the final dose on Day 7 at steady state. Free concentrations represent unbound plasma drug concentrations determined after protein binding separation; total concentrations represent measured plasma concentrations prior to protein binding adjustment. WCK 6777 consists of ertapenem co-administered with zidebactam. Treatment group sample sizes were WCK 6777 2 g (*n* = 6), ERT 2 g (*n* = 6), ZID 2 g (*n* = 6), WCK 6777 4 g (*n* = 6), ERT 3 g (*n* = 6), ZID 3 g (*n* = 6), and WCK 6777 6 g (*n* = 6). Error bars represent standard deviation.

Mean total and unbound plasma ERT concentration–time profiles following single and multiple once-daily intravenous doses of ERT alone (2 or 3 g) and WCK 6777 (ERT co-administered with ZID at 1 + 1, 2 + 2, and 3 + 3 g) are shown in Fig. 3. Across all treatment groups, ERT exhibited consistent biphasic disposition characterized by a rapid distribution phase followed by slower terminal elimination, with no qualitative differences between ERT administered alone and in combination with ZID. Median *T*_max_ occurred at the end of infusion and was determined by infusion duration (0.5 h for 30-min infusions, 1 h for 60-min infusions, and approximately 2 h for 120-min infusions), independent of ZID co-administration. Mean terminal elimination half-life (*t*_1/2_) after repeat dosing was approximately 3.6–4.1 h for total ERT (Table 4) and 3.4–3.9 h for unbound ERT (Table 5) across cohorts.

**TABLE 4:** Summary statistics for total ertapenem single- and multiple-dose PK parameters in plasma by treatment group^*d*^^,^^*e*^

PK parameter (units)	WCK 6777 2 g	ERT 2 g	WCK 6777 4 g	ERT 3 g	WCK 6777 6 g
Total ertapenem single-dose PK parameters					
*C*_max_ (µg/mL)	196.66 (14)	303.43 (9)	354.41 (8)	378.71 (10)	391.87 (13)
*C*_min_ (µg/mL)	2.15 (50)	2.61 (107)	3.10 (60)	7.32 (14)	5.91 (33)
*T*_max_ (h)	0.50 (0.50–0.50)	1.00 (1.00–1.07)	1.00 (1.00–1.00)	2.02 (2.00–2.03)	2.03 (2.02–2.03)
*T*_min_ (h)	23.60 (23.60–23.70)	23.65 (23.60–23.70)	23.60 (23.60–23.60)	23.60 (23.40–23.60)	23.60 (23.60–23.70)
AUC_(0*–t*)_ (µg h/mL)	723.48 (9)	1,241.47 (18)	1,377.99 (14)	2,148.23 (11)	2,024.46 (10)
AUC_(0–inf)_ (µg h/mL)	742.95 (10)	1,265.39 (19)	1,404.86 (15)	2,199.93 (11)	2,065.37 (11)
AUC_(0–24)_ (µg h/mL)	728.97 (9)	1,249.60 (18)	1,386.28 (14)	2,156.38 (11)	2,029.12 (10)
AUC_(0–last)_ (µg h/mL)	728.18 (9)	1,247.86 (18)	1,384.69 (14)	2,153.20 (11)	2,027.57 (10)
*t*_1/2_ (h)	4.34 (12)	3.76 (22)	3.92 (14)	4.20 (4)	4.20 (10)
CL_T_ (L/h)	1.34 (10)	1.58 (19)	1.42 (15)	1.37 (11)	1.45 (11)
ke (1/h)	0.16 (12)	0.18 (22)	0.18 (14)	0.17 (4)	0.17 (10)
*V*_*d*_ (L)	8.43 (7)	8.57 (9)	8.04 (12)	8.28 (10)	8.81 (13)
Total ertapenem multiple-dose PK parameters					
*C*_max,ss_ (µg/mL)	181.90 (11)	341.92 (10)	344.89 (10)	387.57 (9)	343.24 (13)
*C*_max,ss_/dose ([µg/mL]/mg)	0.18 (11)	0.17 (10)	0.17 (10)	0.13 (9)	0.11 (13)
*C*_min,ss_ (µg/mL)	1.75 (30)	1.81 (114)	1.74 (75)	4.93 (2)	3.49 (65)
*C*_avg,ss_ (µg/mL)	29.99 (9)	51.20 (13)	51.40 (16)	80.91 (6)	71.98 (14)
*C*_τ,ss_ (µg/mL)	1.75 (30)	1.85 (111)	1.74 (75)	4.93 (2)	3.50 (64)
*T*_max,ss_ (h)	0.52 (0.50–0.60)	1.00 (1.00–1.00)	1.02 (1.00–1.02)	2.02 (2.02–2.02)	2.07 (2.02–2.23)
*T*_min_ (h)	24.00 (24.00–24.00)	24.05 (24.00–24.30)	24.00 (24.00–24.00)	24.00 (24.00–24.00)	24.00 (24.00–24.10)
AUC_(0–24),ss_ (µg h/mL)	719.86 (9)	1,228.88 (14)	1,233.66 (16)	1,940.84 (6)	1,726.43 (14)
AUC_(0–τ),ss_ (µg h/mL)	719.86 (9)	1,228.88 (14)	1,233.66 (16)	1,940.84 (6)	1,726.43 (14)
AUC_(0–τ),ss_/dose ([µg h/mL]/mg)	0.72 (9)	0.61 (13)	0.62 (16)	0.65 (6)	0.58 (14)
*t*_1/2_ (h)	3.91 (8)	3.64 (19)	3.67 (21)	4.14 (4)	3.86 (18)
CL_T_ (L/h)	1.39 (9)	1.63 (13)	1.62 (16)	1.55 (7)	1.74 (14)
*V*_*d*,ss_ (L)	6.86 (5)	6.73 (11)	6.51 (11)	7.17 (9)	7.58 (10)
Linearity index^*a*^	0.97 (4)	0.97 (7)	0.88 (11)	0.89 (6)	0.84 (9)
RAUC^*b*^	0.99 (5)	0.98 (7)	0.89 (11)	0.91 (6)	0.85 (9)
RC_max_^*c*^	0.92 (17)	1.13 (14)	0.97 (7)	1.01 (8)	0.88 (8)

^*a*^ Linearity index is defined as AUC_(0–τ),ss_ (Dose 7)/AUC_(0–inf)_ (Dose 1).

^*b*^ RAUC is defined as AUC_(0–τ)_ (Dose 7)/AUC_(0–24)_ (Dose 1).

^*c*^ RC_max_ is defined as *C*_max_ (Dose 7)/*C*_max_ (Dose 1).

^*d*^ Single-dose parameters are calculated over the 24-h period following Dose 1. Multi-dose parameters are calculated over the 24-h period following Dose 7. For this study, the dosing interval (τ) was 24 h; therefore, AUC_(0–24),ss_ and AUC_(0–*τ*),ss_ are numerically identical, and *C*_τ,ss_ represents the concentration at the end of the dosing interval (equivalent to *C*_min,ss_). Values of GM (CV%) are shown, except for *T*_max_ and *T*_min_, for which values of median (min-max) are shown.

^*e*^ AUC, area under the concentration-time curve; Cavg, average concentration; CLT, total clearance; Cmax, maximum concentration; Cmin, minimum concentration; CV, coefficient of variation; ERT, ertapenem; GM, geometric mean; ke, elimination rate constant; Tmax, time to maximum concentration; Tmin, time to minimum concentration; Vd, volume of distribution; WCK 6777, ertapenem/zidebactam; τ, dosing interval; ss, steady state.

**TABLE 5:** Summary statistics for free ertapenem single- and multiple-dose PK parameters in plasma by treatment group^*d*^^,^^*e*^

PK parameter (units)	WCK 6777 2 g	ERT 2 g	WCK 6777 4 g	ERT 3 g	WCK 6777 6 g
Free ertapenem single-dose plasma PK parameters					
*C*_max_ (µg/mL)	16.60 (17)	34.00 (17)	42.10 (22)	58.45 (17)	59.42 (19)
*C*_min_ (µg/mL)	0.10 (40)	0.16 (42)	0.14 (67)	0.36 (24)	0.28 (35)
*T*_max_ (h)	0.50 (0.50–0.50)	1.00 (1.00–1.07)	1.00 (1.00–1.00)	2.02 (2.02–2.03)	2.03 (2.02–2.03)
*T*_min_ (h)	23.60 (18.00–23.60)	23.60 (18.00–23.70)	23.60 (23.60–23.60)	23.60 (23.40–23.60)	23.60 (23.60–23.70)
AUC_(0–*t*)_ (µg h/mL)	39.92 (7)	83.95 (19)	102.81 (24)	187.42 (15)	190.43 (14)
AUC_(0–inf)_ (µg h/mL)	41.65 (7)	85.93 (19)	104.94 (24)	190.81 (15)	193.61 (14)
AUC_(0–24)_ (µg h/mL)	41.19 (7)	85.25 (19)	103.96 (23)	188.86 (15)	192.13 (14)
AUC_(0–last)_ (µg h/mL)	41.04 (7)	85.03 (19)	103.94 (23)	188.66 (15)	191.81 (14)
*t*_1/2_ (h)	4.09 (11)	3.83 (21)	3.86 (15)	3.96 (12)	3.87 (10)
CL_T_ (L/h)	23.99 (7)	23.28 (19)	19.06 (24)	15.70 (15)	15.50 (14)
ke (1/h)	0.17 (11)	0.18 (21)	0.18 (15)	0.18 (12)	0.18 (10)
*V*_*d*_ (L)	141.43 (11)	128.25 (12)	106.20 (23)	89.98 (20)	86.55 (16)
Free ertapenem multiple-dose plasma PK parameters					
*C*_max,ss_ (µg/mL)	15.41 (24)	38.18 (13)	36.09 (23)	57.65 (28)	50.36 (28)
*C*_max,ss_/dose ([µg/mL]/mg)	0.02 (24)	0.02 (13)	0.02 (23)	0.02 (28)	0.02 (28)
*C*_min,ss_ (µg/mL)	0.10 (28)	0.12 (35)	0.12 (60)	0.23 (31)	0.15 (59)
*C*_avg,ss_ (µg/mL)	1.64 (6)	3.51 (18)	3.77 (16)	7.01 (23)	6.29 (19)
*C*_τ,ss_ (µg/mL)	0.06 (53)	0.07 (128)	0.07 (88)	0.23 (31)	0.15 (58)
*T*_max,ss_ (h)	0.52 (0.25–0.60)	1.00 (1.00–1.00)	1.02 (0.50–1.02)	2.02 (2.02–2.02)	2.07 (2.02–2.23)
*T*_min_ (h)	24.00 (18.00–24.00)	24.00 (18.00–24.10)	24.00 (18.00–24.00)	24.00 (24.00–24.00)	24.00 (24.00–24.10)
AUC_(0–24),ss_ (µg h/mL)	39.48 (6)	84.22 (18)	90.51 (16)	168.05 (23)	150.93 (19)
AUC_(0–τ),ss_ (µg h/mL)	39.48 (6)	84.22 (18)	90.51 (16)	168.05 (23)	150.93 (19)
AUC_(0–τ),ss_/dose ([µg h/mL]/mg)	0.04 (6)	0.04 (18)	0.05 (16)	0.06 (23)	0.05 (19)
*t*_1/2_ (h)	3.89 (19)	3.45 (19)	3.38 (19)	3.77 (10)	3.76 (17)
CL_T_ (L/h)	25.35 (6)	23.75 (18)	22.09 (16)	17.83 (23)	19.86 (19)
*V*_*d*,ss_ (L)	105.98 (14)	72.51 (16)	69.65 (13)	56.84 (30)	61.59 (29)
Linearity index^*a*^	0.95 (4)	0.98 (4)	0.86 (18)	0.88 (9)	0.78 (17)
RAUC^*b*^	0.96 (4)	0.99 (3)	0.87 (18)	0.89 (9)	0.79 (17)
RC_max_^*c*^	0.93 (11)	1.12 (7)	0.86 (37)	0.99 (12)	0.85 (28)

^*a*^ Linearity index is defined as AUC_(0–τ),ss_ (Dose 7)/AUC_(0–inf)_ (Dose 1).

^*b*^ RAUC is defined as AUC_(0–τ)_ (Dose 7)/AUC_(0–24)_ (Dose 1).

^*c*^ RC_max_ is defined as *C*_max_ (Dose 7)/*C*_max_ (Dose 1).

^*d*^ Single-dose parameters are calculated over the 24-h period following Dose 1. Multi-dose parameters are calculated over the 24-h period following Dose 7. For this study, the dosing interval (*τ*) was 24 h; therefore, AUC_(0–24),ss_ and AUC_(0–*τ*),ss_ are numerically identical, and *C*_τ,ss_ represents the concentration at the end of the dosing interval (equivalent to *C*_min,ss_). Values of GM (CV%) are shown, except for *T*_max_ and *T*_min_ for which values of median (min-max) are shown.

^*e*^ AUC, area under the concentration-time curve; Cavg, average concentration; CLT, total clearance; Cmax, maximum concentration; Cmin, minimum concentration; CV, coefficient of variation; ERT, ertapenem; GM, geometric mean; ke, elimination rate constant; Tmax, time to maximum concentration; Tmin, time to minimum concentration; Vd, volume of distribution; WCK 6777, ertapenem/zidebactam; τ, dosing interval; ss, steady state.

After single-dose administration (Dose 1), mean total plasma *C*_max_ and AUC values increased in a dose-dependent manner with ERT dose and were comparable between ERT-alone and corresponding WCK 6777 cohorts (Table 4). Apparent total clearance (CL_T_), volume of distribution (*V*_*d*_), and elimination rate constants (ke) following the first dose were consistent across cohorts. Following multiple once-daily doses, mean (CV%) total plasma *C*_max,ss_ and AUC_0–*t*,ss_ values on Day 7 increased in a dose-dependent manner with ERT dose and were comparable between ERT-alone and WCK 6777 cohorts (Table 4). Apparent CL_T_ and *V*_*d*_ at steady state were similar across treatment groups, providing no evidence of altered ERT disposition with repeated administration of either ERT alone or in combination with ZID. Dose-normalized *C*_max,ss_ and AUC_(0–*τ*),ss_ metrics were generally similar across treatment groups, supporting approximately linear, dose-dependent PK over the evaluated dose range. Accumulation ratios (RAUC and RC_max_) were close to unity in all cohorts, indicating achievement of steady state by Day 2 and absence of drug accumulation, with no differences attributable to combination dosing. Linearity indices (AUC_0–*τ*,ss_/AUC_0–∞,single_) ranged from approximately 0.84 to 0.97, supporting time-independent PK. Interindividual variability in exposure was low (CV ≤20%) for both *C*_max_ and AUC. Mean concentration–time profiles on Days 1 and 7 closely overlaid, with no evidence of sequence- or time-dependent changes in ERT PK.

Unbound (free) ERT PK following single and multiple doses closely paralleled total-drug findings (Fig. 3; Table 5). After Dose 1, mean unbound *C*_max_ and AUC increased in a dose-dependent manner with dose and were comparable between ERT-alone and WCK 6777 cohorts, with similar unbound CL_T_ and *V*_*d*_ across groups. At steady state, unbound *C*_max,ss_ and AUC_₀–*τ*,ss_ values remained dose dependent and consistent between treatments. Unbound *t*_½_ values (approximately 3.4–4.1 h) were similar to total-drug *t*_1/2_, indicating protein binding that was not influenced by ZID.

**Fig 4:**
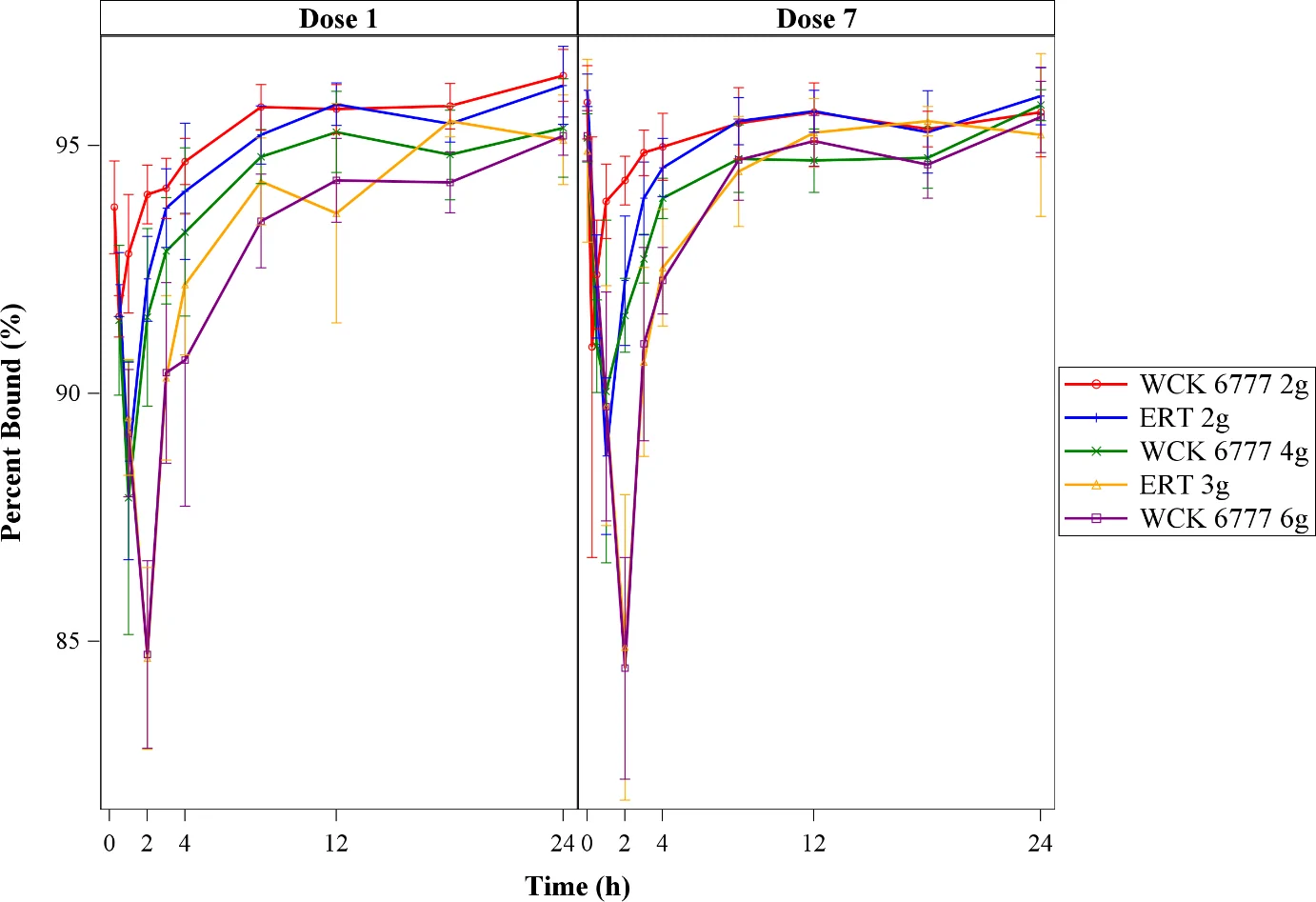
Mean (±SD) percent plasma protein binding of ertapenem over time following the first dose (Day 1) and at steady state (Day 7). Data are presented as mean ± standard deviation percent plasma protein binding of ertapenem at each sampling time. Percent protein binding was calculated from paired total and unbound plasma ertapenem concentrations as: % bound = ([C_total − C_free]/C_total) × 100. Dose 1 represents concentrations following the first dose on Day 1; Dose 7 represents concentrations following the final dose on Day 7 at steady state. WCK 6777 consists of ertapenem co-administered with zidebactam. Treatment group sample sizes were WCK 6777 2 g (*n* = 6), ERT 2 g (*n* = 6), WCK 6777 4 g (*n* = 6), ERT 3 g (*n* = 6), and WCK 6777 6 g (*n* = 6). Error bars represent standard deviation.

Mean plasma protein binding of ERT ranged from approximately 84% to 97% across doses and study days (Fig. 4). Binding exhibited mild concentration dependence, with an increasing unbound fraction at higher total ERT concentrations; however, the magnitude and pattern of protein binding were comparable between ERT-alone and WCK 6777 cohorts, as the mean differences in unbound fraction were <2%. The relationship between total and unbound plasma ERT concentrations was linear across treatment groups (*R*^2^ = 0.997–1).

#### Plasma pharmacokinetics of zidebactam

Mean total and unbound plasma ZID concentration–time profiles following single and multiple once-daily intravenous doses of WCK 6777 (ERT + ZID at 1 + 1, 2 + 2, and 3 + 3 g) and ZID administered alone (2 or 3 g) are shown in Fig. 3. Across all treatment groups, ZID exhibited consistent, monophasic disposition with rapid attainment of peak concentrations and parallel decline phases following the end of infusion. Median *T*_max_ occurred at the end of infusion (0.5 h for 30-min infusions, ~1 h for 60-min infusions, and ~2 h for 120-min infusions) and was driven by infusion duration rather than dose level or co-administration with ERT. Mean terminal elimination *t*_½_ ranged from approximately 2.3–2.7 h for total ZID (Table 6) and 2.6–3.13 h for unbound ZID (Table 7) across cohorts and was not affected by combination dosing.

**TABLE 6:** Summary statistics for total zidebactam single- and multiple-dose PK parameters in plasma by treatment group^*d*^^,^^*e*^

PK parameter (units)	WCK 6777 2 g	ZID 2 g	WCK 6777 4 g	ZID 3 g	WCK 6777 6 g
Total zidebactam single-dose plasma PK parameters					
*C*_max_ (µg/mL)	69.67 (15)	120.41 (22)	133.51 (16)	111.71 (10)	128.42 (11)
*C*_min_ (µg/mL)	0.09 (37)	0.11 (72)	0.10 (44)	0.13 (28)	0.15 (30)
*T*_max_ (h)	0.50 (0.50–0.50)	1.03 (1.02–1.03)	1.00 (1.00–1.00)	2.02 (1.03–2.02)	2.03 (2.02–2.03)
*T*_min_ (h)	18.00 (18.00–23.60)	23.60 (18.00–23.70)	23.60 (18.00–23.60)	23.60 (23.60–23.60)	23.60 (23.60–23.70)
AUC_(0–*t*)_ (µg h/mL)	140.00 (8)	302.40 (18)	320.50 (20)	379.92 (9)	442.32 (14)
AUC_(0–inf)_ (µg h/mL)	141.33 (8)	303.92 (18)	322.04 (20)	381.76 (9)	444.34 (14)
AUC_(0–24)_ (µg h/mL)	141.17 (8)	303.60 (18)	321.76 (20)	381.59 (9)	443.70 (14)
AUC_(0–last)_ (µg h/mL)	141.17 (8)	303.42 (18)	321.35 (20)	381.44 (9)	443.70 (14)
*t*_1/2_ (h)	2.35 (15)	2.60 (14)	2.57 (12)	2.61 (9)	2.71 (6)
CL_T_ (L/h)	7.07 (8)	6.58 (18)	6.21 (20)	7.86 (9)	6.75 (14)
ke (1/h)	0.30 (15)	0.27 (14)	0.27 (12)	0.27 (9)	0.26 (6)
*V*_*d*_ (L)	23.97 (14)	24.67 (28)	23.05 (22)	29.56 (10)	26.43 (16)
Total zidebactam multiple-dose plasma PK parameters					
*C*_max,ss_ (µg/mL)	63.87 (17)	115.71 (16)	130.69 (18)	109.41 (7)	113.79 (16)
*C*_max,ss_/dose ([µg/mL]/mg)	0.06 (17)	0.06 (16)	0.07 (18)	0.04 (7)	0.04 (16)
*C*_min,ss_ (µg/mL)	0.13 (49)	0.12 (68)	0.10 (46)	0.13 (27)	0.12 (36)
*C*_avg,ss_ (µg/mL)	5.56 (8)	11.94 (20)	12.83 (19)	16.05 (7)	16.52 (14)
*C*_τ,ss_ (µg/mL)	0.02 (68)	0.06 (89)	0.05 (176)	0.13 (28)	0.12 (35)
*T*_max,ss_ (h)	0.52 (0.25–0.60)	1.00 (1.00–1.00)	1.02 (1.00–1.02)	2.02 (2.02–2.07)	2.07 (2.02–2.23)
*T*_min_ (h)	18.00 (18.00–24.00)	24.00 (18.00–24.00)	24.00 (18.00–24.00)	24.00 (24.00–24.10)	24.00 (24.00–24.10)
AUC_(0–24),ss_ (µg h/mL)	133.35 (8)	286.34 (20)	308.35 (19)	384.53 (7)	396.60 (14)
AUC_(0–τ),ss_ (µg h/mL)	133.35 (8)	286.34 (20)	308.35 (19)	384.53 (7)	396.60 (14)
AUC_(0–τ),ss_/dose ([µg h/mL]/mg)	0.13 (8)	0.14 (20)	0.15 (19)	0.13 (7)	0.13 (14)
*t*_1/2_ (h)	2.32 (15)	2.64 (12)	2.47 (24)	2.69 (3)	2.65 (10)
CL_T_ (L/h)	7.50 (8)	6.98 (20)	6.49 (19)	7.80 (7)	7.56 (14)
*V*_*d*,ss_ (L)	18.04 (14)	16.57 (18)	13.88 (21)	20.33 (7)	19.35 (13)
Linearity index^*a*^	0.94 (4)	0.94 (13)	0.96 (5)	1.01 (8)	0.89 (11)
RAUC^*b*^	0.94 (4)	0.94 (13)	0.96 (5)	1.01 (8)	0.89 (11)
RC_max_^*c*^	0.92 (15)	0.96 (11)	0.98 (12)	0.98 (13)	0.89 (17)

^*a*^ Linearity index is defined as AUC_(0–τ),ss_ (Dose 7)/AUC_(0–inf)_ (Dose 1).

^*b*^ RAUC is defined as AUC_(0–*τ*)_ (Dose 7)/AUC_(0–24)_ (Dose 1).

^*c*^ RC_max_ is defined as *C*_max_ (Dose 7)/*C*_max_ (Dose 1).

^*d*^ Single-dose parameters are calculated over the 24-h period following Dose 1. Multi-dose parameters are calculated over the 24-h period following Dose 7. For this study, the dosing interval (*τ*) was 24 h; therefore, AUC_(0–24),ss_ and AUC_(0–*τ*),ss_ are numerically identical, and *C*_τ,ss_ represents the concentration at the end of the dosing interval (equivalent to *C*_min,ss_). Values of GM (CV%) are shown, except for *T*_max_ and *T*_min_ for which values of median (min-max) are shown.

^*e*^ AUC, area under the concentration-time curve; Cavg, average concentration; CLT, total clearance; Cmax, maximum concentration; Cmin, minimum concentration; CV, coefficient of variation; GM, geometric mean; ke, elimination rate constant; Tmax, time to maximum concentration; Tmin, time to minimum concentration; Vd, volume of distribution; WCK 6777, ertapenem/zidebactam; ZID, zidebactam; τ, dosing interval; ss, steady state.

**TABLE 7:** Summary statistics for free ZID single- and multiple-dose PK parameters in plasma by treatment group^*d*^ ^*e*^

PK parameter (units)	WCK 6777 2 g	ZID 2 g	WCK 6777 4 g	ZID 3 g	WCK 6777 6 g
Free zidebactam single-dose plasma PK parameters					
*C*_max_ (µg/mL)	64.35 (20)	105.42 (16)	98.41 (55)	108.79 (11)	124.99 (10)
*C*_min_ (µg/mL)	0.09 (22)	0.07 (36)	0.10 (39)	0.14 (26)	0.14 (34)
*T*_max_ (h)	0.50 (0.50–0.50)	1.03 (1.02–1.03)	1.00 (1.00–2.00)	2.02 (1.03–2.02)	2.03 (2.02–2.03)
*T*_min_ (h)	23.60 (18.00–23.70)	23.60 (23.60–23.70)	23.60 (18.00–23.60)	23.60 (23.60–23.60)	23.60 (23.60–23.70)
AUC_(0–*t*)_ (µg h/mL)	125.17 (8)	257.74 (15)	247.40 (42)	379.75 (11)	427.31 (16)
AUC_(0–inf)_ (µg h/mL)	126.50 (8)	259.27 (15)	249.11 (41)	381.44 (11)	429.33 (16)
AUC_(0–24)_ (µg h/mL)	126.34 (8)	259.11 (15)	248.60 (42)	380.91 (11)	428.67 (16)
AUC_(0–last)_ (µg h/mL)	126.34 (8)	259.11 (15)	248.60 (42)	380.91 (11)	428.67 (16)
*t*_1/2_ (h)	2.75 (18)	2.81 (14)	2.63 (15)	2.69 (9)	2.71 (5)
CL_T_ (L/h)	7.91 (8)	7.71 (15)	8.03 (41)	7.86 (11)	6.99 (16)
ke (1/h)	0.25 (18)	0.25 (14)	0.26 (14)	0.26 (9)	0.26 (5)
*V*_*d*_ (L)	31.37 (12)	31.18 (23)	30.42 (40)	30.52 (9)	27.28 (17)
Free zidebactam multiple-dose plasma PK parameters					
*C*_max,ss_ (µg/mL)	57.89 (23)	103.12 (18)	83.36 (57)	111.59 (7)	111.81 (16)
*C*_max,ss_/dose ([µg/mL]/mg)	0.06 (23)	0.05 (18)	0.04 (56)	0.04 (7)	0.04 (15)
*C*_min,ss_ (µg/mL)	0.12 (66)	0.10 (38)	0.17 (864)	0.14 (23)	0.11 (34)
*C*_avg,ss_ (µg/mL)	5.03 (8)	10.48 (20)	9.77 (30)	16.32 (8)	15.83 (14)
*C*_t,ss_ (µg/mL)	0.06 (169)	0.07 (90)	0.11 (2,318)	0.14 (23)	0.11 (34)
*T*_max,ss_ (h)	0.52 (0.25–0.60)	1.00 (1.00–1.00)	1.02 (1.00–2.00)	2.02 (2.02–2.07)	2.07 (2.02–2.23)
*T*_min_ (h)	24.00 (18.00–24.00)	24.00 (18.10–24.00)	24.00 (18.00–24.00)	24.00 (24.00–24.10)	24.00 (24.00–24.10)
AUC_(0–24),ss_ (µg h/mL)	120.70 (8)	251.56 (20)	234.14 (30)	392.09 (8)	380.11 (14)
AUC_(0–*τ*),ss_ (µg h/mL)	120.70 (8)	251.56 (20)	234.14 (30)	392.09 (8)	380.11 (14)
AUC_(0–*τ*),ss_/dose ([µg h/mL]/mg)	0.12 (8)	0.13 (20)	0.12 (30)	0.13 (8)	0.13 (14)
*t*_1/2_ (h)	2.55 (11)	2.78 (15)	3.13 (55)	2.65 (7)	2.70 (8)
CL_T_ (L/h)	8.28 (8)	7.96 (20)	8.54 (30)	7.65 (8)	7.89 (14)
*V*_*d*,ss_ (L)	21.86 (10)	18.79 (14)	30.21 (197)	20.17 (6)	19.69 (13)
Linearity index^*a*^	0.95 (5)	0.97 (13)	0.94 (26)	1.03 (7)	0.89 (13)
RAUC^*b*^	0.96 (5)	0.97 (13)	0.94 (26)	1.03 (7)	0.89 (13)
RC_max_^*c*^	0.90 (15)	0.98 (11)	0.85 (40)	1.03 (10)	0.89 (13)

^*a*^ Linearity index is defined as AUC_(0–*τ*),ss_ (Dose 7)/AUC_(0–inf)_ (Dose 1).

^*b*^ RAUC is defined as AUC_(0–*τ*)_ (Dose 7)/AUC_(0–24)_ (Dose 1).

^*c*^ RC_max_ is defined as *C*_max_ (Dose 7)/*C*_max_ (Dose 1).

^*d*^ Single-dose parameters are calculated over the 24-h period following Dose 1. Multi-dose parameters are calculated over the 24-h period following Dose 7. For this study, the dosing interval (*τ*) was 24 h; therefore, AUC_(0–24),ss_ and AUC_(0–*τ*),ss_ are numerically identical, and *C*_*τ*,ss_ represents the concentration at the end of the dosing interval (equivalent to *C*_min,ss_). Values of GM (CV%) are shown, except for *T*_max_ and *T*_min_ for which values of median (min-max) are shown.

^*e*^ AUC, area under the concentration-time curve; Cavg, average concentration; CLT, total clearance; Cmax, maximum concentration; Cmin, minimum concentration; CV, coefficient of variation; GM, geometric mean; ke, elimination rate constant; Tmax, time to maximum concentration; Tmin, time to minimum concentration; Vd, volume of distribution; WCK 6777, ertapenem/zidebactam; ZID, zidebactam; τ, dosing interval; ss, steady state.

Following single-dose administration (Dose 1), mean total plasma *C*_max_ and AUC values increased in a dose-dependent manner with ZID dose and were comparable between ZID-alone and corresponding WCK 6777 cohorts (Table 6). Apparent CL_T_, *V*_*d*_, and ke were similar across cohorts. Following multiple once-daily doses, mean (CV%) total plasma *C*_max,ss_ and AUC_0–*τ*,ss_ values on Day 7 increased in a dose-dependent manner with ZID dose and were comparable between ZID-alone and WCK 6777 cohorts (Table 6). Apparent CL_T_ and steady-state Vd were similar across treatment groups, demonstrating time-independent PK and no evidence of altered ZID disposition with repeated co-administration of ERT. Dose-normalized *C*_max,ss_ and AUC_(0–*τ*),ss_ metrics were generally similar across treatment groups, supporting approximately linear, dose-dependent PK over the evaluated dose range and indicating no effect of ERT co-administration on the rate or extent of ZID exposure. Accumulation ratios (RAUC and RC_max_) were close to unity across cohorts, indicating achievement of steady state by Day 2 and absence of drug accumulation. Interindividual variability in exposure was modest, with coefficients of variation generally ≤20%. Linearity indices (AUC_0–*τ*,ss_/AUC_₀–∞,single_) were close to unity, further supporting time-independent ZID PK.

**Fig 5:**
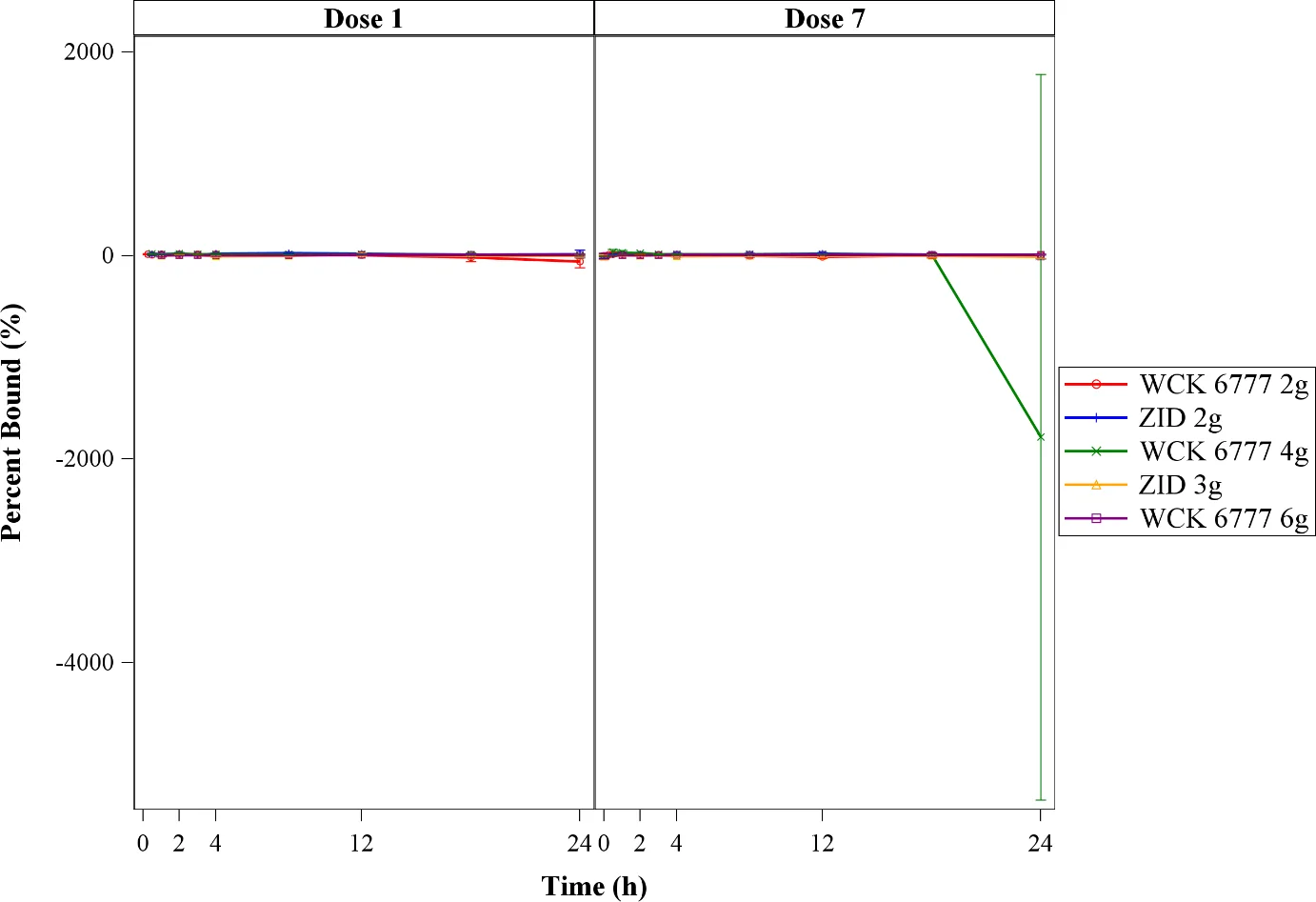
Mean (±SD) percent plasma protein binding of zidebactam over time following the first dose (Day 1) and at steady state (Day 7). Data are presented as mean ± standard deviation percent plasma protein binding of zidebactam at each sampling time. Percent protein binding was calculated from paired total and unbound plasma zidebactam concentrations as: % bound = ([C_total − C_free]/C_total) × 100. Dose 1 represents concentrations following the first dose on Day 1; Dose 7 represents concentrations following the final dose on Day 7 at steady state. WCK 6777 consists of ertapenem co-administered with zidebactam. Treatment group sample sizes were WCK 6777 2 g (*n* = 6), ZID 2 g (*n* = 6), WCK 6777 4 g (*n* = 6), ZID 3 g (*n* = 6), and WCK 6777 6 g (*n* = 6). Error bars represent standard deviation. Negative percent-binding values observed at isolated time points reflect analytical variability when unbound concentrations approached the lower limit of quantification and should not be interpreted as physiologically meaningful.

Unbound (free) ZID PK closely mirrored total-drug findings (Fig. 3; Table 7). After both single and multiple dosing, mean unbound *C*_max_ and AUC values increased in a dose-dependent manner and were similar between ZID-alone and WCK 6777 cohorts. Unbound clearance and volume of distribution were consistent across treatment groups, and unbound *t*_½_ values (approximately 2.6–3.1 h) were comparable to total-drug *t*_½_ values, indicating limited and concentration-independent plasma protein binding. Mean plasma protein binding of ZID was low and consistent across doses and study days, with mean binding generally <10% (Fig. 5). No meaningful differences in protein binding were observed between ZID-alone and WCK 6777 cohorts. The relationship between total and unbound plasma ZID concentrations was linear across treatment groups, supporting reliable derivation of free-drug exposures from total concentrations in both single-agent and combination settings. Isolated negative percent-binding values observed at some time points likely reflect analytical variability when unbound concentrations approached the lower limit of quantification and are not considered physiologically meaningful.

#### Urinary pharmacokinetics

Both ERT and ZID were eliminated predominantly as unchanged parent drug in urine, confirming renal excretion as the primary clearance pathway for both agents (Tables 8 and 9). Following single-dose administration (Day 1), mean cumulative 24-h urinary recovery (Ae_0–24h_) accounted for approximately 52%–59% of the administered dose for ERT and 73%–92% for ZID, with similar recovery observed following multiple dosing at steady state (Day 7). The fraction of dose excreted unchanged (fe_0–24h_) remained consistent across dose levels and dosing days and was comparable between monotherapy and combination (WCK 6777) cohorts, indicating stable renal elimination with repeated dosing and no effect of co-administration.

**TABLE 8:** Summary statistics for ertapenem single- and multiple-dose PK parameters in urine by treatment group^*a*^^,^^*b*^^,^^*c*^

Urine PK Parameters	WCK 6777 2 g	ERT 2 g	WCK 6777 4 g	ERT 3 g	WCK 6777 6 g
Ertapenem single-dose urine PK parameters					
Ae,urine (mg) 0–4 h	339.62 (97.70, 605.00)	748.60 (573.00, 995.00)	794.50 (414.00, 1,620.00)	768.60 (451.00, 1,090.00)	670.13 (87.80, 1,630.00)
Ae,urine (mg) 4–8 h	159.23 (74.20, 268.00)	275.67 (105.00, 414.00)	202.67 (163.00, 272.00)	713.50 (393.00, 1,850.00)	605.83 (246.00, 1,390.00)
Ae,urine (mg) 8–12 h	64.30 (19.80, 83.80)	122.38 (58.80, 204.00)	128.93 (77.50, 202.00)	173.50 (128.00, 227.00)	192.00 (112.00, 305.00)
Ae,urine (mg) 12–24 h	49.80 (25.60, 66.50)	84.22 (49.90, 150.00)	54.38 (19.20, 106.00)	149.15 (84.50, 235.00)	106.95 (58.50, 158.00)
Ae,urine_(0–24)_ (mg)	594.00 (204.00, 856.00)	1,085.67 (321.00, 1,490.00)	1,181.83 (711.00, 2,190.00)	1,676.67 (1,200.00, 2,160.00)	1,572.17 (843.00, 2,400.00)
fe,urine (%) 0–4 h	33.96 (9.77, 60.50)	37.46 (28.70, 49.80)	39.75 (20.70, 81.20)	25.60 (15.00, 36.20)	22.33 (2.93, 54.30)
fe,urine (%) 4–8 h	15.92 (7.42, 26.80)	13.78 (5.27, 20.70)	10.14 (8.14, 13.60)	23.77 (13.10, 61.60)	20.16 (8.18, 46.20)
fe,urine (%) 8–12 h	6.43 (1.98, 8.38)	6.12 (2.94, 10.20)	6.45 (3.88, 10.10)	5.79 (4.28, 7.57)	6.41 (3.74, 10.20)
fe,urine (%) 12–24 h	4.98 (2.56, 6.65)	4.21 (2.49, 7.48)	2.72 (0.96, 5.32)	4.97 (2.82, 7.83)	3.57 (1.95, 5.28)
fe,urine_0–24_ (%)	59.40 (20.40, 85.60)	54.32 (16.10, 74.70)	58.98 (35.50, 109.00)	55.85 (40.10, 71.80)	52.43 (28.10, 79.90)
CL_R0–24_ (L/h)	0.75 (61)	0.78 (57)	0.79 (41)	0.76 (19)	0.73 (42)
Ertapenem multiple-dose urine PK parameters					
Ae,urine (mg) 0–4 h,ss	357.83 (208.00, 525.00)	702.30 (39.50, 1,270.00)	777.80 (433.00, 1,490.00)	1,010.40 (655.00, 1,530.00)	998.17 (467.00, 1,670.00)
Ae,urine (mg) 4–8 h,ss	113.37 (10.10, 205.00)	344.17 (132.00, 911.00)	234.94 (97.70, 473.00)	603.40 (186.00, 1,100.00)	417.33 (209.00, 727.00)
Ae,urine (mg) 8–12 h,ss	54.95 (7.16, 88.10)	136.54 (21.70, 198.00)	88.86 (48.10, 153.00)	171.80 (137.00, 217.00)	113.88 (54.00, 150.00)
Ae,urine (mg) 12–24 h,ss	43.04 (26.70, 68.20)	29.69 (9.35, 72.20)	65.46 (55.00, 81.10)	113.98 (85.60, 159.00)	96.03 (61.50, 167.00)
Ae,urine_0–24,SS_ (mg)	552.83 (325.00, 778.00)	1,067.33 (340.00, 1,700.00)	1,166.60 (652.00, 2,200.00)	1,898.00 (1,060.00, 3,000.00)	1,624.50 (887.00, 2,320.00)
fe,urine (%) 0–4 h,ss	35.78 (20.80, 52.50)	35.09 (1.97, 63.30)	38.94 (21.70, 74.60)	33.68 (21.80, 51.00)	33.28 (15.60, 55.80)
fe,urine (%) 4–8 h,ss	11.34 (1.01, 20.50)	17.19 (6.62, 45.50)	11.72 (4.89, 23.60)	20.08 (6.19, 36.50)	13.91 (6.96, 24.20)
fe,urine (%) 8–12 h,ss	5.50 (0.72, 8.81)	6.83 (1.08, 9.91)	4.45 (2.41, 7.67)	5.73 (4.57, 7.23)	3.80 (1.80, 5.01)
fe,urine (%) 12–24 h,ss	4.30 (2.67, 6.82)	1.48 (0.47, 3.61)	3.27 (2.75, 4.06)	3.80 (2.85, 5.30)	3.20 (2.05, 5.56)
fe,urine_0–24,SS_ (%)	55.28 (32.50, 77.80)	53.35 (17.00, 84.90)	58.36 (32.60, 110.00)	63.28 (35.50, 100.00)	54.20 (29.60, 77.40)
CL_R(0–24),SS_ (L/h)	0.74 (28)	0.78 (53)	0.84 (48)	0.92 (37)	0.90 (44)

^*a*^ Times are relative to time of dosing.

^*b*^ Values of mean (min, max) are shown, except for CL_R_, for which values of GM (CV%) are shown.

^*c*^ Ae, amount excreted in urine; CLR, renal clearance; CV, coefficient of variation; ERT, ertapenem; fe, fraction of dose excreted unchanged in urine; GM, geometric mean; WCK 6777, ertapenem/zidebactam; ss, steady state.

For both compounds, urinary excretion occurred predominantly within the first 8 h post-dose (Tables 8 and 9). Peak urinary recoveries were observed in the 0–4 and 4–8 h collection intervals, followed by progressively lower amounts in later intervals, consistent with plasma concentration–time profiles. The temporal pattern of urinary recovery was similar across dose levels and unaffected by combination dosing. Mean renal clearance (CL_R_) values were stable across cohorts and dosing days, averaging approximately 0.7–0.9 L/h for ERT and ~5–7 L/h for ZID, consistent with elimination primarily via glomerular filtration. No evidence of dose-dependent changes, time-dependent shifts, or altered renal handling was observed following multiple dosing or during co-administration.

**TABLE 9:** Summary statistics for zidebactam single- and multiple-dose PK parameters in urine by treatment group^*a*^^,^^*b*^^,^^*c*^^,^*^d^*

Urine PK Parameters	WCK 6777 2 g	ZID 2 g	WCK 6777 4 g	ZID 3 g	WCK 6777 6 g
Zidebactam single-dose urine PK parameters					
Ae,urine (mg) 0–4 h	544.83 (259.00, 816.00)	1,336.67 (1,060.00, 1,620.00)	1,475.00 (1,080.00, 2,040.00)	1,838.33 (1,270.00, 2,110.00)	1,366.50 (248.00, 2,080.00)
Ae,urine (mg) 4–8 h	150.55 (62.60, 277.00)	360.00 (250.00, 552.00)	259.00 (149.00, 314.00)	631.67 (324.00, 810.00)	1,006.00 (528.00, 2,600.00)
Ae,urine (mg) 8–12 h	33.28 (6.88, 51.30)	80.35 (56.90, 162.00)	83.72 (38.20, 123.00)	143.50 (104.00, 254.00)	167.67 (127.00, 216.00)
Ae,urine (mg) 12–24 h	19.93 (13.30, 24.30)	27.02 (15.10, 54.10)	25.53 (18.00, 37.00)	75.35 (37.60, 110.00)	75.83 (21.50, 176.00)
Ae,urine_(0–24)_ (mg)	733.17 (370.00, 922.00)	1,798.33 (1,470.00, 1,960.00)	1,836.67 (1,420.00, 2,420.00)	2,688.33 (1,820.00, 3,090.00)	2,620.00 (1,040.00, 3,180.00)
fe,urine (%) 0–4 h	54.48 (25.90, 81.60)	66.70 (52.80, 81.00)	73.75 (54.20, 102.00)	61.25 (42.20, 70.40)	45.58 (8.26, 69.40)
fe,urine (%) 4–8 h	15.06 (6.26, 27.70)	17.98 (12.50, 27.60)	12.96 (7.45, 15.70)	21.07 (10.80, 27.00)	33.55 (17.60, 86.80)
fe,urine (%) 8–12 h	3.33 (0.69, 5.13)	4.02 (2.84, 8.11)	4.18 (1.91, 6.13)	4.77 (3.45, 8.46)	5.60 (4.22, 7.22)
fe,urine (%) 12–24 h	1.99 (1.33, 2.43)	1.35 (0.76, 2.70)	1.27 (0.90, 1.85)	2.51 (1.25, 3.65)	2.53 (0.72, 5.86)
fe,urine_(0–24)_ (%)	73.32 (37.00, 92.20)	89.82 (73.30, 97.90)	91.75 (70.90, 121.00)	89.58 (60.60, 103.00)	87.32 (34.50, 106.00)
CL_R(0–24)_ (L/h)	4.98 (39)	5.88 (15)	5.63 (36)	6.95 (25)	5.55 (31)
Zidebactam multiple-dose urine PK parameters					
Ae,urine (mg) 0–4 h,ss	624.50 (383.00, 824.00)	1,210.33 (691.00, 1,570.00)	1,465.00 (1,250.00, 1,640.00)	1,855.00 (1,090.00, 2,200.00)	1,870.00 (1,320.00, 2,280.00)
Ae,urine (mg) 4–8 h,ss	110.20 (7.22, 173.00)	302.00 (302.00, 302.00)	246.00 (219.00, 307.00)	556.83 (403.00, 689.00)	654.33 (253.00, 1,170.00)
Ae,urine (mg) 8–12 h,ss	27.93 (3.55, 45.10)	45.58 (23.10, 55.40)	62.98 (40.20, 93.60)	175.00 (138.00, 212.00)	113.78 (27.70, 160.00)
Ae,urine (mg) 12–24 h,ss	14.75 (7.36, 25.40)	62.37 (18.70, 124.00)	32.48 (13.30, 44.80)	67.67 (45.30, 84.20)	59.50 (34.00, 85.60)
Ae,urine_(0–24),SS_ (mg)	770.17 (495.00, 1,030.00)	1,080.00 (1,080.00, 1,080.00)	1,808.33 (1,580.00, 1,970.00)	2,656.67 (1,770.00, 2,930.00)	2,700.00 (2,200.00, 3,070.00)
fe,urine (%) 0–4 h,ss	62.45 (38.30, 82.40)	60.40 (34.50, 78.40)	73.27 (62.60, 82.10)	61.85 (36.40, 73.40)	62.38 (44.00, 76.00)
fe,urine (%) 4–8 h,ss	11.02 (0.72, 17.30)	15.10 (15.10, 15.10)	12.28 (11.00, 15.30)	18.55 (13.40, 23.00)	21.84 (8.43, 39.10)
fe,urine (%) 8–12 h,ss	2.79 (0.36, 4.51)	2.28 (1.16, 2.77)	3.15 (2.01, 4.68)	5.84 (4.61, 7.05)	3.79 (0.92, 5.34)
fe,urine (%) 12–24 h,ss	1.48 (0.74, 2.54)	3.12 (0.93, 6.20)	1.63 (0.67, 2.24)	2.26 (1.51, 2.81)	1.98 (1.13, 2.85)
fe,urine_(0–24),SS_ (%)	77.02 (49.50, 103.00)	53.80 (53.80, 53.80)	90.35 (78.80, 98.50)	88.48 (59.00, 97.50)	89.93 (73.30, 102.00)
CL_R(0–24),SS_ (L/h)	5.59 (35)	4.68 (NE)	5.84 (25)	6.80 (25)	6.77 (12)

^*a*^ Times are relative to time of dosing.

^*b*^ Values of mean (min, max) are shown, except for CL_R_, for which values of GM (CV%) are shown.

^*c*^ NE, Not estimable.

^*d*^ Ae, amount excreted in urine; CLR, renal clearance; CV, coefficient of variation; fe, fraction of dose excreted unchanged in urine; GM, geometric mean; WCK 6777, ertapenem/zidebactam; ZID, zidebactam; ss, steady state.

## DISCUSSION

This first-in-human study demonstrates that WCK 6777 is safe, well tolerated, and exhibits favorable, predictable PK following once-daily intravenous administration for 7 days in healthy adults. Co-administration did not meaningfully alter the disposition of either component, with plasma exposure and key PK parameters remaining comparable between monotherapy and combination cohorts, thereby supporting further clinical development of WCK 6777. The observed terminal *t*_1/2_ of ERT supports once-daily dosing, and both components achieved steady state rapidly, consistent with time-independent PK. By combining the established antibacterial activity and prolonged half-life of ERT with the β-lactam enhancer activity of ZID, WCK 6777 represents a differentiated β-lactam enhancer strategy (19, 20). Unlike traditional β-lactam/β-lactamase inhibitor combinations that rely solely on enzyme inhibition and lack activity against MBL-producing Enterobacterales, ZID exerts a non-enzymatic mechanism that extends coverage to pathogens expressing both serine and metallo-β-lactamases and lowers the %fT >MIC requirement of the partner β-lactam (23–27). Although aztreonam–avibactam and cefiderocol also provide coverage of metallo-β-lactamase-producing gram-negative organisms, they differ from WCK 6777 in mechanism and administration: aztreonam–avibactam pairs a monobactam with a β-lactamase inhibitor, and cefiderocol is a siderophore cephalosporin, whereas WCK 6777 couples ertapenem with the β-lactam enhancer zidebactam, and its once-daily dosing distinguishes it from these multiple-daily-dose regimens (13–15). Together, these complementary PK and mechanistic features position WCK 6777 as a promising once-daily option with potential utility in both inpatient and OPAT settings. Beyond facilitating earlier discharge or avoiding admission, an effective once-daily outpatient regimen for multidrug-resistant gram-negative infection may also reduce the risk of nosocomial transmission of resistant organisms to other hospitalized patients (16–18, 29).

The safety profile observed across all cohorts was consistent with that of ERT monotherapy and other commercially available β-lactams. No deaths, SAEs, or treatment discontinuations occurred. Reported adverse events were predominantly mild or moderate in severity, self-limited, and most commonly related to infusion-site reactions, which are typical of intravenous β-lactam administration (30, 31) and occurred at frequencies comparable to those reported in Phase I studies of other BL/BLI combinations (11, 12). Transient, asymptomatic elevations in hepatic transaminases were infrequent, reversible, and showed no evidence of dose dependence. Importantly, no neurotoxicity or seizure events were observed at total daily doses up to 6 g (ERT 3 g + ZID 3 g), despite the known association between higher carbapenem exposure and central nervous system effects under certain conditions (11, 30, 32). The protocol-specified, dose-dependent adjustment of infusion duration across cohorts was designed to mitigate peak-related central nervous system risk and may have contributed to the favorable neurologic safety profile observed. Collectively, these findings support the absence of a neurologic safety signal under the studied conditions. However, because the present cohort comprised healthy adults without renal impairment or pre-existing neurologic conditions, evaluation in the target patient populations for clinical use, including individuals with serious gram-negative infections who may have impaired renal function, advanced age, or central nervous system comorbidities that heighten susceptibility to carbapenem-associated neurotoxicity, will be required to confirm this profile and define any neurologic monitoring needs. Finally, asymptomatic QTcF prolongation was observed in two healthy participants and did not require intervention; nonetheless, because QTc effects may carry clinical relevance even in the absence of arrhythmic events, continued ECG monitoring in future studies is warranted, with consideration of additional specific assessments, such as cardiac telemetry or a dedicated thorough QT/QTc study, if warranted.

Pharmacokinetic analyses demonstrated properties supportive of once-daily dosing. Ertapenem exhibited consistent biphasic disposition, with exposure increasing in a dose-dependent manner following both single and multiple dosing, consistent with previous Phase 1 studies (31, 33, 34). Dose-normalized AUC values were generally similar across cohorts, supporting approximately linear PK, while modest differences in *C*_max_ concentrations were primarily attributable to protocol-defined infusion durations rather than altered drug disposition. Unbound ERT exposure closely tracked total drug exposure, with predictable, mildly concentration-dependent protein binding and no evidence of time-dependent changes. Zidebactam exhibited approximately linear, dose-dependent PK across the evaluated dose range, with AUC increasing in a dose-dependent manner and CL remaining consistent following both single and multiple dosing, consistent with previous studies (28). Rapid attainment of steady state and accumulation ratios near unity for both agents indicate efficient elimination and stable systemic exposure over time. Both ERT and ZID were eliminated predominantly as unchanged parent drug in urine, supporting renal excretion as the principal clearance pathway and consistent with CL_R_ values approximating glomerular filtration rate. Renal clearance and fraction excreted unchanged were consistent across dose levels, dosing days, and monotherapy versus combination regimens, providing no evidence of altered renal handling during co-administration. Together with comparable plasma clearance and exposure metrics between monotherapy and combination cohorts, these findings indicate that transporter-mediated or renal PK interactions are unlikely. These findings suggest that renal function will be an important determinant of WCK 6777 dosing. Because the present study enrolled healthy adults with normal renal function, dedicated renal impairment studies and population pharmacokinetic analyses will be required to define dose selection and any necessary dose adjustments across the spectrum of renal function.

Several limitations should be acknowledged. The study population was limited to healthy adults, and the modest sample size and short duration of dosing preclude assessment of rare adverse events or PK variability in patients with comorbidities, altered renal function, or critical illness. In addition, tissue distribution was not directly evaluated and will require further characterization in patient studies. Importantly, dedicated population pharmacokinetic analyses in larger and more clinically diverse patient populations will be necessary to fully characterize inter-individual variability and inform dose selection and optimization for Phase II/III trials. Nonetheless, the randomized, double-blind, placebo-controlled design; inclusion of monotherapy and combination arms; and extensive plasma and urine PK sampling strengthen the reliability of the findings and provide a robust foundation for subsequent clinical evaluation.

In conclusion, WCK 6777 was safe and well tolerated and demonstrated predictable, time-independent PK following once-daily intravenous administration in healthy adults. Co-administration did not alter the disposition of either component, providing no evidence of a clinically meaningful PK interaction. The combination’s complementary mechanisms of action, broad resistance coverage, including activity against MBL-producing Enterobacterales, and compatibility with once-daily dosing support continued clinical development, including evaluation in Phase II/III efficacy studies and potential application in OPAT.

## MATERIALS AND METHODS

### Study design

This was a Phase I, single-center study to investigate the safety, tolerability, and PK of three dose-escalating regimens of WCK 6777 (ERT + ZID) and two dose-escalating regimens of ERT alone and ZID (WCK 5107) alone in 52 healthy adult male and female participants aged 18–45 years old. All participants provided written informed consent prior to any study procedure. The study was sponsored by the Division of Microbiology and Infectious Diseases, National Institute of Allergy and Infectious Diseases, National Institutes of Health, under contract HHSN272201500005I, awarded to DynPort Vaccine Company, Frederick, Maryland.

### Participants

Healthy adult volunteers aged 18–45 years were eligible for enrollment after providing written informed consent and agreeing to comply with all study procedures, restrictions, and confinement requirements. Participants were required to be in good general health based on medical history, physical examination (PE), vital signs (VS), 12-lead ECG, and clinical laboratory assessments performed at screening and prior to dosing, including an estimated creatinine clearance ≥80 mL/min (Cockcroft–Gault). Individuals with a diagnosis of a chronic disorder within 90 days of screening, an unstable chronic disease (including renal failure or seizure disorder), or any other clinically significant disease or disorder, medical or surgical procedure, or trauma within 4 weeks prior to initiation of study drug were excluded from participation. Minor, non-clinically significant Grade 1 laboratory abnormalities were permitted at the investigator’s discretion, with prespecified exclusions for hepatic, renal, and urinary parameters. Participants were required to have adequate venous access for repeated blood sampling and no evidence of acute febrile or infectious illness within 7 days before dosing. Female participants were required to be non-pregnant and non-lactating and either of non-childbearing potential or using a highly effective method of contraception from screening through 30 days after the last dose. Sexually active male participants were required to be vasectomized or to use barrier contraception and to refrain from sperm donation during the same period. Participants agreed to avoid excessive physical activity during the inpatient period and surrounding the final study visit.

Key exclusion criteria included a history of clinically significant drug or food hypersensitivity (including β-lactam antibiotics or ERT), unstable or recently diagnosed chronic disease, clinically significant medical or surgical events within 4 weeks prior to dosing, prior exposure to ZID, seizure disorders (excluding childhood febrile seizures), or recent *Clostridioides difficile* infection. Additional exclusions included recent use of prohibited medications or investigational products, including prescription medications such as probenecid, furosemide, antibiotics, valproic acid, or agents known to interact with human organic anion transporter, as well as non-prescription medications, herbal products, vitamins, nutritional supplements, and illicit or recreational substances within 14 days prior to enrollment, recent blood or plasma donation, positive tests for pregnancy, drugs of abuse, alcohol, HIV, hepatitis B, or hepatitis C, significant tobacco or nicotine use, and a history of binge or heavy alcohol consumption.

### Treatment cohorts

Seven sequential cohorts were evaluated (Table 1; Fig. 1). WCK 6777 (ERT + ZID) was assessed in three cohorts (1, 4, 7); ERT alone in two (2, 5); and ZID alone in two (3, 6). The study was double-blind and placebo-controlled except for ZID-alone cohorts, where placebo was omitted given prior high-dose ZID safety data (28). Each cohort included six participants receiving study drug and, when applicable, two receiving placebo. Single-agent cohorts established baseline safety and PK data and enabled evaluation of potential PK interactions and characterization of dose-dependent PK across the evaluated dose range.

Enrollment proceeded sequentially from Cohorts 1–7, beginning with the lowest WCK 6777 dose. Single-agent cohorts (2, 3, 5, 6) were conducted in parallel and completed before the corresponding combination cohorts (4 and 7). Placebo participants were included only in WCK 6777 and ERT-alone cohorts. Sentinel dosing was used in each WCK 6777 cohort: two participants (one active and one placebo) received the initial dose and were observed for 24 h before full enrollment. All study drugs were administered once daily as IV infusions for seven consecutive days. Infusion volume and duration increased from 100 mL over 30 ± 5 min (Cohort 1) to 250 mL over 60 ± 10 min (Cohorts 2–4) and 250 mL over 120 ± 10 min (Cohorts 5–7). Doses were escalated from 2 to 3 g/day for single agents and from 2 g/day (1 g ERT + 1 g ZID) to 4 g/day (2 g + 2 g) and 6 g/day (3 g + 3 g) for WCK 6777. Dose escalation was overseen by the Safety Monitoring Committee (SMC) and the Division of Microbiology and Infectious Diseases (DMID). Advancement to each subsequent cohort required SMC review of unblinded cumulative safety data through Day 11 (+3 days) and DMID approval based on study-team recommendations.

### Safety assessments

Safety was monitored from the start of study drug infusion on Day 1 through the final visit on Day 11 (±3 days), with all post-dose assessments compared with the pre-dosing baseline (Day −1). Safety evaluations included TEAEs, PE, VS, clinical laboratory testing, and 12-lead ECGs. TEAEs were collected from first dosing through the final visit and assessed by qualified study clinicians for severity, seriousness, duration, outcome, and relationship to study treatment. Symptom-directed PE was performed as clinically indicated from Days 1 through 7, with complete PEs conducted on Days 8 and 11. Vital signs were measured on Days 1 through 8 and on Day 11. Clinical laboratory safety assessments were performed on Days 2, 4, 6, 8, and 11 and included chemistry, hematology, coagulation, and urinalysis parameters. Chemistry assessments included electrolytes, serum creatinine with estimation of CL_CR_ by the Cockcroft–Gault method, blood urea nitrogen, fasting glucose, total and direct bilirubin, ALT, AST, alkaline phosphatase, lactate dehydrogenase, total protein, and albumin. Hematology assessments included hemoglobin, hematocrit, red blood cell count, platelet count, and white blood cell count with differential. Coagulation testing included international normalized ratio with prothrombin time and aPTT. Urinalysis consisted of routine dipstick testing, with urine microscopy performed if dipstick results were abnormal. Twelve-lead ECGs were obtained on Days 8 and 11, recorded in triplicate, and analyzed using mean values. The QT interval was corrected for heart rate using Fridericia’s formula (QTcF).

All TEAEs and SAEs were recorded through the final visit and graded for severity as mild (Grade 1), moderate (Grade 2), or severe (Grade 3) according to predefined criteria. The relationship of each event to study drug was assessed by the investigator based on temporal association, alternative etiologies, biological plausibility, and concomitant medications.

### Pharmacokinetic assessments and analysis

Venous blood samples were collected pre-dose and up to 24 h following completion of infusion on Days 1 and 7 to characterize the plasma PK of ERT and ZID. Sampling schedules were determined by infusion duration. For 30-min infusions (Cohort 1), samples were obtained at 0, 0.25, 0.5 (end of infusion), 1, 2, 3, 4, 8, 12, 18, and 24 h. For 60-min infusions (Cohorts 2–4), samples were obtained at 0, 0.5, 1 (end of infusion), 2, 3, 4, 8, 12, 18, and 24 h. For 120-min infusions (Cohorts 5–7), samples were obtained at 0, 1, 2 (end of infusion), 3, 4, 8, 12, 18, and 24 h following infusion start. From Days 2 through 7, pre-dose (24-h post-dose; trough) and 12-h post-dose plasma samples were collected to assess attainment of steady state. Urine was collected on Days 1 and 7 over 0–4, 4–8, 8–12, and 12–24 h intervals.

Plasma and urine concentrations of ERT and ZID were quantified using validated liquid chromatography-tandem mass spectrometry methods. The lower limits of quantification were 0.1 µg/mL for ERT and 0.05 µg/mL for ZID, with calibration curves demonstrating linearity (*r*² > 0.99). Plasma protein binding was assessed by ultrafiltration using a 10-kDa molecular-weight cutoff, and unbound fractions were calculated as the ratio of ultrafiltrate to total plasma concentration (Data S1 to S6 in the Supplemental material).

The PK analysis population included participants with sufficient data to estimate one or more PK parameters; only estimable parameters were summarized. Pharmacokinetic analyses were performed using noncompartmental methods in Phoenix WinNonlin (version 8.3; Certara, USA) with actual sampling times. Noncompartmental analyses were conducted for both total and unbound plasma concentration–time data. Single-dose plasma PK parameters included *C*_max_, *C*_min_, *T*_max_, *T*_min_, AUC_0–*t*_, AUC_0–24_, AUC_0–last_, AUC_0–∞_, CL_T_, *V*_*d*_, *t*_½_, and ke. Steady-state PK parameters were estimated on Day 7. Multiple-dose (steady-state) PK parameters included *C*_max,ss_, *C*_min,ss_, *C*_avg,ss_, *C*_*τ*,ss_, *T*_max,ss_, AUC_0–24,ss_, AUC_0–*τ*,ss_, CL_T,ss_, *V*_d,ss_, and *t*_½,ss_. PK parameters were summarized descriptively by treatment and dose level using mean, standard deviation, coefficient of variation, median, range, and geometric mean. AUC values were calculated using the linear-up/log-down trapezoidal method, and dose-normalized exposure metrics (*C*_max,ss_/dose and AUC_0–*τ*,ss_/dose for multiple doses) were reported. Accumulation and time linearity were evaluated by comparison of *C*_max_ and AUC_0–*t*_ between Days 1 and 7 using accumulation ratios (RAUC and RC_max_) and a linearity index (AUC_0–*τ*,ss_/AUC_0–∞,single_). Potential PK interactions between ERT and ZID were evaluated by descriptive comparison of exposure (*C*_max_, AUC) and disposition parameters (CL, *V*_*d*_, and *t*_½_) between monotherapy and combination cohorts; the study was not designed or powered for formal bioequivalence testing. Urinary PK parameters included cumulative amount excreted (single dose: Ae_0–4,_ Ae_4–8,_ Ae_8–12,_ Ae_12–24_, and Ae_0–24_; multiple doses: Ae_0–4,ss,_ Ae_4–8,ss,_ Ae_8–12,ss,_ Ae_12–24,ss_, and Ae_0–24,ss_), fraction excreted unchanged (single dose: fe_0–4,_ fe_4–8,_ fe_8–12,_ fe_12–24_, and fe_0–24_; multiple doses: fe_0–4,ss,_ fe_4–8,ss,_ fe_8–12,ss,_ fe_12–24,ss_, and fe_0–24,ss_), and renal clearance (CL_R_ = Ae/AUC), which were summarized descriptively by treatment and dose level.

## Data Availability

No sequence or other database-deposited data sets were generated. De-identified participant-level data that support the findings of this study may be made available in accordance with NIH/NIAID data-sharing recommendations and policies: https://www.niaid.nih.gov/research/data-sharing-recommendations. Requests may be directed to the corresponding author.

## SUPPLEMENTAL MATERIAL

Data S1 aac.00390-26-s0001.pdf The determination of total ertapenem in treated human plasma, with sodium heparin as anticoagulant, by LC-MS/MS bioanalytical method validation report.

Data S2 aac.00390-26-s0002.pdf The determination of free ertapenem in treated human plasma ultrafiltrate, with sodium heparin as anticoagulant, by LC-MS/MS bioanalytical method validation report.

Data S3 aac.00390-26-s0003.pdf The determination of total zidebactam in treated human plasma, with sodium heparin as anticoagulant, by LC-MS/MS bioanalytical method validation report.

Data S4 aac.00390-26-s0004.pdf The determination of free zidebactam in treated human plasma ultrafiltrate, with sodium heparin as anticoagulant, by LC-MS/MS bioanalytical method validation report.

Data S5 aac.00390-26-s0005.pdf The determination of ertapenem in treated human urine by LC-MS/MS bioanalytical method validation report.

Data S6 aac.00390-26-s0006.pdf The determination of zidebactam in treated human urine by LC-MS/MS bioanalytical method validation report.

Supplemental material aac.00390-26-s0007.pdf Legends for all Supplemental data.

Supplemental tables aac.00390-26-s0008.docx Tables S1 to S3.

ASM does not own the copyrights to Supplemental Material that may be linked to, or accessed through, an article. The authors have granted ASM a non-exclusive, world-wide license to publish the Supplemental Material files. Please contact the corresponding author directly for reuse.
