# Supplementary material for: A Phase I, randomized, double-blind, placebo-controlled, dose escalation study to evaluate the safety, tolerability, and pharmacokinetics of intravenous ertapenem and zidebactam in combination (WCK 6777) in healthy adult subjects
Source: Antimicrob Agents Chemother. 2026 Sep 29;70(11):e00390-26. doi: 10.1128/aac.00390-26 (PMC13731237; doi:10.1128/aac.00390-26)
Supplement: Supplementary file 1 [file aac.00390-26-s0001.pdf]

# 1. TITLE: THE DETERMINATION OF TOTAL ERTAPENEM IN TREATED HUMAN PLASMA, WITH SODIUM HEPARIN AS ANTICOAGULANT, BY LC-MS/MS

## BIOANALYTICAL METHOD VALIDATION REPORT

| | |
|---|---|
| Bioanalytical Project Number: | V1872106P5 |
| Sponsor: | DynPort Vaccine Company, LLC<br>110 Thomas Johnson Drive<br>Suite 200<br>Frederick, MD 21702 |
| Bioanalytical Laboratory: | KCAS Bio<br>10830 S Clay Blair Boulevard<br>Olathe, KS 66061 |
| Issue Date: | March 2023 |
| Amendment 1 Issue Date: | October 2023 |
| Amendment 2 Issue Date: | Date of PI Signature |

# 2. SYNOPSIS

This report presents the validation data for an LC-MS/MS method for the determination of total ertapenem in treated human plasma, with sodium heparin as anticoagulant. The assay was analyzed in the range of 0.200 - 200 mcg/mL and is for use on human plasma samples obtained during clinical trials.

**AMENDMENT OUTLINE FORM**

| | |
|---|---|
| 1. Amendment Number 1 | This amendment was created to report:<br>• Long-term matrix stability at -70 °C for up to 60, 88, and 181 days (with comed[2]) and 81, 109, and 202 days (without comed[2]) for total ertapenem in treated human plasma, with sodium heparin as anticoagulant with and without the presence of comed[2]. |
| 2. Amendment Number 2 | This amendment was created to report:<br>• Long-term matrix stability at -70 °C for up to 47 days for total ertapenem in untreated human plasma, with sodium heparin as anticoagulant with and without the presence of comed[2]. |

# 3. TABLE OF CONTENTS FOR THE BIOANALYTICAL METHOD VALIDATION REPORT

1. TITLE: THE DETERMINATION OF TOTAL ERTAPENEM IN TREATED HUMAN PLASMA, WITH SODIUM HEPARIN AS ANTICOAGULANT, BY LC-MS/MS ....1
2. SYNOPSIS ....2
3. TABLE OF CONTENTS FOR THE BIOANALYTICAL METHOD VALIDATION REPORT ....3
4. LIST OF TABLES ....4
5. SIGNATURE PAGE ....5
6. QUALITY ASSURANCE STATEMENT ....6
7. STUDY COMPLIANCE STATEMENT ....7
8. LIST OF ABBREVIATIONS AND DEFINITIONS OF TERMS ....8
9. AMENDMENT ....10
10. EXPERIMENTAL DESIGN AND RESULTS ....10
10.1. Reference Standards ....10
10.2. Calibration Standards and QC Samples Preparation ....10
10.3. Carryover ....11
10.4. Long-Term Matrix Stability at -70 °C ....11
10.5. SOP Deviations and Investigations ....11
11. CONCLUSION ....11
12. PERTINENT SOPS ....11
13. ARCHIVES ....12
14. REFERENCES ....12
15. TABLES ....13

# 4. LIST OF TABLES

Table 1: Abbreviations and Codes ....8
Table 2: Mass Spec Transitions ....13
Table 3: Reference Standards ....13
Table 4: Calibration Standards and QC Samples Preparation ....14
Table 5: Matrix Description ....14
Table 6: Summary of Results and Conclusions ....15
Table 7: Summary of Validation Run Objectives and Outcome ....18
Table 8: Carryover ....18
Table 9: Long-Term Stability at -70 °C - Untreated ....19

# 5. SIGNATURE PAGE

Bioanalytical Project Number: V1872106P5

Amendment 2 Issue Date: Date of PI Signature

**APPROVED**

DocuSigned by:
Anika Pippin
Signer Name: Anika Pippin
Signing Reason: I approve this document
Signing Time: 27-Jun-2024 | 19:05 CDT
BE3AA1198E40459E8A7E3C615D981D82

Anika Pippin, B.S.
Principal Investigator
07Dec2023 to present

Yu-Hui Fu, M.S.
Principal Investigator
04Dec2023 to 06Dec2023

**REVIEWED**

DocuSigned by:
Marsha Luna
Signer Name: Marsha Luna
Signing Reason: I have reviewed this document
Signing Time: 27-Jun-2024 | 18:16 CDT
C9CC738561AF4CC1B375ED013DD1E4BD

Marsha Luna, B.S.
Management

## 6. QUALITY ASSURANCE STATEMENT

This Bioanalytical Method Validation Report has been found to describe accurately the methods and SOPs used and to accurately reflect the raw data collected. The Quality Assurance Unit initiated audits on this study and reported the findings to the Principal Investigator (PI) and Management as follows:

| | | **Date(s) Sent to:** | |
|---|---|---|---|
| **Inspection Date(s)** | **Inspection Phase** | **PI** | **Management** |
| 20May2024 | Data Audit | 20May2024 | 20May2024 |
| 24Jun2024 | Draft Report Audit A2 | 24Jun2024 | 25Jun2024 |
| 26Jun2024 | Final Report Review A2 | 26Jun2024 | 26Jun2024 |

**REVIEWED**

DocuSigned by:

Signer Name: McKenzie Moses
Signing Reason: I have reviewed this document
Signing Time: 27-Jun-2024 | 17:44 CDT
EC84DBFDD7D44D238BCE4363EEA6D90A

McKenzie Moses, B.S.
Quality Assurance Unit

# 7. STUDY COMPLIANCE STATEMENT

I, the undersigned, hereby declare that this study was conducted in compliance with the relevant bioanalytical aspects of FDA Guidance for Industry on Bioanalytical Methods Validation, May 2018 [1] and KCAS Bio (KCAS) procedures and that the work was performed by me, or under my direction, and the report reflects a true and accurate record of the results obtained.

The acceptance criteria are based on the FDA Guidance for Industry, Bioanalytical Method Validation (2018). [1]

**APPROVED**

DocuSigned by:

Anika Pippin

Signer Name: Anika Pippin
Signing Reason: I approve this document
Signing Time: 27-Jun-2024 | 19:05 CDT
BE3AA1198E40459E8A7E3C615D981D82

Anika Pippin, B.S.
Principal Investigator
07Dec2023 to present

Yu-Hui Fu, M.S.
Principal Investigator
04Dec2023 to 06Dec2023

# 8. LIST OF ABBREVIATIONS AND DEFINITIONS OF TERMS

**Table 1: Abbreviations and Codes**

| Abbreviation | Definition |
|---|---|
| < | Concentration less than the lowest acceptable calibration standard |
| > | Concentration exceeded the upper limit of calibration range (> ULOQ) |
| A | Amendment |
| A&P | Accuracy and Precision |
| AQL | Above Quantitation Limit |
| ARQC | Above Range Quality Control |
| Batch | Is the same as Run |
| BQL | Below Quantifiable Limit |
| Cal | Calibration standard |
| Conc. | Concentration |
| Conv. | Conversion |
| %CV | Coefficient of Variation |
| dev | Deviation |
| diff | Difference |
| Dil | Dilution |
| EMA | European Medicines Agency |
| FDA | Food and Drug Administration |
| GLP | Good Laboratory Practice |
| HPLC | High Performance Liquid Chromatography |
| ID | Identification |
| ICH | International Council for Harmonisation of Technical Requirements for Registration of Pharmaceuticals for Human Use |
| IS | Internal Standard |
| ISR | Incurred Sample Reanalysis |
| $K_2EDTA$ or $K_3EDTA$ | Dipotassium or Tripotassium Ethylenediaminetetraacetic Acid |
| KCAS | KCAS Bio |
| LC-MS/MS | Liquid Chromatography Tandem Mass Spectrometry |
| LLOQ | Lower Limit of Quantitation |

**Table 1: Abbreviations and Codes (Continued)**

| Abbreviation | Definition |
|---|---|
| mcg or µg or ug | Microgram |
| mcL or µL or uL | Microliter |
| ME | Matrix Effect |
| MF | Matrix Factor |
| mL | Milliliter |
| MS | Mass Spectrometry |
| n | number |
| N.C. | Not Calculated |
| ND | No difference reported as ISR will be counted |
| ng | nanogram |
| n.report | No Data available to report |
| N/A or NA | Not Applicable |
| N.R. | Not Reported |
| p.a.r. | Peak Area Ratio |
| PI | Principal Investigator |
| QC | Quality Control Sample |
| QCs | Quality Control Samples |
| OECD | Organization for Economic Co-operation and Development |
| RT | Room Temperature |
| sd or SD | Standard deviation |
| Seq | Sequence |
| SOP (s) | Standard Operating Procedure (s) |
| temp | Temperature |
| TP | Test procedure |
| UHPLC | Ultra High Performance Liquid Chromatography |
| ULOQ | Upper Limit of Quantitation |
| WAS | Working Analyte Standard |
| Watson LIMS 7.5 | Watson Laboratory Information Management System, Version 7.5, Service Pack 1 |
| WIS | Working Internal Standard |

This is a basic list. Some of the abbreviations may not have been used in this report.

# 9. AMENDMENT

This amendment was created to report long-term matrix stability at -70 °C for up to 47 days for total ertapenem in untreated human plasma, with sodium heparin as anticoagulant with and without the presence of comed[2].

The validation procedure was designed to meet or exceed the requirements of FDA Guidance for Industry on Bioanalytical Methods Validation, May 2018. The software programs used were Microsoft Office Excel 365, acquisition and processing software Analyst 1.7.2, and data management software Watson LIMS 7.5.

# 10. EXPERIMENTAL DESIGN AND RESULTS

In this method, ertapenem and internal standards, ertapenem-$d_4$, were released from treated human plasma using 96-well protein precipitation. After extraction, the sample was chromatographed using reversed phase HPLC with a Fortis C18 analytical column. Ertapenem and ertapenem-$d_4$ (IS) were detected by monitoring the precursor and product ions described in Table 2 using an Applied Biosystems Sciex API 5000 or API 6500 LC-MS/MS. The quantification was based on setting a calibration graph using the internal standard method based on bioanalytical test procedure TP-80193, entitled “LC-MS/MS Analysis of Ertapenem in Human Sodium Heparin Plasma (100 mcL, 0.200 - 200 mcg/mL; Prior to MES Treatment)”. The test procedure can be found in Annex 1 (V1872106P5).

Human plasma is treated with 0.1M 2-(N-Morpholino) ethane sulphonic acid hydrate (MES) buffer, pH 6.4 at a 1:1 ratio. The validation assay range is calculated based on plasma volume only to mimic unknown study samples. The assay range is 0.200 to 200 mcg/mL in untreated human sodium heparin plasma.

A summary of the validation results is provided in Table 6. A summary describing the objective(s) and outcome of each validation run attempted is provided in Table 7.

The method was validated within the analytical range of 0.200 - 200 mcg/mL in human plasma. No development work was performed after the method was validated.

## 10.1. Reference Standards

Reference standards used during this validation are presented in Table 3.

## 10.2. Calibration Standards and QC Samples Preparation

Calibration standards and quality control pools were prepared by spiking appropriate levels of ertapenem into treated human plasma, with sodium heparin as anticoagulant. Primary reference standards were prepared in at least duplicate, and they need to compare within 5% of each other. Standards and QCs were prepared and stored frozen at KCAS at -70 °C. Standards, QCs, and the

critical solutions used during this validation are described in Table 4, and matrix used is described in Table 5.

## 10.3. Carryover

Carryover is evaluated within each run. It consists of measuring the response for each analyte and if it exceeds 20.0% of the response of LLOQ standard or other sample reflecting the lower sensitivity limit required, a carryover assessment is completed, and affected samples are rejected. The internal standard carryover evaluation is assessed within each run and if it exceeds 5.0% of the average internal standard response of acceptable standards and QCs, impact is evaluated within the run. Data is presented in Table 8.

## 10.4. Long-Term Matrix Stability at -70 °C

To evaluate ertapenem in untreated human plasma with and without the presence of comed[2], with sodium heparin as anticoagulant, stability during sample storage, aliquots of the high and low concentration QC samples were stored frozen at -70 °C nominally for 47 days (with and without the presence of comed[2]) and prior to being analyzed, along with freshly prepared standards and QC samples. To be deemed stable, the mean %Bias at each level must be within ± 15.0% with %CV ≤ 15.0%.

Data are provided in Table 9.

## 10.5. SOP Deviations and Investigations

No SOP deviations occurred. No investigations were conducted during this study.

# 11. CONCLUSION

Ertapenem is stable in untreated human plasma with and without the presence of comed[2], with sodium heparin as anticoagulant, for up to 47 days upon storage in polypropylene tubes at -70 °C.

# 12. PERTINENT SOPS

The following SOPs were followed during the conduct of this study:

SOP-00073 [05-07] - Bioanalytical Method Validation - Chromatographic Assays

SOP-00035 [02] - Procedures, Reporting, and Acceptance Criteria for Bioanalytical Chromatographic Projects

## 13. ARCHIVES

All raw data, including paper and electronic files associated with this study will remain archived at a location specified by KCAS, 10830 S Clay Blair Boulevard, Olathe, Kansas 66061 for a period of at least 10 years, after which the client may be contacted to arrange for continued storage of the files, or disposition according to an agreed procedure. KCAS will maintain an electronic (scanned/true) copy of the method validation report that can be viewed and printed on site. The study plan, documentation, raw data, and final report are indexed under Bioanalytical Project Number V1872106P5A2.

## 14. REFERENCES

1. Guidance for Industry: Bioanalytical Method Validation. U.S. Department of Health and Human Services. Food and Drug Administration. Center for Drug Evaluation and Research. Center for Veterinary Medicine. May 2018.
2. Comed: QCs in the presence of 37.5 mcg/mL zidebactam.

# 15. TABLES

## Table 2: Mass Spec Transitions

| Analyte | Transition |
|---|---|
| Ertapenem | m/z 476.047→431.9 |
| Ertapenem-$d_4$ (IS) | m/z 480.2→436.3 |

## Table 3: Reference Standards

| Analyte | Supplier | Lot/Batch Number | Purity | Expiration or Retest Date | Conversion Factor | Storage Condition |
|---|---|---|---|---|---|---|
| Ertapenem Sodium | MedChemExpress | 229333 | 98.43% | Retest 29Sep2025 | 1.046 | 2-8°C |
| Ertapenem-$d_4$ Sodium Salt (IS) | AlsaChim | DH-ALS-20-002-P2 | 100%* | Retest 26Oct2023** | 1.000* | -20°C |
| Zidebactam | Wockhardt | WK1/WP-0060/016 | 90.40% | Expire 01Mar2024** | 1.000 | 2-8°C |

Certificates of analysis for the above reference standards are on file at KCAS.
*Internal standard reference material was used as is with 100% purity and 1.000 conversion factor.
**Reference material was within retest/expiration at the time of use.

## Table 4: Calibration Standards and QC Samples Preparation

| Critical Solutions | Lot # for Standards (Prep Date) | Lot # for QCs (Prep Date) | Standards Lot (Prep Date) | QCS Lot (Prep Date) | Runs used |
|---|---|---|---|---|---|
| Treated Human Plasma (1:1 MES Buffer:Plasma) | BioChemed, Lot V1872106P5A2-7-2 | | V1872106P5A2-8-1 Set A (06Dec2023) | V1872106P5A2-9-1 Set A (06Dec2023) | 1 |
| Chilled ~0.1M MES Buffer stored at -20 °C in polypropylene | V1872106P5A2-1-1 (05Dec2023) | V1872106P5A2-1-1 (05Dec2023) | | | |
| Treated Human Plasma (1:1 MES Buffer:Plasma) stored at -70 °C in polypropylene | V1872106P5A2-7-1 (06Dec2023) | V1872106P5A2-7-1 (06Dec2023) | | | |
| Treated Human Plasma (1:1 MES Buffer:Plasma) | BioChemed, Lot V1872106P5A2-16-2 | | V1872106P5A2-17-1 Set B (22Jan2024) | V1872106P5A2-18-1 Set B (22Jan2024) | 2 |
| Chilled ~0.1M MES Buffer stored at -20 °C in polypropylene | V1872106P5A2-12-1 (22Jan2024) | V1872106P5A2-12-1 (22Jan2024) | | | |
| Treated Human Plasma (1:1 MES Buffer:Plasma) stored at -70 °C in polypropylene | V1872106P5A2-16-1 (22Jan2024) | V1872106P5A2-16-1 (22Jan2024) | | | |
| Treated Human Plasma (1:1 MES Buffer:Plasma) | BioChemed, Combo-223-1 | | N/A | Untreated QCs V1872106P5A2-10-1 (06Dec2023) | 1, 2 (LTS) |
| Chilled ~0.1M MES Buffer stored at -20 °C in polypropylene | N/A | V1872106P5A2-1-1 (05Dec2023) | | | |
| Treated Human Plasma (1:1 MES Buffer:Plasma) | BioChemed, Combo-223-1 | | N/A | Untreated Comed QCs V1872106P5A2-11-1 (06Dec2023) | 1, 2 (LTS) |
| Chilled ~0.1M MES Buffer stored at -20 °C in polypropylene | N/A | V1872106P5A2-1-1 (05Dec2023) | | | |
| Zidebactam in deionized water stored at -20 °C in polypropylene | N/A | V1872106P2A3-1-1 (05Dec2023) | | | |

## Table 5: Matrix Description

| Matrix | Source Lot # | Date Received |
|---|---|---|
| Sodium Heparin Human Plasma V1872106P5A2-7-2 and V1872106P5A2-16-2 (Combo-223-1) | BioChemed, Lots BC46306NH-1, BC46306NH-2, BC46306NH-4, BC46306NH-6 | Oct2023 |

## Table 6: Summary of Results and Conclusions

| Methodology – TP-80193 | Method Performance – LC-MS/MS | Hyperlink – N/A | Comments |
|---|---|---|---|
| Matrix | Plasma | | |
| Anticoagulant | Sodium heparin | | |
| Assay Volume | 100 mcL | | |
| **Selectivity** | | | |
| Analyte | Ertapenem | | V1872106P5 |
| Internal Standard | Ertapenem-$d_4$ | | V1872106P5 |
| Matrix Effect (6 spiked matrix lots) | Complies | | V1872106P5 |
| Interference | Complies | | V1872106P5 |
| Hemolytic Effect Evaluation | Complies for 0.5% hemolysis<br>Does not comply for 1% and 2% hemolysis | | V1872106P5 |
| Lipemic Effect Evaluation | Complies | | V1872106P5 |
| Carryover | Assessed with every run with standards and QCs | Summary tables Table 8<br><br>Report text Section 10.3 | |
| **Linearity** | | | |
| Weighting | $1/x^2$ | | V1872106P5 |
| Bias at LLOQ | 0.0% | | V1872106P5 |
| Bias above LLOQ | -3.3 to 4.4% | | V1872106P5 |
| Bioanalytical Range | Complies<br>0.200 - 200 mcg/mL | | V1872106P5 |
| **Precision** | | | |
| Intra-assay | 5.0 to 18.0% (LLOQ);<br>3.4 to 13.6% (above LLOQ) | | V1872106P5 |
| Inter-assay | 12.5% (LLOQ);<br>7.4 to 10.0% (above LLOQ) | | V1872106P5 |
| **Accuracy** | | | |
| Intra-assay | -9.5 to 10.0% (LLOQ);<br>-7.3 to 11.3% (above LLOQ) | | V1872106P5 |
| Inter-assay | 4.0% (LLOQ);<br>-1.3 to 1.8% (above LLOQ) | | V1872106P5 |

## Table 6: Summary of Results and Conclusions (Continued)

| Methodology – TP-80193 | Method Performance – LC-MS/MS | Hyperlink – N/A | Comments |
|---|---|---|---|
| **Large Run Size** | Determined to be 120 samples. | | V1872106P5 |
| **Dilution** | | | |
| Dilution factor: 2x | Dilution QC: 150 mcg/mL<br>Precision: 2.8%<br>Accuracy: 5.3% | | V1872106P5 |
| Dilution factor: 5x | Dilution QC: 800 mcg/mL<br>Precision: 4.9%<br>Accuracy: -5.3% | | V1872106P5 |
| **Stability** | | | |
| *Matrix (in polypropylene tubes)* | | | |
| Freeze/Thaw (-20 °C and -70 °C /wet ice) treated with and without the presence of comed[2] | Demonstrated for 4 cycles in treated QCs | | V1872106P5 |
| Freeze/Thaw (-70 °C/wet ice) untreated with and without the presence of comed[2] | Demonstrated for 2 cycles in untreated QCs | | V1872106P5 |
| Bench-top (wet ice) treated with and without the presence of comed[2] | Demonstrated for 8.75 hours in treated QCs | | V1872106P5 |
| Bench-top (wet ice) untreated with and without the presence of comed[2] | Demonstrated for 4 hours in untreated QCs | | V1872106P5 |
| Long-term (-20 °C) treated with and without the presence of comed[2] | Unstable at all durations tested | | V1872106P5 |
| Long-term (-70 °C) treated with and without the presence of comed[2] | Demonstrated for 38 days in treated QCs with comed[2]<br>Demonstrated for 24 days in treated QCs without comed[2] | | V1872106P5 |
| Long-term (-70 °C) treated with and without the presence of comed[2] | Demonstrated for 88 days in treated QCs with comed[2]<br>Demonstrated instability for 181 days in treated QCs with comed[2]<br>Demonstrated for 202 days in treated QCs without comed[2] | | V1872106P5A1 |

## Table 6: Summary of Results and Conclusions (Continued)

| Methodology – TP-80193 | Method Performance – LC-MS/MS | Hyperlink – N/A | Comments |
|---|---|---|---|
| Long-term (-70 °C) untreated with and without the presence of comed[2] | Demonstrated for 29 days in untreated QCs with comed[2]<br>Demonstrated for 28 days in untreated QCs without comed[2] | | V1872106P5 |
| Long-term (-70 °C) untreated with and without the presence of comed[2] | Demonstrated for 47 days in untreated QCs with comed[2]<br>Demonstrated for 47 days in untreated QCs without comed[2] | Summary tables Table 9<br><br>Report text Section 10.4 | |
| *Whole Blood* | No instability observed in ice-water bath or at room temperature in polyethylene terephthalate for up to 1 hour. | | V1872106P5 |
| *Processed Matrix polypropylene* | | | |
| Room Temperature | Demonstrated for 3 days 20 hours | | V1872106P5 |
| Refrigeration | Demonstrated for 3 days 20 hours | | V1872106P5 |
| **Individual Sample Reinjection** | Reproducible | | V1872106P5 |
| **Whole Run Reinjection Integrity** | Reproducible<br>Duration 4 days 4 hours | | V1872106P5 |
| **Column Ruggedness** | Reproducible | | V1872106P5 |
| **Extraction Recovery** | | | |
| Ertapenem | Overall 106.2% | | V1872106P5 |
| Ertapenem-$d_4$ (IS) | Overall 79.2% | | V1872106P5 |
| **Solution Stability** | | | |
| Solution, Primary polypropylene | | | |
| Ertapenem in MES buffer (RT) | Stable 6.75 hours | | V1872106P5 |
| Ertapenem in MES buffer (-20 °C) | Stable 7 days<br>Unstable 29 days | | V1872106P5 |
| Ertapenem-$d_4$ (IS) in MES buffer (RT) | Taken to be same as ertapenem | | |
| Ertapenem-$d_4$ (IS) in MES buffer (-20 °C) | 3 months stability can be assigned per SOP unless low sensitivity is observed | | SOP-00073 |

**Table 7: Summary of Validation Run Objectives and Outcome**

| Run ID | Instrument ID | Extraction Date | Assay Date | Run Objective(s) | Accepted or Rejected | Comment |
|---|---|---|---|---|---|---|
| 1 | LC/MS-22 | 06Dec2023 | 06Dec2023 | Cal, QC, and untreated QC with and without comed[2] qualification | Accepted | This was used as time 0 for untreated long-term stability. |
| 2 | LC/MS-43 | 22Jan2024 | 23Jan2024 | Untreated long-term matrix stability with and without comed[2] | Accepted | |

**Table 8: Carryover**

| Run ID | Result (Extracted Blank after High Standard) | Carryover Results for Internal Standard |
|---|---|---|
| 1 | Seq 13 and Seq 55 are <20.0% of the LLOQ. | Seq 4 and Seq 46 are < 5.0% internal standard peak of acceptable standards. |
| 2 | Seq 13 and Seq 55 are <20.0% of the LLOQ. | Seq 4 and Seq 46 are < 5.0% internal standard peak of acceptable standards. |

Note: Calibration Standards and QCs are arranged in a way to mitigate carryover in all validation runs.

## Table 9: Long-Term Stability at -70 °C - Untreated

Analytical Performance: Back-Calculated Concentrations (µg/mL) of Ertapenem Calibration Standard in (Human) (Plasma) in (Study V1872106P5A2)

| Assay Date | Run ID | Cal 0.200 0.200 µg/mL | Cal 0.400 0.400 µg/mL | Cal 4.00 4.00 µg/mL | Cal 20.0 20.0 µg/mL | Cal 40.0 40.0 µg/mL | Cal 80.0 80.0 µg/mL | Cal 160 160 µg/mL | Cal 200 200 µg/mL |
|---|---|---|---|---|---|---|---|---|---|
| 06-Dec-2023 | 1 | 0.204 | 0.364 | 4.14 | 20.3 | 40.7 | 81.7 | 161 | 196 |
| | | 0.204 | 0.403 | 3.93 | 20.2 | 40.1 | 80.9 | 158 | 195 |
| 23-Jan-2024 | 2 | 0.199 | 0.445 | 4.19 | 20.0 | 40.5 | 76.2 | 160 | 196 |
| | | 0.189 | *0.474 | 4.07 | 19.5 | 39.9 | *38.9 | 159 | 194 |
| Mean | | 0.199 | 0.404 | 4.08 | 20.0 | 40.3 | 79.6 | 160 | 195 |
| S.D. | | 0.00707 | 0.0405 | 0.113 | 0.356 | 0.365 | 2.97 | 1.29 | 0.957 |
| %CV | | 3.6 | 10.0 | 2.8 | 1.8 | 0.9 | 3.7 | 0.8 | 0.5 |
| %Bias | | -0.5 | 1.0 | 2.0 | 0.0 | 0.8 | -0.5 | 0.0 | -2.5 |
| n | | 4 | 3 | 4 | 4 | 4 | 3 | 4 | 4 |
| Reason Deactivated | | | | | | | | | |
| * Excluded due to being outside acceptance criteria | | | | | | | | | |

Calibration Curve Parameters for Ertapenem Calibration Standards in Human Plasma (Study V1872106P5A2)

| Run Date | Run ID | Slope | Intercept | R-Squared | LLOQ | ULOQ |
|---|---|---|---|---|---|---|
| 06-Dec-2023 | 1 | 0.03292 | 0.00061 | 0.9989 | 0.200 | 200 |
| 23-Jan-2024 | 2 | 0.04801 | -0.00136 | 0.9980 | 0.200 | 200 |

Human Plasma Ertapenem Concentrations in Quality Controls for 2 separate batch runs (Study V1872106P5A2)

| Run Date | Run ID | QCS 0.600 0.600 µg/mL | %Bias | QCS 100 100 µg/mL | %Bias | QCS 150 150 µg/mL | %Bias |
|---|---|---|---|---|---|---|---|
| 06-Dec-2023 | 1 | 0.610 | 1.7 | 104 | 4.0 | 145 | -3.3 |
| | | 0.644 | 7.3 | 102 | 2.0 | 149 | -0.7 |
| Intrarun Mean | | 0.627 | | 103 | | 147 | |
| Intrarun %Bias | | 4.5 | | 3.0 | | -2.0 | |
| 23-Jan-2024 | 2 | 0.660 | 10.0 | 104 | 4.0 | 141 | -6.0 |
| | | 0.612 | 2.0 | 99.9 | -0.1 | 156 | 4.0 |
| Intrarun Mean | | 0.636 | | 102 | | 149 | |
| Intrarun %Bias | | 6.0 | | 2.0 | | -0.7 | |

## Table 9: Long-Term Stability at -70 °C – Untreated (Continued)

Untreated QCs with comed[2]

| Run Date | Run ID | QC 0.600 untr comed 0.600 µg/mL | %Bias | QC 150 untr comed 150 µg/mL | %Bias |
|---|---|---|---|---|---|
| 06-Dec-2023 | 1 | 0.603 | 0.5 | 153 | 2.0 |
| (Time 0) | | 0.616 | 2.7 | 150 | 0.0 |
| | | 0.613 | 2.2 | 151 | 0.7 |
| | | 0.640 | 6.7 | 150 | 0.0 |
| | | 0.576 | -4.0 | 148 | -1.3 |
| | | 0.604 | 0.7 | 151 | 0.7 |
| Intrarun Mean | | 0.609 | | 151 | |
| Intrarun SD | | 0.0209 | | 1.64 | |
| Intrarun %CV | | 3.4 | | 1.1 | |
| Intrarun %Bias | | 1.5 | | 0.7 | |
| n | | 6 | | 6 | |
| 23-Jan-2024 | 2 | 0.634 | 5.7 | 150 | 0.0 |
| (47 days) | | 0.622 | 3.7 | 148 | -1.3 |
| | | 0.608 | 1.3 | 142 | -5.3 |
| | | 0.653 | 8.8 | 147 | -2.0 |
| | | 0.615 | 2.5 | 141 | -6.0 |
| | | 0.634 | 5.7 | 139 | -7.3 |
| Intrarun Mean | | 0.628 | | 145 | |
| Intrarun SD | | 0.0161 | | 4.42 | |
| Intrarun %CV | | 2.6 | | 3.0 | |
| Intrarun %Bias | | 4.7 | | -3.3 | |
| n | | 6 | | 6 | |

Untreated QCs without comed[2]

| Run Date | Run ID | QC 0.600 untreated 0.600 µg/mL | %Bias | QC 150 untreated 150 µg/mL | %Bias |
|---|---|---|---|---|---|
| 06-Dec-2023 | 1 | 0.599 | -0.2 | 153 | 2.0 |
| (Time 0) | | 0.613 | 2.2 | 153 | 2.0 |
| | | 0.623 | 3.8 | 155 | 3.3 |
| | | 0.599 | -0.2 | 154 | 2.7 |
| | | 0.611 | 1.8 | 152 | 1.3 |
| | | 0.582 | -3.0 | 153 | 2.0 |
| Intrarun Mean | | 0.605 | | 153 | |
| Intrarun SD | | 0.0143 | | 1.03 | |
| Intrarun %CV | | 2.4 | | 0.7 | |
| Intrarun %Bias | | 0.8 | | 2.0 | |
| n | | 6 | | 6 | |
| 23-Jan-2024 | 2 | 0.615 | 2.5 | 151 | 0.7 |
| (47 days) | | 0.605 | 0.8 | 154 | 2.7 |
| | | 0.607 | 1.2 | 143 | -4.7 |
| | | 0.636 | 6.0 | 148 | -1.3 |
| | | 0.648 | 8.0 | 150 | 0.0 |
| | | 0.659 | 9.8 | 147 | -2.0 |
| Intrarun Mean | | 0.628 | | 149 | |
| Intrarun SD | | 0.0226 | | 3.76 | |
| Intrarun %CV | | 3.6 | | 2.5 | |
| Intrarun %Bias | | 4.7 | | -0.7 | |
| n | | 6 | | 6 | |

Criteria: See Section 10.4.

Results: Ertapenem is stable in untreated human plasma with and without the presence of comed[2], with sodium heparin as anticoagulant, for up to 47 days upon storage in polypropylene tubes at -70 °C.

# 1. TITLE: THE DETERMINATION OF TOTAL ERTAPENEM IN TREATED HUMAN PLASMA, WITH SODIUM HEPARINAS ANTICOAGULANT, BY LC-MS/MS

## BIOANALYTICAL METHOD VALIDATION REPORT

| | |
|---|---|
| Bioanalytical Project Number: | V1872106P5 |
| Sponsor: | DynPort Vaccine Company, LLC<br>110 Thomas Johnson Drive<br>Suite 200<br>Frederick, MD 21702 |
| Bioanalytical Laboratory: | KCAS Bioanalytical & Biomarker Services.<br>10830 S Clay Blair Boulevard<br>Olathe, KS 66061 |
| Issue Date: | March 2023 |
| Amendment 1 Issue Date: | Date of PI Signature |

# 2. SYNOPSIS

This report presents the validation data for an LC-MS/MS method for the determination of total ertapenem in treated human plasma, with sodium heparin as anticoagulant. The assay was analyzed in the range of 0.200 - 200 mcg/mL and is for use on human plasma samples obtained during clinical trials.

**AMENDMENT OUTLINE FORM**

| | |
|---|---|
| 1. Amendment Number 1 | This amendment was created to report:<br>• Long-term matrix stability at -70 °C for up to 60, 88, and 181 days (with comed[2]) and 81, 109, and 202 days (without comed[2]) for total ertapenem in treated human plasma, with sodium heparin as anticoagulant with and without the presence of comed[2]. |

# 3. TABLE OF CONTENTS FOR THE BIOANALYTICAL METHOD VALIDATION REPORT

1. TITLE: THE DETERMINATION OF TOTAL ERTAPENEM IN TREATED HUMAN PLASMA, WITH SODIUM HEPARINAS ANTICOAGULANT, BY LC-MS/MS ........ 1
2. SYNOPSIS ........ 2
3. TABLE OF CONTENTS FOR THE BIOANALYTICAL METHOD VALIDATION REPORT ........ 3
4. LIST OF TABLES ........ 4
5. SIGNATURE PAGE ........ 5
6. QUALITY ASSURANCE STATEMENT ........ 6
7. STUDY COMPLIANCE STATEMENT ........ 7
8. LIST OF ABBREVIATIONS AND DEFINITIONS OF TERMS ........ 8
9. INTRODUCTION ........ 10
10. EXPERIMENTAL DESIGN AND RESULTS ........ 10
10.1. Reference Standards ........ 10
10.2. Calibration Standards and QC Samples Preparation ........ 10
10.2.1. Carryover ........ 11
10.3. Long-Term Matrix Stability at -70 °C ........ 11
10.4. SOP Deviations and Investigations ........ 11
11. CONCLUSION ........ 11
12. PERTINENT SOPS ........ 12
13. ARCHIVES ........ 12
14. REFERENCES ........ 12
15. TABLES ........ 13

# 4. LIST OF TABLES

Table 1: Abbreviations and Codes ........ 8
Table 2: Deviations ........ 11
Table 3: Mass Spec Transitions ........ 13
Table 4: Reference Standards ........ 13
Table 5: Calibration Standards and QC Samples Preparation ........ 14
Table 6: Matrix Description ........ 14
Table 7: Summary of Results and Conclusions ........ 15
Table 8: Summary of Validation Run Objectives and Outcome ........ 18
Table 9: Carryover ........ 18
Table 10: Long-Term Stability at -70 °C - Treated ........ 19

Annex 4 ........ Certificate(s) of Analysis
Annex 5 ........ DEV-2023-0088

# 5. SIGNATURE PAGE

Bioanalytical Project Number: V1872106P5

Amendment 1 Issue Date: Date of PI Signature

**APPROVED**

DocuSigned by:
Anika Pippin
Signer Name: Anika Pippin
Signing Reason: I approve this document
Signing Time: 06-Oct-2023 | 12:19 CDT
BE3AA1198E40459E8A7E3C615D981D82

Anika Pippin, B.S.
Principal Investigator

**REVIEWED**

DocuSigned by:
Marsha Luna
Signer Name: Marsha Luna
Signing Reason: I have reviewed this document
Signing Time: 06-Oct-2023 | 12:01 CDT
C9CC738561AF4CC1B375ED013DD1E4BD

Marsha Luna, B.S.
Management

# 6. QUALITY ASSURANCE STATEMENT

This Bioanalytical Method Validation Report has been found to describe accurately the methods and SOPs used and to accurately reflect the raw data collected. The Quality Assurance Unit initiated audits on this study and reported the findings to the Principal Investigator (PI) and Management as follows:

| | | Date(s) Sent to: | |
|---|---|---|---|
| **Inspection Date(s)** | **Inspection Phase** | **PI** | **Management** |
| 24May2023 | Data Audit | 24May2023 | 04Aug2023 |
| 03Jul2023 | Data Audit | 03Jul2023 | 03Jul2023 |
| 18Sep2023 | Data Audit | 18Sep2023 | 18Sep2023 |
| 19Sep2023 | Draft Report Audit A1 | 19Sep2023 | 21Sep2023 |
| 06Oct2023 | Final Report Review A1 | 06Oct2023 | 06Oct2023 |

**REVIEWED**

DocuSigned by:

Signer Name: Jenae Hayes
Signing Reason: I have reviewed this document
Signing Time: 06-Oct-2023 | 09:58 PDT
E87EF929504341808E814ABD7C1FD695

Jenae' Hayes BA
Quality Assurance Unit

# 7. STUDY COMPLIANCE STATEMENT

I, the undersigned, hereby declare that this study was conducted in compliance with the relevant bioanalytical aspects of FDA GLP regulations 21CFR Part 58, according to the FDA Guidance for Bioanalytical Method Validation and KCAS Bioanalytical & Biomarker Services (KCAS) procedures and that the work was performed by me, or under my direction, and the report reflects a true and accurate record of the results obtained.

The acceptance criteria are based on the FDA Guidance for Industry, Bioanalytical Method Validation (2018). [1]

**APPROVED**

DocuSigned by:
Anika Pippin
Signer Name: Anika Pippin
Signing Reason: I approve this document
Signing Time: 06-Oct-2023 | 12:19 CDT
BE3AA1198E40459E8A7E3C615D981D82

Anika Pippin, B.S.
Principal Investigator

# 8. LIST OF ABBREVIATIONS AND DEFINITIONS OF TERMS

**Table 1: Abbreviations and Codes**

| Abbreviation | Definition |
|---|---|
| < | Concentration less than the lowest acceptable calibration standard |
| > | Concentration exceeded the upper limit of calibration range (> ULOQ) |
| A | Amendment |
| AQL | Above Quantitation Limit |
| %Bias | Percentage measurement valid |
| Batch | Is the same as Run |
| BQL | Below Quantifiable Limit |
| Cal | Calibration standard |
| Conc. | Concentration |
| Conv. | Conversion |
| %CV | Coefficient of Variation |
| dev | Deviation |
| diff | Difference |
| EMA | European Medicines Agency |
| FDA | Food and Drug Administration |
| GLP | Good Laboratory Practice |
| HPLC | High Performance Liquid Chromatography |
| ID | Identification |
| IS | Internal Standard |
| ISR | Incurred Sample Reanalysis |
| $K_2$EDTA or $K_3$EDTA | Dipotassium or Tripotassium Ethylenediaminetetraacetic Acid |
| KCAS | KCAS Bioanalytical & Biomarker Services |
| LC-MS/MS | Liquid Chromatography Tandem Mass Spectrometry |
| LLOQ | Lower Limit of Quantitation |
| mcg or µg or ug | Microgram |
| mcL or µL or uL | Microliter |

**Table 1: Abbreviations and Codes (Continued)**

| Abbreviation | Definition |
|---|---|
| ME | Matrix Effect |
| MF | Matrix Factor |
| MHLW | Ministry of Health, Labour and Welfare of Japan |
| mL | Milliliter |
| MS | Mass Spectrometry |
| n | number |
| N.C. | Not Calculated |
| ND | No difference reported as ISR will be counted |
| ng | nanogram |
| n.report | No Data available to report |
| N/A or NA | Not Applicable |
| N.R. | Not Reported |
| P&A | Precision and Accuracy |
| p.a.r. | Peak Area Ratio |
| PI | Principal Investigator |
| QC | Quality Control Sample |
| QCs | Quality Control Samples |
| OECD | Organization for Economic Co-operation and Development |
| RT | Room Temperature |
| sd or SD | Standard deviation |
| Seq | Sequence |
| SOP (s) | Standard Operating Procedure (s) |
| temp | Temperature |
| TP | Test procedure |
| UHPLC | Ultra High Performance Liquid Chromatography |
| ULOQ | Upper Limit of Quantitation |
| WIS | Working Internal Standard |

This is a basic list. Some of the abbreviations may not have been used in this report.

# 9. INTRODUCTION

This amendment was created to report long-term matrix stability at -70 °C for up to 60, 88, and 181 days (with comed[2]) and 81, 109, and 202 days (without comed[2]) for total ertapenem in treated human plasma, with sodium heparin as anticoagulant with and without the presence of comed[2].

The validation procedure was designed to meet or exceed the requirements of FDA Guidance for Industry on Bioanalytical Methods Validation, May 2018. The software programs used were Microsoft Office Excel 365, acquisition and processing software Analyst 1.7.2, and data management software Watson LIMS 7.5.

# 10. EXPERIMENTAL DESIGN AND RESULTS

In this method, ertapenem and internal standards, ertapenem-$d_4$, were released from treated human plasma using 96 well protein precipitation. After extraction, the sample was chromatographed using reversed phase HPLC with a Fortis C18 analytical column. Ertapenem and ertapenem-$d_4$ (IS) were detected by monitoring the precursor and product ions described in Table 3 using an Applied Biosystems Sciex API 5000 LC-MS/MS. The quantification was based on setting a calibration graph using the internal standard method based on bioanalytical test procedure TP-80193, entitled "LC-MS/MS Analysis of Ertapenem in Human Sodium Heparin Plasma (100 mcL, 0.200 - 200 mcg/mL; Prior to MES Treatment)". The test procedure can be found in Annex 1 (V1872106P5).

Human plasma is treated with 0.1M 2-(N-Morpholino) ethane sulphonic acid hydrate (MES) buffer, pH 6.4 at a 1:1 ratio. The validation assay range is calculated based on plasma volume only to mimic unknown study samples. The assay range is 0.200 to 200 mcg/mL in untreated human sodium heparin plasma.

A summary of the validation results is provided in Table 7. A summary describing the objective(s) and outcome of each validation run attempted is provided in Table 8.

The method was validated within the analytical range of 0.200 - 200 mcg/mL in human plasma. No development work was performed after the method was validated.

## 10.1. Reference Standards

Reference standards used during this validation are presented in Table 4.

## 10.2. Calibration Standards and QC Samples Preparation

Calibration standards and quality control pools were prepared by spiking appropriate levels of ertapenem into treated human plasma, with sodium heparin as anticoagulant. Primary reference standards were prepared in at least duplicate, and they need to compare within 5% of each other. Standards and QCs were prepared and stored frozen at KCAS at -70 °C. Stability QCs were also

stored at -70 °C. Standards, QCs, and the critical solutions used during this validation are described in Table 5, and matrix used is described in Table 6.

### 10.2.1. Carryover

Carryover is evaluated within each run. It consists of measuring the response for each analyte and if it exceeds 20.0% of the response of LLOQ standard or other sample reflecting the lower sensitivity limit required, a carryover assessment is completed, and affected samples are rejected. The internal standard carryover evaluation is assessed within each run and if it exceeds 5.0% of the average internal standard response of acceptable standards and QCs, impact is evaluated within the run. Data is presented in Table 9.

## 10.3. Long-Term Matrix Stability at -70 °C

To evaluate ertapenem in treated human plasma with and without the presence of comed[2], with sodium heparin as anticoagulant, stability during sample storage, aliquots of the high and low concentration QC samples were stored frozen at -70 °C nominally for 60, 88, and 181 days (with comed[2]) and 81, 109, and 202 days (without comed[2]) and prior to being analyzed, along with freshly prepared standards and QC samples. To be deemed stable, the mean %Bias at each level must be within ± 15% with %CV ≤ 15%.

Data are provided in Table 10.

## 10.4. SOP Deviations and Investigations

One SOP deviation occurred, and the description and impact are presented in Table 2. No investigations were conducted during this study. The deviation can be found in Annex 5.

**Table 2: Deviations**

| Deviation # | Description | Impact |
|---|---|---|
| DEV-2023-0088 | The treated comed QC low concentration used in run V1872106P5A1 run 1 for the 60-day stability show %Bias > 15%. This is due to the Time 0 %Bias for the low QC being on the high side (tested in V1872106P5 run 18.) with 11.0%. The %change between Time 0 and 60-day long-term matrix stability data is 9.5% which is within the %Bias acceptance criteria. The sponsor has requested previously that these comed QCs be used for future long-term matrix stability testing, and that both %Bias from nominal and from Time 0 be reported. The stability will be accepted as passing since it is within 15% of the original preparation. | There is no impact on stability since data is only considered acceptable if it is within 15% of the Time 0 data (or within 15% bias from nominal per SOP). |

# 11. CONCLUSION

Ertapenem is stable in treated human plasma with the presence of comed[2], with sodium heparin as anticoagulant, for up to 88 days upon storage in polypropylene tubes at -70 °C.

Ertapenem is stable in treated human plasma without the presence of comed[2], with sodium heparin as anticoagulant, for up to 202 days upon storage in polypropylene tubes at -70 °C.

## 12. PERTINENT SOPS

The following SOPs were followed during the conduct of this study:

SOP-00073 [04] - Bioanalytical Method Validation - Chromatographic Assays

SOP-00035 [02] - Procedures, Reporting, and Acceptance Criteria for Bioanalytical Chromatographic Projects

## 13. ARCHIVES

All raw data, including paper and electronic files associated with this study will remain archived at a location specified by KCAS, 10830 S Clay Blair Boulevard, Olathe, Kansas 66061 for a period of at least 10 years, after which the client may be contacted to arrange for continued storage of the files, or disposition according to an agreed procedure. KCAS will maintain an electronic (scanned/true) copy of the method validation report that can be viewed and printed on site. The study plan, documentation, raw data, and final report are indexed under Bioanalytical Project Number V1872106P5A1.

## 14. REFERENCES

1. Guidance for Industry: Bioanalytical Method Validation. U.S. Department of Health and Human Services. Food and Drug Administration. Center for Drug Evaluation and Research. Center for Veterinary Medicine. May 2018.
2. Comed: QCs in the presence of 37.5 mcg/mL zidebactam.

# 15. TABLES

## Table 3: Mass Spec Transitions

| Analyte | Transition |
|---|---|
| Ertapenem | m/z 476.047→431.9 |
| Ertapenem-$d_4$ (IS) | m/z 480.2→436.3 |

## Table 4: Reference Standards

| Analyte | Supplier | Lot/Batch Number | Purity | Expiration Date or Retest Date` | Conversion Factor |
|---|---|---|---|---|---|
| Ertapenem Sodium | Med Chem Express | 180017 | 98.9% | Retest 11 Aug 2024 | 1.046 |
| | Med Chem Express | 229333 | 98.43% | Retest 29 Sep 2025 | 1.046 |
| Ertapenem-$d_4$ Sodium (IS) | AlsaChim | DH-ALS-20-002-P2 | * | Retest 26 Oct 2023 | * |
| Zidebactam | Wockhardt | WK1/WP-0060/016 | 90.4% | Expire 29 Mar 2023 | 1.000 |

Certificates of analysis for the above reference standards are presented in Annex 4.
*Used as is with 1.000 conversion and 100% purity as IS only.

## Table 5: Calibration Standards and QC Samples Preparation

| Critical Solutions | Lot # for Standards (Prep Date) | Lot # for QCs (Prep Date) | Standards Lot (Prep Date) | QC Lot (Prep Date) | Runs used |
|---|---|---|---|---|---|
| Treated Human Plasma (1:1 MES Buffer:Plasma) | V1872106P1-18-2 | | N/A | V1872106P5-60-1 Set E (16 Dec 2022) | 1, 2, 3 (all LTS) |
| Chilled ~0.1M MES Buffer stored at -20°C in glass | N/A | V1872106P5-52-1 (15 Dec 2022) | | | |
| Treated Human Plasma (1:1 MES Buffer:Plasma) stored at -70°C in polypropylene | N/A | V1872106P5-58-2 (16 Dec 2022) | | | |
| Treated Human Plasma (1:1 MES Buffer:Plasma) | V1872106P2-108-2 | | NA | Treated Comed QCs V1872106P2-109-1 (06 Jan 2023) | 1, 2, 3 (all LTS) |
| Chilled ~0.1M MES Buffer stored at -20°C in polypropylene | NA | V1872106P5-69-1 (03 Jan 2023) | | | |
| Zidebactam in deionized water stored at -20 °C in polypropylene | NA | V1872106P2-98-1 (03 Jan 2023) | | | |
| Treated Human Plasma (1:1 MES Buffer:Plasma) | V1872106P5A1-7-1 | | V1872106P5A1-9-1 Set A (07 Mar 2023) | V1872106P5A1-10-1 Set A (07 Mar 2023) | 1 |
| Chilled ~0.1M MES Buffer stored at -20°C in polypropylene | V1872106P5A1-1-1 (01 Mar 2023) | V1872106P5A1-2-1 (01 Mar 2023) | | | |
| Treated Human Plasma (1:1 MES Buffer:Plasma) stored at -70°C in polypropylene | V1872106P5A1-8-1 (07 Mar 2023) | V1872106P5A1-8-2 (07 Mar 2023) | | | |
| Treated Human Plasma (1:1 MES Buffer:Plasma) | V1872106P5-134-2 | | V1872106P5A1-19-1 Set B (04 Apr 2023) | V1872106P5A1-20-1 Set B (04 Apr 2023) | 2 |
| Chilled ~0.1M MES Buffer stored at -20°C in polypropylene | V1872106P5A1-11-1 (03 Apr 2023) | V1872106P5A1-12-1 (03 Apr 2023) | | | |
| Treated Human Plasma (1:1 MES Buffer:Plasma) stored at -70°C in polypropylene | V1872106P5A1-18-1 (04 Apr 2023) | V1872106P5A1-18-2 (04 Apr 2023) | | | |
| Treated Human Plasma (1:1 MES Buffer:Plasma) | V1872106P5A1-27-1 | | V1872106P5A1-28-1 Set C (06 Jul 2023) | V1872106P5A1-29-1 Set C (06 Jul 2023) | 3 |
| Chilled ~0.1M MES Buffer stored at -20°C in polypropylene | V1872106P5A1-21-1 (05 Jul 2023) | V1872106P5A1-21-1 (05 Jul 2023) | | | |
| Treated Human Plasma (1:1 MES Buffer:Plasma) stored at -70°C in polypropylene | V1872106P5A1-27-2 (06 Jul 2023) | V1872106P5A1-27-2 (06 Jul 2023) | | | |

## Table 6: Matrix Description

| Matrix | Source Lot # | Date Received |
|---|---|---|
| Treated Human Plasma (1:1 MES Buffer:Plasma) V1872106P2-108-2 | BioIVT HMN444008 | Aug2020 |
| Treated Human Plasma (1:1 MES Buffer Plasma) V1872106P1-18-2 | BioIVT, Combo-43-1 Lots HMN769588, HMN769589, HMN769590, HMN769592, HMN769593, HMN769594, HMN769595, HMN769596, HMN769597, HMN769598, HMN769600, HMN769601, HMN769602, HMN769604, HMN769605 | Mar 2022 |
| Treated Human Plasma (1:1 MES Buffer:Plasma) V1872106P5A1-27-1 | BioChemed, Lot BC43938HP-5 | Feb 2023 |
| Treated Human Plasma (1:1 MES Buffer:Plasma) V1872106P5-134-2 | BioChemed, Lot BC43938HP-9 | Feb 2023 |
| Treated Human Plasma (1:1 MES Buffer:Plasma) V1872106P5A1-7-1 | BioIVT, Lot HMN795695 | Apr 2022 |

## Table 7: Summary of Results and Conclusions

| **Methodology – TP-80193** | **Method Performance – LC-MS/MS** | **Hyperlink – N/A** | **Comments** |
|---|---|---|---|
| Matrix | Plasma | | |
| Anticoagulant | Sodium heparin | | |
| Assay Volume | 100 mcL | | |
| **Selectivity** | | | |
| Analyte | Ertapenem | | V1872106P5 |
| Internal Standard | Ertapenem-$d_4$ | | V1872106P5 |
| Matrix Effect (6 spiked matrix lots) | Complies | | V1872106P5 |
| Interference | Complies | | V1872106P5 |
| Hemolytic Effect Evaluation | Complies for 0.5% hemolysis<br>Does not comply for 1% and 2% hemolysis | | V1872106P5 |
| Lipemic Effect Evaluation | Complies | | V1872106P5 |
| Carryover | Assessed with every run with standards and QCs | Summary tables Table 9<br><br>Report text Section 10.2.1 | |
| **Linearity** | | | |
| Weighting | $1/x^2$ | | V1872106P5 |
| Bias at LLOQ | 0.0% | | V1872106P5 |
| Bias above LLOQ | -3.3 to 4.4% | | V1872106P5 |
| Bioanalytical Range | Complies<br>0.200 - 200 mcg/mL | | V1872106P5 |
| **Precision** | | | |
| Intra-assay | 5.0 to 18.0% (LLOQ);<br>3.4 to 13.6% (above LLOQ) | | V1872106P5 |
| Inter-assay | 12.5% (LLOQ);<br>7.4 to 10.0% (above LLOQ) | | V1872106P5 |
| **Accuracy** | | | |
| Intra-assay | -9.5 to 10.0% (LLOQ);<br>-7.3 to 11.3% (above LLOQ) | | V1872106P5 |
| Inter-assay | 4.0% (LLOQ);<br>-1.3 to 1.8% (above LLOQ) | | V1872106P5 |
| **Large Run Size** | Determined to be 120 samples. | | V1872106P5 |

## Table 7: Summary of Results and Conclusions (Continued)

| **Methodology – TP-80193** | **Method Performance – LC-MS/MS** | **Hyperlink – N/A** | **Comments** |
|---|---|---|---|
| **Dilution** | | | |
| Dilution factor: 2x | Dilution QC: 150 mcg/mL<br>Precision: 2.8%<br>Accuracy: 5.3% | | V1872106P5 |
| Dilution factor: 5x | Dilution QC: 800 mcg/mL<br>Precision: 4.9%<br>Accuracy: -5.3% | | V1872106P5 |
| **Stability** | | | |
| *Matrix (in polypropylene tubes)* | | | |
| Freeze/Thaw (-20 °C and -70 °C /wet ice) treated with and without the presence of comed[2] | Demonstrated for 4 cycles in treated QCs | | V1872106P5 |
| Freeze/Thaw (-70 °C/wet ice) untreated with and without the presence of comed[2] | Demonstrated for 2 cycles in untreated QCs | | V1872106P5 |
| Bench-top (wet ice) treated with and without the presence of comed[2] | Demonstrated for 8.75 hours in treated QCs | | V1872106P5 |
| Bench-top (wet ice) untreated with and without the presence of comed[2] | Demonstrated for 4 hours in untreated QCs | | V1872106P5 |
| Long-term (-20 °C) treated with and without the presence of comed[2] | Unstable at all durations tested | | V1872106P5 |
| Long-term (-70 °C) treated with and without the presence of comed[2] | Demonstrated for 38 days in treated QCs with comed[2]<br>Demonstrated for 24 days in treated QCs without comed[2] | | V1872106P5 |
| Long-term (-70 °C) treated with and without the presence of comed[2] | Demonstrated for 88 days in treated QCs with comed[2]<br>Demonstrated instability for 181 days in treated QCs with comed[2]<br>Demonstrated for 202 days in treated QCs without comed[2] | Summary tables Table 10<br><br>Report text Section 10.3 | |
| Long-term (-70 °C) untreated with and without the presence of comed[2] | Demonstrated for 29 days in untreated QCs with comed[2]<br>Demonstrated for 28 days in untreated QCs without comed[2] | | V1872106P5 |
| *Whole Blood* | No instability observed in ice-water bath or at room temperature in polyethylene terephthalate for up to 1 hour. | | V1872106P5 |
| *Processed Matrix polypropylene* | | | |
| Room Temperature | Demonstrated for 3 days 20 hours | | V1872106P5 |

## Table 7: Summary of Results and Conclusions (Continued)

| **Methodology – TP-80193** | **Method Performance – LC-MS/MS** | **Hyperlink – N/A** | **Comments** |
|---|---|---|---|
| Refrigeration | Demonstrated for 3 days 20 hours | | V1872106P5 |
| **Individual Sample Reinjection** | Reproducible | | V1872106P5 |
| **Whole Run Reinjection Integrity** | Reproducible<br>Duration 4 days 4 hours | | V1872106P5 |
| **Column Ruggedness** | Reproducible | | V1872106P5 |
| **Extraction Recovery** | | | |
| Ertapenem | Overall 106.2% | | V1872106P5 |
| Ertapenem-$d_4$ (IS) | Overall 79.2% | | V1872106P5 |
| **Solution Stability** | | | |
| Solution, Primary polypropylene | | | |
| Ertapenem in MES buffer (RT) | Stable 6.75 hours | | V1872106P5 |
| Ertapenem in MES buffer (-20 °C) | Stable 7 days<br>Unstable 29 days | | V1872106P5 |
| Ertapenem-$d_4$ (IS) in MES buffer (RT) | Taken to be same as ertapenem | SOP 00073 | |
| Ertapenem-$d_4$ (IS) in MES buffer (-20 °C) | 1 year stability can be assigned and evaluated thereafter in yearly increments | SOP 00073 | |

## Table 8: Summary of Validation Run Objectives and Outcome

| Run ID | Instrument ID | Extraction Date | Assay Date | Run Objective(s) | Accepted or Rejected | Comment |
|---|---|---|---|---|---|---|
| 1 | LC/MS-23 | 07-Mar-2023 | 07-Mar-2023 | Treated long-term matrix stability with and without comed[2] | Accepted [a] | |
| 2 | LC/MS-39 | 04-Apr-2023 | 06-Apr-2023 | Treated long-term matrix stability with and without comed[2] | Accepted [a] | |
| 3 | LC/MS-22 | 06-Jul-2023 | 06-Jul-2023 | Treated long-term matrix stability with and without comed[2] | Accepted [b] | |

[a] Per sponsor low comed QCs that showed higher %Bias (11%) were used for LTS assessment and per request %Bias and %Change to Time 0 data will both be reported to show stability. The high comed QCs meets all criteria. The low comed QC shows %Bias > 15% however it is only a %Change to the Time 0 data (in V1872106P5 Run 18) and the comed QC is considered stable. This is addressed in deviation DEV-2023-0088.

[b] Treated comed -70 °C long-term matrix stability high QC met acceptance criteria for all 6 replicates. Treated comed -70 °C long-term matrix stability low QC did not meet acceptance criteria at 181 days due to %Change >15% from Time 0.

## Table 9: Carryover

| Run ID | Result (Extracted Blank after High Standard) | Carryover Results for Internal Standard |
|---|---|---|
| 1 | Seq 13 and Seq 56 are <20.0% of the LLOQ. | Seq 4 and Seq 47 are < 5.0% internal standard peak of acceptable standards. |
| 2 | Seq 13 and Seq 56 are <20.0% of the LLOQ. | Seq 4 and Seq 47 are < 5.0% internal standard peak of acceptable standards. |
| 3 | Seq 13 and Seq 56 are <20.0% of the LLOQ. | Seq 4 and Seq 47 are < 5.0% internal standard peak of acceptable standards. |

Note: Calibration Standards and QCs are arranged in a way to mitigate carryover in all validation runs.

## Table 10: Long-Term Stability at -70 °C - Treated

Analytical Performance: Back-Calculated Concentrations (µg/mL) of Ertapenem Calibration Standard in (Human) (Plasma) in (Study V1872106P5A1)

| Assay Date | Run ID | Cal 0.200 0.200 µg/mL | Cal 0.400 0.400 µg/mL | Cal 4.00 4.00 µg/mL | Cal 20.0 20.0 µg/mL | Cal 40.0 40.0 µg/mL | Cal 80.0 80.0 µg/mL | Cal 160 160 µg/mL | Cal 200 200 µg/mL |
|---|---|---|---|---|---|---|---|---|---|
| 07-Mar-2023 | 1 | 0.206 | 0.414 | 3.72 | 20.7 | 41.4 | 81.9 | 159 | 195 |
| | | 0.195 | 0.385 | 3.98 | 20.2 | 40.5 | 81.7 | 156 | 199 |
| 06-Apr-2023 | 2 | 0.187 | 0.437 | 4.27 | 21.3 | 40.2 | 80.1 | 157 | 182 |
| | | 0.195 | 0.431 | 4.13 | 20.4 | 40.4 | 77.0 | 150 | 185 |
| 06-Jul-2023 | 3 | 0.200 | 0.408 | 4.37 | 21.2 | 40.8 | 79.4 | 157 | 195 |
| | | 0.196 | 0.403 | 4.06 | 20.7 | 40.2 | 76.2 | 147 | 188 |
| Mean | | 0.197 | 0.413 | 4.09 | 20.8 | 40.6 | 79.4 | 154 | 191 |
| S.D. | | 0.00628 | 0.0190 | 0.229 | 0.432 | 0.458 | 2.37 | 4.72 | 6.65 |
| %CV | | 3.2 | 4.6 | 5.6 | 2.1 | 1.1 | 3.0 | 3.1 | 3.5 |
| %Bias | | -1.5 | 3.3 | 2.3 | 4.0 | 1.5 | -0.8 | -3.8 | -4.5 |
| n | | 6 | 6 | 6 | 6 | 6 | 6 | 6 | 6 |

Calibration Curve Parameters for Ertapenem Calibration Standards in Human Plasma (Study V1872106P5A1)

| Run Date | Run ID | Slope | Intercept | R-Squared | LLOQ | ULOQ |
|---|---|---|---|---|---|---|
| 07-Mar-2023 | 1 | 0.02040 | -0.00032 | 0.9989 | 0.200 | 200 |
| 06-Apr-2023 | 2 | 0.04553 | 0.00091 | 0.9960 | 0.200 | 200 |
| 06-Jul-2023 | 3 | 0.05849 | 0.00113 | 0.9977 | 0.200 | 200 |

Human Plasma Ertapenem Concentrations in Quality Controls for 3 separate batch runs (Study V1872106P5A1)

| Run Date | Run ID | QCS 0.600 0.600 µg/mL | %Bias | QCS 100 100 µg/mL | %Bias | QCS 150 150 µg/mL | %Bias |
|---|---|---|---|---|---|---|---|
| 07-Mar-2023 | 1 | 0.614 | 2.3 | 100 | 0.0 | 148 | -1.3 |
| | | 0.590 | -1.7 | 104 | 4.0 | 154 | 2.7 |
| Intrarun Mean | | 0.602 | | 102 | | 151 | |
| Intrarun %Bias | | 0.3 | | 2.0 | | 0.7 | |
| 06-Apr-2023 | 2 | 0.654 | 9.0 | 97.0 | -3.0 | 139 | -7.3 |
| | | 0.619 | 3.2 | 97.5 | -2.5 | 144 | -4.0 |
| Intrarun Mean | | 0.637 | | 97.3 | | 142 | |
| Intrarun %Bias | | 6.2 | | -2.7 | | -5.3 | |
| 06-Jul-2023 | 3 | 0.613 | 2.2 | 95.4 | -4.6 | 139 | -7.3 |
| | | 0.579 | -3.5 | 92.3 | -7.7 | 145 | -3.3 |
| Intrarun Mean | | 0.596 | | 93.9 | | 142 | |
| Intrarun %Bias | | -0.7 | | -6.1 | | -5.3 | |

## Table 10: Long-Term Stability at -70 °C – Treated (Continued)

Human Plasma Ertapenem Concentrations in Quality Controls for 1 separate batch runs (Study V1872106P5)

| Run Date | Run ID | Low Comed Time 0 data (from Run 18) |
|---|---|---|
| 09-Jan-2023 | 18 | 0.663 |
| | | # 0.695 |
| | | 0.660 |
| | | 0.656 |
| | | 0.657 |
| | | 0.666 |
| Intrarun Mean | | 0.666 |
| Intrarun SD | | 0.0146 |
| Intrarun %CV | | 2.2 |
| Intrarun %Bias | | 11.0 |
| n | | 6 |

Treated QCs with comed[2]

| Run Date | Run ID | Comed1 LTS -70 low 0.600 µg/mL | %Bias | Comed1 LTS -70 high 150 µg/mL | %Bias |
|---|---|---|---|---|---|
| 07-Mar-2023 | 1 | #0.716 | 19.3 | 169 | 12.7 |
| | | #0.711 | 18.5 | 167 | 11.3 |
| | | #0.744 | 24.0 | 167 | 11.3 |
| | | #0.730 | 21.7 | 164 | 9.3 |
| | | #0.746 | 24.3 | 170 | 13.3 |
| | | *0.922 | | 166 | 10.7 |
| **Without Outlier** Intrarun Mean | | 0.729 | | 167 | |
| Intrarun SD | | 0.0159 | | 2.14 | |
| Intrarun %CV | | 2.2 | | 1.3 | |
| Intrarun %Bias | | 21.5 | | 11.3 | |
| n | | 5 | | 6 | |
| %Change to Time 0 | | 9.5 | | | |
| **With Outlier** Intrarun Mean | | 0.762 | | | |
| Intrarun SD | | 0.0799 | | | |
| Intrarun %CV | | 10.5 | | | |
| Intrarun %Bias | | 27.0 | | | |
| n | | | | | |
| 06-Apr-2023 | 2 | #0.692 | 15.3 | 155 | 3.3 |
| | | #0.704 | 17.3 | 165 | 10.0 |
| | | #0.700 | 16.7 | 163 | 8.7 |
| | | #0.701 | 16.8 | 160 | 6.7 |
| | | #0.693 | 15.5 | 157 | 4.7 |
| | | #0.762 | 27.0 | 164 | 9.3 |
| Intrarun Mean | | 0.709 | | 161 | |
| Intrarun SD | | 0.0265 | | 4.03 | |
| Intrarun %CV | | 3.7 | | 2.5 | |
| Intrarun %Bias | | 18.2 | | 7.3 | |
| n | | 6 | | 6 | |
| %Change to Time 0 | | 6.5 | | | |
| 06-Jul-2023 | 3 | #0.499 | -16.8 | 145 | -3.3 |
| | | #0.273 | -54.5 | 142 | -5.3 |
| | | #0.363 | -39.5 | 141 | -6.0 |
| | | #0.239 | -60.2 | 143 | -4.7 |
| | | 0.634 | 5.7 | 146 | -2.7 |
| | | #0.864 | 44.0 | 141 | -6.0 |
| Intrarun Mean | | 0.479 | | 143 | |
| Intrarun SD | | 0.239 | | 2.10 | |
| Intrarun %CV | | 49.9 | | 1.5 | |
| Intrarun %Bias | | -20.2 | | -4.7 | |
| n | | 6 | | 6 | |
| %Change to Time 0 | | -28.1 | | | |
| Reason Deactivated | | | | | |
| * Statistical Outlier (t-test, p<0.01) | | | | | |
| # > 15%Bias | | | | | |

## Table 10: Long-Term Stability at -70 °C – Treated (Continued)

Treated QCs without comed[2]

| Run Date | Run ID | LTS -70 low 0.600 µg/mL | %Bias | LTS -70 high 150 µg/mL | %Bias |
|---|---|---|---|---|---|
| 07-Mar-2023 | 1 | 0.680 | 13.3 | 159 | 6.0 |
| | | 0.684 | 14.0 | 165 | 10.0 |
| | | 0.627 | 4.5 | 158 | 5.3 |
| | | 0.690 | 15.0 | 159 | 6.0 |
| | | #0.704 | 17.3 | 166 | 10.7 |
| | | 0.651 | 8.5 | 160 | 6.7 |
| Intrarun Mean | | 0.673 | | 161 | |
| Intrarun SD | | 0.0284 | | 3.43 | |
| Intrarun %CV | | 4.2 | | 2.1 | |
| Intrarun %Bias | | 12.2 | | 7.3 | |
| n | | 6 | | 6 | |
| 06-Apr-2023 | 2 | 0.601 | 0.2 | 151 | 0.7 |
| | | 0.638 | 6.3 | 157 | 4.7 |
| | | 0.598 | -0.3 | 155 | 3.3 |
| | | 0.634 | 5.7 | 155 | 3.3 |
| | | 0.633 | 5.5 | 155 | 3.3 |
| | | 0.651 | 8.5 | 157 | 4.7 |
| Intrarun Mean | | 0.626 | | 155 | |
| Intrarun SD | | 0.0214 | | 2.19 | |
| Intrarun %CV | | 3.4 | | 1.4 | |
| Intrarun %Bias | | 4.3 | | 3.3 | |
| n | | 6 | | 6 | |
| 06-Jul-2023 | 3 | 0.547 | -8.8 | 141 | -6.0 |
| | | 0.545 | -9.2 | 137 | -8.7 |
| | | 0.562 | -6.3 | 135 | -10.0 |
| | | 0.571 | -4.8 | 136 | -9.3 |
| | | 0.565 | -5.8 | 135 | -10.0 |
| | | 0.558 | -7.0 | 140 | -6.7 |
| Intrarun Mean | | 0.558 | | 137 | |
| Intrarun SD | | 0.0102 | | 2.58 | |
| Intrarun %CV | | 1.8 | | 1.9 | |
| Intrarun %Bias | | -7.0 | | -8.7 | |
| n | | 6 | | 6 | |
| # > 15%Bias | | | | | |

Criteria: To be deemed stable, the mean %Bias at each level must be within ± 15% with %CV ≤ 15%.

Results: Ertapenem is stable in treated human plasma with comed[2], with sodium heparin as anticoagulant, for up to 88 days upon storage in polypropylene tube at -70 °C.

Ertapenem is unstable in treated human plasma with comed[2], with sodium heparin as anticoagulant at -70 °C at 181 days. See Table 8 for reason for failure.

Ertapenem is stable in treated human plasma without comed[2], with sodium heparin as anticoagulant, for up to 202 days upon storage in polypropylene tube at -70 °C.

MCE®
MedChemExpress

# Certificate of Analysis

Inhibitors • Screening Libraries • Proteins

## Ertapenem sodium

| | |
|---|---|
| Cat. No.: | HY-13625 |
| CAS No.: | 153773-82-1 |
| Batch No.: | 180017 |
| Chemical Name: | 1-Azabicyclo[3.2.0]hept-2-ene-2-carboxylic acid, 3-[[(3S,5S)-5-[[(3-carboxyphenyl)amino]carbonyl]-3-pyrrolidinyl]thio]-6-[(1R)-1-hydroxyethyl]-4-methyl-7-oxo-, sodium salt (1:1), (4R,5S,6S)- |

### PHYSICAL AND CHEMICAL PROPERTIES

| | |
|---|---|
| Molecular Formula: | $C_{22}H_{24}N_3NaO_7S$ |
| Molecular Weight: | **497.50** |
| Storage: | 4°C, sealed storage, away from moisture<br>* In solvent : -80°C, 6 months; -20°C, 1 month (sealed storage, away from moisture) |

Chemical Structure:

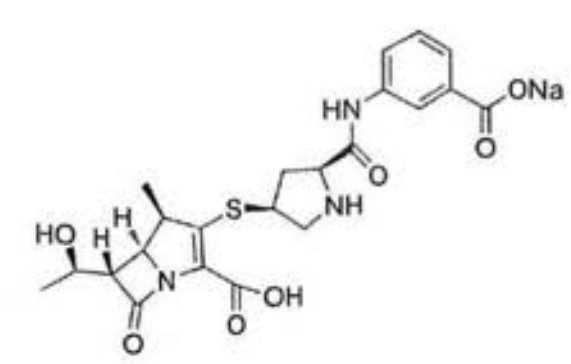


### ANALYTICAL DATA

| | |
|---|---|
| Appearance: | White to light yellow (Solid) |
| $^1$H NMR Spectrum: | Consistent with structure |
| LCMS: | Consistent with structure |
| Purity (LCMS): | **98.86%** |
| Test date: | 2022/08/12 |
| Retest date: | 2024/08/11 |
| Conclusion: | The product has been tested and complies with the given specifications. |

Name: Lingli Ma
Designation: QA Manager
Date: 6/27/2022

**Caution: Product has not been fully validated for medical applications. For research use only.**


Tel: 609-228-6898 Fax: 609-228-5909 E-mail: tech@MedChemExpress.com
Address: 1 Deer Park Dr, Suite Q, Monmouth Junction, NJ 08852, USA

## SAMPLE INFORMATION

| | | | |
|---|---|---|---|
| CAS NO.: | 153773-82-1 | Acquired By: | LY |
| Sample infromation: | | Date Acquired: | 8/12/2022 2:04:10 PM CST |
| Vial: | 2:A,3 | Acq. Method Set: | 1_POS_2MIN |
| Injection #: | 1 | Date Processed: | 8/12/2022 2:36:50 PM CST, |
| Injection Volume: | 0.30 ul | Processing Method: | PDA_01, MS_scan |
| Run Time: | 2.8 Minutes | Channel Name: | PDA Ch1 214nm@4.8nm, MS TIC, |
| Sample Set Name: | 20220812 | Column Name: | A-RP-985 |

**Auto-Scaled Chromatogram**

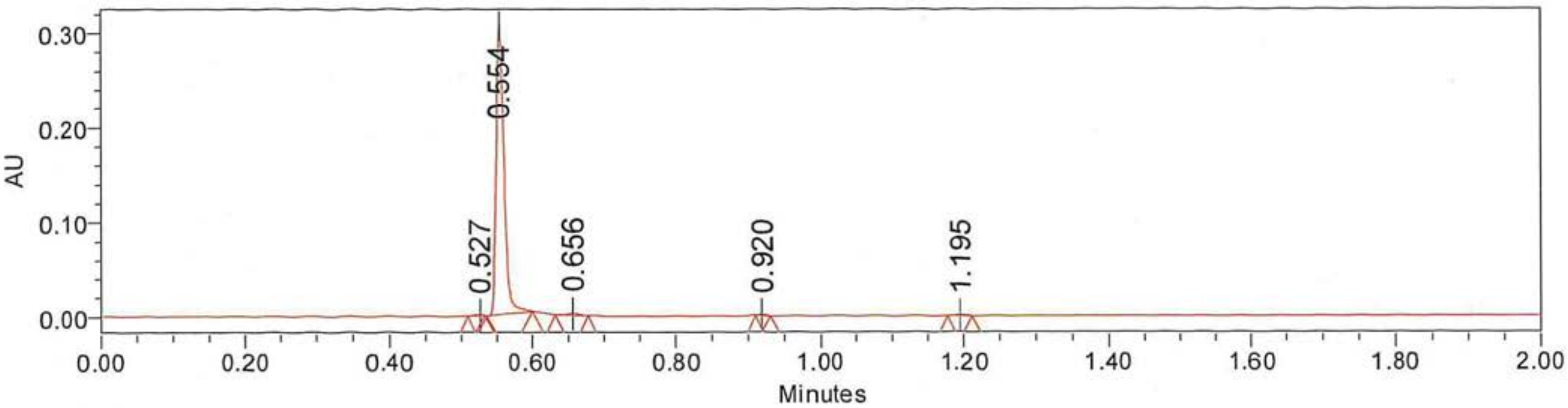


Processed Channel Descr. PDA Ch2 254nm@4.8nm

**Auto-Scaled Chromatogram**

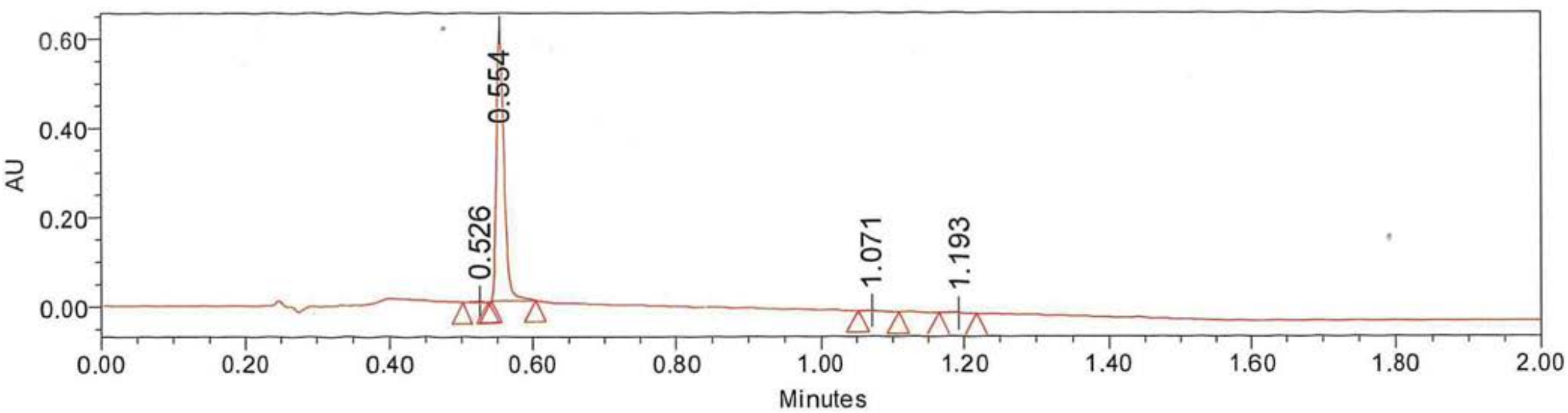


Processed Channel Descr. PDA Ch1 214nm@4.8nm

**Auto-Scaled Chromatogram**

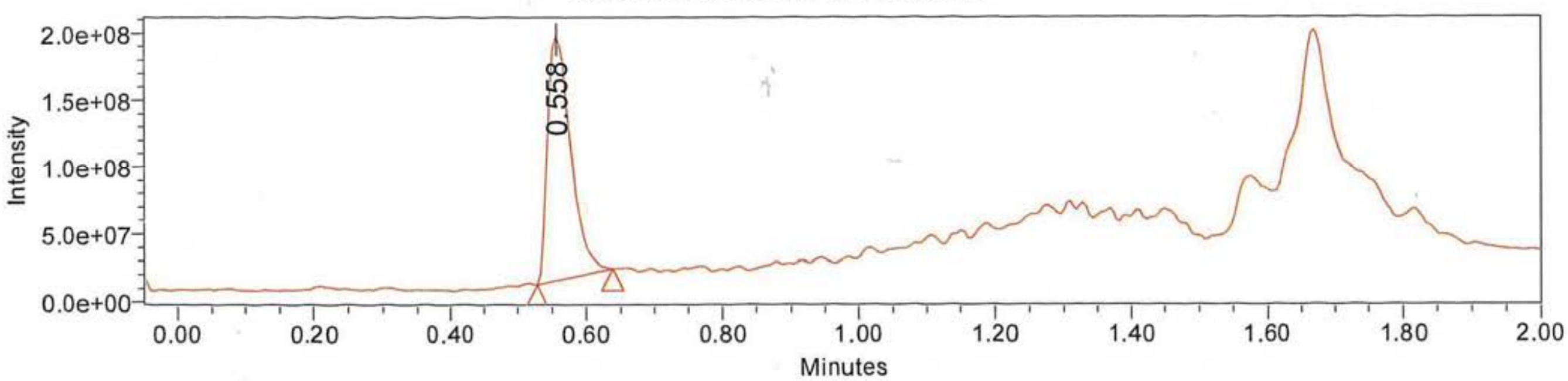


Processed Channel Descr. ACQ-SQD2 1: MS Scan MS TIC, Smoothed by 9 point Savitzky-Golay Filter., Time offset by -0.053 mins. (1: 150.00-1000.00 ES+, Centroid, CV=30)

Peak Results
Channel Name: PDA Ch2 254nm@4.8nm

| | RT | Width (sec) | Area | Height | % Area | Base Peak (Combined) (m/z) | Channel Name |
|---|---|---|---|---|---|---|---|
| 1 | 0.527 | 1.400 | 477 | 640 | 0.20 | 153.83 | PDA Ch2 254nm@4.8nm |
| 2 | 0.554 | 3.800 | 237134 | 308413 | 98.19 | 151.96 | PDA Ch2 254nm@4.8nm |
| 3 | 0.656 | 2.650 | 2446 | 2436 | 1.01 | 151.06 | PDA Ch2 254nm@4.8nm |
| 4 | 0.920 | 1.200 | 632 | 1080 | 0.26 | 152.07 | PDA Ch2 254nm@4.8nm |
| 5 | 1.195 | 2.050 | 823 | 941 | 0.34 | 152.07 | PDA Ch2 254nm@4.8nm |

Peak Results
Channel Name: PDA Ch1 214nm@4.8nm

| | RT | Width (sec) | Area | Height | % Area | Base Peak (Combined) (m/z) | Channel Name |
|---|---|---|---|---|---|---|---|
| 1 | 0.526 | 2.150 | 1796 | 2101 | 0.38 | 153.84 | PDA Ch1 214nm@4.8nm |
| 2 | 0.554 | 3.700 | 471358 | 613577 | 98.86 | 151.97 | PDA Ch1 214nm@4.8nm |
| 3 | 1.071 | 3.350 | 1629 | 1281 | 0.34 | 151.03 | PDA Ch1 214nm@4.8nm |
| 4 | 1.193 | 3.150 | 1997 | 1243 | 0.42 | 152.07 | PDA Ch1 214nm@4.8nm |

Peak Results
Channel Name: MS TIC

| | RT | Width (sec) | Area | Height | % Area | Base Peak (Combined) (m/z) | Channel Name |
|---|---|---|---|---|---|---|---|
| 1 | 0.558 | 6.673 | 431532509 | 182380390 | 100.00 | 476.14 | MS TIC |

**Match Plot**

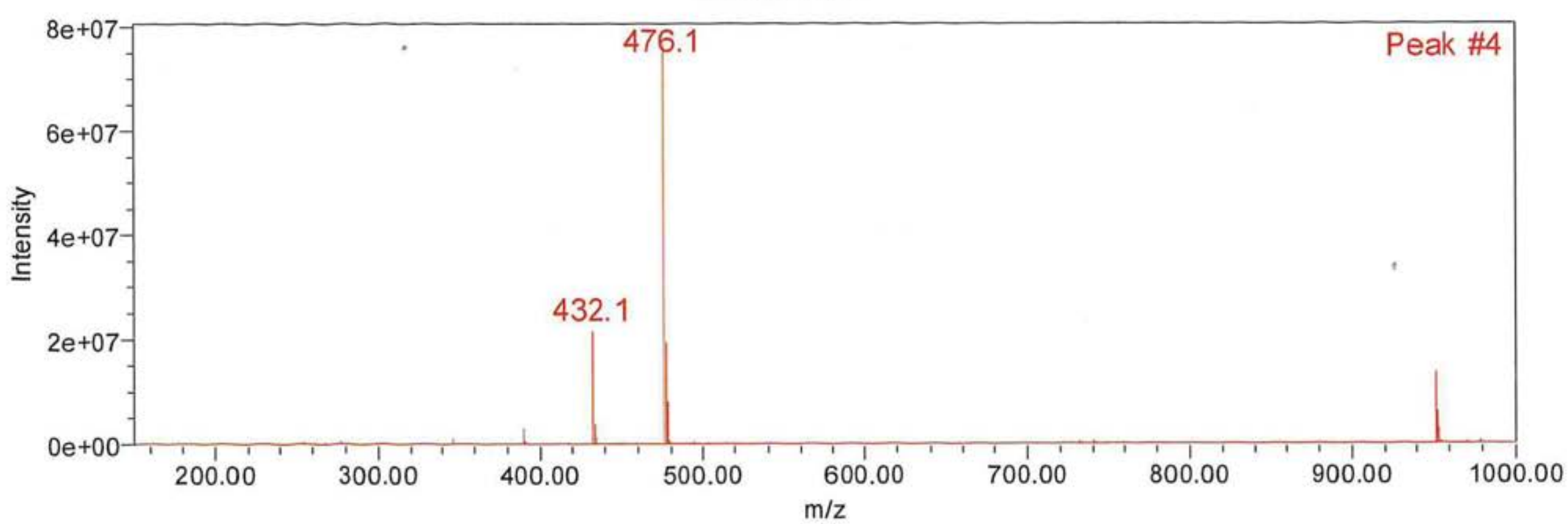


Retention Time 0.558 Channel Name MS TIC

CAS NO.: 153773-82-1, D2O

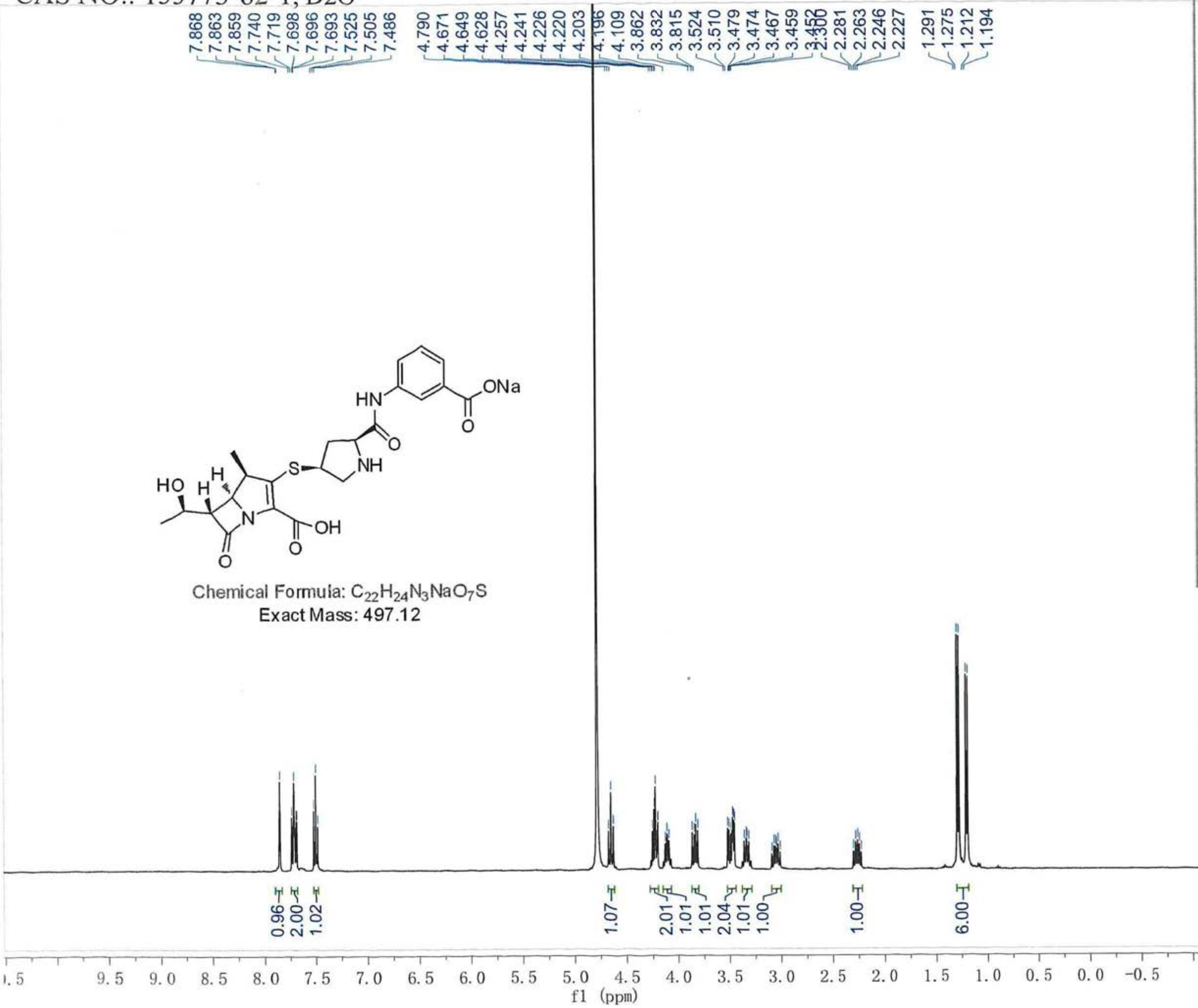


Chemical Formula: $C_{22}H_{24}N_3NaO_7S$
Exact Mass: 497.12

| | Parameter | Value |
|---|---|---|
| 1 | Title | |
| 2 | Origin | Bruker BioSpin GmbH |
| 3 | Owner | nmrsu |
| 4 | Spectrometer | Avance |
| 5 | Author | |
| 6 | Solvent | D2O |
| 7 | Temperature | 298.2 |
| 8 | Pulse Sequence | zg30 |
| 9 | Experiment | 1D |
| 10 | Number of Scans | 8 |
| 11 | Receiver Gain | 101 |
| 12 | Relaxation Delay | 1.0000 |
| 13 | Pulse Width | 8.0000 |
| 14 | Acquisition Time | 3.9977 |
| 15 | Acquisition Date | 2022-08-10T13:49:06 |
| 16 | Modification Date | 2022-08-10T13:53:00 |
| 17 | Spectrometer Frequency | 400.13 |
| 18 | Nucleus | 1H |

Certificate of Analysis

Inhibitors • Screening Libraries • Proteins

## Ertapenem sodium

**Cat. No.:** HY-13625

**CAS No.:** 153773-82-1

**Batch No.:** 229333

**Chemical Name:** 1-Azabicyclo[3.2.0]hept-2-ene-2-carboxylic acid, 3-[[(3S,5S)-5-[[(3-carboxyphenyl)amino]carbonyl]-3-pyrrolidinyl]thio]-6-[(1R)-1-hydroxyethyl]-4-methyl-7-oxo-, sodium salt (1:1), (4R,5S,6S)-

### PHYSICAL AND CHEMICAL PROPERTIES

**Molecular Formula:** $C_{22}H_{24}N_3NaO_7S$

**Molecular Weight:** **497.50**

**Storage:** 4°C, sealed storage, away from moisture
* In solvent : -80°C, 6 months; -20°C, 1 month (sealed storage, away from moisture)

**Test Date:** 2022.09.30

**Retest Date:** 2025.09.29

**Chemical Structure:**

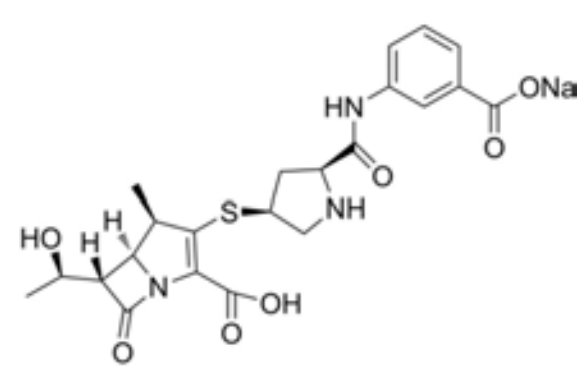


### ANALYTICAL DATA

**Appearance:** White to light yellow (Solid)

**$^1$H NMR Spectrum:** Consistent with structure

**LCMS:** Consistent with structure

**Purity (LCMS):** **98.43%**

**Conclusion:** The product has been tested and complies with the given specifications.

Name: Lingli Ma
Designation: QA Manager
Date: 2/28/2023

**Caution: Product has not been fully validated for medical applications. For research use only.**

Tel: 609-228-6898 Fax: 609-228-5909 E-mail: tech@MedChemExpress.com
Address: 1 Deer Park Dr, Suite Q, Monmouth Junction, NJ 08852, USA

# SAMPLE INFORMATION

| | | | |
|---|---|---|---|
| CAS NO.: | 153773-82-1 | Acquired By: | LY |
| Sample infromation: | | Date Acquired: | 9/30/2022 10:03:14 AM CST |
| Vial: | 2:E,8 | Acq. Method Set: | 1_POS_2MIN |
| Injection #: | 1 | Date Processed: | 9/30/2022 1:46:00 PM CST, |
| Injection Volume: | 0.30 ul | Processing Method: | PDA_01, MS_scan |
| Run Time: | 2.8 Minutes | Channel Name: | PDA Ch1 214nm@4.8nm, MS TIC, |
| Sample Set Name: | 20220930 | Column Name: | A-RP-985 |

**Auto-Scaled Chromatogram**

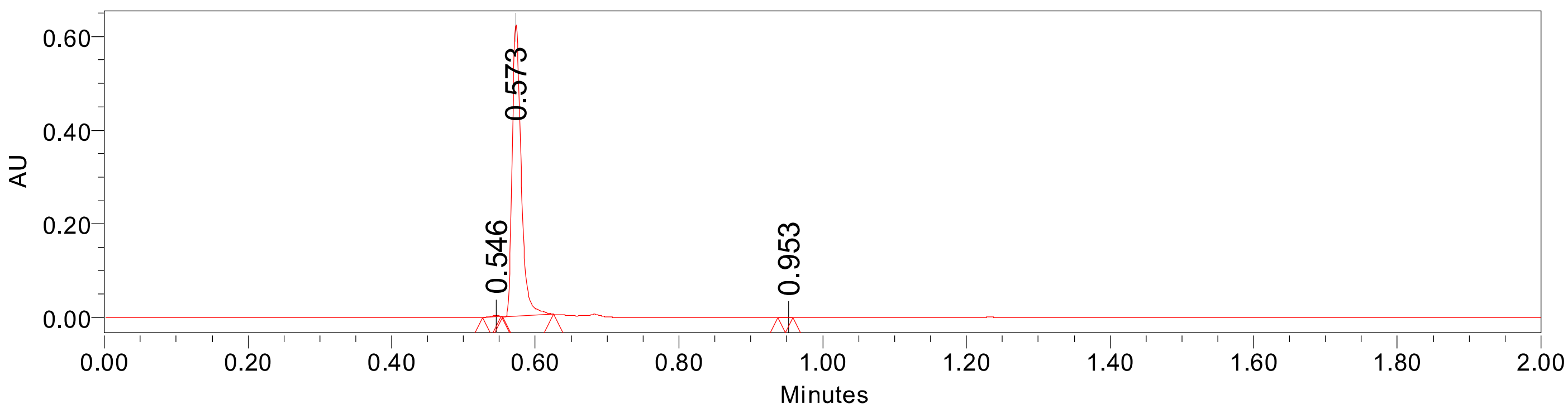


Processed Channel Descr. PDA Ch2 254nm@4.8nm

**Auto-Scaled Chromatogram**

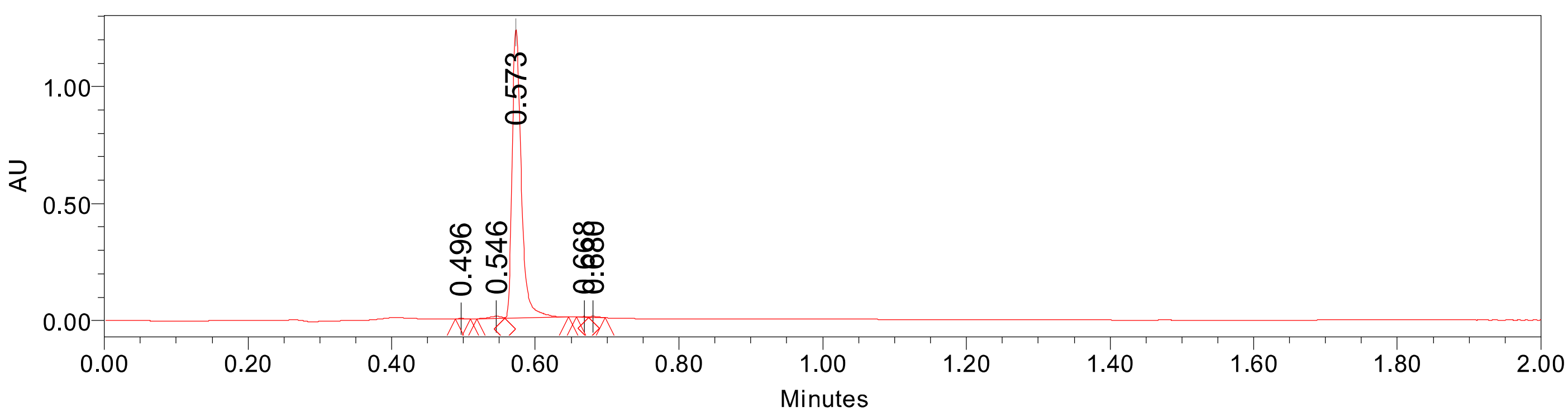


Processed Channel Descr. PDA Ch1 214nm@4.8nm

**Auto-Scaled Chromatogram**

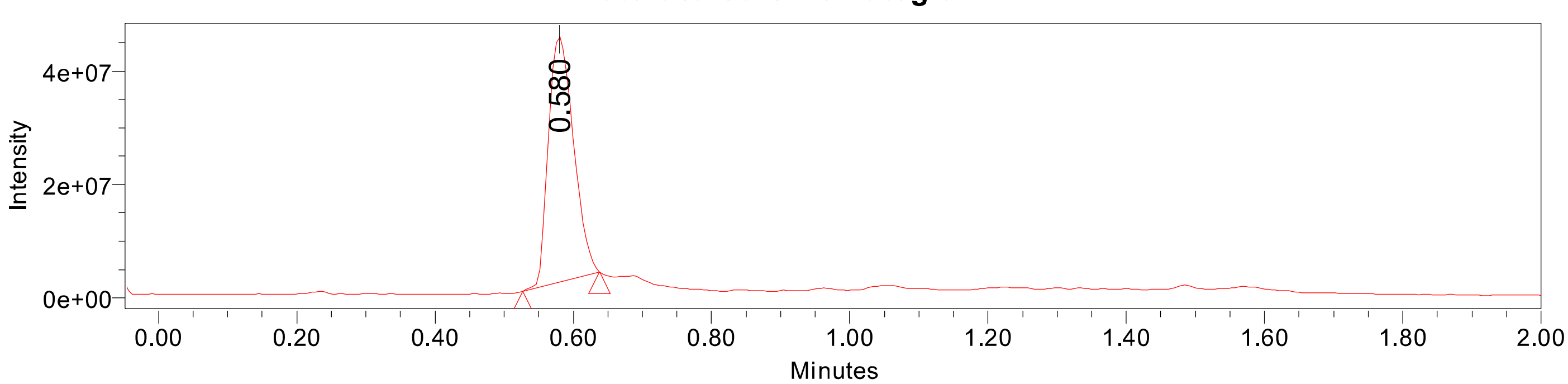


Processed Channel Descr. ACQ-SQD2 1: MS Scan MS TIC, Smoothed by 9 point Savitzky-Golay Filter., Time offset by -0.053 mins. (1: 150.00-1000.00 ES+, Centroid, CV=30)

Peak Results
Channel Name: PDA Ch2 254nm@4.8nm

| | RT | Width (sec) | Area | Height | % Area | Base Peak (Combined) (m/z) | Channel Name |
|---|---|---|---|---|---|---|---|
| 1 | 0.546 | 1.550 | 1772 | 2235 | 0.3283 | 153.13 | PDA Ch2 254nm@4.8nm |
| 2 | 0.573 | 4.200 | 537556 | 621641 | 99.5658 | 247.60 | PDA Ch2 254nm@4.8nm |
| 3 | 0.953 | 1.250 | 572 | 735 | 0.1059 | 279.20 | PDA Ch2 254nm@4.8nm |

Peak Results
Channel Name: PDA Ch1 214nm@4.8nm

| | RT | Width (sec) | Area | Height | % Area | Base Peak (Combined) (m/z) | Channel Name |
|---|---|---|---|---|---|---|---|
| 1 | 0.496 | 1.250 | 468 | 836 | 0.0425 | 222.95 | PDA Ch1 214nm@4.8nm |
| 2 | 0.546 | 2.350 | 9474 | 8900 | 0.8608 | 235.17 | PDA Ch1 214nm@4.8nm |
| 3 | 0.573 | 5.300 | 1083411 | 1232417 | 98.4367 | 247.60 | PDA Ch1 214nm@4.8nm |
| 4 | 0.668 | 1.000 | 2871 | 4011 | 0.2608 | 495.37 | PDA Ch1 214nm@4.8nm |
| 5 | 0.680 | 1.400 | 4392 | 5147 | 0.3991 | 514.34 | PDA Ch1 214nm@4.8nm |

Peak Results
Channel Name: MS TIC

| | RT | Width (sec) | Area | Height | % Area | Base Peak (Combined) (m/z) | Channel Name |
|---|---|---|---|---|---|---|---|
| 1 | 0.580 | 6.673 | 109356458 | 43221408 | 100.0000 | 476.23 | MS TIC |

**Match Plot**

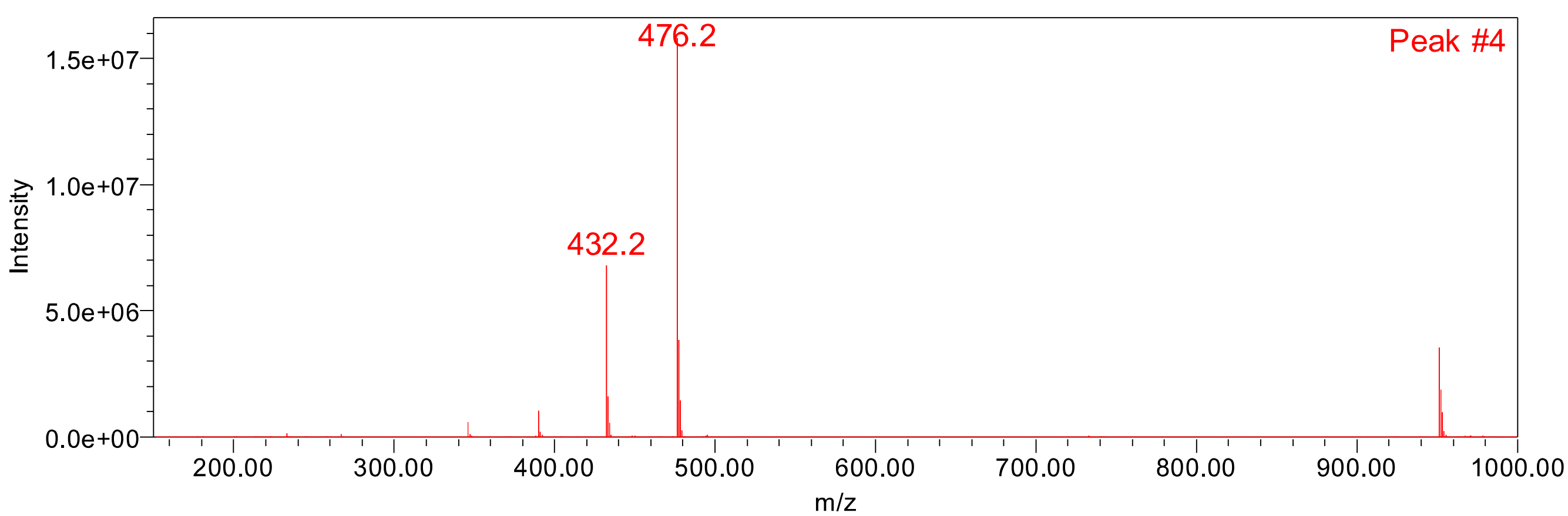


Retention Time 0.580 Channel Name MS TIC

# CAS NO.: 153773-82-1, D2O

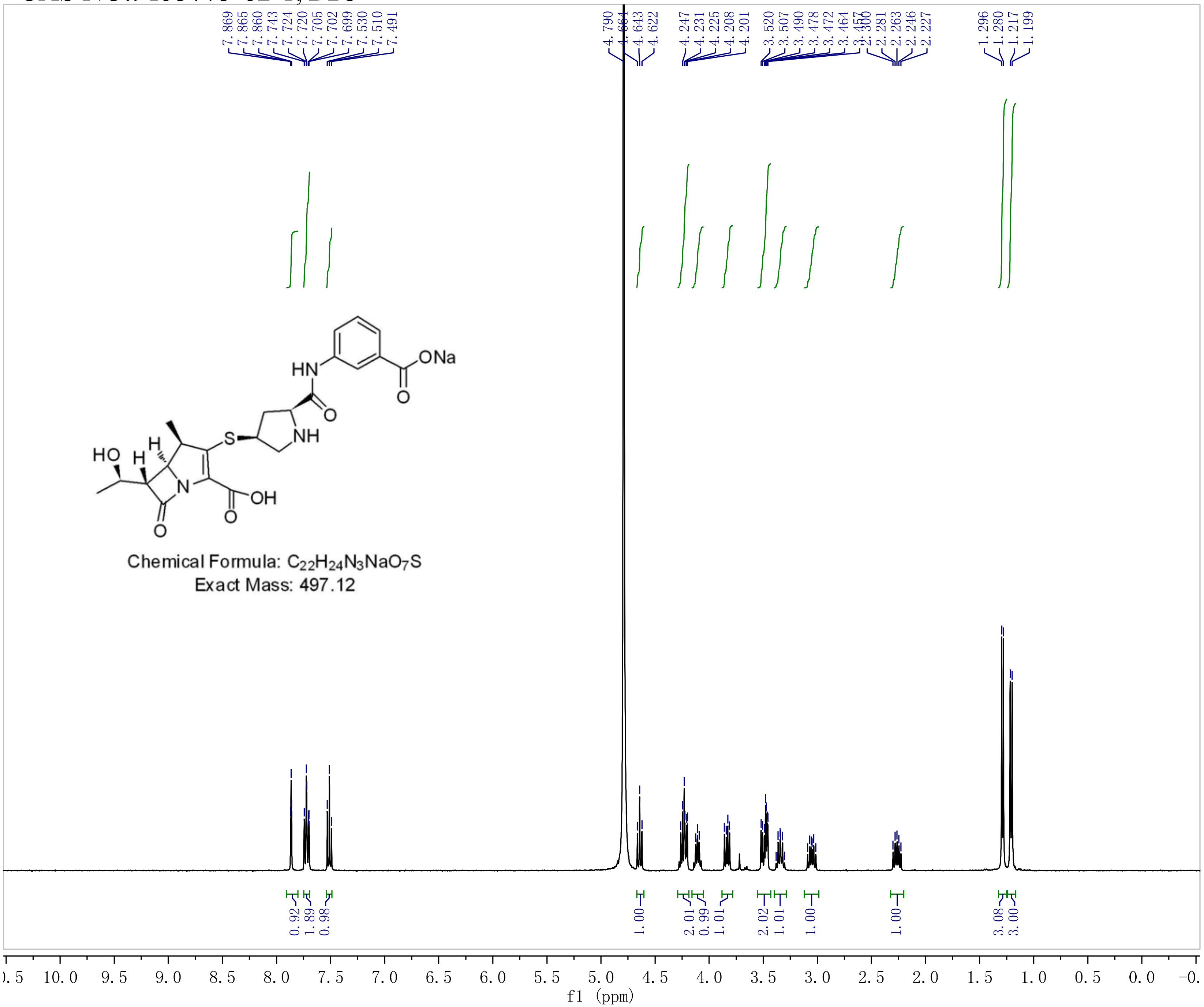


| Parameters | | |
|---|---|---|
| | Parameter | Value |
| 1 | Title | |
| 2 | Spectrometer | Avance |
| 3 | Solvent | D2O |
| 4 | Temperature | 298.4 |
| 5 | Pulse Sequence | zg30 |
| 6 | Number of Scans | 8 |
| 7 | Receiver Gain | 101 |
| 8 | Relaxation Delay | 1.0000 |
| 9 | Pulse Width | 8.0000 |
| 10 | Acquisition Time | 3.9977 |
| 11 | Acquisition Date | 2022-09-29T18:18:21 |
| 12 | Modification Date | 2022-09-29T18:23:12 |
| 13 | Spectrometer Frequency | 400.13 |
| 14 | Spectral Width | 8196.7 |
| 15 | Lowest Frequency | -1627.6 |
| 16 | Nucleus | 1H |
| 17 | Acquired Size | 32768 |
| 18 | Spectral Size | 65536 |

# CERTIFICATE OF ANALYSIS

ALSACHIM
a Shimadzu Group Company

**Product Name:** [$^{2}H_{4}$]-Ertapenem sodium salt

**Molecular Formula:** $C_{22}H_{24}N_{3}O_{7}S$ . Na

**Labeled Molecular Formula:** $C_{22}H_{20}D_{4}N_{3}O_{7}S$ . Na

**Molecular Weight:** 501.52 g/mol

**Structure:**

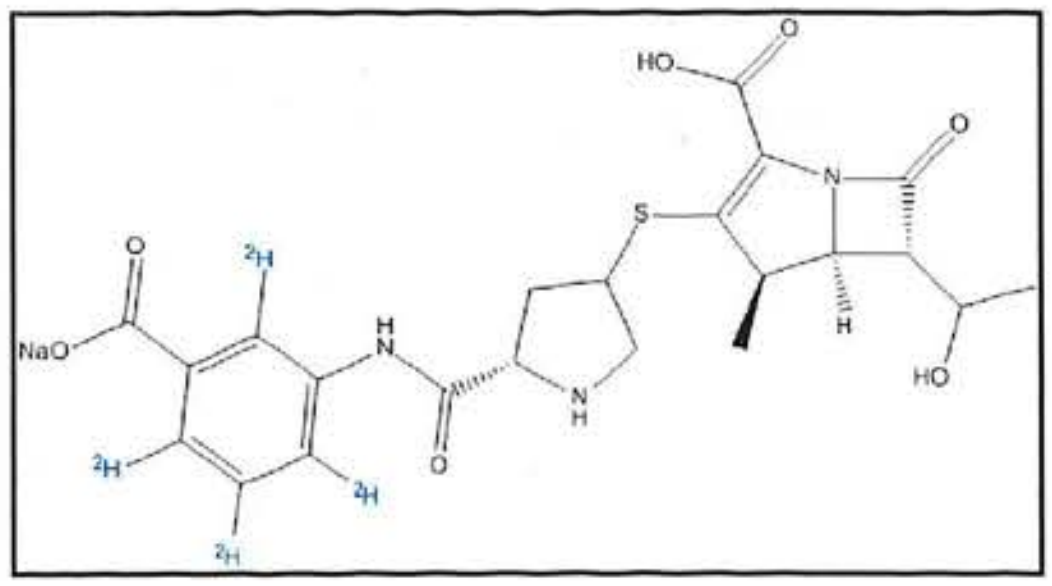


**Batch Number:** DH-ALS-20-002-P2

**Product Number:** C3376

**CAS Number:** 153773-82-1 unlabeled

**Storage:** For a prolonged period, keep at -20°C (±5°C) in a closed container, away from light and moisture

**Stability:** Stable (12 months) (*)

**Extended expiring date:** Saturday April 29, 2023

**Solvent solubility:** Soluble in Water or Acetonitrile/Water (1/1)

| | **Specification:** | **Results:** |
|---|---|---|
| **Appearance:** | Pale grey to white solid | Off white solid |
| **Chemical Purity (HPLC):** | ≥ 80.0% | 89,4% |
| **Isotopic Enrichment:** | ≥ 98.0% $^{2}H$ | 99.0% $^{2}H$ |
| **Unlabeled (M+0) Content:** | Unlabeled (M+0) content < 0.5% | <0.5% |
| **Mass Spectrometry:** | Conform to structure | Conforms |
| **Manufacturing date:** | Tuesday January 7, 2020 | |
| **Reanalysis date:** | Friday April 29, 2022 | |
| **Note:** | For research and development use only, not for use in humans. | |

**Quality Review & Approval**

Francis ROESSLINGER, Quality Manager
Date : Wednesday, May 18, 2022


RECEIVED
SEP 21 REC'D
By

ALSACHIM
a Shimadzu Group Company

# Analysis Report

## <Equipment Information>

LCMS Shimadzu

Alsachim Reference: AN-LC01 + AN-MS01

Pump : LC-40D

Degassing Unit : DGU-405

Auto Sampler : SIL-40C

Diode Array Detector : SPD-M40

Column Oven : CTO-40S

Valve Unit : FCV-20AH2

Liquid Chromatograph Mass Spectrometer : LCMS-2020

Column : Accucore C18 50*4.6 2.6um PN: 17126-054630 SN: 20333623 LOT: 20512

## <Sample Information>

Sample Name : DH-ALS-20-002-P2
Data Filename : DH-ALS-20-002-P2_002.lcd
Method Filename : HP-C18-AF-20MIN.lcm
Batch Filename : 2022-APR-29-3.lcb
Vial # : 2-20
Injection Volume : 0,5 uL
Date Acquired : 29/04/2022 14:31:49
Date Processed : 02/05/2022 08:59:47
Solvent : Water
Concentration : 1 mg/ml
Operator : CD

## <Peak Table >

PDA Ch1 295nm

| Peak# | Ret. Time | Area | Height | Area% |
|---|---|---|---|---|
| 1 | 1,861 | 2224 | 1305 | 0,544 |
| 2 | 2,364 | 16348 | 3642 | 4,001 |
| 3 | 2,684 | 365589 | 157196 | 89,478 |
| 4 | 2,940 | 5546 | 2826 | 1,357 |
| 5 | 3,046 | 2444 | 1326 | 0,598 |
| 6 | 3,202 | 596 | 403 | 0,146 |
| 7 | 3,540 | 15028 | 2285 | 3,678 |
| 8 | 3,776 | 806 | 433 | 0,197 |
| Total | | 408582 | 169417 | 100,000 |

HPLC method and UV Instrument Parameters

<<Pump>>

| | |
|---|---|
| Mode | : Binary gradient |
| Pump A | : LC-40D X3 |
| Pump B | : LC-40D X3 |
| PressMax | : 400 bar |
| PressMin | : 0 bar |
| Pump A Valve Model | : Switching Valve |
| Pump B Valve Model | : Switching Valve |
| Gradient Program: | |

Pump A + B

| # | Time(min) / Flow(mL/min) / B.Conc(%) / B.Curve |
|---|---|
| 1 | 0,00/ 1,2000/ 1,0/ 0 |
| 2 | 9,00/ / 70,0/ |
| 3 | 15,00/ / 99,0/ |
| 4 | 19,00/ / 99,0/ |
| 5 | 19,10/ / 1,0/ |

Pump A Mobile Phase Settings : Water + 0.1% formic acid 0,45 /GPa
Pump B Mobile Phase Settings : Acetonitrile + 0.1% formic acid 1,20 /GPa

<<LC Time Program>>

| Time | Module | Command | Value | Comment |
|---|---|---|---|---|
| 20,00 | Controller | Stop | | |

<<PDA>>

| | |
|---|---|
| Lamp | : D2 & W |
| Start Wavelength | : 190 nm |
| End Wavelength | : 800 nm |

## <HPLC-UV Chromatogram >

Chromatogram

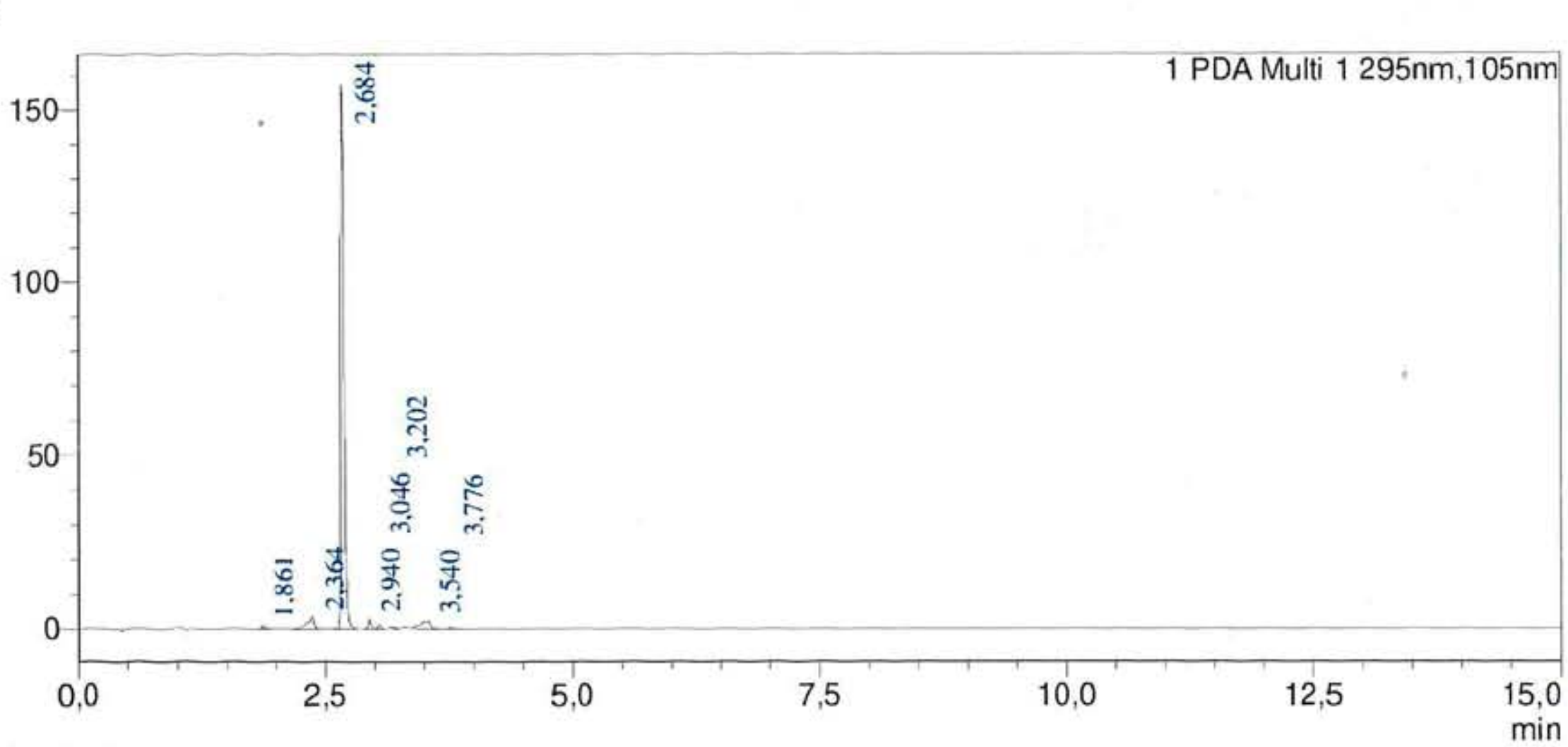


## <UV Spectrum >

UV Spectrum

| | |
|---|---|
| Peak# | : 3 |
| Retention Time | : 2,684 min |
| Spectrum Operation | : None |

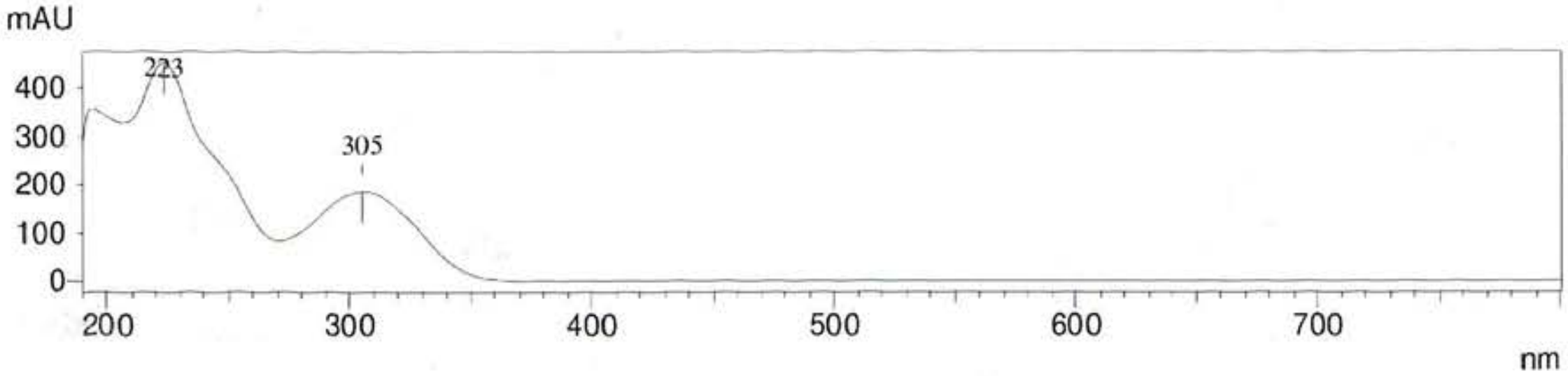

MS Instrument Parameter

| | |
|---|---|
| <<MS Parameter>> | |
| Initial Valve Position | :0 |
| --Segment 1 Event 1-- | |
| Start Time | :0,00 min |
| End Time | :20,00 min |
| Acquisition Mode | :Scan |
| Polarity | :Positive |
| Event Time | :1,00 sec |
| Detector Voltage | :+1,10 kV |
| Threshold | :0 |
| Start m/z | :100,00 |
| End m/z | :1100,00 |
| Scan Speed | :1072 u/sec |
| Interface Volt. | :Use the Data in the Tuning File |
| DL Volt. | :Use the Data in the Tuning File |
| Qarray DC Voltage | :Use the Data in the Tuning File |
| Qarray DC Voltage | :Use the Data in the Tuning File |
| --Segment 1 Event 2-- | |
| Start Time | :0,00 min |
| End Time | :20,00 min |
| Acquisition Mode | :Scan |
| Polarity | :Negative |
| Event Time | :1,00 sec |
| Detector Voltage | :+1,10 kV |
| Threshold | :0 |
| Start m/z | :100,00 |
| End m/z | :1100,00 |
| Scan Speed | :1072 u/sec |
| Interface Volt. | :Use the Data in the Tuning File |
| DL Volt. | :Use the Data in the Tuning File |
| Qarray DC Voltage | :Use the Data in the Tuning File |
| Qarray DC Voltage | :Use the Data in the Tuning File |
| <<Interface>> | |
| Interface | :DUIS |
| DL Temperature | :300 C |
| Nebulizing Gas Flow | :1,50 L/min |
| Heat Block | :400 C |
| Drying Gas | :On |
| | 18,00 L/min |

| Segment | Start Time | End Time | Ionization Mode |
|---|---|---|---|
| 1 | 0,00 | 20,00 | ESI&APCI |

## <HPLC-MS Chromatogram>

MS Chromatogram

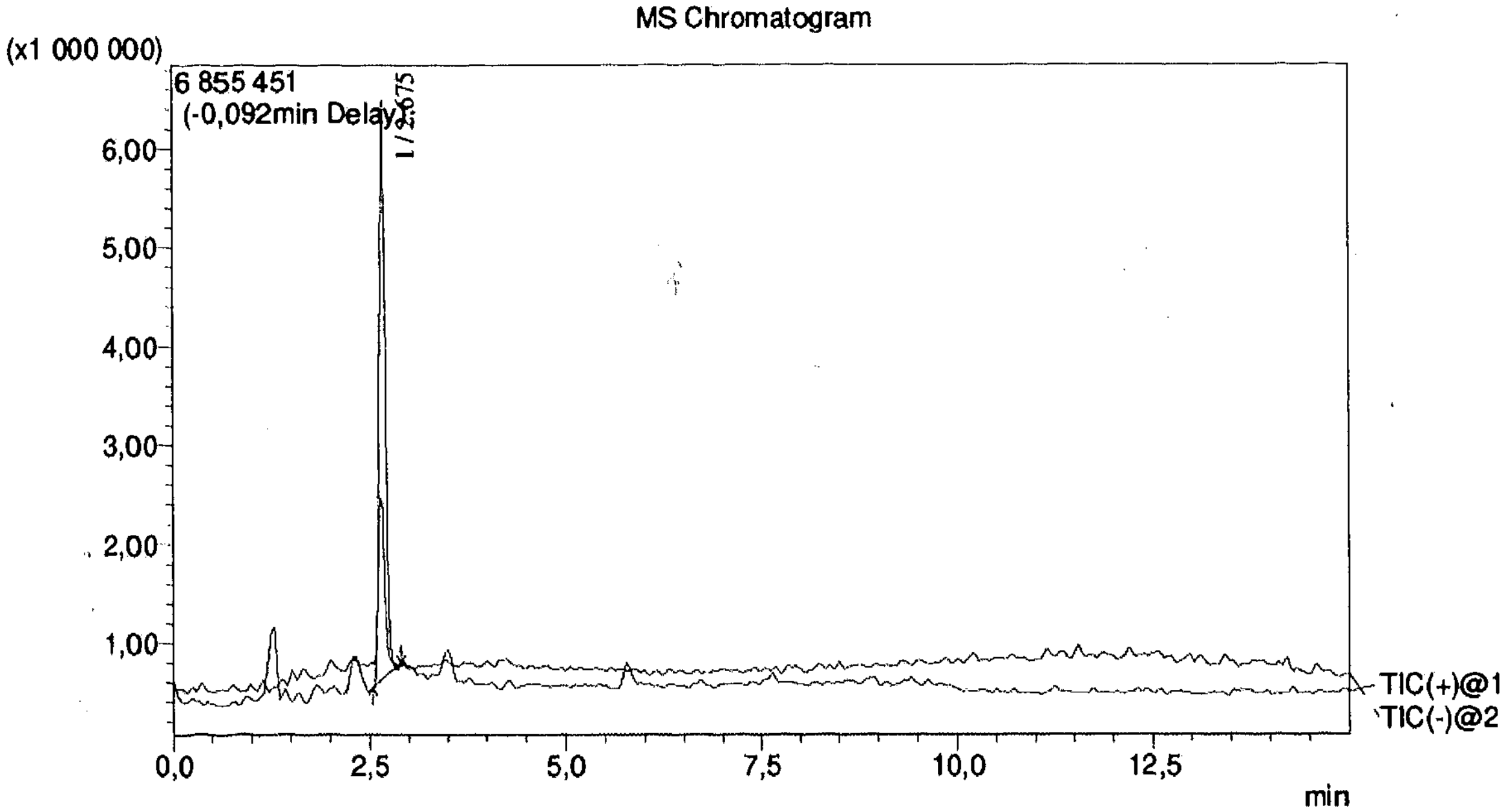

## < Mass spectrum>

MS Spectrum

Peak#:1 R.Time:2,675(Scan#:167)
MassPeaks:660
Spectrum Mode:Averaged 2,641-2,708(165-169)
BG Mode:Calc Segment 1 - Event 1

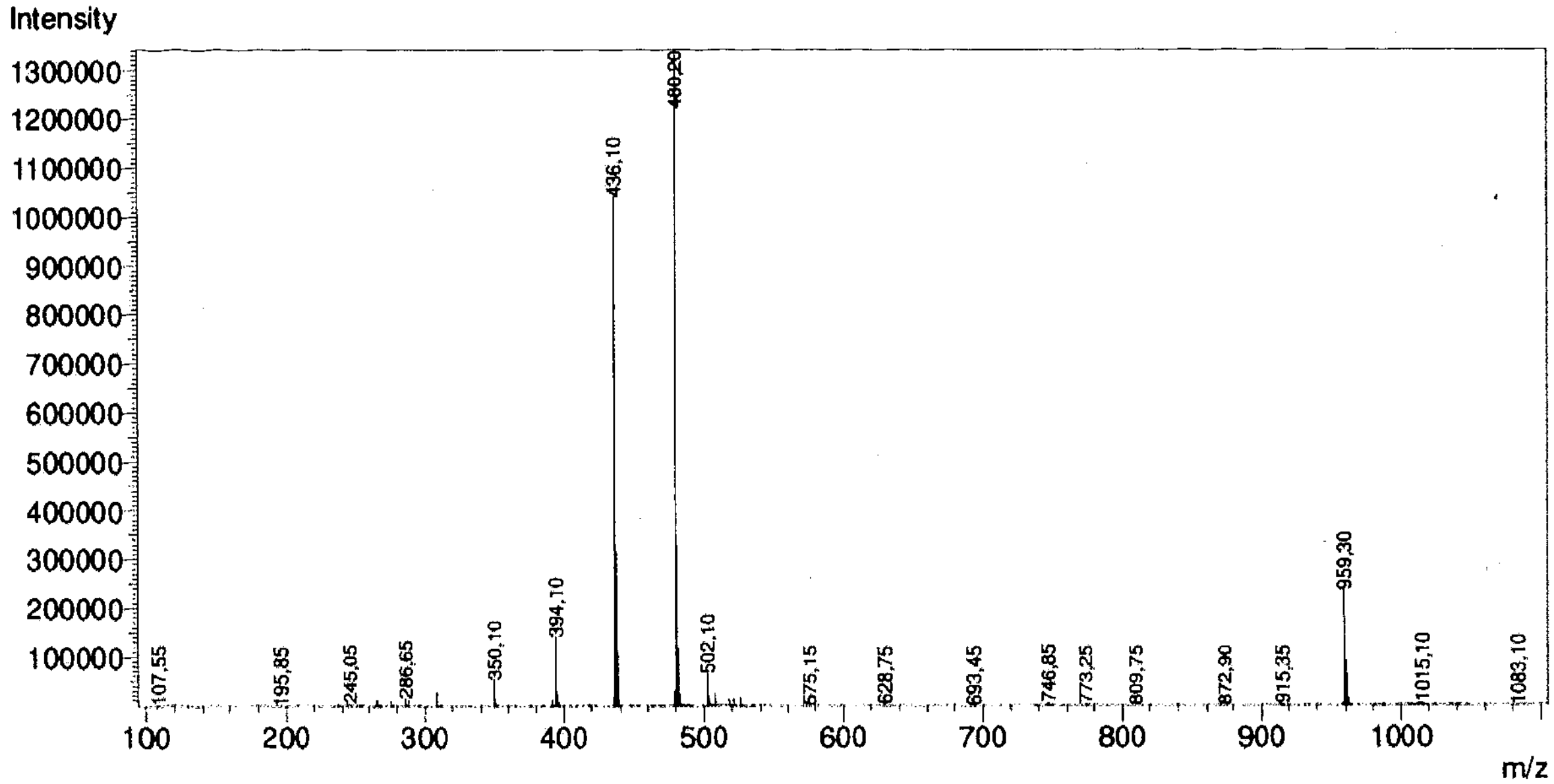


## < Mass Profile Spectrum>

MS Profile Spectrum

Peak#:1 R.Time:2,675(Scan#:167)
MassPeaks:660
Spectrum Mode:Averaged 2,641-2,708(165-169)
BG Mode:Calc Segment 1 - Event 1

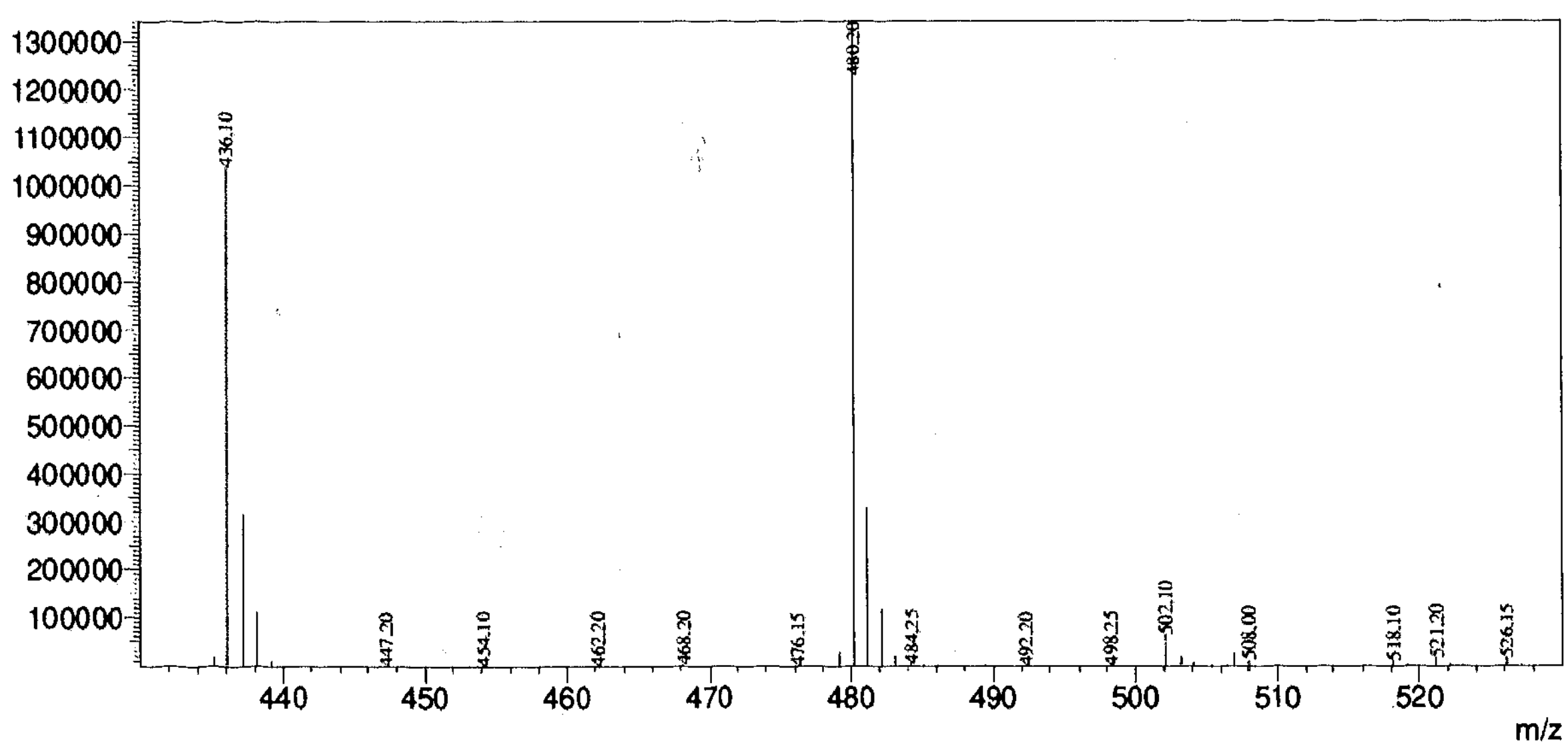

## ANALYTICAL RESEARCH

## CERTIFICATE OF ANALYSIS FOR REFERENCE STANDARD

| **Name** | : | **Zidebactam (WCK 5107)** | | | |
|---|---|---|---|---|---|
| Molecular Formula | : | $C_{13}H_{21}N_5O_7S.2H_2O$ | Batch Number | : | WK1/WP-0060/016 |
| Molecular Weight | : | 427.45 | Laboratory Note Book Reference | : | WA-2004-038 |
| Date of Analysis | : | 30/03/2022 | Month of Manufacturing | : | May' 2019 |
| Valid Up To | : | 29/03/2023 | Reference Standard Number | : | RS/2022-013 |
| Effective Date | : | 23/04/2022 | Page Number | : | Page 1 of 4 |

| Sr. No. | Tests | Specifications | Results |
|---|---|---|---|
| 1 | Description | White to pale yellow powder | White powder |
| 2 | Identification (By IR, Mass & NMR Spectroscopy) | Spectral data should conform to the structure | Spectral data conforms the structure |
| 3 | Solubility | Freely soluble in water, Insoluble in dichloromethane | Freely soluble in water, Insoluble in dichloromethane |
| 4 | Constituted solution (10% w/v solution in WFI)<br>Completeness of solution | The solid dissolves completely, leaving no visible residue as un-dissolved matter | Complies |
| | Clarity of solution | The constituted solution is not significantly less clear than an equal volume of the WFI contained in a similar vessel and examined similarly | Complies |
| 5 | pH of solution | Between 5.0 to 7.0 | 6.0 |
| 6 | Absorbance of solution (1.25 % w/v in water at 420 nm) | Not more than 0.2 AU | 0.01 AU |
| 7 | Water content (By KF Titration) | Not more than 12.0 % w/w | 8.7 % w/w |
| 8 | Specific optical rotation (On anhydrous basis) | Between –28° to -40° | -36° |
| 9 | Heavy metals | Not more than 20 μg/g | Less than 20 μg/g |
| 10 | Palladium content (By ICP-OES) | Not more than 10 μg/g | BLQ (LOQ 2.5 μg/g)* |

| | Prepared by | Checked by | Approved by |
|---|---|---|---|
| Name : | Mr. Sitaram Ubale | Mr. Rajendra Chavan | Mr Subhash Chandra Shukla |
| Department : | AVL | AVL | CRDQ |
| Sign : | [signature] | [signature] | [signature] |
| Date : | 12/04/2022 | 12/04/2022 | 13/04/2022 |

Format No. : AVL-045-F-02-00

Ranked # 1 in Healthcare
GREAT PLACE TO WORK® Best Companies to Work for 2015 India

WOCKHARDT LIFE WINS

ANALYTICAL RESEARCH

## CERTIFICATE OF ANALYSIS FOR REFERENCE STANDARD

| | | | | | |
|---|---|---|---|---|---|
| **Name** | : | **Zidebactam (WCK 5107)** | | | |
| Molecular Formula | : | $C_{13}H_{21}N_5O_7S.2H_2O$ | Batch Number | : | WK1/WP-0060/016 |
| Molecular Weight | : | 427.45 | Laboratory Note Book Reference | : | WA-2004-038 |
| Date of Analysis | : | 30/03/2022 | Month of Manufacturing | : | May' 2019 |
| Valid Up To | : | 29/03/2023 | Reference Standard Number | : | RS/2022-013 |
| Effective Date | : | 23/04/2022 | Page Number | : | Page 3 of 4 |

| Sr. No. | Tests | Specifications | Results |
|---|---|---|---|
| 13 | Sodium content (By ICP-OES) | Not more than 0.5 % w/w | BLQ (LOQ 0.25 % w/w)* |
| 14 | Sulfate content (By Ion chromatography) | Not more than 1.0 % w/w | BLQ (LOQ 0.626 % w/w) |
| 15 | Mono-methyl sulphate content (By Ion chromatography) | Not more than 0.15% w/w | Not detected |
| 16 | Polymorphism (By X-ray Diffraction) | The diffractogram should exhibit characteristic 2 theta values at 13.8, 15.6, 20.3 and 24.3 (± 0.2°) | The diffractogram exhibits characteristic 2 theta values at 13.8, 15.6, 20.3 & 24.3 |
| 17 | Potency (on as is basis) | Not applicable | 90.4 % |

BLQ = Below limit of quantification

BDL = Below disregard limit. Disregard limit 0.05 %

LOQ = Limit of quantification

* LOQ Values are based on analytical method verification report (Report nos. MVR/VP17053 and MVR/VP17054) of Analytical Solutions (CRO).

Specification Reference Number: **ZDB-DSS-112-05**

**Remark:** Potency is calculated on the basis of Total impurities, Water content, Residual solvents, Sulphate, Mono-methyl sulphate, Sodium and Palladium content.

**Storage Condition:** Store at 2°C to 8°C temperature.

| | Prepared by | Checked by | Approved by |
|---|---|---|---|
| Name : | Mr. Sitaram Ubale | Mr. Rajendra Chavan | Mr Subhash Chandra Shukla |
| Department : | AVL | AVL | CRDQ |
| Sign : | [signature] | [signature] | [signature] |
| Date : | 12/04/2022 | 12/04/2022 | 13/04/2022 |

Format No. : AVL-045-F-02-00

Ranked # 1 in Healthcare GREAT PLACE TO WORK Best Companies to Work for 2015 India

# Deviation Detail

All blank fields are considered as data not available. All Signature (Date and Time) shown in this report are in GMT.

| | | | |
|---|---|---|---|
| **Deviation Number** | DEV-2023-0088 | **Status** | CLOSED |
| **Deviation Classification** | Minor | **Deviation For** | Process |
| **Deviation Category** | Other Process | **Incident Date** | 16-Mar-2023 |
| **Deviation Type** | Internal | **Deviation Owner** | PIPPINA Anika Pippin |

## Deviation Information

| | | | | |
|---|---|---|---|---|
| **Site** | KCAS | KCAS | | |
| **Deviation Source** | SOP | | **Project-Study / Non-Project Identifier** | V1872106P5A1 |
| **Process** | Reporting | Reporting | **Department** | Pharma LC-MS/MS |
| **Reported By** | PIPPINA | Anika Pippin | **Reported Date** | 16-Mar-2023 |

**Action Taken?** [No] Initial Containment [No] Immediate Action

**Title**

V1872106P5A1 - Comed Stability for 60 days Acceptance

**Description**

The treated comed QC low concentration used in run V1872106P5A1 run 1 for the 60-day stability shows %bias>15%. This is due to the Time 0 %bias for the low QC being on the high side (tested in V1872106P5 run 18) with 11.0%. The %change between Time 0 and the 60-day LTS data is 9.5% which is within the %bias acceptance criteria. The sponsor has requested previously that these comed QCs be used for future LTS testing (see attached email), and that both, %bias from nominal and from Time 0 be reported. The stability will be accepted as passing since it is within 15% of the original preparation.

### Impacted Product ()

| Primary Product | Product | Batch Number | Lot Number | Affected Quantity | UOM |
|---|---|---|---|---|---|

### Affected Document ()

| Document Number | Document Revision | Title |
|---|---|---|

## Other Affected Item ()

| Item Type | Affected Item | Description |
|---|---|---|

## Immediate Action

**Action Plan**

**Action Plan Description**

**Completed By**

**Completed Date**

# QA Review

**Approval Decision**

**Comment**

**Signature** [GMT]

# Containment ()

| Performed By | Performed Date | Added In |
|---|---|---|

**Comment**

## Disposition (0)

| Product | Batch Number | Lot Number | Affected Quantity | UOM | Disposition | Dispositioned Quantity |
|---|---|---|---|---|---|---|

**Disposition Summary**

## Investigation

### Investigation Tool ()

| Tool | Comment |
|---|---|
| | |

### Result of Investigation (1)

**Result**

No investigation is needed. Root Cause Analysis and Risk Assessment are completed.

| | | | | |
|---|---|---|---|---|
| **Performed By** | PIPPINA | Anika Pippin | **Entered Date** | 16-Mar-2023 |

## Root Cause Analysis (1)

| Root Cause For | Product / Process | Failure Mode (What went wrong) | Root Cause (Why) |
|---|---|---|---|
| Process | Reporting | Acceptance criteria | Client Communication |

| | | |
|---|---|---|
| **Process** | Reporting | Reporting |
| **Failure Mode (What went wrong)** | Acceptance criteria | |
| **Failure Mode Description** | Did not meet established acceptance criteria | |
| **Root Cause (Why)** | Client Communication | |
| **Root Cause Description** | Communication between KCAS and the client | |
| **Root Cause Percentage** | 100 | |
| **Caused By** | | |

## Risk Assessment (1)

| Failure Mode (What went wrong) | Severity Rating | Likelihood of Occurrence Rating | Detectability Rating | Risk Analysis Outcome |
|---|---|---|---|---|
| Acceptance criteria | Minor | Low | Very High | Monitor - No immediate actions |

| | | | |
|---|---|---|---|
| **Severity Rating** | Minor | **Heat Map Values** | ( 2 , 2 ) |
| **Likelihood of Occurrence Rating** | Low | **Risk Priority Number** | 4 |
| **Detectability Rating** | Very High | **Risk Analysis Outcome** | Monitor - No immediate actions (based on RPN) |

**Severity Rationale**

As this is a deviation from acceptance discussed with the sponsor, and it is easily detectable, the severity is minor. There is no impact on stability since data is only considered acceptable if it is within 15% of the Time 0 data (or within 15% bias from nominal per SOP).

**Mitigation**

**Document Number** | **Document Revision**

## Deviation Impact

| Question | Response | Comment |
|---|---|---|
| Are there any compliance issues? | No | Stability will not be accepted as passing if >15% difference to Time 0 data. |
| Are there any known impacts on product quality? | No | Stability will not be accepted as passing if >15% difference to Time 0 data. |

**Final Classification** Minor

| | |
|---|---|
| **Justification** | This was done in discussion with the client and data is not accepted as passing if it is >15% difference to the Time 0 data. |

## Investigation Approval

| | | | | |
|---|---|---|---|---|
| **Approval Decision** | Approve | | | |
| **Comment** | | | | |
| **Signature** | LUNAM | Marsha Luna | 21-Mar-2023 13:21:22 | [GMT] |
| **Approval Decision** | Approve | | | |
| **Comment** | | | | |
| **Signature** | PIPPINA | Anika Pippin | 21-Mar-2023 15:21:04 | [GMT] |

## Action Plan (2)

| Level | Action Plan | Completed By | Completed Date | Verification Required? | Added In |
|---|---|---|---|---|---|
| 1 | Discussion with Sponsor | PIPPINA<br>Anika Pippin | 16-Mar-2023 | No | Investigation |

| | | | | | |
|---|---|---|---|---|---|
| **Action Plan Description** | Immediate action taken was the discussion with the sponsor regarding testing approach and re-preparation. | | | | |
| **Assigned to Role** | DEV OWNER | DEV OWNER | **Assigned to User** | PIPPINA | Anika Pippin |
| **Checklist** | | | **Due (# business days)** | 1 | |
| **Is Plan Complete?** | Yes | | **Due Date** | | |
| **Comment** | Sponsor communication happened previously. | | | | |
| **Signature** | | | | | [GMT] |

| Level | Action Plan | Completed By | Completed Date | Verification Required? | Added In |
|---|---|---|---|---|---|
| 2 | Monitoring via %Change to Time 0 | PIPPINA<br>Anika Pippin | 16-Mar-2023 | No | Investigation |

| | | | | | |
|---|---|---|---|---|---|
| **Action Plan Description** | Per sponsor input, stability is monitored via comparison to the Time 0 data. In addition, another Comed QC was prepared and verified (it did not run as high) if needed for future stability assessments. | | | | |
| **Assigned to Role** | DEV OWNER | DEV OWNER | **Assigned to User** | PIPPINA | Anika Pippin |
| **Checklist** | | | **Due (# business days)** | 1 | |
| **Is Plan Complete?** | Yes | | **Due Date** | | |

**Comment**: All actions completed

**Signature** [GMT]

## Ad Hoc Task ()

| Task Name | Due Date | Completed By | Completed Date |
|---|---|---|---|

**Assigned to Role** **Assigned to User**

**Instruction**

**Completion Comment**

**Signature** [GMT]

## CAPA Escalation

**CAPA Escalation** No **CAPA Number**

**Rationale**

## Closure

| Question | Response | Comment |
|---|---|---|
| Are there any known impacts to the customer? | NA | |
| Are there any issues which would possibly allow for a reoccurrence of this deviation? | NA | |

**Final Risk Assessment Required?** No

**Closure Summary** QA Reviewed

## QA Closure Approval

**Approval Decision**

**Comment**

**Signature** [GMT]

# Additional Information

## Attachments (1)

| File Name | Description | Attached By | Attached Date | Task Name |
|---|---|---|---|---|
| _External__RE_ Repeat Data for 1 month Comed QC LTS (1 month_ V1872106P5).msg | Sponsor email showing that Comed LTS (low) is to be conducted from the original prep and %bias to Time 0 should be reported along with the %bias to nominal. | PIPPINA | 16-Mar-2023 | Initiate Deviation |

## Record Cross Reference ()

| Referenced Record Number | Relation Type | Referenced Entity Name |
|---|---|---|

## Policy

| | | | |
|---|---|---|---|
| **Risk based on** | Risk Priority Number | | |
| **QA Review Required?** | No | **Containment Required?** | No |
| **Investigation Required?** | Yes | **Disposition Required?** | No |
| **Investigation Approval Required?** | Yes | **QA Closure Required?** | No |

## Due Date (# of business days)

| | | |
|---|---|---|
| **Verification Due** | 1 | days from Initiation Date |
| **Investigation Due** | 10 | days from Verification or QA Review Completion Date |
| **Investigation Approval Due** | 1 | days from Investigation or the previous approval Completion Date |
| **Closure Verification Due** | 2 | days from Completion Date of all applicable tasks |

## Due Date Extension Request ()

| Extension Requested for | Original Due Date | Proposed Due Date | Request Status | Requested By |
|---|---|---|---|---|

**Reason for Extension**

### Approval Result

| Action | Comment | Signature |
|---|---|---|
| | | [GMT] |

## Team

| Task | Role | | User | | Level |
|---|---|---|---|---|---|
| Investigate Deviation | DEV INVESTIGATOR | DEV INVESTIGATOR | PIPPINA | Anika Pippin | N/A |
| Verify Deviation Closure | DEV COORDINATOR | DEV COORDINATOR | PIREZM | Monica Pirez | N/A |
| Deviation Owner | DEV OWNER | DEV OWNER | PIPPINA | Anika Pippin | N/A |
| Approve Deviation Investigation | DEV APPROVER | DEV APPROVER | LUNAM | Marsha Luna | 10 |
| Approve Deviation Investigation | DEV APPROVER | DEV APPROVER | PIPPINA | Anika Pippin | 15 |
| Approve Due Date Extension | DEV APPROVER | DEV APPROVER | LUNAM | Marsha Luna | 20 |

## Activity (12)

| Task Name | Action | Signature |
|---|---|---|
| Initiate Deviation | | PIPPINA<br>16-Mar-2023 18:24:44 [GMT] |
| **Comment** | | |
| Verify Deviation | | PIPPINA<br>16-Mar-2023 18:30:25 [GMT] |
| **Comment** | | |
| Investigate Deviation | | PIPPINA<br>16-Mar-2023 18:54:58 [GMT] |
| **Comment** | | |
| Approve Deviation Investigation | Approve | LUNAM<br>16-Mar-2023 19:01:22 [GMT] |
| **Comment** | | |
| Update Team | | PIREZM<br>16-Mar-2023 19:34:15 [GMT] |
| **Comment** | Updated team and removed PirezM from Approve Deviation Investigation as QA only reviews once it is at the Verify Deviation Closure step. Deviation will be QA reviewed once it gets to that step | |
| Approve Deviation Investigation | Reject | PIREZM<br>17-Mar-2023 13:16:01 [GMT] |

| | | | |
|---|---|---|---|
| **Comment** | Please update team to include Approve Deviation Investigation to be routed to management, not QAU. QAU only reviews at Verify Deviation Closure step. | | |
| Investigate Deviation | | PIPPINA<br>21-Mar-2023 13:17:45 | [GMT] |
| **Comment** | | | |
| Approve Deviation Investigation | Approve | LUNAM<br>21-Mar-2023 13:21:22 | [GMT] |
| **Comment** | | | |
| Approve Deviation Investigation | Approve | PIPPINA<br>21-Mar-2023 15:21:04 | [GMT] |
| **Comment** | | | |
| Waiting for Closure | | PIPPINA<br>21-Mar-2023 15:21:06 | [GMT] |
| **Comment** | | | |
| Change Task Owner | Old Task Owner : PIREZM | PIREZM<br>21-Mar-2023 15:54:26 | [GMT] |
| **Comment** | updated deviation reviewer | | |
| Verify Deviation Closure | | ANDERSONC<br>21-Mar-2023 17:09:56 | [GMT] |
| **Comment** | QA Reviewed | | |

# 1. TITLE: THE DETERMINATION OF TOTAL ERTAPENEM IN TREATED HUMAN PLASMA, WITH SODIUM HEPARINAS ANTICOAGULANT, BY LC-MS/MS

## BIOANALYTICAL METHOD VALIDATION REPORT

| | |
|---|---|
| Bioanalytical Project Number: | V1872106P5 |
| Sponsor: | DynPort Vaccine Company, LLC<br>110 Thomas Johnson Drive<br>Suite 200<br>Frederick, MD 21702 |
| Bioanalytical Laboratory: | KCAS Bioanalytical & Biomarker Services.<br>10830 S Clay Blair Boulevard<br>Olathe, KS 66061 |
| Issue Date: | March 2023 |

## 2. SYNOPSIS

This report presents the validation data for an LC-MS/MS method for the determination of total ertapenem in treated human plasma, with sodium heparin as anticoagulant. The assay was analyzed in the range of 0.200 - 200 mcg/mL and is for use on human plasma samples obtained during clinical trials.

# 3. TABLE OF CONTENTS FOR THE BIOANALYTICAL METHOD VALIDATION REPORT


1. TITLE: THE DETERMINATION OF TOTAL ERTAPENEM IN TREATED HUMAN PLASMA, WITH SODIUM HEPARINAS ANTICOAGULANT, BY LC-MS/MS ....1
2. SYNOPSIS ....2
3. TABLE OF CONTENTS FOR THE BIOANALYTICAL METHOD VALIDATION REPORT ....3
4. LIST OF TABLES ....5
5. LIST OF FIGURES ....6
6. SIGNATURE PAGE ....7
7. QUALITY ASSURANCE STATEMENT ....8
8. STUDY COMPLIANCE STATEMENT ....9
9. LIST OF ABBREVIATIONS AND DEFINITIONS OF TERMS ....10
10. INTRODUCTION ....12
11. EXPERIMENTAL DESIGN AND RESULTS ....12
11.1. Reference Standards ....12
11.2. Calibration Standards and QC Samples Preparation ....12
11.3. Selectivity ....13
11.3.1. Blank Evaluation ....13
11.3.2. Matrix Effect ....13
11.3.3. Interference Evaluation ....14
11.3.4. Matrix Evaluation ....14
11.3.5. Carryover ....14
11.4. Linearity and Analytical Range ....14
11.5. Precision and Accuracy ....15
11.6. Sensitivity ....15
11.7. Run Size ....16
11.8. Precision and Accuracy of Dilution ....16
11.9. Freeze/Thaw Stability - Treated ....16

11.10. Freeze/Thaw Stability - Untreated ....17
11.11. Bench-Top Stability in Wet Ice - Treated....17
11.12. Bench-Top Stability in Wet Ice - Untreated ....17
11.13. Long-Term Matrix Stability at -20 °C and -70 °C - Treated ....17
11.14. Long-Term Matrix Stability at -20 °C and -70 °C - Untreated ....18
11.15. Stability During Blood Collection and Processing....19
11.16. Processed Sample Stability at Room Temperature....19
11.17. Processed Sample Stability when Refrigerated ....19
11.18. Individual Sample Reinjection Reproducibility ....20
11.19. Whole Run Reinjection Reproducibility ....20
11.20. Column Ruggedness ....20
11.21. Extraction Recovery ....20
11.22. Solution Stabilities....21
11.23. Calculations ....21
11.24. SOP Deviations and Investigations ....21
12. CONCLUSION....22
13. PERTINENT SOPS ....22
14. ARCHIVES ....23
15. REFERENCES ....23
16. TABLES ....24
17. FIGURES....66

# 4. LIST OF TABLES

Table 1: Abbreviations and Codes ....10
Table 2: Deviations ....22
Table 3: Mass Spec Transitions ....24
Table 4: Reference Standards ....24
Table 5: Calibration Standards and QC Samples Preparation ....25
Table 6: Matrix Description ....28
Table 7: Summary of Results and Conclusions ....29
Table 8: Summary of Validation Run Objectives and Outcome ....33
Table 9: Carryover ....35
Table 10: Selectivity - Blank Assessment ....36
Table 11: Matrix Effect ....37
Table 12: Interference Evaluation ....38
Table 13: Hemolytic Effect Evaluation ....39
Table 14: Lipemic Effect Evaluation ....40
Table 15: Summary of Standard Curves ....41
Table 16: Precision and Accuracy (Intra-Assay) ....42
Table 17: Precision and Accuracy (Inter-Assay) ....43
Table 18: Standard and QC of Failed Runs ....44
Table 19: Standards and QCs of Non-P&A Runs ....45
Table 20: Precision and Accuracy of Sample Dilution ....48
Table 21: Freeze-Thaw Stability, (Frozen At -20 °C and -70 °C Thawed in Wet Ice) - Treated ....49
Table 22: Freeze-Thaw Stability, (Frozen At -70 °C Thawed in Wet Ice) - Untreated ....50
Table 23: Bench-Top Stability in Wet Ice - Treated ....51
Table 24: Bench-Top Stability in Wet Ice - Untreated ....52
Table 25: Long-Term Stability at -20 °C and -70 °C - Treated ....53
Table 26: Long-Term Stability at -70 °C - Untreated ....54
Table 27: Stability Evaluation During Blood Collection and Processing ....57

Table 28: Processed Sample Stability at Room Temperature ....58
Table 29: Refrigerated Processed Sample Stability ....58
Table 30: Individual Sample Reinjection Reproducibility ....59
Table 31: Whole Run Reinjection Reproducibility and Column Ruggedness ....60
Table 32: Extraction Recovery – Ertapenem ....61
Table 33: Extraction Recovery – Ertapenem-$d_4$ ....62
Table 34: Stability of Ertapenem Primary Solution in MES Buffer at Room Temperature ....63
Table 35: Stability of Ertapenem Primary Solution in MES Buffer at -20 °C ....64
Table 36: Stability of Ertapenem Primary Solution in MES Buffer at -20 °C ....65

## 5. LIST OF FIGURES

Figure 1: Typical Calibration Curve ....66
Figure 2: Typical Ion Chromatogram Blank Human Plasma Without Internal Standard ....67
Figure 3: Typical Ion Chromatogram Blank Human Plasma With Internal Standard ....68
Figure 4: Typical Ion Chromatogram LLOQ Calibration Standard, Ertapenem = 0.200 mcg/mL ....69
Figure 5: Typical Ion Chromatogram Calibration Standard, Ertapenem = 80.0 mcg/mL ....70
Figure 6: Typical Ion Chromatogram ULOQ Calibration Standard, Ertapenem = 200 mcg/mL ....71
Figure 7: Typical Ion Chromatogram Cross-Talk Evaluation High QC No IS ....72

Annex 1 .... Test Procedure
Annex 2 .... Certificate(s) of Analysis
Annex 3 .... Interference Testing

# 6. SIGNATURE PAGE

Bioanalytical Project Number: V1872106P5

Issue Date: March 2023

**APPROVED**

DocuSigned by:
Anika Pippin
Signer Name: Anika Pippin
Signing Reason: I approve this document
Signing Time: 10-Mar-2023 | 13:01 CST
BE3AA1198E40459E8A7E3C615D981D82

Anika Pippin, B.S.
Principal Investigator
02Feb2023-Present

Eric Johnson
Principal Investigator
02Nov2022-01Feb2023

Yu-Hui Ann Fu
Principal Investigator
05Aug2022-01Nov2022

**REVIEWED**

DocuSigned by:
Marsha Luna
Signer Name: Marsha Luna
Signing Reason: I have reviewed this document
Signing Time: 10-Mar-2023 | 12:38 CST
C9CC738561AF4CC1B375ED013DD1E4BD

Marsha Luna, B.S.
Management

# 7. QUALITY ASSURANCE STATEMENT

This Bioanalytical Method Validation Report has been found to describe accurately the methods and SOPs used and to accurately reflect the raw data collected. The Quality Assurance Unit initiated audits on this study and reported the findings to the Principal Investigator (PI) and Management as follows:

| | | **Date(s) Sent to:** | |
|---|---|---|---|
| **Inspection Date(s)** | **Inspection Phase** | **PI** | **Management** |
| 29Nov2022 | Data Audit | 29Nov2022 | 20Feb2023 |
| 05Dec2022 | Draft Report Review | 05Dec2022 | 17Feb2023 |
| 20Jan2023 | Data Audit | 20Jan2023 | 20Jan2023 |
| 10Feb2023 | Data Audit | 10Feb2023 | 20Feb2023 |
| 16Feb2023 | Data Audit | 16Feb2023 | 03Mar2023 |
| 16Feb2023 | Data Audit | 16Feb2023 | 16Feb2023 |
| 23Feb2023 | Data Audit | 23Feb2023 | 23Feb2023 |
| 01Mar2023 | Data Audit | 01Mar2023 | 01Mar2023 |
| 07Mar2023 | Updated Draft Report Review | 07Mar2023 | 07Mar2023 |
| 10Mar2023 | Final Report Review | 10Mar2023 | 10Mar2023 |

**REVIEWED**

DocuSigned by:

Mikayla Griesbauer

Signer Name: Mikayla Griesbauer
Signing Reason: I have reviewed this document
Signing Time: 10-Mar-2023 | 10:36 PST
9630D9A8DCC440BCBA93F86194868A8B

Mikayla Griesbauer, B.S.
Quality Assurance Unit

# 8. STUDY COMPLIANCE STATEMENT

I, the undersigned, hereby declare that this study was conducted in compliance with the relevant bioanalytical aspects of FDA GLP regulations 21CFR Part 58, according to the FDA Guidance for Bioanalytical Method Validation and KCAS Bioanalytical & Biomarker Services (KCAS) procedures and that the work was performed by me, or under my direction, and the report reflects a true and accurate record of the results obtained.

The acceptance criteria are based on the FDA Guidance for Industry, Bioanalytical Method Validation (2018). [1]

Hemolytic evaluation failed at 1% and 2%. Hemolytic assessment did pass at 0.5%, and samples will be evaluated upon receipt.

**APPROVED**

DocuSigned by:

Anika Pippin

Signer Name: Anika Pippin
Signing Reason: I approve this document
Signing Time: 10-Mar-2023 | 13:01 CST
BE3AA1198E40459E8A7E3C615D981D82

---

Anika Pippin, B.S.
Principal Investigator
02Feb2023-Present

Eric Johnson
Principal Investigator
02Nov2022-01Feb2023

Yu-Hui Ann Fu
Principal Investigator
05Aug2022-01Nov2022

# 9. LIST OF ABBREVIATIONS AND DEFINITIONS OF TERMS

**Table 1: Abbreviations and Codes**

| Abbreviation | Definition |
|---|---|
| < | Concentration less than the lowest acceptable calibration standard |
| > | Concentration exceeded the upper limit of calibration range (> ULOQ) |
| A | Amendment |
| AQL | Above Quantitation Limit |
| %Bias | Percentage measurement valid |
| Batch | Is the same as Run |
| BQL | Below Quantifiable Limit |
| Cal | Calibration standard |
| Conc. | Concentration |
| Conv. | Conversion |
| %CV | Coefficient of Variation |
| dev | Deviation |
| diff | Difference |
| EMA | European Medicines Agency |
| FDA | Food and Drug Administration |
| GLP | Good Laboratory Practice |
| HPLC | High Performance Liquid Chromatography |
| ID | Identification |
| IS | Internal Standard |
| ISR | Incurred Sample Reanalysis |
| $K_2EDTA$ or $K_3EDTA$ | Dipotassium or Tripotassium Ethylenediaminetetraacetic Acid |
| KCAS | KCAS Bioanalytical & Biomarker Services |
| LC-MS/MS | Liquid Chromatography Tandem Mass Spectrometry |
| LLOQ | Lower Limit of Quantitation |
| mcg or µg or ug | Microgram |
| mcL or µL or uL | Microliter |

**Table 1: Abbreviations and Codes (Continued)**

| Abbreviation | Definition |
|---|---|
| ME | Matrix Effect |
| MF | Matrix Factor |
| MHLW | Ministry of Health, Labour and Welfare of Japan |
| mL | Milliliter |
| MS | Mass Spectrometry |
| n | number |
| N.C. | Not Calculated |
| ND | No difference reported as ISR will be counted |
| ng | nanogram |
| n.report | No Data available to report |
| N/A or NA | Not Applicable |
| N.R. | Not Reported |
| P&A | Precision and Accuracy |
| p.a.r. | Peak Area Ratio |
| PI | Principal Investigator |
| QC | Quality Control Sample |
| QCs | Quality Control Samples |
| OECD | Organization for Economic Co-operation and Development |
| RT | Room Temperature |
| sd or SD | Standard deviation |
| Seq | Sequence |
| SOP (s) | Standard Operating Procedure (s) |
| temp | Temperature |
| TP | Test procedure |
| UHPLC | Ultra High Performance Liquid Chromatography |
| ULOQ | Upper Limit of Quantitation |
| WIS | Working Internal Standard |

This is a basic list. Some of the abbreviations may not have been used in this report.

# 10. INTRODUCTION

The objective of this study was to perform a full validation of a bioanalytical method for the determination of total ertapenem in treated human plasma, with sodium heparin as anticoagulant, to support the Phase 1 clinical trial studying the safety, tolerability and pharmacokinetics of intravenous ertapenem in combination with zidebactam (WCK 6777) in healthy adult subjects. This validation report describes the selectivity, sensitivity, linearity, accuracy, precision, stability and recovery experiments based on an established bioanalytical test procedure.

The validation procedure was designed to meet or exceed the requirements of FDA Guidance for Industry on Bioanalytical Methods Validation, May 2018. The software programs used were Microsoft Office Excel 365, acquisition and processing software Analyst 1.7.2, and data management software Watson LIMS 7.5.

# 11. EXPERIMENTAL DESIGN AND RESULTS

In this method, ertapenem and internal standards, ertapenem-$d_4$, were released from treated human plasma using 96 well protein precipitation. After extraction, the sample was chromatographed using reversed phase HPLC with a Fortis C18 analytical column. Ertapenem and ertapenem-$d_4$ (IS) were detected by monitoring the precursor and product ions described in Table 3 using an Applied Biosystems Sciex API 6500 LC-MS/MS. The quantification was based on setting a calibration graph using the internal standard method based on bioanalytical test procedure TP-80193, entitled "LC-MS/MS Analysis of Ertapenem in Human Sodium Heparin Plasma (100 mcL, 0.200 - 200 mcg/mL; Prior to MES Treatment)". The test procedure can be found in Annex 1.

The human plasma is treated with 0.1M 2-(N-Morpholino) ethane sulphonic acid hydrate (MES) buffer, pH 6.4 at a 1:1 ratio. The validation assay range is calculated based on plasma volume only to mimic unknown study samples. The assay range is 0.200 to 200 mcg/mL in untreated human sodium heparin plasma.

A summary of the validation results is provided in Table 7. A summary describing the objective(s) and outcome of each validation run attempted is provided in Table 8.

The method was validated within the analytical range of 0.200 - 200 mcg/mL in human plasma. No development work was performed after the method was validated.

## 11.1. Reference Standards

Reference standards used during this validation are presented in Table 4.

## 11.2. Calibration Standards and QC Samples Preparation

Calibration standards and quality control pools were prepared by spiking appropriate levels of ertapenem into treated human plasma, with sodium heparin as anticoagulant. Primary reference

standards were prepared in at least duplicate, and they need to compare within 5% of each other. Standards and QCs were prepared and stored frozen at KCAS at -70 °C. Stability QCs were also stored at -20 °C and -70 °C. Standards, QCs, and the critical solutions used during this validation are described in Table 5, and matrix used is described in Table 6.

## 11.3. Selectivity

Selectivity is the ability of the bioanalytical method to measure and differentiate ertapenem in the presence of components that may be expected to be present. These could include metabolites, impurities, degradants, or matrix components. The selectivity of this method was achieved by a sample clean-up procedure using protein precipitation followed by chromatography and specific detection of the precursor and product ions of each analyte using tandem mass spectrometry (MS/MS).

### 11.3.1. Blank Evaluation

A minimum of six different sources of plasma (sodium heparin as anticoagulant) were screened. To be deemed acceptable, the peak response observed must be ≤ 20.0% of the mean response of the lower limit of quantitation (LLOQ) and ≤ 5.0% of the mean response of the internal standard. If any lots fail, further evaluation is needed.

All matrices lots were found to be free of any significant interference at the retention times of the analytes of interest. Data is presented in Table 10. Typical chromatograms for blank plasma, with sodium heparin as anticoagulant, without and with internal standard are presented in Figure 2 and Figure 3, respectively. Cross talk was also evaluated by extracting the high QC with no internal standard. There are no apparent effects of cross talk, and a typical chromatogram is present in Figure 7.

### 11.3.2. Matrix Effect

Matrix effect in MS applications is defined as inter-matrix variation in signal due to co-eluting matrix substances which cause enhancement or suppression of the detector response for the measured analyte.

To evaluate matrix effect, six QC pools were prepared at approximately 3 times the LLOQ and at the high QC concentration, each from a different lot of human plasma, with sodium heparin as anticoagulant. These QC pools were each extracted in replicates of six according to the test procedure, and the mean response ratios were calculated for each matrix. To be deemed free of matrix effect, the %CV of the mean ratios across sources was required to be ≤ 15.0%.

Matrix effect data are presented in Table 11. The matrix effect results meet acceptance criteria.

Note: matrix factor was not used for this determination as outlined by the validation plan. This alternative approach is supported by SOP-00035 and is preferred due to the instability of the drug and the plasma being treated with 0.1M MES buffer (1:1).

#### 11.3.3. Interference Evaluation

Assay selectivity was confirmed by spiking quality control pools with 37.5 mcg/mL zidebactam (based on plasma volume only). These QC pools, containing low and high levels of ertapenem, were analyzed in replicates of six in a single run. To be considered acceptable, the mean %Bias at each level is required to be within ± 15.0% and the %CV is required to be ≤ 15.0%.

The results presented in Table 12, indicate the presence of zidebactam does not affect the accuracy or precision of the data.

Additional interference testing not necessary – see Annex 3 from the Sponsor.

#### 11.3.4. Matrix Evaluation

To evaluate different types of collected matrix, QC pools were prepared with treated hemolytic (0.5%, 1%, and 2% hemolysis) and lipemic human plasma, with sodium heparin as anticoagulant, at approximately three times the LLOQ and the high QC concentration. These QC pools were each extracted in at least replicates of three, and the mean concentrations or responses were calculated for each matrix. To be considered acceptable, the mean %Bias at each level was also required to be within ± 15.0% and the %CV was required to be ≤ 15.0%.

Low hemolytic QCs failed in run 2 and was repeated in run 7. Hemolytic QCs at 1% and 2% do not meet acceptance criteria. The acceptance criteria were met for hemolytic QCs at 0.5%. The results, presented in Table 13 to Table 14, indicate that hemolytic (0.5% hemolysis) and lipemic plasma does not affect the analysis of ertapenem in treated human plasma. Study samples will be evaluated upon receipt and noted in the data due to failure at 1% and 2% hemolysis.

Furthermore, blank lipemic and hemolytic control plasma were extracted with and without internal standard to determine that there were no significant interferences observed at the retention times of the analytes. There were no significant interferences observed.

#### 11.3.5. Carryover

Carryover is evaluated within each run. It consists of measuring the response for each analyte and if it exceeds 20.0% of the response of LLOQ standard or other sample reflecting the lower sensitivity limit required, a carryover assessment is completed, and affected samples are rejected. The internal standard carryover evaluation is assessed within each run and if it exceeds 5.0% of the average internal standard response of acceptable standards and QCs, impact is evaluated within the run. Data is presented in Table 9.

### 11.4. Linearity and Analytical Range

The calibration curve was constructed with eight different, non-zero standard concentrations ranging from 0.100 - 100 mcg/mL in treated human plasma (equivalent to 0.200 to 200 mcg/mL in untreated human plasma), with sodium heparin as anticoagulant. Calibration standards were processed in duplicate and the calibration curve was evaluated using least squares linear

regression with weighting $1/x^2$. The maximum allowable percent deviation from nominal for each standard was ± 15.0%, except at the LLOQ, where ± 20.0% was accepted. Any standard deviating by more than these limits was required to be excluded from the regression line. For the calibration curve to be accepted, at least 75% of the initial number of standards was required to remain in the curve.

The calibration curve was found to be linear. The data are presented in Table 15. A typical calibration curve is presented graphically in Figure 1. Typical chromatograms illustrating blank human plasma standards spiked with ertapenem at the LLOQ, mid, and high concentrations are presented in Figure 4 to Figure 6, respectively.

## 11.5. Precision and Accuracy

The precision of an assay is defined as the closeness in which replicate analyses agree and is reported as percent coefficient of variation (%CV). The accuracy of a method is defined as the closeness of a determined value to its true (nominal) value and is reported as %Bias. Inter-assay (between runs) precision & accuracy and intra-assay (within runs) precision & accuracy were assessed in four runs by analyzing six replicates each of QCs fortified at four separate concentrations (LLOQ, low, mid, and high). For inter- and intra-assay, the mean %Bias at each QC level was required to be within ±15.0% from its respective nominal concentration, except at the LLOQ, where ± 20.0% was accepted. The %CV at each QC level was required to be ≤ 15.0%, except at the LLOQ, where ≤ 20.0% was accepted.

The intra-assay and inter-assay precision and accuracy data for this method met the required criteria and are presented in Table 16 (intra-assay precision and accuracy) and Table 17 (inter-assay precision and accuracy).

One additional P&A run was done to comply with the requirement that calibration standards and QCs have to be prepared from two separate stock solutions on at least one occasion. This was done for the fourth P&A run.

Failed runs are reported in Table 18. Run 12 failed due to large %CV in the QCs throughout the run. Run 12 was reinjected as run 13.

Standards and QCs of non-P&A runs are reported in Table 19.

## 11.6. Sensitivity

The sensitivity of the method was determined in four runs by analyzing six replicates of QCs at the lower limit of quantitation (LLOQ) which were equal in concentration to the lowest calibration standard. The observed response at the LLOQ was required to be at least 5 times the response of noise in the human plasma blank, in addition to the precision and accuracy requirements outlined in the previous section.

The LLOQ for this method was determined to be 0.200 mcg/mL, and the response at the LLOQ was at least 5 times the response of noise in the human matrix blank. Data are presented in Table 16 (intra-assay precision and accuracy) and Table 17 (inter-assay precision and accuracy).

## 11.7. Run Size

In order to evaluate the largest size run that can be used for study sample analysis, a run was processed with a set of 8 calibration standards and a minimum of 3 QC samples, processed in duplicate. In addition, at least 3 matrix blanks (one with internal standard and two without internal standard) were processed with the run. Run 2 was used for this assessment, and additional experiments were also analyzed which made the final run size 120.

In order for the run to be acceptable, at least one QC at each concentration level and two-thirds of the total number of QC samples were required to interpolate within ± 15.0% from nominal.

The standard and QC summary of run 2 is presented in Table 15 and Table 16 and acceptance criteria was met.

## 11.8. Precision and Accuracy of Dilution

To determine the accuracy of dilution, the high concentration QC spiked with ertapenem (150 mcg/mL) as well as an above-range QC (ARQC; 800 mcg/mL) were evaluated. All samples were extracted in six replicates according to the test procedure. To be deemed acceptable, the mean %Bias at each level must be within ± 15.0% with %CV ≤ 15.0%. The replicates were prepared as follows:

- Dilution factor 2x (high QC) = 50 mcL of sample+ 50 mcL of blank (treated) plasma
- Dilution factor 5x (ARQC) = 20 mcL of sample+ 80 mcL of blank (treated) plasma

Dilution precision and accuracy, using dilution factors of 2x and 5x, were proven to be valid. Data are presented in Table 20.

## 11.9. Freeze/Thaw Stability - Treated

Ertapenem stability in treated human plasma with and without the presence of comed[2], with sodium heparin as anticoagulant, during sequential freeze/thaw cycles (frozen separately at -20 °C and -70 °C and thawed unassisted in wet ice) was evaluated. Aliquots of high and low concentration QC samples were frozen for at least 12 hours before the initial thawing. They were re-frozen 3 more times for at least 12 hours each time and were thawed prior to assay along with a freshly prepared set of QCs and calibration standards. To be deemed stable, the mean %Bias at each level must be within ± 15.0% with %CV ≤ 15.0%.

Ertapenem is stable in treated human plasma with and without the presence of comed[2], with sodium heparin as anticoagulant, for 4 cycles of freeze (-20 °C and -70 °C) and thaw (wet ice).

Data are provided in Table 21.

## 11.10. Freeze/Thaw Stability - Untreated

Ertapenem stability in untreated human plasma with and without the presence of comed[2], with sodium heparin as anticoagulant, during sequential freeze/thaw cycles (frozen separately at -70 °C and thawed unassisted in wet ice) was evaluated. Aliquots of high and low concentration QC samples were frozen for at least 12 hours before the initial thawing. They were re-frozen 1 more time for at least 12 hours each time and were thawed prior to assay along with a freshly prepared set of QCs and calibration standards. To be deemed stable, the mean %Bias at each level must be within ± 15.0% with %CV ≤ 15.0%.

Ertapenem is stable in untreated human plasma with and without the presence of comed[2], with sodium heparin as anticoagulant, for 2 cycles of freeze (-70 °C) and thaw (wet ice).

Data are provided in Table 22.

## 11.11. Bench-Top Stability in Wet Ice - Treated

To evaluate ertapenem in treated human plasma with and without the presence of comed[2], with sodium heparin as anticoagulant, stability during sample processing in wet ice, aliquots of the high and low concentration QC pools were allowed to thaw at ambient conditions for 8.75 hours, prior to analysis along with a freshly prepared set of QCs and calibration standards. To be deemed stable, the mean %Bias at each level must be within ± 15.0% with %CV ≤ 15.0%.

Ertapenem in treated human plasma with and without the presence of comed[2], with sodium heparin as anticoagulant, was proven stable in wet ice under normal laboratory lighting over a period of 8.75 hours.

Data are provided in Table 23.

## 11.12. Bench-Top Stability in Wet Ice - Untreated

To evaluate ertapenem in untreated human plasma with and without the presence of comed[2], with sodium heparin as anticoagulant, stability during sample processing in wet ice, aliquots of the high and low concentration QC pools were allowed to thaw at ambient conditions for 4 hours, prior to analysis along with a freshly prepared set of QCs and calibration standards. To be deemed stable, the mean %Bias at each level must be within ± 15.0% with %CV ≤ 15.0%.

Ertapenem in untreated human plasma with and without the presence of comed[2], with sodium heparin as anticoagulant, was proven stable in wet ice under normal laboratory lighting over a period of 4 hours.

Data are provided in Table 24.

## 11.13. Long-Term Matrix Stability at -20 °C and -70 °C - Treated

To evaluate ertapenem in treated human plasma with the presence of comed[2], with sodium heparin as anticoagulant, stability during sample storage, aliquots of the high and low

concentration QC samples were stored frozen at -20 °C and -70 °C nominally for 32 and 38 days prior to being analyzed, along with freshly prepared standards and QC samples. Runs 13 and 16 contained samples that were not handled correctly by flash-freezing upon preparation so they will not be reported.

To evaluate ertapenem in treated human plasma without the presence of comed[2], with sodium heparin as anticoagulant, stability during sample storage, aliquots of the high and low concentration QC samples were stored frozen at -20 °C and -70 °C nominally for 24 days prior to being analyzed, along with freshly prepared standards and QC samples. Runs 13 and 16 contained samples that were not handled correctly by flash-freezing upon preparation so they will not be reported.

To be deemed stable, the mean %Bias at each level must be within ± 15% with %CV ≤ 15%.

Ertapenem in treated human plasma with comed[2], with sodium heparin as anticoagulant, was proven to be unstable at -20 °C in polypropylene tubes. Ertapenem in treated human plasma with comed[2], with sodium heparin as anticoagulant, was proven to be stable at -70 °C over a period of 38 days in polypropylene tubes. Run 23 (32 days) -70 °C failed due to high %Bias and this was verified to not be a stability issue in run 24. The failure was determined to be due to high %bias of the Time 0 preparation. LTS will be evaluated against nominal concentration as well as %change to the Time 0 values.

Ertapenem in treated human plasma without comed[2], with sodium heparin as anticoagulant, was proven to be unstable at -20 °C in polypropylene tubes. Ertapenem in treated human plasma, with sodium heparin as anticoagulant, was proven to be stable at -70 °C over a period of 24 days in polypropylene tubes.

Data are provided in Table 25.

## 11.14. Long-Term Matrix Stability at -20 °C and -70 °C - Untreated

To evaluate ertapenem in untreated human plasma with the presence of comed[2], with sodium heparin as anticoagulant, stability during sample storage, aliquots of the high and low concentration QC samples were stored frozen at -70 °C nominally for 4, 7, 14, 24, 29 days prior to being analyzed, along with freshly prepared standards and QC samples. Runs 17 contained samples that were not handled correctly by flash-freezing upon preparation so they will not be reported.

To evaluate ertapenem in untreated human plasma without the presence of comed[2], with sodium heparin as anticoagulant, stability during sample storage, aliquots of the high and low concentration QC samples were stored frozen at -70 °C nominally for 10, 15, 23, 28 days prior to being analyzed, along with freshly prepared standards and QC samples.

To be deemed stable, the mean %Bias at each level must be within ± 15% with %CV ≤ 15%.

Ertapenem in untreated human plasma without comed[2], with sodium heparin as anticoagulant, was proven to be stable at -70 °C over a period of 28 days in polypropylene tubes.

Ertapenem in untreated human plasma with comed[2], with sodium heparin as anticoagulant, was proven to be stable at -70 °C over a period of 29 days in polypropylene tubes.

Data are provided in Table 26.

## 11.15. Stability During Blood Collection and Processing

Stability of ertapenem in whole blood, with sodium heparin as anticoagulant, was assessed by spiking whole blood at a mid-concentration. These samples were stored immediately at room temperature and in an ice-water bath for 0 hour and at least 60 minutes in polyethylene terephthalate containers. After the designated time, the plasma was harvested, treated with chilled MES Buffer pH 6.4 at 1:1 ratio, and extracted. The % change was calculated with stored data comparing to the 0-hour data. To be considered stable, the % change between stored sample pools and the control 0-hr sample pools must be within ± 15.0%, with %CV ≤ 15.0%.

Ertapenem was shown stable during blood collection processing for up to 60 minutes stored in an ice-water bath or at room temperature in polyethylene terephthalate containers.

Data are provided in Table 27.

## 11.16. Processed Sample Stability at Room Temperature

To evaluate ertapenem stability in processed samples at room temperature, high and low concentration QC samples were processed, placed into polypropylene 96 well plates, stored, and allowed to remain at ambient conditions (~25 °C) before being chromatographed with samples in a subsequent run. To be deemed stable, the mean %Bias at each level must be within ± 15.0% with %CV ≤ 15.0%.

Processed ertapenem samples were proven stable at room temperature stored in polypropylene 96 well plates over a period of 3 days 20 hours. Data are provided in Table 28.

## 11.17. Processed Sample Stability when Refrigerated

To evaluate ertapenem stability in processed samples at refrigerated temperatures, high and low concentration QC pools were processed, placed into polypropylene 96 well plates, and stored at refrigerated temperatures (~4 °C) before being chromatographed with samples in a subsequent run. To be deemed stable, the mean %Bias at each level must be within ± 15.0% with %CV ≤ 15.0%.

Processed ertapenem samples were proven stable when refrigerated (~4 °C) stored in polypropylene 96 well plates over a period of 3 days 20 hours. Data are provided in Table 29.

## 11.18. Individual Sample Reinjection Reproducibility

To evaluate individual sample reinjection reproducibility, low and high QC extracts (6 each) injected during the run were reinjected at the end of the run, within the established stability time window of processed samples in the autosampler/refrigerator. To be deemed reproducible, the mean %Bias at each level must be within ± 15.0% with %CV ≤ 15.0%.

Reinjection of individual samples can be made with adequate precision and accuracy. Data are provided in Table 30.

## 11.19. Whole Run Reinjection Reproducibility

To evaluate whole run reinjection reproducibility, run 1 was reinjected as run 6 on the same MS platform using a different chromatographic column from the same manufacturer, with the same specification as the original, with the same specification within 4 days 4 hours following the original injection. To be deemed acceptable, the reinjected run was required to meet the acceptance criteria outlined in Sections 11.4, 11.5, and 11.6.

It has been demonstrated that entire runs can be reinjected with adequate accuracy and precision. Data are presented in Table 31.

## 11.20. Column Ruggedness

Column ruggedness was assessed by reinjecting run 1 as run 6 on the same MS platform (Applied Biosystems API6500) using a different chromatographic column from the same manufacturer, with the same specification as the original.

Data were required to meet the precision and accuracy criteria outlined in Sections 11.4, 11.5, and 11.6 to be considered as reproducible/rugged method. Data are presented in Table 31. It has been demonstrated that ruggedness has been met.

## 11.21. Extraction Recovery

To evaluate the extraction recovery, the low, mid, and high extracted QC samples were compared to extracted human plasma blanks to which an equal amount of ertapenem at each level, assuming 100% recovery, was added in six replicates.

To evaluate the extraction recovery of ertapenem-$d_4$ (IS), ertapenem was added only for tracking during analysis. Ertapenem-$d_4$ (IS) for the control was added after extraction, while the same amount of ertapenem-$d_4$ (IS) for the extracted QC was added before extraction.

$$\text{\% Recovery} = \frac{\text{Response ratio of "Extracted QC"}}{\text{Average Response Ratio of Control}} \times 100$$

WIS was inadvertently not added to the neat solutions for low, mid, and high QCs in run 2. Extraction recovery for the QCs was repeated in run 7. The mean recovery of ertapenem was determined to be consistent, precise and reproducible at 106.2%.

The recovery of ertapenem-$d_4$ (IS) was determined to be consistent, precise and reproducible at 79.2%. Data are presented in Table 32 to Table 33.

## 11.22. Solution Stabilities

The stability of various solutions of ertapenem in various solvents used during the sample analysis following storage at various temperatures was determined. Ertapenem-$d_4$ is a deuterated analog, so its stability at room temperature is taken as equivalent to ertapenem.

Data showing the ertapenem(s), solvent composition, storage term, and temperature are presented in Table 34 and Table 35.

## 11.23. Calculations

The peak areas of ertapenem and ertapenem-$d_4$ were integrated by Analyst 1.7.2 (Applied Biosystems; Foster City, CA, USA). The data results were imported electronically into Watson LIMS 7.5 for the calculation of ertapenem concentrations and statistical analysis. The calibration curves were obtained by a weighted (1/concentration$^2$) least squares linear regression. After the best fit from data, the concentrations (mcg/mL) were calculated based on the measured peak area ratios of ertapenem/ertapenem-$d_4$ using the following equation:

Response = Slope * Conc + Intercept

$$\text{Mean Value } (\overline{X}) = \frac{\sum_{i=1}^{n} \text{individual data (X)}}{\text{number of data points (n)}}$$

$$\text{Standard Deviation (SD)} = \sqrt{\frac{\sum_{i=1}^{n}\left[\text{individual data (X)} - \text{Mean Value } (\overline{X})\right]^2}{n-1}}$$

$$\text{Coefficient of Variation (\%CV)} = \left[\frac{\text{Standard Deviation (SD)}}{\text{Mean Value } (\overline{X})}\right] \times 100$$

$$\%\text{Bias} = \left(\frac{\text{Calculated Mean of the Observed Values or Observed Value} - \text{Nominal Value}}{\text{Nominal Value}}\right) \times 100$$

All calibration standards and QCs concentrations were reported to three significant figures, and study samples will be reported to three significant figures.

## 11.24. SOP Deviations and Investigations

Two test procedure deviations occurred, and the description and impact are presented in Table 2. No investigations were conducted during this study.

**Table 2: Deviations**

| Deviation # | Description | Impact |
|---|---|---|
| DEV-2023-0011 | TP states to weigh Ertapenem primary into glass and store in polypropylene. All Ertapenem primaries deviated from the TP by weighing into polypropylene. | The severity of the deviation is low due to the fact that all Ertapenem primaries were weighed into polypropylene throughout the validation. All stability was performed using the same type of container that was used for primary preparation. |
| DEV-2023-0014 | TP says to weigh primary into glass but store in polypropylene. Analyst inadvertently also stored primaries in glass instead of polypropylene. Lot numbers affected: V1872106P5-51-1 and V1872106P5-52-1 both weighed out on 15Dec2022. Primaries match each other and response is similar to correctly stored primaries. There is no impact to study. TP has been updated to specify the same container type for weighing and storage to avoid confusion in the future. | TP updated to avoid future confusion by weighing into the same container type as final storage container type. Can be easily detected since storage vessel is documented. |

## 12. CONCLUSION

Validation results obtained in this study indicate that a robust and reliable method has been established for the measurement of total ertapenem in treated human plasma, with sodium heparin as anticoagulant, by LC-MS/MS in the concentration range of 0.200 - 200 mcg/mL. The generally recommended acceptance criteria, FDA Guidance for Industry on Bioanalytical Methods Validation, May 2018, in terms of precision, accuracy, selectivity including matrix effect, recovery and all types of stability have been met. Thus, this validated method is deemed suitable for determination of ertapenem in human plasma, with sodium heparin as anticoagulant, for unknown samples.

## 13. PERTINENT SOPS

The following SOPs were followed during the conduct of this study:

SOP-00073 [02-04] - Bioanalytical Method Validation - Chromatographic Assays

SOP-00035 [00-02] - Procedures, Reporting, and Acceptance Criteria for Bioanalytical Chromatographic Projects

## 14. ARCHIVES

All raw data, including paper and electronic files associated with this study will remain archived at a location specified by KCAS, 10830 S Clay Blair Boulevard, Olathe, Kansas 66061 for a period of at least 10 years, after which the client may be contacted to arrange for continued storage of the files, or disposition according to an agreed procedure. KCAS will maintain an electronic (scanned/true) copy of the method validation report that can be viewed and printed on site. The study plan, documentation, raw data, and final report are indexed under Bioanalytical Project Number V1872106P5.

## 15. REFERENCES

1. Guidance for Industry: Bioanalytical Method Validation. U.S. Department of Health and Human Services. Food and Drug Administration. Center for Drug Evaluation and Research. Center for Veterinary Medicine. May 2018.
2. Comed: QCs in the presence of 37.5 mcg/mL zidebactam.

# 16. TABLES

### Table 3: Mass Spec Transitions

| Analyte | Transition |
|---|---|
| Ertapenem | m/z 476.047→431.9 |
| Ertapenem-$d_4$ (IS) | m/z 480.2→436.3 |

### Table 4: Reference Standards

| Analyte | Supplier | Lot/Batch Number | Purity | Expiration Date or Retest Date` | Conversion Factor |
|---|---|---|---|---|---|
| Ertapenem Sodium Salt | Med Chem Express | 180017 | 98.9% | Retest 11 Aug 2024 | 1.046 |
| | Med Chem Express | 66084 | 99.1% *** | Expires 21 Feb 2025 | 1.046 |
| Ertapenem-$d_4$ Sodium Salt (IS) | Alsa Chim | DH-ALS-20-002-P2 | * | Expire 29 Apr 2023 | * |
| | Alsa Chim | DH-ALS-20-002-P2 | ** | Retest 26 Oct 2023 | ** |
| Zidebactam | Wockhardt | WK1/WP-0060/016 | 90.4% | Expire 29 Mar 2023 | 1.000 |

Certificates of analysis for the above reference standards are presented in Annex 2.
*Used as is with 1.000 conversion and 100% purity as IS only.
**Use as is, no correction for N/A or purity of 83.3%
***Purity updated to 99.09% per updated COA

## Table 5: Calibration Standards and QC Samples Preparation

| Critical Solutions | Lot # for Standards (Prep Date) | Lot # for QCs (Prep Date) | Standards Lot (Prep Date) | QC Lot (Prep Date) | Runs used |
|---|---|---|---|---|---|
| Treated Human Plasma (1:1 MES Buffer:Plasma) | V1872106P5-7-3 | | V1872106P5-8-1 Set A (03Nov2022) | V1872106P5-9-1 Set A (03Nov2022) | 1, 5-7, QCs only 2, 12 (LTS), 16 (LTS) |
| Chilled ~0.1M MES Buffer stored at -20°C in polypropylene | V1872106P5-1-1 (03Nov2022) | V1872106P5-1-1 (03Nov2022) | | | |
| Treated Human Plasma (1:1 MES Buffer:Plasma) stored at -70°C in polypropylene | V1872106P5-7-2 (03Nov2022) | V1872106P5-7-1 (03Nov2022) | | | |
| Treated Human Plasma (1:1 MES Buffer:Plasma) | V1872106P5-15-3 | | V1872106P5-17-1 Set B (04Nov2022) | NA | 2 |
| Chilled MES ~0.1M Buffer stored at -20°C in polypropylene | V1872106P5-1-1 (03Nov2022) | NA | | | |
| Treated Human Plasma (1:1 MES Buffer:Plasma) stored at -70°C in polypropylene | V1872106P5-17-2 (04Nov2022) | NA | | | |
| Treated Human Plasma (1:1 MES Buffer:Plasma) | V1872106P5-35-1 | | V1872106P5-36-1 Set C (09Nov2022) | V1872106P5-37-1 Set C (09Nov2022) | 9 |
| Chilled MES ~0.1M Buffer stored at -20°C in polypropylene | V1872106P5-2-1 (03Nov2022) | V1872106P5-2-1 (03Nov2022) | | | |
| Treated Human Plasma (1:1 MES Buffer:Plasma) stored at -70°C in polypropylene | V1872106P5-35-2 (09Nov2022) | V1872106P5-35-2 (09Nov2022) | | | |
| Treated Human Plasma (1:1 MES Buffer:Plasma) | V1872106P5-7-3 | | NA | V1872106P5-10-1 Set A Comed QC (03Nov2022) | 1, 12 (LTS), 16 (LTS) |
| Chilled MES ~0.1M Buffer stored at -20°C in polypropylene | NA | V1872106P5-1-1 (03Nov2022) | | | |
| Treated Human Plasma (1:1 MES Buffer:Plasma) stored at -70°C in polypropylene | NA | V1872106P5-7-1 (03Nov2022) | | | |
| Zidebactam in Chilled MES ~0.1M Buffer stored at -20°C in polypropylene | | V1872106P5-5-1 (03Nov2022) | | | |
| Treated Human Plasma (1:1 MES Buffer:Plasma) | V1872106P5-48-3 | | V1872106P5-49-1 Set D (05Dec2022) | V1872106P5-50-1 Set D (05Dec2022) | 12 |
| Chilled ~0.1M MES Buffer stored at -20°C in polypropylene | V1872106P5-43-1 (02Dec2022) | V1872106P5-44-1 (02Dec2022) | | | |
| Treated Human Plasma (1:1 MES Buffer:Plasma) stored at -70°C in polypropylene | V1872106P5-48-1 (05Dec2022) | V1872106P5-48-2 (05Dec2022) | | | |
| Treated Human Plasma (1:1 MES Buffer:Plasma) | BioIVT HMN769591 | | NA | V1872106P2-92-1 Untreated Comed QCs (16Dec2022) | 17 (LTS) |
| Chilled ~0.1M MES Buffer stored at -20°C in polypropylene | NA | V1872106P5-51-1 (15Dec2022) | | | |
| Zidebactam in deionized water stored at -20 °C in polypropylene | NA | V1872106P2-79-1 (06Dec2022) | | | |
| Treated Human Plasma (1:1 MES Buffer:Plasma) | V1872106P5-48-3 | | V1872106P5-61-1 Set F (20Dec2022) | V1872106P5-62-1 Set F (20Dec2022) | 15 |
| Chilled ~0.1M MES Buffer stored at -20°C in polypropylene | V1872106P5-51-1 (15Dec2022) | V1872106P5-52-1 (15Dec2022) | | | |
| Treated Human Plasma (1:1 MES Buffer:Plasma) stored at -70°C in polypropylene | V1872106P5-63-1 (20Dec2022) | V1872106P5-63-2 (20Dec2022) | | | |

## Table 5: Calibration Standards and QC Samples Preparation (Continued)

| Critical Solutions | Lot # for Standards (Prep Date) | Lot # for QCs (Prep Date) | Standards Lot (Prep Date) | QC Lot (Prep Date) | Runs used |
|---|---|---|---|---|---|
| Treated Human Plasma (1:1 MES Buffer:Plasma) | V1872106P5-64-1 | | V1872106P5-65-1 Set G (27Dec2022) | V1872106P5-66-1 Set G (27Dec2022) | 16 |
| Chilled ~0.1M MES Buffer stored at -20°C in polypropylene | V1872106U3-42-1 (20Dec2022) | V1872106U3-42-1 (20Dec2022) | | | |
| Treated Human Plasma (1:1 MES Buffer:Plasma) stored at -70°C in polypropylene | V1872106P5-64-2 (27Dec2022) | V1872106P5-64-2 (27Dec2022) | | | |
| Treated Human Plasma (1:1 MES Buffer:Plasma) | V1872106P5-77-2 | | V1872106P5-76-1 Set H (04Jan2023) | V1872106P5-77-1 Set H (04Jan2023) | 17 |
| Chilled ~0.1M MES Buffer stored at -20°C in polypropylene | V1872106P5-68-1 (03Jan2023) | V1872106P5-69-1 (03Jan2023) | | | |
| Treated Human Plasma (1:1 MES Buffer:Plasma) stored at -70°C in polypropylene | V1872106P5-75-1 (04Jan2023) | V1872106P5-75-2 (04Jan2023) | | | |
| Treated Human Plasma (1:1 MES Buffer:Plasma) | V1872106P5-77-2 | | NA | V1872106P5-78-1 Comed QCs (16Dec2022) | 17 (LTS) |
| Chilled ~0.1M MES Buffer stored at -20°C in polypropylene | NA | V1872106P5-68-1 (03Jan2023) | | | |
| Zidebactam in deionized water stored at -20 °C in polypropylene | NA | V1872106P2-97-1 (03Jan2023) | | | |
| Treated Human Plasma (1:1 MES Buffer:Plasma) | V1872106P5-83-1 | | V1872106P5-80-1 Set I (09Jan2023) | V1872106P5-81-1 Set I (09Jan2023) | 18 |
| Chilled ~0.1M MES Buffer stored at -20°C in polypropylene | V1872106P5-68-1 (03Jan2023) | V1872106P5-69-1 (03Jan2023) | | | |
| Treated Human Plasma (1:1 MES Buffer:Plasma) stored at -70°C in polypropylene | V1872106P5-79-1 (09Jan2023) | V1872106P5-79-2 (09Jan2023) | | | |
| Treated Human Plasma (1:1 MES Buffer:Plasma) | BioIVT HMN444009 | | NA | V1872106P5-82-1 Untreated Comed QCs (09Jan2023) | 19 (LTS), 20 (LTS), 21 (LTS), 22 (LTS), 23 (LTS) |
| Chilled ~0.1M MES Buffer stored at -20°C in polypropylene | NA | V1872106P5-68-1 (03Jan2023) | | | |
| Zidebactam in deionized water stored at -20 °C in polypropylene | NA | V1872106P2-97-1 (03Jan2023) | | | |
| Treated Human Plasma (1:1 MES Buffer:Plasma) | V1872106P2-108-2 | | NA | V1872106P2-109-1 Treated Comed QCs (06Jan2023) | 18 (LTS), 23 (LTS), 24 (LTS) |
| Chilled ~0.1M MES Buffer stored at -20°C in polypropylene | NA | V1872106P5-69-1 (03Jan2023) | | | |
| Zidebactam in deionized water stored at -20 °C in polypropylene | NA | V1872106P2-98-1 (03Jan2023) | | | |
| Treated Human Plasma (1:1 MES Buffer:Plasma) | V1872106P5-93-2 | | V1872106P5-92-1 Set J (13Jan2023) | V1872106P5-93-1 Set J (13Jan2023) | 19 |
| Chilled ~0.1M MES Buffer stored at -20°C in polypropylene | V1872106P5-85-1 (12Jan2023) | V1872106P5-84-1 (12Jan2023) | | | |
| Treated Human Plasma (1:1 MES Buffer:Plasma) stored at -70°C in polypropylene | V1872106P5-91-2 (13Jan2023) | V1872106P5-91-1 (13Jan2023) | | | |
| Treated Human Plasma (1:1 MES Buffer:Plasma) | V1872106P5-93-2 | | V1872106P5-95-1 Set K (16Jan2023) | V1872106P5-96-1 Set K (16Jan2023) | 20 |
| Chilled ~0.1M MES Buffer stored at -20°C in polypropylene | V1872106P5-84-1 | V1872106P5-85-1 | | | |
| Treated Human Plasma (1:1 MES Buffer:Plasma) stored at -70°C in polypropylene | V1872106P5-94-1 (16Jan2023) | V1872106P5-94-2 (16Jan2023) | | | |

## Table 5: Calibration Standards and QC Samples Preparation (Continued)

| | | | | | |
|---|---|---|---|---|---|
| Treated Human Plasma (1:1 MES Buffer:Plasma) | V1872106P2-117-1 | | V1872106P5-105-1 Set L (23Jan2023) | V1872106P5-106-1 Set L (23Jan2023) | 21 |
| Chilled ~0.1M MES Buffer stored at -20°C in polypropylene | V1872106P5-97-1 (19Jan2023) | V1872106P5-98-1 (19Jan2023) | | | |
| Treated Human Plasma (1:1 MES Buffer:Plasma) stored at -70°C in polypropylene | V1872106P5-104-1 (23Jan2023) | V1872106P5-104-2 (23Jan2023) | | | |
| Treated Human Plasma (1:1 MES Buffer:Plasma) | BioIVT HMN444012 | | NA | V1872106P5-107-1 Untreated Ertapenem QCs (23Jan2023) | 22 (LTS), 23 (LTS), 25 (LTS), 26 (LTS) |
| Chilled ~0.1M MES Buffer stored at -20°C in polypropylene | NA | V1872106P5-97-1 (19Jan2023) | | | |
| Treated Human Plasma (1:1 MES Buffer:Plasma) stored at -70°C in polypropylene | NA | NA | | | |
| Treated Human Plasma (1:1 MES Buffer:Plasma) | V1872106P5-116-1 | | V1872106P5-118-1 Set M (02Feb2023) | V1872106P5-119-1 Set M (02Feb2023) | 22 |
| Chilled ~0.1M MES Buffer stored at -20°C in polypropylene | V1872106P5-108-1 (01Feb2023) | V1872106P5-109-1 (01Feb2023) | | | |
| Treated Human Plasma (1:1 MES Buffer:Plasma) stored at -70°C in polypropylene | V1872106P5-117-1 (02Feb2023) | V1872106P5-117-2 (02Feb2023) | | | |
| Treated Human Plasma (1:1 MES Buffer:Plasma) | V1872106P5-120-1 | | V1872106P5-121-1 Set N (07Feb2023) | V1872106P5-122-1 Set N (07Feb2023) | 23 |
| Chilled ~0.1M MES Buffer stored at -20°C in polypropylene | V1872106P5-108-1 (01Feb2023) | V1872106P5-108-1 (01Feb2023) | | | |
| Treated Human Plasma (1:1 MES Buffer:Plasma) stored at -70°C in polypropylene | V1872106P5-120-2 (07Feb2023) | V1872106P5-120-2 (07Feb2023) | | | |
| Treated Human Plasma (1:1 MES Buffer:Plasma) | V1872106P5-134-2 | | V1872106P5-135-1 Set O (13Feb2023) | V1872106P5-136-1 Set O (13Feb2023) | 24 |
| Chilled ~0.1M MES Buffer stored at -20°C in polypropylene | V1872106P5-125-1 (10Feb2023) | V1872106P5-125-1 (10Feb2023) | | | |
| Treated Human Plasma (1:1 MES Buffer:Plasma) stored at -70°C in polypropylene | V1872106P5-134-1 (13Feb2023) | V1872106P5-134-1 (13Feb2023) | | | |
| Treated Human Plasma (1:1 MES Buffer:Plasma) | V1872106P5-131-1 | | NA | V1872106P5-131-2 Treated Comed QCs (10Feb2023) | 24 (LTS) |
| Chilled ~0.1M MES Buffer stored at -20°C in polypropylene | NA | V1872106P-124-1 (10Feb2023) | | | |
| Treated Human Plasma (1:1 MES Buffer:Plasma) stored at -70°C in polypropylene | NA | V1872106P2-121-1 (02Feb2023) | | | |
| Treated Human Plasma (1:1 MES Buffer:Plasma) | V1872106P5-137-1 | | V1872106P5-139-1 Set P (15Feb2023) | V1872106P5-140-1 Set P (15Feb2023) | 25 |
| Chilled ~0.1M MES Buffer stored at -20°C in polypropylene | V1872106P5-124-1 (10Feb2023) | V1872106P5-125-1 (10Feb2023) | | | |
| Treated Human Plasma (1:1 MES Buffer:Plasma) stored at -70°C in polypropylene | V1872106P5-138-1 (15Feb2023) | V1872106P5-138-2 (15Feb2023) | | | |
| Treated Human Plasma (1:1 MES Buffer:Plasma) | V1872106P5-134-2 | | V1872106P5-149-1 Set Q (20Feb2023) | V1872106P5-150-1 Set Q (20Feb2023) | 26 |
| Chilled ~0.1M MES Buffer stored at -20°C in polypropylene | V1872106P5-141-1 (17Feb2023) | V1872106P5-141-1 (17Feb2023) | | | |
| Treated Human Plasma (1:1 MES Buffer:Plasma) stored at -70°C in polypropylene | V1872106P5-148-1 (20Feb2023) | V1872106P5-148-1 (20Feb2023) | | | |

## Table 6: Matrix Description

| Matrix | Source Lot # | Date Received |
|---|---|---|
| Treated Human Plasma (1:1 MES Buffer:Plasma)<br>V1872106P5-7-3<br>V1872106P5-15-3<br>V1872106P5-26-1<br>V1872106P5-27-1<br>V1872106P5-35-1<br>V1872106P5-48-3 | Biochemed<br>Combo-73-1<br>BC42768HP-1, BC42768HP-2, BC42768HP-3, BC42768HP-4, BC42768HP-5, BC42768HP-6 | Oct2022 |
| Treated Human Plasma (1:1 MES Buffer:Plasma)<br>V1872106P5-64-1 | BioIVT HMN444010 | Aug2020 |
| Treated Human Plasma (1:1 MES Buffer:Plasma)<br>V1872106P5-77-2<br>V1872106P2-108-2 | BioIVT HMN444008 | Aug2020 |
| Treated Human Plasma (1:1 MES Buffer:Plasma)<br>V1872106P5-83-1<br>V1872106P5-93-2 | BioIVT HMN444009 | Aug2020 |
| Treated Human Plasma (1:1 MES Buffer:Plasma)<br>V1872106P5-116-1 | BioIVT HMN444011 | Aug2020 |
| Treated Human Plasma (1:1 MES Buffer:Plasma)<br>V1872106P5-120-1<br>V1872106P5-131-1 | BioIVT HMN795699 | Apr2022 |
| Treated Human Plasma (1:1 MES Buffer:Plasma)<br>V1872106P5-134-2 | Biochemed BC43938HP-9 | Feb2023 |
| Treated Human Plasma (1:1 MES Buffer:Plasma)<br>V1872106P5-137-1 | BioIVT HMN795696 | Apr2022 |
| Treated Human Plasma (1:1 MES Buffer:Plasma)<br>V1872106P2-117-1 | BioIVT HMN444012 | Aug2020 |
| Human Plasma | BioIVT HMN444009 | Aug2020 |
| Human Plasma | BioIVT HMN444012 | Aug2020 |
| Human Plasma | BioIVT HMN769591 | Mar2022 |

## Table 7: Summary of Results and Conclusions

| Methodology – TP-80193 | Method Performance – LC-MS/MS | Hyperlink – N/A | Comments |
|---|---|---|---|
| Matrix | Plasma | | |
| Anticoagulant | Sodium heparin | | |
| Assay Volume | 100 mcL | | |
| **Selectivity** | | | |
| Analyte | Ertapenem | Summary tables<br>Table 10<br><br>Report text<br>Section 11.3.1 | |
| Internal Standard | Ertapenem-$d_4$ | Summary tables<br>Table 10<br><br>Report text<br>Section 11.3.1 | |
| Matrix Effect<br>(6 spiked matrix lots) | Complies | Summary tables<br>Table 11<br><br>Report text<br>Section 11.3.2 | |
| Interference | Complies | Summary tables<br>Table 12<br><br>Report text<br>Section 11.3.3 | |
| Hemolytic Effect Evaluation | Complies for 0.5% hemolysis<br>Does not comply for 1% and 2% hemolysis | Summary tables<br>Table 13<br><br>Report text<br>Section 11.3.4 | |
| Lipemic Effect Evaluation | Complies | Summary tables<br>Table 14<br><br>Report text<br>Section 11.3.4 | |
| Carryover | Assessed with every run with standards and QCs | Report text<br>Section 11.3.5 | |
| **Linearity** | | | |
| Weighting | $1/x^2$ | Report text<br>Section 11.4 | |
| Bias at LLOQ | 0.0% | Summary tables<br>Table 15<br><br>Report text<br>Section 11.4 | |

## Table 7: Summary of Results and Conclusions (Continued)

| Methodology – TP-80193 | Method Performance – LC-MS/MS | Hyperlink – N/A | Comments |
|---|---|---|---|
| Bias above LLOQ | -3.3 to 4.4% | Summary tables<br>Table 15<br><br>Report text<br>Section 11.4 | |
| Bioanalytical Range | Complies<br>0.200 - 200 mcg/mL | Summary tables<br>Table 15<br><br>Report text<br>Section 11.4 | |
| **Precision** | | | |
| Intra-assay | 5.0 to 18.0% (LLOQ);<br>3.4 to 13.6% (above LLOQ) | Summary tables<br>Table 16<br><br>Report text<br>Section 11.5 | |
| Inter-assay | 12.5% (LLOQ);<br>7.4 to 10.0% (above LLOQ) | Summary tables<br>Table 17<br><br>Report text<br>Section 11.5 | |
| **Accuracy** | | | |
| Intra-assay | -9.5 to 10.0% (LLOQ);<br>-7.3 to 11.3% (above LLOQ) | Summary tables<br>Table 16<br><br>Report text<br>Section 11.5 | |
| Inter-assay | 4.0% (LLOQ);<br>-1.3 to 1.8% (above LLOQ) | Summary tables<br>Table 17<br><br>Report text<br>Section 11.5 | |
| **Large Run Size** | Determined to be 120 samples. | Summary tables<br>Table 15 and Table 16<br>Report text<br>Section 11.7 | |
| **Dilution** | | | |
| Dilution factor: 2x | Dilution QC: 150 mcg/mL<br>Precision: 2.8%<br>Accuracy: 5.3% | Summary tables<br>Table 20<br><br>Report text<br>Section 11.8 | |
| Dilution factor: 5x | Dilution QC: 800 mcg/mL<br>Precision: 4.9%<br>Accuracy: -5.3% | Summary tables<br>Table 20<br><br>Report text<br>Section 11.8 | |

## Table 7: Summary of Results and Conclusions (Continued)

| **Methodology – TP-80193** | **Method Performance – LC-MS/MS** | **Hyperlink – N/A** | **Comments** |
|---|---|---|---|
| **Stability** | | | |
| *Matrix (in polypropylene tubes)* | | | |
| Freeze/Thaw (-20 °C and -70 °C /wet ice) treated with and without the presence of comed[2] | Demonstrated for 4 cycles in treated QCs | Summary tables Table 21<br><br>Report text Section 11.9 | |
| Freeze/Thaw (-70 °C/wet ice) untreated with and without the presence of comed[2] | Demonstrated for 2 cycles in untreated QCs | Summary tables Table 22<br><br>Report text Section 11.10 | |
| Bench-top (wet ice) treated with and without the presence of comed[2] | Demonstrated for 8.75 hours in treated QCs | Summary tables Table 23<br><br>Report text Section 11.11 | |
| Bench-top (wet ice) untreated with and without the presence of comed[2] | Demonstrated for 4 hours in untreated QCs | Summary tables Table 23<br><br>Report text Section 11.12 | |
| Long-term (-20 °C) treated with and without the presence of comed[2] | Unstable at all durations tested | Summary tables Table 25<br><br>Report text Section 11.13 | |
| Long-term (-70 °C) treated with and without the presence of comed[2] | Demonstrated for 38 days in treated QCs with comed[2]<br>Demonstrated for 24 days in treated QCs without comed[2] | Summary tables Table 25<br><br>Report text Section 11.13 | |
| Long-term (-70 °C) untreated with and without the presence of comed[2] | Demonstrated for 29 days in untreated QCs with comed[2]<br>Demonstrated for 28 days in untreated QCs without comed[2] | Summary tables Table 26<br><br>Report text Section 11.14 | |
| *Whole Blood* | No instability observed in ice-water bath or at room temperature in polyethylene terephthalate for up to 1 hour. | Summary tables<br><br>Table 27Table 27<br><br>Report text Section 11.15 | |
| *Processed Matrix polypropylene* | | | |
| Room Temperature | Demonstrated for 3 days 20 hours | Summary tables Table 28<br><br>Report text Section 11.16 | |

## Table 7: Summary of Results and Conclusions (Continued)

| **Methodology – TP-80193** | **Method Performance – LC-MS/MS** | **Hyperlink – N/A** | **Comments** |
|---|---|---|---|
| Refrigeration | Demonstrated for 3 days 20 hours | Summary tables<br>Table 29<br><br>Report text<br>Section 11.17 | |
| **Individual Sample Reinjection** | Reproducible | Summary tables<br>Table 30<br><br>Report text<br>Section 11.18 | |
| **Whole Run Reinjection Integrity** | Reproducible<br>Duration 4 days 4 hours | Summary tables<br>Table 31<br><br>Report text<br>Section 11.19 | |
| **Column Ruggedness** | Reproducible | Summary tables<br>Table 31<br><br>Report text<br>Section 11.20 | |
| **Extraction Recovery** | | | |
| Ertapenem | Overall 106.2% | Summary tables<br>Table 32<br><br>Report text<br>Section 11.21 | |
| Ertapenem-$d_4$ (IS) | Overall 79.2% | Summary tables<br>Table 33<br><br>Report text<br>Section 11.21 | |
| **Solution Stability** | | | |
| Solution, Primary polypropylene | | | |
| Ertapenem in MES buffer (RT) | Stable 6.75 hours | Summary tables<br>Table 34 | |
| Ertapenem in MES buffer (-20 °C) | Stable 7 days<br>Unstable 29 days | Summary tables<br>Table 35 | |
| Ertapenem-$d_4$ (IS) in MES buffer (RT) | Taken to be same as ertapenem | SOP 00073 | |
| Ertapenem-$d_4$ (IS) in MES buffer (-20 °C) | 1 year stability can be assigned and evaluated thereafter in yearly increments | SOP 00073 | |

## Table 8: Summary of Validation Run Objectives and Outcome

| Run ID | Instrument ID | Extraction Date | Assay Date | Run Objective(s) | Accepted or Rejected | Comment |
|---|---|---|---|---|---|---|
| 1 | LC/MS-48 | 03-Nov-2022 | 03-Nov-2022 | Precision and accuracy, IS cross-talk, comed[2] interference, dilution integrity, processed sample stability extraction, and individual sample reinjection reproducibility | Accepted | |
| 2 | LC/MS-48 | 04-Nov-2022 | 04-Nov-2022 | Precision and accuracy, hemolytic and lipemic blank screen, hemolytic and lipemic QCs, selectivity, extraction recovery, and large run size | Accepted | [a] |
| 3 | LC/MS-48 | 04-Nov-2022 | 04-Nov-2022 | Matrix effect | Accepted | |
| 4 | LC/MS-48 | 04-Nov-2022 | 04-Nov-2022 | Bench-top solution stability | Accepted | |
| 5 | LC/MS-48 | 07-Nov-2022 | 07-Nov-2022 | Processed sample stability | Accepted | |
| 6 | LC/MS-48 | 03-Nov-2022 | 07-Nov-2022 | Whole run reinjection reproducibility and column ruggedness | Accepted | |
| 7 | LC/MS-48 | 08-Nov-2022 | 08-Nov-2022 | Hemolytic QCs and extraction recovery (ertapenem only) | Accepted | [b] |
| 8 | LC/MS-48 | 09-Nov-2022 | 09-Nov-2022 | Whole blood stability | Accepted | |
| 9 | LC/MS-48 | 09-Nov-2022 | 09-Nov-2022 | Precision and accuracy, freeze/thaw stability with and without comed[2], bench-top stability with and without comed[2], and large run size | Accepted | |
| 10 | LC/MS-48 | 10-Nov-2022 | 10-Nov-2022 | Primary long term solution stability | Accepted | |
| 11 | LC/MS-42 | 02-Dec-2022 | 05-Dec-2022 | Primary long term solution stability | Rejected | [c] |
| 12 | LC/MS-42 | 05-Dec-2022 | 05-Dec-2022 | Precision and accuracy, Long-term matrix stability with and without comed[2] | Rejected | [d] |
| 13 | LC/MS-42 | 05-Dec-2022 | 07-Dec-2022 | Precision and accuracy, Long-term matrix stability with and without comed[2] | Accepted | [d] |
| 15 | LC/MS-39 | 20-Dec-2022 | 20-Dec-2022 | Untreated freeze/thaw stability with comed[2] and untreated bench-top stability with comed[2] | Accepted | |
| 16 | LC/MS-42 | 27-Dec-2022 | 27-Dec-2022 | Long-term matrix stability with and without comed[2] | Accepted | [e] |
| 17 | LC/MS-22 | 04-Jan-2023 | 04-Jan-2023 | Untreated long-term matrix stability with comed[2] and treated comed[2] QC qualification | Accepted | [f] |
| 18 | LC/MS-39 | 09-Jan-2023 | 09-Jan-2023 | Untreated long-term matrix stability and treated and untreated comed[2] QC qualification | Accepted | |
| 19 | LC/MS-39 | 13-Jan-2023 | 13-Jan-2023 | Untreated long-term matrix stability with comed[2] | Accepted | |
| 20 | LC/MS-39 | 16-Jan-2023 | 16-Jan-2023 | Untreated long-term matrix stability with comed[2] | Accepted | |
| 21 | LC/MS-39 | 23-Jan-2023 | 24-Jan-2023 | Untreated long-term matrix stability with comed[2] and untreated QC qualification | Accepted | |
| 22 | LC/MS-22 | 02-Feb-2023 | 02-Feb-2023 | Untreated long-term matrix stability with and without comed[2] | Accepted | |

## Table 8: Summary of Validation Run Objectives and Outcome (Continued)

| Run ID | Instrument ID | Extraction Date | Assay Date | Run Objective(s) | Accepted or Rejected | Comment |
|---|---|---|---|---|---|---|
| 23 | LC/MS-39 | 07-Feb-2023 | 07-Feb-2023 | Untreated bench-top stability, untreated freeze/thaw stability, untreated long-term matrix stability with and without comed[2], and treated long-term matrix stability with comed[2] | Accepted | g |
| 24 | LC/MS-25 | 13-Feb-2023 | 13-Feb-2023 | Treated long-term matrix stability with comed[2] and treated comed[2] QC qualification | Accepted | h |
| 25 | LC/MS-25 | 15-Feb-2023 | 15-Feb-2023 | Untreated long-term matrix stability | Accepted | |
| 26 | LC/MS-39 | 20-Feb-2023 | 20-Feb-2023 | Untreated long-term matrix stability | Accepted | |

Run 14 was used to qualify untreated comed[2] QCs and will not be reported.
[a]WIS was inadvertently not added to the neat solutions for low, mid, and high QCs. Extraction recovery for the QCs were repeated in run 7. Low Hemolytic QCs failed acceptance criteria and was repeated in Run 7.
[b]Hemolytic QC evaluation at 1% and 2% hemolysis did not meet acceptance criteria.
[c]Ertapenem primary lost 15.7% over 29 days.
[d]Data taken from run 13. Run 12 was reinjected as run 13 due to large %CV in QCs throughout the run. Stability QCs failed due to a combination of high %CV, low %Bias, and high %Bias.
[e]All stability QCs failed due to low %Bias.
[f]Untreated -70 °C LTS QCs failed %Bias criteria and treated comed QC failed due to a preparation error.
[g]Treated comed -70 °C LTS failed %Bias high and this does not point to instability. Per sponsor timepoint will be repeated for confirmation to verify results or see if there was an extraction issue.
[h]Treated comed -20 °C LTS failed low for %Bias. This suggests the treated comed[2] at -20 °C is unstable at 38 days.

## Table 9: Carryover

| Run ID | Result (Extracted Blank after High Standard) | Carryover Results for Internal Standard |
|---|---|---|
| 1 | Seq 13 and Seq 79 are <20.0% of the LLOQ. | Seq 4 and Seq 70 are < 5.0% internal standard peak of acceptable standards. |
| 2 | Seq 29 and Seq 120 are <20.0% of the LLOQ. | Seq 4 and Seq 111 are < 5.0% internal standard peak of acceptable standards. |
| 5 | Seq 13 and Seq 61 are <20.0% of the LLOQ. | Seq 4 and Seq 52 are < 5.0% internal standard peak of acceptable standards. |
| 6 | Seq 13 and Seq 53 are <20.0% of the LLOQ. | Seq 4 and Seq 44 are < 5.0% internal standard peak of acceptable standards. |
| 7 | Seq 13 and Seq 101 are <20.0% of the LLOQ. | Seq 4 and Seq 92 are < 5.0% internal standard peak of acceptable standards. |
| 9 | Seq 13 and Seq 125 are <20.0% of the LLOQ. | Seq 4 and Seq 116 are < 5.0% internal standard peak of acceptable standards. |
| 13 | Seq 13 and Seq 107 are <20.0% of the LLOQ. | Seq 4 and Seq 98 are < 5.0% internal standard peak of acceptable standards. |
| 15 | Seq 13 and Seq 55 are <20.0% of the LLOQ. | Seq 4 and Seq 46 are < 5.0% internal standard peak of acceptable standards. |
| 16 | Seq 13 and Seq 89 are <20.0% of the LLOQ. | Seq 4 and Seq 80 are < 5.0% internal standard peak of acceptable standards. |
| 17 | Seq 13 and Seq 56 are <20.0% of the LLOQ. | Seq 4 and Seq 47 are < 5.0% internal standard peak of acceptable standards. |
| 18 | Seq 13 and Seq 68 are <20.0% of the LLOQ. | Seq 4 and Seq 59 are < 5.0% internal standard peak of acceptable standards. |
| 19 | Seq 13 and Seq 44 are <20.0% of the LLOQ. | Seq 4 and Seq 35 are < 5.0% internal standard peak of acceptable standards. |
| 20 | Seq 13 and Seq 44 are <20.0% of the LLOQ. | Seq 4 and Seq 35 are < 5.0% internal standard peak of acceptable standards. |
| 21 | Seq 13 and Seq 56 are <20.0% of the LLOQ. | Seq 4 and Seq 47 are < 5.0% internal standard peak of acceptable standards. |
| 22 | Seq 13 and Seq 56 are <20.0% of the LLOQ. | Seq 4 and Seq 47 are < 5.0% internal standard peak of acceptable standards. |
| 23 | Seq 13 and Seq 92 are <20.0% of the LLOQ. | Seq 4 and Seq 83 are < 5.0% internal standard peak of acceptable standards. |
| 24 | Seq 13 and Seq 68 are <20.0% of the LLOQ. | Seq 4 and Seq 59 are < 5.0% internal standard peak of acceptable standards. |
| 25 | Seq 13 and Seq 44 are <20.0% of the LLOQ. | Seq 4 and Seq 35 are < 5.0% internal standard peak of acceptable standards. |
| 26 | Seq 13 and Seq 44 are <20.0% of the LLOQ. | Seq 4 and Seq 35 are < 5.0% internal standard peak of acceptable standards. |

Note: Calibration Standards and QCs are arranged in a way to mitigate carryover in all validation runs.

## Table 10: Selectivity - Blank Assessment

Run 2 Selectivity Blank Screen

| Blank ID with or without IS | Blank Lot Number | Peak Area of Analyte | Peak Area of Internal Standard | Percent of LLOQ Peak Area | Percent of LLOQ Peak Area Ratio | Percent of IS in Blanks without IS |
|---|---|---|---|---|---|---|
| Blank 1 NO IS | V1872106P5-21-1 | 0 | 0 | 0.0 | NA | 0.0 |
| Blank 2 NO IS | V1872106P5-21-2 | 0 | 0 | 0.0 | NA | 0.0 |
| Blank 3 NO IS | V1872106P5-21-3 | 0 | 0 | 0.0 | NA | 0.0 |
| Blank 4 NO IS | V1872106P5-21-4 | 0 | 0 | 0.0 | NA | 0.0 |
| Blank 5 NO IS | V1872106P5-21-5 | 0 | 0 | 0.0 | NA | 0.0 |
| Blank 6 NO IS | V1872106P5-21-6 | 0 | 0 | 0.0 | NA | 0.0 |
| Blank 1 | V1872106P5-21-1 | 0 | 732469 | 0.0 | 0.0 | NA |
| Blank 2 | V1872106P5-21-2 | 0 | 782129 | 0.0 | 0.0 | NA |
| Blank 3 | V1872106P5-21-3 | 0 | 743116 | 0.0 | 0.0 | NA |
| Blank 4 | V1872106P5-21-4 | 0 | 807877 | 0.0 | 0.0 | NA |
| Blank 5 | V1872106P5-21-5 | 0 | 882423 | 0.0 | 0.0 | NA |
| Blank 6 | V1872106P5-21-6 | 0 | 762246 | 0.0 | 0.0 | NA |

Note: Average IS response are from all of the accepted standards and QCs

Criteria: A minimum of six different sources of human plasma (sodium heparin as anticoagulant) were screened. To be deemed acceptable, the peak response observed must be ≤ 20.0% of the mean response of the lower limit of quantitation (LLOQ) and ≤ 5.0% of the mean response of the internal standard. If any lots fail, further evaluation is needed.

Results: All matrix lots were found to be free of any significant interference at the retention times of the analytes of interest.

## Table 11: Matrix Effect

| Run Number: 3 | | | | | | | | |
|---|---|---|---|---|---|---|---|---|
| | **Low QC: 0.600 mcg/mL** | | | | **High QC: 150 mcg/mL** | | | |
| | **Drug Area** | **IS Area** | **Drug/IS Ratio** | **Average Drug/IS Ratio** | **Drug Area** | **IS Area** | **Drug/IS Ratio** | **Average Drug/IS Ratio** |
| ME1 | 16569 | 477809 | 0.0347 | 0.0328 | 4595467 | 515015 | 8.9230 | 8.2539 |
| | 14298 | 462690 | 0.0309 | | 5700490 | 686605 | 8.3024 | |
| | 17499 | 550094 | 0.0318 | | 6336071 | 774123 | 8.1848 | |
| | 18140 | 536113 | 0.0338 | | 6136944 | 806919 | 7.6054 | |
| ME2 | 16610 | 424221 | 0.0392 | 0.0380 | 4272192 | 505823 | 8.4460 | 8.3910 |
| | 18200 | 436835 | 0.0417 | | 5779489 | 660145 | 8.7549 | |
| | 18399 | 524650 | 0.0351 | | 6315329 | 773594 | 8.1636 | |
| | 17925 | 493762 | 0.0363 | | 6130570 | 747691 | 8.1993 | |
| ME3 | 14946 | 476714 | 0.0314 | 0.0339 | 4942279 | 522950 | 9.4508 | 8.5702 |
| | 16286 | 477098 | 0.0341 | | 5286771 | 676156 | 7.8189 | |
| | 17831 | 527515 | 0.0338 | | 5916943 | 715991 | 8.2640 | |
| | 17990 | 493532 | 0.0365 | | 6799314 | 777321 | 8.7471 | |
| ME4 | 16587 | 477434 | 0.0347 | 0.0337 | 4795637 | 550286 | 8.7148 | 8.8265 |
| | 14967 | 456439 | 0.0328 | | 6556026 | 654315 | 10.0197 | |
| | 16905 | 494189 | 0.0342 | | 6617255 | 753361 | 8.7836 | |
| | 17027 | 513970 | 0.0331 | | 5998148 | 770173 | 7.7881 | |
| ME5 | 18754 | 478401 | 0.0392 | 0.0380 | 5433520 | 601621 | 9.0315 | 8.7137 |
| | 18274 | 490775 | 0.0372 | | 6325593 | 746216 | 8.4769 | |
| | 18027 | 530572 | 0.0340 | | 6691285 | 743542 | 8.9992 | |
| | 20252 | 486568 | 0.0416 | | 6565809 | 786589 | 8.3472 | |
| ME6 | 13066 | 495287 | 0.0264 | 0.0346 | 5479159 | 633103 | 8.6545 | 8.7043 |
| | 21070 | 497551 | 0.0423 | | 5901274 | 653455 | 9.0309 | |
| | 17259 | 531481 | 0.0325 | | 6115106 | 709617 | 8.6175 | |
| | 17595 | 475341 | 0.0370 | | 6664776 | 782769 | 8.5144 | |
| **Mean** | | | | 0.0352 | | | | 8.5766 |
| **N** | | | | 6 | | | | 6 |
| **SD** | | | | 0.0023 | | | | 0.2173 |
| **%CV** | | | | 6.5 | | | | 2.5 |
| **Meets Acceptance Criteria: %CV of average drug/IS ratio among 6 lots must be <15.0%.** | | | | | | | | |
| Met criteria at Low QC level: **YES** | | | | | | | | |
| Met criteria at High QC level: **YES** | | | | | | | | |

## Table 12: Interference Evaluation

| Run Date | Curve Number | Comed1[2] low 0.600 µg/mL | %Bias | Comed1[2] high 150 µg/mL | %Bias |
|---|---|---|---|---|---|
| 03-Nov-2022 | 1 | 0.567 | -5.5 | 166 | 10.7 |
| | | 0.601 | 0.2 | 138 | -8.0 |
| | | 0.586 | -2.3 | 159 | 6.0 |
| | | 0.633 | 5.5 | 145 | -3.3 |
| | | 0.517 | -13.8 | 148 | -1.3 |
| | | 0.637 | 6.2 | 147 | -2.0 |
| Mean | | 0.590 | | 151 | |
| SD | | 0.0448 | | 10.2 | |
| %CV | | 7.6 | | 6.8 | |
| %Bias | | -1.7 | | 0.7 | |
| n | | 6 | | 6 | |

Criteria: To be considered acceptable, the mean %Bias at each level was also required to be within ± 15.0% and the %CV was required to be ≤ 15.0%.

Results: The acceptance criteria were met.

## Table 13: Hemolytic Effect Evaluation

| Run Date | Curve Number | Hemolytic QC low 0.600 µg/mL | %Bias | Hemolytic QC high 150 µg/mL | %Bias |
|---|---|---|---|---|---|
| 04-Nov-2022 | 2 | 0.511 | -14.8 | 147 | -2.0 |
| | | ##0.489 | -18.5 | 149 | -0.7 |
| | | ##0.445 | -25.8 | 147 | -2.0 |
| | | ##0.458 | -23.7 | 149 | -0.7 |
| | | ##0.435 | -27.5 | 135 | -10.0 |
| | | ##0.451 | -24.8 | 140 | -6.7 |
| Mean | | 0.465 | | 145 | |
| SD | | 0.0291 | | 5.72 | |
| %CV | | 6.3 | | 3.9 | |
| %Bias | | -22.5 | | -3.3 | |
| n | | 6 | | 6 | |
| ## > 15%Bias | | | | | |

| Run Date | Curve Number | 0.5% Hemo QC low 0.600 µg/mL | %Bias | 1% Hemo QC low 0.600 µg/mL | %Bias | 0.5% Hemo QC high 150 µg/mL | %Bias | 1% Hemo QC high 150 µg/mL | %Bias |
|---|---|---|---|---|---|---|---|---|---|
| 08-Nov-2022 | 7 | 0.528 | -12.0 | 0.533 | -11.2 | #121 | -19.3 | #126 | -16.0 |
| | | #0.508 | -15.3 | #0.475 | -20.8 | 130 | -13.3 | #122 | -18.7 |
| | | 0.566 | -5.7 | 0.544 | -9.3 | 132 | -12.0 | #118 | -21.3 |
| | | #0.468 | -22.0 | 0.629 | 4.8 | #121 | -19.3 | 138 | -8.0 |
| | | 0.621 | 3.5 | 0.523 | -12.8 | 135 | -10.0 | 135 | -10.0 |
| | | #0.507 | -15.5 | 0.628 | 4.7 | #126 | -16.0 | #119 | -20.7 |
| Mean | | 0.533 | | 0.555 | | 128 | | 126 | |
| SD | | 0.0536 | | 0.0614 | | 5.82 | | 8.41 | |
| %CV | | 10.1 | | 11.1 | | 4.5 | | 6.7 | |
| %Bias | | -11.2 | | -7.5 | | -14.7 | | -16.0 | |
| n | | 6 | | 6 | | 6 | | 6 | |
| # > 15%Bias | | | | | | | | | |

| Run Date | Curve Number | 2% Hemo QC low 0.600 µg/mL | %Bias | 2% Hemo QC high 150 µg/mL | %Bias |
|---|---|---|---|---|---|
| 08-Nov-2022 | 7 | #0.464 | -22.7 | 131 | -12.7 |
| | | #0.474 | -21.0 | #118 | -21.3 |
| | | #0.509 | -15.2 | #123 | -18.0 |
| | | 0.564 | -6.0 | 135 | -10.0 |
| | | 0.629 | 4.8 | #124 | -17.3 |
| | | #0.475 | -20.8 | 128 | -14.7 |
| Mean | | 0.519 | | 127 | |
| SD | | 0.0651 | | 6.09 | |
| %CV | | 12.5 | | 4.8 | |
| %Bias | | -13.5 | | -15.3 | |
| n | | 6 | | 6 | |
| # > 15%Bias | | | | | |

Criteria: To be considered acceptable, the mean %Bias at each level was also required to be within ± 15.0% and the %CV was required to be ≤ 15.0%.

Results: Low hemolytic QCs failed in run 2 and was repeated in run 7. Hemolytic QCs at 1% and 2% do not meet acceptance criteria. The acceptance criteria were met for hemolytic QCs at 0.5%.

## Table 14: Lipemic Effect Evaluation

| Run Date | Curve Number | Lipemic QC low 0.600 µg/mL | %Bias | Lipemic QC high 150 µg/mL | %Bias |
|---|---|---|---|---|---|
| 04-Nov-2022 | 2 | 0.639 | 6.5 | 145 | -3.3 |
| | | 0.614 | 2.3 | 151 | 0.7 |
| | | 0.612 | 2.0 | 156 | 4.0 |
| | | 0.540 | -10.0 | 137 | -8.7 |
| | | 0.628 | 4.7 | 147 | -2.0 |
| | | 0.615 | 2.5 | 144 | -4.0 |
| Mean | | 0.608 | | 147 | |
| SD | | 0.0349 | | 6.47 | |
| %CV | | 5.7 | | 4.4 | |
| %Bias | | 1.3 | | -2.0 | |
| n | | 6 | | 6 | |

Criteria: To be considered acceptable, the mean %Bias at each level was also required to be within ± 15.0% and the %CV was required to be ≤ 15.0%.

Results: The acceptance criteria were met.

## Table 15: Summary of Standard Curves

Analytical Performance: Back-Calculated Concentrations (µg/mL) of Ertapenem Calibration Standard in (Human) (Plasma) in (Study V1872106P5)

| Assay Date | Analytical Run Number | Cal 0.200 0.200 µg/mL | Cal 0.400 0.400 µg/mL | Cal 4.00 4.00 µg/mL | Cal 20.0 20.0 µg/mL | Cal 40.0 40.0 µg/mL | Cal 80.0 80.0 µg/mL | Cal 160 160 µg/mL | Cal 200 200 µg/mL |
|---|---|---|---|---|---|---|---|---|---|
| 03-Nov-2022 | 1 | 0.197 | 0.424 | 4.28 | 20.7 | 38.8 | 79.8 | 170 | 202 |
| | | 0.195 | 0.405 | 3.96 | 19.9 | 39.1 | 77.4 | 160 | 180 |
| 04-Nov-2022 | 2 | *0.290 | 0.374 | 3.79 | 19.5 | 38.6 | 88.7 | 158 | 211 |
| | | 0.217 | 0.359 | 4.21 | 20.3 | 39.9 | 69.3 | 166 | 215 |
| 09-Nov-2022 | 9 | 0.200 | 0.406 | 4.17 | 19.4 | 41.9 | 74.6 | 167 | *162 |
| | | 0.194 | 0.411 | 4.33 | 19.0 | 39.7 | 73.0 | 169 | 190 |
| 07-Dec-2022 | 13 | *0.271 | 0.392 | 4.12 | 20.2 | 40.9 | 77.8 | 170 | 187 |
| | | 0.195 | 0.425 | 4.12 | 20.4 | 35.6 | 78.7 | 176 | 184 |
| Mean | | 0.200 | 0.400 | 4.12 | 19.9 | 39.3 | 77.4 | 167 | 196 |
| S.D. | | 0.00876 | 0.0233 | 0.175 | 0.580 | 1.86 | 5.71 | 5.78 | 13.8 |
| %CV | | 4.4 | 5.8 | 4.2 | 2.9 | 4.7 | 7.4 | 3.5 | 7.0 |
| %Bias | | 0.0 | 0.0 | 3.0 | -0.5 | -1.8 | -3.3 | 4.4 | -2.0 |
| n | | 6 | 8 | 8 | 8 | 8 | 8 | 8 | 7 |
| Reason Deactivated | | | | | | | | | |
| * Excluded due to being outside acceptance criteria | | | | | | | | | |

Calibration Curve Parameters for Ertapenem Calibration Standards in Human Plasma (Study V1872106P5)

| Run Date | Curve Number | Slope | Intercept | R-Squared | LLOQ | ULOQ |
|---|---|---|---|---|---|---|
| 03-Nov-2022 | 1 | 0.05285 | -0.00002 | 0.9977 | 0.200 | 200 |
| 04-Nov-2022 | 2 | 0.05417 | 0.00011 | 0.9942 | 0.200 | 200 |
| 09-Nov-2022 | 9 | 0.05501 | -0.00043 | 0.9968 | 0.200 | 200 |
| 07-Dec-2022 | 13 | 0.04636 | 0.00164 | 0.9961 | 0.200 | 200 |

See Table 8 for runs not used for precision/accuracy evaluation.

## Table 16: Precision and Accuracy (Intra-Assay)

Human Plasma Ertapenem Concentrations in Quality Controls for 3 separate batch runs (Study V1872106P5)

| Run Date | Curve Number | QCS 0.200 0.200 µg/mL | %Bias | QCS 0.600 0.600 µg/mL | %Bias | QCS 100 100 µg/mL | %Bias | QCS 150 150 µg/mL | %Bias |
|---|---|---|---|---|---|---|---|---|---|
| 03-Nov-2022 | 1 | 0.191 | -4.5 | 0.594 | -1.0 | 108 | 8.0 | 142 | -5.3 |
| | | 0.234 | 17.0 | 0.603 | 0.5 | 105 | 5.0 | 164 | 9.3 |
| | | 0.217 | 8.5 | 0.598 | -0.3 | 106 | 6.0 | 149 | -0.7 |
| | | 0.199 | -0.5 | 0.620 | 3.3 | 103 | 3.0 | 150 | 0.0 |
| | | 0.221 | 10.5 | 0.565 | -5.8 | 104 | 4.0 | 154 | 2.7 |
| | | 0.229 | 14.5 | 0.619 | 3.2 | 95.1 | -4.9 | 146 | -2.7 |
| Intrarun Mean | | 0.215 | | 0.600 | | 104 | | 151 | |
| Intrarun SD | | 0.0169 | | 0.0202 | | 4.47 | | 7.60 | |
| Intrarun %CV | | 7.9 | | 3.4 | | 4.3 | | 5.0 | |
| Intrarun %Bias | | 7.5 | | 0.0 | | 4.0 | | 0.7 | |
| n | | 6 | | 6 | | 6 | | 6 | |
| 04-Nov-2022 | 2 | #0.245 | 22.5 | 0.655 | 9.2 | 108 | 8.0 | 159 | 6.0 |
| | | 0.220 | 10.0 | 0.667 | 11.2 | ##117 | 17.0 | 155 | 3.3 |
| | | 0.237 | 18.5 | ##0.692 | 15.3 | 108 | 8.0 | 153 | 2.0 |
| | | 0.206 | 3.0 | ##0.717 | 19.5 | 102 | 2.0 | 166 | 10.7 |
| | | 0.196 | -2.0 | 0.606 | 1.0 | 91.8 | -8.2 | 132 | -12.0 |
| | | 0.195 | -2.5 | 0.669 | 11.5 | 105 | 5.0 | 146 | -2.7 |
| Intrarun Mean | | 0.217 | | 0.668 | | 105 | | 152 | |
| Intrarun SD | | 0.0212 | | 0.0374 | | 8.30 | | 11.8 | |
| Intrarun %CV | | 9.8 | | 5.6 | | 7.9 | | 7.8 | |
| Intrarun %Bias | | 8.5 | | 11.3 | | 5.0 | | 1.3 | |
| n | | 6 | | 6 | | 6 | | 6 | |
| 09-Nov-2022 | 9 | 0.232 | 16.0 | 0.591 | -1.5 | 101 | 1.0 | 141 | -6.0 |
| | | 0.231 | 15.5 | 0.639 | 6.5 | 86.6 | -13.4 | 138 | -8.0 |
| | | 0.215 | 7.5 | 0.616 | 2.7 | 96.1 | -3.9 | 134 | -10.7 |
| | | 0.225 | 12.5 | 0.680 | 13.3 | 94.5 | -5.5 | 162 | 8.0 |
| | | 0.211 | 5.5 | 0.572 | -4.7 | 97.2 | -2.8 | 128 | -14.7 |
| | | 0.205 | 2.5 | ##0.509 | -15.2 | 92.5 | -7.5 | 130 | -13.3 |
| Intrarun Mean | | 0.220 | | 0.601 | | 94.7 | | 139 | |
| Intrarun SD | | 0.0111 | | 0.0588 | | 4.87 | | 12.3 | |
| Intrarun %CV | | 5.0 | | 9.8 | | 5.1 | | 8.8 | |
| Intrarun %Bias | | 10.0 | | 0.2 | | -5.3 | | -7.3 | |
| n | | 6 | | 6 | | 6 | | 6 | |
| 07-Dec-2022 | 13 | 0.206 | 3.0 | 0.615 | 2.5 | 101 | 1.0 | 149 | -0.7 |
| | | #0.146 | -27.0 | 0.632 | 5.3 | 88.4 | -11.6 | 157 | 4.7 |
| | | 0.189 | -5.5 | 0.546 | -9.0 | 97.3 | -2.7 | 151 | 0.7 |
| | | 0.196 | -2.0 | 0.557 | -7.2 | ##82.5 | -17.5 | 154 | 2.7 |
| | | 0.215 | 7.5 | ##0.444 | -26.0 | 98.6 | -1.4 | 135 | -10.0 |
| | | #0.136 | -32.0 | 0.663 | 10.5 | 97.3 | -2.7 | 154 | 2.7 |
| Intrarun Mean | | 0.181 | | 0.576 | | 94.2 | | 150 | |
| Intrarun SD | | 0.0326 | | 0.0786 | | 7.15 | | 7.85 | |
| Intrarun %CV | | 18.0 | | 13.6 | | 7.6 | | 5.2 | |
| Intrarun %Bias | | -9.5 | | -4.0 | | -5.8 | | 0.0 | |
| n | | 6 | | 6 | | 6 | | 6 | |
| # > 20%Bias | | | | | | | | | |
| ## > 15%Bias | | | | | | | | | |

Criteria: At each level, the %CV was required to be ≤ 15.0%, except at the LLOQ level, where ≤ 20.0% was accepted. The mean %Bias at each level was also required to be within ± 15.0%, except at the LLOQ, where ± 20.0% was accepted.

Results: The acceptance criteria were met. Run size (n=120) was completed in run 2 and met acceptance criteria.

## Table 17: Precision and Accuracy (Inter-Assay)

Human Plasma Ertapenem Concentrations in Quality Controls for 4 separate batch runs (Study V1872106P5)

| Run Date | Curve Number | QCS 0.200 0.200 µg/mL | %Bias | QCS 0.600 0.600 µg/mL | %Bias | QCS 100 100 µg/mL | %Bias | QCS 150 150 µg/mL | %Bias |
|---|---|---|---|---|---|---|---|---|---|
| 03-Nov-2022 | 1 | 0.191 | -4.5 | 0.594 | -1.0 | 108 | 8.0 | 142 | -5.3 |
| | | 0.234 | 17.0 | 0.603 | 0.5 | 105 | 5.0 | 164 | 9.3 |
| | | 0.217 | 8.5 | 0.598 | -0.3 | 106 | 6.0 | 149 | -0.7 |
| | | 0.199 | -0.5 | 0.620 | 3.3 | 103 | 3.0 | 150 | 0.0 |
| | | 0.221 | 10.5 | 0.565 | -5.8 | 104 | 4.0 | 154 | 2.7 |
| | | 0.229 | 14.5 | 0.619 | 3.2 | 95.1 | -4.9 | 146 | -2.7 |
| 04-Nov-2022 | 2 | #0.245 | 22.5 | 0.655 | 9.2 | 108 | 8.0 | 159 | 6.0 |
| | | 0.220 | 10.0 | 0.667 | 11.2 | ##117 | 17.0 | 155 | 3.3 |
| | | 0.237 | 18.5 | ##0.692 | 15.3 | 108 | 8.0 | 153 | 2.0 |
| | | 0.206 | 3.0 | ##0.717 | 19.5 | 102 | 2.0 | 166 | 10.7 |
| | | 0.196 | -2.0 | 0.606 | 1.0 | 91.8 | -8.2 | 132 | -12.0 |
| | | 0.195 | -2.5 | 0.669 | 11.5 | 105 | 5.0 | 146 | -2.7 |
| 09-Nov-2022 | 9 | 0.232 | 16.0 | 0.591 | -1.5 | 101 | 1.0 | 141 | -6.0 |
| | | 0.231 | 15.5 | 0.639 | 6.5 | 86.6 | -13.4 | 138 | -8.0 |
| | | 0.215 | 7.5 | 0.616 | 2.7 | 96.1 | -3.9 | 134 | -10.7 |
| | | 0.225 | 12.5 | 0.680 | 13.3 | 94.5 | -5.5 | 162 | 8.0 |
| | | 0.211 | 5.5 | 0.572 | -4.7 | 97.2 | -2.8 | 128 | -14.7 |
| | | 0.205 | 2.5 | ##0.509 | -15.2 | 92.5 | -7.5 | 130 | -13.3 |
| 07-Dec-2022 | 13 | 0.206 | 3.0 | 0.615 | 2.5 | 101 | 1.0 | 149 | -0.7 |
| | | #0.146 | -27.0 | 0.632 | 5.3 | 88.4 | -11.6 | 157 | 4.7 |
| | | 0.189 | -5.5 | 0.546 | -9.0 | 97.3 | -2.7 | 151 | 0.7 |
| | | 0.196 | -2.0 | 0.557 | -7.2 | ##82.5 | -17.5 | 154 | 2.7 |
| | | 0.215 | 7.5 | ##0.444 | -26.0 | 98.6 | -1.4 | 135 | -10.0 |
| | | #0.136 | -32.0 | 0.663 | 10.5 | 97.3 | -2.7 | 154 | 2.7 |
| Mean Concentration Found (µg/mL) | | 0.208 | | 0.611 | | 99.4 | | 148 | |
| Inter-run SD | | 0.0259 | | 0.0608 | | 7.88 | | 10.9 | |
| Inter-run %CV | | 12.5 | | 10.0 | | 7.9 | | 7.4 | |
| Inter-run %Bias | | 4.0 | | 1.8 | | -0.6 | | -1.3 | |
| n | | 24 | | 24 | | 24 | | 24 | |
| # > 20%Bias | | | | | | | | | |
| ## > 15%Bias | | | | | | | | | |

Criteria: At each level, the %CV was required to be ≤ 15.0%, except at the LLOQ level, where ≤ 20.0% was accepted. In addition, the mean %Bias at each level was required to be within ± 15.0% from nominal value, except at the LLOQ, where ± 20.0% was accepted.

Results: The acceptance criteria were met.

## Table 18: Standard and QC of Failed Runs

Analytical Performance: Back-Calculated Concentrations (µg/mL) of Ertapenem Calibration Standard in (Human) (Plasma) in (Study V1872106P5) (Rejected Runs)

| Assay Date | Analytical Run Number | Cal 0.200 0.200 µg/mL | Cal 0.400 0.400 µg/mL | Cal 4.00 4.00 µg/mL | Cal 20.0 20.0 µg/mL | Cal 40.0 40.0 µg/mL | Cal 80.0 80.0 µg/mL | Cal 160 160 µg/mL | Cal 200 200 µg/mL |
|---|---|---|---|---|---|---|---|---|---|
| 05-Dec-2022 | 12 | 0.199 | *0.481 | 3.85 | 18.8 | 39.6 | 84.4 | 146 | 194 |
| | | 0.210 | 0.367 | 4.10 | 18.7 | 40.3 | 85.1 | 170 | 226 |
| Mean | | 0.205 | 0.367 | 3.98 | 18.8 | 40.0 | 84.8 | 158 | 210 |
| %Bias | | 2.5 | -8.3 | -0.5 | -6.0 | 0.0 | 6.0 | -1.3 | 5.0 |
| Reason Deactivated | | | | | | | | | |
| * Excluded due to being outside acceptance criteria | | | | | | | | | |

Calibration Curve Parameters for Ertapenem Calibration Standards in Human Plasma (Study V1872106P5) (Rejected Runs)

| Run Date | Curve Number | Slope | Intercept | R-Squared | LLOQ | ULOQ |
|---|---|---|---|---|---|---|
| 05-Dec-2022 | 12 | 0.04523 | 0.00068 | 0.9953 | 0.200 | 200 |

Analytical Performance of Ertapenem Quality Control Samples in Human Plasma (Study V1872106P5) (Rejected Runs)

| Run Date | Curve Number | QCS 0.200 0.200 µg/mL | %Bias | QCS 0.600 0.600 µg/mL | %Bias | QCS 100 100 µg/mL | %Bias | QCS 150 150 µg/mL | %Bias |
|---|---|---|---|---|---|---|---|---|---|
| 05-Dec-2022 | 12 | #0.257 | 28.5 | 0.682 | 13.7 | 85.0 | -15.0 | 144 | -4.0 |
| | | #0.244 | 22.0 | 0.664 | 10.7 | 90.7 | -9.3 | 143 | -4.7 |
| | | #0.128 | -36.0 | ##0.733 | 22.2 | 95.9 | -4.1 | 149 | -0.7 |
| | | 0.224 | 12.0 | 0.587 | -2.2 | 86.7 | -13.3 | 131 | -12.7 |
| | | #0.245 | 22.5 | ##0.716 | 19.3 | 100 | 0.0 | 133 | -11.3 |
| | | 0.217 | 8.5 | 0.617 | 2.8 | 95.6 | -4.4 | 143 | -4.7 |
| Mean | | 0.219 | | 0.667 | | 92.3 | | 141 | |
| S.D. | | 0.0470 | | 0.0564 | | 5.84 | | 6.98 | |
| %CV | | 21.5 | | 8.5 | | 6.3 | | 5.0 | |
| %Bias | | 9.5 | | 11.2 | | -7.7 | | -6.0 | |
| n | | 6 | | 6 | | 6 | | 6 | |
| # > 20%Bias | | | | | | | | | |
| ## > 15%Bias | | | | | | | | | |

Criteria: For each run, the calculated concentrations of at least 75% of the calibration standards are required to interpolate within ± 15.0% from nominal with a minimum of six calibration standard levels, except for the LLOQ for which it was required to be within ± 20.0%. In order for the run to be acceptable, at least one QC at each concentration level and two-thirds of the total number of QC samples were required to interpolate within ± 15.0% from nominal.

Results: The acceptance criteria were not met.

## Table 19: Standards and QCs of Non-P&A Runs

Analytical Performance: Back-Calculated Concentrations (µg/mL) of Ertapenem Calibration Standard in (Human) (Plasma)

| Assay Date | Analytical Run Number | Cal 0.200 0.200 µg/mL | Cal 0.400 0.400 µg/mL | Cal 4.00 4.00 µg/mL | Cal 20.0 20.0 µg/mL | Cal 40.0 40.0 µg/mL | Cal 80.0 80.0 µg/mL | Cal 160 160 µg/mL | Cal 200 200 µg/mL |
|---|---|---|---|---|---|---|---|---|---|
| 07-Nov-2022 | 5 | 0.210 | 0.439 | 4.35 | 19.3 | 38.6 | 89.2 | 151 | 201 |
| | | 0.180 | *0.308 | 3.84 | 20.1 | 39.1 | 79.3 | 155 | 194 |
| 08-Nov-2022 | 7 | 0.238 | 0.388 | *4.82 | 20.3 | 40.4 | 82.9 | 157 | 190 |
| | | 0.174 | 0.359 | 4.34 | 20.0 | 42.4 | 74.0 | 138 | 229 |
| 20-Dec-2022 | 15 | 0.196 | 0.395 | 4.13 | 20.9 | 40.7 | 78.8 | 153 | 190 |
| | | *0.355 | 0.420 | 4.07 | 21.2 | 40.6 | 79.8 | 154 | 190 |
| 27-Dec-2022 | 16 | 0.216 | 0.363 | 4.29 | *23.4 | 43.3 | 79.8 | 153 | *164 |
| | | 0.181 | 0.441 | 4.36 | 20.1 | 37.9 | 79.6 | 137 | *153 |
| 04-Jan-2023 | 17 | 0.198 | 0.405 | 4.10 | 21.2 | 40.7 | 78.7 | 152 | 189 |
| | | 0.195 | 0.420 | 4.22 | 20.8 | 40.5 | 79.7 | 149 | 192 |
| 09-Jan-2023 | 18 | 0.194 | 0.436 | 4.11 | 20.1 | 39.5 | 78.6 | 153 | 191 |
| | | 0.193 | 0.416 | 4.25 | 20.2 | 40.8 | 80.3 | 154 | 194 |
| 13-Jan-2023 | 19 | 0.196 | 0.396 | 4.12 | 20.2 | 40.1 | 80.2 | 156 | 191 |
| | | 0.201 | 0.412 | 4.06 | 20.9 | 40.5 | 80.0 | 157 | 192 |
| 16-Jan-2023 | 20 | 0.198 | 0.412 | 4.11 | 20.9 | 40.6 | 78.6 | 152 | 185 |
| | | 0.193 | 0.420 | 4.33 | 20.7 | 41.8 | 80.7 | 154 | 177 |
| 24-Jan-2023 | 21 | 0.205 | 0.413 | 4.14 | 20.6 | 40.9 | 78.6 | 156 | 190 |
| | | 0.191 | 0.399 | 4.21 | 20.0 | 41.3 | 80.4 | 149 | 194 |
| 02-Feb-2023 | 22 | 0.194 | 0.376 | 3.98 | 20.4 | 41.2 | 82.6 | 159 | 192 |
| | | 0.211 | 0.401 | 4.02 | 19.9 | 40.8 | 82.1 | 158 | 193 |
| 07-Feb-2023 | 23 | 0.204 | 0.414 | 4.12 | 20.5 | 41.2 | 80.1 | 157 | 193 |
| | | 0.192 | 0.403 | 4.06 | 20.3 | 40.4 | 79.5 | 156 | 189 |
| 13-Feb-2023 | 24 | 0.207 | 0.422 | 4.03 | 20.5 | 41.0 | 79.1 | 156 | 193 |
| | | 0.189 | 0.391 | 4.17 | 20.3 | 40.0 | 78.6 | 157 | 195 |
| 15-Feb-2023 | 25 | 0.195 | 0.386 | 3.96 | 19.4 | 40.7 | 81.7 | 160 | 193 |
| | | 0.208 | 0.402 | 4.09 | 20.8 | 40.6 | 80.4 | 164 | 189 |
| 20-Feb-2023 | 26 | 0.203 | 0.401 | 3.92 | 19.7 | 41.0 | 77.7 | 159 | 200 |
| | | 0.202 | 0.382 | 3.97 | 20.4 | 41.0 | 81.2 | 161 | 200 |
| Mean | | 0.199 | 0.404 | 4.12 | 20.4 | 40.6 | 80.1 | 154 | 193 |
| S.D. | | 0.0124 | 0.0206 | 0.137 | 0.485 | 1.04 | 2.46 | 5.83 | 8.67 |
| %CV | | 6.2 | 5.1 | 3.3 | 2.4 | 2.6 | 3.1 | 3.8 | 4.5 |
| %Bias | | -0.5 | 1.0 | 3.0 | 2.0 | 1.5 | 0.1 | -3.8 | -3.5 |
| n | | 27 | 27 | 27 | 27 | 28 | 28 | 28 | 26 |
| Reason Deactivated | | | | | | | | | |
| * Excluded due to being outside acceptance criteria | | | | | | | | | |

Calibration Curve Parameters for Ertapenem Calibration Standards in Human Plasma (Study V1872106P5)

| Run Date | Curve Number | Slope | Intercept | R-Squared | LLOQ | ULOQ |
|---|---|---|---|---|---|---|
| 07-Nov-2022 | 5 | 0.05831 | 0.00165 | 0.9957 | 0.200 | 200 |
| 08-Nov-2022 | 7 | 0.05438 | 0.00077 | 0.9888 | 0.200 | 200 |
| 20-Dec-2022 | 15 | 0.05641 | 0.00135 | 0.9985 | 0.200 | 200 |
| 27-Dec-2022 | 16 | 0.40132 | 0.02887 | 0.9912 | 0.200 | 160 |
| 04-Jan-2023 | 17 | 0.04076 | 0.00112 | 0.9979 | 0.200 | 200 |
| 09-Jan-2023 | 18 | 0.06171 | 0.00150 | 0.9980 | 0.200 | 200 |
| 13-Jan-2023 | 19 | 0.06352 | 0.00203 | 0.9992 | 0.200 | 200 |
| 16-Jan-2023 | 20 | 0.06235 | 0.00185 | 0.9966 | 0.200 | 200 |
| 24-Jan-2023 | 21 | 0.05871 | 0.00009 | 0.9984 | 0.200 | 200 |
| 02-Feb-2023 | 22 | 0.02793 | 0.00008 | 0.9988 | 0.200 | 200 |
| 07-Feb-2023 | 23 | 0.03931 | 0.00025 | 0.9991 | 0.200 | 200 |
| 13-Feb-2023 | 24 | 0.04694 | 0.00180 | 0.9989 | 0.200 | 200 |
| 15-Feb-2023 | 25 | 0.04324 | -0.00019 | 0.9990 | 0.200 | 200 |
| 20-Feb-2023 | 26 | 0.03736 | -0.00048 | 0.9995 | 0.200 | 200 |

## Table 19: Standards and QCs of Non-P&A Runs (Continued)

Human Plasma Ertapenem Concentrations in Quality Controls for 14 separate batch runs (Study V1872106P5)

| Run Date | Curve Number | QCS 0.600 0.600 µg/mL | %Bias | QCS 100 100 µg/mL | %Bias | QCS 150 150 µg/mL | %Bias |
|---|---|---|---|---|---|---|---|
| 07-Nov-2022 | 5 | 0.639 | 6.5 | 110 | 10.0 | 141 | -6.0 |
| | | 0.521 | -13.2 | 107 | 7.0 | 164 | 9.3 |
| Intrarun Mean | | 0.580 | | 109 | | 153 | |
| Intrarun %Bias | | -3.3 | | 9.0 | | 2.0 | |
| 08-Nov-2022 | 7 | 0.592 | -1.3 | 110 | 10.0 | 148 | -1.3 |
| | | #1.07 | 78.3 | 114 | 14.0 | 156 | 4.0 |
| | | 0.629 | 4.8 | 98.3 | -1.7 | 149 | -0.7 |
| | | #0.721 | 20.2 | 99.1 | -0.9 | 169 | 12.7 |
| | | #0.487 | -18.8 | 108 | 8.0 | 163 | 8.7 |
| | | 0.643 | 7.2 | 87.6 | -12.4 | 160 | 6.7 |
| Intrarun Mean | | 0.690 | | 103 | | 158 | |
| Intrarun SD | | 0.201 | | 9.69 | | 8.17 | |
| Intrarun %CV | | 29.1 | | 9.4 | | 5.2 | |
| Intrarun %Bias | | 15.0 | | 3.0 | | 5.3 | |
| n | | 6 | | 6 | | 6 | |
| 20-Dec-2022 | 15 | 0.601 | 0.2 | 96.1 | -3.9 | 141 | -6.0 |
| | | 0.608 | 1.3 | 100 | 0.0 | 146 | -2.7 |
| Intrarun Mean | | 0.605 | | 98.1 | | 144 | |
| Intrarun %Bias | | 0.8 | | -1.9 | | -4.0 | |
| 27-Dec-2022 | 16 | 0.596 | -0.7 | 97.4 | -2.6 | 148 | -1.3 |
| | | 0.673 | 12.2 | 98.8 | -1.2 | 137 | -8.7 |
| Intrarun Mean | | 0.635 | | 98.1 | | 143 | |
| Intrarun %Bias | | 5.8 | | -1.9 | | -4.7 | |
| 04-Jan-2023 | 17 | 0.651 | 8.5 | 103 | 3.0 | 151 | 0.7 |
| | | 0.674 | 12.3 | 104 | 4.0 | 151 | 0.7 |
| Intrarun Mean | | 0.663 | | 104 | | 151 | |
| Intrarun %Bias | | 10.5 | | 4.0 | | 0.7 | |
| 09-Jan-2023 | 18 | 0.683 | 13.8 | 107 | 7.0 | 155 | 3.3 |
| | | #0.720 | 20.0 | 106 | 6.0 | 159 | 6.0 |
| Intrarun Mean | | 0.702 | | 107 | | 157 | |
| Intrarun %Bias | | 17.0 | | 7.0 | | 4.7 | |
| 13-Jan-2023 | 19 | 0.651 | 8.5 | 105 | 5.0 | 154 | 2.7 |
| | | 0.644 | 7.3 | 105 | 5.0 | 153 | 2.0 |
| Intrarun Mean | | 0.648 | | 105 | | 154 | |
| Intrarun %Bias | | 8.0 | | 5.0 | | 2.7 | |
| 16-Jan-2023 | 20 | 0.594 | -1.0 | 101 | 1.0 | 145 | -3.3 |
| | | 0.610 | 1.7 | 101 | 1.0 | 144 | -4.0 |
| Intrarun Mean | | 0.602 | | 101 | | 145 | |
| Intrarun %Bias | | 0.3 | | 1.0 | | -3.3 | |
| 24-Jan-2023 | 21 | 0.604 | 0.7 | 94.3 | -5.7 | 138 | -8.0 |
| | | 0.590 | -1.7 | 97.0 | -3.0 | 145 | -3.3 |
| Intrarun Mean | | 0.597 | | 95.7 | | 142 | |
| Intrarun %Bias | | -0.5 | | -4.3 | | -5.3 | |
| 02-Feb-2023 | 22 | 0.603 | 0.5 | 95.6 | -4.4 | 146 | -2.7 |
| | | 0.608 | 1.3 | 97.2 | -2.8 | 143 | -4.7 |
| Intrarun Mean | | 0.606 | | 96.4 | | 145 | |
| Intrarun %Bias | | 1.0 | | -3.6 | | -3.3 | |
| 07-Feb-2023 | 23 | 0.582 | -3.0 | 97.5 | -2.5 | 145 | -3.3 |
| | | 0.580 | -3.3 | 96.1 | -3.9 | 144 | -4.0 |
| Intrarun Mean | | 0.581 | | 96.8 | | 145 | |
| Intrarun %Bias | | -3.2 | | -3.2 | | -3.3 | |
| 13-Feb-2023 | 24 | 0.562 | -6.3 | 97.1 | -2.9 | 146 | -2.7 |
| | | 0.597 | -0.5 | 98.5 | -1.5 | 145 | -3.3 |
| Intrarun Mean | | 0.580 | | 97.8 | | 146 | |
| Intrarun %Bias | | -3.3 | | -2.2 | | -2.7 | |

## Table 19: Standards and QCs of Non-P&A Runs (Continued)

| Run Date | Curve Number | QCS 0.600 0.600 µg/mL | %Bias | QCS 100 100 µg/mL | %Bias | QCS 150 150 µg/mL | %Bias |
|---|---|---|---|---|---|---|---|
| 15-Feb-2023 | 25 | 0.600 | 0.0 | 102 | 2.0 | 152 | 1.3 |
| | | 0.612 | 2.0 | 99.6 | -0.4 | 150 | 0.0 |
| Intrarun Mean | | 0.606 | | 101 | | 151 | |
| Intrarun %Bias | | 1.0 | | 1.0 | | 0.7 | |
| 20-Feb-2023 | 26 | 0.590 | -1.7 | 101 | 1.0 | 148 | -1.3 |
| | | 0.635 | 5.8 | 103 | 3.0 | 149 | -0.7 |
| Intrarun Mean | | 0.613 | | 102 | | 149 | |
| Intrarun %Bias | | 2.2 | | 2.0 | | -0.7 | |
| # > 15%Bias | | | | | | | |

| Run Date | Curve Number | Dil QC 800 x5 800 µg/mL | %Bias |
|---|---|---|---|
| 07-Dec-2022 | 13 | ##670 | -16.3 |
| | | ##640 | -20.0 |
| | | 721 | -9.9 |
| | | 813 | 1.6 |
| | | 800 | 0.0 |
| | | 812 | 1.5 |
| Mean | | 743 | |
| SD | | 76.6 | |
| %CV | | 10.3 | |
| %Bias | | -7.1 | |
| n | | 6 | |
| ## > 15%Bias | | | |

Criteria: For each run, the calculated concentrations of at least 75% of the calibration standards are required to interpolate within ± 15.0% from nominal with a minimum of six calibration standard levels, except for the LLOQ for which it was required to be within ± 20.0%. In order for the run to be acceptable, at least one QC at each concentration level and two-thirds of the total number of QC samples were required to interpolate within ± 15.0% from nominal.

Results: Acceptance criteria were met.

## Table 20: Precision and Accuracy of Sample Dilution

| Dilution Factor | | 2x | | 5x | |
|---|---|---|---|---|---|
| **Run Date** | **Curve Number** | **Dil QC 150 150 µg/mL** | **%Bias** | **Dil QC 800 x5 800 µg/mL** | **%Bias** |
| 03-Nov-2022 | 1 | 156 | 4.0 | 799 | -0.1 |
| | | 160 | 6.7 | 794 | -0.8 |
| | | 165 | 10.0 | 727 | -9.1 |
| | | 155 | 3.3 | 705 | -11.9 |
| | | 161 | 7.3 | 765 | -4.4 |
| | | 153 | 2.0 | 759 | -5.1 |
| Mean | | 158 | | 758 | |
| SD | | 4.46 | | 36.9 | |
| %CV | | 2.8 | | 4.9 | |
| %Bias | | 5.3 | | -5.3 | |
| n | | 6 | | 6 | |

Sample dilution as follows:

2x = 50 mcL of sample+ 50 mcL of blank treated plasma
5x = 20 mcL of sample+ 80 mcL of blank treated plasma

Criteria: To be deemed acceptable, the mean %Bias at each level must be within ± 15.0% with %CV ≤ 15.0%.
Results: The acceptance criteria were met.

## Table 21: Freeze-Thaw Stability, (Frozen At -20 °C and -70 °C Thawed in Wet Ice) - Treated

Treated QCs without comed[2]

| Run Date | Curve Number | F/T -20/RT QC 0.6 0.600 µg/mL | %Bias | F/T -70/RT QC 0.6 0.600 µg/mL | %Bias | F/T -20/RT QC 150 150 µg/mL | %Bias | F/T -70/RT QC 150 150 µg/mL | %Bias |
|---|---|---|---|---|---|---|---|---|---|
| 09-Nov-2022 | 9 | 0.612 | 2.0 | ##0.790 | 31.7 | 141 | -6.0 | 167 | 11.3 |
| | | 0.581 | -3.2 | ##0.770 | 28.3 | 167 | 11.3 | 143 | -4.7 |
| | | 0.604 | 0.7 | 0.577 | -3.8 | 156 | 4.0 | ##180 | 20.0 |
| | | 0.607 | 1.2 | 0.678 | 13.0 | 159 | 6.0 | 157 | 4.7 |
| | | 0.532 | -11.3 | 0.648 | 8.0 | 153 | 2.0 | 156 | 4.0 |
| | | 0.544 | -9.3 | 0.612 | 2.0 | 157 | 4.7 | 170 | 13.3 |
| Mean | | 0.580 | | 0.679 | | 156 | | 162 | |
| SD | | 0.0344 | | 0.0854 | | 8.53 | | 12.9 | |
| %CV | | 5.9 | | 12.6 | | 5.5 | | 8.0 | |
| %Bias | | -3.3 | | 13.2 | | 4.0 | | 8.0 | |
| n | | 6 | | 6 | | 6 | | 6 | |
| ## > 15%Bias | | | | | | | | | |

Treated QCs with comed[2]

| Run Date | Curve Number | Comed F/T -20 QC 0.6 0.600 µg/mL | %Bias | Comed F/T -70 QC 0.6 0.600 µg/mL | %Bias | Comed F/T -20 QC150 150 µg/mL | %Bias | Comed F/T -70 QC150 150 µg/mL | %Bias |
|---|---|---|---|---|---|---|---|---|---|
| 09-Nov-2022 | 9 | 0.632 | 5.3 | ##0.756 | 26.0 | 137 | -8.7 | 169 | 12.7 |
| | | 0.680 | 13.3 | 0.632 | 5.3 | 167 | 11.3 | 161 | 7.3 |
| | | 0.641 | 6.8 | ##0.769 | 28.2 | 143 | -4.7 | 159 | 6.0 |
| | | 0.624 | 4.0 | 0.641 | 6.8 | 142 | -5.3 | 164 | 9.3 |
| | | 0.639 | 6.5 | 0.629 | 4.8 | 144 | -4.0 | 159 | 6.0 |
| | | 0.622 | 3.7 | 0.667 | 11.2 | 156 | 4.0 | 150 | 0.0 |
| Mean | | 0.640 | | 0.682 | | 148 | | 160 | |
| SD | | 0.0212 | | 0.0637 | | 11.2 | | 6.31 | |
| %CV | | 3.3 | | 9.3 | | 7.6 | | 3.9 | |
| %Bias | | 6.7 | | 13.7 | | -1.3 | | 6.7 | |
| n | | 6 | | 6 | | 6 | | 6 | |
| ## > 15%Bias | | | | | | | | | |

Criteria: To be deemed stable, the mean %Bias at each level must be within ± 15.0% with %CV ≤ 15.0%.

Results: Ertapenem is stable in treated human plasma without the presence of comed[2], with sodium heparin as anticoagulant, for at least 4 cycles when frozen at -20 °C and -70 °C and thawed in wet ice.

## Table 22: Freeze-Thaw Stability, (Frozen At -70 °C Thawed in Wet Ice) - Untreated

Untreated QCs without comed[2]

| Run Date | Curve Number | Untr.Ert.onlyFT low 0.600 µg/mL | %Bias | Untr.Ert.onlyFT high 150 µg/mL | %Bias |
|---|---|---|---|---|---|
| 07-Feb-2023 | 23 | 0.591 | -1.5 | 148 | -1.3 |
| | | 0.608 | 1.3 | 148 | -1.3 |
| | | 0.607 | 1.2 | 148 | -1.3 |
| | | 0.590 | -1.7 | 147 | -2.0 |
| | | 0.603 | 0.5 | 147 | -2.0 |
| | | 0.606 | 1.0 | 147 | -2.0 |
| Mean | | 0.601 | | 148 | |
| SD | | 0.00818 | | 0.548 | |
| %CV | | 1.4 | | 0.4 | |
| %Bias | | 0.2 | | -1.3 | |
| n | | 6 | | 6 | |

Untreated QCs with comed[2]

| Run Date | Curve Number | Untr. F/T -70 low 0.600 µg/mL | %Bias | Untr. F/T -70 high 150 µg/mL | %Bias |
|---|---|---|---|---|---|
| 20-Dec-2022 | 15 | 0.594 | -1.0 | 156 | 4.0 |
| | | 0.596 | -0.7 | 158 | 5.3 |
| | | 0.591 | -1.5 | 149 | -0.7 |
| | | 0.576 | -4.0 | 157 | 4.7 |
| | | 0.609 | 1.5 | 153 | 2.0 |
| | | 0.623 | 3.8 | 154 | 2.7 |
| Mean | | 0.598 | | 155 | |
| SD | | 0.0161 | | 3.27 | |
| %CV | | 2.7 | | 2.1 | |
| %Bias | | -0.3 | | 3.3 | |
| n | | 6 | | 6 | |

Criteria: To be deemed stable, the mean %Bias at each level must be within ± 15.0% with %CV ≤ 15.0%.

Results: Ertapenem is stable in untreated human plasma without the presence of comed[2], with sodium heparin as anticoagulant, for at least 2 cycles when frozen at -70 °C and thawed in wet ice.

## Table 23: Bench-Top Stability in Wet Ice - Treated

Treated QCs without comed[2]

| Run Date | Curve Number | BT 8.75 hr QC 0.6 0.600 µg/mL | %Bias | BT 8.75 hr QC 150 150 µg/mL | %Bias |
|---|---|---|---|---|---|
| 09-Nov-2022 | 9 | 0.684 | 14.0 | ##126 | -16.0 |
| | | 0.640 | 6.7 | 154 | 2.7 |
| | | 0.614 | 2.3 | ##179 | 19.3 |
| | | 0.570 | -5.0 | 164 | 9.3 |
| | | ##0.780 | 30.0 | 158 | 5.3 |
| | | 0.655 | 9.2 | 172 | 14.7 |
| Mean | | 0.657 | | 159 | |
| SD | | 0.0715 | | 18.5 | |
| %CV | | 10.9 | | 11.6 | |
| %Bias | | 9.5 | | 6.0 | |
| n | | 6 | | 6 | |
| ## > 15%Bias | | | | | |

Treated QCs with comed[2]

| Run Date | Curve Number | ComedBT 8.75h QC 0.6 0.600 µg/mL | %Bias | ComedBT 8.75h QC150 150 µg/mL | %Bias |
|---|---|---|---|---|---|
| 09-Nov-2022 | 9 | 0.594 | -1.0 | 153 | 2.0 |
| | | 0.608 | 1.3 | 155 | 3.3 |
| | | 0.587 | -2.2 | 161 | 7.3 |
| | | 0.605 | 0.8 | ##175 | 16.7 |
| | | 0.658 | 9.7 | 138 | -8.0 |
| | | 0.622 | 3.7 | 168 | 12.0 |
| Mean | | 0.612 | | 158 | |
| SD | | 0.0254 | | 12.9 | |
| %CV | | 4.2 | | 8.2 | |
| %Bias | | 2.0 | | 5.3 | |
| n | | 6 | | 6 | |
| ## > 15%Bias | | | | | |

Criteria: To be deemed stable, the mean %Bias at each level must be within ± 15.0% with %CV ≤ 15.0%.

Results: Ertapenem is stable in treated human plasma without the presence of comed[2], with sodium heparin as anticoagulant, for at least 8.75 hours in wet ice.

## Table 24: Bench-Top Stability in Wet Ice - Untreated

Untreated QCs with comed[2]

| Run Date | Curve Number | Untr. BT 4hr low 0.600 µg/mL | %Bias | Untr. BT 4hr high 150 µg/mL | %Bias |
|---|---|---|---|---|---|
| 20-Dec-2022 | 15 | #0.702 | 17.0 | 149 | -0.7 |
| | | 0.584 | -2.7 | 155 | 3.3 |
| | | 0.555 | -7.5 | 155 | 3.3 |
| | | 0.572 | -4.7 | 153 | 2.0 |
| | | 0.584 | -2.7 | 150 | 0.0 |
| | | 0.573 | -4.5 | 155 | 3.3 |
| Mean | | 0.595 | | 153 | |
| SD | | 0.0535 | | 2.71 | |
| %CV | | 9.0 | | 1.8 | |
| %Bias | | -0.8 | | 2.0 | |
| n | | 6 | | 6 | |
| # > 15%Bias | | | | | |

Untreated QCs without comed[2]

| Run Date | Curve Number | Untr.Ert.onlyBT low 0.600 µg/mL | %Bias | Untr.Ert.onlyBT high 150 µg/mL | %Bias |
|---|---|---|---|---|---|
| 07-Feb-2023 | 23 | 0.594 | -1.0 | 148 | -1.3 |
| | | 0.595 | -0.8 | 149 | -0.7 |
| | | 0.594 | -1.0 | 149 | -0.7 |
| | | 0.614 | 2.3 | 148 | -1.3 |
| | | 0.569 | -5.2 | 145 | -3.3 |
| | | 0.608 | 1.3 | 144 | -4.0 |
| Mean | | 0.596 | | 147 | |
| SD | | 0.0155 | | 2.14 | |
| %CV | | 2.6 | | 1.5 | |
| %Bias | | -0.7 | | -2.0 | |
| n | | 6 | | 6 | |

Criteria: To be deemed stable, the mean %Bias at each level must be within ± 15.0% with %CV ≤ 15.0%.

Results: Ertapenem is stable in untreated human plasma with the presence of comed[2], with sodium heparin as anticoagulant, for at least 4 hours in wet ice.

## Table 25: Long-Term Stability at -20 °C and -70 °C - Treated

Treated QCs with comed[2]

| Run Date | Curve Number | Comed1 LTS -20 low 0.600 µg/mL | %Bias | Comed1 LTS -70 low 0.600 µg/mL | %Bias | Comed1 LTS -20 high 150 µg/mL | %Bias | Comed1 LTS -70 high 150 µg/mL | %Bias |
|---|---|---|---|---|---|---|---|---|---|
| 07-Feb-2023 | 23 | | | #0.757 | 26.2 | | | #175 | 16.7 |
| (32 days) | | | | #0.756 | 26.0 | | | #174 | 16.0 |
| | | | | #0.747 | 24.5 | | | #174 | 16.0 |
| | | | | #0.767 | 27.8 | | | #173 | 15.3 |
| | | | | #0.751 | 25.2 | | | 172 | 14.7 |
| | | | | #0.764 | 27.3 | | | #173 | 15.3 |
| Intrarun Mean | | | | 0.757 | | | | 174 | |
| Intrarun SD | | | | 0.00756 | | | | 1.05 | |
| Intrarun %CV | | | | 1.0 | | | | 0.6 | |
| Intrarun %Bias | | | | 26.2 | | | | 16.0 | |
| n | | | | 6 | | | | 6 | |
| 13-Feb-2023 | 24 | #0.369 | -38.5 | 0.667 | 11.2 | #92.9 | -38.1 | 163 | 8.7 |
| (38 days) | | #0.371 | -38.2 | 0.646 | 7.7 | #93.8 | -37.5 | 165 | 10.0 |
| | | #0.373 | -37.8 | 0.682 | 13.7 | #94.0 | -37.3 | 165 | 10.0 |
| | | #0.376 | -37.3 | 0.667 | 11.2 | #94.0 | -37.3 | 167 | 11.3 |
| | | #0.382 | -36.3 | #0.697 | 16.2 | #94.1 | -37.3 | 167 | 11.3 |
| | | #0.368 | -38.7 | #0.705 | 17.5 | #93.4 | -37.7 | 171 | 14.0 |
| Intrarun Mean | | 0.373 | | 0.677 | | 93.7 | | 166 | |
| Intrarun SD | | 0.00519 | | 0.0218 | | 0.465 | | 2.73 | |
| Intrarun %CV | | 1.4 | | 3.2 | | 0.5 | | 1.6 | |
| Intrarun %Bias | | -37.8 | | 12.8 | | -37.5 | | 10.7 | |
| n | | 6 | | 6 | | 6 | | 6 | |
| # > 15%Bias | | | | | | | | | |

Treated QCs without comed[2]

| Run Date | Curve Number | LTS -20 low 0.600 µg/mL | %Bias | LTS -70 low 0.600 µg/mL | %Bias | LTS -20 high 150 µg/mL | %Bias | LTS -70 high 150 µg/mL | %Bias |
|---|---|---|---|---|---|---|---|---|---|
| 09-Jan-2023 | 18 | | | 0.623 | 3.8 | | | 150 | 0.0 |
| (24 days) | | | | 0.627 | 4.5 | | | 149 | -0.7 |
| | | | | 0.619 | 3.2 | | | 153 | 2.0 |
| | | | | 0.630 | 5.0 | | | 150 | 0.0 |
| | | | | 0.603 | 0.5 | | | 152 | 1.3 |
| | | | | 0.615 | 2.5 | | | 149 | -0.7 |
| Intrarun Mean | | | | 0.620 | | | | 151 | |
| Intrarun SD | | | | 0.00971 | | | | 1.64 | |
| Intrarun %CV | | | | 1.6 | | | | 1.1 | |
| Intrarun %Bias | | | | 3.3 | | | | 0.7 | |
| n | | | | 6 | | | | 6 | |
| # > 15%Bias | | | | | | | | | |

Criteria: To be deemed stable, the mean %Bias at each level must be within ± 15% with %CV ≤ 15%.

Results: Ertapenem is stable in treated human plasma with comed[2], with sodium heparin as anticoagulant, for up to 38 days upon storage in polypropylene tube at -70 °C.
Ertapenem is unstable in treated human plasma with comed[2], with sodium heparin as anticoagulant at -20 °C.
Ertapenem is stable in treated human plasma without comed[2], with sodium heparin as anticoagulant, for up to 24 days upon storage in polypropylene tube at -70 °C.
Ertapenem is stable in treated human plasma without comed[2], with sodium heparin as anticoagulant, at -20 °C.

## Table 26: Long-Term Stability at -70 °C - Untreated

Untreated with comed[2]

| Run Date | Curve Number | Untr. LTS -70 low 0.600 µg/mL | %Bias | Untr. LTS -70 high 150 µg/mL | %Bias |
|---|---|---|---|---|---|
| 13-Jan-2023 | 19 | 0.534 | -11.0 | 137 | -8.7 |
| (4 days) | | 0.581 | -3.2 | 140 | -6.7 |
| | | 0.557 | -7.2 | 143 | -4.7 |
| | | 0.574 | -4.3 | 140 | -6.7 |
| | | 0.546 | -9.0 | 139 | -7.3 |
| | | 0.582 | -3.0 | 137 | -8.7 |
| Intrarun Mean | | 0.562 | | 139 | |
| Intrarun SD | | 0.0198 | | 2.25 | |
| Intrarun %CV | | 3.5 | | 1.6 | |
| Intrarun %Bias | | -6.3 | | -7.3 | |
| n | | 6 | | 6 | |
| 16-Jan-2023 | 20 | 0.580 | -3.3 | 135 | -10.0 |
| (7 days) | | 0.588 | -2.0 | 139 | -7.3 |
| | | 0.563 | -6.2 | 139 | -7.3 |
| | | 0.591 | -1.5 | 139 | -7.3 |
| | | 0.586 | -2.3 | 139 | -7.3 |
| | | 0.585 | -2.5 | 138 | -8.0 |
| Intrarun Mean | | 0.582 | | 138 | |
| Intrarun SD | | 0.0101 | | 1.60 | |
| Intrarun %CV | | 1.7 | | 1.2 | |
| Intrarun %Bias | | -3.0 | | -8.0 | |
| n | | 6 | | 6 | |
| # > 15%Bias | | | | | |

## Table 26: Long-Term Stability at -70 °C – Untreated (Continued)

Untreated QCs with comed[2]

| Run Date | Curve Number | Combo Untr LTS Low 0.600 µg/mL | %Bias | Untr Comed LTS low 0.600 µg/mL | %Bias | Combo Untr LTS High 150 µg/mL | %Bias | Untr Comed LTS high 150 µg/mL | %Bias |
|---|---|---|---|---|---|---|---|---|---|
| 24-Jan-2023 | 21 | 0.640 | 6.7 | | | 147 | -2.0 | | |
| (14 days) | | 0.628 | 4.7 | | | 147 | -2.0 | | |
| | | 0.612 | 2.0 | | | 152 | 1.3 | | |
| | | 0.572 | -4.7 | | | 152 | 1.3 | | |
| | | 0.613 | 2.2 | | | 150 | 0.0 | | |
| | | 0.593 | -1.2 | | | 149 | -0.7 | | |
| Intrarun Mean | | 0.610 | | | | 150 | | | |
| Intrarun SD | | 0.0244 | | | | 2.26 | | | |
| Intrarun %CV | | 4.0 | | | | 1.5 | | | |
| Intrarun %Bias | | 1.7 | | | | 0.0 | | | |
| n | | 6 | | | | 6 | | | |
| 02-Feb-2023 | 22 | | | 0.564 | -6.0 | | | 144 | -4.0 |
| (24 days) | | | | 0.596 | -0.7 | | | 143 | -4.7 |
| | | | | 0.597 | -0.5 | | | 144 | -4.0 |
| | | | | 0.628 | 4.7 | | | 141 | -6.0 |
| | | | | 0.582 | -3.0 | | | 144 | -4.0 |
| | | | | 0.590 | -1.7 | | | 143 | -4.7 |
| Intrarun Mean | | | | 0.593 | | | | 143 | |
| Intrarun SD | | | | 0.0211 | | | | 1.17 | |
| Intrarun %CV | | | | 3.6 | | | | 0.8 | |
| Intrarun %Bias | | | | -1.2 | | | | -4.7 | |
| n | | | | 6 | | | | 6 | |
| 07-Feb-2023 | 23 | | | 0.669 | 11.5 | | | 156 | 4.0 |
| (29 days) | | | | 0.658 | 9.7 | | | 155 | 3.3 |
| | | | | #0.717 | 19.5 | | | 156 | 4.0 |
| | | | | 0.657 | 9.5 | | | 158 | 5.3 |
| | | | | 0.668 | 11.3 | | | 158 | 5.3 |
| | | | | 0.666 | 11.0 | | | 157 | 4.7 |
| Intrarun Mean | | | | 0.673 | | | | 157 | |
| Intrarun SD | | | | 0.0224 | | | | 1.21 | |
| Intrarun %CV | | | | 3.3 | | | | 0.8 | |
| Intrarun %Bias | | | | 12.2 | | | | 4.7 | |
| n | | | | 6 | | | | 6 | |
| # > 15%Bias | | | | | | | | | |

## Table 26: Long-Term Stability at -70 °C – Untreated (Continued)

Untreated QCs without comed[2]

| Run Date | Curve Number | Untr LTS low 0.600 µg/mL | %Bias | Untr LTS high 150 µg/mL | %Bias |
|---|---|---|---|---|---|
| 02-Feb-2023 | 22 | 0.547 | -8.8 | 135 | -10.0 |
| (10 days) | | 0.527 | -12.2 | 134 | -10.7 |
| | | 0.532 | -11.3 | 136 | -9.3 |
| | | 0.539 | -10.2 | 138 | -8.0 |
| | | 0.516 | -14.0 | 135 | -10.0 |
| | | 0.528 | -12.0 | 133 | -11.3 |
| Intrarun Mean | | 0.532 | | 135 | |
| Intrarun SD | | 0.0107 | | 1.72 | |
| Intrarun %CV | | 2.0 | | 1.3 | |
| Intrarun %Bias | | -11.3 | | -10.0 | |
| n | | 6 | | 6 | |
| 07-Feb-2023 | 23 | 0.613 | 2.2 | 148 | -1.3 |
| (15 days) | | 0.606 | 1.0 | 149 | -0.7 |
| | | 0.596 | -0.7 | 145 | -3.3 |
| | | 0.605 | 0.8 | 147 | -2.0 |
| | | 0.584 | -2.7 | 145 | -3.3 |
| | | 0.586 | -2.3 | 148 | -1.3 |
| Intrarun Mean | | 0.598 | | 147 | |
| Intrarun SD | | 0.0117 | | 1.67 | |
| Intrarun %CV | | 2.0 | | 1.1 | |
| Intrarun %Bias | | -0.3 | | -2.0 | |
| n | | 6 | | 6 | |
| 15-Feb-2023 | 25 | 0.607 | 1.2 | 153 | 2.0 |
| (23 days) | | 0.625 | 4.2 | 154 | 2.7 |
| | | 0.600 | 0.0 | 152 | 1.3 |
| | | 0.632 | 5.3 | 150 | 0.0 |
| | | 0.625 | 4.2 | 151 | 0.7 |
| | | 0.612 | 2.0 | 148 | -1.3 |
| Intrarun Mean | | 0.617 | | 151 | |
| Intrarun SD | | 0.0124 | | 2.16 | |
| Intrarun %CV | | 2.0 | | 1.4 | |
| Intrarun %Bias | | 2.8 | | 0.7 | |
| n | | 6 | | 6 | |
| 20-Feb-2023 | 26 | 0.605 | 0.8 | 143 | -4.7 |
| (28 days) | | 0.586 | -2.3 | 146 | -2.7 |
| | | 0.642 | 7.0 | 142 | -5.3 |
| | | 0.576 | -4.0 | 147 | -2.0 |
| | | 0.596 | -0.7 | 144 | -4.0 |
| | | 0.539 | -10.2 | 141 | -6.0 |
| Intrarun Mean | | 0.591 | | 144 | |
| Intrarun SD | | 0.0340 | | 2.32 | |
| Intrarun %CV | | 5.8 | | 1.6 | |
| Intrarun %Bias | | -1.5 | | -4.0 | |
| n | | 6 | | 6 | |

Criteria: To be deemed stable, the mean %Bias at each level must be within ± 15% with %CV ≤ 15%.

Results: Ertapenem is stable in untreated human plasma with comed[2], with sodium heparin as anticoagulant, for up to 29 days upon storage in polypropylene tube at -70 °C.

Ertapenem is stable in untreated human plasma without comed[2], with sodium heparin as anticoagulant, for up to 28 days upon storage in polypropylene tube at -70 °C. There was failure in run 17 and the QC pools were reprepared and long term stability duration restarted.

## Table 27: Stability Evaluation During Blood Collection and Processing

Run: 8

Evaluation of Ertapenem Stability During Blood Collection and Processing in Ice Water Bath, Centrifuged at Room Temperature

| **Mid QCS, 100 mcg/mL** | | | | | | | | | |
|---|---|---|---|---|---|---|---|---|---|
| Stability Data: 60 minutes Ice Water Bath | | | | | Comparison Data: 0 minutes | | | | |
| Sample ID | Seq # | Drug Peak Area | IS Peak Area | Peak Area Ratio | Sample ID | Seq # | Drug Peak Area | IS Peak Area | Peak Area Ratio |
| Mid-RT 60 Min | 4 | 3091442 | 687558 | 4.4963 | Mid-RT 0 Hr | 3 | 2963859 | 668367 | 4.4345 |
| Mid-RT 60 Min | 8 | 3135744 | 668395 | 4.6915 | Mid-RT 0 Hr | 7 | 2877301 | 686253 | 4.1928 |
| Mid-RT 60 Min | 12 | 2991805 | 718708 | 4.1628 | Mid-RT 0 Hr | 11 | 2961991 | 715097 | 4.1421 |
| Mid-RT 60 Min | 16 | 3026589 | 697066 | 4.3419 | Mid-RT 0 Hr | 15 | 3414573 | 651598 | 5.2403 |
| | | | Mean | 4.4231 | | | | Mean | 4.5024 |
| | | | SD | 0.2249 | | | | SD | 0.5082 |
| | | | %CV | 5.1 | | | | %CV | 11.3 |
| | | | **% Change** | **-1.8** | | | | | |

Evaluation of Ertapenem Stability During Blood Collection and Processing at Room Temperature, Centrifuged at Room Temperature

| **Mid QCS, 100 mcg/mL** | | | | | | | | | |
|---|---|---|---|---|---|---|---|---|---|
| Stability Data: 60 minutes Room Temperature | | | | | Comparison Data: 0 minutes | | | | |
| Sample ID | Seq # | Drug Peak Area | IS Peak Area | Peak Area Ratio | Sample ID | Seq # | Drug Peak Area | IS Peak Area | Peak Area Ratio |
| Mid-IceWater 60 Min | 6 | 2931832 | 651309 | 4.5014 | Mid-IceWater 0 Hr | 5 | 3425396 | 700756 | 4.8881 |
| Mid-IceWater 60 Min | 10 | 2959579 | 685028 | 4.3204 | Mid-IceWater 0 Hr | 9 | 2919931 | 700911 | 4.1659 |
| Mid-IceWater 60 Min | 14 | 3010154 | 679587 | 4.4294 | Mid-IceWater 0 Hr | 13 | 3104319 | 700948 | 4.4287 |
| Mid-IceWater 60 Min | 18 | 2904449 | 697709 | 4.1628 | Mid-IceWater 0 Hr | 17 | 3051669 | 670694 | 4.5500 |
| | | | Mean | 4.3535 | | | | Mean | 4.5082 |
| | | | SD | 0.1473 | | | | SD | 0.2998 |
| | | | %CV | 3.4 | | | | %CV | 6.6 |
| | | | **% Change** | **-3.4** | | | | | |

$$\%\ \text{Change} = \frac{(\text{Peak Area Ratio Stored} - \text{Peak Area Ratio Time 0})}{\text{Peak Area Ratio Time 0}} \times 100$$

Criteria: To be considered stable, the % change between 60 minute sample pools and the control 0-hr sample pools must be within ± 15.0%, with %CV ≤ 15.0%.

Results: Ertapenem appears to be stable during the blood collection process at room temperature or in an ice-water bath for at least 60 minutes in polyethylene terephthalate containers.

## Table 28: Processed Sample Stability at Room Temperature

| Run Date | Curve Number | Processed RT low 0.600 µg/mL | %Bias | Processed RT high 150 µg/mL | %Bias |
|---|---|---|---|---|---|
| 07-Nov-2022 | 5 | #0.497 | -17.2 | 163 | 8.7 |
| | | 0.600 | 0.0 | 153 | 2.0 |
| | | #0.509 | -15.2 | 156 | 4.0 |
| | | 0.660 | 10.0 | 133 | -11.3 |
| | | 0.587 | -2.2 | 136 | -9.3 |
| | | 0.540 | -10.0 | 138 | -8.0 |
| Mean | | 0.566 | | 147 | |
| SD | | 0.0618 | | 12.4 | |
| %CV | | 10.9 | | 8.4 | |
| %Bias | | -5.7 | | -2.0 | |
| n | | 6 | | 6 | |
| # > 15%Bias | | | | | |

Samples were processed with run 1 then stored for 3 days 20 hours in polypropylene 96 well plates at ambient conditions prior to injection with run 5.

Criteria: To be deemed stable, the mean %Bias at each level must be within ± 15.0% with %CV ≤ 15.0%.

Results: The acceptance criteria were met.

## Table 29: Refrigerated Processed Sample Stability

| Run Date | Curve Number | Processed Refr low 0.600 µg/mL | %Bias | Processed Refr high 150 µg/mL | %Bias |
|---|---|---|---|---|---|
| 07-Nov-2022 | 5 | #0.495 | -17.5 | 129 | -14.0 |
| | | 0.578 | -3.7 | 146 | -2.7 |
| | | #0.497 | -17.2 | 152 | 1.3 |
| | | 0.554 | -7.7 | 156 | 4.0 |
| | | 0.536 | -10.7 | 130 | -13.3 |
| | | #0.718 | 19.7 | 137 | -8.7 |
| Mean | | 0.563 | | 142 | |
| SD | | 0.0825 | | 11.4 | |
| %CV | | 14.7 | | 8.0 | |
| %Bias | | -6.2 | | -5.3 | |
| n | | 6 | | 6 | |
| # > 15%Bias | | | | | |

Samples were processed with run 1, and then stored for 3 days 20 hours in polypropylene 96 well plates at refrigerated temperatures prior to injection with run 5.

Criteria: To be deemed stable, the mean %Bias at each level must be within ± 15.0% with %CV ≤ 15.0%.

Results: The acceptance criteria were met.

## Table 30: Individual Sample Reinjection Reproducibility

| Run Date | Curve Number | Indiv Reinj low 0.600 µg/mL | %Bias | Indiv Reinj high 150 µg/mL | %Bias |
|---|---|---|---|---|---|
| 03-Nov-2022 | 1 | 0.621 | 3.5 | 157 | 4.7 |
| | | 0.585 | -2.5 | 153 | 2.0 |
| | | 0.583 | -2.8 | 166 | 10.7 |
| | | 0.639 | 6.5 | 144 | -4.0 |
| | | 0.625 | 4.2 | 150 | 0.0 |
| | | 0.623 | 3.8 | 149 | -0.7 |
| Mean | | 0.613 | | 153 | |
| SD | | 0.0231 | | 7.63 | |
| %CV | | 3.8 | | 5.0 | |
| %Bias | | 2.2 | | 2.0 | |
| n | | 6 | | 6 | |

Low and high QC extracts (6 each) were injected during the run and then reinjected at the end of the same run.
Criteria: To be deemed reproducible, the mean %Bias at each level must be within ± 15.0% with %CV ≤ 15.0%.
Results: The acceptance criteria were met.

## Table 31: Whole Run Reinjection Reproducibility and Column Ruggedness

Analytical Performance: Back-Calculated Concentrations (µg/mL) of Ertapenem Calibration Standard in (Human) (Plasma) in (Study V1872106P5)

| Assay Date | Analytical Run Number | Cal 0.200 0.200 µg/mL | Cal 0.400 0.400 µg/mL | Cal 4.00 4.00 µg/mL | Cal 20.0 20.0 µg/mL | Cal 40.0 40.0 µg/mL | Cal 80.0 80.0 µg/mL | Cal 160 160 µg/mL | Cal 200 200 µg/mL |
|---|---|---|---|---|---|---|---|---|---|
| 07-Nov-2022 | 6 | 0.204 | 0.391 | 3.79 | 21.1 | 45.2 | 72.3 | 166 | 186 |
| | | 0.193 | 0.427 | 3.66 | 19.8 | 40.8 | 86.3 | 149 | 206 |
| Mean | | 0.199 | 0.409 | 3.73 | 20.5 | 43.0 | 79.3 | 158 | 196 |
| %Bias | | -0.5 | 2.3 | -6.8 | 2.5 | 7.5 | -0.9 | -1.3 | -2.0 |

Calibration Curve Parameters for Ertapenem Calibration Standards in Human Plasma (Study V1872106P5)

| Run Date | Curve Number | Slope | Intercept | R-Squared | LLOQ | ULOQ |
|---|---|---|---|---|---|---|
| 07-Nov-2022 | 6 | 0.05404 | 0.00020 | 0.9946 | 0.200 | 200 |

Analytical Performance of Ertapenem Quality Control Samples in Human Plasma (Study V1872106P5)

| Run Date | Curve Number | QCS 0.200 0.200 µg/mL | %Bias | QCS 0.600 0.600 µg/mL | %Bias | QCS 100 100 µg/mL | %Bias | QCS 150 150 µg/mL | %Bias |
|---|---|---|---|---|---|---|---|---|---|
| 07-Nov-2022 | 6 | 0.179 | -10.5 | 0.668 | 11.3 | 97.4 | -2.6 | 169 | 12.7 |
| | | 0.168 | -16.0 | #0.489 | -18.5 | 105 | 5.0 | 147 | -2.0 |
| | | 0.188 | -6.0 | 0.590 | -1.7 | 99.1 | -0.9 | 171 | 14.0 |
| | | 0.165 | -17.5 | #0.698 | 16.3 | 101 | 1.0 | 143 | -4.7 |
| | | 0.181 | -9.5 | 0.536 | -10.7 | 114 | 14.0 | 156 | 4.0 |
| | | 0.195 | -2.5 | 0.643 | 7.2 | #119 | 19.0 | #176 | 17.3 |
| Mean | | 0.179 | | 0.604 | | 106 | | 160 | |
| S.D. | | 0.0115 | | 0.0806 | | 8.72 | | 13.6 | |
| %CV | | 6.4 | | 13.3 | | 8.2 | | 8.5 | |
| %Bias | | -10.5 | | 0.7 | | 6.0 | | 6.7 | |
| n | | 6 | | 6 | | 6 | | 6 | |
| # > 15%Bias | | | | | | | | | |

Note: Run 6 is a reinjection of run 1 on a different analytical column of the same manufacturer, dimensions, and type, using same MS platform (Applied Biosystems API6500) – reinjected within 4 days 4 hours

Criteria: At each level, the %CV was required to be ≤ 15.0% from the nominal value, except at the LLOQ level, where ≤ 20.0% was accepted. The mean %Bias at each level was also required to be within ± 15.0%, except at the LLOQ, where ± 20.0% was accepted.

Results: The acceptance criteria were met.

## Table 32: Extraction Recovery – Ertapenem

Run: 7

| **Control** | | | **Extracted QCs** | | |
|---|---|---|---|---|---|
| QC Conc. *mcg/mL* | | Drug Peak Area | QC Conc. *mcg/mL* | Drug Peak Area | Extraction Recovery % |
| Low QC | | | Low QC | | |
| 0.600 | | 23886 | 0.600 | 21688 | 101.4 |
| 0.600 | | 20603 | 0.600 | 23190 | 108.4 |
| 0.600 | | 19534 | 0.600 | 26006 | 121.6 |
| 0.600 | | 19289 | 0.600 | 22204 | 103.8 |
| 0.600 | | 22875 | 0.600 | 21021 | 98.3 |
| 0.600 | | 22168 | 0.600 | 23602 | 110.3 |
| | Average | 21393 | | Average | 107.3 |
| Mid QC | | | Mid QC | | |
| 100 | | 4094282 | 100 | 4059841 | 105.8 |
| 100 | | 3303795 | 100 | 4092040 | 106.7 |
| 100 | | 4295561 | 100 | 3705451 | 96.6 |
| 100 | | 3819508 | 100 | 4131147 | 107.7 |
| 100 | | 3586993 | 100 | 4050610 | 105.6 |
| 100 | | 3918128 | 100 | 3958688 | 103.2 |
| | Average | 3836378 | | Average | 104.3 |
| High QC | | | High QC | | |
| 150 | | 5610822 | 150 | 5822233 | 101.7 |
| 150 | | 6472913 | 150 | 5948900 | 103.9 |
| 150 | | 5670982 | 150 | 6449185 | 112.6 |
| 150 | | 5728639 | 150 | 6256417 | 109.3 |
| 150 | | 5556829 | 150 | 6457425 | 112.8 |
| 150 | | 5318170 | 150 | 5840248 | 102.0 |
| | Average | 5726393 | | Average | 107.0 |
| | | | | | ====== |
| | | | | n: | 18 |
| | | | | Overall Mean Recovery: | **106.2** |
| | | | | SD | **5.9** |
| | | | | Overall % CV: | **5.6** |

$$\%\ \text{Recovery} = \frac{\text{Response Ratio of "Extracted QC"}}{\text{Average Response Ratio of Control}} \times 100$$

## Table 33: Extraction Recovery – Ertapenem-$d_4$

Run: 2

| Control (PES; post extraction spike) | | | | Extracted QCs | | | | IS |
|---|---|---|---|---|---|---|---|---|
| Ertapenem QC Conc. *mcg/mL* | IS Peak Area | Ertapenem Peak Area | Peak Area Ratio | Ertapenem QC Conc. *mcg/mL* | IS Peak Area | Ertapenem Peak Area | Peak Area Ratio | Extraction Recovery % |
| Mid QC | | | | Mid QC | | | | |
| 100.00 | 939933 | 4398433 | 0.2137 | 100.00 | 792120 | 4649113 | 0.1704 | 76.6 |
| 100.00 | 1004218 | 4143573 | 0.2424 | 100.00 | 864951 | 5470428 | 0.1581 | 71.1 |
| 100.00 | 947568 | 4675820 | 0.2027 | 100.00 | 772782 | 4526745 | 0.1707 | 76.7 |
| 100.00 | 993650 | 4283105 | 0.2320 | 100.00 | 880916 | 4866753 | 0.1810 | 81.4 |
| 100.00 | 1007765 | 4822372 | 0.2090 | 100.00 | 832722 | 4141420 | 0.2011 | 90.4 |
| 100.00 | 1090859 | 4642748 | 0.2350 | 100.00 | 796409 | 4548134 | 0.1751 | 78.7 |
| | | Average | 0.2224 | | | | Average | 79.2 |
| | | | | | | | SD | 6.5 |
| | | | | | | | %CV | 8.2 |
| | | | | | | | | ====== |
| | | | | | | | n: | 6 |

$$\% \text{ Recovery} = \frac{\text{Response Ratio of "Extracted QC"}}{\text{Average Response Ratio of Control}} \times 100$$

## Table 34: Stability of Ertapenem Primary Solution in MES Buffer at Room Temperature

Run: 4
Container: Polypropylene

| Primary Solution - Solution Stability at Room Temperature | | | | | | | | | |
|---|---|---|---|---|---|---|---|---|---|
| Concentration: 8.00 mg/mL | | | | | | | | | |
| Stability Data (Room Temperature) | | | | | Comparison Data (4°C) | | | | |
| Lot #: | V1872106P5-14-1 | | | | Lot #: | V1872106P5-14-2 | | | |
| Prepared: | 3-Nov-2022 | | | | Prepared: | 3-Nov-2022 | | | |
| Sample ID | Seq # | Drug Peak Area | IS Peak Area | Peak Area Ratio | Sample ID | Seq # | Drug Peak Area | IS Peak Area | Peak Area Ratio |
| Primary BT-1 | 7 | 5620573 | 88122 | 63.7817 | Primary Refr-1 | 5 | 5960585 | 91974 | 64.8073 |
| Primary BT-1 | 13 | 6621884 | 95052 | 69.6659 | Primary Refr-1 | 11 | 5865724 | 92488 | 63.4215 |
| Primary BT-1 | 19 | 7062212 | 113045 | 62.4726 | Primary Refr-1 | 17 | 6074087 | 95377 | 63.6850 |
| Primary BT-2 | 8 | 5736744 | 88649 | 64.7130 | Primary Refr-2 | 6 | 4583100 | 70979 | 64.5698 |
| Primary BT-2 | 14 | 6152635 | 92769 | 66.3221 | Primary Refr-2 | 12 | 5209699 | 84342 | 61.7687 |
| Primary BT-2 | 20 | 7002169 | 106053 | 66.0252 | Primary Refr-2 | 18 | 7361633 | 107453 | 68.5103 |
| | | | Mean | 65.4968 | | | | Mean | 64.4604 |
| | | | n | 6 | | | | n | 6 |
| | | | SD | 2.4917 | | | | SD | 2.2565 |
| | | | %CV | 3.8 | | | | %CV | 3.5 |
| %Change = | 1.6 | | | | | | | | |
| Duration: | 6.75 | Hours | | | | | | | |

$$\%\text{Change} = \frac{\text{mean response of stored solutions} - \text{mean response of comparison solutions}}{\text{mean response of comparison solutions}} \times 100$$

Criteria: To be deemed stable, % Change upon storage for analyte must be within ±15.0% and %CV must be within ≤15.0%.
Meets Criteria: YES

## Table 35: Stability of Ertapenem Primary Solution in MES Buffer at -20 °C

Run: 11
Container: Polypropylene

| Primary Solution - Solution Stability at -20°C | | | | | | | | | |
|---|---|---|---|---|---|---|---|---|---|
| Concentration: 8.00 mg/mL | | | | | | | | | |
| Stability Data (Stored) | | | | | Comparison Data (Fresh) | | | | |
| Lot #: | V1872106P5-2-1 | | | | Lot #: | V1872106P5-43-1 | | | |
| Prepared: | 3-Nov-2022 | | | | Prepared: | 2-Dec-2022 | | | |
| Sample ID | Seq # | Drug Peak Area | IS Peak Area | Peak Area Ratio | Sample ID | Seq # | Drug Peak Area | IS Peak Area | Peak Area Ratio |
| 11 004 Primary stored-1 1 1 | 4 | 55516156 | 875137 | 63.4371 | 11 003 Primary fresh-1 1 1 | 3 | 67757503 | 873169 | 77.5995 |
| 11 008 Primary stored-1 2 1 | 8 | 52823174 | 847513 | 62.3273 | 11 007 Primary fresh-1 2 1 | 7 | 68112017 | 919844 | 74.0474 |
| 11 012 Primary stored-1 3 1 | 12 | 53341277 | 774119 | 68.9058 | 11 011 Primary fresh-1 3 1 | 11 | 72642640 | 887000 | 81.8970 |
| 11 006 Primary stored-2 1 1 | 6 | 52714862 | 803755 | 65.5857 | 11 005 Primary fresh-2 1 1 | 5 | 69731780 | 909591 | 76.6628 |
| 11 010 Primary stored-2 2 1 | 10 | 57904954 | 843854 | 68.6196 | 11 009 Primary fresh-2 2 1 | 9 | 69026745 | 849068 | 81.2971 |
| 11 014 Primary stored-2 3 1 | 14 | 54906358 | 827878 | 66.3218 | 11 013 Primary fresh-2 3 1 | 13 | 70678817 | 916186 | 77.1446 |
| | | | Mean | 65.8662 | | | | Mean | 78.1081 |
| | | | n | 6 | | | | n | 6 |
| | | | SD | 2.6658 | | | | SD | 2.9763 |
| | | | %CV | 4.0 | | | | %CV | 3.8 |
| %Change = | -15.7 | | | | | | | | |
| Duration: | 29 | Days | | | | | | | |

$$\%\text{Change} = \frac{\text{mean response of stored solutions} - \text{mean response of comparison solutions}}{\text{mean response of comparison solutions}} \times 100$$

Criteria: To be deemed stable, % Change upon storage for analyte must be within ±15.0% and %CV must be within ≤15.0%.
Meets Criteria: No

## Table 36: Stability of Ertapenem Primary Solution in MES Buffer at -20 °C

Run: 10
Container: Polypropylene

| Concentration: 8.00 mg/mL | | | | | | | | | |
|---|---|---|---|---|---|---|---|---|---|
| Stability Data (Stored -20°C) | | | | | Comparison Data (Fresh) | | | | |
| Lot #: | V1872106P5-2-1 | | | | Lot #: | V1872106P5-38-1 | | | |
| Prepared: | 3-Nov-2022 | | | | Prepared: | 10-Nov-2022 | | | |
| Sample ID | Seq # | Drug Peak Area | IS Peak Area | Peak Area Ratio | Sample ID | Seq # | Drug Peak Area | IS Peak Area | Peak Area Ratio |
| Primary stored-1 | 4 | 5817264 | 30968 | 187.8476 | Primary fresh-1 | 3 | 5482273 | 32017 | 171.2301 |
| Primary stored-1 | 8 | 5954724 | 31229 | 190.6793 | Primary fresh-1 | 7 | 6302709 | 32203 | 195.7181 |
| Primary stored-1 | 12 | 5658237 | 34334 | 164.7998 | Primary fresh-1 | 11 | 6405482 | 36041 | 177.7276 |
| Primary stored-2 | 6 | 5097485 | 30613 | 166.5137 | Primary fresh-2 | 5 | 6041908 | 32145 | 187.9579 |
| Primary stored-2 | 10 | 4919923 | 28241 | 174.2121 | Primary fresh-2 | 9 | 5971914 | 32011 | 186.5582 |
| Primary stored-2 | 14 | 5479678 | 31178 | 175.7546 | Primary fresh-2 | 13 | 6214568 | 31401 | 197.9099 |
| | | | Mean | 176.6345 | | | | Mean | 186.1836 |
| | | | n | 6 | | | | n | 6 |
| | | | SD | 10.6969 | | | | SD | 10.2646 |
| | | | %CV | 6.1 | | | | %CV | 5.5 |
| %Change = | -5.1 | | | | | | | | |
| Duration: | 7 | Days | | | | | | | |

$$\%\text{Change} = \frac{\text{mean response of stored solutions} - \text{mean response of comparison solutions}}{\text{mean response of comparison solutions}} \times 100$$

Criteria: To be deemed stable, % Change upon storage for analyte must be within ±15.0% and %CV must be within ≤15.0%.
Meets Criteria: YES

# 17. FIGURES

## Figure 1: Typical Calibration Curve

Analytical Run 1 Run Acceptance Parameters for Ertapenem (µg/mL) (Study V1872106P5)

| | |
|---|---|
| Regression Type | LINEAR |
| Weighting Factor | 1/X**2 |
| Slope | 0.052848469560109600 |
| Intercept | -0.000015493499713561 |
| R-Squared | 0.9977092651577 |
| LLOQ | 0.2 |
| ULOQ | 200 |
| Mean Std %Diff | 3.1 |
| S.D. Std %Diff | 2.9 |
| Mean Std Response | 0.052833 |
| S.D. Std Response | 0.002271 |

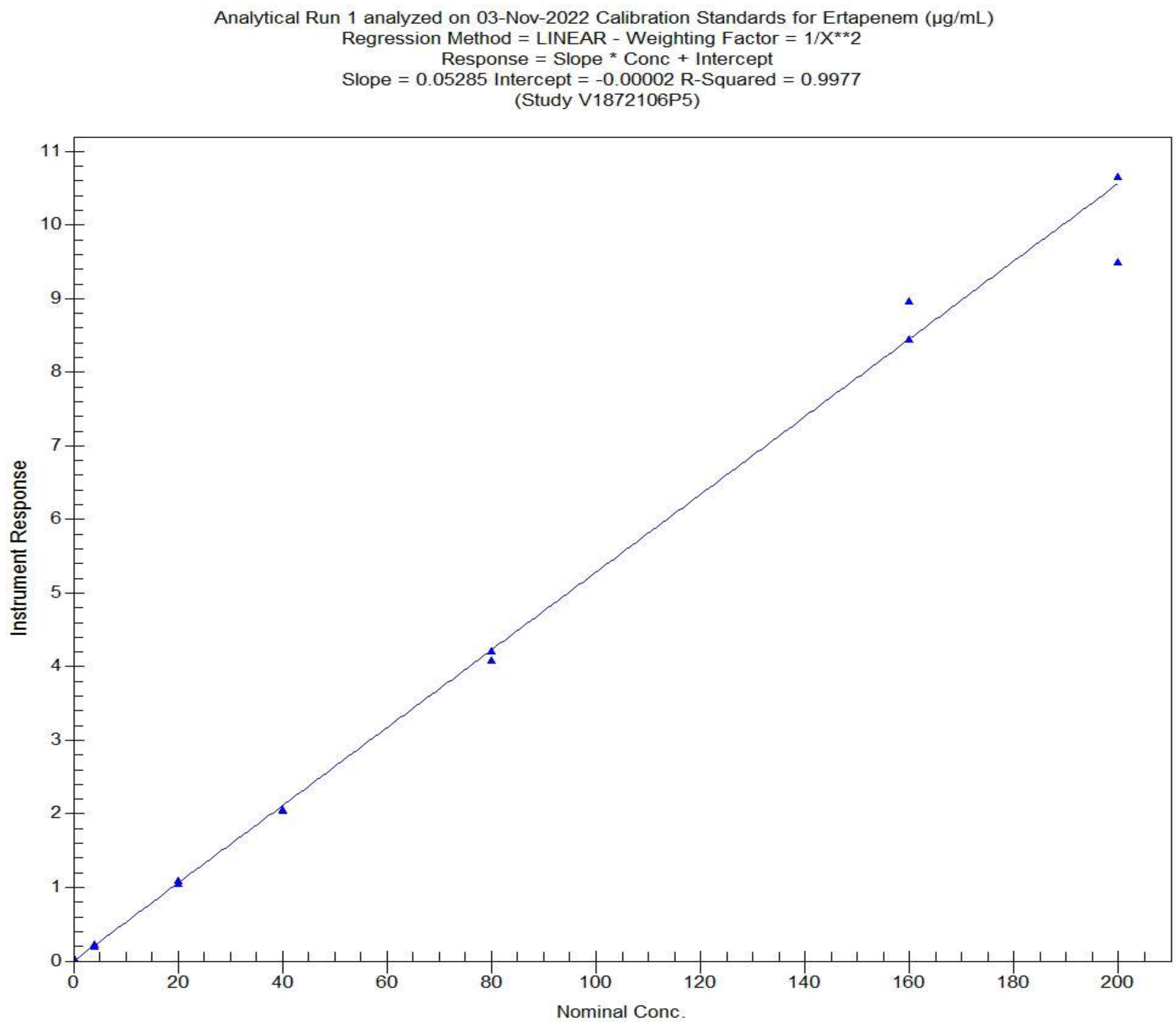

## Figure 2: Typical Ion Chromatogram Blank Human Plasma Without Internal Standard

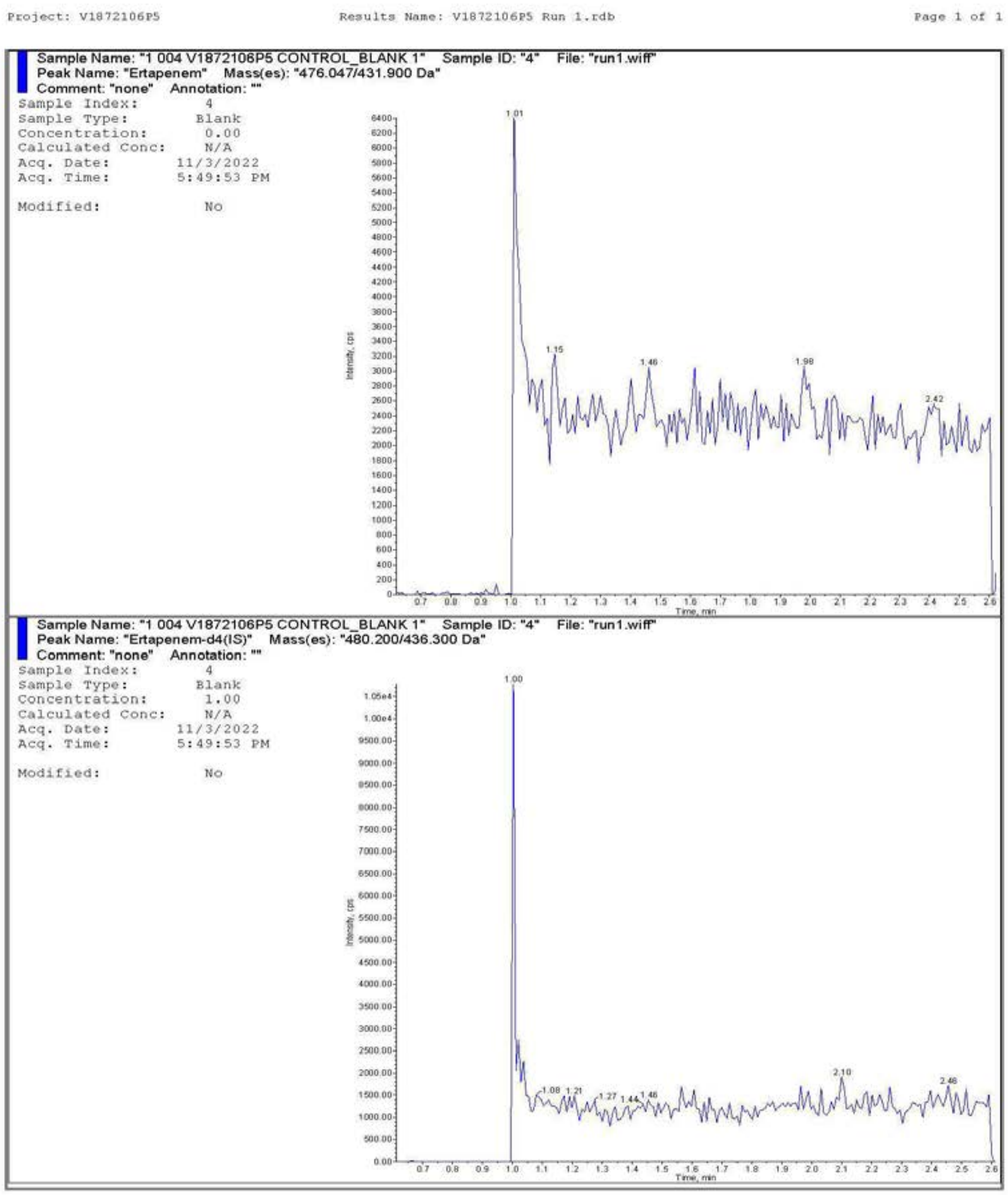

**Figure 3: Typical Ion Chromatogram Blank Human Plasma With Internal Standard**

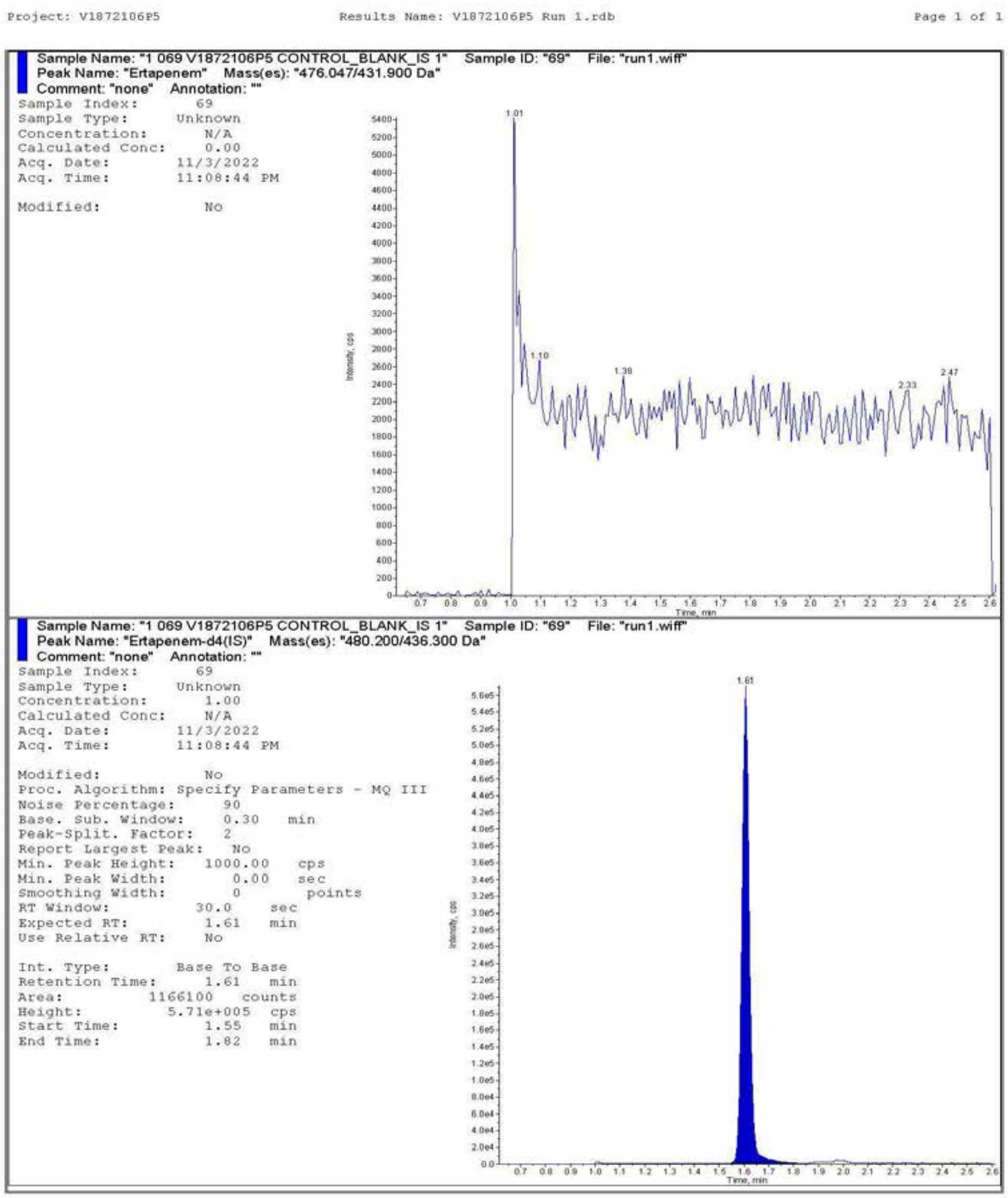

**Figure 4: Typical Ion Chromatogram LLOQ Calibration Standard, Ertapenem = 0.200 mcg/mL**

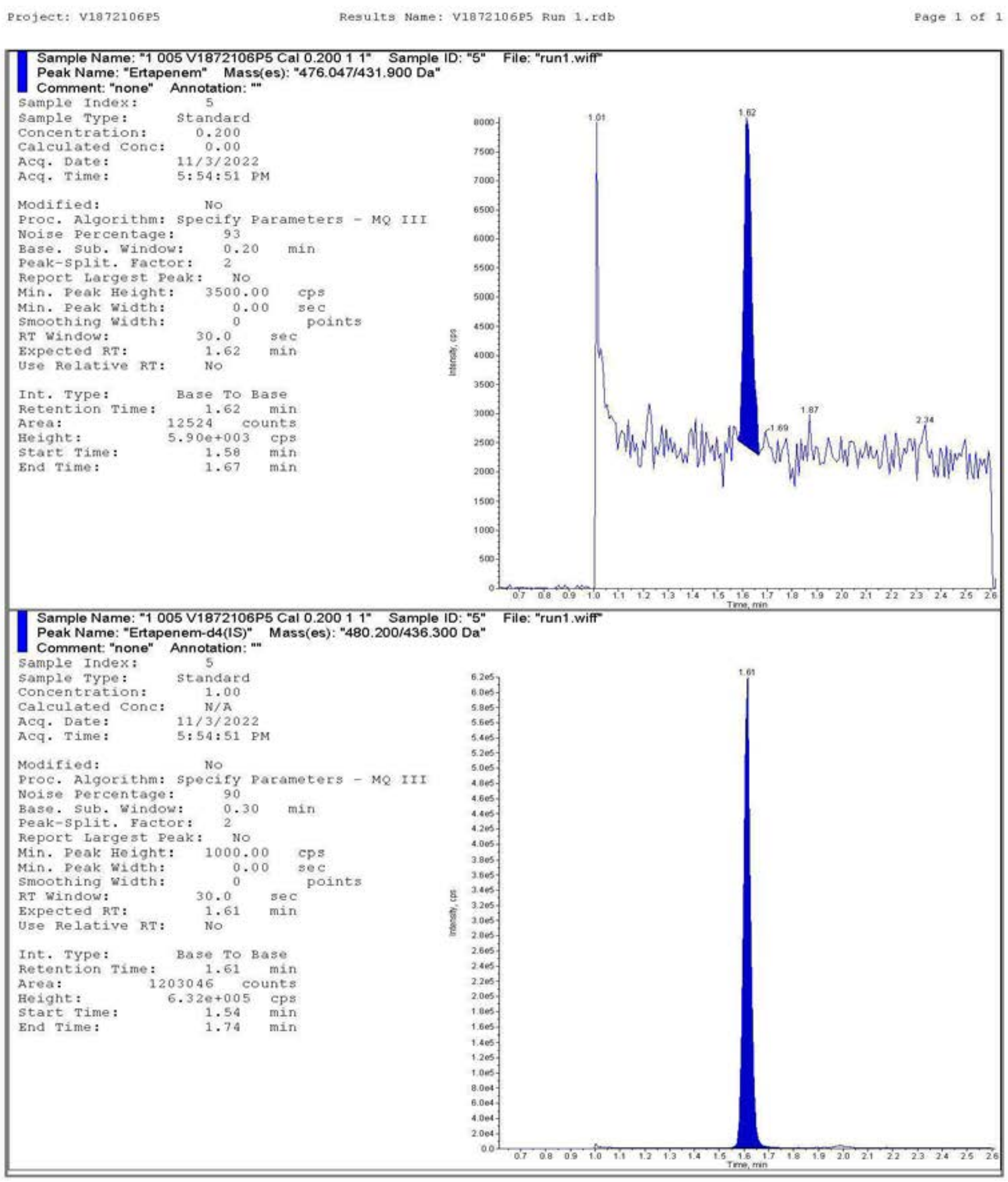

## Figure 5: Typical Ion Chromatogram Calibration Standard, Ertapenem = 80.0 mcg/mL

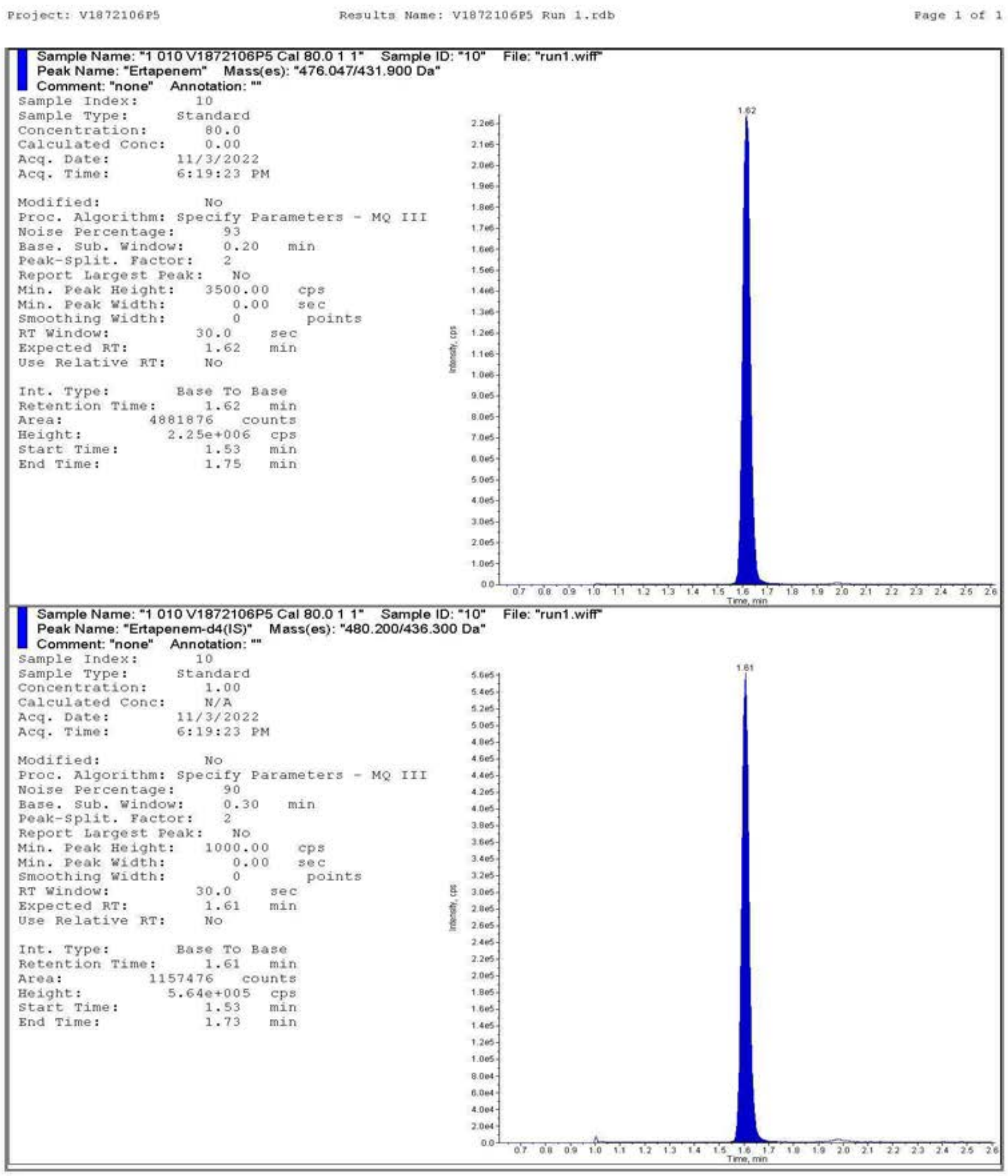

**Figure 6: Typical Ion Chromatogram ULOQ Calibration Standard, Ertapenem = 200 mcg/mL**

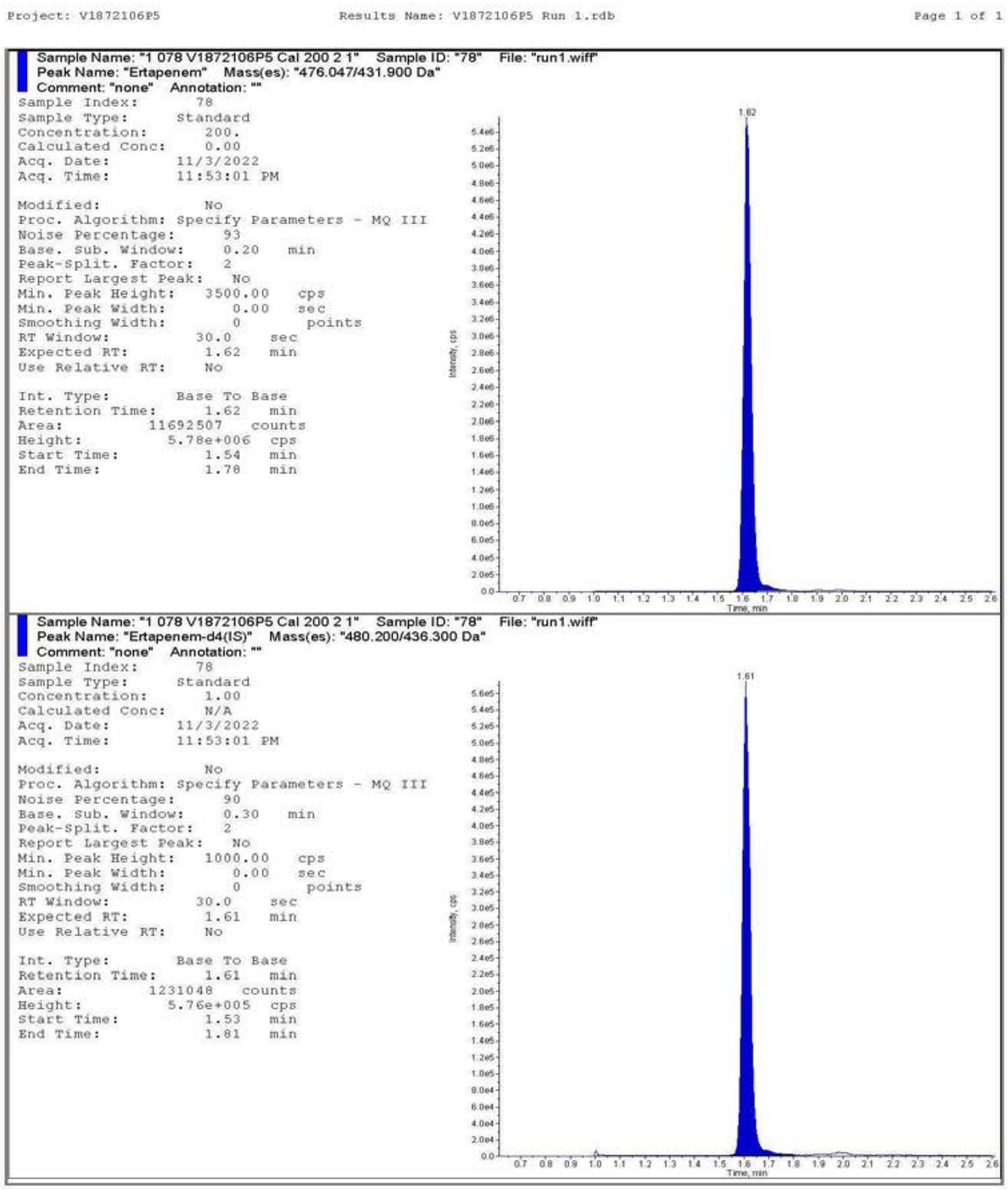

## Figure 7: Typical Ion Chromatogram Cross-Talk Evaluation High QC No IS

Project: V1872106P5    Results Name: V1872106P5 Run 1.rdb    Page 1 of 1

**Sample Name: "1 063 V1872106P5 IS Cross High NO IS 4 1"    Sample ID: "63"    File: "run1.wiff"**
**Peak Name: "Ertapenem"    Mass(es): "476.047/431.900 Da"**
**Comment: "none"    Annotation: ""**

Sample Index: 63
Sample Type: QC
Concentration: 150.
Calculated Conc: 0.00
Acq. Date: 11/3/2022
Acq. Time: 10:39:26 PM

Modified: No
Proc. Algorithm: Specify Parameters - MQ III
Noise Percentage: 93
Base. Sub. Window: 0.20 min
Peak-Split. Factor: 2
Report Largest Peak: No
Min. Peak Height: 3500.00 cps
Min. Peak Width: 0.00 sec
Smoothing Width: 0 points
RT Window: 30.0 sec
Expected RT: 1.62 min
Use Relative RT: No

Int. Type: Base To Base
Retention Time: 1.62 min
Area: 9787587 counts
Height: 4.88e+006 cps
Start Time: 1.52 min
End Time: 1.85 min

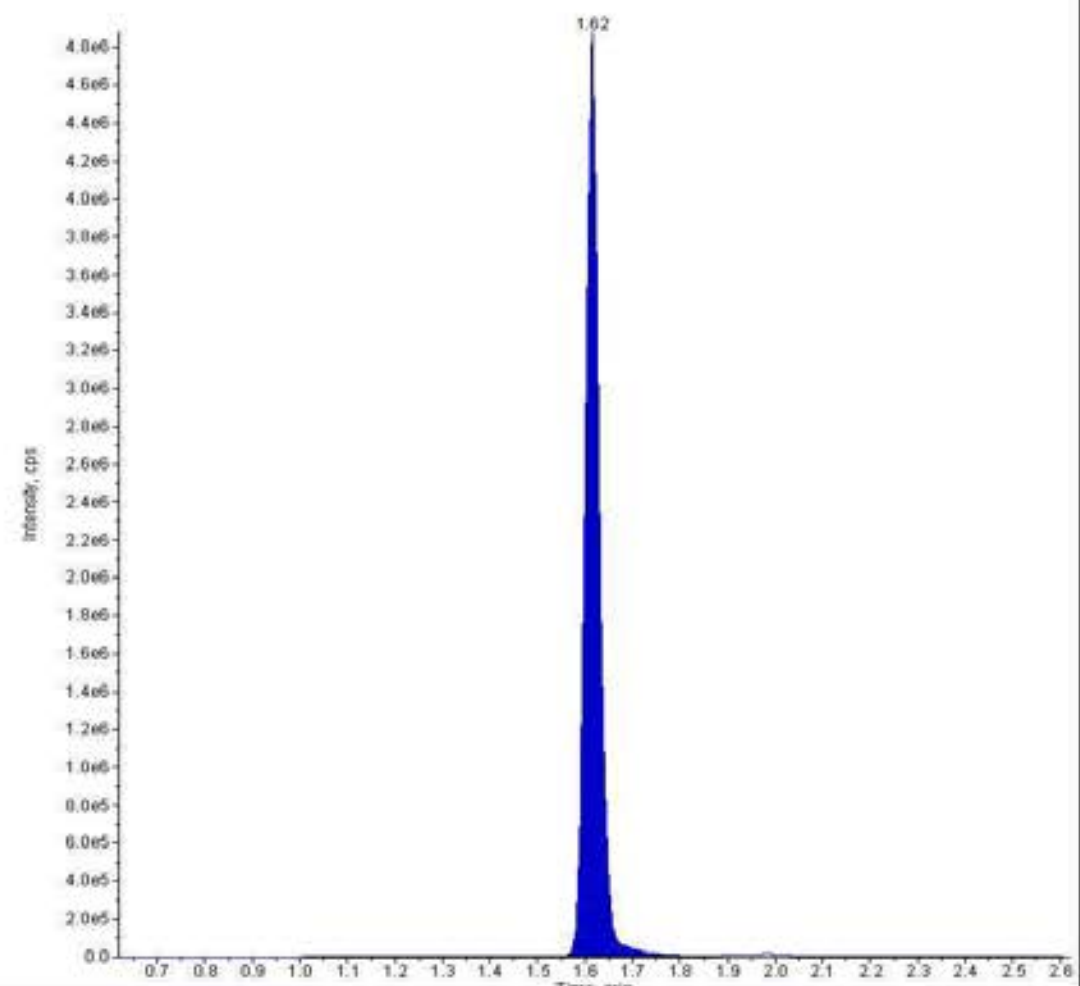


**Sample Name: "1 063 V1872106P5 IS Cross High NO IS 4 1"    Sample ID: "63"    File: "run1.wiff"**
**Peak Name: "Ertapenem-d4(IS)"    Mass(es): "480.200/436.300 Da"**
**Comment: "none"    Annotation: ""**

Sample Index: 63
Sample Type: QC
Concentration: 1.00
Calculated Conc: N/A
Acq. Date: 11/3/2022
Acq. Time: 10:39:26 PM

Modified: No
Proc. Algorithm: Specify Parameters - MQ III
Noise Percentage: 90
Base. Sub. Window: 0.30 min
Peak-Split. Factor: 2
Report Largest Peak: No
Min. Peak Height: 1000.00 cps
Min. Peak Width: 0.00 sec
Smoothing Width: 0 points
RT Window: 30.0 sec
Expected RT: 1.61 min
Use Relative RT: No

Int. Type: Base To Base
Retention Time: 1.61 min
Area: 32127 counts
Height: 1.71e+004 cps
Start Time: 1.55 min
End Time: 1.67 min

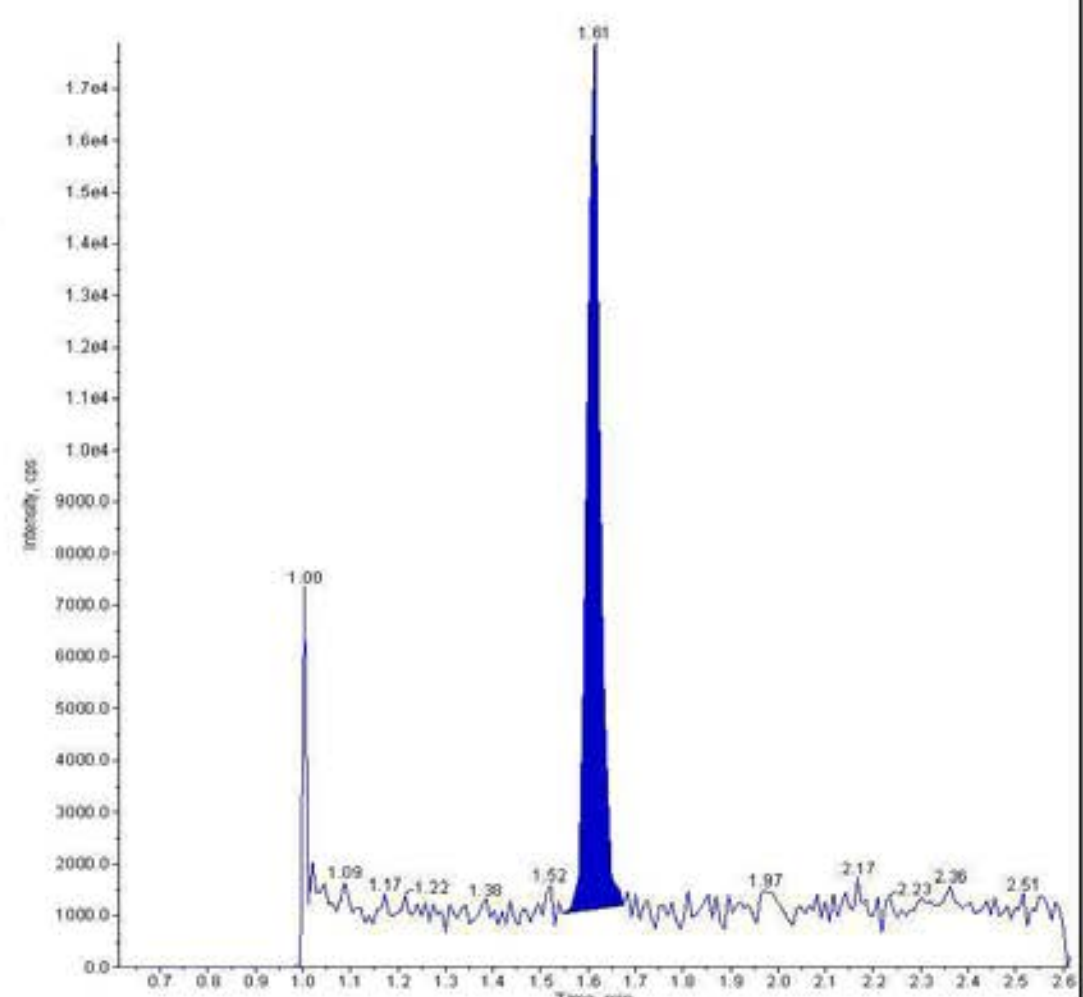

# 1. GENERAL INFORMATION

## 1.1. Client Name: Dynport Vaccine Company

## 1.2. Reference: Validation V1872106P5

# 2. TABLE OF CONTENTS


1. GENERAL INFORMATION ........ 1
1.1. Client Name: Dynport Vaccine Company ........ 1
1.2. Reference: Validation V1872106P5 ........ 1
2. TABLE OF CONTENTS ........ 1
3. SAMPLE REQUIREMENTS ........ 3
4. METHOD SUMMARY ........ 3
5. SPECIAL HANDLING OF BIOLOGICAL SAMPLES ........ 3
6. DATA COLLECTION AND ANALYSIS SOFTWARE ........ 3
7. INSTRUMENTS, EQUIPMENT, AND CONDITIONS ........ 4
7.1. LC and MS Conditions ........ 4
Table 1: HPLC System ........ 4
7.1.1. Gradient Table ........ 5
7.1.2. Autosampler Settings ........ 5
7.1.3. Mass Spectrometer System ........ 5
8. REAGENTS, STANDARDS, AND SUPPLIES ........ 7
Table 2: Reagents, Standards, and Supplies ........ 7
9. SAFETY ........ 7
10. PROCEDURE ........ 8
10.1. Preparation of Analyte Stock Solutions and Dilutions ........ 8


CONFIDENTIAL



Serial Number: f07c17ae-63f9-4a60-86ea-00fa341ccc84 accessed by Eric Johnson (JOHNSONE) on Wed, 23 Nov 2022 17:47:00 GMT.

10.1.1. Ertapenem Primary Solution, 8.00 mg/mL ........ 8
10.1.2. Working Internal Standard Solution, ~ 20 mcg/mL Ertapenem-$d_4$ ........ 8
10.2. Preparation of Other Reagents ........ 8
10.2.1. Mobile Phase A: 0.5% Formic Acid in Deionized Water ........ 8
10.2.2. Mobile Phase B: 90/10 Acetonitrile/Deionized Water ........ 8
10.2.3. Rinse: 90/10 Acetone/ Deionized Water ........ 9
10.2.4. 1 N Sodium Hydroxide (NaOH) in Deionized Water ........ 9
10.2.5. MES ([2-(N-Morpholino) ethane sulphonic acid hydrate]) Buffer pH 6.4, ~0.1M ...... 9
10.2.6. Reconstitution Solvent: 2/98 Acetonitrile/Deionized Water ........ 9
10.3. Preparation of Standards and QC Samples in Ice Water Bath ........ 9
10.3.1. Treated Human Sodium Heparin Plasma (Human Sodium Heparin Plasma: MES Buffer 1:1) ........ 9
10.3.2. Preparation of Secondary Solution 400 mcg/mL (Reported as 800 mcg/mL) ........ 9
10.3.3. Preparation of Standards and QC Samples in Sodium Heparin Plasma Matrix in Ice Water Bath ........ 10
Table 3: Example of Standard Serial Dilution Preparation ........ 10
Table 4: Example of QC Serial Dilution Preparation ........ 11
10.4. Extraction Procedure ........ 11
10.4.1. Dilutions ........ 12
10.5. Calculations ........ 12
11. REFERENCE CHROMATOGRAMS ........ 13
Figure 1: Blank without IS ........ 13
Figure 2: Blank with IS ........ 13
Figure 3: LLOQ ........ 14
Figure 4: ULOQ ........ 14
12. REVISION HISTORY ........ 14

CONFIDENTIAL



Serial Number: f07c17ae-63f9-4a60-86ea-00fa341ccc84 accessed by Eric Johnson (JOHNSONE) on Wed, 23 Nov 2022 17:47:00 GMT.

Table 5: Revision History ....................14

## 3. SAMPLE REQUIREMENTS

Human sodium heparin plasma samples (treated with 1:1 MES Buffer pH 6.4, ~0.1M), minimum volume 100 mcL each, for determination of ertapenem concentration.

Refer to the relevant study protocol for a description of expected samples.

## 4. METHOD SUMMARY

The method involves 96-well protein precipitation followed by using the Sciex 6500 or 5000 LC/MS/MS systems. The concentration range is 0.200 - 200 mcg/mL for ertapenem in human plasma, prior to MES treatment.

## 5. SPECIAL HANDLING OF BIOLOGICAL SAMPLES

The method requires no special handling. Please ensure all "Universal Precautions" are taken. Unknown plasma samples need to be diluted 1:1 with MES buffer after sample collection. The calibration standard and QC concentrations are adjusted in this test procedure, so the unknown samples do not need to be adjusted for the dilution factor of 2x for the treatment with MES buffer.

## 6. DATA COLLECTION AND ANALYSIS SOFTWARE

| Description | Version |
|---|---|
| Watson LIMS™ (Thermo Fisher Scientific) | 7.5 or higher |
| Analyst® (AB Sciex) | 1.7.2 or higher |

CONFIDENTIAL



Serial Number: f07c17ae-63f9-4a60-86ea-00fa341ccc84 accessed by Eric Johnson (JOHNSONE) on Wed, 23 Nov 2022 17:47:00 GMT.

# 7. INSTRUMENTS, EQUIPMENT, AND CONDITIONS

Equivalent equipment can be substituted, except where noted in Section 7.1. Minor modifications of conditions are permitted to optimize chromatography, except where noted in Section 7.1. Refer to SOP-00073 for allowable minor modifications that do not require validation.

## 7.1. LC and MS Conditions

**Table 1: HPLC System**

| System Controller | Shimadzu Controller, CBM20A |
|---|---|
| Pump | Shimadzu LC-30AD |
| Autosampler | Shimadzu SIL-30AC |
| Column Oven | Shimadzu CTO-30A |
| MS Interface | ESI |
| Analytical Column | Fortis Technology Ltd. Fortis C18 4.6 x 50mm, 5 µ |
| Column Guard Cartridge | Security Guard Cartridge AQ C18, 4.0 x 2.0 mm |
| Column Temperature (set at) | 35°C |
| Mobile Phase A | 0.5% Formic Acid in Deionized Water |
| Mobile Phase B | 90/10 Acetonitrile/Deionized Water |
| Needle Wash Solvent (R0, R3) | 90/10 Acetone/Deionized Water or equivalent |
| Total Run Time (approximate) | 4.5 min |
| Injection Volume (µl) | 0.1-2.0 (6500), 0.1-2.0 mcL (5000) |
| Flow Rate | 1.0 mL/min |
| Pressure, kgf/cm2 (approximate) | ~ 100 |
| Approximate Retention Time (min.) | Ertapenem: 1.66 |
| | Ertapenem-d4: 1.66 |

CONFIDENTIAL



Serial Number: f07c17ae-63f9-4a60-86ea-00fa341ccc84 accessed by Eric Johnson (JOHNSONE) on Wed, 23 Nov 2022 17:47:00 GMT.

### 7.1.1. Gradient Table

Time Program:

| Time | Module | Events | Parameter |
|---|---|---|---|
| 0.0 | Pumps | Pump B Conc. | 18 |
| 0.5 | Pumps | Pump B Conc. | 18 |
| 1.0 | Controller | Event | 1 |
| 2.4 | Pumps | Pump B Conc. | 45 |
| 2.5 | Pumps | Pump B Conc. | 100 |
| 2.6 | Controller | Event | 2 |
| 3.5 | Pumps | Pump B Conc. | 100 |
| 3.6 | Pumps | Pump B Conc. | 18 |
| 4.2 | System Controller | Stop | |

### 7.1.2. Autosampler Settings

| | |
|---|---|
| Temperature: | Set at 4°C |
| Rinse Type: | External |
| Rinse Mode: | Before and after aspiration |
| Rinse Dip Time: | 0 sec |
| Rinse Method: | Rinse Pump Then Port |
| Rinse Time: | 2 sec |
| Rinsing Speed: | 35 µL/sec |
| Rinsing Volume: | 500 µL |

### 7.1.3. Mass Spectrometer System

| | |
|---|---|
| Mass Spectrometer | Sciex 5000 or 6500 (Low Mass Mode) |
| Source Type: | Turbo spray |
| Scan Type: | MRM (MRM) |
| Polarity: | Positive |
| Resolution Q1: | Unit |
| Resolution Q3: | Unit |

CONFIDENTIAL



Serial Number: f07c17ae-63f9-4a60-86ea-00fa341ccc84 accessed by Eric Johnson (JOHNSONE) on Wed, 23 Nov 2022 17:47:00 GMT.

*All the below parameters in this section may be adjusted to optimize the instrument conditions.*

**Ertapenem:**

| Q1 Mass (Da) | Q3 Mass (Da) | Dwell (msec) |
|---|---|---|
| 476.047 | 431.9 | 250.00 |
| DP | 56.00 | |
| CE | 13.00 | |
| CXP | 13.00 | |

**Ertapenem-$d_4$:**

| Q1 Mass (Da) | Q3 Mass (Da) | Dwell (msec) |
|---|---|---|
| 480.2 | 436.3 | 250.00 |
| DP | 56.00 | |
| CE | 13.00 | |
| CXP | 13.00 | |

**Parameter Table:**

| | |
|---|---|
| CAD: | 12.00 |
| CUR: | 40.00 |
| GS1: | 50.00 |
| GS2: | 60.00 |
| IS: | 5500 |
| TEM: | 600 |
| EP | 12 |

CONFIDENTIAL



Serial Number: f07c17ae-63f9-4a60-86ea-00fa341ccc84 accessed by Eric Johnson (JOHNSONE) on Wed, 23 Nov 2022 17:47:00 GMT.

# 8. REAGENTS, STANDARDS, AND SUPPLIES

Reagents from alternate supplier and equivalent grade can be substituted.

**Table 2: Reagents, Standards, and Supplies**

| Chemical / Grade | Manufacturer/Supplier |
|---|---|
| Ertapenem Sodium Salt | Medchem Express |
| Ertapenem-$d_4$ Sodium Salt | Alsa Chim |
| MES ([2-(N-Morpholino) ethane sulphonic acid hydrate]) buffer | Sigma |
| Acetone | Honeywell |
| Acetonitrile, HPLC grade | Honeywell |
| Ammonium Acetate ≥ 99.9% | Sigma |
| Deionized Water | Millipore |
| Formic Acid, 98+% | EMD Millipore |
| Methanol | Honeywell |
| 1 N Sodium Hydroxide | Fisher Scientific |
| Human Sodium Heparin Plasma | BioChemed/BioIVT |
| Animal tubes | Sarstedt D51588 |
| Advantage Siliguard Coated 1 mL 96 well Polypropylene plate; round top, round wells | Analytical Sales and Services, Catalog # 17P687GC |
| Advantage SiliGuard Coated 2 mL 96 well Polypropylene plate: Square Top, Round Bottom | Analytical Sales and Services, Catalog # 27P687GC |
| Purple, pre-slit, square well, soft silicone/PTFE Cap Mats | Analytical Sales & Services, Inc., Catalog# 965085 |
| Pre-slit, round well, soft silicone/PTFE Cap Mats | Analytical Sales & Services, Inc., Catalog# 965075 |

CONFIDENTIAL

# 9. SAFETY

Always observe safe laboratory practices. Consult the appropriate SDS before handling any chemical if you are unfamiliar with the proper safety precautions.



Serial Number: f07c17ae-63f9-4a60-86ea-00fa341ccc84 accessed by Eric Johnson (JOHNSONE) on Wed, 23 Nov 2022 17:47:00 GMT.

# 10. PROCEDURE

## 10.1. Preparation of Analyte Stock Solutions and Dilutions

The quantities of analyte solution prepared may be scaled up or down proportionally as needed. Alternate dilution schemes can be used to achieve the appropriate final concentrations of standards, QCs, working internal standard solution. For assignment of expiration dates to analyte stocks and their dilutions, refer to the latest version of the validation report.

### 10.1.1. Ertapenem Primary Solution, 8.00 mg/mL

Check drying conditions prior to use. Weigh approximately 60 mg of Ertapenem (correcting for purity and correction factor if necessary) in a glass container and add the corresponding volume of MES buffer, approximately 7.50 mL. Mix well and sonicate if necessary. Mix well, aliquot, and store at -20°C in polypropylene container.

### 10.1.2. Working Internal Standard Solution, ~ 20 mcg/mL Ertapenem-$d_4$

Ertapenem-$d_4$ is received in 1.0 mg pre-weighed vials. Add deionized water to transfer the whole content to the 50 mL polypropylene container, rinse the vial three times and transfer with MES buffer. Bring to volume with MES buffer to make a final concentration of ~ 20 mcg/mL. Mix well, **aliquot**, and store at -20°C in polypropylene container.

## 10.2. Preparation of Other Reagents

Prepared quantities of all reagents may be scaled up or down as necessary. Volumes provided are examples. For assignment of expiration dates, refer to SOP-00094.

### 10.2.1. Mobile Phase A: 0.5% Formic Acid in Deionized Water

Fill a 4-L Volumetric flask partially with deionized water. Add 20 mL of Formic Acid, then dilute to volume with deionized water. Mix well, transfer to a 4-L glass container and store at room temperature.

### 10.2.2. Mobile Phase B: 90/10 Acetonitrile/Deionized Water

Combine 3600 mL of acetonitrile with 400 mL of deionized water in a 4-L glass bottle. Mix well and store at room temperature.

CONFIDENTIAL



Serial Number: f07c17ae-63f9-4a60-86ea-00fa341ccc84 accessed by Eric Johnson (JOHNSONE) on Wed, 23 Nov 2022 17:47:00 GMT.

### 10.2.3. Rinse: 90/10 Acetone/ Deionized Water

Combine 3600 mL of acetone and 400 mL of deionized water in a 4-L Container. Mix well and store at room temperature.

### 10.2.4. 1 N Sodium Hydroxide (NaOH) in Deionized Water

Weight ~ 4.00 g of sodium hydroxide (F.W. 40.00) and gradually transfer it to a 100-mL volumetric flask containing ~50 mL of deionized water. Mix to dissolve and wait until it reaches room temperature. Dilute to volume with deionized water and mix well. Store in a polypropylene container at room temperature.

### 10.2.5. MES ([2-(N-Morpholino) ethane sulphonic acid hydrate]) Buffer pH 6.4, ~0.1M

Weigh ~ 4000 mg of MES hydrate (F.W. 195.24) and transfer to a 200-mL volumetric flask containing ~150 mL of deionized water. Dissolve and adjust the pH to 6.4 with 1N NaOH. Dilute to volume with deionized water. Mix well and store refrigerated.

### 10.2.6. Reconstitution Solvent: 2/98 Acetonitrile/Deionized Water

Transfer 40 mL of acetonitrile into 2-L volumetric flask containing about 1500 of deionized water. Bring to volume with deionized water. Mix well. Store at room temperature.

## 10.3. Preparation of Standards and QC Samples in Ice Water Bath

### 10.3.1. Treated Human Sodium Heparin Plasma (Human Sodium Heparin Plasma: MES Buffer 1:1)

Add 100 mL of chilled human sodium heparin plasma to 100 mL of chilled 0.1 M MES Buffer pH 6.4 in an appropriate polypropylene container. Mix well and store at -20°C.

### 10.3.2. Preparation of Secondary Solution 400 mcg/mL (Reported as 800 mcg/mL)

Combine 250 mcL of the Ertapenem primary (8.00 mg/mL) and 4.75 mL of blank treated (1:1 MES buffer: plasma) human sodium heparin plasma using an adjustable pipet and polypropylene tubes. Mix well and store solutions in polypropylene at -70°C.

CONFIDENTIAL



Serial Number: f07c17ae-63f9-4a60-86ea-00fa341ccc84 accessed by Eric Johnson (JOHNSONE) on Wed, 23 Nov 2022 17:47:00 GMT.

### 10.3.3. Preparation of Standards and QC Samples in Sodium Heparin Plasma Matrix in Ice Water Bath

Use treated human sodium heparin plasma that has been diluted 1:1 with MES buffer and screened for the lack of interference at the retention times of ertapenem and ertapenem-$d_4$ (IS). Perform a serial dilution from the secondary solution (400 mcg/mL) to create Standards and QC Samples. Since unknown samples are treated with the MES buffer after collection at a 1:1 ratio, the calibration standard and QC concentrations are based on the plasma volume only. Unknown samples do not need to be adjusted for the dilution with MES buffer. The prepared concentrations in plasma only, not accounting for MES buffer, will be as follows: For standards: 200, 160, 80.0, 40.0, 20.0, 4.00, 0.400 and 0.200 mcg/mL (the actual standard drug concentrations in the MES/plasma mixture are 100, 80.0, 40.0, 20.0, 10.0, 2.00, 0.200, and 0.100 mcg/mL). For QCs: 150, 100, and 0.600 mcg/mL, with 0.200 mcg/mL for the LLOQ QC when needed (the actual QC drug concentrations in the MES/plasma mixture are 75.0, 50.0, and 0.300 mcg/mL, with 0.100 mcg/mL for the LLOQ QC). Other concentrations may be prepared, provided they meet the requirements of SOP-00035 and provided they remain within the range of 0.200 to 200 mcg/mL in the plasma, prior to MES treatment. Store solutions in polypropylene at -70°C or -20°C. Examples of serial dilution schemes are provided below. Volumes for these preps can be adjusted.

**Table 3: Example of Standard Serial Dilution Preparation**

| Final Standard Conc. mcg/mL | Concentrate Conc. mcg/mL | Pipette Volume mL (Spike) | Treated Plasma Volume mL | Watson Concentration |
|---|---|---|---|---|
| 100 | 400 | 2 | 6 | 200 |
| 80 | 100 | 4 | 1 | 160 |
| 40 | 80 | 3 | 3 | 80.0 |
| 20 | 40 | 3 | 3 | 40.0 |
| 10 | 20 | 2 | 2 | 20.0 |
| 2 | 10 | 1 | 4 | 4.00 |
| 0.800 | 2 | 2 | 3 | - |
| 0.200 | 0.800 | 1 | 3 | 0.400 |
| 0.100 | 0.200 | 2 | 2 | 0.200 |

CONFIDENTIAL



Serial Number: f07c17ae-63f9-4a60-86ea-00fa341ccc84 accessed by Eric Johnson (JOHNSONE) on Wed, 23 Nov 2022 17:47:00 GMT.

**Table 4: Example of QC Serial Dilution Preparation**

| Final QC Conc. mcg/mL | Concentrate Conc. mcg/mL | Pipette Volume mL (Spike) | Treated Plasma Volume mL | Watson Concentration |
|---|---|---|---|---|
| 100 | 400 | 4 | 12 | - |
| 75.0 | 100 | 9 | 3 | 150 |
| 50.0 | 100 | 4 | 4 | 100 |
| 15.0 | 50 | 1.5 | 3.5 | - |
| 1.50 | 15 | 0.5 | 4.5 | - |
| 0.300 | 1.5 | 2 | 8 | 0.600 |
| 0.100 | 0.3 | 2 | 4 | 0.200 |

## 10.4. Extraction Procedure

1. Thaw MES treated human plasma samples on ice water bath. Once samples are thawed, either vortex the batch as a whole for ~2 minutes, followed by vortexing each for 2 seconds, or vortex each sample for ~ 10 seconds each.
2. Pipet 100 mcL of sample or blank to corresponding wells in a 2 mL Siliguard 96-well plate on ice.
3. Add 50 mcL of COLD WIS (~20 mcg/mL Ertapenem-d4 in MES buffer) to all applicable wells. For Control Blanks, add 50 mcL of MES buffer instead.
4. Seal the plate with adhesive pad (or equivalent) and vortex at 1300 rpm for 3 minutes using a plate shaker.
5. Add 250 mcL of acetonitrile to all wells. Seal the plate with an adhesive pad (or equivalent) and mix the samples by using a plate shaker. Start at 800 rpm, then slowly increase speed to 1200 rpm. Maintain 1200 rpm for at least 5 min.
6. Set Plate to equilibrate at room temperature for 10 minutes.
7. Centrifuge the plate for 10 minutes at the setting of 4000 rpm at room temperature.
8. Add 250 mcL of reconstitution solvent (2/98 acetonitrile/deionized water) into a new 1-mL 96-well Siliguard plate.

CONFIDENTIAL



Serial Number: f07c17ae-63f9-4a60-86ea-00fa341ccc84 accessed by Eric Johnson (JOHNSONE) on Wed, 23 Nov 2022 17:47:00 GMT.

9. Transfer 40 mcL of supernatant to the new 1-mL 96-well Siliguard plate using an automated liquid handler or a multichannel pipette (or equivalent).
10. Seal the plate with a purple lid. Mix on a plate shaker. Start at 800 rpm, then slowly increase speed to 1300 rpm. Maintain at 1300 rpm for at least 3 minutes.
11. Store samples in refrigerator until ready for LC-MS injection.

### 10.4.1. Dilutions

2x = 50 mcL sample + 50 mcL blank matrix (treated)

5x = 20 mcL sample + 80 mcL of blank matrix (treated)

## 10.5. Calculations

Linear regression is performed with a $1/x^2$ weighting factor.

Equation: $y = a + bx$

Where y = analyte/internal standard area ratio

x = concentration (mcg/mL)

b = slope

a = intercept

CONFIDENTIAL



Serial Number: f07c17ae-63f9-4a60-86ea-00fa341ccc84 accessed by Eric Johnson (JOHNSONE) on Wed, 23 Nov 2022 17:47:00 GMT.

## 11. REFERENCE CHROMATOGRAMS

**Figure 1: Blank without IS**

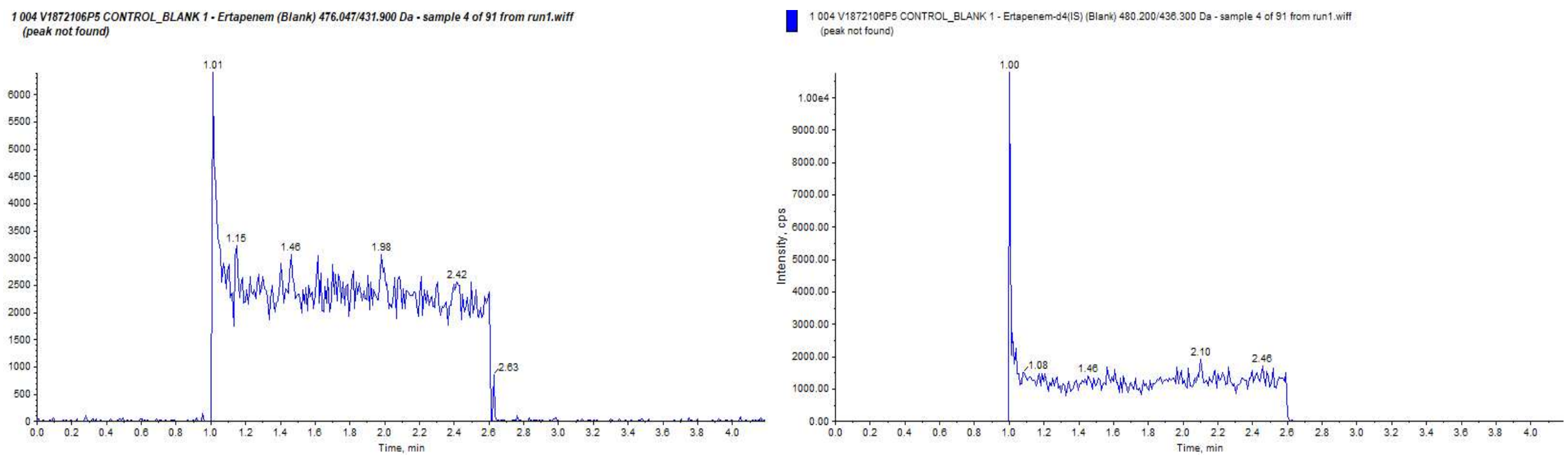


**Figure 2: Blank with IS**

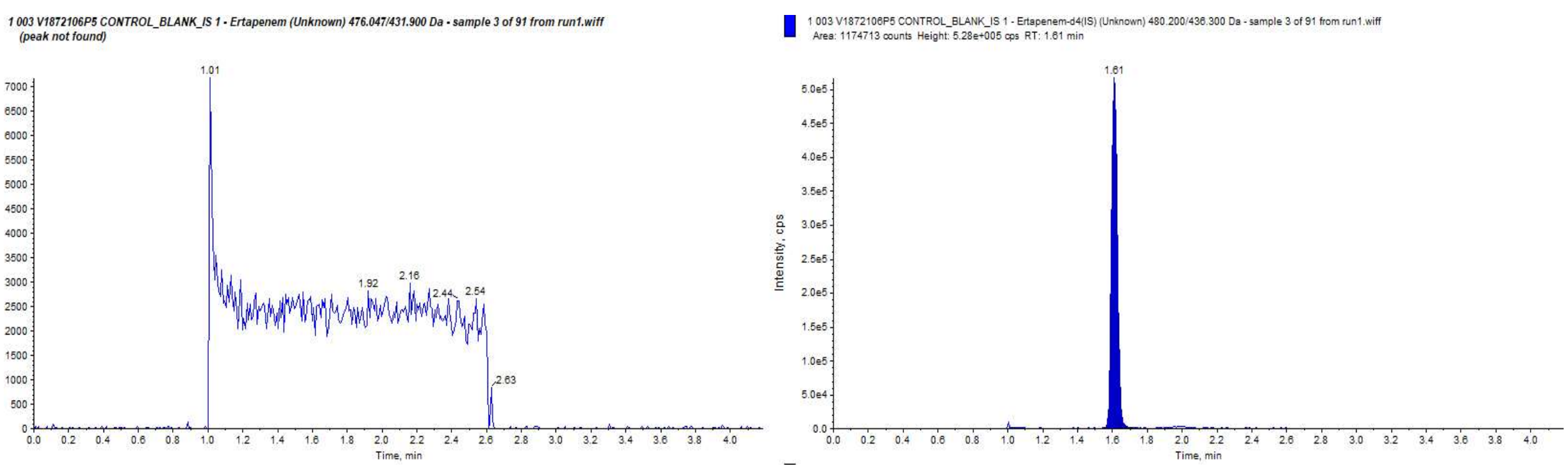


CONFIDENTIAL



Serial Number: f07c17ae-63f9-4a60-86ea-00fa341ccc84 accessed by Eric Johnson (JOHNSONE) on Wed, 23 Nov 2022 17:47:00 GMT.

**Figure 3: LLOQ**

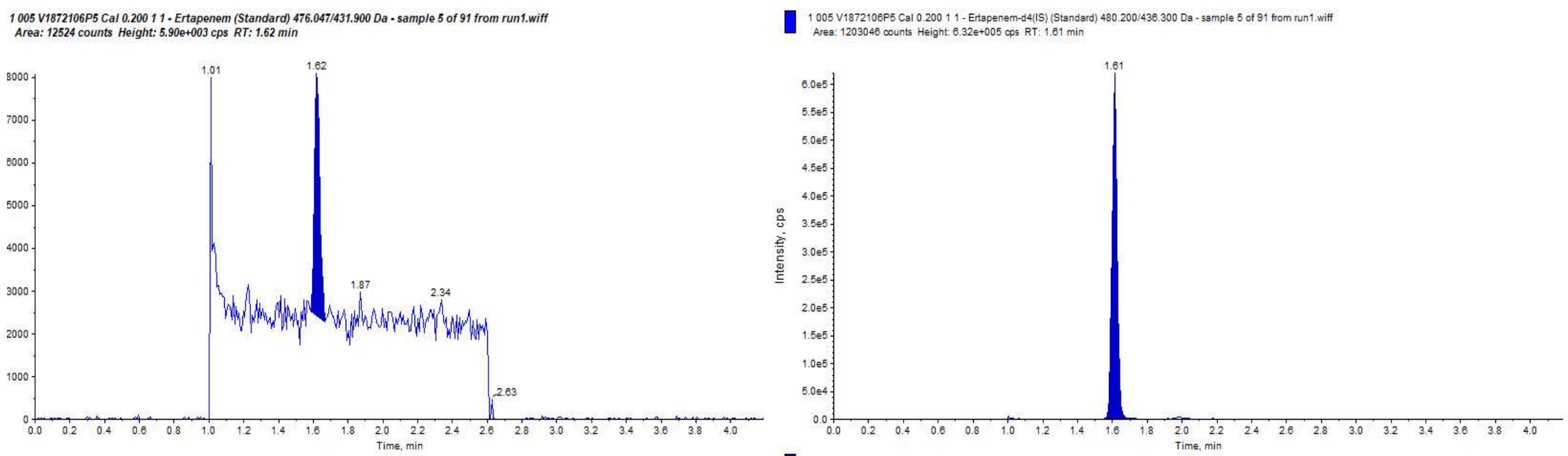


**Figure 4: ULOQ**

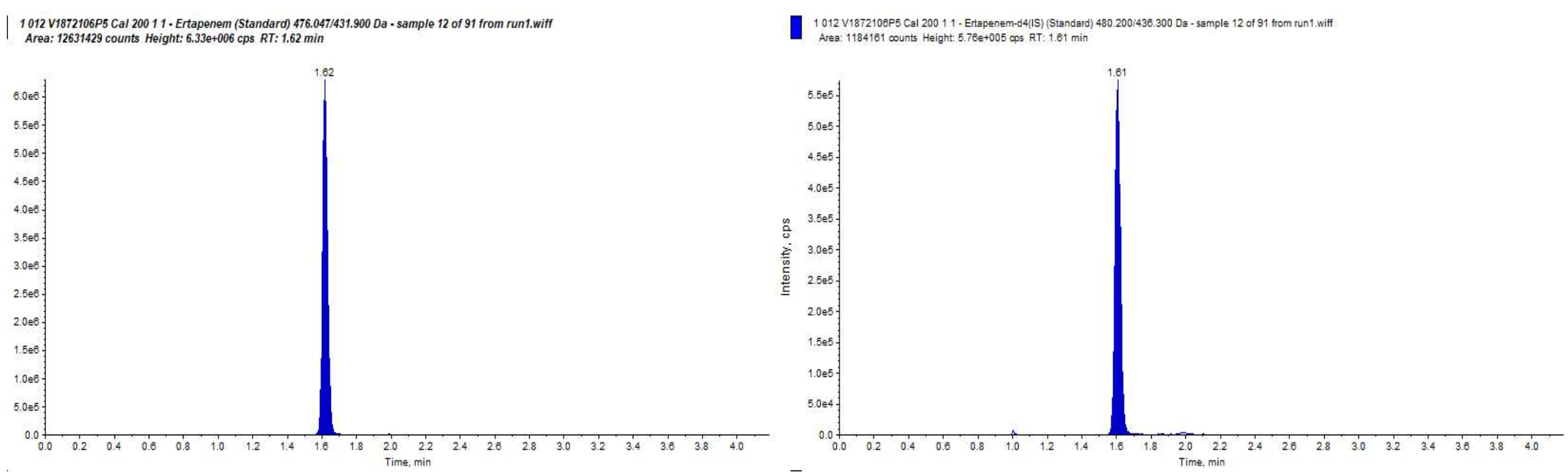


## 12. REVISION HISTORY

**Table 5: Revision History**

| Revision | Effective Date | Section | Revision Summary |
|---|---|---|---|
| 00 | See Header | NA | New Procedure |

CONFIDENTIAL



Serial Number: f07c17ae-63f9-4a60-86ea-00fa341ccc84 accessed by Eric Johnson (JOHNSONE) on Wed, 23 Nov 2022 17:47:00 GMT.

MedChemExpress

# Certificate of Analysis

Inhibitors • Screening Libraries • Proteins

## Ertapenem sodium

| | |
|---|---|
| Cat. No.: | HY-13625 |
| CAS No.: | 153773-82-1 |
| Batch No.: | 180017 |
| Chemical Name: | 1-Azabicyclo[3.2.0]hept-2-ene-2-carboxylic acid, 3-[[(3S,5S)-5-[[(3-carboxyphenyl)amino]carbonyl]-3-pyrrolidinyl]thio]-6-[(1R)-1-hydroxyethyl]-4-methyl-7-oxo-, sodium salt (1:1), (4R,5S,6S)- |

### PHYSICAL AND CHEMICAL PROPERTIES

| | |
|---|---|
| Molecular Formula: | $C_{22}H_{24}N_3NaO_7S$ |
| Molecular Weight: | **497.50** |
| Storage: | 4°C, sealed storage, away from moisture<br>* In solvent : -80°C, 6 months; -20°C, 1 month (sealed storage, away from moisture) |

Chemical Structure:

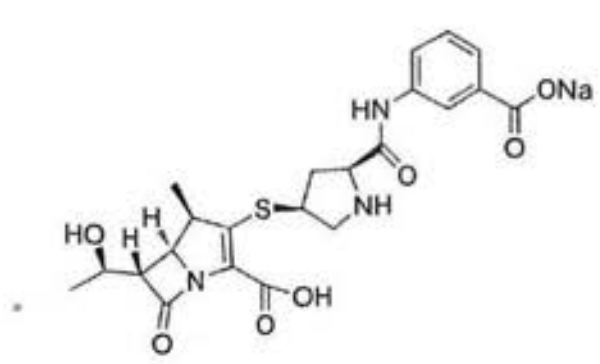


### ANALYTICAL DATA

| | |
|---|---|
| Appearance: | White to light yellow (Solid) |
| $^1$H NMR Spectrum: | Consistent with structure |
| LCMS: | Consistent with structure |
| Purity (LCMS): | **98.86%** |
| Test date: | 2022/08/12 |
| Retest date: | 2024/08/11 |
| Conclusion: | The product has been tested and complies with the given specifications. |

Name: Lingli Ma
Designation: QA Manager
Date: 6/27/2022

**Caution: Product has not been fully validated for medical applications. For research use only.**

Tel: 609-228-6898 Fax: 609-228-5909 E-mail: tech@MedChemExpress.com
Address: 1 Deer Park Dr, Suite Q, Monmouth Junction, NJ 08852, USA

## SAMPLE INFORMATION

| | | | |
|---|---|---|---|
| CAS NO.: | 153773-82-1 | Acquired By: | LY |
| Sample infromation: | | Date Acquired: | 8/12/2022 2:04:10 PM CST |
| Vial: | 2:A,3 | Acq. Method Set: | 1_POS_2MIN |
| Injection #: | 1 | Date Processed: | 8/12/2022 2:36:50 PM CST, |
| Injection Volume: | 0.30 ul | Processing Method: | PDA_01, MS_scan |
| Run Time: | 2.8 Minutes | Channel Name: | PDA Ch1 214nm@4.8nm, MS TIC, |
| Sample Set Name: | 20220812 | Column Name: | A-RP-985 |

### Auto-Scaled Chromatogram

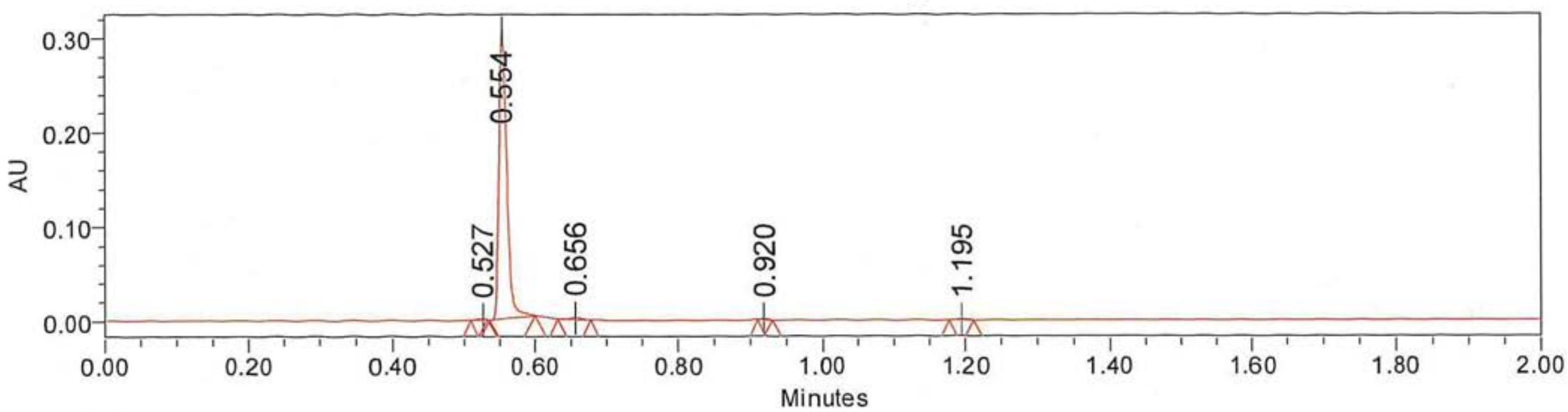


Processed Channel Descr. PDA Ch2 254nm@4.8nm

### Auto-Scaled Chromatogram

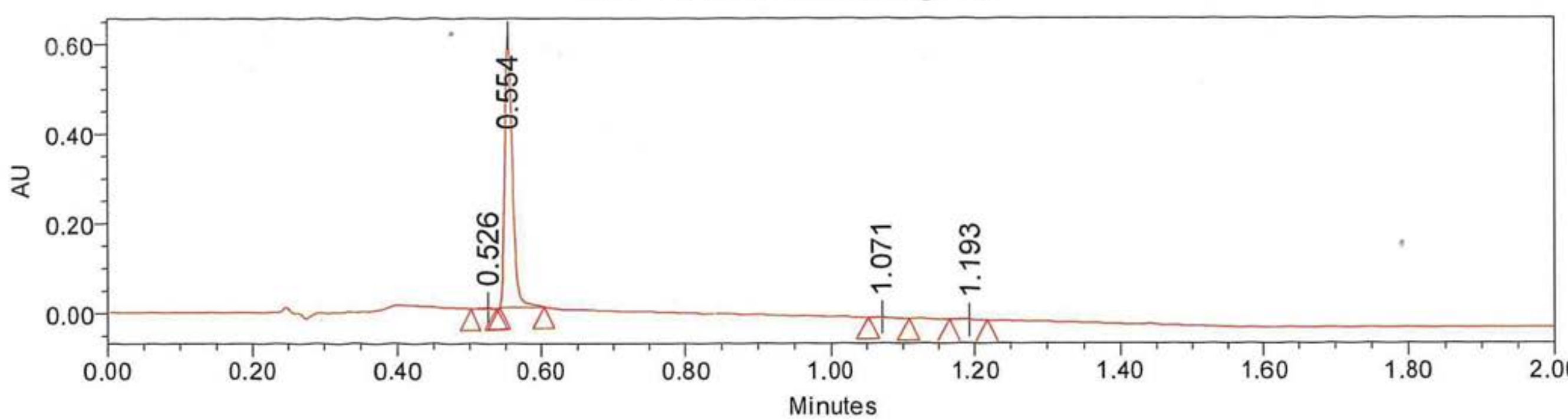


Processed Channel Descr. PDA Ch1 214nm@4.8nm

### Auto-Scaled Chromatogram

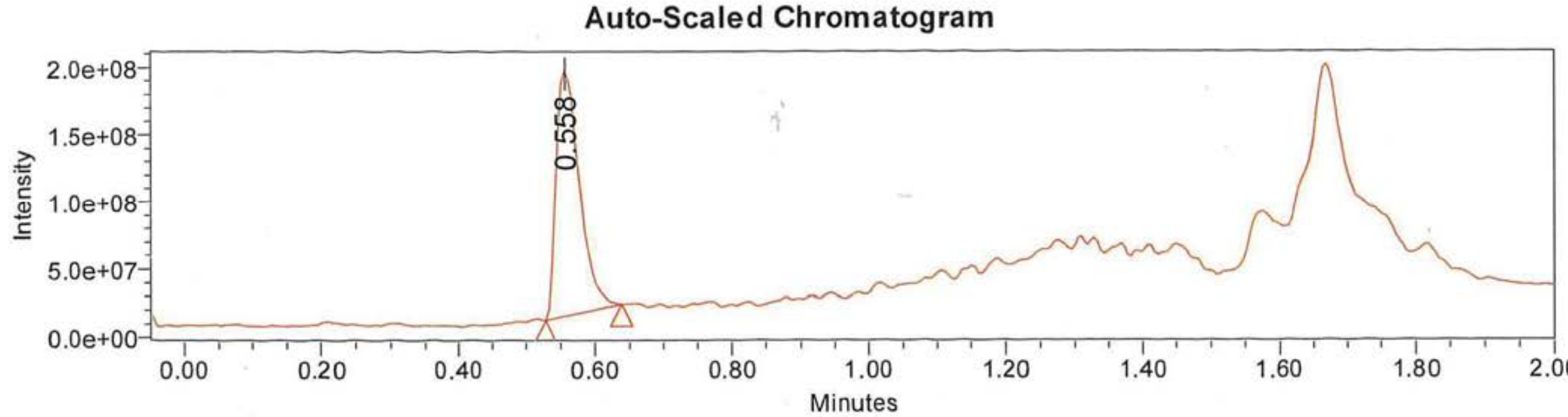


Processed Channel Descr. ACQ-SQD2 1: MS Scan MS TIC, Smoothed by 9 point Savitzky-Golay Filter., Time offset by -0.053 mins. (1: 150.00-1000.00 ES+, Centroid, CV=30)

Peak Results
Channel Name: PDA Ch2 254nm@4.8nm

| | RT | Width (sec) | Area | Height | % Area | Base Peak (Combined) (m/z) | Channel Name |
|---|---|---|---|---|---|---|---|
| 1 | 0.527 | 1.400 | 477 | 640 | 0.20 | 153.83 | PDA Ch2 254nm@4.8nm |
| 2 | 0.554 | 3.800 | 237134 | 308413 | 98.19 | 151.96 | PDA Ch2 254nm@4.8nm |
| 3 | 0.656 | 2.650 | 2446 | 2436 | 1.01 | 151.06 | PDA Ch2 254nm@4.8nm |
| 4 | 0.920 | 1.200 | 632 | 1080 | 0.26 | 152.07 | PDA Ch2 254nm@4.8nm |
| 5 | 1.195 | 2.050 | 823 | 941 | 0.34 | 152.07 | PDA Ch2 254nm@4.8nm |

Peak Results
Channel Name: PDA Ch1 214nm@4.8nm

| | RT | Width (sec) | Area | Height | % Area | Base Peak (Combined) (m/z) | Channel Name |
|---|---|---|---|---|---|---|---|
| 1 | 0.526 | 2.150 | 1796 | 2101 | 0.38 | 153.84 | PDA Ch1 214nm@4.8nm |
| 2 | 0.554 | 3.700 | 471358 | 613577 | 98.86 | 151.97 | PDA Ch1 214nm@4.8nm |
| 3 | 1.071 | 3.350 | 1629 | 1281 | 0.34 | 151.03 | PDA Ch1 214nm@4.8nm |
| 4 | 1.193 | 3.150 | 1997 | 1243 | 0.42 | 152.07 | PDA Ch1 214nm@4.8nm |

Peak Results
Channel Name: MS TIC

| | RT | Width (sec) | Area | Height | % Area | Base Peak (Combined) (m/z) | Channel Name |
|---|---|---|---|---|---|---|---|
| 1 | 0.558 | 6.673 | 431532509 | 182380390 | 100.00 | 476.14 | MS TIC |

**Match Plot**

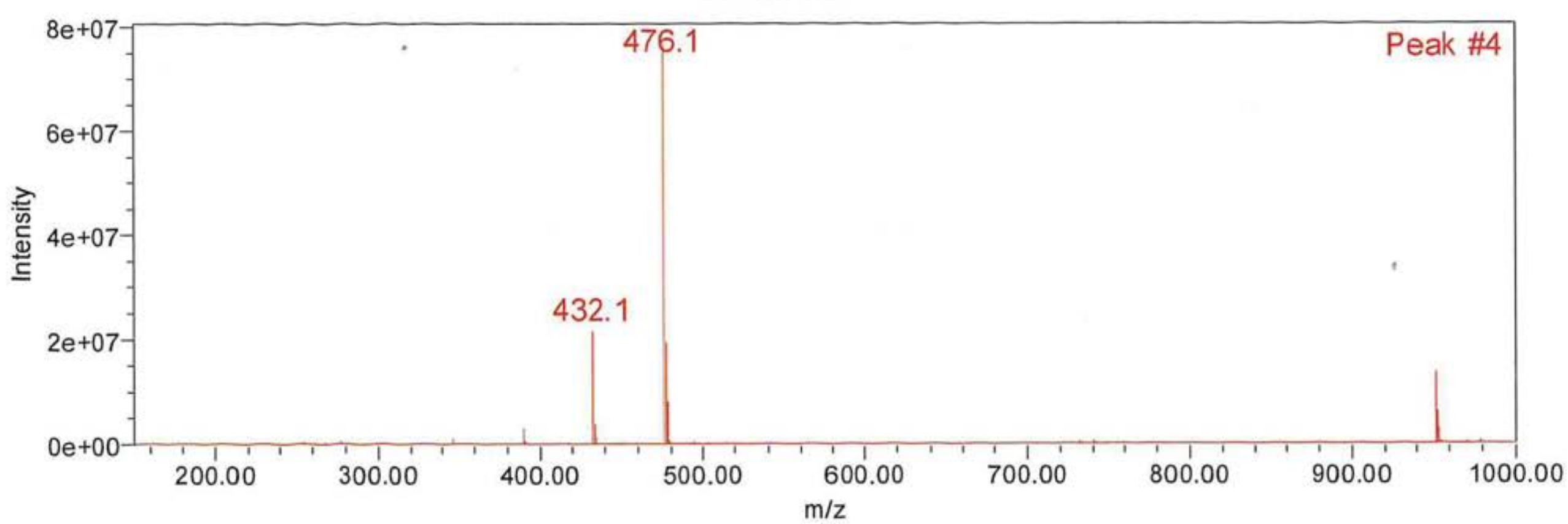


Retention Time 0.558 Channel Name MS TIC

CAS NO.: 153773-82-1, D2O

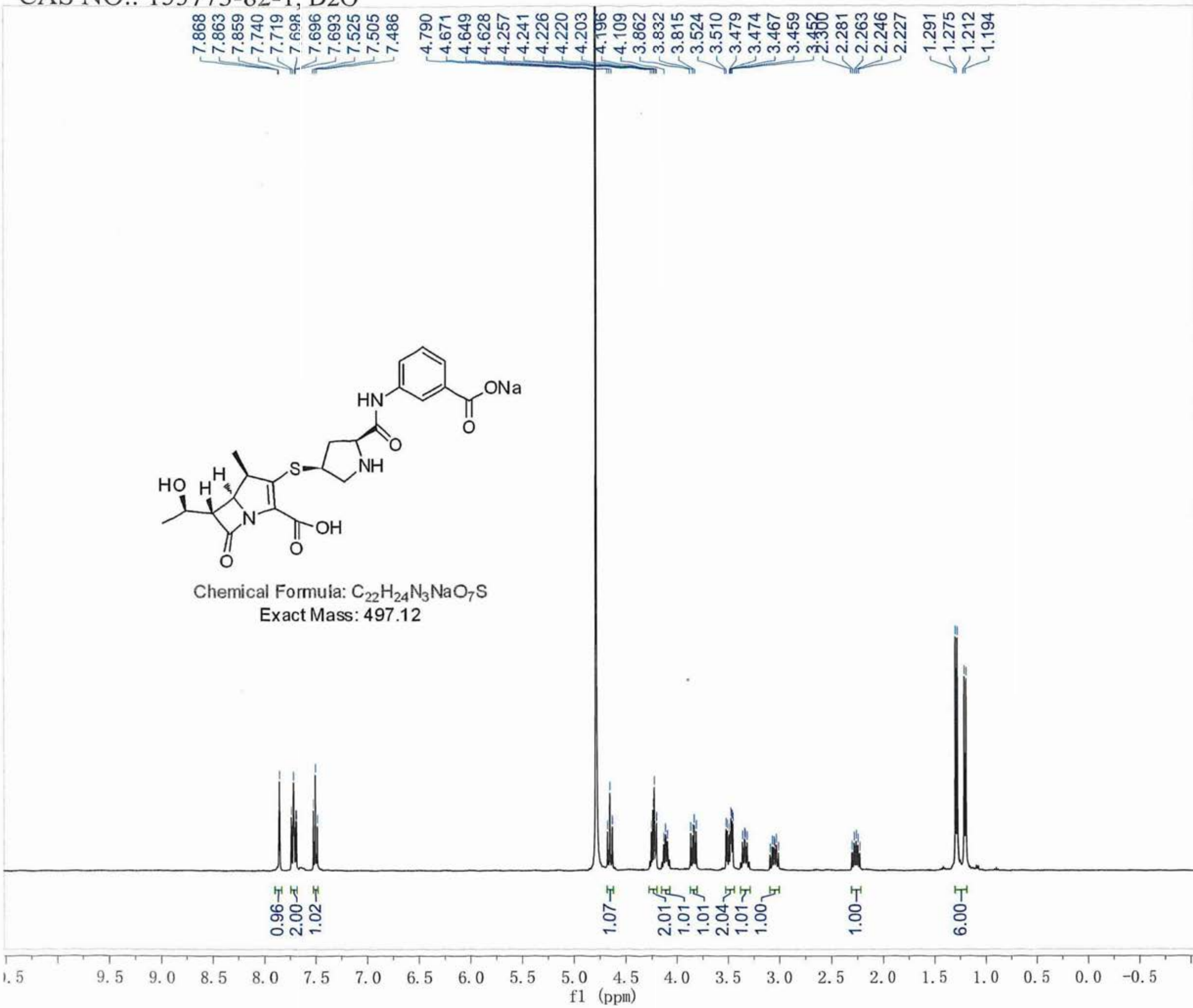


Chemical Formula: $C_{22}H_{24}N_3NaO_7S$
Exact Mass: 497.12

| | Parameter | Value |
|---|---|---|
| 1 | Title | |
| 2 | Origin | Bruker BioSpin GmbH |
| 3 | Owner | nmrsu |
| 4 | Spectrometer | Avance |
| 5 | Author | |
| 6 | Solvent | D2O |
| 7 | Temperature | 298.2 |
| 8 | Pulse Sequence | zg30 |
| 9 | Experiment | 1D |
| 10 | Number of Scans | 8 |
| 11 | Receiver Gain | 101 |
| 12 | Relaxation Delay | 1.0000 |
| 13 | Pulse Width | 8.0000 |
| 14 | Acquisition Time | 3.9977 |
| 15 | Acquisition Date | 2022-08-10T13:49:06 |
| 16 | Modification Date | 2022-08-10T13:53:00 |
| 17 | Spectrometer Frequency | 400.13 |
| 18 | Nucleus | 1H |

ALSACHIM
a Shimadzu Group Company

# CERTIFICATE OF ANALYSIS

| | |
|---|---|
| **Product Name:** | [$^{2}H_{4}$]-Ertapenem sodium salt |
| **Molecular Formula:** | $C_{22}H_{24}N_{3}O_{7}S$ . Na |
| **Labeled Molecular Formula:** | $C_{22}H_{20}D_{4}N_{3}O_{7}S$ . Na |
| **Molecular Weight:** | 501.52 g/mol |
| **Structure:** | |

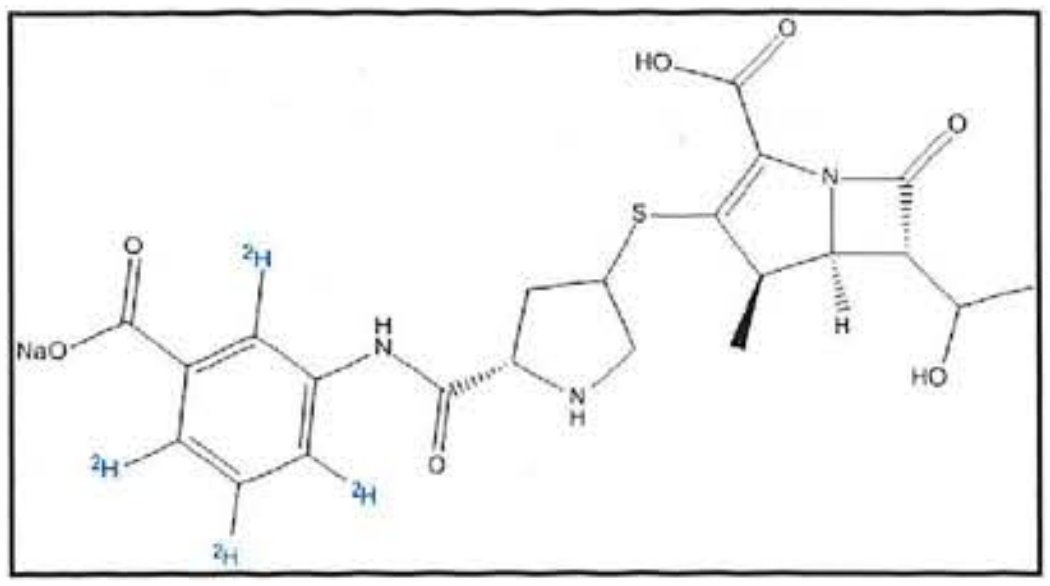


| | | |
|---|---|---|
| **Batch Number:** | DH-ALS-20-002-P2 | |
| **Product Number:** | C3376 | |
| **CAS Number:** | 153773-82-1 unlabeled | |
| **Storage:** | For a prolonged period, keep at -20°C (±5°C) in a closed container, away from light and moisture | |
| **Stability:** | Stable (12 months) (*) | |
| **Extended expiring date:** | Saturday April 29, 2023 | |
| **Solvent solubility:** | Soluble in Water or Acetonitrile/Water (1/1) | |
| | **Specification:** | **Results:** |
| **Appearance:** | Pale grey to white solid | Off white solid |
| **Chemical Purity (HPLC):** | ≥ 80.0% | 89,4% |
| **Isotopic Enrichment:** | ≥ 98.0% $^{2}H$ | 99.0% $^{2}H$ |
| **Unlabeled (M+0) Content:** | Unlabeled (M+0) content < 0.5% | <0.5% |
| **Mass Spectrometry:** | Conform to structure | Conforms |
| **Manufacturing date:** | Tuesday January 7, 2020 | |
| **Reanalysis date:** | Friday April 29, 2022 | |
| **Note:** | For research and development use only, not for use in humans. | |

**Quality Review & Approval**

Francis ROESSLINGER, Quality Manager
Date : Wednesday, May 18, 2022


RECEIVED
SEP 21 REC'D
By

ALSACHIM
a Shimadzu Group Company

# Analysis Report

## <Equipment Information>

LCMS Shimadzu

Alsachim Reference: AN-LC01 + AN-MS01

Pump : LC-40D

Degassing Unit : DGU-405

Auto Sampler : SIL-40C

Diode Array Detector : SPD-M40

Column Oven : CTO-40S

Valve Unit : FCV-20AH2

Liquid Chromatograph Mass Spectrometer : LCMS-2020

Column : Accucore C18 50*4.6 2.6um PN: 17126-054630 SN: 20333623 LOT: 20512

## <Sample Information>

Sample Name : DH-ALS-20-002-P2
Data Filename : DH-ALS-20-002-P2_002.lcd
Method Filename : HP-C18-AF-20MIN.lcm
Batch Filename : 2022-APR-29-3.lcb
Vial # : 2-20
Injection Volume : 0,5 uL
Date Acquired : 29/04/2022 14:31:49
Date Processed : 02/05/2022 08:59:47
Solvent : Water
Concentration : 1 mg/ml
Operator : CD

## <Peak Table >

PDA Ch1 295nm

| Peak# | Ret. Time | Area | Height | Area% |
|---|---|---|---|---|
| 1 | 1,861 | 2224 | 1305 | 0,544 |
| 2 | 2,364 | 16348 | 3642 | 4,001 |
| 3 | 2,684 | 365589 | 157196 | 89,478 |
| 4 | 2,940 | 5546 | 2826 | 1,357 |
| 5 | 3,046 | 2444 | 1326 | 0,598 |
| 6 | 3,202 | 596 | 403 | 0,146 |
| 7 | 3,540 | 15028 | 2285 | 3,678 |
| 8 | 3,776 | 806 | 433 | 0,197 |
| Total | | 408582 | 169417 | 100,000 |

HPLC method and UV Instrument Parameters

<<Pump>>

| | |
|---|---|
| Mode | : Binary gradient |
| Pump A | : LC-40D X3 |
| Pump B | : LC-40D X3 |
| PressMax | : 400 bar |
| PressMin | : 0 bar |
| Pump A Valve Model | : Switching Valve |
| Pump B Valve Model | : Switching Valve |
| Gradient Program: | |

Pump A + B

| # | Time(min) / Flow(mL/min) / B.Conc(%) / B.Curve |
|---|---|
| 1 | 0,00/ 1,2000/ 1,0/ 0 |
| 2 | 9,00/ / 70,0/ |
| 3 | 15,00/ / 99,0/ |
| 4 | 19,00/ / 99,0/ |
| 5 | 19,10/ / 1,0/ |

Pump A Mobile Phase Settings : Water + 0.1% formic acid 0,45 /GPa
Pump B Mobile Phase Settings : Acetonitrile + 0.1% formic acid 1,20 /GPa

<<LC Time Program>>

| Time | Module | Command | Value | Comment |
|---|---|---|---|---|
| 20,00 | Controller | Stop | | |

<<PDA>>

| | |
|---|---|
| Lamp | : D2 & W |
| Start Wavelength | : 190 nm |
| End Wavelength | : 800 nm |

## <HPLC-UV Chromatogram >

Chromatogram

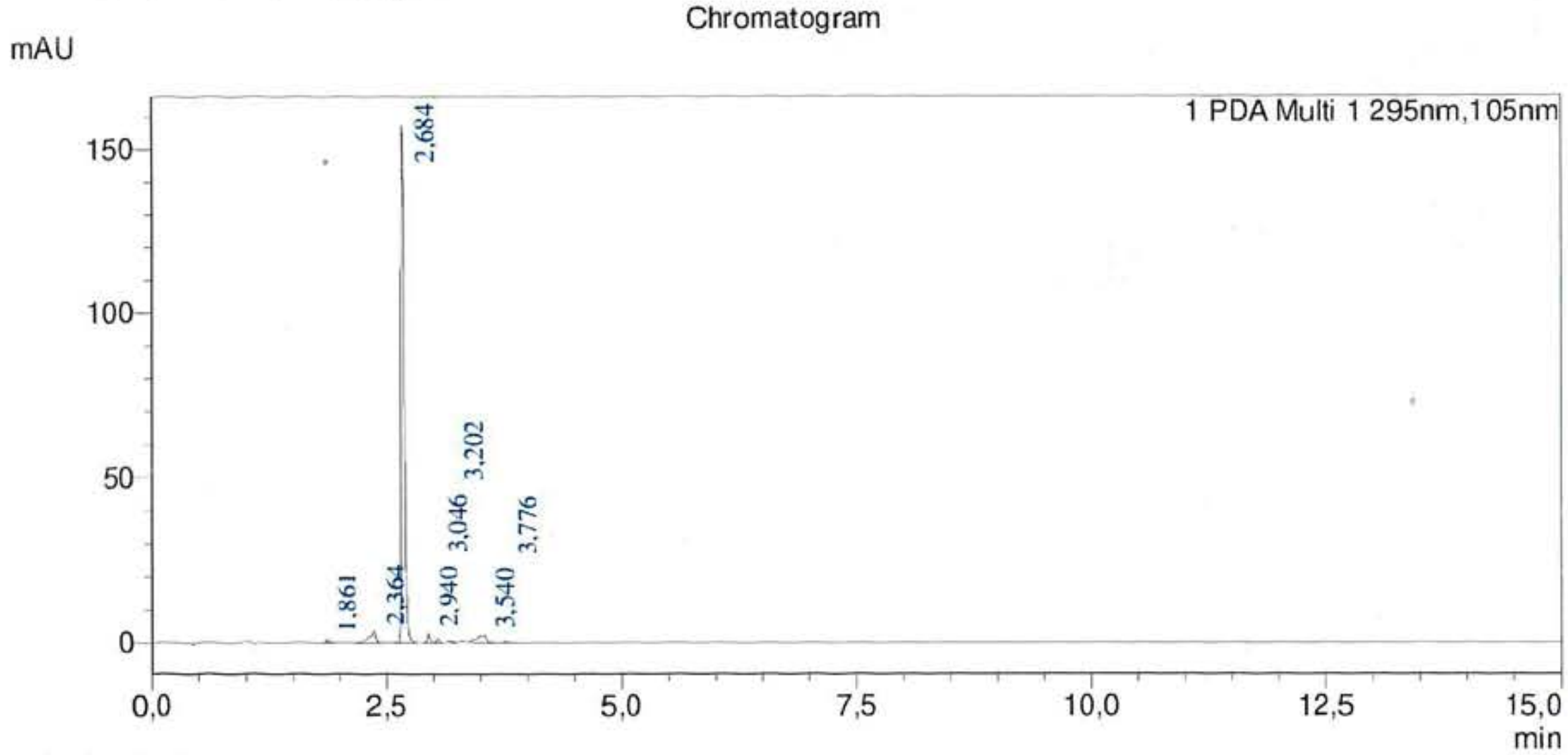


## <UV Spectrum >

UV Spectrum

| | |
|---|---|
| Peak# | : 3 |
| Retention Time | : 2,684 min |
| Spectrum Operation | : None |

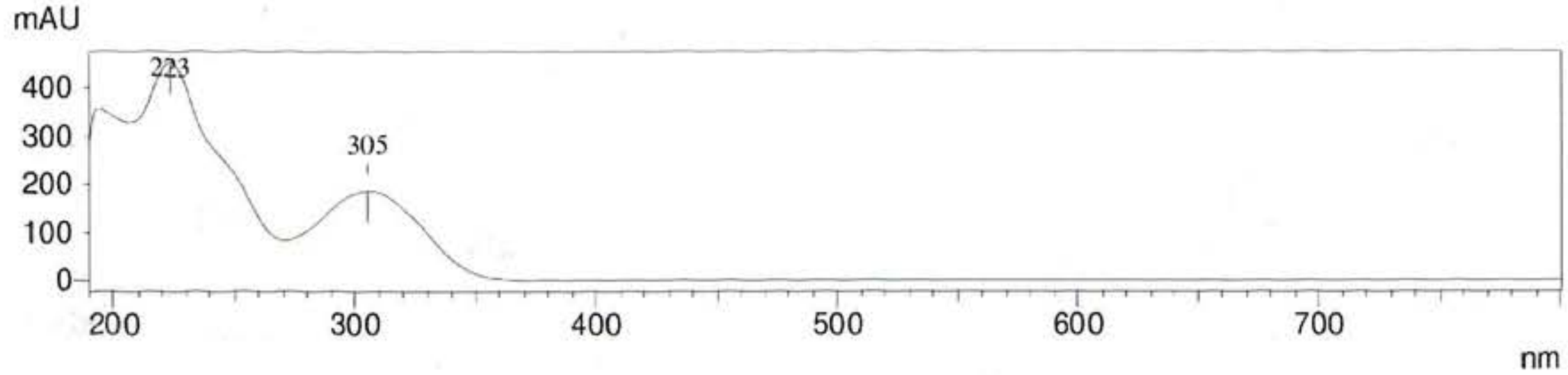

MS Instrument Parameter

<<MS Parameter>>

| | |
|---|---|
| Initial Valve Position | :0 |
| --Segment 1 Event 1-- | |
| Start Time | :0,00 min |
| End Time | :20,00 min |
| Acquisition Mode | :Scan |
| Polarity | :Positive |
| Event Time | :1,00 sec |
| Detector Voltage | :+1,10 kV |
| Threshold | :0 |
| Start m/z | :100,00 |
| End m/z | :1100,00 |
| Scan Speed | :1072 u/sec |
| Interface Volt. | :Use the Data in the Tuning File |
| DL Volt. | :Use the Data in the Tuning File |
| Qarray DC Voltage | :Use the Data in the Tuning File |
| Qarray DC Voltage | :Use the Data in the Tuning File |
| --Segment 1 Event 2-- | |
| Start Time | :0,00 min |
| End Time | :20,00 min |
| Acquisition Mode | :Scan |
| Polarity | :Negative |
| Event Time | :1,00 sec |
| Detector Voltage | :+1,10 kV |
| Threshold | :0 |
| Start m/z | :100,00 |
| End m/z | :1100,00 |
| Scan Speed | :1072 u/sec |
| Interface Volt. | :Use the Data in the Tuning File |
| DL Volt. | :Use the Data in the Tuning File |
| Qarray DC Voltage | :Use the Data in the Tuning File |
| Qarray DC Voltage | :Use the Data in the Tuning File |

<<Interface>>

| | |
|---|---|
| Interface | :DUIS |
| DL Temperature | :300 C |
| Nebulizing Gas Flow | :1,50 L/min |
| Heat Block | :400 C |
| Drying Gas | :On |
| | 18,00 L/min |

| Segment | Start Time | End Time | Ionization Mode |
|---|---|---|---|
| 1 | 0,00 | 20,00 | ESI&APCI |

## <HPLC-MS Chromatogram>

MS Chromatogram

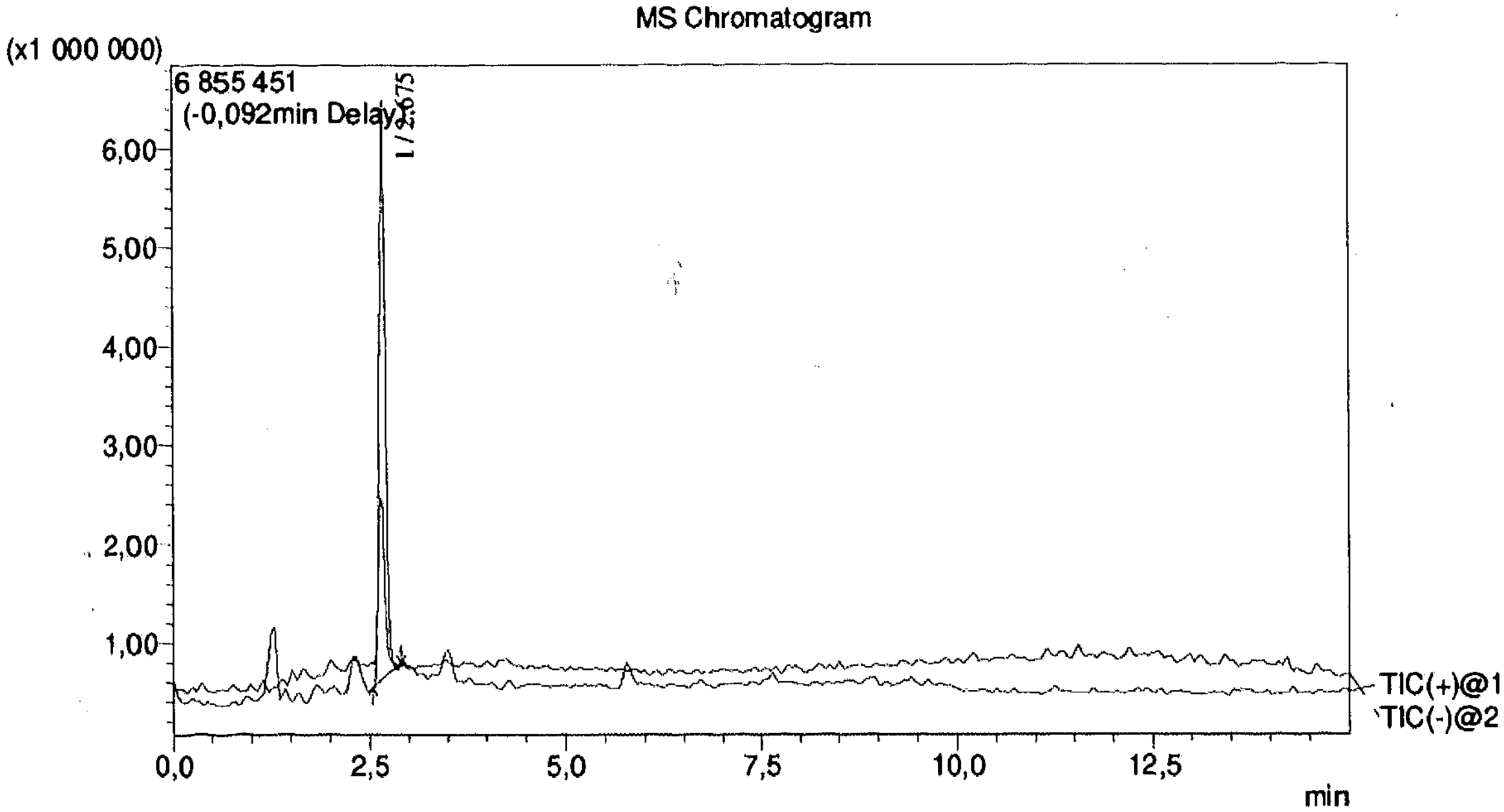

## < Mass spectrum>

MS Spectrum

Peak#:1 R.Time:2,675(Scan#:167)
MassPeaks:660
Spectrum Mode:Averaged 2,641-2,708(165-169)
BG Mode:Calc Segment 1 - Event 1

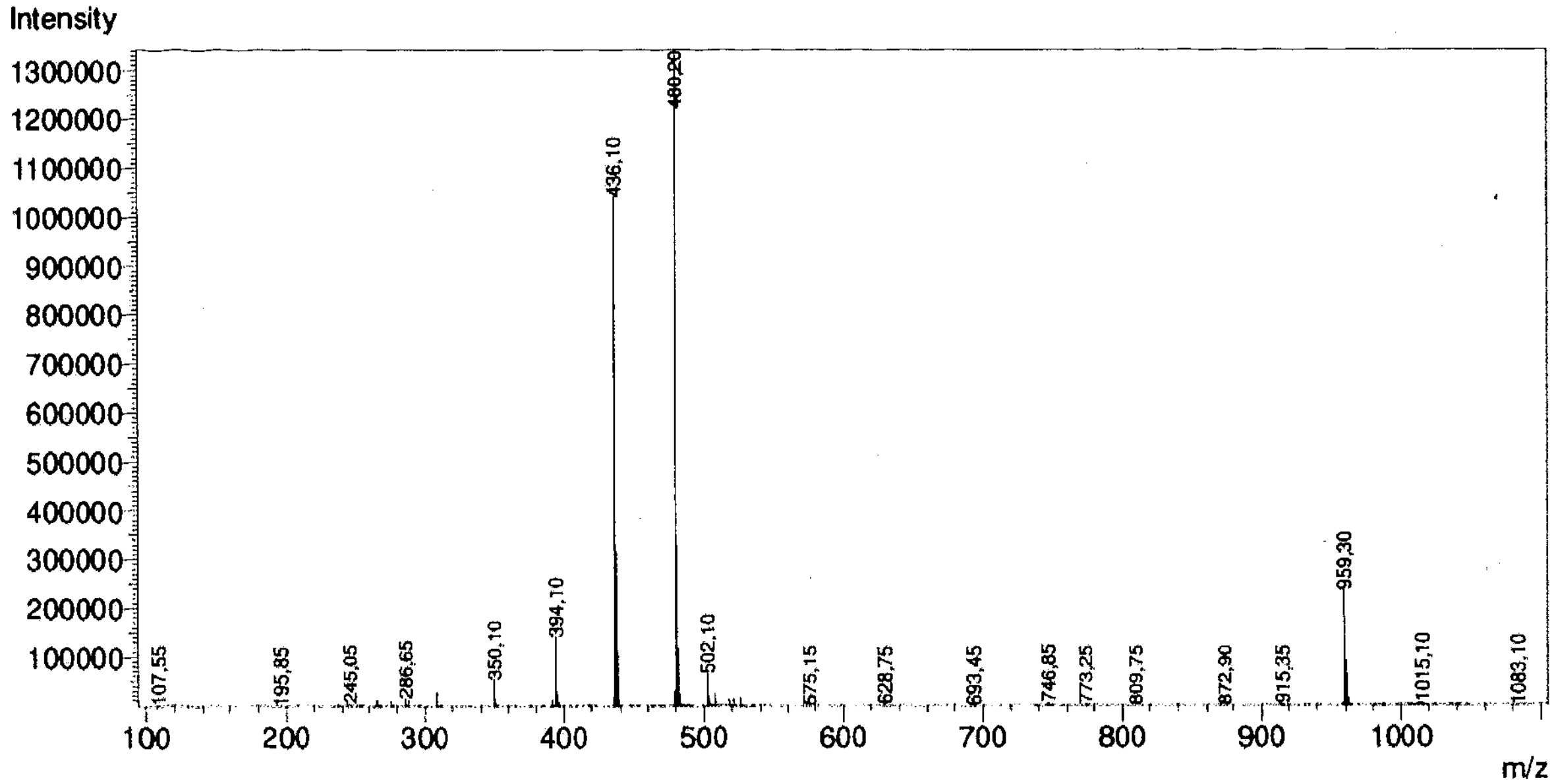


## < Mass Profile Spectrum>

MS Profile Spectrum

Peak#:1 R.Time:2,675(Scan#:167)
MassPeaks:660
Spectrum Mode:Averaged 2,641-2,708(165-169)
BG Mode:Calc Segment 1 - Event 1

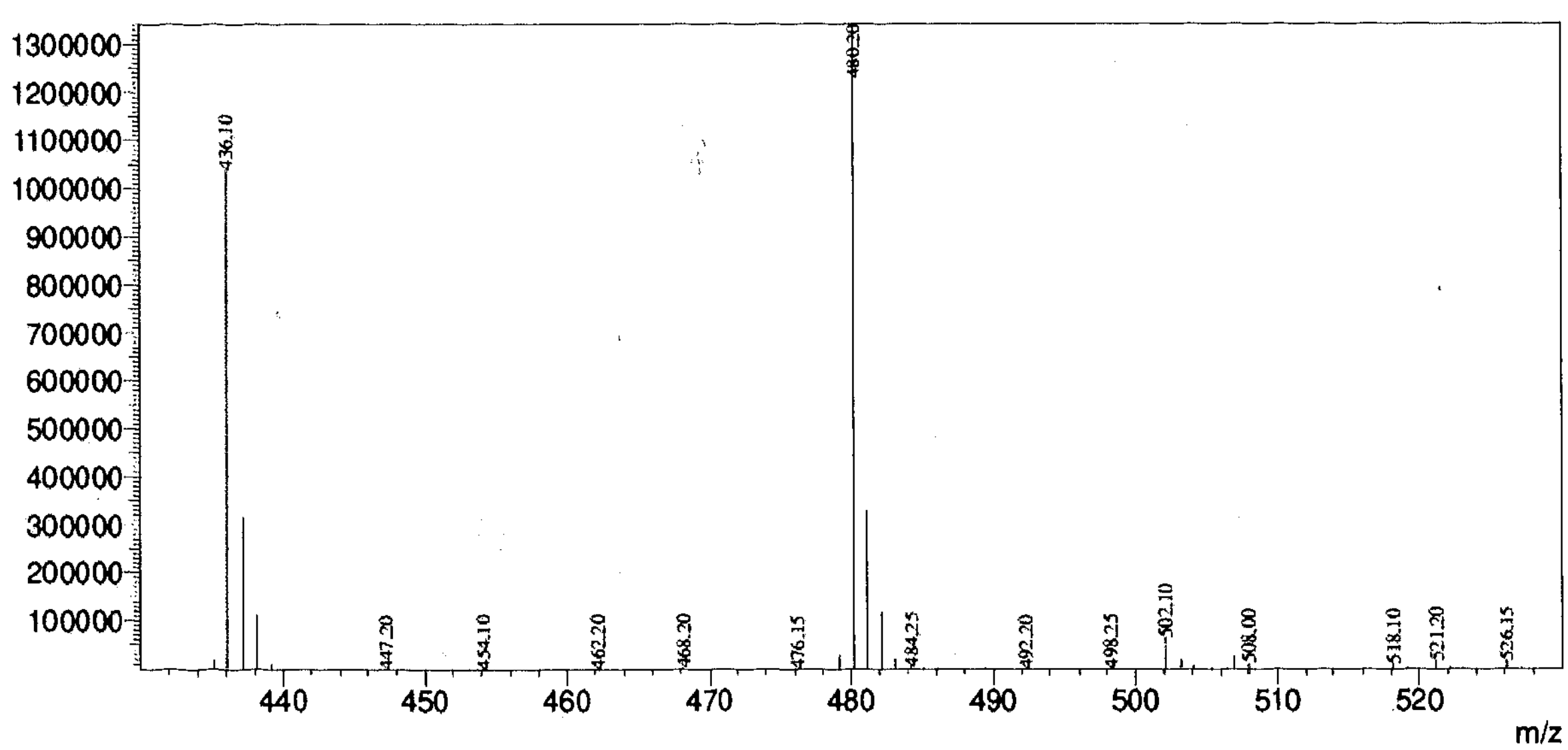

WOCKHARDT LIFE WINS

ANALYTICAL RESEARCH

## CERTIFICATE OF ANALYSIS FOR REFERENCE STANDARD

| **Name** | : | **Zidebactam (WCK 5107)** | | | |
|---|---|---|---|---|---|
| Molecular Formula | : | $C_{13}H_{21}N_5O_7S.2H_2O$ | Batch Number | : | WK1/WP-0060/016 |
| Molecular Weight | : | 427.45 | Laboratory Note Book Reference | : | WA-2004-038 |
| Date of Analysis | : | 30/03/2022 | Month of Manufacturing | : | May' 2019 |
| Valid Up To | : | 29/03/2023 | Reference Standard Number | : | RS/2022-013 |
| Effective Date | : | 23/04/2022 | Page Number | : | Page 1 of 4 |

| Sr. No. | Tests | Specifications | Results |
|---|---|---|---|
| 1 | Description | White to pale yellow powder | White powder |
| 2 | Identification (By IR, Mass & NMR Spectroscopy) | Spectral data should conform to the structure | Spectral data conforms the structure |
| 3 | Solubility | Freely soluble in water, Insoluble in dichloromethane | Freely soluble in water, Insoluble in dichloromethane |
| 4 | Constituted solution (10% w/v solution in WFI)<br>Completeness of solution<br><br>Clarity of solution | <br>The solid dissolves completely, leaving no visible residue as un-dissolved matter<br>The constituted solution is not significantly less clear than an equal volume of the WFI contained in a similar vessel and examined similarly | <br>Complies<br><br>Complies |
| 5 | pH of solution | Between 5.0 to 7.0 | 6.0 |
| 6 | Absorbance of solution (1.25 % w/v in water at 420 nm) | Not more than 0.2 AU | 0.01 AU |
| 7 | Water content (By KF Titration) | Not more than 12.0 % w/w | 8.7 % w/w |
| 8 | Specific optical rotation (On anhydrous basis) | Between –28° to -40° | -36° |
| 9 | Heavy metals | Not more than 20 μg/g | Less than 20 μg/g |
| 10 | Palladium content (By ICP-OES) | Not more than 10 μg/g | BLQ (LOQ 2.5 μg/g)* |

| | Prepared by | Checked by | Approved by |
|---|---|---|---|
| Name : | Mr. Sitaram Ubale | Mr. Rajendra Chavan | Mr Subhash Chandra Shukla |
| Department : | AVL | AVL | CRDQ |
| Sign : | [signature] | [signature] | [signature] |
| Date : | 12/04/2022 | 12/04/2022 | 13/04/2022 |

Format No. : AVL-045-F-02-00

Ranked # 1 in Healthcare
GREAT PLACE TO WORK® Best Companies to Work for 2015 India

ANALYTICAL RESEARCH

## CERTIFICATE OF ANALYSIS FOR REFERENCE STANDARD

| | | | | | |
|---|---|---|---|---|---|
| **Name** | : | **Zidebactam (WCK 5107)** | | | |
| Molecular Formula | : | $C_{13}H_{21}N_5O_7S.2H_2O$ | Batch Number | : | WK1/WP-0060/016 |
| Molecular Weight | : | 427.45 | Laboratory Note Book Reference | : | WA-2004-038 |
| Date of Analysis | : | 30/03/2022 | Month of Manufacturing | : | May' 2019 |
| Valid Up To | : | 29/03/2023 | Reference Standard Number | : | RS/2022-013 |
| Effective Date | : | 23/04/2022 | Page Number | : | Page 3 of 4 |

| Sr. No. | Tests | Specifications | Results |
|---|---|---|---|
| 13 | Sodium content (By ICP-OES) | Not more than 0.5 % w/w | BLQ (LOQ 0.25 % w/w)* |
| 14 | Sulfate content (By Ion chromatography) | Not more than 1.0 % w/w | BLQ (LOQ 0.626 % w/w) |
| 15 | Mono-methyl sulphate content (By Ion chromatography) | Not more than 0.15% w/w | Not detected |
| 16 | Polymorphism (By X-ray Diffraction) | The diffractogram should exhibit characteristic 2 theta values at 13.8, 15.6, 20.3 and 24.3 (± 0.2°) | The diffractogram exhibits characteristic 2 theta values at 13.8, 15.6, 20.3 & 24.3 |
| 17 | Potency (on as is basis) | Not applicable | 90.4 % |

BLQ = Below limit of quantification
BDL = Below disregard limit. Disregard limit 0.05 %
LOQ = Limit of quantification

* LOQ Values are based on analytical method verification report (Report nos. MVR/VP17053 and MVR/VP17054) of Analytical Solutions (CRO).

Specification Reference Number: **ZDB-DSS-112-05**

**Remark:** Potency is calculated on the basis of Total impurities, Water content, Residual solvents, Sulphate, Mono-methyl sulphate, Sodium and Palladium content.

**Storage Condition:** Store at 2°C to 8°C temperature.

| | Prepared by | Checked by | Approved by |
|---|---|---|---|
| Name : | Mr. Sitaram Ubale | Mr. Rajendra Chavan | Mr Subhash Chandra Shukla |
| Department : | AVL | AVL | CRDQ |
| Sign : | [signature] | [signature] | [signature] |
| Date : | 12/04/2022 | 12/04/2022 | 13/04/2022 |

Format No. : AVL-045-F-02-00

Ranked # 1 in Healthcare GREAT PLACE TO WORK Best Companies to Work for 2015 India

# Record of Decision – Interference Testing
# Study 21-0013: Ertapenem

**Memo Date:** November 10, 2022

RE: Interference Testing of Clinically Authorized Medications for Study 21-0013 (Ertapenem)

For this clinical study KCAS is developing several analytical LC-MS/MS assays for the detection and quantitation of ertapenem in plasma and urine and falls under the Bioanalytical Method Validation, FDA Guidance for Industry (May 2018) which states:

> *Potential interfering substances in a biological matrix include endogenous matrix components such as metabolites, decomposition products – and from the actual study – concomitant medications and other xenobiotics. If a stabilizer or enzyme inhibitor is used during sample collection, the sponsor should evaluate the potential for interference on the quantitation of the analyte. Sponsors should make a scientific judgment about the need to assess these (and any other) potential interferences during method development.*

The clinical trial has stringent screening and exclusion criteria but allows all volunteers to use acetaminophen (paracetamol) for temporary pain and headaches and allows women to continue using commercially available birth control.

An industry paper quoted de Zwart. M Lausecker *et al*, Co-medication and interference testing in bioanalysis: a European Bioanalysis Forum recommendation *Bioanalysis* 8(19), 2065-2070 (2016) contains a decision flow chart (Figure 1) that based on whether co-medication is scheduled or unscheduled and potential interference based on physical properties. The Figure 1 chart indicates that:

1. These inferring substances may not be considered scheduled co-medication because definition within the paper of “drugs which dose, time and route of administration are defined in the clinical study protocol”, however the CDC estimates that ~ 25% of women aged, age 18-49 years old use hormone containing contraceptive, so it is prudent to expect ~12.5% of clinical samples to contain one or more of the contraceptives listed below when adjusting for study enrollment of both genders.
2. Therefore, following the “Scheduled co-medication” decision tree the interference testing is not required because the significant differences in the molecular weights of the test analyte and the ConMeds (see table below).

Zidebactam MASS (MW) vs CONMEDS (source: www.ChemSpider.com)

| DRUG/Analyte | Formula | Avg. Mass | Monoisotopic Mass |
|---|---|---|---|
| Ertapenem | $C_{22}H_{25}N_3O_7S$ | 475.55 Da | 475.142327 Da |
| Zidebactam | $C_{13}H_{21}N_5O_7S$ | 391.400 Da | 391.116180 Da |
| Urea | $CH_4N_{20}$ | 60.0553 Da | 60.055 Da |
| Ethinylestradiol | $C_{20}H_{24}O_2$ | 296.403 Da | 296.177643 Da |
| Levonorgestrel | $C_{21}H_{28}O_2$ | 312.446 Da | 312.208923 Da |
| Etonogestrel | $C_{22}H_{28}O_2$ | 324.457 Da | 324.208923 Da |
| Norethindrone | $C_{20}H_{26}O_2$ | 298.419 Da | 298.193268 Da |
| Drospirenone | $C_{24}H_{30}O_3$ | 366.493 Da | 366.219482 Da |
| Paracetamol | $C_8H_9NO_2$ | 151.163 Da | 151.063324 Da |


*These items are controlled by the U.S. Government and authorized for export only to the country of ultimate destination for use by the ultimate consignee or end-user(s) herein identified. They may not be resold, transferred, or otherwise disposed of, to any other country or to any person other than the authorized ultimate consignee or end-user(s), either in their original form or after being incorporated into other items, without first obtaining approval from the U.S. Government or as otherwise authorized by U.S. law and regulations.*

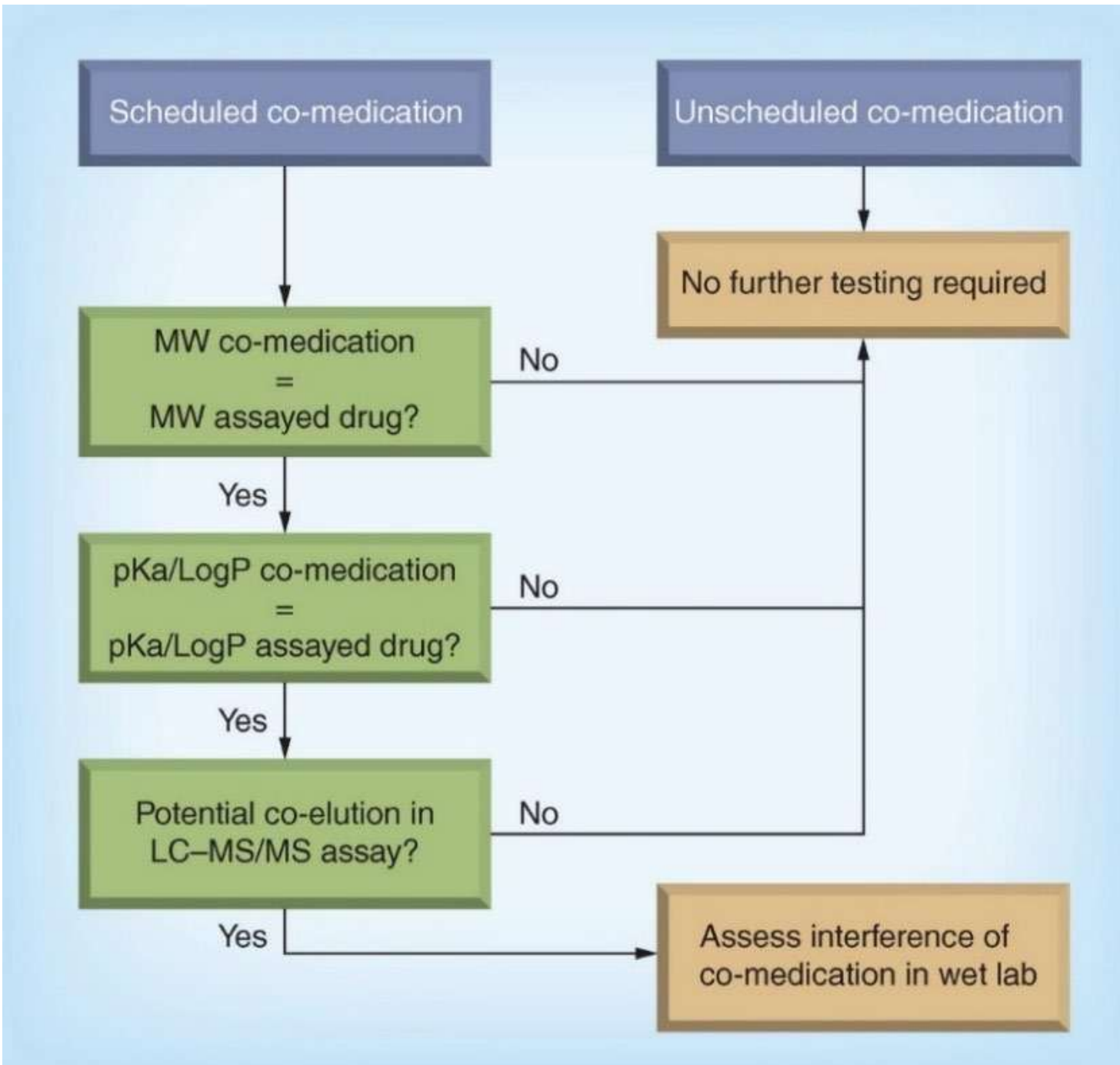


**Figure 1.** Recommendation for scheduled and unscheduled co-medication testing for LC–MS/MS-based assays.

Therefore, based on both sides of the decision tree in Figure 1, further interference testing other than the evaluation of the planned co-administered drug, zidebactam, is not required.

Decision issued by:

______________________________________________

Matthew E. Hinz, M.S. Sr. Manager
DVC LLC, A GDIT Company


*These items are controlled by the U.S. Government and authorized for export only to the country of ultimate destination for use by the ultimate consignee or end-user(s) herein identified. They may not be resold, transferred, or otherwise disposed of, to any other country or to any person other than the authorized ultimate consignee or end-user(s), either in their original form or after being incorporated into other items, without first obtaining approval from the U.S. Government or as otherwise authorized by U.S. law and regulations.*
