# Supplementary material for: A Phase I, randomized, double-blind, placebo-controlled, dose escalation study to evaluate the safety, tolerability, and pharmacokinetics of intravenous ertapenem and zidebactam in combination (WCK 6777) in healthy adult subjects
Source: Antimicrob Agents Chemother. 2026 Sep 29;70(11):e00390-26. doi: 10.1128/aac.00390-26 (PMC13731237; doi:10.1128/aac.00390-26)
Supplement: Supplementary file 2 [file aac.00390-26-s0002.pdf]

1. TITLE: THE DETERMINATION OF FREE ERTAPENEM IN TREATED HUMAN PLASMA ULTRAFILTRATE, WITH SODIUM HEPARIN AS ANTICOAGULANT, BY LC-MS/MS

BIOANALYTICAL METHOD VALIDATION REPORT

Bioanalytical Project Number:	V1872106P3
Sponsor:	DynPort Vaccine Company, LLC<br>110 Thomas Johnson Drive<br>Suite 200<br>Frederick, MD 21702
Bioanalytical Laboratory:	KCAS Bio<br>10830 S Clay Blair Boulevard<br>Olathe, KS 66061
Issue Date:	February 2023
Amendment 1 Issue Date:	June 2023
Amendment 2 Issue Date:	Date of PI Signature

2. SYNOPSIS

This report presents the validation data for an LC-MS/MS method for the determination of free ertapenem in treated human plasma ultrafiltrate, with sodium heparin as anticoagulant. The assay was analyzed in the range of 0.0500 to 50.0 mcg/mL and is for use on treated human plasma ultrafiltrate samples obtained during clinical trials.

AMENDMENT OUTLINE FORM

1. Amendment Number 1	This amendment was created to report:<br>• Long-term matrix stability at -70 °C for up to 186 days (with comed[2]) and 187 days (without comed[2]) for free ertapenem in treated human plasma ultrafiltrate, with sodium heparin as anticoagulant with and without the presence of comed[2].
2. Amendment Number 2	This amendment was created to report:<br>• Platform change (API5000 vs API6500).

3. TABLE OF CONTENTS FOR THE BIOANALYTICAL METHOD VALIDATION REPORT

1. TITLE: THE DETERMINATION OF FREE ERTAPENEM IN TREATED HUMAN PLASMA ULTRAFILTRATE, WITH SODIUM HEPARIN AS ANTICOAGULANT, BY LC-MS/MS 1
2. SYNOPSIS 2
3. TABLE OF CONTENTS FOR THE BIOANALYTICAL METHOD VALIDATION REPORT 3
4. LIST OF TABLES 4
5. SIGNATURE PAGE 5
6. QUALITY ASSURANCE STATEMENT 6
7. STUDY COMPLIANCE STATEMENT 7
8. LIST OF ABBREVIATIONS AND DEFINITIONS OF TERMS 8
9. AMENDMENT 10
10. EXPERIMENTAL DESIGN AND RESULTS 10
10.1. Reference Standards 10
10.2. Calibration Standards and QC Samples Preparation 10
10.3. Carryover 11
10.4. Platform Change 11
10.5. SOP Deviations and Investigations 11
11. CONCLUSION 11
12. PERTINENT SOPS 11
13. ARCHIVES 12
14. REFERENCES 12
15. TABLES 13

4. LIST OF TABLES

Table 1: Abbreviations and Codes 8
Table 2: Mass Spec Transitions 13
Table 3: Reference Standards 13
Table 4: Calibration Standards and QC Samples Preparation 14
Table 5: Matrix Description 14
Table 6: Summary of Results and Conclusions 15
Table 7: Summary of Validation Run Objectives and Outcome 17
Table 8: Carryover 17
Table 9: Platform Change 18

Test Procedure ....Annex 4

5. SIGNATURE PAGE

Bioanalytical Project Number: V1872106P3

Amendment 2 Issue Date: Date of PI Signature

APPROVED

DocuSigned by:

Anika Pippin

Signer Name: Anika Pippin
Signing Reason: I approve this document
Signing Time: 27-Jun-2024 | 17:35 CDT
BE3AA1198E40459E8A7E3C615D981D82

Anika Pippin, B.S.
Principal Investigator

REVIEWED

DocuSigned by:

Marsha Luna

Signer Name: Marsha Luna
Signing Reason: I have reviewed this document
Signing Time: 27-Jun-2024 | 16:58 CDT
C9CC738561AF4CC1B375ED013DD1E4BD

Marsha Luna, B.S.
Management

6. QUALITY ASSURANCE STATEMENT

This Bioanalytical Method Validation Report has been found to describe accurately the methods and SOPs used and to accurately reflect the raw data collected. The Quality Assurance Unit initiated audits on this study and reported the findings to the Principal Investigator (PI) and Management as follows:

		Date(s) Sent to:	
**Inspection Date(s)**	**Inspection Phase**	**PI**	**Management**
19Feb2024	Data Audit	19Feb2024	19Feb2024
24Jun2024	Draft Report Audit	24Jun2024	24Jun2024
26Jun2024	Final Report Review	26Jun2024	26Jun2024

REVIEWED

DocuSigned by:
Stephanie Bryan
Signer Name: Stephanie Bryan
Signing Reason: I have reviewed this document
Signing Time: 27-Jun-2024 | 16:55 CDT
8DCA3FD0EF7844F0A60EBB4A85B9FD67

Stephanie Bryan, B.S.
Quality Assurance Unit

7. STUDY COMPLIANCE STATEMENT

I, the undersigned, hereby declare that this study was conducted in compliance with the relevant bioanalytical aspects of FDA Guidance for Industry on Bioanalytical Methods Validation, May 2018 [1] and KCAS Bio (KCAS) procedures and that the work was performed by me, or under my direction, and the report reflects a true and accurate record of the results obtained.

The acceptance criteria are based on the FDA Guidance for Industry, Bioanalytical Method Validation (2018). [1]

APPROVED

DocuSigned by:
Anika Pippin
Signer Name: Anika Pippin
Signing Reason: I approve this document
Signing Time: 27-Jun-2024 | 17:35 CDT
BE3AA1198E40459E8A7E3C615D981D82

Anika Pippin, B.S.
Principal Investigator

8. LIST OF ABBREVIATIONS AND DEFINITIONS OF TERMS

Table 1: Abbreviations and Codes

Abbreviation	Definition
<	Concentration less than the lowest acceptable calibration standard
>	Concentration exceeded the upper limit of calibration range (> ULOQ)
A	Amendment
A&P	Accuracy and Precision
AQL	Above Quantitation Limit
ARQC	Above Range Quality Control
Batch	Is the same as Run
BQL	Below Quantifiable Limit
Cal	Calibration standard
Conc.	Concentration
Conv.	Conversion
%CV	Coefficient of Variation
dev	Deviation
diff	Difference
Dil	Dilution
EMA	European Medicines Agency
FDA	Food and Drug Administration
GLP	Good Laboratory Practice
HPLC	High Performance Liquid Chromatography
ID	Identification
ICH	International Council for Harmonisation of Technical Requirements for Registration of Pharmaceuticals for Human Use
IS	Internal Standard
ISR	Incurred Sample Reanalysis
K_2EDTA or K_3EDTA	Dipotassium or Tripotassium Ethylenediaminetetraacetic Acid
KCAS	KCAS Bio
LC-MS/MS	Liquid Chromatography Tandem Mass Spectrometry
LLOQ	Lower Limit of Quantitation

Table 1: Abbreviations and Codes (Continued)

Abbreviation	Definition
mcg or µg or ug	Microgram
mcL or µL or uL	Microliter
ME	Matrix Effect
MF	Matrix Factor
mL	Milliliter
MS	Mass Spectrometry
n	number
N.C.	Not Calculated
ND	No difference reported as ISR will be counted
ng	nanogram
n.report	No Data available to report
N/A or NA	Not Applicable
N.R.	Not Reported
p.a.r.	Peak Area Ratio
PI	Principal Investigator
QC	Quality Control Sample
QCs	Quality Control Samples
OECD	Organization for Economic Co-operation and Development
RT	Room Temperature
sd or SD	Standard deviation
Seq	Sequence
SOP (s)	Standard Operating Procedure (s)
temp	Temperature
TP	Test procedure
UHPLC	Ultra High Performance Liquid Chromatography
ULOQ	Upper Limit of Quantitation
WAS	Working Analyte Standard
Watson LIMS 7.5	Watson Laboratory Information Management System, Version 7.5, Service Pack 1
WIS	Working Internal Standard

This is a basic list. Some of the abbreviations may not have been used in this report.

9. AMENDMENT

This amendment was created to report platform change (API5000 vs API6500) results.

The validation procedure was designed to meet or exceed the requirements of FDA Guidance for Industry on Bioanalytical Methods Validation, May 2018. The software programs used were Microsoft Office Excel 365, acquisition and processing software Analyst 1.7.2, and data management software Watson LIMS 7.5.

10. EXPERIMENTAL DESIGN AND RESULTS

In this method, ertapenem and internal standard, ertapenem-d_4 (IS), were released from human plasma ultrafiltrate using 96-well protein precipitation. After extraction, the sample was chromatographed using reversed phase HPLC with a Fortis Technology Ltd. Fortis C18 analytical column. Ertapenem and ertapenem-d_4 (IS) were detected by monitoring the precursor and product ions described in Table 2 using an Applied Biosystems Sciex API 5000 LC-MS/MS. The quantification was based on setting a calibration graph using the internal standard method based on bioanalytical test procedure TP-80238, entitled "LC-MS/MS Analysis of Free Ertapenem in Treated Human Plasma Ultrafiltrate (Sodium Heparin, 100 mcL, (0.0500 – 50.0 mcg/mL; Prior to MES Treatment)". The test procedure can be found in Annex 4.

Human plasma ultrafiltrate, screened for the lack of interference at the retention times of ertapenem and ertapenem-d_4 (IS), was diluted 1:1 with MES buffer, pH 6.4 for stabilization. Since the standards and QCs concentrations were already adjusted for the addition of MES during the preparation and are reported based on plasma ultrafiltrate volume only, the obtained free ertapenem concentration of the unknown study samples do not need to be adjusted for the dilution with MES buffer.

A summary of the validation results is provided in Table 6. A summary describing the objective(s) and outcome of each validation run attempted is provided in Table 7.

The method was validated within the analytical range of 0.0500 to 50.0 mcg/mL in human plasma ultrafiltrate. No development work was performed after the method was validated.

10.1. Reference Standards

Reference standards used during this validation are presented in Table 3.

10.2. Calibration Standards and QC Samples Preparation

Calibration standards and quality control pools were prepared by spiking appropriate levels of ertapenem into treated human plasma ultrafiltrate, with sodium heparin as anticoagulant. Primary reference standards were prepared in at least duplicate and compared within 5% of each other. Standards and QCs were prepared and stored frozen at KCAS at -70 °C. Standards, QCs,

and the critical solutions used during this validation are described in Table 4, and matrix used is described in Table 5.

10.3. Carryover

Carryover is evaluated within each run. It consists of measuring the response for each analyte and if it exceeds 20% of the response of LLOQ standard or other sample reflecting the lower sensitivity limit required, a carryover assessment is completed, and affected samples are rejected. The internal standard carryover evaluation is assessed within each run and if it exceeds 5% of the average internal standard response of acceptable standards and QCs, impact is evaluated within the run. Data are presented in Table 8.

10.4. Platform Change

Platform change was assessed by injecting run 1 on a different MS platform (Applied Biosystems API6500).

The maximum allowable percent deviation from nominal for each standard was ± 15%, except at the LLOQ, where ± 20% was accepted. Any standard deviating by more than these limits was required to be excluded from the regression line. For the calibration curve to be accepted, at least 75% of the initial number of standards was required to remain in the curve. In addition, for accuracy and precision (A&P) runs, the criteria (within ± 15% or ± 20% for LLOQ) should also be fulfilled for at least 50% of the calibration standards tested per concentration level. For the QCs the accuracy at each concentration level should be within ± 15% of the nominal concentration, except at the LLOQ, where it should be within ± 20%. The precision (%CV) of the concentrations determined at each level should not exceed 15%, except at the LLOQ, where it should not exceed 20%. Sensitivity QCs also need to have a signal to noise of at least 5 to be considered an acceptable platform to be used.

Data are presented in Table 9 for the precision/accuracy portion of this experiment.

10.5. SOP Deviations and Investigations

No process deviation occurred during this study. No investigations were conducted during this study.

11. CONCLUSION

Platform change (API5000 vs API6500) has been proven.

12. PERTINENT SOPS

The following SOPs were followed during the conduct of this study:

SOP-00073 [05] - Bioanalytical Method Validation - Chromatographic Assays

SOP-00035 [02] - Procedures, Reporting, and Acceptance Criteria for Bioanalytical Chromatographic Projects

13. ARCHIVES

All raw data, including paper and electronic files associated with this study will remain archived at a location specified by KCAS, 10830 S Clay Blair Boulevard, Olathe, KS 66061 for a period of at least 10 years, after which the client may be contacted to arrange for continued storage of the files, or disposition according to an agreed procedure. KCAS will maintain an electronic (scanned/true) copy of the method validation report that can be viewed and printed on site. The study plan, documentation, raw data, and final report are indexed under Bioanalytical Project Number V1872106P3A2.

14. REFERENCES

1. Guidance for Industry: Bioanalytical Method Validation. U.S. Department of Health and Human Services. Food and Drug Administration. Center for Drug Evaluation and Research. Center for Veterinary Medicine. May 2018.
2. Comeds: QCs in the presence of 150 mcg/mL zidebactam.

15. TABLES

Table 2: Mass Spec Transitions

Analyte	Transition
Ertapenem	m/z 476.047→431.9
Ertapenem-d_4 (IS)	m/z 480.2→436.3

Table 3: Reference Standards

Analyte	Supplier	Lot/Batch Number	Purity	Expiration or Retest Date	Conv. Factor	Storage Condition
Ertapenem Sodium	MedChemExpress	229333	98.43%	Retest 29Sep2025	1.046	2-8 °C
Ertapenem-d_4 Sodium Salt (IS)	AlsaChim	DH-ALS-20-002-P2	100%*	Expire 29Apr2023**	1.000*	-20 °C

Certificates of analysis for the above reference standards are on file at KCAS Bio.
*Used as is with 1.000 conversion and 100% purity as IS only.
**Reference material was within retest at the time of use.

Table 4: Calibration Standards and QC Samples Preparation

<table>
<tr><th>Critical Solutions</th><th>Lot # for Standards (Prep Date)</th><th>Lot # for QCs (Prep Date)</th><th>Standards Lot (Prep Date)</th><th>QCS Lot (Prep Date)</th><th>Runs used</th></tr>
<tr><td>Treated Sodium Heparin Human Plasma Ultrafiltrate</td><td colspan="2">BioIVT, Lot V1872106P4-29-2</td><td rowspan="3">V1872106P3A1-14-1
Set C
(16May2023)</td><td rowspan="3">V1872106P3A1-15-1
Set C
(16May2023)</td><td rowspan="3">1</td></tr>
<tr><td>MES Buffer, pH 6.4 stored frozen (-20°C) in polypropylene</td><td>V1872106P3A1-7-1
(15May2023)</td><td>V1872106P3A1-7-1
(15May2023)</td></tr>
<tr><td>Human Plasma Ultrafiltrate Treated with MES Buffer, ~0.1M, pH 6.4 (1:1) stored frozen (-70°C) in polypropylene</td><td>V1872106P3A1-13-1
(16May2023)</td><td>V1872106P3A1-13-1
(16May2023)</td></tr>
</table>

Table 5: Matrix Description

Matrix	Source Lot #	Date Received
Sodium Heparin Human Plasma Ultrafiltrate V1872106P4-29-2	BioIVT, Lot HMN790516	Apr2022
Sodium Heparin Human Plasma Ultrafiltrate V1872106P4A1-3-1	BioIVT, Lot HMN790518	Apr2022

Table 6: Summary of Results and Conclusions

**Methodology – TP-80238**	**Method Performance – LC-MS/MS**	**Hyperlink – N/A**	**Comments**
Matrix	Plasma ultrafiltrate		
Anticoagulant	Sodium heparin		
Assay Volume	100 mcL		
**Selectivity**			
Analyte	Ertapenem		V1872106P3
Internal Standard	Ertapenem-d_4 (IS)		V1872106P3
Matrix Effect<br>(6 spiked matrix lots)	Complies		V1872106P3
Interference	Complies		V1872106P3
Carryover	Assessed with every run with standards and QCs	Summary tables<br>Table 8<br><br>Report text<br>Section 10.3	
**Linearity**			
Weighting	$1/x^2$		V1872106P3
Bias at LLOQ	-0.6%		V1872106P3
Bias above LLOQ	-4.4 to 3.0%		V1872106P3
Bioanalytical Range	Complies<br>0.0500 to 50.0 mcg/mL		V1872106P3
**Precision**			
Intra-assay	5.5 to 10.2% (LLOQ);<br>1.0 to 5.2% (above LLOQ)		V1872106P3
Inter-assay	16.0% (LLOQ);<br>4.3 to 9.0% (above LLOQ)		V1872106P3
**Accuracy**			
Intra-assay	-15.6 to 13.4% (LLOQ);<br>-11.3 to 4.7% (above LLOQ)		V1872106P3
Inter-assay	-5.2% (LLOQ);<br>-7.5 to -6.0% (above LLOQ)		V1872106P3
**Large Run Size**	Determined to be 158 samples.		V1872106P3

Table 6: Summary of Results and Conclusions (Continued)

Methodology – TP-80238	Method Performance – LC-MS/MS	Hyperlink – N/A	Comments
**Dilution**			
Dilution factor: 2x	Dilution QC: 37.5 mcg/mL<br>Precision: 2.1%<br>Accuracy: -0.8%		V1872106P3
Dilution factor: 5x	Dilution QC: 200 mcg/mL<br>Precision: 2.7%<br>Accuracy: -2.0%		V1872106P3
**Stability**			
Matrix (in polypropylene tubes)			
Freeze/Thaw (-20 °C/ice-water bath)	Demonstrated instability at 3 cycles		V1872106P3
Freeze/Thaw (-70 °C/ice-water bath) with and without the presence of comed[2]	Demonstrated for 3 cycles		V1872106P3
Ice-Water Bath	Demonstrated for 24 hours		V1872106P3
Long-term (-20 °C) with and without the presence of comed[2]	Demonstrated instability at 28 days without comed[2]<br>Demonstrated instability at 27 days with comed[2]		V1872106P3
Long-term (-70 °C) with and without the presence of comed[2]	Demonstrated for 187 days without comed[2]<br>Demonstrated for 186 days with comed[2]		V1872106P3A1
Processed Matrix polypropylene			
Refrigeration	Demonstrated for 5 days 22 hours		V1872106P3
**Individual Sample Reinjection**	Reproducible		V1872106P3
**Whole Run Reinjection Integrity**	Reproducible<br>Duration 4 days 11 hours		V1872106P3
**Column Ruggedness**	Reproducible		V1872106P3

Table 6: Summary of Results and Conclusions (Continued)

Methodology – TP-80238	Method Performance – LC-MS/MS	Hyperlink – N/A	Comments
**Platform Change (API50000 vs API6500)**	Reproducible	Summary tables Table 9<br><br>Report text Section 10.4	
**Extraction Recovery**			
Ertapenem	Overall 91.1%		V1872106P3
Ertapenem-d_4 (IS)	Overall 134.5%		V1872106P3
**Solution Stability**			
Solution, Primary polypropylene			
Ertapenem in MES buffer (RT)	Stable 6.75 hours		V1872106P5
Ertapenem in MES buffer (-20 °C)	Stable 7 days		V1872106P5
Ertapenem in MES buffer (-20 °C)	Instability observed at 28 days		V1872106P3
Ertapenem-d_4 (IS) in MES buffer (RT)	Taken to be same as ertapenem		
Ertapenem-d_4 (IS) in MES buffer (-20 °C)	3 months stability can be assigned per SOP unless low sensitivity is observed		SOP-00073

Table 7: Summary of Validation Run Objectives and Outcome

Run ID	Instrument ID	Extraction Date	Assay Date	Run Objective(s)	Accepted or Rejected	Comment
1	LC/MS-31	21Aug2023	22Aug2023	Platform Change (API5000 vs API6500)	Accepted	

Table 8: Carryover

Run ID	Result (Extracted Blank after High Standard)	Carryover Results for Internal Standard
1	Seq 13 and Seq 50 are <20.0% of the LLOQ.	Seq 4 and Seq 41 are < 5.0% internal standard peak of acceptable standards.

Note: Calibration Standards and QCs are arranged in a way to mitigate carryover in all validation runs.

Table 9: Platform Change

Analytical Performance: Back-Calculated Concentrations (µg/mL) of Ertapenem Calibration Standard in (Human) (Plasma Ultrafiltrate) in (Study V1872106P3 A2)

Assay Date	Run ID	Cal 0.05 0.0500 µg/mL	Cal 0.1 0.100 µg/mL	Cal 1 1.00 µg/mL	Cal 5 5.00 µg/mL	Cal 10 10.0 µg/mL	Cal 20 20.0 µg/mL	Cal 40 40.0 µg/mL	Cal 50 50.0 µg/mL
22-Aug-2023	1	0.0476	0.111	1.01	5.31	10.7	19.1	36.8	51.9
		0.0531	0.0855	0.965	5.58	10.5	19.2	38.2	46.1
Mean		0.0504	0.0983	0.988	5.45	10.6	19.2	37.5	49.0
%Bias		0.8	-1.7	-1.2	9.0	6.0	-4.0	-6.3	-2.0

Calibration Curve Parameters for Ertapenem Calibration Standards in Human Plasma Ultrafiltrate (Study V1872106P3 A2)

Run Date	Run ID	Slope	Intercept	R-Squared	LLOQ	ULOQ
22-Aug-2023	1	0.27848	0.00163	0.9930	0.0500	50.0

Human Plasma Ultrafiltrate Ertapenem Concentrations in Quality Controls for 1 separate batch runs (Study V1872106P3 A2)

Run Date	Run ID	QCS 0.05 0.0500 µg/mL	%Bias	QCS 0.15 0.150 µg/mL	%Bias	QCS 25 25.0 µg/mL	%Bias	QCS 37.5 37.5 µg/mL	%Bias
22-Aug-2023	1	0.0455	-9.0	0.147	-2.0	24.8	-0.8	36.0	-4.0
		0.0470	-6.0	0.154	2.7	23.7	-5.2	34.0	-9.3
		0.0532	6.4	#0.124	-17.3	24.4	-2.4	32.3	-13.9
		0.0501	0.2	0.141	-6.0	25.0	0.0	32.8	-12.5
		0.0493	-1.4	0.132	-12.0	24.9	-0.4	32.5	-13.3
		0.0506	1.2	0.136	-9.3	21.8	-12.8	33.1	-11.7
Mean		0.0493		0.139		24.1		33.5	
SD		0.00273		0.0107		1.22		1.38	
%CV		5.5		7.7		5.1		4.1	
%Bias		-1.4		-7.3		-3.6		-10.7	
n		6		6		6		6	
# > 15%Bias									

Note: Run 1 is injected on a different MS platform (Applied Biosystems API5000 vs API6500).

Criteria: Data were required to meet the accuracy and precision criteria outlined in Section 10.4 to be the considered as reproducible/rugged method.

Results: The acceptance criteria were met.

1. GENERAL INFORMATION

1.1. Client Name: Dynport Vaccine Company

1.2. Reference: Validation V1872106P3

2. TABLE OF CONTENTS

1. GENERAL INFORMATION V1872106P3 1
1.1. Client Name: Dynport Vaccine Company 1
1.2. Reference: Validation V1872106P3 1
2. TABLE OF CONTENTS 1
3. SAMPLE REQUIREMENTS 3
4. METHOD SUMMARY 3
5. SPECIAL HANDLING OF BIOLOGICAL SAMPLES 3
6. DATA COLLECTION AND ANALYSIS SOFTWARE 3
7. INSTRUMENTS, EQUIPMENT, AND CONDITIONS 4
7.1. LC and MS Conditions 4
Table 1: HPLC System 4
7.1.1. Gradient Table 5
7.1.2. Autosampler Settings 5
7.1.3. Mass Spectrometer System 5
8. REAGENTS, STANDARDS, AND SUPPLIES 7
Table 2: Reagents, Standards, and Supplies 7
9. SAFETY 7
10. PROCEDURE 8
10.1. Preparation of Analyte Stock Solutions and Dilutions 8

CONFIDENTIAL

Serial Number: 0cfbae28-298d-4a49-9fe8-26a0ef3e0e64 accessed by Anika Pippin (PIPPINA) on Wed, 19 Jun 2024 13:45:54 GMT.

10.1.1. Ertapenem Primary Solution, 2.00 mg/mL (Prepare on wet ice) 8
10.1.2. Working Internal Standard, ~5.0 mcg/mL Ertapenem-d_4 (Prepare on wet ice) 8
10.2. Preparation of Other Reagents 8
10.2.1. Mobile Phase A: 0.5% Formic Acid in Deionized Water 8
10.2.2. Mobile Phase B: 90/10 Acetonitrile / Deionized Water 8
10.2.3. Rinse: 90/10 Acetone/Deionized Water 9
10.2.4. 1 N Sodium Hydroxide (NaOH) in Deionized Water 9
10.2.5. MES (2-(N-Morpholino) Ethanesulphonic Acid Hydrate) Buffer pH 6.4, ~0.1M 9
10.2.6. 5/95 Acetonitrile/Deionized Water 9
10.3. Preparation of Standards and QC Samples 9
10.3.1. Ertapenem Secondary Solution, 100 mcg/mL in Treated Human Plasma Ultrafiltrate (Reported as 200 mcg/mL; Prepare on wet ice) 9
10.3.2. Treated Human Plasma Ultrafiltrate (Ultrafiltrate: MES Buffer 1:1) 9
10.3.3. Preparation of Standards and QC Samples in Human Plasma Ultrafiltrate 10
10.3.4. Ultrafiltration of Human Plasma Samples using Centrifugal Filters 10
10.4. Extraction Procedure 11
10.5. Dilutions 11
10.6. Calculations 12
11. REFERENCE CHROMATOGRAMS 12
Figure 1: Blank with IS 12
Figure 2: LLOQ – Ertapenem 0.050 mcg/mL 13
Figure 3: ULOQ – Ertapenem 50.0 mcg/mL 13
12. REVISION HISTORY 14
Table 3: Revision History 14

CONFIDENTIAL

Serial Number: 0cfbae28-298d-4a49-9fe8-26a0ef3e0e64 accessed by Anika Pippin (PIPPINA) on Wed, 19 Jun 2024 13:45:54 GMT.

3. SAMPLE REQUIREMENTS

Ultrafiltered human sodium heparin plasma samples (treated with 1:1 MES Buffer, pH 6.4, ~0.1M) minimum volume 100 mcL each, for determination of free ertapenem concentration.

Refer to the relevant study protocol for a description of expected samples.

4. METHOD SUMMARY

The method involves 96-well protein precipitation followed by chromatographic separation and tandem MS detection using the Sciex 5000 LC/MS/MS system. The concentration range is 0.0500 – 50.0 mcg/mL in human plasma ultrafiltrate only. Since unknown samples are treated with the MES buffer after collection at a 1:1 ratio, the calibration standard and QC concentrations are based on the ultrafiltrate volume only. Unknown samples do not need to be adjusted for the dilution with MES buffer.

5. SPECIAL HANDLING OF BIOLOGICAL SAMPLES

The method requires no special handling. Please ensure all "Universal Precautions" are taken.

Primaries, spiking solutions, calibration standards and QCs should be prepared on wet ice, and each prepared level should be aliquoted immediately and aliquots should be flash frozen in a dry ice/acetone bath for ~1 minute and stored in the freezer immediately after flash freezing.

6. DATA COLLECTION AND ANALYSIS SOFTWARE

Description	Version
Watson LIMS™ (Thermo Fisher Scientific)	7.5 or higher
Analyst® (AB Sciex)	1.7.2 or higher

CONFIDENTIAL

Serial Number: 0cfbae28-298d-4a49-9fe8-26a0ef3e0e64 accessed by Anika Pippin (PIPPINA) on Wed, 19 Jun 2024 13:45:54 GMT.

7. INSTRUMENTS, EQUIPMENT, AND CONDITIONS

Equivalent equipment can be substituted, except where noted in Section 7.1. Minor modifications of conditions are permitted to optimize chromatography, except where noted in Section 7.1. Refer to SOP-00073 for allowable minor modifications that do not require validation.

7.1. LC and MS Conditions

Table 1: HPLC System

**System Controller**	**Shimadzu Controller, CBM20A**
Pump	Shimadzu LC-30AD
Autosampler	Shimadzu SIL-30AC
Column Oven	Shimadzu CTO-30A
MS Interface	ESI
Analytical Column	Fortis Technology Ltd. Fortis C18 4.6 x 50mm, 5 µ
Column Guard Cartridge	Security Guard Cartridge AQ C18, 4.0 x 2.0 mm
Column Temperature (set at)	35°C
Mobile Phase A	0.5% Formic acid in deionized water
Mobile Phase B	90/10 Acetonitrile/deionized water
Needle Wash Solvent (R0, R3)	90/10 Acetone/deionized water (or other acceptable solution)
Total Run Time (approximate)	4.5 min
Injection Volume (µl)	0.1-5.0
Flow Rate	1.0 mL/min
Pressure, kgf/cm2 (approximate)	~100
Approximate Retention Time (min.)	Ertapenem: 1.66
	Ertapenem-d4: 1.66

CONFIDENTIAL

Serial Number: 0cfbae28-298d-4a49-9fe8-26a0ef3e0e64 accessed by Anika Pippin (PIPPINA) on Wed, 19 Jun 2024 13:45:54 GMT.

7.1.1. Gradient Table

Time Program

Time	Module	Events	Parameter
0.00	Pumps	Pump B Conc.	18
0.50	Pumps	Pump B Conc.	18
1.00	Controller	Event 1	
2.40	Pumps	Pump B Conc.	45
2.50	Pumps	Pump B Conc.	100
2.60	Controller	Event 2	
3.50	Pumps	Pump B Conc.	100
3.60	Pumps	Pump B Conc.	18
4.20	System Controller	Stop	

7.1.2. Autosampler Settings

Temperature: Set at 4°C
Rinse Type: External
Rinse Mode: Before and after aspiration
Rinse Dip Time: 0 sec
Rinse Method: Rinse Pump Then Port
Rinse Time: 2 sec
Rinsing Speed: 35 µL/sec
Rinsing Volume: 500 µL

7.1.3. Mass Spectrometer System

Mass Spectrometer Sciex 5000 or 6500 (Low Mass Mode)
Source Type: Turbo spray
Scan Type: MRM (MRM)
Polarity: Positive
Resolution Q1: Unit
Resolution Q3: Unit

CONFIDENTIAL

Serial Number: 0cfbae28-298d-4a49-9fe8-26a0ef3e0e64 accessed by Anika Pippin (PIPPINA) on Wed, 19 Jun 2024 13:45:54 GMT.

All the below parameters in this section may be adjusted to optimize the instrument conditions.

Ertapenem:

Q1 Mass (Da)	Q3 Mass (Da)	Dwell (msec)
476.047	431.9	250.00
DP	56.00	
CE	13.00	
CXP	13.00	

Ertapenem-d_4 (IS):

Q1 Mass (Da)	Q3 Mass (Da)	Dwell (msec)
480.2	436.3	250.00
DP	56.00	
CE	13.00	
CXP	13.00	

Dummy Ions (to counter-act potential crosstalk):

Q1 Mass (Da)	Q3 Mass (Da)	Dwell (msec)
250	200	100
254	204	100

Parameter Table:

CAD:	12.00
CUR:	40.00
GS1:	50.00
GS2:	60.00
IS:	5500
TEM:	600
EP	12

CONFIDENTIAL

Serial Number: 0cfbae28-298d-4a49-9fe8-26a0ef3e0e64 accessed by Anika Pippin (PIPPINA) on Wed, 19 Jun 2024 13:45:54 GMT.

8. REAGENTS, STANDARDS, AND SUPPLIES

Reagents from alternate supplier and equivalent grade can be substituted.

Table 2: Reagents, Standards, and Supplies

Chemical / Grade	Manufacturer/Supplier
Ertapenem	Performa
Ertapenem-d_4	Sigma Aldrich
MES ([2-(N-Morpholino) ethane sulphonic acid hydrate]) buffer	Sigma
Acetonitrile, HPLC grade	Honeywell
Formic Acid, 98+%	EMD Millipore
Deionized Water	Millipore
Ammonium Acetate ≥ 99.9%	Sigma
Methanol	Honeywell
Acetone	Honeywell
Ultrafiltered Human Sodium Heparin Plasma	BioIVT
Centrifugal Filters	Merck Millipore, Lot: 0000158890
Advantage SiliGuard Coated 2 mL 96 well Polypropylene plate: Square Top, Round Bottom	Analytical Sales and Services, Catalog # 27P687GC
Purple, pre-slit, square well, soft silicone/PTFE Cap Mats	Analytical Sales & Services, Inc., Catalog# 965085

9. SAFETY

Always observe safe laboratory practices. Consult the appropriate SDS before handling any chemical if you are unfamiliar with the proper safety precautions.

CONFIDENTIAL

Serial Number: 0cfbae28-298d-4a49-9fe8-26a0ef3e0e64 accessed by Anika Pippin (PIPPINA) on Wed, 19 Jun 2024 13:45:54 GMT.

10. PROCEDURE

10.1. Preparation of Analyte Stock Solutions and Dilutions

The quantities of analyte solution prepared may be scaled up or down proportionally as needed. Alternate dilution schemes can be used to achieve the appropriate final concentrations of standards, QCs, working internal standard, and system verification solution (SVS). For assignment of expiration dates to analyte stocks and their dilutions, refer to the latest version of the validation report.

10.1.1. Ertapenem Primary Solution, 2.00 mg/mL (Prepare on wet ice)

Check drying conditions prior to use. Weigh approximately 20.0 mg of ertapenem reference standard (correcting for purity and correction factor if necessary) into a polypropylene container and add 10.0 mL of MES buffer, pH 6.4, ~0.1M. Mix well and sonicate if necessary. Store solution at -20°C in a polypropylene container.

10.1.2. Working Internal Standard, ~5.0 mcg/mL Ertapenem-d_4 (Prepare on wet ice)

Check drying conditions prior to use. Weigh 1.00 mg of ertapenem-d_4 reference standard into a polypropylene container. Add 200 mL of MES buffer, pH 6.4, ~0.1M (do not correct for conversion factor and purity). Mix well and sonicate if necessary. **Aliquot** and store at or below -20 °C in polypropylene containers.

10.2. Preparation of Other Reagents

Prepared quantities of all reagents may be scaled up or down as necessary. Volumes provided are examples. For assignment of expiration dates, refer to SOP-00094.

10.2.1. Mobile Phase A: 0.5% Formic Acid in Deionized Water

Add 20 mL of formic acid to a 4-L volumetric flask containing ~3800 mL of deionized water. Dilute to volume with deionized water. Mix well and store at room temperature.

10.2.2. Mobile Phase B: 90/10 Acetonitrile / Deionized Water

Combine 3600 mL of acetonitrile with 400 mL of deionized water in a 4-L flask. Mix well and store at room temperature.

CONFIDENTIAL

Serial Number: 0cfbae28-298d-4a49-9fe8-26a0ef3e0e64 accessed by Anika Pippin (PIPPINA) on Wed, 19 Jun 2024 13:45:54 GMT.

10.2.3. Rinse: 90/10 Acetone/Deionized Water

Combine 3600 mL of acetone and 400 mL of deionized water in a 1-L flask. Mix well and store at room temperature.

10.2.4. 1 N Sodium Hydroxide (NaOH) in Deionized Water

Weight ~ 4.00 g of sodium hydroxide (F.W. 40.00) and gradually transfer it to a 100-mL volumetric flask containing ~50 mL of deionized water. Mix to dissolve and wait until it reaches room temperature. Dilute to volume with deionized water and mix well. Store in a polypropylene container at room temperature.

10.2.5. MES (2-(N-Morpholino) Ethanesulphonic Acid Hydrate) Buffer pH 6.4, ~0.1M

Weigh ~ 4000 mg of MES hydrate (F.W. 195.24) and transfer to a 200-mL volumetric flask containing ~150 mL of deionized water. Dissolve and adjust the pH to 6.4 with 1N NaOH. Dilute to volume with deionized water. Mix well and store refrigerated.

10.2.6. 5/95 Acetonitrile/Deionized Water

Combine 50 mL of acetonitrile and 950 mL of deionized water in a 1-L flask. Mix well and store at room temperature.

10.3. Preparation of Standards and QC Samples

10.3.1. Ertapenem Secondary Solution, 100 mcg/mL in Treated Human Plasma Ultrafiltrate (Reported as 200 mcg/mL; Prepare on wet ice)

Transfer 1.0 mL of ertapenem primary solution (2.00 mg/mL) to a polypropylene tube containing 19.0 mL of **chilled** treated sodium heparin human plasma ultrafiltrate on wet ice. Mix well, aliquot, and store at -70 °C or -20 °C.

10.3.2. Treated Human Plasma Ultrafiltrate (Ultrafiltrate: MES Buffer 1:1)

Add 200 mL of **chilled** sodium heparin human plasma ultrafiltrate to a polypropylene container containing 200 mL of **chilled** 0.1M MES Buffer, pH 6.4. Mix well and store at -20°C.

It is recommended to prepare the treated human plasma ultrafiltrate fresh prior to usage.

CONFIDENTIAL

Serial Number: 0cfbae28-298d-4a49-9fe8-26a0ef3e0e64 accessed by Anika Pippin (PIPPINA) on Wed, 19 Jun 2024 13:45:54 GMT.

10.3.3. Preparation of Standards and QC Samples in Human Plasma Ultrafiltrate

Use treated sodium heparin human plasma ultrafiltrate that has been diluted 1:1 with MES buffer, screened for the lack of interference at the retention times of ertapenem and ertapenem-d_4 (IS). Starting from the ertapenem secondary in treated human plasma ultrafiltrate (200 mcg/mL), prepare a series of calibration standards and QCs with treated sodium heparin human plasma ultrafiltrate on wet ice. Since unknown samples are treated with the MES buffer after collection at a 1:1 ratio, the calibration standard and QC concentrations are based on the ultrafiltrate volume only. Unknown samples do not need to be adjusted for the dilution with MES buffer. The prepared concentrations in plasma ultrafiltrate only, not accounting for MES buffer, will be as follows:

For standards: 50.0, 40.0, 20.0, 10.0, 5.00, 1.00, 0.100, and 0.0500 mcg/mL.

For QCs: 37.5, 25.0, and 0.150 mcg/mL, with 0.0500 mcg/mL for the LLOQ QCs when needed. Other concentrations may be prepared, provided they meet the requirements of SOP-00035 and provided they remain within the range of 0.0500 to 50.0 mcg/mL.

Store solutions in polypropylene at -70°C or -20°C.

10.3.4. Ultrafiltration of Human Plasma Samples using Centrifugal Filters

1. Thaw human plasma sample on wet ice. Once sample is thawed, vortex each sample for ~ 20 seconds.
2. Add ~1 mL of sample into a centrifugal filter. Hold the tube at ~45° with pipette tip touching the tube wall and add sample slowly with even flow to avoid air locking.
3. Cap the filter. The caps should extend no more than 3-4 mm the top. Further compression can cause prefiltration, which could introduce analytical error.
4. Centrifuge sample at 2000 x g for 30 minutes, this will yield ~75 mcL of filtrate.
5. Add **chilled** 0.1M MES Buffer, pH 6.4, at a 1:1 ratio (Sample Filtrate: MES Buffer). Vortex to Mix.
6. Use the resulting plasma filtrate samples for the extraction procedure in Section 10.4.

CONFIDENTIAL

Serial Number: 0cfbae28-298d-4a49-9fe8-26a0ef3e0e64 accessed by Anika Pippin (PIPPINA) on Wed, 19 Jun 2024 13:45:54 GMT.

10.4. Extraction Procedure

1. Thaw treated human plasma ultrafiltrate samples on wet ice. Once samples are thawed, vortex each sample for ~10 seconds immediately prior to pipetting.
2. Pipet 100 mcL of human plasma ultrafiltrate sample or blank to the corresponding wells of the 1-mL 96-well plate on wet ice.
3. Add 50 mcL of **COLD** working IS (5.0 mcg/mL ertapenem-d_4 in MES buffer, pH 6.4, ~0.1M) to all applicable wells. For **Control Blanks** add 50 mcL of MES buffer, pH 6.4, ~0.1M instead.
4. Seal the plate with an adhesive pad (or equivalent) and vortex at 1300 rpm for 5 minutes using a plate shaker.
5. Add 200 mcL of acetonitrile to all wells. Seal the plate with an adhesive pad (or equivalent) and vortex for 10 minutes at 1200 rpm.
6. Centrifuge the plate for 10 minutes at a setting of 4000 rpm (ambient conditions).
7. Transfer 200 mcL of the supernatant to a new 1-mL 96-well plate using a multichannel pipette (or equivalent).
8. Evaporate the sample using a 96-well SPE dryer at 37 °C at 60-80 psi. This generally takes around 70 minutes.
9. Reconstitute the plate with 130 mcL of 5/95 acetonitrile/deionized water in each well. Seal the plate with a cap mat.
10. Vortex the final plate at 1300 rpm for 5 minutes using a plate shaker.
11. Centrifuge the plate for 2 minutes at a setting of 3300 rpm (ambient conditions).
12. Refrigerate prior to LC-MS injection according to the conditions outlined in Section 7.

10.5. Dilutions

2x = 50 mcL of sample + 50 mcL of blank treated human plasma ultrafiltrate

5x = 20 mcL of sample + 80 mcL of blank treated human plasma ultrafiltrate

CONFIDENTIAL

Serial Number: 0cfbae28-298d-4a49-9fe8-26a0ef3e0e64 accessed by Anika Pippin (PIPPINA) on Wed, 19 Jun 2024 13:45:54 GMT.

10.6. Calculations

Linear regression is performed with a $1/x^2$ weighting factor.

Equation: Response = Slope * Conc + Intercept

11. REFERENCE CHROMATOGRAMS

Figure 1: Blank with IS

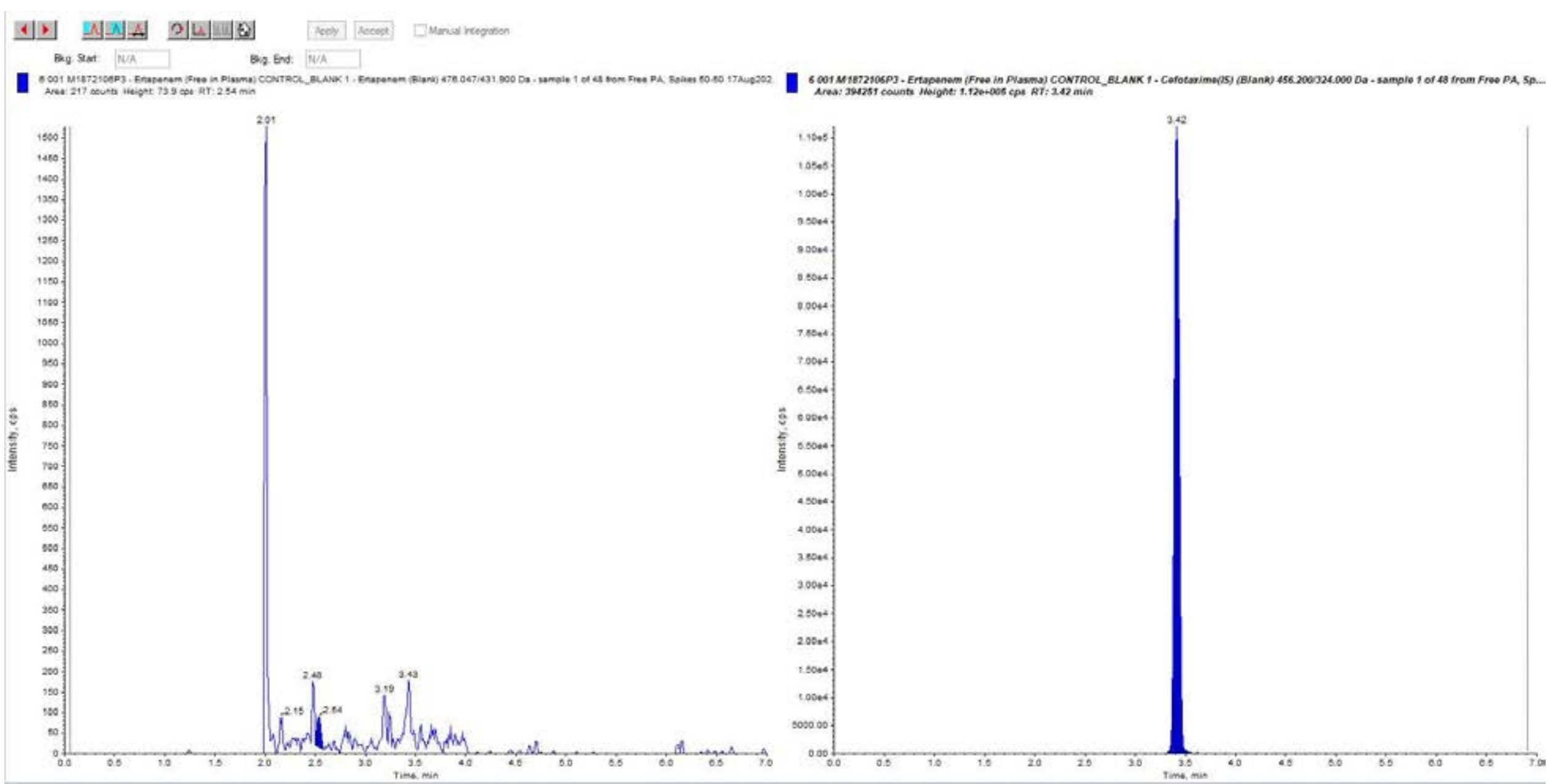

CONFIDENTIAL

Serial Number: 0cfbae28-298d-4a49-9fe8-26a0ef3e0e64 accessed by Anika Pippin (PIPPINA) on Wed, 19 Jun 2024 13:45:54 GMT.

Figure 2: LLOQ – Ertapenem 0.050 mcg/mL

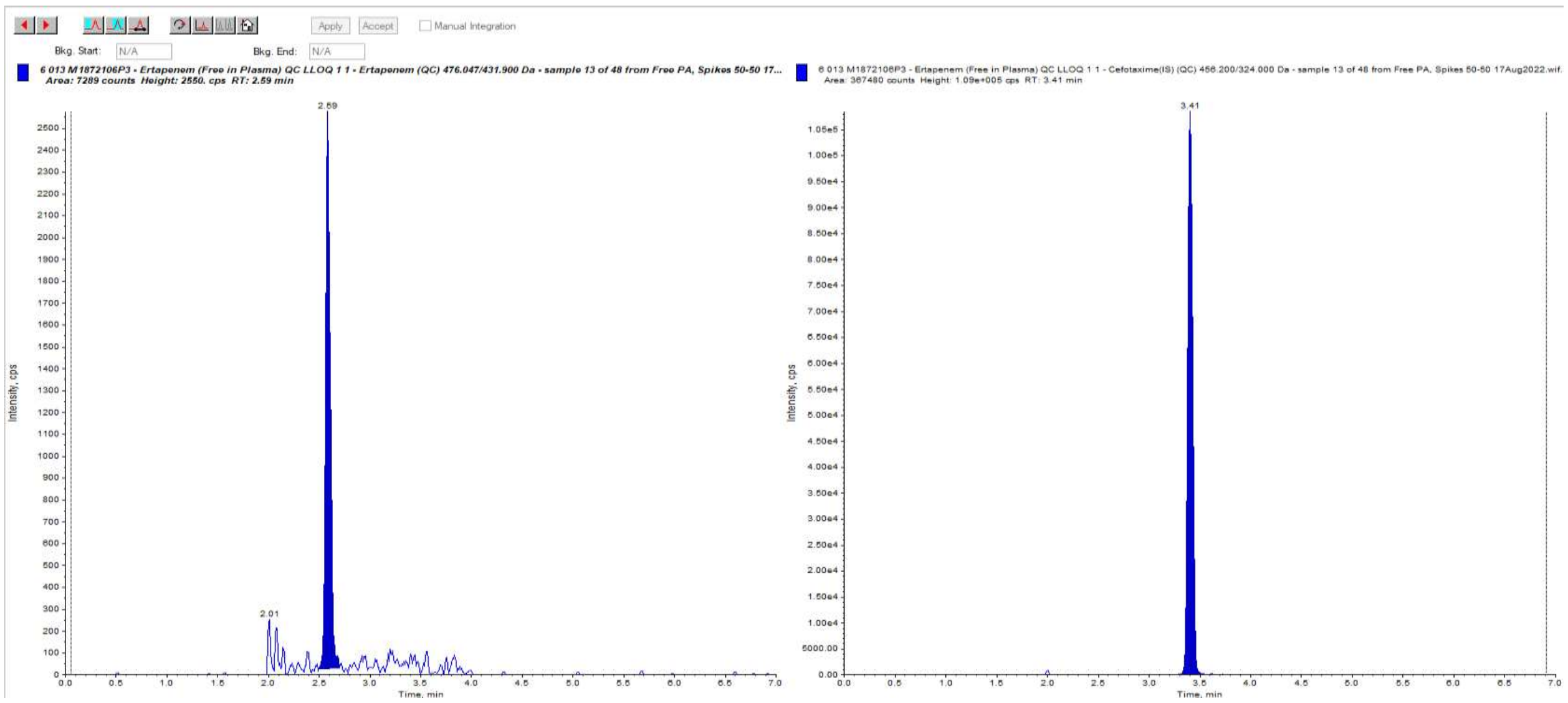

Figure 3: ULOQ – Ertapenem 50.0 mcg/mL

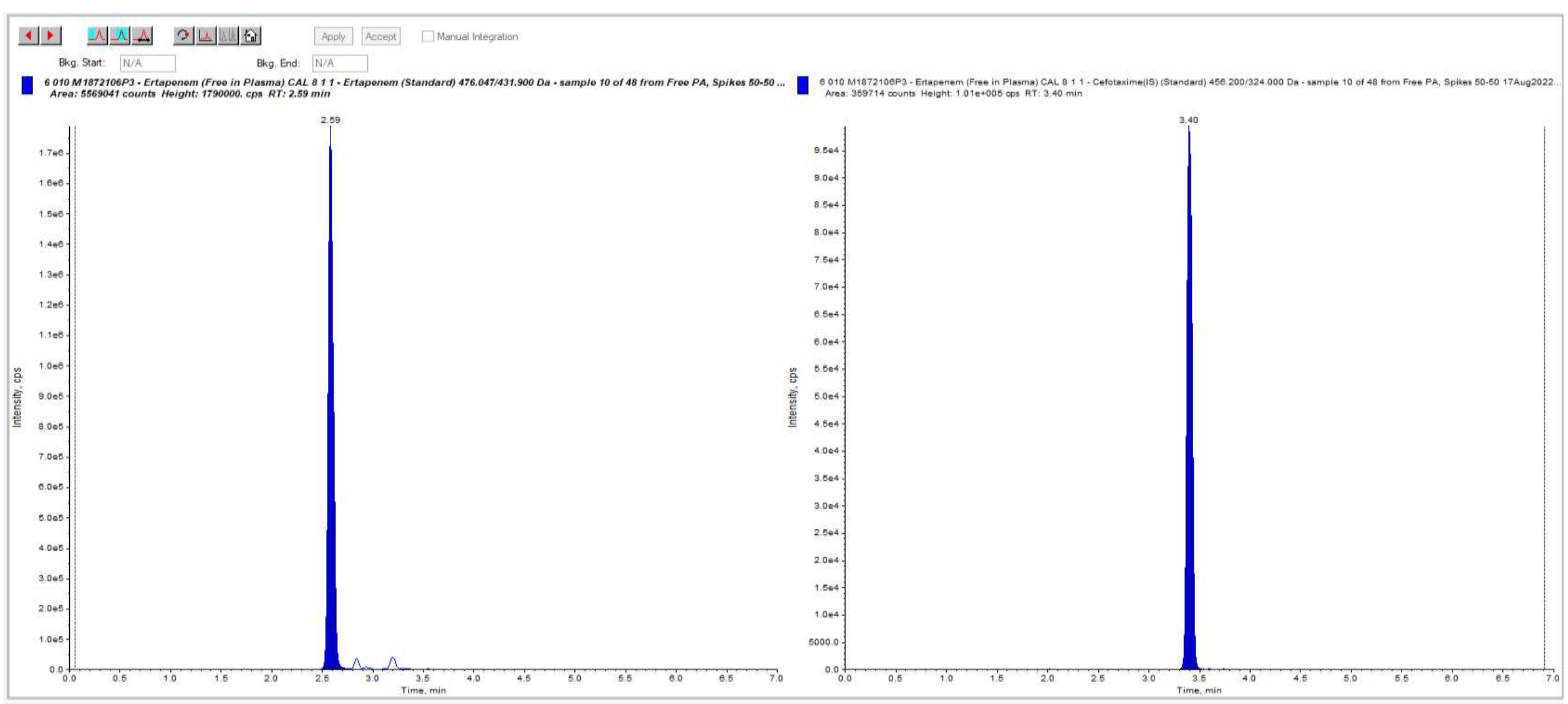

CONFIDENTIAL

Serial Number: 0cfbae28-298d-4a49-9fe8-26a0ef3e0e64 accessed by Anika Pippin (PIPPINA) on Wed, 19 Jun 2024 13:45:54 GMT.

12. REVISION HISTORY

Table 3: Revision History

Revision	Effective Date	Section	Revision Summary
01	See Header	10.3.4	(Ultrafiltration of Human Plasma Samples using Centrifugal Filters) Added a step for clarification to add MES buffer to filtered samples after filtration as the collected samples are not treated and are treated at KCAS. Clarified filtrate volume prior to MES buffer addition. These adjustments were made to clarify actual sample collection. No additional work is needed since sample composition is not changing.
00	28 Nov 2022	NA	New Procedure

CONFIDENTIAL

Serial Number: 0cfbae28-298d-4a49-9fe8-26a0ef3e0e64 accessed by Anika Pippin (PIPPINA) on Wed, 19 Jun 2024 13:45:54 GMT.

TP-80238 [01]: V1872106P3 - Ertapenem in Treated Human Plasma Ultrafiltrate (Sodium Heparin, 100 mcL, 0.0500 – 50.0 mcg/mL)

All blank fields are considered as data not available.

All Signature (Date and Time) shown in this report are in GMT.

**Type**	TP	**Status**	CURRENT	
**Effective Date**	19-Oct-2023	**Periodic Review Date**	05-Mar-2051	
**Owner**	PIPPINA	**Department Name**	Pharma LC-MS/MS	Pharma LC-MS/MS

Description

Full Title: LC-MS/MS Analysis of Free Ertapenem in Treated Human Plasma Ultrafiltrate (Sodium Heparin, 100 mcL, 0.0500 – 50.0 mcg/mL; Prior to MES Treatment)

Revision Notes (5)

Task Name	Action	Comment	Signature
Revise Document	Original Document Revision : 00	Trey Vinke indicated "It has come to our attention that TP-80238 (LC-MS/MS Analysis of Free Ertapenem in Treated Human Plasma Ultrafiltrate) requires a correction. "	DAVISONB<br>16-Oct-2023 18:48:19 [GMT]
Check Out	Document Profile Preparation	Editing and collaboration will occur outside of the system.	DAVISONB<br>16-Oct-2023 18:49:00 [GMT]
Check In	Document Profile Preparation	Edits made outside of the system.	NEVINSC<br>19-Oct-2023 17:22:30 [GMT]
Check Out	Document Finalization	Update header	NEVINSC<br>19-Oct-2023 17:30:24 [GMT]
Check In	Document Finalization	Update header completed.	NEVINSC<br>19-Oct-2023 17:31:18 [GMT]

Serial Number: 0cfbae28-298d-4a49-9fe8-26a0ef3e0e64 accessed by Anika Pippin (PIPPINA) on Wed, 19 Jun 2024 13:45:54 GMT.

Approval Information

Note to Approver

Reviewer: Bryan Parmentier
Management: Marsha Luna
PI: Anika Pippin

Approval Decision	Comment	Signature
Approve		PARMENTIERB<br>19-Oct-2023 19:34:13 [GMT]
Approve		LUNAM<br>19-Oct-2023 19:41:02 [GMT]
Approve		PIPPINA<br>19-Oct-2023 19:48:39 [GMT]

Serial Number: 0cfbae28-298d-4a49-9fe8-26a0ef3e0e64 accessed by Anika Pippin (PIPPINA) on Wed, 19 Jun 2024 13:45:54 GMT.

1. TITLE: THE DETERMINATION OF FREE ERTAPENEM IN TREATED HUMAN PLASMA ULTRAFILTRATE, WITH SODIUM HEPARIN AS ANTICOAGULANT, BY LC-MS/MS

BIOANALYTICAL METHOD VALIDATION REPORT

Bioanalytical Project Number:	V1872106P3
Sponsor:	DynPort Vaccine Company, LLC<br>110 Thomas Johnson Drive<br>Suite 200<br>Frederick, MD 21702
Bioanalytical Laboratory:	KCAS Bioanalytical & Biomarker Services.<br>10830 S Clay Blair Boulevard<br>Olathe, KS 66061
Issue Date:	February 2023
Amendment 1 Issue Date:	Date of PI Signature

2. SYNOPSIS

This report presents the validation data for an LC-MS/MS method for the determination of free ertapenem in treated human plasma ultrafiltrate, with sodium heparin as anticoagulant. The assay was analyzed in the range of 0.0500 to 50.0 mcg/mL and is for use on treated human plasma ultrafiltrate samples obtained during clinical trials.

AMENDMENT OUTLINE FORM

1. Amendment Number 1

This amendment was created to report:

- Long-term matrix stability at -70 °C for up to 186 days (with comed[2]) and 187 days (without comed[2]) for free ertapenem in treated human plasma ultrafiltrate, with sodium heparin as anticoagulant with and without the presence of comed[2].

3. TABLE OF CONTENTS FOR THE BIOANALYTICAL METHOD VALIDATION REPORT

1. TITLE: THE DETERMINATION OF FREE ERTAPENEM IN TREATED HUMAN PLASMA ULTRAFILTRATE, WITH SODIUM HEPARIN AS ANTICOAGULANT, BY LC-MS/MS 1
2. SYNOPSIS 2
3. TABLE OF CONTENTS FOR THE BIOANALYTICAL METHOD VALIDATION REPORT 3
4. LIST OF TABLES 4
5. SIGNATURE PAGE 5
6. QUALITY ASSURANCE STATEMENT 6
7. STUDY COMPLIANCE STATEMENT 7
8. LIST OF ABBREVIATIONS AND DEFINITIONS OF TERMS 8
9. INTRODUCTION 10
10. EXPERIMENTAL DESIGN AND RESULTS 10
10.1. Reference Standards 10
10.2. Calibration Standards and QC Samples Preparation 10
10.2.1. Carryover 11
10.3. Long-Term Matrix Stability at -70 °C 11
10.4. SOP Deviations and Investigations 11
11. CONCLUSION 12
12. PERTINENT SOPS 12
13. ARCHIVES 12
14. REFERENCES 13
15. TABLES 14

4. LIST OF TABLES

Table 1: Abbreviations and Codes 8
Table 2: Deviations 12
Table 3: Mass Spec Transitions 14
Table 4: Reference Standards 14
Table 5: Calibration Standards and QC Samples Preparation 15
Table 6: Matrix Description 16
Table 7: Summary of Results and Conclusions 17
Table 8: Summary of Validation Run Objectives and Outcome 19
Table 9: Carryover 19
Table 10: Long-Term Stability at -70 °C 20

Annex 3 .... Certificate(s) of Analysis

5. SIGNATURE PAGE

Bioanalytical Project Number: V1872106P3

Amendment 1 Issue Date: Date of PI Signature

APPROVED

DocuSigned by:
Anika Pippin
Signer Name: Anika Pippin
Signing Reason: I approve this document
Signing Time: 19-Jun-2023 | 13:16 CDT
BE3AA1198E40459E8A7E3C615D981D82

Anika Pippin, B.S.
Principal Investigator

REVIEWED

DocuSigned by:
Marsha Luna
Signer Name: Marsha Luna
Signing Reason: I have reviewed this document
Signing Time: 19-Jun-2023 | 09:20 CDT
C9CC738561AF4CC1B375ED013DD1E4BD

Marsha Luna, B.S.
Management

6. QUALITY ASSURANCE STATEMENT

This Bioanalytical Method Validation Report has been found to describe accurately the methods and SOPs used and to accurately reflect the raw data collected. The Quality Assurance Unit initiated audits on this study and reported the findings to the Principal Investigator (PI) and Management as follows:

		**Date(s) Sent to:**	
**Inspection Date(s)**	**Inspection Phase**	**PI**	**Management**
01Mar2023	Data Audit	01Mar2023	01Mar2023
16Mar2023	Data Audit	16Mar2023	09May2023
23May2023	Data Audit	23May2023	15Jun2023
08Jun2023	Data Audit	08Jun2023	15Jun2023
09Jun2023	Draft Report Review	09Jun2023	15Jun2023
15Jun2023	Final Report Review	15Jun2023	15Jun2023

REVIEWED

DocuSigned by:

Jessica Lopez

Signer Name: Jessica Lopez
Signing Reason: I have reviewed this document
Signing Time: 19-Jun-2023 | 08:39 CDT
95C7FCBE477F4555996DA2F7A85A8F26

Jessica Lopez
Quality Assurance Unit

7. STUDY COMPLIANCE STATEMENT

I, the undersigned, hereby declare that this study was conducted in compliance with the relevant bioanalytical aspects of FDA Guidance for Industry on Bioanalytical Methods Validation, May 2018 [1] and KCAS Bioanalytical & Biomarker Services (KCAS) procedures and that the work was performed by me, or under my direction, and the report reflects a true and accurate record of the results obtained.

The acceptance criteria are based on the FDA Guidance for Industry, Bioanalytical Method Validation (2018). [1]

APPROVED

DocuSigned by:

Anika Pippin

Signer Name: Anika Pippin
Signing Reason: I approve this document
Signing Time: 19-Jun-2023 | 13:16 CDT
BE3AA1198E40459E8A7E3C615D981D82

Anika Pippin, B.S.
Principal Investigator

8. LIST OF ABBREVIATIONS AND DEFINITIONS OF TERMS

Table 1: Abbreviations and Codes

Abbreviation	Definition
<	Concentration less than the lowest acceptable calibration standard
>	Concentration exceeded the upper limit of calibration range (> ULOQ)
A	Amendment
AQL	Above Quantitation Limit
%Bias	Percentage measurement valid
Batch	Is the same as Run
BQL	Below Quantifiable Limit
Cal	Calibration standard
Conc.	Concentration
Conv.	Conversion
%CV	Coefficient of Variation
Dev	Deviation
Diff	Difference
EMA	European Medicines Agency
FDA	Food and Drug Administration
GLP	Good Laboratory Practice
HPLC	High Performance Liquid Chromatography
ID	Identification
IS	Internal Standard
ISR	Incurred Sample Reanalysis
K_2EDTA or K_3EDTA	Dipotassium or Tripotassium Ethylenediaminetetraacetic Acid
KCAS	KCAS Bioanalytical & Biomarker Services
LC-MS/MS	Liquid Chromatography Tandem Mass Spectrometry
LLOQ	Lower Limit of Quantitation
mcg or µg or ug	Microgram
mcL or µL or uL	Microliter

Table 1: Abbreviations and Codes (Continued)

Abbreviation	Definition
ME	Matrix Effect
MF	Matrix Factor
MHLW	Ministry of Health, Labour and Welfare of Japan
mL	Milliliter
MS	Mass Spectrometry
n	number
N.C.	Not Calculated
ND	No difference reported as ISR will be counted
ng	nanogram
n.report	No Data available to report
N/A or NA	Not Applicable
N.R.	Not Reported
P&A	Precision and Accuracy
p.a.r.	Peak Area Ratio
PI	Principal Investigator
QC	Quality Control Sample
QCs	Quality Control Samples
OECD	Organization for Economic Co-operation and Development
RT	Room Temperature
sd or SD	Standard deviation
Seq	Sequence
SOP (s)	Standard Operating Procedure (s)
temp	Temperature
TP	Test procedure
UHPLC	Ultra High Performance Liquid Chromatography
ULOQ	Upper Limit of Quantitation
WIS	Working Internal Standard

This is a basic list. Some of the abbreviations may not have been used in this report.

9. INTRODUCTION

This amendment was created to report long-term matrix stability at -70 °C for up to 186 days (with comed[2]) and 187 days (without comed[2]) for free ertapenem in treated human plasma ultrafiltrate, with sodium heparin as anticoagulant with and without the presence of comed[2].

The validation procedure was designed to meet or exceed the requirements of FDA Guidance for Industry on Bioanalytical Methods Validation, May 2018. The software programs used were Microsoft Office Excel 365, acquisition and processing software Analyst 1.7.2, and data management software Watson LIMS 7.5.

10. EXPERIMENTAL DESIGN AND RESULTS

In this method, ertapenem and internal standard, ertapenem-d_4 (IS), were released from human plasma ultrafiltrate using 96-well protein precipitation. After extraction, the sample was chromatographed using reversed phase HPLC with a Fortis Technology Ltd. Fortis C18 analytical column. Ertapenem and ertapenem-d_4 (IS) were detected by monitoring the precursor and product ions described in Table 3 using an Applied Biosystems Sciex API 5000 LC-MS/MS. The quantification was based on setting a calibration graph using the internal standard method based on bioanalytical test procedure TP-80238, entitled "LC-MS/MS Analysis of Free Ertapenem in Treated Human Plasma Ultrafiltrate (Sodium Heparin, 100 mcL, (0.0500 – 50.0 mcg/mL; Prior to MES Treatment)". The test procedure can be found in Annex 1 (V1872106P3).

Human plasma ultrafiltrate, screened for the lack of interference at the retention times of ertapenem and ertapenem-d_4 (IS), was diluted 1:1 with MES buffer, pH 6.4 for stabilization. Since the standards and QCs concentrations were already adjusted for the addition of MES during the preparation and are reported based on plasma ultrafiltrate volume only, the obtained free ertapenem concentration of the unknown study samples do not need to be adjusted for the dilution with MES buffer.

A summary of the validation results is provided in Table 7. A summary describing the objective(s) and outcome of each validation run attempted is provided in Table 8.

The method was validated within the analytical range of 0.0500 to 50.0 mcg/mL in human plasma ultrafiltrate. No development work was performed after the method was validated.

10.1. Reference Standards

Reference standards used during this validation are presented in Table 4.

10.2. Calibration Standards and QC Samples Preparation

Calibration standards and quality control pools were prepared by spiking appropriate levels of ertapenem into treated human plasma ultrafiltrate, with sodium heparin as anticoagulant.

Primary reference standards were prepared in at least duplicate and compared within 5% of each other. Standards and QCs were prepared and stored frozen at KCAS at -70 °C. Stability QCs were also stored at -20 °C. Standards, QCs, and the critical solutions used during this validation are described in Table 5, and matrix used is described in Table 6.

10.2.1. Carryover

Carryover is evaluated within each run. It consists of measuring the response for each analyte and if it exceeds 20.0% of the response of LLOQ standard or other sample reflecting the lower sensitivity limit required, a carryover assessment is completed, and affected samples are rejected. The internal standard carryover evaluation is assessed within each run and if it exceeds 5.0% of the average internal standard response of acceptable standards and QCs, impact is evaluated within the run. Data are presented in Table 9.

10.3. Long-Term Matrix Stability at -70 °C

To evaluate ertapenem in human plasma ultrafiltrate, with sodium heparin as anticoagulant, stability during sample storage, aliquots of the high and low concentration QC pools with and without the presence of comed[2] were stored frozen at -70 °C nominally for 98, 112, and 187 days (without comed[2]) and 97, 111, and 186 days (with comed[2]) prior to being analyzed, along with freshly prepared standards and QC samples. To be deemed stable, the mean %Bias at each level must be within ± 15.0% with %CV ≤ 15.0%.

Data are provided in Table 10.

See Section 10.4 for reason for run 1 stability failure.

10.4. SOP Deviations and Investigations

One process deviation occurred during this study. No investigations were conducted during this study.

Table 2: Deviations

Deviation #	Description	Impact
DEV-2023-0104	Run 1 both comed and regular LTS showed high %Bias compared to the fresh calibration standards and QCs, 30.7% bias and 22.4% bias for the comed low and high, respectively, and 27.3% bias and 21.1% bias for the regular LTS at the low and high level, respectively. This was repeated in run 2 with passing data, 6.0% bias and -2.7% bias for the comed low and high, respectively, and 5.3% bias and -1.9% bias for the regular LTS at the low and high level, respectively.<br>The high failure was attributed to human error during preparation or extraction (pipetting sample) and the repeat run shows passing data, further showing that this was a preparation error. This deviation was written as documentation to fulfill contract requirements for 1872106, no deviation occurred.	There is no impact.

11. CONCLUSION

Ertapenem in treated human plasma ultrafiltrate with and without the presence of comed[2], with sodium heparin as anticoagulant, was proven to be stable at -70 °C over a period of 186 days (with comed[2]) and 187 days (without comed[2]) in polypropylene tubes.

12. PERTINENT SOPS

The following SOPs were followed during the conduct of this study:

SOP-00073 [04] - Bioanalytical Method Validation - Chromatographic Assays

SOP-00035 [02] - Procedures, Reporting, and Acceptance Criteria for Bioanalytical Chromatographic Projects

13. ARCHIVES

All raw data, including paper and electronic files associated with this study will remain archived at a location specified by KCAS, 10830 S Clay Blair Boulevard, Olathe, KS 66061 for a period of at least 10 years, after which the client may be contacted to arrange for continued storage of the files, or disposition according to an agreed procedure. KCAS will maintain an electronic (scanned/true) copy of the method validation report that can be viewed and printed on site. The study plan, documentation, raw data, and final report are indexed under Bioanalytical Project Number V1872106P3A1.

14. REFERENCES

1. Guidance for Industry: Bioanalytical Method Validation. U.S. Department of Health and Human Services. Food and Drug Administration. Center for Drug Evaluation and Research. Center for Veterinary Medicine. May 2018.
2. Comeds: QCs in the presence of 150 mcg/mL zidebactam.

15. TABLES

Table 3: Mass Spec Transitions

Analyte	Transition
Ertapenem	m/z 476.047→431.9
Ertapenem-d_4 (IS)	m/z 480.2→436.3

Table 4: Reference Standards

<table>
<tr><th>Analyte</th><th>Supplier</th><th>Lot/Batch Number</th><th>Purity</th><th>Expiration Date or Retest Date</th><th>Conversion Factor</th></tr>
<tr><td rowspan="2">Ertapenem Sodium</td><td rowspan="2">MedChemExpress</td><td>180017</td><td>98.9%</td><td>Retest 11 Aug 2024</td><td rowspan="2">1.046</td></tr>
<tr><td>229333</td><td>98.43%</td><td>Retest 29 Sep 2025</td></tr>
<tr><td>Ertapenem-d_4 Sodium Salt (IS)</td><td>AlsaChim</td><td>DH-ALS-20-002-P2</td><td>*</td><td>Expire 29 Apr 2023</td><td>*</td></tr>
<tr><td rowspan="2">Zidebactam</td><td rowspan="2">Wockhardt</td><td rowspan="2">WK1/WP-0060/016</td><td>90.4%</td><td>Expire 29 Mar 2023</td><td rowspan="2">1.000</td></tr>
<tr><td>90.4%</td><td>Retest 01 Mar 2024</td></tr>
</table>

Certificates of analysis for the above reference standards are presented in Annex 3.
*Used as is with 1.000 conversion and 100% purity as IS only.

Table 5: Calibration Standards and QC Samples Preparation

<table>
<tr><th>Critical Solutions</th><th>Lot # for Standards
(Prep Date)</th><th>Lot # for QCs
(Prep Date)</th><th>Standards Lot
(Prep Date)</th><th>QCS Lot
(Prep Date)</th><th>Runs used</th></tr>
<tr><td colspan="3">Human Plasma Ultrafiltrate Treated with MES Buffer, ~0.1M, pH 6.4 (1:1)
Lot V1872106P3-15-1</td><td rowspan="3">N/A</td><td rowspan="3">V1872106P3-14-1
Set A
(10 Nov 2022)</td><td rowspan="3">1, 2, 4
(LTS)</td></tr>
<tr><td>MES Buffer, pH 6.4 stored frozen (-20°C) in polypropylene</td><td>N/A</td><td>V1872106P3-2-1
(09 Nov 2022)</td></tr>
<tr><td>Human Plasma Ultrafiltrate Treated with MES Buffer, ~0.1M, pH 6.4 (1:1) stored frozen (-70°C) in polypropylene</td><td>N/A</td><td>V1872106P3-12-2
(10 Nov 2022)</td></tr>
<tr><td colspan="3">Human Plasma Ultrafiltrate Treated with MES Buffer, ~0.1M, pH 6.4 (1:1)
Lot V1872106P3-15-1</td><td rowspan="4">NA</td><td rowspan="4">Comed QCs
V1872106P3-22-1
Set A
(11 Nov 2022)</td><td rowspan="4">1, 2, 4
(LTS)</td></tr>
<tr><td>Deionized water stored frozen (-20°C) in polypropylene</td><td>NA</td><td>V1872106P4-1-1
(27 Oct 2022)</td></tr>
<tr><td>MES Buffer, pH 6.4 stored frozen (-20°C) in polypropylene</td><td>NA</td><td>V1872106P3-2-1
(09 Nov 2022)</td></tr>
<tr><td>Human Plasma Ultrafiltrate Treated with MES Buffer, ~0.1M, pH 6.4 (1:1) stored frozen (-70°C) in polypropylene</td><td>NA</td><td>V1872106P3-12-2
(10 Nov 2022)</td></tr>
<tr><td colspan="3">Human Plasma Ultrafiltrate Treated with MES Buffer, ~0.1M, pH 6.4 (1:1)
Lot V1872106P3A1-1-1</td><td rowspan="3">V1872106P3A1-2-1
Set A
(16 Feb 2023)</td><td rowspan="3">V1872106P3A1-3-1
Set A
(16 Feb 2023)</td><td rowspan="3">1</td></tr>
<tr><td>MES Buffer, pH 6.4 stored frozen (-20°C) in polypropylene</td><td>V1872106P5-124-1
(10 Feb 2023)</td><td>V1872106P5-125-1
(10 Feb 2023)</td></tr>
<tr><td>Human Plasma Ultrafiltrate Treated with MES Buffer, ~0.1M, pH 6.4 (1:1) stored frozen (-70°C) in polypropylene</td><td>V1872106P3A1-1-2
(16 Feb 2023)</td><td>V1872106P3A1-1-3
(16 Feb 2023)</td></tr>
<tr><td colspan="3">Human Plasma Ultrafiltrate Treated with MES Buffer, ~0.1M, pH 6.4 (1:1)
Lot V1872106P3A1-1-1</td><td rowspan="3">V1872106P3A1-5-1
Set B
(02 Mar 2023)</td><td rowspan="3">V1872106P3A1-6-1
Set B
(02 Mar 2023)</td><td rowspan="3">2</td></tr>
<tr><td>MES Buffer, pH 6.4 stored frozen (-20°C) in polypropylene</td><td>V1872106P5A1-1-1
(01 Mar 2023)</td><td>V1872106P5A1-2-1
(01 Mar 2023)</td></tr>
<tr><td>Human Plasma Ultrafiltrate Treated with MES Buffer, ~0.1M, pH 6.4 (1:1) stored frozen (-70°C) in polypropylene</td><td>V1872106P3A1-4-1
(02 Mar 2023)</td><td>V1872106P3A1-4-2
(02 Mar 2023)</td></tr>
<tr><td colspan="3">Human Plasma Ultrafiltrate Treated with MES Buffer, ~0.1M, pH 6.4 (1:1)
Lot V1872106P4-29-2</td><td rowspan="3">V1872106P3A1-14-1
Set C
(16 May 2023)</td><td rowspan="3">V1872106P3A1-15-1
Set C
(16 May 2023)</td><td rowspan="3">4</td></tr>
<tr><td>MES Buffer, pH 6.4 stored frozen (-20°C) in polypropylene</td><td>V1872106P3A1-7-1
(15 May 2023)</td><td>V1872106P3A1-7-1
(15 May 2023)</td></tr>
<tr><td>Human Plasma Ultrafiltrate Treated with MES Buffer, ~0.1M, pH 6.4 (1:1) stored frozen (-70°C) in polypropylene</td><td>V1872106P3A1-13-1
(16 May 2023)</td><td>V1872106P3A1-13-1
(16 May 2023)</td></tr>
</table>

Table 6: Matrix Description

Matrix	Source Lot #	Date Received
Human Plasma Ultrafiltrate Treated with MES Buffer, ~0.1M, pH 6.4 (1:1) Lot V1872106P3-15-1	BioIVT Combo-70-1 (HMN790515, HMN790517, HMN790518, HMN790520, HMN790521, HMN790523)	Apr 2022
Human Plasma Ultrafiltrate Treated with MES Buffer, ~0.1M, pH 6.4 (1:1) Lot V1872106P4-29-2	BioIVT, Lot HMN790516	Apr 2022
Human Plasma Ultrafiltrate Treated with MES Buffer, ~0.1M, pH 6.4 (1:1) Lot V1872106P4A1-3-1	BioIVT, Lot HMN790518	Apr 2022
Human Plasma Ultrafiltrate Treated with MES Buffer, ~0.1M, pH 6.4 (1:1) Lot V1872106P3A1-1-1	BioIVT, Lot HMN790524	Apr 2022

Table 7: Summary of Results and Conclusions

Methodology – TP-80238[D00]	Method Performance – LC-MS/MS	Hyperlink – N/A	Comments
Matrix	Plasma ultrafiltrate		
Anticoagulant	Sodium heparin		
Assay Volume	100 mcL		
**Selectivity**			
Analyte	Ertapenem		V1872106P3
Internal Standard	Ertapenem-d_4 (IS)		V1872106P3
Matrix Effect<br>(6 spiked matrix lots)	Complies		V1872106P3
Interference	Complies		V1872106P3
Carryover	Assessed with every run with standards and QCs	Summary tables<br>Table 9<br><br>Report text<br>Section 10.2.1	
**Linearity**			
Weighting	$1/x^2$		V1872106P3
Bias at LLOQ	-0.6%		V1872106P3
Bias above LLOQ	-4.4 to 3.0%		V1872106P3
Bioanalytical Range	Complies<br>0.0500 to 50.0 mcg/mL		V1872106P3
**Precision**			
Intra-assay	5.5 to 10.2% (LLOQ);<br>1.0 to 5.2% (above LLOQ)		V1872106P3
Inter-assay	16.0% (LLOQ);<br>4.3 to 9.0% (above LLOQ)		V1872106P3
**Accuracy**			
Intra-assay	-15.6 to 13.4% (LLOQ);<br>-11.3 to 4.7% (above LLOQ)		V1872106P3
Inter-assay	-5.2% (LLOQ);<br>-7.5 to -6.0% (above LLOQ)		V1872106P3
**Large Run Size**	Determined to be 158 samples.		V1872106P3
**Dilution**			
Dilution factor: 2x	Dilution QC: 37.5 mcg/mL<br>Precision: 2.1%<br>Accuracy: -0.8%		V1872106P3
Dilution factor: 5x	Dilution QC: 200 mcg/mL<br>Precision: 2.7%<br>Accuracy: -2.0%		V1872106P3

Table 7: Summary of Results and Conclusions (Continued)

Methodology – TP-80238[D00]	Method Performance – LC-MS/MS	Hyperlink – N/A	Comments
**Stability**			
Matrix (in polypropylene tubes)			
Freeze/Thaw (-20 °C/ice-water bath)	Demonstrated instability at 3 cycles		V1872106P3
Freeze/Thaw (-70 °C/ice-water bath) with and without the presence of comed[2]	Demonstrated for 3 cycles		V1872106P3
Ice-Water Bath	Demonstrated for 24 hours		V1872106P3
Long-term (-20 °C) with and without the presence of comed[2]	Demonstrated instability at 28 days without comed[2]<br>Demonstrated instability at 27 days with comed[2]		V1872106P3
Long-term (-70 °C) with and without the presence of comed[2]	Demonstrated for 187 days without comed[2]<br>Demonstrated for 186 days with comed[2]	Summary tables Table 10<br>Report text Section 10.3	
Processed Matrix polypropylene			
Refrigeration	Demonstrated for 5 days 22 hours		V1872106P3
**Individual Sample Reinjection**	Reproducible		V1872106P3
**Whole Run Reinjection Integrity**	Reproducible<br>Duration 4 days 11 hours		V1872106P3
**Column Ruggedness**	Reproducible		V1872106P3
**Extraction Recovery**			
Ertapenem	Overall 91.1%		V1872106P3
Ertapenem-d_4 (IS)	Overall 134.5%		V1872106P3
**Solution Stability**			
Solution, Primary polypropylene			
Ertapenem in MES buffer (RT)	Stable 6.75 hours		V1872106P5
Ertapenem in MES buffer (-20 °C)	Stable 7 days		V1872106P5
Ertapenem in MES buffer (-20 °C)	Instability observed at 28 days		V1872106P3
Ertapenem-d_4 (IS) in MES buffer (RT)	Taken to be same as ertapenem	SOP 00073	
Ertapenem-d_4 (IS) in MES buffer (-20 °C)	1 year stability can be assigned and evaluated thereafter in yearly increments	SOP 00073	

Table 8: Summary of Validation Run Objectives and Outcome

Run ID	Instrument ID	Extraction Date	Assay Date	Run Objective(s)	Accepted or Rejected	Comment
1	LC/MS-39	16-Feb-2023	17-Feb-2023	Long-term matrix stability with and without comed[2]	Accepted	a
2	LC/MS-39	02-Mar-2023	04-Mar-2023	Long-term matrix stability with and without comed[2]	Accepted	
4	LC/MS-24	16-May-2023	16-May-2023	Long-term matrix stability with and without comed[2]	Accepted	

Run 3 was created in error and was not used.
[a]The stability failed high and this did not point to instability. This was informally investigated and repeated for confirmation to verify results or see if there was an extraction issue as requested by sponsor.

Table 9: Carryover

Run ID	Result (Extracted Blank after High Standard)	Carryover Results for Internal Standard
1	Seq 13 and Seq 56 are <20.0% of the LLOQ.	Seq 4 and Seq 47 are < 5.0% internal standard peak of acceptable standards.
2	Seq 13 is <20.0% of the LLOQ and Seq 56 is 28.7% of the LLOQ.	Seq 4 and Seq 47 are < 5.0% internal standard peak of acceptable standards.
4	Seq 13 and Seq 56 are <20.0% of the LLOQ.	Seq 4 and Seq 47 are < 5.0% internal standard peak of acceptable standards.

Note: Calibration Standards and QCs are arranged in a way to mitigate carryover in all validation runs.
Carryover is evaluated within each run. It consists of measuring the response for each analyte and if it exceeds 20% of the response of LLOQ standard or other sample reflecting the lower sensitivity limit required, a carryover assessment is completed, and affected samples are rejected. The internal standard carryover evaluation is assessed within each run and if it exceeds 5% of the average internal standard response of acceptable standards and QCs, impact is evaluated within the run.
Carryover evaluation of the internal standard are within 5% in each run. Calibration Standards and QCs are arranged in a way to mitigate carryover in all validation runs, and no QCs was deactivated due to carry-over assessments.

Table 10: Long-Term Stability at -70 °C

Analytical Performance: Back-Calculated Concentrations (µg/mL) of Ertapenem Calibration Standard in (Human) (Plasma Ultrafiltrate) in (Study V1872106P3 A1)

Assay Date	Run ID	Cal 0.05 0.0500 µg/mL	Cal 0.1 0.100 µg/mL	Cal 1 1.00 µg/mL	Cal 5 5.00 µg/mL	Cal 10 10.0 µg/mL	Cal 20 20.0 µg/mL	Cal 40 40.0 µg/mL	Cal 50 50.0 µg/mL
17-Feb-2023	1	0.0478	0.108	0.996	5.38	10.4	20.7	39.5	49.2
		0.0498	0.101	0.990	5.09	9.65	19.2	38.1	47.9
04-Mar-2023	2	0.0450	0.109	1.04	4.91	10.3	20.6	39.1	48.7
		0.0522	0.102	0.998	5.21	9.46	19.8	37.2	50.5
16-May-2023	4	0.0503	0.109	1.12	5.51	10.2	19.9	37.1	46.0
		0.0502	0.0872	1.10	5.28	10.1	19.3	36.6	45.1
Mean		0.0492	0.103	1.04	5.23	10.0	19.9	37.9	47.9
S.D.		0.00250	0.00838	0.0569	0.213	0.377	0.631	1.17	2.03
%CV		5.1	8.1	5.5	4.1	3.8	3.2	3.1	4.2
%Bias		-1.6	3.0	4.0	4.6	0.0	-0.5	-5.3	-4.2
n		6	6	6	6	6	6	6	6

Calibration Curve Parameters for Ertapenem Calibration Standards in Human Plasma Ultrafiltrate (Study V1872106P3 A1)

Run Date	Run ID	Slope	Intercept	R-Squared	LLOQ	ULOQ
17-Feb-2023	1	0.07128	-0.00011	0.9979	0.0500	50.0
04-Mar-2023	2	0.15395	0.00015	0.9971	0.0500	50.0
16-May-2023	4	0.01265	0.00008	0.9925	0.0500	50.0

Human Plasma Ultrafiltrate Ertapenem Concentrations in Quality Controls for 3 separate batch runs (Study V1872106P3 A1)

Run Date	Run ID	QCS 0.15 0.150 µg/mL	%Bias	QCS 25 25.0 µg/mL	%Bias	QCS 37.5 37.5 µg/mL	%Bias
17-Feb-2023	1	0.159	6.0	26.0	4.0	39.2	4.5
		0.156	4.0	25.0	0.0	36.7	-2.1
Intrarun Mean		0.158		25.5		38.0	
Intrarun %Bias		5.3		2.0		1.3	
04-Mar-2023	2	0.157	4.7	25.1	0.4	37.0	-1.3
		0.151	0.7	26.2	4.8	33.8	-9.9
Intrarun Mean		0.154		25.7		35.4	
Intrarun %Bias		2.7		2.8		-5.6	
16-May-2023	4	0.149	-0.7	25.8	3.2	35.7	-4.8
		0.161	7.3	24.0	-4.0	35.6	-5.1
Intrarun Mean		0.155		24.9		35.7	
Intrarun %Bias		3.3		-0.4		-4.8	

Table 10: Long-Term Stability at -70 °C (Continued)

Treated with and without comed[2]

Run Date	Run ID	Comed1 LTS -70 low 0.150 µg/mL	%Bias	LTS -70 low 0.150 µg/mL	%Bias	Comed1 LTS -70 high 37.5 µg/mL	%Bias	LTS -70 high 37.5 µg/mL	%Bias
17-Feb-2023	1	#0.206	37.3	#0.190	26.7	#47.9	27.7	#47.2	25.9
(98 days without comed[2])		#0.200	33.3	#0.188	25.3	#46.1	22.9	#45.5	21.3
(97 days with comed[2])		#0.193	28.7	#0.202	34.7	#45.5	21.3	#45.7	21.9
		#0.186	24.0	#0.183	22.0	#44.8	19.5	#44.3	18.1
		#0.193	28.7	#0.187	24.7	#46.0	22.7	#43.9	17.1
				#0.197	31.3	#44.9	19.7	#45.5	21.3
Intrarun Mean		0.196		0.191		45.9		45.4	
Intrarun SD		0.00764		0.00703		1.13		1.17	
Intrarun %CV		3.9		3.7		2.5		2.6	
Intrarun %Bias		30.7		27.3		22.4		21.1	
n		5		6		6		6	
04-Mar-2023	2	#0.180	20.0	0.171	14.0	38.3	2.1	37.1	-1.1
(112 days without comed[2])		0.149	-0.7	0.155	3.3	36.5	-2.7	35.7	-4.8
(111 days with comed[2])		0.149	-0.7	0.145	-3.3	36.7	-2.1	36.2	-3.5
		0.144	-4.0	0.154	2.7	34.2	-8.8	38.4	2.4
		0.169	12.7	0.163	8.7	38.3	2.1	36.4	-2.9
		0.162	8.0	0.162	8.0	35.1	-6.4	37.2	-0.8
Intrarun Mean		0.159		0.158		36.5		36.8	
Intrarun SD		0.0140		0.00898		1.66		0.952	
Intrarun %CV		8.8		5.7		4.5		2.6	
Intrarun %Bias		6.0		5.3		-2.7		-1.9	
n		6		6		6		6	
16-May-2023	4	0.167	11.3	0.166	10.7	36.7	-2.1	37.3	-0.5
(187 days without comed[2])		0.159	6.0	0.164	9.3	34.3	-8.5	37.6	0.3
(186 days with comed[2])		0.164	9.3	0.150	0.0	37.1	-1.1	38.9	3.7
		#0.176	17.3	0.160	6.7	35.6	-5.1	38.3	2.1
		0.164	9.3	0.161	7.3	36.9	-1.6	38.1	1.6
		0.160	6.7	0.157	4.7	36.5	-2.7	37.7	0.5
Intrarun Mean		0.165		0.160		36.2		38.0	
Intrarun SD		0.00613		0.00568		1.06		0.574	
Intrarun %CV		3.7		3.6		2.9		1.5	
Intrarun %Bias		10.0		6.7		-3.5		1.3	
n		6		6		6		6	
# > 15%Bias									

Criteria: To be deemed stable, the mean %Bias at each level must be within ± 15.0% with %CV ≤ 15.0%.

Results: Ertapenem is stable in treated human plasma ultrafiltrate with and without presence comed[2], with sodium heparin as anticoagulant, for up to 186 days (with comed[2]) and 187 days (without comed[2]) upon storage in polypropylene tube at -70 °C. See Section 10.4 for reason for run 1 stability failure.

MedChemExpress

Certificate of Analysis

Inhibitors • Screening Libraries • Proteins

Ertapenem sodium

Cat. No.:	HY-13625
CAS No.:	153773-82-1
Batch No.:	180017
Chemical Name:	1-Azabicyclo[3.2.0]hept-2-ene-2-carboxylic acid, 3-[[(3S,5S)-5-[[(3-carboxyphenyl)amino]carbonyl]-3-pyrrolidinyl]thio]-6-[(1R)-1-hydroxyethyl]-4-methyl-7-oxo-, sodium salt (1:1), (4R,5S,6S)-

PHYSICAL AND CHEMICAL PROPERTIES

Molecular Formula:	$C_{22}H_{24}N_3NaO_7S$
Molecular Weight:	**497.50**
Storage:	4°C, sealed storage, away from moisture<br>* In solvent : -80°C, 6 months; -20°C, 1 month (sealed storage, away from moisture)

Chemical Structure:

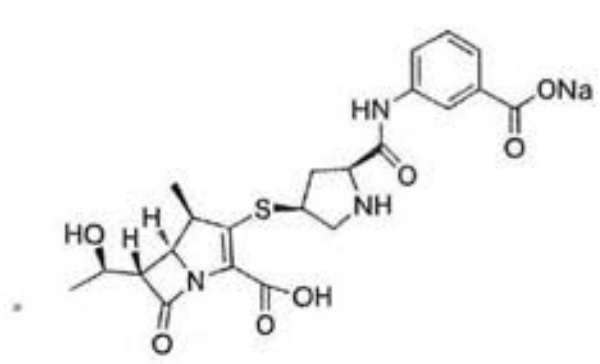

ANALYTICAL DATA

Appearance:	White to light yellow (Solid)
^{1}H NMR Spectrum:	Consistent with structure
LCMS:	Consistent with structure
Purity (LCMS):	**98.86%**
Test date:	2022/08/12
Retest date:	2024/08/11
Conclusion:	The product has been tested and complies with the given specifications.

Name: Lingli Ma
Designation: QA Manager
Date: 6/27/2022

Caution: Product has not been fully validated for medical applications. For research use only.

Tel: 609-228-6898 Fax: 609-228-5909 E-mail: tech@MedChemExpress.com
Address: 1 Deer Park Dr, Suite Q, Monmouth Junction, NJ 08852, USA

Ertapenem sodium

Cat. No.:	HY-13625
CAS No.:	153773-82-1
Molecular Formula:	$C_{22}H_{24}N_3NaO_7S$
Molecular Weight:	497.5
Target:	Bacterial; Antibiotic
Pathway:	Anti-infection
Storage:	4°C, sealed storage, away from moisture<br>* In solvent : -80°C, 6 months; -20°C, 1 month (sealed storage, away from moisture)

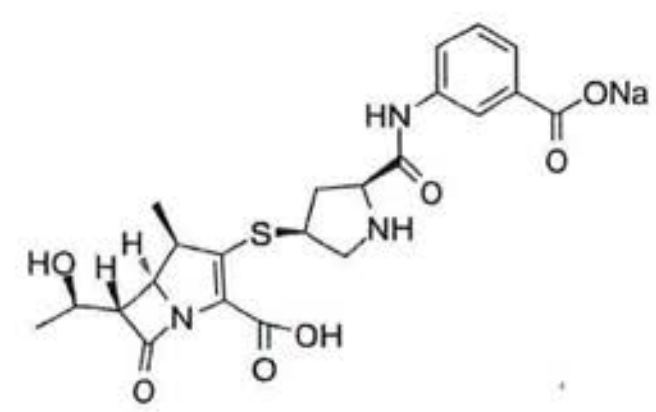

SOLVENT & SOLUBILITY

In Vitro

H_2O : 50 mg/mL (100.50 mM; Need ultrasonic)

	Solvent / Mass / Concentration	1 mg	5 mg	10 mg
Preparing Stock Solutions	1 mM	2.0101 mL	10.0503 mL	20.1005 mL
	5 mM	0.4020 mL	2.0101 mL	4.0201 mL
	10 mM	0.2010 mL	1.0050 mL	2.0101 mL

Please refer to the solubility information to select the appropriate solvent.

BIOLOGICAL ACTIVITY

Description

Ertapenem sodium (L-749345) is a broad spectrum and long acting β-lactam antibiotic. Ertapenem sodium has a broad-spectrum anti-anaerobic activity against a variety of anaerobes with a mode MIC of 0.12 μg/mL. Ertapenem sodium can be used for the research of severe infections caused by bacteria in the skin, lungs, stomach, pelvis, and urinary tract[1][2].

In Vitro

Ertapenem sodium (0-100 μg/mL approximately, 48 h) is active against 99.1% of all anaerobes with a mode MIC of 0.12 μg/mL and MIC_{90} of 1 μg/mL, and MIC's ≥8 μg/mL for B.fragilis and B.vulgatus species, respectively[1].

MCE has not independently confirmed the accuracy of these methods. They are for reference only.

Cell Viability Assay[1]

Cell Line:	B. fragilis (ATCC 25285), B. thetaiotaomicron (ATCC 29741), and Eubacterium lentum (ATCC 43055)
Concentration:	0-100 μg/mL approximately
Incubation Time:	48 h
Result:	Inhibited 99.1% of all isolate with a mode MIC of 0.12 μg/mL and MIC_{90} of 1 μg/mL, and

98.8% of the isolates were susceptible among the B. fragilis group.

In Vivo

Ertapenem sodium (Subcutaneous injection, 0-10 mg/kg, 0-120 h after infection, S. aureus thigh tissue infection model) shows > 3 $\log_{10}$ CFU reduction of organism at 10 mg/kg, and maintains the activity with 3.3 and 4.4 $\log_{10}$ CFU eliminated at 2 mg/kg[2].

Ertapenem sodium (Subcutaneous injection, 4h after infection, systemic infection model) is active against all gram-positive organisms, and is also active against gram-negative organisms tested with ED_{50}s of <0.25 mg/kg/dose[2].

MCE has not independently confirmed the accuracy of these methods. They are for reference only.

Animal Model:	S. aureus thigh tissue infection model (DBA/2 mice)[2]
Dosage:	0.5,1, 2, 5, 10 mg/kg (given at 2, 6, 10, 24, 48, 72, 96, 120 h)
Administration:	Subcutaneous injection (0.5 mL after infection)
Result:	Displayed > 3 $\log_{10}$ CFU reduction of organism compared to non-antibiotic-treated controls at 10 mg/kg.<br>Maintained the activity with 3.3 and 4.4 $\log_{10}$ CFU eliminated at 2 mg/kg.

Animal Model:	Systemic infection model (DBA/2 female mice, viral antibody-free CD-1 female mice)[2]
Dosage:	0-3 mg/kg approximately
Administration:	Subcutaneous injection (0.5 mL, begin immediately and 4 h after infection)
Result:	Showed activity against all gram-positive organisms, and also ram-negative organisms tested with ED_{50}s of <0.25 mg/kg/dose.

Animal Model:	CD-1 mice, rats[2]
Dosage:	10 mg/kg approximately
Administration:	Intraperitoneal injection (pharmacokinetic assay)
Result:	Exhibited an $AUC_{0-\infty}$ ranging from 1.8-21.82 µg•hr/mL in tissue in mice following a 10-mg/kg i.p. dose.<br>Exhibited slow clearance rate with a $t_{1/2\beta}$ of 3.2 h, Clp of 0.47 mL/min/kg, AUC_{0-8} of 284.15 µg•hr/mL.

CUSTOMER VALIDATION

- Nat Commun. 2022 Mar 2;13(1):1116.
- Nat Commun. 2021 Jul 22;12(1):4461.
- J Antimicrob Chemother. 2020 Jul 1;75(7):1850-1858.
- Biomed Res Int. 2018 Jul 2;2018:3579832.

See more customer validations on www.MedChemExpress.com

REFERENCES

[1]. Kenneth E Aldridge. Ertapenem (MK-0826), a new carbapenem: comparative in vitro activity against clinically significant anaerobes. Diagn Microbiol Infect Dis. 2002 Oct;44(2):181-6.

[2]. C J Gill, et al. In vivo activity and pharmacokinetic evaluation of a novel long-acting carbapenem antibiotic, MK-826 (L-749,345). Antimicrob Agents Chemother. 1998 Aug;42(8):1996-2001.

Caution: Product has not been fully validated for medical applications. For research use only.

Tel: 609-228-6898 Fax: 609-228-5909 E-mail: tech@MedChemExpress.com

Address: 1 Deer Park Dr, Suite Q, Monmouth Junction, NJ 08852, USA

MCE® MedChemExpress

Safety Data Sheet

Revision Date: Aug.-15-2022
Print Date: Aug.-18-2022

1. PRODUCT AND COMPANY IDENTIFICATION

1.1 Product identifier

Product name :	Ertapenem sodium
Catalog No. :	HY-13625
CAS No. :	153773-82-1

1.2 Relevant identified uses of the substance or mixture and uses advised against

Identified uses :	Laboratory chemicals, manufacture of substances.

1.3 Details of the supplier of the safety data sheet

Company:	MedChemExpress USA
Tel:	609-228-6898
Fax:	609-228-5909
E-mail:	sales@medchemexpress.com

1.4 Emergency telephone number

Emergency Phone #:	609-228-6898

2. HAZARDS IDENTIFICATION

2.1 Classification of the substance or mixture

Not a hazardous substance or mixture.

2.2 GHS Label elements, including precautionary statements

Not a hazardous substance or mixture.

2.3 Other hazards

None.

3. COMPOSITION/INFORMATION ON INGREDIENTS

3.1 Substances

Synonyms:	L-749345; MK-826
Formula:	$C_{22}H_{24}N_3NaO_7S$
Molecular Weight:	497.50
CAS No. :	153773-82-1

4. FIRST AID MEASURES

4.1 Description of first aid measures

Eye contact

Remove any contact lenses, locate eye-wash station, and flush eyes immediately with large amounts of water. Separate eyelids with fingers to ensure adequate flushing. Promptly call a physician.

Skin contact

Rinse skin thoroughly with large amounts of water. Remove contaminated clothing and shoes and call a physician.

Inhalation

Immediately relocate self or casualty to fresh air. If breathing is difficult, give cardiopulmonary resuscitation (CPR). Avoid mouth-to-mouth resuscitation.

Ingestion

Wash out mouth with water; Do NOT induce vomiting; call a physician.

4.2 Most important symptoms and effects, both acute and delayed

The most important known symptoms and effects are described in the labelling (see section 2.2).

4.3 Indication of any immediate medical attention and special treatment needed

Treat symptomatically.

5. FIRE FIGHTING MEASURES

5.1 Extinguishing media

Suitable extinguishing media

Use water spray, dry chemical, foam, and carbon dioxide fire extinguisher.

5.2 Special hazards arising from the substance or mixture

During combustion, may emit irritant fumes.

5.3 Advice for firefighters

Wear self-contained breathing apparatus and protective clothing.

6. ACCIDENTAL RELEASE MEASURES

6.1 Personal precautions, protective equipment and emergency procedures

Use full personal protective equipment. Avoid breathing vapors, mist, dust or gas. Ensure adequate ventilation. Evacuate personnel to safe areas.

Refer to protective measures listed in sections 8.

6.2 Environmental precautions

Try to prevent further leakage or spillage. Keep the product away from drains or water courses.

6.3 Methods and materials for containment and cleaning up

Absorb solutions with finely-powdered liquid-binding material (diatomite, universal binders); Decontaminate surfaces and equipment by scrubbing with alcohol; Dispose of contaminated material according to Section 13.

7. HANDLING AND STORAGE

7.1 Precautions for safe handling

Avoid inhalation, contact with eyes and skin. Avoid dust and aerosol formation. Use only in areas with appropriate exhaust ventilation.

7.2 Conditions for safe storage, including any incompatibilities

Keep container tightly sealed in cool, well-ventilated area. Keep away from direct sunlight and sources of ignition.

Recommended storage temperature: 4°C, sealed storage, away from moisture

* In solvent : -80°C, 6 months; -20°C, 1 month (sealed storage, away from moisture)

Shipping at room temperature if less than 2 weeks.

7.3 Specific end use(s)

No data available.

8. EXPOSURE CONTROLS/PERSONAL PROTECTION

8.1 Control parameters

Components with workplace control parameters

This product contains no substances with occupational exposure limit values.

8.2 Exposure controls

Engineering controls

Ensure adequate ventilation. Provide accessible safety shower and eye wash station.

Personal protective equipment

**Eye protection**	Safety goggles with side-shields.
**Hand protection**	Protective gloves.
**Skin and body protection**	Impervious clothing.
**Respiratory protection**	Suitable respirator.
**Environmental exposure controls**	Keep the product away from drains, water courses or the soil. Clean spillages in a safe way as soon as possible.

9. PHYSICAL AND CHEMICAL PROPERTIES

9.1 Information on basic physical and chemical properties

**Appearance**	Solid
**Odor**	No data available
**Odor threshold**	No data available
**pH**	No data available
**Melting/freezing point**	No data available
**Boiling point/range**	No data available
**Flash point**	No data available
**Evaporation rate**	No data available
**Flammability (solid, gas)**	No data available
**Upper/lower flammability or explosive limits**	No data available
**Vapor pressure**	No data available
**Vapor density**	No data available
**Relative density**	No data available
**Water Solubility**	No data available
**Partition coefficient**	No data available
**Auto-ignition temperature**	No data available
**Decomposition temperature**	No data available
**Viscosity**	No data available
**Explosive properties**	No data available
**Oxidizing properties**	No data available

9.2 Other safety information

No data available.

10. STABILITY AND REACTIVITY

10.1 Reactivity

No data available.

10.2 Chemical stability

Stable under recommended storage conditions.

10.3 Possibility of hazardous reactions

No data available.

10.4 Conditions to avoid

No data available.

10.5 Incompatible materials

Strong acids/alkalis, strong oxidising/reducing agents.

10.6 Hazardous decomposition products

Under fire conditions, may decompose and emit toxic fumes.

Other decomposition products - no data available.

11.TOXICOLOGICAL INFORMATION

11.1 Information on toxicological effects

Acute toxicity

Classified based on available data. For more details, see section 2

Skin corrosion/irritation

Classified based on available data. For more details, see section 2

Serious eye damage/irritation

Classified based on available data. For more details, see section 2

Respiratory or skin sensitization

Classified based on available data. For more details, see section 2

Germ cell mutagenicity

Classified based on available data. For more details, see section 2

Carcinogenicity

IARC: No component of this product present at a level equal to or greater than 0.1% is identified as probable, possible or confirmed human carcinogen by IARC.

ACGIH: No component of this product present at a level equal to or greater than 0.1% is identified as a potential or confirmed carcinogen by ACGIH.

NTP: No component of this product present at a level equal to or greater than 0.1% is identified as a anticipated or confirmed carcinogen by NTP.

OSHA: No component of this product present at a level equal to or greater than 0.1% is identified as a potential or confirmed carcinogen by OSHA.

Reproductive toxicity

Classified based on available data. For more details, see section 2

Specific target organ toxicity - single exposure

Classified based on available data. For more details, see section 2

Specific target organ toxicity - repeated exposure

Classified based on available data. For more details, see section 2

Aspiration hazard

Classified based on available data. For more details, see section 2

Additional information

This information is based on our current knowledge. However the chemical, physical, and toxicological properties have not been completely investigated.

12. ECOLOGICAL INFORMATION

12.1 Toxicity

No data available.

12.2 Persistence and degradability

No data available.

12.3 Bioaccumlative potential

No data available.

12.4 Mobility in soil

No data available.

12.5 Results of PBT and vPvB assessment

PBT/vPvB assessment unavailable as chemical safety assessment not required or not conducted.

12.6 Other adverse effects

No data available.

13. DISPOSAL CONSIDERATIONS

13.1 Waste treatment methods

Product

Dispose substance in accordance with prevailing country, federal, state and local regulations.

Contaminated packaging

Conduct recycling or disposal in accordance with prevailing country, federal, state and local regulations.

14. TRANSPORT INFORMATION

DOT (US)

Proper shipping name: Not dangerous goods

UN number: -

Class: -

Packing group: -

IMDG

Proper shipping name: Not dangerous goods

UN number: -

Class: -

Packing group: -

IATA

Proper shipping name: Not dangerous goods

UN number: -

Class: -

Packing group: -

15. REGULATORY INFORMATION

SARA 302 Components:

No chemicals in this material are subject to the reporting requirements of SARA Title III, Section 302.

SARA 313 Components:

This material does not contain any chemical components with known CAS numbers that exceed the threshold (De Minimis) reporting levels established by SARA Title III, Section 313.

SARA 311/312 Hazards:

No SARA Hazards.

Massachusetts Right To Know Components:

No components are subject to the Massachusetts Right to Know Act.

Pennsylvania Right To Know Components:

No components are subject to the Pennsylvania Right to Know Act.

New Jersey Right To Know Components:

No components are subject to the New Jersey Right to Know Act.

California Prop. 65 Components:

This product does not contain any chemicals known to State of California to cause cancer, birth defects, or anyother reproductive harm.

16. OTHER INFORMATION

Copyright 2022 MedChemExpress. The above information is correct to the best of our present knowledge but does not purport to be all inclusive and should be used only as a guide. The product is for research use only and for experienced personnel. It must only be handled by suitably qualified experienced scientists in appropriately equipped and authorized facilities. The burden of safe use of this material rests entirely with the user. MedChemExpress disclaims all liability for any damage resulting from handling or from contact with this product.

SAMPLE INFORMATION

CAS NO.:	153773-82-1	Acquired By:	LY
Sample infromation:		Date Acquired:	8/12/2022 2:04:10 PM CST
Vial:	2:A,3	Acq. Method Set:	1_POS_2MIN
Injection #:	1	Date Processed:	8/12/2022 2:36:50 PM CST,
Injection Volume:	0.30 ul	Processing Method:	PDA_01, MS_scan
Run Time:	2.8 Minutes	Channel Name:	PDA Ch1 214nm@4.8nm, MS TIC,
Sample Set Name:	20220812	Column Name:	A-RP-985

Auto-Scaled Chromatogram

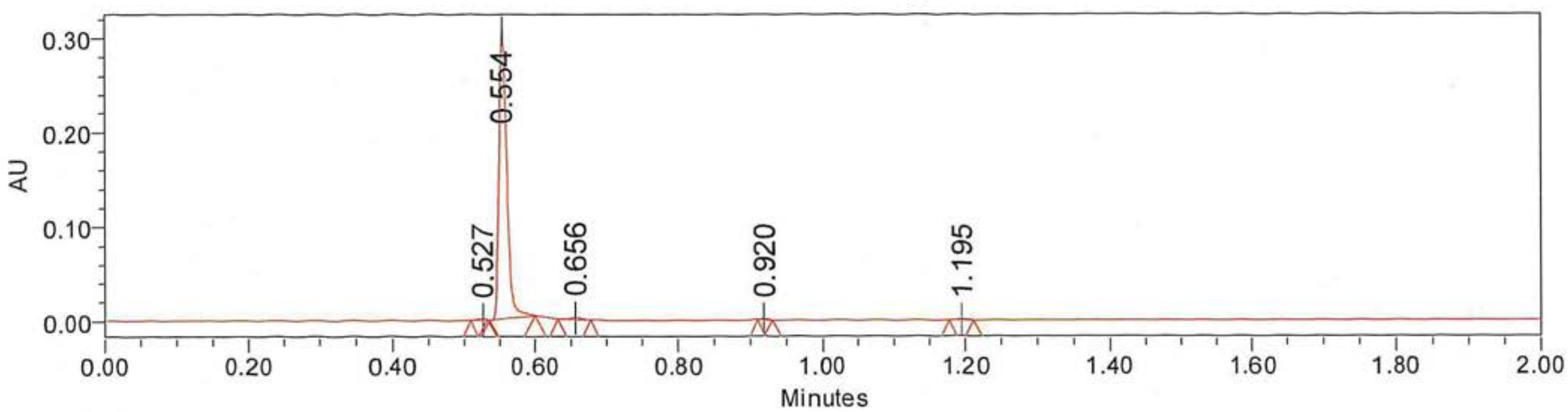

Processed Channel Descr. PDA Ch2 254nm@4.8nm

Auto-Scaled Chromatogram

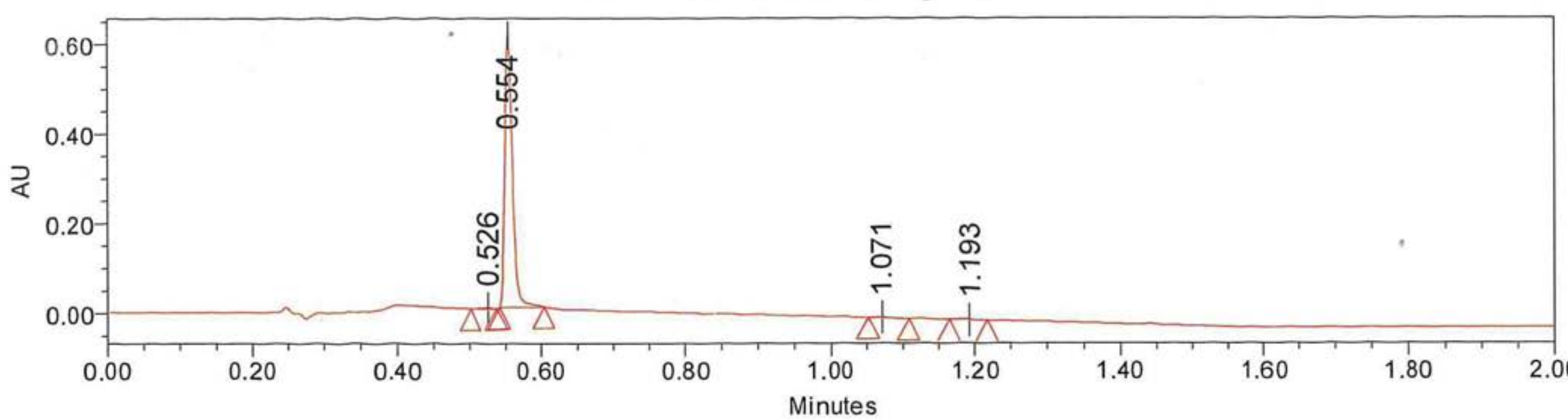

Processed Channel Descr. PDA Ch1 214nm@4.8nm

Auto-Scaled Chromatogram

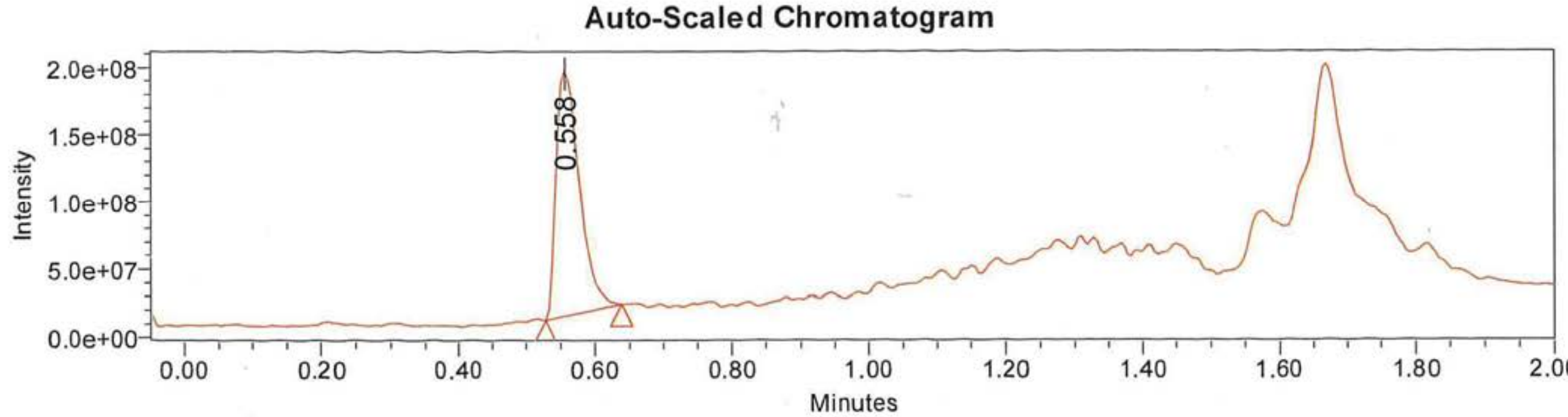

Processed Channel Descr. ACQ-SQD2 1: MS Scan MS TIC, Smoothed by 9 point Savitzky-Golay Filter., Time offset by -0.053 mins. (1: 150.00-1000.00 ES+, Centroid, CV=30)

Peak Results
Channel Name: PDA Ch2 254nm@4.8nm

	RT	Width (sec)	Area	Height	% Area	Base Peak (Combined) (m/z)	Channel Name
1	0.527	1.400	477	640	0.20	153.83	PDA Ch2 254nm@4.8nm
2	0.554	3.800	237134	308413	98.19	151.96	PDA Ch2 254nm@4.8nm
3	0.656	2.650	2446	2436	1.01	151.06	PDA Ch2 254nm@4.8nm
4	0.920	1.200	632	1080	0.26	152.07	PDA Ch2 254nm@4.8nm
5	1.195	2.050	823	941	0.34	152.07	PDA Ch2 254nm@4.8nm

Peak Results
Channel Name: PDA Ch1 214nm@4.8nm

	RT	Width (sec)	Area	Height	% Area	Base Peak (Combined) (m/z)	Channel Name
1	0.526	2.150	1796	2101	0.38	153.84	PDA Ch1 214nm@4.8nm
2	0.554	3.700	471358	613577	98.86	151.97	PDA Ch1 214nm@4.8nm
3	1.071	3.350	1629	1281	0.34	151.03	PDA Ch1 214nm@4.8nm
4	1.193	3.150	1997	1243	0.42	152.07	PDA Ch1 214nm@4.8nm

Peak Results
Channel Name: MS TIC

	RT	Width (sec)	Area	Height	% Area	Base Peak (Combined) (m/z)	Channel Name
1	0.558	6.673	431532509	182380390	100.00	476.14	MS TIC

Match Plot

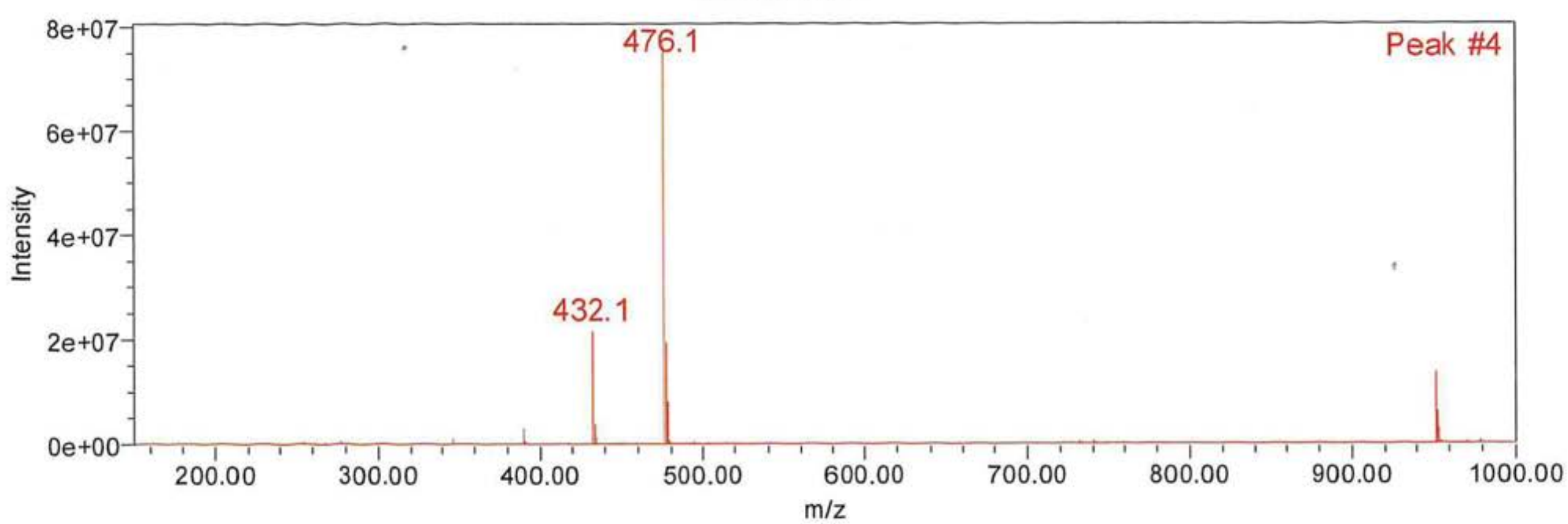

Retention Time 0.558 Channel Name MS TIC

CAS NO.: 153773-82-1, D2O

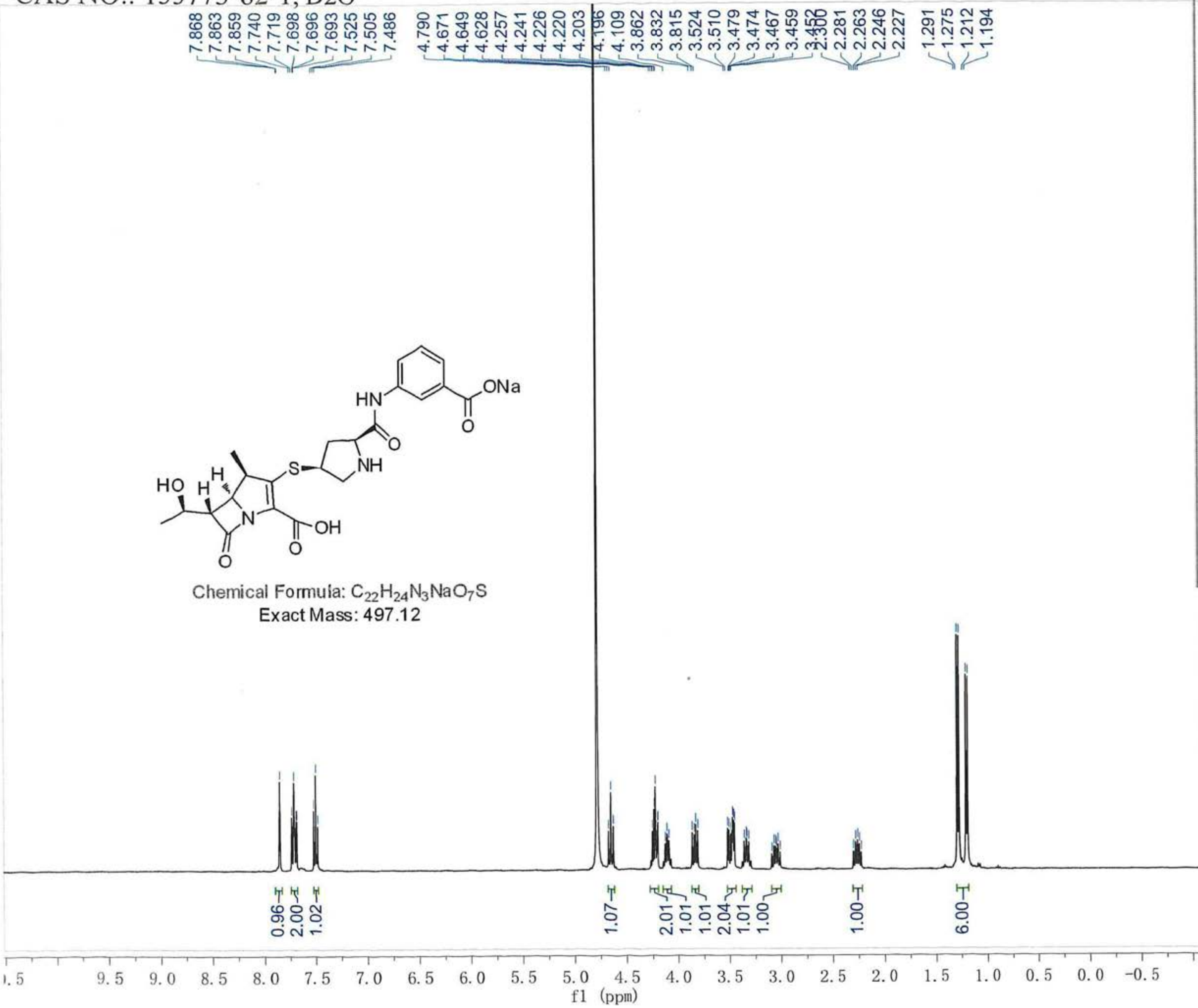

Chemical Formula: $C_{22}H_{24}N_3NaO_7S$
Exact Mass: 497.12

	Parameter	Value
1	Title	
2	Origin	Bruker BioSpin GmbH
3	Owner	nmrsu
4	Spectrometer	Avance
5	Author	
6	Solvent	D2O
7	Temperature	298.2
8	Pulse Sequence	zg30
9	Experiment	1D
10	Number of Scans	8
11	Receiver Gain	101
12	Relaxation Delay	1.0000
13	Pulse Width	8.0000
14	Acquisition Time	3.9977
15	Acquisition Date	2022-08-10T13:49:06
16	Modification Date	2022-08-10T13:53:00
17	Spectrometer Frequency	400.13
18	Nucleus	1H

MCE MedChem Express

PO: 42461

Packing List

From:

MedChemExpress LLC
1 Deer Park Dr, Suite Q,
Monmouth Junction,
NJ 08852, USA
Tel: 609-228-6898
Fax: 609-228-5909
E-mail: sales@medchemexpress.com

To:

KCAS Bioanalytical and Biomarker Services
Contact Name: Receiving
10830 S. Clay Blair Blvd
Olathe Kansas 66061
United States
Tel:
Email:

Purchase PO No.:
PO42461
Customer-Ref No.:

Invoice No.:
M2225195
Date of Issue:
Sept-14-2022
Ship Date:
Sept-20-2022

#	Cat. No.	Product Name	Batch	Size	Quantity
1	HY-13625	Ertapenem sodium	180017	10 g	1
2	Technical documentation for listed products				1

RECEIVED
SEP 21 REC'D
By

Total: 2

1. Packages and products should be inspected immediately upon receipt.
2. Please notify us immediately of any damage, error in quantity, or product defects by email or fax.
3. If you are not satisfied with our products, you may return or exchange any items within 30 days of receiving the products.

Thank You for Selecting MCE!

MedChemExpress

Certificate of Analysis

Inhibitors • Screening Libraries • Proteins

Ertapenem sodium

Cat. No.: HY-13625

CAS No.: 153773-82-1

Batch No.: 229333

Chemical Name: 1-Azabicyclo[3.2.0]hept-2-ene-2-carboxylic acid, 3-[[(3S,5S)-5-[[(3-carboxyphenyl)amino]carbonyl]-3-pyrrolidinyl]thio]-6-[(1R)-1-hydroxyethyl]-4-methyl-7-oxo-, sodium salt (1:1), (4R,5S,6S)-

PHYSICAL AND CHEMICAL PROPERTIES

Molecular Formula: $C_{22}H_{24}N_3NaO_7S$

Molecular Weight: **497.50**

Storage: 4°C, sealed storage, away from moisture

* In solvent : -80°C, 6 months; -20°C, 1 month (sealed storage, away from moisture)

Test Date: 2022.09.30

Retest Date: 2025.09.29

Chemical Structure:

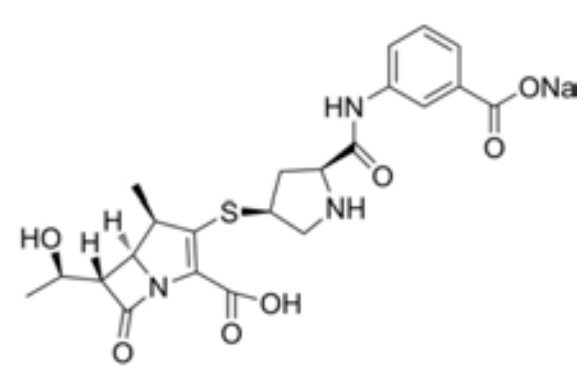

ANALYTICAL DATA

Appearance: White to light yellow (Solid)

[1] H NMR Spectrum: Consistent with structure

LCMS: Consistent with structure

Purity (LCMS): **98.43%**

Conclusion: The product has been tested and complies with the given specifications.

Name: Lingli Ma

Designation: QA Manager

Date: 2/28/2023

Caution: Product has not been fully validated for medical applications. For research use only.

Tel: 609-228-6898 Fax: 609-228-5909 E-mail: tech@MedChemExpress.com

Address: 1 Deer Park Dr, Suite Q, Monmouth Junction, NJ 08852, USA

Safety Data Sheet

Revision Date: Aug.-15-2022
Print Date: Jan.-23-2023

1. PRODUCT AND COMPANY IDENTIFICATION

1.1 Product identifier

Product name :	Ertapenem sodium
Catalog No. :	HY-13625
CAS No. :	153773-82-1

1.2 Relevant identified uses of the substance or mixture and uses advised against

Identified uses :	Laboratory chemicals, manufacture of substances.

1.3 Details of the supplier of the safety data sheet

Company:	MedChemExpress USA
Tel:	609-228-6898
Fax:	609-228-5909
E-mail:	sales@medchemexpress.com

1.4 Emergency telephone number

Emergency Phone #:	609-228-6898

2. HAZARDS IDENTIFICATION

2.1 Classification of the substance or mixture

Not a hazardous substance or mixture.

2.2 GHS Label elements, including precautionary statements

Not a hazardous substance or mixture.

2.3 Other hazards

None.

3. COMPOSITION/INFORMATION ON INGREDIENTS

3.1 Substances

Synonyms:	L-749345; MK-826
Formula:	$C_{22}H_{24}N_3NaO_7S$
Molecular Weight:	497.50
CAS No. :	153773-82-1

4. FIRST AID MEASURES

4.1 Description of first aid measures

Eye contact

Remove any contact lenses, locate eye-wash station, and flush eyes immediately with large amounts of water. Separate eyelids with fingers to ensure adequate flushing. Promptly call a physician.

Skin contact

Rinse skin thoroughly with large amounts of water. Remove contaminated clothing and shoes and call a physician.

Inhalation

Immediately relocate self or casualty to fresh air. If breathing is difficult, give cardiopulmonary resuscitation (CPR). Avoid mouth-to-mouth resuscitation.

Ingestion

Wash out mouth with water; Do NOT induce vomiting; call a physician.

4.2 Most important symptoms and effects, both acute and delayed

The most important known symptoms and effects are described in the labelling (see section 2.2).

4.3 Indication of any immediate medical attention and special treatment needed

Treat symptomatically.

5. FIRE FIGHTING MEASURES

5.1 Extinguishing media

Suitable extinguishing media

Use water spray, dry chemical, foam, and carbon dioxide fire extinguisher.

5.2 Special hazards arising from the substance or mixture

During combustion, may emit irritant fumes.

5.3 Advice for firefighters

Wear self-contained breathing apparatus and protective clothing.

6. ACCIDENTAL RELEASE MEASURES

6.1 Personal precautions, protective equipment and emergency procedures

Use full personal protective equipment. Avoid breathing vapors, mist, dust or gas. Ensure adequate ventilation. Evacuate personnel to safe areas.

Refer to protective measures listed in sections 8.

6.2 Environmental precautions

Try to prevent further leakage or spillage. Keep the product away from drains or water courses.

6.3 Methods and materials for containment and cleaning up

Absorb solutions with finely-powdered liquid-binding material (diatomite, universal binders); Decontaminate surfaces and equipment by scrubbing with alcohol; Dispose of contaminated material according to Section 13.

7. HANDLING AND STORAGE

7.1 Precautions for safe handling

Avoid inhalation, contact with eyes and skin. Avoid dust and aerosol formation. Use only in areas with appropriate exhaust ventilation.

7.2 Conditions for safe storage, including any incompatibilities

Keep container tightly sealed in cool, well-ventilated area. Keep away from direct sunlight and sources of ignition.

Recommended storage temperature: 4°C, sealed storage, away from moisture

* In solvent : -80°C, 6 months; -20°C, 1 month (sealed storage, away from moisture)

Shipping at room temperature if less than 2 weeks.

7.3 Specific end use(s)

No data available.

8. EXPOSURE CONTROLS/PERSONAL PROTECTION

8.1 Control parameters

Components with workplace control parameters

This product contains no substances with occupational exposure limit values.

8.2 Exposure controls

Engineering controls

Ensure adequate ventilation. Provide accessible safety shower and eye wash station.

Personal protective equipment

**Eye protection**	Safety goggles with side-shields.
**Hand protection**	Protective gloves.
**Skin and body protection**	Impervious clothing.
**Respiratory protection**	Suitable respirator.
**Environmental exposure controls**	Keep the product away from drains, water courses or the soil. Clean spillages in a safe way as soon as possible.

9. PHYSICAL AND CHEMICAL PROPERTIES

9.1 Information on basic physical and chemical properties

**Appearance**	Solid
**Odor**	No data available
**Odor threshold**	No data available
**pH**	No data available
**Melting/freezing point**	No data available
**Boiling point/range**	No data available
**Flash point**	No data available
**Evaporation rate**	No data available
**Flammability (solid, gas)**	No data available
**Upper/lower flammability or explosive limits**	No data available
**Vapor pressure**	No data available
**Vapor density**	No data available
**Relative density**	No data available
**Water Solubility**	No data available
**Partition coefficient**	No data available
**Auto-ignition temperature**	No data available
**Decomposition temperature**	No data available
**Viscosity**	No data available
**Explosive properties**	No data available
**Oxidizing properties**	No data available

9.2 Other safety information

No data available.

10. STABILITY AND REACTIVITY

10.1 Reactivity

No data available.

10.2 Chemical stability

Stable under recommended storage conditions.

10.3 Possibility of hazardous reactions

No data available.

10.4 Conditions to avoid

No data available.

10.5 Incompatible materials

Strong acids/alkalis, strong oxidising/reducing agents.

10.6 Hazardous decomposition products

Under fire conditions, may decompose and emit toxic fumes.

Other decomposition products - no data available.

11.TOXICOLOGICAL INFORMATION

11.1 Information on toxicological effects

Acute toxicity

Classified based on available data. For more details, see section 2

Skin corrosion/irritation

Classified based on available data. For more details, see section 2

Serious eye damage/irritation

Classified based on available data. For more details, see section 2

Respiratory or skin sensitization

Classified based on available data. For more details, see section 2

Germ cell mutagenicity

Classified based on available data. For more details, see section 2

Carcinogenicity

IARC: No component of this product present at a level equal to or greater than 0.1% is identified as probable, possible or confirmed human carcinogen by IARC.

ACGIH: No component of this product present at a level equal to or greater than 0.1% is identified as a potential or confirmed carcinogen by ACGIH.

NTP: No component of this product present at a level equal to or greater than 0.1% is identified as a anticipated or confirmed carcinogen by NTP.

OSHA: No component of this product present at a level equal to or greater than 0.1% is identified as a potential or confirmed carcinogen by OSHA.

Reproductive toxicity

Classified based on available data. For more details, see section 2

Specific target organ toxicity - single exposure

Classified based on available data. For more details, see section 2

Specific target organ toxicity - repeated exposure

Classified based on available data. For more details, see section 2

Aspiration hazard

Classified based on available data. For more details, see section 2

Additional information

This information is based on our current knowledge. However the chemical, physical, and toxicological properties have not been completely investigated.

12. ECOLOGICAL INFORMATION

12.1 Toxicity

No data available.

12.2 Persistence and degradability

No data available.

12.3 Bioaccumlative potential

No data available.

12.4 Mobility in soil

No data available.

12.5 Results of PBT and vPvB assessment

PBT/vPvB assessment unavailable as chemical safety assessment not required or not conducted.

12.6 Other adverse effects

No data available.

13. DISPOSAL CONSIDERATIONS

13.1 Waste treatment methods

Product

Dispose substance in accordance with prevailing country, federal, state and local regulations.

Contaminated packaging

Conduct recycling or disposal in accordance with prevailing country, federal, state and local regulations.

14. TRANSPORT INFORMATION

DOT (US)

Proper shipping name: Not dangerous goods

UN number: -

Class: -

Packing group: -

IMDG

Proper shipping name: Not dangerous goods

UN number: -

Class: -

Packing group: -

IATA

Proper shipping name: Not dangerous goods

UN number: -

Class: -

Packing group: -

15. REGULATORY INFORMATION

SARA 302 Components:

No chemicals in this material are subject to the reporting requirements of SARA Title III, Section 302.

SARA 313 Components:

This material does not contain any chemical components with known CAS numbers that exceed the threshold (De Minimis) reporting levels established by SARA Title III, Section 313.

SARA 311/312 Hazards:

No SARA Hazards.

Massachusetts Right To Know Components:

No components are subject to the Massachusetts Right to Know Act.

Pennsylvania Right To Know Components:

No components are subject to the Pennsylvania Right to Know Act.

New Jersey Right To Know Components:

No components are subject to the New Jersey Right to Know Act.

California Prop. 65 Components:

This product does not contain any chemicals known to State of California to cause cancer, birth defects, or anyother reproductive harm.

16. OTHER INFORMATION

Copyright 2023 MedChemExpress. The above information is correct to the best of our present knowledge but does not purport to be all inclusive and should be used only as a guide. The product is for research use only and for experienced personnel. It must only be handled by suitably qualified experienced scientists in appropriately equipped and authorized facilities. The burden of safe use of this material rests entirely with the user. MedChemExpress disclaims all liability for any damage resulting from handling or from contact with this product.

CERTIFICATE OF ANALYSIS

ALSACHIM
a Shimadzu Group Company

**Product Name:**	[$^2H_4$]-Ertapenem sodium salt
**Molecular Formula:**	$C_{22}H_{24}N_3O_7S$. Na
**Labeled Molecular Formula:**	$C_{22}H_{20}D_4N_3O_7S$. Na
**Molecular Weight:**	501.52 g/mol
**Structure:**	

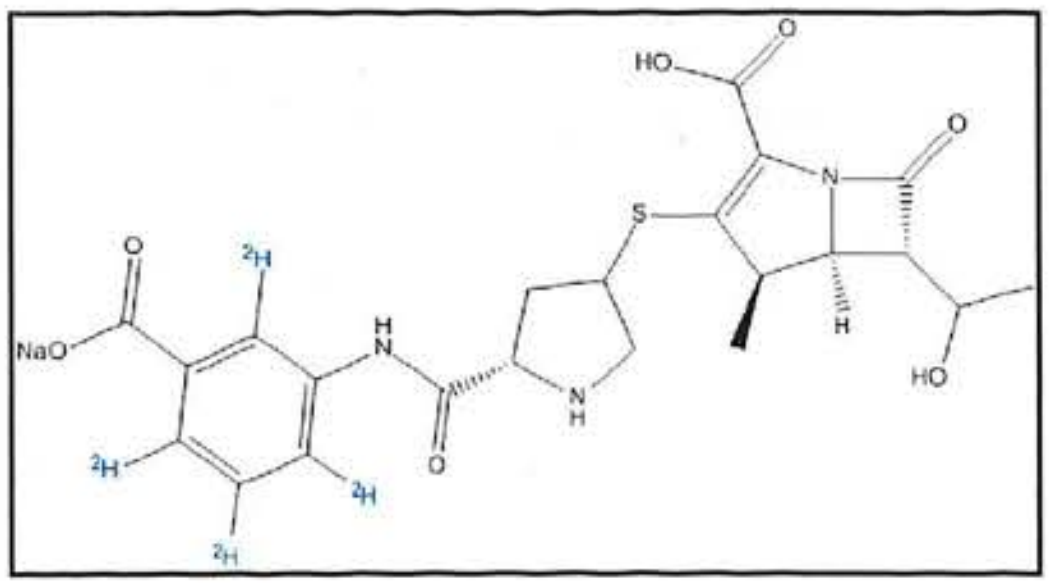

**Batch Number:**	DH-ALS-20-002-P2	
**Product Number:**	C3376	
**CAS Number:**	153773-82-1 unlabeled	
**Storage:**	For a prolonged period, keep at -20°C (±5°C) in a closed container, away from light and moisture	
**Stability:**	Stable (12 months) (*)	
**Extended expiring date:**	Saturday April 29, 2023	
**Solvent solubility:**	Soluble in Water or Acetonitrile/Water (1/1)	
	**Specification:**	**Results:**
**Appearance:**	Pale grey to white solid	Off white solid
**Chemical Purity (HPLC):**	≥ 80.0%	89,4%
**Isotopic Enrichment:**	≥ 98.0% ^2H	99.0% ^2H
**Unlabeled (M+0) Content:**	Unlabeled (M+0) content < 0.5%	<0.5%
**Mass Spectrometry:**	Conform to structure	Conforms
**Manufacturing date:**	Tuesday January 7, 2020	
**Reanalysis date:**	Friday April 29, 2022	
**Note:**	For research and development use only, not for use in humans.	

Quality Review & Approval

Francis ROESSLINGER, Quality Manager
Date : Wednesday, May 18, 2022

RECEIVED
SEP 21 REC'D
By

ALSACHIM
a Shimadzu Group Company

Analysis Report

<Equipment Information>

LCMS Shimadzu

Alsachim Reference: AN-LC01 + AN-MS01

Pump : LC-40D

Degassing Unit : DGU-405

Auto Sampler : SIL-40C

Diode Array Detector : SPD-M40

Column Oven : CTO-40S

Valve Unit : FCV-20AH2

Liquid Chromatograph Mass Spectrometer : LCMS-2020

Column : Accucore C18 50*4.6 2.6um PN: 17126-054630 SN: 20333623 LOT: 20512

<Sample Information>

Sample Name : DH-ALS-20-002-P2
Data Filename : DH-ALS-20-002-P2_002.lcd
Method Filename : HP-C18-AF-20MIN.lcm
Batch Filename : 2022-APR-29-3.lcb
Vial # : 2-20
Injection Volume : 0,5 uL
Date Acquired : 29/04/2022 14:31:49
Date Processed : 02/05/2022 08:59:47
Solvent : Water
Concentration : 1 mg/ml
Operator : CD

<Peak Table >

PDA Ch1 295nm

Peak#	Ret. Time	Area	Height	Area%
1	1,861	2224	1305	0,544
2	2,364	16348	3642	4,001
3	2,684	365589	157196	89,478
4	2,940	5546	2826	1,357
5	3,046	2444	1326	0,598
6	3,202	596	403	0,146
7	3,540	15028	2285	3,678
8	3,776	806	433	0,197
Total		408582	169417	100,000

HPLC method and UV Instrument Parameters

<<Pump>>

Mode	: Binary gradient
Pump A	: LC-40D X3
Pump B	: LC-40D X3
PressMax	: 400 bar
PressMin	: 0 bar
Pump A Valve Model	: Switching Valve
Pump B Valve Model	: Switching Valve
Gradient Program:	

Pump A + B

#	Time(min) / Flow(mL/min) / B.Conc(%) / B.Curve
1	0,00/ 1,2000/ 1,0/ 0
2	9,00/ / 70,0/
3	15,00/ / 99,0/
4	19,00/ / 99,0/
5	19,10/ / 1,0/

Pump A Mobile Phase Settings : Water + 0.1% formic acid 0,45 /GPa
Pump B Mobile Phase Settings : Acetonitrile + 0.1% formic acid 1,20 /GPa

<<LC Time Program>>

Time	Module	Command	Value	Comment
20,00	Controller	Stop		

<<PDA>>

Lamp	: D2 & W
Start Wavelength	: 190 nm
End Wavelength	: 800 nm

<HPLC-UV Chromatogram >

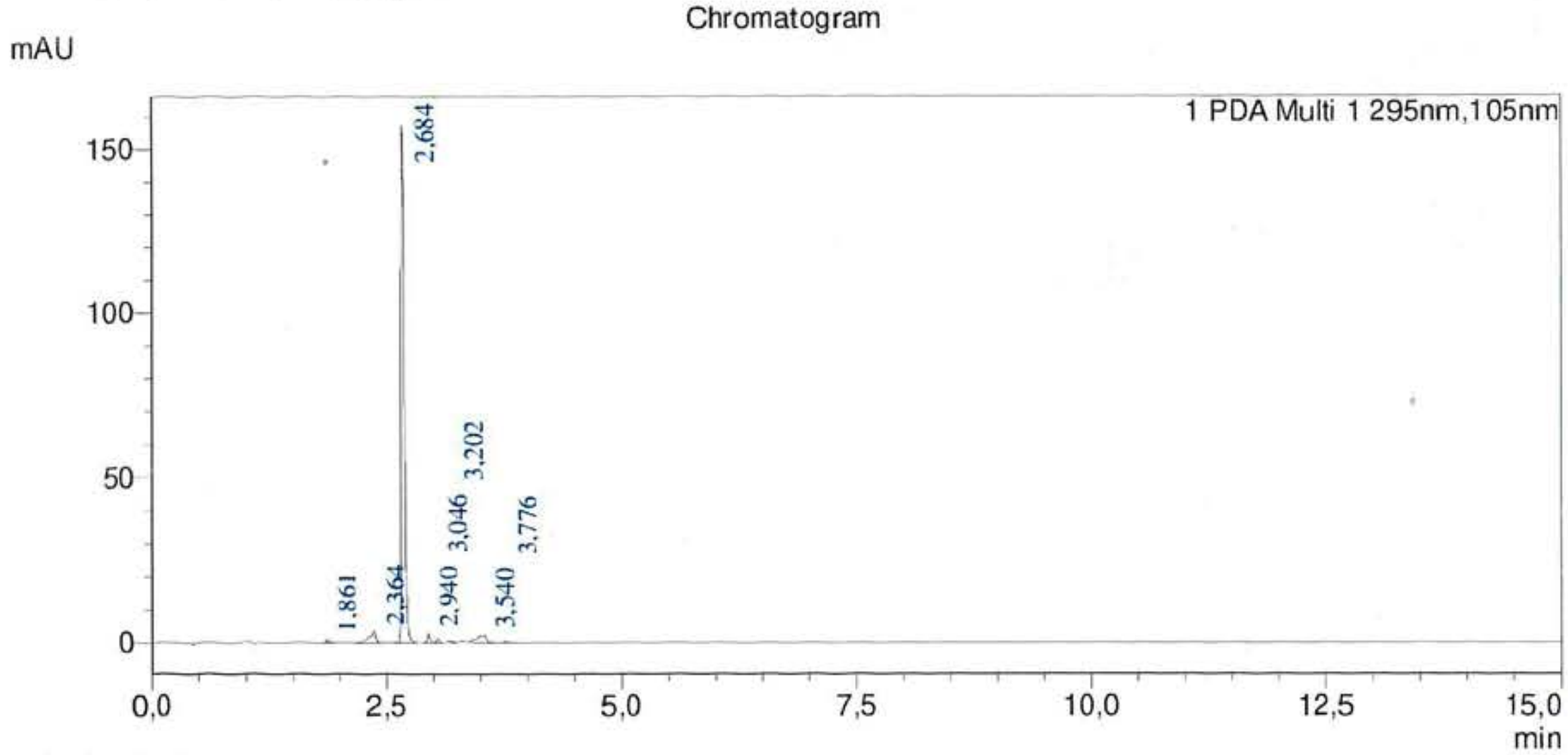

<UV Spectrum >

UV Spectrum

Peak#	: 3
Retention Time	: 2,684 min
Spectrum Operation	: None

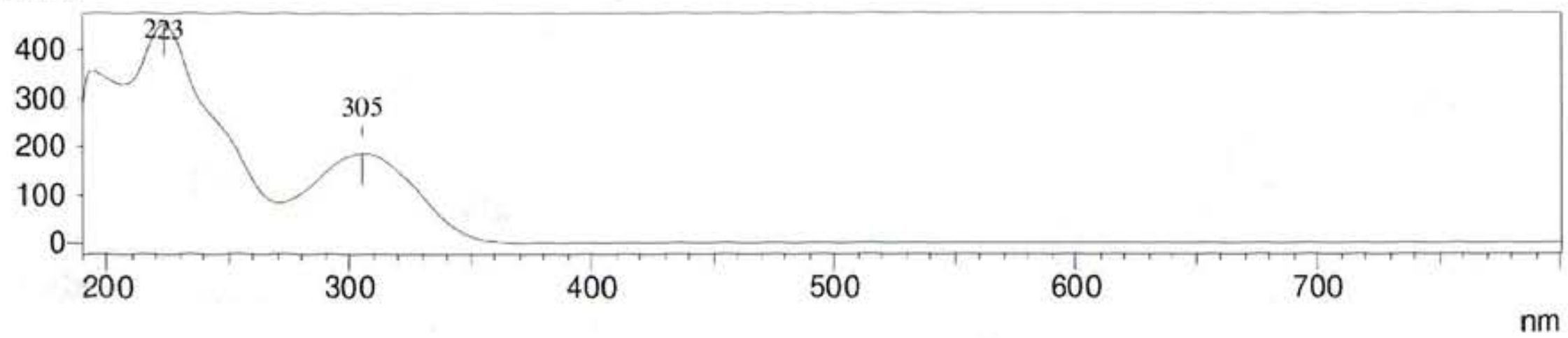

MS Instrument Parameter

<<MS Parameter>>	
Initial Valve Position	:0
--Segment 1 Event 1--	
Start Time	:0,00 min
End Time	:20,00 min
Acquisition Mode	:Scan
Polarity	:Positive
Event Time	:1,00 sec
Detector Voltage	:+1,10 kV
Threshold	:0
Start m/z	:100,00
End m/z	:1100,00
Scan Speed	:1072 u/sec
Interface Volt.	:Use the Data in the Tuning File
DL Volt.	:Use the Data in the Tuning File
Qarray DC Voltage	:Use the Data in the Tuning File
Qarray DC Voltage	:Use the Data in the Tuning File
--Segment 1 Event 2--	
Start Time	:0,00 min
End Time	:20,00 min
Acquisition Mode	:Scan
Polarity	:Negative
Event Time	:1,00 sec
Detector Voltage	:+1,10 kV
Threshold	:0
Start m/z	:100,00
End m/z	:1100,00
Scan Speed	:1072 u/sec
Interface Volt.	:Use the Data in the Tuning File
DL Volt.	:Use the Data in the Tuning File
Qarray DC Voltage	:Use the Data in the Tuning File
Qarray DC Voltage	:Use the Data in the Tuning File
<<Interface>>	
Interface	:DUIS
DL Temperature	:300 C
Nebulizing Gas Flow	:1,50 L/min
Heat Block	:400 C
Drying Gas	:On
	18,00 L/min

Segment	Start Time	End Time	Ionization Mode
1	0,00	20,00	ESI&APCI

<HPLC-MS Chromatogram>

MS Chromatogram

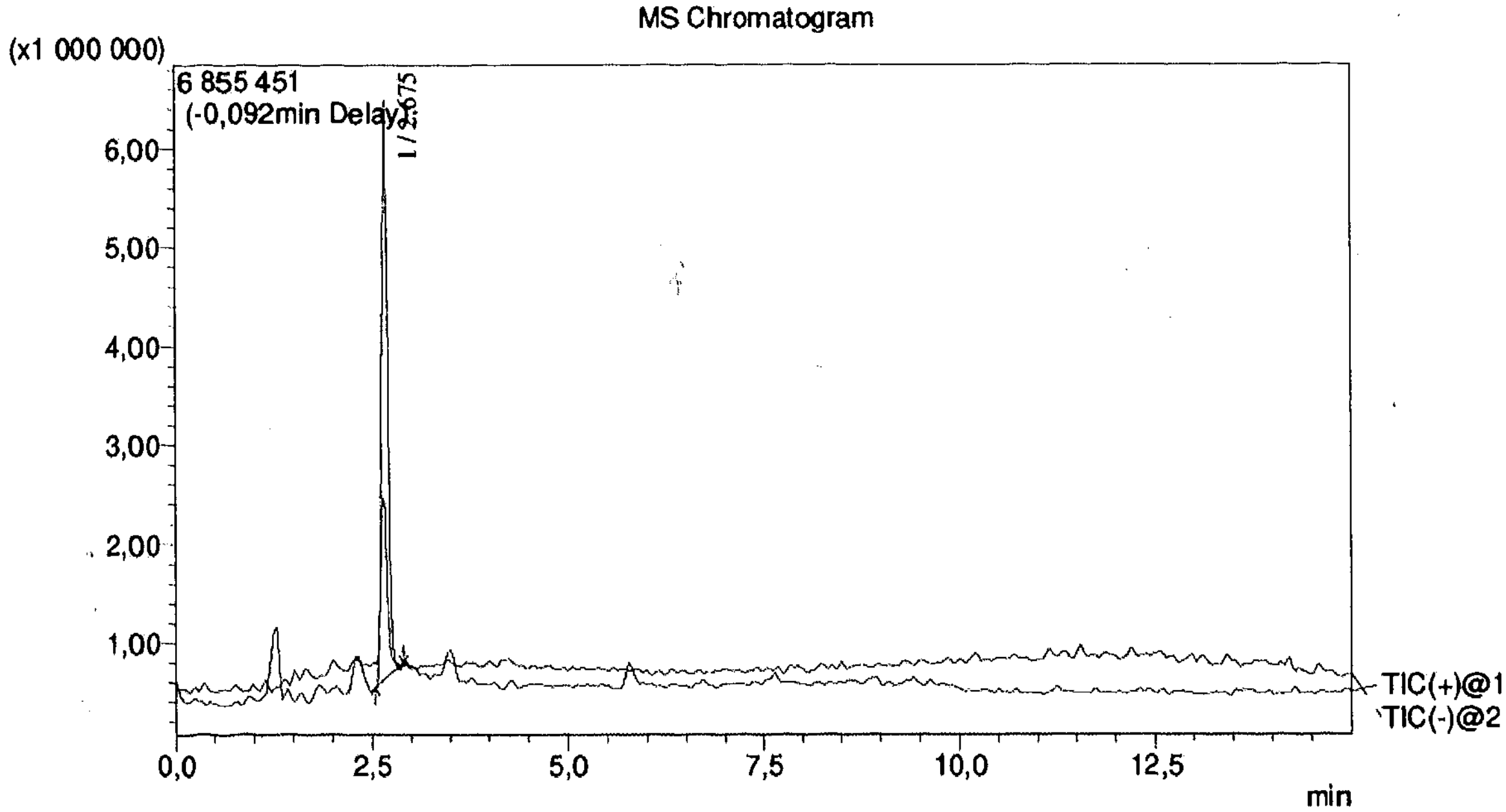

< Mass spectrum>

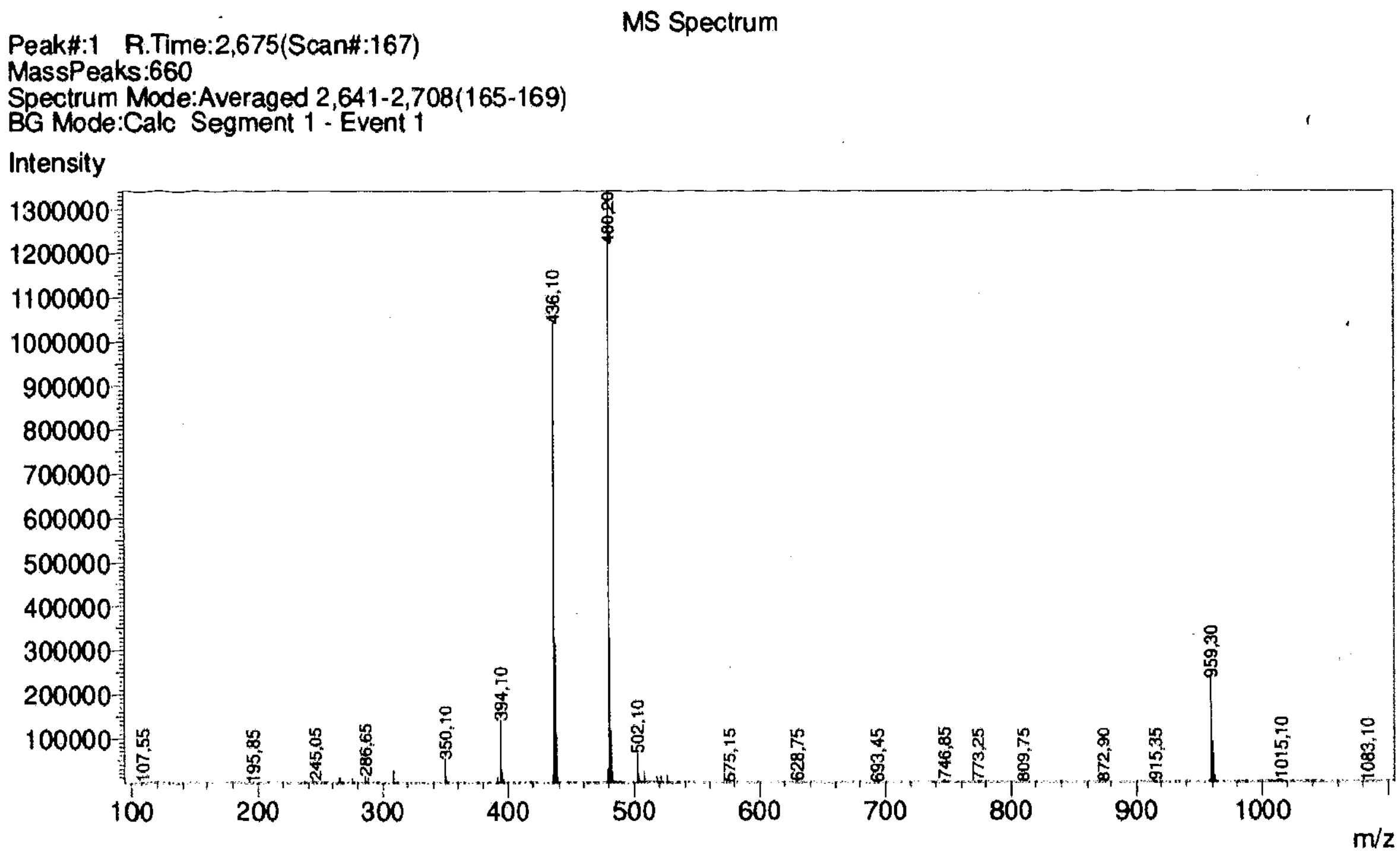

< Mass Profile Spectrum>

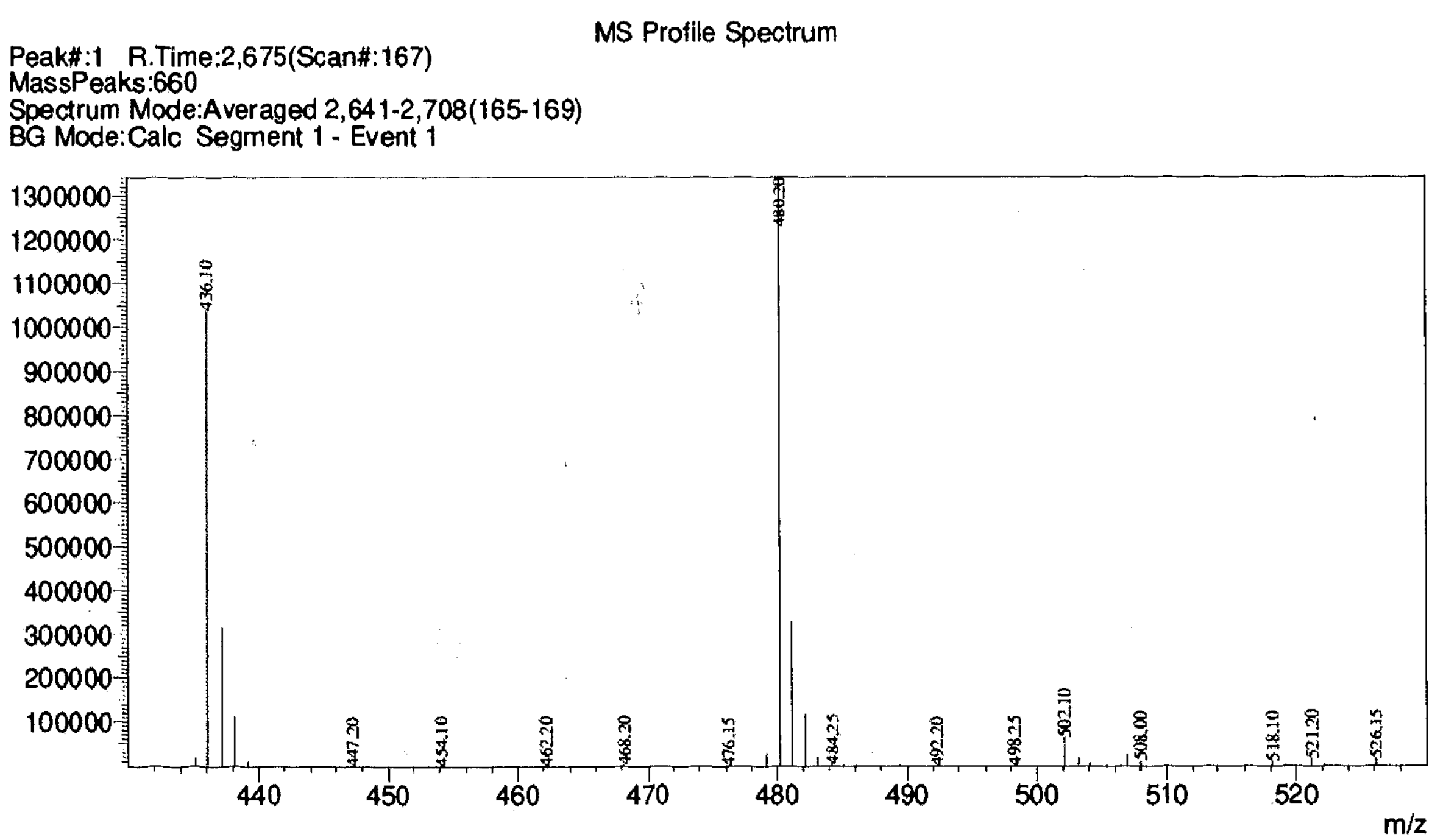

ALSACHIM
a Shimadzu Group Company

SAFETY DATA SHEET

(REACH regulation (EC) n° 1907/2006 - n° 2020/878)

SECTION 1 : IDENTIFICATION OF THE SUBSTANCE/MIXTURE AND OF THE COMPANY/UNDERTAKING

1.1. Product identifier

Product name : *[2H4]-ERTAPENEM SODIUM SALT*

Product code : C3376.

N° CAS 153773-82-1 unlabeled

1.2. Relevant identified uses of the substance or mixture and uses advised against

Identified uses : Laboratory chemicals, Manufacture of substances

1.3. Details of the supplier of the safety data sheet

Registered company name : Alsachim.

Address : 160 rue Tobias Stimmer.67400.Illkirch Graffenstaden.France.

Telephone : +33 (0) 390 402 200.   Fax : +33 (0) 390 402 199 .

E-mail : contact@alsachim.com

1.4. Emergency telephone number : +33 (0)1 45 42 59 59.

Association/Organisation : INRS / ORFILA   http://www.centres-antipoison.net.

SECTION 2 : HAZARDS IDENTIFICATION

2.1. Classification of the substance or mixture

In compliance with EC regulation No. 1272/2008 and its amendments.

Respiratory sensitisation, Category 1 (Resp. Sens. 1, H334).

Hazardous to the aquatic environment - Acute hazard, Category 1 (Aquatic Acute 1, H400).

Hazardous to the aquatic environment - Chronic hazard, Category 2 (Aquatic Chronic 2, H411).

This substance does not present a physical hazard. Refer to the recommendations regarding the other products present on the site.

2.2. Label elements

In compliance with EC regulation No. 1272/2008 and its amendments.

Hazard pictograms :

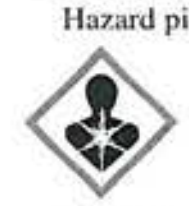 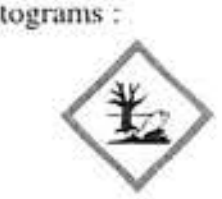

GHS08   GHS09

Signal Word :

DANGER

Product identifiers :

[2H4]-ERTAPENEM SODIUM SALT

Hazard statements :

H334	May cause allergy or asthma symptoms or breathing difficulties if inhaled.
H410	Very toxic to aquatic life with long lasting effects.
Precautionary statements - Prevention :	
P261	Avoid breathing dust.
P273	Avoid release to the environment.
P284	[In case of inadequate ventilation] wear respiratory protection.
Precautionary statements - Response :	
P304 + P340	IF INHALED: Remove person to fresh air and keep comfortable for breathing.
P342 + P311	If experiencing respiratory symptoms: Call a POISON CENTER or doctor.
P391	Collect spillage.
Precautionary statements - Disposal :	
P501	Dispose of contents/container to an approved waste disposal facility.

2.3. Other hazards

The substance does not fulfil the PBT or vPvP criteria in accordance with annexe XIII of the REACH regulations EC 1907/2006.

SECTION 3 : COMPOSITION/INFORMATION ON INGREDIENTS

3.1. Substances

N° CAS 153773-82-1 unlabeled

* Molecular weight 501,52 g/mol

Composition :

Identification	(EC) 1272/2008	Note	%
[2H4]-ERTAPENEM SODIUM SALT	GHS09, GHS08<br>Dgr<br>Resp. Sens. 1, H334<br>Aquatic Chronic 2, H411<br>Aquatic Acute 1, H400<br>M Acute = 1		100%

SECTION 4 : FIRST AID MEASURES

As a general rule, in case of doubt or if symptoms persist, always call a doctor.

NEVER induce swallowing by an unconscious person.

4.1. description of first aid measures

In the event of exposure by inhalation :

In the event of massive inhalation of dust, remove the person exposed to fresh air. Keep warm and at rest.

If breathing is irregular or has stopped, effect mouth-to-mouth resuscitation and call a doctor.

In the event of an allergic reaction, seek medical attention.

In the event of splashes or contact with eyes :

Wash thoroughly with fresh, clean water for 15 minutes holding the eyelids open.

If there is any redness, pain or visual impairment, consult an ophthalmologist.

In the event of splashes or contact with skin :

Watch out for any remaining product between skin and clothing, watches, shoes, etc.

In the event of swallowing :

Seek medical attention, showing the label.

4.2. Most important symptoms and effects, both acute and delayed

No data available.

4.3. Indication of any immediate medical attention and special treatment needed

No data available.

SECTION 5 : FIREFIGHTING MEASURES

Non-flammable.

5.1. Extinguishing media

5.2. Special hazards arising from the substance or mixture

A fire will often produce a thick black smoke. Exposure to decomposition products may be hazardous to health.

Do not breathe in smoke.

In the event of a fire, the following may be formed :

- carbon monoxide (CO)
- carbon dioxide (CO_2)

5.3. Advice for firefighters

No data available.

SECTION 6 : ACCIDENTAL RELEASE MEASURES

6.1. Personal precautions, protective equipment and emergency procedures

Consult the safety measures listed under headings 7 and 8.

For non first aid worker

Avoid inhaling dust.

If a large quantity has been spilt, evacuate all personnel and only allow intervention by trained operators equipped with safety apparatus.

14.7. Maritime transport in bulk according to IMO instruments

No data available.

SECTION 15: Regulatory information

15.1. Safety, health and environmental regulations/legislation specific for the substance or mixture

- Classification and labelling information included in section 2:

The following regulations have been used:

- EU Regulation No. 1272/2008 amended by EU Regulation No. 2021/643 (ATP 16)
- EU Regulation No. 1272/2008 amended by EU Regulation No. 2021/849 (ATP 17)

- Container information:

The substance is packed in an interior packaging, not exceeding 10 ml, the required labelling elements appear on the outer packaging.

- Particular provisions :

No data available.

15.2. Chemical safety assessment

No data available.

SECTION 16 : OTHER INFORMATION

Since the user's working conditions are not known by us, the information supplied on this safety data sheet is based on our current knowledge and on national and community regulations.

It is at all times the responsibility of the user to take all necessary measures to comply with legal requirements and local regulations.

The information in this safety data sheet must be regarded as a description of the safety requirements relating to the substance and guarantee of the properties thereof.

Wording of the phrases mentioned in section 3 :

H334	May cause allergy or asthma symptoms or breathing difficulties if inhaled.
H400	Very toxic to aquatic life.
H411	Toxic to aquatic life with long lasting effects.

Abbreviations :

REACH : Registration, Evaluation, Authorization and Restriction of Chemical Substances.
ADR : European agreement concerning the international carriage of dangerous goods by Road.
IMDG : International Maritime Dangerous Goods.
IATA : International Air Transport Association.
ICAO : International Civil Aviation Organisation
RID : Regulations concerning the International carriage of Dangerous goods by rail.
WGK : Wassergefahrdungsklasse (Water Hazard Class).
GHS08 : Health hazard
GHS09 : Environment
PBT: Persistent, bioaccumulable and toxic.
vPvB : Very persistent, very bioaccumulable.
SVHC : Substances of very high concern.

espiratory protection

void inhaling dust.

the ventilation is insufficient, wear appropriate breathing apparatus.

/hen workers are confronted with concentrations that are above occupational exposure limits, they must wear a suitable, approved, espiratory protection device.

ype of FFP mask :

/ear a disposable half-mask dust filter in accordance with standard EN149/A1.

ategory :

FFP1

nti-gas and vapour filter(s) (Combined filters) in accordance with standard EN14387 :

A1 (Brown)

TION 9 : PHYSICAL AND CHEMICAL PROPERTIES

Information on basic physical and chemical properties

**ysical state**	
hysical state :	Solid.
**lour**	
olor	Pale grey to white
**our**	
dour threshold :	Not stated.
**lting point**	
lelting point/melting range :	Not relevant.
**ezing point**	
reezing point / Freezing range :	Not stated.
**iling point or initial boiling point and boiling range**	
oiling point/boiling range :	Not relevant.
**mmability**	
lammability (solid, gas) :	Not stated.
**wer and upper explosion limit**	
xplosive properties, lower explosivity limit (%) :	Not stated.
xplosive properties, upper explosivity limit (%) :	Not stated.
**sh point**	
lash point interval :	Not relevant.
**to-ignition temperature**	
elf-ignition temperature :	Not relevant.
**composition temperature**	
ecomposition point/decomposition range :	Not relevant.
H (aqueous solution) :	Not stated.
H :	Not relevant.
**nematic viscosity**	
iscosity :	Not stated.
**ubility**	
/ater solubility :	Soluble.
at solubility :	Not stated.
olubility	Mixture of Acetonitrile and Water (1/1)
**rtition coefficient n-octanol/water (log value)**	
artition coefficient: n-octanol/water :	Not stated.
**pour pressure**	
apour pressure (50°C) :	Below 110 kPa (1.10 bar).
**nsity and/or relative density**	
ensity :	= 1
**lative vapour density**	
apour density :	Not stated.

9.2. Other information

Molecular formula : C22H20D4N3O7S . Na

9.2.1. Information with regard to physical hazard classes

No data available.

9.2.2. Other safety characteristics

No data available.

SECTION 10 : STABILITY AND REACTIVITY

10.1. Reactivity

No data available.

10.2. Chemical stability

This substance is stable under the recommended handling and storage conditions in section 7.

10.3. Possibility of hazardous reactions

No data available.

10.4. Conditions to avoid

Avoid :

- formation of dusts

Dusts can form an explosive mixture with air.

10.5. Incompatible materials

No data available.

10.6. Hazardous decomposition products

The thermal decomposition may release/form :

- carbon monoxide (CO)
- carbon dioxide (CO2)

SECTION 11 : TOXICOLOGICAL INFORMATION

11.1. Information on hazard classes as defined in Regulation (EC) No 1272/2008

May cause hypersensitivity of the respiratory tracts with effects taking the form of asthma, rhinitis/conjunctivitis or alveolitis.

11.1.1. Substances

No toxicological data available for the substances.

SECTION 12 : ECOLOGICAL INFORMATION

Very toxic to aquatic life with long lasting effects.

The product must not be allowed to run into drains or waterways.

12.1. Toxicity

12.2. Persistence and degradability

No data available.

12.3. Bioaccumulative potential

No data available.

12.4. Mobility in soil

No data available.

12.5. Results of PBT and vPvB assessment

No data available.

12.6. Endocrine disrupting properties

No data available.

12.7. Other adverse effects

No data available.

SECTION 13 : DISPOSAL CONSIDERATIONS

Proper waste management of the substance and/or its container must be determined in accordance with Directive 2008/98/EC.

13.1. Waste treatment methods

Do not pour into drains or waterways.

Waste :

Waste management is carried out without endangering human health, without harming the environment and, in particular without risk to water, air, soil, plants or animals.

Recycle or dispose of waste in compliance with current legislation, preferably via a certified collector or company.

Do not contaminate the ground or water with waste, do not dispose of waste into the environment.

Soiled packaging :

Empty container completely. Keep label(s) on container.

Give to a certified disposal contractor.

SECTION 14 : TRANSPORT INFORMATION

Transport product in compliance with provisions of the ADR for road, RID for rail, IMDG for sea and ICAO/IATA for air transport (ADR 2021 - IMDG 2020 - ICAO/IATA 2021).

14.1. UN number or ID number

3077

14.2. UN proper shipping name

UN3077=ENVIRONMENTALLY HAZARDOUS SUBSTANCE, SOLID, N.O.S.

([2h4]-ertapenem sodium salt)

14.3. Transport hazard class(es)

- Classification :

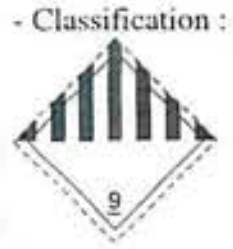

9

14.4. Packing group

III

14.5. Environmental hazards

- Environmentally hazardous material :

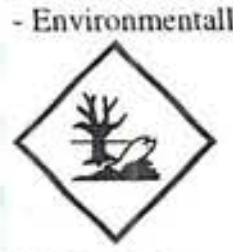

14.6. Special precautions for user

ADR/RID	Class	Code	Pack gr.	Label	Ident.	LQ	Provis.	EQ	Cat.	Tunnel
	9	M7	III	9	90	5 kg	274 335 375 601	E1	3	-

Not subject to this regulation if Q <= 5 l / 5 kg (ADR 3.3.1 - DS 375)

IMDG	Class	2°Label	Pack gr.	LQ	EMS	Provis.	EQ	Stowage Handling	Segregation
	9	-	III	5 kg	F-A, S-F	274 335 966 967 969	E1	Category A SW23	-

Not subject to this regulation if Q <= 5 l / 5 kg (IMDG 3.3.1 - 2.10.2.7)

IATA	Class	2°Label	Pack gr.	Passager	Passager	Cargo	Cargo	note	EQ
	9	-	III	956	400 kg	956	400 kg	A97 A158 A179 A197 A215	E1
	9	-	III	Y956	30 kg G	-	-	A97 A158 A179 A197 A215	E1

Not subject to this regulation if Q <= 5 l / 5 kg (IATA 4.4.4 - DS A197)

For limited quantities, see part 2.7 of the OACI/IATA and chapter 3.4 of the ADR and IMDG.

For excepted quantities, see part 2.6 of the OACI/IATA and chapter 3.5 of the ADR and IMDG.

For first aid worker

First aid workers will be equipped with suitable personal protective equipment (See section 8).

6.2. Environmental precautions

Prevent any material from entering drains or waterways.

6.3. Methods and material for containment and cleaning up

Retrieve the product by mechanical means (sweeping/vacuuming).

6.4. Reference to other sections

No data available.

SECTION 7 : HANDLING AND STORAGE

Requirements relating to storage premises apply to all facilities where the substance is handled.

7.1. Precautions for safe handling

Always wash hands after handling.

Remove and wash contaminated clothing before re-using.

Fire prevention :

Handle in well-ventilated areas.

Prevent access by unauthorised personnel.

Recommended equipment and procedures :

For personal protection, see section 8.

Observe precautions stated on label and also industrial safety regulations.

Also provide breathing apparatus for certain short tasks of an exceptional nature and for emergency interventions.

In all cases, recover emissions at source.

Prohibited equipment and procedures :

No smoking, eating or drinking in areas where the substance is used.

7.2. Conditions for safe storage, including any incompatibilities

No data available.

Storage

Keep the container tightly closed in a dry, well-ventilated place.

For a prolonged period, keep at -20°C (±5°C) in a closed container, away from light and moisture.

Packaging

Always keep in packaging made of an identical material to the original.

7.3. Specific end use(s)

No data available.

SECTION 8 : EXPOSURE CONTROLS/PERSONAL PROTECTION

8.1. Control parameters

No data available.

8.2. Exposure controls

Personal protection measures, such as personal protective equipment

Use personal protective equipment that is clean and has been properly maintained.

Store personal protective equipment in a clean place, away from the work area.

Never eat, drink or smoke during use. Remove and wash contaminated clothing before re-using. Ensure that there is adequate venti especially in confined areas.

- Eye / face protection

Avoid contact with eyes.

Before handling powders or dust emission, wear mask goggles in accordance with standard EN166.

- Hand protection

Use suitable protective gloves that are resistant to chemical agents in accordance with standard EN ISO 374-1.

Gloves must be selected according to the application and duration of use at the workstation.

Protective gloves need to be selected according to their suitability for the workstation in question : other chemical products that m handled, necessary physical protections (cutting, pricking, heat protection), level of dexterity required.

- Body protection

Work clothing worn by personnel shall be laundered regularly.

After contact with the product, all parts of the body that have been soiled must be washed.

WOCKHARDT | LIFE WINS

ANALYTICAL RESEARCH

CERTIFICATE OF ANALYSIS FOR REFERENCE STANDARD

**Name**	:	**Zidebactam (WCK 5107)**			
Molecular Formula	:	$C_{13}H_{21}N_5O_7S.2H_2O$	Batch Number	:	WK1/WP-0060/016
Molecular Weight	:	427.45	Laboratory Note Book Reference	:	WA-2004-038
Date of Analysis	:	30/03/2022	Month of Manufacturing	:	May' 2019
Valid Up To	:	29/03/2023	Reference Standard Number	:	RS/2022-013
Effective Date	:	23/04/2022	Page Number	:	Page 1 of 4

Sr. No.	Tests	Specifications	Results
1	Description	White to pale yellow powder	White powder
2	Identification<br>(By IR, Mass & NMR Spectroscopy)	Spectral data should conform to the structure	Spectral data conforms the structure
3	Solubility	Freely soluble in water,<br>Insoluble in dichloromethane	Freely soluble in water, Insoluble in dichloromethane
4	Constituted solution<br>(10% w/v solution in WFI)<br>Completeness of solution<br><br>Clarity of solution	 <br>The solid dissolves completely, leaving no visible residue as un-dissolved matter<br>The constituted solution is not significantly less clear than an equal volume of the WFI contained in a similar vessel and examined similarly	 <br>Complies<br><br>Complies
5	pH of solution	Between 5.0 to 7.0	6.0
6	Absorbance of solution (1.25 % w/v in water at 420 nm)	Not more than 0.2 AU	0.01 AU
7	Water content (By KF Titration)	Not more than 12.0 % w/w	8.7 % w/w
8	Specific optical rotation (On anhydrous basis)	Between –28° to -40°	-36°
9	Heavy metals	Not more than 20 μg/g	Less than 20 μg/g
10	Palladium content (By ICP-OES)	Not more than 10 μg/g	BLQ (LOQ 2.5 μg/g)*

	Prepared by	Checked by	Approved by
Name :	Mr. Sitaram Ubale	Mr. Rajendra Chavan	Mr Subhash Chandra Shukla
Department :	AVL	AVL	CRDQ
Sign :	[signature]	[signature]	[signature]
Date :	12/04/2022	12/04/2022	13/04/2022

Format No. : AVL-045-F-02-00

Ranked # 1 in Healthcare
Best Companies to Work for 2015 India

ANALYTICAL RESEARCH

CERTIFICATE OF ANALYSIS FOR REFERENCE STANDARD

Name	:	**Zidebactam (WCK 5107)**			
Molecular Formula	:	$C_{13}H_{21}N_5O_7S.2H_2O$	Batch Number	:	WK1/WP-0060/016
Molecular Weight	:	427.45	Laboratory Note Book Reference	:	WA-2004-038
Date of Analysis	:	30/03/2022	Month of Manufacturing	:	May' 2019
Valid Up To	:	29/03/2023	Reference Standard Number	:	RS/2022-013
Effective Date	:	23/04/2022	Page Number	:	Page 2 of 4

Sr. No.	Tests	Specifications	Results
11	Related substances (By HPLC) :		
	i. Impurity -1	Not more than 2.00 % w/w	0.28 % w/w
	ii. Impurity -2	Not more than 1.00 % w/w	Not detected
	iii. Impurity -3	Not more than 1.00 % w/w	0.10 % w/w
	iv. Impurity -4	Not more than 0.10 % w/w	Not detected
	v. Specified impurity at RRT 2.16-2.28 (MW 782)	Not more than 0.50 % w/w	0.03 % w/w
	vi. Any unspecified impurity	Not more than 0.15 % w/w	BDL
	vii. Total impurities	Not more than 4.00 % w/w	0.41 % w/w
12	Residual solvents (By GC) :		
	Group A		
	i. Isopropyl alcohol	Not more than 5000 µg/g	Not detected
	ii. Dichloromethane	Not more than 600 µg/g	Not detected
	iii. Methanol	Not more than 3000 µg/g	1762 µg/g
	iv. Ethyl acetate	Not more than 5000 µg/g	Not detected
	v. Cyclohexane	Not more than 3880 µg/g	Not detected
	vi. Acetone	Not more than 5000 µg/g	Not detected
	vii. Methyl tertiary butyl ether	Not more than 5000 µg/g	Not detected
	viii. Toluene	Not more than 890 µg/g	Not detected
	ix. Ethanol	Not more than 5000 µg/g	2990 µg/g
	Group B		
	i. Triethylamine	Not more than 250 µg/g	Not detected
	ii. Pyridine	Not more than 200 µg/g	Not detected

	Prepared by	Checked by	Approved by
Name :	Mr. Sitaram Ubale	Mr. Rajendra Chavan	Mr Subhash Chandra Shukla
Department :	AVL	AVL	CRDQ
Sign :	[signature]	[signature]	[signature]
Date :	12/04/2022	12/04/2022	13/04/2022

Format No. : AVL-045-F-02-00

Ranked # 1 In Healthcare
GREAT PLACE TO WORK
Best Companies to Work for 2015 India

ANALYTICAL RESEARCH

CERTIFICATE OF ANALYSIS FOR REFERENCE STANDARD

**Name**	:	**Zidebactam (WCK 5107)**			
Molecular Formula	:	$C_{13}H_{21}N_5O_7S.2H_2O$	Batch Number	:	WK1/WP-0060/016
Molecular Weight	:	427.45	Laboratory Note Book Reference	:	WA-2004-038
Date of Analysis	:	30/03/2022	Month of Manufacturing	:	May' 2019
Valid Up To	:	29/03/2023	Reference Standard Number	:	RS/2022-013
Effective Date	:	23/04/2022	Page Number	:	Page 3 of 4

Sr. No.	Tests	Specifications	Results
13	Sodium content (By ICP-OES)	Not more than 0.5 % w/w	BLQ (LOQ 0.25 % w/w)*
14	Sulfate content (By Ion chromatography)	Not more than 1.0 % w/w	BLQ (LOQ 0.626 % w/w)
15	Mono-methyl sulphate content (By Ion chromatography)	Not more than 0.15% w/w	Not detected
16	Polymorphism (By X-ray Diffraction)	The diffractogram should exhibit characteristic 2 theta values at 13.8, 15.6, 20.3 and 24.3 (± 0.2°)	The diffractogram exhibits characteristic 2 theta values at 13.8, 15.6, 20.3 & 24.3
17	Potency (on as is basis)	Not applicable	90.4 %

BLQ = Below limit of quantification
BDL = Below disregard limit. Disregard limit 0.05 %
LOQ = Limit of quantification

* LOQ Values are based on analytical method verification report (Report nos. MVR/VP17053 and MVR/VP17054) of Analytical Solutions (CRO).

Specification Reference Number: **ZDB-DSS-112-05**

Remark: Potency is calculated on the basis of Total impurities, Water content, Residual solvents, Sulphate, Mono-methyl sulphate, Sodium and Palladium content.

Storage Condition: Store at 2°C to 8°C temperature.

	Prepared by	Checked by	Approved by
Name :	Mr. Sitaram Ubale	Mr. Rajendra Chavan	Mr Subhash Chandra Shukla
Department :	AVL	AVL	CRDQ
Sign :	[signature]	[signature]	[signature]
Date :	12/04/2022	12/04/2022	13/04/2022

Format No. : AVL-045-F-02-00

Ranked # 1 In Healthcare
Great Place to Work Best Companies to Work for 2015 India

ANALYTICAL RESEARCH

CERTIFICATE OF ANALYSIS FOR REFERENCE STANDARD

**Name**	:	**Zidebactam (WCK 5107)**			
Molecular Formula	:	$C_{13}H_{21}N_5O_7S.2H_2O$	Batch Number	:	WK1/WP-0060/016
Molecular Weight	:	427.45	Laboratory Note Book Reference	:	WA-2004-038
Date of Analysis	:	30/03/2022	Month of Manufacturing	:	May' 2019
Valid Up To	:	29/03/2023	Reference Standard Number	:	RS/2022-013
Effective Date	:	23/04/2022	Page Number	:	Page 4 of 4

Chemical Names:

i. **WCK 5107**: (2S,5R)-7-Oxo-6-sulphoxy-2-[N'-((R)-piperidin-3-carbonyl)-hydrazinocarbonyl]-1,6-diaza-bicyclo[3.2.1]octane dehydrate

ii. **Impurity-1**: (2S,5R)-5-sulphooxyamino-2-[N'-((R)-piperidin-3-carbonyl)-hydrazinocarbonyl]-piperidine

iii. **Impurity-2**: (2S,5R)-7-Oxo-6-hydroxy-2-[N'-((R)-piperidin-3-carbonyl)-hydrazinocarbonyl]-1,6-diaza-bicyclo[3.2.1]octane

iv. **Impurity-3**: (2S,5R)-7-Oxo-6-sulphooxy-2-[N'-((S)-piperidin-3-carbonyl)-hydrazinocarbonyl]-1,6-diaza-bicyclo[3.2.1]octane

v. **Impurity-4**: (2S,5R)-7-Oxo-6-sulphooxy-2-[N'-((R)-1-tert-butoxycarbonyl-piperidin-3-yl)-carbonyl)-hydrazinocarbonyl] -1,6-diaza-bicyclo[3.2.1]octane

	Prepared by	Checked by	Approved by
Name :	Mr. Sitaram Ubale	Mr. Rajendra Chavan	Mr Subhash Chandra Shukla
Department :	AVL	AVL	CRDQ
Sign :	[signature]	[signature]	[signature]
Date :	12/04/2022	12/04/2022	13/04/2022

Format No. : AVL-045-F-02-00

Ranked # 1 in Healthcare
GREAT PLACE TO WORK Best Companies to Work for 2015 India

ANALYTICAL RESEARCH

CERTIFICATE OF ANALYSIS FOR REFERENCE STANDARD

**Name**	:	**Zidebactam (WCK 5107)**			
Molecular Formula	:	$C_{13}H_{21}N_5O_7S.2H_2O$	Batch Number	:	WK1/WP-0060/016
Molecular Weight	:	427.45	Laboratory Note Book Reference	:	WA-2004-038
Date of Analysis	:	30/03/2022	Month of Manufacturing	:	May' 2019
Valid Up To	:	29/03/2023	Reference Standard Number	:	RS/2022-013
Effective Date	:	23/04/2022	Page Number	:	Page 1 of 4

Sr. No.	Tests	Specifications	Results
1	Description	White to pale yellow powder	White powder
2	Identification (By IR, Mass & NMR Spectroscopy)	Spectral data should conform to the structure	Spectral data conforms the structure
3	Solubility	Freely soluble in water, Insoluble in dichloromethane	Freely soluble in water, Insoluble in dichloromethane
4	Constituted solution (10% w/v solution in WFI)<br>Completeness of solution<br><br>Clarity of solution	The solid dissolves completely, leaving no visible residue as un-dissolved matter<br>The constituted solution is not significantly less clear than an equal volume of the WFI contained in a similar vessel and examined similarly	Complies<br><br>Complies
5	pH of solution	Between 5.0 to 7.0	6.0
6	Absorbance of solution (1.25 % w/v in water at 420 nm)	Not more than 0.2 AU	0.01 AU
7	Water content (By KF Titration)	Not more than 12.0 % w/w	8.7 % w/w
8	Specific optical rotation (On anhydrous basis)	Between –28° to -40°	-36°
9	Heavy metals	Not more than 20 μg/g	Less than 20 μg/g
10	Palladium content (By ICP-OES)	Not more than 10 μg/g	BLQ (LOQ 2.5 μg/g)*

	Prepared by	Checked by	Approved by
Name :	Mr. Sitaram Ubale	Mr. Rajendra Chavan	Mr Subhash Chandra Shukla
Department :	AVL	AVL	CRDQ
Sign :	[signature]	[signature]	[signature]
Date :	12/04/2022	12/04/2022	13/04/2022

Format No. : AVL-045-F-02-00

Ranked # 1 in Healthcare
GREAT PLACE TO WORK Best Companies to Work for 2015 India

WOCKHARDT LIFE WINS

ANALYTICAL RESEARCH

CERTIFICATE OF ANALYSIS FOR REFERENCE STANDARD

**Name**	:	**Zidebactam (WCK 5107)**			
Molecular Formula	:	$C_{13}H_{21}N_5O_7S.2H_2O$	Batch Number	:	WK1/WP-0060/016
Molecular Weight	:	427.45	Laboratory Note Book Reference	:	WA-2004-038
Date of Analysis	:	30/03/2022	Month of Manufacturing	:	May' 2019
Valid Up To	:	29/03/2023	Reference Standard Number	:	RS/2022-013
Effective Date	:	23/04/2022	Page Number	:	Page 3 of 4

Sr. No.	Tests	Specifications	Results
13	Sodium content (By ICP-OES)	Not more than 0.5 % w/w	BLQ (LOQ 0.25 % w/w)*
14	Sulfate content (By Ion chromatography)	Not more than 1.0 % w/w	BLQ (LOQ 0.626 % w/w)
15	Mono-methyl sulphate content (By Ion chromatography)	Not more than 0.15% w/w	Not detected
16	Polymorphism (By X-ray Diffraction)	The diffractogram should exhibit characteristic 2 theta values at 13.8, 15.6, 20.3 and 24.3 (± 0.2°)	The diffractogram exhibits characteristic 2 theta values at 13.8, 15.6, 20.3 & 24.3
17	Potency (on as is basis)	Not applicable	90.4 %

BLQ = Below limit of quantification

BDL = Below disregard limit. Disregard limit 0.05 %

LOQ = Limit of quantification

* LOQ Values are based on analytical method verification report (Report nos. MVR/VP17053 and MVR/VP17054) of Analytical Solutions (CRO).

Specification Reference Number: **ZDB-DSS-112-05**

Remark: Potency is calculated on the basis of Total impurities, Water content, Residual solvents, Sulphate, Mono-methyl sulphate, Sodium and Palladium content.

Storage Condition: Store at 2°C to 8°C temperature.

	Prepared by	Checked by	Approved by
Name :	Mr. Sitaram Ubale	Mr. Rajendra Chavan	Mr Subhash Chandra Shukla
Department :	AVL	AVL	CRDQ
Sign :	[signature]	[signature]	[signature]
Date :	12/04/2022	12/04/2022	13/04/2022

Format No. : AVL-045-F-02-00

Ranked # 1 in Healthcare GREAT PLACE TO WORK Best Companies to Work for 2015 India

WOCKHARDT | LIFE WINS

June 19,2022

PROFORMA INVOICE

International Air Waybill No	Date of Exportation: **June 19,2022**
**Shipper / Exporter:**<br>Sachin Bhagwat<br>Wockhardt Research Centre<br>D-4 MIDC Chikalthana<br>Aurangabad 431 210, M.S. INDIA<br>Tel : 91-240-6632222<br>Fax: 91-240-2485292	**Consignee Compounding:/Contact**<br>Test Materials Management (TMM)<br>ATTN: Bryan Parmentier, Anika Pippin, Lana Goehl ,KCAS Bioanalytical & Biomarker Services 12400 Shawnee Mission Parkway Shawnee, KS 66216, USA<br>913.248.3066 / 913-248-3000

No of Packs	Full Description of Goods		Total Value
01	Zidebactam (WCK 5107)	10 gm X 1 vial = 10 gm	10
	**Total Invoice Value**		**USD 10**

NON-HAZARDOUS COMPOUND
NOT FOR RE-SALE

MATERIAL IS EXTRACTED FROM chemicals DERIVED SUBSTANCE ONE TIME LAB TESTING ONLY ENTIRE AMOUNT TO BE CONSUMED IN TESTING

NON-INFECTIOUS/NON CONTAGIOUS, NO COMMERCIAL VALUE. NON TOXIC MATERIAL DOES NOT CONTAIN ANY ANIMAL OR CELL CULTURE DERIVETIVE PRODUCTS , NOT INFLAMMABLE NON-CORROSIVE, NON-DENGEROUS

For Wockhardt Research Centre

WOCKHARDT LTD. WOCKHARDT AURANGABAD

WOCKHARDT | LIFE WINS

June 19,2022

PROFORMA INVOICE

International Air Waybill No	Date of Exportation: June 19,2022
Shipper / Exporter:<br>Sachin Bhagwat<br>Wockhardt Research Centre<br>D-4 MIDC Chikalthana<br>Aurangabad 431 210, M.S. INDIA<br>Tel : 91-240-6632222<br>Fax: 91-240-2485292	Consignee Compounding:/Contact<br>Test Materials Management (TMM)<br>ATTN: Bryan Parmentier, Anika Pippin, Lana Goehl ,KCAS Bioanalytical & Biomarker Services 12400 Shawnee Mission Parkway Shawnee, KS 66216, USA<br>913.248.3066 / 913-248-3000

No of Packs	Full Description of Goods		Total Value
01	Zidebactam (WCK 5107)	10 gm X 1 vial = 10 gm	10
	Total Invoice Value		USD 10

NON-HAZARDOUS COMPOUND
NOT FOR RE-SALE

MATERIAL IS EXTRACTED FROM chemicals DERIVED SUBSTANCE ONE TIME LAB TESTING ONLY ENTIRE AMOUNT TO BE CONSUMED IN TESTING

NON-INFECTIOUS/NON CONTAGIOUS, NO COMMERCIAL VALUE. NON TOXIC MATERIAL DOES NOT CONTAIN ANY ANIMAL OR CELL CULTURE DERIVETIVE PRODUCTS , NOT INFLAMMABLE NON-CORROSIVE, NON-DENGEROUS

For Wockhardt Research Centre

WOCKHARDT LTD. ★ AURANGABAD ★ WOCKHARDT

WOCKHARDT

Material Safety Data Sheet (MSDS)

==

MSDS of Zidebactam (WCK 5107)

SECTION 1: CHEMICAL PRODUCT IDENTIFICATION

SUBSTANCE: WCK 5107

INN: Zidebactam

CHEMICAL NAME: (2S, 5R)-7-Oxo-6-sulphooxy -2-[N'-((R)-piperidin-3-carbonyl)-hydrazinocarbonyl]-1,6-diaza-bicyclo[3.2.1]octane dihydrate

MOLECULAR FORMULA: $C_{13}H_{21}N_5O_7S.2H_2O$

MOLECULAR WEIGHT: 427.4

PERCENT COMPOSITION: C 39.89%, H 17.89%, N 5.41%

TRADE NAMES/SYNONYMS: Not available.

CAS NUMBER: 1996664-59-5

CHEMICAL FAMILY: 1,6-Diaza-Bicyclo Octane (DBO)

SUBSTANCE USE: As a beta-lactam enhancer.

SECTION 2 COMPOSITION, INFORMATION ON INGREDIENTS

COMPONENT: Single component.

EC NUMBER (EINECS): Not available

PERCENTAGE: >98%.

SECTION 3 HAZARDS IDENTIFICATION

MAJOR PHYSICAL AND HEALTH HAZARDS: May cause irritation to eyes, skin, and respiratory tract.

ROUTE OF ENTRY: May be absorbed via contact with skin or eyes, inhalation of aerosols or accidental ingestion. Normal use under supervision substance presents little hazards.

WOCKHARDT LTD. ★ AURANGABAD ★ WOCKHARDT

SIGNS AND SYMPTOMS OF EXPOSURE: Acute signs and symptoms of exposure may include eye, skin and respiratory irritation.

CHEMICAL LISTED AS CARCINOGEN: NTP: IARC: OSHA listing: Not known.
MEDICAL CONDITIONS GENERALLY AGGRAVATED BY EXPOSURE: Not known.

SECTION 4 FIRST AID MEASURES

INHALATION: If difficulty with breathing, remove from exposure. Seek attention of physician immediately.

SKIN CONTACT: Remove contaminated clothing, wash skin with water and soap for 15 minutes. Seek medical attention if irritation or signs of exposure are noted.

EYE CONTACT: Flush eyes with large volumes of water for 15 minutes or more. Seek medical attention if irritation or signs of exposure are noted.

INGESTION: If ingestion occurs, flush mouth with water and seek medical attention if any symptoms appear.

INJECTION: In case of accidental injection, wash area and seek medical attention.

SECTION 5 FIRE FIGHTING MEASURES

FLASH POINT: Not applicable.

AUTOIGNITION TEMPERATURE: Not applicable.

FLAMABLE LIMITS IN AIR: Lower and Upper %: Not applicable

FLAMABLE LIMITS: Not applicable

EXTINGUISHING MEDIA: Use of water spray or multi purpose ABC extinguisher is recommended.

SPECIAL FIRE FIGHTING PRCEDURES: As with all fires, evacuate personnel to safe area. Fire fighters should wear self-contained breathing apparatus to avoid inhalation of smoke.

HAZARDOUS COMBUSTION PRODUCTS: Not available.

WOCKHARDT LTD. WOCKHARDT AURANGABAD

WOCKHARDT

UNUSAL FIRE/ EXPLOSION HAZARDS: None.

SECTION 6 ACCIDENTAL RELEASE MEASURES

SPILLS: Collect spillage maximum with suitable precautions such as using mask, safety goggles and gloves. Spry water and absorb liquid with suitable material and clean affected area with soap and water. Dispose of material according to the applicable laws and regulations.

RELESASE TO AIR: If aerosolized, reduce exposure by ventilating area. Wear respiratory protection as necessary.

RELEASE TO WATER: Refer to the local water authority. Drain disposal is not recommended.

SECTION 7 HANDLING AND STORAGE

GENERAL HANDLING: While handling, avoid all contacts and inhalation of dust, fumes, mist and/or vapors associated with the substance.

WASTE DISPOSAL METHOD: Incineration in an approved incinerator is recommended. Refer to the local rules and regulations.

STORAGE CONDITION: Store between 2- 8 deg Celsius.

SECTION 8 EXPOSURE CONTROLS, PERSONAL PROTECTION

Under normal use and handling conditions, no protective equipments are required. The following is recommended for production settings.

RESPIRATORY PROTECTION: Under normal use, respirator may not be required, if adequate ventilation is available. A dust/mist respirator may be required if aerosols are generated.

EYE PROTECTION: Chemical goggles or face shield is recommended, Safety goggles are always required.

VENTILATION: Handle the substance in well ventilated area.

CLOTHING: Handle the substance in work uniform or laboratory coat.

GLOVES: Use protective latex or nitrile gloves.

ADDITIONAL EXPOSURE PRECAUSIONS: Wash hands following use by

WOCKHARDT LTD. ★ AURANGABAD ★

water and soap. Eating, drinking is not recommended while handling the substance.

SECTION 9 PHYSICAL AND CHEMICAL PROPERTIES

PHYSICAL STATE: Solid
COLOR: Pale yellow to white powder.

CHANGE IN APPEARANCE: No change in appearance upon exposure to atmosphere.
PHYSICAL FORM: Solid
ODOR: Odorless
MOLECULAR FORMULA: $C_{13}H_{21}N_3O_7S.2H_2O$
MOLECULAR WEIGHT: 427.4
BOILING POINT: Not available
MELTING POINT: >200° C
DECOMPOSITION POINT: Not available
VAPOR PRESSURE: Not available
VAPOR DENSITY: Not available
SPECIFIC GRAVITY: Not available
WATER SOLUBILITY: more than 200 mg in 1.0 ml water.
pH: 5-7 (0.5% in water)
VOLATILITY: Not applicable
SOLVENT SOLUBILITY: Soluble in water and in methanol; Insoluble in acetone.

SECTION 10 STABILITY AND REACTIVITY

REACTIVITY: Stable at normal temperature and pressure.

INCOMPATIBILITIES: Not identified

ACIDS (STRONG): Unstable

BASES (STRONG): Unstable

OXIDIZERS (STRONG): Unstable

HAZARDOUS DECOMPOSITION: May emit fumes when heated to decomposition.

POLYMERIZATION: will not occur.

SECTION 11 TOXICOLOGICAL INFORMATION

WOCKHARDT

SIGNS AND SYMPTOMS OF EXPOSURE AND OVEREXPOSURE:
Immediate effect is eye, skin and respiratory irritation may occur.

ACUTE TOXICITY:

LD50 in rat (oral): >2000 mg/kg.
LD50 in mice (oral): >2000 mg/kg.

LD50 in rat (IV): >2000 mg/kg.
LD50 in mice (IV): >2000 mg/kg.

TARGET ORGANS: Not found.

MEDICAL CONDITIONS AGGRAVATED BY EXPOSURE: Not available

TUMORIGENIC DATA: Not available

MUTAGENIC DATA: Non-mutagenic

REPRODUCTIVE EFFECTS DATA: Not available

SECTION 12 ECOLOGICAL INFORMATION

ECOTOXICITY DATA: Not available
INVERTEBRATE TOXICITY: Not available

SECTION 13 DISPOSAL CONSIDERATIONS:

WASTE DISPOSAL: Dispose of any clean up material and waste residue according to all applicable laws and regulations. Incineration at approved, permitted facility is recommended.

SECTION 14 TRANSPORT INFORMATION

Information with regulatory organizations DOT, ICAO/IATA and IMO is not available

SECTION 15 REGULATORY INFORMATION

This section is not complete analysis or reference to all applicable regulatory information. Please consider all applicable laws and regulations for your country/state.

SECTION 16 OTHER INFORMATION

Disclaimer:

To the best of our knowledge, the information contained herein is accurate. However, neither Wockhardt nor any of its subsidiaries assume any liability whatsoever for the accuracy or completeness of the information contained herein. Final determination of suitability of any material is the sole

WOCKHARDT LTD. ★ AURANGABAD ★

responsibility of the user. All materials may present unknown hazards and should be used with caution. Although certain hazards are described herein, Wockhardt cannot guarantee that these are the only hazards that exist.

WOCKHARDT LTD. AURANGABAD

WOCKHARDT LIFE WINS

ANALYTICAL RESEARCH

CERTIFICATE OF ANALYSIS FOR REFERENCE STANDARD

**Name**	:	**Zidebactam (WCK 5107)**			
Molecular Formula	:	$C_{13}H_{21}N_5O_7S.2H_2O$	Batch Number	:	WK1/WP-0060/016
Molecular Weight	:	427.45	Laboratory Note Book Reference	:	WA-2362-020
Date of Analysis	:	02/03/2023	Month of Manufacturing	:	May' 2019
Valid Up To	:	01/03/2024	Reference Standard Number	:	RS/2023-017
Effective Date	:	30/03/2023	Page Number	:	Page 1 of 4

Sr. No.	Tests	Specifications	Results
1	Description	White to pale yellow powder	White powder
2	Identification (By IR, Mass & NMR Spectroscopy)	Spectral data should conform to the structure	Spectral data conforms the structure
3	Solubility	Freely soluble in water, Insoluble in dichloromethane	Freely soluble in water, Insoluble in dichloromethane
4	Completeness and Clarity of Solutions (10% w/v solution in Water For Injection (WFI))	The solid dissolves completely, leaving no undissolved matter.	Complies
		The constituted solution is not significantly less clear than an equal volume of the WFI contained in a similar vessel and examined similarly.	Complies
		The constituted solution is free from particulate matter that can be observed on visual inspection.	Complies
5	pH of Solution	5.0 to 7.0	6.0
6	Absorbance of Solution (1.25 % w/v in water at 420 nm)	Not more than 0.2 AU	0.01 AU
7	Water Content (By KF Titration)	Not more than 12.0 % w/w	8.6 % w/w
8	Specific Optical Rotation (On anhydrous basis) (0.5 % w/v solution in water)	–28° to –40°	–36°
9	Palladium Content (By ICP-OES)	Not more than 10 μg/g	BLQ (LOQ 2.5 μg/g)*

	Prepared by	Checked by	Approved by
Name :	Mr. Sitaram Ubale	Mr. Rajendra Chavan	Ms. Mohini Khatik
Department :	AVL	AVL	CRDQ
Sign :	[signature]	[signature]	[signature]
Date :	05/04/2023	05/04/2023	05/04/2023

Format No. : AVL-045-F-02-00

Ranked # 1 in Healthcare
GREAT PLACE TO WORK® Best Companies to Work for 2015 India

ANALYTICAL RESEARCH

CERTIFICATE OF ANALYSIS FOR REFERENCE STANDARD

Name	:	Zidebactam (WCK 5107)			
Molecular Formula	:	$C_{13}H_{21}N_5O_7S.2H_2O$	Batch Number	:	WK1/WP-0060/016
Molecular Weight	:	427.45	Laboratory Note Book Reference	:	WA-2362-020
Date of Analysis	:	02/03/2023	Month of Manufacturing	:	May' 2019
Valid Up To	:	01/03/2024	Reference Standard Number	:	RS/2023-017
Effective Date	:	30/03/2023	Page Number	:	Page 2 of 4

Sr. No.	Tests	Specifications	Results
10	Related Substances (By HPLC) :		
	i. Impurity -1	Not more than 2.00 % w/w	0.24 % w/w
	ii. Impurity -2	Not more than 1.00 % w/w	Not detected
	iii. Impurity -3	Not more than 1.00 % w/w	0.20 % w/w
	iv. Impurity -4	Not more than 0.10 % w/w	Not detected
	v. Specified Impurity at RRT 2.16-2.28 (MW 782)	Not more than 0.50 % w/w	0.02 % w/w
	vi. Any Unspecified Impurity #		
	Individual Unspecified Impurity	Not more than 0.15 % w/w	0.04 % w/w (RRT 0.59)
	vii. Total Impurities	Not more than 4.00 % w/w	0.50 % w/w
11	Residual Solvents (By GC) :		
	Group A		
	i. Isopropyl alcohol	Not more than 5000 µg/g	Not detected
	ii. Dichloromethane	Not more than 600 µg/g	Not detected
	iii. Methanol	Not more than 3000 µg/g	1762 µg/g
	iv. Ethyl acetate	Not more than 5000 µg/g	Not detected
	v. Cyclohexane	Not more than 3880 µg/g	Not detected
	vi. Acetone	Not more than 5000 µg/g	Not detected
	vii. Methyl tertiary butyl ether	Not more than 5000 µg/g	Not detected
	viii. Toluene	Not more than 890 µg/g	Not detected
	ix. Ethanol	Not more than 5000 µg/g	2990 µg/g
	Group B		
	i. Triethylamine	Not more than 250 µg/g	Not detected
	ii. Pyridine	Not more than 200 µg/g	Not detected

	Prepared by	Checked by	Approved by
Name :	Mr. Sitaram Ubale	Mr. Rajendra Chavan	Ms. Mohini Khatik
Department :	AVL	AVL	CRDQ
Sign :	[signature]	[signature]	[signature]
Date :	05/04/2023	05/04/2023	05/04/2023

Format No. : AVL-045-F-02-00

Ranked # 1 in Healthcare
GREAT PLACE TO WORK® Best Companies to Work for 2015 India

WOCKHARDT LIFE WINS

ANALYTICAL RESEARCH

CERTIFICATE OF ANALYSIS FOR REFERENCE STANDARD

**Name**	:	**Zidebactam (WCK 5107)**			
Molecular Formula	:	$C_{13}H_{21}N_5O_7S.2H_2O$	Batch Number	:	WK1/WP-0060/016
Molecular Weight	:	427.45	Laboratory Note Book Reference	:	WA-2362-020
Date of Analysis	:	02/03/2023	Month of Manufacturing	:	May' 2019
Valid Up To	:	01/03/2024	Reference Standard Number	:	RS/2023-017
Effective Date	:	30/03/2023	Page Number	:	Page 3 of 4

Sr. No.	Tests	Specifications	Results
12	Sodium Content (By ICP-OES)	Not more than 0.5 % w/w	BLQ (LOQ 0.25 % w/w)*
13	Sulfate Content (By Ion Chromatography)	Not more than 1.0 % w/w	BLQ (LOQ 0.626 % w/w)
14	Monomethyl sulphate Content (By Ion Chromatography)	Not more than 0.15% w/w	Not detected
15	Polymorphism (By X-ray Diffraction)	The diffractogram should exhibit characteristic 2 θ values at 13.8°, 15.6°, 20.3° and 24.3° (± 0.2°)	The diffractogram exhibits characteristic 2 θ values at 13.8°, 15.6°, 20.3° & 24.3°
16	Potency (On "as is" basis)	Not applicable	90.4 %

BLQ = Below Limit of Quantitation
LOQ = Limit of Quantitation

# All Unspecified Impurities > 0.03 % reported individually along with their respective Relative Retention Times (RRT's).

* LOQ Values are based on analytical method verification report (Report nos. MVR/VP17053 and MVR/VP17054) of Analytical Solutions (CRO).

Specification Reference Number: **ZDB-DSS-112-06**

Remark: Potency is calculated on the basis of Total Impurities, Water Content and Residual Solvents.

Storage Condition: Store at 2°C to 8°C temperature.

	Prepared by	Checked by	Approved by
Name :	Mr. Sitaram Ubale	Mr. Rajendra Chavan	Ms. Mohini Khatik
Department :	AVL	AVL	CRDQ
Sign :	[signature]	[signature]	[signature]
Date :	05/04/2023	05/04/2023	05/04/2023

Format No. : AVL-045-F-02-00

Ranked # 1 in Healthcare
GREAT PLACE TO WORK® Best Companies to Work for 2015 India

ANALYTICAL RESEARCH

CERTIFICATE OF ANALYSIS FOR REFERENCE STANDARD

Name	:	Zidebactam (WCK 5107)			
Molecular Formula	:	$C_{13}H_{21}N_5O_7S.2H_2O$	Batch Number	:	WK1/WP-0060/016
Molecular Weight	:	427.45	Laboratory Note Book Reference	:	WA-2362-020
Date of Analysis	:	02/03/2023	Month of Manufacturing	:	May' 2019
Valid Up To	:	01/03/2024	Reference Standard Number	:	RS/2023-017
Effective Date	:	30/03/2023	Page Number	:	Page 4 of 4

Chemical Names:

i. **WCK 5107**: (2S,5R)-7-Oxo-6-sulphoxy-2-[N'-((R)-piperidin-3-carbonyl)hydrazinocarbonyl]-1,6-diaza-bicyclo[3.2.1]octane dihydrate

ii. **Impurity-1**: (2S,5R)-5-Sulphooxyamino-2-[N'-((R)-piperidin-3-carbonyl)hydrazinocarbonyl] piperidine

iii. **Impurity-2**: (2S,5R)-7-Oxo-6-hydroxy-2-[N'-((R)-piperidin-3-carbonyl)hydrazinocarbonyl]-1,6-diaza-bicyclo[3.2.1]octane

iv. **Impurity-3**: (2S,5R)-7-Oxo-6-sulphooxy-2-[N'-((S)-piperidin-3-carbonyl)hydrazinocarbonyl]-1,6-diaza-bicyclo[3.2.1]octane

v. **Impurity-4**: (2S,5R)-7-Oxo-6-sulphooxy-2-[N'-((R)-1-tert-butoxycarbonylpiperidin-3-yl)carbonyl) hydrazinocarbonyl]-1,6-diaza-bicyclo[3.2.1]octane

	Prepared by	Checked by	Approved by
Name :	Mr. Sitaram Ubale	Mr. Rajendra Chavan	Ms. Mohini Khatik
Department :	AVL	AVL	CRDQ
Sign :	[signature]	[signature]	[signature]
Date :	05/04/2023	05/04/2023	05/04/2023

Format No. : AVL-045-F-02-00

Ranked # 1 in Healthcare
GREAT PLACE TO WORK Best Companies to Work for 2015 India

1. TITLE: THE DETERMINATION OF FREE ERTAPENEM IN TREATED HUMAN PLASMA ULTRAFILTRATE, WITH SODIUM HEPARIN AS ANTICOAGULANT, BY LC-MS/MS

BIOANALYTICAL METHOD VALIDATION REPORT

Bioanalytical Project Number:	V1872106P3
Sponsor:	DynPort Vaccine Company, LLC<br>110 Thomas Johnson Drive<br>Suite 200<br>Frederick, MD 21702
Bioanalytical Laboratory:	KCAS Bioanalytical & Biomarker Services.<br>10830 S Clay Blair Boulevard<br>Olathe, KS 66061
Issue Date:	February 2023

2. SYNOPSIS

This report presents the validation data for an LC-MS/MS method for the determination of free ertapenem in treated human plasma ultrafiltrate, with sodium heparin as anticoagulant. The assay was analyzed in the range of 0.0500 to 50.0 mcg/mL and is for use on treated human plasma ultrafiltrate samples obtained during clinical trials.

3. TABLE OF CONTENTS FOR THE BIOANALYTICAL METHOD VALIDATION REPORT

1. TITLE: THE DETERMINATION OF FREE ERTAPENEM IN TREATED HUMAN PLASMA ULTRAFILTRATE, WITH SODIUM HEPARIN AS ANTICOAGULANT, BY LC-MS/MS 1

2. SYNOPSIS 2

3. TABLE OF CONTENTS FOR THE BIOANALYTICAL METHOD VALIDATION REPORT 3

4. LIST OF TABLES....5

5. LIST OF FIGURES 7

6. SIGNATURE PAGE 8

7. QUALITY ASSURANCE STATEMENT 9

8. STUDY COMPLIANCE STATEMENT 10

9. LIST OF ABBREVIATIONS AND DEFINITIONS OF TERMS....11

10. INTRODUCTION 13

11. EXPERIMENTAL DESIGN AND RESULTS 13

11.1. Reference Standards 13

11.2. Calibration Standards and QC Samples Preparation 14

11.3. Selectivity 14

11.3.1. Blank Evaluation 14

11.3.2. Matrix Effect....14

11.3.3. Interference Evaluation....15

11.3.4. Carryover 15

11.4. Linearity and Analytical Range 15

11.5. Precision and Accuracy 16

11.6. Sensitivity 16

11.7. Run Size....16

11.8. Precision and Accuracy of Dilution....17

11.9. Freeze/Thaw Stability....17

11.10. Ice-Water Bath Stability 17

11.11. Long-Term Matrix Stability at -20 °C and -70 °C 18
11.12. Processed Sample Stability when Refrigerated 18
11.13. Individual Sample Reinjection Reproducibility 18
11.14. Whole Run Reinjection Reproducibility 19
11.15. Column Ruggedness 19
11.16. Extraction Recovery 19
11.17. Solution Stabilities 20
11.18. Calculations 20
11.19. SOP Deviations and Investigations 20
12. CONCLUSION 21
13. PERTINENT SOPS 21
14. ARCHIVES 21
15. REFERENCES 22
16. TABLES 23
17. FIGURES 51

4. LIST OF TABLES

Table 1: Abbreviations and Codes 11
Table 2: Deviations 21
Table 3: Mass Spec Transitions 23
Table 4: Reference Standards 23
Table 5: Calibration Standards and QC Samples Preparation 24
Table 6: Matrix Description 25
Table 7: Summary of Results and Conclusions 26
Table 8: Summary of Validation Run Objectives and Outcome 30
Table 9: Carryover 31
Table 10: Selectivity - Blank Assessment 32
Table 11: Matrix Effect – Low Concentration 33
Table 12: Matrix Effect – High Concentration 34
Table 13: Interference Evaluation 35
Table 14: Summary of Standard Curves 36
Table 15: Precision and Accuracy (Intra-Assay) 37
Table 16: Precision and Accuracy (Inter-Assay) 38
Table 17: Standards and QCs of Non-P&A Runs 39
Table 18: Precision and Accuracy of Sample Dilution 40
Table 19: Freeze-Thaw Stability, (Frozen At -20 °C and -70 °C Thawed in Ice-Water Bath) 41
Table 20: Ice-Water Bath Stability 42
Table 21: Long-Term Stability at -20 °C and -70 °C 43
Table 22: Refrigerated Processed Sample Stability 44
Table 23: Individual Sample Reinjection Reproducibility 44
Table 24: Whole Run Reinjection Reproducibility and Column Ruggedness 45
Table 25: Extraction Recovery – Ertapenem 46
Table 26: Extraction Recovery – Ertapenem-d_4 (IS) 47

Table 27: Stability of Ertapenem Primary Solution in ~0.1M MES Buffer, pH 6.4 at Room Temperature 48
Table 28: Stability of Ertapenem Primary Solution in ~0.1M MES Buffer, pH 6.4 at -20 °C 49

5. LIST OF FIGURES

Figure 1: Typical Calibration Curve 51

Figure 2: Typical Ion Chromatogram Blank Human Plasma Ultrafiltrate Without Internal Standard 52

Figure 3: Typical Ion Chromatogram Blank Human Plasma Ultrafiltrate With Internal Standard 53

Figure 4: Typical Ion Chromatogram LLOQ Calibration Standard, Ertapenem = 0.0500 mcg/mL 54

Figure 5: Typical Ion Chromatogram Calibration Standard, Ertapenem = 20.0 mcg/mL 55

Figure 6: Typical Ion Chromatogram ULOQ Calibration Standard, ertapenem = 50.0 mcg/mL 56

Figure 7: Typical Ion Chromatogram Cross-Talk Evaluation High QC No IS 57

Annex 1 .... Test Procedure

Annex 2 .... Certificate(s) of Analysis

6. SIGNATURE PAGE

Bioanalytical Project Number: V1872106P3

Issue Date: February 2023

APPROVED

DocuSigned by:

Anika Pippin

Signer Name: Anika Pippin
Signing Reason: I approve this document
Signing Time: 08-Feb-2023 | 14:39 CST
BE3AA1198E40459E8A7E3C615D981D82

Anika Pippin, B.S.
Principal Investigator

REVIEWED

DocuSigned by:

Marsha Luna

Signer Name: Marsha Luna
Signing Reason: I have reviewed this document
Signing Time: 08-Feb-2023 | 14:26 CST
C9CC738561AF4CC1B375ED013DD1E4BD

Marsha Luna, B.S.
Management

7. QUALITY ASSURANCE STATEMENT

This Bioanalytical Method Validation Report has been found to describe accurately the methods and SOPs used and to accurately reflect the raw data collected. The Quality Assurance Unit initiated audits on this study and reported the findings to the Principal Investigator (PI) and Management as follows:

		Date(s) Sent to:	
**Inspection Date(s)**	**Inspection Phase**	**PI**	**Management**
30Nov2022	Data Audit	30Nov2022	12Dec2022
19Dec2022 and 20Dec2022	Data Audit	20Dec2022	29Dec2022
21Dec2022	Data Audit	21Dec2022	29Dec2022
05Jan2023	Draft Report Review	05Jan2023	30Jan2023
06Feb2023	Final Report Review	06Feb2023	06Feb2023

REVIEWED

DocuSigned by:

Laura Garrett

Signer Name: Laura Garrett
Signing Reason: I have reviewed this document
Signing Time: 08-Feb-2023 | 14:20 CST
25EEB8B628934C508B49FD5392D40731

Laura Garrett, B.S.
Quality Assurance Unit

8. STUDY COMPLIANCE STATEMENT

I, the undersigned, hereby declare that this study was conducted in compliance with the relevant bioanalytical aspects of FDA Guidance for Industry on Bioanalytical Methods Validation, May 2018 [1] and KCAS Bioanalytical & Biomarker Services (KCAS) procedures and that the work was performed by me, or under my direction, and the report reflects a true and accurate record of the results obtained.

The acceptance criteria are based on the FDA Guidance for Industry, Bioanalytical Method Validation (2018). [1]

APPROVED

DocuSigned by:

Anika Pippin

Signer Name: Anika Pippin
Signing Reason: I approve this document
Signing Time: 08-Feb-2023 | 14:39 CST
BE3AA1198E40459E8A7E3C615D981D82

Anika Pippin, B.S.
Principal Investigator

9. LIST OF ABBREVIATIONS AND DEFINITIONS OF TERMS

Table 1: Abbreviations and Codes

Abbreviation	Definition
<	Concentration less than the lowest acceptable calibration standard
>	Concentration exceeded the upper limit of calibration range (> ULOQ)
A	Amendment
AQL	Above Quantitation Limit
%Bias	Percentage measurement valid
Batch	Is the same as Run
BQL	Below Quantifiable Limit
Cal	Calibration standard
Conc.	Concentration
Conv.	Conversion
%CV	Coefficient of Variation
Dev	Deviation
Diff	Difference
EMA	European Medicines Agency
FDA	Food and Drug Administration
GLP	Good Laboratory Practice
HPLC	High Performance Liquid Chromatography
ID	Identification
IS	Internal Standard
ISR	Incurred Sample Reanalysis
K_2EDTA or K_3EDTA	Dipotassium or Tripotassium Ethylenediaminetetraacetic Acid
KCAS	KCAS Bioanalytical & Biomarker Services
LC-MS/MS	Liquid Chromatography Tandem Mass Spectrometry
LLOQ	Lower Limit of Quantitation
mcg or µg or ug	Microgram
mcL or µL or uL	Microliter

Table 1: Abbreviations and Codes (Continued)

Abbreviation	Definition
ME	Matrix Effect
MF	Matrix Factor
MHLW	Ministry of Health, Labour and Welfare of Japan
mL	Milliliter
MS	Mass Spectrometry
n	number
N.C.	Not Calculated
ND	No difference reported as ISR will be counted
ng	nanogram
n.report	No Data available to report
N/A or NA	Not Applicable
N.R.	Not Reported
P&A	Precision and Accuracy
p.a.r.	Peak Area Ratio
PI	Principal Investigator
QC	Quality Control Sample
QCs	Quality Control Samples
OECD	Organization for Economic Co-operation and Development
RT	Room Temperature
sd or SD	Standard deviation
Seq	Sequence
SOP (s)	Standard Operating Procedure (s)
temp	Temperature
TP	Test procedure
UHPLC	Ultra High Performance Liquid Chromatography
ULOQ	Upper Limit of Quantitation
WIS	Working Internal Standard

This is a basic list. Some of the abbreviations may not have been used in this report.

10. INTRODUCTION

The objective of this study was to perform a full validation of a bioanalytical method for the determination of free ertapenem in MES (0.1M [2-(N-Morpholino) ethane sulphonic acid hydrate buffer, pH 6.4])-treated human plasma ultrafiltrate (at a 1:1 ratio), with sodium heparin as anticoagulant. This validation report describes the selectivity, sensitivity, linearity, accuracy, precision, stability and recovery experiments based on an established bioanalytical test procedure.

The validation procedure was designed to meet or exceed the requirements of FDA Guidance for Industry on Bioanalytical Methods Validation, May 2018. The software programs used were Microsoft Office Excel 365, acquisition and processing software Analyst 1.7.2, and data management software Watson LIMS 7.5.

11. EXPERIMENTAL DESIGN AND RESULTS

In this method, ertapenem and internal standard, ertapenem-d_4 (IS), were released from human plasma ultrafiltrate using 96-well protein precipitation. After extraction, the sample was chromatographed using reversed phase HPLC with a Fortis Technology Ltd. Fortis C18 analytical column. Ertapenem and ertapenem-d_4 (IS) were detected by monitoring the precursor and product ions described in Table 3 using an Applied Biosystems Sciex API 5000 LC-MS/MS. The quantification was based on setting a calibration graph using the internal standard method based on bioanalytical test procedure TP-80238, entitled "LC-MS/MS Analysis of Free Ertapenem in Treated Human Plasma Ultrafiltrate (Sodium Heparin, 100 mcL, (0.0500 – 50.0 mcg/mL; Prior to MES Treatment)". The test procedure can be found in Annex 1.

Human plasma ultrafiltrate, screened for the lack of interference at the retention times of ertapenem and ertapenem-d_4 (IS), was diluted 1:1 with MES buffer, pH 6.4 for stabilization. Since the standards and QCs concentrations were already adjusted for the addition of MES during the preparation and are reported based on plasma ultrafiltrate volume only, the obtained free ertapenem concentration of the unknown study samples do not need to be adjusted for the dilution with MES buffer.

A summary of the validation results is provided in Table 7. A summary describing the objective(s) and outcome of each validation run attempted is provided in Table 8.

The method was validated within the analytical range of 0.0500 to 50.0 mcg/mL in human plasma ultrafiltrate. No development work was performed after the method was validated.

11.1. Reference Standards

Reference standards used during this validation are presented in Table 4.

11.2. Calibration Standards and QC Samples Preparation

Calibration standards and quality control pools were prepared by spiking appropriate levels of ertapenem into treated human plasma ultrafiltrate, with sodium heparin as anticoagulant. Primary reference standards were prepared in at least duplicate and compared within 5% of each other. Standards and QCs were prepared and stored frozen at KCAS at -70 °C. Stability QCs were also stored at -20 °C. Standards, QCs, and the critical solutions used during this validation are described in Table 5, and matrix used is described in Table 6.

11.3. Selectivity

Selectivity is the ability of the bioanalytical method to measure and differentiate ertapenem in the presence of components that may be expected to be present. These could include metabolites, impurities, degradants, or matrix components. The selectivity of this method was achieved by a sample clean-up procedure using protein precipitation followed by chromatography and specific detection of the precursor and product ions of each analyte using tandem mass spectrometry (MS/MS).

11.3.1. Blank Evaluation

A minimum of six different sources of plasma ultrafiltrate (sodium heparin as anticoagulant) were screened. To be deemed acceptable, the peak response observed must be ≤ 20.0% of the mean response of the lower limit of quantitation (LLOQ) and ≤ 5.0% of the mean response of the internal standard. If any lots fail, further evaluation is needed.

All matrix lots were found to be free of any significant interference at the retention times of the analytes of interest. Data is presented in Table 10. Typical chromatograms for blank plasma ultrafiltrate, with sodium heparin as anticoagulant, without and with internal standard are presented in Figure 2 and Figure 3, respectively. Cross talk was also evaluated by extracting the high QC with no internal standard. There are no apparent effects of cross talk, and a typical chromatogram is present in Figure 7.

11.3.2. Matrix Effect

Matrix effect in MS applications is defined as inter-matrix variation in signal due to co-eluting matrix substances which cause enhancement or suppression of the detector response for the measured analyte.

To evaluate matrix effect using matrix factor, ertapenem and ertapenem-d_4 (IS) were spiked in four replicates into six different lots of extracted human plasma ultrafiltrate, with sodium heparin as anticoagulant. Ertapenem and ertapenem-d_4 (IS) were also spiked in four replicates into a proper extracted solvent or neat solution for comparison.

For ertapenem and ertapenem-d_4 (IS), the matrix factor (MF) was calculated for each lot of human plasma ultrafiltrate using the following equation:

$$MF = \frac{\text{Peak Response in Presence of Matrix Substances}}{\text{Mean Peak Response in Absence of Matrix}}$$

The IS normalized MF was calculated using the following equation:

$$\text{IS Normalized MF} = \frac{MF_{Analyte}}{MF_{IS}}$$

The evaluation was conducted for ertapenem at a high (close to ULOQ) and a low concentration (maximum of three times the LLOQ) and then the MF and IS Normalized MF were calculated for each lot of matrix. To be deemed acceptable for matrix effect test, the %CV for IS Normalized MF must be ≤ 15.0% over all six lots.

Matrix effect data are presented in Table 11 to Table 12. The matrix effect results meet acceptance criteria.

11.3.3. Interference Evaluation

Assay selectivity was confirmed by spiking quality control pools with 150 mcg/mL zidebactam. These QC pools, containing low and high levels of ertapenem, were analyzed in replicates of six in a single run. To be considered acceptable, the mean %Bias at each level was also required to be within ± 15.0% and the %CV was required to be ≤ 15.0%.

The results presented in Table 13, indicate the presence of 150 mcg/mL zidebactam does not affect the accuracy or precision of the data.

11.3.4. Carryover

Carryover is evaluated within each run. It consists of measuring the response for each analyte and if it exceeds 20.0% of the response of LLOQ standard or other sample reflecting the lower sensitivity limit required, a carryover assessment is completed, and affected samples are rejected. The internal standard carryover evaluation is assessed within each run and if it exceeds 5.0% of the average internal standard response of acceptable standards and QCs, impact is evaluated within the run. Data are presented in Table 9.

11.4. Linearity and Analytical Range

The calibration curve was constructed with eight different, non-zero standard concentrations ranging from 0.0250 to 25.0 mcg/mL in treated human plasma ultrafiltrate (equivalent to 0.0500 to 50.0 mcg/mL in untreated human plasma ultrafiltrate), with sodium heparin as anticoagulant. Calibration standards were processed in duplicate and the calibration curve was evaluated using least squares linear regression with weighting $1/x^2$. The maximum allowable percent deviation from nominal for each standard was ± 15.0%, except at the LLOQ, where ± 20.0% was accepted. Any standard deviating by more than these limits was required to be excluded from the regression line. For the calibration curve to be accepted, at least 75% of the initial number of standards was required to remain in the curve.

The calibration curve was found to be linear. The data are presented in Table 14. A typical calibration curve is presented graphically in Figure 1. Typical chromatograms illustrating blank treated human plasma ultrafiltrate standards spiked with ertapenem at the LLOQ, mid, and high concentrations are presented in Figure 4 to Figure 6, respectively.

11.5. Precision and Accuracy

The precision of an assay is defined as the closeness in which replicate analyses agree and is reported as percent coefficient of variation (%CV). The accuracy of a method is defined as the closeness of a determined value to its true (nominal) value and is reported as %Bias. At least one precision and accuracy run was performed using standards prepared from one primary stock and the QCs were prepared from a separate primary stock. Inter-assay (between runs) precision and accuracy and intra-assay (within runs) precision and accuracy were assessed in three runs by analyzing six replicates each of QCs fortified at four separate concentrations (LLOQ, low, mid, and high). For inter- and intra-assay, the mean %Bias at each QC level was required to be within ±15.0% from its respective nominal concentration, except at the LLOQ, where ± 20.0% was accepted. The %CV at each QC level was required to be ≤ 15.0%, except at the LLOQ, where ≤ 20.0% was accepted.

The intra-assay and inter-assay precision and accuracy data for this method met the required criteria and are presented in Table 15 (intra-assay precision and accuracy) and Table 16 (inter-assay precision and accuracy).

Standards and QCs of non-P&A runs are reported in Table 17.

11.6. Sensitivity

The sensitivity of the method was determined in three runs by analyzing six replicates of QCs at the lower limit of quantitation (LLOQ) which were equal in concentration to the lowest calibration standard. The observed response at the LLOQ was required to be at least 5 times the response of noise in the human plasma ultrafiltrate blank, in addition to the precision and accuracy requirements outlined in the previous section.

The LLOQ for this method was determined to be 0.0500 mcg/mL, and the response at the LLOQ was at least 5 times the response of noise in the human matrix blank. Data are presented in Table 15 (intra-assay precision and accuracy) and Table 16 (inter-assay precision and accuracy).

11.7. Run Size

In order to evaluate the largest size run that can be used for study sample analysis, a run was processed with a set of 8 calibration standards and a minimum of 3 QC samples, processed in duplicate. In addition, at least 3 matrix blanks (one with internal standard and two without internal standard) were processed with the run. Run 5 was used for this assessment, and additional experiments were also analyzed which made the final run size 158.

In order for the run to be acceptable, at least one QC at each concentration level and two-thirds of the total number of QC samples were required to interpolate within ± 15.0% from nominal.

The standard and QC summary of run 5 is presented in Table 14 and Table 15 and acceptance criteria was met.

11.8. Precision and Accuracy of Dilution

To determine the accuracy of dilution, the high concentration QC spiked with ertapenem (37.5 mcg/mL) as well as an above-range QC (ARQC; 200 mcg/mL) were evaluated. All samples were extracted in six replicates according to the test procedure. To be deemed acceptable, the mean %Bias at each level must be within ± 15.0% with %CV ≤ 15.0%. The replicates were prepared as follows:

- Dilution factor 2x (high QC) = 50 mcL of sample+ 50 mcL of blank treated human plasma ultrafiltrate
- Dilution factor 5x (ARQC) = 20 mcL of sample+ 80 mcL of blank treated human plasma ultrafiltrate

Dilution precision and accuracy, using dilution factors of 2x and 5x, were proven to be valid. Data are presented in Table 18.

11.9. Freeze/Thaw Stability

Ertapenem stability in treated human plasma ultrafiltrate, with sodium heparin as anticoagulant, during sequential freeze/thaw cycles (frozen separately at -20 °C and -70 °C and thawed unassisted in an ice-water bath) with and without the presence of comeds[2] was evaluated. Aliquots of high and low concentration QC samples were frozen for at least 12 hours before the initial thawing. They were re-frozen 2 more times for at least 12 hours each time and were thawed prior to assay along with a freshly prepared set of QCs and calibration standards. To be deemed stable, the mean %Bias at each level must be within ± 15.0% with %CV ≤ 15.0%.

Ertapenem in treated human plasma ultrafiltrate with and without the presence of comeds[2], with sodium heparin as anticoagulant, was proven stable over 3 cycles of freeze (-70 °C) and thaw (ice-water bath).

Ertapenem in treated human plasma ultrafiltrate with and without the presence of comeds[2], with sodium heparin as anticoagulant, was proven unstable over 3 cycles of freeze (-20 °C) and thaw (ice-water bath).

Data are provided in Table 19.

11.10. Ice-Water Bath Stability

To evaluate ertapenem in treated human plasma ultrafiltrate, with sodium heparin as anticoagulant, stability during sample processing in an ice-water bath, aliquots of the high and

low concentration QC pools with and without the presence of comeds[2] were allowed to thaw in an ice-water bath for 24 hours, prior to analysis along with a freshly prepared set of QCs and calibration standards. To be deemed stable, the mean %Bias at each level must be within ± 15.0% with %CV ≤ 15.0%.

Ertapenem in treated human plasma ultrafiltrate with and without the presence of comeds[2], with sodium heparin as anticoagulant, was proven stable in an ice-water bath under normal laboratory lighting over a period of 24 hours. Data are provided in Table 20.

11.11. Long-Term Matrix Stability at -20 °C and -70 °C

To evaluate ertapenem in human plasma ultrafiltrate, with sodium heparin as anticoagulant, stability during sample storage, aliquots of the high and low concentration QC pools with and without the presence of comeds[2] were stored frozen at -20 °C and -70 °C nominally for 28 days (without comeds[2]) and 27 days (with comeds[2]) prior to being analyzed, along with freshly prepared standards and QC samples. To be deemed stable, the mean %Bias at each level must be within ± 15.0% with %CV ≤ 15.0%.

Ertapenem in treated human plasma ultrafiltrate with and without the presence of comeds[2], with sodium heparin as anticoagulant, was proven to be stable at -70 °C over a period of 27 days (with comeds[2]) and 28 days (without comeds[2]) in polypropylene tubes.

Ertapenem in treated human plasma ultrafiltrate with and without the presence of comeds[2], with sodium heparin as anticoagulant, was proven not to be stable at -20 °C over a period of 27 days (with comeds[2]) and 28 days (without comeds[2]) in polypropylene tubes. All -20 °C LTS in this run failed %Bias criteria. Unknown samples should be stored at -70 °C.

Data are provided in Table 21.

11.12. Processed Sample Stability when Refrigerated

To evaluate ertapenem stability in processed samples at refrigerated temperatures, high and low concentration QC pools were processed, placed into polypropylene 96 well plates, and stored at refrigerated temperatures before being chromatographed with samples in a subsequent run. To be deemed stable, the mean %Bias at each level must be within ± 15.0% with %CV ≤ 15.0%.

Processed ertapenem samples were proven stable when refrigerated stored in polypropylene 96-well plates over a period of 5 days 22 hours. Data are provided in Table 22.

11.13. Individual Sample Reinjection Reproducibility

To evaluate individual sample reinjection reproducibility, low and high QC extracts (6 each) injected during the run were reinjected at the end of the run, within the established stability time window of processed samples in the refrigerator. To be deemed reproducible, the mean %Bias at each level must be within ± 15.0% with %CV ≤ 15.0%.

Reinjection of individual samples can be made with adequate precision and accuracy. Data are provided in Table 23.

11.14. Whole Run Reinjection Reproducibility

To evaluate whole run reinjection reproducibility, run 4 was reinjected as run 7 on the same MS platform using a different chromatographic column from the same manufacturer, with the same specification as the original within 4 days 11 hours following the original injection. To be deemed acceptable, the reinjected run was required to meet the acceptance criteria outlined in Sections 11.4, 11.5, and 11.6.

It has been demonstrated that entire runs can be reinjected with adequate accuracy and precision. Data are presented in Table 24.

11.15. Column Ruggedness

Column ruggedness was assessed by reinjecting run 4 as run 7 on the same MS platform (Applied Biosystems API5000) using a different chromatographic column from the same manufacturer, with the same specification as the original.

Data were required to meet the precision and accuracy criteria outlined in Sections 11.4, 11.5, and 11.6 to be considered as reproducible/rugged method. Data are presented in Table 24. It has been demonstrated that ruggedness has been met.

11.16. Extraction Recovery

To evaluate the extraction recovery, the low, mid, and high extracted QC samples were compared to extracted human plasma ultrafiltrate blanks to which an equal amount of ertapenem at each level, assuming 100% recovery, was added in six replicates.

To evaluate the extraction recovery of ertapenem-d_4 (IS), ertapenem was added only for tracking during analysis. Ertapenem-d_4 (IS) (IS) for the control was added after extraction, while the same amount of ertapenem-d_4 (IS) (IS) for the extracted QC was added before extraction.

$$\% \text{ Recovery} = \frac{\text{Response ratio of "Extracted QC"}}{\text{Average Response Ratio of Control}} \times 100$$

The mean recovery of ertapenem was determined to be consistent, precise and reproducible at 91.1%.

The recovery of ertapenem-d_4 (IS) (IS) was determined to be consistent, precise and reproducible at 134.5%. Data are presented in Table 25 to Table 26.

11.17. Solution Stabilities

The stability of various solutions of ertapenem in various solvents used during the sample analysis following storage at various temperatures was determined. Ertapenem-d_4 (IS) is a deuterated analog, so its stability at room temperature is taken as equivalent to ertapenem.

Data showing the ertapenem(s), solvent composition, storage term, and temperature are presented in Table 27 and Table 28.

Solution stabilities previously conducted in other studies.

11.18. Calculations

The peak areas of ertapenem and ertapenem-d_4 (IS) were integrated by Analyst 1.7.2 (Applied Biosystems; Foster City, CA, USA). The data results were imported electronically into Watson LIMS 7.5 for the calculation of ertapenem concentrations and statistical analysis. The calibration curves were obtained by a weighted (1/concentration^2) least squares linear regression. After the best fit from data, the concentrations (mcg/mL) were calculated based on the measured peak area ratios of ertapenem/ertapenem-d_4 (IS) using the following equation:

Response = Slope * Conc + Intercept

$$\text{Mean Value } (\overline{\text{X}}) = \frac{\sum_{i=1}^{n} \text{individual data (X)}}{\text{number of data points (n)}}$$

$$\text{Standard Deviation (SD)} = \sqrt{\frac{\sum_{i=1}^{n} \left[\text{individual data (X)} - \text{Mean Value } (\overline{\text{X}})\right]^2}{\text{n-1}}}$$

$$\text{Coefficient of Variation (\%CV)} = \left[\frac{\text{Standard Deviation (SD)}}{\text{Mean Value } (\overline{\text{X}})}\right] \times 100$$

$$\text{\%Bias} = \left(\frac{\text{Calculated Mean of the Observed Values or Observed Value} - \text{Nominal Value}}{\text{Nominal Value}}\right) \times 100$$

All calibration standards and QCs concentrations were reported to three significant figures, and study samples will be reported to three significant figures.

11.19. SOP Deviations and Investigations

One TP deviation occurred during this study. No investigations were conducted during this study.

Table 2: Deviations

Deviation #	Description	Impact
DEV-2022-0275	Per test procedure 90/10 Acetonitrile/deionized water is to be used as mobile phase B. In runs 6 and 7 per lot# documentation on the acquisition sheet, 90/10 Acetone/deionized water was used instead. Both runs meet all acceptance criteria and the chromatography (including retention time) is very similar to previous runs. Data is accepted.	There is no impact.

12. CONCLUSION

Validation results obtained in this study indicate that a robust and reliable method has been established for the measurement of ertapenem in human plasma Ultrafiltrate, with sodium heparin as anticoagulant, by LC-MS/MS in the concentration range of 0.0500 to 50.0 mcg/mL. The generally recommended acceptance criteria, FDA Guidance for Industry on Bioanalytical Methods Validation, May 2018, in terms of precision, accuracy, selectivity including matrix effect, recovery and all types of stability have been met. Thus, this validated method is deemed suitable for determination of ertapenem in human plasma ultrafiltrate, with sodium heparin as anticoagulant, for unknown samples.

13. PERTINENT SOPS

The following SOPs were followed during the conduct of this study:

SOP-00073 [02] - Bioanalytical Method Validation - Chromatographic Assays

SOP-00035 [00] - Procedures, Reporting, and Acceptance Criteria for Bioanalytical Chromatographic Projects

14. ARCHIVES

All raw data, including paper and electronic files associated with this study will remain archived at a location specified by KCAS, 10830 S Clay Blair Boulevard, Olathe, KS 66061 for a period of at least 10 years, after which the client may be contacted to arrange for continued storage of the files, or disposition according to an agreed procedure. KCAS will maintain an electronic (scanned/true) copy of the method validation report that can be viewed and printed on site. The study plan, documentation, raw data, and final report are indexed under Bioanalytical Project Number V1872106P3.

15. REFERENCES

1. Guidance for Industry: Bioanalytical Method Validation. U.S. Department of Health and Human Services. Food and Drug Administration. Center for Drug Evaluation and Research. Center for Veterinary Medicine. May 2018.
2. Comeds: QCs in the presence of 150 mcg/mL zidebactam.

16. TABLES

Table 3: Mass Spec Transitions

Analyte	Transition
Ertapenem	m/z 476.047→431.9
Ertapenem-d_4 (IS)	m/z 480.2→436.3

Table 4: Reference Standards

Analyte	Supplier	Lot/Batch Number	Purity	Expiration Date or Retest Date	Conversion Factor
Ertapenem Sodium	MedChemExpress	180017	98.9%	Retest 11 Aug 2024	1.046
Ertapenem-d_4 Sodium Salt (IS)	AlsaChim	DH-ALS-20-002-P2	*	Expire 29 Apr 2023	*
Zidebactam	Wockhardt	WK1/WP-0060/016	90.4%	Expire 29 Mar 2023	1.000

Certificates of analysis for the above reference standards are presented in Annex 2.
*Used as is with 1.000 conversion and 100% purity as IS only.

Table 5: Calibration Standards and QC Samples Preparation

Critical Solutions	Lot # for Standards (Prep Date)	Lot # for QCs (Prep Date)	Standards Lot (Prep Date)	QCS Lot (Prep Date)	Runs used
Human Plasma Ultrafiltrate Treated with MES Buffer, ~0.1M, pH 6.4 (1:1)<br>Lot V1872106P3-15-1			V1872106P3-13-1<br>Set A<br>(10Nov2022)	V1872106P3-14-1<br>Set A<br>(10Nov2022)	1, 3<br>QCs only:<br>2, 9 (LTS)
MES Buffer, pH 6.4 stored frozen (-20°C) in polypropylene	V1872106P3-1-1<br>(09Nov2022)	V1872106P3-2-1<br>(09Nov2022)			
Human Plasma Ultrafiltrate Treated with MES Buffer, ~0.1M, pH 6.4 (1:1) stored frozen (-70°C) in polypropylene	V1872106P3-12-1<br>(10Nov2022)	V1872106P3-12-2<br>(10Nov2022)			
Human Plasma Ultrafiltrate Treated with MES Buffer, ~0.1M, pH 6.4 (1:1)<br>Lot V1872106P3-15-1			NA	V1872106P3-22-1<br>Set A<br>(11Nov2022)	Comed QCs:<br>3, 9 (LTS)
Deionized water stored frozen (-20°C) in polypropylene	NA	V1872106P4-1-1<br>(27Oct2022)			
MES Buffer, pH 6.4 stored frozen (-20°C) in polypropylene	NA	V1872106P3-2-1<br>(09Nov2022)			
Human Plasma Ultrafiltrate Treated with MES Buffer, ~0.1M, pH 6.4 (1:1) stored frozen (-70°C) in polypropylene	NA	V1872106P3-12-2<br>(10Nov2022)			
Human Plasma Ultrafiltrate Treated with MES Buffer, ~0.1M, pH 6.4 (1:1)<br>Lot V1872106P3-25-1			V1872106P3-25-2<br>Set B<br>(14Nov2022)	V1872106P3-26-1<br>Set B<br>(14Nov2022)	4
MES Buffer, pH 6.4 stored frozen (-20°C) in polypropylene	V1872106P3-1-1<br>(09Nov2022)	V1872106P3-2-1<br>(09Nov2022)			
Human Plasma Ultrafiltrate Treated with MES Buffer, ~0.1M, pH 6.4 (1:1) stored frozen (-70°C) in polypropylene	V1872106P3-12-1<br>(10Nov2022)	V1872106P3-12-2<br>(10Nov2022)			
Human Plasma Ultrafiltrate Treated with MES Buffer, ~0.1M, pH 6.4 (1:1)<br>Lot V1872106P3-25-1			V1872106P3-27-1<br>Set C<br>(16Nov2022)	V1872106P3-28-1<br>Set C<br>(16Nov2022)	5, 6
MES Buffer, pH 6.4 stored frozen (-20°C) in polypropylene	V1872106P3-1-1<br>(09Nov2022)	V1872106P3-1-1<br>(09Nov2022)			
Human Plasma Ultrafiltrate Treated with MES Buffer, ~0.1M, pH 6.4 (1:1) stored frozen (-70°C) in polypropylene	V1872106P3-12-1<br>(10Nov2022)	V1872106P3-30-1<br>(16Nov2022)			
Human Plasma Ultrafiltrate Treated with MES Buffer, ~0.1M, pH 6.4 (1:1)<br>Lot V1872106P4-29-2			V1872106P3-36-1<br>Set D<br>(08Dec2022)	V1872106P3-37-1<br>Set D<br>(08Dec2022)	9
MES Buffer, pH 6.4 stored frozen (-20°C) in polypropylene	V1872106P3-31-1<br>(07Dec2022)	V1872106P3-32-1<br>(07Dec2022)			
Human Plasma Ultrafiltrate Treated with MES Buffer, ~0.1M, pH 6.4 (1:1) stored frozen (-70°C) in polypropylene	V1872106P3-35-1<br>(08Dec2022)	V1872106P3-35-2<br>(08Dec2022)			

Table 6: Matrix Description

Matrix	Source Lot #	Date Received
Human Plasma Ultrafiltrate Treated with MES Buffer, ~0.1M, pH 6.4 (1:1) Lot V1872106P3-15-1	BioIVT Combo-70-1 (HMN790515, HMN790517, HMN790518, HMN790520, HMN790521, HMN790523)	Apr 2022
Human Plasma Ultrafiltrate Treated with MES Buffer, ~0.1M, pH 6.4 (1:1) Lot V1872106P3-25-1	BioIVT Combo-70-1 (HMN790515, HMN790517, HMN790518, HMN790520, HMN790521, HMN790523)	Apr 2022
Human Plasma Ultrafiltrate Treated with MES Buffer, ~0.1M, pH 6.4 (1:1) Lot V1872106P4-29-2	BioIVT HMN790516	Apr 2022

Table 7: Summary of Results and Conclusions

Methodology – TP-80238[D00]	Method Performance – LC-MS/MS	Hyperlink – N/A	Comments
Matrix	Plasma ultrafiltrate		
Anticoagulant	Sodium heparin		
Assay Volume	100 mcL		
**Selectivity**			
Analyte	Ertapenem	Summary tables<br>Table 10<br><br>Report text<br>Section 11.3.1	
Internal Standard	Ertapenem-d_4 (IS)	Summary tables<br>Table 10<br><br>Report text<br>Section 11.3.1	
Matrix Effect<br>(6 spiked matrix lots)	Complies	Summary tables<br>Table 11,Table 12<br><br>Report text<br>Section 11.3.2	
Interference	Complies	Summary tables<br>Table 13<br><br>Report text<br>Section 11.3.3	
Carryover	Assessed with every run with standards and QCs	Summary tables<br>Table 9<br><br>Report text<br>Section 11.3.4	
**Linearity**			
Weighting	$1/x^2$	Report text<br>Section 11.4	
Bias at LLOQ	-0.6%	Summary tables<br>Table 14<br><br>Report text<br>Section 11.4	
Bias above LLOQ	-4.4 to 3.0%	Summary tables<br>Table 14<br><br>Report text<br>Section 11.4	
Bioanalytical Range	Complies<br>0.0500 to 50.0 mcg/mL	Summary tables<br>Table 14<br><br>Report text<br>Section 11.4	

Table 7: Summary of Results and Conclusions (Continued)

**Methodology – TP-80238[D00]**	**Method Performance – LC-MS/MS**	**Hyperlink – N/A**	**Comments**
**Precision**			
Intra-assay	5.5 to 10.2% (LLOQ);<br>1.0 to 5.2% (above LLOQ)	Summary tables<br>Table 15<br><br>Report text<br>Section 11.5	
Inter-assay	16.0% (LLOQ);<br>4.3 to 9.0% (above LLOQ)	Summary tables<br>Table 16<br><br>Report text<br>Section 11.5	
**Accuracy**			
Intra-assay	-15.6 to 13.4% (LLOQ);<br>-11.3 to 4.7% (above LLOQ)	Summary tables<br>Table 15<br><br>Report text<br>Section 11.5	
Inter-assay	-5.2% (LLOQ);<br>-7.5 to -6.0% (above LLOQ)	Summary tables<br>Table 16<br><br>Report text<br>Section 11.5	
**Large Run Size**	Determined to be 158 samples.	Summary tables<br>Table 14 and Table 15<br><br>Report text<br>Section 11.7	
**Dilution**			
Dilution factor: 2x	Dilution QC: 37.5 mcg/mL<br>Precision: 2.1%<br>Accuracy: -0.8%	Summary tables<br>Table 18<br><br>Report text<br>Section 11.8	
Dilution factor: 5x	Dilution QC: 200 mcg/mL<br>Precision: 2.7%<br>Accuracy: -2.0%	Summary tables<br>Table 18<br><br>Report text<br>Section 11.8	
**Stability**			
Matrix (in polypropylene tubes)			
Freeze/Thaw (-20 °C/ice-water bath)	Demonstrated instability at 3 cycles	Summary tables<br>Table 19<br><br>Report text<br>Section 11.9	

Table 7: Summary of Results and Conclusions (Continued)

**Methodology – TP-80238[D00]**	**Method Performance – LC-MS/MS**	**Hyperlink – N/A**	**Comments**
Freeze/Thaw (-70 °C/ice-water bath) with and without the presence of comeds[2]	Demonstrated for 3 cycles	Summary tables<br>Table 19<br><br>Report text<br>Section 11.9	
Ice-Water Bath	Demonstrated for 24 hours	Summary tables<br>Table 20<br><br>Report text<br>Section 11.10	
Long-term (-20 °C) with and without the presence of comeds[2]	Demonstrated instability at 28 days without comeds[2]<br>Demonstrated instability at 27 days with comeds[2]	Summary tables<br>Table 21<br><br>Report text<br>Section 11.11	
Long-term (-70 °C) with and without the presence of comeds[2]	Demonstrated for 28 days without comeds[2]<br>Demonstrated for 27 days with comeds[2]	Summary tables<br>Table 21<br><br>Report text<br>Section 11.11	
Processed Matrix polypropylene			
Refrigeration	Demonstrated for 5 days 22 hours	Summary tables<br>Table 22<br><br>Report text<br>Section 11.12	
**Individual Sample Reinjection**	Reproducible	Summary tables<br>Table 23<br><br>Report text<br>Section 11.13	
**Whole Run Reinjection Integrity**	Reproducible<br>Duration 4 days 11 hours	Summary tables<br>Table 24<br><br>Report text<br>Section 11.14	
**Column Ruggedness**	Reproducible	Summary tables<br>Table 24<br><br>Report text<br>Section 11.15	

Table 7: Summary of Results and Conclusions (Continued)

**Methodology – TP-80238[D00]**	**Method Performance – LC-MS/MS**	**Hyperlink – N/A**	**Comments**
**Extraction Recovery**			
Ertapenem	Overall 91.1%	Summary tables<br>Table 25<br><br>Report text<br>Section 11.16	
Ertapenem-d_4 (IS)	Overall 134.5%	Summary tables<br>Table 26<br><br>Report text<br>Section 11.16	
**Solution Stability**			
Solution, Primary polypropylene			
Ertapenem in MES buffer (RT)	Stable 6.75 hours	Summary tables<br>Table 27	V1872106P5
Ertapenem in MES buffer (-20 °C)	Stable 7 days	Summary tables<br>Table 28	V1872106P5
Ertapenem in MES buffer (-20 °C)	Instability observed at 28 days	Summary tables<br>Table 28	V1872106P3
Ertapenem-d_4 (IS) in MES buffer (RT)	Taken to be same as ertapenem	SOP 00073	
Ertapenem-d_4 (IS) in MES buffer (-20 °C)	1 year stability can be assigned and evaluated thereafter in yearly increments	SOP 00073	

Table 8: Summary of Validation Run Objectives and Outcome

Run ID	Instrument ID	Extraction Date	Assay Date	Run Objective(s)	Accepted or Rejected	Comment
1	LC/MS-22	10-Nov-2022	10-Nov-2022	Precision and accuracy, cross-talk, blank selectivity, individual sample reinjection reproducibility, extraction of refrigerated processed stability samples	Accepted	[a]
2	LC/MS-22	10-Nov-2022	11-Nov-2022	Matrix effect with matrix factor and recovery	Accepted	
3	LC/MS-22	11-Nov-2022	14-Nov-2022	Comed[2] interference	Accepted	
4	LC/MS-22	14-Nov-2022	14-Nov-2022	Precision and accuracy and dilution integrity	Accepted	
5	LC/MS-22	16-Nov-2022	16-Nov-2022	Precision and accuracy, bench-top stability with and without comeds[2], freeze/thaw stability with and without comeds[2], injection of refrigerated processed stability, large run size	Accepted	[b]
6	LC/MS-22	18-Nov-2022	19-Nov-2022	Precision and accuracy	Accepted	
7	LC/MS-22	14-Nov-2022	19-Nov-2022	Whole run reinjection reproducibility and column ruggedness	Accepted	
8	LC/MS-22	07-Dec-2022	07-Dec-2022	Primary comparison and primary long-term solution stability	Rejected	%Change >15.0%
9	LC/MS-39	08-Dec-2022	09-Dec-2022	Long-term matrix stability with and without comeds[2]	Accepted	[c]

[a]Batch fails criteria for all LLOQ QCs and cannot be used for precision and accuracy, but all run acceptance criteria are met and the run passes. The LLOQ QC failure appears to be due to a preparation error, not a method issue as the %CV across all six extracted aliquots meets criteria (8.7%), but the %Bias for all individual samples as well as the overall &Bias are outside of the ± 20% criterion. Cross-talk samples show a peak at the IS retention time, but it is below 5% and acceptance criteria are met.

[b]Freeze/thaw for comed and analyte at -20 °C/ice-water failed %Bias criteria for both high and low concentrations. Analyte does not appear stable at -20 °C freeze/thaw. Unknown samples should be stored at -70 °C

[c]All -20 °C LTS in this run fail %Bias criteria. Unknown samples should be stored at -70 °C.

Table 9: Carryover

Run ID	Result (Extracted Blank after High Standard)	Carryover Results for Internal Standard
1	Seq 13 and Seq 64 are <20.0% of the LLOQ.	Seq 4 and Seq 55 are < 5.0% internal standard peak of acceptable standards.
3	Seq 13 and Seq 44 are <20.0% of the LLOQ.	Seq 4 and Seq 35 are < 5.0% internal standard peak of acceptable standards.
4	Seq 13 and Seq 62 are <20.0% of the LLOQ.	Seq 4 and Seq 53 are < 5.0% internal standard peak of acceptable standards.
5	Seq 13 and Seq 146 are <20.0% of the LLOQ.	Seq 4 and Seq 137 are < 5.0% internal standard peak of acceptable standards.
6	Seq 13 and Seq 50 are <20.0% of the LLOQ.	Seq 4 and Seq 41 are < 5.0% internal standard peak of acceptable standards.
7	Seq 13 and Seq 50 are <20.0% of the LLOQ.	Seq 4 and Seq 41 are < 5.0% internal standard peak of acceptable standards.
9	Seq 13 and Seq 80 are <20.0% of the LLOQ.	Seq 4 and Seq 71 are < 5.0% internal standard peak of acceptable standards.

Note: Calibration Standards and QCs are arranged in a way to mitigate carryover in all validation runs.

Table 10: Selectivity - Blank Assessment

Run 1 Selectivity Blank Screen

Blank ID with or without IS	Blank Lot Number	Peak Area of Analyte	Peak Area of Internal Standard	Percent of LLOQ Peak Area	Percent of LLOQ Peak Area Ratio	Percent of IS in Blanks without IS
Blank 1 NO IS	V1872106P4-8-2	0	0	0.0	NA	0.0
Blank 2 NO IS	V1872106P4-8-3	0	0	0.0	NA	0.0
Blank 3 NO IS	V1872106P4-8-4	0	0	0.0	NA	0.0
Blank 4 NO IS	V1872106P4-8-5	0	0	0.0	NA	0.0
Blank 5 NO IS	V1872106P4-8-6	0	0	0.0	NA	0.0
Blank 6 NO IS	V1872106P4-8-7	0	0	0.0	NA	0.0
Blank 1	V1872106P4-8-2	0	507823	0.0	0.0	NA
Blank 2	V1872106P4-8-3	0	482617	0.0	0.0	NA
Blank 3	V1872106P4-8-4	0	488367	0.0	0.0	NA
Blank 4	V1872106P4-8-5	0	502241	0.0	0.0	NA
Blank 5	V1872106P4-8-6	0	460301	0.0	0.0	NA
Blank 6	V1872106P4-8-7	0	471912	0.0	0.0	NA

Note: Average IS response are from all of the accepted standards and QCs

Criteria: A minimum of six different sources of plasma ultrafiltrate (sodium heparin as anticoagulant) were screened. To be deemed acceptable, the peak response observed must be ≤ 20.0% of the mean response of the lower limit of quantitation (LLOQ) and ≤ 5.0% of the mean response of the internal standard. If any lots fail, further evaluation is needed.

Results: All matrix lots were found to be free of any significant interference at the retention times of the analytes of interest.

Table 11: Matrix Effect – Low Concentration

Run Number: 2
Concentration: 0.150 mcg/mL

Sample ID	Drug in neat/matrix Area	Average Drug in Neat	Individual MF analyte	Average MF analyte	IS in neat/ matrix Area	Average IS in neat Area	Individual IS MF	Average IS MF	Average IS Normalized MF
Neat	12890	13126			343067	344636			
	12846				341987				
	13865				347462				
	12901				346029				
ME1	12840		0.9782	0.9619	337012		0.9779	0.9493	1.0133
	12550		0.9562		315917		0.9167		
	12433		0.9472		324397		0.9413		
	12679		0.9660		331318		0.9614		
ME2	13136		1.0008	0.9902	331804		0.9628	0.9600	1.0315
	12673		0.9655		327623		0.9506		
	13268		1.0109		332168		0.9638		
	12912		0.9837		331812		0.9628		
ME3	12853		0.9792	0.9787	330663		0.9595	0.9614	1.0180
	12834		0.9778		338495		0.9822		
	12776		0.9734		329280		0.9554		
	12920		0.9843		326875		0.9485		
ME4	12963		0.9876	0.9794	330757		0.9597	0.9661	1.0137
	12958		0.9872		334498		0.9706		
	12798		0.9750		336407		0.9761		
	12699		0.9675		330162		0.9580		
ME5	12819		0.9766	0.9760	323014		0.9373	0.9598	1.0169
	13168		1.0032		332993		0.9662		
	12598		0.9598		333739		0.9684		
	12658		0.9644		333344		0.9672		
ME6	13355		1.0175	0.9947	337568		0.9795	0.9701	1.0254
	12786		0.9741		332331		0.9643		
	13109		0.9987		330508		0.9590		
	12975		0.9885		336905		0.9776		
**Mean**									1.0198
**N**									6
**SD**									0.00720
**%CV**									0.7

neat = in absence of matrix, matrix = in presence of extracted matrix
Criteria: %CV of Average IS Normalized MF among 6 lots of matrix must be ≤ 15.0%.
Meets Acceptance Criteria: YES MF=matrix factor

$$\text{MF} = \frac{\text{Peak Response in Presence of Matrix Substances}}{\text{Mean Peak Response in Absence of Matrix}}$$

$$\text{IS Normalized MF} = \frac{\text{MF}_{Analyte}}{\text{MF}_{IS}}$$

Table 12: Matrix Effect – High Concentration

Run Number:		2							
Concentration:		37.5 mcg/mL							
**Sample ID**	**Drug in neat/matrix Area**	**Average Drug in Neat**	**Individual MF analyte**	**Average MF analyte**	**IS in neat/ matrix Area**	**Average IS in neat Area**	**Individual IS MF**	**Average IS MF**	**Average IS Normalized MF**
Neat	3178202	3216679			353437	358322			
	3237667				366097				
	3220849				355132				
	3229997				358621				
ME1	3020362		0.9390	0.9692	339310		0.9469	0.9521	1.0180
	3153817		0.9805		343128		0.9576		
	3148635		0.9788		339998		0.9489		
	3147996		0.9786		342170		0.9549		
ME2	3150425		0.9794	0.9676	345022		0.9629	0.9583	1.0096
	3095150		0.9622		342536		0.9559		
	3102009		0.9644		338124		0.9436		
	3101805		0.9643		347895		0.9709		
ME3	3093929		0.9618	0.9614	343429		0.9584	0.9561	1.0055
	3098969		0.9634		345553		0.9644		
	3109072		0.9665		345775		0.9650		
	3068280		0.9539		335637		0.9367		
ME4	3074875		0.9559	0.9527	338072		0.9435	0.9456	1.0075
	2859271		0.8889		321614		0.8976		
	3168818		0.9851		352875		0.9848		
	3154723		0.9807		342717		0.9565		
ME5	3073281		0.9554	0.9554	347235		0.9691	0.9562	0.9992
	3138593		0.9757		342681		0.9563		
	3025920		0.9407		342522		0.9559		
	3054838		0.9497		338019		0.9433		
ME6	3100812		0.9640	0.9560	341830		0.9540	0.9332	1.0244
	3089522		0.9605		328377		0.9164		
	3066273		0.9532		334596		0.9338		
	3043886		0.9463		332807		0.9288		
**Mean**									1.0107
**N**									6
**SD**									0.00907
**%CV**									0.9

neat = in absence of matrix, matrix = in presence of extracted matrix
Criteria: %CV of Average IS Normalized MF among 6 lots of matrix must be ≤ 15.0%.
Meets Acceptance Criteria: YES MF=matrix factor

$$MF = \frac{\text{Peak Response in Presence of Matrix Substances}}{\text{Mean Peak Response in Absence of Matrix}}$$

$$\text{IS Normalized MF} = \frac{MF_{Analyte}}{MF_{IS}}$$

Table 13: Interference Evaluation

Run Date	Curve Number	Comed1 low 0.150 µg/mL	%Bias	Comed1 high 37.5 µg/mL	%Bias
14-Nov-2022	3	0.152	1.3	35.1	-6.4
		0.153	2.0	35.7	-4.8
		0.151	0.7	35.1	-6.4
		0.152	1.3	35.0	-6.7
		0.153	2.0	35.1	-6.4
		0.162	8.0	35.0	-6.7
Mean		0.154		35.2	
SD		0.00407		0.266	
%CV		2.6		0.8	
%Bias		2.7		-6.1	
n		6		6	

Criteria: To be considered acceptable, the mean %Bias at each level was also required to be within ± 15.0% and the %CV was required to be ≤ 15.0%.

Results: The acceptance criteria were met.

Table 14: Summary of Standard Curves

Analytical Performance: Back-Calculated Concentrations (µg/mL) of Ertapenem Calibration Standard in (Human) (Plasma Ultrafiltrate) in (Study V1872106P3)

Assay Date	Analytical Run Number	Cal 0.05 0.0500 µg/mL	Cal 0.1 0.100 µg/mL	Cal 1 1.00 µg/mL	Cal 5 5.00 µg/mL	Cal 10 10.0 µg/mL	Cal 20 20.0 µg/mL	Cal 40 40.0 µg/mL	Cal 50 50.0 µg/mL
14-Nov-2022	4	0.0486	0.104	0.978	5.17	10.2	19.8	39.5	46.4
		0.0496	0.103	1.01	5.15	10.2	20.1	39.1	48.9
16-Nov-2022	5	0.0462	0.0972	0.976	4.99	9.75	18.9	37.1	44.8
		0.0530	0.105	1.08	5.37	10.6	21.1	40.6	49.9
19-Nov-2022	6	0.0515	0.103	1.01	5.16	10.5	20.0	39.1	48.6
		0.0491	0.0942	1.03	5.02	10.3	19.8	38.6	48.0
Mean		0.0497	0.101	1.01	5.14	10.3	20.0	39.0	47.8
S.D.		0.00236	0.00433	0.0384	0.135	0.297	0.706	1.15	1.86
%CV		4.7	4.3	3.8	2.6	2.9	3.5	2.9	3.9
%Bias		-0.6	1.0	1.0	2.8	3.0	0.0	-2.5	-4.4
n		6	6	6	6	6	6	6	6

Calibration Curve Parameters for Ertapenem Calibration Standards in Human Plasma Ultrafiltrate (Study V1872106P3)

Run Date	Curve Number	Slope	Intercept	R-Squared	LLOQ	ULOQ
14-Nov-2022	4	0.09055	0.00003	0.9989	0.0500	50.0
16-Nov-2022	5	0.09863	0.00037	0.9958	0.0500	50.0
19-Nov-2022	6	0.10628	0.00016	0.9988	0.0500	50.0

See Table 8 for runs not used for precision/accuracy evaluation.

Table 15: Precision and Accuracy (Intra-Assay)

Human Plasma Ultrafiltrate Ertapenem Concentrations in Quality Controls for 3 separate batch runs (Study V1872106P3)

Run Date	Curve Number	QCS 0.05 0.0500 µg/mL	%Bias	QCS 0.15 0.150 µg/mL	%Bias	QCS 25 25.0 µg/mL	%Bias	QCS 37.5 37.5 µg/mL	%Bias
14-Nov-2022	4	0.0590	18.0	0.155	3.3	25.1	0.4	37.0	-1.3
		0.0546	9.2	0.149	-0.7	24.4	-2.4	37.9	1.1
		0.0566	13.2	0.162	8.0	24.7	-1.2	36.8	-1.9
		0.0590	18.0	0.158	5.3	25.0	0.0	35.8	-4.5
		0.0595	19.0	0.161	7.3	24.8	-0.8	37.2	-0.8
		0.0516	3.2	0.156	4.0	24.6	-1.6	36.4	-2.9
Intrarun Mean		0.0567		0.157		24.8		36.9	
Intrarun SD		0.00313		0.00471		0.258		0.715	
Intrarun %CV		5.5		3.0		1.0		1.9	
Intrarun %Bias		13.4		4.7		-0.8		-1.6	
n		6		6		6		6	
16-Nov-2022	5	0.0421	-15.8	##0.126	-16.0	22.7	-9.2	33.8	-9.9
		0.0421	-15.8	0.129	-14.0	22.8	-8.8	33.2	-11.5
		0.0401	-19.8	0.132	-12.0	23.0	-8.0	32.6	-13.1
		0.0453	-9.4	0.140	-6.7	23.4	-6.4	33.5	-10.7
		0.0490	-2.0	0.141	-6.0	23.2	-7.2	33.1	-11.7
		0.0418	-16.4	0.133	-11.3	22.4	-10.4	34.5	-8.0
Intrarun Mean		0.0434		0.134		22.9		33.5	
Intrarun SD		0.00322		0.00596		0.360		0.653	
Intrarun %CV		7.4		4.4		1.6		1.9	
Intrarun %Bias		-13.2		-10.7		-8.4		-10.7	
n		6		6		6		6	
19-Nov-2022	6	0.0474	-5.2	0.140	-6.7	23.0	-8.0	34.3	-8.5
		0.0462	-7.6	##0.126	-16.0	22.9	-8.4	33.3	-11.2
		#0.0379	-24.2	0.135	-10.0	21.8	-12.8	33.7	-10.1
		#0.0377	-24.6	##0.127	-15.3	23.0	-8.0	34.4	-8.3
		0.0443	-11.4	0.129	-14.0	22.6	-9.6	34.1	-9.1
		#0.0395	-21.0	0.142	-5.3	22.9	-8.4	33.7	-10.1
Intrarun Mean		0.0422		0.133		22.7		33.9	
Intrarun SD		0.00432		0.00685		0.465		0.422	
Intrarun %CV		10.2		5.2		2.0		1.2	
Intrarun %Bias		-15.6		-11.3		-9.2		-9.6	
n		6		6		6		6	
# > 20%Bias									
## > 15%Bias									

Criteria: At each level, the %CV was required to be ≤ 15.0%, except at the LLOQ level, where ≤ 20.0% was accepted. The mean %Bias at each level was also required to be within ± 15.0%, except at the LLOQ, where ± 20.0% was accepted.

Results: The acceptance criteria were met. Run size (n=158) was completed in run 5 and met acceptance criteria.

Table 16: Precision and Accuracy (Inter-Assay)

Human Plasma Ultrafiltrate Ertapenem Concentrations in Quality Controls for 3 separate batch runs (Study V1872106P3)

Run Date	Curve Number	QCS 0.05 0.0500 µg/mL	%Bias	QCS 0.15 0.150 µg/mL	%Bias	QCS 25 25.0 µg/mL	%Bias	QCS 37.5 37.5 µg/mL	%Bias
14-Nov-2022	4	0.0590	18.0	0.155	3.3	25.1	0.4	37.0	-1.3
		0.0546	9.2	0.149	-0.7	24.4	-2.4	37.9	1.1
		0.0566	13.2	0.162	8.0	24.7	-1.2	36.8	-1.9
		0.0590	18.0	0.158	5.3	25.0	0.0	35.8	-4.5
		0.0595	19.0	0.161	7.3	24.8	-0.8	37.2	-0.8
		0.0516	3.2	0.156	4.0	24.6	-1.6	36.4	-2.9
16-Nov-2022	5	0.0421	-15.8	##0.126	-16.0	22.7	-9.2	33.8	-9.9
		0.0421	-15.8	0.129	-14.0	22.8	-8.8	33.2	-11.5
		0.0401	-19.8	0.132	-12.0	23.0	-8.0	32.6	-13.1
		0.0453	-9.4	0.140	-6.7	23.4	-6.4	33.5	-10.7
		0.0490	-2.0	0.141	-6.0	23.2	-7.2	33.1	-11.7
		0.0418	-16.4	0.133	-11.3	22.4	-10.4	34.5	-8.0
19-Nov-2022	6	0.0474	-5.2	0.140	-6.7	23.0	-8.0	34.3	-8.5
		0.0462	-7.6	##0.126	-16.0	22.9	-8.4	33.3	-11.2
		#0.0379	-24.2	0.135	-10.0	21.8	-12.8	33.7	-10.1
		#0.0377	-24.6	##0.127	-15.3	23.0	-8.0	34.4	-8.3
		0.0443	-11.4	0.129	-14.0	22.6	-9.6	34.1	-9.1
		#0.0395	-21.0	0.142	-5.3	22.9	-8.4	33.7	-10.1
Mean Concentration Found (µg/mL)		0.0474		0.141		23.5		34.7	
Inter-run SD		0.00757		0.0127		1.02		1.65	
Inter-run %CV		16.0		9.0		4.3		4.8	
Inter-run %Bias		-5.2		-6.0		-6.0		-7.5	
n		18		18		18		18	
# > 20%Bias									
## > 15%Bias									

Criteria: At each level, the %CV was required to be ≤ 15.0%, except at the LLOQ level, where ≤ 20.0% was accepted. In addition, the mean %Bias at each level was required to be within ± 15.0% from nominal value, except at the LLOQ, where ± 20.0% was accepted.

Results:The acceptance criteria were met.

Table 17: Standards and QCs of Non-P&A Runs

Analytical Performance: Back-Calculated Concentrations (μg/mL) of Ertapenem Calibration Standard in (Human) (Plasma Ultrafiltrate) in (Study V1872106P3)

Assay Date	Analytical Run Number	Cal 0.05 0.0500 μg/mL	Cal 0.1 0.100 μg/mL	Cal 1 1.00 μg/mL	Cal 5 5.00 μg/mL	Cal 10 10.0 μg/mL	Cal 20 20.0 μg/mL	Cal 40 40.0 μg/mL	Cal 50 50.0 μg/mL
10-Nov-2022	1	0.0505	0.0882	0.999	4.89	9.77	19.1	38.2	45.4
		0.0512	0.104	1.08	5.41	10.7	20.5	40.3	50.3
14-Nov-2022	3	0.0493	0.0982	0.998	5.17	10.0	20.0	39.2	47.2
		0.0487	0.109	1.04	5.33	9.99	19.6	38.6	48.0
09-Dec-2022	9	0.0516	0.113	1.01	5.53	9.25	19.4	39.6	48.9
		0.0476	0.0898	1.02	5.08	9.94	20.6	39.2	48.2
Mean		0.0498	0.100	1.02	5.24	9.94	19.9	39.2	48.0
S.D.		0.00155	0.0101	0.0313	0.234	0.467	0.606	0.739	1.65
%CV		3.1	10.1	3.1	4.5	4.7	3.0	1.9	3.4
%Bias		-0.4	0.0	2.0	4.8	-0.6	-0.5	-2.0	-4.0
n		6	6	6	6	6	6	6	6

Calibration Curve Parameters for Ertapenem Calibration Standards in Human Plasma Ultrafiltrate (Study V1872106P3)

Run Date	Curve Number	Slope	Intercept	R-Squared	LLOQ	ULOQ
10-Nov-2022	1	0.08883	0.00014	0.9959	0.0500	50.0
14-Nov-2022	3	0.09661	0.00019	0.9980	0.0500	50.0
09-Dec-2022	9	0.17233	0.00059	0.9957	0.0500	50.0

Human Plasma Ultrafiltrate Ertapenem Concentrations in Quality Controls for 3 separate batch runs (Study V1872106P3)

Run Date	Curve Number	QCS 0.05 0.0500 μg/mL	%Bias	QCS 0.15 0.150 μg/mL	%Bias	QCS 25 25.0 μg/mL	%Bias	QCS 37.5 37.5 μg/mL	%Bias
10-Nov-2022	1	#0.0657	31.4	0.146	-2.7	23.5	-6.0	34.8	-7.2
		#0.0608	21.6	0.145	-3.3	22.9	-8.4	34.6	-7.7
		#0.0634	26.8	0.149	-0.7	23.5	-6.0	35.0	-6.7
		#0.0768	53.6	0.150	0.0	23.7	-5.2	35.2	-6.1
		#0.0680	36.0	0.151	0.7	23.9	-4.4	36.4	-2.9
		#0.0724	44.8	0.144	-4.0	23.8	-4.8	36.2	-3.5
Intrarun Mean		0.0679		0.148		23.6		35.4	
Intrarun SD		0.00592		0.00288		0.356		0.753	
Intrarun %CV		8.7		1.9		1.5		2.1	
Intrarun %Bias		35.8		-1.3		-5.6		-5.6	
n		6		6		6		6	
14-Nov-2022	3			0.151	0.7	23.8	-4.8	35.2	-6.1
				0.158	5.3	24.3	-2.8	35.7	-4.8
Intrarun Mean				0.155		24.1		35.5	
Intrarun %Bias				3.3		-3.6		-5.3	
09-Dec-2022	9			0.157	4.7	25.1	0.4	38.0	1.3
				0.158	5.3	25.1	0.4	37.0	-1.3
Intrarun Mean				0.158		25.1		37.5	
Intrarun %Bias				5.3		0.4		0.0	
# > 20%Bias									

Criteria: For each run, the calculated concentrations of at least 75% of the calibration standards are required to interpolate within ± 15.0% from nominal with a minimum of six calibration standard levels, except for the LLOQ for which it was required to be within ± 20.0%. In order for the run to be acceptable, at least one QC at each concentration level and two-thirds of the total number of QC samples were required to interpolate within ± 15.0% from nominal.

Results: Acceptance criteria were met.

Table 18:Precision and Accuracy of Sample Dilution

**Dilution factor**		**X2**		**X5**	
**Run Date**	**Curve Number**	**Dil QC 37.5 37.5 µg/mL**	**%Bias**	**Dil QC 200 200 µg/mL**	**%Bias**
14-Nov-2022	4	36.8	-1.9	199	-0.5
		36.1	-3.7	201	0.5
		37.9	1.1	197	-1.5
		36.7	-2.1	188	-6.0
		38.2	1.9	191	-4.5
		37.3	-0.5	200	0.0
Mean		37.2		196	
SD		0.789		5.29	
%CV		2.1		2.7	
%Bias		-0.8		-2.0	
n		6		6	

Sample dilution as follows:

2x =50 mcL of sample+ 50 mcL of blank treated human plasma ultrafiltrate
5x =20 mcL of sample+ 80 mcL of blank treated human plasma ultrafiltrate

Criteria: To be deemed acceptable, the mean %Bias at each level must be within ± 15.0% with %CV ≤ 15.0%.
Results: The acceptance criteria were met.

Table 19: Freeze-Thaw Stability, (Frozen At -20 °C and -70 °C Thawed in Ice-Water Bath)

Without comeds[2]

Run Date	Curve Number	F/T -20/RT low 0.150 µg/mL	%Bias	F/T -70/RT low 0.150 µg/mL	%Bias	F/T -20/RT high 37.5 µg/mL	%Bias	F/T -70/RT high 37.5 µg/mL	%Bias
16-Nov-2022	5	##0.119	-20.7	0.150	0.0	##30.1	-19.7	35.0	-6.7
		##0.124	-17.3	0.141	-6.0	##30.6	-18.4	35.8	-4.5
		##0.123	-18.0	0.141	-6.0	##29.8	-20.5	35.3	-5.9
		##0.127	-15.3	0.151	0.7	##30.5	-18.7	37.3	-0.5
		##0.127	-15.3	0.154	2.7	##30.2	-19.5	36.5	-2.7
		0.128	-14.7	0.155	3.3	##30.0	-20.0	35.6	-5.1
Mean		0.125		0.149		30.2		35.9	
SD		0.00339		0.00622		0.303		0.847	
%CV		2.7		4.2		1.0		2.4	
%Bias		-16.7		-0.7		-19.5		-4.3	
n		6		6		6		6	
## > 15%Bias									

With comeds[2]

Run Date	Curve Number	Comed1 F/T -20 low 0.150 µg/mL	%Bias	Comed1 F/T -70 low 0.150 µg/mL	%Bias	Comed1 F/T -20 high 37.5 µg/mL	%Bias	Comed1 F/T -70 high 37.5 µg/mL	%Bias
16-Nov-2022	5	0.128	-14.7	0.151	0.7	##30.1	-19.7	36.6	-2.4
		##0.122	-18.7	0.149	-0.7	##29.7	-20.8	35.8	-4.5
		0.128	-14.7	0.151	0.7	##30.0	-20.0	34.9	-6.9
		0.130	-13.3	0.156	4.0	##30.2	-19.5	36.4	-2.9
		##0.123	-18.0	0.150	0.0	##31.4	-16.3	36.4	-2.9
		##0.126	-16.0	0.166	10.7	##30.3	-19.2	36.0	-4.0
Mean		0.126		0.154		30.3		36.0	
SD		0.00313		0.00643		0.585		0.621	
%CV		2.5		4.2		1.9		1.7	
%Bias		-16.0		2.7		-19.2		-4.0	
n		6		6		6		6	
## > 15%Bias									

Criteria: To be deemed stable, the mean %Bias at each level must be within ± 15.0% with %CV ≤ 15.0%.

Results: Ertapenem is stable in treated human plasma ultrafiltrate with and without presence comeds[2], with sodium heparin as anticoagulant, for at least 3 cycles when frozen at -70 °C and thawed in ice-water bath.
Ertapenem is unstable in treated human plasma ultrafiltrate with and without presence comeds[2], with sodium heparin as anticoagulant, for at least 3 cycles when frozen at -20 °C and thawed in ice-water bath.

Table 20: Ice-Water Bath Stability

Without comeds[2]

Run Date	Curve Number	BT 24hr low 0.150 µg/mL	%Bias	BT 6hr low 0.150 µg/mL	%Bias	BT 24hr high 37.5 µg/mL	%Bias	BT 6hr high 37.5 µg/mL	%Bias
16-Nov-2022	5	0.153	2.0	0.149	-0.7	36.3	-3.2	35.7	-4.8
		0.139	-7.3	0.144	-4.0	35.4	-5.6	34.4	-8.3
		0.148	-1.3	0.144	-4.0	35.7	-4.8	35.8	-4.5
		0.145	-3.3	0.156	4.0	37.2	-0.8	35.7	-4.8
		0.150	0.0	0.155	3.3	35.0	-6.7	35.1	-6.4
		0.149	-0.7	0.148	-1.3	36.1	-3.7	36.8	-1.9
Mean		0.147		0.149		36.0		35.6	
SD		0.00484		0.00520		0.771		0.799	
%CV		3.3		3.5		2.1		2.2	
%Bias		-2.0		-0.7		-4.0		-5.1	
n		6		6		6		6	

With comeds[2]

Run Date	Curve Number	Comed1 BT 24hr low 0.150 µg/mL	%Bias	Comed1 BT 6hr low 0.150 µg/mL	%Bias	Comed1 BT 24hr high 37.5 µg/mL	%Bias	Comed1 BT 6hr high 37.5 µg/mL	%Bias
16-Nov-2022	5	0.156	4.0	0.158	5.3	35.7	-4.8	36.4	-2.9
		0.144	-4.0	0.150	0.0	35.5	-5.3	35.2	-6.1
		0.148	-1.3	0.153	2.0	34.3	-8.5	36.4	-2.9
		0.158	5.3	0.165	10.0	36.6	-2.4	36.8	-1.9
		0.156	4.0	0.154	2.7	35.5	-5.3	36.0	-4.0
		0.149	-0.7	0.151	0.7	35.9	-4.3	36.4	-2.9
Mean		0.152		0.155		35.6		36.2	
SD		0.00560		0.00556		0.749		0.551	
%CV		3.7		3.6		2.1		1.5	
%Bias		1.3		3.3		-5.1		-3.5	
n		6		6		6		6	

Criteria: To be deemed stable, the mean %Bias at each level must be within ± 15.0% with %CV ≤ 15.0%.

Results: Ertapenem is stable in treated human plasma ultrafiltrate with and without presence comeds[2], with sodium heparin as anticoagulant, for at least 24 hours in an ice-water bath.

Table 21: Long-Term Stability at -20 °C and -70 °C

Without comeds[2]

Run Date	Curve Number	LTS -20 low 0.150 µg/mL	%Bias	LTS -70 low 0.150 µg/mL	%Bias	LTS -20 high 37.5 µg/mL	%Bias	LTS -70 high 37.5 µg/mL	%Bias
09-Dec-2022	9	##0.0621	-58.6	0.160	6.7	##16.5	-56.0	41.0	9.3
		##0.0682	-54.5	0.167	11.3	##15.7	-58.1	39.8	6.1
		##0.0719	-52.1	0.158	5.3	##16.7	-55.5	40.0	6.7
		##0.0707	-52.9	0.171	14.0	##16.2	-56.8	38.5	2.7
		##0.0719	-52.1	##0.175	16.7	##16.2	-56.8	39.3	4.8
		##0.0592	-60.5	0.169	12.7	##14.9	-60.3	39.8	6.1
Mean		0.0673		0.167		16.0		39.7	
SD		0.00543		0.00653		0.650		0.824	
%CV		8.1		3.9		4.1		2.1	
%Bias		-55.1		11.3		-57.3		5.9	
n		6		6		6		6	
## > 15%Bias									

With comeds[2]

Run Date	Curve Number	Comed1 LTS -20 low 0.150 µg/mL	%Bias	Comed1 LTS -70 low 0.150 µg/mL	%Bias	Comed1 LTS -20 high 37.5 µg/mL	%Bias	Comed1 LTS -70 high 37.5 µg/mL	%Bias
09-Dec-2022	9	##0.0820	-45.3	##0.184	22.7	##17.0	-54.7	40.5	8.0
		##0.0703	-53.1	0.160	6.7	##17.5	-53.3	37.8	0.8
		##0.0795	-47.0	0.172	14.7	##16.0	-57.3	38.3	2.1
		##0.0718	-52.1	0.164	9.3	##16.5	-56.0	38.7	3.2
		##0.0681	-54.6	0.164	9.3	##17.1	-54.4	38.1	1.6
		##0.0665	-55.7	0.170	13.3	##16.2	-56.8	40.1	6.9
Mean		0.0730		0.169		16.7		38.9	
SD		0.00630		0.00856		0.578		1.12	
%CV		8.6		5.1		3.5		2.9	
%Bias		-51.3		12.7		-55.5		3.7	
n		6		6		6		6	
## > 15%Bias									

Criteria: To be deemed stable, the mean %Bias at each level must be within ± 15.0% with %CV ≤ 15.0%.

Results: Ertapenem is stable in treated human plasma ultrafiltrate with and without presence comeds[2], with sodium heparin as anticoagulant, for up to 27 days (with comeds[2]) and 28 days (without comeds[2]) upon storage in polypropylene tube at -70 °C.
Ertapenem is not stable in treated human plasma ultrafiltrate with and without presence comeds[2], with sodium heparin as anticoagulant, for up to 27 days (with comeds[2]) and 28 days (without comeds[2]) upon storage in polypropylene tube at -20 °C. All -20 °C LTS in this run failed %Bias criteria.

Table 22: Refrigerated Processed Sample Stability

Run Date	Curve Number	Processed Refr low 0.150 µg/mL	%Bias	Processed Refr high 37.5 µg/mL	%Bias
16-Nov-2022	5	0.144	-4.0	36.3	-3.2
		0.149	-0.7	36.7	-2.1
		0.153	2.0	37.1	-1.1
		0.150	0.0	35.8	-4.5
		0.146	-2.7	36.7	-2.1
		0.149	-0.7	35.9	-4.3
Mean		0.149		36.4	
SD		0.00315		0.508	
%CV		2.1		1.4	
%Bias		-0.7		-2.9	
n		6		6	

Samples were processed with run 1, and then stored for 5 days 22 hours in polypropylene 96 well plates at refrigerated temperatures prior to injection with run 5.

Criteria: To be deemed stable, the mean %Bias at each level must be within ± 15.0% with %CV ≤ 15.0%.
Results: The acceptance criteria were met.

Table 23: Individual Sample Reinjection Reproducibility

Run Date	Curve Number	Indiv Reinj low 0.150 µg/mL	%Bias	Indiv Reinj high 37.5 µg/mL	%Bias
10-Nov-2022	1	0.156	4.0	35.9	-4.3
		0.154	2.7	36.3	-3.2
		0.148	-1.3	36.2	-3.5
		0.159	6.0	35.6	-5.1
		0.161	7.3	36.1	-3.7
		0.149	-0.7	37.5	0.0
Mean		0.155		36.3	
SD		0.00524		0.653	
%CV		3.4		1.8	
%Bias		3.3		-3.2	
n		6		6	

Low and high QC extracts (6 each) were injected during the run and then reinjected at the end of the same run.
Criteria: To be deemed reproducible, the mean %Bias at each level must be within ± 15.0% with %CV ≤ 15.0%.
Results: The acceptance criteria were met.

Table 24: Whole Run Reinjection Reproducibility and Column Ruggedness

Analytical Performance: Back-Calculated Concentrations (µg/mL) of Ertapenem Calibration Standard in (Human) (Plasma Ultrafiltrate) in (Study V1872106P3)

Assay Date	Analytical Run Number	Cal 0.05 0.0500 µg/mL	Cal 0.1 0.100 µg/mL	Cal 1 1.00 µg/mL	Cal 5 5.00 µg/mL	Cal 10 10.0 µg/mL	Cal 20 20.0 µg/mL	Cal 40 40.0 µg/mL	Cal 50 50.0 µg/mL
19-Nov-2022	7	0.0488	0.0922	1.00	5.04	10.5	20.2	39.1	49.5
		0.0538	0.0975	0.998	5.10	10.1	20.0	40.5	48.9
Mean		0.0513	0.0949	0.999	5.07	10.3	20.1	39.8	49.2
%Bias		2.6	-5.1	-0.1	1.4	3.0	0.5	-0.5	-1.6

Calibration Curve Parameters for Ertapenem Calibration Standards in Human Plasma Ultrafiltrate (Study V1872106P3)

Run Date	Curve Number	Slope	Intercept	R-Squared	LLOQ	ULOQ
19-Nov-2022	7	0.10469	-0.00059	0.9986	0.0500	50.0

Human Plasma Ultrafiltrate Ertapenem Concentrations in Quality Controls for 1 separate batch runs (Study V1872106P3)

Run Date	Curve Number	QCS 0.05 0.0500 µg/mL	%Bias	QCS 0.15 0.150 µg/mL	%Bias	QCS 25 25.0 µg/mL	%Bias	QCS 37.5 37.5 µg/mL	%Bias
19-Nov-2022	7	0.0520	4.0	#0.173	15.3	25.7	2.8	37.1	-1.1
		0.0565	13.0	0.151	0.7	25.8	3.2	37.6	0.3
		0.0547	9.4	#0.177	18.0	24.6	-1.6	37.1	-1.1
		0.0562	12.4	0.151	0.7	25.4	1.6	37.6	0.3
		0.0532	6.4	0.148	-1.3	25.6	2.4	36.9	-1.6
		0.0587	17.4	0.165	10.0	25.4	1.6	36.6	-2.4
Mean		0.0552		0.161		25.4		37.2	
SD		0.00243		0.0125		0.431		0.394	
%CV		4.4		7.8		1.7		1.1	
%Bias		10.4		7.3		1.6		-0.8	
n		6		6		6		6	
# > 15%Bias									

Note: Run 7 is a reinjection of run 4 on a different analytical column of the same manufacturer, dimensions, and type, using same MS platform (Applied Biosystems API5000) – reinjected within 4 days 11 hours

Criteria: At each level, the %CV was required to be ≤ 15.0% from the nominal value, except at the LLOQ level, where ≤ 20.0% was accepted. The mean %Bias at each level was also required to be within ± 15.0%, except at the LLOQ, where ± 20.0% was accepted.

Results: The acceptance criteria were met.

Table 25: Extraction Recovery – Ertapenem

Run: 2

Control (Post-extraction Spike)					Extracted QCs				
QC Conc. *mcg/mL*	Drug Peak Area	Drug Peak Area (corrected)	IS Peak Area	Peak Area Ratio	QC Conc. *mcg/mL*	Drug Peak Area	IS Peak Area	Peak Area Ratio	Extraction Recovery %
Low QC					Low QC				
0.150	12376	6188	376267	0.0164	0.150	5519	395211	0.0140	87.1
	12997	6499	396093	0.0164		5496	361515	0.0152	94.8
	12570	6285	383745	0.0164		6498	455779	0.0143	88.9
	12146	6073	385368	0.0158		6251	438607	0.0143	88.9
	12245	6123	439052	0.0139		5212	387789	0.0134	83.8
	12530	6265	362408	0.0173		5070	374153	0.0136	84.5
			Average	0.0160				Average	88.0
Mid QC					Mid QC				
25.0	1997616	998808	406541	2.4568	25.0	957888	430918	2.2229	92.4
	2104720	1052360	449475	2.3413		929909	421807	2.2046	91.6
	2005344	1002672	448873	2.2338		1056136	468175	2.2559	93.8
	2060851	1030426	393964	2.6155		1004821	454091	2.2128	92.0
	2072662	1036331	413699	2.5050		1017155	439504	2.3143	96.2
	2031612	1015806	444997	2.2827		848637	387784	2.1884	91.0
			Average	2.4059				Average	92.8
High QC					High QC				
37.5	3046525	1523263	407791	3.7354	37.5	1497614	450465	3.3246	93.3
	3008830	1504415	418843	3.5918		1496751	442781	3.3803	94.9
	3027576	1513788	462535	3.2728		1242868	383178	3.2436	91.0
	2975735	1487868	401401	3.7067		1263367	380992	3.3160	93.1
	2982422	1491211	394296	3.7820		1352994	410627	3.2949	92.5
	2951808	1475904	448421	3.2913		1323737	412711	3.2074	90.0
			Average	3.5633				Average	92.5
									======
								n:	18
								Overall Mean Recovery:	**91.1**
								Overall Standard Deviation:	**3.4**
								Overall % CV:	**3.7**

It was found that recovery solutions were calculated based on reported concentrations in plasma instead of actual concentrations in treated matrix (which are half of the reported concentrations). A correction factor of 0.5 is applied to the areas of the post extraction spike samples.

$$\% \text{ Recovery} = \frac{\text{Response Ratio of "Extracted QC"}}{\text{Average Response Ratio of Control}} \times 100$$

Table 26: Extraction Recovery – Ertapenem-d_4 (IS)

Run: 2

Control				Extracted QCs				
WIS Conc.	IS Peak Area	Drug Peak Area	Peak Area Ratio	WIS Conc.	IS Peak Area	Drug Peak Area	Peak Area Ratio	Extraction Recovery
5.0 mcg/mL				*5.0 mcg/mL*				%
	310831	920914	0.3375		430918	957888	0.4499	135.1
	317681	1038482	0.3059		421807	929909	0.4536	136.2
	328740	1059601	0.3102		468175	1056136	0.4433	133.1
	312650	914365	0.3419		454091	1004821	0.4519	135.7
	326147	899390	0.3626		439504	1017155	0.4321	129.7
	312949	920794	0.3399		387784	848637	0.4569	137.2
		Average	0.3330					======
							n:	6
							Mean Recovery:	**134.5**
							Standard Deviation:	**2.7**
							%CV:	**2.0**

$$\% \text{ Recovery} = \frac{\text{Response Ratio of "Extracted QC"}}{\text{Average Response Ratio of Control}} \times 100$$

Table 27: Stability of Ertapenem Primary Solution in ~0.1M MES Buffer, pH 6.4 at Room Temperature

V1872106P5 Run: 4
Container: Polypropylene

Primary Solution - Solution Stability at Room Temperature									
Concentration: 8.00 mg/mL									
Stability Data (Room Temperature)					Comparison Data (4°C)				
Lot #:	V1872106P5-14-1				Lot #:	V1872106P5-14-2			
Prepared:	3-Nov-2022				Prepared:	3-Nov-2022			
Sample ID	Seq #	Drug Peak Area	IS Peak Area	Peak Area Ratio	Sample ID	Seq #	Drug Peak Area	IS Peak Area	Peak Area Ratio
Primary BT-1	7	5620573	88122	63.7817	Primary Refr-1	5	5960585	91974	64.8073
Primary BT-1	13	6621884	95052	69.6659	Primary Refr-1	11	5865724	92488	63.4215
Primary BT-1	19	7062212	113045	62.4726	Primary Refr-1	17	6074087	95377	63.6850
Primary BT-2	8	5736744	88649	64.7130	Primary Refr-2	6	4583100	70979	64.5698
Primary BT-2	14	6152635	92769	66.3221	Primary Refr-2	12	5209699	84342	61.7687
Primary BT-2	20	7002169	106053	66.0252	Primary Refr-2	18	7361633	107453	68.5103
			Mean	65.4968				Mean	64.4604
			n	6				n	6
			SD	2.4917				SD	2.2565
			%CV	3.8				%CV	3.5
%Change =	1.6								
Duration:	6.75	Hours							

$$\%Change = \frac{\text{mean response of stored solutions} - \text{mean response of comparison solutions}}{\text{mean response of comparison solutions}} \times 100$$

Criteria: To be deemed stable, % Change upon storage for analyte must be within ±15.0% and %CV must be within ≤15.0%.
Meets Criteria: YES

Table 28: Stability of Ertapenem Primary Solution in ~0.1M MES Buffer, pH 6.4 at -20 °C

V1872106P5 Run: 10
Container: Polypropylene

Concentration: 8.00 mg/mL									
Stability Data (Stored -20°C)					Comparison Data (Fresh)				
Lot #:	V1872106P5-2-1				Lot #:	V1872106P5-38-1			
Prepared:	3-Nov-2022				Prepared:	10-Nov-2022			
Sample ID	Seq #	Drug Peak Area	IS Peak Area	Peak Area Ratio	Sample ID	Seq #	Drug Peak Area	IS Peak Area	Peak Area Ratio
Primary stored-1	4	5817264	30968	187.8476	Primary fresh-1	3	5482273	32017	171.2301
Primary stored-1	8	5954724	31229	190.6793	Primary fresh-1	7	6302709	32203	195.7181
Primary stored-1	12	5658237	34334	164.7998	Primary fresh-1	11	6405482	36041	177.7276
Primary stored-2	6	5097485	30613	166.5137	Primary fresh-2	5	6041908	32145	187.9579
Primary stored-2	10	4919923	28241	174.2121	Primary fresh-2	9	5971914	32011	186.5582
Primary stored-2	14	5479678	31178	175.7546	Primary fresh-2	13	6214568	31401	197.9099
			Mean	176.6345				Mean	186.1836
			n	6				n	6
			SD	10.6969				SD	10.2646
			%CV	6.1				%CV	5.5
%Change =	-5.1								
Duration:	7	Days							

$$\%\text{Change} = \frac{\text{mean response of stored solutions} - \text{mean response of comparison solutions}}{\text{mean response of comparison solutions}} \times 100$$

Criteria: To be deemed stable, % Change upon storage for analyte must be within ±15.0% and %CV must be within ≤15.0%.
Meets Criteria: YES

Table 28: Stability of Ertapenem Primary Solution in ~0.1M MES Buffer, pH 6.4 at -20 °C (Continued)

V1872106P3 Run: 8
Container: Polypropylene

Concentration: 2.00 mg/mL									
Comparison Data (Fresh)<br>Lot A#:<br>Lot B#:<br>Prepared:	V1872106P3-31-1, Aliquot#1<br>V1872106P3-32-1, Aliquot#1<br>07Dec2022				Stability Data (Stored)<br>Lot #:<br>Prepared:	V1872106P3-2-1, Aliquot#2<br>09Nov2022			
Sample ID	Seq #	Drug Peak Area	IS Peak Area	Peak Area Ratio	Sample ID	Seq #	Drug Peak Area	IS Peak Area	Peak Area Ratio
8 003 V1872106P3 Primary Comp A-1 1	3	264722	221207	1.1967	8 015 V1872106P3 Primary LTS-1 1	15	232805	234939	0.9909
8 004 V1872106P3 Primary Comp A-1 1	4	263152	223928	1.1752	8 016 V1872106P3 Primary LTS-1 1	16	227469	233390	0.9746
8 005 V1872106P3 Primary Comp A-1 1	5	278647	231788	1.2022	8 017 V1872106P3 Primary LTS-1 1	17	235699	243720	0.9671
8 006 V1872106P3 Primary Comp A-2 1	6	269478	234103	1.1511	8 018 V1872106P3 Primary LTS-2 1	18	232952	238850	0.9753
8 007 V1872106P3 Primary Comp A-2 1	7	279094	234714	1.1891	8 019 V1872106P3 Primary LTS-2 1	19	234257	241490	0.9700
8 008 V1872106P3 Primary Comp A-2 1	8	277949	229934	1.2088	8 020 V1872106P3 Primary LTS-2 1	20	232843	243220	0.9573
8 009 V1872106P3 Primary Comp B-1 1	9	281644	232449	1.2116					
8 010 V1872106P3 Primary Comp B-1 1	10	289058	241748	1.1957					
8 011 V1872106P3 Primary Comp B-1 1	11	283251	228527	1.2395					
8 012 V1872106P3 Primary Comp B-2 1	12	281654	235563	1.1957					
8 013 V1872106P3 Primary Comp B-2 1	13	291038	239932	1.2130					
8 014 V1872106P3 Primary Comp B-2 1	14	291568	236234	1.2342					
			Mean	1.2011				Mean	0.9726
			n	12				n	6
			SD	0.0239				SD	0.0111
			%CV	2.0				%CV	1.1
%Change =	-19.0								
Duration:	28	Days							

$$\%\text{Change} = \frac{\text{mean response of stored solutions} - \text{mean response of comparison solutions}}{\text{mean response of comparison solutions}} \times 100$$

Criteria: To be deemed stable, % Change upon storage for analyte must be within ±15.0% and %CV must be within ≤15.0%.
Meets Criteria: NO

17. FIGURES

Figure 1: Typical Calibration Curve

Analytical Run 4 Run Acceptance Parameters for Ertapenem (µg/mL) (Study V1872106P3)

Regression Type	LINEAR
Weighting Factor	1/X**2
Slope	0.090549877515347500
Intercept	0.000029657823768757
R-Squared	0.998873789353787
LLOQ	0.05
ULOQ	50
Mean Std %Diff	2.4
S.D. Std %Diff	1.6
Mean Std Response	0.090666
S.D. Std Response	0.002735

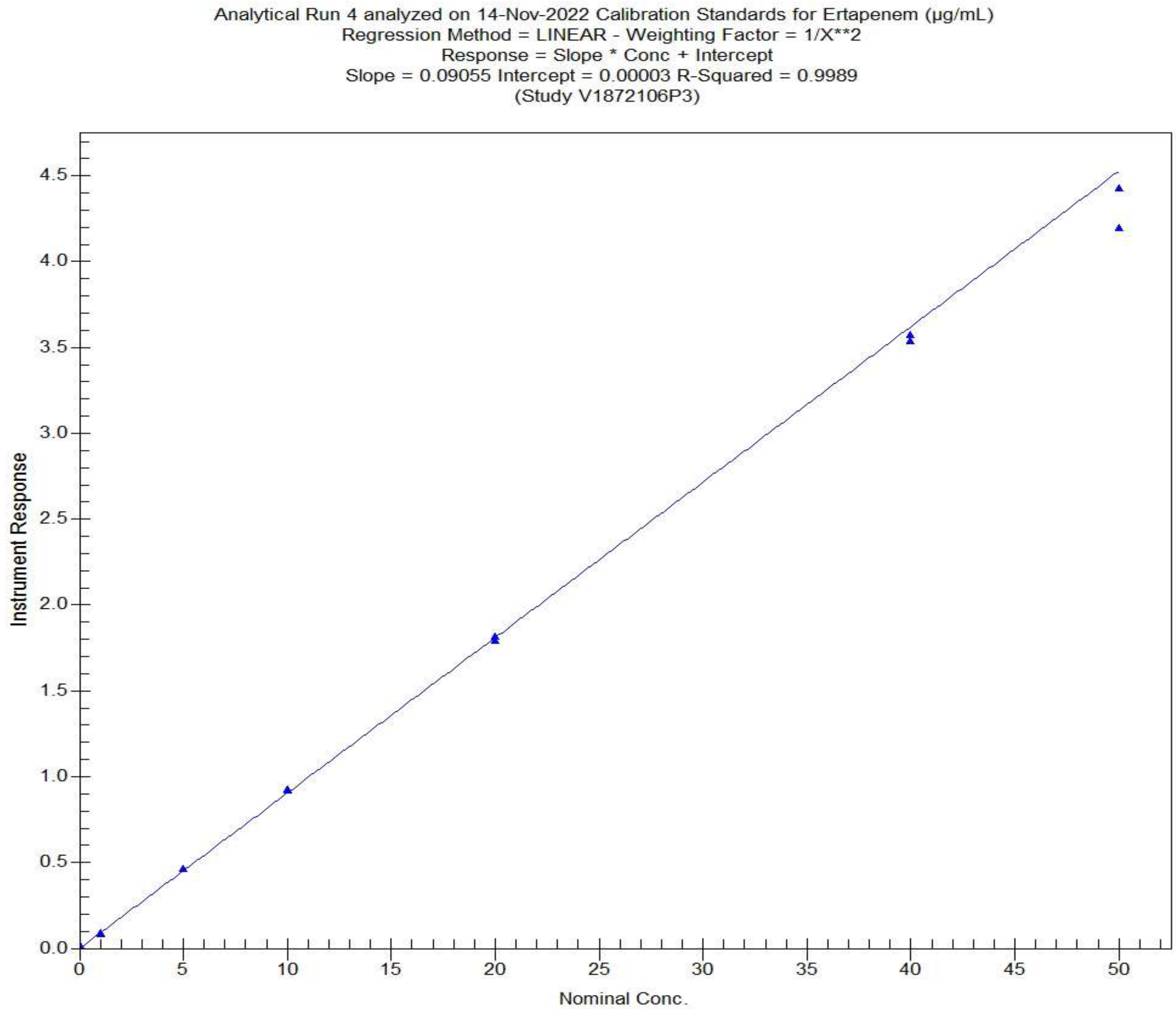

Figure 2: Typical Ion Chromatogram Blank Human Plasma Ultrafiltrate Without Internal Standard

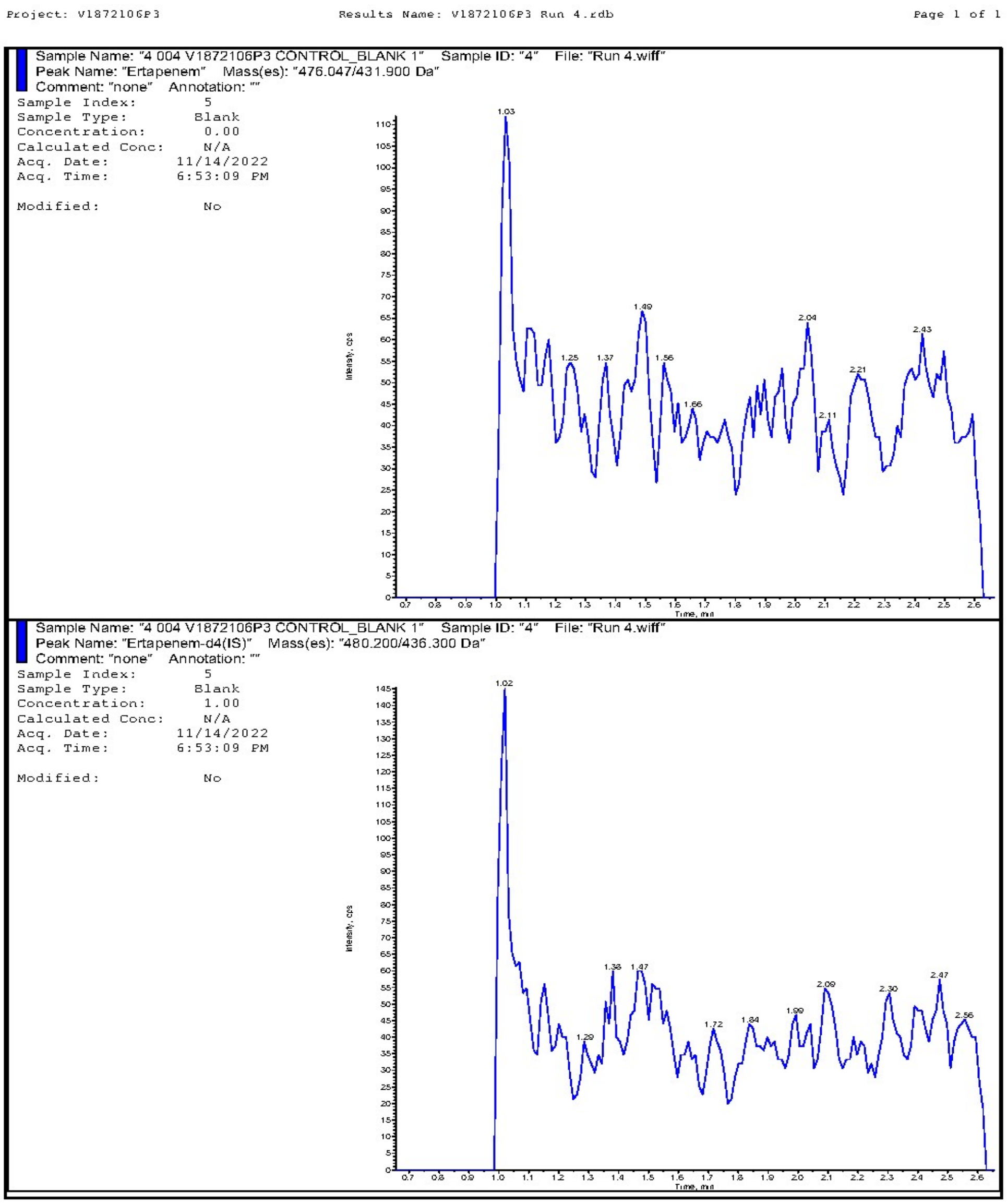

Figure 3: Typical Ion Chromatogram Blank Human Plasma Ultrafiltrate With Internal Standard

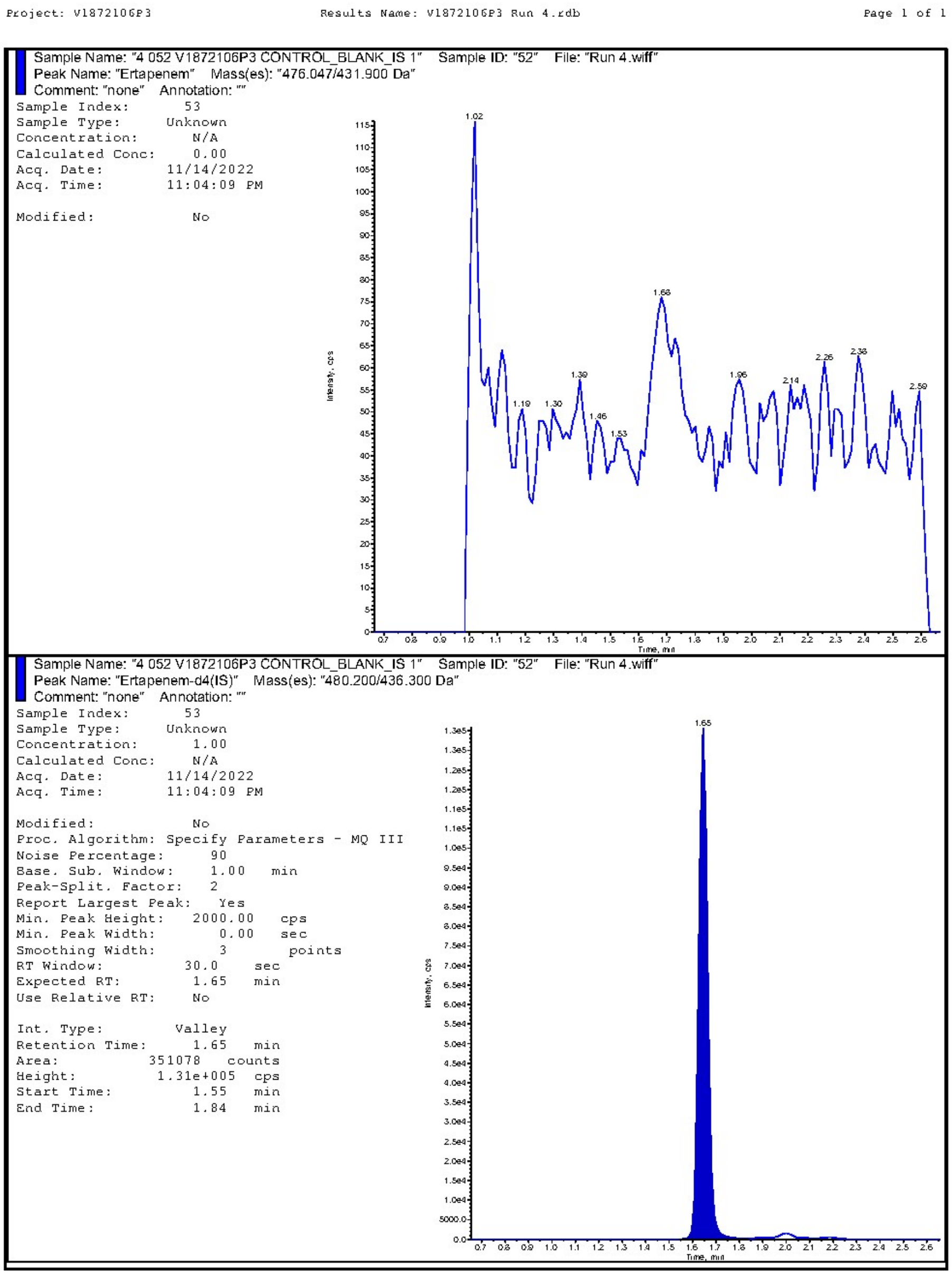

Figure 4: Typical Ion Chromatogram LLOQ Calibration Standard, Ertapenem = 0.0500 mcg/mL

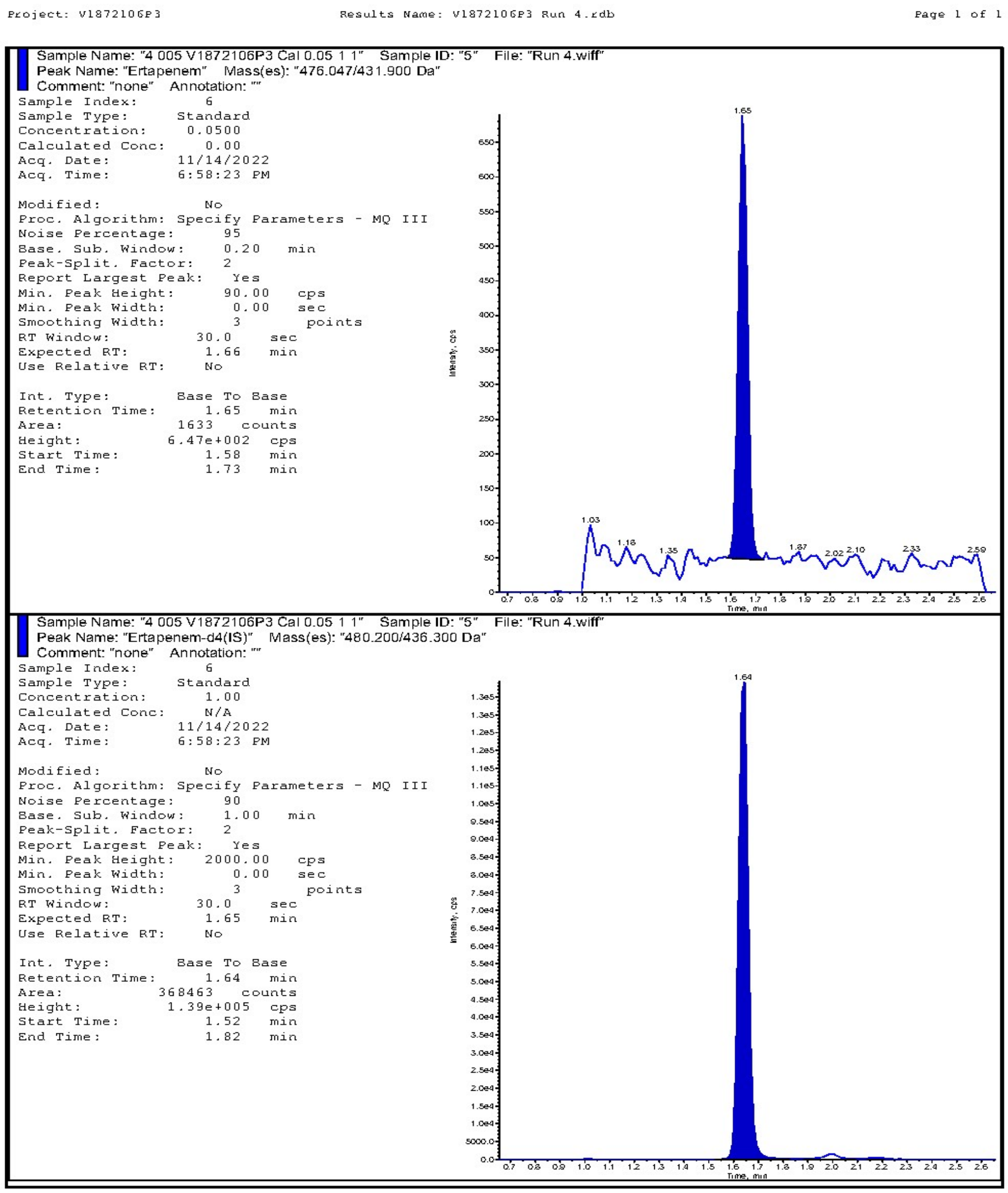

Figure 5: Typical Ion Chromatogram Calibration Standard, Ertapenem = 20.0 mcg/mL

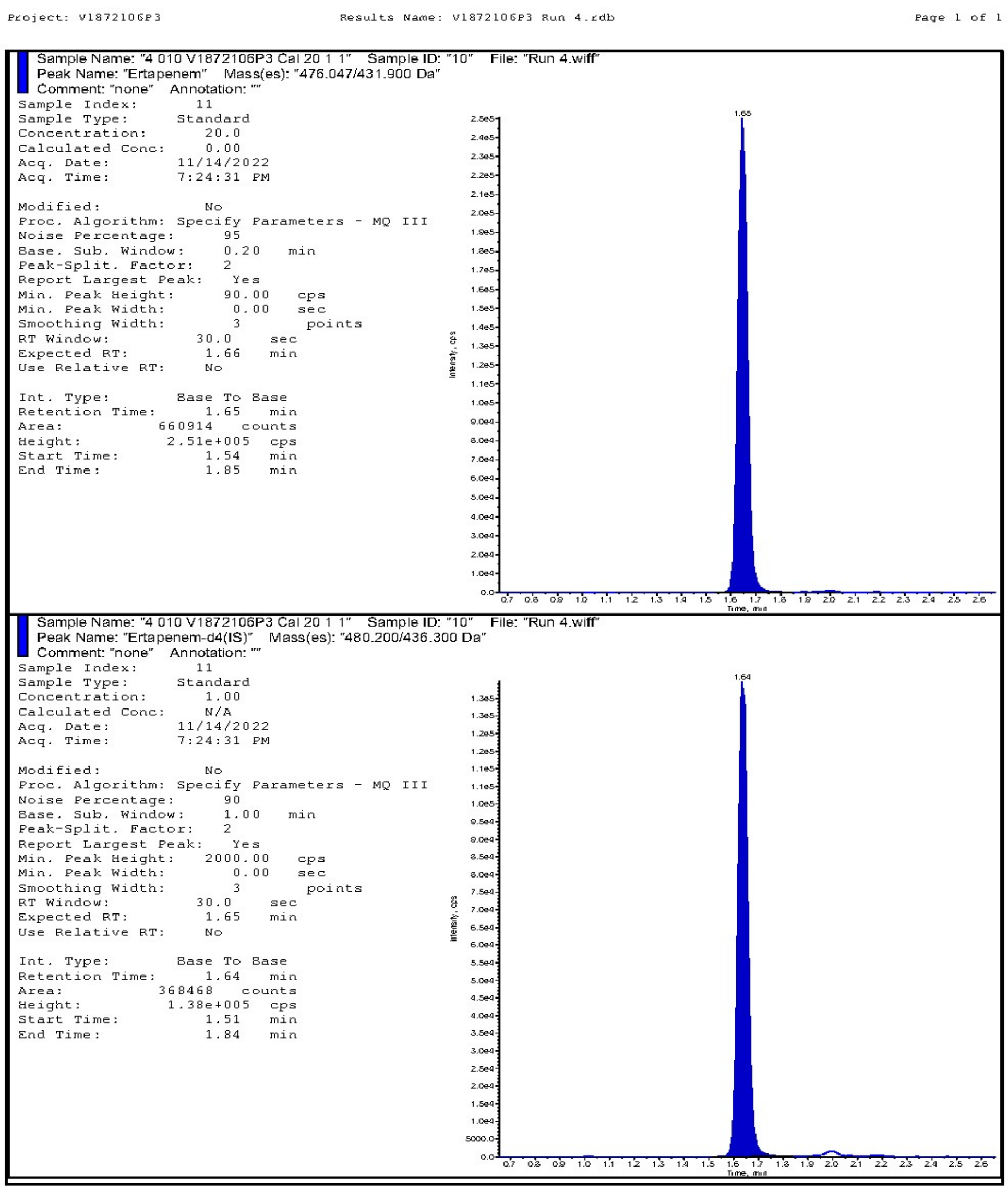

Figure 6: Typical Ion Chromatogram ULOQ Calibration Standard, ertapenem = 50.0 mcg/mL

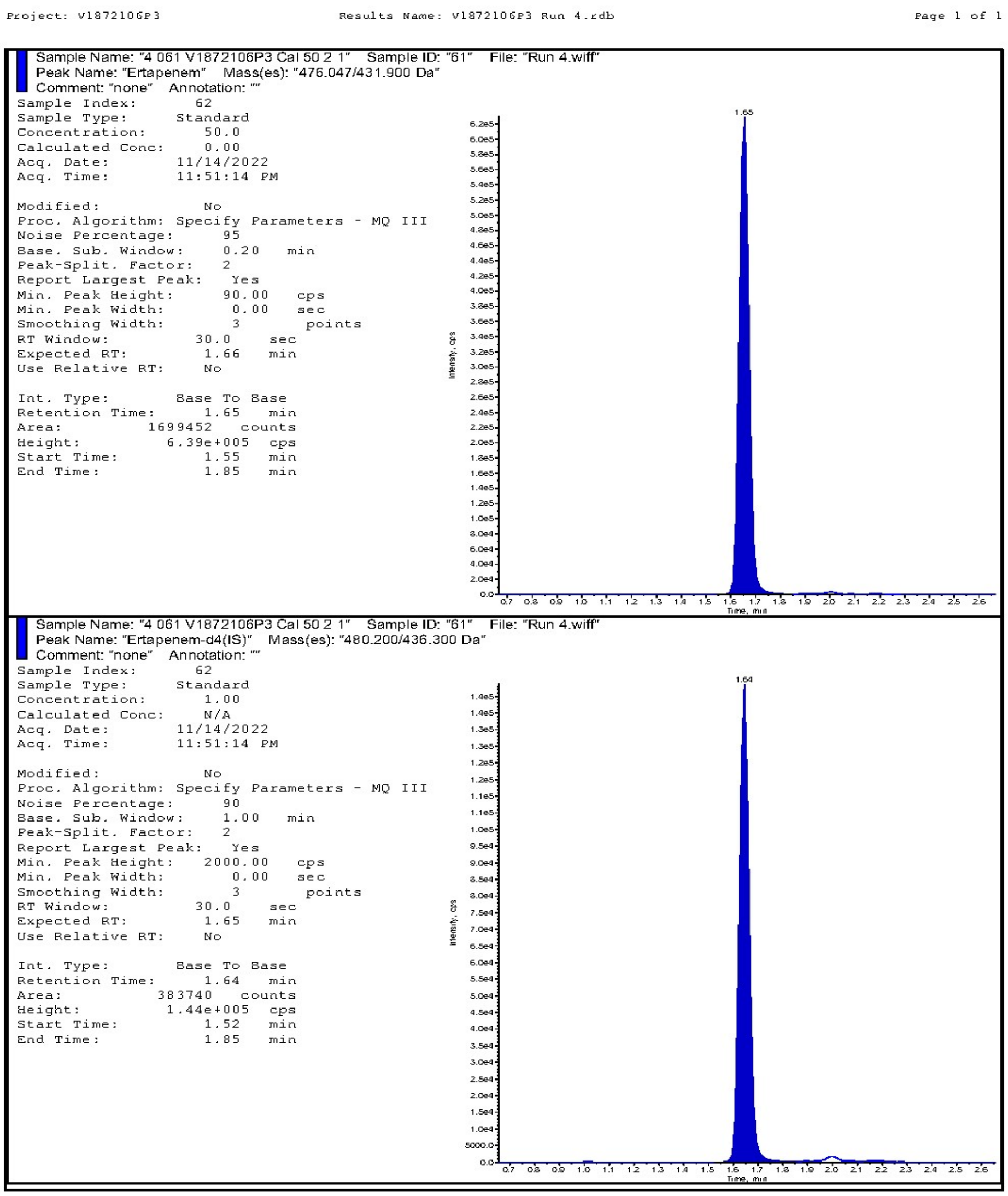

Figure 7: Typical Ion Chromatogram Cross-Talk Evaluation High QC No IS

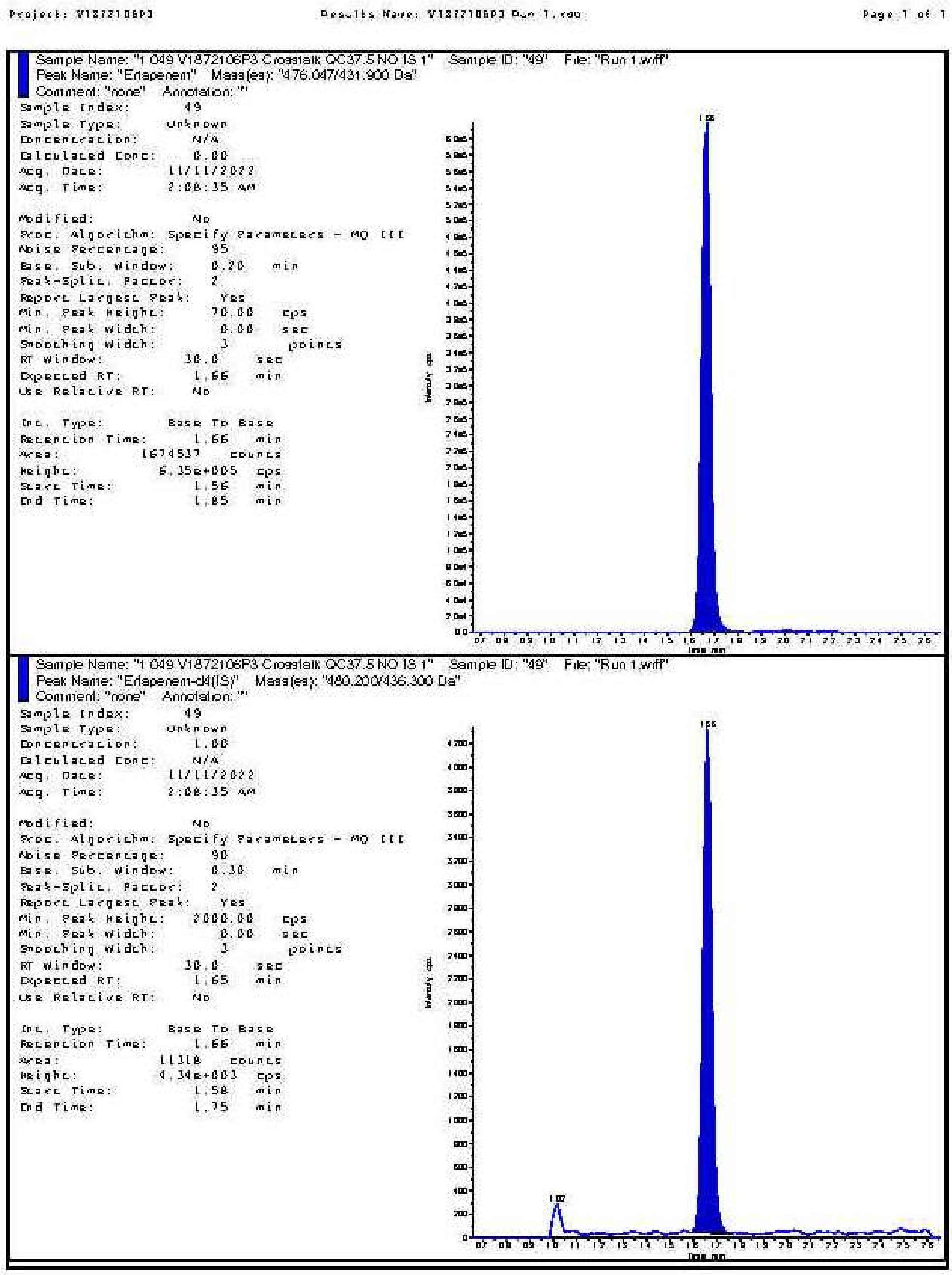

1. GENERAL INFORMATION V1872106P3

1.1. Client Name: Dynport Vaccine Company

1.2. Reference: Validation V1872106P3

2. TABLE OF CONTENTS

1. GENERAL INFORMATION V1872106P3 1
1.1. Client Name: Dynport Vaccine Company 1
1.2. Reference: Validation V1872106P3 1
2. TABLE OF CONTENTS 1
3. SAMPLE REQUIREMENTS 3
4. METHOD SUMMARY 3
5. SPECIAL HANDLING OF BIOLOGICAL SAMPLES 3
6. DATA COLLECTION AND ANALYSIS SOFTWARE 3
7. INSTRUMENTS, EQUIPMENT, AND CONDITIONS 4
7.1. LC and MS Conditions 4
Table 1: HPLC System 4
7.1.1. Gradient Table 5
7.1.2. Autosampler Settings 5
7.1.3. Mass Spectrometer System 5
8. REAGENTS, STANDARDS, AND SUPPLIES 7
Table 2: Reagents, Standards, and Supplies 7
9. SAFETY 7
10. PROCEDURE 8
10.1. Preparation of Analyte Stock Solutions and Dilutions 8

CONFIDENTIAL

Serial Number: 84ada1b0-9e24-4433-aceb-aca0b619f78f accessed by Scott Hammers (HAMMERSS) on Tue, 06 Dec 2022 17:45:55 GMT

10.1.1. Ertapenem Primary Solution, 2.00 mg/mL (Prepare on wet ice) 8
10.1.2. Working Internal Standard, ~5.0 mcg/mL Ertapenem-d_4 (Prepare on wet ice) 8
10.2. Preparation of Other Reagents 8
10.2.1. Mobile Phase A: 0.5% Formic Acid in Deionized Water 8
10.2.2. Mobile Phase B: 90/10 Acetonitrile / Deionized Water 8
10.2.3. Rinse: 90/10 Acetone/Deionized Water 9
10.2.4. 1 N Sodium Hydroxide (NaOH) in Deionized Water 9
10.2.5. MES (2-(N-Morpholino) Ethanesulphonic Acid Hydrate) Buffer pH 6.4, ~0.1M....9
10.2.6. 5/95 Acetonitrile/Deionized Water 9
10.3. Preparation of Standards and QC Samples 9
10.3.1. Ertapenem Secondary Solution, 100 mcg/mL in Treated Human Plasma Ultrafiltrate (Reported as 200 mcg/mL; Prepare on wet ice) 9
10.3.2. Treated Human Plasma Ultrafiltrate (Ultrafiltrate: MES Buffer 1:1) 9
10.3.3. Preparation of Standards and QC Samples in Human Plasma Ultrafiltrate....10
10.3.4. Ultrafiltration of Human Plasma Samples using Centrifugal Filters....10
10.4. Extraction Procedure 11
10.5. Dilutions 11
10.6. Calculations 12
11. REFERENCE CHROMATOGRAMS 12
Figure 1: Blank with IS 12
Figure 2: LLOQ – Ertapenem 0.050 mcg/mL....13
Figure 3: ULOQ – Ertapenem 50.0 mcg/mL 13
12. REVISION HISTORY 14
Table 3: Revision History....14

CONFIDENTIAL

Serial Number: 84ada1b0-9e24-4433-aceb-aca0b619f78f accessed by Scott Hammers (HAMMERSS) on Tue, 06 Dec 2022 17:45:55 GMT

3. SAMPLE REQUIREMENTS

Ultrafiltered human sodium heparin plasma samples (treated with 1:1 MES Buffer, pH 6.4, ~0.1M) minimum volume 100 mcL each, for determination of free ertapenem concentration.

Refer to the relevant study protocol for a description of expected samples.

4. METHOD SUMMARY

The method involves 96-well protein precipitation followed by chromatographic separation and tandem MS detection using the Sciex 5000 LC/MS/MS system. The concentration range is 0.0500 – 50.0 mcg/mL in human plasma ultrafiltrate only. Since unknown samples are treated with the MES buffer after collection at a 1:1 ratio, the calibration standard and QC concentrations are based on the ultrafiltrate volume only. Unknown samples do not need to be adjusted for the dilution with MES buffer.

5. SPECIAL HANDLING OF BIOLOGICAL SAMPLES

The method requires no special handling. Please ensure all "Universal Precautions" are taken.

Primaries, spiking solutions, calibration standards and QCs should be prepared on wet ice, and each prepared level should be aliquoted immediately and aliquots should be flash frozen in a dry-ice/acetone bath for ~1 minute and stored in the freezer immediately after flash freezing.

6. DATA COLLECTION AND ANALYSIS SOFTWARE

Description	Version
Watson LIMS™ (Thermo Fisher Scientific)	7.5 or higher
Analyst® (AB Sciex)	1.7.2 or higher

CONFIDENTIAL

Serial Number: 84ada1b0-9e24-4433-aceb-aca0b619f78f accessed by Scott Hammers (HAMMERSS) on Tue, 06 Dec 2022 17:45:55 GMT

7. INSTRUMENTS, EQUIPMENT, AND CONDITIONS

Equivalent equipment can be substituted, except where noted in Section 7.1. Minor modifications of conditions are permitted to optimize chromatography, except where noted in Section 7.1. Refer to SOP-00073 for allowable minor modifications that do not require validation.

7.1. LC and MS Conditions

Table 1: HPLC System

**System Controller**	**Shimadzu Controller, CBM20A**
Pump	Shimadzu LC-30AD
Autosampler	Shimadzu SIL-30AC
Column Oven	Shimadzu CTO-30A
MS Interface	ESI
Analytical Column	Fortis Technology Ltd. Fortis C18 4.6 x 50mm, 5 µ
Column Guard Cartridge	Security Guard Cartridge AQ C18, 4.0 x 2.0 mm
Column Temperature (set at)	35°C
Mobile Phase A	0.5% Formic acid in deionized water
Mobile Phase B	90/10 Acetonitrile/deionized water
Needle Wash Solvent (R0, R3)	90/10 Acetone/deionized water (or other acceptable solution)
Total Run Time (approximate)	4.5 min
Injection Volume (µl)	0.1-5.0
Flow Rate	1.0 mL/min
Pressure, kgf/cm2 (approximate)	~100
Approximate Retention Time (min.)	Ertapenem: 1.66
	Ertapenem-d4: 1.66

CONFIDENTIAL

Serial Number: 84ada1b0-9e24-4433-aceb-aca0b619f78f accessed by Scott Hammers (HAMMERSS) on Tue, 06 Dec 2022 17:45:55 GMT

7.1.1. Gradient Table

Time Program

Time	Module	Events	Parameter
0.00	Pumps	Pump B Conc.	18
0.50	Pumps	Pump B Conc.	18
1.00	Controller	Event 1	
2.40	Pumps	Pump B Conc.	45
2.50	Pumps	Pump B Conc.	100
2.60	Controller	Event 2	
3.50	Pumps	Pump B Conc.	100
3.60	Pumps	Pump B Conc.	18
4.20	System Controller	Stop	

7.1.2. Autosampler Settings

Temperature: Set at 4°C
Rinse Type: External
Rinse Mode: Before and after aspiration
Rinse Dip Time: 0 sec
Rinse Method: Rinse Pump Then Port
Rinse Time: 2 sec
Rinsing Speed: 35 µL/sec
Rinsing Volume: 500 µL

7.1.3. Mass Spectrometer System

Mass Spectrometer Sciex 5000 or 6500 (Low Mass Mode)
Source Type: Turbo spray
Scan Type: MRM (MRM)
Polarity: Positive
Resolution Q1: Unit
Resolution Q3: Unit

CONFIDENTIAL

Serial Number: 84ada1b0-9e24-4433-aceb-aca0b619f78f accessed by Scott Hammers (HAMMERSS) on Tue, 06 Dec 2022 17:45:55 GMT

All the below parameters in this section may be adjusted to optimize the instrument conditions.

Ertapenem:

Q1 Mass (Da)	Q3 Mass (Da)	Dwell (msec)
476.047	431.9	250.00
DP	56.00	
CE	13.00	
CXP	13.00	

Ertapenem-d$_4$ (IS):

Q1 Mass (Da)	Q3 Mass (Da)	Dwell (msec)
480.2	436.3	250.00
DP	56.00	
CE	13.00	
CXP	13.00	

Dummy Ions (to counter-act potential crosstalk):

Q1 Mass (Da)	Q3 Mass (Da)	Dwell (msec)
250	200	100
254	204	100

Parameter Table:

CAD: 12.00
CUR: 40.00
GS1: 50.00
GS2: 60.00
IS: 5500
TEM: 600
EP 12

CONFIDENTIAL

Serial Number: 84ada1b0-9e24-4433-aceb-aca0b619f78f accessed by Scott Hammers (HAMMERSS) on Tue, 06 Dec 2022 17:45:55 GMT

8. REAGENTS, STANDARDS, AND SUPPLIES

Reagents from alternate supplier and equivalent grade can be substituted.

Table 2: Reagents, Standards, and Supplies

Chemical / Grade	Manufacturer/Supplier
Ertapenem	Performa
Ertapenem-d_4	Sigma Aldrich
MES ([2-(N-Morpholino) ethane sulphonic acid hydrate]) buffer	Sigma
Acetonitrile, HPLC grade	Honeywell
Formic Acid, 98+%	EMD Millipore
Deionized Water	Millipore
Ammonium Acetate ≥ 99.9%	Sigma
Methanol	Honeywell
Acetone	Honeywell
Ultrafiltered Human Sodium Heparin Plasma	BioIVT
Centrifugal Filters	Merck Millipore, Lot: 0000158890
Advantage SiliGuard Coated 2 mL 96 well Polypropylene plate: Square Top, Round Bottom	Analytical Sales and Services, Catalog # 27P687GC
Purple, pre-slit, square well, soft silicone/PTFE Cap Mats	Analytical Sales & Services, Inc., Catalog# 965085

9. SAFETY

Always observe safe laboratory practices. Consult the appropriate SDS before handling any chemical if you are unfamiliar with the proper safety precautions.

CONFIDENTIAL

Serial Number: 84ada1b0-9e24-4433-aceb-aca0b619f78f accessed by Scott Hammers (HAMMERSS) on Tue, 06 Dec 2022 17:45:55 GMT

10. PROCEDURE

10.1. Preparation of Analyte Stock Solutions and Dilutions

The quantities of analyte solution prepared may be scaled up or down proportionally as needed. Alternate dilution schemes can be used to achieve the appropriate final concentrations of standards, QCs, working internal standard, and system verification solution (SVS). For assignment of expiration dates to analyte stocks and their dilutions, refer to the latest version of the validation report.

10.1.1. Ertapenem Primary Solution, 2.00 mg/mL (Prepare on wet ice)

Check drying conditions prior to use. Weigh approximately 20.0 mg of ertapenem reference standard (correcting for purity and correction factor if necessary) into a polypropylene container and add 10.0 mL of MES buffer, pH 6.4, ~0.1M. Mix well and sonicate if necessary. Store solution at -20°C in a polypropylene container.

10.1.2. Working Internal Standard, ~5.0 mcg/mL Ertapenem-d_4 (Prepare on wet ice)

Check drying conditions prior to use. Weigh 1.00 mg of ertapenem-d_4 reference standard into a polypropylene container. Add 200 mL of MES buffer, pH 6.4, ~0.1M (do not correct for conversion factor and purity). Mix well and sonicate if necessary. **Aliquot** and store at or below -20 °C in polypropylene containers.

10.2. Preparation of Other Reagents

Prepared quantities of all reagents may be scaled up or down as necessary. Volumes provided are examples. For assignment of expiration dates, refer to SOP-00094.

10.2.1. Mobile Phase A: 0.5% Formic Acid in Deionized Water

Add 20 mL of formic acid to a 4-L volumetric flask containing ~3800 mL of deionized water. Dilute to volume with deionized water. Mix well and store at room temperature.

10.2.2. Mobile Phase B: 90/10 Acetonitrile / Deionized Water

Combine 3600 mL of acetonitrile with 400 mL of deionized water in a 4-L flask. Mix well and store at room temperature.

CONFIDENTIAL

Serial Number: 84ada1b0-9e24-4433-aceb-aca0b619f78f accessed by Scott Hammers (HAMMERSS) on Tue, 06 Dec 2022 17:45:55 GMT

10.2.3. Rinse: 90/10 Acetone/Deionized Water

Combine 3600 mL of acetone and 400 mL of deionized water in a 1-L flask. Mix well and store at room temperature.

10.2.4. 1 N Sodium Hydroxide (NaOH) in Deionized Water

Weight ~ 4.00 g of sodium hydroxide (F.W. 40.00) and gradually transfer it to a 100-mL volumetric flask containing ~50 mL of deionized water. Mix to dissolve and wait until it reaches room temperature. Dilute to volume with deionized water and mix well. Store in a polypropylene container at room temperature.

10.2.5. MES (2-(N-Morpholino) Ethanesulphonic Acid Hydrate) Buffer pH 6.4, ~0.1M

Weigh ~ 4000 mg of MES hydrate (F.W. 195.24) and transfer to a 200-mL volumetric flask containing ~150 mL of deionized water. Dissolve and adjust the pH to 6.4 with 1N NaOH. Dilute to volume with deionized water. Mix well and store refrigerated.

10.2.6. 5/95 Acetonitrile/Deionized Water

Combine 50 mL of acetonitrile and 950 mL of deionized water in a 1-L flask. Mix well and store at room temperature.

10.3. Preparation of Standards and QC Samples

10.3.1. Ertapenem Secondary Solution, 100 mcg/mL in Treated Human Plasma Ultrafiltrate (Reported as 200 mcg/mL; Prepare on wet ice)

Transfer 1.0 mL of ertapenem primary solution (2.00 mg/mL) to a polypropylene tube containing 19.0 mL of **chilled** treated sodium heparin human plasma ultrafiltrate on wet ice. Mix well, aliquot, and store at -70 °C or -20 °C.

10.3.2. Treated Human Plasma Ultrafiltrate (Ultrafiltrate: MES Buffer 1:1)

Add 200 mL of **chilled** sodium heparin human plasma ultrafiltrate to a polypropylene container containing 200 mL of **chilled** 0.1M MES Buffer, pH 6.4. Mix well and store at -20°C.

It is recommended to prepare the treated human plasma ultrafiltrate fresh prior to usage.

CONFIDENTIAL

Serial Number: 84ada1b0-9e24-4433-aceb-aca0b619f78f accessed by Scott Hammers (HAMMERSS) on Tue, 06 Dec 2022 17:45:55 GMT

10.3.3. Preparation of Standards and QC Samples in Human Plasma Ultrafiltrate

Use treated sodium heparin human plasma ultrafiltrate that has been diluted 1:1 with MES buffer, screened for the lack of interference at the retention times of ertapenem and ertapenem-d_4 (IS). Starting from the ertapenem secondary in treated human plasma ultrafiltrate (200 mcg/mL), prepare a series of calibration standards and QCs with treated sodium heparin human plasma ultrafiltrate on wet ice. Since unknown samples are treated with the MES buffer after collection at a 1:1 ratio, the calibration standard and QC concentrations are based on the ultrafiltrate volume only. Unknown samples do not need to be adjusted for the dilution with MES buffer. The prepared concentrations in plasma ultrafiltrate only, not accounting for MES buffer, will be as follows: For standards: 50.0, 40.0, 20.0, 10.0, 5.00, 1.00, 0.100, and 0.0500 mcg/mL. For QCs: 37.5, 25.0, and 0.150 mcg/mL, with 0.0500 mcg/mL for the LLOQ QCs when needed. Other concentrations may be prepared, provided they meet the requirements of SOP-00035 and provided they remain within the range of 0.0500 to 50.0 mcg/mL. Store solutions in polypropylene at -70°C or -20°C.

10.3.4. Ultrafiltration of Human Plasma Samples using Centrifugal Filters

1. Thaw human plasma sample on wet ice. Once sample is thawed, vortex each sample for ~ 20 seconds.
2. Add ~1 mL of sample into a centrifugal filter. Hold the tube at ~45° with pipette tip touching the tube wall and add sample slowly with even flow to avoid air locking.
3. Cap the filter. The caps should extend no more than 3-4 mm the top. Further compression can cause prefiltration, which could introduce analytical error.
4. Centrifuge sample at 2000 x g for 30 minutes, this will yield ~150 mcL of filtrate.
5. Use the resulting plasma filtrate samples for the extraction procedure in Section 10.4.

CONFIDENTIAL

Serial Number: 84ada1b0-9e24-4433-aceb-aca0b619f78f accessed by Scott Hammers (HAMMERSS) on Tue, 06 Dec 2022 17:45:55 GMT

10.4. Extraction Procedure

1. Thaw treated human plasma ultrafiltrate samples on wet ice. Once samples are thawed, vortex each sample for ~10 seconds immediately prior to pipetting.
2. Pipet 100 mcL of human plasma ultrafiltrate sample or blank to the corresponding wells of the 1-mL 96-well plate on wet ice.
3. Add 50 mcL of **COLD** working IS (5.0 mcg/mL ertapenem-d_4 in MES buffer, pH 6.4, ~0.1M) to all applicable wells. For **Control Blanks** add 50 mcL of MES buffer, pH 6.4, ~0.1M instead.
4. Seal the plate with an adhesive pad (or equivalent) and vortex at 1300 rpm for 5 minutes using a plate shaker.
5. Add 200 mcL of acetonitrile to all wells. Seal the plate with an adhesive pad (or equivalent) and vortex for 10 minutes at 1200 rpm.
6. Centrifuge the plate for 10 minutes at a setting of 4000 rpm (ambient conditions).
7. Transfer 200 mcL of the supernatant to a new 1-mL 96-well plate using a multichannel pipette (or equivalent).
8. Evaporate the sample using a 96-well SPE dryer at 37 °C at 60-80 psi. This generally takes around 70 minutes.
9. Reconstitute the plate with 130 mcL of 5/95 acetonitrile/deionized water in each well. Seal the plate with a cap mat.
10. Vortex the final plate at 1300 rpm for 5 minutes using a plate shaker.
11. Centrifuge the plate for 2 minutes at a setting of 3300 rpm (ambient conditions).
12. Refrigerate prior to LC-MS injection according to the conditions outlined in Section 7.

10.5. Dilutions

2x = 50 mcL of sample + 50 mcL of blank treated human plasma ultrafiltrate

5x = 20 mcL of sample + 80 mcL of blank treated human plasma ultrafiltrate

CONFIDENTIAL

Serial Number: 84ada1b0-9e24-4433-aceb-aca0b619f78f accessed by Scott Hammers (HAMMERSS) on Tue, 06 Dec 2022 17:45:55 GMT

10.6. Calculations

Linear regression is performed with a $1/x^2$ weighting factor.

Equation: Response = Slope * Conc + Intercept

11. REFERENCE CHROMATOGRAMS

Figure 1: Blank with IS

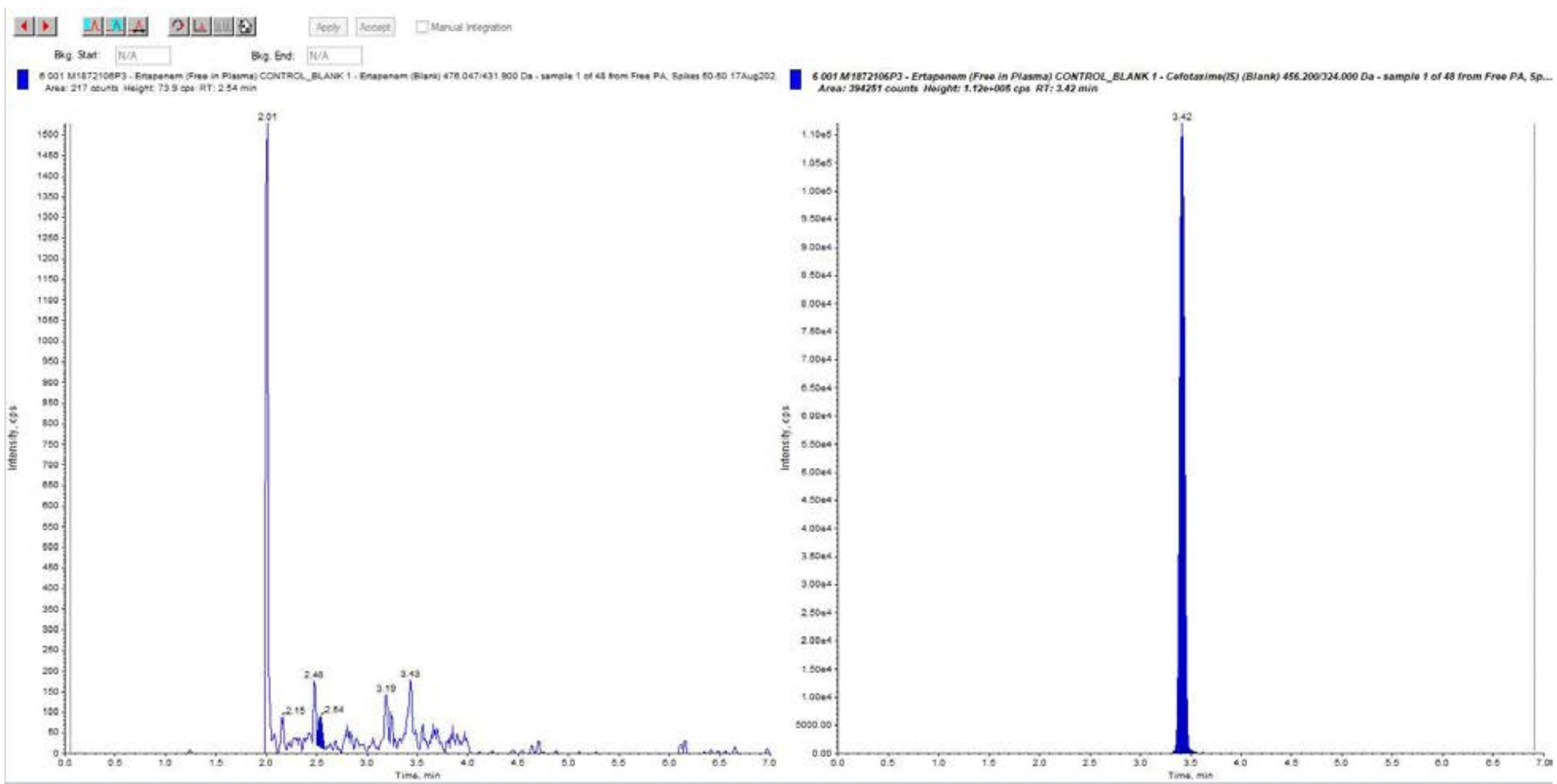

CONFIDENTIAL

Serial Number: 84ada1b0-9e24-4433-aceb-aca0b619f78f accessed by Scott Hammers (HAMMERSS) on Tue, 06 Dec 2022 17:45:55 GMT

Figure 2: LLOQ – Ertapenem 0.050 mcg/mL

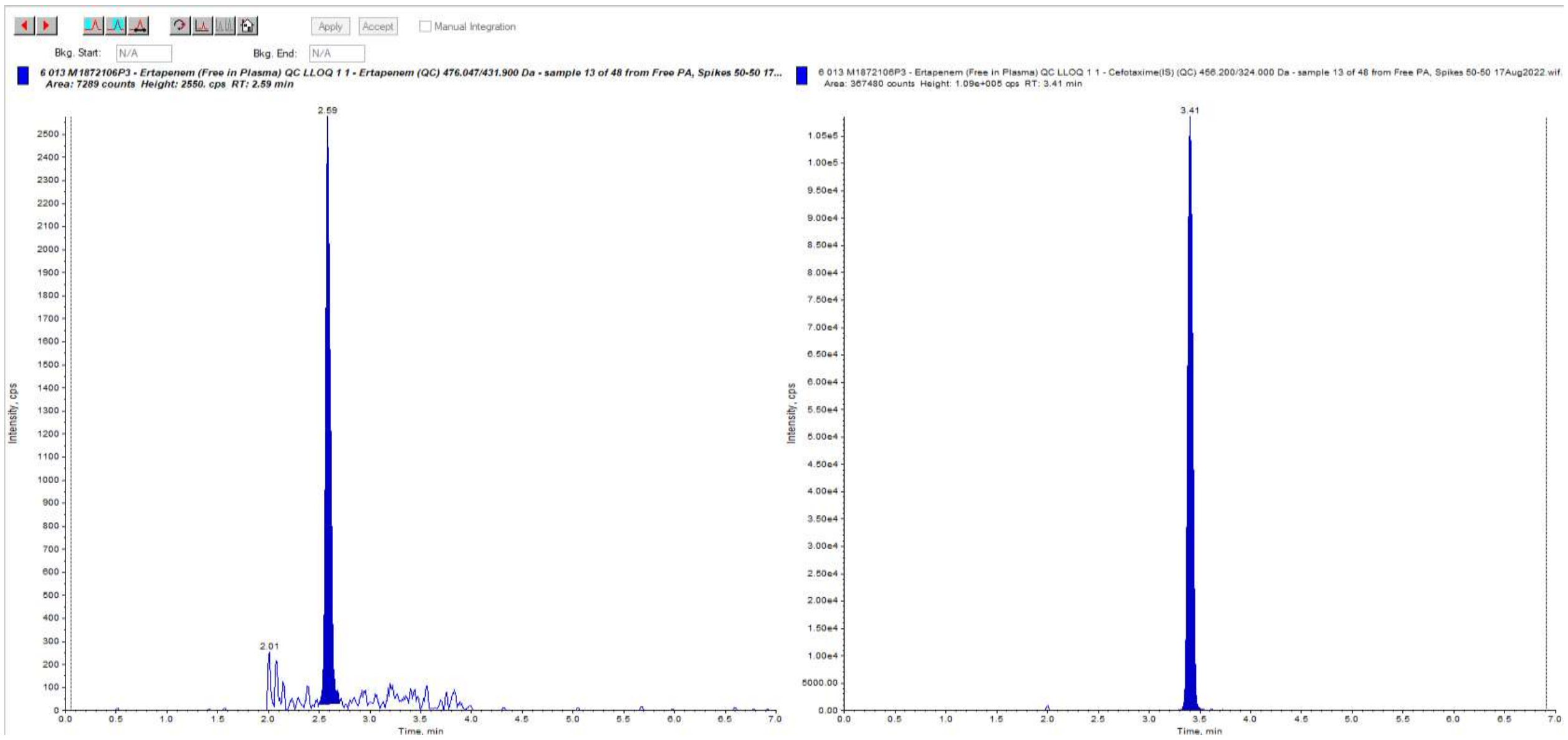

Figure 3: ULOQ – Ertapenem 50.0 mcg/mL

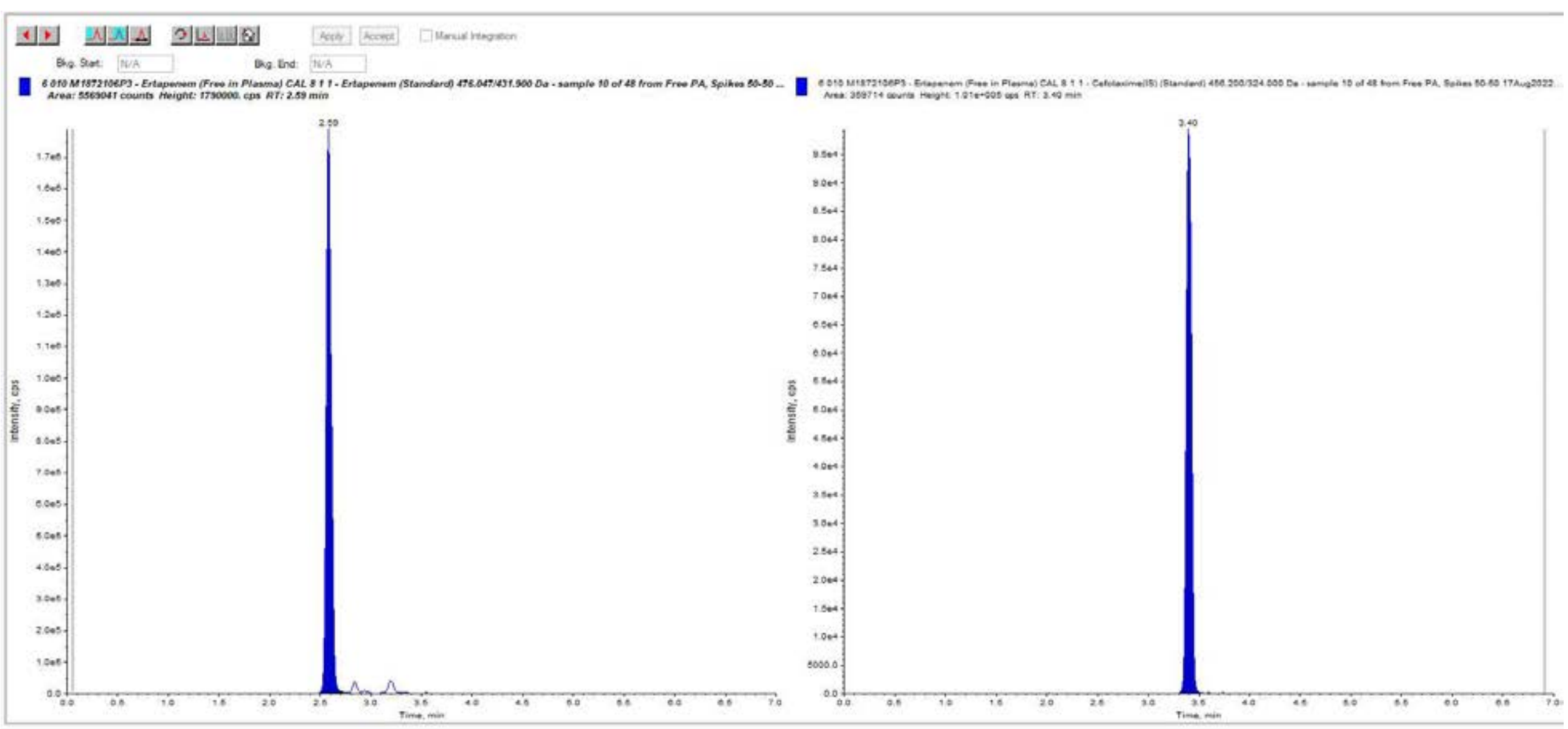

CONFIDENTIAL

Serial Number: 84ada1b0-9e24-4433-aceb-aca0b619f78f accessed by Scott Hammers (HAMMERSS) on Tue, 06 Dec 2022 17:45:55 GMT

12. REVISION HISTORY

Table 3: Revision History

Revision	Effective Date	Section	Revision Summary
00	See Header	NA	New Procedure

CONFIDENTIAL

Serial Number: 84ada1b0-9e24-4433-aceb-aca0b619f78f accessed by Scott Hammers (HAMMERSS) on Tue, 06 Dec 2022 17:45:55 GMT

TP-80238 [00]: V1872106P3 - Ertapenem in Treated Human Plasma Ultrafiltrate (Sodium Heparin, 100 mcL, 0.0500 – 50.0 mcg/mL)

All blank fields are considered as data not available.

All Signature (Date and Time) shown in this report are in GMT.

**Type**	TP	**Status**	CURRENT	
**Effective Date**	28-Nov-2022	**Periodic Review Date**	14-Apr-2050	
**Owner**	PIPPINA	**Department Name**	Pharma LC-MS/MS	Pharma LC-MS/MS
**Description**	Full Title: LC-MS/MS Analysis of Free Ertapenem in Treated Human Plasma Ultrafiltrate (Sodium Heparin, 100 mcL, 0.0500 – 50.0 mcg/mL; Prior to MES Treatment)			

Revision Notes (5)

Task Name	Action	Comment	Signature
Check In	Document Profile Preparation	Created outside of the system.	NEVINSC<br>23-Nov-2022 23:25:38 [GMT]
Check Out	Document Finalization	Correct TOC.	NEVINSC<br>23-Nov-2022 23:32:52 [GMT]
Check In	Document Finalization	Corrected TOC.	NEVINSC<br>23-Nov-2022 23:33:23 [GMT]
Check Out	Document Finalization	Correct TOC.	NEVINSC<br>23-Nov-2022 23:36:26 [GMT]
Check In	Document Finalization	Corrected TOC.	NEVINSC<br>23-Nov-2022 23:36:57 [GMT]

Serial Number: 84ada1b0-9e24-4433-aceb-aca0b619f78f accessed by Scott Hammers (HAMMERSS) on Tue, 06 Dec 2022 17:45:55 GMT.

Approval Information

Note to Approver

Reviewer: Bryan Parmentier, MLT (ASCP)
Management: Marsha Luna, B.S.
Author & PI: Anika Pippin, B.S.

Approval Decision	Comment	Signature
Approve		LUNAM<br>28-Nov-2022 1:24:11 [GMT]
Approve		PIPPINA<br>28-Nov-2022 16:28:31 [GMT]
Approve		PARMENTIERB<br>23-Nov-2022 23:51:13 [GMT]

Serial Number: 84ada1b0-9e24-4433-aceb-aca0b619f78f accessed by Scott Hammers (HAMMERSS) on Tue, 06 Dec 2022 17:45:55 GMT.

Certificate of Analysis

Inhibitors • Screening Libraries • Proteins

Ertapenem sodium

Cat. No.:	HY-13625
CAS No.:	153773-82-1
Batch No.:	180017
Chemical Name:	1-Azabicyclo[3.2.0]hept-2-ene-2-carboxylic acid, 3-[[(3S,5S)-5-[[(3-carboxyphenyl)amino]carbonyl]-3-pyrrolidinyl]thio]-6-[(1R)-1-hydroxyethyl]-4-methyl-7-oxo-, sodium salt (1:1), (4R,5S,6S)-

PHYSICAL AND CHEMICAL PROPERTIES

Molecular Formula:	$C_{22}H_{24}N_3NaO_7S$
Molecular Weight:	**497.50**
Storage:	4°C, sealed storage, away from moisture<br>* In solvent : -80°C, 6 months; -20°C, 1 month (sealed storage, away from moisture)

Chemical Structure:

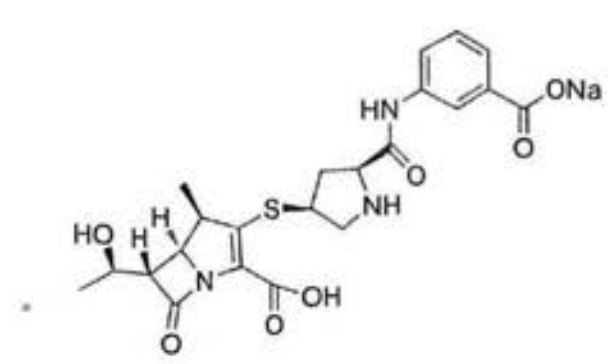

ANALYTICAL DATA

Appearance:	White to light yellow (Solid)
^{1}H NMR Spectrum:	Consistent with structure
LCMS:	Consistent with structure
Purity (LCMS):	**98.86%**
Test date:	2022/08/12
Retest date:	2024/08/11
Conclusion:	The product has been tested and complies with the given specifications.

Name: Lingli Ma
Designation: QA Manager
Date: 6/27/2022

Caution: Product has not been fully validated for medical applications. For research use only.

Tel: 609-228-6898 Fax: 609-228-5909 E-mail: tech@MedChemExpress.com
Address: 1 Deer Park Dr, Suite Q, Monmouth Junction, NJ 08852, USA

Ertapenem sodium

Cat. No.:	HY-13625
CAS No.:	153773-82-1
Molecular Formula:	$C_{22}H_{24}N_3NaO_7S$
Molecular Weight:	497.5
Target:	Bacterial; Antibiotic
Pathway:	Anti-infection
Storage:	4°C, sealed storage, away from moisture<br>* In solvent : -80°C, 6 months; -20°C, 1 month (sealed storage, away from moisture)

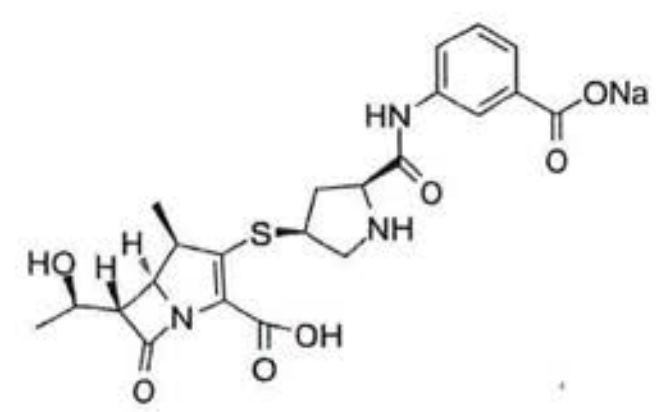

SOLVENT & SOLUBILITY

In Vitro

H_2O : 50 mg/mL (100.50 mM; Need ultrasonic)

	Solvent Mass / Concentration	1 mg	5 mg	10 mg
Preparing Stock Solutions	1 mM	2.0101 mL	10.0503 mL	20.1005 mL
	5 mM	0.4020 mL	2.0101 mL	4.0201 mL
	10 mM	0.2010 mL	1.0050 mL	2.0101 mL

Please refer to the solubility information to select the appropriate solvent.

BIOLOGICAL ACTIVITY

Description

Ertapenem sodium (L-749345) is a broad spectrum and long acting β-lactam antibiotic. Ertapenem sodium has a broad-spectrum anti-anaerobic activity against a variety of anaerobes with a mode MIC of 0.12 μg/mL. Ertapenem sodium can be used for the research of severe infections caused by bacteria in the skin, lungs, stomach, pelvis, and urinary tract[1][2].

In Vitro

Ertapenem sodium (0-100 μg/mL approximately, 48 h) is active against 99.1% of all anaerobes with a mode MIC of 0.12 μg/mL and MIC_{90} of 1 μg/mL, and MIC's ≥8 μg/mL for B.fragilis and B.vulgatus species, respectively[1].

MCE has not independently confirmed the accuracy of these methods. They are for reference only.

Cell Viability Assay[1]

Cell Line:	B. fragilis (ATCC 25285), B. thetaiotaomicron (ATCC 29741), and Eubacterium lentum (ATCC 43055)
Concentration:	0-100 μg/mL approximately
Incubation Time:	48 h
Result:	Inhibited 99.1% of all isolate with a mode MIC of 0.12 μg/mL and MIC_{90} of 1 μg/mL, and

98.8% of the isolates were susceptible among the B. fragilis group.

In Vivo

Ertapenem sodium (Subcutaneous injection, 0-10 mg/kg, 0-120 h after infection, S. aureus thigh tissue infection model) shows > 3 log_{10} CFU reduction of organism at 10 mg/kg, and maintains the activity with 3.3 and 4.4 log_{10} CFU eliminated at 2 mg/kg[2].

Ertapenem sodium (Subcutaneous injection, 4h after infection, systemic infection model) is active against all gram-positive organisms, and is also active against gram-negative organisms tested with ED_{50}s of <0.25 mg/kg/dose[2].

MCE has not independently confirmed the accuracy of these methods. They are for reference only.

Animal Model:	S. aureus thigh tissue infection model (DBA/2 mice)[2]
Dosage:	0.5,1, 2, 5, 10 mg/kg (given at 2, 6, 10, 24, 48, 72, 96, 120 h)
Administration:	Subcutaneous injection (0.5 mL after infection)
Result:	Displayed > 3 log_{10} CFU reduction of organism compared to non-antibiotic-treated controls at 10 mg/kg.<br>Maintained the activity with 3.3 and 4.4 log_{10} CFU eliminated at 2 mg/kg.

Animal Model:	Systemic infection model (DBA/2 female mice, viral antibody-free CD-1 female mice)[2]
Dosage:	0-3 mg/kg approximately
Administration:	Subcutaneous injection (0.5 mL, begin immediately and 4 h after infection)
Result:	Showed activity against all gram-positive organisms, and also ram-negative organisms tested with ED_{50}s of <0.25 mg/kg/dose.

Animal Model:	CD-1 mice, rats[2]
Dosage:	10 mg/kg approximately
Administration:	Intraperitoneal injection (pharmacokinetic assay)
Result:	Exhibited an $AUC_{0-\infty}$ ranging from 1.8-21.82 µg•hr/mL in tissue in mice following a 10-mg/kg i.p. dose.<br>Exhibited slow clearance rate with a $t_{1/2\beta}$ of 3.2 h, Clp of 0.47 mL/min/kg, AUC_{0-8} of 284.15 µg•hr/mL.

CUSTOMER VALIDATION

- Nat Commun. 2022 Mar 2;13(1):1116.
- Nat Commun. 2021 Jul 22;12(1):4461.
- J Antimicrob Chemother. 2020 Jul 1;75(7):1850-1858.
- Biomed Res Int. 2018 Jul 2;2018:3579832.

See more customer validations on www.MedChemExpress.com

REFERENCES

[1]. Kenneth E Aldridge. Ertapenem (MK-0826), a new carbapenem: comparative in vitro activity against clinically significant anaerobes. Diagn Microbiol Infect Dis. 2002 Oct;44(2):181-6.

[2]. C J Gill, et al. In vivo activity and pharmacokinetic evaluation of a novel long-acting carbapenem antibiotic, MK-826 (L-749,345). Antimicrob Agents Chemother. 1998 Aug;42(8):1996-2001.

Caution: Product has not been fully validated for medical applications. For research use only.

Tel: 609-228-6898 Fax: 609-228-5909 E-mail: tech@MedChemExpress.com

Address: 1 Deer Park Dr, Suite Q, Monmouth Junction, NJ 08852, USA

MCE® MedChemExpress

Safety Data Sheet

Revision Date: Aug.-15-2022
Print Date: Aug.-18-2022

1. PRODUCT AND COMPANY IDENTIFICATION

1.1 Product identifier

Product name :	Ertapenem sodium
Catalog No. :	HY-13625
CAS No. :	153773-82-1

1.2 Relevant identified uses of the substance or mixture and uses advised against

Identified uses :	Laboratory chemicals, manufacture of substances.

1.3 Details of the supplier of the safety data sheet

Company:	MedChemExpress USA
Tel:	609-228-6898
Fax:	609-228-5909
E-mail:	sales@medchemexpress.com

1.4 Emergency telephone number

Emergency Phone #:	609-228-6898

2. HAZARDS IDENTIFICATION

2.1 Classification of the substance or mixture

Not a hazardous substance or mixture.

2.2 GHS Label elements, including precautionary statements

Not a hazardous substance or mixture.

2.3 Other hazards

None.

3. COMPOSITION/INFORMATION ON INGREDIENTS

3.1 Substances

Synonyms:	L-749345; MK-826
Formula:	$C_{22}H_{24}N_3NaO_7S$
Molecular Weight:	497.50
CAS No. :	153773-82-1

4. FIRST AID MEASURES

4.1 Description of first aid measures

Eye contact

Remove any contact lenses, locate eye-wash station, and flush eyes immediately with large amounts of water. Separate eyelids with fingers to ensure adequate flushing. Promptly call a physician.

Skin contact

Rinse skin thoroughly with large amounts of water. Remove contaminated clothing and shoes and call a physician.

Inhalation

Immediately relocate self or casualty to fresh air. If breathing is difficult, give cardiopulmonary resuscitation (CPR). Avoid mouth-to-mouth resuscitation.

Ingestion

Wash out mouth with water; Do NOT induce vomiting; call a physician.

4.2 Most important symptoms and effects, both acute and delayed

The most important known symptoms and effects are described in the labelling (see section 2.2).

4.3 Indication of any immediate medical attention and special treatment needed

Treat symptomatically.

5. FIRE FIGHTING MEASURES

5.1 Extinguishing media

Suitable extinguishing media

Use water spray, dry chemical, foam, and carbon dioxide fire extinguisher.

5.2 Special hazards arising from the substance or mixture

During combustion, may emit irritant fumes.

5.3 Advice for firefighters

Wear self-contained breathing apparatus and protective clothing.

6. ACCIDENTAL RELEASE MEASURES

6.1 Personal precautions, protective equipment and emergency procedures

Use full personal protective equipment. Avoid breathing vapors, mist, dust or gas. Ensure adequate ventilation. Evacuate personnel to safe areas.

Refer to protective measures listed in sections 8.

6.2 Environmental precautions

Try to prevent further leakage or spillage. Keep the product away from drains or water courses.

6.3 Methods and materials for containment and cleaning up

Absorb solutions with finely-powdered liquid-binding material (diatomite, universal binders); Decontaminate surfaces and equipment by scrubbing with alcohol; Dispose of contaminated material according to Section 13.

7. HANDLING AND STORAGE

7.1 Precautions for safe handling

Avoid inhalation, contact with eyes and skin. Avoid dust and aerosol formation. Use only in areas with appropriate exhaust ventilation.

7.2 Conditions for safe storage, including any incompatibilities

Keep container tightly sealed in cool, well-ventilated area. Keep away from direct sunlight and sources of ignition.

Recommended storage temperature: 4°C, sealed storage, away from moisture

* In solvent : -80°C, 6 months; -20°C, 1 month (sealed storage, away from moisture)

Shipping at room temperature if less than 2 weeks.

7.3 Specific end use(s)

No data available.

8. EXPOSURE CONTROLS/PERSONAL PROTECTION

8.1 Control parameters

Components with workplace control parameters

This product contains no substances with occupational exposure limit values.

8.2 Exposure controls

Engineering controls

Ensure adequate ventilation. Provide accessible safety shower and eye wash station.

Personal protective equipment

**Eye protection**	Safety goggles with side-shields.
**Hand protection**	Protective gloves.
**Skin and body protection**	Impervious clothing.
**Respiratory protection**	Suitable respirator.
**Environmental exposure controls**	Keep the product away from drains, water courses or the soil. Clean spillages in a safe way as soon as possible.

9. PHYSICAL AND CHEMICAL PROPERTIES

9.1 Information on basic physical and chemical properties

Appearance	Solid
Odor	No data available
Odor threshold	No data available
pH	No data available
Melting/freezing point	No data available
Boiling point/range	No data available
Flash point	No data available
Evaporation rate	No data available
Flammability (solid, gas)	No data available
Upper/lower flammability or explosive limits	No data available
Vapor pressure	No data available
Vapor density	No data available
Relative density	No data available
Water Solubility	No data available
Partition coefficient	No data available
Auto-ignition temperature	No data available
Decomposition temperature	No data available
Viscosity	No data available
Explosive properties	No data available
Oxidizing properties	No data available

9.2 Other safety information

No data available.

10. STABILITY AND REACTIVITY

10.1 Reactivity

No data available.

10.2 Chemical stability

Stable under recommended storage conditions.

10.3 Possibility of hazardous reactions

No data available.

10.4 Conditions to avoid

No data available.

10.5 Incompatible materials

Strong acids/alkalis, strong oxidising/reducing agents.

10.6 Hazardous decomposition products

Under fire conditions, may decompose and emit toxic fumes.

Other decomposition products - no data available.

11.TOXICOLOGICAL INFORMATION

11.1 Information on toxicological effects

Acute toxicity

Classified based on available data. For more details, see section 2

Skin corrosion/irritation

Classified based on available data. For more details, see section 2

Serious eye damage/irritation

Classified based on available data. For more details, see section 2

Respiratory or skin sensitization

Classified based on available data. For more details, see section 2

Germ cell mutagenicity

Classified based on available data. For more details, see section 2

Carcinogenicity

IARC: No component of this product present at a level equal to or greater than 0.1% is identified as probable, possible or confirmed human carcinogen by IARC.

ACGIH: No component of this product present at a level equal to or greater than 0.1% is identified as a potential or confirmed carcinogen by ACGIH.

NTP: No component of this product present at a level equal to or greater than 0.1% is identified as a anticipated or confirmed carcinogen by NTP.

OSHA: No component of this product present at a level equal to or greater than 0.1% is identified as a potential or confirmed carcinogen by OSHA.

Reproductive toxicity

Classified based on available data. For more details, see section 2

Specific target organ toxicity - single exposure

Classified based on available data. For more details, see section 2

Specific target organ toxicity - repeated exposure

Classified based on available data. For more details, see section 2

Aspiration hazard

Classified based on available data. For more details, see section 2

Additional information

This information is based on our current knowledge. However the chemical, physical, and toxicological properties have not been completely investigated.

12. ECOLOGICAL INFORMATION

12.1 Toxicity

No data available.

12.2 Persistence and degradability

No data available.

12.3 Bioaccumlative potential

No data available.

12.4 Mobility in soil

No data available.

12.5 Results of PBT and vPvB assessment

PBT/vPvB assessment unavailable as chemical safety assessment not required or not conducted.

12.6 Other adverse effects

No data available.

13. DISPOSAL CONSIDERATIONS

13.1 Waste treatment methods

Product

Dispose substance in accordance with prevailing country, federal, state and local regulations.

Contaminated packaging

Conduct recycling or disposal in accordance with prevailing country, federal, state and local regulations.

14. TRANSPORT INFORMATION

DOT (US)

Proper shipping name: Not dangerous goods

UN number: -

Class: -

Packing group: -

IMDG

Proper shipping name: Not dangerous goods

UN number: -

Class: -

Packing group: -

IATA

Proper shipping name: Not dangerous goods

UN number: -

Class: -

Packing group: -

15. REGULATORY INFORMATION

SARA 302 Components:

No chemicals in this material are subject to the reporting requirements of SARA Title III, Section 302.

SARA 313 Components:

This material does not contain any chemical components with known CAS numbers that exceed the threshold (De Minimis) reporting levels established by SARA Title III, Section 313.

SARA 311/312 Hazards:

No SARA Hazards.

Massachusetts Right To Know Components:

No components are subject to the Massachusetts Right to Know Act.

Pennsylvania Right To Know Components:

No components are subject to the Pennsylvania Right to Know Act.

New Jersey Right To Know Components:

No components are subject to the New Jersey Right to Know Act.

California Prop. 65 Components:

This product does not contain any chemicals known to State of California to cause cancer, birth defects, or anyother reproductive harm.

16. OTHER INFORMATION

Copyright 2022 MedChemExpress. The above information is correct to the best of our present knowledge but does not purport to be all inclusive and should be used only as a guide. The product is for research use only and for experienced personnel. It must only be handled by suitably qualified experienced scientists in appropriately equipped and authorized facilities. The burden of safe use of this material rests entirely with the user. MedChemExpress disclaims all liability for any damage resulting from handling or from contact with this product.

SAMPLE INFORMATION			
CAS NO.:	153773-82-1	Acquired By:	LY
Sample infromation:		Date Acquired:	8/12/2022 2:04:10 PM CST
Vial:	2:A,3	Acq. Method Set:	1_POS_2MIN
Injection #:	1	Date Processed:	8/12/2022 2:36:50 PM CST,
Injection Volume:	0.30 ul	Processing Method:	PDA_01, MS_scan
Run Time:	2.8 Minutes	Channel Name:	PDA Ch1 214nm@4.8nm, MS TIC,
Sample Set Name:	20220812	Column Name:	A-RP-985

Auto-Scaled Chromatogram

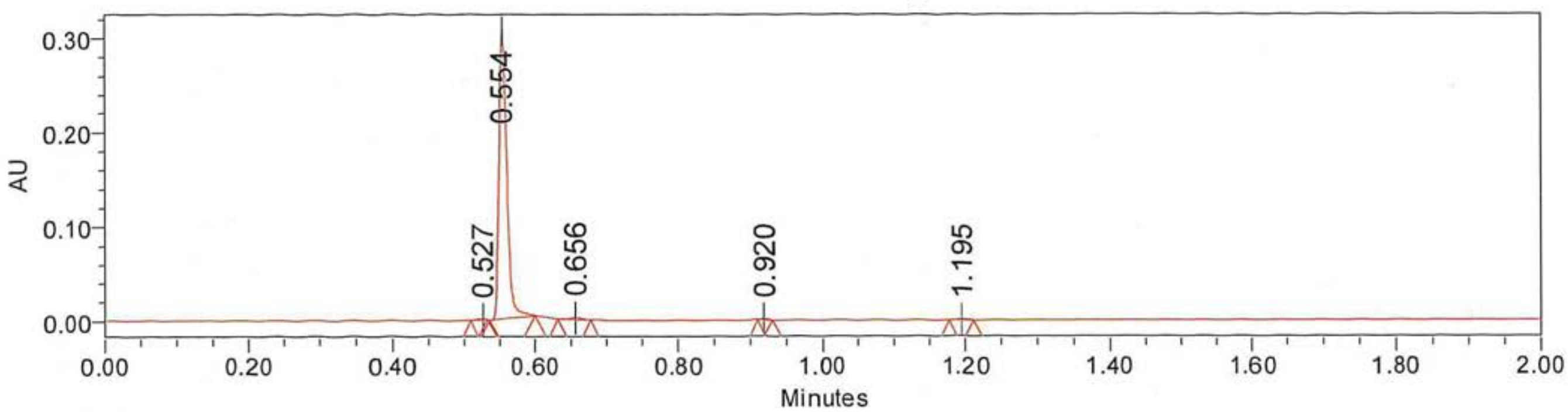

Processed Channel Descr. PDA Ch2 254nm@4.8nm

Auto-Scaled Chromatogram

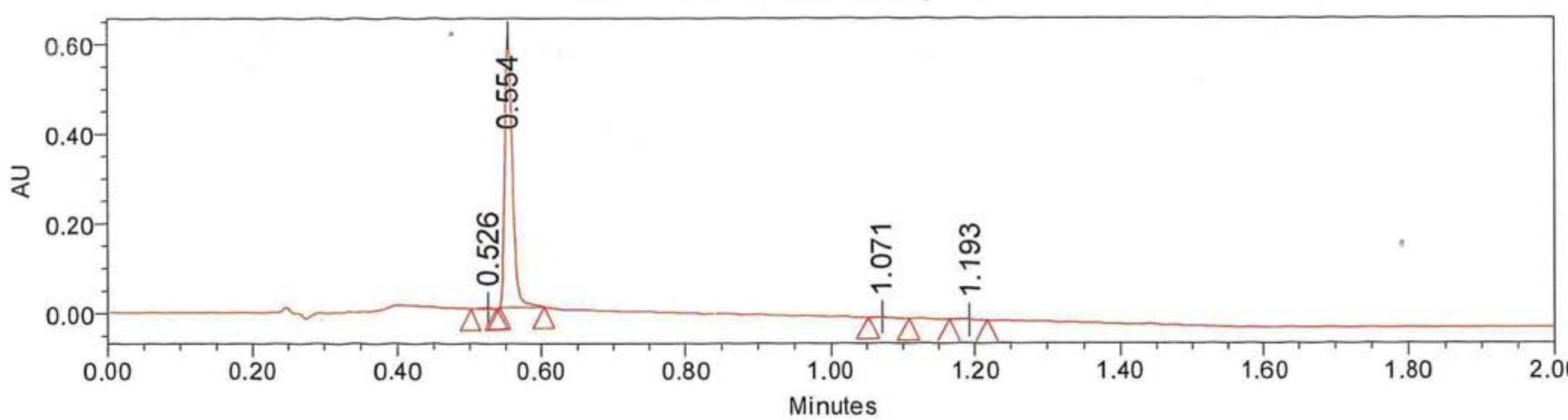

Processed Channel Descr. PDA Ch1 214nm@4.8nm

Auto-Scaled Chromatogram

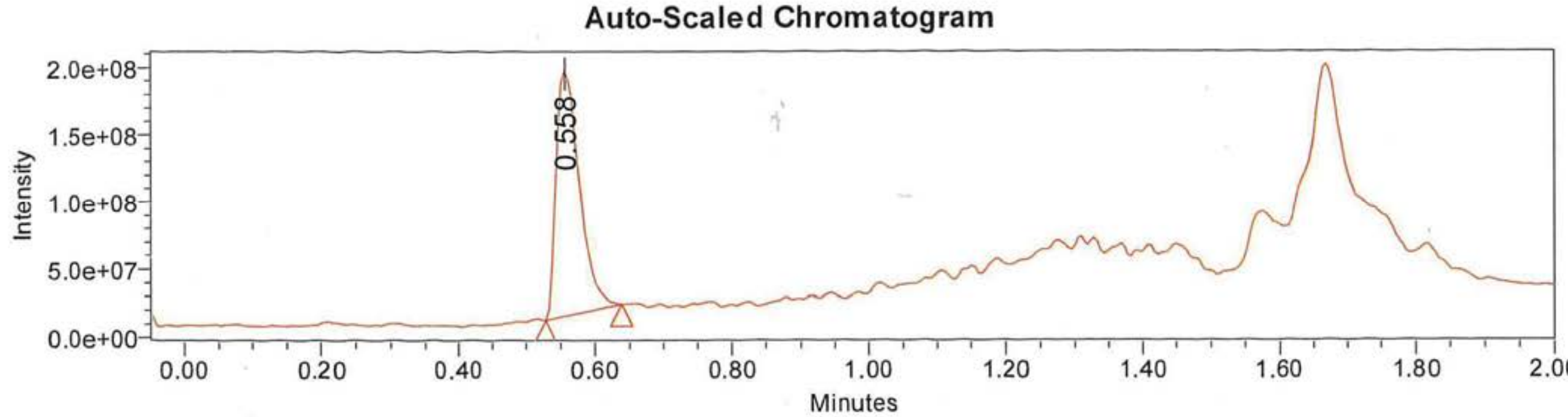

Processed Channel Descr. ACQ-SQD2 1: MS Scan MS TIC, Smoothed by 9 point Savitzky-Golay Filter., Time offset by -0.053 mins. (1: 150.00-1000.00 ES+, Centroid, CV=30)

Peak Results
Channel Name: PDA Ch2 254nm@4.8nm

	RT	Width (sec)	Area	Height	% Area	Base Peak (Combined) (m/z)	Channel Name
1	0.527	1.400	477	640	0.20	153.83	PDA Ch2 254nm@4.8nm
2	0.554	3.800	237134	308413	98.19	151.96	PDA Ch2 254nm@4.8nm
3	0.656	2.650	2446	2436	1.01	151.06	PDA Ch2 254nm@4.8nm
4	0.920	1.200	632	1080	0.26	152.07	PDA Ch2 254nm@4.8nm
5	1.195	2.050	823	941	0.34	152.07	PDA Ch2 254nm@4.8nm

Peak Results
Channel Name: PDA Ch1 214nm@4.8nm

	RT	Width (sec)	Area	Height	% Area	Base Peak (Combined) (m/z)	Channel Name
1	0.526	2.150	1796	2101	0.38	153.84	PDA Ch1 214nm@4.8nm
2	0.554	3.700	471358	613577	98.86	151.97	PDA Ch1 214nm@4.8nm
3	1.071	3.350	1629	1281	0.34	151.03	PDA Ch1 214nm@4.8nm
4	1.193	3.150	1997	1243	0.42	152.07	PDA Ch1 214nm@4.8nm

Peak Results
Channel Name: MS TIC

	RT	Width (sec)	Area	Height	% Area	Base Peak (Combined) (m/z)	Channel Name
1	0.558	6.673	431532509	182380390	100.00	476.14	MS TIC

Match Plot

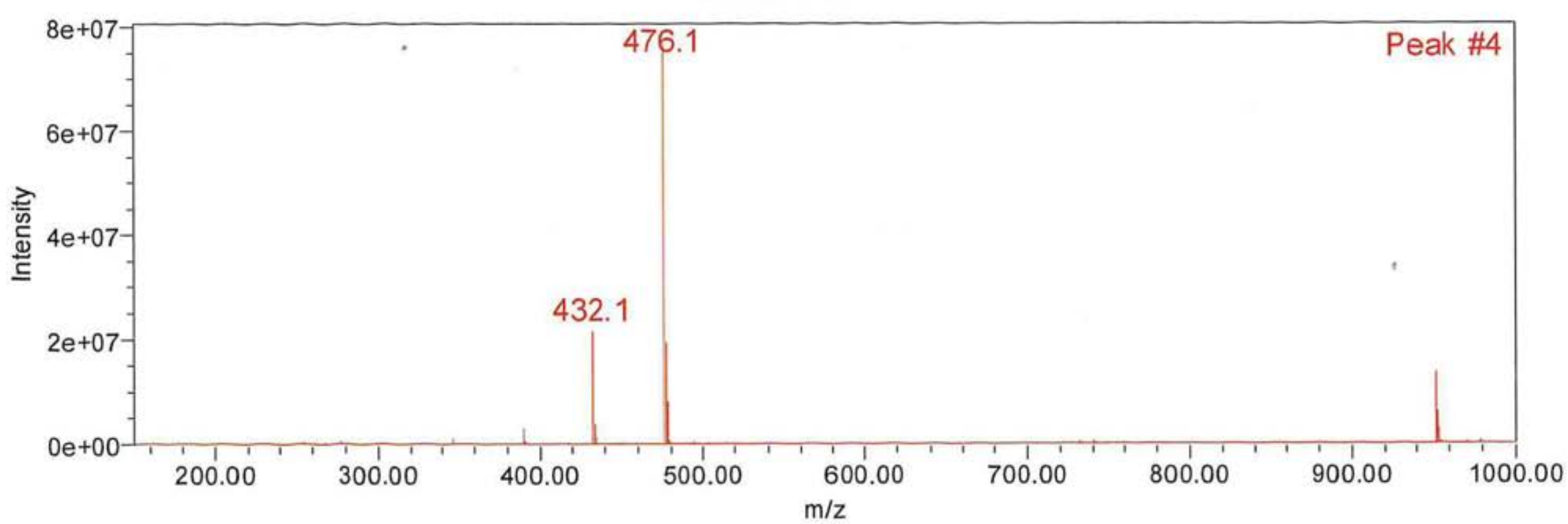

Retention Time 0.558 Channel Name MS TIC

CAS NO.: 153773-82-1, D2O

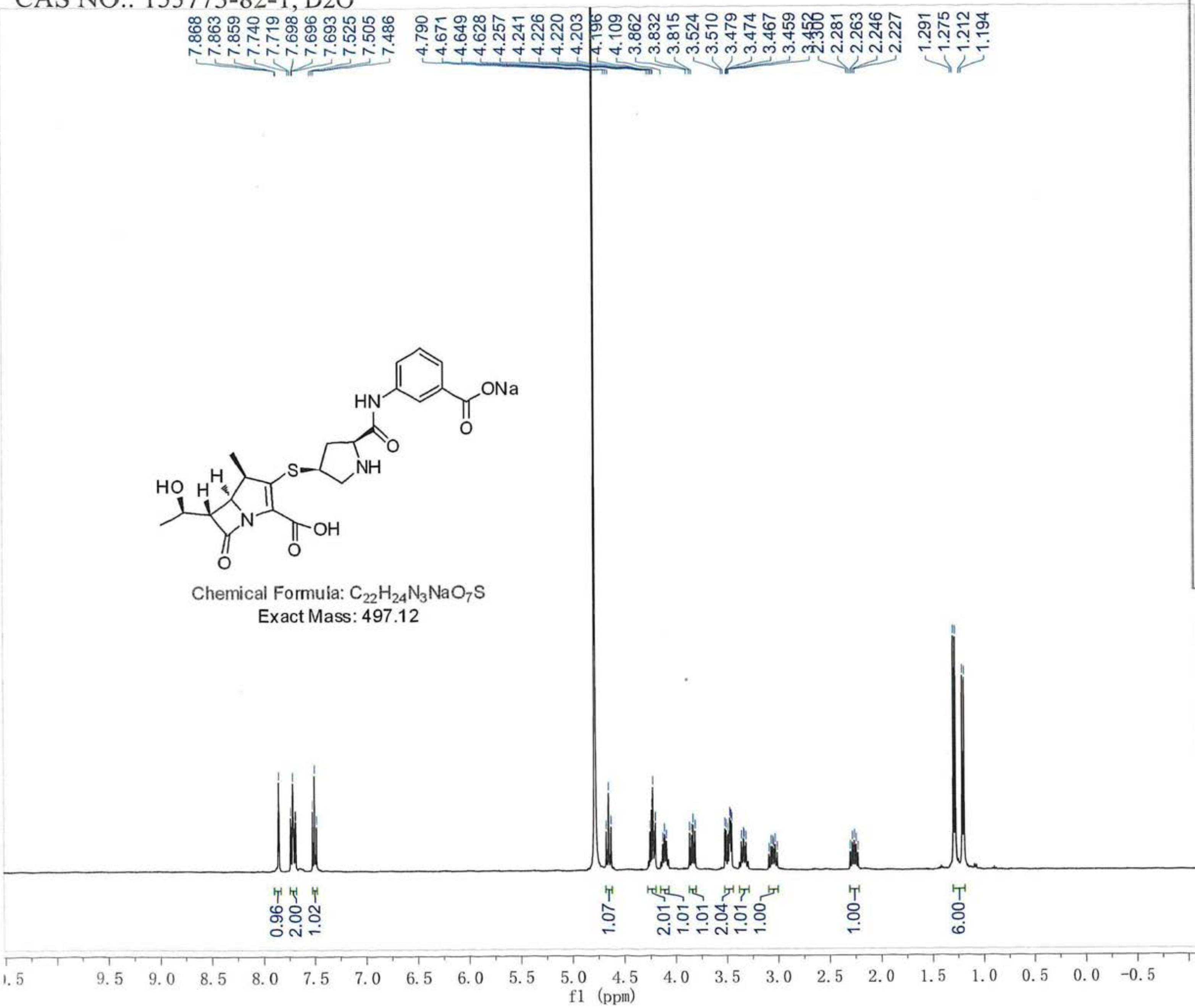

	Parameter	Value
1	Title	
2	Origin	Bruker BioSpin GmbH
3	Owner	nmrsu
4	Spectrometer	Avance
5	Author	
6	Solvent	D2O
7	Temperature	298.2
8	Pulse Sequence	zg30
9	Experiment	1D
10	Number of Scans	8
11	Receiver Gain	101
12	Relaxation Delay	1.0000
13	Pulse Width	8.0000
14	Acquisition Time	3.9977
15	Acquisition Date	2022-08-10T13:49:06
16	Modification Date	2022-08-10T13:53:00
17	Spectrometer Frequency	400.13
18	Nucleus	1H

Inhibitors, Agonists, Screening Libraries
www.MedChemExpress.com

MCE MedChem Express

PO: 42461

Packing List

From:	To:	Purchase PO No.:
MedChemExpress LLC	KCAS Bioanalytical and Biomarker Services	PO42461
1 Deer Park Dr, Suite Q,	Contact Name: Receiving	Customer-Ref No.:
Monmouth Junction,	10830 S. Clay Blair Blvd	
NJ 08852, USA	Olathe Kansas 66061	Invoice No.:
Tel: 609-228-6898	United States	M2225195
Fax: 609-228-5909	Tel:	Date of Issue:
E-mail: sales@medchemexpress.com	Email:	Sept-14-2022
		Ship Date:
		Sept-20-2022

#	Cat. No.	Product Name	Batch	Size	Quantity
1	HY-13625	Ertapenem sodium	180017	10 g	1
2	Technical documentation for listed products				1

RECEIVED
SEP 21 REC'D
By

Total: 2

1. Packages and products should be inspected immediately upon receipt.
2. Please notify us immediately of any damage, error in quantity, or product defects by email or fax.
3. If you are not satisfied with our products, you may return or exchange any items within 30 days of receiving the products.

Thank You for Selecting MCE!

ALSACHIM
a Shimadzu Group Company

CERTIFICATE OF ANALYSIS

**Product Name:**	[$^2H_4$]-Ertapenem sodium salt
**Molecular Formula:**	$C_{22}H_{24}N_3O_7S$. Na
**Labeled Molecular Formula:**	$C_{22}H_{20}D_4N_3O_7S$. Na
**Molecular Weight:**	501.52 g/mol
**Structure:**	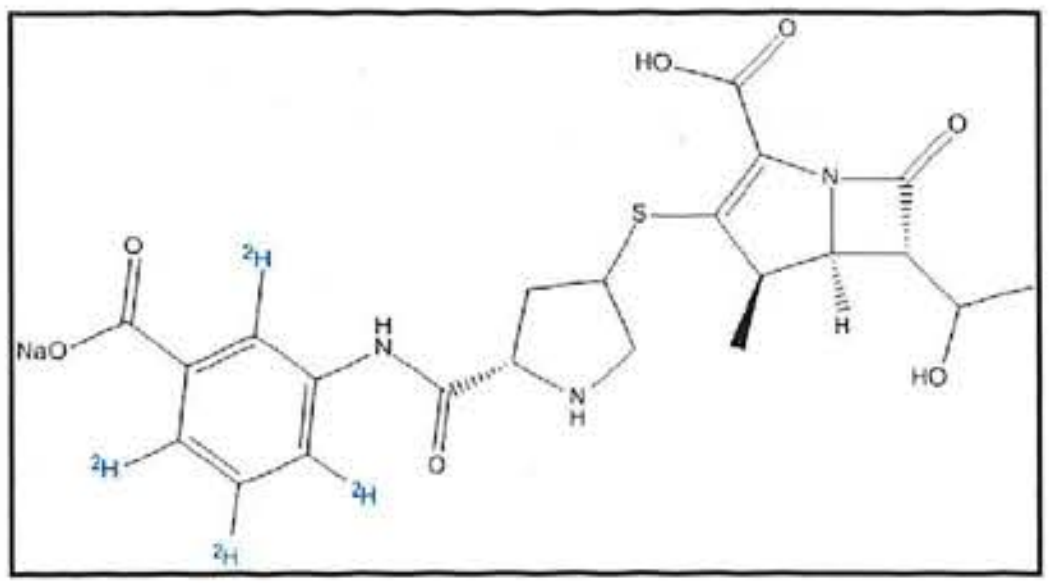

**Batch Number:**	DH-ALS-20-002-P2	
**Product Number:**	C3376	
**CAS Number:**	153773-82-1 unlabeled	
**Storage:**	For a prolonged period, keep at -20°C (±5°C) in a closed container, away from light and moisture	
**Stability:**	Stable (12 months) (*)	
**Extended expiring date:**	Saturday April 29, 2023	
**Solvent solubility:**	Soluble in Water or Acetonitrile/Water (1/1)	
	**Specification:**	**Results:**
**Appearance:**	Pale grey to white solid	Off white solid
**Chemical Purity (HPLC):**	≥ 80.0%	89,4%
**Isotopic Enrichment:**	≥ 98.0% ^{2}H	99.0% ^{2}H
**Unlabeled (M+0) Content:**	Unlabeled (M+0) content < 0.5%	<0.5%
**Mass Spectrometry:**	Conform to structure	Conforms
**Manufacturing date:**	Tuesday January 7, 2020	
**Reanalysis date:**	Friday April 29, 2022	
**Note:**	For research and development use only, not for use in humans.	

Quality Review & Approval

Francis ROESSLINGER, Quality Manager
Date : Wednesday, May 18, 2022

RECEIVED
SEP 21 REC'D
By

ALSACHIM
a Shimadzu Group Company

Analysis Report

<Equipment Information>

LCMS Shimadzu

Alsachim Reference: AN-LC01 + AN-MS01

Pump : LC-40D

Degassing Unit : DGU-405

Auto Sampler : SIL-40C

Diode Array Detector : SPD-M40

Column Oven : CTO-40S

Valve Unit : FCV-20AH2

Liquid Chromatograph Mass Spectrometer : LCMS-2020

Column : Accucore C18 50*4.6 2.6um PN: 17126-054630 SN: 20333623 LOT: 20512

<Sample Information>

Sample Name : DH-ALS-20-002-P2
Data Filename : DH-ALS-20-002-P2_002.lcd
Method Filename : HP-C18-AF-20MIN.lcm
Batch Filename : 2022-APR-29-3.lcb
Vial # : 2-20
Injection Volume : 0,5 uL
Date Acquired : 29/04/2022 14:31:49
Date Processed : 02/05/2022 08:59:47
Solvent : Water
Concentration : 1 mg/ml
Operator : CD

<Peak Table >

PDA Ch1 295nm

Peak#	Ret. Time	Area	Height	Area%
1	1,861	2224	1305	0,544
2	2,364	16348	3642	4,001
3	2,684	365589	157196	89,478
4	2,940	5546	2826	1,357
5	3,046	2444	1326	0,598
6	3,202	596	403	0,146
7	3,540	15028	2285	3,678
8	3,776	806	433	0,197
Total		408582	169417	100,000

HPLC method and UV Instrument Parameters

<<Pump>>

Mode	: Binary gradient
Pump A	: LC-40D X3
Pump B	: LC-40D X3
PressMax	: 400 bar
PressMin	: 0 bar
Pump A Valve Model	: Switching Valve
Pump B Valve Model	: Switching Valve
Gradient Program:	

Pump A + B

#	Time(min) / Flow(mL/min) / B.Conc(%) / B.Curve
1	0,00/ 1,2000/ 1,0/ 0
2	9,00/ / 70,0/
3	15,00/ / 99,0/
4	19,00/ / 99,0/
5	19,10/ / 1,0/

Pump A Mobile Phase Settings	: Water + 0.1% formic acid 0,45 /GPa
Pump B Mobile Phase Settings	: Acetonitrile + 0.1% formic acid 1,20 /GPa

<<LC Time Program>>

Time	Module	Command	Value	Comment
20,00	Controller	Stop		

<<PDA>>

Lamp	: D2 & W
Start Wavelength	: 190 nm
End Wavelength	: 800 nm

<HPLC-UV Chromatogram >

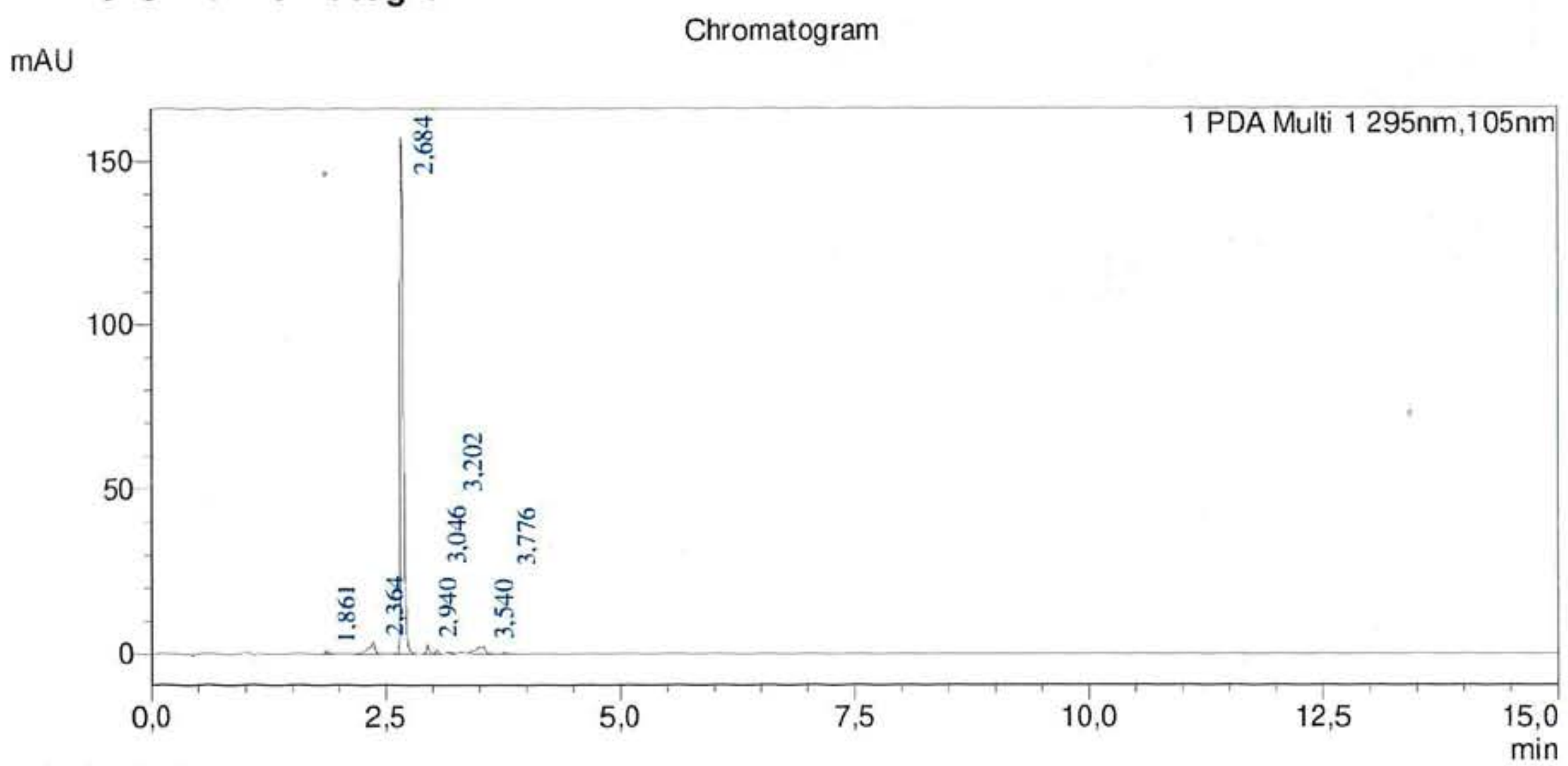

<UV Spectrum >

UV Spectrum

Peak#	: 3
Retention Time	: 2,684 min
Spectrum Operation	: None

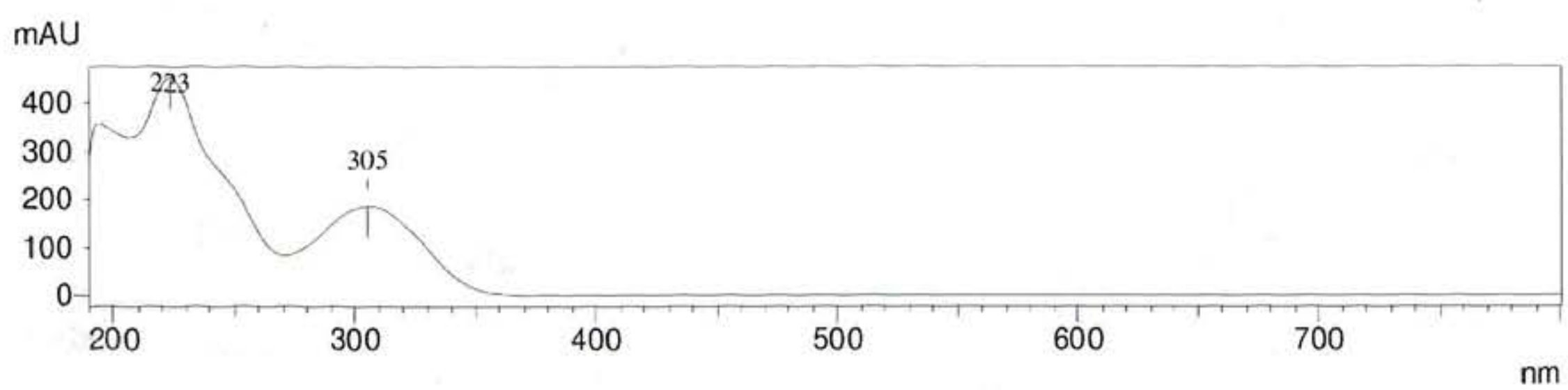

MS Instrument Parameter

<<MS Parameter>>

Initial Valve Position	:0
--Segment 1 Event 1--	
Start Time	:0,00 min
End Time	:20,00 min
Acquisition Mode	:Scan
Polarity	:Positive
Event Time	:1,00 sec
Detector Voltage	:+1,10 kV
Threshold	:0
Start m/z	:100,00
End m/z	:1100,00
Scan Speed	:1072 u/sec
Interface Volt.	:Use the Data in the Tuning File
DL Volt.	:Use the Data in the Tuning File
Qarray DC Voltage	:Use the Data in the Tuning File
Qarray DC Voltage	:Use the Data in the Tuning File
--Segment 1 Event 2--	
Start Time	:0,00 min
End Time	:20,00 min
Acquisition Mode	:Scan
Polarity	:Negative
Event Time	:1,00 sec
Detector Voltage	:+1,10 kV
Threshold	:0
Start m/z	:100,00
End m/z	:1100,00
Scan Speed	:1072 u/sec
Interface Volt.	:Use the Data in the Tuning File
DL Volt.	:Use the Data in the Tuning File
Qarray DC Voltage	:Use the Data in the Tuning File
Qarray DC Voltage	:Use the Data in the Tuning File

<<Interface>>

Interface	:DUIS
DL Temperature	:300 C
Nebulizing Gas Flow	:1,50 L/min
Heat Block	:400 C
Drying Gas	:On
	18,00 L/min

Segment	Start Time	End Time	Ionization Mode
1	0,00	20,00	ESI&APCI

<HPLC-MS Chromatogram>

MS Chromatogram

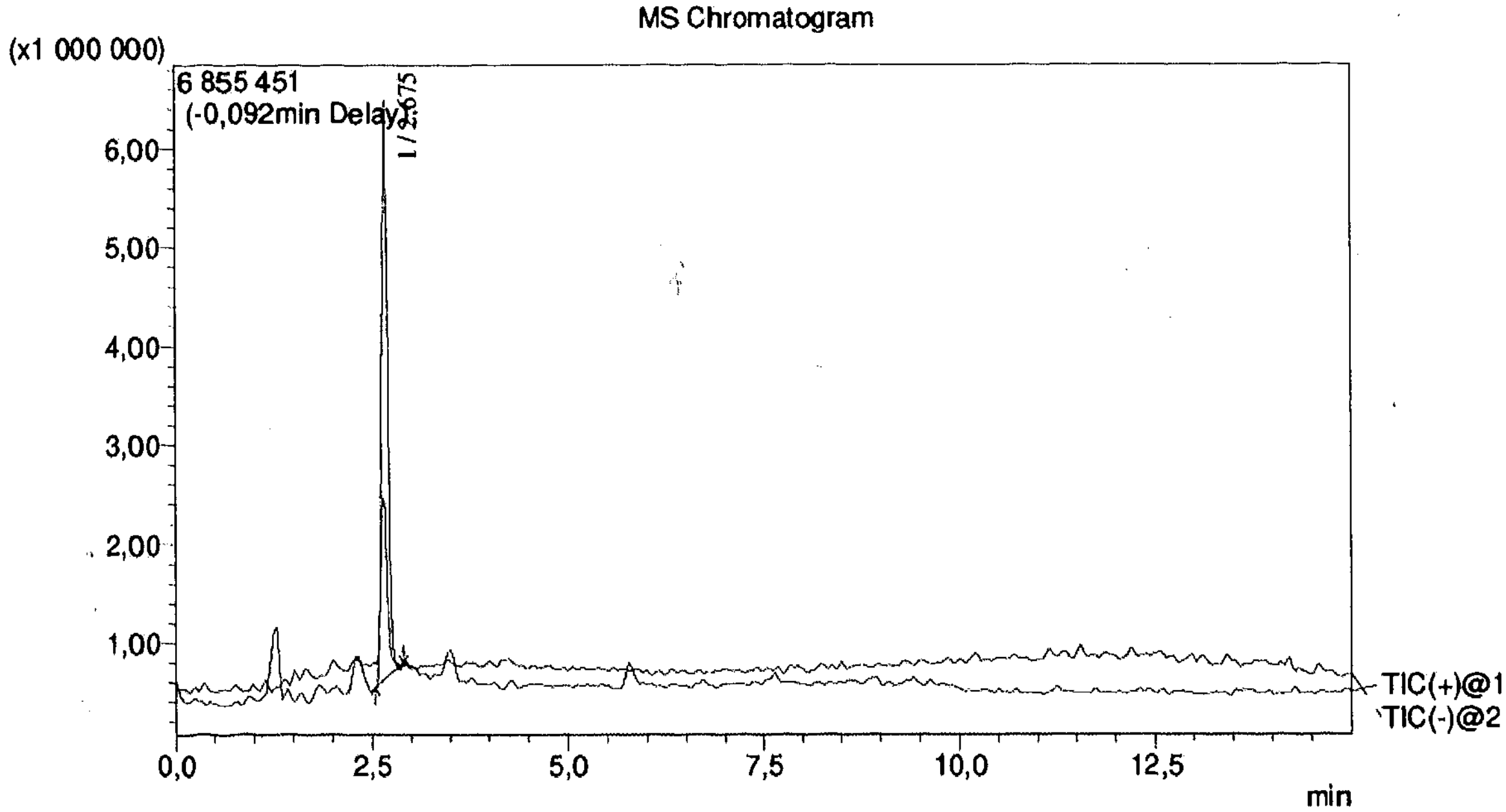

< Mass spectrum>

Peak#:1 R.Time:2,675(Scan#:167)
MassPeaks:660
Spectrum Mode:Averaged 2,641-2,708(165-169)
BG Mode:Calc Segment 1 - Event 1

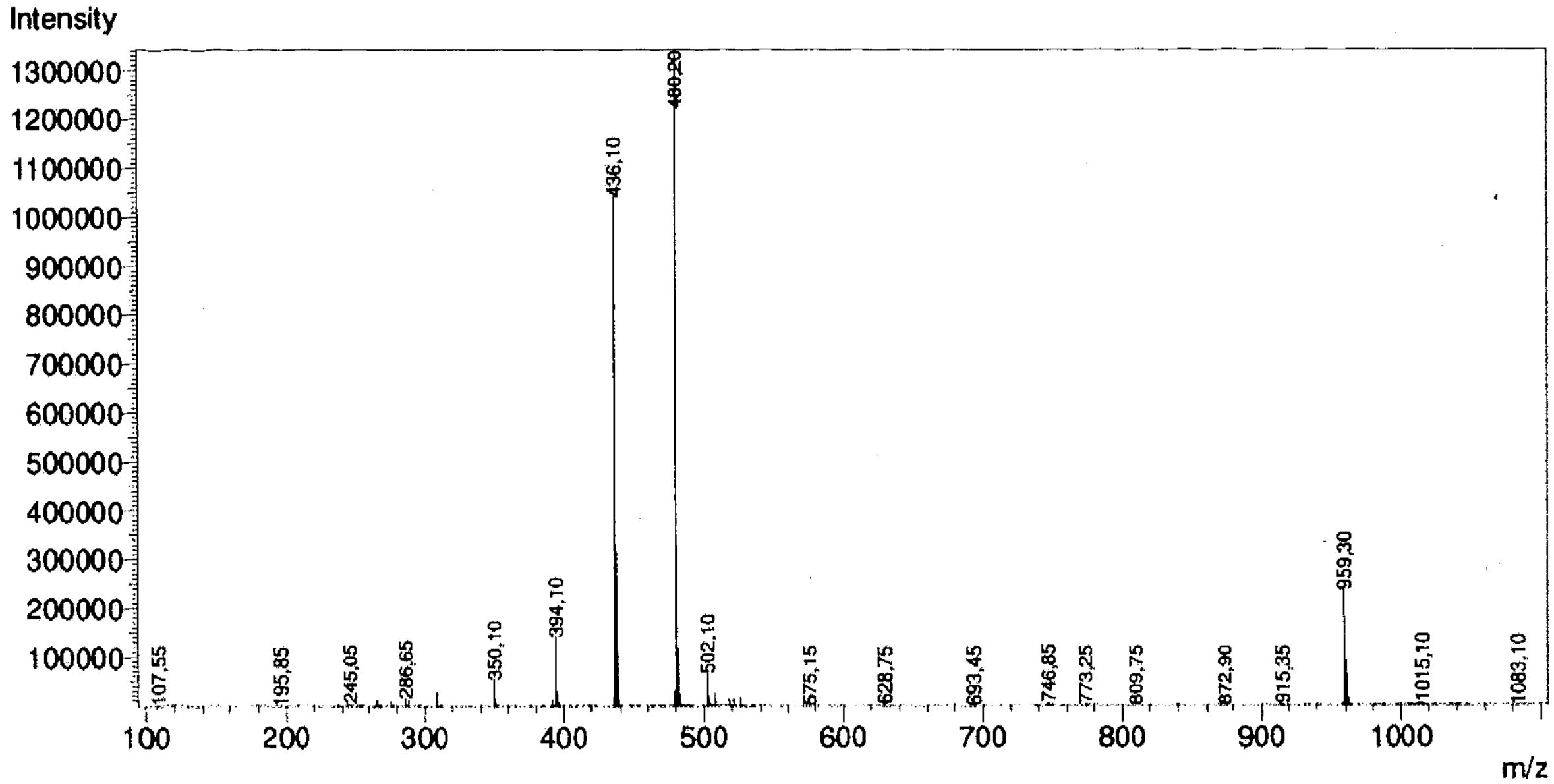

< Mass Profile Spectrum>

Peak#:1 R.Time:2,675(Scan#:167)
MassPeaks:660
Spectrum Mode:Averaged 2,641-2,708(165-169)
BG Mode:Calc Segment 1 - Event 1

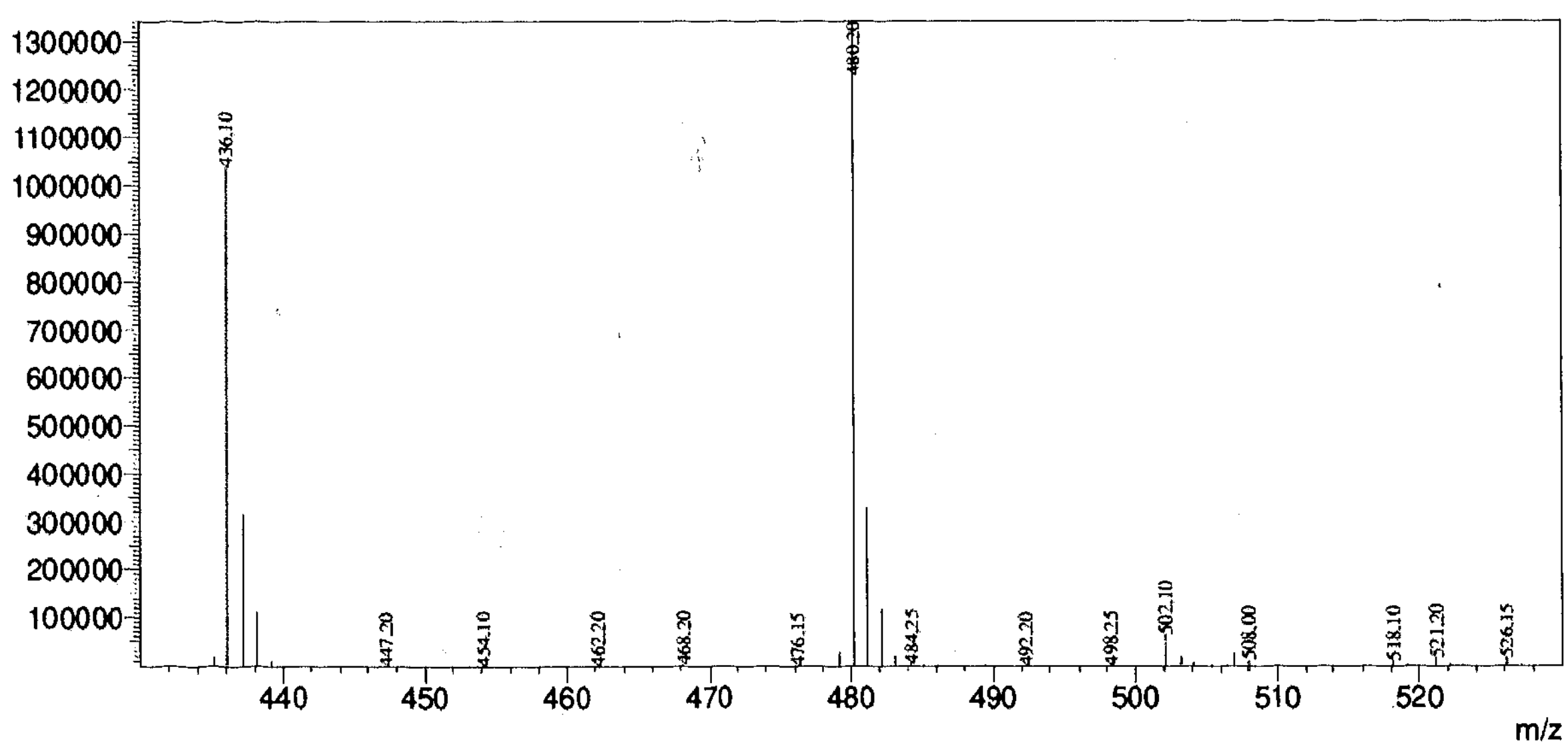

ALSACHIM
a Shimadzu Group Company

SAFETY DATA SHEET

(REACH regulation (EC) n° 1907/2006 - n° 2020/878)

SECTION 1 : IDENTIFICATION OF THE SUBSTANCE/MIXTURE AND OF THE COMPANY/UNDERTAKING

1.1. Product identifier

Product name : *[2H4]-ERTAPENEM SODIUM SALT*

Product code : C3376.

N° CAS 153773-82-1 unlabeled

1.2. Relevant identified uses of the substance or mixture and uses advised against

Identified uses : Laboratory chemicals, Manufacture of substances

1.3. Details of the supplier of the safety data sheet

Registered company name : Alsachim.

Address : 160 rue Tobias Stimmer.67400.Illkirch Graffenstaden.France.

Telephone : +33 (0) 390 402 200. Fax : +33 (0) 390 402 199 .

E-mail : contact@alsachim.com

1.4. Emergency telephone number : +33 (0)1 45 42 59 59.

Association/Organisation : INRS / ORFILA http://www.centres-antipoison.net.

SECTION 2 : HAZARDS IDENTIFICATION

2.1. Classification of the substance or mixture

In compliance with EC regulation No. 1272/2008 and its amendments.

Respiratory sensitisation, Category 1 (Resp. Sens. 1, H334).

Hazardous to the aquatic environment - Acute hazard, Category 1 (Aquatic Acute 1, H400).

Hazardous to the aquatic environment - Chronic hazard, Category 2 (Aquatic Chronic 2, H411).

This substance does not present a physical hazard. Refer to the recommendations regarding the other products present on the site.

2.2. Label elements

In compliance with EC regulation No. 1272/2008 and its amendments.

Hazard pictograms :

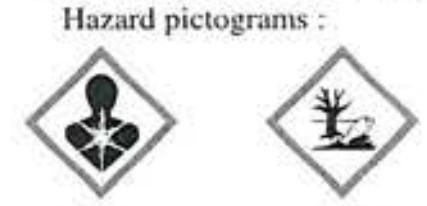

GHS08 GHS09

Signal Word :

DANGER

Product identifiers :

[2H4]-ERTAPENEM SODIUM SALT

Hazard statements :

H334	May cause allergy or asthma symptoms or breathing difficulties if inhaled.
H410	Very toxic to aquatic life with long lasting effects.

Precautionary statements - Prevention :

P261	Avoid breathing dust.
P273	Avoid release to the environment.
P284	[In case of inadequate ventilation] wear respiratory protection.

Precautionary statements - Response :

P304 + P340	IF INHALED: Remove person to fresh air and keep comfortable for breathing.
P342 + P311	If experiencing respiratory symptoms: Call a POISON CENTER or doctor.
P391	Collect spillage.

Precautionary statements - Disposal :

P501	Dispose of contents/container to an approved waste disposal facility.

2.3. Other hazards

The substance does not fulfil the PBT or vPvP criteria in accordance with annexe XIII of the REACH regulations EC 1907/2006.

SECTION 3 : COMPOSITION/INFORMATION ON INGREDIENTS

3.1. Substances

N° CAS 153773-82-1 unlabeled

* Molecular weight 501,52 g/mol

Composition :

Identification	(EC) 1272/2008	Note	%
[2H4]-ERTAPENEM SODIUM SALT	GHS09, GHS08<br>Dgr<br>Resp. Sens. 1, H334<br>Aquatic Chronic 2, H411<br>Aquatic Acute 1, H400<br>M Acute = 1		100%

SECTION 4 : FIRST AID MEASURES

As a general rule, in case of doubt or if symptoms persist, always call a doctor.

NEVER induce swallowing by an unconscious person.

4.1. description of first aid measures

In the event of exposure by inhalation :

In the event of massive inhalation of dust, remove the person exposed to fresh air. Keep warm and at rest.

If breathing is irregular or has stopped, effect mouth-to-mouth resuscitation and call a doctor.

In the event of an allergic reaction, seek medical attention.

In the event of splashes or contact with eyes :

Wash thoroughly with fresh, clean water for 15 minutes holding the eyelids open.

If there is any redness, pain or visual impairment, consult an ophthalmologist.

In the event of splashes or contact with skin :

Watch out for any remaining product between skin and clothing, watches, shoes, etc.

In the event of swallowing :

Seek medical attention, showing the label.

4.2. Most important symptoms and effects, both acute and delayed

No data available.

4.3. Indication of any immediate medical attention and special treatment needed

No data available.

SECTION 5 : FIREFIGHTING MEASURES

Non-flammable.

5.1. Extinguishing media

5.2. Special hazards arising from the substance or mixture

A fire will often produce a thick black smoke. Exposure to decomposition products may be hazardous to health.

Do not breathe in smoke.

In the event of a fire, the following may be formed :

- carbon monoxide (CO)
- carbon dioxide (CO_2)

5.3. Advice for firefighters

No data available.

SECTION 6 : ACCIDENTAL RELEASE MEASURES

6.1. Personal precautions, protective equipment and emergency procedures

Consult the safety measures listed under headings 7 and 8.

For non first aid worker

Avoid inhaling dust.

If a large quantity has been spilt, evacuate all personnel and only allow intervention by trained operators equipped with safety apparatus.

14.7. Maritime transport in bulk according to IMO instruments

No data available.

SECTION 15: Regulatory information

15.1. Safety, health and environmental regulations/legislation specific for the substance or mixture

- Classification and labelling information included in section 2:

The following regulations have been used:

- EU Regulation No. 1272/2008 amended by EU Regulation No. 2021/643 (ATP 16)
- EU Regulation No. 1272/2008 amended by EU Regulation No. 2021/849 (ATP 17)

- Container information:

The substance is packed in an interior packaging, not exceeding 10 ml, the required labelling elements appear on the outer packaging.

- Particular provisions :

No data available.

15.2. Chemical safety assessment

No data available.

SECTION 16 : OTHER INFORMATION

Since the user's working conditions are not known by us, the information supplied on this safety data sheet is based on our current knowledge and on national and community regulations.

It is at all times the responsibility of the user to take all necessary measures to comply with legal requirements and local regulations.

The information in this safety data sheet must be regarded as a description of the safety requirements relating to the substance and guarantee of the properties thereof.

Wording of the phrases mentioned in section 3 :

H334	May cause allergy or asthma symptoms or breathing difficulties if inhaled.
H400	Very toxic to aquatic life.
H411	Toxic to aquatic life with long lasting effects.

Abbreviations :

REACH : Registration, Evaluation, Authorization and Restriction of Chemical Substances.

ADR : European agreement concerning the international carriage of dangerous goods by Road.

IMDG : International Maritime Dangerous Goods.

IATA : International Air Transport Association.

ICAO : International Civil Aviation Organisation

RID : Regulations concerning the International carriage of Dangerous goods by rail.

WGK : Wassergefahrdungsklasse (Water Hazard Class).

GHS08 : Health hazard

GHS09 : Environment

PBT: Persistent, bioaccumulable and toxic.

vPvB : Very persistent, very bioaccumulable.

SVHC : Substances of very high concern.

espiratory protection

void inhaling dust.

the ventilation is insufficient, wear appropriate breathing apparatus.

/hen workers are confronted with concentrations that are above occupational exposure limits, they must wear a suitable, approved, espiratory protection device.

ype of FFP mask :

/ear a disposable half-mask dust filter in accordance with standard EN149/A1.

ategory :

FFP1

nti-gas and vapour filter(s) (Combined filters) in accordance with standard EN14387 :

A1 (Brown)

TION 9 : PHYSICAL AND CHEMICAL PROPERTIES

Information on basic physical and chemical properties

**ysical state**	
hysical state :	Solid.
**lour**	
olor	Pale grey to white
**our**	
dour threshold :	Not stated.
**lting point**	
lelting point/melting range :	Not relevant.
**ezing point**	
reezing point / Freezing range :	Not stated.
**iling point or initial boiling point and boiling range**	
oiling point/boiling range :	Not relevant.
**mmability**	
lammability (solid, gas) :	Not stated.
**wer and upper explosion limit**	
xplosive properties, lower explosivity limit (%) :	Not stated.
xplosive properties, upper explosivity limit (%) :	Not stated.
**sh point**	
lash point interval :	Not relevant.
**to-ignition temperature**	
elf-ignition temperature :	Not relevant.
**composition temperature**	
ecomposition point/decomposition range :	Not relevant.
H (aqueous solution) :	Not stated.
H :	Not relevant.
**nematic viscosity**	
iscosity :	Not stated.
**ubility**	
/ater solubility :	Soluble.
at solubility :	Not stated.
olubility	Mixture of Acetonitrile and Water (1/1)
**rtition coefficient n-octanol/water (log value)**	
artition coefficient: n-octanol/water :	Not stated.
**pour pressure**	
/apour pressure (50°C) :	Below 110 kPa (1.10 bar).
**nsity and/or relative density**	
Density :	= 1
**lative vapour density**	
/apour density :	Not stated.

9.2. Other information

Molecular formula : C22H20D4N3O7S . Na

9.2.1. Information with regard to physical hazard classes

No data available.

9.2.2. Other safety characteristics

No data available.

SECTION 10 : STABILITY AND REACTIVITY

10.1. Reactivity

No data available.

10.2. Chemical stability

This substance is stable under the recommended handling and storage conditions in section 7.

10.3. Possibility of hazardous reactions

No data available.

10.4. Conditions to avoid

Avoid :

- formation of dusts

Dusts can form an explosive mixture with air.

10.5. Incompatible materials

No data available.

10.6. Hazardous decomposition products

The thermal decomposition may release/form :

- carbon monoxide (CO)
- carbon dioxide (CO_2)

SECTION 11 : TOXICOLOGICAL INFORMATION

11.1. Information on hazard classes as defined in Regulation (EC) No 1272/2008

May cause hypersensitivity of the respiratory tracts with effects taking the form of asthma, rhinitis/conjunctivitis or alveolitis.

11.1.1. Substances

No toxicological data available for the substances.

SECTION 12 : ECOLOGICAL INFORMATION

Very toxic to aquatic life with long lasting effects.

The product must not be allowed to run into drains or waterways.

12.1. Toxicity

12.2. Persistence and degradability

No data available.

12.3. Bioaccumulative potential

No data available.

12.4. Mobility in soil

No data available.

12.5. Results of PBT and vPvB assessment

No data available.

12.6. Endocrine disrupting properties

No data available.

12.7. Other adverse effects

No data available.

SECTION 13 : DISPOSAL CONSIDERATIONS

Proper waste management of the substance and/or its container must be determined in accordance with Directive 2008/98/EC.

13.1. Waste treatment methods

Do not pour into drains or waterways.

Waste :

Waste management is carried out without endangering human health, without harming the environment and, in particular without risk to water, air, soil, plants or animals.

Recycle or dispose of waste in compliance with current legislation, preferably via a certified collector or company.

Do not contaminate the ground or water with waste, do not dispose of waste into the environment.

Soiled packaging :

Empty container completely. Keep label(s) on container.

Give to a certified disposal contractor.

SECTION 14 : TRANSPORT INFORMATION

Transport product in compliance with provisions of the ADR for road, RID for rail, IMDG for sea and ICAO/IATA for air transport (ADR 2021 - IMDG 2020 - ICAO/IATA 2021).

14.1. UN number or ID number

3077

14.2. UN proper shipping name

UN3077=ENVIRONMENTALLY HAZARDOUS SUBSTANCE, SOLID, N.O.S.

([2h4]-ertapenem sodium salt)

14.3. Transport hazard class(es)

- Classification :

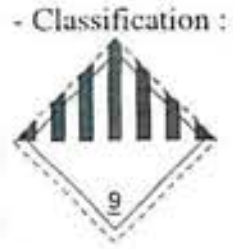

9

14.4. Packing group

III

14.5. Environmental hazards

- Environmentally hazardous material :

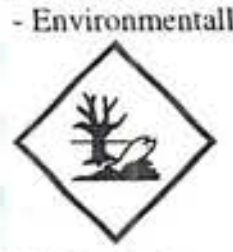

14.6. Special precautions for user

ADR/RID	Class	Code	Pack gr.	Label	Ident.	LQ	Provis.	EQ	Cat.	Tunnel
	9	M7	III	9	90	5 kg	274 335 375 601	E1	3	-

Not subject to this regulation if Q <= 5 l / 5 kg (ADR 3.3.1 - DS 375)

IMDG	Class	2°Label	Pack gr.	LQ	EMS	Provis.	EQ	Stowage Handling	Segregation
	9	-	III	5 kg	F-A, S-F	274 335 966 967 969	E1	Category A SW23	-

Not subject to this regulation if Q <= 5 l / 5 kg (IMDG 3.3.1 - 2.10.2.7)

IATA	Class	2°Label	Pack gr.	Passager	Passager	Cargo	Cargo	note	EQ
	9	-	III	956	400 kg	956	400 kg	A97 A158 A179 A197 A215	E1
	9	-	III	Y956	30 kg G	-	-	A97 A158 A179 A197 A215	E1

Not subject to this regulation if Q <= 5 l / 5 kg (IATA 4.4.4 - DS A197)

For limited quantities, see part 2.7 of the OACI/IATA and chapter 3.4 of the ADR and IMDG.

For excepted quantities, see part 2.6 of the OACI/IATA and chapter 3.5 of the ADR and IMDG.

For first aid worker

First aid workers will be equipped with suitable personal protective equipment (See section 8).

6.2. Environmental precautions

Prevent any material from entering drains or waterways.

6.3. Methods and material for containment and cleaning up

Retrieve the product by mechanical means (sweeping/vacuuming).

6.4. Reference to other sections

No data available.

SECTION 7 : HANDLING AND STORAGE

Requirements relating to storage premises apply to all facilities where the substance is handled.

7.1. Precautions for safe handling

Always wash hands after handling.

Remove and wash contaminated clothing before re-using.

Fire prevention :

Handle in well-ventilated areas.

Prevent access by unauthorised personnel.

Recommended equipment and procedures :

For personal protection, see section 8.

Observe precautions stated on label and also industrial safety regulations.

Also provide breathing apparatus for certain short tasks of an exceptional nature and for emergency interventions.

In all cases, recover emissions at source.

Prohibited equipment and procedures :

No smoking, eating or drinking in areas where the substance is used.

7.2. Conditions for safe storage, including any incompatibilities

No data available.

Storage

Keep the container tightly closed in a dry, well-ventilated place.

For a prolonged period, keep at -20°C (±5°C) in a closed container, away from light and moisture.

Packaging

Always keep in packaging made of an identical material to the original.

7.3. Specific end use(s)

No data available.

SECTION 8 : EXPOSURE CONTROLS/PERSONAL PROTECTION

8.1. Control parameters

No data available.

8.2. Exposure controls

Personal protection measures, such as personal protective equipment

Use personal protective equipment that is clean and has been properly maintained.

Store personal protective equipment in a clean place, away from the work area.

Never eat, drink or smoke during use. Remove and wash contaminated clothing before re-using. Ensure that there is adequate venti especially in confined areas.

- Eye / face protection

Avoid contact with eyes.

Before handling powders or dust emission, wear mask goggles in accordance with standard EN166.

- Hand protection

Use suitable protective gloves that are resistant to chemical agents in accordance with standard EN ISO 374-1.

Gloves must be selected according to the application and duration of use at the workstation.

Protective gloves need to be selected according to their suitability for the workstation in question : other chemical products that n handled, necessary physical protections (cutting, pricking, heat protection), level of dexterity required.

- Body protection

Work clothing worn by personnel shall be laundered regularly.

After contact with the product, all parts of the body that have been soiled must be washed.

ANALYTICAL RESEARCH

CERTIFICATE OF ANALYSIS FOR REFERENCE STANDARD

**Name**	:	**Zidebactam (WCK 5107)**			
Molecular Formula	:	$C_{13}H_{21}N_5O_7S.2H_2O$	Batch Number	:	WK1/WP-0060/016
Molecular Weight	:	427.45	Laboratory Note Book Reference	:	WA-2004-038
Date of Analysis	:	30/03/2022	Month of Manufacturing	:	May' 2019
Valid Up To	:	29/03/2023	Reference Standard Number	:	RS/2022-013
Effective Date	:	13/04/2022	Page Number	:	Page 1 of 4

Sr. No.	Tests	Specifications	Results
1	Description	White to pale yellow powder	White powder
2	Identification<br>(By IR, Mass & NMR Spectroscopy)	Spectral data should conform to the structure	Spectral data conforms the structure
3	Solubility	Freely soluble in water,<br>Insoluble in dichloromethane	Freely soluble in water, Insoluble in dichloromethane
4	Constituted solution<br>(10% w/v solution in WFI)<br>Completeness of solution<br><br>Clarity of solution	 <br>The solid dissolves completely, leaving no visible residue as un-dissolved matter<br>The constituted solution is not significantly less clear than an equal volume of the WFI contained in a similar vessel and examined similarly	 <br>Complies<br><br>Complies
5	pH of solution	Between 5.0 to 7.0	6.0
6	Absorbance of solution (1.25 % w/v in water at 420 nm)	Not more than 0.2 AU	0.01 AU
7	Water content (By KF Titration)	Not more than 12.0 % w/w	8.7 % w/w
8	Specific optical rotation (On anhydrous basis)	Between –28° to -40°	-36°
9	Heavy metals	Not more than 20 µg/g	Less than 20 µg/g
10	Palladium content (By ICP-OES)	Not more than 10 µg/g	BLQ (LOQ 2.5 µg/g)*

	Prepared by	Checked by	Approved by
Name :	Mr. Sitaram Ubale	Mr. Rajendra Chavan	Mr Subhash Chandra Shukla
Department :	AVL	AVL	CRDQ
Sign :	[signature]	[signature]	[signature]
Date :	12/04/2022	12/04/2022	13/04/2022

Format No. : AVL-045-F-02-00

Ranked # 1 In Healthcare Best Companies to Work for 2015 India

ANALYTICAL RESEARCH

CERTIFICATE OF ANALYSIS FOR REFERENCE STANDARD

**Name**	:	**Zidebactam (WCK 5107)**			
Molecular Formula	:	$C_{13}H_{21}N_5O_7S.2H_2O$	Batch Number	:	WK1/WP-0060/016
Molecular Weight	:	427.45	Laboratory Note Book Reference	:	WA-2004-038
Date of Analysis	:	30/03/2022	Month of Manufacturing	:	May' 2019
Valid Up To	:	29/03/2023	Reference Standard Number	:	RS/2022-013
Effective Date	:	23/04/2022	Page Number	:	Page 2 of 4

Sr. No.	Tests	Specifications	Results
11	Related substances (By HPLC) :		
	i. Impurity -1	Not more than 2.00 % w/w	0.28 % w/w
	ii. Impurity -2	Not more than 1.00 % w/w	Not detected
	iii. Impurity -3	Not more than 1.00 % w/w	0.10 % w/w
	iv. Impurity -4	Not more than 0.10 % w/w	Not detected
	v. Specified impurity at RRT 2.16-2.28 (MW 782)	Not more than 0.50 % w/w	0.03 % w/w
	vi. Any unspecified impurity	Not more than 0.15 % w/w	BDL
	vii. Total impurities	Not more than 4.00 % w/w	0.41 % w/w
12	Residual solvents (By GC) :		
	Group A		
	i. Isopropyl alcohol	Not more than 5000 µg/g	Not detected
	ii. Dichloromethane	Not more than 600 µg/g	Not detected
	iii. Methanol	Not more than 3000 µg/g	1762 µg/g
	iv. Ethyl acetate	Not more than 5000 µg/g	Not detected
	v. Cyclohexane	Not more than 3880 µg/g	Not detected
	vi. Acetone	Not more than 5000 µg/g	Not detected
	vii. Methyl tertiary butyl ether	Not more than 5000 µg/g	Not detected
	viii. Toluene	Not more than 890 µg/g	Not detected
	ix. Ethanol	Not more than 5000 µg/g	2990 µg/g
	Group B		
	i. Triethylamine	Not more than 250 µg/g	Not detected
	ii. Pyridine	Not more than 200 µg/g	Not detected

	Prepared by	Checked by	Approved by
Name :	Mr. Sitaram Ubale	Mr. Rajendra Chavan	Mr Subhash Chandra Shukla
Department :	AVL	AVL	CRDQ
Sign :	[signature]	[signature]	[signature]
Date :	12/04/2022	12/04/2022	13/04/2022

Format No. : AVL-045-F-02-00

Ranked # 1 in Healthcare
GREAT PLACE TO WORK Best Companies to Work for 2015 India

WOCKHARDT LIFE WINS

ANALYTICAL RESEARCH

CERTIFICATE OF ANALYSIS FOR REFERENCE STANDARD

**Name**	:	**Zidebactam (WCK 5107)**			
Molecular Formula	:	$C_{13}H_{21}N_5O_7S.2H_2O$	Batch Number	:	WK1/WP-0060/016
Molecular Weight	:	427.45	Laboratory Note Book Reference	:	WA-2004-038
Date of Analysis	:	30/03/2022	Month of Manufacturing	:	May' 2019
Valid Up To	:	29/03/2023	Reference Standard Number	:	RS/2022-013
Effective Date	:	23/04/2022	Page Number	:	Page 3 of 4

Sr. No.	Tests	Specifications	Results
13	Sodium content (By ICP-OES)	Not more than 0.5 % w/w	BLQ (LOQ 0.25 % w/w)*
14	Sulfate content (By Ion chromatography)	Not more than 1.0 % w/w	BLQ (LOQ 0.626 % w/w)
15	Mono-methyl sulphate content (By Ion chromatography)	Not more than 0.15% w/w	Not detected
16	Polymorphism (By X-ray Diffraction)	The diffractogram should exhibit characteristic 2 theta values at 13.8, 15.6, 20.3 and 24.3 (± 0.2°)	The diffractogram exhibits characteristic 2 theta values at 13.8, 15.6, 20.3 & 24.3
17	Potency (on as is basis)	Not applicable	90.4 %

BLQ = Below limit of quantification
BDL = Below disregard limit. Disregard limit 0.05 %
LOQ = Limit of quantification

* LOQ Values are based on analytical method verification report (Report nos. MVR/VP17053 and MVR/VP17054) of Analytical Solutions (CRO).

Specification Reference Number: **ZDB-DSS-112-05**

Remark: Potency is calculated on the basis of Total impurities, Water content, Residual solvents, Sulphate, Mono-methyl sulphate, Sodium and Palladium content.

Storage Condition: Store at 2°C to 8°C temperature.

	Prepared by	Checked by	Approved by
Name :	Mr. Sitaram Ubale	Mr. Rajendra Chavan	Mr Subhash Chandra Shukla
Department :	AVL	AVL	CRDQ
Sign :	[signature]	[signature]	[signature]
Date :	12/04/2022	12/04/2022	13/04/2022

Format No. : AVL-045-F-02-00

Ranked # 1 In Healthcare
Best Companies to Work for 2015 India

ANALYTICAL RESEARCH

CERTIFICATE OF ANALYSIS FOR REFERENCE STANDARD

Name	:	**Zidebactam (WCK 5107)**			
Molecular Formula	:	$C_{13}H_{21}N_5O_7S.2H_2O$	Batch Number	:	WK1/WP-0060/016
Molecular Weight	:	427.45	Laboratory Note Book Reference	:	WA-2004-038
Date of Analysis	:	30/03/2022	Month of Manufacturing	:	May' 2019
Valid Up To	:	29/03/2023	Reference Standard Number	:	RS/2022-013
Effective Date	:	23/04/2022	Page Number	:	Page 4 of 4

Chemical Names:

i. **WCK 5107**: (2S,5R)-7-Oxo-6-sulphoxy-2-[N'-((R)-piperidin-3-carbonyl)-hydrazinocarbonyl]-1,6-diaza-bicyclo[3.2.1]octane dehydrate

ii. **Impurity-1**: (2S,5R)-5-sulphooxyamino-2-[N'-((R)-piperidin-3-carbonyl)-hydrazinocarbonyl]-piperidine

iii. **Impurity-2**: (2S,5R)-7-Oxo-6-hydroxy-2-[N'-((R)-piperidin-3-carbonyl)-hydrazinocarbonyl]-1,6-diaza-bicyclo[3.2.1]octane

iv. **Impurity-3**: (2S,5R)-7-Oxo-6-sulphooxy-2-[N'-((S)-piperidin-3-carbonyl)-hydrazinocarbonyl]-1,6-diaza-bicyclo[3.2.1]octane

v. **Impurity-4**: (2S,5R)-7-Oxo-6-sulphooxy-2-[N'-((R)-1-tert-butoxycarbonyl-piperidin-3-yl)-carbonyl)-hydrazinocarbonyl] -1,6-diaza-bicyclo[3.2.1]octane

	Prepared by	Checked by	Approved by
Name :	Mr. Sitaram Ubale	Mr. Rajendra Chavan	Mr Subhash Chandra Shukla
Department :	AVL	AVL	CRDQ
Sign :	[signature]	[signature]	[signature]
Date :	12/04/2022	12/04/2022	13/04/2022

Format No. : AVL-045-F-02-00

Ranked # 1 in Healthcare
Best Companies to Work for 2015 India

ANALYTICAL RESEARCH

CERTIFICATE OF ANALYSIS FOR REFERENCE STANDARD

**Name**	**:**	**Zidebactam (WCK 5107)**			
Molecular Formula	:	$C_{13}H_{21}N_5O_7S.2H_2O$	Batch Number	:	WK1/WP-0060/016
Molecular Weight	:	427.45	Laboratory Note Book Reference	:	WA-2004-038
Date of Analysis	:	30/03/2022	Month of Manufacturing	:	May' 2019
Valid Up To	:	29/03/2023	Reference Standard Number	:	RS/2022-013
Effective Date	:	23/04/2022	Page Number	:	Page 1 of 4

Sr. No.	Tests	Specifications	Results
1	Description	White to pale yellow powder	White powder
2	Identification (By IR, Mass & NMR Spectroscopy)	Spectral data should conform to the structure	Spectral data conforms the structure
3	Solubility	Freely soluble in water, Insoluble in dichloromethane	Freely soluble in water, Insoluble in dichloromethane
4	Constituted solution (10% w/v solution in WFI)<br>Completeness of solution<br><br>Clarity of solution	The solid dissolves completely, leaving no visible residue as un-dissolved matter<br>The constituted solution is not significantly less clear than an equal volume of the WFI contained in a similar vessel and examined similarly	Complies<br><br>Complies
5	pH of solution	Between 5.0 to 7.0	6.0
6	Absorbance of solution (1.25 % w/v in water at 420 nm)	Not more than 0.2 AU	0.01 AU
7	Water content (By KF Titration)	Not more than 12.0 % w/w	8.7 % w/w
8	Specific optical rotation (On anhydrous basis)	Between –28° to -40°	-36°
9	Heavy metals	Not more than 20 µg/g	Less than 20 µg/g
10	Palladium content (By ICP-OES)	Not more than 10 µg/g	BLQ (LOQ 2.5 µg/g)*

	Prepared by	Checked by	Approved by
Name :	Mr. Sitaram Ubale	Mr. Rajendra Chavan	Mr Subhash Chandra Shukla
Department :	AVL	AVL	CRDQ
Sign :	[signature]	[signature]	[signature]
Date :	12/04/2022	12/04/2022	13/04/2022

Format No. : AVL-045-F-02-00

Ranked # 1 in Healthcare
GREAT PLACE TO WORK Best Companies to Work for 2015 India

ANALYTICAL RESEARCH

CERTIFICATE OF ANALYSIS FOR REFERENCE STANDARD

Name	:	**Zidebactam (WCK 5107)**			
Molecular Formula	:	$C_{13}H_{21}N_5O_7S.2H_2O$	Batch Number	:	WK1/WP-0060/016
Molecular Weight	:	427.45	Laboratory Note Book Reference	:	WA-2004-038
Date of Analysis	:	30/03/2022	Month of Manufacturing	:	May' 2019
Valid Up To	:	29/03/2023	Reference Standard Number	:	RS/2022-013
Effective Date	:	23/04/2022	Page Number	:	Page 3 of 4

Sr. No.	Tests	Specifications	Results
13	Sodium content (By ICP-OES)	Not more than 0.5 % w/w	BLQ (LOQ 0.25 % w/w)*
14	Sulfate content (By Ion chromatography)	Not more than 1.0 % w/w	BLQ (LOQ 0.626 % w/w)
15	Mono-methyl sulphate content (By Ion chromatography)	Not more than 0.15% w/w	Not detected
16	Polymorphism (By X-ray Diffraction)	The diffractogram should exhibit characteristic 2 theta values at 13.8, 15.6, 20.3 and 24.3 (± 0.2°)	The diffractogram exhibits characteristic 2 theta values at 13.8, 15.6, 20.3 & 24.3
17	Potency (on as is basis)	Not applicable	90.4 %

BLQ = Below limit of quantification
BDL = Below disregard limit. Disregard limit 0.05 %
LOQ = Limit of quantification

* LOQ Values are based on analytical method verification report (Report nos. MVR/VP17053 and MVR/VP17054) of Analytical Solutions (CRO).

Specification Reference Number: **ZDB-DSS-112-05**

Remark: Potency is calculated on the basis of Total impurities, Water content, Residual solvents, Sulphate, Mono-methyl sulphate, Sodium and Palladium content.

Storage Condition: Store at 2°C to 8°C temperature.

	Prepared by	Checked by	Approved by
Name :	Mr. Sitaram Ubale	Mr. Rajendra Chavan	Mr Subhash Chandra Shukla
Department :	AVL	AVL	CRDQ
Sign :	[signature]	[signature]	[signature]
Date :	12/04/2022	12/04/2022	13/04/2022

Format No. : AVL-045-F-02-00

Ranked # 1 in Healthcare
GREAT PLACE TO WORK Best Companies to Work for 2015 India

WOCKHARDT | LIFE WINS

June 19,2022

PROFORMA INVOICE

International Air Waybill No	Date of Exportation: **June 19,2022**
**Shipper / Exporter:**<br>Sachin Bhagwat<br>Wockhardt Research Centre<br>D-4 MIDC Chikalthana<br>Aurangabad 431 210, M.S. INDIA<br>Tel : 91-240-6632222<br>Fax: 91-240-2485292	**Consignee Compounding:/Contact**<br>Test Materials Management (TMM)<br>ATTN: Bryan Parmentier, Anika Pippin, Lana Goehl ,KCAS Bioanalytical & Biomarker Services 12400 Shawnee Mission Parkway Shawnee, KS 66216, USA<br>913.248.3066 / 913-248-3000

No of Packs	Full Description of Goods		Total Value
01	Zidebactam (WCK 5107)	10 gm X 1 vial = 10 gm	10
	**Total Invoice Value**		**USD 10**

NON-HAZARDOUS COMPOUND
NOT FOR RE-SALE

MATERIAL IS EXTRACTED FROM chemicals DERIVED SUBSTANCE ONE TIME LAB TESTING ONLY ENTIRE AMOUNT TO BE CONSUMED IN TESTING

NON-INFECTIOUS/NON CONTAGIOUS, NO COMMERCIAL VALUE. NON TOXIC MATERIAL DOES NOT CONTAIN ANY ANIMAL OR CELL CULTURE DERIVETIVE PRODUCTS , NOT INFLAMMABLE NON-CORROSIVE, NON-DENGEROUS

For Wockhardt Research Centre

WOCKHARDT LTD. ★ WOCKHARDT ★ AURANGABAD

WOCKHARDT | LIFE WINS

June 19,2022

PROFORMA INVOICE

International Air Waybill No	Date of Exportation: June 19,2022
**Shipper / Exporter:**<br>Sachin Bhagwat<br>Wockhardt Research Centre<br>D-4 MIDC Chikalthana<br>Aurangabad 431 210, M.S. INDIA<br>Tel : 91-240-6632222<br>Fax: 91-240-2485292	**Consignee Compounding:/Contact**<br>Test Materials Management (TMM)<br>ATTN: Bryan Parmentier, Anika Pippin, Lana Goehl ,KCAS Bioanalytical & Biomarker Services 12400 Shawnee Mission Parkway Shawnee, KS 66216, USA<br>913.248.3066 / 913-248-3000

No of Packs	Full Description of Goods		Total Value
01	Zidebactam (WCK 5107)	10 gm X 1 vial = 10 gm	10
**Total Invoice Value**			**USD 10**

NON-HAZARDOUS COMPOUND
NOT FOR RE-SALE

MATERIAL IS EXTRACTED FROM chemicals DERIVED SUBSTANCE ONE TIME LAB TESTING ONLY ENTIRE AMOUNT TO BE CONSUMED IN TESTING

NON-INFECTIOUS/NON CONTAGIOUS, NO COMMERCIAL VALUE. NON TOXIC MATERIAL DOES NOT CONTAIN ANY ANIMAL OR CELL CULTURE DERIVETIVE PRODUCTS , NOT INFLAMMABLE NON-CORROSIVE, NON-DENGEROUS

For Wockhardt Research Centre

WOCKHARDT LTD. ★ AURANGABAD ★ WOCKHARDT

WOCKHARDT

Material Safety Data Sheet (MSDS)

==

MSDS of Zidebactam (WCK 5107)

SECTION 1: CHEMICAL PRODUCT IDENTIFICATION

SUBSTANCE: WCK 5107

INN: Zidebactam

CHEMICAL NAME: (2S, 5R)-7-Oxo-6-sulphooxy -2-[N'-((R)-piperidin-3-carbonyl)-hydrazinocarbonyl]-1,6-diaza-bicyclo[3.2.1]octane dihydrate

MOLECULAR FORMULA: $C_{13}H_{21}N_5O_7S.2H_2O$

MOLECULAR WEIGHT: 427.4

PERCENT COMPOSITION: C 39.89%, H 17.89%, N 5.41%

TRADE NAMES/SYNONYMS: Not available.

CAS NUMBER: 1996664-59-5

CHEMICAL FAMILY: 1,6-Diaza-Bicyclo Octane (DBO)

SUBSTANCE USE: As a beta-lactam enhancer.

SECTION 2 COMPOSITION, INFORMATION ON INGREDIENTS

COMPONENT: Single component.

EC NUMBER (EINECS): Not available

PERCENTAGE: >98%.

SECTION 3 HAZARDS IDENTIFICATION

MAJOR PHYSICAL AND HEALTH HAZARDS: May cause irritation to eyes, skin, and respiratory tract.

ROUTE OF ENTRY: May be absorbed via contact with skin or eyes, inhalation of aerosols or accidental ingestion. Normal use under supervision substance presents little hazards.

WOCKHARDT

SIGNS AND SYMPTOMS OF EXPOSURE: Acute signs and symptoms of exposure may include eye, skin and respiratory irritation.

CHEMICAL LISTED AS CARCINOGEN: NTP: IARC: OSHA listing: Not known.

MEDICAL CONDITIONS GENERALLY AGGRAVATED BY EXPOSURE: Not known.

SECTION 4 FIRST AID MEASURES

INHALATION: If difficulty with breathing, remove from exposure. Seek attention of physician immediately.

SKIN CONTACT: Remove contaminated clothing, wash skin with water and soap for 15 minutes. Seek medical attention if irritation or signs of exposure are noted.

EYE CONTACT: Flush eyes with large volumes of water for 15 minutes or more. Seek medical attention if irritation or signs of exposure are noted.

INGESTION: If ingestion occurs, flush mouth with water and seek medical attention if any symptoms appear.

INJECTION: In case of accidental injection, wash area and seek medical attention.

SECTION 5 FIRE FIGHTING MEASURES

FLASH POINT: Not applicable.

AUTOIGNITION TEMPERATURE: Not applicable.

FLAMABLE LIMITS IN AIR: Lower and Upper %: Not applicable

FLAMABLE LIMITS: Not applicable

EXTINGUISHING MEDIA: Use of water spray or multi purpose ABC extinguisher is recommended.

SPECIAL FIRE FIGHTING PRCEDURES: As with all fires, evacuate personnel to safe area. Fire fighters should wear self-contained breathing apparatus to avoid inhalation of smoke.

HAZARDOUS COMBUSTION PRODUCTS: Not available.

WOCKHARDT LTD. WOCKHARDT AURANGABAD

WOCKHARDT

UNUSAL FIRE/ EXPLOSION HAZARDS: None.

SECTION 6 ACCIDENTAL RELEASE MEASURES

SPILLS: Collect spillage maximum with suitable precautions such as using mask, safety goggles and gloves. Spry water and absorb liquid with suitable material and clean affected area with soap and water. Dispose of material according to the applicable laws and regulations.

RELESASE TO AIR: If aerosolized, reduce exposure by ventilating area. Wear respiratory protection as necessary.

RELEASE TO WATER: Refer to the local water authority. Drain disposal is not recommended.

SECTION 7 HANDLING AND STORAGE

GENERAL HANDLING: While handling, avoid all contacts and inhalation of dust, fumes, mist and/or vapors associated with the substance.

WASTE DISPOSAL METHOD: Incineration in an approved incinerator is recommended. Refer to the local rules and regulations.

STORAGE CONDITION: Store between 2- 8 deg Celsius.

SECTION 8 EXPOSURE CONTROLS, PERSONAL PROTECTION

Under normal use and handling conditions, no protective equipments are required. The following is recommended for production settings.

RESPIRATORY PROTECTION: Under normal use, respirator may not be required, if adequate ventilation is available. A dust/mist respirator may be required if aerosols are generated.

EYE PROTECTION: Chemical goggles or face shield is recommended, Safety goggles are always required.

VENTILATION: Handle the substance in well ventilated area.

CLOTHING: Handle the substance in work uniform or laboratory coat.

GLOVES: Use protective latex or nitrile gloves.

ADDITIONAL EXPOSURE PRECAUSIONS: Wash hands following use by

WOCKHARDT LTD. ★ WOCKHARDT ★ AURANGABAD

water and soap. Eating, drinking is not recommended while handling the substance.

SECTION 9 PHYSICAL AND CHEMICAL PROPERTIES

PHYSICAL STATE: Solid
COLOR: Pale yellow to white powder.

CHANGE IN APPEARANCE: No change in appearance upon exposure to atmosphere.
PHYSICAL FORM: Solid
ODOR: Odorless
MOLECULAR FORMULA: $C_{13}H_{21}N_3O_7S.2H_2O$
MOLECULAR WEIGHT: 427.4
BOILING POINT: Not available
MELTING POINT: >200° C
DECOMPOSITION POINT: Not available
VAPOR PRESSURE: Not available
VAPOR DENSITY: Not available
SPECIFIC GRAVITY: Not available
WATER SOLUBILITY: more than 200 mg in 1.0 ml water.
pH: 5-7 (0.5% in water)
VOLATILITY: Not applicable
SOLVENT SOLUBILITY: Soluble in water and in methanol; Insoluble in acetone.

SECTION 10 STABILITY AND REACTIVITY

REACTIVITY: Stable at normal temperature and pressure.

INCOMPATIBILITIES: Not identified

ACIDS (STRONG): Unstable

BASES (STRONG): Unstable

OXIDIZERS (STRONG): Unstable

HAZARDOUS DECOMPOSITION: May emit fumes when heated to decomposition.

POLYMERIZATION: will not occur.

SECTION 11 TOXICOLOGICAL INFORMATION

WOCKHARDT LTD. ★ AURANGABAD

Wockhardt

SIGNS AND SYMPTOMS OF EXPOSURE AND OVEREXPOSURE:
Immediate effect is eye, skin and respiratory irritation may occur.

ACUTE TOXICITY:

LD50 in rat (oral): >2000 mg/kg.
LD50 in mice (oral): >2000 mg/kg.

LD50 in rat (IV): >2000 mg/kg.
LD50 in mice (IV): >2000 mg/kg.

TARGET ORGANS: Not found.

MEDICAL CONDITIONS AGGRAVATED BY EXPOSURE: Not available

TUMORIGENIC DATA: Not available

MUTAGENIC DATA: Non-mutagenic

REPRODUCTIVE EFFECTS DATA: Not available

SECTION 12 ECOLOGICAL INFORMATION

ECOTOXICITY DATA: Not available
INVERTEBRATE TOXICITY: Not available

SECTION 13 DISPOSAL CONSIDERATIONS:

WASTE DISPOSAL: Dispose of any clean up material and waste residue according to all applicable laws and regulations. Incineration at approved, permitted facility is recommended.

SECTION 14 TRANSPORT INFORMATION

Information with regulatory organizations DOT, ICAO/IATA and IMO is not available

SECTION 15 REGULATORY INFORMATION

This section is not complete analysis or reference to all applicable regulatory information. Please consider all applicable laws and regulations for your country/state.

SECTION 16 OTHER INFORMATION

Disclaimer:

To the best of our knowledge, the information contained herein is accurate. However, neither Wockhardt nor any of its subsidiaries assume any liability whatsoever for the accuracy or completeness of the information contained herein. Final determination of suitability of any material is the sole

WOCKHARDT LTD. ★ WOCKHARDT ★ AURANGABAD

WOCKHARDT

responsibility of the user. All materials may present unknown hazards and should be used with caution. Although certain hazards are described herein, Wockhardt cannot guarantee that these are the only hazards that exist.

WOCKHARDT LTD. ★ AURANGABAD ★ WOCKHARDT
