# Supplementary material for: A Phase I, randomized, double-blind, placebo-controlled, dose escalation study to evaluate the safety, tolerability, and pharmacokinetics of intravenous ertapenem and zidebactam in combination (WCK 6777) in healthy adult subjects
Source: Antimicrob Agents Chemother. 2026 Sep 29;70(11):e00390-26. doi: 10.1128/aac.00390-26 (PMC13731237; doi:10.1128/aac.00390-26)
Supplement: Supplementary file 3 [file aac.00390-26-s0003.pdf]

1. TITLE: THE DETERMINATION OF TOTAL ZIDEBACTAM IN TREATED HUMAN PLASMA, WITH SODIUM HEPARIN AS ANTICOAGULANT, BY LC-MS/MS

BIOANALYTICAL METHOD VALIDATION REPORT

Bioanalytical Project Number:	V1872106P2
Sponsor:	DynPort Vaccine Company, LLC<br>110 Thomas Johnson Drive<br>Suite 200<br>Frederick, MD 21702
Bioanalytical Laboratory:	KCAS Bio<br>10830 S Clay Blair Boulevard<br>Olathe, KS 66061
Issue Date:	February 2023
Amendment 1 Issue Date:	March 2023
Amendment 2 Issue Date:	March 2023
Amendment 3 Issue Date:	Date of PI Signature

2. SYNOPSIS

This report presents the validation data for an LC-MS/MS method for the determination of total zidebactam in treated human plasma, with sodium heparin as anticoagulant. The assay was analyzed in the range of 0.0500 – 50.0 mcg/mL and is for use on human plasma samples obtained during clinical trials.

AMENDMENT OUTLINE FORM

1. Amendment Number 1	This amendment was created to report:<br>• Complete reissue of the original report to add more stability data per sponsor request.
2. Amendment Number 2	This amendment was created to report:<br>• Long-term matrix stability at -70 °C for up to 180 days for total zidebactam in treated human plasma, with sodium heparin as anticoagulant with and without the presence of comeds[2].
3. Amendment Number 3	This amendment was created to report:<br>• Long-term matrix stability at -70 °C for up to 49 days (without comeds[2]) and 50 days (with comeds[2]) for total zidebactam in untreated human plasma, with sodium heparin as anticoagulant with and without the presence of comeds[2].

3. TABLE OF CONTENTS FOR THE BIOANALYTICAL METHOD VALIDATION REPORT

1. TITLE: THE DETERMINATION OF TOTAL ZIDEBACTAM IN TREATED HUMAN PLASMA, WITH SODIUM HEPARIN AS ANTICOAGULANT, BY LC-MS/MS 1
2. SYNOPSIS 2
3. TABLE OF CONTENTS FOR THE BIOANALYTICAL METHOD VALIDATION REPORT 3
4. LIST OF TABLES 4
5. SIGNATURE PAGE 5
6. QUALITY ASSURANCE STATEMENT 6
7. STUDY COMPLIANCE STATEMENT 7
8. LIST OF ABBREVIATIONS AND DEFINITIONS OF TERMS 8
9. AMENDMENT 10
10. EXPERIMENTAL DESIGN AND RESULTS 10
10.1. Reference Standards 10
10.2. Calibration Standards and QC Samples Preparation 10
10.3. Carryover 11
10.4. Long-Term Matrix Stability at -20 °C and -70 °C - Untreated 11
10.5. SOP Deviations and Investigations 11
11. CONCLUSION 11
12. PERTINENT SOPS 11
13. ARCHIVES 12
14. REFERENCES 12
15. TABLES 13

4. LIST OF TABLES

Table 1: Abbreviations and Codes 8
Table 2: Mass Spec Transitions 13
Table 3: Reference Standards 13
Table 4: Calibration Standards and QC Samples Preparation 14
Table 5: Matrix Description 14
Table 6: Summary of Results and Conclusions 15
Table 7: Summary of Validation Run Objectives and Outcome 18
Table 8: Carryover 18
Table 9: Long-Term Stability at -70 °C - Untreated 19

5. SIGNATURE PAGE

Bioanalytical Project Number: V1872106P2

Amendment 3 Issue Date: Date of PI Signature

APPROVED

DocuSigned by:
Anika Pippin
Signer Name: Anika Pippin
Signing Reason: I approve this document
Signing Time: 28-Jun-2024 | 11:15 CDT
BE3AA1198E40459E8A7E3C615D981D82

Anika Pippin, B.S.
Principal Investigator

REVIEWED

DocuSigned by:
Marsha Luna
Signer Name: Marsha Luna
Signing Reason: I have reviewed this document
Signing Time: 28-Jun-2024 | 09:38 CDT
C9CC738561AF4CC1B375ED013DD1E4BD

Marsha Luna, B.S.
Management

6. QUALITY ASSURANCE STATEMENT

This Bioanalytical Method Validation Report has been found to describe accurately the methods and SOPs used and to accurately reflect the raw data collected. The Quality Assurance Unit initiated audits on this study and reported the findings to the Principal Investigator (PI) and Management as follows:

		**Date(s) Sent to:**	
**Inspection Date(s)**	**Inspection Phase**	**PI**	**Management**
21May2024	Data Audit	21May2024	18Jun2024
25Jun2024	Draft Report Audit	25Jun2024	25Jun2024
27Jun2024	Final Report Review	27Jun2024	27Jun2024

REVIEWED

DocuSigned by:
Jessica Lopez
Signer Name: Jessica Lopez
Signing Reason: I have reviewed this document
Signing Time: 28-Jun-2024 | 09:36 CDT
95C7FCBE477F4555996DA2F7A85A8F26

Jessica Lopez
Quality Assurance Unit

7. STUDY COMPLIANCE STATEMENT

I, the undersigned, hereby declare that this study was conducted in compliance with the relevant bioanalytical aspects of FDA Guidance for Industry on Bioanalytical Methods Validation, May 2018 [1] and KCAS Bio (KCAS) procedures and that the work was performed by me, or under my direction, and the report reflects a true and accurate record of the results obtained.

The acceptance criteria are based on the FDA Guidance for Industry, Bioanalytical Method Validation (2018). [1]

APPROVED

DocuSigned by:

Anika Pippin

Signer Name: Anika Pippin
Signing Reason: I approve this document
Signing Time: 28-Jun-2024 | 11:16 CDT
BE3AA1198E40459E8A7E3C615D981D82

Anika Pippin, B.S.
Principal Investigator

8. LIST OF ABBREVIATIONS AND DEFINITIONS OF TERMS

Table 1: Abbreviations and Codes

Abbreviation	Definition
<	Concentration less than the lowest acceptable calibration standard
>	Concentration exceeded the upper limit of calibration range (> ULOQ)
A	Amendment
A&P	Accuracy and Precision
AQL	Above Quantitation Limit
ARQC	Above Range Quality Control
Batch	Is the same as Run
BQL	Below Quantifiable Limit
Cal	Calibration standard
Conc.	Concentration
Conv.	Conversion
%CV	Coefficient of Variation
dev	Deviation
diff	Difference
Dil	Dilution
EMA	European Medicines Agency
FDA	Food and Drug Administration
GLP	Good Laboratory Practice
HPLC	High Performance Liquid Chromatography
ID	Identification
ICH	International Council for Harmonisation of Technical Requirements for Registration of Pharmaceuticals for Human Use
IS	Internal Standard
ISR	Incurred Sample Reanalysis
K_2EDTA or K_3EDTA	Dipotassium or Tripotassium Ethylenediaminetetraacetic Acid
KCAS	KCAS Bio
LC-MS/MS	Liquid Chromatography Tandem Mass Spectrometry
LLOQ	Lower Limit of Quantitation

Table 1: Abbreviations and Codes (Continued)

Abbreviation	Definition
mcg or µg or ug	Microgram
mcL or µL or uL	Microliter
ME	Matrix Effect
MF	Matrix Factor
mL	Milliliter
MS	Mass Spectrometry
n	number
N.C.	Not Calculated
ND	No difference reported as ISR will be counted
ng	nanogram
n.report	No Data available to report
N/A or NA	Not Applicable
N.R.	Not Reported
p.a.r.	Peak Area Ratio
PI	Principal Investigator
QC	Quality Control Sample
QCs	Quality Control Samples
OECD	Organization for Economic Co-operation and Development
RT	Room Temperature
sd or SD	Standard deviation
Seq	Sequence
SOP (s)	Standard Operating Procedure (s)
temp	Temperature
TP	Test procedure
UHPLC	Ultra High Performance Liquid Chromatography
ULOQ	Upper Limit of Quantitation
WAS	Working Analyte Standard
Watson LIMS 7.5	Watson Laboratory Information Management System, Version 7.5, Service Pack 1
WIS	Working Internal Standard

This is a basic list. Some of the abbreviations may not have been used in this report.

9. AMENDMENT

This amendment was created to long-term matrix stability at -70 °C for up to 49 days (without comeds[2]) and 50 days (with comeds[2]) for total zidebactam in untreated human plasma, with sodium heparin as anticoagulant with and without the presence of comeds[2].

The validation procedure was designed to meet or exceed the requirements of FDA Guidance for Industry on Bioanalytical Methods Validation, May 2018. The software programs used were Microsoft Office Excel 365, acquisition and processing software Analyst 1.7.2, and data management software Watson LIMS 7.5.

10. EXPERIMENTAL DESIGN AND RESULTS

In this method, zidebactam and internal standards, cefotaxime, were released from human plasma using protein precipitation. After extraction, the sample was chromatographed using reversed phase HPLC with a Fortis C18 analytical column. Zidebactam and cefotaxime (IS) were detected by monitoring the precursor and product ions described in Table 2 using an Applied Biosystems Sciex API 5000 or API 6500 LC-MS/MS. The quantification was based on setting a calibration graph using the internal standard method based on bioanalytical test procedure TP-80194, entitled "LC-MS/MS Analysis of Zidebactam in Treated Human Sodium Heparin Plasma (100 mcL, 0.0500 – 50.0 mcg/mL; Prior to MES Treatment)". The test procedure can be found in Annex 1 (V1872106P1 A1).

Human plasma, screened for the lack of interference at the retention times of ertapenem and cefotaxime (IS), was diluted 1:1 with MES buffer, pH 6.4 for stabilization. Since the standards and QCs concentrations were already adjusted for the addition of MES during the preparation, the obtained total zidebactam concentration of the unknown study samples do not need to be adjusted for the dilution with MES buffer.

A summary of the validation results is provided in Table 6. A summary describing the objective(s) and outcome of each validation run attempted is provided in Table 7.

The method was validated within the analytical range of 0.0500 – 50.0 mcg/mL in human plasma. No development work was performed after the method was validated.

10.1. Reference Standards

Reference standards used during this validation are presented in Table 3.

10.2. Calibration Standards and QC Samples Preparation

Calibration standards and quality control pools were prepared by spiking appropriate levels of zidebactam into human plasma, with sodium heparin as anticoagulant. Primary reference standards were prepared in at least duplicate and compared within 5% of each other. Standards and QCs were prepared and stored frozen at KCAS at -70 °C. Standards, QCs, and the critical

solutions used during this validation are described in Table 4, and matrix used is described in Table 5.

10.3. Carryover

Carryover is evaluated within each run. It consists of measuring the response for each analyte and if it exceeds 20.0% of the response of LLOQ standard or other sample reflecting the lower sensitivity limit required, a carryover assessment is completed, and affected samples are rejected. The internal standard carryover evaluation is assessed within each run and if it exceeds 5.0% of the average internal standard response of acceptable standards and QCs, impact is evaluated within the run. Data is presented in Table 8.

10.4. Long-Term Matrix Stability at -20 °C and -70 °C - Untreated

To evaluate zidebactam in untreated human plasma, with sodium heparin as anticoagulant, stability during sample storage, aliquots of the high and low concentration QC pools with and without the presence of comed[2] were stored frozen at -70 °C nominally for 49 days (without comeds[2]) and 50 days (with comeds[2]) prior to being analyzed, along with freshly prepared standards and QC samples. To be deemed stable, the mean %Bias at each level must be within ± 15.0% with %CV ≤ 15.0%.

Data will be provided in Table 9.

10.5. SOP Deviations and Investigations

No SOP deviations occurred. No investigations were conducted during this study.

11. CONCLUSION

Zidebactam is stable in untreated human plasma with and without the presence of comed[2], with sodium heparin as anticoagulant, for up to 49 days (without comeds[2]) and 50 days (with comeds[2]) upon storage in polypropylene tube at -70 °C.

12. PERTINENT SOPS

The following SOPs were followed during the conduct of this study:

SOP-00073 [05-07] - Bioanalytical Method Validation - Chromatographic Assays

SOP-00035 [02] - Procedures, Reporting, and Acceptance Criteria for Bioanalytical Chromatographic Projects

13. ARCHIVES

All raw data, including paper and electronic files associated with this study will remain archived at a location specified by KCAS, 10830 S Clay Blair Boulevard, Olathe, KS 66061 for a period of at least 10 years, after which the client may be contacted to arrange for continued storage of the files, or disposition according to an agreed procedure. KCAS will maintain an electronic (scanned/true) copy of the method validation report that can be viewed and printed on site. The study plan, documentation, raw data, and final report are indexed under Bioanalytical Project Number V1872106P2A3.

14. REFERENCES

1. Guidance for Industry: Bioanalytical Method Validation. U.S. Department of Health and Human Services. Food and Drug Administration. Center for Drug Evaluation and Research. Center for Veterinary Medicine. May 2018.
2. Comed: QCs in the presence of 150 mcg/mL ertapenem.

15. TABLES

Table 2: Mass Spec Transitions

Analyte	Transition
Zidebactam	m/z 392.028→312.000
Cefotaxime (IS)	m/z 456.200→324.000

Table 3: Reference Standards

Analyte	Supplier	Lot/Batch Number	Purity	Expiration or Retest Date	Conversion Factor	Storage Condition
Zidebactam	Wockhardt	WK1/WP-0060/016	90.40%	Expire 01Mar2024**	1.000	2-8 °C
Cefotaxime Sodium (IS)	Sigma-Aldrich	146942	95.9%*	Retest 18Oct2025	1.048*	2-8 °C
Ertapenem Sodium	MedChemExpress	229333	98.43%	Retest 29Sep2025	1.046	2-8 °C

Certificates of analysis for the above reference standards are on file at KCAS. *Internal standard reference material was used as is with 100% purity and 1.000 conversion factor. **Reference material was within retest at the time of use.

Table 4: Calibration Standards and QC Samples Preparation

Critical Solutions	Lot # for Standards (Prep Date)	Lot # for QCs (Prep Date)	Standards Lot (Prep Date)	QCS Lot (Prep Date)	Runs used
Treated Human Plasma (1:1 MES Buffer:Plasma)	BioIVT, Lot B1872106P2-13-1		B1872106P2-14-1 Set B (23Oct2023)	B1872106P2-15-1 Set B (23Oct2023)	2
Deionized water stored at -20°C in polypropylene	B1872106P2-5-1 (18Oct2023) B1872106P2-8-1 (18Oct2023)	B1872106P2-6-1 (18Oct2023) B1872106P2-12-1 (23Oct2023)			
Treated Human Plasma (1:1 MES Buffer:Plasma)	BioChemed, Lot V1872106P5A2-16-2		V1872106P2A3-10-1 Set A (25Jan2024)	V1872106P2A3-11-1 Set A (25Jan2024)	4
Deionized water stored at -20°C in polypropylene	V1872106P2A3-1-1 (05Dec2023) V1872106P2A3-8-1 (25Jan2024)	V1872106P2A3-2-1 (05Dec2023) V1872106P2A3-9-1 (25Jan2024)			
Treated Human Plasma (1:1 MES Buffer:Plasma)	BioChemed, Combo-223-1		N/A	Untreated QCs V1872106P2A3-7-1 (07Dec2023)	2, 4 (LTS)
Deionized water stored at -20°C in polypropylene	N/A	V1872106P2A3-2-1 (05Dec2023)			
Treated Human Plasma (1:1 MES Buffer:Plasma)	BioChemed, Combo-223-1		N/A	Untreated Comed QCs V1872106P5A2-11-1 (06Dec2023)	2, 4 (LTS)
Deionized water stored at -20°C in polypropylene	N/A	V1872106P2A3-1-1 (05Dec2023)			
Ertapenem in chilled ~0.1M MES buffer, pH 6.4 stored at -20 °C in polypropylene	N/A	V1872106P5A2-1-1 (05Dec2023)			

Table 5: Matrix Description

Matrix	Source Lot #	Date Received
Sodium Heparin Human Plasma B1872106P2-13-1	BioIVT, Lot HMN795697	Apr2022
Sodium Heparin Human Plasma V1872106P5A2-16-2 (Combo-223-1)	BioChemed, Lots BC46306NH-1, BC46306NH-2, BC46306NH-4, BC46306NH-6	Oct2023

Table 6: Summary of Results and Conclusions

**Methodology – TP-80194**	**Method Performance – LC-MS/MS**	**Hyperlink – N/A**	**Comments**
Matrix	Plasma		
Anticoagulant	Sodium heparin		
Assay Volume	100 mcL		
**Selectivity**			
Analyte	Zidebactam		V1872106P2
Internal Standard	Cefotaxime		V1872106P2
Matrix Effect<br>(6 spiked matrix lots)	Complies		V1872106P2
Interference	Complies		V1872106P2
Hemolytic Effect Evaluation	Complies		V1872106P2
Lipemic Effect Evaluation	Complies		V1872106P2
Carryover	Assessed with every run with standards and QCs	Summary tables<br>Table 8<br><br>Report text<br>Section 10.3	
**Linearity**			
Weighting	$1/x^2$		V1872106P2
Bias at LLOQ	-3.6%		V1872106P2
Bias above LLOQ	-9.4 to 9.0%		V1872106P2
Bioanalytical Range	Complies<br>0.0500 – 50.0 mcg/mL		V1872106P2
**Precision**			
Intra-assay	0.6 to 8.9% (LLOQ);<br>0.9 to 3.4% (above LLOQ)		V1872106P2
Inter-assay	10.1% (LLOQ);<br>3.5 to 4.8% (above LLOQ)		V1872106P2
**Accuracy**			
Intra-assay	-9.6 to 11.4% (LLOQ);<br>-9.1 to 12.0% (above LLOQ)		V1872106P2
Inter-assay	-2.0% (LLOQ);<br>-5.1 to 6.0% (above LLOQ)		V1872106P2
**Large Run Size**	Determined to be 125 samples.		V1872106P2

Table 6: Summary of Results and Conclusions (Continued)

Methodology – TP-80194	Method Performance – LC-MS/MS	Hyperlink – N/A	Comments
**Dilution**			
Dilution factor: 2x	Dilution QC: 37.5 mcg/mL<br>Precision: 1.4%<br>Accuracy: 1.9%		V1872106P2
Dilution factor: 10x	Dilution QC: 400 mcg/mL<br>Precision: 1.3%<br>Accuracy: -2.8%		V1872106P2
**Stability**			
Matrix (in polypropylene tubes)			
Freeze/Thaw (-20 °C/ice-water bath) with and without the presence of comed[2]	Demonstrated for 4 cycles for treated QCs		V1872106P2
Freeze/Thaw (-70 °C/ice-water bath) with and without the presence of comed[2]	Demonstrated for 4 cycles for treated QCs<br>Demonstrated for 2 cycles for untreated QCs		V1872106P2 and V1872106P2A1
Bench-top in wet ice without the presence of comed[2]	Demonstrated for 19.75 hours for treated QCs<br>Demonstrated for 4 hours for untreated QCs		V1872106P2 and V1872106P2A1
Bench-top in wet ice with the presence of comed[2]	Demonstrated for 8.25 hours for treated QCs<br>Demonstrated for 4 hours for untreated QCs		V1872106P2 and V1872106P2A1
Long-term (-20 °C) with and without the presence of comed[2]	Demonstrated instability at 62 days		V1872106P2
Long-term (-70 °C) with and without the presence of comed[2]	Demonstrated for 90 days for treated QCs<br>Demonstrated for 27 days for untreated QCs without comed[2]<br>Demonstrated for 35 days for untreated QCs with comed[2]		V1872106P2 and V1872106P2A1
Long-term (-70 °C) with and without the presence of comed[2]	Demonstrated for 180 days for treated QCs with and without comed[2]		V1872106P2A2

Table 6: Summary of Results and Conclusions (Continued)

Methodology – TP-80194	Method Performance – LC-MS/MS	Hyperlink – N/A	Comments
Long-term (-70 °C) with and without the presence of comed[2]	Demonstrated for 49 days for untreated QCs without comed[2]<br>Demonstrated for 50 days for untreated QCs with comed[2]	Summary tables Table 9<br>Report text Section 10.4	
Whole Blood	No instability observed in ice-water bath or at room temperature in polyethylene terephthalate for up to 1.5 hour.		V1872106P2
Processed Matrix polypropylene			
Room Temperature	Demonstrated for 3 days		V1872106P2
Refrigeration	Demonstrated for 3 days		V1872106P2
**Individual Sample Reinjection**	Reproducible		V1872106P2
**Whole Run Reinjection Integrity**	Reproducible<br>Duration 2 days 7 hours		V1872106P2
**Column Ruggedness**	Reproducible		V1872106P2
**Extraction Recovery**			
Zidebactam	Overall 95.1%		V1872106P2
Cefotaxime (IS)	Overall 99.0%		V1872106P2
**Solution Stability**			
Zidebactam 1° in deionized water (RT) - polypropylene	Stable 17.75 hours		V1872106U2 and V1872106P2
Zidebactam 1° in deionized water (-20 °C) - polypropylene	Stable 61 days		V1872106U2
Zidebactam Spike in deionized water (RT) - polypropylene	Stable 17.75 hours		V1872106U2 and V1872106P2
Zidebactam Spike in deionized water (-20 °C) - polypropylene	Stable 61 days		V1872106U2
Cefotaxime (IS) in deionized water in glass (RT) - polypropylene	Stable 15.5 hours<br>Stable 19.0 hours		V1872106U1 and V1872106P2A1
Cefotaxime (IS) in deionized water in glass (-20°C) - polypropylene	3 months stability can be assigned per SOP unless low sensitivity is observed		SOP-00073

Table 7: Summary of Validation Run Objectives and Outcome

Run ID	Instrument ID	Extraction Date	Assay Date	Run Objective(s)	Accepted or Rejected	Comment
2	LC/MS-22	07Dec2023	11Dec2023	Cal, QC, and untreated QC with and without comed[2] qualification	Accepted	This was used as Time 0 for long-term stability.
4	LC/MS-44	25Jan2024	25Jan2024	Untreated long-term matrix stability with and without comed[2]	Accepted	

Run 1 was used for primary comparison and spike linearity check and is not reported.
Run 3 was created to reinject run 1, but this was not done, and the run was not used. It is not reported.

Table 8: Carryover

Run ID	Result (Extracted Blank after High Standard)	Carryover Results for Internal Standard
2	Seq 13 and Seq 56 are <20.0% of the LLOQ.	Seq 4 and Seq 47 are < 5.0% internal standard peak of acceptable standards.
4	Seq 13 and Seq 56 are <20.0% of the LLOQ.	Seq 4 and Seq 47 are < 5.0% internal standard peak of acceptable standards.

Note: Calibration Standards and QCs are arranged in a way to mitigate carryover in all validation runs.

Table 9: Long-Term Stability at -70 °C - Untreated

Analytical Performance: Back-Calculated Concentrations (µg/mL) of Zidebactam Calibration Standard in (Human) (Plasma) in (Study V1872106P2A3)

Assay Date	Run ID	Cal 0.0500 0.0500 µg/mL	Cal 0.100 0.100 µg/mL	Cal 1.00 1.00 µg/mL	Cal 5.00 5.00 µg/mL	Cal 10.0 10.0 µg/mL	Cal 20.0 20.0 µg/mL	Cal 40.0 40.0 µg/mL	Cal 50.0 50.0 µg/mL
11-Dec-2023	2	0.0502	0.101	1.01	5.22	10.1	19.9	39.6	48.3
		0.0484	0.105	1.00	5.21	9.70	20.3	39.4	47.3
25-Jan-2024	4	0.0424	0.0947	0.880	4.35	9.95	18.4	35.3	50.1
		0.0562	0.112	1.01	5.65	10.4	20.6	43.7	55.6
Mean		0.0493	0.103	0.975	5.11	10.0	19.8	39.5	50.3
S.D.		0.00568	0.00725	0.0635	0.545	0.293	0.976	3.43	3.70
%CV		11.5	7.0	6.5	10.7	2.9	4.9	8.7	7.4
%Bias		-1.4	3.0	-2.5	2.2	0.0	-1.0	-1.3	0.6
n		4	4	4	4	4	4	4	4

Calibration Curve Parameters for Zidebactam Calibration Standards in Human Plasma (Study V1872106P2A3)

Run Date	Run ID	Slope	Intercept	R-Squared	LLOQ	ULOQ
11-Dec-2023	2	0.10651	-0.00053	0.9989	0.0500	50.0
25-Jan-2024	4	0.01094	0.00001	0.9879	0.0500	50.0

Human Plasma Zidebactam Concentrations in Quality Controls for 2 separate batch runs (Study V1872106P2A3)

Run Date	Run ID	QCS 0.150 0.150 µg/mL	%Bias	QCS 25.0 25.0 µg/mL	%Bias	QCS 37.5 37.5 µg/mL	%Bias
11-Dec-2023	2	0.148	-1.3	24.7	-1.2	35.7	-4.8
		0.153	2.0	23.7	-5.2	35.9	-4.3
Intrarun Mean		0.151		24.2		35.8	
Intrarun %Bias		0.7		-3.2		-4.5	
25-Jan-2024	4	0.131	-12.7	26.5	6.0	37.4	-0.3
		0.142	-5.3	23.6	-5.6	37.0	-1.3
Intrarun Mean		0.137		25.1		37.2	
Intrarun %Bias		-8.7		0.4		-0.8	

Table 9: Long-Term Stability at -70 °C – Untreated (Continued)

Untreated without comed[2]

Run Date	Run ID	QC 0.150 Untreated 0.150 µg/mL	%Bias	QC 37.5 Untreated 37.5 µg/mL	%Bias
11-Dec-2023	2	0.163	8.7	37.6	0.3
(Time 0)		0.167	11.3	38.0	1.3
		0.156	4.0	37.5	0.0
		0.159	6.0	37.0	-1.3
		0.153	2.0	38.0	1.3
		0.154	2.7	38.2	1.9
Intrarun Mean		0.159		37.7	
Intrarun SD		0.00547		0.440	
Intrarun %CV		3.4		1.2	
Intrarun %Bias		6.0		0.5	
n		6		6	
25-Jan-2024	4	0.137	-8.7	38.8	3.5
(49 days)		0.161	7.3	36.3	-3.2
		0.147	-2.0	36.3	-3.2
		0.149	-0.7	39.9	6.4
		0.159	6.0	36.9	-1.6
		0.150	0.0	38.9	3.7
Intrarun Mean		0.151		37.9	
Intrarun SD		0.00871		1.54	
Intrarun %CV		5.8		4.1	
Intrarun %Bias		0.7		1.1	
n		6		6	

Table 9: Long-Term Stability at -70 °C – Untreated (Continued)

Untreated with comed[2]

Run Date	Run ID	QC 0.150 Comed Untr 0.150 µg/mL	%Bias	QC 37.5 Comed Untr 37.5 µg/mL	%Bias
11-Dec-2023	2	0.162	8.0	36.7	-2.1
(Time 0)		0.163	8.7	37.6	0.3
		0.158	5.3	36.3	-3.2
		0.164	9.3	37.6	0.3
		0.160	6.7	36.9	-1.6
		0.152	1.3	37.2	-0.8
Intrarun Mean		0.160		37.1	
Intrarun SD		0.00440		0.517	
Intrarun %CV		2.8		1.4	
Intrarun %Bias		6.7		-1.1	
n		6		6	
25-Jan-2024	4	0.130	-13.3	37.5	0.0
(50 days)		0.156	4.0	36.4	-2.9
		0.147	-2.0	33.2	-11.5
		0.139	-7.3	36.8	-1.9
		0.157	4.7	35.0	-6.7
		0.162	8.0	39.7	5.9
Intrarun Mean		0.149		36.4	
Intrarun SD		0.0122		2.21	
Intrarun %CV		8.2		6.1	
Intrarun %Bias		-0.7		-2.9	
n		6		6	

Criteria: See Section 10.4.

Results: Zidebactam is stable in untreated human plasma with and without the presence of comed[2], with sodium heparin as anticoagulant, for up to 49 days (without comeds[2]) and 50 days (with comeds[2]) upon storage in polypropylene tube at -70 °C.

1. TITLE: THE DETERMINATION OF TOTAL ZIDEBACTAM IN TREATED HUMAN PLASMA, WITH SODIUM HEPARIN AS ANTICOAGULANT, BY LC-MS/MS

BIOANALYTICAL METHOD VALIDATION REPORT

Bioanalytical Project Number:	V1872106P2
Sponsor:	DynPort Vaccine Company, LLC<br>110 Thomas Johnson Drive<br>Suite 200<br>Frederick, MD 21702
Bioanalytical Laboratory:	KCAS Bioanalytical & Biomarker Services.<br>10830 S Clay Blair Boulevard<br>Olathe, KS 66061
Issue Date:	February 2023
Amendment 1 Issue Date:	March 2023
Amendment 2 Issue Date:	March 2023

2. SYNOPSIS

This report presents the validation data for an LC-MS/MS method for the determination of total zidebactam in treated human plasma, with sodium heparin as anticoagulant. The assay was analyzed in the range of 0.0500 – 50.0 mcg/mL and is for use on human plasma samples obtained during clinical trials.

AMENDMENT OUTLINE FORM

1. Amendment Number 1	March 2023 - This amendment was created to report:<br>• Complete reissue of the original report to add more stability data per sponsor request.
2. Amendment Number 2	March 2023 - This amendment was created to report:<br>• Long-term matrix stability at -70 °C for up to 180 days for total zidebactam in treated human plasma, with sodium heparin as anticoagulant with and without the presence of comeds[2].

3. TABLE OF CONTENTS FOR THE BIOANALYTICAL METHOD VALIDATION REPORT

1. TITLE: THE DETERMINATION OF TOTAL ZIDEBACTAM IN TREATED HUMAN PLASMA, WITH SODIUM HEPARIN AS ANTICOAGULANT, BY LC-MS/MS 1
2. SYNOPSIS 2
3. TABLE OF CONTENTS FOR THE BIOANALYTICAL METHOD VALIDATION REPORT 3
4. LIST OF TABLES 4
5. SIGNATURE PAGE 5
6. QUALITY ASSURANCE STATEMENT 6
7. STUDY COMPLIANCE STATEMENT 7
8. LIST OF ABBREVIATIONS AND DEFINITIONS OF TERMS 8
9. AMENDMENT 10
10. EXPERIMENTAL DESIGN AND RESULTS 10
10.1. Reference Standards 10
10.2. Calibration Standards and QC Samples Preparation 10
10.2.1. Carryover 11
10.3. Long-Term Matrix Stability at -20 °C and -70 °C - Treated 11
10.4. SOP Deviations and Investigations 11
11. CONCLUSION 11
12. PERTINENT SOPS 12
13. ARCHIVES 12
14. REFERENCES 12
15. TABLES 13

4. LIST OF TABLES

Table 1: Abbreviations and Codes 8
Table 2: Mass Spec Transitions 13
Table 3: Reference Standards 13
Table 4: Calibration Standards and QC Samples Preparation 14
Table 5: Matrix Description 14
Table 6: Summary of Results and Conclusions 15
Table 7: Summary of Validation Run Objectives and Outcome 17
Table 8: Carryover 17
Table 9: Long-Term Stability at -70 °C - Treated 18

5. SIGNATURE PAGE

Bioanalytical Project Number: V1872106P2

Amendment 2 Issue Date: March 2023

APPROVED

DocuSigned by:
Anika Pippin
Signer Name: Anika Pippin
Signing Reason: I approve this document
Signing Time: 21-Mar-2023 | 13:50 CDT
BE3AA1198E40459E8A7E3C615D981D82

Anika Pippin, B.S.
Principal Investigator

REVIEWED

DocuSigned by:
Marsha Luna
Signer Name: Marsha Luna
Signing Reason: I have reviewed this document
Signing Time: 21-Mar-2023 | 13:40 CDT
C9CC738561AF4CC1B375ED013DD1E4BD

Marsha Luna, B.S.
Management

6. QUALITY ASSURANCE STATEMENT

This Bioanalytical Method Validation Report has been found to describe accurately the methods and SOPs used and to accurately reflect the raw data collected. The Quality Assurance Unit initiated audits on this study and reported the findings to the Principal Investigator (PI) and Management as follows:

		Date(s) Sent to:	
**Inspection Date(s)**	**Inspection Phase**	**PI**	**Management**
17Mar2023	Data Audit	17Mar2023	17Mar2023
21Mar2023	Draft Report Review	21Mar2023	21Mar2023
21Mar2023	Final Report Review	21Mar2023	21Mar2023

REVIEWED

DocuSigned by:
Connie Anderson
Signer Name: Connie Anderson
Signing Reason: I have reviewed this document
Signing Time: 21-Mar-2023 | 13:38 CDT
9429F81BF6DE47B3A3DEEE0F97D96287

Connie Anderson, B.S.
Quality Assurance Unit

7. STUDY COMPLIANCE STATEMENT

I, the undersigned, hereby declare that this study was conducted in compliance with the relevant bioanalytical aspects of FDA guidelines, GLP documentation and reporting GLP regulations 21CFR Part 58 and KCAS Bioanalytical & Biomarker Services (KCAS) procedures and that the work was performed by me, or under my direction, and the report reflects a true and accurate record of the results obtained.

The acceptance criteria are based on the FDA Guidance for Industry, Bioanalytical Method Validation (2018). [1]

APPROVED

DocuSigned by:

Anika Pippin

Signer Name: Anika Pippin
Signing Reason: I approve this document
Signing Time: 21-Mar-2023 | 13:50 CDT
BE3AA1198E40459E8A7E3C615D981D82

Anika Pippin, B.S.
Principal Investigator

8. LIST OF ABBREVIATIONS AND DEFINITIONS OF TERMS

Table 1: Abbreviations and Codes

Abbreviation	Definition
<	Concentration less than the lowest acceptable calibration standard
>	Concentration exceeded the upper limit of calibration range (> ULOQ)
A	Amendment
AQL	Above Quantitation Limit
%Bias	Percentage measurement valid
Batch	Is the same as Run
BQL	Below Quantifiable Limit
Cal	Calibration standard
Conc.	Concentration
Conv.	Conversion
%CV	Coefficient of Variation
dev	Deviation
diff	Difference
EMA	European Medicines Agency
FDA	Food and Drug Administration
F/T	Freeze/Thaw
GLP	Good Laboratory Practice
HPLC	High Performance Liquid Chromatography
ID	Identification
IS	Internal Standard
ISR	Incurred Sample Reanalysis
K_2EDTA or K_3EDTA	Dipotassium or Tripotassium Ethylenediaminetetraacetic Acid
KCAS	KCAS Bioanalytical & Biomarker Services
LC-MS/MS	Liquid Chromatography Tandem Mass Spectrometry
LLOQ	Lower Limit of Quantitation
mcg or μg or ug	Microgram
mcL or μL or uL	Microliter

Table 1: Abbreviations and Codes (Continued)

Abbreviation	Definition
ME	Matrix Effect
MF	Matrix Factor
MHLW	Ministry of Health, Labour and Welfare of Japan
mL	Milliliter
MS	Mass Spectrometry
n	number
N.C.	Not Calculated
ND	No difference reported as ISR will be counted
ng	nanogram
n.report	No Data available to report
N/A or NA	Not Applicable
N.R.	Not Reported
OECD	Organization for Economic Co-operation and Development
P&A	Precision and Accuracy
p.a.r.	Peak Area Ratio
PI	Principal Investigator
QC	Quality Control Sample
QCs	Quality Control Samples
RT	Room Temperature
sd or SD	Standard deviation
Seq	Sequence
SOP (s)	Standard Operating Procedure (s)
temp	Temperature
TP	Test procedure
UHPLC	Ultra High Performance Liquid Chromatography
ULOQ	Upper Limit of Quantitation
WIS	Working Internal Standard

This is a basic list. Some of the abbreviations may not have been used in this report.

9. AMENDMENT

This amendment was created to report long-term matrix stability at -70 °C for up to 180 days for total zidebactam in treated human plasma, with sodium heparin as anticoagulant with and without the presence of comeds[2].

The validation procedure was designed to meet or exceed the requirements of FDA Guidance for Industry on Bioanalytical Methods Validation, May 2018. The software programs used were Microsoft Office Excel 365, acquisition and processing software Analyst 1.7.2, and data management software Watson LIMS 7.5.

10. EXPERIMENTAL DESIGN AND RESULTS

In this method, zidebactam and internal standards, cefotaxime, were released from human plasma using protein precipitation. After extraction, the sample was chromatographed using reversed phase HPLC with a Fortis C18 analytical column. Zidebactam and cefotaxime (IS) were detected by monitoring the precursor and product ions described in Table 2 using an Applied Biosystems Sciex API 5000 LC-MS/MS. The quantification was based on setting a calibration graph using the internal standard method based on bioanalytical test procedure TP-80194, entitled “LC-MS/MS Analysis of Zidebactam in Treated Human Sodium Heparin Plasma (100 mcL, 0.0500 – 50.0 mcg/mL; Prior to MES Treatment)”. The test procedure can be found in Annex 1 (V1872106P1 A1).

Human plasma, screened for the lack of interference at the retention times of ertapenem and cefotaxime (IS), was diluted 1:1 with MES buffer, pH 6.4 for stabilization. Since the standards and QCs concentrations were already adjusted for the addition of MES during the preparation, the obtained total zidebactam concentration of the unknown study samples do not need to be adjusted for the dilution with MES buffer.

A summary of the validation results is provided in Table 6. A summary describing the objective(s) and outcome of each validation run attempted is provided in Table 7.

The method was validated within the analytical range of 0.0500 – 50.0 mcg/mL in human plasma. No development work was performed after the method was validated.

10.1. Reference Standards

Reference standards used during this validation are presented in Table 3.

10.2. Calibration Standards and QC Samples Preparation

Calibration standards and quality control pools were prepared by spiking appropriate levels of zidebactam into human plasma, with sodium heparin as anticoagulant. Primary reference standards were prepared in at least duplicate and compared within 5% of each other. Standards and QCs were prepared and stored frozen at KCAS at -70 °C. Standards, QCs, and the critical

solutions used during this validation are described in Table 4, and matrix used is described in Table 5.

10.2.1. Carryover

Carryover is evaluated within each run. It consists of measuring the response for each analyte and if it exceeds 20% of the response of LLOQ standard or other sample reflecting the lower sensitivity limit required, a carryover assessment is completed, and affected samples are rejected. The internal standard carryover evaluation is assessed within each run and if it exceeds 5% of the average internal standard response of acceptable standards and QCs, impact is evaluated within the run. Data is presented in Table 8.

10.3. Long-Term Matrix Stability at -20 °C and -70 °C - Treated

To evaluate zidebactam in treated human plasma, with sodium heparin as anticoagulant, stability during sample storage, aliquots of the high and low concentration QC pools with and without the presence of comed[2] were stored frozen at -70 °C nominally for 180 days prior to being analyzed, along with freshly prepared standards and QC samples. To be deemed stable, the mean %Bias at each level must be within ± 15% with %CV ≤ 15%.

Zidebactam is stable in treated human plasma with and without the presence of comed[2], with sodium heparin as anticoagulant, for up to 180 days upon storage in polypropylene tube at -70 °C.

Data will be provided in Table 9.

10.4. SOP Deviations and Investigations

No SOP deviations occurred. No investigations were conducted during this study.

11. CONCLUSION

Zidebactam is stable in treated human plasma with and without the presence of comed[2], with sodium heparin as anticoagulant, for up to 180 days upon storage in polypropylene tube at -70 °C.

12. PERTINENT SOPS

The following SOPs were followed during the conduct of this study:

SOP-00073 [04] - Bioanalytical Method Validation - Chromatographic Assays

SOP-00035 [02] - Procedures, Reporting, and Acceptance Criteria for Bioanalytical Chromatographic Projects

13. ARCHIVES

All raw data, including paper and electronic files associated with this study will remain archived at a location specified by KCAS, 10830 S Clay Blair Boulevard, Olathe, KS 66061 for a period of at least 10 years, after which the client may be contacted to arrange for continued storage of the files, or disposition according to an agreed procedure. KCAS will maintain an electronic (scanned/true) copy of the method validation report that can be viewed and printed on site. The study plan, documentation, raw data, and final report are indexed under Bioanalytical Project Number V1872106P2A2.

14. REFERENCES

1. Guidance for Industry: Bioanalytical Method Validation. U.S. Department of Health and Human Services. Food and Drug Administration. Center for Drug Evaluation and Research. Center for Veterinary Medicine. May 2018.
2. Comed: QCs in the presence of 150 mcg/mL ertapenem.

15. TABLES

Table 2: Mass Spec Transitions

Analyte	Transition
Zidebactam	m/z 392.028→312.000
Cefotaxime (IS)	m/z 456.200→324.000

Table 3: Reference Standards

Analyte	Supplier	Lot/Batch Number	Purity	Expiration Date or Retest Date	Conversion Factor
Zidebactam	Wockhardt	WK1/WP-0060/016	90.4%	Expire 29 Mar 2023	1.000
Cefotaxime Sodium Salt (IS)	Sigma Aldrich	0000146942	95.9%	Retest 30 Sep 2023	1.048
Ertapenem Sodium	Med Chem Express	155576	98.9%	Retest 20 Jun 2024	1.046

Certificates of analysis for the above reference standards are presented in Annex 2 (V1872106P2 A1).

Table 4: Calibration Standards and QC Samples Preparation

<table>
<tr><th>Critical Solutions</th><th>Lot # for Standards (Prep Date)</th><th>Lot # for QCs (Prep Date)</th><th>Standards Lot (Prep Date)</th><th>QCS Lot (Prep Date)</th><th>Runs used</th></tr>
<tr><td>Treated Human Sodium Heparin Plasma (1:1 MES buffer:plasma)</td><td colspan="2">V1872106P2-56-1</td><td rowspan="2">NA</td><td rowspan="2">Comed QCs
V1872106P2-58-1
Set F
(08Sep2022)</td><td rowspan="2">1 (LTS)</td></tr>
<tr><td>Deionized Water stored at -20°C in polypropylene</td><td>NA</td><td>V1872106P2-41-1
(01Sep2022)
V1872106P2-51-1
(06Sep2022)
V1872106P2-53-1
(07Sep2022)</td></tr>
<tr><td>Treated Human Sodium Heparin Plasma (1:1 MES buffer:plasma)</td><td colspan="2">V1872106P2-56-1</td><td rowspan="2">NA</td><td rowspan="2">V1872106P2-57-1
Set F
(08Sep2022)</td><td rowspan="2">1 (LTS)</td></tr>
<tr><td>Deionized Water stored at -20°C in polypropylene</td><td>NA</td><td>V1872106P2-41-1
(01Sep2022)
V1872106P2-51-1
(06Sep2022)</td></tr>
<tr><td>Treated Human Sodium Heparin Plasma (1:1 MES buffer:plasma)</td><td colspan="2">V1872106P2-117-1</td><td rowspan="2">V1872106P2A2-1-1
Set A
(07 Mar 2023)</td><td rowspan="2">V1872106P2A2-2-1
Set A
(07 Mar 2023)</td><td rowspan="2">1</td></tr>
<tr><td>Deionized Water stored at -20°C in polypropylene</td><td>V1872106P2-120-1
(02Feb2023)
V1872106P2-122-1
(02Feb2023)</td><td>V1872106P2-121-1
(02Feb2023)
V1872106P2-123-1
(02Feb2023)</td></tr>
</table>

Table 5: Matrix Description

Matrix	Source Lot #	Date Received
Treated Human Sodium Heparin Plasma (1:1 MES Buffer:Plasma)<br>V1872106P2-56-1	BioIVT<br>Combo-43-1<br>(HMN769588, HMN769589, HMN769590, HMN769592, HMN769593, HMN769594, HMN769595, HMN769596, HMN769597, HMN769598, HMN769600, HMN769601, HMN769602, HMN769604, HMN769605)	Mar2022
Treated Human Sodium Heparin Plasma (1:1 MES Buffer:Plasma)<br>V1872106P2-117-1	BioIVT, Lot HMN444012	Aug2020

Table 6: Summary of Results and Conclusions

**Methodology – TP-80194**	**Method Performance – LC-MS/MS**	**Hyperlink – N/A**	**Comments**
Matrix	Plasma		
Anticoagulant	Sodium heparin		
Assay Volume	100 mcL		
**Selectivity**			
Analyte	Zidebactam		V1872106P2
Internal Standard	Cefotaxime		V1872106P2
Matrix Effect (6 spiked matrix lots)	Complies		V1872106P2
Interference	Complies		V1872106P2
Hemolytic Effect Evaluation	Complies		V1872106P2
Lipemic Effect Evaluation	Complies		V1872106P2
Carryover	Assessed with every run with standards and QCs	Summary tables Table 8<br><br>Report text Section 10.2.1	V1872106P2
**Linearity**			
Weighting	$1/x^2$		V1872106P2
Bias at LLOQ	-3.6%		V1872106P2
Bias above LLOQ	-9.4 to 9.0%		V1872106P2
Bioanalytical Range	Complies<br>0.0500 – 50.0 mcg/mL		V1872106P2
**Precision**			
Intra-assay	0.6 to 8.9% (LLOQ);<br>0.9 to 3.4% (above LLOQ)		V1872106P2
Inter-assay	10.1% (LLOQ);<br>3.5 to 4.8% (above LLOQ)		V1872106P2
**Accuracy**			
Intra-assay	-9.6 to 11.4% (LLOQ);<br>-9.1 to 12.0% (above LLOQ)		V1872106P2
Inter-assay	-2.0% (LLOQ);<br>-5.1 to 6.0% (above LLOQ)		V1872106P2
**Large Run Size**	Determined to be 125 samples.		V1872106P2

Table 6: Summary of Results and Conclusions (Continued)

**Methodology – TP-80194**	**Method Performance – LC-MS/MS**	**Hyperlink – N/A**	**Comments**
**Dilution**			
Dilution factor: 2x	Dilution QC: 37.5 mcg/mL<br>Precision: 1.4%<br>Accuracy: 1.9%		V1872106P2
Dilution factor: 10x	Dilution QC: 400 mcg/mL<br>Precision: 1.3%<br>Accuracy: -2.8%		V1872106P2
**Stability**			
Matrix (in polypropylene tubes)			
Freeze/Thaw (-20 °C/ice-water bath) with and without the presence of comed[2]	Demonstrated for 4 cycles for treated QCs		V1872106P2
Freeze/Thaw (-70 °C/ice-water bath) with and without the presence of comed[2]	Demonstrated for 4 cycles for treated QCs<br>Demonstrated for 2 cycles for untreated QCs		V1872106P2 and V1872106P2A1
Bench-top in wet ice without the presence of comed[2]	Demonstrated for 19.75 hours for treated QCs<br>Demonstrated for 4 hours for untreated QCs		V1872106P2 and V1872106P2A1
Bench-top in wet ice with the presence of comed[2]	Demonstrated for 8.25 hours for treated QCs<br>Demonstrated for 4 hours for untreated QCs		V1872106P2 and V1872106P2A1
Long-term (-20 °C) with and without the presence of comed[2]	Demonstrated instability at 62 days		V1872106P2
Long-term (-70 °C) with and without the presence of comed[2]	Demonstrated for 90 days for treated QCs<br>Demonstrated for 27 days for untreated QCs without comed[2]<br>Demonstrated for 35 days for untreated QCs with comed[2]		V1872106P2 and V1872106P2A1
Long-term (-70 °C) with and without the presence of comed[2]	Demonstrated for 180 days for treated QCs with and without comed[2]	Summary tables Table 9<br><br>Report text Section 10.3	
Whole Blood	No instability observed in ice-water bath or at room temperature in polyethylene terephthalate for up to 1.5 hour.		V1872106P2
Processed Matrix polypropylene			
Room Temperature	Demonstrated for 3 days 0 hours		V1872106P2
Refrigeration	Demonstrated for 3 days 0 hours		V1872106P2

Table 6: Summary of Results and Conclusions (Continued)

**Methodology – TP-80194**	**Method Performance – LC-MS/MS**	**Hyperlink – N/A**	**Comments**
**Individual Sample Reinjection**	Reproducible		V1872106P2
**Whole Run Reinjection Integrity**	Reproducible<br>Duration 2 days 7 hours		V1872106P2
**Column Ruggedness**	Reproducible		V1872106P2
**Extraction Recovery**			
Zidebactam	Overall 95.1%		V1872106P2
Cefotaxime (IS)	Overall 99.0%		V1872106P2
**Solution Stability**			
**Solution, Primary polypropylene**			
Zidebactam in deionized water (RT)	Stable 17.75 hours		V1872106U2 and V1872106P2
Zidebactam in deionized water (-20 °C)	Stable 25 days<br>Stable 61 days		V1872106U2
**Solution, Working polypropylene**			
Zidebactam Spike in deionized water (RT)	Stable 17.75 hours		V1872106U2 and V1872106P2
Zidebactam Spike in deionized water (-20 °C)	Stable 25 days<br>Stable 61 days		V1872106U2
Cefotaxime (IS) in deionized water in glass (RT)	Stable 15.5 hours<br>Stable 19.0 hours		V1872106U1 and V1872106P2A1

Table 7: Summary of Validation Run Objectives and Outcome

Run ID	Instrument ID	Extraction Date	Assay Date	Run Objective(s)	Accepted or Rejected	Comment
1	LC/MS-22	07 Mar 2023	07 Mar 2023	Treated long-term matrix stability	Accepted	

Table 8: Carryover

Run ID	Result (Extracted Blank after High Standard)	Carryover Results for Internal Standard
1	Seq 13 and Seq 56 are <20.0% of the LLOQ	Seq 4 and Seq 47 are < 5.0% internal standard peak of acceptable standards.

Carryover is evaluated within each run. It consists of measuring the response for each analyte and if it exceeds 20% of the response of LLOQ standard or other sample reflecting the lower sensitivity limit required, a carryover assessment is completed, and affected samples are rejected. The internal standard carryover evaluation is assessed within each run and if it exceeds 5% of the average internal standard response of acceptable standards and QCs, impact is evaluated within the run.

Carryover evaluation of the internal standard are within 5% in each run. Calibration Standards and QCs are arranged in a way to mitigate carryover in all validation runs, and no QCs was deactivated due to carry-over assessments.

Table 9: Long-Term Stability at -70 °C - Treated

Treated with and without comed[2]
Analytical Performance: Back-Calculated Concentrations (µg/mL) of Zidebactam Calibration Standard in (Human) (Plasma) in (Study V1872106P2A2)

Assay Date	Run ID	Cal 0.0500 0.0500 µg/mL	Cal 0.100 0.100 µg/mL	Cal 1.00 1.00 µg/mL	Cal 5.00 5.00 µg/mL	Cal 10.0 10.0 µg/mL	Cal 20.0 20.0 µg/mL	Cal 40.0 40.0 µg/mL	Cal 50.0 50.0 µg/mL
07-Mar-2023	1	0.0492	0.0961	0.942	4.98	10.2	19.9	38.7	47.8
		0.0518	0.101	0.995	5.25	10.4	20.9	41.1	48.7
Mean		0.0505	0.0986	0.969	5.12	10.3	20.4	39.9	48.3
%Bias		1.0	-1.4	-3.1	2.4	3.0	2.0	-0.3	-3.4

Calibration Curve Parameters for Zidebactam Calibration Standards in Human Plasma (Study V1872106P2A2)

Run Date	Run ID	Slope	Intercept	R-Squared	LLOQ	ULOQ
07-Mar-2023	1	0.07653	-0.00096	0.9985	0.0500	50.0

Run Date	Run ID	QCS 0.150 0.150 µg/mL	%Bias	QCS 25.0 25.0 µg/mL	%Bias	QCS 37.5 37.5 µg/mL	%Bias
07-Mar-2023	1	0.140	-6.7	25.0	0.0	36.3	-3.2
		0.143	-4.7	24.2	-3.2	38.2	1.9
Intrarun Mean		0.142		24.6		37.3	
Intrarun %Bias		-5.3		-1.6		-0.5	

Run Date	Run ID	LTS -70 low 0.150 µg/mL	%Bias	LTS -70 high 37.5 µg/mL	%Bias
07-Mar-2023	1	0.156	4.0	33.5	-10.7
		0.159	6.0	35.3	-5.9
		0.162	8.0	34.5	-8.0
		0.160	6.7	35.7	-4.8
		0.155	3.3	33.8	-9.9
		0.159	6.0	36.3	-3.2
Intrarun Mean		0.159		34.9	
Intrarun SD		0.00259		1.10	
Intrarun %CV		1.6		3.2	
Intrarun %Bias		6.0		-6.9	
n		6		6	

Run Date	Run ID	Comed1 LTS -70 low 0.150 µg/mL	%Bias	Comed1 LTS -70 high 37.5 µg/mL	%Bias
07-Mar-2023	1	0.158	5.3	34.7	-7.5
		0.153	2.0	36.3	-3.2
		0.166	10.7	37.3	-0.5
		0.160	6.7	36.2	-3.5
		0.159	6.0	34.1	-9.1
		0.156	4.0	37.2	-0.8
Intrarun Mean		0.159		36.0	
Intrarun SD		0.00437		1.31	
Intrarun %CV		2.7		3.6	
Intrarun %Bias		6.0		-4.0	
n		6		6	

Criteria: To be deemed stable, the mean %Bias at each level must be within ± 15% with %CV ≤ 15%.
Results: Zidebactam is stable in treated human plasma with and without the presence of comed[2], with sodium heparin as anticoagulant, for up to 180 days upon storage in polypropylene tube at -70 °C.

1. TITLE: THE DETERMINATION OF TOTAL ZIDEBACTAM IN TREATED HUMAN PLASMA, WITH SODIUM HEPARIN AS ANTICOAGULANT, BY LC-MS/MS

BIOANALYTICAL METHOD VALIDATION REPORT

Bioanalytical Project Number:	V1872106P2
Sponsor:	DynPort Vaccine Company, LLC<br>110 Thomas Johnson Drive<br>Suite 200<br>Frederick, MD 21702
Bioanalytical Laboratory:	KCAS Bioanalytical & Biomarker Services.<br>10830 S Clay Blair Boulevard<br>Olathe, KS 66061
Issue Date:	February 2023
Amendment 1 Issue Date:	March 2023

2. SYNOPSIS

This report presents the validation data for an LC-MS/MS method for the determination of total zidebactam in treated human plasma, with sodium heparin as anticoagulant. The assay was analyzed in the range of 0.0500 – 50.0 mcg/mL and is for use on human plasma samples obtained during clinical trials.

AMENDMENT OUTLINE FORM

1. Amendment Number 1	March 2023 - This amendment was created to report:<br>• Complete reissue of the original report to add more stability data per sponsor request.

3. TABLE OF CONTENTS FOR THE BIOANALYTICAL METHOD VALIDATION REPORT

1. TITLE: THE DETERMINATION OF TOTAL ZIDEBACTAM IN TREATED HUMAN PLASMA, WITH SODIUM HEPARIN AS ANTICOAGULANT, BY LC-MS/MS 1
2. SYNOPSIS 2
3. TABLE OF CONTENTS FOR THE BIOANALYTICAL METHOD VALIDATION REPORT 3
4. LIST OF TABLES 5
5. LIST OF FIGURES 6
6. SIGNATURE PAGE 7
7. QUALITY ASSURANCE STATEMENT 8
8. STUDY COMPLIANCE STATEMENT 9
9. LIST OF ABBREVIATIONS AND DEFINITIONS OF TERMS 10
10. AMENDMENT 12
11. EXPERIMENTAL DESIGN AND RESULTS 12
11.1. Reference Standards 12
11.2. Calibration Standards and QC Samples Preparation 13
11.3. Selectivity 13
11.3.1. Blank Evaluation 13
11.3.2. Matrix Effect 13
11.3.3. Interference Evaluation 14
11.3.4. Matrix Evaluation 14
11.3.5. Carryover 14
11.4. Linearity and Analytical Range 14
11.5. Precision and Accuracy 15
11.6. Sensitivity 16
11.7. Run Size 16
11.8. Precision and Accuracy of Dilution 16
11.9. Freeze/Thaw Stability - Treated 17

11.10. Freeze/Thaw Stability – Untreated 17
11.11. Bench-Top Stability in Wet Ice - Treated....................17
11.12. Bench-Top Stability in Wet Ice - Untreated 18
11.13. Long-Term Matrix Stability at -20 °C and -70 °C - Treated 18
11.14. Long-Term Matrix Stability at -70 °C - Untreated....................19
11.15. Stability During Blood Collection and Processing....................19
11.16. Processed Sample Stability at Room Temperature....................19
11.17. Processed Sample Stability when Refrigerated 20
11.18. Individual Sample Reinjection Reproducibility 20
11.19. Whole Run Reinjection Reproducibility 20
11.20. Column Ruggedness 20
11.21. Extraction Recovery 21
11.22. Solution Stabilities....................21
11.23. Calculations 21
11.24. SOP Deviations and Investigations 22
12. CONCLUSION....................22
13. PERTINENT SOPS 23
14. ARCHIVES 23
15. REFERENCES 23
16. TABLES 24
17. FIGURES....................78

4. LIST OF TABLES

Table 1: Abbreviations and Codes 10
Table 2: Mass Spec Transitions 24
Table 3: Reference Standards 24
Table 4: Calibration Standards and QC Samples Preparation 25
Table 5: Matrix Description 29
Table 6: Summary of Results and Conclusions 31
Table 7: Summary of Validation Run Objectives and Outcome 36
Table 8: Carryover 38
Table 9: Selectivity - Blank Assessment 39
Table 10: Matrix Effect 40
Table 11: Interference Evaluation 41
Table 12: Hemolytic Effect Evaluation 42
Table 13: Lipemic Effect Evaluation 42
Table 14: Summary of Standard Curves 43
Table 15: Precision and Accuracy (Intra-Assay) 44
Table 16: Precision and Accuracy (Inter-Assay) 45
Table 17: Standard and QC of Failed Runs 46
Table 18: Standards and QCs of Non-P&A Runs 47
Table 19: Precision and Accuracy of Sample Dilution 49
Table 20: Freeze-Thaw Stability, (Frozen At -20 °C and -70 °C Thawed in Wet Ice) - Treated 50
Table 21: Freeze-Thaw Stability, (Frozen At -70 °C Thawed in Wet Ice) - Untreated 52
Table 22: Bench-Top Stability in Wet Ice- Treated 53
Table 23: Bench-Top Stability in Wet Ice- Untreated 55
Table 24: Long-Term Stability at -20 °C and -70 °C - Treated 56
Table 25: Long-Term Stability at -20 °C and -70 °C - Untreated 58
Table 26: Stability Evaluation During Blood Collection and Processing 60
Table 27: Processed Sample Stability at Room Temperature 61

Table 28: Refrigerated Processed Sample Stability 61
Table 29: Individual Sample Reinjection Reproducibility 62
Table 30: Whole Run Reinjection Reproducibility and Column Ruggedness 63
Table 31: Extraction Recovery – Zidebactam 64
Table 32: Extraction Recovery – Cefotaxime 65
Table 33: Stability of Zidebactam Primary Solution in Deionized Water at Room Temperature 66
Table 34: Stability of Zidebactam Primary Solution in Deionized Water at -20 °C 68
Table 35: Stability of Zidebactam Spiking Solution in Deionized Water at Room Temperature 70
Table 36: Stability of Zidebactam Spiking Solution in Deionized Water at -20 °C 73
Table 37: Stability of Cefotaxime Working Internal Standard Solution in Deionized Water at Room Temperature 76

5. LIST OF FIGURES

Figure 1: Typical Calibration Curve 78
Figure 2: Typical Ion Chromatogram Blank Human Plasma without Internal Standard 79
Figure 3: Typical Ion Chromatogram Blank Human Plasma with Internal Standard 80
Figure 4: Typical Ion Chromatogram LLOQ Calibration Standard, Zidebactam = 0.0500 mcg/mL 81
Figure 5: Typical Ion Chromatogram Calibration Standard, Zidebactam = 20.0 mcg/mL 82
Figure 6: Typical Ion Chromatogram ULOQ Calibration Standard, Zidebactam = 50.0 mcg/mL 83
Figure 7: Typical Ion Chromatogram Cross-Talk Evaluation High QC No IS 84

Annex 1 .... Test Procedure
Annex 2 .... Certificate(s) of Analysis
Annex 3 .... Interference Testing

6. SIGNATURE PAGE

Bioanalytical Project Number: V1872106P2

Amendment 1 Issue Date: March 2023

APPROVED

DocuSigned by:
Anika Pippin
Signer Name: Anika Pippin
Signing Reason: I approve this document
Signing Time: 10-Mar-2023 | 09:45 CST
BE3AA1198E40459E8A7E3C615D981D82

Anika Pippin, B.S.
Principal Investigator

REVIEWED

DocuSigned by:
Marsha Luna
Signer Name: Marsha Luna
Signing Reason: I have reviewed this document
Signing Time: 10-Mar-2023 | 09:27 CST
C9CC738561AF4CC1B375ED013DD1E4BD

Marsha Luna, B.S.
Management

7. QUALITY ASSURANCE STATEMENT

This Bioanalytical Method Validation Report has been found to describe accurately the methods and SOPs used and to accurately reflect the raw data collected. The Quality Assurance Unit initiated audits on this study and reported the findings to the Principal Investigator (PI) and Management as follows:

		Date(s) Sent to:	
**Inspection Date(s)**	**Inspection Phase**	**PI**	**Management**
13Oct2022	Data Audit	13Oct2022	18Jan2023
18Oct2022	Data Audit	18Oct2022	21Oct2022
20Oct2022	Data Audit	20Oct2022	28Oct2022
20Oct2022	Draft Report Review	20Oct2022	23Nov2022
28Nov2022	Data Audit	28Nov2022	28Nov2022
05Dec2022	Updated Draft Report Review	05Dec2022	03Feb2023
03Feb2023	Final Report Review	03Feb2023	03Feb2023
10Feb2023	Data Audit	10Feb2023	10Feb2023
14Feb2023	Data Audit	14Feb2023	14Feb2023
15Feb2023	Data Audit	15Feb2023	15Feb2023
23Feb2023	Data Audit	23Feb2023	23Feb2023
01Mar2023	Data Audit	01Mar2023	01Mar2023
03Mar2023 and 06Mar2023	A1 Draft Report Review	06Mar2023	06Mar2023
08Mar2023	A1 Final Report Review	08Mar2023	08Mar2023

REVIEWED

DocuSigned by:

Connie Anderson

Signer Name: Connie Anderson
Signing Reason: I have reviewed this document
Signing Time: 10-Mar-2023 | 08:30 CST
9429F81BF6DE47B3A3DEEE0F97D96287

Connie Anderson, B.S., ASQ-CQA
Quality Assurance Unit

8. STUDY COMPLIANCE STATEMENT

I, the undersigned, hereby declare that this study was conducted in compliance with the relevant bioanalytical aspects of FDA guidelines, GLP documentation and reporting GLP regulations 21CFR Part 58 and KCAS Bioanalytical & Biomarker Services (KCAS) procedures and that the work was performed by me, or under my direction, and the report reflects a true and accurate record of the results obtained.

The acceptance criteria are based on the FDA Guidance for Industry, Bioanalytical Method Validation (2018). [1]

APPROVED

DocuSigned by:

Anika Pippin

Signer Name: Anika Pippin
Signing Reason: I approve this document
Signing Time: 10-Mar-2023 | 09:45 CST
BE3AA1198E40459E8A7E3C615D981D82

Anika Pippin, B.S.
Principal Investigator

9. LIST OF ABBREVIATIONS AND DEFINITIONS OF TERMS

Table 1: Abbreviations and Codes

Abbreviation	Definition
<	Concentration less than the lowest acceptable calibration standard
>	Concentration exceeded the upper limit of calibration range (> ULOQ)
A	Amendment
AQL	Above Quantitation Limit
%Bias	Percentage measurement valid
Batch	Is the same as Run
BQL	Below Quantifiable Limit
Cal	Calibration standard
Conc.	Concentration
Conv.	Conversion
%CV	Coefficient of Variation
dev	Deviation
diff	Difference
EMA	European Medicines Agency
FDA	Food and Drug Administration
F/T	Freeze/Thaw
GLP	Good Laboratory Practice
HPLC	High Performance Liquid Chromatography
ID	Identification
IS	Internal Standard
ISR	Incurred Sample Reanalysis
K_2EDTA or K_3EDTA	Dipotassium or Tripotassium Ethylenediaminetetraacetic Acid
KCAS	KCAS Bioanalytical & Biomarker Services
LC-MS/MS	Liquid Chromatography Tandem Mass Spectrometry
LLOQ	Lower Limit of Quantitation
mcg or µg or ug	Microgram
mcL or µL or uL	Microliter

Table 1: Abbreviations and Codes (Continued)

Abbreviation	Definition
ME	Matrix Effect
MF	Matrix Factor
MHLW	Ministry of Health, Labour and Welfare of Japan
mL	Milliliter
MS	Mass Spectrometry
n	number
N.C.	Not Calculated
ND	No difference reported as ISR will be counted
ng	nanogram
n.report	No Data available to report
N/A or NA	Not Applicable
N.R.	Not Reported
OECD	Organization for Economic Co-operation and Development
P&A	Precision and Accuracy
p.a.r.	Peak Area Ratio
PI	Principal Investigator
QC	Quality Control Sample
QCs	Quality Control Samples
RT	Room Temperature
sd or SD	Standard deviation
Seq	Sequence
SOP (s)	Standard Operating Procedure (s)
temp	Temperature
TP	Test procedure
UHPLC	Ultra High Performance Liquid Chromatography
ULOQ	Upper Limit of Quantitation
WIS	Working Internal Standard

This is a basic list. Some of the abbreviations may not have been used in this report.

10. AMENDMENT

This amendment was created to report a complete reissue of the original report to add more stability data per sponsor request.

The objective of this study was to perform a full validation of a bioanalytical method for the determination of total zidebactam in MES ([2-(N-Morpholino) ethane sulphonic acid hydrate])-treated human plasma, with sodium heparin as anticoagulant. This validation report describes the selectivity, sensitivity, linearity, accuracy, precision, stability and recovery experiments based on an established bioanalytical test procedure.

The validation procedure was designed to meet or exceed the requirements of FDA Guidance for Industry on Bioanalytical Methods Validation, May 2018. The software programs used were Microsoft Office Excel 365, acquisition and processing software Analyst 1.7.2, and data management software Watson LIMS 7.5.

11. EXPERIMENTAL DESIGN AND RESULTS

In this method, zidebactam and internal standards, cefotaxime, were released from human plasma using protein precipitation. After extraction, the sample was chromatographed using reversed phase HPLC with a Fortis C18 analytical column. Zidebactam and cefotaxime (IS) were detected by monitoring the precursor and product ions described in Table 2 using an Applied Biosystems Sciex API 5000 or API 6500 LC-MS/MS. The quantification was based on setting a calibration graph using the internal standard method based on bioanalytical test procedure TP-80194, entitled “LC-MS/MS Analysis of Zidebactam in Treated Human Sodium Heparin Plasma (100 mcL, 0.0500 – 50.0 mcg/mL; Prior to MES Treatment)”. The test procedure can be found in Annex 1.

Human plasma, screened for the lack of interference at the retention times of ertapenem and cefotaxime (IS), was diluted 1:1 with MES buffer, pH 6.4 for stabilization. Since the standards and QCs concentrations were already adjusted for the addition of MES during the preparation, the obtained total zidebactam concentration of the unknown study samples do not need to be adjusted for the dilution with MES buffer.

A summary of the validation results is provided in Table 6. A summary describing the objective(s) and outcome of each validation run attempted is provided in Table 7.

The method was validated within the analytical range of 0.0500 – 50.0 mcg/mL in human plasma. No development work was performed after the method was validated.

11.1. Reference Standards

Reference standards used during this validation are presented in Table 3.

11.2. Calibration Standards and QC Samples Preparation

Calibration standards and quality control pools were prepared by spiking appropriate levels of zidebactam into human plasma, with sodium heparin as anticoagulant. Primary reference standards were prepared in at least duplicate and compared within 5% of each other. Standards and QCs were prepared and stored frozen at KCAS at -70 °C. Stability QCs were also stored at -20 °C and -70 °C. Standards, QCs, and the critical solutions used during this validation are described in Table 4, and matrix used is described in Table 5.

11.3. Selectivity

Selectivity is the ability of a method to detect and quantify the desired analytes without excessive false positives or negatives due to endogenous substances, co-administered analytes, metabolites, or other substances introduced/removed during sample work up. The selectivity of this method was achieved by a sample clean-up procedure using protein precipitation followed by chromatography and specific detection of the precursor and product ions of each analyte using tandem mass spectrometry (MS/MS).

11.3.1. Blank Evaluation

A minimum of six different sources of plasma (sodium heparin as anticoagulant) were screened. To be deemed acceptable, the peak response observed must be ≤ 20.0% of the mean response of the lower limit of quantitation (LLOQ) and ≤ 5.0% of the mean response of the internal standard. If any lots fail, further evaluation is needed.

All six matrices lots were found to be free of any significant interference at the retention times of the analytes of interest. Data is presented in Table 9. Typical chromatograms for blank plasma, with sodium heparin as anticoagulant, without and with internal standard are presented in Figure 2 and Figure 3, respectively. Cross talk was also evaluated by extracting the high QC with no internal standard. There are no apparent effects of cross talk, and a typical chromatogram is present in Figure 7.

11.3.2. Matrix Effect

Matrix effect in MS applications is defined as inter-matrix variation in signal due to co-eluting matrix substances which cause enhancement or suppression of the detector response for the measured analyte.

To evaluate matrix effect, six QC pools were prepared at approximately 3 times the LLOQ and at the high QC concentration, each from a different lot of human plasma, with sodium heparin as anticoagulant. These QC pools were each extracted in replicates of four according to the test procedure, and the mean response ratios were calculated for each matrix. To be deemed free of matrix effect, the %CV of the mean ratios across sources was required to be ≤ 15.0%.

Matrix effect data are presented in Table 10. The matrix effect results meet acceptance criteria.

Note: matrix factor (comparison between extracted blank plasma versus neat solution after they were spiked at the end of the extraction) was not used for this determination as outlined by the validation plan. This alternative approach (extraction of the spiked QCs from 6 lots of plasma to calculate their % CV) is supported by SOP-00035 and is preferred due to stability of the drug and being treated.

11.3.3. Interference Evaluation

Assay selectivity was confirmed by spiking quality control pools with 150 mcg/mL ertapenem. These QC pools, containing low and high levels of zidebactam, were analyzed in replicates of six in a single run. To be considered acceptable, the mean %Bias at each level is required to be within ± 15.0% and the %CV is required to be ≤ 15.0%.

The results presented in Table 11, indicate the presence of 150 mcg/mL ertapenem does not affect the accuracy or precision of the data.

Additional interference testing not necessary – see Annex 3 from the Sponsor.

11.3.4. Matrix Evaluation

To evaluate different types of collected matrix, QC pools were prepared with treated hemolytic (2% hemolysis) and lipemic human plasma, with sodium heparin as anticoagulant, at approximately three times the LLOQ and the high QC concentration. These QC pools were each extracted in at least replicates of three, and the mean concentrations or responses were calculated for each matrix. To be considered acceptable, the mean %Bias at each level was also required to be within ± 15.0% and the %CV was required to be ≤ 15.0%.

The results, presented in Table 12 to Table 13, indicate that treated hemolytic and lipemic plasma does not affect the analysis of zidebactam in human plasma.

Furthermore, blank lipemic and treated hemolytic control plasma were extracted with and without internal standard to determine that there were no significant interferences observed at the retention times of the analytes. There were no significant interferences observed.

11.3.5. Carryover

Carryover is evaluated within each run. It consists of measuring the response for each analyte and if it exceeds 20.0% of the response of LLOQ standard or other sample reflecting the lower sensitivity limit required, a carryover assessment is completed, and affected samples are rejected. The internal standard carryover evaluation is assessed within each run and if it exceeds 5.0% of the average internal standard response of acceptable standards and QCs, impact is evaluated within the run. Data is presented in Table 8.

11.4. Linearity and Analytical Range

The calibration curve was constructed with eight different, non-zero standard concentrations ranging from 0.0250 – 25.0 mcg/mL in treated human plasma (equivalent to 0.0500 to 50.0

mcg/mL in untreated human plasma), with sodium heparin as anticoagulant. Concentrations are based on plasma volume only and no adjustments need to be made to unknown sample concentrations. Calibration standards were processed in duplicate and the calibration curve was evaluated using least squares linear regression with weighting $1/x^2$. The maximum allowable percent deviation from nominal for each standard was ± 15.0%, except at the LLOQ, where ± 20.0% was accepted. Any standard deviating by more than these limits was required to be excluded from the regression line. For the calibration curve to be accepted, at least 75% of the initial number of standards was required to remain in the curve.

The calibration curve was found to be linear. The data are presented in Table 14. A typical calibration curve is presented graphically in Figure 1. Typical chromatograms illustrating blank human plasma standards spiked with zidebactam at the LLOQ, mid, and high concentrations are presented in Figure 4 to Figure 6, respectively.

11.5. Precision and Accuracy

The precision of an assay is defined as the closeness in which replicate analyses agree and is reported as percent coefficient of variation (%CV). The accuracy of a method is defined as the closeness of a determined value to its true (nominal) value and is reported as %Bias. At least one precision and accuracy run was performed using standards prepared from one primary stock and the QCs were prepared from a separate primary stock. Inter-assay (between runs) precision & accuracy and intra-assay (within runs) precision & accuracy were assessed in four runs by analyzing six (6) replicates each of QCs fortified at four separate concentrations (LLOQ, low, mid, and high). For inter- and intra-assay, the mean %Bias at each QC level was required to be within ±15.0% from its respective nominal concentration, except at the LLOQ, where ± 20.0% was accepted. The %CV at each QC level was required to be ≤ 15.0%, except at the LLOQ, where ≤ 20.0% was accepted.

The intra-assay and inter-assay precision and accuracy data for this method met the required criteria and are presented in Table 15 (intra-assay precision and accuracy) and Table 16 (inter-assay precision and accuracy).

Failed runs are reported in Table 17. See Table 7 for reasons of failure.

For each non-P&A run, the calculated concentrations of at least 75% of the calibration standards are required to interpolate within ± 15.0% from nominal with a minimum of six calibration standard levels, except for the LLOQ for which it was required to be within ± 20.0%. In order for the run to be acceptable, at least one QC at each concentration level and two-thirds of the total number of QC samples were required to interpolate within ± 15.0% from nominal.

Standards and QCs of non-P&A runs are reported in Table 18 and acceptance criteria was met.

11.6. Sensitivity

The sensitivity of the method was determined in four runs by analyzing six (6) replicates of QCs at the lower limit of quantitation (LLOQ) which were equal in concentration to the lowest calibration standard. The observed response at the LLOQ was required to be at least 5 times the response of noise in the human plasma blank, in addition to the precision and accuracy requirements outlined in the previous section.

The LLOQ for this method was determined to be 0.0500 mcg/mL, and the response at the LLOQ was at least 5 times the response of noise in the human matrix blank. Data are presented in Table 15 (intra-assay precision and accuracy) and Table 16 (inter-assay precision and accuracy).

11.7. Run Size

In order to evaluate the largest size run that can be used for study sample analysis, a run was processed with a set of 8 calibration standards and a minimum of 3 QC samples, processed in duplicate. In addition, at least 3 matrix blanks (one with internal standard and two without internal standard) were processed with the run. Run 12 was used for this assessment, and additional experiments were also analyzed which made the final run size 125.

In order for the run to be acceptable, at least one QC at each concentration level and two-thirds of the total number of QC samples were required to interpolate within ± 15.0% from nominal.

The standard and QC summary of run 12 is presented in Table 14 and Table 15 and acceptance criteria was met.

11.8. Precision and Accuracy of Dilution

To determine the accuracy of dilution, the high concentration QC spiked with zidebactam (37.5 mcg/mL) as well as an above-range QC (ARQC; 400 mcg/mL) were evaluated. All samples were extracted in six replicates according to the test procedure. To be deemed acceptable, the mean %Bias at each level must be within ± 15.0% with %CV ≤ 15.0%. The replicates were prepared as follows:

- Dilution factor 2x (high QC) = 50 mcL sample + 50 mcL blank treated human plasma
- Dilution factor 10x (ARQC) = 20 mcL sample + 180 mcL blank treated human plasma, mix well and take 100 mcL for analysis.

Dilution precision and accuracy, using dilution factors of 2x and 10x, were proven to be valid. Data are presented in Table 19.

See Table 7 for reasons for the failure of runs 7 and 8. Dilution linearity was repeated and established in run 9 which met acceptance criteria.

11.9. Freeze/Thaw Stability - Treated

Zidebactam stability in treated human plasma, with sodium heparin as anticoagulant, during sequential freeze/thaw cycles (frozen separately at -20 °C and -70 °C and thawed in wet ice) with and without the presence of comed[2] was evaluated. Aliquots of high, low, and ARQC concentration QC samples were frozen for at least 12 hours before the initial thawing. They were re-frozen 3 more times for at least 12 hours each time and were thawed prior to assay along with a freshly prepared set of QCs and calibration standards. To be deemed stable, the mean %Bias at each level must be within ± 15.0% with %CV ≤ 15.0%.

During initial F/T evaluation in Run 12, the low F/T QCs failed. After re-prepare the QCs with flash freeze, the re-evaluation in Run 24 indicated zidebactam in treated human plasma with and without the presence of comed[2], with sodium heparin as anticoagulant, was proven stable over 4 cycles of freeze (-20 °C and -70 °C) and thaw (wet ice).

Zidebactam is stable in treated human plasma with the presence of comed[2], with sodium heparin as anticoagulant, for at least 4 cycles when frozen at -20 °C and -70 °C and thawed in wet ice. The matrix stability in run 12 failed for the low QC and passed for the high QC. Freeze/thaw at the low and high QCs were repeated in run 24.

Data are provided in Table 20.

11.10. Freeze/Thaw Stability – Untreated

Zidebactam stability in untreated human plasma, with sodium heparin as anticoagulant, during sequential freeze/thaw cycles (frozen separately at -70 °C and thawed in wet ice) with and without the presence of comed[2] was evaluated. Aliquots of high and low concentration QC samples were frozen for at least 12 hours before the initial thawing. They were re-frozen 1 more time for at least 12 hours each time and were thawed prior to assay along with a freshly prepared set of QCs and calibration standards. To be deemed stable, the mean %Bias at each level must be within ± 15.0% with %CV ≤ 15.0%.

Zidebactam is stable in untreated human plasma without the presence of comed[2], with sodium heparin as anticoagulant, for at least 2 cycles when frozen at -70 °C and thawed in wet ice.

Zidebactam is stable in untreated human plasma with the presence of comed[2], with sodium heparin as anticoagulant, for at least 2 cycles when frozen at -70 °C and thawed in wet ice.

Data are provided in Table 21.

11.11. Bench-Top Stability in Wet Ice - Treated

To evaluate zidebactam in treated human plasma, with sodium heparin as anticoagulant, stability during sample processing in wet ice, aliquots of the high, low and ARQC concentration QC pools with and without the presence of comed[2] were allowed to thaw in wet ice for 8.25 hours and 19.75 hours, prior to analysis along with a freshly prepared set of QCs and calibration

standards. To be deemed stable, the mean %Bias at each level must be within ± 15.0% with %CV ≤ 15.0%.

Zidebactam is stable in treated human plasma without the presence of comed[2], with sodium heparin as anticoagulant, for at least 19.75 hours in wet ice.

During initial benchtop evaluation in Run 12, the low QCs failed. After the QCs were reprepared with flash freeze, the re-evaluation in Run 24 indicated zidebactam is stable in treated human plasma with the presence of comed[2], with sodium heparin as anticoagulant, for at least 8.25 hours in wet ice.

Data are provided in Table 22.

11.12. Bench-Top Stability in Wet Ice - Untreated

To evaluate zidebactam in untreated human plasma, with sodium heparin as anticoagulant, stability during sample processing in wet ice, aliquots of the high and low concentration QC pools with and without the presence of comed[2] were allowed to thaw in wet ice for 4 hours, prior to analysis along with a freshly prepared set of QCs and calibration standards. To be deemed stable, the mean %Bias at each level must be within ± 15.0% with %CV ≤ 15.0%.

Zidebactam is stable in untreated human plasma without the presence of comed[2], with sodium heparin as anticoagulant, for at least 4 hours in wet ice.

Zidebactam is stable in untreated human plasma with the presence of comed[2], with sodium heparin as anticoagulant, for at least 4 hours in wet ice.

Data are provided in Table 23.

11.13. Long-Term Matrix Stability at -20 °C and -70 °C - Treated

To evaluate zidebactam in treated human plasma, with sodium heparin as anticoagulant, stability during sample storage, aliquots of the high and low concentration QC pools with and without the presence of comed[2] were stored frozen at -20 °C and -70 °C nominally for 34, 62, and 90 days. prior to being analyzed, along with freshly prepared standards and QC samples. To be deemed stable, the mean %Bias at each level must be within ± 15.0% with %CV ≤ 15.0%.

Zidebactam is stable in treated human plasma without the presence of comed[2], with sodium heparin as anticoagulant, for up to 90 days upon storage in polypropylene tube at -70 °C.

Zidebactam is unstable in treated human plasma without the presence of comed[2], with sodium heparin as anticoagulant, for up to 62 days upon storage in polypropylene tube at -20 °C. Run 30 -20 °C LTS did not meet criteria due to high %Bias at the low and high QC.

Zidebactam is stable in treated human plasma with the presence of comed[2], with sodium heparin as anticoagulant, for up to 90 days upon storage in polypropylene tube at -70 °C. Zidebactam is unstable in treated human plasma with the presence of comed[2], with sodium heparin as

anticoagulant, for up to 62 days upon storage in polypropylene tube at -20 °C. Run 30 -20 °C LTS QCs did not meet criteria at 62 days due to high %Bias at the low and high QC.

Due to the batch not meeting acceptance criteria in Run 25, the 34 days long-term stability evaluation of zidebactam in treated human plasma cannot be accepted and were not reported.

Data will be provided in Table 24

11.14. Long-Term Matrix Stability at -70 °C - Untreated

To evaluate zidebactam in untreated human plasma, with sodium heparin as anticoagulant, stability during sample storage, aliquots of the high and low concentration QC pools with and without the presence of comed[2] were stored frozen at -70 °C nominally for 17, 21, 24, 27, 28, and 35 days. prior to being analyzed, along with freshly prepared standards and QC samples. To be deemed stable, the mean %Bias at each level must be within ± 15.0% with %CV ≤ 15.0%.

Zidebactam is stable in untreated human plasma without the presence of comed[2], with sodium heparin as anticoagulant, for up to 27 days upon storage in polypropylene tube at -70 °C.

Zidebactam is stable in untreated human plasma with the presence of comed[2], with sodium heparin as anticoagulant, for up to 35 days upon storage in polypropylene tube at -70 °C. Run 42 -70 °C LTS failed criteria for high %Bias at the low and high QC, and high %CV at the high QC. This was likely a prep error and additional LTS was performed using these QCs.

Data will be provided in Table 25.

11.15. Stability During Blood Collection and Processing

Stability of zidebactam in whole blood, with sodium heparin as anticoagulant, was assessed by spiking whole blood at the mid-level concentration. These samples were stored immediately at room temperature and in an ice-water bath for 0 hour and at least 90 minutes in polyethylene terephthalate containers. After the designated time, the plasma was harvested, treated with chilled MES Buffer pH 6.4 at 1:1 ratio, and extracted. The % change was calculated with stored data comparing to the 0 hour data. To be considered stable, the % change between stored sample pools and the control 0-hr sample pools must be within ± 15.0%, with %CV ≤ 15.0%.

Zidebactam was shown stable during blood collection processing for up to 90 minutes stored in an ice-water bath or at room temperature in polyethylene terephthalate containers.

Data are provided in Table 26.

11.16. Processed Sample Stability at Room Temperature

To evaluate zidebactam stability in processed samples at room temperature, high and low concentration QC samples were processed, placed into polypropylene 96 well plates, stored, and allowed to remain at ambient conditions (~25 °C) before being chromatographed with samples in

a subsequent run. To be deemed stable, the mean %Bias at each level must be within ± 15.0% with %CV ≤ 15.0%.

Processed zidebactam samples were proven stable at room temperature stored in polypropylene 96 well plates over a period of 3 days 0 hours. Data are provided in Table 27.

11.17. Processed Sample Stability when Refrigerated

To evaluate zidebactam stability in processed samples at refrigerated temperatures, high and low concentration QC pools were processed, placed into polypropylene 96 well plates, and stored at refrigerated temperatures (~4 °C) before being chromatographed with samples in a subsequent run. To be deemed stable, the mean %Bias at each level must be within ± 15.0% with %CV ≤ 15.0%.

Processed zidebactam samples were proven stable when refrigerated (~4 °C) stored in polypropylene 96 well plates over a period of 3 days 0 hours. Data are provided in Table 28.

11.18. Individual Sample Reinjection Reproducibility

To evaluate individual sample reinjection reproducibility, low and high QC extracts (6 each) injected during the run were reinjected at the end of the run, within the established stability time window of processed samples in the autosampler/refrigerator. To be deemed reproducible, the mean %Bias at each level must be within ± 15.0% with %CV ≤ 15.0%.

Reinjection of individual samples can be made with adequate precision and accuracy. Data are provided in Table 29.

11.19. Whole Run Reinjection Reproducibility

To evaluate whole run reinjection reproducibility, run 13 was reinjected as run 15 on the same MS platform using a different chromatographic column from the same manufacturer, with the same specification as the original within 2 days 7 hours following the original injection. To be deemed acceptable, the reinjected run was required to meet the acceptance criteria outlined in Sections 11.4, 11.5, and 11.6.

It has been demonstrated that entire runs can be reinjected with adequate accuracy and precision. Data are presented in Table 30.

11.20. Column Ruggedness

Column ruggedness was assessed by reinjecting run 13 as run 15 on the same MS platform (Applied Biosystems API5000) using a different chromatographic column from the same manufacturer, with the same specification as the original.

Data were required to meet the precision and accuracy criteria outlined in Sections 11.4, 11.5, and 11.6 to be considered as reproducible/rugged method. Data are presented in Table 30. It has been demonstrated that ruggedness has been met.

11.21. Extraction Recovery

To evaluate the extraction recovery, the low, mid, and high extracted QC samples were compared to extracted human plasma blanks to which an equal amount of zidebactam at each level, assuming 100% recovery, was added in six replicates.

To evaluate the extraction recovery of cefotaxime (IS), zidebactam was added only for tracking during analysis. Cefotaxime (IS) for the control was added after extraction, while the same amount of cefotaxime (IS) for the extracted QC was added before extraction.

$$\%\ \text{Recovery} = \frac{\text{Response ratio of "Extracted QC"}}{\text{Average Response Ratio of Control}} \times 100$$

The mean recovery of zidebactam was determined to be consistent, precise and reproducible at 95.1%.

The recovery of cefotaxime (IS) was determined to be consistent, precise and reproducible at 99.0%. Data are presented in Table 31 to Table 32.

11.22. Solution Stabilities

The stability of various solutions of zidebactam in various solvents used during the sample analysis following storage at various temperatures was determined.

Data showing the zidebactam(s), solvent composition, storage term, and temperature are presented in Table 33 to Table 37.

11.23. Calculations

The peak areas of zidebactam and cefotaxime were integrated by Analyst 1.7.2 (Applied Biosystems; Foster City, CA, USA). The data results were imported electronically into Watson LIMS 7.5 for the calculation of zidebactam concentrations and statistical analysis. The calibration curves were obtained by a weighted (1/concentration^2) least squares linear regression. After the best fit from data, the concentrations (mcg/mL) were calculated based on the measured peak area ratios of zidebactam/cefotaxime using the following equation:

$$\text{Response} = \text{Slope} * \text{Conc} + \text{Intercept}$$

$$\text{Mean Value } (\overline{X}) = \frac{\sum_{i=1}^{n} \text{individual data } (X)}{\text{number of data points } (n)}$$

$$\text{Standard Deviation (SD)}=\sqrt{\frac{\sum_{i=1}^{n}\left[\text{individual data (X)-Mean Value }(\overline{X})\right]^2}{\text{n-1}}}$$

$$\text{Coefficient of Variation (\%CV)}=\left[\frac{\text{Standard Deviation (SD)}}{\text{Mean Value }(\overline{X})}\right]\times 100$$

$$\text{\%Bias}=\left(\frac{\text{Calculated Mean of the Observed Values or Observed Value-Nominal Value}}{\text{Nominal Value}}\right)\times 100$$

All calibration standards and QCs concentrations were reported to three significant figures, and study samples will be reported to three significant figures.

11.24. SOP Deviations and Investigations

No SOP deviations occurred. No investigations were conducted during this study.

12. CONCLUSION

Validation results obtained in this study indicate that a robust and reliable method has been established for the measurement of total zidebactam in treated human plasma, with sodium heparin as anticoagulant, by LC-MS/MS in the concentration range of 0.0500 – 50.0 mcg/mL. The generally recommended acceptance criteria, FDA Guidance for Industry on Bioanalytical Methods Validation, May 2018, in terms of precision, accuracy, selectivity including matrix effect, recovery and all types of stability have been met. Thus, this validated method is deemed suitable for determination of zidebactam in human plasma, with sodium heparin as anticoagulant, for unknown samples.

13. PERTINENT SOPS

The following SOPs were followed during the conduct of this study:

SOP-00073 [02-04] - Bioanalytical Method Validation - Chromatographic Assays

SOP-00035 [00-02] - Procedures, Reporting, and Acceptance Criteria for Bioanalytical Chromatographic Projects

14. ARCHIVES

All raw data, including paper and electronic files associated with this study will remain archived at a location specified by KCAS, 10830 S Clay Blair Boulevard, Olathe, KS 66061 for a period of at least 10 years, after which the client may be contacted to arrange for continued storage of the files, or disposition according to an agreed procedure. KCAS will maintain an electronic (scanned/true) copy of the method validation report that can be viewed and printed on site. The study plan, documentation, raw data, and final report are indexed under Bioanalytical Project Number V1872106P2A1 and V1872106P2.

15. REFERENCES

1. Guidance for Industry: Bioanalytical Method Validation. U.S. Department of Health and Human Services. Food and Drug Administration. Center for Drug Evaluation and Research. Center for Veterinary Medicine. May 2018.
2. Comed: QCs in the presence of 150 mcg/mL ertapenem.

16. TABLES

Table 2: Mass Spec Transitions

Analyte	Transition
Zidebactam	m/z 392.028→312.000
Cefotaxime (IS)	m/z 456.200→324.000

Table 3: Reference Standards

Analyte	Supplier	Lot/Batch Number	Purity	Expiration Date or Retest Date	Conversion Factor
Zidebactam	Wockhardt	WK1/WP-0060/016	90.4%	Expire 29 Mar 2023	1.000
Cefotaxime Sodium Salt (IS)	Sigma Aldrich	0000146942	95.9%	Retest 30 Sep 2023	1.048
Ertapenem Sodium	Med Chem Express	155576	98.9%	Retest 20 Jun 2024	1.046
	Med Chem Express	180017	98.9%	Retest 11 Aug 2024	1.046

Certificates of analysis for the above reference standards are presented in Annex 2.

Table 4: Calibration Standards and QC Samples Preparation

Critical Solutions	Lot # for Standards (Prep Date)	Lot # for QCs (Prep Date)	Standards Lot # (Prep Date)	QCS Lot # (Prep Date)	Runs used
Treated Human Sodium Heparin Plasma (1:1 MES buffer:plasma)	V1872106P1-18-2		V1872106P2-13-1 (Set A) (15Aug2022)	V1872106P2-14-1 (Set A) (15Aug2022)	5, only QCs used for 7-8
Deionized Water stored at -20°C in polypropylene	V1872106P2-2-1 (09Aug2022) V1872106P2-4-1 (09Aug2022)	V1872106P2-1-1 (09Aug2022) V1872106P2-5-1 (09Aug2022)			
Treated Human Sodium Heparin Plasma (1:1 MES buffer:plasma)	V1872106P1-18-2		V1872106P2-25-1 (Set B) (17Aug2022)	NA	7
Deionized Water stored at -20°C in polypropylene	V1872106P2-2-1 (09Aug2022) V1872106P2-4-1 (09Aug2022)	NA			
Treated Human Sodium Heparin Plasma (1:1 MES buffer:plasma)	V1872106P1-18-2		V1872106P2-27-1 (Set C) (18Aug2022)	NA	8
Deionized Water stored at -20°C in polypropylene	V1872106P2-2-1 (09Aug2022) V1872106P2-4-1 (09Aug2022)	NA			
Treated Human Sodium Heparin Plasma (1:1 MES buffer:plasma)	V1872106P2-28-1		V1872106P2-29-1 (Set D) (22Aug2022)	V1872106P2-30-1 (Set D) (22Aug2022)	9, only cals used for 10
Deionized Water stored at -20°C in polypropylene	V1872106P2-2-1 (09Aug2022) V1872106P2-4-1 (09Aug2022)	V1872106P2-1-1 (09Aug2022) V1872106P2-5-1 (09Aug2022)			
Treated Human Sodium Heparin Plasma (1:1 MES buffer:plasma)	V1872106P2-38-2		V1872106P2-38-1 (Set E) (26Aug2022)	V1872106P2-40-1 (Set E) (26Aug2022)	12-14
Deionized Water stored at -20°C in polypropylene	V1872106P2-2-1 (09Aug2022) V1872106P2-4-1 (09Aug2022)	V1872106P2-1-1 (09Aug2022) V1872106P2-5-1 (09Aug2022)			
Treated Human Sodium Heparin Plasma (1:1 MES buffer:plasma)	V1872106P2-64-2		V1872106P2-64-1 (Set G) (20Sep2022)	V1872106P2-65-1 (Set G) (20Sep2022)	24
Deionized Water stored at -20°C in polypropylene	V1872106P2-41-1 (01Sep2022) V1872106P2-43-1 (01Sep2022)	V1872106P2-41-1 (01Sep2022) V1872106P2-51-1 (06Sep2022)			

Table 4: Calibration Standards and QC Samples Preparation (Continued)

Critical Solutions	Lot # for Standards (Prep Date)	Lot # for QCs (Prep Date)	Standards Lot # (Prep Date)	QCS Lot # (Prep Date)	Runs used
Treated Human Sodium Heparin Plasma (1:1 MES buffer:plasma)	V1872106P1-18-2	V1872106P1-18-2	NA	V1872106P2-15-1 Comed QCs	5
Deionized Water stored at -20°C in polypropylene	NA	V1872106P2-1-1 (09Aug2022) V1872106P2-5-1 (09Aug2022) V1872106P1-11-1 (08Aug2022)			
Treated Human Sodium Heparin Plasma (1:1 MES buffer:plasma)	V1872106P2-28-1	V1872106P2-28-1	NA	V1872106P2-32-1 Comed QCs	14
Deionized Water stored at -20°C in polypropylene	NA	V1872106P2-1-1 (09Aug2022) V1872106P2-5-1 (09Aug2022) V1872106P1-11-1 (08Aug2022)			
Treated Human Sodium Heparin Plasma (1:1 MES buffer:plasma)	V1872106P2-66-2	V1872106P2-66-2	V1872106P2-67-1 (Set H) (12Oct2022)	V1872106P2-68-1 (Set H) (12Oct2022)	25
Deionized Water stored at -20°C in polypropylene	V1872106P2-41-1 (01Sep2022) V1872106P2-43-1 (01Sep2022)	V1872106P2-41-1 (01Sep2022) V1872106P2-51-1 (06Sep2022)			
Treated Human Sodium Heparin Plasma (1:1 MES buffer:plasma)	V1872106P5-35-1	V1872106P5-35-1	V1872106P2-77-1 (Set I) (09Nov2022)	V1872106P2-78-1 (Set I) (09Nov2022)	30
Deionized Water stored at -20°C in polypropylene	V1872106P2-69-1 (08Nov2022) V1872106P2-73-1 (08Nov2022)	V1872106P2-69-1 (08Nov2022) V1872106P2-74-1 (08Nov2022)			
Treated Human Sodium Heparin Plasma (1:1 MES buffer:plasma)	V1872106P2-56-1	V1872106P2-56-1	NA	V1872106P2-58-1 (Set F) (08Sep2022) Comed QCs	25 (LTS), 30 (LTS) 31 (LTS)
Deionized Water stored at -20°C in polypropylene	NA	V1872106P2-41-1 (01Sep2022) V1872106P2-51-1 (06Sep2022) V1872106P2-53-1 (07Sep2022)			
Treated Human Sodium Heparin Plasma (1:1 MES buffer:plasma)	V1872106P2-56-1	V1872106P2-56-1	NA	V1872106P2-57-1 (Set F) (08Sep2022)	25 (LTS), 30 (LTS) 31 (LTS)
Deionized Water stored at -20°C in polypropylene	NA	V1872106P2-41-1 (01Sep2022) V1872106P2-51-1 (06Sep2022)			

Table 4: Calibration Standards and QC Samples Preparation (Continued)

Critical Solutions	Lot # for Standards (Prep Date)	Lot # for QCs (Prep Date)	Standards Lot # (Prep Date)	QCS Lot # (Prep Date)	Runs used
Treated Human Sodium Heparin Plasma<br>(1:1 MES buffer:plasma)	V1872106P2-88-2		V1872106P2-88-1<br>Set I<br>(07Dec2022)	V1872106P2-89-1<br>Set I<br>(07Dec2022)	31
Deionized Water stored at -20°C in polypropylene	V1872106P2-79-1<br>(06Dec2022)<br>V1872106P2-83-1<br>(06Dec2022)	V1872106P2-80-1<br>(06Dec2022)<br>V1872106P2-84-1<br>(06Dec2022)			
Sodium Heparin Plasma	BioIVT HMN769591		NA	V1872106P2-92-1<br>Untreated QCs<br>(16Dec2022)	37 (LTS),<br>40 (LTS),<br>42 (LTS),<br>43 (LTS)
Deionized Water stored at -20°C in polypropylene	NA	V1872016P2-79-1<br>(06Dec2022)			
Ertapenem in ~0.1M MES Buffer, pH 6.4 stored at -20 °C in glass		V1872106P5-51-1 (15Dec2022)			
Treated Human Sodium Heparin Plasma<br>(1:1 MES buffer:plasma)	V1872106P2-94-1		V1872106-95-1<br>Set L<br>(20Dec2022)	V1872106-96-1<br>Set L<br>(20Dec2022)	36
Deionized Water stored at -20°C in polypropylene	V1872106P2-79-1<br>(06Dec2022)<br>V1872106P2-83-1<br>(06Dec2022)	V1872106P2-80-1<br>(06Dec2022)<br>V1872106P2-84-1<br>(06Dec2022)			
Treated Human Sodium Heparin Plasma<br>(1:1 MES buffer:plasma)	V1872106P2-108-2		V1872106P2-107-1<br>Set M<br>(06Jan2023)	V1872106P2-108-1<br>Set M<br>(06Jan2023)	37
Deionized Water stored at -20°C in polypropylene	V1872106P2-97-1<br>(03Jan2023)<br>V1872106P2-99-1<br>(03Jan2023)	V1872106P2-98-1<br>(03Jan2023)<br>V1872106P2-105-1<br>(05Jan2023)			
Treated Human Sodium Heparin Plasma<br>(1:1 MES buffer:plasma)	V1872106P5-83-1		V1872106P2-110-1<br>Set N<br>(09Jan2023)	V1872106P2-111-1<br>Set N<br>(09Jan2023)	40
Deionized Water stored at -20°C in polypropylene	V1872106P2-97-1<br>(03Jan2023)<br>V1872106P2-99-1<br>(03Jan2023)	V1872106P2-98-1<br>(03Jan2023)<br>V1872106P2-105-1<br>(05Jan2023)			

Table 4: Calibration Standards and QC Samples Preparation (Continued)

Critical Solutions	Lot # for Standards (Prep Date)	Lot # for QCs (Prep Date)	Standards Lot # (Prep Date)	QCS Lot # (Prep Date)	Runs used
Treated Human Sodium Heparin Plasma (1:1 MES buffer:plasma)	V1872106P2-108-2		V1872106P2-112-1 Set O (13Jan2023)	V1872106P2-113-1 Set O (13Jan2023)	42
Deionized Water stored at -20°C in polypropylene	V1872106P2-97-1 (03Jan2023) V1872106P2-99-1 (03Jan2023)	V1872106P2-98-1 (03Jan2023) V1872106P2-105-1 (05Jan2023)			
Treated Human Sodium Heparin Plasma (1:1 MES buffer:plasma)	V1872106P2-117-1		V1872106P2-114-1 Set P (20Jan2023)	V1872106P2-115-1 Set P (20Jan2023)	43
Deionized Water stored at -20°C in polypropylene	V1872106P2-97-1 (03Jan2023) V1872106P2-99-1 (03Jan2023)	V1872106P2-98-1 (03Jan2023) V1872106P2-105-1 (05Jan2023)			
Human Sodium Heparin Plasma	Bio IVT HMN444012		NA	V1872106P2-116-1 Untreated QCs (20Jan2023)	446 (LTS), 47 (LTS)
Deionized Water stored at -20°C in polypropylene	NA	V1872106P2-97-1 (03Jan2023)			
Treated Human Sodium Heparin Plasma (1:1 MES buffer:plasma)	V1872106P2-117-1		V1872106P2-126-1 Set Q (02Feb2023)	V1872106P2-127-1 Set Q (02Feb2023)	46
Deionized Water stored at -20°C in polypropylene	V1872106-120-1 (02Feb2023) V1872106P2-122-1 (02Feb2023)	V1872106-121-1 (02Feb2023) V1872106P2-123-1 (02Feb2023)			
Treated Human Sodium Heparin Plasma (1:1 MES buffer:plasma)	V1872106P5-137-1		V1872106P2-128-1 Set R (16Feb2023)	V1872106P2-129-1 Set R (16Feb2023)	47
Deionized Water stored at -20°C in polypropylene	V1872106-120-1 (02Feb2023) V1872106P2-122-1 (02Feb2023)	V1872106-121-1 (02Feb2023) V1872106P2-123-1 (02Feb2023)			

Table 5: Matrix Description

Matrix	Source Lot #	Date Received
Treated Human Sodium Heparin Plasma (1:1 MES Buffer:Plasma)<br>V1872106P1-18-2<br>V1872106P2-28-1<br>V1872106P2-38-2<br>V1872106P2-56-1<br>V1872106P2-64-2<br>V1872106P2-66-2	BioIVT<br>Combo-43-1<br>(HMN769588, HMN769589, HMN769590, HMN769592, HMN769593, HMN769594, HMN769595, HMN769596, HMN769597, HMN769598, HMN769600, HMN769601, HMN769602, HMN769604, HMN769605)	Mar2022
Treated Human Sodium Heparin Plasma (1:1 MES Buffer:Plasma)<br>V1872106P5-35-1	BioIVT<br>Combo-73-1<br>(BC42768HP-1, BC42768HP-2, BC42768HP-3, BC42768HP-4, BC42768HP-5, BC42768HP-6	Oct2022
Treated Human Sodium Heparin Plasma (1:1 MES Buffer:Plasma)<br>V1872106P2-88-2	BioIVT<br>HMN444012	Aug2020
Treated Human Sodium Heparin Plasma (1:1 MES Buffer:Plasma)<br>V1872106P2-94-1	BioIVT<br>HMN444010	Aug2020
Treated Human Sodium Heparin Plasma (1:1 MES Buffer:Plasma)<br>V1872106P2-108-2	BioIVT<br>HMN444008	Aug2020
Treated Human Sodium Heparin Plasma (1:1 MES Buffer:Plasma)<br>V1872106P2-117-1	BioIVT<br>HMN444012	Aug2020

Table 5: Matrix Description (Continued)

Matrix	Source Lot #	Date Received
Treated Human Sodium Heparin Plasma (1:1 MES Buffer:Plasma)<br>V1872106P5-83-1	BioIVT<br>HMN444009	Aug2020
Treated Human Sodium Heparin Plasma (1:1 MES Buffer:Plasma)<br>V1872106P5-137-1	BioIVT<br>HMN795696	Apr2022
Human Sodium Heparin Plasma	BioIVT HMN769591	Mar2022

Table 6: Summary of Results and Conclusions

Methodology – TP-80194	Method Performance – LC-MS/MS	Hyperlink – N/A	Comments
Matrix	Plasma		
Anticoagulant	Sodium heparin		
Assay Volume	100 mcL		
**Selectivity**			
Analyte	Zidebactam	Summary tables<br>Table 9<br><br>Report text<br>Section 11.3.1	
Internal Standard	Cefotaxime	Summary tables<br>Table 9<br><br>Report text<br>Section 11.3.1	
Matrix Effect<br>(6 spiked matrix lots)	Complies	Summary tables<br>Table 10<br><br>Report text<br>Section 11.3.2	
Interference	Complies	Summary tables<br>Table 11<br><br>Report text<br>Section 11.3.3	
Hemolytic Effect Evaluation	Complies	Summary tables<br>Table 12<br><br>Report text<br>Section 11.3.4	
Lipemic Effect Evaluation	Complies	Summary tables<br>Table 13<br><br>Report text<br>Section 11.3.4	
Carryover	Assessed with every run with standards and QCs	Summary tables<br>Table 8<br><br>Report text<br>Section 11.3.5	
**Linearity**			
Weighting	$1/x^2$	Report text<br>Section 11.4	
Bias at LLOQ	-3.6%	Summary tables<br>Table 14<br><br>Report text<br>Section 11.4	

Table 6: Summary of Results and Conclusions (Continued)

Methodology – TP-80194	Method Performance – LC-MS/MS	Hyperlink – N/A	Comments
Bias above LLOQ	-9.4 to 9.0%	Summary tables<br>Table 14<br><br>Report text<br>Section 11.4	
Bioanalytical Range	Complies<br>0.0500 – 50.0 mcg/mL	Summary tables<br>Table 14<br><br>Report text<br>Section 11.4	
**Precision**			
Intra-assay	0.6 to 8.9% (LLOQ);<br>0.9 to 3.4% (above LLOQ)	Summary tables<br>Table 15<br><br>Report text<br>Section 11.5	
Inter-assay	10.1% (LLOQ);<br>3.5 to 4.8% (above LLOQ)	Summary tables<br>Table 16<br><br>Report text<br>Section 11.5	
**Accuracy**			
Intra-assay	-9.6 to 11.4% (LLOQ);<br>-9.1 to 12.0% (above LLOQ)	Summary tables<br>Table 15<br><br>Report text<br>Section 11.5	
Inter-assay	-2.0% (LLOQ);<br>-5.1 to 6.0% (above LLOQ)	Summary tables<br>Table 16<br><br>Report text<br>Section 11.5	
**Large Run Size**	Determined to be 125 samples.	Summary tables<br>Table 14 and Table 15<br><br>Report text<br>Section 11.7	

Table 6: Summary of Results and Conclusions (Continued)

**Methodology – TP-80194**	**Method Performance – LC-MS/MS**	**Hyperlink – N/A**	**Comments**
**Dilution**			
Dilution factor: 2x	Dilution QC: 37.5 mcg/mL<br>Precision: 1.4%<br>Accuracy: 1.9%	Summary tables<br>Table 19<br><br>Report text<br>Section 11.8	
Dilution factor: 10x	Dilution QC: 400 mcg/mL<br>Precision: 1.3%<br>Accuracy: -2.8%	Summary tables<br>Table 19<br><br>Report text<br>Section 11.8	
**Stability**			
Matrix (in polypropylene tubes)			
Freeze/Thaw (-20 °C/ice-water bath) with and without the presence of comed[2]	Demonstrated for 4 cycles for treated QCs	Summary tables<br>Table 20<br><br>Report text<br>Section 11.9	
Freeze/Thaw (-70 °C/ice-water bath) with and without the presence of comed[2]	Demonstrated for 4 cycles for treated QCs<br>Demonstrated for 2 cycles for untreated QCs	Summary tables<br>Table 20<br><br>Report text<br>Section 11.9	
Bench-top in wet ice without the presence of comed[2]	Demonstrated for 19.75 hours for treated QCs<br>Demonstrated for 4 hours for untreated QCs	Summary tables<br>Table 22<br><br>Report text<br>Section 11.11	
Bench-top in wet ice with the presence of comed[2]	Demonstrated for 8.25 hours for treated QCs<br>Demonstrated for 4 hours for untreated QCs	Summary tables<br>Table 22<br><br>Report text<br>Section 11.11	
Long-term (-20 °C) with and without the presence of comed[2]	Demonstrated instability at 62 days	Summary tables<br>Table 24<br><br>Report text<br>Section 11.13	
Long-term (-70 °C) with and without the presence of comed[2]	Demonstrated for 90 days for treated QCs<br>Demonstrated for 27 days for untreated QCs without comed[2]<br>Demonstrated for 35 days for untreated QCs with comed[2]	Summary tables<br>Table 24<br><br>Report text<br>Section 11.13	

Table 6: Summary of Results and Conclusions (Continued)

**Methodology – TP-80194**	**Method Performance – LC-MS/MS**	**Hyperlink – N/A**	**Comments**
Whole Blood	No instability observed in ice-water bath or at room temperature in polyethylene terephthalate for up to 1.5 hour.	Summary tables<br>Table 26<br><br>Report text<br>Section 11.15	
Processed Matrix polypropylene			
Room Temperature	Demonstrated for 3 days 0 hours	Summary tables<br>Table 27<br><br>Report text<br>Section 11.16	
Refrigeration	Demonstrated for 3 days 0 hours	Summary tables<br>Table 28<br><br>Report text<br>Section 11.17	
**Individual Sample Reinjection**	Reproducible	Summary tables<br>Table 29<br><br>Report text<br>Section 11.18	
**Whole Run Reinjection Integrity**	Reproducible<br>Duration 2 days 7 hours	Summary tables<br>Table 30<br><br>Report text<br>Section 11.19	
**Column Ruggedness**	Reproducible	Summary tables<br>Table 30<br><br>Report text<br>Section 11.20	
**Extraction Recovery**			
Zidebactam	Overall 95.1%	Summary tables<br>Table 31<br><br>Report text<br>Section 11.21	
Cefotaxime (IS)	Overall 99.0%	Summary tables<br>Table 32<br><br>Report text<br>Section 11.21	

Table 6: Summary of Results and Conclusions (Continued)

Methodology – TP-80194	Method Performance – LC-MS/MS	Hyperlink – N/A	Comments
**Solution Stability**			
**Solution, Primary polypropylene**			
Zidebactam in deionized water (RT)	Stable 17.75 hours	Summary tables Table 33	V1872106U2
Zidebactam in deionized water (-20 °C)	Stable 25 days<br>Stable 61 days	Summary tables Table 34	V1872106U2
**Solution, Working polypropylene**			
Zidebactam Spike in deionized water (RT)	Stable 17.75 hours	Summary tables Table 35	V1872106U2
Zidebactam Spike in deionized water (-20 °C)	Stable 25 days<br>Stable 61 days	Summary tables Table 36	V1872106U2
Cefotaxime (IS) in deionized water in glass (RT)	Stable 15.5 hours<br>Stable 19.0 hours	Summary tables Table 37	V1872106U1

Table 7: Summary of Validation Run Objectives and Outcome

Run ID	Instrument ID	Extraction Date	Assay Date	Run Objective(s)	Accepted or Rejected	Comment
2	LC/MS-24	10-Aug-2022	10-Aug-2022	Benchtop solution stability	Rejected	Failed due to high CV
5	LC/MS-31	15-Aug-2022	15-Aug-2022	P&A, hemolytic and lipemic QC evaluation, hemolytic and lipemic plasma screen, comed[2] interference, individual sample reinjection reproducibility	Accepted	
7	LC/MS-31	17-Aug-2022	17-Aug-2022	P&A, dilution integrity, and IS cross-talk	Rejected	Preparation error in standard preparation caused the standards to be 10x lower
8	LC/MS-24	18-Aug-2022	18-Aug-2022	P&A, dilution integrity, and IS cross-talk	Rejected	[a, b]
9	LC/MS-23	22-Aug-2022	23-Aug-2022	P&A, dilution integrity, and IS cross-talk	Accepted	
10	LC/MS-23	23-Aug-2022	23-Aug-2022	Matrix effect and selectivity	Accepted	
12	LC/MS-24	26-Aug-2022	26-Aug-2022	P&A, freeze-thaw stability with and without comed[2], bench-top stability with and without comed[2], and large run size	Accepted	Freeze/Thaw and benchtop matrix stability failed for low QC, and passed for high QC
13	LC/MS-24	26-Aug-2022	27-Aug-2022	P&A, extraction recovery, freeze/thaw and bench-top stability for dilution QC only	Accepted	[c, d]
14	LC/MS-24	29-Aug-2022	29-Aug-2022	Comed[2] qualification and processed sample stability	Accepted	Low comed[2] QC failed
15	LC/MS-24	26-Aug-2022	29-Aug-2022	Whole batch reinjection and column ruggedness	Accepted	
22	LC/MS-31	07-Sep-2022	07-Sep-2022	Whole blood stability	Accepted	
24	LC/MS-42	20-Sep-2022	20-Sep-2022	Freeze thaw stability with and without comed[2] and bench-top stability with and without comed[2]	Accepted	
25	LC/MS-29	12 Oct 2022	12 Oct 2022	Long-term matrix stability	Rejected	[e]
30	LC/MS-24	09 Nov 2022	09 Nov 2022	Long-term matrix stability	Accepted	Long-term stability at -20 °C did not meet criteria
31	LC/MS-23	07-Dec-2022	07-Dec-2022	Long-term matrix stability	Accepted	
34	LC/MS-23	07-Dec-2022	07-Dec-2022	WIS bench-top solution stability	Accepted	
36	LC/MS-22	20-Dec-2022	20-Dec-2022	Untreated freeze/thaw stability with comed[2] and untreated bench-top stability with comed[2]	Accepted	
37	LC/MS-22	06-Jan-2023	06-Jan-2023	Untreated long-term matrix stability with comed[2]	Accepted	This run was also used to qualify treated comed[2] QCs and that data will not be reported

Table 7: Summary of Validation Run Objectives and Outcome (Continued)

Run ID	Instrument ID	Extraction Date	Assay Date	Run Objective(s)	Accepted or Rejected	Comment
40	LC/MS-22	09-Jan-2023	09-Jan-2023	Untreated long-term matrix stability with comed[2]	Accepted	
42	LC/MS-22	13-Jan-2023	15-Jan-2023	Untreated long-term matrix stability with comed[2]	Accepted	-70 °C LTS failed criteria for being too high at the %Bias. This is likely a prep error and additional LTS was performed using these QCs
43	LC/MS-39	20-Jan-2023	21-Jan-2023	Untreated long-term matrix stability with and without comed[2]	Accepted	This run was also used to qualify untreated QCs and that data will not be reported
46	LC/MS-22	06-Feb-2023	07-Feb-2023	Untreated long-term matrix stability, untreated freeze/thaw stability, and untreated bench-top stability	Accepted	
47	LC/MS-39	16-Feb-2023	17-Feb-2023	Untreated long-term matrix stability	Accepted	

Runs 1, 16, 20, 21, 29, 33, 39, and 41 were used for a spike check and will not be reported.
Runs 3, 4, 6, 17, 19, 28, and 32 were used for a primary check and will not be reported.
Runs 38 and 45 were used for a primary solution check and spike linearity check and will not be reported.
Runs 11, 23, 26, 27, and 44 were not used.
Run 18 was used to qualify standards and QCs and will not be reported.
Run 35 was used to qualify untreated QCs and will not be reported.
[a]IS cross-talk failed due to WIS being added.
[b]LLOQ and low QCs all fail with average %Bias of 32.0% and 20.7% respectively and suspect the preparation of standard using frozen treated plasma may not be sufficient to stabilize drug in the prepared standards, resulting in the QC pools having high %Bias.
[c]ARQC freeze/thaw and bench-top pass acceptance criteria, however freeze/thaw and bench-top stability do not pass for QC 0.150 in run 12.
[d]ARQC freeze/thaw and bench-top evaluation pass the study criteria and will be reported. The low and high QC freeze/thaw and bench-top stability were repeated with less freeze/thaw cycles and shorter bench-top duration to confirm the stability.
[e] Due to the batch not meeting acceptance criteria in Run 25, the 34 days long-term stability evaluation of zidebactam in treated human plasma cannot be accepted and were not reported.

Table 8: Carryover

Run ID	Result (Extracted Blank after High Standard)	Carryover Results for Internal Standard
5	Seq 15 is <20.0% of the LLOQ and Seq 93 is 50.4% of the LLOQ.	Seq 6 and Seq 82 are < 5.0% internal standard peak of acceptable standards.
7	Seq 13 and Seq 67 are <20.0% of the LLOQ.	Seq 4 and Seq 58 are < 5.0% internal standard peak of acceptable standards.
8	Seq 13 and Seq 67 are <20.0% of the LLOQ.	Seq 4 and Seq 58 are < 5.0% internal standard peak of acceptable standards.
9	Seq 13 and Seq 67 are <20.0% of the LLOQ.	Seq 4 and Seq 58 are < 5.0% internal standard peak of acceptable standards.
12	Seq 13 and Seq 125 are <20.0% of the LLOQ.	Seq 4 and Seq 116 are < 5.0% internal standard peak of acceptable standards.
13	Seq 13 is <20.0% of the LLOQ and Seq 94 is 35.5% of the LLOQ.	Seq 4 and Seq 85 are < 5.0% internal standard peak of acceptable standards.
14	Seq 13 and Seq 73 are <20.0% of the LLOQ.	Seq 4 and Seq 64 are < 5.0% internal standard peak of acceptable standards.
15	Seq 13 is <20.0% of the LLOQ and Seq 52 is 37.8% of the LLOQ.	Seq 4 and Seq 43 are < 5.0% internal standard peak of acceptable standards.
24	Seq 13 and Seq 107 are <20.0% of the LLOQ.	Seq 4 and Seq 98 are < 5.0% internal standard peak of acceptable standards.
30	Seq 13 and Seq 95 are <20.0% of the LLOQ.	Seq 4 and Seq 86 are < 5.0% internal standard peak of acceptable standards.
31	Seq 13 and Seq 65 are <20.0% of the LLOQ.	Seq 4 and Seq 56 are < 5.0% internal standard peak of acceptable standards.
36	Seq 13 and Seq 55 are <20.0% of the LLOQ.	Seq 4 and Seq 46 are < 5.0% internal standard peak of acceptable standards.
37	Seq 13 and Seq 56 are <20.0% of the LLOQ.	Seq 4 and Seq 47 are < 5.0% internal standard peak of acceptable standards.
40	Seq 13 and Seq 44 are <20.0% of the LLOQ.	Seq 4 and Seq 35 are < 5.0% internal standard peak of acceptable standards.
42	Seq 13 and Seq 44 are <20.0% of the LLOQ.	Seq 4 and Seq 35 are < 5.0% internal standard peak of acceptable standards.
43	Seq 13 and Seq 56 are <20.0% of the LLOQ.	Seq 4 and Seq 47 are < 5.0% internal standard peak of acceptable standards.
46	Seq 13 and Seq 68 are <20.0% of the LLOQ.	Seq 4 and Seq 59 are < 5.0% internal standard peak of acceptable standards.
47	Seq 13 and Seq 44 are <20.0% of the LLOQ.	Seq 4 and Seq 35 are < 5.0% internal standard peak of acceptable standards.

Carryover is evaluated within each run. It consists of measuring the response for each analyte and if it exceeds 20.0% of the response of LLOQ standard or other sample reflecting the lower sensitivity limit required, a carryover assessment is completed, and affected samples are rejected. The internal standard carryover evaluation is assessed within each run and if it exceeds 5.0% of the average internal standard response of acceptable standards and QCs, impact is evaluated within the run.

Carryover evaluation of the internal standard are within 5.0% in each run. Calibration Standards and QCs are arranged in a way to mitigate carryover in all validation runs, and no QCs was deactivated due to carry-over assessments.

Table 9: Selectivity - Blank Assessment

Run 10 Selectivity Blank Screen

Blank ID with or without IS	Blank Lot Number	Peak Area of Analyte	Peak Area of Internal Standard	Percent of LLOQ Peak Area	Percent of LLOQ Peak Area Ratio	Percent of IS in Blanks without IS
Blank 1 NO IS	V1872106P2-36-1	0	0	0.0	NA	0.0
Blank 2 NO IS	V1872106P2-36-2	0	0	0.0	NA	0.0
Blank 3 NO IS	V1872106P2-36-3	0	0	0.0	NA	0.0
Blank 4 NO IS	V1872106P2-36-4	0	0	0.0	NA	0.0
Blank 5 NO IS	V1872106P2-36-5	0	0	0.0	NA	0.0
Blank 6 NO IS	V1872106P2-36-6	0	0	0.0	NA	0.0
Blank 1	V1872106P2-36-1	0	4258422	0.0	0.0	NA
Blank 2	V1872106P2-36-2	0	4223577	0.0	0.0	NA
Blank 3	V1872106P2-36-3	0	4217270	0.0	0.0	NA
Blank 4	V1872106P2-36-4	0	4192238	0.0	0.0	NA
Blank 5	V1872106P2-36-5	0	4201569	0.0	0.0	NA
Blank 6	V1872106P2-36-6	0	4112414	0.0	0.0	NA

Note: Average IS response are from all of the accepted standards and QCs

Criteria: A minimum of six different sources of sodium heparin treated plasma were screened. To be deemed acceptable, the peak response observed must be ≤ 20.0% of the mean response of the lower limit of quantitation (LLOQ) and ≤ 5.0% of the mean response of the internal standard. If any lots fail, further evaluation is needed.

Results: All matrix lots were found to be free of any significant interference at the retention times of the analytes of interest.

Table 10: Matrix Effect

Run Number: 10								
	Low QC: 0.15 mcg/mL				High QC: 37.5 mcg/mL			
	Drug Area	IS Area	Drug/IS Ratio	Average Drug/IS Ratio	Drug Area	IS Area	Drug/IS Ratio	Average Drug/IS Ratio
ME1	58892	4230537	0.0139	0.0140	13405936	4280150	3.1321	3.0892
	59251	4248188	0.0139		12775703	4228586	3.0213	
	58281	4118700	0.0142		14373053	4648991	3.0916	
	60304	4292265	0.0140		13066734	4198952	3.1119	
ME2	57070	4143333	0.0138	0.0138	13199705	4265537	3.0945	3.0951
	58717	4288509	0.0137		13537430	4235888	3.1959	
	56429	4219198	0.0134		13218247	4284350	3.0852	
	60990	4297039	0.0142		12797325	4258839	3.0049	
ME3	59470	4453119	0.0134	0.0134	12931467	4190265	3.0861	3.0692
	57175	4273528	0.0134		13121291	4336659	3.0257	
	58214	4398229	0.0132		12816304	4165250	3.0770	
	58302	4325222	0.0135		12915475	4182458	3.0880	
ME4	59646	4363534	0.0137	0.0136	13581709	4335589	3.1326	3.0866
	59969	4353344	0.0138		13409190	4346911	3.0848	
	58408	4334281	0.0135		13320774	4366725	3.0505	
	57798	4262956	0.0136		13691869	4447747	3.0784	
ME5	59872	4250492	0.0141	0.0136	13339958	4298780	3.1032	3.0620
	59199	4311672	0.0137		12959629	4334205	2.9901	
	56367	4245323	0.0133		12821983	4167010	3.0770	
	57668	4319354	0.0134		12772102	4149815	3.0778	
ME6	56878	4186003	0.0136	0.0134	13049249	4223298	3.0898	3.0911
	55182	4221045	0.0131		12734296	4038023	3.1536	
	57065	4293953	0.0133		12980418	4271292	3.0390	
	54915	4073896	0.0135		13045818	4232668	3.0822	
**Mean**				0.0136				3.0822
**N**				6				6
**SD**				0.0002				0.0134
**%CV**				1.8				0.4

Meets Acceptance Criteria: %CV of average drug/IS ratio among 6 lots must be <15.0%.
Met criteria at Low QC level: **YES**
Met criteria at High QC level: **YES**

Table 11: Interference Evaluation

Run Date	Curve Number	Comed1[2] low 0.150 µg/mL	%Bias	Comed1[2] high 37.5 µg/mL	%Bias
15-Aug-2022	5	0.154	2.7	37.4	-0.3
		0.152	1.3	38.5	2.7
		0.158	5.3	38.8	3.5
		0.162	8.0	37.4	-0.3
		0.162	8.0	38.4	2.4
		0.156	4.0	38.3	2.1
Mean		0.157		38.1	
SD		0.00413		0.592	
%CV		2.6		1.6	
%Bias		4.7		1.6	
n		6		6	

Criteria: To be considered acceptable, the mean %Bias at each level was also required to be within ± 15.0% and the %CV was required to be ≤ 15.0%.

Results: The acceptance criteria were met.

Table 12: Hemolytic Effect Evaluation

Run Date	Curve Number	Hemolytic QC low 0.150 µg/mL	%Bias	Hemolytic QC high 37.5 µg/mL	%Bias
15-Aug-2022	5	0.145	-3.3	35.6	-5.1
		0.148	-1.3	36.0	-4.0
		0.150	0.0	36.5	-2.7
		0.144	-4.0	37.0	-1.3
		0.148	-1.3	36.9	-1.6
		0.154	2.7	36.2	-3.5
Mean		0.148		36.4	
SD		0.00360		0.539	
%CV		2.4		1.5	
%Bias		-1.3		-2.9	
n		6		6	

Criteria: To be considered acceptable, the mean %Bias at each level was also required to be within ± 15.0% and the %CV was required to be ≤ 15.0%.

Results: The acceptance criteria were met.

Table 13: Lipemic Effect Evaluation

Run Date	Curve Number	Lipemic QC low 0.150 µg/mL	%Bias	Lipemic QC high 37.5 µg/mL	%Bias
15-Aug-2022	5	0.155	3.3	37.3	-0.5
		0.159	6.0	38.1	1.6
		0.157	4.7	38.4	2.4
		*0.312		38.0	1.3
		0.155	3.3	38.5	2.7
		0.156	4.0	38.2	1.9
With outlier					
Mean		0.182			
SD		0.0635			
%CV		34.9			
%Bias		21.3			
n		6			
Without outlier					
Mean		0.156		38.1	
SD		0.00167		0.426	
%CV		1.1		1.1	
%Bias		4.0		1.6	
n		5		6	
Reason Deactivated					
* Statistical Outlier (t-test, p<0.01)					

Criteria: To be considered acceptable, the mean %Bias at each level was also required to be within ± 15.0% and the %CV was required to be ≤ 15.0%.

Results: The acceptance criteria were met.

Table 14: Summary of Standard Curves

Analytical Performance: Back-Calculated Concentrations (µg/mL) of Zidebactam Calibration Standard in (Human) (Plasma) in (Study V1872106P2)

Assay Date	Analytical Run Number	Cal 0.0500 0.0500 µg/mL	Cal 0.100 0.100 µg/mL	Cal 1.00 1.00 µg/mL	Cal 5.00 5.00 µg/mL	Cal 10.0 10.0 µg/mL	Cal 20.0 20.0 µg/mL	Cal 40.0 40.0 µg/mL	Cal 50.0 50.0 µg/mL
15-Aug-2022	5	0.0487	0.105	0.998	4.93	10.1	19.4	40.0	45.6
		0.0467	0.113	1.03	5.01	10.1	20.3	40.9	47.9
23-Aug-2022	9	0.0502	*0.134	1.06	5.41	10.4	20.3	37.1	45.8
		0.0493	*0.134	1.11	5.21	10.4	19.9	37.0	43.2
26-Aug-2022	12	0.0456	0.109	1.10	5.52	9.90	19.1	35.7	43.6
		0.0485	0.113	1.09	*5.92	10.5	19.9	37.6	45.4
27-Aug-2022	13	0.0491	0.107	1.10	5.33	10.4	19.3	39.1	46.0
		0.0471	0.106	1.05	5.28	10.2	18.8	36.2	44.8
Mean		0.0482	0.109	1.07	5.24	10.3	19.6	38.0	45.3
S.D.		0.00154	0.00349	0.0397	0.211	0.207	0.557	1.86	1.47
%CV		3.2	3.2	3.7	4.0	2.0	2.8	4.9	3.2
%Bias		-3.6	9.0	7.0	4.8	3.0	-2.0	-5.0	-9.4
n		8	6	8	7	8	8	8	8
Reason Deactivated									
* Excluded due to being outside acceptance criteria									

Calibration Curve Parameters for Zidebactam Calibration Standards in Human Plasma (Study V1872106P2)

Run Date	Curve Number	Slope	Intercept	R-Squared	LLOQ	ULOQ
15-Aug-2022	5	0.06625	-0.00055	0.9969	0.0500	50.0
23-Aug-2022	9	0.13604	-0.00409	0.9947	0.0500	50.0
26-Aug-2022	12	0.04082	0.00031	0.9904	0.0500	50.0
27-Aug-2022	13	0.04210	0.00029	0.9944	0.0500	50.0

See Table 7 for runs not used for precision/accuracy evaluation.

Table 15: Precision and Accuracy (Intra-Assay)

Human Plasma Zidebactam Concentrations in Quality Controls for 4 separate batch runs (Study V1872106P2)

Run Date	Curve Number	QCS 0.0500 0.0500 µg/mL	%Bias	QCS 0.150 0.150 µg/mL	%Bias	QCS 25.0 25.0 µg/mL	%Bias	QCS 37.5 37.5 µg/mL	%Bias
15-Aug-2022	5	0.0546	9.2	0.157	4.7	25.3	1.2	38.2	1.9
		#0.0650	30.0	0.161	7.3	25.2	0.8	38.6	2.9
		0.0530	6.0	0.153	2.0	25.6	2.4	37.7	0.5
		0.0572	14.4	0.154	2.7	25.2	0.8	37.9	1.1
		0.0529	5.8	0.158	5.3	25.5	2.0	38.4	2.4
		0.0516	3.2	0.160	6.7	24.9	-0.4	38.1	1.6
Intrarun Mean		0.0557		0.157		25.3		38.2	
Intrarun SD		0.00494		0.00319		0.248		0.327	
Intrarun %CV		8.9		2.0		1.0		0.9	
Intrarun %Bias		11.4		4.7		1.2		1.9	
n		6		6		6		6	
23-Aug-2022	9	0.0490	-2.0	0.168	12.0	26.0	4.0	37.0	-1.3
		0.0493	-1.4	0.167	11.3	24.3	-2.8	34.9	-6.9
		0.0489	-2.2	0.172	14.7	25.5	2.0	35.7	-4.8
		0.0493	-1.4	0.168	12.0	24.4	-2.4	35.5	-5.3
		0.0495	-1.0	0.168	12.0	24.7	-1.2	33.8	-9.9
		0.0487	-2.6	0.164	9.3	25.2	0.8	35.3	-5.9
Intrarun Mean		0.0491		0.168		25.0		35.4	
Intrarun SD		0.000299		0.00256		0.668		1.05	
Intrarun %CV		0.6		1.5		2.7		3.0	
Intrarun %Bias		-1.8		12.0		0.0		-5.6	
n		6		6		6		6	
26-Aug-2022	12	0.0470	-6.0	0.149	-0.7	23.6	-5.6	34.3	-8.5
		0.0443	-11.4	0.149	-0.7	23.3	-6.8	35.1	-6.4
		0.0444	-11.2	0.158	5.3	24.0	-4.0	34.4	-8.3
		0.0439	-12.2	0.157	4.7	23.6	-5.6	34.5	-8.0
		0.0454	-9.2	0.152	1.3	24.9	-0.4	36.0	-4.0
		0.0463	-7.4	0.153	2.0	24.2	-3.2	34.7	-7.5
Intrarun Mean		0.0452		0.153		23.9		34.8	
Intrarun SD		0.00124		0.00385		0.572		0.638	
Intrarun %CV		2.7		2.5		2.4		1.8	
Intrarun %Bias		-9.6		2.0		-4.4		-7.2	
n		6		6		6		6	
27-Aug-2022	13	0.0429	-14.2	0.167	11.3	23.0	-8.0	34.7	-7.5
		0.0476	-4.8	0.154	2.7	23.9	-4.4	35.0	-6.7
		0.0468	-6.4	0.153	2.0	23.8	-4.8	33.7	-10.1
		0.0470	-6.0	0.163	8.7	23.6	-5.6	33.9	-9.6
		0.0464	-7.2	0.158	5.3	24.8	-0.8	33.7	-10.1
		0.0440	-12.0	0.157	4.7	23.4	-6.4	33.5	-10.7
Intrarun Mean		0.0458		0.159		23.8		34.1	
Intrarun SD		0.00188		0.00539		0.606		0.615	
Intrarun %CV		4.1		3.4		2.5		1.8	
Intrarun %Bias		-8.4		6.0		-4.8		-9.1	
n		6		6		6		6	
# > 20%Bias									

Criteria: At each level, the %CV was required to be ≤ 15.0%, except at the LLOQ level, where ≤ 20.0% was accepted. The mean %Bias at each level was also required to be within ± 15.0%, except at the LLOQ, where ± 20.0% was accepted.

Results: The acceptance criteria were met. Run size (n=125) was completed in run 12 and met acceptance criteria.

Table 16: Precision and Accuracy (Inter-Assay)

Human Plasma Zidebactam Concentrations in Quality Controls for 4 separate batch runs (Study V1872106P2)

Run Date	Curve Number	QCS 0.0500 0.0500 µg/mL	%Bias	QCS 0.150 0.150 µg/mL	%Bias	QCS 25.0 25.0 µg/mL	%Bias	QCS 37.5 37.5 µg/mL	%Bias
15-Aug-2022	5	0.0546	9.2	0.157	4.7	25.3	1.2	38.2	1.9
		#0.0650	30.0	0.161	7.3	25.2	0.8	38.6	2.9
		0.0530	6.0	0.153	2.0	25.6	2.4	37.7	0.5
		0.0572	14.4	0.154	2.7	25.2	0.8	37.9	1.1
		0.0529	5.8	0.158	5.3	25.5	2.0	38.4	2.4
		0.0516	3.2	0.160	6.7	24.9	-0.4	38.1	1.6
23-Aug-2022	9	0.0490	-2.0	0.168	12.0	26.0	4.0	37.0	-1.3
		0.0493	-1.4	0.167	11.3	24.3	-2.8	34.9	-6.9
		0.0489	-2.2	0.172	14.7	25.5	2.0	35.7	-4.8
		0.0493	-1.4	0.168	12.0	24.4	-2.4	35.5	-5.3
		0.0495	-1.0	0.168	12.0	24.7	-1.2	33.8	-9.9
		0.0487	-2.6	0.164	9.3	25.2	0.8	35.3	-5.9
26-Aug-2022	12	0.0470	-6.0	0.149	-0.7	23.6	-5.6	34.3	-8.5
		0.0443	-11.4	0.149	-0.7	23.3	-6.8	35.1	-6.4
		0.0444	-11.2	0.158	5.3	24.0	-4.0	34.4	-8.3
		0.0439	-12.2	0.157	4.7	23.6	-5.6	34.5	-8.0
		0.0454	-9.2	0.152	1.3	24.9	-0.4	36.0	-4.0
		0.0463	-7.4	0.153	2.0	24.2	-3.2	34.7	-7.5
27-Aug-2022	13	0.0429	-14.2	0.167	11.3	23.0	-8.0	34.7	-7.5
		0.0476	-4.8	0.154	2.7	23.9	-4.4	35.0	-6.7
		0.0468	-6.4	0.153	2.0	23.8	-4.8	33.7	-10.1
		0.0470	-6.0	0.163	8.7	23.6	-5.6	33.9	-9.6
		0.0464	-7.2	0.158	5.3	24.8	-0.8	33.7	-10.1
		0.0440	-12.0	0.157	4.7	23.4	-6.4	33.5	-10.7
Mean Concentration Found (µg/mL)		0.0490		0.159		24.5		35.6	
Inter-run SD		0.00496		0.00662		0.849		1.70	
Inter-run %CV		10.1		4.2		3.5		4.8	
Inter-run %Bias		-2.0		6.0		-2.0		-5.1	
n		24		24		24		24	
# > 20%Bias									

Criteria: At each level, the %CV was required to be ≤ 15.0%, except at the LLOQ level, where ≤ 20.0% was accepted. In addition, the mean %Bias at each level was required to be within ± 15.0% from nominal value, except at the LLOQ, where ± 20.0% was accepted.

Results: The acceptance criteria were met.

Table 17: Standard and QC of Failed Runs

Analytical Performance: Back-Calculated Concentrations (µg/mL) of Zidebactam Calibration Standard in (Human) (Plasma) in (Study V1872106P2) (Rejected Runs)

Assay Date	Analytical Run Number	Cal 0.0500 0.0500 µg/mL	Cal 0.100 0.100 µg/mL	Cal 1.00 1.00 µg/mL	Cal 5.00 5.00 µg/mL	Cal 10.0 10.0 µg/mL	Cal 20.0 20.0 µg/mL	Cal 40.0 40.0 µg/mL	Cal 50.0 50.0 µg/mL
17-Aug-2022	7	0.0501	0.0998	0.985	4.92	10.1	19.7	39.3	49.9
		0.0502	0.0994	0.957	5.04	10.2	19.8	40.6	53.6
18-Aug-2022	8	0.0486	0.105	1.06	5.31	10.3	19.8	37.4	46.4
		0.0503	0.0983	1.05	5.26	10.3	19.7	38.0	45.8
12-Oct-2022	25	0.0512	0.104	0.936	5.36	10.0	21.3	41.7	51.9
		0.0477	0.102	0.884	4.98	9.77	19.2	40.2	48.9
Mean		0.0497	0.101	0.979	5.15	10.1	19.9	39.5	49.4
S.D.		0.00128	0.00269	0.0678	0.187	0.204	0.714	1.63	3.04
%CV		2.6	2.7	6.9	3.6	2.0	3.6	4.1	6.2
%Bias		-0.6	1.0	-2.1	3.0	1.0	-0.5	-1.3	-1.2
n		6	6	6	6	6	6	6	6

Calibration Curve Parameters for Zidebactam Calibration Standards in Human Plasma (Study V1872106P2) (Rejected Runs)

Run Date	Curve Number	Slope	Intercept	R-Squared	LLOQ	ULOQ
17-Aug-2022	7	0.00608	0.00002	0.9993	0.0500	50.0
18-Aug-2022	8	0.05047	-0.00091	0.9969	0.0500	50.0
12-Oct-2022	25	0.05418	-0.00141	0.9969	0.0500	50.0

Human Plasma Zidebactam Concentrations in Quality Controls for 3 separate batch runs (Study V1872106P2) (Rejected Runs)

Run Date	Curve Number	QCS 0.0500 0.0500 µg/mL	%Bias	QCS 0.150 0.150 µg/mL	%Bias	QCS 25.0 25.0 µg/mL	%Bias	QCS 37.5 37.5 µg/mL	%Bias
17-Aug-2022	7	#0.455	810.0	##1.47	880.0	##231	824.0	##350	833.3
		#0.403	706.0	##1.43	853.3	##232	828.0	##347	825.3
		#0.412	724.0	##1.41	840.0	##234	836.0	##349	830.7
		#0.443	786.0	##1.50	900.0	##244	876.0	##358	854.7
		#0.433	766.0	##1.49	893.3	##238	852.0	##355	846.7
		#0.453	806.0	##1.52	913.3	##237	848.0	##351	836.0
Intrarun Mean		0.433		1.47		236		352	
Intrarun SD		0.0216		0.0424		4.77		4.08	
Intrarun %CV		5.0		2.9		2.0		1.2	
Intrarun %Bias		766.0		880.0		844.0		838.7	
n		6		6		6		6	
18-Aug-2022	8	#0.0650	30.0	##0.177	18.0	26.0	4.0	35.9	-4.3
		#0.0669	33.8	##0.184	22.7	24.8	-0.8	35.9	-4.3
		#0.0675	35.0	##0.180	20.0	24.9	-0.4	35.7	-4.8
		#0.0651	30.2	##0.183	22.0	24.7	-1.2	37.0	-1.3
		#0.0663	32.6	##0.185	23.3	24.6	-1.6	36.6	-2.4
		#0.0650	30.0	##0.174	16.0	25.0	0.0	36.0	-4.0
Intrarun Mean		0.0660		0.181		25.0		36.2	
Intrarun SD		0.00109		0.00432		0.510		0.504	
Intrarun %CV		1.7		2.4		2.0		1.4	
Intrarun %Bias		32.0		20.7		0.0		-3.5	
n		6		6		6		6	
12-Oct-2022	25			##0.189	26.0	24.8	-0.8	37.4	-0.3
				##0.191	27.3	23.7	-5.2	36.9	-1.6
Intrarun Mean				0.190		24.3		37.2	
Intrarun %Bias				26.7		-2.8		-0.8	
# > 20%Bias									
## > 15%Bias									

Criteria: For each run, the calculated concentrations of at least 75% of the calibration standards are required to interpolate within ± 15.0% from nominal with a minimum of six calibration standard levels, except for the LLOQ for which it was required to be within ± 20.0%. In order for the run to be acceptable, at least one QC at each concentration level and two-thirds of the total number of QC samples were required to interpolate within ± 15.0% from nominal.

Results: The acceptance criteria were not met. See Table 7 for reasons of failure.

Table 18: Standards and QCs of Non-P&A Runs

Analytical Performance: Back-Calculated Concentrations (µg/mL) of Zidebactam Calibration Standard in (Human) (Plasma) in (Study V1872106P2)

Assay Date	Analytical Run Number	Cal 0.0500 0.0500 µg/mL	Cal 0.100 0.100 µg/mL	Cal 1.00 1.00 µg/mL	Cal 5.00 5.00 µg/mL	Cal 10.0 10.0 µg/mL	Cal 20.0 20.0 µg/mL	Cal 40.0 40.0 µg/mL	Cal 50.0 50.0 µg/mL
29-Aug-2022	14	0.0512	0.0983	1.03	5.19	10.1	20.3	38.5	46.4
		0.0495	0.0981	1.03	5.32	10.2	19.8	39.8	46.6
20-Sep-2022	24	0.0515	*0.0808	0.948	5.42	11.5	21.5	44.2	52.7
		0.0486	*0.0698	*0.831	4.52	9.10	18.3	38.8	44.3
09-Nov-2022	30	0.0490	0.0988	1.04	5.21	10.2	20.0	39.0	47.7
		0.0506	0.102	1.03	5.12	10.2	19.9	38.7	46.7
07-Dec-2022	31	0.0507	0.0989	1.02	5.21	10.3	20.0	39.5	48.9
		*0.114	0.0978	1.01	5.10	9.89	19.9	39.8	47.6
20-Dec-2022	36	0.0485	0.106	1.01	5.39	10.3	19.4	37.4	47.0
		0.0494	0.101	1.07	5.35	11.3	18.9	36.7	43.4
06-Jan-2023	37	0.0503	0.0927	1.00	5.10	10.2	20.1	39.3	45.3
		0.0529	0.0945	1.00	5.28	10.5	20.7	40.3	49.1
09-Jan-2023	40	0.0473	0.0919	0.989	5.16	10.7	21.2	40.7	52.0
		0.0545	0.102	0.924	5.05	9.53	19.6	*49.8	47.9
15-Jan-2023	42	0.0435	0.0891	1.03	5.49	10.4	20.7	*54.9	49.8
		0.0566	0.110	0.969	*6.42	*14.1	18.5	38.5	47.6
21-Jan-2023	43	0.0532	0.0881	0.982	5.21	10.2	20.6	41.3	50.5
		0.0524	0.0900	0.971	5.02	10.2	20.2	40.1	49.1
07-Feb-2023	46	0.0468	0.0945	0.936	4.78	9.85	19.0	38.6	46.5
		0.0538	0.103	1.05	5.19	10.7	20.9	42.6	50.5
17-Feb-2023	47	0.0547	0.0896	1.01	5.33	10.2	20.3	38.0	46.0
		*0.0626	0.0910	1.03	5.43	10.4	20.1	40.9	47.5
Mean		0.0508	0.0969	1.00	5.18	10.3	20.0	39.6	47.9
S.D.		0.00311	0.00607	0.0379	0.223	0.516	0.817	1.75	2.32
%CV		6.1	6.3	3.8	4.3	5.0	4.1	4.4	4.8
%Bias		1.6	-3.1	0.0	3.6	3.0	0.0	-1.0	-4.2
n		20	20	21	21	21	22	20	22
Reason Deactivated									
* Excluded due to being outside acceptance criteria									

Calibration Curve Parameters for Zidebactam Calibration Standards in Human Plasma (Study V1872106P2)

Run Date	Curve Number	Slope	Intercept	R-Squared	LLOQ	ULOQ
29-Aug-2022	14	0.06670	-0.00005	0.9983	0.0500	50.0
20-Sep-2022	24	0.02392	0.00000	0.9916	0.0500	50.0
09-Nov-2022	30	0.05320	0.00014	0.9988	0.0500	50.0
07-Dec-2022	31	0.19367	0.00018	0.9994	0.0500	50.0
20-Dec-2022	36	0.09313	0.00015	0.9938	0.0500	50.0
06-Jan-2023	37	0.09759	-0.00039	0.9976	0.0500	50.0
09-Jan-2023	40	0.07589	-0.00049	0.9965	0.0500	50.0
15-Jan-2023	42	0.08621	-0.00049	0.9913	0.0500	50.0
21-Jan-2023	43	0.13055	-0.00218	0.9969	0.0500	50.0
07-Feb-2023	46	0.07404	-0.00034	0.9963	0.0500	50.0
17-Feb-2023	47	0.10274	-0.00145	0.9955	0.0500	50.0

Table 18: Standards and QCs of Non-P&A Runs (Continued)

Human Plasma Zidebactam Concentrations in Quality Controls for 10 separate batch runs (Study V1872106P2)

Run Date	Curve Number	QCS 0.150 0.150 µg/mL	%Bias	QCS 25.0 25.0 µg/mL	%Bias	QCS 37.5 37.5 µg/mL	%Bias
29-Aug-2022	14	0.159	6.0	24.8	-0.8	36.4	-2.9
		0.150	0.0	24.3	-2.8	35.6	-5.1
Intrarun Mean		0.155		24.6		36.0	
Intrarun %Bias		3.3		-1.6		-4.0	
20-Sep-2022	24	#0.174	16.0	28.6	14.4	40.0	6.7
		0.161	7.3	24.2	-3.2	38.0	1.3
Intrarun Mean		0.168		26.4		39.0	
Intrarun %Bias		12.0		5.6		4.0	
09-Nov-2022	30	0.154	2.7	25.5	2.0	36.4	-2.9
		0.166	10.7	24.6	-1.6	37.7	0.5
Intrarun Mean		0.160		25.1		37.1	
Intrarun %Bias		6.7		0.4		-1.1	
07-Dec-2022	31	0.156	4.0	25.6	2.4	38.6	2.9
		0.150	0.0	25.1	0.4	37.6	0.3
Intrarun Mean		0.153		25.4		38.1	
Intrarun %Bias		2.0		1.6		1.6	
20-Dec-2022	36	0.154	2.7	23.0	-8.0	35.3	-5.9
		0.160	6.7	23.2	-7.2	34.7	-7.5
Intrarun Mean		0.157		23.1		35.0	
Intrarun %Bias		4.7		-7.6		-6.7	
06-Jan-2023	37	0.152	1.3	26.4	5.6	37.0	-1.3
		0.151	0.7	26.5	6.0	37.5	0.0
Intrarun Mean		0.152		26.5		37.3	
Intrarun %Bias		1.3		6.0		-0.5	
09-Jan-2023	40	0.147	-2.0	24.3	-2.8	38.8	3.5
		0.158	5.3	#51.1	104.4	36.8	-1.9
Intrarun Mean		0.153		37.7		37.8	
Intrarun %Bias		2.0		50.8		0.8	
15-Jan-2023	42	0.157	4.7	25.5	2.0	#53.2	41.9
		#0.217	44.7	24.5	-2.0	37.7	0.5
Intrarun Mean		0.187		25.0		45.5	
Intrarun %Bias		24.7		0.0		21.3	
21-Jan-2023	43	0.160	6.7	25.3	1.2	37.3	-0.5
		0.163	8.7	27.1	8.4	39.5	5.3
Intrarun Mean		0.162		26.2		38.4	
Intrarun %Bias		8.0		4.8		2.4	
07-Feb-2023	46	0.132	-12.0	26.1	4.4	39.7	5.9
		0.145	-3.3	26.1	4.4	40.8	8.8
Intrarun Mean		0.139		26.1		40.3	
Intrarun %Bias		-7.3		4.4		7.5	
17-Feb-2023	47	0.134	-10.7	24.1	-3.6	#31.7	-15.5
		0.153	2.0	25.2	0.8	37.7	0.5
Intrarun Mean		0.144		24.7		34.7	
Intrarun %Bias		-4.0		-1.2		-7.5	
# > 15%Bias							

Criteria: For each run, the calculated concentrations of at least 75% of the calibration standards are required to interpolate within ± 15.0% from nominal with a minimum of six calibration standard levels, except for the LLOQ for which it was required to be within ± 20.0%. In order for the run to be acceptable, at least one QC at each concentration level and two-thirds of the total number of QC samples were required to interpolate within ± 15.0% from nominal.

Results: Acceptance criteria were met.

Table 19: Precision and Accuracy of Sample Dilution

Run Date	Curve Number	Dil QC 37.5 37.5 µg/mL	%Bias	Dil QC 400 400 µg/mL	%Bias
17-Aug-2022	7	##361	862.7	##3780	845.0
		##503	1241.3	##3660	815.0
		##276	636.0	##3660	815.0
		##386	929.3	##3760	840.0
		##382	918.7	##3700	825.0
		##365	873.3	##3770	842.5
Intrarun Mean		379		3720	
Intrarun SD		72.9		55.3	
Intrarun %CV		19.2		1.5	
Intrarun %Bias		910.7		830.0	
n		6		6	
18-Aug-2022	8	39.7	5.9	364	-9.0
		37.7	0.5	361	-9.8
		38.7	3.2	362	-9.5
		38.3	2.1	367	-8.3
		38.3	2.1	375	-6.3
		38.9	3.7	381	-4.8
Intrarun Mean		38.6		368	
Intrarun SD		0.678		7.99	
Intrarun %CV		1.8		2.2	
Intrarun %Bias		2.9		-8.0	
n		6		6	
## > 15%Bias					

Run Date	Curve Number	Dil QC 37.5 37.5 µg/mL	%Bias	Dil QC 400 400 µg/mL	%Bias
23-Aug-2022	9	38.2	1.9	388	-3.0
		37.5	0.0	385	-3.8
		39.0	4.0	397	-0.8
		38.4	2.4	383	-4.3
		38.3	2.1	388	-3.0
		37.7	0.5	391	-2.3
Mean		38.2		389	
SD		0.534		4.93	
%CV		1.4		1.3	
%Bias		1.9		-2.8	
n		6		6	

Sample dilution as follows:

2x = 50 mcL sample + 50 mcL blank treated human plasma
10x = 20 mcL sample + 180 mcL blank treated human plasma, mix well and take 100 mcL for analysis.

Criteria: To be deemed acceptable, the mean %Bias at each level must be within ± 15.0% with %CV ≤ 15.0%.
Results: See Table 7 for reason of failure in run 7 and 8. Dilution linearity was established in run 9. The acceptance criteria were met.

Table 20: Freeze-Thaw Stability, (Frozen At -20 °C and -70 °C Thawed in Wet Ice) - Treated

Treated QCs without comed[2]

Run Date	Curve Number	F/T -20/RT low 0.150 µg/mL	%Bias	F/T -70/RT low 0.150 µg/mL	%Bias	F/T -20/RT high 37.5 µg/mL	%Bias	F/T -70/RT high 37.5 µg/mL	%Bias
26-Aug-2022	12	0.129	-14.0	##0.122	-18.7	34.0	-9.3	35.5	-5.3
		0.132	-12.0	##0.125	-16.7	33.6	-10.4	35.0	-6.7
		0.131	-12.7	##0.126	-16.0	33.5	-10.7	34.0	-9.3
		##0.125	-16.7	0.132	-12.0	33.4	-10.9	33.4	-10.9
		0.130	-13.3	##0.126	-16.0	32.7	-12.8	33.3	-11.2
		##0.123	-18.0	0.130	-13.3	33.4	-10.9	33.2	-11.5
Intrarun Mean		0.128		0.127		33.4		34.1	
Intrarun SD		0.00356		0.00360		0.423		0.971	
Intrarun %CV		2.8		2.8		1.3		2.8	
Intrarun %Bias		-14.7		-15.3		-10.9		-9.1	
n		6		6		6		6	
20-Sep-2022	24	0.147	-2.0	#0.173	15.3	36.5	-2.7	39.9	6.4
		0.147	-2.0	#0.174	16.0	35.8	-4.5	37.5	0.0
		0.134	-10.7	#0.174	16.0	34.4	-8.3	37.6	0.3
		0.137	-8.7	0.156	4.0	33.0	-12.0	36.8	-1.9
		0.141	-6.0	0.147	-2.0	#30.8	-17.9	34.3	-8.5
		0.134	-10.7	0.143	-4.7	32.7	-12.8	35.7	-4.8
Intrarun Mean		0.140		0.161		33.9		37.0	
Intrarun SD		0.00600		0.0143		2.12		1.90	
Intrarun %CV		4.3		8.9		6.3		5.1	
Intrarun %Bias		-6.7		7.3		-9.6		-1.3	
n		6		6		6		6	

> 15%Bias

Run Date	Curve Number	ARQC 400 F/T -20 400 µg/mL	%Bias	ARQC 400 F/T -70 400 µg/mL	%Bias
27-Aug-2022	13	##339	-15.3	363	-9.3
		363	-9.3	385	-3.8
		354	-11.5	372	-7.0
		368	-8.0	374	-6.5
		366	-8.5	363	-9.3
		373	-6.8	356	-11.0
Mean		361		369	
SD		12.3		10.3	
%CV		3.4		2.8	
%Bias		-9.8		-7.8	
n		6		6	

> 15%Bias

Criteria: To be deemed stable, the mean %Bias at each level must be within ± 15.0% with %CV ≤ 15.0%.

Results: During initial F/T evaluation in Run 12, the low F/T QCs failed. After re-prepare the QCs with flash freeze, the re-evaluation in Run 24 indicated zidebactam is stable in treated human plasma without the presence of comed[2], with sodium heparin as anticoagulant, for at least 4 cycles when frozen at -20 °C and -70 °C and thawed in wet ice.

Table 20: Freeze-Thaw Stability, (Frozen At -20 °C and -70 °C Thawed in Wet Ice) – Treated (Continued)

Treated QCs with comed[2]

Run Date	Curve Number	Comed1 F/T -20 low 0.150 µg/mL	%Bias	Comed1 F/T -70 low 0.150 µg/mL	%Bias	Comed1 F/T -20 high 37.5 µg/mL	%Bias	Comed1 F/T -70 high 37.5 µg/mL	%Bias
26-Aug-2022	12	##0.107	-28.7	##0.104	-30.7	33.3	-11.2	33.3	-11.2
		##0.104	-30.7	##0.107	-28.7	33.3	-11.2	34.9	-6.9
		##0.106	-29.3	##0.107	-28.7	33.1	-11.7	35.2	-6.1
		##0.111	-26.0	##0.115	-23.3	32.4	-13.6	34.8	-7.2
		##0.192	28.0	##0.112	-25.3	32.9	-12.3	34.5	-8.0
		##0.107	-28.7	##0.102	-32.0	33.7	-10.1	33.1	-11.7
Intrarun Mean		0.121		0.108		33.1		34.3	
Intrarun SD		0.0348		0.00488		0.440		0.883	
Intrarun %CV		28.8		4.5		1.3		2.6	
Intrarun %Bias		-19.3		-28.0		-11.7		-8.5	
n		6		6		6		6	
20-Sep-2022	24	0.154	2.7	0.157	4.7	36.4	-2.9	36.1	-3.7
		0.144	-4.0	0.151	0.7	37.5	0.0	37.9	1.1
		0.155	3.3	0.154	2.7	36.3	-3.2	36.4	-2.9
		0.136	-9.3	0.149	-0.7	33.9	-9.6	36.0	-4.0
		0.139	-7.3	0.138	-8.0	32.5	-13.3	36.1	-3.7
		0.139	-7.3	0.140	-6.7	32.8	-12.5	33.4	-10.9
Intrarun Mean		0.145		0.148		34.9		36.0	
Intrarun SD		0.00817		0.00763		2.10		1.45	
Intrarun %CV		5.6		5.2		6.0		4.0	
Intrarun %Bias		-3.3		-1.3		-6.9		-4.0	
n		6		6		6		6	

> 15%Bias

Criteria: To be deemed stable, the mean %Bias at each level must be within ± 15.0% with %CV ≤ 15.0%.

Results: During initial F/T evaluation in Run 12, the low F/T QCs failed. After re-prepare the QCs with flash freeze, the re-evaluation in Run 24 indicated zidebactam is stable in treated human plasma with the presence of comed[2], with sodium heparin as anticoagulant, for at least 4 cycles when frozen at -20 °C and -70 °C and thawed in wet ice.

Table 21: Freeze-Thaw Stability, (Frozen At -70 °C Thawed in Wet Ice) - Untreated

Untreated QCs without comed[2]

Run Date	Curve Number	Untr. F/T -70 low 0.150 µg/mL	%Bias	Untr. F/T -70 high 37.5 µg/mL	%Bias
07-Feb-2023	46	0.157	4.7	36.5	-2.7
		0.158	5.3	37.0	-1.3
		0.152	1.3	38.2	1.9
		0.151	0.7	37.2	-0.8
		0.155	3.3	38.2	1.9
		0.156	4.0	39.3	4.8
Mean		0.155		37.7	
SD		0.00279		1.02	
%CV		1.8		2.7	
%Bias		3.3		0.5	
n		6		6	

Criteria: To be deemed stable, the mean %Bias at each level must be within ± 15.0% with %CV ≤ 15.0%.
Results: Zidebactam is stable in untreated human plasma without the presence of comed[2], with sodium heparin as anticoagulant, for at least 2 cycles when frozen at -70 °C and thawed in wet ice.

Untreated QCs with comed[2]

Run Date	Curve Number	Untr. F/T -70 low 0.150 µg/mL	%Bias	Untr. F/T -70 high 37.5 µg/mL	%Bias
20-Dec-2022	36	0.144	-4.0	33.1	-11.7
		0.140	-6.7	34.1	-9.1
		0.151	0.7	34.0	-9.3
		0.148	-1.3	35.1	-6.4
		0.140	-6.7	33.9	-9.6
		0.152	1.3	35.4	-5.6
Mean		0.146		34.3	
SD		0.00531		0.845	
%CV		3.6		2.5	
%Bias		-2.7		-8.5	
n		6		6	

Criteria: To be deemed stable, the mean %Bias at each level must be within ± 15.0% with %CV ≤ 15.0%.
Results: Zidebactam is stable in untreated human plasma with the presence of comed[2], with sodium heparin as anticoagulant, for at least 2 cycles when frozen at -70 °C and thawed in wet ice.

Table 22: Bench-Top Stability in Wet Ice- Treated

Treated QCs without comed[2]

Run Date	Curve Number	BT 19.75hr low 0.150 µg/mL	%Bias	BT 19.75hr high 37.5 µg/mL	%Bias	ARQC 400 BT 19.75hr 400 µg/mL	%Bias
26-Aug-2022	12	0.128	-14.7	34.4	-8.3		
		0.133	-11.3	34.7	-7.5		
		0.135	-10.0	33.5	-10.7		
		0.133	-11.3	35.0	-6.7		
		0.133	-11.3	35.1	-6.4		
		##0.126	-16.0	34.5	-8.0		
Intrarun Mean		0.131		34.5			
Intrarun SD		0.00350		0.575			
Intrarun %CV		2.7		1.7			
Intrarun %Bias		-12.7		-8.0			
n		6		6			
27-Aug-2022	13					342	-14.5
						363	-9.3
						401	0.3
						364	-9.0
						381	-4.8
						379	-5.3
Intrarun Mean						372	
Intrarun SD						20.1	
Intrarun %CV						5.4	
Intrarun %Bias						-7.0	
n						6	
## > 15%Bias							

Run Date	Curve Number	BT 8.25hr low 0.150 µg/mL	%Bias	BT 8.25hr high 37.5 µg/mL	%Bias
20-Sep-2022	24	0.162	8.0	36.4	-2.9
		0.153	2.0	36.5	-2.7
		0.156	4.0	38.0	1.3
		0.148	-1.3	34.1	-9.1
		0.147	-2.0	34.6	-7.7
		0.141	-6.0	32.9	-12.3
Intrarun Mean		0.151		35.4	
Intrarun SD		0.00741		1.87	
Intrarun %CV		4.9		5.3	
Intrarun %Bias		0.7		-5.6	
n		6		6	

Criteria: To be deemed stable, the mean %Bias at each level must be within ± 15.0% with %CV ≤ 15.0%.

Results: Zidebactam is stable in treated human plasma without the presence of comed[2], with sodium heparin as anticoagulant, for at least 19.75 hours in wet ice.

Table 22: Bench-Top Stability in Wet Ice – Treated (Continued)

Treated QCs with comed[2]

Run Date	Curve Number	Comed1BT19.75hr low 0.150 µg/mL	%Bias	Comed1BT19.75hr high 37.5 µg/mL	%Bias
26-Aug-2022	12	##0.111	-26.0	33.0	-12.0
		##0.112	-25.3	33.5	-10.7
		##0.109	-27.3	34.1	-9.1
		##0.107	-28.7	35.4	-5.6
		##0.105	-30.0	35.7	-4.8
		##0.108	-28.0	34.8	-7.2
Intrarun Mean		0.109		34.4	
Intrarun SD		0.00258		1.07	
Intrarun %CV		2.4		3.1	
Intrarun %Bias		-27.3		-8.3	
n		6		6	
## > 15%Bias					

Run Date	Curve Number	Comed1 BT 8.25hr low 0.150 µg/mL	%Bias	Comed1BT 8.25hr high 37.5 µg/mL	%Bias
20-Sep-2022	24	#0.175	16.7	38.2	1.9
		0.164	9.3	36.4	-2.9
		0.159	6.0	37.3	-0.5
		0.154	2.7	34.0	-9.3
		0.148	-1.3	35.4	-5.6
		0.140	-6.7	33.2	-11.5
Intrarun Mean		0.157		35.8	
Intrarun SD		0.0123		1.92	
Intrarun %CV		7.8		5.4	
Intrarun %Bias		4.7		-4.5	
n		6		6	
# > 15%Bias					

Criteria: To be deemed stable, the mean %Bias at each level must be within ± 15.0% with %CV ≤ 15.0%.

Results: Zidebactam is stable in treated human plasma with the presence of comed[2], with sodium heparin as anticoagulant, for at least 8.25 hours in wet ice.

Table 23: Bench-Top Stability in Wet Ice- Untreated

Untreated QCs without comed[2]

Run Date	Curve Number	Untr. BT low 0.150 µg/mL	%Bias	Untr. BT high 37.5 µg/mL	%Bias
07-Feb-2023	46	0.153	2.0	37.0	-1.3
		0.154	2.7	39.4	5.1
		0.156	4.0	38.3	2.1
		0.157	4.7	38.6	2.9
		0.162	8.0	37.5	0.0
		0.172	14.7	38.3	2.1
Intrarun Mean		0.159		38.2	
Intrarun SD		0.00710		0.842	
Intrarun %CV		4.5		2.2	
Intrarun %Bias		6.0		1.9	
n		6		6	

Criteria: To be deemed stable, the mean %Bias at each level must be within ± 15.0% with %CV ≤ 15.0%.
Results: Zidebactam is stable in untreated human plasma without the presence of comed[2], with sodium heparin as anticoagulant, for at least 4 hours in wet ice.

Untreated QCs with comed[2]

Run Date	Curve Number	Untr. BT low 0.150 µg/mL	%Bias	Untr. BT high 37.5 µg/mL	%Bias
20-Dec-2022	36	0.141	-6.0	33.2	-11.5
		0.151	0.7	34.9	-6.9
		0.148	-1.3	32.9	-12.3
		0.136	-9.3	33.8	-9.9
		0.149	-0.7	32.5	-13.3
		0.146	-2.7	32.9	-12.3
Mean		0.145		33.4	
SD		0.00564		0.866	
%CV		3.9		2.6	
%Bias		-3.3		-10.9	
n		6		6	

Criteria: To be deemed stable, the mean %Bias at each level must be within ± 15.0% with %CV ≤ 15.0%.
Results: Zidebactam is stable in untreated human plasma with the presence of comed[2], with sodium heparin as anticoagulant, for at least 4 hours in wet ice.

Table 24: Long-Term Stability at -20 °C and -70 °C - Treated

Treated without comed[2]

Run Date	Curve Number	LTS -20 low 0.150 µg/mL	%Bias	LTS -70 low 0.150 µg/mL	%Bias	LTS -20 high 37.5 µg/mL	%Bias	LTS -70 high 37.5 µg/mL	%Bias
09-Nov-2022	30	#0.0875	-41.7	0.149	-0.7	#22.4	-40.3	34.7	-7.5
(62 days)		#0.0797	-46.9	0.154	2.7	#21.9	-41.6	34.5	-8.0
		#0.0824	-45.1	0.143	-4.7	#22.0	-41.3	34.3	-8.5
		#0.0933	-37.8	0.158	5.3	#21.5	-42.7	33.8	-9.9
		#0.0897	-40.2	0.149	-0.7	#22.5	-40.0	34.0	-9.3
		#0.0843	-43.8	0.150	0.0	#22.4	-40.3	34.0	-9.3
Intrarun Mean		0.0862		0.151		22.1		34.2	
Intrarun SD		0.00499		0.00509		0.387		0.343	
Intrarun %CV		5.8		3.4		1.8		1.0	
Intrarun %Bias		-42.5		0.7		-41.1		-8.8	
n		6		6		6		6	
07-Dec-2022	31			0.140	-6.7			34.5	-8.0
(90 days)				0.144	-4.0			33.9	-9.6
				0.145	-3.3			34.0	-9.3
				0.137	-8.7			33.8	-9.9
				0.139	-7.3			34.1	-9.1
				0.139	-7.3			33.8	-9.9
Intrarun Mean				0.141				34.0	
Intrarun SD				0.00314				0.264	
Intrarun %CV				2.2				0.8	
Intrarun %Bias				-6.0				-9.3	
n				6				6	
# > 15%Bias									

Run Date	Curve Number	Dil QC 400 LTS -70 400 µg/mL	%Bias
07-Dec-2022	31	378	-5.5
(90 days)		391	-2.3
		388	-3.0
		379	-5.3
		398	-0.5
		392	-2.0
Mean		388	
SD		7.81	
%CV		2.0	
%Bias		-3.0	
n		6	

Criteria: To be deemed stable, the mean %Bias at each level must be within ± 15.0% with %CV ≤ 15.0%.

Results: Zidebactam is stable in treated human plasma without the presence of comed[2], with sodium heparin as anticoagulant, for up to 90 days upon storage in polypropylene tube at -70 °C. Zidebactam is unstable in treated human plasma without the presence of comed[2], with sodium heparin as anticoagulant, for up to 62 days upon storage in polypropylene tube at -20 °C.

Table 24: Long-Term Stability at -20 °C and -70 °C – Treated (Continued)

Treated QCs with comed[2]

Run Date	Curve Number	Comed1 LTS -20 low 0.150 µg/mL	%Bias	Comed1 LTS -70 low 0.150 µg/mL	%Bias	Comed1 LTS -20 high 37.5 µg/mL	%Bias	Comed1 LTS -70 high 37.5 µg/mL	%Bias
09-Nov-2022	30	#0.105	-30.0	0.151	0.7	#25.7	-31.5	35.5	-5.3
(62 days)		#0.105	-30.0	0.144	-4.0	#25.1	-33.1	35.2	-6.1
		#0.100	-33.3	0.149	-0.7	#25.2	-32.8	35.5	-5.3
		#0.0966	-35.6	0.148	-1.3	#25.6	-31.7	35.2	-6.1
		#0.103	-31.3	0.147	-2.0	#25.2	-32.8	35.1	-6.4
		#0.109	-27.3	0.149	-0.7	#25.7	-31.5	35.0	-6.7
Intrarun Mean		0.103		0.148		25.4		35.3	
Intrarun SD		0.00433		0.00237		0.279		0.207	
Intrarun %CV		4.2		1.6		1.1		0.6	
Intrarun %Bias		-31.3		-1.3		-32.3		-5.9	
n		6		6		6		6	
07-Dec-2022	31			0.144	-4.0			34.5	-8.0
(90 days)				0.141	-6.0			35.2	-6.1
				0.139	-7.3			34.1	-9.1
				0.137	-8.7			33.9	-9.6
				0.141	-6.0			33.8	-9.9
				0.136	-9.3			33.8	-9.9
Intrarun Mean				0.140				34.2	
Intrarun SD				0.00294				0.549	
Intrarun %CV				2.1				1.6	
Intrarun %Bias				-6.7				-8.8	
n				6				6	
# > 15%Bias									

Criteria: To be deemed stable, the mean %Bias at each level must be within ± 15.0% with %CV ≤ 15.0%.

Results: Zidebactam is stable in treated human plasma with the presence of comed[2], with sodium heparin as anticoagulant, for up to 90 days upon storage in polypropylene tube at -70 °C.
Run 30 -20 °C LTS QCs did not meet criteria at 62 days due to high %Bias at the low and high LTS QC. Zidebactam is not stable in treated human plasma with the presence of comed[2], with sodium heparin as anticoagulant, for up to 62 days upon storage in polypropylene tube at -20 °C.

Table 25: Long-Term Stability at -20 °C and -70 °C - Untreated

Untreated QCs without comed[2]

Run Date	Curve Number	Untr. LTS -70 low 0.150 µg/mL	%Bias	Untr. LTS -70 high 37.5 µg/mL	%Bias
07-Feb-2023	46	0.167	11.3	37.1	-1.1
(17 days)		#0.175	16.7	38.3	2.1
		0.164	9.3	39.1	4.3
		0.163	8.7	39.6	5.6
		0.161	7.3	38.2	1.9
		0.161	7.3	39.7	5.9
Intrarun Mean		0.165		38.7	
Intrarun SD		0.00531		0.993	
Intrarun %CV		3.2		2.6	
Intrarun %Bias		10.0		3.2	
n		6		6	
17-Feb-2023	47	0.169	12.7	36.4	-2.9
(27 days)		0.148	-1.3	32.8	-12.5
		0.164	9.3	35.7	-4.8
		0.156	4.0	35.9	-4.3
		0.155	3.3	37.8	0.8
		0.169	12.7	37.9	1.1
Intrarun Mean		0.160		36.1	
Intrarun SD		0.00852		1.86	
Intrarun %CV		5.3		5.2	
Intrarun %Bias		6.7		-3.7	
n		6		6	
# > 15%Bias					

Criteria: To be deemed stable, the mean %Bias at each level must be within ± 15.0% with %CV ≤ 15.0%.

Results: Zidebactam is stable in untreated human plasma without the presence of comed[2], with sodium heparin as anticoagulant, for up to 27 days upon storage in polypropylene tube at -70 °C.

Table 24: Long-Term Stability at -20 °C and -70 °C – Untreated (Continued)

Untreated QCs with comed[2]

Run Date	Curve Number	Untr. LTS -70 low 0.150 µg/mL	%Bias	Untr. LTS -70 high 37.5 µg/mL	%Bias
06-Jan-2023	37	0.149	-0.7	37.3	-0.5
(21 days)		0.143	-4.7	35.1	-6.4
		0.160	6.7	36.8	-1.9
		0.155	3.3	37.6	0.3
		0.157	4.7	36.6	-2.4
		0.156	4.0	38.2	1.9
Intrarun Mean		0.153		36.9	
Intrarun SD		0.00622		1.07	
Intrarun %CV		4.1		2.9	
Intrarun %Bias		2.0		-1.6	
n		6		6	
09-Jan-2023	40	0.144	-4.0	34.6	-7.7
(24 days)		0.148	-1.3	34.1	-9.1
		0.154	2.7	34.5	-8.0
		0.144	-4.0	33.7	-10.1
		0.157	4.7	#47.1	25.6
		0.152	1.3	33.4	-10.9
Intrarun Mean		0.150		36.2	
Intrarun SD		0.00538		5.34	
Intrarun %CV		3.6		14.8	
Intrarun %Bias		0.0		-3.5	
n		6		6	
15-Jan-2023	42	#0.187	24.7	38.0	1.3
(28 days)		#0.210	40.0	#53.0	41.3
		#0.213	42.0	#54.1	44.3
		#0.215	43.3	#51.4	37.1
		#0.223	48.7	40.4	7.7
		#0.212	41.3	39.1	4.3
Intrarun Mean		0.210		46.0	
Intrarun SD		0.0121		7.57	
Intrarun %CV		5.8		16.5	
Intrarun %Bias		40.0		22.7	
n		6		6	
21-Jan-2023	43	0.158	5.3	36.4	-2.9
(35 days)		0.155	3.3	37.3	-0.5
		0.156	4.0	37.6	0.3
		0.162	8.0	37.7	0.5
		0.161	7.3	36.3	-3.2
		0.158	5.3	38.0	1.3
Intrarun Mean		0.158		37.2	
Intrarun SD		0.00273		0.708	
Intrarun %CV		1.7		1.9	
Intrarun %Bias		5.3		-0.8	
n		6		6	

Criteria: To be deemed stable, the mean %Bias at each level must be within ± 15.0% with %CV ≤ 15.0%.

Results: Zidebactam is stable in untreated human plasma with the presence of comed[2], with sodium heparin as anticoagulant, for up to 35 days upon storage in polypropylene tube at -70 °C.

Table 26: Stability Evaluation During Blood Collection and Processing

Run: 22

Evaluation of Zidebactam Stability During Blood Collection and Processing in Ice Water Bath, Centrifuged at Room Temperature

**Mid QCS, 25.0 mcg/mL**									
Stability Data: 90 minutes Ice Water Bath					Comparison Data: 0 minutes				
Sample ID	Seq #	Drug Peak Area	IS Peak Area	Peak Area Ratio	Sample ID	Seq #	Drug Peak Area	IS Peak Area	Peak Area Ratio
MidQC-IceWater 90min	6	9321489	2602181	3.582	MidQC-IceWater 0hr	5	9420212	2525447	3.730
MidQC-IceWater 90min	10	8891879	2521867	3.526	MidQC-IceWater 0hr	9	9017807	2552087	3.534
MidQC-IceWater 90min	14	8972279	2577871	3.480	MidQC-IceWater 0hr	13	9707517	2726874	3.560
MidQC-IceWater 90min	18	8597532	2530990	3.397	MidQC-IceWater 0hr	17	8738290	2468928	3.539
			Mean	3.496				Mean	3.591
			SD	0.07828				SD	0.09362
			%CV	2.2				%CV	2.6
			**% Change**	**-2.6**					

Evaluation of Zidebactam Stability During Blood Collection and Processing at Room Temperature, Centrifuged at Room Temperature

**Mid QCS, 25.0 mcg/mL**									
Stability Data: 90 minutes Room Temperature					Comparison Data: 0 minutes				
Sample ID	Seq #	Drug Peak Area	IS Peak Area	Peak Area Ratio	Sample ID	Seq #	Drug Peak Area	IS Peak Area	Peak Area Ratio
MidQC-RT 90min	4	9445147	2602200	3.6	MidQC-RT 0hr	3	9541404	2653703	3.6
MidQC-RT 90min	8	8868631	2522974	3.5	MidQC-RT 0hr	7	9547807	2648311	3.6
MidQC-RT 90min	12	8642952	2492399	3.5	MidQC-RT 0hr	11	9329805	2642535	3.5
MidQC-RT 90min	16	8917718	2567144	3.5	MidQC-RT 0hr	15	9119916	2605152	3.5
			Mean	3.5				Mean	3.6
			SD	0.0751				SD	0.0506
			%CV	2.1				%CV	1.4
			**% Change**	**-1.0**					

$$\%\ \text{Change} = \frac{(\text{Peak Area Ratio Stored} - \text{Peak Area Ratio Time 0})}{\text{Peak Area Ratio Time 0}} \times 100$$

Criteria: To be considered stable, the % change between 90 minute sample pools and the control 0-hr sample pools must be within ± 15.0%, with %CV ≤ 15.0%.

Results: Zidebactam appears to be stable during the blood collection process at room temperature or in an ice-water bath for at least 90 minutes in polyethylene terephthalate containers.

Table 27: Processed Sample Stability at Room Temperature

Run Date	Curve Number	Processed RT low 0.150 µg/mL	%Bias	Processed RT high 37.5 µg/mL	%Bias
29-Aug-2022	14	0.170	13.3	40.7	8.5
		0.170	13.3	41.8	11.5
		0.155	3.3	40.9	9.1
		0.171	14.0	39.5	5.3
		0.164	9.3	39.0	4.0
		0.166	10.7	40.1	6.9
Mean		0.166		40.3	
SD		0.00603		1.01	
%CV		3.6		2.5	
%Bias		10.7		7.5	
n		6		6	

Samples were processed with run 13 then stored for 3 days 0 hours in polypropylene 96 well plates at ambient conditions prior to injection with run 14.

Criteria: To be deemed stable, the mean %Bias at each level must be within ± 15.0% with %CV ≤ 15.0%.

Results: The acceptance criteria were met.

Table 28: Refrigerated Processed Sample Stability

Run Date	Curve Number	Processed Refr low 0.150 µg/mL	%Bias	Processed Refr high 37.5 µg/mL	%Bias
29-Aug-2022	14	0.145	-3.3	35.4	-5.6
		0.138	-8.0	34.0	-9.3
		0.146	-2.7	35.5	-5.3
		0.145	-3.3	35.0	-6.7
		0.172	14.7	35.5	-5.3
		0.144	-4.0	34.4	-8.3
Mean		0.148		35.0	
SD		0.0119		0.635	
%CV		8.0		1.8	
%Bias		-1.3		-6.7	
n		6		6	

Samples were processed with run 13, and then stored for 3 days 0 hours in polypropylene 96 well plates at refrigerated temperatures prior to injection with run 14.

Criteria: To be deemed stable, the mean %Bias at each level must be within ± 15.0% with %CV ≤ 15.0%.

Results: The acceptance criteria were met.

Table 29: Individual Sample Reinjection Reproducibility

Run Date	Curve Number	Indiv Reinj low 0.150 µg/mL	%Bias	Indiv Reinj high 37.5 µg/mL	%Bias
15-Aug-2022	5	0.161	7.3	39.0	4.0
		0.167	11.3	39.3	4.8
		0.166	10.7	38.4	2.4
		0.158	5.3	38.8	3.5
		0.163	8.7	39.3	4.8
		*0.253		38.9	3.7
With outlier					
Mean		0.178			
SD		0.0369			
%CV		20.7			
%Bias		18.7			
n		6			
Without outlier					
Mean		0.163		39.0	
SD		0.00367		0.339	
%CV		2.3		0.9	
%Bias		8.7		4.0	
n		5		6	
Reason Deactivated: * Statistical Outlier (t-test. p<0.01)					

Low and high QC extracts (6 each) were injected during the run and then reinjected at the end of the same run.
Criteria: To be deemed reproducible, the mean %Bias at each level must be within ± 15.0% with %CV ≤ 15.0%.
Results: The acceptance criteria were met.

Table 30: Whole Run Reinjection Reproducibility and Column Ruggedness

Analytical Performance: Back-Calculated Concentrations (µg/mL) of Zidebactam Calibration Standard in (Human) (Plasma) in (Study V1872106P2)

Assay Date	Analytical Run Number	Cal 0.0500 0.0500 µg/mL	Cal 0.100 0.100 µg/mL	Cal 1.00 1.00 µg/mL	Cal 5.00 5.00 µg/mL	Cal 10.0 10.0 µg/mL	Cal 20.0 20.0 µg/mL	Cal 40.0 40.0 µg/mL	Cal 50.0 50.0 µg/mL
29-Aug-2022	15	0.0470	0.0961	1.00	4.89	10.1	19.4	40.2	47.1
		0.0525	0.105	1.05	5.19	10.4	19.6	39.8	49.2
Mean		0.0498	0.101	1.03	5.04	10.3	19.5	40.0	48.2
%Bias		-0.4	1.0	3.0	0.8	3.0	-2.5	0.0	-3.6

Calibration Curve Parameters for Zidebactam Calibration Standards in Human Plasma (Study V1872106P2)

Run Date	Curve Number	Slope	Intercept	R-Squared	LLOQ	ULOQ
29-Aug-2022	15	0.06519	-0.00004	0.9982	0.0500	50.0

Analytical Performance of Zidebactam Quality Control Samples in Human Plasma (Study V1872106P2)

Run Date	Curve Number	QCS 0.0500 0.0500 µg/mL	%Bias	QCS 0.150 0.150 µg/mL	%Bias	QCS 25.0 25.0 µg/mL	%Bias	QCS 37.5 37.5 µg/mL	%Bias
29-Aug-2022	15	0.0487	-2.6	0.161	7.3	24.7	-1.2	36.7	-2.1
		0.0534	6.8	0.156	4.0	24.9	-0.4	36.1	-3.7
		0.0479	-4.2	0.152	1.3	25.4	1.6	35.3	-5.9
		0.0484	-3.2	0.153	2.0	26.1	4.4	38.3	2.1
		0.0476	-4.8	0.155	3.3	24.7	-1.2	36.2	-3.5
		0.0541	8.2	0.150	0.0	26.0	4.0	35.8	-4.5
Mean		0.0500		0.155		25.3		36.4	
S.D.		0.00293		0.00383		0.636		1.04	
%CV		5.9		2.5		2.5		2.9	
%Bias		0.0		3.3		1.2		-2.9	
n		6		6		6		6	

Note: Run 15 is a reinjection of run 13 on a different analytical column of the same manufacturer, dimensions, and type, using same MS platform (Applied Biosystems API5000) – reinjected within 2 days 7 hours

Criteria: At each level, the %CV was required to be ≤ 15.0% from the nominal value, except at the LLOQ level, where ≤ 20.0% was accepted. The mean %Bias at each level was also required to be within ± 15.0%, except at the LLOQ, where ± 20.0% was accepted.

Results: The acceptance criteria were met.

Table 31: Extraction Recovery – Zidebactam

Run: 13

Control (PES; post extraction spike)				Extracted QCs				Zidebactam
Zidebactam QC Conc. *mcg/mL*	Zidebactam Peak Area	IS Peak Area	Peak Area Ratio	Zidebactam QC Conc. *mcg/mL*	Zidebactam Peak Area	IS Peak Area	Peak Area Ratio	Extraction Recovery %
Low QC				Low QC				
0.150	14803	2093643	0.0071	0.150	15068	2053276	0.0073	99.0
0.150	15116	2002531	0.0075	0.150	13732	2021690	0.0068	91.6
0.150	14781	2023973	0.0073	0.150	13662	2024549	0.0067	91.0
0.150	15170	2005351	0.0076	0.150	14356	2005589	0.0072	96.5
0.150	15010	2001043	0.0075	0.150	14059	2029174	0.0069	93.4
0.150	14925	1989727	0.0075	0.150	13976	2023215	0.0069	93.2
		Average	0.0074				Average	94.1
Mid QC				Mid QC				
25.0	2124139	2030951	1.0459	25.0	1994652	2063125	0.9668	92.9
25.0	2070052	2032945	1.0183	25.0	2031420	2015310	1.0080	96.8
25.0	2128712	2019876	1.0539	25.0	2000741	1992100	1.0043	96.5
25.0	2121747	2015251	1.0528	25.0	1995571	2007504	0.9941	95.5
25.0	2132744	2049496	1.0406	25.0	2093711	2006231	1.0436	100.3
25.0	2088791	2020012	1.0340	25.0	1996520	2028787	0.9841	94.5
		Average	1.0409				Average	96.1
High QC				High QC				
37.5	3011103	1972848	1.5263	37.5	2910784	1990972	1.4620	97.0
37.5	2968101	2040520	1.4546	37.5	2962208	2007504	1.4756	97.9
37.5	3049067	1954268	1.5602	37.5	2869822	2021678	1.4195	94.1
37.5	3076109	2017580	1.5247	37.5	2847632	1996543	1.4263	94.6
37.5	3012011	1989263	1.5141	37.5	2892815	2038423	1.4191	94.1
37.5	2988367	2036584	1.4673	37.5	2836285	2009758	1.4113	93.6
		Average	1.5079				Average	95.2
								======
							n:	18
							Overall Mean Recovery:	**95.1**
							Overall Standard Deviation:	**2.5**
							Overall % CV:	**2.6**

$$\% \text{ Recovery} = \frac{\text{Response Ratio of "Extracted QC"}}{\text{Average Response Ratio of Control}} \times 100$$

Table 32: Extraction Recovery – Cefotaxime

Run: 13

Control (PES; post extraction spike)				Extracted QCs				IS
Zidebactam QC Conc. *mcg/mL*	IS Peak Area	Zidebactam Peak Area	Peak Area Ratio	Zidebactam QC Conc. *mcg/mL*	IS Peak Area	Zidebactam Peak Area	Peak Area Ratio	Extraction Recovery %
Mid QC				Mid QC				
25.0	1980621	2050433	0.9660	25.0	2063125	1994652	1.0343	102.4
25.0	2030364	2057374	0.9869	25.0	2015310	2031420	0.9921	98.2
25.0	2030928	2031814	0.9996	25.0	1992100	2000741	0.9957	98.6
25.0	2073166	2022113	1.0252	25.0	2007504	1995571	1.0060	99.6
25.0	2085201	1989598	1.0481	25.0	2006231	2093711	0.9582	94.9
25.0	2067469	1997324	1.0351	25.0	2028787	1996520	1.0162	100.6
		Average	1.0101				Average	99.0
							SD	2.5
							%CV	2.6
								======
							n:	6

$$\% \text{ Recovery} = \frac{\text{Response Ratio of "Extracted QC"}}{\text{Average Response Ratio of Control}} \times 100$$

Table 33: Stability of Zidebactam Primary Solution in Deionized Water at Room Temperature

Study: V1872106P2
Run: 2
Container: Polypropylene

Analyte	Sample Name	Sequence Number	Analyte Peak Area	IS Peak Area	Peak Area Ratio		Bench Top	Refrigerated	-20 °C Reference
Zidebactam 2 mg/mL	Primary -20°C-1	23	2567191	528700	4.8557	Lot	V1872106P2-7-4 compared to Refr	V1872106P2-7-5 Compared to -20°C	V1872106P2-7-6
	Primary -20°C-1	29	2633556	527079	4.9965				
	Primary -20°C-1	35	2609761	526757	4.9544				
	Primary -20°C-2	24	2557707	295453	8.6569				
	Primary -20°C-2	30	2528712	518220	4.8796				
	Primary -20°C-2	36	2568839	534612	4.8051	Avg	4.8975	5.5715	5.5247
	Primary Refr-1	25	2511017	521633	4.8138	Std Dev	0.052	1.705	1.536
	Primary Refr-1	31	2600821	524492	4.9587	%CV	1.1	30.6	27.8
	Primary Refr-1	37	2560732	527358	4.8558	%Change	-12.1	0.8	N/A
	Primary Refr-2	26	2559192	522788	4.8953				
	Primary Refr-2	32	2550769	525413	4.8548				
	Primary Refr-2	38	2575883	284602	9.0508	Duration	21 hours		
	Primary BT-1	27	2527304	521507	4.8462				
	Primary BT-1	33	2586010	523130	4.9433				
	Primary BT-1	39	2565561	525638	4.8809				
	Primary BT-2	28	2550205	527432	4.8351				
	Primary BT-2	34	2563285	521255	4.9175				
	Primary BT-2	40	2619701	527943	4.9621				

$$\%\text{Change} = \frac{\text{mean response of stored solutions} - \text{mean response of comparison solutions}}{\text{mean response of comparison solutions}} \times 100$$

Criteria: To be deemed stable, % Change upon storage for analyte must be within ±15.0% and %CV must be within ≤15.0%.
Meets Criteria: NO. Failed due to high %CV. Data from V1872106U2 Zidebactam Primary Solution in Deionized Water at Room Temperature for 17.5 hours is reported in next page.

Table 33: Stability of Zidebactam Primary Solution in Deionized Water at Room Temperature (Continued)

Study: V1872106U2
Run: 3
Container: Glass

Analyte	Sample Name	Sequence Number	Analyte Peak Area	IS Peak Area	Peak Area Ratio		Bench Top	Refrigerated	-20 °C Reference
Zidebactam 160 mg/mL	3 040 V1872106U2 Primary -20°C-1 1 1	40	5873361	70659	83.1226	Lot	V1872106U2-7-1 compared to Refr	V1872106U2-7-2 Compared to -20°C	V1872106U2-7-3
	3 041 V1872106U2 Primary -20°C-1 2 1	41	5955067	72984	81.5941				
	3 042 V1872106U2 Primary -20°C-1 3 1	42	6037661	72006	83.8494				
	3 043 V1872106U2 Primary -20°C-2 1 1	43	6159684	73349	83.9778				
	3 044 V1872106U2 Primary -20°C-2 2 1	44	6042317	70926	85.1918				
	3 045 V1872106U2 Primary -20°C-2 3 1	45	6229043	73072	85.2453	Avg	82.8448	87.6236	83.8302
	3 046 V1872106U2 Primary Refr-1 1 1	46	6399570	70861	90.3116	Std Dev	2.236	1.673	1.370
	3 047 V1872106U2 Primary Refr-1 2 1	47	6360185	73071	87.0412	%CV	2.7	1.9	1.6
	3 048 V1872106U2 Primary Refr-1 3 1	48	6146003	71623	85.8105	%Change	-5.5	4.5	N/A
	3 049 V1872106U2 Primary Refr-2 1 1	49	6284414	71539	87.8460				
	3 050 V1872106U2 Primary Refr-2 2 1	50	6330916	71469	88.5827				
	3 051 V1872106U2 Primary Refr-2 3 1	51	6223524	72241	86.1495	Duration	17.75 hours		
	3 052 V1872106U2 Primary BT-1 1 1	52	5820122	72929	79.8053				
	3 053 V1872106U2 Primary BT-1 2 1	53	5878923	71780	81.9020				
	3 054 V1872106U2 Primary BT-1 3 1	54	5916822	71690	82.5334				
	3 055 V1872106U2 Primary BT-2 1 1	55	6102441	70418	86.6602				
	3 056 V1872106U2 Primary BT-2 2 1	56	6029362	72377	83.3049				
	3 057 V1872106U2 Primary BT-2 3 1	57	6075182	73316	82.8630				

$$\%\text{Change} = \frac{\text{mean response of stored solutions} - \text{mean response of comparison solutions}}{\text{mean response of comparison solutions}} \times 100$$

Criteria: To be deemed stable, % Change upon storage for analyte must be within ±15.0% and %CV must be within ≤15.0%.
Meets Criteria: YES

Table 34: Stability of Zidebactam Primary Solution in Deionized Water at -20 °C

Study: V1872106U2
Run: 9
Container: Glass

Concentration: 160 mg/mL									
Stability Data (Stored)					Comparison Data (Fresh)				
Lot #:	V1872106U2-2-1				Lot #:	V1872106U2-28-1			
Prepared:	21-Jul-2022				Prepared:	15-Aug-2022			
Sample ID	Seq #	Drug Peak Area	IS Peak Area	Peak Area Ratio	Sample ID	Seq #	Drug Peak Area	IS Peak Area	Peak Area Ratio
9 034 V1872106U2 Primary stored-1 1 1	34	4889962	53131	92.0359	9 028 V1872106U2 Primary fresh-1 1 1	28	4929982	53808	91.6217
9 035 V1872106U2 Primary stored-1 2 1	35	4930025	55191	89.3266	9 029 V1872106U2 Primary fresh-1 2 1	29	4982471	54565	91.3126
9 036 V1872106U2 Primary stored-1 3 1	36	4936729	54750	90.1686	9 030 V1872106U2 Primary fresh-1 3 1	30	4928925	52976	93.0407
9 037 V1872106U2 Primary stored-2 1 1	37	4912770	55707	88.1895	9 031 V1872106U2 Primary fresh-2 1 1	31	5019924	54404	92.2712
9 038 V1872106U2 Primary stored-2 2 1	38	4956650	56101	88.3523	9 032 V1872106U2 Primary fresh-2 2 1	32	4992694	54774	91.1508
9 039 V1872106U2 Primary stored-2 3 1	39	4975100	55073	90.3365	9 033 V1872106U2 Primary fresh-2 3 1	33	4959224	54632	90.7751
			Mean	89.7349				Mean	91.6954
			n	6				n	6
			SD	1.4361				SD	0.8291
			%CV	1.6				%CV	0.9
%Change =	-2.1								
Duration:	25	Days							

$$\%Change = \frac{\text{mean response of stored solutions} - \text{mean response of comparison solutions}}{\text{mean response of comparison solutions}} \times 100$$

Criteria: To be deemed stable, % Change upon storage for analyte must be within ±15.0% and %CV must be within ≤15.0%.
Meets Criteria: YES

Table 34: Stability of Zidebactam Primary Solution in Deionized Water at -20 °C (Continued)

Study: V1872106U2
Run: 12
Container: Glass

Analyte	Sample Name	Sequence Number	Analyte Peak Area	IS Peak Area	Peak Area Ratio		Stored	Fresh
Zidebactam 160 mg/mL	12 057 V1872106U2 Primary fresh-1 1 1	57	6395288	43481	147.0824	Lot	V1872106U2-2-1	V1872106U2-31-1
	12 058 V1872106U2 Primary fresh-1 2 1	58	6510560	43940	148.1693	Prep Date	21-Jul-22	20-Sep-22
	12 059 V1872106U2 Primary fresh-1 3 1	59	6638070	45716	145.2023			
	12 060 V1872106U2 Primary fresh-2 1 1	60	6591301	44820	147.0616			
	12 061 V1872106U2 Primary fresh-2 2 1	61	6519385	45470	143.3777			
	12 062 V1872106U2 Primary fresh-2 3 1	62	6416012	45079	142.3282	Avg	153.1481	145.5369
	12 063 V1872106U2 Primary stored-1 1 1	63	7142353	44327	161.1287	Std Dev	7.50048	2.31139
	12 064 V1872106U2 Primary stored-1 2 1	64	7211902	46450	155.2616	%CV	4.9	1.6
	12 065 V1872106U2 Primary stored-1 3 1	65	7203453	45510	158.2829	%Change	5.2	
	12 066 V1872106U2 Primary stored-2 1 1	66	6547190	46825	139.8225			
	12 067 V1872106U2 Primary stored-2 2 1	67	6945315	45065	154.1177			
	12 068 V1872106U2 Primary stored-2 3 1	68	6751274	44926	150.2754	Duration	61	days

$$\%\text{Change} = \frac{\text{mean response of stored solutions} - \text{mean response of comparison solutions}}{\text{mean response of comparison solutions}} \times 100$$

Criteria: To be deemed stable, % Change upon storage for analyte must be within ±15.0% and %CV must be within ≤15.0%.
Meets Criteria: YES

Table 35: Stability of Zidebactam Spiking Solution in Deionized Water at Room Temperature

Study: V1872106P2
Run: 2
Container: Polypropylene

Analyte	Sample Name	Sequence Number	Analyte Peak Area	IS Peak Area	Peak Area Ratio		Bench Top	Refrigerated	-20 °C Reference
Zidebactam	Spike low -20°C-1	3	9462	491079	0.0193	Lot	V1872106P2-7-1	V1872106P2-7-2	V1872106P2-7-3
1.5 mcg/mL	Spike low -20°C-1	9	8801	501538	0.0175		compared to Refr	Compared to -20°C	
	Spike low -20°C-1	15	8647	502203	0.0172				
	Spike low -20°C-2	4	11966	487948	0.0245				
	Spike low -20°C-2	10	11868	495240	0.0240				
	Spike low -20°C-2	16	12302	494351	0.0249	Avg	0.0186	0.0215	0.0212
	Spike low Refr-1	5	9424	497618	0.0189	Std Dev	0.00078	0.00711	0.00361
	Spike low Refr-1	11	8973	492660	0.0182	%CV	4.2	33.1	17.0
	Spike low Refr-1	17	9345	259831	0.0360	%Change	-13.5	1.1	N/A
	Spike low Refr-2	6	9172	497896	0.0184				
	Spike low Refr-2	12	9363	491895	0.0190				
	Spike low Refr-2	18	9206	504247	0.0183	Duration	21 hours		
	Spike low RT-1	7	8885	510117	0.0174				
	Spike low RT-1	13	9601	497905	0.0193				
	Spike low RT-1	19	9519	504086	0.0189				
	Spike low RT-2	8	9069	508325	0.0178				
	Spike low RT-2	14	9406	502063	0.0187				
	Spike low RT-2	20	9679	500883	0.0193				

$$\%\text{Change} = \frac{\text{mean response of stored solutions} - \text{mean response of comparison solutions}}{\text{mean response of comparison solutions}} \times 100$$

Criteria: To be deemed stable, % Change upon storage for analyte must be within ±15.0% and %CV must be within ≤15.0%.
Meets Criteria: NO. Failed due to high %CV. Data from V1872106U2 Zidebactam Spiking Solution in Deionized Water at Room Temperature for 17.75 hours is reported in the next page.

Table 35: Stability of Zidebactam Spiking Solution in Deionized Water at Room Temperature (Continued)

Study: V1872106U2
Run: 3
Container: Polypropylene

Analyte	Sample Name	Sequence Number	Analyte Peak Area	IS Peak Area	Peak Area Ratio		Bench Top	Refrigerated	-20 °C Reference
Zidebactam 300 mcg/mL	3 003 V1872106U2 Spike low -20°C-1 1 1	3	48648	71445	0.6809	Lot	V18721062U2-7-4 compared to Refr	V18721062U2-7-5 Compared to -20°C	V18721062U2-7-6
	3 004 V1872106U2 Spike low -20°C-1 2 1	4	47266	71530	0.6608				
	3 005 V1872106U2 Spike low -20°C-1 3 1	5	48604	71728	0.6776				
	3 006 V1872106U2 Spike low -20°C-2 1 1	6	46856	71893	0.6517				
	3 007 V1872106U2 Spike low -20°C-2 2 1	7	47254	72325	0.6534				
	3 008 V1872106U2 Spike low -20°C-2 3 1	8	47607	69761	0.6824	Avg	0.6592	0.6617	0.6678
	3 009 V1872106U2 Spike low Refr-1 1 1	9	46091	71404	0.6455	Std Dev	0.00742	0.01150	0.01413
	3 010 V1872106U2 Spike low Refr-1 2 1	10	47706	70985	0.6721	%CV	1.1	1.7	2.1
	3 011 V1872106U2 Spike low Refr-1 3 1	11	46231	70901	0.6521	%Change	-0.4	-0.9	N/A
	3 012 V1872106U2 Spike low Refr-2 1 1	12	47641	72348	0.6585				
	3 013 V1872106U2 Spike low Refr-2 2 1	13	48275	71681	0.6735				
	3 014 V1872106U2 Spike low Refr-2 3 1	14	47749	71398	0.6688	Duration	17.75 hours		
	3 015 V1872106U2 Spike low RT-1 1 1	15	46353	70274	0.6596				
	3 016 V1872106U2 Spike low RT-1 2 1	16	46100	71065	0.6487				
	3 017 V1872106U2 Spike low RT-1 3 1	17	46824	70742	0.6619				
	3 018 V1872106U2 Spike low RT-2 1 1	18	47068	70552	0.6671				
	3 019 V1872106U2 Spike low RT-2 2 1	19	46743	71676	0.6521				
	3 020 V1872106U2 Spike low RT-2 3 1	20	47764	71727	0.6659				

Criteria: To be deemed stable, % Change upon storage for analyte must be within ±15.0% and %CV must be within ≤15.0%.
Meets Criteria: YES

Table 35: Stability of Zidebactam Spiking Solution in Deionized Water at Room Temperature (Continued)

Study: V1872106U2
Run: 3
Container: Polypropylene

Analyte	Sample Name	Sequence Number	Analyte Peak Area	IS Peak Area	Peak Area Ratio		Bench Top	Refrigerated	-20 °C Reference
Zidebactam	3 021 V1872106U2 Spike high -20°C-1 1 1	21	4572243	70263	65.0733	Lot	V18721062U2-8-1	V18721062U2-8-2	V18721062U2-8-3
30,000 mcg/mL	3 022 V1872106U2 Spike high -20°C-1 2 1	22	4491468	71708	62.6355		compared to Refr	Compared to -20°C	
	3 023 V1872106U2 Spike high -20°C-1 3 1	23	4518595	71162	63.4973				
	3 024 V1872106U2 Spike high -20°C-2 1 1	24	4459474	71160	62.6683				
	3 025 V1872106U2 Spike high -20°C-2 2 1	25	4567708	71493	63.8903				
	3 026 V1872106U2 Spike high -20°C-2 3 1	26	4443614	71038	62.5526	Avg	64.4876	64.0624	63.3862
	3 027 V1872106U2 Spike high Refr-1 1 1	27	4384160	71276	61.5096	Std Dev	0.65091	2.68461	0.98858
	3 028 V1872106U2 Spike high Refr-1 2 1	28	4294992	70371	61.0336	%CV	1.0	4.2	1.6
	3 029 V1872106U2 Spike high Refr-1 3 1	29	4491832	71960	62.4212	%Change	0.7	1.1	N/A
	3 030 V1872106U2 Spike high Refr-2 1 1	30	4673188	70197	66.5725				
	3 031 V1872106U2 Spike high Refr-2 2 1	31	4760824	71302	66.7699				
	3 032 V1872106U2 Spike high Refr-2 3 1	32	4733414	71645	66.0676	Duration	17.75 hours		
	3 033 V1872106U2 Spike high RT-1 1 1	33	4528619	70459	64.2731				
	3 034 V1872106U2 Spike high RT-1 2 1	34	4641357	73083	63.5080				
	3 035 V1872106U2 Spike high RT-1 3 1	35	4610917	71399	64.5796				
	3 036 V1872106U2 Spike high RT-2 1 1	36	4619486	71895	64.2532				
	3 037 V1872106U2 Spike high RT-2 2 1	37	4659281	71204	65.4357				
	3 038 V1872106U2 Spike high RT-2 3 1	38	4668585	71962	64.8757				

$$\%\text{Change} = \frac{\text{mean response of stored solutions} - \text{mean response of comparison solutions}}{\text{mean response of comparison solutions}} \times 100$$

Criteria: To be deemed stable, % Change upon storage for analyte must be within ±15.0% and %CV must be within ≤15.0%.
Meets Criteria: YES

Table 36: Stability of Zidebactam Spiking Solution in Deionized Water at -20 °C

Study: V1872106U2
Run: 9
Container: Polypropylene

Concentration: 300 mcg/mL									
Stability Data (Stored)					Comparison Data (Fresh)				
Lot #:	V1872106U2-4-1				Lot #:	V1872106U2-22-1			
Prepared:	21-Jul-2022				Prepared:	15-Aug-2022			
Sample ID	Seq #	Drug Peak Area	IS Peak Area	Peak Area Ratio	Sample ID	Seq #	Drug Peak Area	IS Peak Area	Peak Area Ratio
9 009 V1872106U2 Spike low stored-1 1 1	9	38263	53582	0.7141	9 003 V1872106U2 Spike low fresh-1 1 1	3	40256	54025	0.7451
9 010 V1872106U2 Spike low stored-1 2 1	10	38757	54465	0.7116	9 004 V1872106U2 Spike low fresh-1 2 1	4	39981	54394	0.7350
9 011 V1872106U2 Spike low stored-1 3 1	11	38892	54006	0.7201	9 005 V1872106U2 Spike low fresh-1 3 1	5	39548	53018	0.7459
9 012 V1872106U2 Spike low stored-2 1 1	12	38202	53365	0.7159	9 006 V1872106U2 Spike low fresh-2 1 1	6	40384	52849	0.7641
9 013 V1872106U2 Spike low stored-2 2 1	13	39876	54343	0.7338	9 007 V1872106U2 Spike low fresh-2 2 1	7	40030	54425	0.7355
9 014 V1872106U2 Spike low stored-2 3 1	14	37889	54140	0.6998	9 008 V1872106U2 Spike low fresh-2 3 1	8	39846	54579	0.7301
			Mean	0.7159				Mean	0.7426
			n	6				n	6
			SD	0.0111				SD	0.0122
			%CV	1.6				%CV	1.6
%Change =	-3.6								
Duration:	25	Days							

$$\%\text{Change} = \frac{\text{mean response of stored solutions} - \text{mean response of comparison solutions}}{\text{mean response of comparison solutions}} \times 100$$

Criteria: To be deemed stable, % Change upon storage for analyte must be within ±15.0% and %CV must be within ≤15.0%.
Meets Criteria: YES

Table 36: Stability of Zidebactam Spiking Solution in Deionized Water at -20 °C (Continued)

Study: V1872106U2
Run: 9
Container: Polypropylene

Concentration: 30,000 mcg/mL									
Stability Data (Stored)					Comparison Data (Fresh)				
Lot #:	V1872106U2-4-1				Lot #:	V1872106U2-22-1			
Prepared:	21-Jul-2022				Prepared:	15-Aug-2022			
Sample ID	Seq #	Drug Peak Area	IS Peak Area	Peak Area Ratio	Sample ID	Seq #	Drug Peak Area	IS Peak Area	Peak Area Ratio
9 021 V1872106U2 Spike high stored-1 1 1	21	3782495	54413	69.5145	9 015 V1872106U2 Spike high fresh-1 1 1	15	3759255	53829	69.8370
9 022 V1872106U2 Spike high stored-1 2 1	22	3741223	54289	68.9131	9 016 V1872106U2 Spike high fresh-1 2 1	16	3793491	54048	70.1874
9 023 V1872106U2 Spike high stored-1 3 1	23	3741273	54767	68.3125	9 017 V1872106U2 Spike high fresh-1 3 1	17	3762946	55443	67.8705
9 024 V1872106U2 Spike high stored-2 1 1	24	3519263	54803	64.2166	9 018 V1872106U2 Spike high fresh-2 1 1	18	3813334	55022	69.3056
9 025 V1872106U2 Spike high stored-2 2 1	25	3627002	54256	66.8498	9 019 V1872106U2 Spike high fresh-2 2 1	19	3775447	54443	69.3468
9 026 V1872106U2 Spike high stored-2 3 1	26	3591727	54910	65.4112	9 020 V1872106U2 Spike high fresh-2 3 1	20	3809358	53956	70.6012
			Mean	67.2030				Mean	69.5248
			n	6				n	6
			SD	2.0858				SD	0.9499
			%CV	3.1				%CV	1.4
%Change =	-3.3								
Duration:	25	Days							

$$\%\text{Change} = \frac{\text{mean response of stored solutions} - \text{mean response of comparison solutions}}{\text{mean response of comparison solutions}} \times 100$$

Criteria: To be deemed stable, % Change upon storage for analyte must be within ±15.0% and %CV must be within ≤15.0%.
Meets Criteria: YES

Table 36: Stability of Zidebactam Spiking Solution in Deionized Water at -20 °C (Continued)

Study: V1872106U2
Run: 12
Container: Polypropylene

Analyte	Sample Name	Sequence Number	Analyte Peak Area	IS Peak Area	Peak Area Ratio		Stored	Fresh
Zidebactam 30.0 mcg/mL	12 031 V1872106U2 Spike low fresh-1 1 1	31	61273	41966	1.4601	Lot	V1872106U2-4-1	V1872106U2-34-1
	12 032 V1872106U2 Spike low fresh-1 2 1	32	65893	43994	1.4978	Prep Date	21-Jul-22	20-Sep-22
	12 033 V1872106U2 Spike low fresh-1 3 1	33	65670	44788	1.4662			
	12 034 V1872106U2 Spike low fresh-2 1 1	34	62602	44627	1.4028			
	12 035 V1872106U2 Spike low fresh-2 2 1	35	61173	44881	1.3630			
	12 036 V1872106U2 Spike low fresh-2 3 1	36	63454	44837	1.4152	Avg	1.2110	1.4342
	12 037 V1872106U2 Spike low stored-1 1 1	37	56861	44926	1.2657	Std Dev	0.05267	0.04930
	12 038 V1872106U2 Spike low stored-1 2 1	38	57797	44849	1.2887	%CV	4.3	3.4
	12 039 V1872106U2 Spike low stored-1 3 1	39	55195	46147	1.1961	%Change	-15.6	
	12 040 V1872106U2 Spike low stored-2 1 1	40	52701	44774	1.1770			
	12 041 V1872106U2 Spike low stored-2 2 1	41	53169	45499	1.1686			
	12 042 V1872106U2 Spike low stored-2 3 1	42	52331	44722	1.1701	Duration	61	days
Zidebactam 3,000 mcg/mL	12 044 V1872106U2 Spike high fresh-1 1 1	44	5070025	44066	115.0553	Lot	V1872106U2-4-1	V1872106U2-34-1
	12 045 V1872106U2 Spike high fresh-1 2 1	45	5297563	44976	117.7864	Prep Date	21-Jul-22	20-Sep-22
	12 046 V1872106U2 Spike high fresh-1 3 1	46	5549046	42693	129.9755			
	12 047 V1872106U2 Spike high fresh-2 1 1	47	5610729	43504	128.9704			
	12 048 V1872106U2 Spike high fresh-2 2 1	48	4763342	43658	109.1058			
	12 049 V1872106U2 Spike high fresh-2 3 1	49	5160410	43236	119.3545	Avg	111.6635	120.0413
	12 050 V1872106U2 Spike high stored-1 1 1	50	5001460	45790	109.2260	Std Dev	6.65019	8.10456
	12 051 V1872106U2 Spike high stored-1 2 1	51	4952186	44738	110.6931	%CV	6.0	6.8
	12 052 V1872106U2 Spike high stored-1 3 1	52	5187790	44557	116.4304	%Change	-7.0	
	12 053 V1872106U2 Spike high stored-2 1 1	53	4618099	45003	102.6176			
	12 054 V1872106U2 Spike high stored-2 2 1	54	4955409	45389	109.1764			
	12 055 V1872106U2 Spike high stored-2 3 1	55	5328551	43735	121.8372	Duration	61	days

$$\%\text{Change} = \frac{\text{mean response of stored solutions} - \text{mean response of comparison solutions}}{\text{mean response of comparison solutions}} \times 100$$

Criteria: To be deemed stable, % Change upon storage for analyte must be within ±15.0% and %CV must be within ≤15.0%.
Meets Criteria: YES for the high concentration, but not at the low concentration. Spikes should only be used for the 25 days established previously.

Table 37: Stability of Cefotaxime Working Internal Standard Solution in Deionized Water at Room Temperature

Study: V1872106U1
Run 3
Container: Glass

Analyte	Sample Name	Sequence Number	Analyte Peak Area	IS Peak Area	Peak Area Ratio		Bench Top	Refrigerated	-20 °C Reference
Cefotaxime ~500 mcg/mL	3 059 V1872106U1 WIS -20°C-1 1 1	59	1313192	485422	2.7053	Lot	V1872106U1-11-1 compared to Refr	V1872106U1-11-2 Compared to -20°C	V1872106U1-11-3
	3 060 V1872106U1 WIS -20°C-1 3 1	60	1339652	522363	2.5646				
	3 061 V1872106U1 WIS -20°C-1 2 1	61	1340502	550053	2.4370				
	3 062 V1872106U1 WIS -20°C-2 2 1	62	1336674	522445	2.5585				
	3 063 V1872106U1 WIS -20°C-2 3 1	63	1341412	560588	2.3929				
	3 064 V1872106U1 WIS -20°C-2 1 1	64	1343379	587756	2.2856	Avg	2.5534	2.7884	2.4906
	3 065 V1872106U1 WIS Refr-1 1 1	65	1335207	450429	2.9643	Std Dev	0.129	0.232	0.149
	3 066 V1872106U1 WIS Refr-1 3 1	66	1348441	467684	2.8832	%CV	5.0	8.3	6.0
	3 067 V1872106U1 WIS Refr-1 2 1	67	1335092	554007	2.4099	%Change	-8.4	12.0	N/A
	3 068 V1872106U1 WIS Refr-2 2 1	68	1321946	453246	2.9166				
	3 069 V1872106U1 WIS Refr-2 3 1	69	1326727	447448	2.9651				
	3 070 V1872106U1 WIS Refr-2 1 1	70	1335122	515294	2.5910	Duration	15.5 hours		
	3 071 V1872106U1 WIS RT-1 1 1	71	1336567	547268	2.4423				
	3 072 V1872106U1 WIS RT-1 2 1	72	1341696	504955	2.6571				
	3 073 V1872106U1 WIS RT-1 3 1	73	1329061	557140	2.3855				
	3 074 V1872106U1 WIS RT-2 3 1	74	1340202	529252	2.5323				
	3 075 V1872106U1 WIS RT-2 2 1	75	1319308	483530	2.7285				
	3 076 V1872106U1 WIS RT-2 1 1	76	1347636	523394	2.5748				

$$\%\text{Change} = \frac{\text{mean response of stored solutions} - \text{mean response of comparison solutions}}{\text{mean response of comparison solutions}} \times 100$$

Criteria: To be deemed stable, % Change upon storage for analyte must be within ±20.0% and %CV must be within ≤15.0%.
Meets Criteria: YES

Table 37: Stability of Cefotaxime Working Internal Standard Solution in Deionized Water at Room Temperature (Continued)

Study: V1872106P2A1
Run 34
Container: Glass

Concentration: 50 mcg/mL									
Stability Data (RT)					Comparison Data (Refrigerated)				
Lot #:	V1872106P2-83-3				Lot #:	V1872106P2-83-4			
Prepared:					Prepared:				
Sample ID	Seq #	IS Peak Area	Analyte Peak Area	Peak Area Ratio	Sample ID	Seq #	IS Peak Area	Analyte Peak Area	Peak Area Ratio
34 007 WIS RT-1 1 1	7	2151178	612037	3.5148	34 005 WIS Refr-1 1 1	5	2150392	627659	3.4261
34 013 WIS RT-1 2 1	13	2223690	631316	3.5223	34 011 WIS Refr-1 2 1	11	2155987	632806	3.4070
34 019 WIS RT-1 3 1	19	2198539	615941	3.5694	34 017 WIS Refr-1 3 1	17	2170613	632882	3.4297
34 008 WIS RT-2 1 1	8	2176660	610682	3.5643	34 006 WIS Refr-2 1 1	6	2165986	627640	3.4510
34 014 WIS RT-2 2 1	14	2194030	618080	3.5498	34 012 WIS Refr-2 2 1	12	2189975	628746	3.4831
34 020 WIS RT-2 3 1	20	2177484	614087	3.5459	34 018 WIS Refr-2 3 1	18	2124927	620077	3.4269
			Mean	3.5444				Mean	3.4373
			n	6				n	6
			SD	0.0220				SD	0.0264
			%CV	0.6				%CV	0.8
%Change =	3.1								
Duration:	19	Hours							

$$\%\text{Change} = \frac{\text{mean response of stored solutions} - \text{mean response of comparison solutions}}{\text{mean response of comparison solutions}} \times 100$$

Criteria: To be deemed stable, % Change upon storage for analyte must be within ±20.0% and %CV must be within ≤15.0%.
Meets Criteria: YES

17. FIGURES

Figure 1: Typical Calibration Curve

Analytical Run 5 Run Acceptance Parameters for Zidebactam (µg/mL) (Study V1872106P2)

Regression Type	LINEAR
Weighting Factor	1/X**2
Slope	0.066251815168961900
Intercept	-0.000547046228752823
R-Squared	0.996895952383572
LLOQ	0.05
ULOQ	50
Mean Std %Diff	3.4
S.D. Std %Diff	3.5
Mean Std Response	0.064105
S.D. Std Response	0.005106

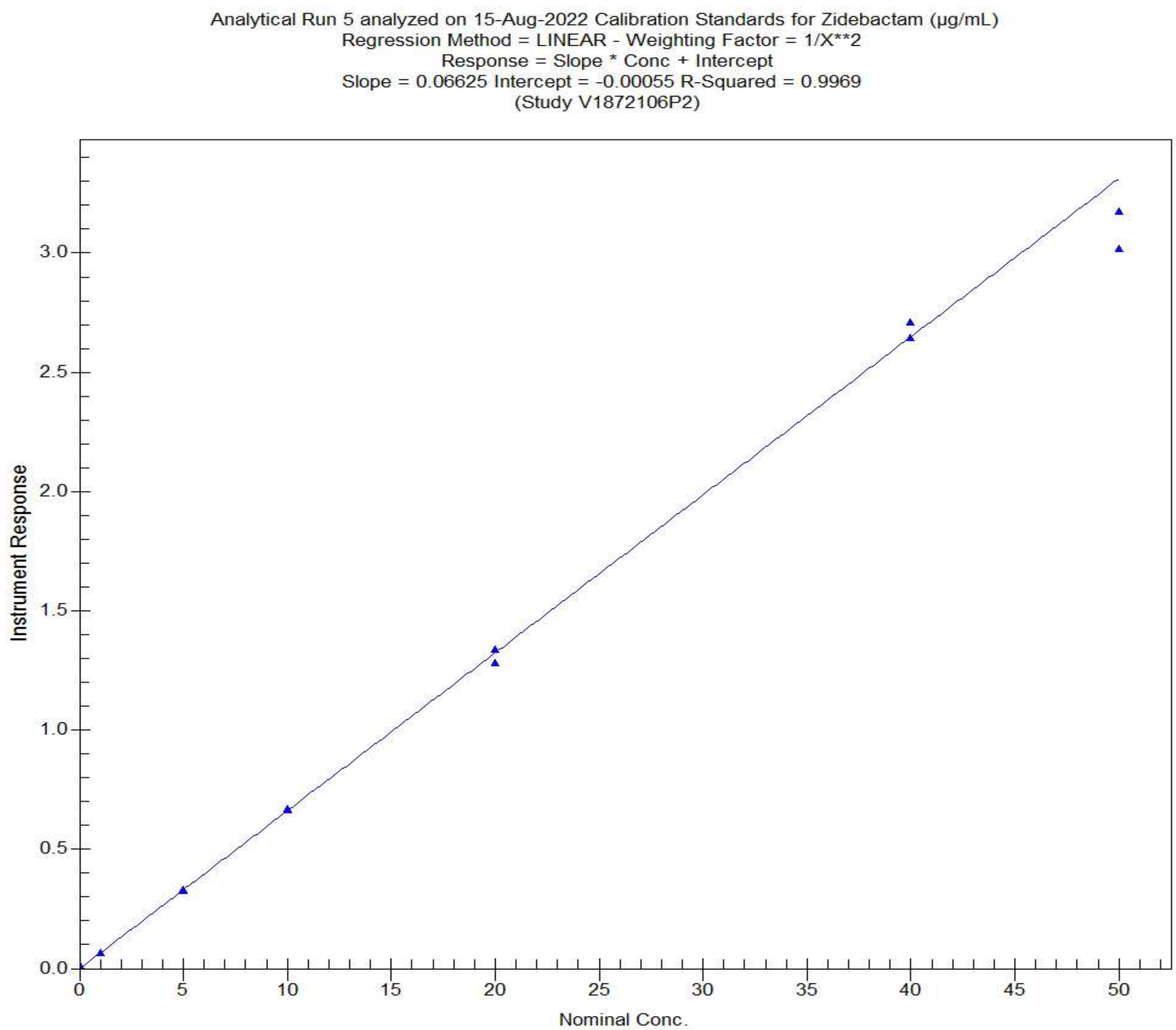

Figure 2: Typical Ion Chromatogram Blank Human Plasma without Internal Standard

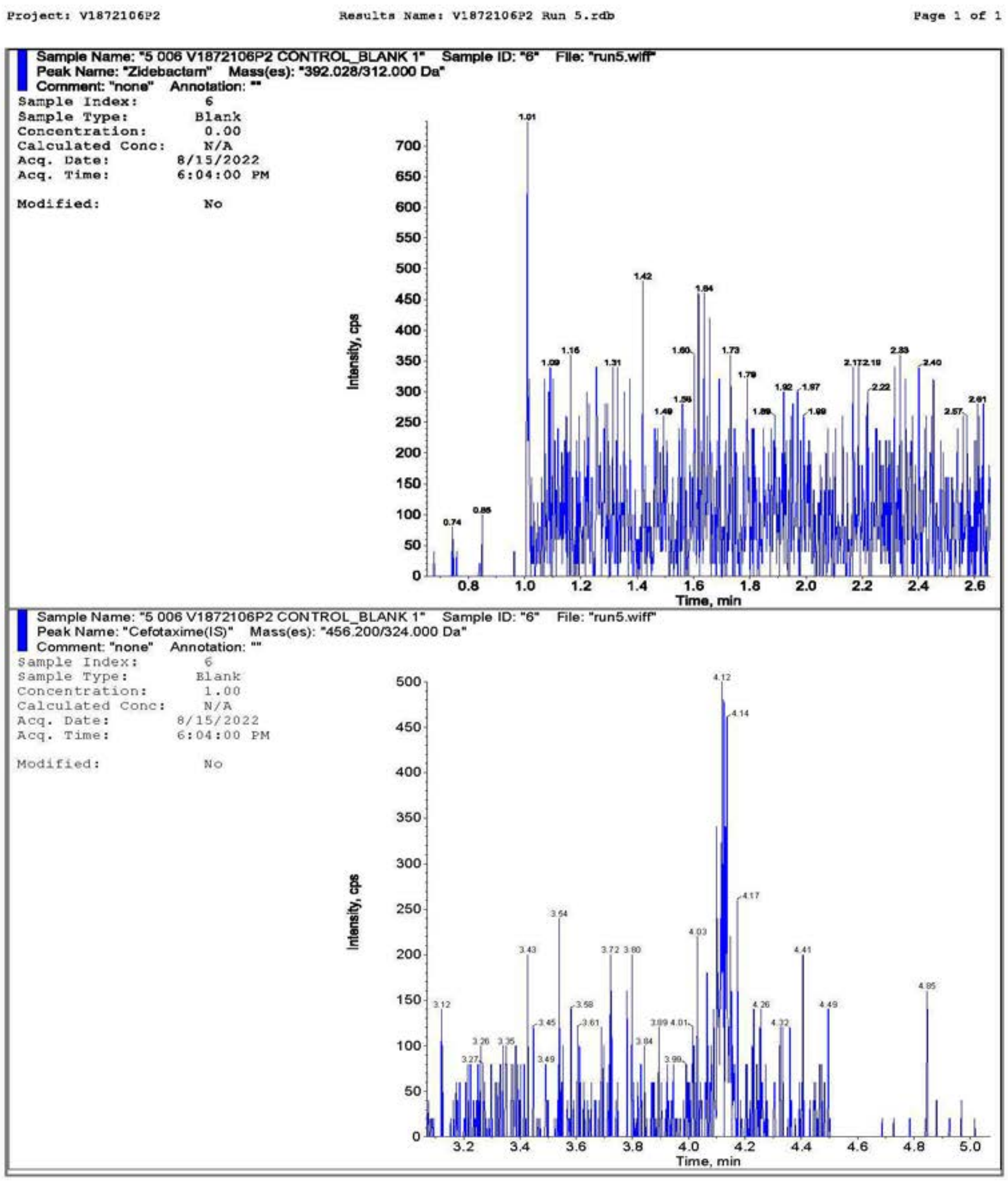

Figure 3: Typical Ion Chromatogram Blank Human Plasma with Internal Standard

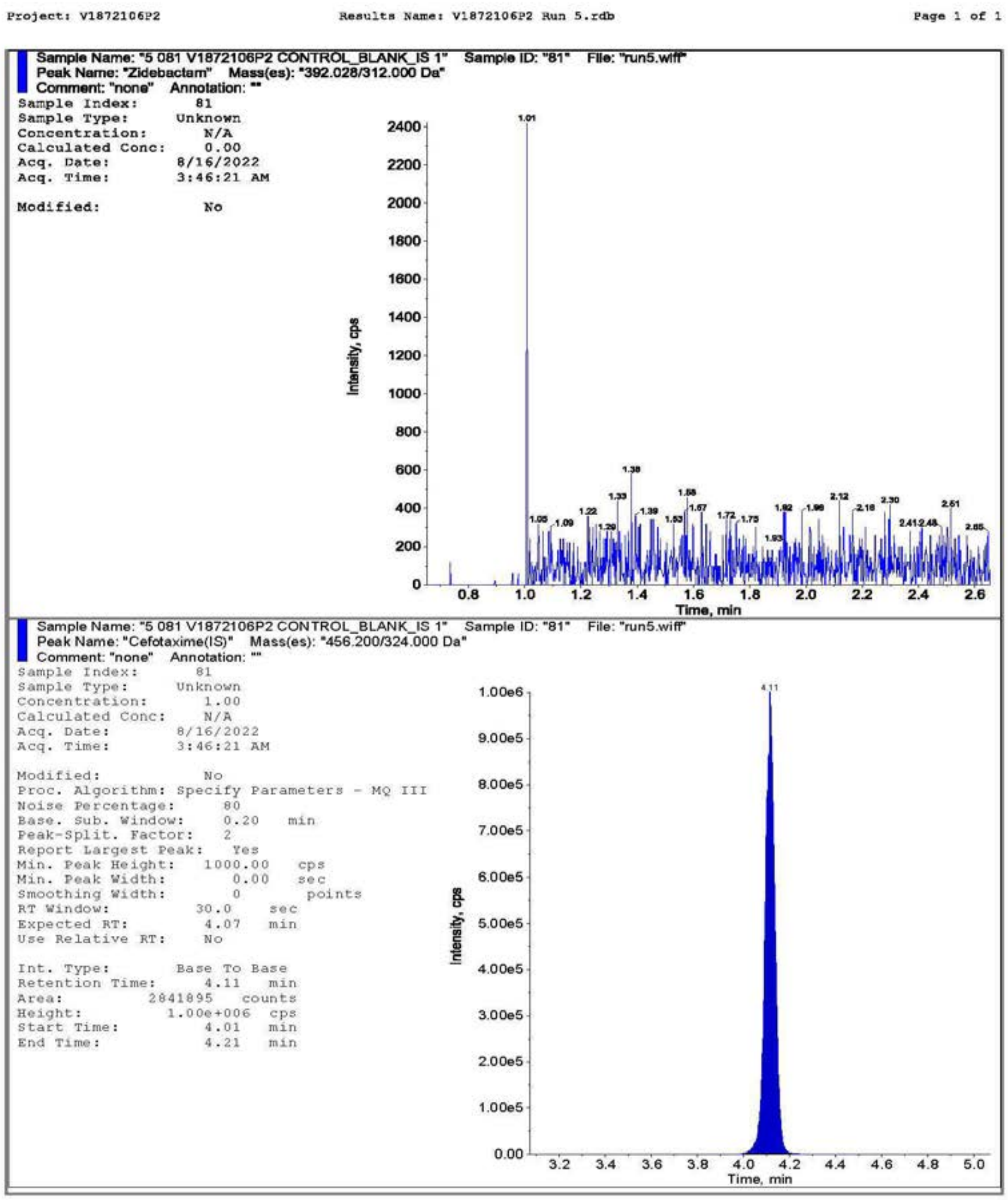

Figure 4: Typical Ion Chromatogram LLOQ Calibration Standard, Zidebactam = 0.0500 mcg/mL

Project: V1872106P2 Results Name: V1872106P2 Run 5.rdb Page 1 of 1

Sample Name: "5 007 V1872106P2 Cal 0.0500 1 1" Sample ID: "7" File: "run5.wiff"
Peak Name: "Zidebactam" Mass(es): "392.028/312.000 Da"
Comment: "none" Annotation: ""

```
Sample Index:        7
Sample Type:         Standard
Concentration:       0.0500
Calculated Conc:     0.00
Acq. Date:           8/15/2022
Acq. Time:           6:11:46 PM

Modified:            No
Proc. Algorithm: Specify Parameters - MQ III
Noise Percentage:    90
Base. Sub. Window:   0.30   min
Peak-Split. Factor:  4
Report Largest Peak: Yes
Min. Peak Height:    560.00   cps
Min. Peak Width:     0.00     sec
Smoothing Width:     0        points
RT Window:           30.0     sec
Expected RT:         1.65     min
Use Relative RT:     No

Int. Type:           Base To Base
Retention Time:      1.60     min
Area:                7874     counts
Height:              2.19e+003   cps
Start Time:          1.53     min
End Time:            1.68     min
```

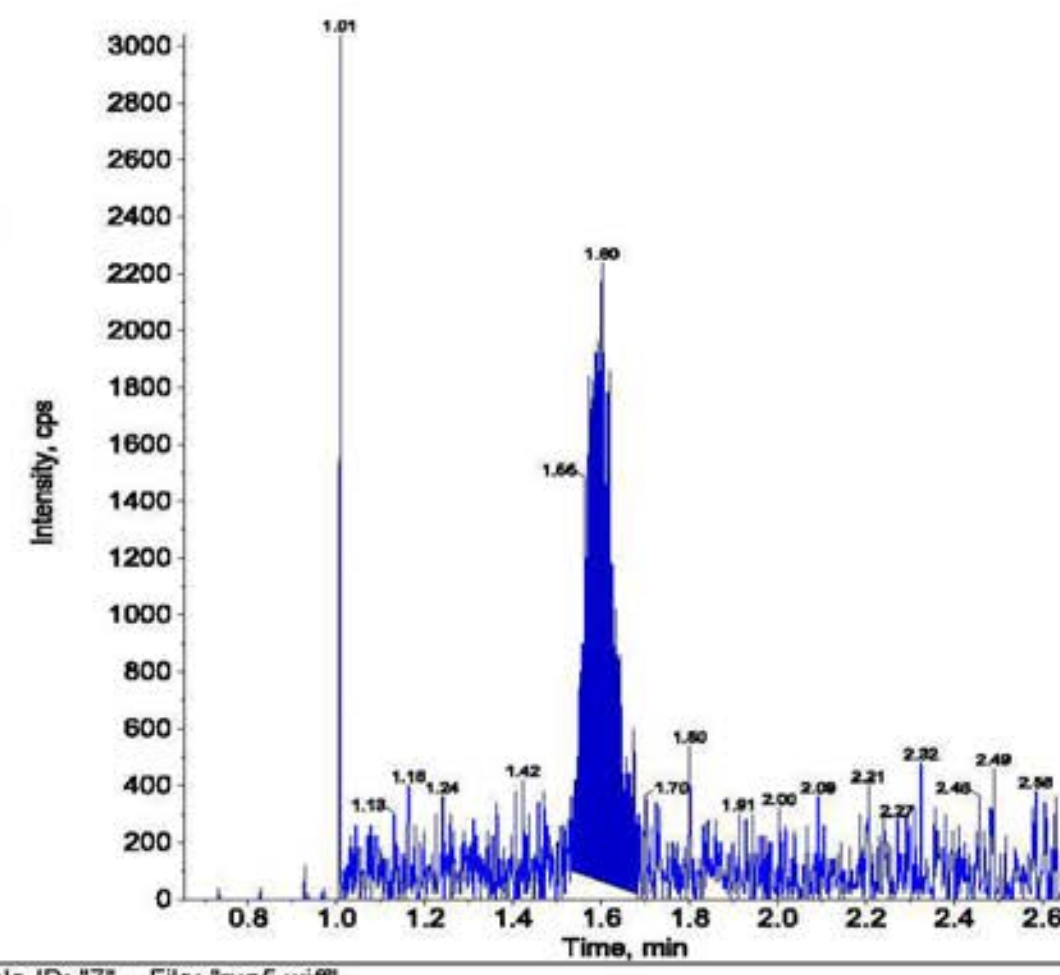

Sample Name: "5 007 V1872106P2 Cal 0.0500 1 1" Sample ID: "7" File: "run5.wiff"
Peak Name: "Cefotaxime(IS)" Mass(es): "456.200/324.000 Da"
Comment: "none" Annotation: ""

```
Sample Index:        7
Sample Type:         Standard
Concentration:       1.00
Calculated Conc:     N/A
Acq. Date:           8/15/2022
Acq. Time:           6:11:46 PM

Modified:            No
Proc. Algorithm: Specify Parameters - MQ III
Noise Percentage:    80
Base. Sub. Window:   0.20   min
Peak-Split. Factor:  2
Report Largest Peak: Yes
Min. Peak Height:    1000.00  cps
Min. Peak Width:     0.00     sec
Smoothing Width:     0        points
RT Window:           30.0     sec
Expected RT:         4.07     min
Use Relative RT:     No

Int. Type:           Base To Base
Retention Time:      4.13     min
Area:                2941794  counts
Height:              9.87e+005   cps
Start Time:          4.02     min
End Time:            4.22     min
```

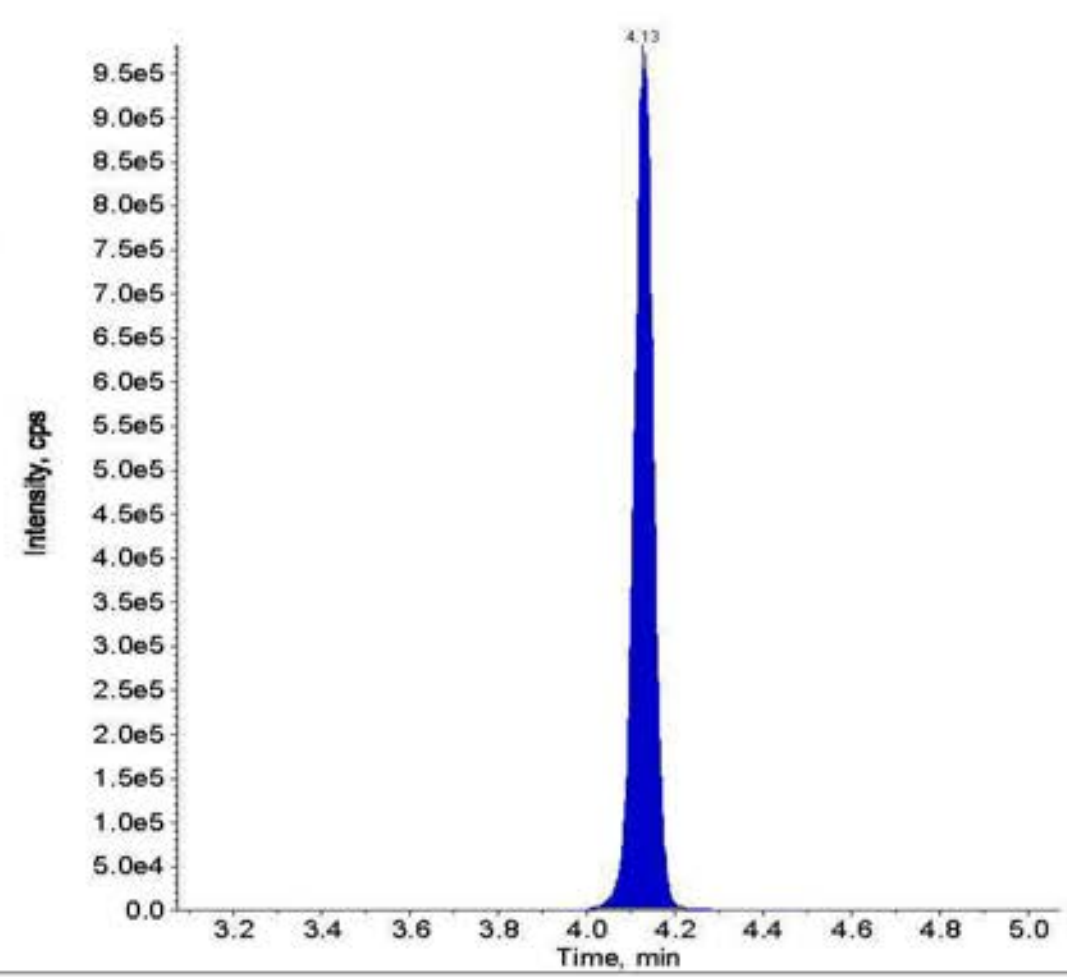

Figure 5: Typical Ion Chromatogram Calibration Standard, Zidebactam = 20.0 mcg/mL

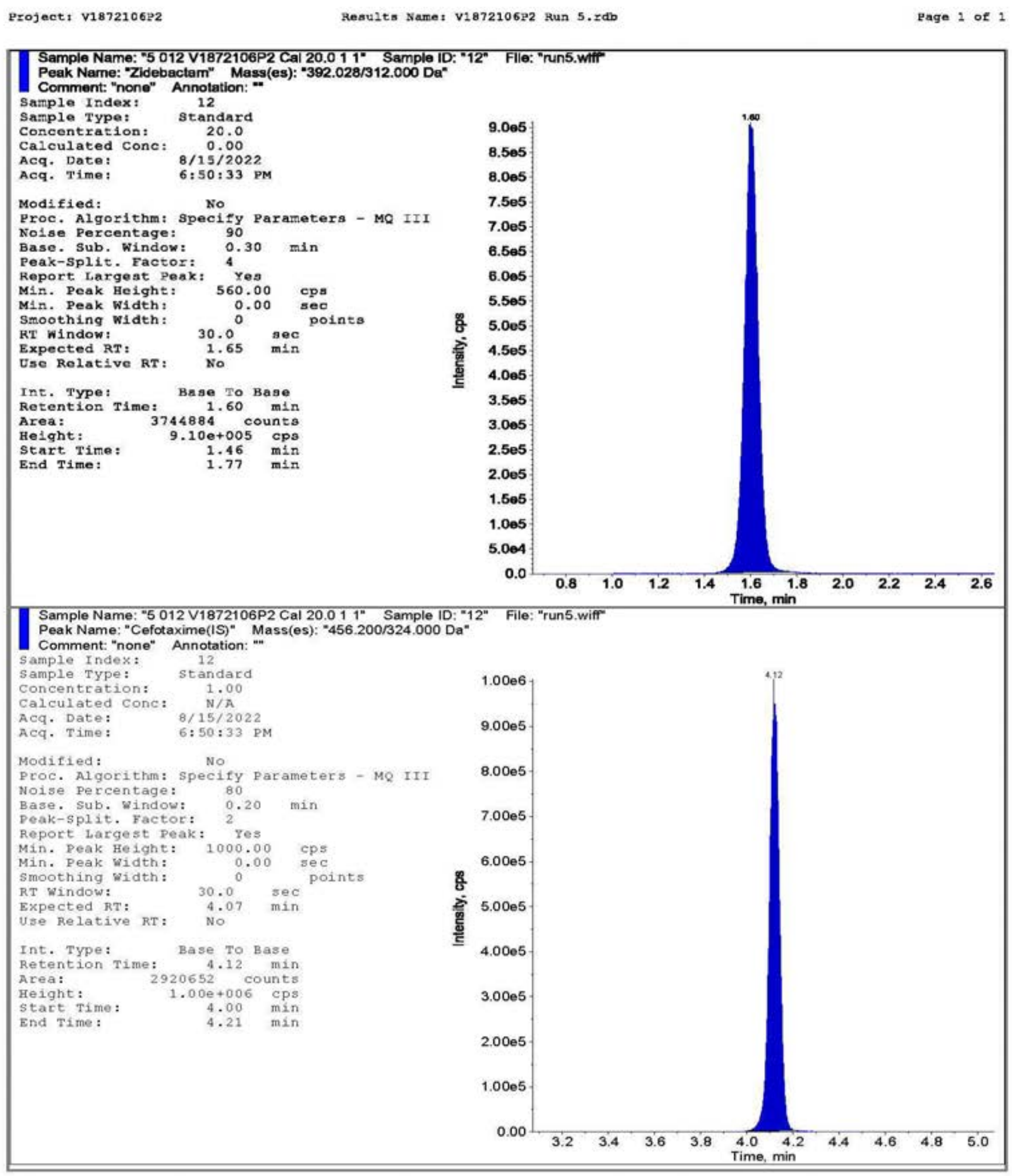

Figure 6: Typical Ion Chromatogram ULOQ Calibration Standard, Zidebactam = 50.0 mcg/mL

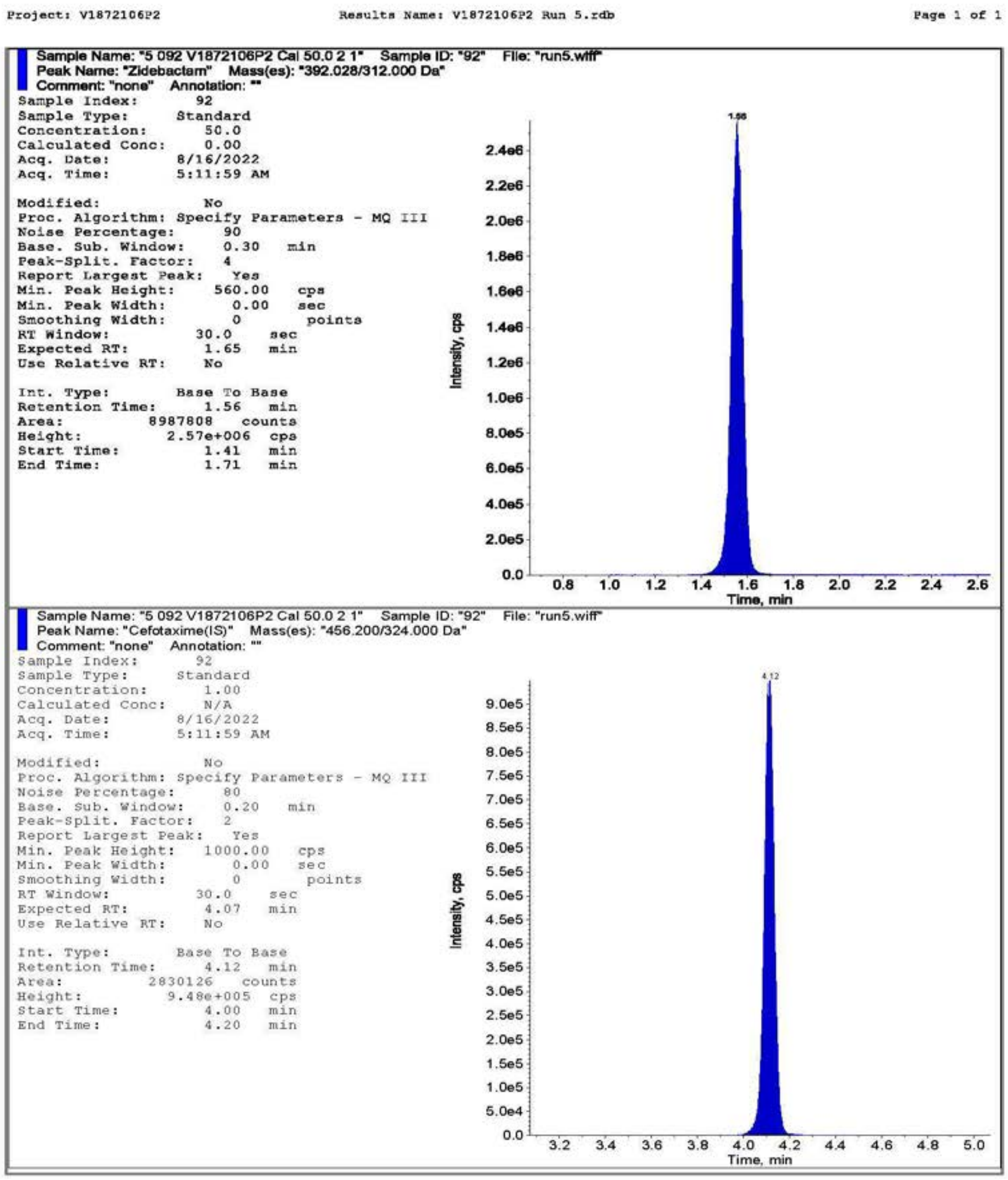

Figure 7: Typical Ion Chromatogram Cross-Talk Evaluation High QC No IS

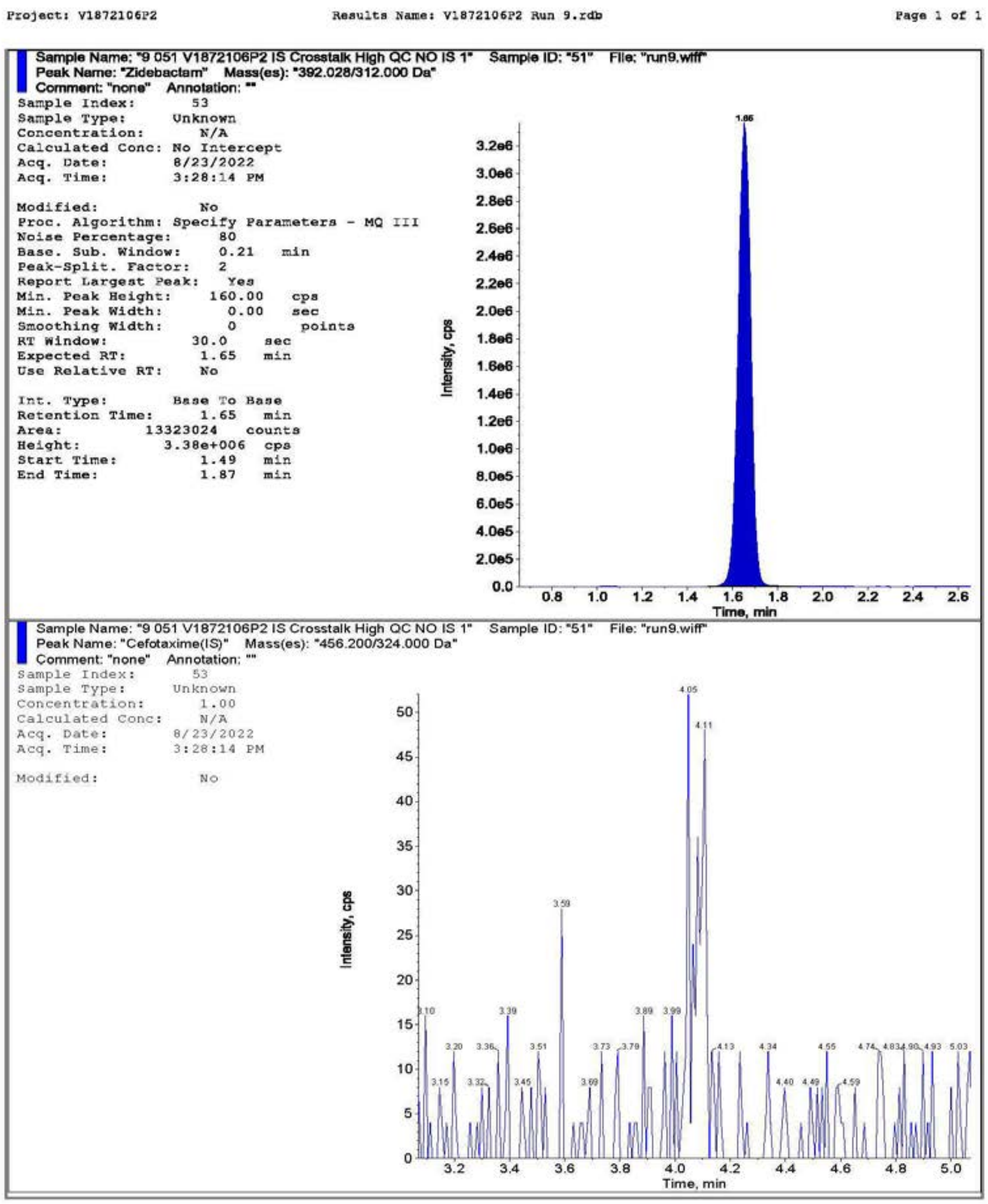

KCAS Bioanalytical & Biomarker Services	**TEST PROCEDURE**	Doc #: **TP-80194**
		Rev #: **01**
		Effective Date: **1/13/2023**

Title: **LC-MS/MS Analysis of Zidebactam in Treated Human Sodium Heparin Plasma (100 mcL, 0.0500 – 50.0 mcg/mL; Prior to MES Treatment)**
Validation Number: V1872106P2

1. GENERAL INFORMATION

1.1. Client Name: Dynport Vaccine Company

1.2. Reference: Validation V1872106P2

2. TABLE OF CONTENTS

1. GENERAL INFORMATION 1
1.1. Client Name: Dynport Vaccine Company 1
1.2. Reference: Validation V1872106P2 1
2. TABLE OF CONTENTS 1
3. SAMPLE REQUIREMENTS 3
4. METHOD SUMMARY 3
5. SPECIAL HANDLING OF BIOLOGICAL SAMPLES 3
6. DATA COLLECTION AND ANALYSIS SOFTWARE 3
7. INSTRUMENTS, EQUIPMENT, AND CONDITIONS 4
7.1. LC and MS Conditions 4
Table 1: HPLC System 4
7.1.1. Gradient Table 5
7.1.2. Autosampler Settings 5
7.1.3. Mass Spectrometer System 5
8. REAGENTS, STANDARDS, AND SUPPLIES 7
Table 2: Reagents, Standards, and Supplies 7
9. SAFETY 7
10. PROCEDURE 8
10.1. Preparation of Analyte Stock Solutions and Dilutions 8

CONFIDENTIAL

Serial Number: c4d37b88-d6c5-4833-95e6-1964235b97b0 accessed by Bryan Sitzmann (SITZMANNB) on Tue, 17 Jan 2023 23:46:48 GMT.

10.1.1. Zidebactam Primary Solution, 2.00 mg/mL 8
10.1.2. Internal Standard Primary Solution, 5 mg/mL Cefotaxime Sodium 8
10.1.3. Internal Standard Working Solution, 50.0 mcg/mL Cefotaxime Sodium 8
10.2. Preparation of Other Reagents 8
10.2.1. Mobile Phase A: 10 mM Ammonium Acetate in Deionized Water 8
10.2.2. Mobile Phase B: 95/5 Acetonitrile / 10mM Ammonium Acetate 9
10.2.3. Rinse: 90/10 Acetone/ Deionized Water 9
10.2.4. 1 N Sodium Hydroxide (NaOH) in Deionized Water 9
10.2.5. MES ([2-(N-Morpholino) ethane sulphonic acid hydrate]) Buffer pH 6.4, ~0.1M 9
10.2.6. Reconstitution Solvent: 2/98 Acetonitrile/Deionized Water 9
10.3. Preparation of Standards and QC Samples in Ice Water Bath 9
10.3.1. Treated Human Sodium Heparin Plasma (Human Sodium Heparin Plasma: MES Buffer 1:1) 9
10.3.2. Preparation of Standard and QC Spiking Solutions 10
10.3.3. Preparation of Standards and QC Samples in Sodium Heparin Plasma Matrix 10
10.4. Extraction Procedure 11
10.5. Dilutions 11
10.6. Calculations 12
11. REFERENCE CHROMATOGRAMS 12
Figure 1: Blank without IS 12
Figure 2: Blank with IS 12
Figure 3: LLOQ 13
12. REVISION HISTORY 13
Table 3: Revision History 13

CONFIDENTIAL

Serial Number: c4d37b88-d6c5-4833-95e6-1964235b97b0 accessed by Bryan Sitzmann (SITZMANNB) on Tue, 17 Jan 2023 23:46:48 GMT.

3. SAMPLE REQUIREMENTS

Human sodium heparin plasma samples (treated with 1:1 MES Buffer pH 6.4, ~0.1M), minimum volume 100 mcL each, for determination of Zidebactam concentration.

Refer to the relevant study protocol for a description of expected samples.

4. METHOD SUMMARY

The method involves 96-well protein precipitation followed by using the Sciex 6500 or 5000 LC/MS/MS systems. The concentration range is 0.0250 – 25.0 mcg/mL for Zidebactam in human plasma, prior to MES treatment.

5. SPECIAL HANDLING OF BIOLOGICAL SAMPLES

The method requires no special handling. Please ensure all "Universal Precautions" are taken.

Unknown plasma samples need to be diluted 1:1 with MES buffer after sample collection. The calibration standard and QC concentrations are adjusted in this test procedure, so the unknown samples do not need to be adjusted for the dilution factor of 2x for the treatment with MES buffer.

Primaries, spiking solutions, calibration standards and QCs should be prepared on wet ice, and each prepared level should be aliquoted immediately and aliquots should be flash frozen in a dry ice/acetone bath for ~1 minute and stored in the freezer immediately after flash freezing.

6. DATA COLLECTION AND ANALYSIS SOFTWARE

Description	Version
Watson LIMS™ (Thermo Fisher Scientific)	7.5 or higher
Analyst® (AB Sciex)	1.7.2 or higher

CONFIDENTIAL

Serial Number: c4d37b88-d6c5-4833-95e6-1964235b97b0 accessed by Bryan Sitzmann (SITZMANNB) on Tue, 17 Jan 2023 23:46:48 GMT.

7. INSTRUMENTS, EQUIPMENT, AND CONDITIONS

Equivalent equipment can be substituted, except where noted in Section 7.1. Minor modifications of conditions are permitted to optimize chromatography, except where noted in Section 7.1. Refer to SOP-00073 for allowable minor modifications that do not require validation.

7.1. LC and MS Conditions

Table 1: HPLC System

**Parameter**	**Parameter Value**
System Controller	Shimadzu Controller, CBM20A
Pump	Shimadzu LC-30AD
Autosampler	Shimadzu SIL-30AC
Column Oven	Shimadzu CTO-30A
MS Interface	ESI
Analytical Column	Fortis Technology Ltd. Fortis C18 4.6 x 50mm, 5 µ
Column Guard Cartridge	Security Guard Cartridge AQ C18, 4.0 x 2.0 mm
Column Temperature (set at)	35°C
Mobile Phase A	10 mM Ammonium Acetate in Deionized Water
Mobile Phase B	95/5 Acetonitrile / 10 mM Ammonium Acetate
Needle Wash Solvent (R0, R3)	90/10 Acetone / Deionized Water
Total Run Time (approximate)	7.9 min
Injection Volume (µl)	0.50-10 mcL (6500), 1-20 mcL (5000)
Flow Rate	1.0 mL/min
Pressure, kgf/cm2 (approximate)	~80
Approximate Retention Time (min.)	Zidebactam: 1.6
	Cefotaxime: 4.0

CONFIDENTIAL

Serial Number: c4d37b88-d6c5-4833-95e6-1964235b97b0 accessed by Bryan Sitzmann (SITZMANNB) on Tue, 17 Jan 2023 23:46:48 GMT.

7.1.1. Gradient Table

Time Program

Time	Module	Events	Parameter
1.00	Controller	Event	1
1.20	Pumps	Pump B Conc.	2
2.80	Pumps	Pump B Conc.	16
3.80	Pumps	Pump B Conc.	16
4.50	Pumps	Pump B Conc.	100
4.50	Controller	Event	2
5.80	Pumps	Pump B Conc.	100
6.00	Pumps	Pump B Conc.	2
7.00	System Controller	Stop	

7.1.2. Autosampler Settings

Temperature:	Set at 4°C
Rinse Type:	External
Rinse Mode:	Before and after aspiration
Rinse Dip Time:	0 sec
Rinse Method:	Rinse Pump Then Port
Rinse Time:	2 sec
Rinsing Speed:	35 µL/sec
Rinsing Volume:	500 µL

7.1.3. Mass Spectrometer System

Mass Spectrometer	Sciex 5000 or 6500
Source Type:	Turbo spray
Scan Type:	MRM (MRM)
Polarity:	Positive
Resolution Q1:	Unit
Resolution Q3:	Unit

CONFIDENTIAL

Serial Number: c4d37b88-d6c5-4833-95e6-1964235b97b0 accessed by Bryan Sitzmann (SITZMANNB) on Tue, 17 Jan 2023 23:46:48 GMT.

All the below parameters in this section may be adjusted to optimize the instrument conditions.

Zidebactam:

Q1 Mass (Da)	Q3 Mass (Da)	Dwell (msec)
392.028	312.000	250
DP	66.00	
CE	21.00	
CXP	16.00	

Cefotaxime (IS):

Q1 Mass (Da)	Q3 Mass (Da)	Dwell (msec)
456.200	324.000	250
DP	50.00	
CE	22.00	
CXP	10.00	

Parameter Table:

CAD:	12.00
CUR:	40.00
GS1:	50.00
GS2:	60.00
IS:	5500
TEM:	600
EP:	12

CONFIDENTIAL

Serial Number: c4d37b88-d6c5-4833-95e6-1964235b97b0 accessed by Bryan Sitzmann (SITZMANNB) on Tue, 17 Jan 2023 23:46:48 GMT.

8. REAGENTS, STANDARDS, AND SUPPLIES

Reagents from alternate supplier and equivalent grade can be substituted.

Table 2: Reagents, Standards, and Supplies

**Chemical / Grade**	**Manufacturer/Supplier**
Zidebactam	Performa
Cefotaxime Sodium	Sigma Aldrich
MES ([2-(N-Morpholino) ethane sulphonic acid hydrate]) buffer	Sigma
Acetonitrile, HPLC grade	Honeywell
1 N Sodium Hydroxide	Fisher Scientific
Formic Acid, 98+%	EMD Millipore
Deionized Water	Millipore
Ammonium Acetate ≥ 99.9%	Sigma
Methanol	Honeywell
Acetone	Honeywell
Animal tubes	Sarstedt D51588
Human Sodium Heparin Plasma	BioChemed/BioIVT
Advantage SiliGuard Coated 2 mL 96 well Polypropylene plate: Square Top, Round Bottom	Analytical Sales and Services, Catalog # 27P687GC
Purple, pre-slit, square well, soft silicone/PTFE Cap Mats	Analytical Sales & Services, Inc., Catalog# 965085
Advantage Siliguard Coated 1 mL 96 well Polypropylene plate; round top, round wells	Analytical Sales and Services, Catalog # 17P687GC
Pre-slit, round well, soft silicone/PTFE Cap Mats	Analytical Sales & Services, Inc., Catalog# 965075

9. SAFETY

Always observe safe laboratory practices. Consult the appropriate SDS before handling any chemical if you are unfamiliar with the proper safety precautions.

CONFIDENTIAL

Serial Number: c4d37b88-d6c5-4833-95e6-1964235b97b0 accessed by Bryan Sitzmann (SITZMANNB) on Tue, 17 Jan 2023 23:46:48 GMT.

10. PROCEDURE

10.1. Preparation of Analyte Stock Solutions and Dilutions

The quantities of analyte solution prepared may be scaled up or down proportionally as needed. Alternate dilution schemes can be used to achieve the appropriate final concentrations of standards, QCs, working internal standard solution. For assignment of expiration dates to analyte stocks and their dilutions, refer to the latest version of the validation report.

10.1.1. Zidebactam Primary Solution, 2.00 mg/mL

Check drying conditions prior to use. Weigh approximately 20 mg of Zidebactam (correcting for purity and correction factor if necessary) in a polypropylene container and add the corresponding volume of deionized water, approximately 10.0 mL. Mix well and sonicate if necessary. Store solution at-20°C in a polypropylene container.

Primaries should be prepared on wet ice, and each prepared level should be aliquoted immediately and aliquots should be flash frozen in a dry ice/acetone bath for ~1 minute and stored in the freezer immediately after flash freezing.

10.1.2. Internal Standard Primary Solution, 5 mg/mL Cefotaxime Sodium

Check drying conditions prior to use. Weigh 50 mg salt of Cefotaxime Sodium. Add the calculated volume of deionized water (don't need to correct for conversion factor and purity), approximately 10 mL. Mix well and sonicate if necessary. Store at or below -20 °C in a glass container.

10.1.3. Internal Standard Working Solution, 50.0 mcg/mL Cefotaxime Sodium

Add 500 µL of 5 mg/mL Cefotaxime Sodium primary solution to a 50 mL volumetric flask. Dilute to volume with deionized water. Mix well and store at -20 °C in glass.

10.2. Preparation of Other Reagents

Prepared quantities of all reagents may be scaled up or down as necessary. Volumes provided are examples. For assignment of expiration dates, refer to SOP-00094.

10.2.1. Mobile Phase A: 10 mM Ammonium Acetate in Deionized Water

Weigh out ~3.08 g of Ammonium Acetate and transfer to a 4-L volumetric flask. Dilute to volume with deionized water. Mix well and store at room temperature.

CONFIDENTIAL

Serial Number: c4d37b88-d6c5-4833-95e6-1964235b97b0 accessed by Bryan Sitzmann (SITZMANNB) on Tue, 17 Jan 2023 23:46:48 GMT.

10.2.2. Mobile Phase B: 95/5 Acetonitrile / 10mM Ammonium Acetate

Combine 3800 mL of acetonitrile with 200 mL of 10 mM Ammonium Acetate in a 4-L glass bottle. Mix well and store at room temperature.

10.2.3. Rinse: 90/10 Acetone/ Deionized Water

Combine 900 mL of acetone and 100 mL of deionized water in a 1-L Container. Mix well and store at room temperature.

10.2.4. 1 N Sodium Hydroxide (NaOH) in Deionized Water

Weight ~ 4.00 g of sodium hydroxide (F.W. 40.00) and gradually transfer it to a 100-mL volumetric flask containing ~50 mL of deionized water. Mix to dissolve and wait until it reaches room temperature. Dilute to volume with deionized water and mix well. Store in a polypropylene container at room temperature.

10.2.5. MES ([2-(N-Morpholino) ethane sulphonic acid hydrate]) Buffer pH 6.4, ~0.1M

Weigh and transfer accurately about 2000 mg of MES hydrate to 100 mL volumetric flask. Dissolve and adjust the pH to 6.4 with 1N NaOH, make up the volume with deionized water. Mix well and store refrigerated.

10.2.6. Reconstitution Solvent: 2/98 Acetonitrile/Deionized Water

Combine 40 mL of acetonitrile and 1960 mL of deionized water in a 2-L container. Mix well. Store at room temperature for up to 1 month.

10.3. Preparation of Standards and QC Samples in Ice Water Bath

10.3.1. Treated Human Sodium Heparin Plasma (Human Sodium Heparin Plasma: MES Buffer 1:1)

Add 100 mL of **chilled** human sodium heparin plasma to 100 mL of **chilled** MES Buffer pH 6.4 in an appropriate polypropylene container. Mix well and store at -20°C.

It is recommended to freshly prepare the treated human plasma prior to usage.

CONFIDENTIAL

Serial Number: c4d37b88-d6c5-4833-95e6-1964235b97b0 accessed by Bryan Sitzmann (SITZMANNB) on Tue, 17 Jan 2023 23:46:48 GMT.

10.3.2. Preparation of Standard and QC Spiking Solutions

Starting from the 2.00 mg/mL primary solution of Zidebactam in deionized water, prepare the STD/QC Spiking Solutions with deionized water as the diluent on ice water bath. The following concentrations for the standards spiking solution;. 500, 400, 200, 100, 50.0, 10.0, 1.00, and 0.500 mcg/mL. For QC spiking solutions: 375, 250, 1.5, and 0.500 mcg/mL for the LLOQ QC. Store solutions in polypropylene at -20°C.

Spiking solutions should be prepared on wet ice, and each prepared level should be aliquoted immediately and aliquots should be flash frozen in a dry ice/acetone bath for ~1 minute and stored in the freezer immediately after flash freezing.

10.3.3. Preparation of Standards and QC Samples in Sodium Heparin Plasma Matrix

Use treated human plasma that has been diluted 1:1 with MES buffer and screened for the lack of interference at the retention times of ertapenem and cefotaxime (IS). Dilute each spiking solution by a factor of 20 into treated human plasma on crushed ice and mix well. For example, combine 0.500 mL of spiking solution and 9.5 mL of treated human plasma. Since unknown samples are treated with the MES buffer after collection at a 1:1 ratio, the calibration standard and QC concentrations are based on the plasma volume only. Unknown samples do not need to be adjusted for the dilution with MES buffer. The prepared concentrations in plasma only, not accounting for MES buffer, will be as follows: For standards: 50.0, 40.0, 20.0, 10.0, 5.00, 1.00, 0.100 and 0.0500 mcg/mL (the actual standard drug concentrations in the MES/plasma mixture are 25.0, 20.0, 10.0, 5.00, 2.50, 0.500, 0.0500, and 0.0250 mcg/mL). For QCs: 37.5, 25.0, and 0.150 mcg/mL, with 0.0500 mcg/mL for the LLOQ QC when needed (the actual QC drug concentrations in the MES/plasma mixture are (18.8, 12.5, and 0.0750 mcg/mL, with 0.0250 mcg/mL for the LLOQ QC). Other concentrations may be prepared, provided they meet the requirements of SOP-00035 and provided they remain within the range of 0.0500 to 50.0 mcg/mL in the plasma, prior to MES treatment. Store solutions in polypropylene at -70°C.

Calibration standards and QCs should be prepared on wet ice, and each prepared level should be aliquoted immediately and aliquots should be flash frozen in a dry ice/acetone bath for ~1 minute and stored in the freezer immediately after flash freezing.

CONFIDENTIAL

Serial Number: c4d37b88-d6c5-4833-95e6-1964235b97b0 accessed by Bryan Sitzmann (SITZMANNB) on Tue, 17 Jan 2023 23:46:48 GMT.

10.4. Extraction Procedure

1. Thaw MES treated human plasma samples on ice water bath. Once samples are thawed, either vortex the batch as a whole for ~2 minutes followed by vortexing each for 10 seconds, or vortex each sample for ~ 20 seconds each.
2. Pipet 100 mcL of sample or blank to corresponding wells in a 2 mL Siliguard 96-well plate. Flash freeze the whole run in a dry-ice/acetone bath as soon as pipetting is completed prior to returning it to the freezer.
3. Add 20 mcL of WIS (50.0 mcg/mL Cefotaxime Sodium in deionized water) to all applicable wells. For **Control Blanks**, add 20 mcL of deionized water instead.
4. Seal the plate with adhesive pad (or equivalent) and vortex at 1300 rpm for 3 minutes using a plate shaker.
5. Add 400 mcL of acetonitrile to all wells. Seal the plate with an adhesive pad (or equivalent) and mix the samples by using a plate shaker. Start at 800 rpm, then slowly increase speed to 1200 rpm. Maintain 1200 rpm for at least 5 min.
6. Keep the plate at room temperature for 10 minutes.
7. Centrifuge the plate for 10 minutes at the setting of 4000 rpm at room temperature.
8. Transfer 300 mcL of supernatant to a new 1-mL 96-well SiliGuard plate using an automated liquid handler or a multichannel pipette (or equivalent).
9. Completely dry down supernatant in the plate using single unit (or equivalent) Biotage SPE Dry upper flow of ~60 psi at 37°C, and a lower flow of ~60 psi at 37°C.
10. Reconstitute with 100 mcL reconstitution solvent (2/98 acetonitrile/deionized water).
11. Seal the plate with a purple lid. Store samples in refrigerator until ready for LC-MS injection.

10.5. Dilutions

2x = 50 mcL sample + 50 mcL blank matrix (treated)

10x = 20 mcL sample + 180 mcL of blank matrix (treated). Mix well. Pipet 100 mcL as sample.

CONFIDENTIAL

Serial Number: c4d37b88-d6c5-4833-95e6-1964235b97b0 accessed by Bryan Sitzmann (SITZMANNB) on Tue, 17 Jan 2023 23:46:48 GMT.

10.6. Calculations

Linear regression is performed with a $1/x^2$ weighting factor.

Equation: $y = a + bx$

Where y = analyte/internal standard area ratio

x = concentration (mcg/mL)

b = slope

a = intercept

11. REFERENCE CHROMATOGRAMS

Figure 1: Blank without IS

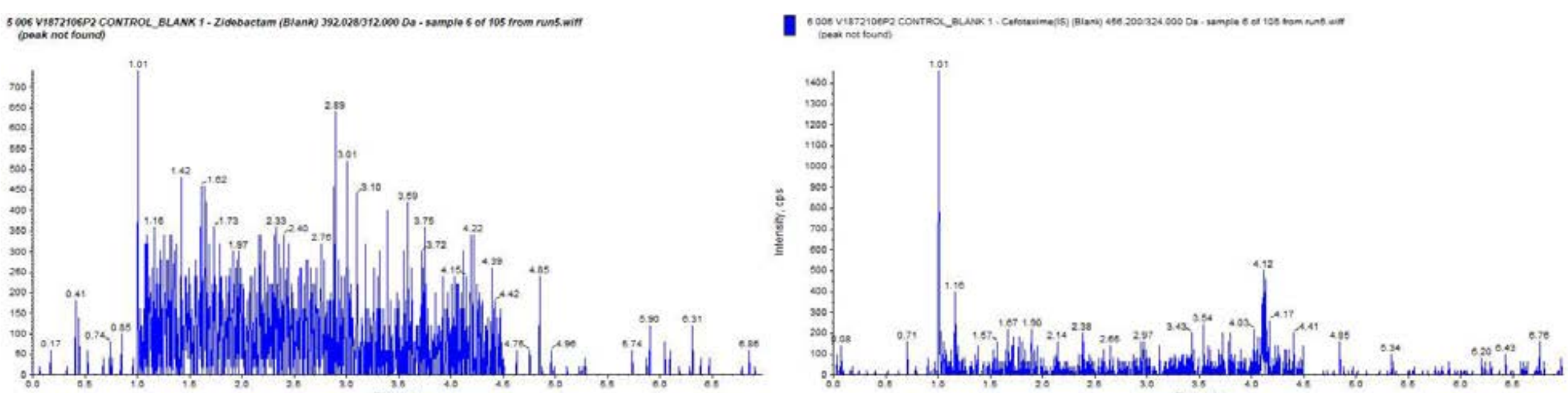

Figure 2: Blank with IS

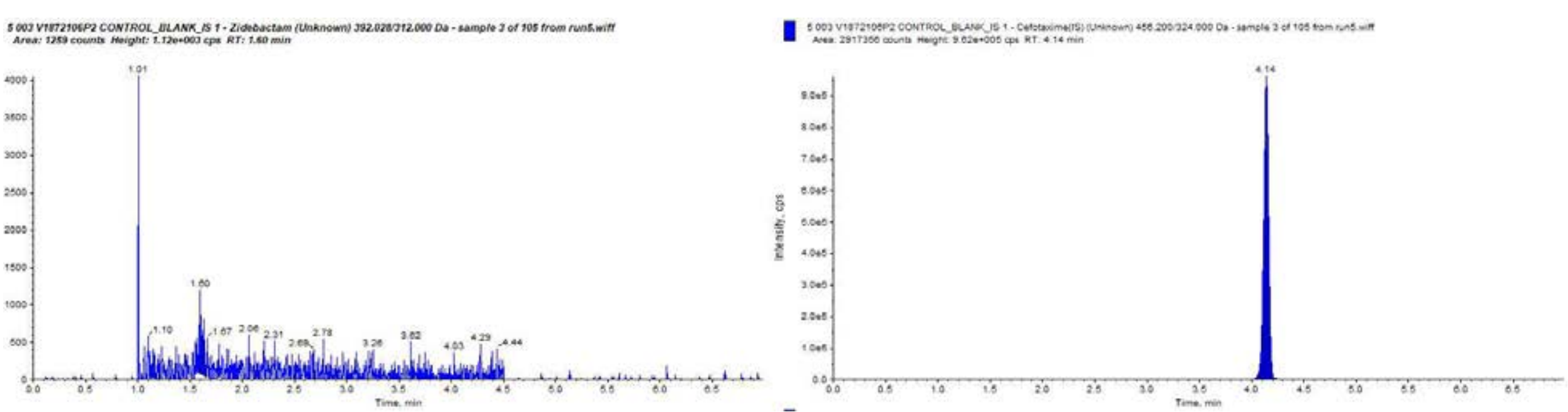

CONFIDENTIAL

Serial Number: c4d37b88-d6c5-4833-95e6-1964235b97b0 accessed by Bryan Sitzmann (SITZMANNB) on Tue, 17 Jan 2023 23:46:48 GMT.

Figure 3: LLOQ

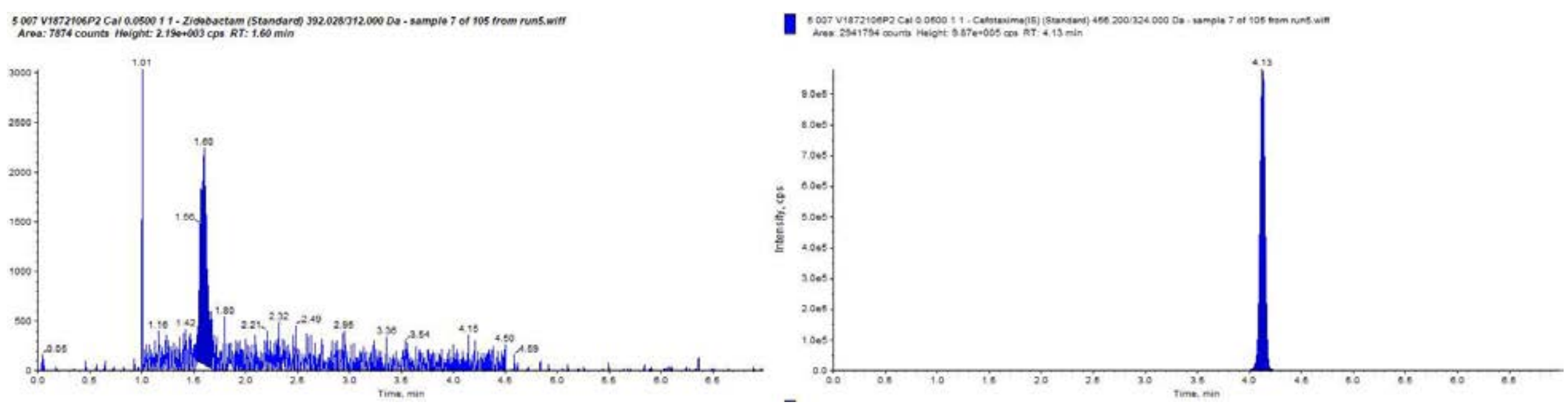

12. REVISION HISTORY

Table 3: Revision History

Revision	Effective Date	Section	Revision Summary
01	See Header	Throughout	Minor formatting edits to emphasize key details. **Note: All changes in this TP are being made for clarification. They reflect actual process used during validation and no procedure updates are made. No additional validation is needed.**
		5	(Special Handling of Biological Samples) Corrected section to say that samples do not need dilution factor applied.<br>Added instructions to prepare on wet ice and flash freeze for storage.
		10.1.1	(Zidebactam Primary Solution, 2.00 mg/mL) Added instructions to prepare on wet ice and flash freeze for storage
		10.3.1	(Treated Human Sodium Heparin Plasma) Added recommendation to prepare treated human plasma ultrafiltrate fresh.
		10.3.2	(Preparation of Standard and QC Spiking Solutions) Added instructions to prepare on wet ice and flash freeze for storage
		10.3.3	(Preparation of Standards and QC Samples in Sodium Heparin Plasma Matrix) Added instructions to prepare on wet ice and flash freeze for storage. Removed wording for storage at -20°C
		10.4	(Extraction Procedure) Added clarification to Step 2 to flash-freeze run after pipetting is complete.
00	23 Nov 2022	NA	New Procedure

CONFIDENTIAL

Serial Number: c4d37b88-d6c5-4833-95e6-1964235b97b0 accessed by Bryan Sitzmann (SITZMANNB) on Tue, 17 Jan 2023 23:46:48 GMT.

TP-80194 [01]: V1872106P2 - Zidebactam in Treated Human Sodium Heparin Plasma (100 mcL, 0.050 0 – 50.0 mcg/mL)

All blank fields are considered as data not available.

All Signature (Date and Time) shown in this report are in GMT.

**Type**	TP	**Status**	CURRENT	
**Effective Date**	13-Jan-2023	**Periodic Review Date**	30-May-2050	
**Owner**	PARMENTIERB	**Department Name**	Pharma LC-MS/MS	Pharma LC-MS/MS
**Description**	Full Title: LC-MS/MS Analysis of Zidebactam in Treated Human Sodium Heparin Plasma (100 mcL, 0.0500 – 50.0 mcg/mL; Prior to MES Treatment)			

Revision Notes (5)

Task Name	Action	Comment	Signature
Check In	Document Profile Preparation	Edited outside of the system.	NEVINSC<br>10-Jan-2023 15:16:41 [GMT]
Check Out	Document Finalization	More changes needed.	NEVINSC<br>10-Jan-2023 16:21:35 [GMT]
Check In	Document Finalization	Edited outside of the system.	NEVINSC<br>13-Jan-2023 20:41:24 [GMT]
Revise Document	Original Document Revision : 00	Revision requested.	NEVINSC<br>05-Jan-2023 21:05:19 [GMT]
Check Out	Document Profile Preparation	Revision requested.	NEVINSC<br>05-Jan-2023 21:05:40 [GMT]

Serial Number: c4d37b88-d6c5-4833-95e6-1964235b97b0 accessed by Bryan Sitzmann (SITZMANNB) on Tue, 17 Jan 2023 23:46:48 GMT.

Approval Information

Note to Approver

Reviewer: Bryan Parmentier, MLT (ASCP)
Management: Marsha Luna, B.S.
Author & PI: Anika Pippin, B.S.

Approval Decision	Comment	Signature
Approve		PARMENTIERB<br>13-Jan-2023 21:29:47 [GMT]
Approve		LUNAM<br>13-Jan-2023 23:35:08 [GMT]
Approve		PIPPINA<br>14-Jan-2023 0:24:47 [GMT]

Serial Number: c4d37b88-d6c5-4833-95e6-1964235b97b0 accessed by Bryan Sitzmann (SITZMANNB) on Tue, 17 Jan 2023 23:46:48 GMT.

3050 Spruce Street, Saint Louis, MO 63103, USA
Website: www.sigma-aldrich.com
Email USA: techserv@sial.com
Outside USA: eurtechserv@sial.com

Certificate of Analysis

Product Name : **Cefotaxime sodium salt suitable for plant cell culture, BioReagent, powder**
Product Number : **C7039-500MG**
Batch Number : **0000146942**
Source Batch : 157206
CAS Number : 64485-93-4
Storage Temperature : Cooler/Refrigerated
Molecular Formula : $C_{16}H_{16}N_5NaO_7S_2$
Formula Weight : 477.45
Recommended Retest Date : Sep 2023
Quality Release Date : 02 Jan 2022

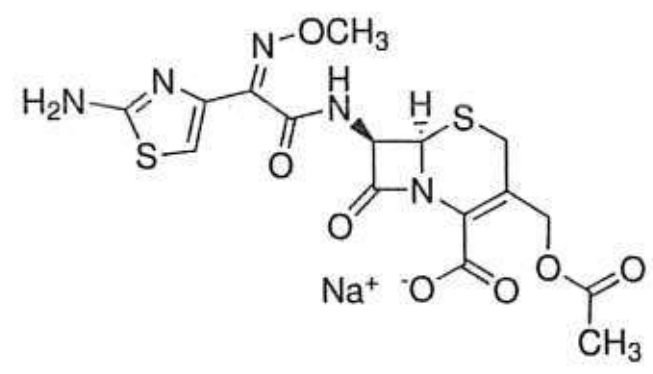

Test	Specification	Result
Appearance (Form)	Powder or Crystals	Powder
Appearance (Color)<br>Off-white to pale yellow	Conforms	Conforms
Solubility<br>10% in water	Clear	Clear
Infrared Spectrum	Conforms to Structure	Conforms
Total Impurity	≤ 3.0 %	1.7 %
Specific Rotation<br>[a]20/D	58 - 64 °	61 °
Assay (Dry Basis)	916 - 964 µg/mg	955 µg/mg
Loss on Drying	≤ 3.0 %	2.4 %
Plant Cell Culture Test	Pass	Pass

Anna Bendersky, Quality Control Manager
Quallity Control
Israel
IL

Sigma-Aldrich warrants, that at the time of the quality release or subsequent retest date this product conformed to the information contained in this publication. The current Specification sheet may be available at Sigma-Aldrich.com. For further inquiries, please contact Technical Service. Purchase must determine the suitability of the product for its particular use. See reverse side of website or packing slip for additional terms and conditions of sale

Version Number: 01 Doc: 1074711

The branding on the header and/or footer of this document may temporarily not visually match the product purchased as we transition our branding. However, all of the information in the document regarding the product remains unchanged and matches the product ordered.
For further information please contact mlsbranding@sial.com

Certificate of Analysis

Inhibitors • Screening Libraries • Proteins

Ertapenem sodium

**Cat. No.:**	HY-13625
**CAS No.:**	153773-82-1
**Batch No.:**	155576
**Chemical Name:**	1-Azabicyclo[3.2.0]hept-2-ene-2-carboxylic acid, 3-[[(3S,5S)-5-[[(3-carboxyphenyl)amino]carbonyl]-3-pyrrolidinyl]thio]-6-[(1R)-1-hydroxyethyl]-4-methyl-7-oxo-, sodium salt (1:1), (4R,5S,6S)-

PHYSICAL AND CHEMICAL PROPERTIES

**Molecular Formula:**	$C_{22}H_{24}N_3NaO_7S$
**Molecular Weight:**	497.50
**Storage:**	4°C, sealed storage, away from moisture<br>* The compound is unstable in solutions, freshly prepared is recommended.

Chemical Structure:

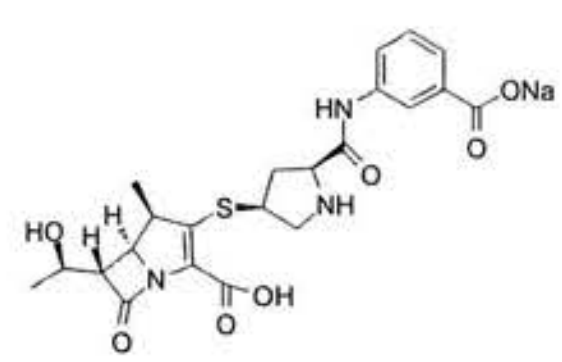

ANALYTICAL DATA

**Appearance:**	White to light yellow (Solid)
**¹H NMR Spectrum:**	Consistent with structure
**LCMS:**	Consistent with structure
**Purity (HPLC):**	98.94%
**Test Date:**	2022/06/21
**Retest Date:**	2024/06/20
**Conclusion:**	The product has been tested and complies with the given specifications.

Name: Lingli Ma
Designation: QA Manager
Date: 7/6/2022

Caution: Product has not been fully validated for medical applications. For research use only.

Tel: 609-228-6898 Fax: 609-228-5909 E-mail: tech@MedChemExpress.com
Address: 1 Deer Park Dr, Suite Q, Monmouth Junction, NJ 08852, USA

Chromatogram and Results

Injection Details

Injection Name:	**BIZ2022-617-WLQ3**	*Run Time (min):*	**16.00**
Vial Number:	**GB1**	*Injection Volume:*	**2.00**
Injection Type:	**Unknown**	*Channel:*	**EXT308NM**
User:	**HuXinQuan**	*Wavelength:*	**n.a.**
CAS NO.:	**153773-82-1 A-RP-760 0.8MG/ML**	*Bandwidth:*	**n.a.**
Instrument Method:	**10-90A**	*Dilution Factor:*	**1.0000**
Processing Method:	**3D Quantitative**	*Sample Weight:*	**1.0000**
Injection Date/Time:	**21/Jun/22 14:33**		

chm.ra **chrom://hy583/HPLC-63/202206/20220621.seq/469.smp/EXT308NM.channel/EXT308NM.chm**

Chromatogram

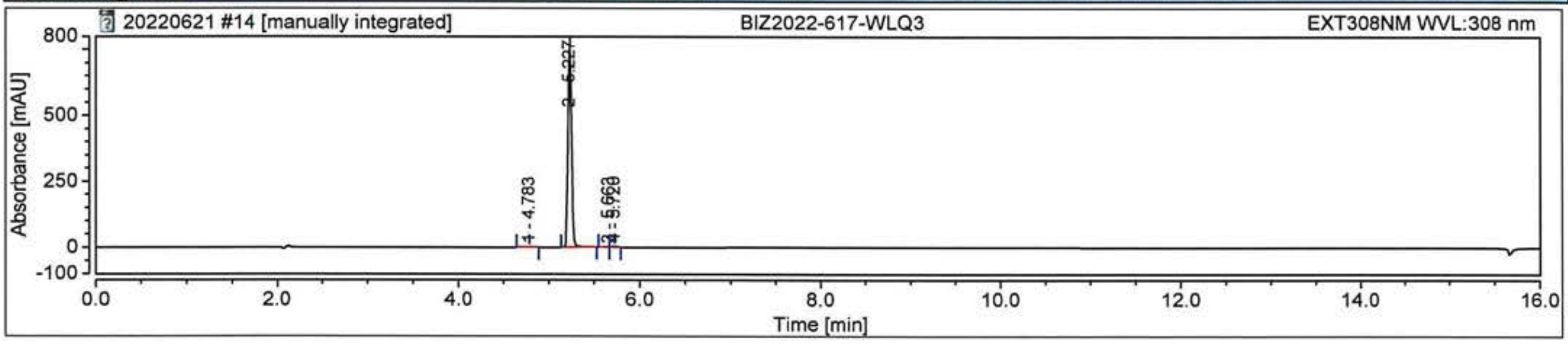

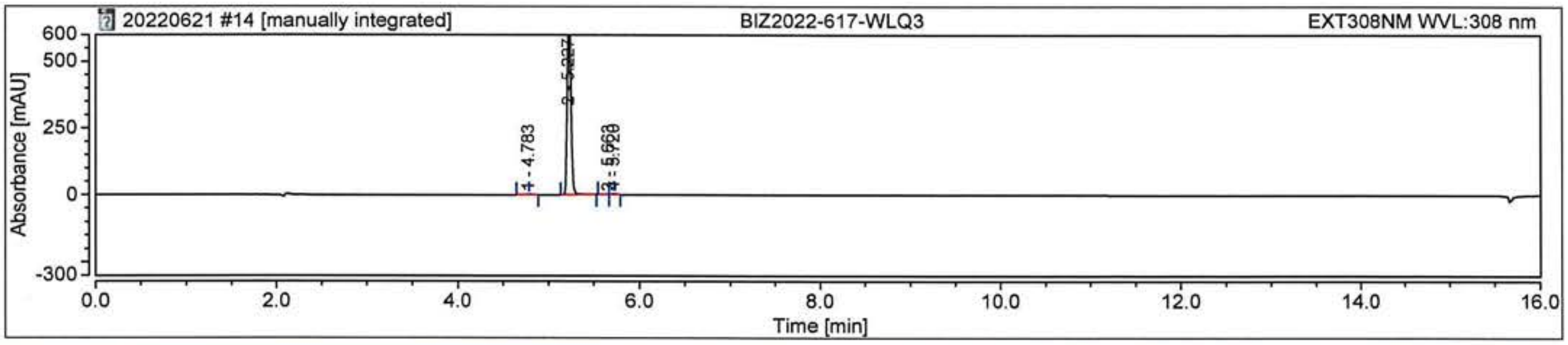

Integration Results

No.	Peak Name	Retention Time min	Area mAU*min	Height mAU	Match	Resolution (SM)	Rel.Area %
1		4.783	0.073	0.951	997	4.29	0.200
2		5.227	36.337	758.223	1000	4.39	98.941
3		5.663	0.090	1.177	990	0.54	0.246
4		5.720	0.225	3.358	996	n.a.	0.614
**Total:**			**36.726**	**763.709**	**3983.01**	**9.22**	

CAS NO.: 153773-82-1 ,D2O

Chemical Formula: $C_{22}H_{24}N_3NaO_7S$
Exact Mass: 497.12

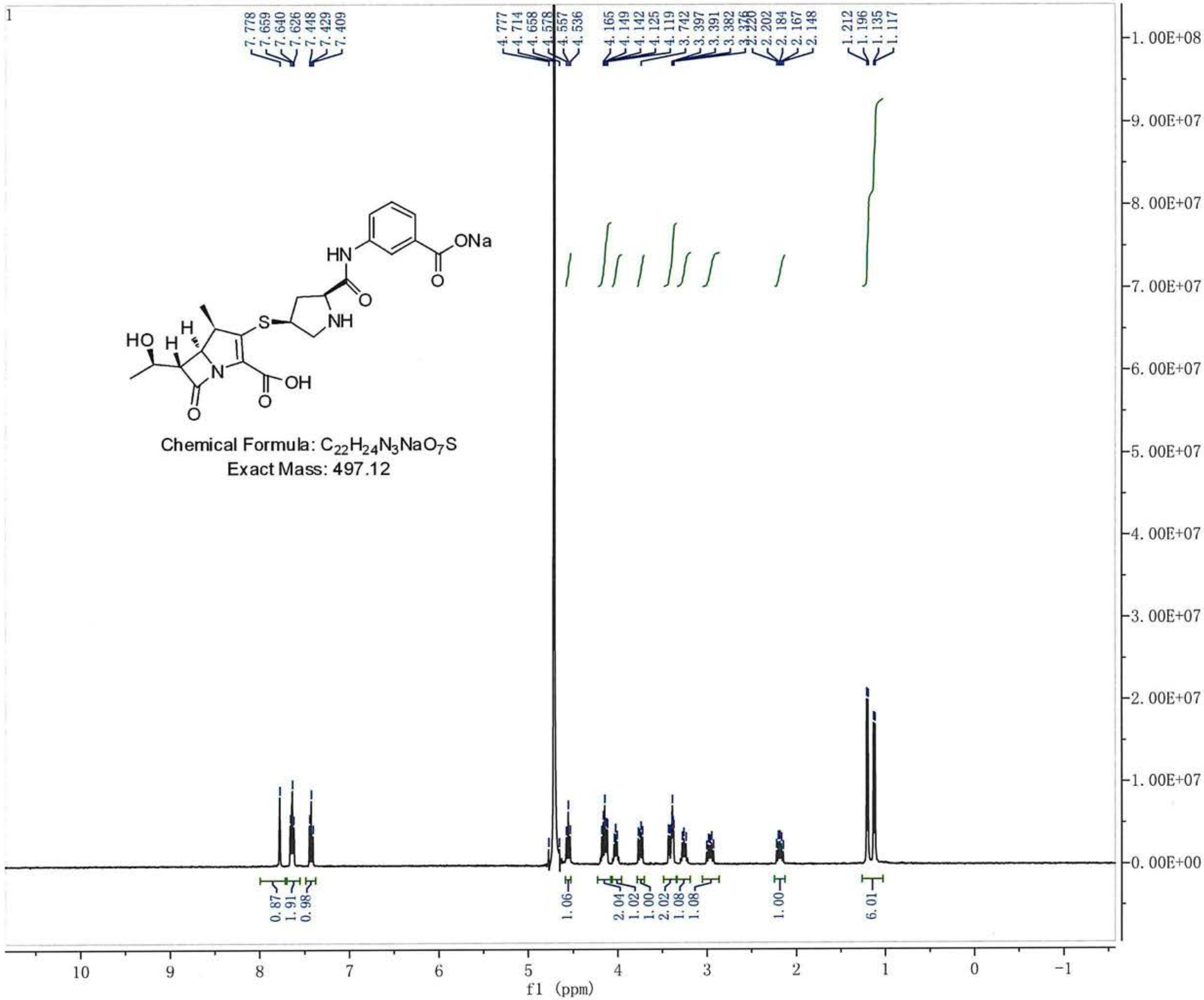

	Parameter	Value
1	Title	1
2	Comment	
3	Spectrometer	magnet
4	Solvent	D20
5	Temperature	298.0
6	Pulse Sequence	s1pul30
7	Number of Scans	64
8	Receiver Gain	64
9	Relaxation Delay	1.0000
10	Pulse Width	9.8000
11	Acquisition Time	3.9999
12	Acquisition Date	2022-06-17T16:21:42
13	Modification Date	2022-06-17T16:29:29
14	Spectrometer Frequency	400.00
15	Spectral Width	8108.0
16	Lowest Frequency	-1654.0
17	Nucleus	1H
18	Acquired Size	32431
19	Spectral Size	65536

MCE® MedChemExpress

Certificate of Analysis

Inhibitors • Screening Libraries • Proteins

Ertapenem sodium

Cat. No.:	HY-13625
CAS No.:	153773-82-1
Batch No.:	180017
Chemical Name:	1-Azabicyclo[3.2.0]hept-2-ene-2-carboxylic acid, 3-[[(3S,5S)-5-[[(3-carboxyphenyl)amino]carbonyl]-3-pyrrolidinyl]thio]-6-[(1R)-1-hydroxyethyl]-4-methyl-7-oxo-, sodium salt (1:1), (4R,5S,6S)-

PHYSICAL AND CHEMICAL PROPERTIES

Molecular Formula:	$C_{22}H_{24}N_3NaO_7S$
Molecular Weight:	**497.50**
Storage:	4°C, sealed storage, away from moisture<br>* In solvent : -80°C, 6 months; -20°C, 1 month (sealed storage, away from moisture)

Chemical Structure:

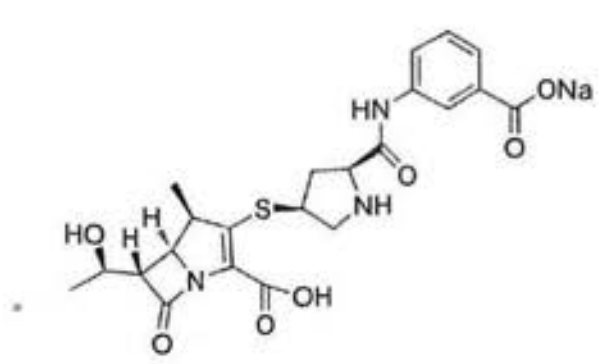

ANALYTICAL DATA

Appearance:	White to light yellow (Solid)
^{1}H NMR Spectrum:	Consistent with structure
LCMS:	Consistent with structure
Purity (LCMS):	**98.86%**
Test date:	2022/08/12
Retest date:	2024/08/11
Conclusion:	The product has been tested and complies with the given specifications.

Name: Lingli Ma

Designation: QA Manager

Date: 6/27/2022

Caution: Product has not been fully validated for medical applications. For research use only.

Tel: 609-228-6898 Fax: 609-228-5909 E-mail: tech@MedChemExpress.com

Address: 1 Deer Park Dr, Suite Q, Monmouth Junction, NJ 08852, USA

SAMPLE INFORMATION

CAS NO.:	153773-82-1	Acquired By:	LY
Sample infromation:		Date Acquired:	8/12/2022 2:04:10 PM CST
Vial:	2:A,3	Acq. Method Set:	1_POS_2MIN
Injection #:	1	Date Processed:	8/12/2022 2:36:50 PM CST,
Injection Volume:	0.30 ul	Processing Method:	PDA_01, MS_scan
Run Time:	2.8 Minutes	Channel Name:	PDA Ch1 214nm@4.8nm, MS TIC,
Sample Set Name:	20220812	Column Name:	A-RP-985

Auto-Scaled Chromatogram

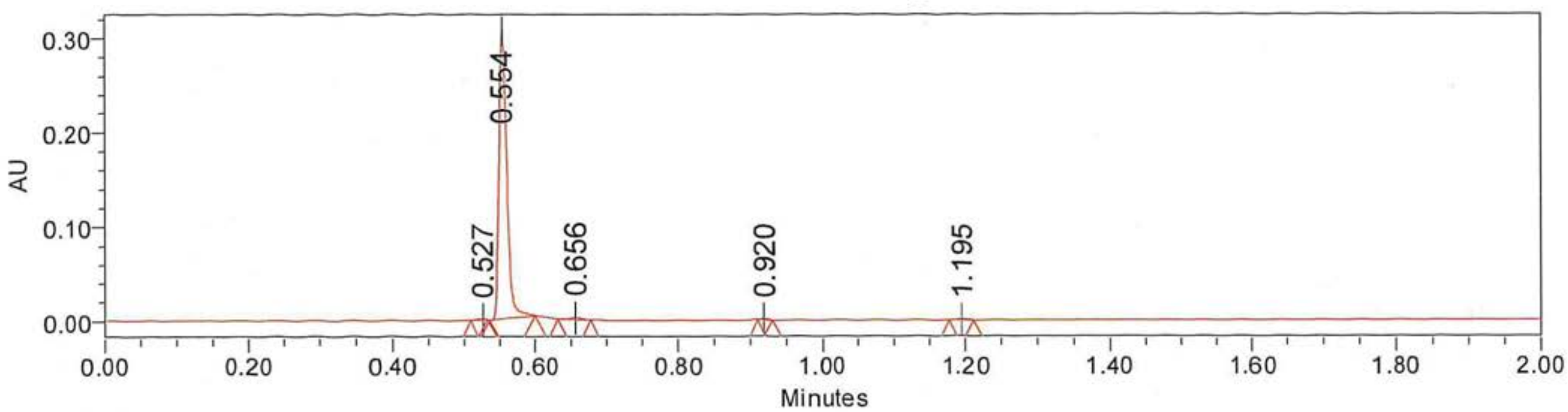

Processed Channel Descr. PDA Ch2 254nm@4.8nm

Auto-Scaled Chromatogram

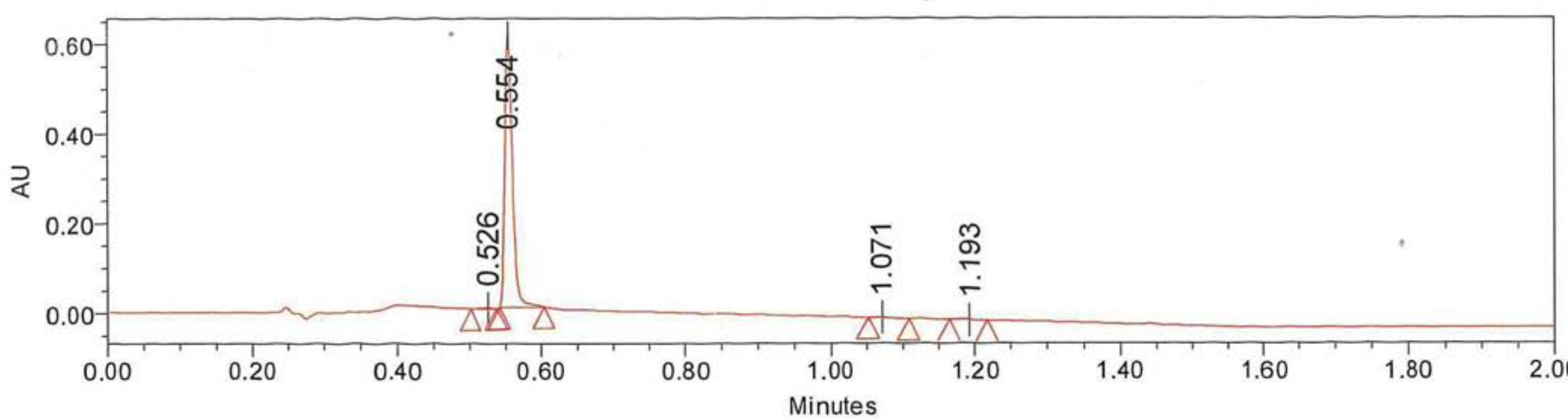

Processed Channel Descr. PDA Ch1 214nm@4.8nm

Auto-Scaled Chromatogram

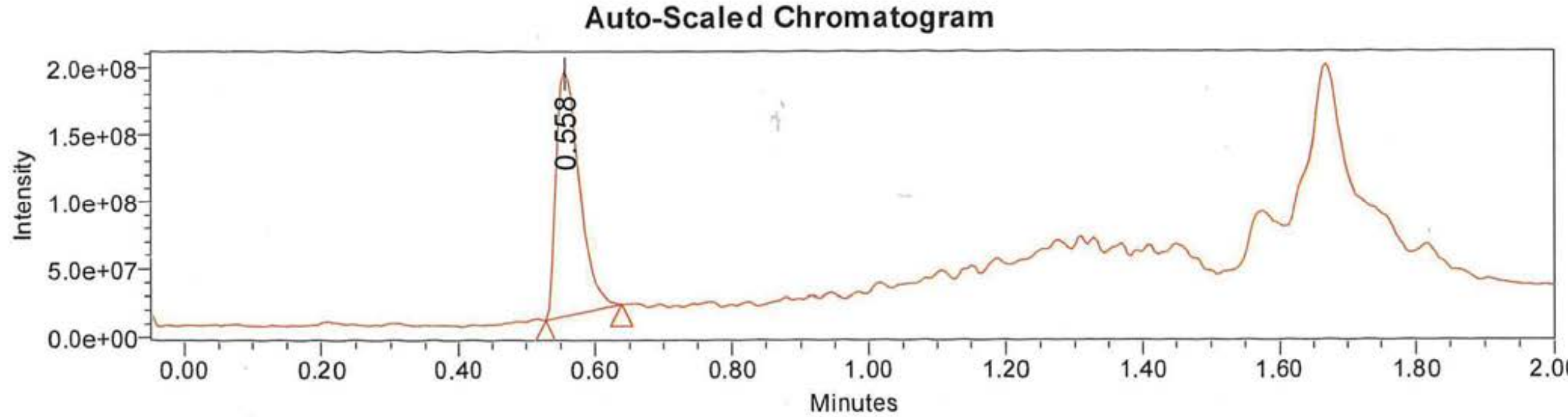

Processed Channel Descr. ACQ-SQD2 1: MS Scan MS TIC, Smoothed by 9 point Savitzky-Golay Filter., Time offset by -0.053 mins. (1: 150.00-1000.00 ES+, Centroid, CV=30)

Peak Results
Channel Name: PDA Ch2 254nm@4.8nm

	RT	Width (sec)	Area	Height	% Area	Base Peak (Combined) (m/z)	Channel Name
1	0.527	1.400	477	640	0.20	153.83	PDA Ch2 254nm@4.8nm
2	0.554	3.800	237134	308413	98.19	151.96	PDA Ch2 254nm@4.8nm
3	0.656	2.650	2446	2436	1.01	151.06	PDA Ch2 254nm@4.8nm
4	0.920	1.200	632	1080	0.26	152.07	PDA Ch2 254nm@4.8nm
5	1.195	2.050	823	941	0.34	152.07	PDA Ch2 254nm@4.8nm

Peak Results
Channel Name: PDA Ch1 214nm@4.8nm

	RT	Width (sec)	Area	Height	% Area	Base Peak (Combined) (m/z)	Channel Name
1	0.526	2.150	1796	2101	0.38	153.84	PDA Ch1 214nm@4.8nm
2	0.554	3.700	471358	613577	98.86	151.97	PDA Ch1 214nm@4.8nm
3	1.071	3.350	1629	1281	0.34	151.03	PDA Ch1 214nm@4.8nm
4	1.193	3.150	1997	1243	0.42	152.07	PDA Ch1 214nm@4.8nm

Peak Results
Channel Name: MS TIC

	RT	Width (sec)	Area	Height	% Area	Base Peak (Combined) (m/z)	Channel Name
1	0.558	6.673	431532509	182380390	100.00	476.14	MS TIC

Match Plot

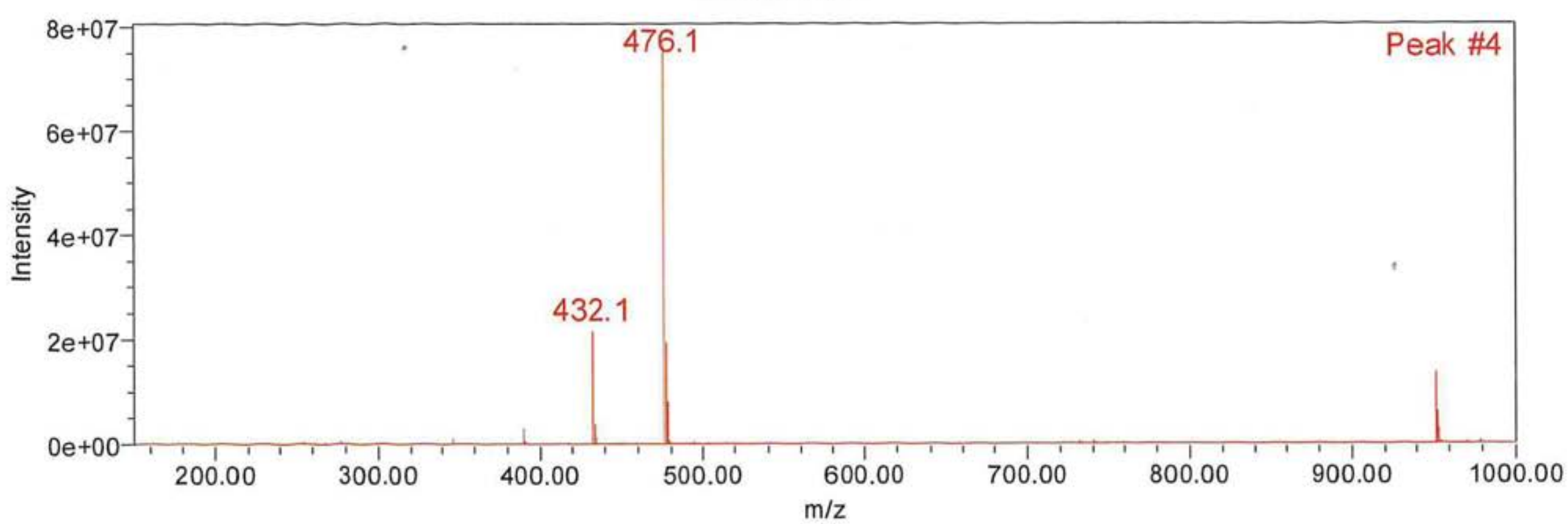

Retention Time 0.558 Channel Name MS TIC

CAS NO.: 153773-82-1, D2O

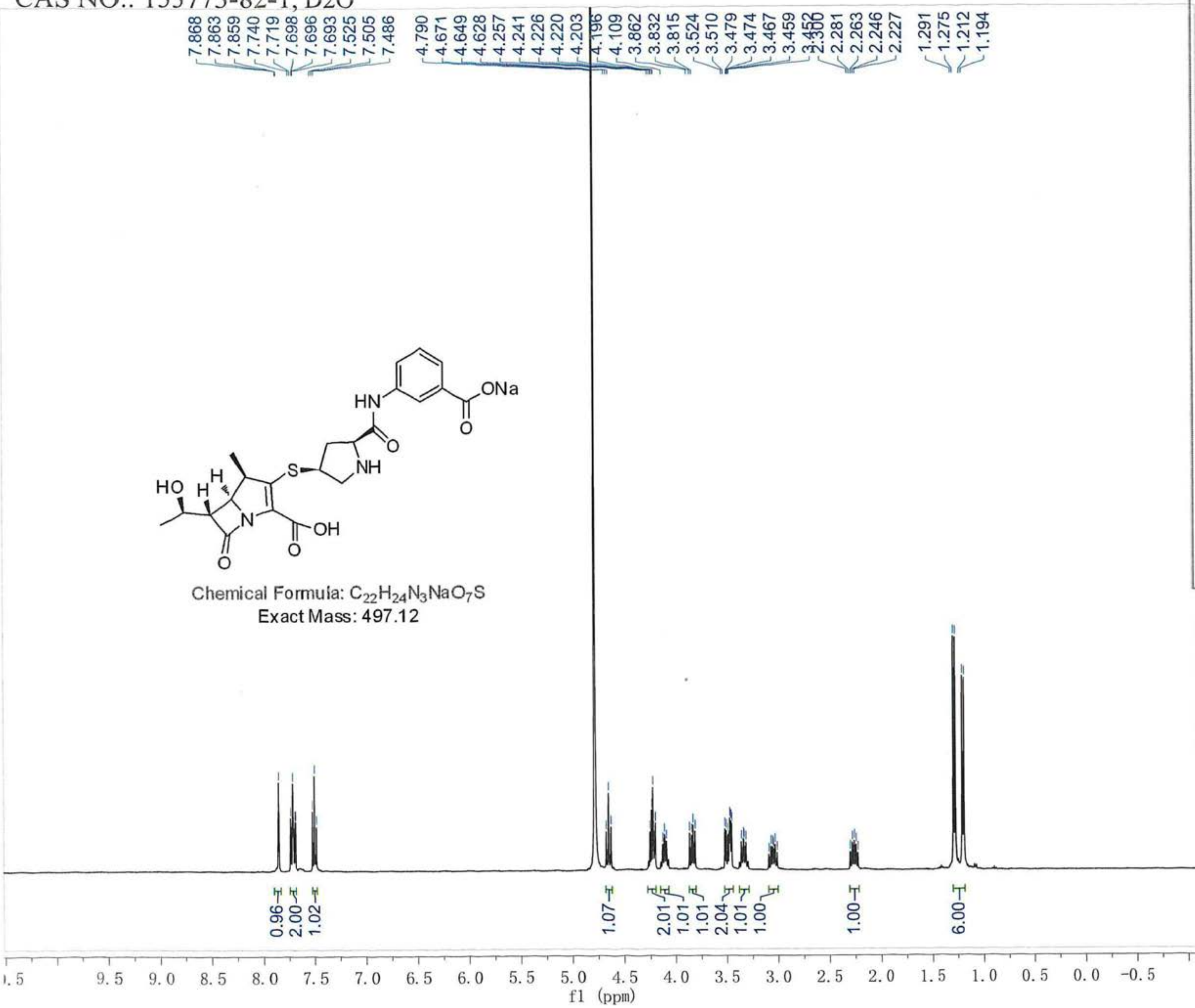

	Parameter	Value
1	Title	
2	Origin	Bruker BioSpin GmbH
3	Owner	nmrsu
4	Spectrometer	Avance
5	Author	
6	Solvent	D2O
7	Temperature	298.2
8	Pulse Sequence	zg30
9	Experiment	1D
10	Number of Scans	8
11	Receiver Gain	101
12	Relaxation Delay	1.0000
13	Pulse Width	8.0000
14	Acquisition Time	3.9977
15	Acquisition Date	2022-08-10T13:49:06
16	Modification Date	2022-08-10T13:53:00
17	Spectrometer Frequency	400.13
18	Nucleus	1H

WOCKHARDT LIFE WINS

ANALYTICAL RESEARCH

CERTIFICATE OF ANALYSIS FOR REFERENCE STANDARD

**Name**	:	**Zidebactam (WCK 5107)**			
Molecular Formula	:	$C_{13}H_{21}N_5O_7S.2H_2O$	Batch Number	:	WK1/WP-0060/016
Molecular Weight	:	427.45	Laboratory Note Book Reference	:	WA-2004-038
Date of Analysis	:	30/03/2022	Month of Manufacturing	:	May' 2019
Valid Up To	:	29/03/2023	Reference Standard Number	:	RS/2022-013
Effective Date	:	13/04/2022	Page Number	:	Page 1 of 4

Sr. No.	Tests	Specifications	Results
1	Description	White to pale yellow powder	White powder
2	Identification<br>(By IR, Mass & NMR Spectroscopy)	Spectral data should conform to the structure	Spectral data conforms the structure
3	Solubility	Freely soluble in water,<br>Insoluble in dichloromethane	Freely soluble in water, Insoluble in dichloromethane
4	Constituted solution<br>(10% w/v solution in WFI)<br>Completeness of solution<br><br>Clarity of solution	 <br>The solid dissolves completely, leaving no visible residue as un-dissolved matter<br>The constituted solution is not significantly less clear than an equal volume of the WFI contained in a similar vessel and examined similarly	 <br>Complies<br><br>Complies
5	pH of solution	Between 5.0 to 7.0	6.0
6	Absorbance of solution (1.25 % w/v in water at 420 nm)	Not more than 0.2 AU	0.01 AU
7	Water content (By KF Titration)	Not more than 12.0 % w/w	8.7 % w/w
8	Specific optical rotation (On anhydrous basis)	Between –28° to -40°	-36°
9	Heavy metals	Not more than 20 μg/g	Less than 20 μg/g
10	Palladium content (By ICP-OES)	Not more than 10 μg/g	BLQ (LOQ 2.5 μg/g)*

	Prepared by	Checked by	Approved by
Name :	Mr. Sitaram Ubale	Mr. Rajendra Chavan	Mr Subhash Chandra Shukla
Department :	AVL	AVL	CRDQ
Sign :	[signature]	[signature]	[signature]
Date :	12/04/2022	12/04/2022	13/04/2022

Format No. : AVL-045-F-02-00

Ranked # 1 in Healthcare Best Companies to Work for 2015 India

ANALYTICAL RESEARCH

CERTIFICATE OF ANALYSIS FOR REFERENCE STANDARD

Name	:	Zidebactam (WCK 5107)			
Molecular Formula	:	$C_{13}H_{21}N_5O_7S.2H_2O$	Batch Number	:	WK1/WP-0060/016
Molecular Weight	:	427.45	Laboratory Note Book Reference	:	WA-2004-038
Date of Analysis	:	30/03/2022	Month of Manufacturing	:	May' 2019
Valid Up To	:	29/03/2023	Reference Standard Number	:	RS/2022-013
Effective Date	:	23/04/2022	Page Number	:	Page 2 of 4

Sr. No.	Tests	Specifications	Results
11	Related substances (By HPLC) :		
	i. Impurity -1	Not more than 2.00 % w/w	0.28 % w/w
	ii. Impurity -2	Not more than 1.00 % w/w	Not detected
	iii. Impurity -3	Not more than 1.00 % w/w	0.10 % w/w
	iv. Impurity -4	Not more than 0.10 % w/w	Not detected
	v. Specified impurity at RRT 2.16-2.28 (MW 782)	Not more than 0.50 % w/w	0.03 % w/w
	vi. Any unspecified impurity	Not more than 0.15 % w/w	BDL
	vii. Total impurities	Not more than 4.00 % w/w	0.41 % w/w
12	Residual solvents (By GC) :		
	Group A		
	i. Isopropyl alcohol	Not more than 5000 µg/g	Not detected
	ii. Dichloromethane	Not more than 600 µg/g	Not detected
	iii. Methanol	Not more than 3000 µg/g	1762 µg/g
	iv. Ethyl acetate	Not more than 5000 µg/g	Not detected
	v. Cyclohexane	Not more than 3880 µg/g	Not detected
	vi. Acetone	Not more than 5000 µg/g	Not detected
	vii. Methyl tertiary butyl ether	Not more than 5000 µg/g	Not detected
	viii. Toluene	Not more than 890 µg/g	Not detected
	ix. Ethanol	Not more than 5000 µg/g	2990 µg/g
	Group B		
	i. Triethylamine	Not more than 250 µg/g	Not detected
	ii. Pyridine	Not more than 200 µg/g	Not detected

	Prepared by	Checked by	Approved by
Name :	Mr. Sitaram Ubale	Mr. Rajendra Chavan	Mr Subhash Chandra Shukla
Department :	AVL	AVL	CRDQ
Sign :	[signature]	[signature]	[signature]
Date :	12/04/2022	12/04/2022	13/04/2022

Format No. : AVL-045-F-02-00

Ranked # 1 in Healthcare
Best Companies to Work for 2015 India

ANALYTICAL RESEARCH

CERTIFICATE OF ANALYSIS FOR REFERENCE STANDARD

**Name**	:	**Zidebactam (WCK 5107)**			
Molecular Formula	:	$C_{13}H_{21}N_5O_7S.2H_2O$	Batch Number	:	WK1/WP-0060/016
Molecular Weight	:	427.45	Laboratory Note Book Reference	:	WA-2004-038
Date of Analysis	:	30/03/2022	Month of Manufacturing	:	May' 2019
Valid Up To	:	29/03/2023	Reference Standard Number	:	RS/2022-013
Effective Date	:	23/04/2022	Page Number	:	Page 3 of 4

Sr. No.	Tests	Specifications	Results
13	Sodium content (By ICP-OES)	Not more than 0.5 % w/w	BLQ (LOQ 0.25 % w/w)*
14	Sulfate content (By Ion chromatography)	Not more than 1.0 % w/w	BLQ (LOQ 0.626 % w/w)
15	Mono-methyl sulphate content (By Ion chromatography)	Not more than 0.15% w/w	Not detected
16	Polymorphism (By X-ray Diffraction)	The diffractogram should exhibit characteristic 2 theta values at 13.8, 15.6, 20.3 and 24.3 (± 0.2°)	The diffractogram exhibits characteristic 2 theta values at 13.8, 15.6, 20.3 & 24.3
17	Potency (on as is basis)	Not applicable	90.4 %

BLQ = Below limit of quantification
BDL = Below disregard limit. Disregard limit 0.05 %
LOQ = Limit of quantification

* LOQ Values are based on analytical method verification report (Report nos. MVR/VP17053 and MVR/VP17054) of Analytical Solutions (CRO).

Specification Reference Number: **ZDB-DSS-112-05**

Remark: Potency is calculated on the basis of Total impurities, Water content, Residual solvents, Sulphate, Mono-methyl sulphate, Sodium and Palladium content.

Storage Condition: Store at 2°C to 8°C temperature.

	Prepared by	Checked by	Approved by
Name :	Mr. Sitaram Ubale	Mr. Rajendra Chavan	Mr Subhash Chandra Shukla
Department :	AVL	AVL	CRDQ
Sign :	[signature]	[signature]	[signature]
Date :	12/04/2022	12/04/2022	13/04/2022

Format No. : AVL-045-F-02-00

Ranked # 1 In Healthcare
Great Place to Work
Best Companies to Work for 2015 India

ANALYTICAL RESEARCH

CERTIFICATE OF ANALYSIS FOR REFERENCE STANDARD

Name	:	**Zidebactam (WCK 5107)**			
Molecular Formula	:	$C_{13}H_{21}N_5O_7S.2H_2O$	Batch Number	:	WK1/WP-0060/016
Molecular Weight	:	427.45	Laboratory Note Book Reference	:	WA-2004-038
Date of Analysis	:	30/03/2022	Month of Manufacturing	:	May' 2019
Valid Up To	:	29/03/2023	Reference Standard Number	:	RS/2022-013
Effective Date	:	23/04/2022	Page Number	:	Page 4 of 4

Chemical Names:

i. **WCK 5107**: (2S,5R)-7-Oxo-6-sulphoxy-2-[N'-((R)-piperidin-3-carbonyl)-hydrazinocarbonyl]-1,6-diaza-bicyclo[3.2.1]octane dehydrate

ii. **Impurity-1**: (2S,5R)-5-sulphooxyamino-2-[N'-((R)-piperidin-3-carbonyl)-hydrazinocarbonyl]-piperidine

iii. **Impurity-2**: (2S,5R)-7-Oxo-6-hydroxy-2-[N'-((R)-piperidin-3-carbonyl)-hydrazinocarbonyl]-1,6-diaza-bicyclo[3.2.1]octane

iv. **Impurity-3**: (2S,5R)-7-Oxo-6-sulphooxy-2-[N'-((S)-piperidin-3-carbonyl)-hydrazinocarbonyl]-1,6-diaza-bicyclo[3.2.1]octane

v. **Impurity-4**: (2S,5R)-7-Oxo-6-sulphooxy-2-[N'-((R)-1-tert-butoxycarbonyl-piperidin-3-yl)-carbonyl)-hydrazinocarbonyl] -1,6-diaza-bicyclo[3.2.1]octane

	Prepared by	Checked by	Approved by
Name :	Mr. Sitaram Ubale	Mr. Rajendra Chavan	Mr Subhash Chandra Shukla
Department :	AVL	AVL	CRDQ
Sign :	[signature]	[signature]	[signature]
Date :	12/04/2022	12/04/2022	13/04/2022

Format No. : AVL-045-F-02-00

Ranked # 1 in Healthcare
GREAT PLACE TO WORK Best Companies to Work for 2015 India

Record of Decision – Interference Testing
Study 21-0013: Zidebactam

Memo Date: November 10, 2022

RE: Interference Testing of Clinically Authorized Medications for Study 21-0013 (Zidebactam)

For this clinical study KCAS is developing several analytical LC-MS/MS assays for the detection and quantitation of zidebactam in plasma and urine and falls under the Bioanalytical Method Validation, FDA Guidance for Industry (May 2018) which states:

> *Potential interfering substances in a biological matrix include endogenous matrix components such as metabolites, decomposition products – and from the actual study – concomitant medications and other xenobiotics. If a stabilizer or enzyme inhibitor is used during sample collection, the sponsor should evaluate the potential for interference on the quantitation of the analyte. Sponsors should make a scientific judgment about the need to assess these (and any other) potential interferences during method development.*

The clinical trial has stringent screening and exclusion criteria but allows all volunteers to use acetaminophen (paracetamol) for temporary pain and headaches and allows women to continue using commercially available birth control.

An industry paper quoted de Zwart. M Lausecker *et al*, Co-medication and interference testing in bioanalysis: a European Bioanalysis Forum recommendation *Bioanalysis* 8(19), 2065-2070 (2016) contains a decision flow chart (Figure 1) that based on whether co-medication is scheduled or unscheduled and potential interference based on physical properties. The Figure 1 chart indicates that:

1. These inferring substances may not be considered scheduled co-medication because definition within the paper of "drugs which dose, time and route of administration are defined in the clinical study protocol", however the CDC estimates that ~ 25% of women aged, age 18-49 years old use hormone containing contraceptive, so it is prudent to expect ~12.5% of clinical samples to contain one or more of the contraceptives listed below when adjusting for study enrollment of both genders.
2. Therefore, following the "Scheduled co-medication" decision tree the interference testing is not required because the significant differences in the molecular weights of the test analyte and the ConMeds (see table below).

Zidebactam MASS (MW) vs CONMEDS (source: www.ChemSpider.com)

DRUG/Analyte	Formula	Avg. Mass	Monoisotopic Mass
Zidebactam	C13H21N5O7S	391.400 Da	391.116180 Da
Ertapenem	C22H25N3O7S	475.55 Da	475.142327 Da
Urea	CH4N20	60.0553 Da	60.055 Da
Ethinylestradiol	C20H24O2	296.403 Da	296.177643 Da
Levonorgestrel	C21H28O2	312.446 Da	312.208923 Da
Etonogestrel	C22H28O2	324.457 Da	324.208923 Da
Norethindrone	C20H26O2	298.419 Da	298.193268 Da
Drospirenone	C24H30O3	366.493 Da	366.219482 Da

These items are controlled by the U.S. Government and authorized for export only to the country of ultimate destination for use by the ultimate consignee or end-user(s) herein identified. They may not be resold, transferred, or otherwise disposed of, to any other country or to any person other than the authorized ultimate consignee or end-user(s), either in their original form or after being incorporated into other items, without first obtaining approval from the U.S. Government or as otherwise authorized by U.S. law and regulations.

Paracetamol	C8H9NO2	151.163 Da	151.063324 Da

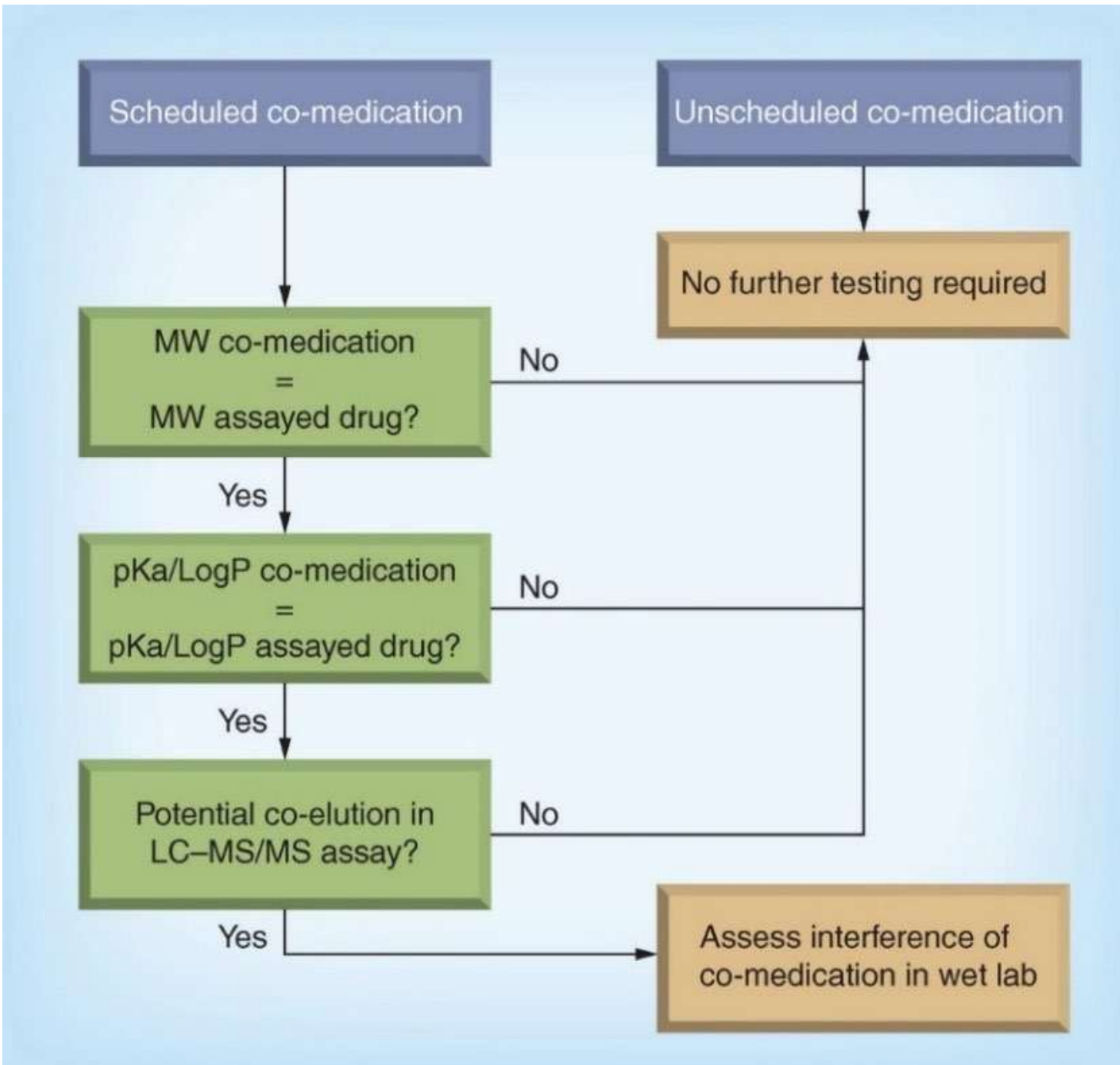

Figure 1. Recommendation for scheduled and unscheduled co-medication testing for LC–MS/MS-based assays.

Therefore, based on both sides of the decision tree in Figure 1, further interference testing other than the evaluation of the planned co-administered drug, ertapenem, is not required.

Decision issued by:

____

Matthew E. Hinz, M.S. Sr. Manager
DVC LLC, A GDIT Company

These items are controlled by the U.S. Government and authorized for export only to the country of ultimate destination for use by the ultimate consignee or end-user(s) herein identified. They may not be resold, transferred, or otherwise disposed of, to any other country or to any person other than the authorized ultimate consignee or end-user(s), either in their original form or after being incorporated into other items, without first obtaining approval from the U.S. Government or as otherwise authorized by U.S. law and regulations.
