# Supplementary material for: A Phase I, randomized, double-blind, placebo-controlled, dose escalation study to evaluate the safety, tolerability, and pharmacokinetics of intravenous ertapenem and zidebactam in combination (WCK 6777) in healthy adult subjects
Source: Antimicrob Agents Chemother. 2026 Sep 29;70(11):e00390-26. doi: 10.1128/aac.00390-26 (PMC13731237; doi:10.1128/aac.00390-26)
Supplement: Supplementary file 4 [file aac.00390-26-s0004.pdf]

1. TITLE: THE DETERMINATION OF FREE ZIDEBACTAM IN TREATED HUMAN PLASMA ULTRAFILTRATE, WITH SODIUM HEPARIN AS ANTICOAGULANT, BY LC-MS/MS

BIOANALYTICAL METHOD VALIDATION REPORT

Bioanalytical Project Number:	V1872106P4
Sponsor:	DynPort Vaccine Company, LLC<br>110 Thomas Johnson Drive<br>Suite 200<br>Frederick, MD 21702
Bioanalytical Laboratory:	KCAS Bio<br>10830 S Clay Blair Boulevard<br>Olathe, KS 66061
Issue Date:	February 2023
Amendment 1 Issue Date:	June 2023
Amendment 2 Issue Date:	Date of PI Signature

2. SYNOPSIS

This report presents the validation data for an LC-MS/MS method for the determination of free zidebactam in treated human plasma ultrafiltrate, with sodium heparin as anticoagulant to support the Phase 1 clinical trial studying the safety, tolerability, and pharmacokinetics of intravenous zidebactam in combination with ertapenem (WCK 6777) in healthy adult subjects. The assay was analyzed in the range of 0.0500 – 50.0 mcg/mL and is for use on treated human plasma ultrafiltrate samples obtained during clinical trials.

AMENDMENT OUTLINE FORM

1. Amendment Number 1	This amendment was created to report:<br>• Long-term matrix stability at -70 °C for up to 201 days for free zidebactam in treated human plasma ultrafiltrate, with sodium heparin as anticoagulant with and without the presence of comed[2].
2. Amendment Number 2	This amendment was created to report:<br>• Accuracy and precision to support linear regression.<br>• Platform change (API5000 vs API6500).

3. TABLE OF CONTENTS FOR THE BIOANALYTICAL METHOD VALIDATION REPORT

1. TITLE: THE DETERMINATION OF FREE ZIDEBACTAM IN TREATED HUMAN PLASMA ULTRAFILTRATE, WITH SODIUM HEPARIN AS ANTICOAGULANT, BY LC-MS/MS 1
2. SYNOPSIS 2
3. TABLE OF CONTENTS FOR THE BIOANALYTICAL METHOD VALIDATION REPORT 3
4. LIST OF TABLES 4
5. SIGNATURE PAGE 5
6. QUALITY ASSURANCE STATEMENT 6
7. STUDY COMPLIANCE STATEMENT 7
8. LIST OF ABBREVIATIONS AND DEFINITIONS OF TERMS 8
9. INTRODUCTION 10
10. EXPERIMENTAL DESIGN AND RESULTS 10
10.1. Reference Standards 10
10.2. Calibration Standards and QC Samples Preparation 10
10.3. Calibration Curve and Range 11
10.4. Accuracy and Precision (A&P) 11
10.5. Carryover 12
10.6. Platform Change 12
10.7. SOP Deviations and Investigations 12
11. CONCLUSION 12
12. PERTINENT SOPS 12
13. ARCHIVES 13
14. REFERENCES 13
15. TABLES 14

4. LIST OF TABLES

Table 1: Abbreviations and Codes 8
Table 2: Mass Spec Transitions 14
Table 3: Reference Standards 14
Table 4: Calibration Standards and QC Samples Preparation 14
Table 5: Matrix Description 14
Table 6: Summary of Results and Conclusions 15
Table 7: Summary of Validation Run Objectives and Outcome 18
Table 8: Summary of Standard Curves 19
Table 9: Accuracy and Precision (Intra-Assay) 20
Table 10: Accuracy and Precision (Inter-Assay) 21
Table 11: Carryover 22

Annex 4 .... Certificate(s) of Analysis
Annex 5 .... Test Procedure

5. SIGNATURE PAGE

Bioanalytical Project Number: V1872106P4

Amendment 2 Issue Date: Date of PI Signature

APPROVED

DocuSigned by:
Anika Pippin
Signer Name: Anika Pippin
Signing Reason: I approve this document
Signing Time: 28-Jun-2024 | 11:20 CDT
BE3AA1198E40459E8A7E3C615D981D82

Anika Pippin, B.S.
Principal Investigator

REVIEWED

DocuSigned by:
Marsha Luna
Signer Name: Marsha Luna
Signing Reason: I have reviewed this document
Signing Time: 28-Jun-2024 | 09:34 CDT
C9CC738561AF4CC1B375ED013DD1E4BD

Marsha Luna, B.S.
Management

6. QUALITY ASSURANCE STATEMENT

This Bioanalytical Method Validation Report has been found to describe accurately the methods and SOPs used and to accurately reflect the raw data collected. The Quality Assurance Unit initiated audits on this study and reported the findings to the Principal Investigator (PI) and Management as follows:

		Date(s) Sent to:	
**Inspection Date(s)**	**Inspection Phase**	**PI**	**Management**
21May2024	Data Audit	21May2024	27Jun2024
19Jun2024	Data Audit	19Jun2024	26Jun2024
19Jun2024	Draft Report Audit	19Jun2024	27Jun2024
27Jun2024 and 28Jun2024	Final Report Review	28Jun2024	28Jun2024

REVIEWED

DocuSigned by:
McKenzie Moses
Signer Name: McKenzie Moses
Signing Reason: I have reviewed this document
Signing Time: 28-Jun-2024 | 09:33 CDT
EC84DBFDD7D44D238BCE4363EEA6D90A

McKenzie Moses, B.S.
Quality Assurance Unit

7. STUDY COMPLIANCE STATEMENT

I, the undersigned, hereby declare that this study was conducted in compliance with the relevant bioanalytical aspects of FDA Guidance for Industry on Bioanalytical Methods Validation, May 2018 [1] and KCAS Bio (KCAS) procedures and that the work was performed by me, or under my direction, and the report reflects a true and accurate record of the results obtained.

The acceptance criteria are based on the FDA Guidance for Industry, Bioanalytical Method Validation (2018). [1]

APPROVED

DocuSigned by:

Anika Pippin

Signer Name: Anika Pippin
Signing Reason: I approve this document
Signing Time: 28-Jun-2024 | 11:21 CDT
BE3AA1198E40459E8A7E3C615D981D82

Anika Pippin, B.S.
Principal Investigator

8. LIST OF ABBREVIATIONS AND DEFINITIONS OF TERMS

Table 1: Abbreviations and Codes

Abbreviation	Definition
<	Concentration less than the lowest acceptable calibration standard
>	Concentration exceeded the upper limit of calibration range (> ULOQ)
A	Amendment
A&P	Accuracy and Precision
AQL	Above Quantitation Limit
ARQC	Above Range Quality Control
Batch	Is the same as Run
BQL	Below Quantifiable Limit
Cal	Calibration standard
Conc.	Concentration
Conv.	Conversion
%CV	Coefficient of Variation
dev	Deviation
diff	Difference
Dil	Dilution
EMA	European Medicines Agency
FDA	Food and Drug Administration
GLP	Good Laboratory Practice
HPLC	High Performance Liquid Chromatography
ID	Identification
ICH	International Council for Harmonisation of Technical Requirements for Registration of Pharmaceuticals for Human Use
IS	Internal Standard
ISR	Incurred Sample Reanalysis
K_2EDTA or K_3EDTA	Dipotassium or Tripotassium Ethylenediaminetetraacetic Acid
KCAS	KCAS Bio
LC-MS/MS	Liquid Chromatography Tandem Mass Spectrometry
LLOQ	Lower Limit of Quantitation

Table 1: Abbreviations and Codes (Continued)

Abbreviation	Definition
mcg or µg or ug	Microgram
mcL or µL or uL	Microliter
ME	Matrix Effect
MF	Matrix Factor
mL	Milliliter
MS	Mass Spectrometry
n	number
N.C.	Not Calculated
ND	No difference reported as ISR will be counted
ng	nanogram
n.report	No Data available to report
N/A or NA	Not Applicable
N.R.	Not Reported
p.a.r.	Peak Area Ratio
PI	Principal Investigator
QC	Quality Control Sample
QCs	Quality Control Samples
OECD	Organization for Economic Co-operation and Development
RT	Room Temperature
sd or SD	Standard deviation
Seq	Sequence
SOP (s)	Standard Operating Procedure (s)
temp	Temperature
TP	Test procedure
UHPLC	Ultra High Performance Liquid Chromatography
ULOQ	Upper Limit of Quantitation
WAS	Working Analyte Standard
Watson LIMS 7.5	Watson Laboratory Information Management System, Version 7.5, Service Pack 1
WIS	Working Internal Standard

This is a basic list. Some of the abbreviations may not have been used in this report.

9. INTRODUCTION

This amendment was created to report accuracy and precision to support linear regression and platform change (API 5000 vs API 6500).

The validation procedure was designed to meet or exceed the requirements of FDA Guidance for Industry on Bioanalytical Methods Validation, May 2018. The software programs used were Microsoft Office Excel 365, acquisition and processing software Analyst 1.7.2, and data management software Watson LIMS 7.5.

10. EXPERIMENTAL DESIGN AND RESULTS

In this method, free zidebactam and internal standard, cefotaxime, were released from treated human plasma ultrafiltrate using 96 well protein precipitation. After extraction, the sample was chromatographed using reversed phase HPLC with a Fortis C18 analytical column. Zidebactam and cefotaxime (IS) were detected by monitoring the precursor and product ions described in Table 2 using an Applied Biosystems Sciex API 5000 LC-MS/MS. The quantification was based on setting a calibration graph using the internal standard method based on bioanalytical test procedure TP-80217, entitled “LC-MS/MS Analysis of Free Zidebactam in Treated Human Plasma Ultrafiltrate (Sodium Heparin, 50 mcL, 0.0500 – 50.0 mcg/mL; Prior to MES Treatment)”. The updated test procedure can be found in Annex 5.

Human plasma, screened for the lack of interference at the retention times of zidebactam and cefotaxime (IS), was diluted 1:1 with MES buffer, pH 6.4 for stabilization. Since the standards and QCs concentrations were already adjusted for the addition of MES during the preparation, the obtained free zidebactam concentration of the unknown study samples do not need to be adjusted for the dilution with MES buffer.

A summary of the validation results is provided in Table 6. A summary describing the objective(s) and outcome of each validation run attempted is provided in Table 7.

The method was validated within the analytical range of 0.0500 – 50.0 mcg/mL in treated human plasma ultrafiltrate. No development work was performed after the method was validated.

10.1. Reference Standards

Reference standards used during this validation are presented in Table 3.

10.2. Calibration Standards and QC Samples Preparation

Calibration standards and quality control pools were prepared by spiking appropriate levels of zidebactam into human plasma ultrafiltrate, with sodium heparin as anticoagulant. Primary reference standards were prepared in at least duplicate and compared within 5% of each other. Standards and QCs were prepared and stored frozen at KCAS at -70 °C. Standards, QCs, and the

critical solutions used during this validation are described in Table 4, and matrix used is described in Table 5.

10.3. Calibration Curve and Range

The calibration curve demonstrates the relationship between the nominal analyte concentration and the response of the analytical platform to the analyte. Calibration standards, prepared by spiking matrix with a known quantity of analyte(s), span the calibration range and comprise the calibration curve.

The calibration curve was constructed with eight different, non-zero standard concentrations ranging from 0.0500 – 50.0 mcg/mL in treated human plasma ultrafiltrate. Calibration standards were processed in duplicate and the calibration curve was evaluated using linear regression with weighting $1/x^2$. The maximum allowable percent deviation from nominal for each standard was ± 15%, except at the LLOQ, where ± 20% was accepted. Any standard deviating by more than these limits was required to be excluded from the regression line. For the calibration curve to be accepted, at least 75% of the initial number of standards was required to remain in the curve. In addition, for accuracy and precision (A&P) runs, the criteria (within ± 15% or ± 20% for LLOQ) should also be fulfilled for at least 50% of the calibration standards tested per concentration level. In addition, run acceptance must be met. If both replicates of either the LLOQ or the ULOQ are rejected, the run cannot be used as an A&P run.

Linear regression was used for the calibration curve with $1/x^2$ weighting for the runs reported in this amendment. The acceptance criteria were met, and the data are presented in Table 8.

10.4. Accuracy and Precision (A&P)

The accuracy of a method describes the closeness of a measured result to its true value. For the purpose of validation, mean %Bias is the statistical measure of accuracy. The precision of a method describes the closeness of multiple results from the same sample to each other. The % Coefficient of Variation (%CV) is the statistical measure of precision.

A&P to support linear regression for this method was determined by analyzing the QCs within each run (within-run) and in different runs (between-run). A&P was evaluated using the same runs and data. Within-run A&P was evaluated by analyzing at least five replicates at each QC concentration level (LLOQ, low, mid, and high) in each analytical run. Between-run accuracy and precision to support linear regression were evaluated by analyzing each QC concentration level in two analytical runs.

The accuracy at each concentration level should be within ± 15% of the nominal concentration, except at the LLOQ, where it should be within ± 20%. The precision (%CV) of the concentrations determined at each level should not exceed 15%, except at the LLOQ, where it should not exceed 20%.

The acceptance criteria were met. The intra-assay and inter-assay accuracy and precision data for this method met the required criteria and are presented in Table 9 (intra-assay accuracy and precision) and Table 10 (inter-assay accuracy and precision).

10.5. Carryover

Carryover is evaluated within each run. It consists of measuring the response for each analyte and if it exceeds 20% of the response of LLOQ standard or other sample reflecting the lower sensitivity limit required, a carryover assessment is completed, and affected samples are rejected. The internal standard carryover evaluation is assessed within each run and if it exceeds 5% of the average internal standard response for the acceptable LLOQ standard, impact is evaluated within the run. Data are presented in Table 11.

10.6. Platform Change

Platform change was assessed by injecting run 1 on a different MS platform (Applied Biosystems API 6500).

Data were required to meet the accuracy and precision criteria outlined in Section 10.3 and Section 10.4, along with the sensitivity QC having signal to noise of at least 5, to be considered an acceptable platform to be used.

The acceptance criteria were met and the data are provided in data are provided in Table 9 to Table 10 for the precision/accuracy portion of this experiment.

10.7. SOP Deviations and Investigations

No protocol deviations occurred during this study. No investigations were conducted during this study.

11. CONCLUSION

The intra-assay and inter-assay accuracy and precision data for this method met the required criteria to support linear regression. Platform change (API 5000 vs API 6500) has been proven.

12. PERTINENT SOPS

The following SOPs were followed during the conduct of this study:

SOP-00073 [05-07] - Bioanalytical Method Validation - Chromatographic Assays

SOP-00035 [02-03] - Procedures, Reporting, and Acceptance Criteria for Bioanalytical Chromatographic Projects

13. ARCHIVES

All raw data, including paper and electronic files associated with this study will remain archived at a location specified by KCAS, 10830 S Clay Blair Boulevard, Olathe, KS 66061 for a period of at least 10 years, after which the client may be contacted to arrange for continued storage of the files, or disposition according to an agreed procedure. KCAS will maintain an electronic (scanned/true) copy of the method validation report that can be viewed and printed on site. The study plan, documentation, raw data, and final report are indexed under Bioanalytical Project Number V1872106P4A2.

14. REFERENCES

1. Guidance for Industry: Bioanalytical Method Validation. U.S. Department of Health and Human Services. Food and Drug Administration. Center for Drug Evaluation and Research. Center for Veterinary Medicine. May 2018.
2. Comeds: QCs in the presence of 150 mcg/mL ertapenem.

15. TABLES

Table 2: Mass Spec Transitions

Analyte	Transition
Zidebactam	m/z 392.028→312.000
Cefotaxime (IS)	m/z 456.200→324.000

Table 3: Reference Standards

Analyte	Supplier	Lot/Batch Number	Purity	Expiration or Retest Date	Conversion Factor	Storage Condition
Zidebactam	Wockhardt	WK1/WP-0060/016	90.4%	Expire 01Mar2024	1.000	2-8°C
Zidebactam	Wockhardt	WK1/A-956/153	90.7%	Retest 10Jan2025	1.000	2-8°C
Cefotaxime (IS)	Sigma-Aldrich	0000146942	95.9%	Retest 18Oct2025<br>Retest 30Sep2023*	1.048	2-8°C

Certificates of analysis for the above reference standards are presented in Annex 4.
*Reference material was within retest at the time of use.

Table 4: Calibration Standards and QC Samples Preparation

Critical Solutions	Lot # for Standards (Prep Date)	Lot # for QCs (Prep Date)	Standards Lot (Prep Date)	QCS Lot (Prep Date)	Runs used
Treated Sodium Heparin Human Plasma	V1872106P4A1-11-2		V1872106P4A1-11-1 Set C (17May2023)	V1872106P4A1-12-1 Set C (17May2023)	1
Deionized water stored at -20 °C in polypropylene	V1872106P4A1-5-1 (15May2023) V1872106P4A1-7-1 (15May2023)	V1872106P4A1-6-1 (15May2023) V1872106P4A1-8-1 (15May2023)			
Treated Sodium Heparin Human Plasma	V1872106P4A2-14-2		V1872106P4A2-13-1 Set A (05Jun2024)	V1872106P4A2-14-1 Set A (05Jun2024)	4
Deionized water stored at -20 °C in polypropylene	V1872106P4A2-8-1 (04Jun2024) V1872106P4A2-9-1 (04Jun2024)	V1872106P4A2-7-1 (04Jun2024) V1872106P4A2-10-1 (04Jun2024)			

Table 5: Matrix Description

Matrix	Source Lot #	Date Received
Sodium Heparin Human Plasma V1872106P4A2-11-2	BioIVT, Lot HMN1086396	Mar2023
Sodium Heparin Human Plasma V1872106P4A2-14-2	BioIVT, Lot HMN1086398	Mar2023

Table 6: Summary of Results and Conclusions

Methodology – TP-80217	Method Performance – LC-MS/MS	Hyperlink – N/A	Comments
Matrix	Plasma ultrafiltrate		
Anticoagulant	Sodium heparin		
Assay Volume	50 mcL		
**Selectivity**			
Analyte	Zidebactam		V1872106P4
Internal Standard	Cefotaxime		V1872106P4
Matrix Effect (6 spiked matrix lots)	Complies		V1872106P4
Interference	Complies		V1872106P4
Hemolytic Effect Evaluation	Complies		V1872106P4
Lipemic Effect Evaluation	Complies		V1872106P4
Carryover	Assessed with every run with standards and QCs	Summary tables Table 11<br><br>Report text Section 10.5	
**Linearity**			
Weighting	Quadratic $1/x^2$		V1872106P4
Bias at LLOQ	0.8%		V1872106P4
Bias above LLOQ	-2.6 to 2.0%		V1872106P4
Bioanalytical Range	Complies<br>0.0500 – 50.0 mcg/mL		V1872106P4
**Precision**			
Intra-assay	3.2 to 9.4% (LLOQ);<br>3.5 to 9.9% (above LLOQ)		V1872106P4
Inter-assay	6.1% (LLOQ);<br>5.4 to 6.3% (above LLOQ)		V1872106P4
**Accuracy**			
Intra-assay	8.4 to 9.2% (LLOQ);<br>1.2 to 11.3% (above LLOQ)		V1872106P4
Inter-assay	9.0% (LLOQ);<br>2.4 to 6.7% (above LLOQ)		V1872106P4
**Large Run Size**	Determined to be 158 samples.		V1872106P4

Table 6: Summary of Results and Conclusions (Continued)

Methodology – TP-80217	Method Performance – LC-MS/MS	Hyperlink – N/A	Comments
**Dilution**			
Dilution factor: 2x	Dilution QC: 37.5 mcg/mL<br>Precision: 3.0%<br>Accuracy: 3.5%		V1872106P4
Dilution factor: 10x	Dilution QC: 400 mcg/mL<br>Precision: 6.7%<br>Accuracy: 1.5%		V1872106P4
**Regression Change**			
Weighting	Linear $1/x^2$	Table 8<br>Report text<br>Section 10.3	
Bias at LLOQ	1.2%	Table 8<br>Report text<br>Section 10.3	
Bias above LLOQ	-4.4 to 3.0%	Table 8<br>Report text<br>Section 10.3	
Bioanalytical Range	Complies<br>0.0500 – 50.0 mcg/mL	Table 8<br>Report text<br>Section 10.3	
**Precision**			
Intra-assay	4.1 to 10.9% (LLOQ);<br>0.6 to 5.0% (above LLOQ)	Table 9<br>Report text<br>Section 10.4	
Inter-assay	10.6% (LLOQ);<br>4.3 to 5.0% (above LLOQ)	Table 10<br>Report text<br>Section 10.4	
**Accuracy**			
Intra-assay	-9.2 to 3.2% (LLOQ);<br>-6.9 to 2.8% (above LLOQ)	Table 9<br>Report text<br>Section 10.4	
Inter-assay	-3.0% (LLOQ);<br>-3.2 to 0.0% (above LLOQ)	Table 10<br>Report text<br>Section 10.4	

Table 6: Summary of Results and Conclusions (Continued)

Methodology – TP-80217	Method Performance – LC-MS/MS	Hyperlink – N/A	Comments
**Stability**			
Matrix (in polypropylene tubes)			
Freeze/Thaw (-20 °C/wet ice) with and without the presence of comeds[2]	Demonstrated for 3 cycles		V1872106P4
Freeze/Thaw (-70 °C/wet ice) with and without the presence of comeds[2]	Demonstrated for 3 cycles		V1872106P4
Bench-top stability in wet ice with and without the presence of comeds[2]	Demonstrated for 24 hours		V1872106P4
Long-term (-20 °C) with and without the presence of comeds[2]	Demonstrated instability at 39 days		V1872106P4
Long-term (-70 °C) with and without the presence of comeds[2]	Demonstrated for 201 days		V1872106P4A1
Processed Matrix polypropylene			
Refrigeration	Demonstrated for 5 day 21 hours		V1872106P4
**Individual Sample Reinjection**	Reproducible		V1872106P4
**Whole Run Reinjection Integrity**	Reproducible<br>Duration 6 days 20 hours		V1872106P4
**Column Ruggedness**	Reproducible		V1872106P4
**Platform Change (API5000 vs API6500)**	Reproducible	Summary tables<br>Table 9<br><br>Report text<br>Section 10.6	
**Extraction Recovery**			
Zidebactam	Overall 109.3%		V1872106P4
Cefotaxime (IS)	Overall 115.7%		V1872106P4

Table 6: Summary of Results and Conclusions (Continued)

Methodology – TP-80217	Method Performance – LC-MS/MS	Hyperlink – N/A	Comments
**Solution Stability**			
Solution, Primary polypropylene or glass			
Zidebactam in deionized water (RT) - glass	Stable 17.75 hours		V1872106U2
Zidebactam in deionized water (-20 °C) - glass	Stable 61 days		V1872106U2
Zidebactam in deionized water (-20 °C) - polypropylene	Stable 39 days		V1872106P4
Zidebactam Spike in deionized water (RT) - polypropylene	Stable 17.75 hours		V1872106P4
Zidebactam Spike in deionized water (-20 °C) - glass	Stable 25 days<br>Stable 61 days (high QC only)		V1872106P4
Cefotaxime (IS) in deionized water in glass (RT) - glass	Stable 15.5 hours		V1872106P4
Cefotaxime (IS) in deionized water in glass (RT) - glass	Can be used for up to 3 months per SOP-00073.		SOP-00073

Table 7: Summary of Validation Run Objectives and Outcome

Run ID	Instrument ID	Extraction Date	Assay Date	Run Objective(s)	Accepted or Rejected	Comment
1	LC/MS-31	21Aug2023	23Aug2023	Accuracy & precision for linear regression and platform change (API500 vs API6500)	Accepted	
4	LC/MS-22	05Jun2024	05Jun2024	Accuracy & precision for linear regression	Accepted	

Runs 2 and 3 were used for spike check and primary comparison and will not be reported.

Table 8: Summary of Standard Curves

Analytical Performance: Back-Calculated Concentrations (µg/mL) of Zidebactam Calibration Standard in (Human) (Plasma Ultrafiltrate) in (Study V1872106P4 A2)

Assay Date	Run ID	Cal 0.05 0.0500 µg/mL	Cal 0.1 0.100 µg/mL	Cal 1 1.00 µg/mL	Cal 5 5.00 µg/mL	Cal 10 10.0 µg/mL	Cal 20 20.0 µg/mL	Cal 40 40.0 µg/mL	Cal 50 50.0 µg/mL
23-Aug-2023	1	0.0518	0.0980	0.990	4.74	9.37	*16.4	36.1	43.9
		0.0484	0.101	1.04	5.38	10.7	21.1	42.6	52.3
05-Jun-2024	4	0.0547	0.0931	1.09	5.22	10.6	21.1	39.8	47.6
		0.0476	0.0970	0.977	4.92	9.75	19.6	39.3	47.5
Mean		0.0506	0.0973	1.02	5.07	10.1	20.6	39.5	47.8
S.D.		0.00327	0.00326	0.0516	0.289	0.649	0.866	2.66	3.44
%CV		6.5	3.4	5.1	5.7	6.4	4.2	6.7	7.2
%Bias		1.2	-2.7	2.0	1.4	1.0	3.0	-1.3	-4.4
n		4	4	4	4	4	3	4	4
Reason Deactivated									
* Excluded due to being outside acceptance criteria									

See Table 7 for runs not used for accuracy/precision evaluation.

Calibration Curve Parameters for Zidebactam Calibration Standards in Human Plasma Ultrafiltrate (Study V1872106P4 A2)

Run Date	Run ID	Slope	Intercept	R-Squared	LLOQ	ULOQ
23-Aug-2023	1	0.18273	-0.00018	0.9950	0.0500	50.0
05-Jun-2024	4	0.26043	0.00280	0.9966	0.0500	50.0

Table 9: Accuracy and Precision (Intra-Assay)

Human Plasma Ultrafiltrate Zidebactam Concentrations in Quality Controls for 2 separate batch runs (Study V1872106P4 A2)

Run Date	Run ID	QC 0.05 0.0500 µg/mL	%Bias	QC 0.15 0.150 µg/mL	%Bias	QC 25 25.0 µg/mL	%Bias	QC 37.5 37.5 µg/mL	%Bias
23-Aug-2023	1	0.0463	-7.4	0.158	5.3	23.9	-4.4	35.1	-6.4
		0.0445	-11.0	0.143	-4.7	23.9	-4.4	34.3	-8.5
		0.0425	-15.0	0.146	-2.7	23.0	-8.0	34.7	-7.5
		0.0476	-4.8	0.137	-8.7	23.7	-5.2	35.5	-5.3
		0.0448	-10.4	0.153	2.0	24.0	-4.0	34.6	-7.7
		0.0468	-6.4	0.147	-2.0	24.8	-0.8	35.1	-6.4
Intrarun Mean		0.0454		0.147		23.9		34.9	
Intrarun SD		0.00185		0.00739		0.578		0.431	
Intrarun %CV		4.1		5.0		2.4		1.2	
Intrarun %Bias		-9.2		-2.0		-4.4		-6.9	
n		6		6		6		6	
05-Jun-2024	4	0.0485	-3.0	0.156	4.0	27.0	8.0	37.9	1.1
		#0.0619	23.8	0.155	3.3	26.4	5.6	38.0	1.3
		0.0508	1.6	0.143	-4.7	24.9	-0.4	37.6	0.3
		0.0522	4.4	0.147	-2.0	25.2	0.8	37.5	0.0
		0.0511	2.2	0.163	8.7	25.1	0.4	38.0	1.3
		0.0452	-9.6	0.152	1.3	25.7	2.8	37.8	0.8
Intrarun Mean		0.0516		0.153		25.7		37.8	
Intrarun SD		0.00562		0.00706		0.828		0.210	
Intrarun %CV		10.9		4.6		3.2		0.6	
Intrarun %Bias		3.2		2.0		2.8		0.8	
n		6		6		6		6	
# > 20%Bias									

Note: Run 1 was used to assess platform change on a different MS platform (Applied Biosystems API 5000 vs API 6500).

Criteria: See Section 10.4.
Results: The acceptance criteria were met.

Table 10: Accuracy and Precision (Inter-Assay)

Run Date	Run ID	QC 0.05 0.0500 µg/mL	%Bias	QC 0.15 0.150 µg/mL	%Bias	QC 25 25.0 µg/mL	%Bias	QC 37.5 37.5 µg/mL	%Bias
23-Aug-2023	1	0.0463	-7.4	0.158	5.3	23.9	-4.4	35.1	-6.4
		0.0445	-11.0	0.143	-4.7	23.9	-4.4	34.3	-8.5
		0.0425	-15.0	0.146	-2.7	23.0	-8.0	34.7	-7.5
		0.0476	-4.8	0.137	-8.7	23.7	-5.2	35.5	-5.3
		0.0448	-10.4	0.153	2.0	24.0	-4.0	34.6	-7.7
		0.0468	-6.4	0.147	-2.0	24.8	-0.8	35.1	-6.4
05-Jun-2024	4	0.0485	-3.0	0.156	4.0	27.0	8.0	37.9	1.1
		#0.0619	23.8	0.155	3.3	26.4	5.6	38.0	1.3
		0.0508	1.6	0.143	-4.7	24.9	-0.4	37.6	0.3
		0.0522	4.4	0.147	-2.0	25.2	0.8	37.5	0.0
		0.0511	2.2	0.163	8.7	25.1	0.4	38.0	1.3
		0.0452	-9.6	0.152	1.3	25.7	2.8	37.8	0.8
Mean Concentration Found (µg/mL)		0.0485		0.150		24.8		36.3	
Inter-run SD		0.00514		0.00743		1.17		1.56	
Inter-run %CV		10.6		5.0		4.7		4.3	
Inter-run %Bias		-3.0		0.0		-0.8		-3.2	
n		12		12		12		12	
# > 20%Bias									

Criteria: See Section 10.4.
Results: The acceptance criteria were met.

Table 11: Carryover

Run ID	Result (Extracted Blank after High Standard)	Carryover Results for Internal Standard
1	Seq 13 and Seq 50 are <20% of the LLOQ.	Seq 4 and Seq 41 are < 5% internal standard peak of acceptable standards.
4	Seq 14 is <20% of the LLOQ and Seq 51 is 29% of the LLOQ.	Seq 5 and Seq 42 are < 5% internal standard peak of acceptable standards.

Note: Calibration Standards and QCs are arranged in a way to mitigate carryover in all validation runs.

ANALYTICAL RESEARCH

CERTIFICATE OF ANALYSIS FOR REFERENCE STANDARD

**Name**	:	**Zidebactam (WCK 5107)**			
Molecular Formula	:	$C_{13}H_{21}N_5O_7S.2H_2O$	Batch Number	:	WK1/A-956/153
Molecular Weight	:	427.45	Laboratory Note Book Reference	:	WA-2362-078
Date of Analysis	:	11/01/2024	Month of Manufacturing	:	May' 2022
Valid Up To	:	10/01/2025	Reference Standard Number	:	RS/2024-005
Effective Date	:	08/02/2024	Page Number	:	Page 1 of 4

Sr. No.	Tests	Specifications	Results
1	Description	White to pale yellow powder	White powder
2	Identification (By IR, Mass & NMR Spectroscopy)	Spectral data should conform to the structure	Spectral data conforms the structure
3	Solubility	Freely soluble in water, Insoluble in dichloromethane	Freely soluble in water, Insoluble in dichloromethane
4	Completeness and Clarity of Solutions (10% w/v solution in Water For Injection (WFI))	The solid dissolves completely, leaving no undissolved matter.	Complies
		The constituted solution is not significantly less clear than an equal volume of the WFI contained in a similar vessel and examined similarly.	Complies
		The constituted solution is free from particulate matter that can be observed on visual inspection.	Complies
5	pH of Solution	5.0 to 7.0	5.4
6	Absorbance of Solution (1.25 % w/v in water at 420 nm)	Not more than 0.2 AU	0.01 AU
7	Water Content (By KF Titration)	Not more than 12.0 % w/w	8.6 % w/w
8	Specific Optical Rotation (On anhydrous basis) (0.5 % w/v solution in water)	–28° to –40°	–37°
9	Palladium Content (By ICP-MS)	Not more than 10 µg/g	0.3 µg/g

	Prepared by	Checked by	Approved by
Name :	Mr. Sitaram Ubale	Mr. Rajendra Chavan	Mr. Subhash Chandra Shukla
Department :	AVL	AVL	CRDQ
Sign :	[signature]	[signature]	[signature]
Date :	08/02/2024	08/02/2024	08/02/2024

Format No. : AVL-045-F-02-00

Ranked # 1 in Healthcare
GREAT PLACE TO WORK Best Companies to Work for 2015 India

WOCKHARDT | LIFE WINS

ANALYTICAL RESEARCH

CERTIFICATE OF ANALYSIS FOR REFERENCE STANDARD

Name	:	**Zidebactam (WCK 5107)**			
Molecular Formula	:	$C_{13}H_{21}N_5O_7S.2H_2O$	Batch Number	:	WK1/A-956/153
Molecular Weight	:	427.45	Laboratory Note Book Reference	:	WA-2362-078
Date of Analysis	:	11/01/2024	Month of Manufacturing	:	May' 2022
Valid Up To	:	10/01/2025	Reference Standard Number	:	RS/2024-005
Effective Date	:	08/02/2024	Page Number	:	Page 2 of 4

Sr. No.	Tests	Specifications	Results
10	Related Substances (By HPLC) :		
	i. Impurity -1	Not more than 2.0 % w/w	0.21 % w/w
	ii. Impurity -2	Not more than 1.0 % w/w	Not detected
	iii. Impurity -3	Not more than 1.0 % w/w	0.03 % w/w
	iv. Impurity -4	Not more than 0.10 % w/w	Not detected
	v. Specified Impurity at RRT 2.16-2.28 (MW 782)	Not more than 0.50 % w/w	0.03 % w/w
	vi. Any Unspecified Impurity # Individual Unspecified Impurity	Not more than 0.15 % w/w	BRL
	vii. Total Impurities	Not more than 4.0 % w/w	0.28 % w/w
11	Residual Solvents (By GC) :		
	Group A		
	i. Isopropyl alcohol	Not more than 5000 ppm	Not detected
	ii. Dichloromethane	Not more than 600 ppm	Not detected
	iii. Methanol	Not more than 3000 ppm	1403 ppm
	iv. Ethyl acetate	Not more than 5000 ppm	Not detected
	v. Cyclohexane	Not more than 3880 ppm	Not detected
	vi. Acetone	Not more than 5000 ppm	Not detected
	vii. Methyl tertiary butyl ether	Not more than 5000 ppm	Not detected
	viii. Toluene	Not more than 890 ppm	Not detected
	ix. Ethanol	Not more than 5000 ppm	2945 ppm
	Group B		
	i. Triethylamine	Not more than 250 ppm	Not detected
	ii. Pyridine	Not more than 200 ppm	Not detected

	Prepared by	Checked by	Approved by
Name :	Mr. Sitaram Ubale	Mr. Rajendra Chavan	Mr. Subhash Chandra Shukla
Department :	AVL	AVL	CRDQ
Sign :	[signature]	[signature]	[signature]
Date :	08/02/2024	08/02/2024	08/02/2024

Format No. : AVL-045-F-02-00

WOCKHARDT | LIFE WINS

ANALYTICAL RESEARCH

CERTIFICATE OF ANALYSIS FOR REFERENCE STANDARD

**Name**	:	**Zidebactam (WCK 5107)**			
Molecular Formula	:	$C_{13}H_{21}N_5O_7S.2H_2O$	Batch Number	:	WK1/A-956/153
Molecular Weight	:	427.45	Laboratory Note Book Reference	:	WA-2362-078
Date of Analysis	:	11/01/2024	Month of Manufacturing	:	May' 2022
Valid Up To	:	10/01/2025	Reference Standard Number	:	RS/2024-005
Effective Date	:	08/02/2024	Page Number	:	Page 3 of 4

Sr. No.	Tests	Specifications	Results
12	Sodium Content (By ICP-MS)	Not more than 0.5 % w/w	BLQ (LOQ 100 ppm)
13	Sulfate Content (By Ion Chromatography)	Not more than 1.0 % w/w	BLQ (LOQ 0.626 % w/w)
14	Monomethyl sulphate Content (By Ion Chromatography)	Not more than 0.15% w/w	Not detected
15	Polymorphism (By X-ray Powder Diffraction)	The diffractogram should exhibit characteristic 2 θ values at 13.8°, 15.6°, 20.3° and 24.3° (± 0.2°)	The diffractogram exhibits characteristic 2 θ values at 13.8°, 15.6°, 20.4° & 24.3°
16	Potency (On "as is" basis)	Not applicable	90.7 %

BLQ = Below Limit of Quantitation

BRL = Below Reporting Level

LOQ = Limit of Quantitation

All Unspecified Impurities ≥ 0.02 % w/w reported individually along with their respective Relative Retention Times (RRT's).

Specification Reference Number: ZDB-DSS-112-07

Remark: Potency is calculated on the basis of Total Impurities, Water Content and Residual Solvents.

Storage Condition: Store at 2 °C to 8 °C temperature.

	Prepared by	Checked by	Approved by
Name :	Mr. Sitaram Ubale	Mr. Rajendra Chavan	Mr. Subhash Chandra Shukla
Department :	AVL	AVL	CRDQ
Sign :	[signature]	[signature]	[signature]
Date :	08/02/2024	08/02/2024	08/02/2024

Format No. : AVL-045-F-02-00

Ranked # 1 in Healthcare
GREAT PLACE TO WORK Best Companies to Work for 2015 India

WOCKHARDT | LIFE WINS

ANALYTICAL RESEARCH

CERTIFICATE OF ANALYSIS FOR REFERENCE STANDARD

Name	:	**Zidebactam (WCK 5107)**			
Molecular Formula	:	$C_{13}H_{21}N_5O_7S.2H_2O$	Batch Number	:	WK1/A-956/153
Molecular Weight	:	427.45	Laboratory Note Book Reference	:	WA-2362-078
Date of Analysis	:	11/01/2024	Month of Manufacturing	:	May' 2022
Valid Up To	:	10/01/2025	Reference Standard Number	:	RS/2024-005
Effective Date	:	08/02/2024	Page Number	:	Page 4 of 4

Chemical Names:

i. **WCK 5107**: (2S,5R)-7-Oxo-6-sulphooxy-2-[N'-((R)-piperidin-3-carbonyl)hydrazinocarbonyl]-1,6-diaza-bicyclo[3.2.1]octane dihydrate

ii. **Impurity-1**: (2S,5R)-5-Sulphooxyamino-2-[N'-((R)-piperidin-3-carbonyl)hydrazinocarbonyl] piperidine

iii. **Impurity-2**: (2S,5R)-7-Oxo-6-hydroxy-2-[N'-((R)-piperidin-3-carbonyl)hydrazinocarbonyl]-1,6-diaza-bicyclo[3.2.1]octane

iv. **Impurity-3**: (2S,5R)-7-Oxo-6-sulphooxy-2-[N'-((S)-piperidin-3-carbonyl)hydrazinocarbonyl]-1,6-diaza-bicyclo[3.2.1]octane

v. **Impurity-4**: (2S,5R)-7-Oxo-6-sulphooxy-2-[N'-((R)-1-tert-butoxycarbonylpiperidin-3-yl)carbonyl) hydrazinocarbonyl]-1,6-diaza-bicyclo[3.2.1]octane

	Prepared by	Checked by	Approved by
Name :	Mr. Sitaram Ubale	Mr. Rajendra Chavan	Mr. Subhash Chandra Shukla
Department :	AVL	AVL	CRDQ
Sign :	[signature]	[signature]	[signature]
Date :	08/02/2024	08/02/2024	08/02/2024

Format No. : AVL-045-F-02-00

Ranked # 1 In Healthcare
GREAT PLACE TO WORK Best Companies to Work for 2015 India

WOCKHARDT LIFE WINS

ANALYTICAL RESEARCH

CERTIFICATE OF ANALYSIS FOR REFERENCE STANDARD

**Name**	:	**Zidebactam (WCK 5107)**			
Molecular Formula	:	$C_{13}H_{21}N_5O_7S.2H_2O$	Batch Number	:	WK1/WP-0060/016
Molecular Weight	:	427.45	Laboratory Note Book Reference	:	WA-2362-010
Date Of Analysis	:	02/03/2023	Month Of Manufacturing	:	May' 2019.
Valid Up To	:	01/03/2024	Reference Standard Number	:	RS/2023-009
Effective Date	:	30/03/2023	Page Number	:	Page 1 of 4

Sr. No.	Tests	Specifications	Results
1	Description	White to pale yellow powder	White powder
2	Identification (By IR, Mass & NMR Spectroscopy)	Spectral data should conform to the structure	Spectral data conforms the structure
3	Solubility	Freely soluble in water, Insoluble in dichloromethane	Freely soluble in water, Insoluble in dichloromethane
4	Completeness and Clarity of Solutions (10% w/v solution in Water for Injection (WFI))	The solid dissolves completely, leaving no undissolved matter.	Complies
		The constituted solution is not significantly less clear than an equal volume of the WFI contained in a similar vessel and examined similarly.	Complies
		The constituted solution is free from particulate matter that can be observed on visual inspection.	Complies
5	pH of Solution	Between 5.0 to 7.0	6.0
6	Absorbance of Solution (1.25 % w/v in water at 420 nm)	Not more than 0.2 AU	0.01 AU
7	Water Content (By KF Titration)	Not more than 12.0 % w/w	8.6 % w/w
8	Specific Optical Rotation (On anhydrous basis) (0.5 % w/v solution in Water)	Between –28° to -40°	-36°
9	Palladium Content (By ICP-OES)	Not more than 10 µg/g	BLQ (LOQ 2.5 µg/g)*

	Prepared by	Checked by	Approved by
Name :	Mr. Sitaram Ubale	Mr. Rajendra Chavan	Ms. Mohini Khatik
Department :	AVL	AVL	CRDQ
Sign :	[signature]	[signature]	[signature]
Date :	10/03/2023	10/03/2023	11/03/2023

Format No. : AVL-045-F-02-00

Ranked # 1 in Healthcare
GREAT PLACE TO WORK Best Companies to Work for 2015 India

ANALYTICAL RESEARCH

CERTIFICATE OF ANALYSIS FOR REFERENCE STANDARD

**Name**	:	**Zidebactam (WCK 5107)**			
Molecular Formula	:	$C_{13}H_{21}N_5O_7S.2H_2O$	Batch Number	:	WK1/WP-0060/016
Molecular Weight	:	427.45	Laboratory Note Book Reference	:	WA-2362-010
Date Of Analysis	:	02/03/2023	Month Of Manufacturing	:	May' 2019
Valid Up To	:	01/03/2024	Reference Standard Number	:	RS/2023-009
Effective Date	:	30/03/2023	Page Number	:	Page 2 of 4

Sr. No.	Tests	Specifications	Results
10	Related Substances (By HPLC) :		
	i. Impurity -1	Not more than 2.00 % w/w	0.24 % w/w
	ii. Impurity -2	Not more than 1.00 % w/w	Not detected
	iii. Impurity -3	Not more than 1.00 % w/w	0.20 % w/w
	iv. Impurity -4	Not more than 0.10 % w/w	Not detected
	v. Specified Impurity at RRT 2.16-2.28 (MW 782)	Not more than 0.50 % w/w	0.02 % w/w
	vi. Any Unspecified Impurity at RRT 0.59	Not more than 0.15 % w/w	0.04 % w/w
	vii. Total Impurities	Not more than 4.00 % w/w	0.50 % w/w
11	Residual Solvents (By GC) :		
	Group A		
	i. Isopropyl alcohol	Not more than 5000 µg/g	Not detected
	ii. Dichloromethane	Not more than 600 µg/g	Not detected
	iii. Methanol	Not more than 3000 µg/g	1762 µg/g
	iv. Ethyl acetate	Not more than 5000 µg/g	Not detected
	v. Cyclohexane	Not more than 3880 µg/g	Not detected
	vi. Acetone	Not more than 5000 µg/g	Not detected
	vii. Methyl tertiary butyl ether	Not more than 5000 µg/g	Not detected
	viii. Toluene	Not more than 890 µg/g	Not detected
	ix. Ethanol	Not more than 5000 µg/g	2990 µg/g
	Group B		
	i. Triethylamine	Not more than 250 µg/g	Not detected
	ii. Pyridine	Not more than 200 µg/g	Not detected

	Prepared by	Checked by	Approved by
Name :	Mr. Sitaram Ubale	Mr. Rajendra Chavan	Ms. Mohini Khatik
Department :	AVL	AVL	CRDQ
Sign :	[signature]	[signature]	[signature]
Date :	10/03/2023	10/03/2023	11/03/2023

Format No : AVL-045-F-02-00

Ranked # 1 in Healthcare
GREAT PLACE TO WORK Best Companies to Work for 2015 India

ANALYTICAL RESEARCH

CERTIFICATE OF ANALYSIS FOR REFERENCE STANDARD

**Name**	:	**Zidebactam (WCK 5107)**			
Molecular Formula	:	$C_{13}H_{21}N_5O_7S.2H_2O$	Batch Number	:	WK1/WP-0060/016
Molecular Weight	:	427.45	Laboratory Note Book Reference	:	WA-2362-010
Date Of Analysis	:	02/03/2023	Month Of Manufacturing	:	May' 2019
Valid Up To	:	01/03/2024	Reference Standard Number	:	RS/2023-009
Effective Date	:	30/03/2023	Page Number	:	Page 3 of 4

Sr. No.	Tests	Specifications	Results
12	Sodium Content (By ICP-OES)	Not more than 0.5 % w/w	BLQ (LOQ 0.25 % w/w)*
13	Sulfate Content (By Ion chromatography)	Not more than 1.0 % w/w	BLQ (LOQ 0.626 % w/w)
14	Mono-methyl Sulphate Content (By Ion chromatography)	Not more than 0.15% w/w	Not detected
15	Polymorphism (By X-ray Diffraction)	The diffractogram should exhibit characteristic 2 theta values at 13.8, 15.6, 20.3 and 24.3 (± 0.2°)	The diffractogram exhibits characteristic 2 theta values at 13.8, 15.6, 20.3 & 24.3
16	Potency (On as is basis)	Not applicable	90.4 %

BLQ = Below limit of quantification

LOQ = Limit of quantification

* LOQ Values are based on analytical method verification report (Report nos. MVR/VP17053 and MVR/VP17054) of Analytical Solutions (CRO).

Specification Reference Number: **ZDB-DSS-112-06**

Remark: Potency is calculated on the basis of Total Impurities, Water Content and Residual solvents.

Storage Condition: Store at 2°C to 8°C temperature.

	Prepared by	Checked by	Approved by
Name :	Mr. Sitaram Ubale	Mr. Rajendra Chavan	Ms. Mohini Khatik
Department :	AVL	AVL	CRDQ
Sign :	[signature]	[signature]	[signature]
Date :	10/03/2023	10/03/2023	11/03/2023

Format No. : AVL-045-F-02-00

Ranked # 1 in Healthcare
GREAT PLACE TO WORK Best Companies to Work for 2015 India

ANALYTICAL RESEARCH

CERTIFICATE OF ANALYSIS FOR REFERENCE STANDARD

Name	:	**Zidebactam (WCK 5107)**			
Molecular Formula	:	$C_{13}H_{21}N_5O_7S.2H_2O$	Batch Number	:	WK1/WP-0060/016
Molecular Weight	:	427.45	Laboratory Note Book Reference	:	WA-2362-010
Date Of Analysis	:	02/03/2023	Month Of Manufacturing	:	May' 2019
Valid Up To	:	01/03/2024	Reference Standard Number	:	RS/2023-009
Effective Date	:	30/03/2023	Page Number	:	Page 4 of 4

Chemical Names:

i. **WCK 5107**: (2S,5R)-7-Oxo-6-sulphoxy-2-[N'-((R)-piperidin-3-carbonyl)-hydrazinocarbonyl]-1,6-diaza-bicyclo[3.2.1]octane dehydrate

ii. **Impurity-1**: (2S,5R)-5-sulphooxyamino-2-[N'-((R)-piperidin-3-carbonyl)-hydrazinocarbonyl]-piperidine

iii. **Impurity-2**: (2S,5R)-7-Oxo-6-hydroxy-2-[N'-((R)-piperidin-3-carbonyl)-hydrazinocarbonyl]-1,6-diaza-bicyclo[3.2.1]octane

iv. **Impurity-3**: (2S,5R)-7-Oxo-6-sulphooxy-2-[N'-((S)-piperidin-3-carbonyl)-hydrazinocarbonyl]-1,6-diaza-bicyclo[3.2.1]octane

v. **Impurity-4**: (2S,5R)-7-Oxo-6-sulphooxy-2-[N'-((R)-1-tert-butoxycarbonyl-piperidin-3-yl)-carbonyl)-hydrazinocarbonyl] -1,6-diaza-bicyclo[3.2.1]octane

	Prepared by	Checked by	Approved by
Name :	Mr. Sitaram Ubale	Mr. Rajendra Chavan	Ms. Mohini Khatik
Department :	AVL	AVL	CRDQ
Sign :	[signature]	[signature]	[signature]
Date :	10/03/2023	10/03/2023	11/03/2023

Format No. : AVL-045-F-02-00

Ranked # 1 in Healthcare
GREAT PLACE TO WORK Best Companies to Work for 2015 India

Sigma-Aldrich.

3050 Spruce Street, Saint Louis, MO 63103, USA
Website: www.sigma-aldrich.com
Email USA: techserv@sial.com
Outside USA: eurtechserv@sial.com

Certificate of Analysis

Product Name : **Cefotaxime sodium salt suitable for plant cell culture, BioReagent, powder**
Product Number : **C7039-500MG**
Batch Number : **0000146942**
Source Batch : 157206
CAS Number : 64485-93-4
Storage Temperature : Cooler/Refrigerated
Molecular Formula : $C_{16}H_{16}N_5NaO_7S_2$
Formula Weight : 477.45
Recommended Retest Date : Sep 2023
Quality Release Date : 02 Jan 2022

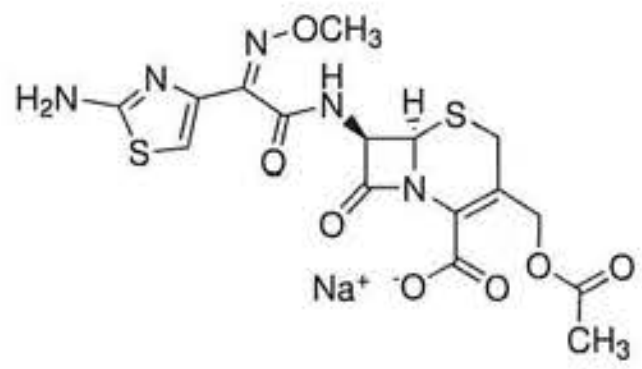

Test	Specification	Result
Appearance (Form)	Powder or Crystals	Powder
Appearance (Color)<br>Off-white to pale yellow	Conforms	Conforms
Solubility<br>10% in water	Clear	Clear
Infrared Spectrum	Conforms to Structure	Conforms
Total Impurity	≤ 3.0 %	1.7 %
Specific Rotation<br>[a]20/D	58 - 64 °	61 °
Assay (Dry Basis)	916 - 964 µg/mg	955 µg/mg
Loss on Drying	≤ 3.0 %	2.4 %
Plant Cell Culture Test	Pass	Pass

Anna Bendersky, Quality Control Manager
Quallity Control
Israel
IL

Sigma-Aldrich warrants, that at the time of the quality release or subsequent retest date this product conformed to the information contained in this publication. The current Specification sheet may be available at Sigma-Aldrich.com. For further inquiries, please contact Technical Service. Purchase must determine the suitability of the product for its particular use. See reverse side of website or packing slip for additional terms and conditions of sale

The branding on the header and/or footer of this document may temporarily not visually match the product purchased as we transition our branding. However, all of the information in the document regarding the product remains unchanged and matches the product ordered.
For further information please contact mlsbranding@sial.com

	TEST PROCEDURE - Confidential	Doc #: TP-80217
Kcas bio	Title: **LC-MS/MS Analysis of Free Zidebactam in Treated Human Plasma Ultrafiltrate (Sodium Heparin, 50 mcL, 0.0500 – 50.0 mcg/mL; Prior to MES Treatment)**	Rev #: **02**
		Page Numbering: **1 / 12**
		Effective Date: **18-Jun-2024**
		Validation #: **V1872106P4**

1. GENERAL INFORMATION

1.1. Client Name: Dynport Vaccine Company

1.2. Reference: Validation V1872106P4

2. TABLE OF CONTENTS

1. GENERAL INFORMATION 1
1.1. Client Name: Dynport Vaccine Company 1
1.2. Reference: Validation V1872106P4 1
2. TABLE OF CONTENTS 1
3. SAMPLE REQUIREMENTS 2
4. METHOD SUMMARY 3
5. SPECIAL HANDLING OF BIOLOGICAL SAMPLES 3
6. DATA COLLECTION AND ANALYSIS SOFTWARE 3
7. INSTRUMENTS, EQUIPMENT, AND CONDITIONS 3
7.1. LC and MS Conditions 4
Table 1: HPLC System 4
7.1.1. Gradient Table 4
7.1.2. Autosampler Settings 5
7.1.3. Mass Spectrometer System 5
8. REAGENTS, STANDARDS, AND SUPPLIES 6
Table 2: Reagents, Standards, and Supplies 6
9. SAFETY 7
10. PROCEDURE 7
10.1. Preparation of Analyte Stock Solutions and Dilutions 7
10.1.1. Zidebactam Primary Solution, 4.00 mg/mL (Prepare on wet ice) 7
10.1.2. Working Internal Standard Solution, ~50 mcg/mL Cefotaxime 7

Serial Number: 9eac9dba-a078-4e76-bd9b-708a05c884ae accessed by Anika Pippin (PIPPINA) on Mon, 24 Jun 2024 23:29:37 GMT.

10.2. Preparation of Other Reagents 7
10.2.1. Mobile Phase A: 10 mM Ammonium Acetate in Deionized Water 7
10.2.2. Mobile Phase B: 95/5 Acetonitrile / 10mM Ammonium Acetate 7
10.2.3. Rinse: 90/10 Acetone/Deionized Water 8
10.2.4. 1 N Sodium Hydroxide (NaOH) in Deionized Water 8
10.2.5. MES (2-(N-Morpholino) Ethanesulphonic Acid Hydrate) Buffer pH 6.4, ~0.1M 8
10.2.6. 5/95 Acetonitrile/Deionized Water 8
10.3. Preparation of Standards and QC Samples 8
10.3.1. Preparation of Standard and QC Spiking Solutions on Wet Ice 8
10.3.2. Treated Human Plasma Ultrafiltrate (Ultrafiltrate: MES Buffer 1:1) 8
10.3.3. Preparation of Standards and QC Samples in Human Plasma Ultrafiltrate 9
10.3.4. Ultrafiltration of Human Plasma Samples using Centrifugal Filters 9
10.4. Extraction Procedure 9
10.5. Dilutions 10
10.6. Calculations 10
11. REFERENCE CHROMATOGRAMS 11
Figure 1: Blank with IS 11
Figure 2: Carryover Blank with IS 11
Figure 3: LLOQ – Zidebactam 0.050 mcg/mL 12
12. REVISION HISTORY 12
Table 3: Revision History 12

3. SAMPLE REQUIREMENTS

Ultrafiltered human sodium heparin plasma samples (treated with 1:1 MES Buffer, pH 6.4, ~0.1M) minimum volume 50 mcL each, for determination of free zidebactam concentration.

Refer to the relevant study protocol for a description of expected samples.

Serial Number: 9eac9dba-a078-4e76-bd9b-708a05c884ae accessed by Anika Pippin (PIPPINA) on Mon, 24 Jun 2024 23:29:37 GMT.

4. METHOD SUMMARY

The method involves 96-well protein precipitation followed by chromatographic separation and tandem MS detection using the Sciex 5000 LC/MS/MS system. The concentration range is 0.0500 – 50.0 mcg/mL in human plasma ultrafiltrate only. Since unknown samples are treated with the MES buffer after collection at a 1:1 ratio, the calibration standard and QC concentrations are based on the ultrafiltrate volume only. Unknown samples do not need to be adjusted for the dilution with MES buffer.

5. SPECIAL HANDLING OF BIOLOGICAL SAMPLES

The method requires no special handling. Please ensure all “Universal Precautions” are taken.

Primaries, spiking solutions, calibration standards and QCs should be prepared on wet ice, and each prepared level should be aliquoted immediately and aliquots should be flash frozen in a dry-ice/acetone bath for ~1 minute and stored in the freezer immediately after flash freezing.

6. DATA COLLECTION AND ANALYSIS SOFTWARE

Description	Version
Watson LIMS™ (Thermo Fisher Scientific)	7.5 or higher
Analyst® (AB Sciex)	1.7.2 or higher

7. INSTRUMENTS, EQUIPMENT, AND CONDITIONS

Equivalent equipment can be substituted, except where noted in Section 7.1. Minor modifications of conditions are permitted to optimize chromatography, except where noted in Section 7.1. Refer to SOP-00073 for allowable minor modifications that do not require validation.

Serial Number: 9eac9dba-a078-4e76-bd9b-708a05c884ae accessed by Anika Pippin (PIPPINA) on Mon, 24 Jun 2024 23:29:37 GMT.

7.1. LC and MS Conditions

Table 1: HPLC System

**System Controller**	**Shimadzu Controller, CBM20A**
Pump	Shimadzu LC-30AD
Autosampler	Shimadzu SIL-30AC
Column Oven	Shimadzu CTO-30A
MS Interface	ESI
Analytical Column	Fortis Technology Ltd. Fortis C18 4.6 x 50mm, 5 µ
Column Guard Cartridge	Security Guard Cartridge AQ C18, 4.0 x 2.0 mm
Column Temperature (set at)	35°C
Mobile Phase A	10 mM Ammonium Acetate in Deionized Water
Mobile Phase B	95/5 Acetonitrile / 10 mM Ammonium Acetate
Needle Wash Solvent (R0, R3)	90/10 Acetone /Deionized Water
Total Run Time (approximate)	7.9 min
Injection Volume (µl)	1-10 mcL
Flow Rate	1.0 mL/min
Pressure, kgf/cm2 (approximate)	~250
Approximate Retention Time (min.)	Zidebactam: 1.80
	Cefotaxime: 3.50

7.1.1. Gradient Table

Time Program

**Time**	**Module**	**Events**	**Parameter**
1.00	Controller	Event 1*	
1.50	Pumps	Pump B Conc.	2
2.50	Pumps	Pump B Conc.	16
3.80	Pumps	Pump B Conc.	100
4.00	Controller	Event 2*	
5.80	Pumps	Pump B Conc.	100
6.00	Pumps	Pump B Conc.	2
7.00	System Controller	Stop	

* Mobile phase flow is directed to the mass spectrometer from 1.00 min (Event 1) to 4.00 min (Event 2). The rest of the gradient time, the mobile phase flow goes to waste.

Serial Number: 9eac9dba-a078-4e76-bd9b-708a05c884ae accessed by Anika Pippin (PIPPINA) on Mon, 24 Jun 2024 23:29:37 GMT.

7.1.2. Autosampler Settings

Temperature:	Set at 4°C
Rinse Type:	External
Rinse Mode:	Before and after aspiration
Rinse Dip Time:	2 sec
Rinse Method:	Rinse Pump Then Port
Rinse Time:	2 sec
Rinsing Speed:	35 µL/sec
Rinsing Volume:	800 µL

7.1.3. Mass Spectrometer System

Mass Spectrometer	Sciex 5000
Source Type:	Turbo spray
Scan Type:	MRM (MRM)
Polarity:	Positive
Resolution Q1:	Unit
Resolution Q3:	Unit

All the below parameters in this section may be adjusted to optimize the instrument conditions.

Zidebactam:

Q1 Mass (Da)	Q3 Mass (Da)	Dwell (msec)
392.028	312.000	250.00
DP	66.00	
CE	21.00	
CXP	16.00	

Cefotaxime (IS):

Q1 Mass (Da)	Q3 Mass (Da)	Dwell (msec)
456.200	324.000	250.00
DP	50.00	
CE	32.00	
CXP	10.00	

Serial Number: 9eac9dba-a078-4e76-bd9b-708a05c884ae accessed by Anika Pippin (PIPPINA) on Mon, 24 Jun 2024 23:29:37 GMT.

Parameter Table:

CAD:	12.00
CUR:	40.00
GS1:	50.00
GS2:	60.00
IS:	5500
TEM:	600
EP:	12

8. REAGENTS, STANDARDS, AND SUPPLIES

Reagents from alternate supplier and equivalent grade can be substituted.

Table 2: Reagents, Standards, and Supplies

Chemical / Grade	Manufacturer/Supplier
Zidebactam	Performa
Cefotaxime Sodium	Sigma Aldrich
MES ([2-(N-Morpholino) ethane sulphonic acid hydrate]) buffer	Sigma
Acetonitrile, HPLC grade	Honeywell
Formic Acid, 98+%	EMD Millipore
Deionized Water	Millipore
Ammonium Acetate ≥ 99.9%	Sigma
Methanol	Honeywell
Acetone	Honeywell
Ultrafiltered Human Sodium Heparin Plasma	BioIVT
Centrifugal Filters	Merck Millipore, Lot: 0000158890
Advantage SiliGuard Coated 2 mL 96 well Polypropylene plate: Square Top, Round Bottom	Analytical Sales and Services, Catalog # 27P687GC
Purple, pre-slit, square well, soft silicone/PTFE Cap Mats	Analytical Sales & Services, Inc., Catalog# 965085

Serial Number: 9eac9dba-a078-4e76-bd9b-708a05c884ae accessed by Anika Pippin (PIPPINA) on Mon, 24 Jun 2024 23:29:37 GMT.

9. SAFETY

Always observe safe laboratory practices. Consult the appropriate SDS before handling any chemical if you are unfamiliar with the proper safety precautions.

10. PROCEDURE

10.1. Preparation of Analyte Stock Solutions and Dilutions

The quantities of analyte solution prepared may be scaled up or down proportionally as needed. Alternate dilution schemes can be used to achieve the appropriate final concentrations of standards, QCs, working internal standard, and system verification solution (SVS). For assignment of expiration dates to analyte stocks and their dilutions, refer to the latest version of the validation report.

10.1.1. Zidebactam Primary Solution, 4.00 mg/mL (Prepare on wet ice)

Check drying conditions prior to use. Weigh approximately 40.0 mg of zidebactam reference standard (correcting for purity and correction factor if necessary) in a polypropylene container and add 10.0 mL of deionized water. Mix well and sonicate if necessary. Store solution at -20°C in a polypropylene container.

10.1.2. Working Internal Standard Solution, ~50 mcg/mL Cefotaxime

Check drying conditions prior to use. Weigh 10.0 mg of cefotaxime reference standard into a glass container. Add 200 mL of deionized water (do not correct for conversion factor and purity). Mix well and sonicate if necessary. Store at or below -20°C in a glass container.

10.2. Preparation of Other Reagents

Prepared quantities of all reagents may be scaled up or down as necessary. Volumes provided are examples. For assignment of expiration dates, refer to SOP-00094.

10.2.1. Mobile Phase A: 10 mM Ammonium Acetate in Deionized Water

Weigh ~3.08 g of ammonium acetate (F.W. 77.08) and transfer to a 4-L volumetric flask. Dilute to volume with deionized water. Mix well and store at room temperature.

10.2.2. Mobile Phase B: 95/5 Acetonitrile / 10mM Ammonium Acetate

Combine 3800 mL of acetonitrile with 200 mL of 10 mM ammonium acetate in a 4-L flask. Mix well and store at room temperature.

Serial Number: 9eac9dba-a078-4e76-bd9b-708a05c884ae accessed by Anika Pippin (PIPPINA) on Mon, 24 Jun 2024 23:29:37 GMT.

10.2.3. Rinse: 90/10 Acetone/Deionized Water

Combine 900 mL of acetone and 100 mL of deionized water in a 1-L flask. Mix well and store at room temperature.

10.2.4. 1 N Sodium Hydroxide (NaOH) in Deionized Water

Weight ~4.00 g of sodium hydroxide (F.W. 40.00) and gradually transfer it to a 100-mL volumetric flask containing ~50 mL of deionized water. Mix to dissolve and wait until it reaches room temperature. Dilute to volume with deionized water and mix well. Store in a polypropylene container at room temperature.

10.2.5. MES (2-(N-Morpholino) Ethanesulphonic Acid Hydrate) Buffer pH 6.4, ~0.1M

Weigh ~4000 mg of MES hydrate (F.W. 195.24) and transfer to a 200-mL volumetric flask containing ~150 mL of deionized water. Dissolve and adjust the pH to 6.4 with 1N NaOH. Dilute to volume with deionized water. Mix well and store refrigerated.

10.2.6. 5/95 Acetonitrile/Deionized Water

Combine 50 mL of acetonitrile and 950 mL of deionized water in a 1-L flask. Mix well and store at room temperature.

10.3. Preparation of Standards and QC Samples

10.3.1. Preparation of Standard and QC Spiking Solutions on Wet Ice

Starting from the zidebactam primary solution (4.00 mg/mL) prepare a series of zidebactam spiking solutions in deionized water on wet ice. The following concentrations are recommended but can be altered as necessary:

For standards: 500, 400, 200, 100, 50.0, 10.0, 1.00, and 0.500 mcg/mL.

For QC spiking solutions: 375, 250, and 1.50 mcg/mL, and 0.500 mcg/mL for the LLOQ QC when needed. Store solutions in polypropylene at -20°C.

10.3.2. Treated Human Plasma Ultrafiltrate (Ultrafiltrate: MES Buffer 1:1)

Add 200 mL of chilled sodium heparin human plasma ultrafiltrate to a polypropylene container containing 200 mL of chilled 0.1M MES Buffer, pH 6.4. Mix well and store at -20°C.

It is recommended to prepare the treated human plasma ultrafiltrate fresh prior to usage.

Serial Number: 9eac9dba-a078-4e76-bd9b-708a05c884ae accessed by Anika Pippin (PIPPINA) on Mon, 24 Jun 2024 23:29:37 GMT.

10.3.3. Preparation of Standards and QC Samples in Human Plasma Ultrafiltrate

Use treated sodium heparin human plasma ultrafiltrate that has been diluted 1:1 with MES buffer, screened for the lack of interference at the retention times of zidebactam and cefotaxime (IS). Dilute each spiking solution by a factor of 20 into treated human plasma ultrafiltrate on wet ice and mix well. For example, combine 0.500 mL of spiking solution and 9.5 mL of treated ultrafiltrate. Since unknown samples are treated with the MES buffer after collection at a 1:1 ratio, the calibration standard and QC concentrations are based on the ultrafiltrate volume only. Unknown samples do not need to be adjusted for the dilution with MES buffer. The prepared concentrations in ultrafiltrate only, not accounting for MES buffer, will be as follows:

For standards: 50.0, 40.0, 20.0, 10.0, 5.00, 1.00, 0.100, and 0.0500 mcg/mL.

For QCs: 37.5, 25.0, and 0.150 mcg/mL, with 0.0500 mcg/mL for the LLOQ QCs when needed. Other concentrations may be prepared, provided they meet the requirements of SOP-00035 and provided they remain within the range of 0.0500 to 50.0 mcg/mL.

Store solutions in polypropylene at -70°C or -20°C.

10.3.4. Ultrafiltration of Human Plasma Samples using Centrifugal Filters

1. Thaw human plasma samples on wet ice. Once samples are thawed, vortex each sample for ~20 seconds.
2. Add ~1 mL of sample into a centrifugal filter. Hold the tube at ~45° with pipette tip touching the tube wall and add sample slowly with even flow to avoid air locking.
3. Cap the filter. The caps should extend no more than 3-4 mm from the top. Further compression can cause prefiltration, which could introduce analytical error.
4. Centrifuge sample at 2000 x g for 30 minutes, this will yield ~75 mcL of filtrate.
5. Add **chilled** 0.1M MES Buffer, pH 6.4, at a 1:1 ratio (Sample Filtrate: MES Buffer). Vortex to Mix.
6. Use the resulting plasma filtrate samples for the extraction procedure in Section 10.4.

10.4. Extraction Procedure

1. Thaw treated human plasma ultrafiltrate samples on wet ice. Once samples are thawed, either vortex the batch as a whole for ~5 minutes followed by vortexing each for 10 seconds, or vortex each sample for ~20 seconds each.
2. Pipet 50 mcL of MES Buffer, pH 6.4 into each well in a 1-mL 96-well plate.
3. Pipet 50 mcL of ultrafiltered human plasma sample or blank to the corresponding wells of the 1-mL 96-well plate.

Serial Number: 9eac9dba-a078-4e76-bd9b-708a05c884ae accessed by Anika Pippin (PIPPINA) on Mon, 24 Jun 2024 23:29:37 GMT.

4. Add 50 mcL of working IS (50 mcg/mL cefotaxime in deionized water) to all applicable wells. For **Control Blanks,** add 50 mcL of deionized water instead.
5. Seal the plate with an adhesive pad (or equivalent) and vortex at 1300 rpm for 5 minutes using a plate shaker.
6. Add 200 mcL of acetonitrile to all wells. Seal the plate with an adhesive pad (or equivalent) and vortex for 10 minutes at 1200 rpm.
7. Centrifuge the plate for 10 minutes at a setting of 4000 rpm (ambient conditions).
8. Transfer 200 mcL of the supernatant to a new 1-mL 96-well plate using a multichannel pipette (or equivalent).
9. Evaporate the sample using a 96-well SPE dryer at 37°C at 60-80 psi. This generally takes around 60 minutes.
10. Reconstitute the plate with 150 mcL of 5/95 acetonitrile/deionized water in each well. Seal the plate with a cap mat.
11. Vortex the final plate at 1300 rpm for 5 minutes using a plate shaker.
12. Refrigerate prior to LC-MS injection according to the conditions outlined in Section 7.

10.5. Dilutions

2x = 25 mcL of sample + 25 mcL of blank treated human plasma ultrafiltrate

10x = 20 mcL of sample + 180 mcL of blank treated human plasma ultrafiltrate, mix well and use 50 mcL for analysis

10.6. Calculations

Linear regression is performed with a 1/x2 weighting factor.

Equation: $y = a + bx$

Where y = analyte/internal standard area ratio

x = concentration of analyte (mcg/mL)

b = Slope

a = Intercept

Serial Number: 9eac9dba-a078-4e76-bd9b-708a05c884ae accessed by Anika Pippin (PIPPINA) on Mon, 24 Jun 2024 23:29:37 GMT.

11. REFERENCE CHROMATOGRAMS

Figure 1: Blank with IS

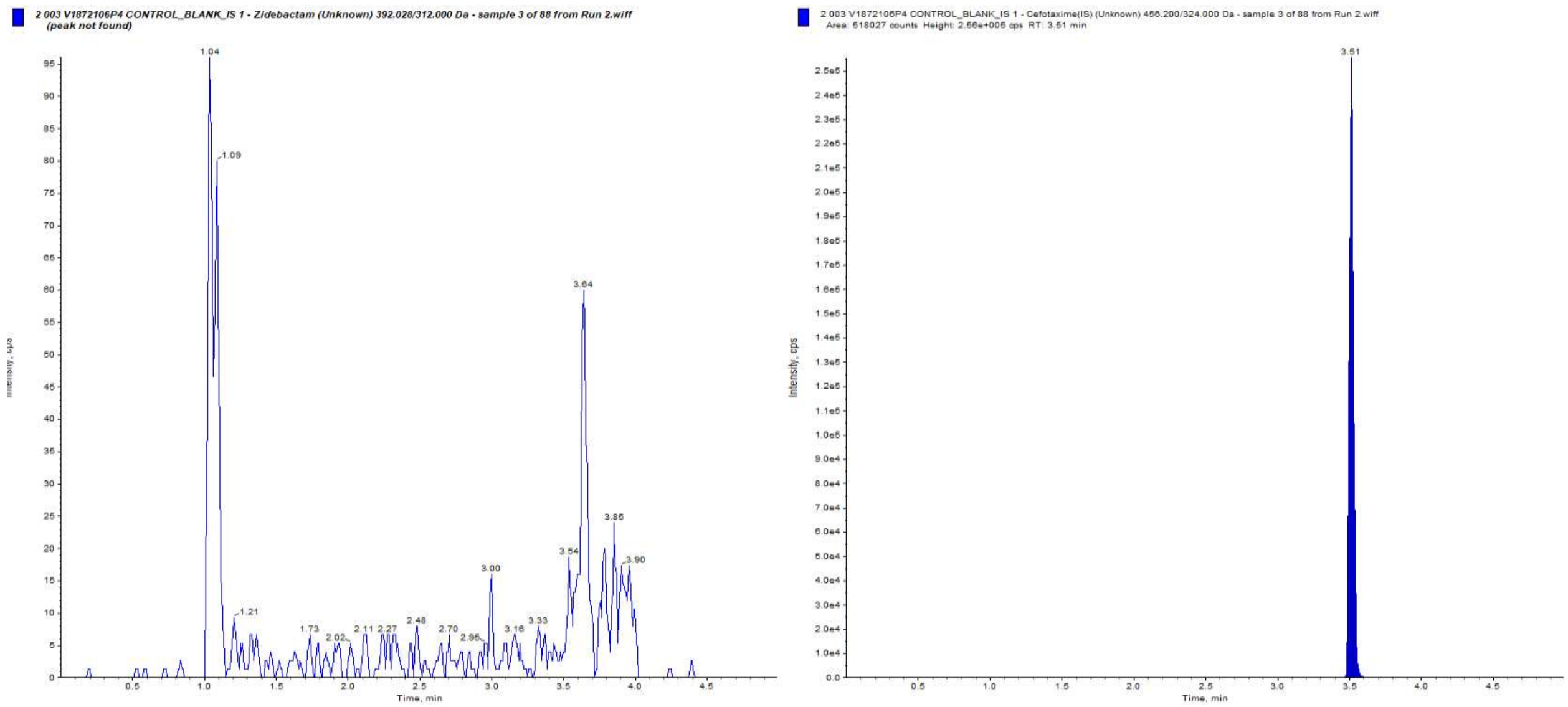

Figure 2: Carryover Blank with IS

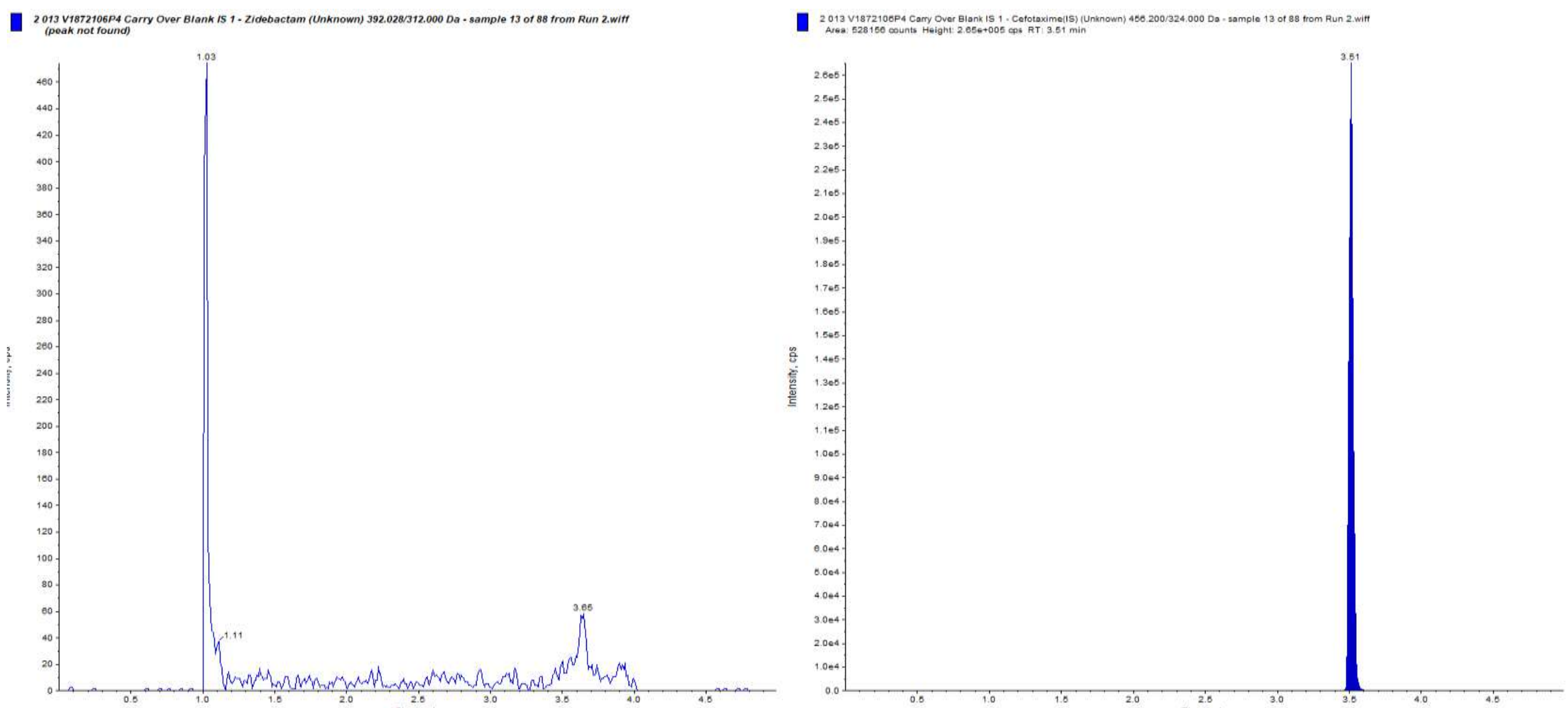

Serial Number: 9eac9dba-a078-4e76-bd9b-708a05c884ae accessed by Anika Pippin (PIPPINA) on Mon, 24 Jun 2024 23:29:37 GMT.

Figure 3: LLOQ – Zidebactam 0.050 mcg/mL

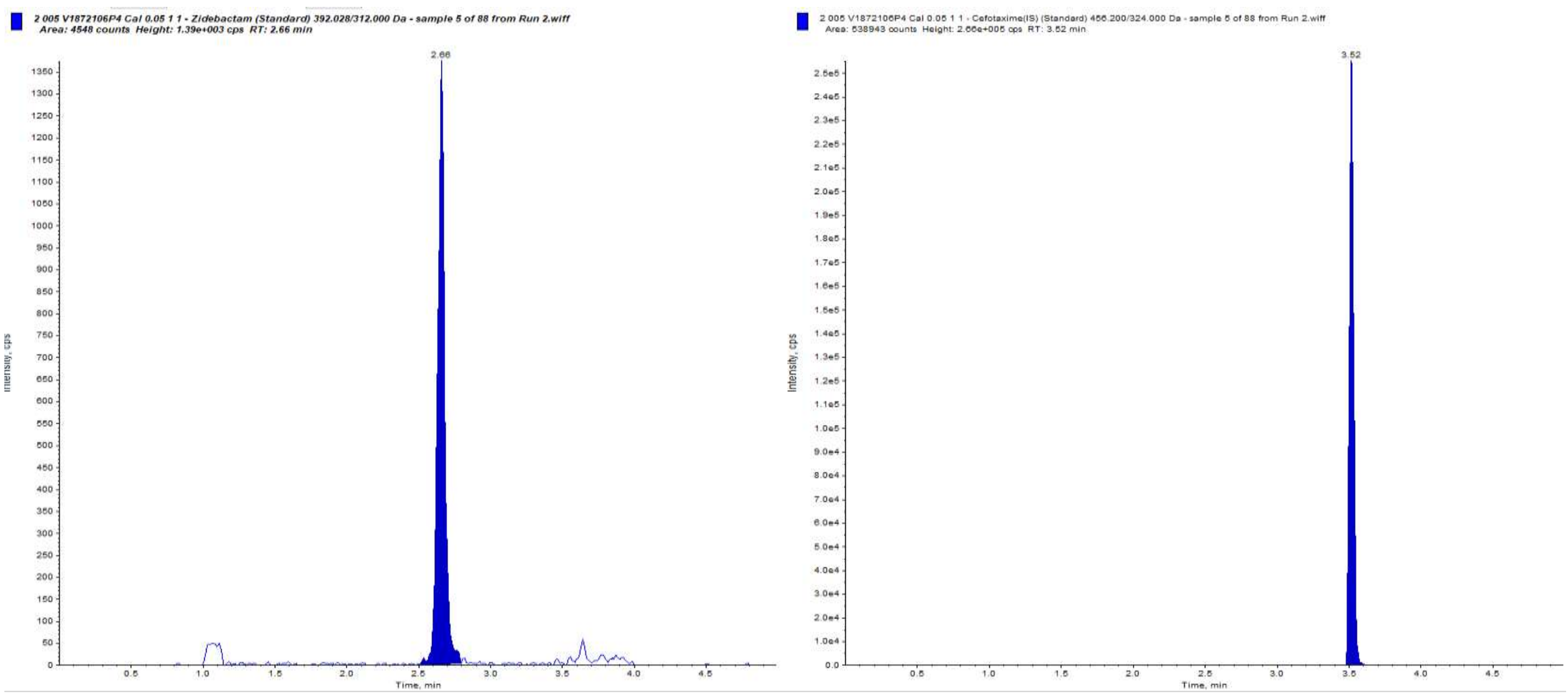

12. REVISION HISTORY

Table 3: Revision History

Revision	Effective Date	Section	Revision Summary
02	See Header	Throughout	Updated template and added header with new company logo.
		10.6	(Calculations) Calculations were updated to reflect Linear Regression. Additional validation work for this update has been completed.
01	19 Oct 2023	10.3.4	(Ultrafiltration of Human Plasma Samples using Centrifugal Filters) Added a step for clarification to add MES buffer to filtered samples after filtration as the collected samples are not treated and are treated at KCAS. Clarified filtrate volume prior to MES buffer addition. These adjustments were made to clarify actual sample collection. No additional work is needed since sample composition is not changing.
00	25 Nov 2022	NA	New Procedure

Serial Number: 9eac9dba-a078-4e76-bd9b-708a05c884ae accessed by Anika Pippin (PIPPINA) on Mon, 24 Jun 2024 23:29:37 GMT.

TP-80217 [02]: V1872106P4 - Free Zidebactam in Treated Human Plasma Ultrafiltrate

All blank fields are considered as data not available. All Signature (Date and Time) shown in this report are in GMT.

**Type**	TP	**Status**	CURRENT
**Effective Date**	18-Jun-2024	**Periodic Review Date**	05-Mar-2051
**Owner**	PARMENTIERB	**Department Name**	Pharma LC-MS/MS Pharma LC-MS/MS
**Description**	Full Title: LC-MS/MS Analysis of Free Zidebactam in Treated Human Plasma Ultrafiltrate (Sodium Heparin, 50 mcL, 0.0500 – 50.0 mcg/mL; Prior to MES Treatment)		

Revision Notes (5)

Task Name	Action	Comment	Signature
Revise Document	Original Document Revision : 01	Revision requested by Anika P.	NEVINSC 17-Jun-2024 14:35:53 [GMT]
Check Out	Document Profile Preparation	Revision requested by Anika P.	NEVINSC 17-Jun-2024 14:36:21 [GMT]
Check In	Document Profile Preparation	Edits made outside of the system.	NEVINSC 17-Jun-2024 20:50:36 [GMT]
Check Out	Document Finalization	Correct hyperlink.	NEVINSC 17-Jun-2024 20:58:49 [GMT]
Check In	Document Finalization	Corrected hyperlink.	NEVINSC 17-Jun-2024 20:59:15 [GMT]

Approval Information

Note to Approver

Reviewer: Bryan Parmentier
Management: Marsha Luna
Author & PI: Anika Pippin

Approval Decision	Comment	Assigned Role	Approved By	Signature
Approve		Document Approver	Bryan Parmentier	PARMENTIERB<br>17-Jun-2024 21:28:13 [GMT]
Approve		Document Approver	Marsha Luna	LUNAM<br>17-Jun-2024 21:38:29 [GMT]
Approve		Document Approver	Anika Pippin	PIPPINA<br>18-Jun-2024 20:23:49 [GMT]

Serial Number: 9eac9dba-a078-4e76-bd9b-708a05c884ae accessed by Anika Pippin (PIPPINA) on Mon, 24 Jun 2024 23:29:37 GMT.

1. TITLE: THE DETERMINATION OF FREE ZIDEBACTAM IN TREATED HUMAN PLASMA ULTRAFILTRATE, WITH SODIUM HEPARIN AS ANTICOAGULANT, BY LC-MS/MS

BIOANALYTICAL METHOD VALIDATION REPORT

Bioanalytical Project Number: V1872106P4

Sponsor: DynPort Vaccine Company, LLC
110 Thomas Johnson Drive
Suite 200
Frederick, MD 21702

Bioanalytical Laboratory: KCAS Bioanalytical & Biomarker Services.
10830 S Clay Blair Boulevard
Olathe, KS 66061

Issue Date: February 2023

Amendment 1 Issue Date: Date of PI Signature

2. SYNOPSIS

This report presents the validation data for an LC-MS/MS method for the determination of free zidebactam in treated human plasma ultrafiltrate, with sodium heparin as anticoagulant to support the Phase 1 clinical trial studying the safety, tolerability, and pharmacokinetics of intravenous zidebactam in combination with ertapenem (WCK 6777) in healthy adult subjects. The assay was analyzed in the range of 0.0500 – 50.0 mcg/mL and is for use on treated human plasma ultrafiltrate samples obtained during clinical trials.

AMENDMENT OUTLINE FORM

1. Amendment Number 1	This amendment was created to report:<br>• Long-term matrix stability at -70 °C for up to 201 days for free zidebactam in treated human plasma ultrafiltrate, with sodium heparin as anticoagulant with and without the presence of comed[2].

3. TABLE OF CONTENTS FOR THE BIOANALYTICAL METHOD VALIDATION REPORT

1. TITLE: THE DETERMINATION OF FREE ZIDEBACTAM IN TREATED HUMAN PLASMA ULTRAFILTRATE, WITH SODIUM HEPARIN AS ANTICOAGULANT, BY LC-MS/MS 1
2. SYNOPSIS 2
3. TABLE OF CONTENTS FOR THE BIOANALYTICAL METHOD VALIDATION REPORT 3
4. LIST OF TABLES 4
5. SIGNATURE PAGE 5
6. QUALITY ASSURANCE STATEMENT 6
7. STUDY COMPLIANCE STATEMENT 7
8. LIST OF ABBREVIATIONS AND DEFINITIONS OF TERMS 8
9. INTRODUCTION 10
10. EXPERIMENTAL DESIGN AND RESULTS 10
10.1. Reference Standards 10
10.2. Calibration Standards and QC Samples Preparation 10
10.2.1. Carryover 11
10.3. Long-Term Matrix Stability at -70 °C 11
10.4. SOP Deviations and Investigations 11
11. CONCLUSION 12
12. PERTINENT SOPS 12
13. ARCHIVES 12
14. REFERENCES 12
15. TABLES 13

4. LIST OF TABLES

Table 1: Abbreviations and Codes 8
Table 2: Deviations 11
Table 3: Mass Spec Transitions 13
Table 4: Reference Standards 13
Table 5: Calibration Standards and QC Samples Preparation 14
Table 6: Matrix Description 15
Table 7: Summary of Results and Conclusions 16
Table 8: Summary of Validation Run Objectives and Outcome 19
Table 9: Carryover 20
Table 10: Long-Term Stability at -70 °C 21

Annex 3 .... Certificate(s) of Analysis

5. SIGNATURE PAGE

Bioanalytical Project Number: V1872106P4

Amendment 1 Issue Date: Date of PI Signature

APPROVED

DocuSigned by:
Anika Pippin
Signer Name: Anika Pippin
Signing Reason: I approve this document
Signing Time: 15-Jun-2023 | 17:38 CDT
BE3AA1198E40459E8A7E3C615D981D82

Anika Pippin, B.S.
Principal Investigator

REVIEWED

DocuSigned by:
Marsha Luna
Signer Name: Marsha Luna
Signing Reason: I have reviewed this document
Signing Time: 15-Jun-2023 | 15:50 CDT
C9CC738561AF4CC1B375ED013DD1E4BD

Marsha Luna, B.S.
Management

6. QUALITY ASSURANCE STATEMENT

This Bioanalytical Method Validation Report has been found to describe accurately the methods and SOPs used and to accurately reflect the raw data collected. The Quality Assurance Unit initiated audits on this study and reported the findings to the Principal Investigator (PI) and Management as follows:

		Date(s) Sent to:	
**Inspection Date(s)**	**Inspection Phase**	**PI**	**Management**
17Mar2023	Data Audit	17Mar2023	15May2023
23May2023	Data Audit	23May2023	23May2023
08Jun2023	Data Audit	08Jun2023	12Jun2023
09Jun2023	Draft Report Review	09Jun2023	09Jun2023
13Jun2023	Final Report Review	13Jun2023	13Jun2023

REVIEWED

DocuSigned by:
Monica Pirez
Signer Name: Monica Pirez
Signing Reason: I have reviewed this document
Signing Time: 15-Jun-2023 | 15:49 CDT
518486CF3F7A41039BB7811A9B22618B

Monica Pirez, B.S.
Quality Assurance Unit

7. STUDY COMPLIANCE STATEMENT

I, the undersigned, hereby declare that this study was conducted in compliance with the relevant bioanalytical aspects of FDA Guidance for Industry on Bioanalytical Methods Validation, May 2018 [1] and KCAS Bioanalytical & Biomarker Services (KCAS) procedures and that the work was performed by me, or under my direction, and the report reflects a true and accurate record of the results obtained.

The acceptance criteria are based on the FDA Guidance for Industry, Bioanalytical Method Validation (2018). [1]

APPROVED

DocuSigned by:

Anika Pippin

Signer Name: Anika Pippin
Signing Reason: I approve this document
Signing Time: 15-Jun-2023 | 17:38 CDT
BE3AA1198E40459E8A7E3C615D981D82

Anika Pippin, B.S.
Principal Investigator

8. LIST OF ABBREVIATIONS AND DEFINITIONS OF TERMS

Table 1: Abbreviations and Codes

Abbreviation	Definition
<	Concentration less than the lowest acceptable calibration standard
>	Concentration exceeded the upper limit of calibration range (> ULOQ)
A	Amendment
AQL	Above Quantitation Limit
%Bias	Percentage measurement valid
Batch	Is the same as Run
BQL	Below Quantifiable Limit
Cal	Calibration standard
Conc.	Concentration
Conv.	Conversion
%CV	Coefficient of Variation
dev	Deviation
diff	Difference
EMA	European Medicines Agency
FDA	Food and Drug Administration
GLP	Good Laboratory Practice
HPLC	High Performance Liquid Chromatography
ID	Identification
IS	Internal Standard
ISR	Incurred Sample Reanalysis
K_2EDTA or K_3EDTA	Dipotassium or Tripotassium Ethylenediaminetetraacetic Acid
KCAS	KCAS Bioanalytical & Biomarker Services
LC-MS/MS	Liquid Chromatography Tandem Mass Spectrometry
LLOQ	Lower Limit of Quantitation
mcg or µg or ug	Microgram
mcL or µL or uL	Microliter

Table 1: Abbreviations and Codes (Continued)

Abbreviation	Definition
ME	Matrix Effect
MF	Matrix Factor
MHLW	Ministry of Health, Labour and Welfare of Japan
mL	Milliliter
MS	Mass Spectrometry
n	number
N.C.	Not Calculated
ND	No difference reported as ISR will be counted
ng	nanogram
n.report	No Data available to report
N/A or NA	Not Applicable
N.R.	Not Reported
P&A	Precision and Accuracy
p.a.r.	Peak Area Ratio
PI	Principal Investigator
QC	Quality Control Sample
QCs	Quality Control Samples
OECD	Organization for Economic Co-operation and Development
RT	Room Temperature
sd or SD	Standard deviation
Seq	Sequence
SOP (s)	Standard Operating Procedure (s)
temp	Temperature
TP	Test procedure
UHPLC	Ultra High Performance Liquid Chromatography
ULOQ	Upper Limit of Quantitation
WIS	Working Internal Standard

This is a basic list. Some of the abbreviations may not have been used in this report.

9. INTRODUCTION

This amendment was created to report long-term matrix stability at -70 °C for up to 201 days for free zidebactam in treated human plasma ultrafiltrate, with sodium heparin as anticoagulant with and without the presence of comed[2].

The validation procedure was designed to meet or exceed the requirements of FDA Guidance for Industry on Bioanalytical Methods Validation, May 2018. The software programs used were Microsoft Office Excel 365, acquisition and processing software Analyst 1.7.2, and data management software Watson LIMS 7.5.

10. EXPERIMENTAL DESIGN AND RESULTS

In this method, free zidebactam and internal standards, cefotaxime, were released from treated human plasma ultrafiltrate using 96 well protein precipitation. After extraction, the sample was chromatographed using reversed phase HPLC with a Fortis C18 analytical column. Zidebactam and cefotaxime (IS) were detected by monitoring the precursor and product ions described in Table 3 using an Applied Biosystems Sciex API 5000 LC-MS/MS. The quantification was based on setting a calibration graph using the internal standard method based on bioanalytical test procedure TP-80217, entitled "LC-MS/MS Analysis of Free Zidebactam in Treated Human Plasma Ultrafiltrate (Sodium Heparin, 50 mcL, 0.0500 – 50.0 mcg/mL; Prior to MES Treatment)". The test procedure can be found in Annex 1 (V1872106P4).

Human plasma, screened for the lack of interference at the retention times of zidebactam and cefotaxime (IS), was diluted 1:1 with MES buffer, pH 6.4 for stabilization. Since the standards and QCs concentrations were already adjusted for the addition of MES during the preparation, the obtained free zidebactam concentration of the unknown study samples do not need to be adjusted for the dilution with MES buffer.

A summary of the validation results is provided in Table 7. A summary describing the objective(s) and outcome of each validation run attempted is provided in Table 8.

The method was validated within the analytical range of 0.0500 – 50.0 mcg/mL in treated human plasma ultrafiltrate. No development work was performed after the method was validated.

10.1. Reference Standards

Reference standards used during this validation are presented in Table 4.

10.2. Calibration Standards and QC Samples Preparation

Calibration standards and quality control pools were prepared by spiking appropriate levels of zidebactam into human plasma ultrafiltrate, with sodium heparin as anticoagulant. Primary reference standards were prepared in at least duplicate and compared within 5% of each other. Standards and QCs were prepared and stored frozen at KCAS at -70 °C. Standards, QCs, and the

critical solutions used during this validation are described in Table 5, and matrix used is described in Table 6.

10.2.1. Carryover

Carryover is evaluated within each run. It consists of measuring the response for each analyte and if it exceeds 20.0% of the response of LLOQ standard or other sample reflecting the lower sensitivity limit required, a carryover assessment is completed, and affected samples are rejected. The internal standard carryover evaluation is assessed within each run and if it exceeds 5.0% of the average internal standard response of acceptable standards and QCs, impact is evaluated within the run. Data is presented in Table 9.

10.3. Long-Term Matrix Stability at -70 °C

To evaluate free zidebactam in human plasma ultrafiltrate with and without the presence of comed[2], with sodium heparin as anticoagulant, stability during sample storage, aliquots of the high and low concentration QC pools were stored frozen at -70 °C nominally for 112, 126 (without comed[2] only), and 201 days prior to being analyzed, along with freshly prepared standards and QC samples. To be deemed stable, the mean %Bias at each level must be within ± 15.0% with %CV ≤ 15.0%.

Data are provided in Table 10.

See Section 10.4 for reason for run 1 stability failure.

10.4. SOP Deviations and Investigations

One process deviation occurred during this study. No investigations were conducted during this study.

Table 2: Deviations

Deviation #	Description	Impact
DEV-2023-0104	In run 1 the comed LTS meets criteria, but the regular LTS shows a high %Bias at the low level (16.7% bias). The experiment was repeated in run 2 with passing results (10.7% bias).<br>The high failure was attributed to human error during preparation or extraction (pipetting sample) and the repeat run shows passing data, further showing that this was a preparation error. This deviation was written as documentation to fulfill contract requirements for 1872106, no deviation occurred.	There is no impact.

11. CONCLUSION

Free zidebactam in human plasma ultrafiltrate with and without the presence of comed[2], with sodium heparin as anticoagulant, was proven to be stable at -70 °C over a period of 201 days in polypropylene tubes.

12. PERTINENT SOPS

The following SOPs were followed during the conduct of this study:

SOP-00073 [04] - Bioanalytical Method Validation - Chromatographic Assays

SOP-00035 [02] - Procedures, Reporting, and Acceptance Criteria for Bioanalytical Chromatographic Projects

13. ARCHIVES

All raw data, including paper and electronic files associated with this study will remain archived at a location specified by KCAS, 10830 S Clay Blair Boulevard, Olathe, KS 66061 for a period of at least 10 years, after which the client may be contacted to arrange for continued storage of the files, or disposition according to an agreed procedure. KCAS will maintain an electronic (scanned/true) copy of the method validation report that can be viewed and printed on site. The study plan, documentation, raw data, and final report are indexed under Bioanalytical Project Number V1872106P4A1.

14. REFERENCES

1. Guidance for Industry: Bioanalytical Method Validation. U.S. Department of Health and Human Services. Food and Drug Administration. Center for Drug Evaluation and Research. Center for Veterinary Medicine. May 2018.
2. Comeds: QCs in the presence of 150 mcg/mL ertapenem.

15. TABLES

Table 3: Mass Spec Transitions

Analyte	Transition
Zidebactam	m/z 392.028→312.000
Cefotaxime (IS)	m/z 456.200→324.000

Table 4: Reference Standards

Analyte	Supplier	Lot/Batch Number	Purity	Exp. Date or Retest Date	Conversion Factor	Storage Temp.
Zidebactam	Wockhardt	WK1/WP-0060/016	90.4%	Exp. 01 Mar 2024	1.000	2-8 °C
			90.4%	Exp. 29 Mar 2023		
Cefotaxime Sodium Salt (IS)	Sigma-Aldrich	0000146942	95.9%	Retest 30 Sep 2023	1.048	2-8 °C
Ertapenem Sodium	Med Chem Express	180017	98.9%	Retest 11 Aug 2024	1.046	2-8 °C

Certificates of analysis for the above reference standards are presented in Annex 3.

Table 5: Calibration Standards and QC Samples Preparation

<table>
<tr><th>Critical Solutions</th><th>Lot # for Standards (Prep Date)</th><th>Lot # for QCs (Prep Date)</th><th>Standards Lot (Prep Date)</th><th>QCS Lot (Prep Date)</th><th>Runs used</th></tr>
<tr><td>Sodium Heparin Human Plasma Ultrafiltrate</td><td colspan="2">BioIVT, Lot V1872106P4-8-1</td><td rowspan="2">N/A</td><td rowspan="2">V1872106P4-10-1
Set A
(28 Oct 2022)</td><td rowspan="2">1, 2, 3
(LTS)</td></tr>
<tr><td>Deionized water stored at -20°C in polypropylene</td><td>N/A</td><td>V1872106P4-1-1
(27 Oct 2022)
V1872106P4-2-1
(27 Oct 2022)
V1872106P4-5-1
(27 Oct 2022)</td></tr>
<tr><td>Sodium Heparin Human Plasma Ultrafiltrate</td><td colspan="2">BioIVT, Lot V1872106P4-8-1</td><td rowspan="3">NA</td><td rowspan="3">V1872106P4-11-1
Set A
Comed QCs
(28 Oct 2022)</td><td rowspan="3">1, 3
(LTS)</td></tr>
<tr><td>Deionized water stored at -20°C in polypropylene</td><td rowspan="2">NA</td><td>V1872106P4-2-1
(27 Oct 2022)
V1872106P4-5-1
(27 Oct 2022)</td></tr>
<tr><td>Ertapenem in deionized water stored at -20 °C in glass</td><td>V1872106U1-54-1
(12 Oct 2022)</td></tr>
<tr><td>Sodium Heparin Human Plasma Ultrafiltrate</td><td colspan="2">BioIVT, Lot V1872106P4A1-2-2</td><td rowspan="2">V1872106P4A1-1-1
Set A
(17 Feb 2023)</td><td rowspan="2">V1872106P4A1-2-1
Set A
(17 Feb 2023)</td><td rowspan="2">1</td></tr>
<tr><td>Deionized water stored at -20°C in polypropylene</td><td>V1872106P2-120-1
(02 Feb 2023)
V1872106P2-122-1
(02 Feb 2023)</td><td>V1872106P2-121-1
(02 Feb 2023)
V1872106P2-123-1
(02 Feb 2023)</td></tr>
<tr><td>Sodium Heparin Human Plasma Ultrafiltrate</td><td colspan="2">BioIVT, Lot V1872106P4A1-3-1</td><td rowspan="2">V1872106P4A1-4-1
Set B
(03 Mar 2023)</td><td rowspan="2">V1872106P4A1-3-2
Set B
(03 Mar 2023)</td><td rowspan="2">2</td></tr>
<tr><td>Deionized water stored at -20°C in polypropylene</td><td>V1872106P2-120-1
(02 Feb 2023)
V1872106P2-122-1
(02 Feb 2023)</td><td>V1872106P2-121-1
(02 Feb 2023)
V1872106P2-123-1
(02 Feb 2023)</td></tr>
<tr><td>Sodium Heparin Human Plasma Ultrafiltrate</td><td colspan="2">BioIVT, Lot V1872106P4A1-11-2</td><td rowspan="2">V1872106P4A1-11-1
Set C
(17 May 2023)</td><td rowspan="2">V1872106P4A1-12-1
Set C
(17 May 2023)</td><td rowspan="2">3</td></tr>
<tr><td>Deionized water stored at -20°C in polypropylene</td><td>V1872106P4A1-5-1
(15 May 2023)
V1872106P4A1-7-1
(15 May 2023)</td><td>V1872106P4A1-6-1
(15 May 2023)
V1872106P4A1-8-1
(15 May 2023)</td></tr>
</table>

Table 6: Matrix Description

Matrix	Source Lot #	Date Received
Sodium Heparin Human Plasma Ultrafiltrate Lot V1872106P4-8-1 (Combo-70-1) and V1872106P4A1-2-2 (Combo-70-1)	BioIVT, Lots HMN790515, HMN790517, HMN790518, HMN790520, HMN790521, HMN790523	Apr 2022
Sodium Heparin Human Plasma Ultrafiltrate Lot V1872106P4A1-3-1	BioIVT, Lots HMN790518	Apr 2022
Sodium Heparin Human Plasma Ultrafiltrate Lot V1872106P4A1-11-2	BioIVT, Lot HMN1086396	Mar 2023

Table 7: Summary of Results and Conclusions

**Methodology – TP-80217**	**Method Performance – LC-MS/MS**	**Hyperlink – N/A**	**Comments**
Matrix	Plasma ultrafiltrate		
Anticoagulant	Sodium heparin		
Assay Volume	50 mcL		
**Selectivity**			
Analyte	Zidebactam		V1872106P4
Internal Standard	Cefotaxime		V1872106P4
Matrix Effect<br>(6 spiked matrix lots)	Complies		V1872106P4
Interference	Complies		V1872106P4
Hemolytic Effect Evaluation	Complies		V1872106P4
Lipemic Effect Evaluation	Complies		V1872106P4
Carryover	Assessed with every run with standards and QCs	Summary tables<br>Table 9<br><br>Report text<br>Section 10.2.1	
**Linearity**			
Weighting	$1/x^2$		V1872106P4
Bias at LLOQ	0.8%		V1872106P4
Bias above LLOQ	-2.6 to 2.0%		V1872106P4
Bioanalytical Range	Complies<br>0.0500 – 50.0 mcg/mL		V1872106P4
**Precision**			
Intra-assay	3.2 to 9.4% (LLOQ);<br>3.5 to 9.9% (above LLOQ)		V1872106P4
Inter-assay	6.1% (LLOQ);<br>5.4 to 6.3% (above LLOQ)		V1872106P4
**Accuracy**			
Intra-assay	8.4 to 9.2% (LLOQ);<br>1.2 to 11.3% (above LLOQ)		V1872106P4
Inter-assay	9.0% (LLOQ);<br>2.4 to 6.7% (above LLOQ)		V1872106P4
**Large Run Size**	Determined to be 158 samples.		V1872106P4
**Dilution**			
Dilution factor: 2x	Dilution QC: 37.5 mcg/mL<br>Precision: 3.0%<br>Accuracy: 3.5%		V1872106P4

Table 7: Summary of Results and Conclusions (Continued)

Methodology – TP-80217	Method Performance – LC-MS/MS	Hyperlink – N/A	Comments
Dilution factor: 10x	Dilution QC: 400 mcg/mL<br>Precision: 6.7%<br>Accuracy: 1.5%		V1872106P4
**Stability**			
Matrix (in polypropylene tubes)			
Freeze/Thaw (-20 °C/wet ice) with and without the presence of comeds[2]	Demonstrated for 3 cycles		V1872106P4
Freeze/Thaw (-70 °C/wet ice) with and without the presence of comeds[2]	Demonstrated for 3 cycles		V1872106P4
Bench-top stability in wet ice with and without the presence of comeds[2]	Demonstrated for 24 hours		V1872106P4
Long-term (-20 °C) with and without the presence of comeds[2]	Demonstrated instability at 39 days		V1872106P4
Long-term (-70 °C) with and without the presence of comeds[2]	Demonstrated for 201 days	Summary tables<br>Table 10<br><br>Report text<br>Section 10.3	
Processed Matrix polypropylene			
Refrigeration	Demonstrated for 5 day 21 hours		V1872106P4
**Individual Sample Reinjection**	Reproducible		V1872106P4
**Whole Run Reinjection Integrity**	Reproducible<br>Duration 6 days 20 hours		V1872106P4
**Column Ruggedness**	Reproducible		V1872106P4
**Extraction Recovery**			
Zidebactam	Overall 109.3%		V1872106P4
Cefotaxime (IS)	Overall 115.7%		V1872106P4
**Solution Stability**			
Solution, Primary polypropylene or glass			
Zidebactam in deionized water (RT) - glass	Stable 17.75 hours		V1872106U2
Zidebactam in deionized water (-20 °C) - glass	Stable 25 days<br>Stable 61 days		V1872106U2
Zidebactam in deionized water (-20 °C) - polypropylene	Stable 39 days		V1872106P4

Table 7: Summary of Results and Conclusions (Continued)

**Methodology – TP-80217**	**Method Performance – LC-MS/MS**	**Hyperlink – N/A**	**Comments**
Solution, Working polypropylene or glass			
Zidebactam Spike in deionized water (RT) - polypropylene	Stable 17.75 hours		V1872106P4
Zidebactam Spike in deionized water (-20 °C) - glass	Stable 25 days<br>Stable 61 days (high QC only)		V1872106P4
Cefotaxime (IS) in deionized water in glass (RT) - glass	Stable 15.5 hours		V1872106P4

Table 8: Summary of Validation Run Objectives and Outcome

Run ID	Instrument ID	Extraction Date	Assay Date	Run Objective(s)	Accepted or Rejected	Comment
1	LC/MS-39	17-Feb-2023	18-Feb-2023	Long-term matrix stability with and without comed[2]	Accepted	[a]
2	LC/MS-39	03-Mar-2023	04-Mar-2023	Long-term matrix stability without comed[2]	Accepted	
3	LC/MS-25	17-May-2023	17-May-2023	Long-term matrix stability with and without comed[2]	Accepted	

Run 4 was used for a spike linearity check and primary comparison and is not reported.

[a]LTS -70 °C failed %Bias criteria slightly high. This doesn't suggest instability. The experiment was repeated since the high bias may be due to an extraction error. See Section 10.4 for more details.

Table 9: Carryover

Run ID	Result (Extracted Blank after High Standard)	Carryover Results for Internal Standard
1	Seq 13 and Seq 56 are <20.0% of the LLOQ.	Seq 4 and Seq 47 are < 5.0% internal standard peak of acceptable standards.
2	Seq 13 is 25.5% of the LLOQ and Seq 44 is <20.0% of the LLOQ.	Seq 4 and Seq 35 are < 5.0% internal standard peak of acceptable standards.
3	Seq 13 and Seq 56 are <20.0% of the LLOQ.	Seq 4 and Seq 47 are < 5.0% internal standard peak of acceptable standards.

Note: Calibration Standards and QCs are arranged in a way to mitigate carryover in all validation runs.

Carryover is evaluated within each run. It consists of measuring the response for each analyte and if it exceeds 20% of the response of LLOQ standard or other sample reflecting the lower sensitivity limit required, a carryover assessment is completed, and affected samples are rejected. The internal standard carryover evaluation is assessed within each run and if it exceeds 5% of the average internal standard response of acceptable standards and QCs, impact is evaluated within the run.

Carryover evaluation of the internal standard are within 5% in each run. Calibration Standards and QCs are arranged in a way to mitigate carryover in all validation runs, and no QCs was deactivated due to carry-over assessments.

Table 10: Long-Term Stability at -70 °C

Analytical Performance: Back-Calculated Concentrations (µg/mL) of Zidebactam Calibration Standard in (Human) (Plasma Ultrafiltrate) in (Study V1872106P4 A1)

Assay Date	Run ID	Cal 0.05 0.0500 µg/mL	Cal 0.1 0.100 µg/mL	Cal 1 1.00 µg/mL	Cal 5 5.00 µg/mL	Cal 10 10.0 µg/mL	Cal 20 20.0 µg/mL	Cal 40 40.0 µg/mL	Cal 50 50.0 µg/mL
18-Feb-2023	1	0.0548	0.0921	0.944	4.90	10.4	19.4	39.9	51.2
		0.0496	0.0902	1.04	5.25	10.4	21.0	39.3	48.4
04-Mar-2023	2	*0.0337	*0.0758	0.925	5.11	10.3	20.5	37.4	50.4
		0.0532	0.0872	1.06	5.03	9.18	22.7	43.6	45.9
17-May-2023	3	0.0483	0.0968	1.01	5.17	10.4	20.3	41.0	50.9
		0.0543	0.0925	0.993	4.97	10.1	19.3	39.4	48.6
Mean		0.0520	0.0918	0.995	5.07	10.1	20.5	40.1	49.2
S.D.		0.00292	0.00351	0.0529	0.130	0.480	1.25	2.07	2.01
%CV		5.6	3.8	5.3	2.6	4.8	6.1	5.2	4.1
%Bias		4.0	-8.2	-0.5	1.4	1.0	2.5	0.3	-1.6
n		5	5	6	6	6	6	6	6
Reason Deactivated									
* Excluded due to being outside acceptance criteria									

Calibration Curve Parameters for Zidebactam Calibration Standards in Human Plasma Ultrafiltrate (Study V1872106P4 A1)

Run Date	Run ID	A	B	C	R-Squared	LLOQ	ULOQ
18-Feb-2023	1	0.00024	0.20366	-0.00180	0.9967	0.0500	50.0
04-Mar-2023	2	-0.00002	0.09024	0.00101	0.9937	0.0500	50.0
17-May-2023	3	-0.00003	0.09702	0.00052	0.9982	0.0500	50.0

Human Plasma Ultrafiltrate Zidebactam Concentrations in Quality Controls for 3 separate batch runs (Study V1872106P4 A1)

Run Date	Run ID	QC 0.15 0.150 µg/mL	%Bias	QC 25 25.0 µg/mL	%Bias	QC 37.5 37.5 µg/mL	%Bias
18-Feb-2023	1	0.148	-1.3	23.4	-6.4	36.8	-1.9
		0.142	-5.3	25.8	3.2	36.5	-2.7
Intrarun Mean		0.145		24.6		36.7	
Intrarun %Bias		-3.3		-1.6		-2.1	
04-Mar-2023	2	#0.127	-15.3	27.0	8.0	38.2	1.9
		0.130	-13.3	26.1	4.4	38.2	1.9
Intrarun Mean		0.129		26.6		38.2	
Intrarun %Bias		-14.0		6.4		1.9	
17-May-2023	3	#0.181	20.7	24.8	-0.8	39.1	4.3
		0.146	-2.7	25.7	2.8	37.6	0.3
Intrarun Mean		0.164		25.3		38.4	
Intrarun %Bias		9.3		1.2		2.4	
# > 15%Bias							

Table 10: Long-Term Stability at -70 °C (Continued)

Treated with and without comed[2]

Run Date	Run ID	Comed1 LTS -70 low 0.150 µg/mL	%Bias	LTS -70 low 0.150 µg/mL	%Bias	Comed1 LTS -70 high 37.5 µg/mL	%Bias	LTS -70 high 37.5 µg/mL	%Bias
18-Feb-2023	1	0.149	-0.7	0.172	14.7	40.1	6.9	#44.0	17.3
(112 days)		0.169	12.7	#0.182	21.3	37.6	0.3	40.4	7.7
		0.167	11.3	#0.194	29.3	38.7	3.2	40.1	6.9
		#0.188	25.3	#0.174	16.0	38.6	2.9	38.8	3.5
		0.161	7.3	0.158	5.3	37.0	-1.3	38.7	3.2
		#0.174	16.0	0.168	12.0	38.6	2.9	38.5	2.7
Intrarun Mean		0.168		0.175		38.4		40.1	
Intrarun SD		0.0130		0.0123		1.06		2.07	
Intrarun %CV		7.7		7.0		2.8		5.2	
Intrarun %Bias		12.0		16.7		2.4		6.9	
n		6		6		6		6	
04-Mar-2023	2			#0.187	24.7			36.5	-2.7
(126 days)				0.161	7.3			39.2	4.5
				#0.177	18.0			36.5	-2.7
				#0.175	16.7			40.7	8.5
				0.147	-2.0			38.8	3.5
				0.148	-1.3			36.9	-1.6
Intrarun Mean				0.166				38.1	
Intrarun SD				0.0164				1.73	
Intrarun %CV				9.9				4.5	
Intrarun %Bias				10.7				1.6	
n				6				6	
17-May-2023	3	0.142	-5.3	0.158	5.3	36.8	-1.9	37.5	0.0
(201 days)		0.138	-8.0	0.156	4.0	36.5	-2.7	37.7	0.5
		0.142	-5.3	0.146	-2.7	37.5	0.0	36.7	-2.1
		0.138	-8.0	0.151	0.7	36.7	-2.1	39.0	4.0
		0.141	-6.0	0.150	0.0	37.3	-0.5	37.0	-1.3
		0.148	-1.3	0.143	-4.7	36.5	-2.7	37.2	-0.8
Intrarun Mean		0.142		0.151		36.9		37.5	
Intrarun SD		0.00367		0.00572		0.422		0.808	
Intrarun %CV		2.6		3.8		1.1		2.2	
Intrarun %Bias		-5.3		0.7		-1.6		0.0	
n		6		6		6		6	
# > 15%Bias									

Criteria: To be deemed stable, the mean %Bias at each level must be within ± 15.0% with %CV ≤ 15.0%.

Results: Free zidebactam is stable in human plasma ultrafiltrate with and without the presence of comed[2], with sodium heparin as anticoagulant, for up to 201 days upon storage in polypropylene tube at -70 °C. See Section 10.4 for reason for run 1 stability failure.

WOCKHARDT LIFE WINS

ANALYTICAL RESEARCH

CERTIFICATE OF ANALYSIS FOR REFERENCE STANDARD

**Name**	**:**	**Zidebactam (WCK 5107)**			
Molecular Formula	:	$C_{13}H_{21}N_5O_7S.2H_2O$	Batch Number	:	WK1/WP-0060/016
Molecular Weight	:	427.45	Laboratory Note Book Reference	:	WA-2362-010
Date Of Analysis	:	02/03/2023	Month Of Manufacturing	:	May' 2019.
Valid Up To	:	01/03/2024	Reference Standard Number	:	RS/2023-009
Effective Date	:	30/03/2023	Page Number	:	Page 1 of 4

Sr. No.	Tests	Specifications	Results
1	Description	White to pale yellow powder	White powder
2	Identification (By IR, Mass & NMR Spectroscopy)	Spectral data should conform to the structure	Spectral data conforms the structure
3	Solubility	Freely soluble in water, Insoluble in dichloromethane	Freely soluble in water, Insoluble in dichloromethane
4	Completeness and Clarity of Solutions (10% w/v solution in Water for Injection (WFI))	The solid dissolves completely, leaving no undissolved matter.	Complies
		The constituted solution is not significantly less clear than an equal volume of the WFI contained in a similar vessel and examined similarly.	Complies
		The constituted solution is free from particulate matter that can be observed on visual inspection.	Complies
5	pH of Solution	Between 5.0 to 7.0	6.0
6	Absorbance of Solution (1.25 % w/v in water at 420 nm)	Not more than 0.2 AU	0.01 AU
7	Water Content (By KF Titration)	Not more than 12.0 % w/w	8.6 % w/w
8	Specific Optical Rotation (On anhydrous basis) (0.5 % w/v solution in Water)	Between –28° to -40°	-36°
9	Palladium Content (By ICP-OES)	Not more than 10 µg/g	BLQ (LOQ 2.5 µg/g)*

	Prepared by	Checked by	Approved by
Name :	Mr. Sitaram Ubale	Mr. Rajendra Chavan	Ms. Mohini Khatik
Department :	AVL	AVL	CRDQ
Sign :	[signature]	[signature]	[signature]
Date :	10/03/2023	10/03/2023	11/03/2023

Format No.: AVL-045-F-02-00

Ranked # 1 in Healthcare
GREAT PLACE TO WORK Best Companies to Work for 2015 India

ANALYTICAL RESEARCH

CERTIFICATE OF ANALYSIS FOR REFERENCE STANDARD

**Name**	:	**Zidebactam (WCK 5107)**			
Molecular Formula	:	$C_{13}H_{21}N_5O_7S.2H_2O$	Batch Number	:	WK1/WP-0060/016
Molecular Weight	:	427.45	Laboratory Note Book Reference	:	WA-2362-010
Date Of Analysis	:	02/03/2023	Month Of Manufacturing	:	May' 2019
Valid Up To	:	01/03/2024	Reference Standard Number	:	RS/2023-009
Effective Date	:	30/03/2023	Page Number	:	Page 2 of 4

Sr. No.	Tests	Specifications	Results
10	Related Substances (By HPLC) :		
	i. Impurity -1	Not more than 2.00 % w/w	0.24 % w/w
	ii. Impurity -2	Not more than 1.00 % w/w	Not detected
	iii. Impurity -3	Not more than 1.00 % w/w	0.20 % w/w
	iv. Impurity -4	Not more than 0.10 % w/w	Not detected
	v. Specified Impurity at RRT 2.16-2.28 (MW 782)	Not more than 0.50 % w/w	0.02 % w/w
	vi. Any Unspecified Impurity at RRT 0.59	Not more than 0.15 % w/w	0.04 % w/w
	vii. Total Impurities	Not more than 4.00 % w/w	0.50 % w/w
11	Residual Solvents (By GC) :		
	Group A		
	i. Isopropyl alcohol	Not more than 5000 µg/g	Not detected
	ii. Dichloromethane	Not more than 600 µg/g	Not detected
	iii. Methanol	Not more than 3000 µg/g	1762 µg/g
	iv. Ethyl acetate	Not more than 5000 µg/g	Not detected
	v. Cyclohexane	Not more than 3880 µg/g	Not detected
	vi. Acetone	Not more than 5000 µg/g	Not detected
	vii. Methyl tertiary butyl ether	Not more than 5000 µg/g	Not detected
	viii. Toluene	Not more than 890 µg/g	Not detected
	ix. Ethanol	Not more than 5000 µg/g	2990 µg/g
	Group B		
	i. Triethylamine	Not more than 250 µg/g	Not detected
	ii. Pyridine	Not more than 200 µg/g	Not detected

	Prepared by	Checked by	Approved by
Name :	Mr. Sitaram Ubale	Mr. Rajendra Chavan	Ms. Mohini Khatik
Department :	AVL	AVL	CRDQ
Sign :	[signature]	[signature]	[signature]
Date :	10/03/2023	10/03/2023	11/03/2023

Format No : AVL-045-F-02-00

Ranked # 1 in Healthcare
GREAT PLACE TO WORK Best Companies to Work for 2015 India

ANALYTICAL RESEARCH

CERTIFICATE OF ANALYSIS FOR REFERENCE STANDARD

**Name**	:	**Zidebactam (WCK 5107)**			
Molecular Formula	:	$C_{13}H_{21}N_5O_7S.2H_2O$	Batch Number	:	WK1/WP-0060/016
Molecular Weight	:	427.45	Laboratory Note Book Reference	:	WA-2362-010
Date Of Analysis	:	02/03/2023	Month Of Manufacturing	:	May' 2019
Valid Up To	:	01/03/2024	Reference Standard Number	:	RS/2023-009
Effective Date	:	30/03/2023	Page Number	:	Page 3 of 4

Sr. No.	Tests	Specifications	Results
12	Sodium Content (By ICP-OES)	Not more than 0.5 % w/w	BLQ (LOQ 0.25 % w/w)*
13	Sulfate Content (By Ion chromatography)	Not more than 1.0 % w/w	BLQ (LOQ 0.626 % w/w)
14	Mono-methyl Sulphate Content (By Ion chromatography)	Not more than 0.15% w/w	Not detected
15	Polymorphism (By X-ray Diffraction)	The diffractogram should exhibit characteristic 2 theta values at 13.8, 15.6, 20.3 and 24.3 (± 0.2°)	The diffractogram exhibits characteristic 2 theta values at 13.8, 15.6, 20.3 & 24.3
16	Potency (On as is basis)	Not applicable	90.4 %

BLQ = Below limit of quantification

LOQ = Limit of quantification

* LOQ Values are based on analytical method verification report (Report nos. MVR/VP17053 and MVR/VP17054) of Analytical Solutions (CRO).

Specification Reference Number: **ZDB-DSS-112-06**

Remark: Potency is calculated on the basis of Total Impurities, Water Content and Residual solvents.

Storage Condition: Store at 2°C to 8°C temperature.

	Prepared by	Checked by	Approved by
Name :	Mr. Sitaram Ubale	Mr. Rajendra Chavan	Ms. Mohini Khatik
Department :	AVL	AVL	CRDQ
Sign :	[signature]	[signature]	[signature]
Date :	10/03/2023	10/03/2023	11/03/2023

Format No. : AVL-045-F-02-00

Ranked # 1 in Healthcare
GREAT PLACE TO WORK Best Companies to Work for 2015 India

ANALYTICAL RESEARCH

CERTIFICATE OF ANALYSIS FOR REFERENCE STANDARD

**Name**	:	**Zidebactam (WCK 5107)**			
Molecular Formula	:	$C_{13}H_{21}N_5O_7S.2H_2O$	Batch Number	:	WK1/WP-0060/016
Molecular Weight	:	427.45	Laboratory Note Book Reference	:	WA-2362-010
Date Of Analysis	:	02/03/2023	Month Of Manufacturing	:	May' 2019
Valid Up To	:	01/03/2024	Reference Standard Number	:	RS/2023-009
Effective Date	:	30/03/2023	Page Number	:	Page 4 of 4

Chemical Names:

i. **WCK 5107**: (2S,5R)-7-Oxo-6-sulphoxy-2-[N'-((R)-piperidin-3-carbonyl)-hydrazinocarbonyl]-1,6-diaza-bicyclo[3.2.1]octane dehydrate

ii. **Impurity-1**: (2S,5R)-5-sulphooxyamino-2-[N'-((R)-piperidin-3-carbonyl)-hydrazinocarbonyl]-piperidine

iii. **Impurity-2**: (2S,5R)-7-Oxo-6-hydroxy-2-[N'-((R)-piperidin-3-carbonyl)-hydrazinocarbonyl]-1,6-diaza-bicyclo[3.2.1]octane

iv. **Impurity-3**: (2S,5R)-7-Oxo-6-sulphooxy-2-[N'-((S)-piperidin-3-carbonyl)-hydrazinocarbonyl]-1,6-diaza-bicyclo[3.2.1]octane

v. **Impurity-4**: (2S,5R)-7-Oxo-6-sulphooxy-2-[N'-((R)-1-tert-butoxycarbonyl-piperidin-3-yl)-carbonyl)-hydrazinocarbonyl] -1,6-diaza-bicyclo[3.2.1]octane

	Prepared by	Checked by	Approved by
Name :	Mr. Sitaram Ubale	Mr. Rajendra Chavan	Ms. Mohini Khatik
Department :	AVL	AVL	CRDQ
Sign :	[signature]	[signature]	[signature]
Date :	10/03/2023	10/03/2023	11/03/2023

Format No. : AVL-045-F-02-00

Ranked # 1 in Healthcare GREAT PLACE TO WORK Best Companies to Work for 2015 India

WOCKHARDT | LiFE WiNS

ANALYTICAL RESEARCH

CERTIFICATE OF ANALYSIS FOR REFERENCE STANDARD

**Name**	:	**Zidebactam (WCK 5107)**			
Molecular Formula	:	$C_{13}H_{21}N_5O_7S.2H_2O$	Batch Number	:	WK1/WP-0060/016
Molecular Weight	:	427.45	Laboratory Note Book Reference	:	WA-2004-038
Date of Analysis	:	30/03/2022	Month of Manufacturing	:	May' 2019
Valid Up To	:	29/03/2023	Reference Standard Number	:	RS/2022-013
Effective Date	:	23/04/2022	Page Number	:	Page 1 of 4

Sr. No.	Tests	Specifications	Results
1	Description	White to pale yellow powder	White powder
2	Identification (By IR, Mass & NMR Spectroscopy)	Spectral data should conform to the structure	Spectral data conforms the structure
3	Solubility	Freely soluble in water, Insoluble in dichloromethane	Freely soluble in water, Insoluble in dichloromethane
4	Constituted solution (10% w/v solution in WFI)<br>Completeness of solution<br><br>Clarity of solution	 <br>The solid dissolves completely, leaving no visible residue as un-dissolved matter<br>The constituted solution is not significantly less clear than an equal volume of the WFI contained in a similar vessel and examined similarly	 <br>Complies<br><br>Complies
5	pH of solution	Between 5.0 to 7.0	6.0
6	Absorbance of solution (1.25 % w/v in water at 420 nm)	Not more than 0.2 AU	0.01 AU
7	Water content (By KF Titration)	Not more than 12.0 % w/w	8.7 % w/w
8	Specific optical rotation (On anhydrous basis)	Between –28° to -40°	-36°
9	Heavy metals	Not more than 20 µg/g	Less than 20 µg/g
10	Palladium content (By ICP-OES)	Not more than 10 µg/g	BLQ (LOQ 2.5 µg/g)*

	Prepared by	Checked by	Approved by
Name :	Mr. Sitaram Ubale	Mr. Rajendra Chavan	Mr Subhash Chandra Shukla
Department :	AVL	AVL	CRDQ
Sign :	[signature]	[signature]	[signature]
Date :	12/04/2022	12/04/2022	13/04/2022

Format No. : AVL-045-F-02-00

Ranked # 1 in Healthcare Best Companies to Work for 2015 India

WOCKHARDT | LIFE WINS

ANALYTICAL RESEARCH

CERTIFICATE OF ANALYSIS FOR REFERENCE STANDARD

**Name**	:	**Zidebactam (WCK 5107)**			
Molecular Formula	:	$C_{13}H_{21}N_5O_7S.2H_2O$	Batch Number	:	WK1/WP-0060/016
Molecular Weight	:	427.45	Laboratory Note Book Reference	:	WA-2004-038
Date of Analysis	:	30/03/2022	Month of Manufacturing	:	May' 2019
Valid Up To	:	29/03/2023	Reference Standard Number	:	RS/2022-013
Effective Date	:	23/04/2022	Page Number	:	Page 2 of 4

Sr. No.	Tests	Specifications	Results
11	Related substances (By HPLC) :		
	i. Impurity -1	Not more than 2.00 % w/w	0.28 % w/w
	ii. Impurity -2	Not more than 1.00 % w/w	Not detected
	iii. Impurity -3	Not more than 1.00 % w/w	0.10 % w/w
	iv. Impurity -4	Not more than 0.10 % w/w	Not detected
	v. Specified impurity at RRT 2.16-2.28 (MW 782)	Not more than 0.50 % w/w	0.03 % w/w
	vi. Any unspecified impurity	Not more than 0.15 % w/w	BDL
	vii. Total impurities	Not more than 4.00 % w/w	0.41 % w/w
12	Residual solvents (By GC) :		
	Group A		
	i. Isopropyl alcohol	Not more than 5000 µg/g	Not detected
	ii. Dichloromethane	Not more than 600 µg/g	Not detected
	iii. Methanol	Not more than 3000 µg/g	1762 µg/g
	iv. Ethyl acetate	Not more than 5000 µg/g	Not detected
	v. Cyclohexane	Not more than 3880 µg/g	Not detected
	vi. Acetone	Not more than 5000 µg/g	Not detected
	vii. Methyl tertiary butyl ether	Not more than 5000 µg/g	Not detected
	viii. Toluene	Not more than 890 µg/g	Not detected
	ix. Ethanol	Not more than 5000 µg/g	2990 µg/g
	Group B		
	i. Triethylamine	Not more than 250 µg/g	Not detected
	ii. Pyridine	Not more than 200 µg/g	Not detected

	Prepared by	Checked by	Approved by
Name :	Mr. Sitaram Ubale	Mr. Rajendra Chavan	Mr Subhash Chandra Shukla
Department :	AVL	AVL	CRDQ
Sign :	[signature]	[signature]	[signature]
Date :	12/04/2022	12/04/2022	13/04/2022

Format No. : AVL-045-F-02-00

Ranked # 1 in Healthcare
GREAT PLACE TO WORK
Best Companies to Work for 2015 India

ANALYTICAL RESEARCH

CERTIFICATE OF ANALYSIS FOR REFERENCE STANDARD

**Name**	:	**Zidebactam (WCK 5107)**			
Molecular Formula	:	$C_{13}H_{21}N_5O_7S.2H_2O$	Batch Number	:	WK1/WP-0060/016
Molecular Weight	:	427.45	Laboratory Note Book Reference	:	WA-2004-038
Date of Analysis	:	30/03/2022	Month of Manufacturing	:	May' 2019
Valid Up To	:	29/03/2023	Reference Standard Number	:	RS/2022-013
Effective Date	:	23/04/2022	Page Number	:	Page 3 of 4

Sr. No.	Tests	Specifications	Results
13	Sodium content (By ICP-OES)	Not more than 0.5 % w/w	BLQ (LOQ 0.25 % w/w)*
14	Sulfate content (By Ion chromatography)	Not more than 1.0 % w/w	BLQ (LOQ 0.626 % w/w)
15	Mono-methyl sulphate content (By Ion chromatography)	Not more than 0.15% w/w	Not detected
16	Polymorphism (By X-ray Diffraction)	The diffractogram should exhibit characteristic 2 theta values at 13.8, 15.6, 20.3 and 24.3 (± 0.2°)	The diffractogram exhibits characteristic 2 theta values at 13.8, 15.6, 20.3 & 24.3
17	Potency (on as is basis)	Not applicable	90.4 %

BLQ = Below limit of quantification
BDL = Below disregard limit. Disregard limit 0.05 %
LOQ = Limit of quantification

* LOQ Values are based on analytical method verification report (Report nos. MVR/VP17053 and MVR/VP17054) of Analytical Solutions (CRO).

Specification Reference Number: **ZDB-DSS-112-05**

Remark: Potency is calculated on the basis of Total impurities, Water content, Residual solvents, Sulphate, Mono-methyl sulphate, Sodium and Palladium content.

Storage Condition: Store at 2°C to 8°C temperature.

	Prepared by	Checked by	Approved by
Name :	Mr. Sitaram Ubale	Mr. Rajendra Chavan	Mr Subhash Chandra Shukla
Department :	AVL	AVL	CRDQ
Sign :	[signature]	[signature]	[signature]
Date :	12/04/2022	12/04/2022	13/04/2022

Format No. : AVL-045-F-02-00

Ranked # 1 In Healthcare
Great Place to Work
Best Companies to Work for 2015 India

WOCKHARDT LiFE WiNS

ANALYTICAL RESEARCH

CERTIFICATE OF ANALYSIS FOR REFERENCE STANDARD

**Name**	:	**Zidebactam (WCK 5107)**			
Molecular Formula	:	$C_{13}H_{21}N_5O_7S.2H_2O$	Batch Number	:	WK1/WP-0060/016
Molecular Weight	:	427.45	Laboratory Note Book Reference	:	WA-2004-038
Date of Analysis	:	30/03/2022	Month of Manufacturing	:	May' 2019
Valid Up To	:	29/03/2023	Reference Standard Number	:	RS/2022-013
Effective Date	:	23/04/2022	Page Number	:	Page 4 of 4

Chemical Names:

i. **WCK 5107**: (2S,5R)-7-Oxo-6-sulphoxy-2-[N'-((R)-piperidin-3-carbonyl)-hydrazinocarbonyl]-1,6-diaza-bicyclo[3.2.1]octane dehydrate

ii. **Impurity-1**: (2S,5R)-5-sulphooxyamino-2-[N'-((R)-piperidin-3-carbonyl)-hydrazinocarbonyl]-piperidine

iii. **Impurity-2**: (2S,5R)-7-Oxo-6-hydroxy-2-[N'-((R)-piperidin-3-carbonyl)-hydrazinocarbonyl]-1,6-diaza-bicyclo[3.2.1]octane

iv. **Impurity-3**: (2S,5R)-7-Oxo-6-sulphooxy-2-[N'-((S)-piperidin-3-carbonyl)-hydrazinocarbonyl]-1,6-diaza-bicyclo[3.2.1]octane

v. **Impurity-4**: (2S,5R)-7-Oxo-6-sulphooxy-2-[N'-((R)-1-tert-butoxycarbonyl-piperidin-3-yl)-carbonyl)-hydrazinocarbonyl] -1,6-diaza-bicyclo[3.2.1]octane

	Prepared by	Checked by	Approved by
Name :	Mr. Sitaram Ubale	Mr. Rajendra Chavan	Mr Subhash Chandra Shukla
Department :	AVL	AVL	CRDQ
Sign :	[signature]	[signature]	[signature]
Date :	12/04/2022	12/04/2022	13/04/2022

Format No. : AVL-045-F-02-00

Ranked # 1 in Healthcare
GREAT PLACE TO WORK Best Companies to Work for 2015 India

3050 Spruce Street, Saint Louis, MO 63103, USA
Website: www.sigma-aldrich.com
Email USA: techserv@sial.com
Outside USA: eurtechserv@sial.com

Certificate of Analysis

Product Name : **Cefotaxime sodium salt suitable for plant cell culture, BioReagent, powder**
Product Number : **C7039-500MG**
Batch Number : **0000146942**
Source Batch : 157206
CAS Number : 64485-93-4
Storage Temperature : Cooler/Refrigerated
Molecular Formula : $C_{16}H_{16}N_5NaO_7S_2$
Formula Weight : 477.45
Recommended Retest Date : Sep 2023
Quality Release Date : 02 Jan 2022

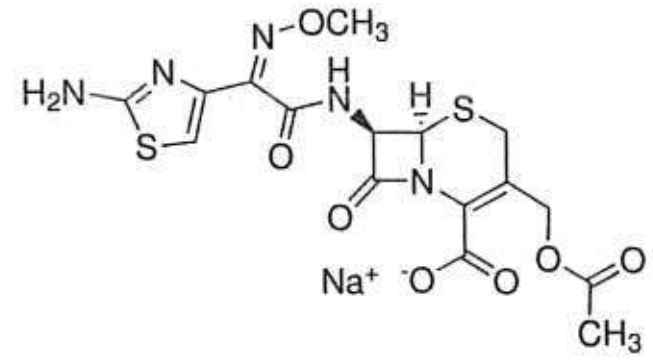

Test	Specification	Result
Appearance (Form)	Powder or Crystals	Powder
Appearance (Color)<br>Off-white to pale yellow	Conforms	Conforms
Solubility<br>10% in water	Clear	Clear
Infrared Spectrum	Conforms to Structure	Conforms
Total Impurity	≤ 3.0 %	1.7 %
Specific Rotation<br>[a]20/D	58 - 64 °	61 °
Assay (Dry Basis)	916 - 964 µg/mg	955 µg/mg
Loss on Drying	≤ 3.0 %	2.4 %
Plant Cell Culture Test	Pass	Pass

Anna Bendersky, Quality Control Manager
Quallity Control
Israel
IL

Sigma-Aldrich warrants, that at the time of the quality release or subsequent retest date this product conformed to the information contained in this publication. The current Specification sheet may be available at Sigma-Aldrich.com. For further inquiries, please contact Technical Service. Purchase must determine the suitability of the product for its particular use. See reverse side of website or packing slip for additional terms and conditions of sale

The branding on the header and/or footer of this document may temporarily not visually match the product purchased as we transition our branding. However, all of the information in the document regarding the product remains unchanged and matches the product ordered.
For further information please contact mlsbranding@sial.com

MedChemExpress

Certificate of Analysis

Inhibitors • Screening Libraries • Proteins

Ertapenem sodium

Cat. No.:	HY-13625
CAS No.:	153773-82-1
Batch No.:	180017
Chemical Name:	1-Azabicyclo[3.2.0]hept-2-ene-2-carboxylic acid, 3-[[(3S,5S)-5-[[(3-carboxyphenyl)amino]carbonyl]-3-pyrrolidinyl]thio]-6-[(1R)-1-hydroxyethyl]-4-methyl-7-oxo-, sodium salt (1:1), (4R,5S,6S)-

PHYSICAL AND CHEMICAL PROPERTIES

Molecular Formula:	$C_{22}H_{24}N_3NaO_7S$
Molecular Weight:	**497.50**
Storage:	4°C, sealed storage, away from moisture<br>* In solvent : -80°C, 6 months; -20°C, 1 month (sealed storage, away from moisture)

Chemical Structure:

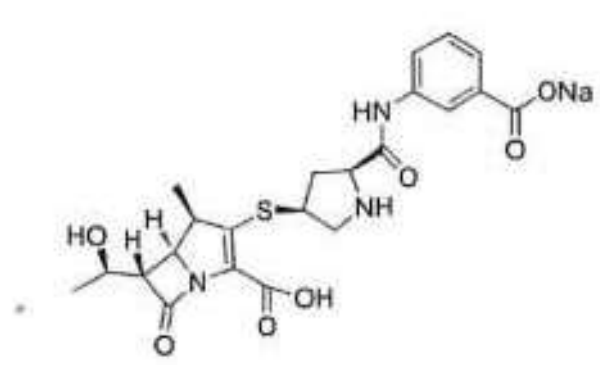

ANALYTICAL DATA

Appearance:	White to light yellow (Solid)
^{1}H NMR Spectrum:	Consistent with structure
LCMS:	Consistent with structure
Purity (LCMS):	**98.86%**
Test date:	2022/08/12
Retest date:	2024/08/11
Conclusion:	The product has been tested and complies with the given specifications.

Name: Lingli Ma
Designation: QA Manager
Date: 6/27/2022

Caution: Product has not been fully validated for medical applications. For research use only.

Tel: 609-228-6898 Fax: 609-228-5909 E-mail: tech@MedChemExpress.com
Address: 1 Deer Park Dr, Suite Q, Monmouth Junction, NJ 08852, USA

1. TITLE: THE DETERMINATION OF FREE ZIDEBACTAM IN TREATED HUMAN PLASMA ULTRAFILTRATE, WITH SODIUM HEPARIN AS ANTICOAGULANT, BY LC-MS/MS

BIOANALYTICAL METHOD VALIDATION REPORT

Bioanalytical Project Number: V1872106P4

Sponsor: DynPort Vaccine Company, LLC
110 Thomas Johnson Drive
Suite 200
Frederick, MD 21702

Bioanalytical Laboratory: KCAS Bioanalytical & Biomarker Services.
10830 S Clay Blair Boulevard
Olathe, Kansas 66061

Issue Date: February 2023

2. SYNOPSIS

This report presents the validation data for an LC-MS/MS method for the determination of free zidebactam in treated human plasma ultrafiltrate, with sodium heparin as anticoagulant to support the Phase 1 clinical trial studying the safety, tolerability, and pharmacokinetics of intravenous zidebactam in combination with ertapenem (WCK 6777) in healthy adult subjects. The assay was analyzed in the range of 0.0500 – 50.0 mcg/mL and is for use on treated human plasma ultrafiltrate samples obtained during clinical trials.

3. TABLE OF CONTENTS FOR THE BIOANALYTICAL METHOD VALIDATION REPORT

1. TITLE: THE DETERMINATION OF FREE ZIDEBACTAM IN TREATED HUMAN PLASMA ULTRAFILTRATE, WITH SODIUM HEPARIN AS ANTICOAGULANT, BY LC-MS/MS 1

2. SYNOPSIS 2

3. TABLE OF CONTENTS FOR THE BIOANALYTICAL METHOD VALIDATION REPORT 3

4. LIST OF TABLES 5

5. LIST OF FIGURES 7

6. SIGNATURE PAGE 8

7. QUALITY ASSURANCE STATEMENT 9

8. STUDY COMPLIANCE STATEMENT 10

9. LIST OF ABBREVIATIONS AND DEFINITIONS OF TERMS 11

10. INTRODUCTION 13

11. EXPERIMENTAL DESIGN AND RESULTS 13

11.1. Reference Standards 13

11.2. Calibration Standards and QC Samples Preparation 13

11.3. Selectivity 14

11.3.1. Blank Evaluation 14

11.3.2. Matrix Effect 14

11.3.3. Interference Evaluation 15

11.3.4. Matrix Evaluation 15

11.3.5. Carryover 15

11.4. Linearity and Analytical Range 16

11.5. Precision and Accuracy 16

11.6. Sensitivity 16

11.7. Run Size 17

11.8. Precision and Accuracy of Dilution 17

11.9. Freeze/Thaw Stability 17

11.10. Ice-Water Bath Stability 18
11.11. Long-Term Matrix Stability at -20 °C and -70 °C 18
11.12. Processed Sample Stability when Refrigerated 18
11.13. Individual Sample Reinjection Reproducibility 19
11.14. Whole Run Reinjection Reproducibility 19
11.15. Column Ruggedness 19
11.16. Extraction Recovery 19
11.17. Solution Stabilities 20
11.18. Calculations 20
11.19. SOP Deviations and Investigations 20
12. CONCLUSION 21
13. PERTINENT SOPS 21
14. ARCHIVES 21
15. REFERENCES 21
16. TABLES 22
17. FIGURES 54

4. LIST OF TABLES

Table 1: Abbreviations and Codes 11
Table 2: Mass Spec Transitions 22
Table 3: Reference Standards 22
Table 4: Calibration Standards and QC Samples Preparation 23
Table 5: Matrix Description 23
Table 6: Summary of Results and Conclusions 24
Table 7: Summary of Validation Run Objectives and Outcome 28
Table 8: Carryover 29
Table 9: Selectivity - Blank Assessment 29
Table 10: Matrix Effect – Low Concentration 30
Table 11: Matrix Effect – High Concentration 31
Table 12: Interference Evaluation (Presence of 150 mcg/mL Ertapenem) 32
Table 13: Hemolytic Effect Evaluation (2% Hemolyzation) 32
Table 14: Lipemic Effect Evaluation 32
Table 15: Summary of Standard Curves 33
Table 16: Precision and Accuracy (Intra-Assay) 34
Table 17: Precision and Accuracy (Inter-Assay) 35
Table 18: Precision and Accuracy of Sample Dilution 36
Table 19: Freeze-Thaw Stability, (Frozen At -20 °C and -70 °C Thawed in Wet Ice) 37
Table 20: Bench-Top Stability in Wet Ice 38
Table 21: Long-Term Stability at -20 °C and -70 °C 39
Table 22: Refrigerated Processed Sample Stability 40
Table 23: Individual Sample Reinjection Reproducibility 40
Table 24: Whole Run Reinjection Reproducibility and Column Ruggedness 41
Table 25: Extraction Recovery – Zidebactam 42
Table 26: Extraction Recovery – Cefotaxime 43
Table 27: Stability of Zidebactam Primary Solution in Deionized Water at Room Temperature 44

Table 28: Stability of Zidebactam Primary Solution in Deionized Water at -20 °C 45

Table 29: Stability of Zidebactam Spiking Solution in Deionized Water at Room Temperature 48

Table 30: Stability of Zidebactam Spiking Solution in Deionized Water at -20 °C 50

Table 31: Stability of Cefotaxime Working Internal Standard Solution in Deionized Water at Room Temperature 53

5. LIST OF FIGURES

Figure 1: Typical Calibration Curve 54

Figure 2: Typical Ion Chromatogram Blank Human Plasma Ultrafiltrate Without Internal Standard 55

Figure 3: Typical Ion Chromatogram Blank Human Plasma Ultrafiltrate With Internal Standard 56

Figure 4: Typical Ion Chromatogram LLOQ Calibration Standard, Zidebactam = 0.0500 mcg/mL 57

Figure 5: Typical Ion Chromatogram Calibration Standard, Zidebactam = 20.0 mcg/mL 58

Figure 6: Typical Ion Chromatogram ULOQ Calibration Standard, Zidebactam = 50.0 mcg/mL 59

Figure 7: Typical Ion Chromatogram Cross-Talk Evaluation High QC No IS 60

Annex 1 .... Test Procedure

Annex 2 .... Certificate(s) of Analysis

6. SIGNATURE PAGE

Bioanalytical Project Number: V1872106P4

Issue Date: February 2023

APPROVED

DocuSigned by:

Anika Pippin

Signer Name: Anika Pippin
Signing Reason: I approve this document
Signing Time: 09-Feb-2023 | 16:49 CST
BE3AA1198E40459E8A7E3C615D981D82

Anika Pippin, B.S.
Principal Investigator

REVIEWED

DocuSigned by:

Marsha Luna

Signer Name: Marsha Luna
Signing Reason: I have reviewed this document
Signing Time: 09-Feb-2023 | 16:42 CST
C9CC738561AF4CC1B375ED013DD1E4BD

Marsha Luna, B.S.
Management

7. QUALITY ASSURANCE STATEMENT

This Bioanalytical Method Validation Report has been found to describe accurately the methods and SOPs used and to accurately reflect the raw data collected. The Quality Assurance Unit initiated audits on this study and reported the findings to the Principal Investigator (PI) and Management as follows:

		**Date(s) Sent to:**	
**Inspection Date(s)**	**Inspection Phase**	**PI**	**Management**
30Nov2022	Data Audit	30Nov2022	30Nov2022
27Dec2022	Data Audit	27Dec2022	30Dec2022
05Jan2023	Draft Report Review	05Jan2023	24Jan2023
08Feb2023	Final Report Review	08Feb2023	08Feb2023

REVIEWED

DocuSigned by:

Signer Name: Jessica Lopez
Signing Reason: I have reviewed this document
Signing Time: 09-Feb-2023 | 16:33 CST
95C7FCBE477F4555996DA2F7A85A8F26

Jessica Lopez
Quality Assurance Unit

8. STUDY COMPLIANCE STATEMENT

I, the undersigned, hereby declare that this study was conducted in compliance with the relevant bioanalytical aspects of FDA Guidance for Industry on Bioanalytical Methods Validation, May 2018 [1] and KCAS Bioanalytical & Biomarker Services (KCAS) procedures and that the work was performed by me, or under my direction, and the report reflects a true and accurate record of the results obtained.

The acceptance criteria are based on the FDA Guidance for Industry, Bioanalytical Method Validation (2018). [1]

APPROVED

DocuSigned by:

Anika Pippin

Signer Name: Anika Pippin
Signing Reason: I approve this document
Signing Time: 09-Feb-2023 | 16:49 CST
BE3AA1198E40459E8A7E3C615D981D82

Anika Pippin, B.S.
Principal Investigator

9. LIST OF ABBREVIATIONS AND DEFINITIONS OF TERMS

Table 1: Abbreviations and Codes

Abbreviation	Definition
<	Concentration less than the lowest acceptable calibration standard
>	Concentration exceeded the upper limit of calibration range (> ULOQ)
A	Amendment
AQL	Above Quantitation Limit
%Bias	Percentage measurement valid
Batch	Is the same as Run
BQL	Below Quantifiable Limit
Cal	Calibration standard
Conc.	Concentration
Conv.	Conversion
%CV	Coefficient of Variation
dev	Deviation
diff	Difference
EMA	European Medicines Agency
FDA	Food and Drug Administration
GLP	Good Laboratory Practice
HPLC	High Performance Liquid Chromatography
ID	Identification
IS	Internal Standard
ISR	Incurred Sample Reanalysis
K_2EDTA or K_3EDTA	Dipotassium or Tripotassium Ethylenediaminetetraacetic Acid
KCAS	KCAS Bioanalytical & Biomarker Services
LC-MS/MS	Liquid Chromatography Tandem Mass Spectrometry
LLOQ	Lower Limit of Quantitation
mcg or µg or ug	Microgram
mcL or µL or uL	Microliter

Table 1: Abbreviations and Codes (Continued)

Abbreviation	Definition
ME	Matrix Effect
MF	Matrix Factor
MHLW	Ministry of Health, Labour and Welfare of Japan
mL	Milliliter
MS	Mass Spectrometry
n	number
N.C.	Not Calculated
ND	No difference reported as ISR will be counted
ng	nanogram
n.report	No Data available to report
N/A or NA	Not Applicable
N.R.	Not Reported
P&A	Precision and Accuracy
p.a.r.	Peak Area Ratio
PI	Principal Investigator
QC	Quality Control Sample
QCs	Quality Control Samples
OECD	Organization for Economic Co-operation and Development
RT	Room Temperature
sd or SD	Standard deviation
Seq	Sequence
SOP (s)	Standard Operating Procedure (s)
temp	Temperature
TP	Test procedure
UHPLC	Ultra High Performance Liquid Chromatography
ULOQ	Upper Limit of Quantitation
WIS	Working Internal Standard

This is a basic list. Some of the abbreviations may not have been used in this report.

10. INTRODUCTION

The objective of this study was to perform a full validation of a bioanalytical method for the determination of free zidebactam in MES ([2-(N-Morpholino) ethane sulphonic acid hydrate])-treated human plasma ultrafiltrate, with sodium heparin as anticoagulant. This validation report describes the selectivity, sensitivity, linearity, accuracy, precision, stability and recovery experiments based on an established bioanalytical test procedure.

The validation procedure was designed to meet or exceed the requirements of FDA Guidance for Industry on Bioanalytical Methods Validation, May 2018. The software programs used were Microsoft Office Excel 365, acquisition and processing software Analyst 1.7.2, and data management software Watson LIMS 7.5.

11. EXPERIMENTAL DESIGN AND RESULTS

In this method, free zidebactam and internal standards, cefotaxime, were released from treated human plasma ultrafiltrate using 96 well protein precipitation. After extraction, the sample was chromatographed using reversed phase HPLC with a Fortis C18 analytical column. Zidebactam and cefotaxime (IS) were detected by monitoring the precursor and product ions described in Table 2 using an Applied Biosystems Sciex API 5000 LC-MS/MS. The quantification was based on setting a calibration graph using the internal standard method based on bioanalytical test procedure TP-80217, entitled "LC-MS/MS Analysis of Free Zidebactam in Treated Human Plasma Ultrafiltrate (Sodium Heparin, 50 mcL, 0.0500 – 50.0 mcg/mL; Prior to MES Treatment)". The test procedure can be found in Annex 1.

Human plasma, screened for the lack of interference at the retention times of zidebactam and cefotaxime (IS), was diluted 1:1 with MES buffer, pH 6.4 for stabilization. Since the standards and QCs concentrations were already adjusted for the addition of MES during the preparation, the obtained free zidebactam concentration of the unknown study samples do not need to be adjusted for the dilution with MES buffer.

A summary of the validation results is provided in Table 6. A summary describing the objective(s) and outcome of each validation run attempted is provided in Table 7.

The method was validated within the analytical range of 0.0500 – 50.0 mcg/mL in treated human plasma ultrafiltrate. No development work was performed after the method was validated.

11.1. Reference Standards

Reference standards used during this validation are presented in Table 3.

11.2. Calibration Standards and QC Samples Preparation

Calibration standards and quality control pools were prepared by spiking appropriate levels of zidebactam into human plasma ultrafiltrate, with sodium heparin as anticoagulant. Primary

reference standards were prepared in at least duplicate and compared within 5% of each other. Standards and QCs were prepared and stored frozen at KCAS at -70 °C. Stability QCs were also stored at -20 °C. Standards, QCs, and the critical solutions used during this validation are described in Table 4, and matrix used is described in Table 5.

11.3. Selectivity

Selectivity is the ability of the bioanalytical method to measure and differentiate zidebactam in the presence of components that may be expected to be present. These could include metabolites, impurities, degradants, or matrix components. The selectivity of this method was achieved by a sample clean-up procedure using protein precipitation followed by chromatography and specific detection of the precursor and product ions of each analyte using tandem mass spectrometry (MS/MS).

11.3.1. Blank Evaluation

A minimum of six different sources of plasma ultrafiltrate (sodium heparin as anticoagulant) were screened. To be deemed acceptable, the peak response observed must be ≤ 20.0% of the mean response of the lower limit of quantitation (LLOQ) and ≤ 5.0% of the mean response of the internal standard. If any lots fail, further evaluation is needed.

All matrix lots were found to be free of any significant interference at the retention times of the analytes of interest. Data is presented in Table 9. Typical chromatograms for blank plasma ultrafiltrate, with sodium heparin as anticoagulant, without and with internal standard are presented in Figure 2 and Figure 3, respectively. Cross talk was also evaluated by extracting the high QC with no internal standard. There are no apparent effects of cross talk, and a typical chromatogram is present in Figure 7.

11.3.2. Matrix Effect

Matrix effect in MS applications is defined as inter-matrix variation in signal due to co-eluting matrix substances which cause enhancement or suppression of the detector response for the measured analyte.

To evaluate matrix effect using matrix factor, zidebactam and cefotaxime (IS) were spiked in four replicates into six different lots of extracted human plasma ultrafiltrate, with sodium heparin as anticoagulant. Zidebactam and cefotaxime (IS) were also spiked in four replicates into a proper extracted solvent or neat solution for comparison.

For zidebactam and cefotaxime (IS), the matrix factor (MF) was calculated for each lot of human plasma ultrafiltrate using the following equation:

$$\text{MF}=\frac{\text{Peak Response in Presence of Matrix Substances}}{\text{Mean Peak Response in Absence of Matrix}}$$

The IS normalized MF was calculated using the following equation:

$$\text{IS Normalized MF} = \frac{MF_{Analyte}}{MF_{IS}}$$

The evaluation was conducted for zidebactam at a high (close to ULOQ) and a low concentration (maximum of three times the LLOQ) and then the MF and IS Normalized MF were calculated for each lot of matrix. To be deemed acceptable for matrix effect test, the %CV for IS Normalized MF must be ≤ 15.0% over all six lots.

Matrix effect data are presented in Table 10 to Table 11. The matrix effect results meet acceptance criteria.

11.3.3. Interference Evaluation

Assay selectivity was confirmed by spiking quality control pools with 150 mcg/mL ertapenem. These QC pools, containing low and high levels of zidebactam, were analyzed in replicates of six in a single run. To be considered acceptable, the mean %Bias at each level is required to be within ± 15.0% and the %CV is required to be ≤ 15.0%.

The results presented in Table 12, indicate the presence of 150 mcg/mL ertapenem does not affect the accuracy or precision of the data.

11.3.4. Matrix Evaluation

To evaluate different types of collected matrix, QC pools were prepared with treated hemolytic (2% hemolysis) and lipemic human plasma ultrafiltrate, with sodium heparin as anticoagulant, at approximately three times the LLOQ and the high QC concentration. These QC pools were each extracted in at least replicates of three, and the mean concentrations or responses were calculated for each matrix. To be considered acceptable, the mean %Bias at each level was also required to be within ± 15.0% and the %CV was required to be ≤ 15.0%.

The results, presented in Table 13 to Table 14, indicate that hemolytic and lipemic plasma does not affect the analysis of zidebactam in treated human plasma ultrafiltrate.

Furthermore, blank lipemic and hemolytic control plasma were extracted with and without internal standard to determine that there were no significant interferences observed at the retention times of the analytes. There were no significant interferences observed.

11.3.5. Carryover

Carryover is evaluated within each run. It consists of measuring the response for each analyte and if it exceeds 20.0% of the response of LLOQ standard or other sample reflecting the lower sensitivity limit required, a carryover assessment is completed, and affected samples are rejected. The internal standard carryover evaluation is assessed within each run and if it exceeds 5.0% of the average internal standard response of acceptable standards and QCs, impact is evaluated within the run. Data is presented in Table 8.

11.4. Linearity and Analytical Range

The calibration curve was constructed with eight different, non-zero standard concentrations ranging from 0.0500 – 50.0 mcg/mL in treated human plasma ultrafiltrate, with sodium heparin as anticoagulant. Calibration standards were processed in duplicate and the calibration curve was evaluated using quadratic regression with weighting $1/x^2$. The maximum allowable percent deviation from nominal for each standard was ± 15.0%, except at the LLOQ, where ± 20.0% was accepted. Any standard deviating by more than these limits was required to be excluded from the regression line. For the calibration curve to be accepted, at least 75% of the initial number of standards was required to remain in the curve.

Quadratic regression was used for the calibration curve. The data are presented in Table 15. A typical calibration curve is presented graphically in Figure 1. Typical chromatograms illustrating blank human plasma ultrafiltrate standards spiked with zidebactam at the LLOQ, mid, and high concentrations are presented in Figure 4 to Figure 6, respectively.

11.5. Precision and Accuracy

The precision of an assay is defined as the closeness in which replicate analyses agree and is reported as percent coefficient of variation (%CV). The accuracy of a method is defined as the closeness of a determined value to its true (nominal) value and is reported as %Bias. At least one precision and accuracy run was performed using standards prepared from one primary stock and the QCs were prepared from a separate primary stock. Inter-assay (between runs) precision and accuracy and intra-assay (within runs) precision and accuracy were assessed in three runs by analyzing six replicates each of QCs fortified at four separate concentrations (LLOQ, low, mid, and high). For inter- and intra-assay, the mean %Bias at each QC level was required to be within ±15.0% from its respective nominal concentration, except at the LLOQ, where ± 20.0% was accepted. The %CV at each QC level was required to be ≤ 15.0%, except at the LLOQ, where ≤ 20.0% was accepted.

The intra-assay and inter-assay precision and accuracy data for this method met the required criteria and are presented in Table 16 (intra-assay precision and accuracy) and Table 17 (inter-assay precision and accuracy).

11.6. Sensitivity

The sensitivity of the method was determined in three runs by analyzing six replicates of QCs at the lower limit of quantitation (LLOQ) which were equal in concentration to the lowest calibration standard. The observed response at the LLOQ was required to be at least 5 times the response of noise in the human plasma ultrafiltrate blank, in addition to the precision and accuracy requirements outlined in the previous section.

The LLOQ for this method was determined to be 0.0500 mcg/mL, and the response at the LLOQ was at least 5 times the response of noise in the human matrix blank. Data are presented in Table 16 (intra-assay precision and accuracy) and Table 17 (inter-assay precision and accuracy).

11.7. Run Size

In order to evaluate the largest size run that can be used for study sample analysis, a run was processed with a set of 8 calibration standards and a minimum of 3 QC samples, processed in duplicate. In addition, at least 3 matrix blanks (one with internal standard and two without internal standard) were processed with the run. Run 5 was used for this assessment, and additional experiments were also analyzed which made the final run size 158.

In order for the run to be acceptable, at least one QC at each concentration level and two-thirds of the total number of QC samples were required to interpolate within ± 15.0% from nominal.

The standard and QC summary of run 5 is presented in Table 15 and Table 16 and acceptance criteria was met.

11.8. Precision and Accuracy of Dilution

To determine the accuracy of dilution, the high concentration QC spiked with zidebactam (37.5 mcg/mL) as well as an above-range QC (ARQC; 400 mcg/mL) were evaluated. All samples were extracted in six replicates according to the test procedure. To be deemed acceptable, the mean %Bias at each level must be within ± 15.0% with %CV ≤ 15.0%. The replicates were prepared as follows:

- Dilution factor 2x (high QC) = 25 mcL sample + 25 mcL blank human plasma ultrafiltrate
- Dilution factor 10x (ARQC) = 20 mcL sample + 180 mcL blank human plasma ultrafiltrate, mix well, then pipette 50 mcL as sample

Dilution precision and accuracy, using dilution factors of 2x and 10x, were proven to be valid. Data are presented in Table 18.

11.9. Freeze/Thaw Stability

Zidebactam stability in treated human plasma ultrafiltrate with and without the presence of comeds[2], with sodium heparin as anticoagulant, during sequential freeze/thaw cycles (frozen separately at -20 °C and -70 °C and thawed unassisted in wet ice) was evaluated. Aliquots of high and low concentration QC samples were frozen for at least 12 hours before the initial thawing. They were re-frozen 2 more times for at least 12 hours each time and were thawed prior to assay along with a freshly prepared set of QCs and calibration standards. To be deemed stable, the mean %Bias at each level must be within ± 15.0% with %CV ≤ 15.0%.

Zidebactam in treated human plasma ultrafiltrate, with sodium heparin as anticoagulant, was proven stable over 3 cycles of freeze (-20 °C and -70 °C) and thaw (wet ice). Data are provided in Table 19.

11.10. Ice-Water Bath Stability

To evaluate zidebactam in treated human plasma ultrafiltrate with and without the presence of comeds[2], with sodium heparin as anticoagulant, stability during sample processing in an ice-water bath, aliquots of the high and low concentration QC pools were allowed to thaw in an ice-water bath for 6 hours and 24 hours, prior to analysis along with a freshly prepared set of QCs and calibration standards. To be deemed stable, the mean %Bias at each level must be within ± 15.0% with %CV ≤ 15.0%.

Zidebactam in treated human plasma ultrafiltrate with and without the presence of comeds[2], with sodium heparin as anticoagulant, was proven stable in an ice-water bath under normal laboratory lighting over a period of 24 hours. Data are provided in Table 20.

11.11. Long-Term Matrix Stability at -20 °C and -70 °C

To evaluate free zidebactam in human plasma ultrafiltrate with and without the presence of comeds[2], with sodium heparin as anticoagulant, stability during sample storage, aliquots of the high and low concentration QC pools were stored frozen at -20 °C and -70 °C nominally for 39 days prior to being analyzed, along with freshly prepared standards and QC samples. To be deemed stable, the mean %Bias at each level must be within ± 15.0% with %CV ≤ 15.0%.

Free zidebactam in human plasma ultrafiltrate with and without the presence of comeds[2], with sodium heparin as anticoagulant, was proven to be stable at -70 °C over a period of 39 days in polypropylene tubes.

Free zidebactam in human plasma ultrafiltrate with and without the presence of comeds[2], with sodium heparin as anticoagulant, was proven not to be stable at -20 °C over a period of 39 days in polypropylene tubes. Criteria not met at the high QC level for both, regular and comed QCs %Bias is low. Unknown samples should be stored at -70 °C.

Data are provided in Table 21.

11.12. Processed Sample Stability when Refrigerated

To evaluate zidebactam stability in processed samples at refrigerated temperatures, high and low concentration QC pools were processed, placed into polypropylene 96 well plates, and stored at refrigerated temperatures before being chromatographed with samples in a subsequent run. To be deemed stable, the mean %Bias at each level must be within ± 15.0% with %CV ≤ 15.0%.

Processed zidebactam samples were proven stable when refrigerated stored in polypropylene 96-well plates over a period of 5 days 21 hours. Data are provided in Table 22.

11.13. Individual Sample Reinjection Reproducibility

To evaluate individual sample reinjection reproducibility, low and high QC extracts (6 each) injected during the run were reinjected at the end of the run, within the established stability time window of processed samples in the refrigerator. To be deemed reproducible, the mean %Bias at each level must be within ± 15.0% with %CV ≤ 15.0%.

Reinjection of individual samples can be made with adequate precision and accuracy. Data are provided in Table 23.

11.14. Whole Run Reinjection Reproducibility

To evaluate whole run reinjection reproducibility, run 2 was reinjected as run 6 on the same MS platform using a different chromatographic column from the same manufacturer, with the same specification as the original within 6 days 20 hours following the original injection. To be deemed acceptable, the reinjected run was required to meet the acceptance criteria outlined in Sections 11.4, 11.5, and 11.6.

It has been demonstrated that entire runs can be reinjected with adequate accuracy and precision. Data are presented in Table 24.

11.15. Column Ruggedness

Column ruggedness was assessed by reinjecting run 2 as run 6 on the same MS platform (Applied Biosystems API5000) using a different chromatographic column from the same manufacturer, with the same specification as the original.

Data were required to meet the precision and accuracy criteria outlined in Sections 11.4, 11.5, and 11.6 to be considered as reproducible/rugged method. Data are presented in Table 24. It has been demonstrated that ruggedness has been met.

11.16. Extraction Recovery

To evaluate the extraction recovery, the low, mid, and high extracted QC were compared to extracted human plasma ultrafiltrate blanks to which an equal amount of zidebactam at each level, assuming 100% recovery, was added in six replicates.

To evaluate the extraction recovery of cefotaxime (IS), zidebactam was added only for tracking during analysis. Cefotaxime (IS) for the control was added after extraction, while the same amount of cefotaxime (IS) for the extracted QC was added before extraction.

$$\%\ \text{Recovery} = \frac{\text{Response ratio of "Extracted QC"}}{\text{Average Response Ratio of Control}} \times 100$$

The mean recovery of zidebactam was determined to be consistent, precise and reproducible at 109.3%.

The recovery of cefotaxime (IS) was determined to be consistent, precise and reproducible at 115.7%. Data are presented in Table 25 to Table 26.

11.17. Solution Stabilities

The stability of various solutions of zidebactam in various solvents used during the sample analysis following storage at various temperatures was determined.

Data showing the zidebactam(s), solvent composition, storage term, and temperature are presented in Table 27 to Table 31.

11.18. Calculations

The peak areas of zidebactam and cefotaxime were integrated by Analyst 1.7.2 (Applied Biosystems; Foster City, CA, USA). The data results were imported electronically into Watson LIMS 7.5 for the calculation of free zidebactam concentrations and statistical analysis. The calibration curves were obtained by a weighted (1/concentration^2) quadratic regression. After the best fit from data, the concentrations (mcg/mL) were calculated based on the measured peak area ratios of free zidebactam/cefotaxime using the following equation:

$$y = ax^2 + bx + c$$

Where x = Concentration of zidebactam(mcg/mL)
a, b, c = coefficients of regression

$$\text{Mean Value } (\overline{X}) = \frac{\sum_{i=1}^{n} \text{individual data (X)}}{\text{number of data points (n)}}$$

$$\text{Standard Deviation (SD)} = \sqrt{\frac{\sum_{i=1}^{n} \left[\text{individual data (X)} - \text{Mean Value } (\overline{X})\right]^2}{n-1}}$$

$$\text{Coefficient of Variation (\%CV)} = \left[\frac{\text{Standard Deviation (SD)}}{\text{Mean Value } (\overline{X})}\right] \times 100$$

$$\text{\%Bias} = \left(\frac{\text{Calculated Mean of the Observed Values or Observed Value} - \text{Nominal Value}}{\text{Nominal Value}}\right) \times 100$$

All calibration standards and QCs concentrations were reported to three significant figures, and study samples will be reported to three significant figures.

11.19. SOP Deviations and Investigations

No SOP deviations occurred. No investigations were conducted during this study.

12. CONCLUSION

Validation results obtained in this study indicate that a robust and reliable method has been established for the measurement of free zidebactam in treated human plasma ultrafiltrate, with sodium heparin as anticoagulant, by LC-MS/MS in the concentration range of 0.0500 – 50.0 mcg/mL. The generally recommended acceptance criteria, FDA Guidance for Industry on Bioanalytical Methods Validation, May 2018, in terms of precision, accuracy, selectivity including matrix effect, recovery and all types of stability have been met. Thus, this validated method is deemed suitable for determination of free zidebactam in treated human plasma ultrafiltrate, with sodium heparin as anticoagulant, for unknown samples.

13. PERTINENT SOPS

The following SOPs were followed during the conduct of this study:

SOP-00073 [02] - Bioanalytical Method Validation - Chromatographic Assays

SOP-00035 [00] - Procedures, Reporting, and Acceptance Criteria for Bioanalytical Chromatographic Projects

14. ARCHIVES

All raw data, including paper and electronic files associated with this study will remain archived at a location specified by KCAS, 10830 S Clay Blair Boulevard, Olathe, Kansas 66061 for a period of at least 10 years, after which the client may be contacted to arrange for continued storage of the files, or disposition according to an agreed procedure. KCAS will maintain an electronic (scanned/true) copy of the method validation report that can be viewed and printed on site. The study plan, documentation, raw data, and final report are indexed under Bioanalytical Project Number V1872106P4.

15. REFERENCES

1. Guidance for Industry: Bioanalytical Method Validation. U.S. Department of Health and Human Services. Food and Drug Administration. Center for Drug Evaluation and Research. Center for Veterinary Medicine. May 2018.
2. Comeds: QCs in the presence of 150 mcg/mL ertapenem.

16. TABLES

Table 2: Mass Spec Transitions

Analyte	Transition
Zidebactam	m/z 392.028→312.000
Cefotaxime (IS)	m/z 456.200→324.000

Table 3: Reference Standards

Analyte	Supplier	Lot/Batch Number	Purity	Expiration Date or Retest Date	Conversion Factor
Zidebactam	Wockhardt	WK1/WP-0060/016	90.4%	Expire 29 Mar 2023	1.000
Cefotaxime Sodium Salt (IS)	Sigma-Aldrich	0000146942	95.9%	Retest 30 Sep 2023	1.048
Ertapenem Sodium	Med Chem Express	180017	98.9%	Retest 11 Aug 2024	1.046

Certificates of analysis for the above reference standards are presented in Annex 2.

Table 4: Calibration Standards and QC Samples Preparation

<table>
<tr><th>Critical Solutions</th><th>Lot # for Standards (Prep Date)</th><th>Lot # for QCs (Prep Date)</th><th>Standards Lot (Prep Date)</th><th>QCS Lot (Prep Date)</th><th>Runs used</th></tr>
<tr><td>Sodium Heparin Human Plasma Ultrafiltrate</td><td colspan="2">BioIVT, Lot V1872106P4-8-1</td><td rowspan="2">V1872106P4-9-1
Set A
(28 Oct 2022)</td><td rowspan="2">V1872106P4-10-1
Set A
(28 Oct 2022)</td><td rowspan="2">2, QCs only:
3, 4, 8 (LTS)</td></tr>
<tr><td>Deionized water stored at -20°C in polypropylene</td><td>V1872106P4-1-1
(27 Oct 2022)
V1872106P4-4-1
(27 Oct 2022)</td><td>V1872106P4-1-1
(27 Oct 2022)
V1872106P4-2-1
(27 Oct 2022)
V1872106P4-5-1
(27 Oct 2022)</td></tr>
<tr><td>Sodium Heparin Human Plasma Ultrafiltrate</td><td colspan="2">BioIVT, Lot V1872106P4-8-1</td><td rowspan="2">V1872106P4-17-1
Set B
(31 Oct 2022)</td><td rowspan="2">N/A</td><td rowspan="2">4</td></tr>
<tr><td>Deionized water stored at -20°C in polypropylene</td><td>V1872106P4-1-1
(27 Oct 2022)
V1872106P4-4-1
(27 Oct 2022)</td><td>N/A</td></tr>
<tr><td>Sodium Heparin Human Plasma Ultrafiltrate</td><td colspan="2">BioIVT, Lot V1872106P4-8-1</td><td rowspan="2">V1872106P4-20-1
Set C
(03 Nov 2022)</td><td rowspan="2">V1872106P4-21-1
Set B
(03 Nov 2022)</td><td rowspan="2">5</td></tr>
<tr><td>Deionized water stored at -20°C in polypropylene</td><td>V1872106P4-1-1
(27 Oct 2022)
V1872106P4-4-1
(27 Oct 2022)</td><td>V1872106P4-2-1
(27 Oct 2022)
V1872106P4-5-1
(27 Oct 2022)</td></tr>
<tr><td>Sodium Heparin Human Plasma Ultrafiltrate</td><td colspan="2">BioIVT, Lot V1872106P4-8-1</td><td rowspan="3">NA</td><td rowspan="3">V1872106P4-11-1
Set A
Comed QCs
(28 Oct 2022)</td><td rowspan="3">2, 8 (LTS)</td></tr>
<tr><td>Deionized water stored at -20°C in polypropylene</td><td rowspan="2">NA</td><td>V1872106P4-2-1
(27 Oct 2022)
V1872106P4-5-1
(27 Oct 2022)</td></tr>
<tr><td>Ertapenem in deionized water stored at -20 °C in glass</td><td>V1872106U1-54-1
(12 Oct 2022)</td></tr>
<tr><td>Sodium Heparin Human Plasma Ultrafiltrate</td><td colspan="2">BioIVT, Lot V1872106P4-29-2</td><td rowspan="2">V1872106P4-30-1
Set D
(06 Dec 2022)</td><td rowspan="2">V1872106P4-29-1
Set C
(06 Dec 2022)</td><td rowspan="2">8</td></tr>
<tr><td>Deionized water stored at -20°C in polypropylene</td><td>V1872106P4-22-1
(05 Dec 2022)
V1872106P4-24-1
(05 Dec 2022)</td><td>V1872106P4-23-1
(05 Dec 2022)
V1872106P4-25-1
(05 Dec 2022)</td></tr>
</table>

Table 5: Matrix Description

Matrix	Source Lot #	Date Received
Sodium Heparin Human Plasma Ultrafiltrate Lot V1872106P4-8-1 (Combo-70-1)	BioIVT, Lots HMN790515, HMN790517, HMN790518, HMN790520, HMN790521, HMN790523	Apr 2022
Sodium Heparin Human Plasma Ultrafiltrate Lot V1872106P4-29-2	BioIVT, Lot HMN790516	Apr 2022

Table 6: Summary of Results and Conclusions

**Methodology – TP-80217**	**Method Performance – LC-MS/MS**	**Hyperlink – N/A**	**Comments**
Matrix	Plasma ultrafiltrate		
Anticoagulant	Sodium heparin		
Assay Volume	50 mcL		
**Selectivity**			
Analyte	Zidebactam	Summary tables<br>Table 9<br><br>Report text<br>Section 11.3.1	
Internal Standard	Cefotaxime	Summary tables<br>Table 9<br><br>Report text<br>Section 11.3.1	
Matrix Effect<br>(6 spiked matrix lots)	Complies	Summary tables<br>Table 10,Table 11<br><br>Report text<br>Section 11.3.2	
Interference	Complies	Summary tables<br>Table 12<br><br>Report text<br>Section 11.3.3	
Hemolytic Effect Evaluation	Complies	Summary tables<br>Table 13<br><br>Report text<br>Section 11.3.4	
Lipemic Effect Evaluation	Complies	Summary tables<br>Table 14<br><br>Report text<br>Section 11.3.4	
Carryover	Assessed with every run with standards and QCs	Summary tables<br>Table 8<br><br>Report text<br>Section 11.3.5	
**Linearity**			
Weighting	$1/x^2$	Report text<br>Section 11.4	
Bias at LLOQ	0.8%	Summary tables<br>Table 15<br><br>Report text<br>Section 11.4	

Table 6: Summary of Results and Conclusions (Continued)

Methodology – TP-80217	Method Performance – LC-MS/MS	Hyperlink – N/A	Comments
Bias above LLOQ	-2.6 to 2.0%	Summary tables<br>Table 15<br><br>Report text<br>Section 11.4	
Bioanalytical Range	Complies<br>0.0500 – 50.0 mcg/mL	Summary tables<br>Table 15<br><br>Report text<br>Section 11.4	
**Precision**			
Intra-assay	3.2 to 9.4% (LLOQ);<br>3.5 to 9.9% (above LLOQ)	Summary tables<br>Table 16<br><br>Report text<br>Section 11.5	
Inter-assay	6.1% (LLOQ);<br>5.4 to 6.3% (above LLOQ)	Summary tables<br>Table 17<br><br>Report text<br>Section 11.5	
**Accuracy**			
Intra-assay	8.4 to 9.2% (LLOQ);<br>1.2 to 11.3% (above LLOQ)	Summary tables<br>Table 16<br><br>Report text<br>Section 11.5	
Inter-assay	9.0% (LLOQ);<br>2.4 to 6.7% (above LLOQ)	Summary tables<br>Table 17<br><br>Report text<br>Section 11.5	
**Large Run Size**	Determined to be 158 samples.	Summary tables<br>Table 15 and Table 16<br><br>Report text<br>Section 11.7	
**Dilution**			
Dilution factor: 2x	Dilution QC: 37.5 mcg/mL<br>Precision: 3.0%<br>Accuracy: 3.5%	Summary tables<br>Table 18<br><br>Report text<br>Section 11.8	
Dilution factor: 10x	Dilution QC: 400 mcg/mL<br>Precision: 6.7%<br>Accuracy: 1.5%	Summary tables<br>Table 18<br><br>Report text<br>Section 11.8	

Table 6: Summary of Results and Conclusions (Continued)

Methodology – TP-80217	Method Performance – LC-MS/MS	Hyperlink – N/A	Comments
**Stability**			
Matrix (in polypropylene tubes)			
Freeze/Thaw (-20 °C/wet ice) with and without the presence of comeds[2]	Demonstrated for 3 cycles	Summary tables<br>Table 19<br><br>Report text<br>Section 11.9	
Freeze/Thaw (-70 °C/wet ice) with and without the presence of comeds[2]	Demonstrated for 3 cycles	Summary tables<br>Table 19<br><br>Report text<br>Section 11.9	
Bench-top stability in wet ice with and without the presence of comeds[2]	Demonstrated for 24 hours	Summary tables<br>Table 20<br><br>Report text<br>Section 11.10	
Long-term (-20 °C) with and without the presence of comeds[2]	Demonstrated instability at 39 days	Summary tables<br>Table 21<br><br>Report text<br>Section 11.11	
Long-term (-70 °C) with and without the presence of comeds[2]	Demonstrated for 39 days	Summary tables<br>Table 21<br><br>Report text<br>Section 11.11	
Processed Matrix polypropylene			
Refrigeration	Demonstrated for 5 day 21 hours	Summary tables<br>Table 22<br><br>Report text<br>Section 11.12	
**Individual Sample Reinjection**	Reproducible	Summary tables<br>Table 23<br><br>Report text<br>Section 11.13	
**Whole Run Reinjection Integrity**	Reproducible<br>Duration 6 days 20 hours	Summary tables<br>Table 24<br><br>Report text<br>Section 11.14	

Table 6: Summary of Results and Conclusions (Continued)

Methodology – TP-80217	Method Performance – LC-MS/MS	Hyperlink – N/A	Comments
**Column Ruggedness**	Reproducible	Summary tables<br>Table 24<br><br>Report text<br>Section 11.15	
**Extraction Recovery**			
Zidebactam	Overall 109.3%	Summary tables<br>Table 25<br><br>Report text<br>Section 11.16	
Cefotaxime (IS)	Overall 115.7%	Summary tables<br>Table 26<br><br>Report text<br>Section 11.16	
**Solution Stability**			
Solution, Primary polypropylene or glass			
Zidebactam in deionized water (RT) - glass	Stable 17.75 hours	Summary tables<br>Table 27	V1872106U2
Zidebactam in deionized water (-20 °C) - glass	Stable 25 days<br>Stable 61 days	Summary tables<br>Table 28	V1872106U2
Zidebactam in deionized water (-20 °C) - polypropylene	Stable 39 days	Summary tables<br>Table 28	
Solution, Working polypropylene or glass			
Zidebactam Spike in deionized water (RT) - polypropylene	Stable 17.75 hours	Summary tables<br>Table 29	V1872106U2
Zidebactam Spike in deionized water (-20 °C) - glass	Stable 25 days<br>Stable 61 days (high QC only)	Summary tables<br>Table 30	V1872106U2
Cefotaxime (IS) in deionized water in glass (RT) - glass	Stable 15.5 hours	Summary tables<br>Table 31	V1872106U2

Table 7: Summary of Validation Run Objectives and Outcome

Run ID	Instrument ID	Extraction Date	Assay Date	Run Objective(s)	Accepted or Rejected	Comment
2	LC/MS-39	28-Oct-2022	28-Oct-2022	Precision and accuracy, IS cross-talk, selectivity, individual sample reinjection reproducibility, processed sample stability extraction, and comed[2] interference	Accepted	[a]
3	LC/MS-39	28-Oct-2022	29-Oct-2022	Matrix factor and recovery	Accepted	
4	LC/MS-39	31-Oct-2022	31-Oct-2022	Precision and accuracy, dilution integrity, lipemic QCs, and hemolytic QCs	Accepted	
5	LC/MS-22	03-Nov-2022	03-Nov-2022	Precision and accuracy, freeze/thaw stability with and without comeds[2], bench-top stability with and without comeds[2], processed sample injection, and large run size	Accepted	
6	LC/MS-22	28-Oct-2022	03-Nov-2022	Whole run reinjection reproducibility and column ruggedness	Accepted	
7	LC/MS-22	05-Dec-2022	05-Dec-2022	Primary long-term solution stability, primary comparison, and spike linearity check	Accepted	
8	LC/MS-39	06-Dec-2022	06-Dec-2022	Long-term matrix stability with and without comeds[2]	Accepted	[b]

Run 1 was used for a spike linearity check and primary comparison and is not reported.
[a]Analyte response is not linear and quadratic regression will be used for this study.
[b]Criteria for LTS at -20 °C with and without comeds did not meet criteria at the high QC level as %Bias is low. Unknown samples should be stored at -70 °C.

Table 8: Carryover

Run ID	Result (Extracted Blank after High Standard)	Carryover Results for Internal Standard
2	Seq 13 and Seq 76 are <20.0% of the LLOQ.	Seq 4 and Seq 67 are < 5.0% internal standard peak of acceptable standards.
4	Seq 13 and Seq 86 are <20.0% of the LLOQ.	Seq 4 and Seq 77 are < 5.0% internal standard peak of acceptable standards.
5	Seq 13 and Seq 146 are <20.0% of the LLOQ.	Seq 4 and Seq 137 are < 5.0% internal standard peak of acceptable standards.
6	Seq 13 and Seq 50 are <20.0% of the LLOQ.	Seq 4 and Seq 41 are < 5.0% internal standard peak of acceptable standards.
8	Seq 13 and Seq 80 are <20.0% of the LLOQ.	Seq 4 and Seq 71 are < 5.0% internal standard peak of acceptable standards.

Note: Calibration Standards and QCs are arranged in a way to mitigate carryover in all validation runs.

Table 9: Selectivity - Blank Assessment

Run 2 Selectivity Blank Screen

Blank ID with or without IS	Blank Lot Number	Peak Area of Analyte	Peak Area of Internal Standard	Percent of LLOQ Peak Area	Percent of LLOQ Peak Area Ratio	Percent of IS in Blanks without IS
Blank 1 NO IS	V1872106P4-8-2	0	0	0.0	NA	0.0
Blank 2 NO IS	V1872106P4-8-3	0	0	0.0	NA	0.0
Blank 3 NO IS	V1872106P4-8-4	0	0	0.0	NA	0.0
Blank 4 NO IS	V1872106P4-8-5	0	0	0.0	NA	0.0
Blank 5 NO IS	V1872106P4-8-6	0	0	0.0	NA	0.0
Blank 6 NO IS	V1872106P4-8-7	0	0	0.0	NA	0.0
Blank 1	V1872106P4-8-2	0	502891	0.0	0.0	NA
Blank 2	V1872106P4-8-3	0	521172	0.0	0.0	NA
Blank 3	V1872106P4-8-4	0	520013	0.0	0.0	NA
Blank 4	V1872106P4-8-5	0	497296	0.0	0.0	NA
Blank 5	V1872106P4-8-6	0	530544	0.0	0.0	NA
Blank 6	V1872106P4-8-7	0	519992	0.0	0.0	NA

Note: Average IS response are from all of the accepted standards and QCs

Criteria: A minimum of six different sources of plasma ultrafiltrate (sodium heparin as anticoagulant) were screened. To be deemed acceptable, the peak response observed must be ≤ 20.0% of the mean response of the lower limit of quantitation (LLOQ) and ≤ 5.0% of the mean response of the internal standard. If any lots fail, further evaluation is needed.

Results: All matrix lots were found to be free of any significant interference at the retention times of the analytes of interest.

Table 10: Matrix Effect – Low Concentration

Run Number: 3

Concentration: 0.150mcg/mL

Sample ID	Drug in neat/matrix Area	Average Drug in Neat	Individual MF analyte	Average MF analyte	IS in neat/ matrix Area	Average IS in neat Area	Individual IS MF	Average IS MF	Average IS Normalized MF
Neat	12884	13066			511637	514655			
	12886				526451				
	12997				498738				
	13495				521793				
ME1	12886		0.9863	1.0168	492991		0.9579	0.9882	1.0290
	13062		0.9997		522417		1.0151		
	12569		0.9620		519613		1.0096		
	14625		1.1194		499259		0.9701		
ME2	12152		0.9301	0.9895	504360		0.9800	0.9970	0.9924
	13290		1.0172		516138		1.0029		
	14377		1.1004		519791		1.0100		
	11894		0.9103		512251		0.9953		
ME3	11654		0.8920	0.9223	514414		0.9995	0.9903	0.9313
	11968		0.9160		500054		0.9716		
	11818		0.9045		519794		1.0100		
	12759		0.9765		504380		0.9800		
ME4	12190		0.9330	0.9557	514471		0.9996	0.9880	0.9674
	11917		0.9121		505360		0.9819		
	13685		1.0474		508758		0.9885		
	12157		0.9305		505240		0.9817		
ME5	11125		0.8515	0.9150	503436		0.9782	0.9810	0.9328
	12529		0.9589		509852		0.9907		
	11966		0.9158		503596		0.9785		
	12202		0.9339		502543		0.9765		
ME6	12620		0.9659	0.9547	504864		0.9810	0.9708	0.9835
	11360		0.8695		503261		0.9779		
	13266		1.0153		496091		0.9639		
	12650		0.9682		494235		0.9603		
**Mean**									0.9727
**N**									6
**SD**									0.0374
**%CV**									3.8

neat = in absence of matrix, matrix = in presence of extracted matrix

Criteria: %CV of Average IS Normalized MF among 6 lots of matrix must be ≤ 15.0%.

Meets Acceptance Criteria: YES MF=matrix factor

$$MF = \frac{\text{Peak Response in Presence of Matrix Substances}}{\text{Mean Peak Response in Absence of Matrix}}$$

$$\text{IS Normalized MF} = \frac{MF_{Analyte}}{MF_{IS}}$$

Table 11: Matrix Effect – High Concentration

Run Number:		3							
Concentration:		40.0 mcg/mL							
**Sample ID**	**Drug in neat/matrix Area**	**Average Drug in Neat**	**Individual MF $_{analyte}$**	**Average MF $_{analyte}$**	**IS in neat/ matrix Area**	**Average IS in neat Area**	**Individual IS MF**	**Average IS MF**	**Average IS Normalized MF**
Neat	2310581	2422945			496340	497280			
	2461461				504150				
	2288072				486906				
	2631665				501725				
ME1	2321101		0.9580	1.0078	503165		1.0118	0.9916	1.0163
	2693770		1.1118		485387		0.9761		
	2359579		0.9738		490851		0.9871		
	2392496		0.9874		493052		0.9915		
ME2	2364479		0.9759	0.9995	500266		1.0060	1.0016	0.9979
	2248995		0.9282		499818		1.0051		
	2607801		1.0763		500754		1.0070		
	2465697		1.0176		491446		0.9883		
ME3	2287900		0.9443	1.0126	505722		1.0170	0.9958	1.0169
	2498782		1.0313		481448		0.9682		
	2444644		1.0090		507390		1.0203		
	2582819		1.0660		486284		0.9779		
ME4	2456058		1.0137	1.0730	497756		1.0010	0.9910	1.0827
	2714093		1.1202		486917		0.9792		
	2481366		1.0241		491964		0.9893		
	2747351		1.1339		494612		0.9946		
ME5	2528261		1.0435	1.0232	490807		0.9870	0.9951	1.0283
	2389315		0.9861		493766		0.9929		
	2403991		0.9922		490316		0.9860		
	2595452		1.0712		504398		1.0143		
ME6	2544400		1.0501	1.0239	489847		0.9851	0.9942	1.0299
	2538002		1.0475		491962		0.9893		
	2488055		1.0269		493352		0.9921		
	2353206		0.9712		502439		1.0104		
**Mean**									1.0287
**N**									6
**SD**									0.0288
**%CV**									2.8

neat = in absence of matrix, matrix = in presence of extracted matrix
Criteria: %CV of Average IS Normalized MF among 6 lots of matrix must be ≤ 15.0%.
Meets Acceptance Criteria: YES MF=matrix factor

$$\text{MF} = \frac{\text{Peak Response in Presence of Matrix Substances}}{\text{Mean Peak Response in Absence of Matrix}}$$

$$\text{IS Normalized MF} = \frac{\text{MF}_{Analyte}}{\text{MF}_{IS}}$$

Table 12: Interference Evaluation (Presence of 150 mcg/mL Ertapenem)

Run Date	Curve Number	Comed1[2] low 0.150 µg/mL	%Bias	Comed1[2] high 37.5 µg/mL	%Bias
28-Oct-2022	2	##0.177	18.0	32.6	-13.1
		0.139	-7.3	##30.2	-19.5
		0.145	-3.3	33.6	-10.4
		0.170	13.3	34.4	-8.3
		0.157	4.7	36.6	-2.4
		##0.178	18.7	37.5	0.0
Mean		0.161		34.2	
SD		0.0166		2.67	
%CV		10.3		7.8	
%Bias		7.3		-8.8	
n		6		6	
## > 15%Bias					

Criteria: To be considered acceptable, the mean %Bias at each level was also required to be within ± 15.0% and the %CV was required to be ≤ 15.0%.

Results: The acceptance criteria were met.

Table 13: Hemolytic Effect Evaluation (2% Hemolyzation)

Run Date	Curve Number	Hemo QC 0.15 0.150 µg/mL	%Bias	Hemo QC 37.5 37.5 µg/mL	%Bias
31-Oct-2022	4	0.150	0.0	33.9	-9.6
		0.142	-5.3	35.3	-5.9
		0.147	-2.0	37.9	1.1
		0.151	0.7	36.8	-1.9
		0.134	-10.7	36.4	-2.9
		0.143	-4.7	37.0	-1.3
Mean		0.145		36.2	
SD		0.00628		1.42	
%CV		4.3		3.9	
%Bias		-3.3		-3.5	
n		6		6	

Criteria: To be considered acceptable, the mean %Bias at each level was also required to be within ± 15.0% and the %CV was required to be ≤ 15.0%.

Results: The acceptance criteria were met.

Table 14: Lipemic Effect Evaluation

Run Date	Curve Number	Lipemic QC 0.15 0.150 µg/mL	%Bias	Lipemic QC 37.5 37.5 µg/mL	%Bias
31-Oct-2022	4	0.149	-0.7	35.8	-4.5
		0.151	0.7	42.7	13.9
		0.133	-11.3	37.1	-1.1
		0.136	-9.3	33.9	-9.6
		0.157	4.7	38.1	1.6
		0.156	4.0	38.1	1.6
Mean		0.147		37.6	
SD		0.0102		2.96	
%CV		6.9		7.9	
%Bias		-2.0		0.3	
n		6		6	

Criteria: To be considered acceptable, the mean %Bias at each level was also required to be within ± 15.0% and the %CV was required to be ≤ 15.0%.

Results: The acceptance criteria were met.

Table 15: Summary of Standard Curves

Analytical Performance: Back-Calculated Concentrations (µg/mL) of Zidebactam Calibration Standard in (Human) (Plasma Ultrafiltrate) in (Study V1872106P4)

Assay Date	Analytical Run Number	Cal 0.05 0.0500 µg/mL	Cal 0.1 0.100 µg/mL	Cal 1 1.00 µg/mL	Cal 5 5.00 µg/mL	Cal 10 10.0 µg/mL	Cal 20 20.0 µg/mL	Cal 40 40.0 µg/mL	Cal 50 50.0 µg/mL
28-Oct-2022	2	0.0536	0.0976	1.07	4.97	10.3	20.3	37.4	51.6
		0.0483	0.0943	0.976	4.87	10.1	19.8	42.2	48.4
31-Oct-2022	4	0.0488	0.101	0.956	4.61	10.7	20.3	*51.4	45.0
		0.0511	0.0996	1.04	4.63	10.3	21.3	43.2	49.1
03-Nov-2022	5	0.0488	0.0953	0.981	5.06	9.54	19.7	38.2	46.6
		0.0515	0.104	1.02	5.08	9.95	20.7	43.2	52.0
Mean		0.0504	0.0986	1.01	4.87	10.1	20.4	40.8	48.8
S.D.		0.00207	0.00364	0.0435	0.208	0.390	0.592	2.82	2.74
%CV		4.1	3.7	4.3	4.3	3.9	2.9	6.9	5.6
%Bias		0.8	-1.4	1.0	-2.6	1.0	2.0	2.0	-2.4
n		6	6	6	6	6	6	5	6
Reason Deactivated									
* Excluded due to being outside acceptance criteria									

Calibration Curve Parameters for Zidebactam Calibration Standards in Human Plasma Ultrafiltrate (Study V1872106P4)

Run Date	Curve Number	A	B	C	R-Squared	LLOQ	ULOQ
28-Oct-2022	2	-0.00074	0.15368	0.00020	0.9980	0.0500	50.0
31-Oct-2022	4	-0.00064	0.17368	-0.00053	0.9967	0.0500	50.0
03-Nov-2022	5	-0.00035	0.17362	-0.00067	0.9983	0.0500	50.0

See Table 7 for runs not used for precision/accuracy evaluation.

Table 16: Precision and Accuracy (Intra-Assay)

Human Plasma Ultrafiltrate Zidebactam Concentrations in Quality Controls for 3 separate batch runs (Study V1872106P4)

Run Date	Curve Number	QC 0.05 0.0500 µg/mL	%Bias	QC 0.15 0.150 µg/mL	%Bias	QC 25 25.0 µg/mL	%Bias	QC 37.5 37.5 µg/mL	%Bias
28-Oct-2022	2	0.0576	15.2	##0.181	20.7	23.6	-5.6	40.7	8.5
		#0.0629	25.8	0.157	4.7	25.1	0.4	36.9	-1.6
		0.0506	1.2	0.159	6.0	22.1	-11.6	36.9	-1.6
		0.0543	8.6	0.171	14.0	##29.5	18.0	36.5	-2.7
		0.0539	7.8	0.172	14.7	26.0	4.0	38.0	1.3
		0.0485	-3.0	0.161	7.3	25.5	2.0	39.4	5.1
Intrarun Mean		0.0546		0.167		25.3		38.1	
Intrarun SD		0.00513		0.00935		2.50		1.67	
Intrarun %CV		9.4		5.6		9.9		4.4	
Intrarun %Bias		9.2		11.3		1.2		1.6	
n		6		6		6		6	
31-Oct-2022	4	0.0524	4.8	0.148	-1.3	26.7	6.8	40.2	7.2
		0.0495	-1.0	0.146	-2.7	26.4	5.6	42.9	14.4
		0.0564	12.8	0.160	6.7	24.9	-0.4	33.6	-10.4
		0.0549	9.8	0.162	8.0	25.3	1.2	38.6	2.9
		0.0564	12.8	0.155	3.3	25.8	3.2	37.5	0.0
		0.0555	11.0	0.160	6.7	23.5	-6.0	38.5	2.7
Intrarun Mean		0.0542		0.155		25.4		38.6	
Intrarun SD		0.00273		0.00677		1.16		3.07	
Intrarun %CV		5.0		4.4		4.6		8.0	
Intrarun %Bias		8.4		3.3		1.6		2.9	
n		6		6		6		6	
03-Nov-2022	5	0.0533	6.6	0.153	2.0	26.0	4.0	37.3	-0.5
		0.0577	15.4	0.157	4.7	25.2	0.8	38.9	3.7
		0.0531	6.2	0.150	0.0	24.9	-0.4	39.9	6.4
		0.0534	6.8	0.164	9.3	25.9	3.6	41.1	9.6
		0.0550	10.0	0.158	5.3	26.7	6.8	38.2	1.9
		0.0550	10.0	0.165	10.0	27.4	9.6	39.9	6.4
Intrarun Mean		0.0546		0.158		26.0		39.2	
Intrarun SD		0.00175		0.00591		0.928		1.36	
Intrarun %CV		3.2		3.7		3.6		3.5	
Intrarun %Bias		9.2		5.3		4.0		4.5	
n		6		6		6		6	
# > 20%Bias									
## > 15%Bias									

Criteria: At each level, the %CV was required to be ≤ 15.0%, except at the LLOQ level, where ≤ 20.0% was accepted. The mean %Bias at each level was also required to be within ± 15.0%, except at the LLOQ, where ± 20.0% was accepted.

Results: The acceptance criteria were met. Run size (n=158) was completed in run 5 and met acceptance criteria.

Table 17: Precision and Accuracy (Inter-Assay)

Human Plasma Ultrafiltrate Zidebactam Concentrations in Quality Controls for 3 separate batch runs (Study V1872106P4)

Run Date	Curve Number	QC 0.05 0.0500 µg/mL	%Bias	QC 0.15 0.150 µg/mL	%Bias	QC 25 25.0 µg/mL	%Bias	QC 37.5 37.5 µg/mL	%Bias
28-Oct-2022	2	0.0576	15.2	##0.181	20.7	23.6	-5.6	40.7	8.5
		#0.0629	25.8	0.157	4.7	25.1	0.4	36.9	-1.6
		0.0506	1.2	0.159	6.0	22.1	-11.6	36.9	-1.6
		0.0543	8.6	0.171	14.0	##29.5	18.0	36.5	-2.7
		0.0539	7.8	0.172	14.7	26.0	4.0	38.0	1.3
		0.0485	-3.0	0.161	7.3	25.5	2.0	39.4	5.1
31-Oct-2022	4	0.0524	4.8	0.148	-1.3	26.7	6.8	40.2	7.2
		0.0495	-1.0	0.146	-2.7	26.4	5.6	42.9	14.4
		0.0564	12.8	0.160	6.7	24.9	-0.4	33.6	-10.4
		0.0549	9.8	0.162	8.0	25.3	1.2	38.6	2.9
		0.0564	12.8	0.155	3.3	25.8	3.2	37.5	0.0
		0.0555	11.0	0.160	6.7	23.5	-6.0	38.5	2.7
03-Nov-2022	5	0.0533	6.6	0.153	2.0	26.0	4.0	37.3	-0.5
		0.0577	15.4	0.157	4.7	25.2	0.8	38.9	3.7
		0.0531	6.2	0.150	0.0	24.9	-0.4	39.9	6.4
		0.0534	6.8	0.164	9.3	25.9	3.6	41.1	9.6
		0.0550	10.0	0.158	5.3	26.7	6.8	38.2	1.9
		0.0550	10.0	0.165	10.0	27.4	9.6	39.9	6.4
Mean Concentration Found (µg/mL)		0.0545		0.160		25.6		38.6	
Inter-run SD		0.00330		0.00871		1.61		2.09	
Inter-run %CV		6.1		5.4		6.3		5.4	
Inter-run %Bias		9.0		6.7		2.4		2.9	
n		18		18		18		18	
# > 20%Bias									
## > 15%Bias									

Criteria: At each level, the %CV was required to be ≤ 15.0%, except at the LLOQ level, where ≤ 20.0% was accepted. In addition, the mean %Bias at each level was required to be within ± 15.0% from nominal value, except at the LLOQ, where ± 20.0% was accepted.

Results: The acceptance criteria were met.

Table 18: Precision and Accuracy of Sample Dilution

Run Date	Curve Number	Dil QC 2x (High QC) 37.5 µg/mL	%Bias	Dil QC 10x (ARQC) 400 µg/mL	%Bias
31-Oct-2022	4	39.4	5.1	394	-1.5
		40.7	8.5	397	-0.8
		37.5	0.0	423	5.8
		38.6	2.9	453	13.3
		38.4	2.4	380	-5.0
		37.9	1.1	390	-2.5
Mean		38.8		406	
SD		1.15		27.0	
%CV		3.0		6.7	
%Bias		3.5		1.5	
n		6		6	

Sample dilution as follows:

2x = 25 mcL sample + 25 mcL blank human plasma ultrafiltrate
10x = 20 mcL sample + 180 mcL blank human plasma ultrafiltrate, mix well, then pipette 50 mcL as sample

Criteria: To be deemed acceptable, the mean %Bias at each level must be within ± 15.0% with %CV ≤ 15.0%.
Results: The acceptance criteria were met.

Table 19: Freeze-Thaw Stability, (Frozen At -20 °C and -70 °C Thawed in Wet Ice)

Without Comeds[2]

Run Date	Curve Number	F/T -20 low 0.150 µg/mL	%Bias	F/T -70 low 0.150 µg/mL	%Bias	F/T -20 high 37.5 µg/mL	%Bias	F/T -70 high 37.5 µg/mL	%Bias
03-Nov-2022	5	0.151	0.7	0.151	0.7	36.3	-3.2	39.1	4.3
		0.142	-5.3	0.159	6.0	38.4	2.4	39.3	4.8
		0.155	3.3	0.157	4.7	37.1	-1.1	39.2	4.5
		0.156	4.0	0.153	2.0	38.2	1.9	40.4	7.7
		0.148	-1.3	0.145	-3.3	36.9	-1.6	38.8	3.5
		0.145	-3.3	0.151	0.7	37.5	0.0	37.8	0.8
Mean		0.150		0.153		37.4		39.1	
SD		0.00554		0.00497		0.800		0.839	
%CV		3.7		3.2		2.1		2.1	
%Bias		0.0		2.0		-0.3		4.3	
n		6		6		6		6	

With Comeds[2]

Run Date	Curve Number	Comed1 F/T -20 low 0.150 µg/mL	%Bias	Comed1 F/T -70 low 0.150 µg/mL	%Bias	Comed1 F/T -20 high 37.5 µg/mL	%Bias	Comed1 F/T -70 high 37.5 µg/mL	%Bias
03-Nov-2022	5	0.145	-3.3	0.150	0.0	38.8	3.5	39.0	4.0
		0.151	0.7	0.150	0.0	37.0	-1.3	38.0	1.3
		0.148	-1.3	0.150	0.0	36.1	-3.7	39.4	5.1
		0.138	-8.0	0.152	1.3	37.3	-0.5	37.8	0.8
		0.145	-3.3	0.147	-2.0	37.8	0.8	39.2	4.5
		0.143	-4.7	0.145	-3.3	36.1	-3.7	38.0	1.3
Mean		0.145		0.149		37.2		38.6	
SD		0.00443		0.00253		1.04		0.709	
%CV		3.1		1.7		2.8		1.8	
%Bias		-3.3		-0.7		-0.8		2.9	
n		6		6		6		6	

Criteria: To be deemed stable, the mean %Bias at each level must be within ± 15.0% with %CV ≤ 15.0%.

Results: Zidebactam is stable in treated human plasma ultrafiltrate with and without the presence of comeds[2], with sodium heparin as anticoagulant, for at least 3 cycles when frozen at -20 °C and -70 °C and thawed in wet ice.

Table 20: Bench-Top Stability in Wet Ice

Without Comeds[2]

Run Date	Curve Number	BT 24hr low 0.150 µg/mL	%Bias	BT 6hr low 0.150 µg/mL	%Bias	BT 24hr high 37.5 µg/mL	%Bias	BT 6hr high 37.5 µg/mL	%Bias
03-Nov-2022	5	0.154	2.7	0.154	2.7	36.3	-3.2	39.0	4.0
		0.159	6.0	0.162	8.0	40.8	8.8	38.6	2.9
		0.150	0.0	0.150	0.0	40.6	8.3	38.9	3.7
		0.149	-0.7	0.151	0.7	39.4	5.1	39.0	4.0
		0.141	-6.0	0.158	5.3	40.6	8.3	40.4	7.7
		0.156	4.0	0.148	-1.3	38.7	3.2	38.6	2.9
Mean		0.152		0.154		39.4		39.1	
SD		0.00635		0.00531		1.73		0.671	
%CV		4.2		3.4		4.4		1.7	
%Bias		1.3		2.7		5.1		4.3	
n		6		6		6		6	

With Comeds[2]

Run Date	Curve Number	Comed1 BT 24hr low 0.150 µg/mL	%Bias	Comed1 BT 6hr low 0.150 µg/mL	%Bias	Comed1 BT 24hr high 37.5 µg/mL	%Bias	Comed1 BT 6hr high 37.5 µg/mL	%Bias
03-Nov-2022	5	0.145	-3.3	0.145	-3.3	36.4	-2.9	37.9	1.1
		0.150	0.0	0.155	3.3	36.2	-3.5	39.3	4.8
		0.152	1.3	0.151	0.7	36.6	-2.4	37.7	0.5
		0.151	0.7	0.154	2.7	37.3	-0.5	38.0	1.3
		0.151	0.7	0.154	2.7	39.3	4.8	36.3	-3.2
		0.151	0.7	0.150	0.0	39.8	6.1	37.8	0.8
Mean		0.150		0.152		37.6		37.8	
SD		0.00253		0.00373		1.56		0.954	
%CV		1.7		2.5		4.1		2.5	
%Bias		0.0		1.3		0.3		0.8	
n		6		6		6		6	

Criteria: To be deemed stable, the mean %Bias at each level must be within ± 15.0% with %CV ≤ 15.0%.

Results: Zidebactam is stable in treated human plasma ultrafiltrate with and without the presence of comeds[2], with sodium heparin as anticoagulant, for at least 24 hours in wet ice.

Table 21: Long-Term Stability at -20 °C and -70 °C

Analytical Performance: Back-Calculated Concentrations (µg/mL) of Zidebactam Calibration Standard in (Human) (Plasma Ultrafiltrate)

Assay Date	Analytical Run Number	Cal 0.05 0.0500 µg/mL	Cal 0.1 0.100 µg/mL	Cal 1 1.00 µg/mL	Cal 5 5.00 µg/mL	Cal 10 10.0 µg/mL	Cal 20 20.0 µg/mL	Cal 40 40.0 µg/mL	Cal 50 50.0 µg/mL
06-Dec-2022	8	0.0491	0.0973	1.02	4.95	9.99	20.6	35.8	47.7
		0.0501	0.104	1.14	5.00	10.8	19.4	40.6	45.8
Mean		0.0496	0.101	1.08	4.98	10.4	20.0	38.2	46.8
%Bias		-0.8	1.0	8.0	-0.4	4.0	0.0	-4.5	-6.4

Calibration Curve Parameters for Zidebactam Calibration Standards in Human Plasma Ultrafiltrate (Study V1872106P4)

Run Date	Curve Number	Slope	Intercept	R-Squared	LLOQ	ULOQ
06-Dec-2022	8	0.20948	-0.00004	0.9958	0.0500	50.0

Analytical Performance of Zidebactam Quality Control Samples in Human Plasma Ultrafiltrate (Study V1872106P4)

Run Date	Curve Number	QC 0.15 0.150 µg/mL	%Bias	QC 25 25.0 µg/mL	%Bias	QC 37.5 37.5 µg/mL	%Bias
06-Dec-2022	8	0.144	-4.0	25.6	2.4	32.4	-13.6
		0.142	-5.3	25.3	1.2	32.9	-12.3
Mean		0.143		25.5		32.7	
%Bias		-4.7		2.0		-12.8	

Without Comeds[2]

Run Date	Curve Number	LTS -20 low 0.150 µg/mL	%Bias	LTS -70 low 0.150 µg/mL	%Bias	LTS -20 high 37.5 µg/mL	%Bias	LTS -70 high 37.5 µg/mL	%Bias
06-Dec-2022	8	#0.121	-19.3	0.165	10.0	#29.9	-20.3	38.6	2.9
		#0.126	-16.0	#0.173	15.3	#28.7	-23.5	37.0	-1.3
		0.158	5.3	0.170	13.3	32.8	-12.5	38.1	1.6
		0.151	0.7	#0.178	18.7	32.4	-13.6	36.9	-1.6
		#0.125	-16.7	0.154	2.7	#26.0	-30.7	39.5	5.3
		#0.125	-16.7	#0.188	25.3	#28.7	-23.5	40.3	7.5
Mean		0.134		0.171		29.8		38.4	
S.D.		0.0159		0.0116		2.55		1.35	
%CV		11.9		6.8		8.6		3.5	
%Bias		-10.7		14.0		-20.5		2.4	
n		6		6		6		6	
# > 15%Bias									

With Comeds[2]

Run Date	Curve Number	Comed1 LTS -20 low 0.150 µg/mL	%Bias	Comed1 LTS -70 low 0.150 µg/mL	%Bias	Comed1 LTS -20 high 37.5 µg/mL	%Bias	Comed1 LTS -70 high 37.5 µg/mL	%Bias
06-Dec-2022	8	0.131	-12.7	0.156	4.0	#29.5	-21.3	34.9	-6.9
		0.143	-4.7	0.157	4.7	32.7	-12.8	37.7	0.5
		0.141	-6.0	0.158	5.3	32.5	-13.3	42.1	12.3
		0.149	-0.7	#0.175	16.7	#31.2	-16.8	37.4	-0.3
		0.141	-6.0	#0.178	18.7	#29.4	-21.6	#30.0	-20.0
		#0.119	-20.7	0.156	4.0	31.9	-14.9	40.3	7.5
Mean		0.137		0.163		31.2		37.1	
S.D.		0.0107		0.0103		1.45		4.26	
%CV		7.8		6.3		4.6		11.5	
%Bias		-8.7		8.7		-16.8		-1.1	
n		6		6		6		6	
# > 15%Bias									

Criteria: To be deemed stable, the mean %Bias at each level must be within ± 15.0% with %CV ≤ 15.0%.

Results: Free zidebactam is stable in human plasma ultrafiltrate with and without the presence of comeds[2], with sodium heparin as anticoagulant, for up to 39 days upon storage in polypropylene tube at -70 °C.

Free zidebactam is not stable in human plasma ultrafiltrate with and without the presence of comeds[2], with sodium heparin as anticoagulant, for up to 39 days upon storage in polypropylene tube at -20 °C. Criteria not met at the high QC level for both, regular and comed QCs %Bias is low.

Table 22: Refrigerated Processed Sample Stability

Run Date	Curve Number	Processed Refr low 0.150 µg/mL	%Bias	Processed Refr high 37.5 µg/mL	%Bias
03-Nov-2022	5	0.168	12.0	41.3	10.1
		0.163	8.7	41.3	10.1
		0.171	14.0	41.4	10.4
		0.161	7.3	41.0	9.3
		0.156	4.0	40.1	6.9
		0.167	11.3	40.0	6.7
Mean		0.164		40.9	
SD		0.00543		0.635	
%CV		3.3		1.6	
%Bias		9.3		9.1	
n		6		6	

Samples were processed with run 2, and then stored for 5 days 21 hours in polypropylene 96 well plates at refrigerated temperatures prior to injection with run 5.

Criteria: To be deemed stable, the mean %Bias at each level must be within ± 15.0% with %CV ≤ 15.0%.
Results: The acceptance criteria were met.

Table 23: Individual Sample Reinjection Reproducibility

Run Date	Curve Number	Indiv Reinj low 0.150 µg/mL	%Bias	Indiv Reinj high 37.5 µg/mL	%Bias
28-Oct-2022	2	0.140	-6.7	33.5	-10.7
		##0.177	18.0	35.5	-5.3
		0.152	1.3	39.5	5.3
		##0.173	15.3	39.6	5.6
		0.158	5.3	43.0	14.7
		0.149	-0.7	39.8	6.1
Mean		0.158		38.5	
SD		0.0143		3.41	
%CV		9.1		8.9	
%Bias		5.3		2.7	
n		6		6	
## > 15%Bias					

Low and high QC extracts (6 each) were injected during the run and then reinjected at the end of the same run.
Criteria: To be deemed reproducible, the mean %Bias at each level must be within ± 15.0% with %CV ≤ 15.0%.
Results: The acceptance criteria were met.

Table 24: Whole Run Reinjection Reproducibility and Column Ruggedness

Analytical Performance: Back-Calculated Concentrations (µg/mL) of Zidebactam Calibration Standard in (Human) (Plasma Ultrafiltrate) in (Study V1872106P4)

Assay Date	Analytical Run Number	Cal 0.05 0.0500 µg/mL	Cal 0.1 0.100 µg/mL	Cal 1 1.00 µg/mL	Cal 5 5.00 µg/mL	Cal 10 10.0 µg/mL	Cal 20 20.0 µg/mL	Cal 40 40.0 µg/mL	Cal 50 50.0 µg/mL
03-Nov-2022	6	0.0496	0.0975	0.934	4.97	10.5	19.6	40.8	47.4
		0.0496	0.106	1.02	5.02	9.93	19.7	42.2	49.8
Mean		0.0496	0.102	0.977	5.00	10.2	19.7	41.5	48.6
%Bias		-0.8	2.0	-2.3	0.0	2.0	-1.5	3.8	-2.8

Calibration Curve Parameters for Zidebactam Calibration Standards in Human Plasma Ultrafiltrate (Study V1872106P4)

Run Date	Curve Number	A	B	C	R-Squared	LLOQ	ULOQ
03-Nov-2022	6	-0.00022	0.16077	-0.00023	0.9985	0.0500	50.0

Analytical Performance of Zidebactam Quality Control Samples in Human Plasma Ultrafiltrate (Study V1872106P4)

Run Date	Curve Number	QC 0.05 0.0500 µg/mL	%Bias	QC 0.15 0.150 µg/mL	%Bias	QC 25 25.0 µg/mL	%Bias	QC 37.5 37.5 µg/mL	%Bias
03-Nov-2022	6	0.0505	1.0	0.161	7.3	25.7	2.8	37.9	1.1
		0.0565	13.0	0.155	3.3	24.4	-2.4	38.8	3.5
		0.0534	6.8	0.159	6.0	25.2	0.8	38.1	1.6
		0.0570	14.0	0.158	5.3	26.0	4.0	36.4	-2.9
		0.0563	12.6	0.151	0.7	24.8	-0.8	37.8	0.8
		0.0518	3.6	0.155	3.3	27.2	8.8	37.3	-0.5
Mean		0.0543		0.157		25.6		37.7	
S.D.		0.00274		0.00356		0.995		0.808	
%CV		5.0		2.3		3.9		2.1	
%Bias		8.6		4.7		2.4		0.5	
n		6		6		6		6	

Note: Run 6 is a reinjection of run 2 on a different analytical column of the same manufacturer, dimensions, and type, using same MS platform (Applied Biosystems API5000) – reinjected within 6 days 20 hours

Criteria: At each level, the %CV was required to be ≤ 15.0% from the nominal value, except at the LLOQ level, where ≤ 20.0% was accepted. The mean %Bias at each level was also required to be within ± 15.0%, except at the LLOQ, where ± 20.0% was accepted.

Results: The acceptance criteria were met.

Table 25: Extraction Recovery – Zidebactam

Run: 3

Control		Extracted QCs		
QC Conc. *mcg/mL*	Drug Peak Area	QC Conc. *mcg/mL*	Drug Peak Area	Extraction Recovery *%*
Low QC		Low QC		
0.150	12783	0.150	10863	117.7
	12576		11447	109.9
	11373		11602	98.0
	13309		9885	134.6
	13628		10526	129.5
	13411		10575	126.8
Average	12847			119.4
Mid QC		Mid QC		
25.0	0	25.0	1529286	NA*
	1763391		1723765	102.3
	1776917		1764079	100.7
	1619281		1590265	101.8
	1649893		1673777	98.6
	1755448		1701406	103.2
				101.3
High QC		High QC		
37.5	2617356	37.5	2194707	119.3
	2463445		2475306	99.5
	2687034		2672417	100.5
	2391725		2456896	97.3
	2616319		2451732	106.7
	2388397		2136029	111.8
				105.9
				======
			n:	17
			Overall Mean Recovery:	**109.3**
			Overall Standard Deviation:	**12.0**
			Overall % CV:	**11.0**

*The control Mid QC did not contain analyte and so the extraction recovery will not be calculated for this sample.
Control QC did not contain IS, so recovery calculated with analyte peak area only

$$\%\ \text{Recovery} = \frac{\text{Response Ratio of "Extracted QC"}}{\text{Average Response Ratio of Control}} \times 100$$

Table 26: Extraction Recovery – Cefotaxime

Run: 3

Control				Extracted QCs				
WIS Conc. *50 mcg/mL*	IS Peak Area	Drug Peak Area	Peak Area Ratio	WIS Conc. *50 mcg/mL*	IS Peak Area	Drug Peak Area	Peak Area Ratio	Extraction Recovery %
	1592555	516680	3.0823		1529286	459357	3.3292	106.2
	1669499	525069	3.1796		1723765	475565	3.6247	115.7
	1685373	522444	3.2259		1764079	439576	4.0131	128.0
	1745567	520615	3.3529		1590265	457048	3.4794	111.0
	1460380	529675	2.7571		1673777	454565	3.6822	117.5
	1676033	522596	3.2071		1701406	468777	3.6295	115.8
		Average	3.1342					======
							n:	6
							Mean Recovery:	**115.7**
							Standard Deviation:	**7.3**
							%CV:	**6.3**

$$\% \text{ Recovery} = \frac{\text{Response Ratio of "Extracted QC"}}{\text{Average Response Ratio of Control}} \times 100$$

Table 27: Stability of Zidebactam Primary Solution in Deionized Water at Room Temperature

Study: V1872106U2
Run: 3
Container: Glass

Analyte	Sample Name	Sequence Number	Analyte Peak Area	IS Peak Area	Peak Area Ratio		Bench Top	Refrigerated	-20 °C Reference
Zidebactam 160 mg/mL	3 040 V1872106U2 Primary -20°C-1 1 1	40	5873361	70659	83.1226	Lot	V1872106U2-7-1 compared to Refr	V1872106U2-7-2 Compared to -20°C	V1872106U2-7-3
	3 041 V1872106U2 Primary -20°C-1 2 1	41	5955067	72984	81.5941				
	3 042 V1872106U2 Primary -20°C-1 3 1	42	6037661	72006	83.8494				
	3 043 V1872106U2 Primary -20°C-2 1 1	43	6159684	73349	83.9778				
	3 044 V1872106U2 Primary -20°C-2 2 1	44	6042317	70926	85.1918				
	3 045 V1872106U2 Primary -20°C-2 3 1	45	6229043	73072	85.2453	Avg	82.8448	87.6236	83.8302
	3 046 V1872106U2 Primary Refr-1 1 1	46	6399570	70861	90.3116	Std Dev	2.236	1.673	1.370
	3 047 V1872106U2 Primary Refr-1 2 1	47	6360185	73071	87.0412	%CV	2.7	1.9	1.6
	3 048 V1872106U2 Primary Refr-1 3 1	48	6146003	71623	85.8105	%Change	-5.5	4.5	N/A
	3 049 V1872106U2 Primary Refr-2 1 1	49	6284414	71539	87.8460				
	3 050 V1872106U2 Primary Refr-2 2 1	50	6330916	71469	88.5827				
	3 051 V1872106U2 Primary Refr-2 3 1	51	6223524	72241	86.1495	Duration	17.75 hours		
	3 052 V1872106U2 Primary BT-1 1 1	52	5820122	72929	79.8053				
	3 053 V1872106U2 Primary BT-1 2 1	53	5878923	71780	81.9020				
	3 054 V1872106U2 Primary BT-1 3 1	54	5916822	71690	82.5334				
	3 055 V1872106U2 Primary BT-2 1 1	55	6102441	70418	86.6602				
	3 056 V1872106U2 Primary BT-2 2 1	56	6029362	72377	83.3049				
	3 057 V1872106U2 Primary BT-2 3 1	57	6075182	73316	82.8630				

$$\%\text{Change} = \frac{\text{mean response of stored solutions} - \text{mean response of comparison solutions}}{\text{mean response of comparison solutions}} \times 100$$

Criteria: To be deemed stable, % Change upon storage for analyte must be within ±15.0% and %CV must be within ≤15.0%.
Meets Criteria: YES

Table 28: Stability of Zidebactam Primary Solution in Deionized Water at -20 °C

Study: V1872106U2
Run: 9
Container: Glass

Concentration: 160 mg/mL									
Stability Data (Stored)<br>Lot #:<br>Prepared:	V1872106U2-2-1<br>21-Jul-2022				Comparison Data (Fresh)<br>Lot #:<br>Prepared:	V1872106U2-28-1<br>15-Aug-2022			
Sample ID	Seq #	Drug Peak Area	IS Peak Area	Peak Area Ratio	Sample ID	Seq #	Drug Peak Area	IS Peak Area	Peak Area Ratio
9 034 V1872106U2 Primary stored-1 1 1	34	4889962	53131	92.0359	9 028 V1872106U2 Primary fresh-1 1 1	28	4929982	53808	91.6217
9 035 V1872106U2 Primary stored-1 2 1	35	4930025	55191	89.3266	9 029 V1872106U2 Primary fresh-1 2 1	29	4982471	54565	91.3126
9 036 V1872106U2 Primary stored-1 3 1	36	4936729	54750	90.1686	9 030 V1872106U2 Primary fresh-1 3 1	30	4928925	52976	93.0407
9 037 V1872106U2 Primary stored-2 1 1	37	4912770	55707	88.1895	9 031 V1872106U2 Primary fresh-2 1 1	31	5019924	54404	92.2712
9 038 V1872106U2 Primary stored-2 2 1	38	4956650	56101	88.3523	9 032 V1872106U2 Primary fresh-2 2 1	32	4992694	54774	91.1508
9 039 V1872106U2 Primary stored-2 3 1	39	4975100	55073	90.3365	9 033 V1872106U2 Primary fresh-2 3 1	33	4959224	54632	90.7751
			Mean	89.7349				Mean	91.6954
			n	6				n	6
			SD	1.4361				SD	0.8291
			%CV	1.6				%CV	0.9
%Change =	-2.1								
Duration:	25	Days							

$$\%\text{Change} = \frac{\text{mean response of stored solutions} - \text{mean response of comparison solutions}}{\text{mean response of comparison solutions}} \times 100$$

Criteria: To be deemed stable, % Change upon storage for analyte must be within ±15.0% and %CV must be within ≤15.0%.
Meets Criteria: YES

Table 28: Stability of Zidebactam Primary Solution in Deionized Water at -20 °C (Continued)

Study: V1872106U2
Run: 12
Container: Glass

Analyte	Sample Name	Sequence Number	Analyte Peak Area	IS Peak Area	Peak Area Ratio		Stored	Fresh
Zidebactam 160 mg/mL	12 057 V1872106U2 Primary fresh-1 1 1	57	6395288	43481	147.0824	Lot	V1872106U2-2-1	V1872106U2-31-1
	12 058 V1872106U2 Primary fresh-1 2 1	58	6510560	43940	148.1693	Prep Date	21-Jul-22	20-Sep-22
	12 059 V1872106U2 Primary fresh-1 3 1	59	6638070	45716	145.2023			
	12 060 V1872106U2 Primary fresh-2 1 1	60	6591301	44820	147.0616			
	12 061 V1872106U2 Primary fresh-2 2 1	61	6519385	45470	143.3777			
	12 062 V1872106U2 Primary fresh-2 3 1	62	6416012	45079	142.3282	Avg	153.1481	145.5369
	12 063 V1872106U2 Primary stored-1 1 1	63	7142353	44327	161.1287	Std Dev	7.50048	2.31139
	12 064 V1872106U2 Primary stored-1 2 1	64	7211902	46450	155.2616	%CV	4.9	1.6
	12 065 V1872106U2 Primary stored-1 3 1	65	7203453	45510	158.2829	%Change	5.2	
	12 066 V1872106U2 Primary stored-2 1 1	66	6547190	46825	139.8225			
	12 067 V1872106U2 Primary stored-2 2 1	67	6945315	45065	154.1177			
	12 068 V1872106U2 Primary stored-2 3 1	68	6751274	44926	150.2754	Duration	61	days

$$\%\text{Change} = \frac{\text{mean response of stored solutions} - \text{mean response of comparison solutions}}{\text{mean response of comparison solutions}} \times 100$$

Criteria: To be deemed stable, % Change upon storage for analyte must be within ±15.0% and %CV must be within ≤15.0%.
Meets Criteria: YES

Table 28: Stability of Zidebactam Primary Solution in Deionized Water at -20 °C (Continued)

Study: V1872106P4
Run: 7
Container: Polypropylene

Concentration: 4.0 mg/mL									
Stability Data (Stored)<br>Lot #: V1872106P4-1-1<br>Prepared: 27-Oct-2022					Comparison Data (Fresh)<br>Lot #: V1872106P4-22-1<br>Prepared: 5-Dec-2022				
Sample ID	Seq #	Drug Peak Area	IS Peak Area	Peak Area Ratio	Sample ID	Seq #	Drug Peak Area	IS Peak Area	Peak Area Ratio
7 042 V1872106P4 Primary LTS-1 1	42	2409976	288466	8.3545	7 030 V1872106P4 Primary Comp A-1 1	30	2551331	274657	9.2892
7 043 V1872106P4 Primary LTS-1 1	43	2678547	283002	9.4648	7 031 V1872106P4 Primary Comp A-1 1	31	2494102	271470	9.1874
7 044 V1872106P4 Primary LTS-1 1	44	2408567	285138	8.4470	7 032 V1872106P4 Primary Comp A-1 1	32	2485387	285866	8.6942
7 045 V1872106P4 Primary LTS-2 1	45	2523482	282874	8.9209	7 033 V1872106P4 Primary Comp A-2 1	33	2608612	283858	9.1898
7 046 V1872106P4 Primary LTS-2 1	46	2555077	270476	9.4466	7 034 V1872106P4 Primary Comp A-2 1	34	2352174	281853	8.3454
7 047 V1872106P4 Primary LTS-2 1	47	2512113	276545	9.0839	7 035 V1872106P4 Primary Comp A-2 1	35	2379728	281696	8.4479
			Mean	8.9529				Mean	8.8590
			n	6				n	6
			SD	0.4771				SD	0.4153
			%CV	5.3				%CV	4.7
%Change =	1.1								
Duration:	39	Days							

$$\%\text{Change} = \frac{\text{mean response of stored solutions} - \text{mean response of comparison solutions}}{\text{mean response of comparison solutions}} \times 100$$

Criteria: To be deemed stable, % Change upon storage for analyte must be within ±15.0% and %CV must be within ≤15.0%.
Meets Criteria: YES

Table 29: Stability of Zidebactam Spiking Solution in Deionized Water at Room Temperature

Study: V1872106U2
Run: 3
Container: Polypropylene

Analyte	Sample Name	Sequence Number	Analyte Peak Area	IS Peak Area	Peak Area Ratio		Bench Top	Refrigerated	-20 °C Reference
Zidebactam 300 mcg/mL	3 003 V1872106U2 Spike low -20°C-1 1 1	3	48648	71445	0.6809	Lot	V18721062U2-7-4 compared to Refr	V18721062U2-7-5 Compared to -20°C	V18721062U2-7-6
	3 004 V1872106U2 Spike low -20°C-1 2 1	4	47266	71530	0.6608				
	3 005 V1872106U2 Spike low -20°C-1 3 1	5	48604	71728	0.6776				
	3 006 V1872106U2 Spike low -20°C-2 1 1	6	46856	71893	0.6517				
	3 007 V1872106U2 Spike low -20°C-2 2 1	7	47254	72325	0.6534				
	3 008 V1872106U2 Spike low -20°C-2 3 1	8	47607	69761	0.6824	Avg	0.6592	0.6617	0.6678
	3 009 V1872106U2 Spike low Refr-1 1 1	9	46091	71404	0.6455	Std Dev	0.00742	0.01150	0.01413
	3 010 V1872106U2 Spike low Refr-1 2 1	10	47706	70985	0.6721	%CV	1.1	1.7	2.1
	3 011 V1872106U2 Spike low Refr-1 3 1	11	46231	70901	0.6521	%Change	-0.4	-0.9	N/A
	3 012 V1872106U2 Spike low Refr-2 1 1	12	47641	72348	0.6585				
	3 013 V1872106U2 Spike low Refr-2 2 1	13	48275	71681	0.6735				
	3 014 V1872106U2 Spike low Refr-2 3 1	14	47749	71398	0.6688	Duration	17.75 hours		
	3 015 V1872106U2 Spike low RT-1 1 1	15	46353	70274	0.6596				
	3 016 V1872106U2 Spike low RT-1 2 1	16	46100	71065	0.6487				
	3 017 V1872106U2 Spike low RT-1 3 1	17	46824	70742	0.6619				
	3 018 V1872106U2 Spike low RT-2 1 1	18	47068	70552	0.6671				
	3 019 V1872106U2 Spike low RT-2 2 1	19	46743	71676	0.6521				
	3 020 V1872106U2 Spike low RT-2 3 1	20	47764	71727	0.6659				

Criteria: To be deemed stable, % Change upon storage for analyte must be within ±15.0% and %CV must be within ≤15.0%.
Meets Criteria: YES

Table 29: Stability of Zidebactam Spiking Solution in Deionized Water at Room Temperature (Continued)

Study: V1872106U2
Run: 3
Container: Polypropylene

Analyte	Sample Name	Sequence Number	Analyte Peak Area	IS Peak Area	Peak Area Ratio		Bench Top	Refrigerated	-20 °C Reference
Zidebactam 30,000 mcg/mL	3 021 V1872106U2 Spike high -20°C-1 1 1	21	4572243	70263	65.0733	Lot	V18721062U2-8-1 compared to Refr	V18721062U2-8-2 Compared to -20°C	V18721062U2-8-3
	3 022 V1872106U2 Spike high -20°C-1 2 1	22	4491468	71708	62.6355				
	3 023 V1872106U2 Spike high -20°C-1 3 1	23	4518595	71162	63.4973				
	3 024 V1872106U2 Spike high -20°C-2 1 1	24	4459474	71160	62.6683				
	3 025 V1872106U2 Spike high -20°C-2 2 1	25	4567708	71493	63.8903				
	3 026 V1872106U2 Spike high -20°C-2 3 1	26	4443614	71038	62.5526	Avg	64.4876	64.0624	63.3862
	3 027 V1872106U2 Spike high Refr-1 1 1	27	4384160	71276	61.5096	Std Dev	0.65091	2.68461	0.98858
	3 028 V1872106U2 Spike high Refr-1 2 1	28	4294992	70371	61.0336	%CV	1.0	4.2	1.6
	3 029 V1872106U2 Spike high Refr-1 3 1	29	4491832	71960	62.4212	%Change	0.7	1.1	N/A
	3 030 V1872106U2 Spike high Refr-2 1 1	30	4673188	70197	66.5725				
	3 031 V1872106U2 Spike high Refr-2 2 1	31	4760824	71302	66.7699				
	3 032 V1872106U2 Spike high Refr-2 3 1	32	4733414	71645	66.0676	Duration	17.75 hours		
	3 033 V1872106U2 Spike high RT-1 1 1	33	4528619	70459	64.2731				
	3 034 V1872106U2 Spike high RT-1 2 1	34	4641357	73083	63.5080				
	3 035 V1872106U2 Spike high RT-1 3 1	35	4610917	71399	64.5796				
	3 036 V1872106U2 Spike high RT-2 1 1	36	4619486	71895	64.2532				
	3 037 V1872106U2 Spike high RT-2 2 1	37	4659281	71204	65.4357				
	3 038 V1872106U2 Spike high RT-2 3 1	38	4668585	71962	64.8757				

$$\%\text{Change} = \frac{\text{mean response of stored solutions} - \text{mean response of comparison solutions}}{\text{mean response of comparison solutions}} \times 100$$

Criteria: To be deemed stable, % Change upon storage for analyte must be within ±15.0% and %CV must be within ≤15.0%.
Meets Criteria: YES

Table 30: Stability of Zidebactam Spiking Solution in Deionized Water at -20 °C

Study: V1872106U2
Run: 9
Container: Polypropylene

Concentration: 300 mcg/mL									
Stability Data (Stored)<br>Lot #: V1872106U2-4-1<br>Prepared: 21-Jul-2022					Comparison Data (Fresh)<br>Lot #: V1872106U2-22-1<br>Prepared: 15-Aug-2022				
Sample ID	Seq #	Drug Peak Area	IS Peak Area	Peak Area Ratio	Sample ID	Seq #	Drug Peak Area	IS Peak Area	Peak Area Ratio
9 009 V1872106U2 Spike low stored-1 1 1	9	38263	53582	0.7141	9 003 V1872106U2 Spike low fresh-1 1 1	3	40256	54025	0.7451
9 010 V1872106U2 Spike low stored-1 2 1	10	38757	54465	0.7116	9 004 V1872106U2 Spike low fresh-1 2 1	4	39981	54394	0.7350
9 011 V1872106U2 Spike low stored-1 3 1	11	38892	54006	0.7201	9 005 V1872106U2 Spike low fresh-1 3 1	5	39548	53018	0.7459
9 012 V1872106U2 Spike low stored-2 1 1	12	38202	53365	0.7159	9 006 V1872106U2 Spike low fresh-2 1 1	6	40384	52849	0.7641
9 013 V1872106U2 Spike low stored-2 2 1	13	39876	54343	0.7338	9 007 V1872106U2 Spike low fresh-2 2 1	7	40030	54425	0.7355
9 014 V1872106U2 Spike low stored-2 3 1	14	37889	54140	0.6998	9 008 V1872106U2 Spike low fresh-2 3 1	8	39846	54579	0.7301
			Mean	0.7159				Mean	0.7426
			n	6				n	6
			SD	0.0111				SD	0.0122
			%CV	1.6				%CV	1.6
%Change =	-3.6								
Duration:	25	Days							

$$\%\text{Change} = \frac{\text{mean response of stored solutions} - \text{mean response of comparison solutions}}{\text{mean response of comparison solutions}} \times 100$$

Criteria: To be deemed stable, % Change upon storage for analyte must be within ±15.0% and %CV must be within ≤15.0%.
Meets Criteria: YES

Table 30: Stability of Zidebactam Spiking Solution in Deionized Water at -20 °C (Continued)

Study: V1872106U2
Run: 9
Container: Polypropylene

Concentration: 30,000 mcg/mL									
Stability Data (Stored)<br>Lot #: V1872106U2-4-1<br>Prepared: 21-Jul-2022					Comparison Data (Fresh)<br>Lot #: V1872106U2-22-1<br>Prepared: 15-Aug-2022				
Sample ID	Seq #	Drug Peak Area	IS Peak Area	Peak Area Ratio	Sample ID	Seq #	Drug Peak Area	IS Peak Area	Peak Area Ratio
9 021 V1872106U2 Spike high stored-1 1 1	21	3782495	54413	69.5145	9 015 V1872106U2 Spike high fresh-1 1 1	15	3759255	53829	69.8370
9 022 V1872106U2 Spike high stored-1 2 1	22	3741223	54289	68.9131	9 016 V1872106U2 Spike high fresh-1 2 1	16	3793491	54048	70.1874
9 023 V1872106U2 Spike high stored-1 3 1	23	3741273	54767	68.3125	9 017 V1872106U2 Spike high fresh-1 3 1	17	3762946	55443	67.8705
9 024 V1872106U2 Spike high stored-2 1 1	24	3519263	54803	64.2166	9 018 V1872106U2 Spike high fresh-2 1 1	18	3813334	55022	69.3056
9 025 V1872106U2 Spike high stored-2 2 1	25	3627002	54256	66.8498	9 019 V1872106U2 Spike high fresh-2 2 1	19	3775447	54443	69.3468
9 026 V1872106U2 Spike high stored-2 3 1	26	3591727	54910	65.4112	9 020 V1872106U2 Spike high fresh-2 3 1	20	3809358	53956	70.6012
			Mean	67.2030				Mean	69.5248
			n	6				n	6
			SD	2.0858				SD	0.9499
			%CV	3.1				%CV	1.4
%Change =	-3.3								
Duration:	25	Days							

$$\%\text{Change} = \frac{\text{mean response of stored solutions} - \text{mean response of comparison solutions}}{\text{mean response of comparison solutions}} \times 100$$

Criteria: To be deemed stable, % Change upon storage for analyte must be within ±15.0% and %CV must be within ≤15.0%.
Meets Criteria: YES

Table 30: Stability of Zidebactam Spiking Solution in Deionized Water at -20 °C (Continued)

Study: V1872106U2
Run: 12
Container: Polypropylene

Analyte	Sample Name	Sequence Number	Analyte Peak Area	IS Peak Area	Peak Area Ratio		Stored	Fresh
Zidebactam 30.0 mcg/mL	12 031 V1872106U2 Spike low fresh-1 1 1	31	61273	41966	1.4601	Lot	V1872106U2-4-1	V1872106U2-34-1
	12 032 V1872106U2 Spike low fresh-1 2 1	32	65893	43994	1.4978	Prep Date	21-Jul-22	20-Sep-22
	12 033 V1872106U2 Spike low fresh-1 3 1	33	65670	44788	1.4662			
	12 034 V1872106U2 Spike low fresh-2 1 1	34	62602	44627	1.4028			
	12 035 V1872106U2 Spike low fresh-2 2 1	35	61173	44881	1.3630			
	12 036 V1872106U2 Spike low fresh-2 3 1	36	63454	44837	1.4152	Avg	1.2110	1.4342
	12 037 V1872106U2 Spike low stored-1 1 1	37	56861	44926	1.2657	Std Dev	0.05267	0.04930
	12 038 V1872106U2 Spike low stored-1 2 1	38	57797	44849	1.2887	%CV	4.3	3.4
	12 039 V1872106U2 Spike low stored-1 3 1	39	55195	46147	1.1961	%Change	-15.6	
	12 040 V1872106U2 Spike low stored-2 1 1	40	52701	44774	1.1770			
	12 041 V1872106U2 Spike low stored-2 2 1	41	53169	45499	1.1686			
	12 042 V1872106U2 Spike low stored-2 3 1	42	52331	44722	1.1701	Duration	61	days
Zidebactam 3,000 mcg/mL	12 044 V1872106U2 Spike high fresh-1 1 1	44	5070025	44066	115.0553	Lot	V1872106U2-4-1	V1872106U2-34-1
	12 045 V1872106U2 Spike high fresh-1 2 1	45	5297563	44976	117.7864	Prep Date	21-Jul-22	20-Sep-22
	12 046 V1872106U2 Spike high fresh-1 3 1	46	5549046	42693	129.9755			
	12 047 V1872106U2 Spike high fresh-2 1 1	47	5610729	43504	128.9704			
	12 048 V1872106U2 Spike high fresh-2 2 1	48	4763342	43658	109.1058			
	12 049 V1872106U2 Spike high fresh-2 3 1	49	5160410	43236	119.3545	Avg	111.6635	120.0413
	12 050 V1872106U2 Spike high stored-1 1 1	50	5001460	45790	109.2260	Std Dev	6.65019	8.10456
	12 051 V1872106U2 Spike high stored-1 2 1	51	4952186	44738	110.6931	%CV	6.0	6.8
	12 052 V1872106U2 Spike high stored-1 3 1	52	5187790	44557	116.4304	%Change	-7.0	
	12 053 V1872106U2 Spike high stored-2 1 1	53	4618099	45003	102.6176			
	12 054 V1872106U2 Spike high stored-2 2 1	54	4955409	45389	109.1764			
	12 055 V1872106U2 Spike high stored-2 3 1	55	5328551	43735	121.8372	Duration	61	days

$$\%\text{Change} = \frac{\text{mean response of stored solutions} - \text{mean response of comparison solutions}}{\text{mean response of comparison solutions}} \times 100$$

Criteria: To be deemed stable, % Change upon storage for analyte must be within ±15.0% and %CV must be within ≤15.0%.
Meets Criteria: YES for the high concentration, but not at the low concentration. Spikes should only be used for the 25 days established previously.

Table 31: Stability of Cefotaxime Working Internal Standard Solution in Deionized Water at Room Temperature

Study: V1872106U1
Run 3
Container: Glass

Analyte	Sample Name	Sequence Number	Analyte Peak Area	IS Peak Area	Peak Area Ratio		Bench Top	Refrigerated	-20 °C Reference
Cefotaxime	3 059 V1872106U1 WIS -20°C-1 1 1	59	1313192	485422	2.7053	Lot	V1872106U1-11-1	V1872106U1-11-2	V1872106U1-11-3
~500 mcg/mL	3 060 V1872106U1 WIS -20°C-1 3 1	60	1339652	522363	2.5646		compared to Refr	Compared to -20°C	
	3 061 V1872106U1 WIS -20°C-1 2 1	61	1340502	550053	2.4370				
	3 062 V1872106U1 WIS -20°C-2 2 1	62	1336674	522445	2.5585				
	3 063 V1872106U1 WIS -20°C-2 3 1	63	1341412	560588	2.3929				
	3 064 V1872106U1 WIS -20°C-2 1 1	64	1343379	587756	2.2856	Avg	2.5534	2.7884	2.4906
	3 065 V1872106U1 WIS Refr-1 1 1	65	1335207	450429	2.9643	Std Dev	0.129	0.232	0.149
	3 066 V1872106U1 WIS Refr-1 3 1	66	1348441	467684	2.8832	%CV	5.0	8.3	6.0
	3 067 V1872106U1 WIS Refr-1 2 1	67	1335092	554007	2.4099	%Change	-8.4	12.0	N/A
	3 068 V1872106U1 WIS Refr-2 2 1	68	1321946	453246	2.9166				
	3 069 V1872106U1 WIS Refr-2 3 1	69	1326727	447448	2.9651				
	3 070 V1872106U1 WIS Refr-2 1 1	70	1335122	515294	2.5910	Duration	15.5 hours		
	3 071 V1872106U1 WIS RT-1 1 1	71	1336567	547268	2.4423				
	3 072 V1872106U1 WIS RT-1 2 1	72	1341696	504955	2.6571				
	3 073 V1872106U1 WIS RT-1 3 1	73	1329061	557140	2.3855				
	3 074 V1872106U1 WIS RT-2 3 1	74	1340202	529252	2.5323				
	3 075 V1872106U1 WIS RT-2 2 1	75	1319308	483530	2.7285				
	3 076 V1872106U1 WIS RT-2 1 1	76	1347636	523394	2.5748				

$$\%\text{Change} = \frac{\text{mean response of stored solutions} - \text{mean response of comparison solutions}}{\text{mean response of comparison solutions}} \times 100$$

Criteria: To be deemed stable, % Change upon storage for analyte must be within ±20.0% and %CV must be within ≤15.0%.
Meets Criteria: YES

17. FIGURES

Figure 1: Typical Calibration Curve

Analytical Run 2 Run Acceptance Parameters for Zidebactam (µg/mL) (Study V1872106P4)

Regression Type	QUADRATIC
Weighting Factor	1/X**2
A	-0.000741836230156934
B	0.153683756633259
C	0.000199909296646651
R-Squared	0.997957925860746
LLOQ	0.05
ULOQ	50
Mean Std %Diff	3.5
S.D. Std %Diff	2.2
Mean Std Response	0.142770
S.D. Std Response	0.015701

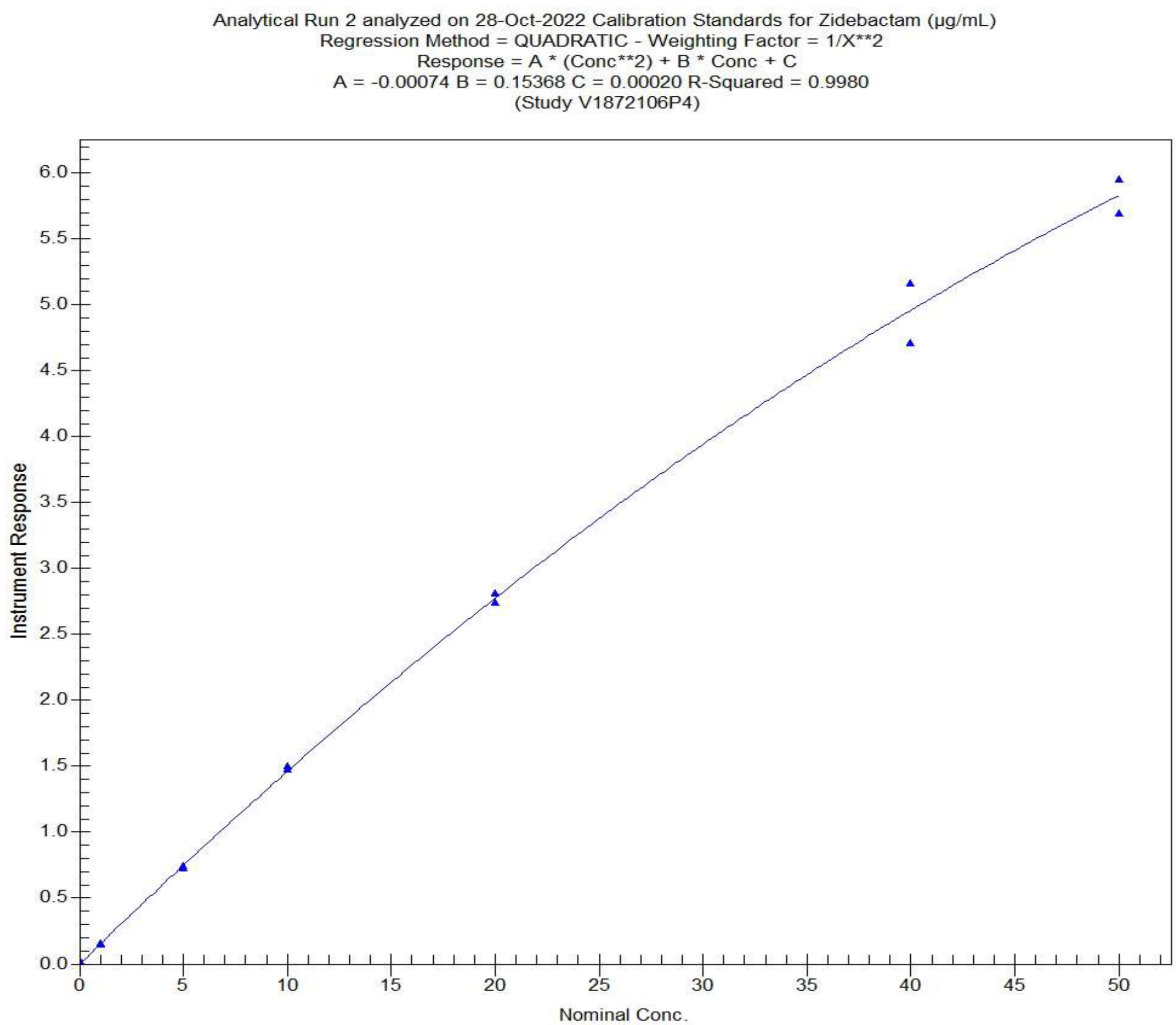

Figure 2: Typical Ion Chromatogram Blank Human Plasma Ultrafiltrate Without Internal Standard

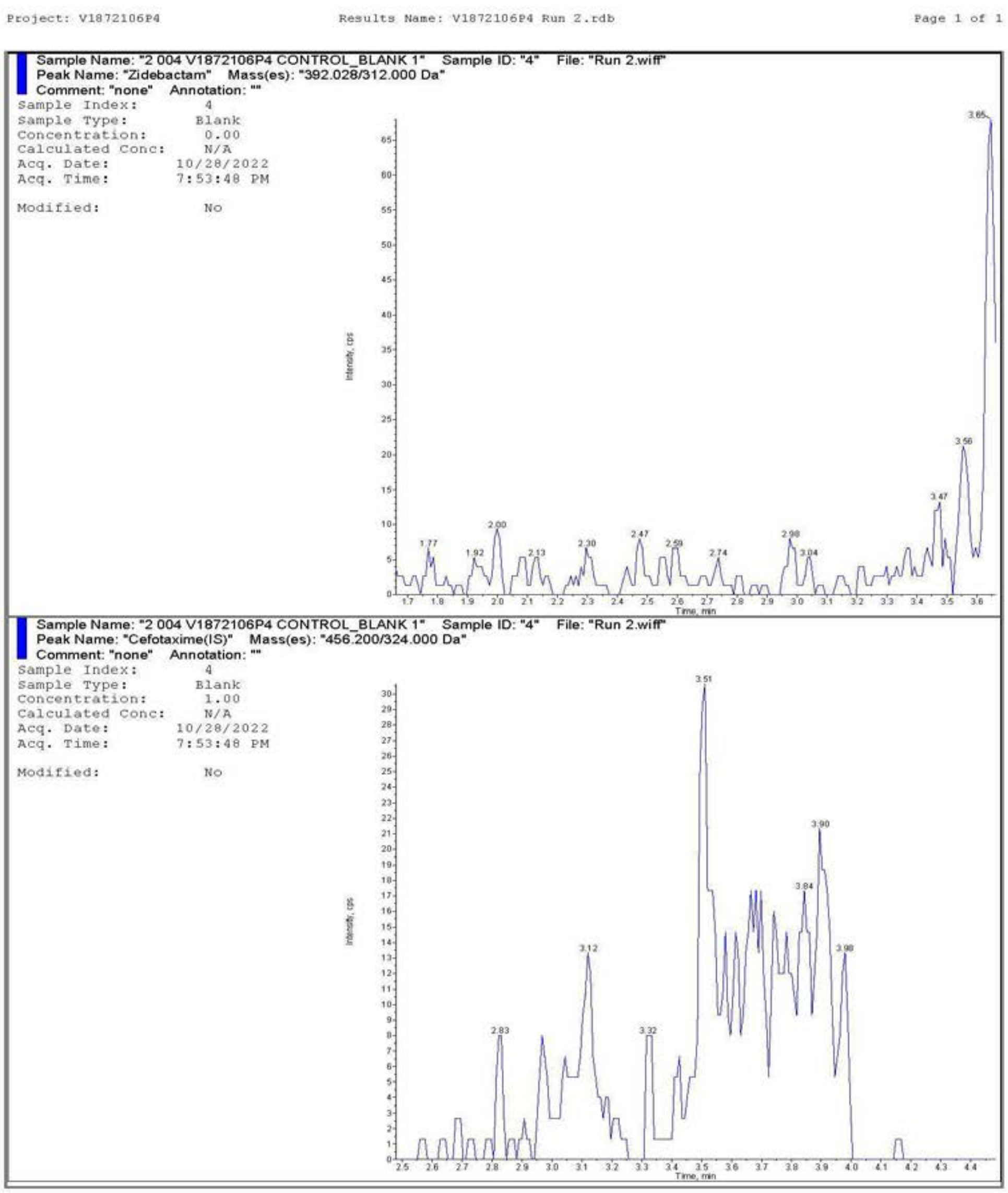

Figure 3: Typical Ion Chromatogram Blank Human Plasma Ultrafiltrate With Internal Standard

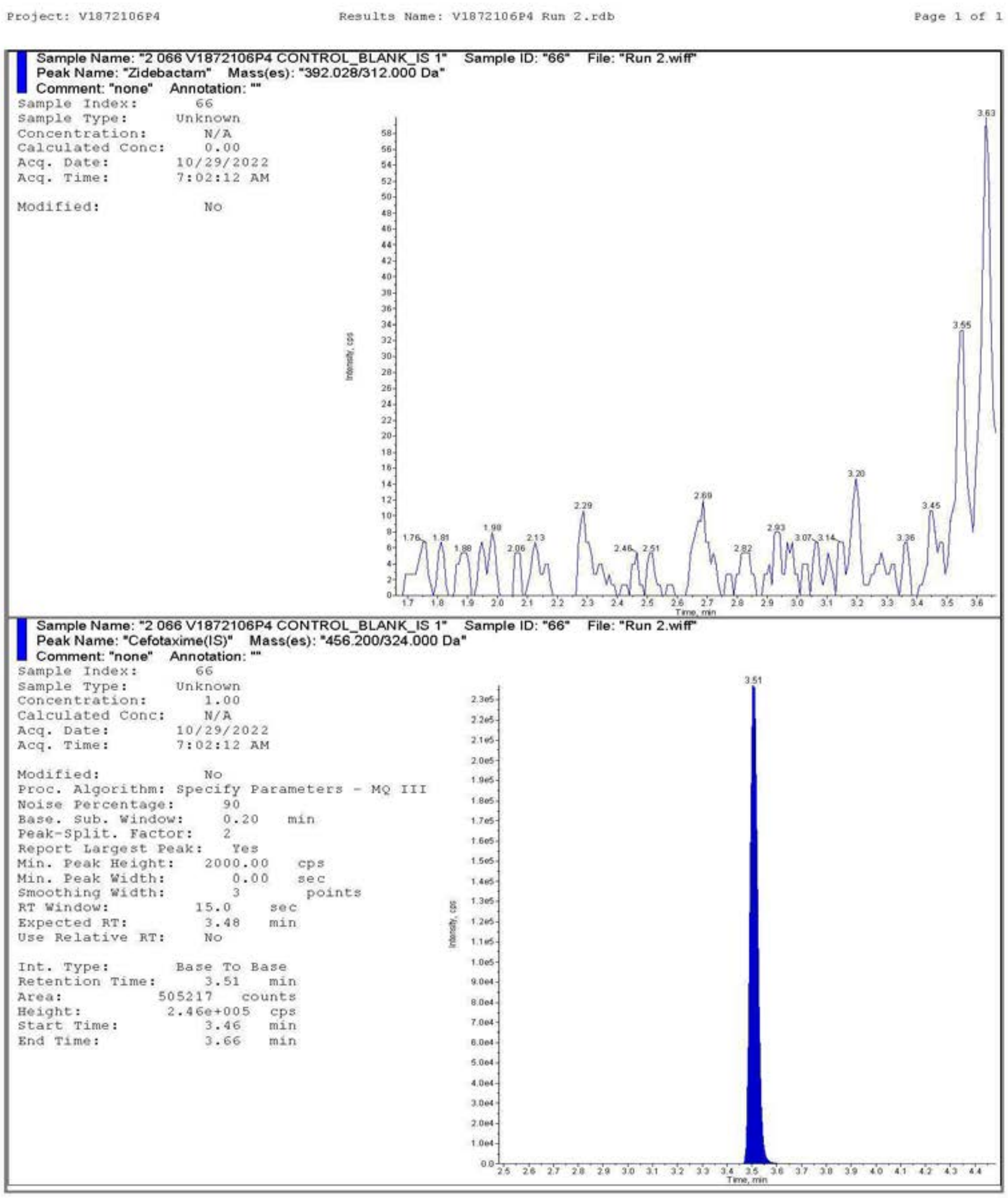

Figure 4: Typical Ion Chromatogram LLOQ Calibration Standard, Zidebactam = 0.0500 mcg/mL

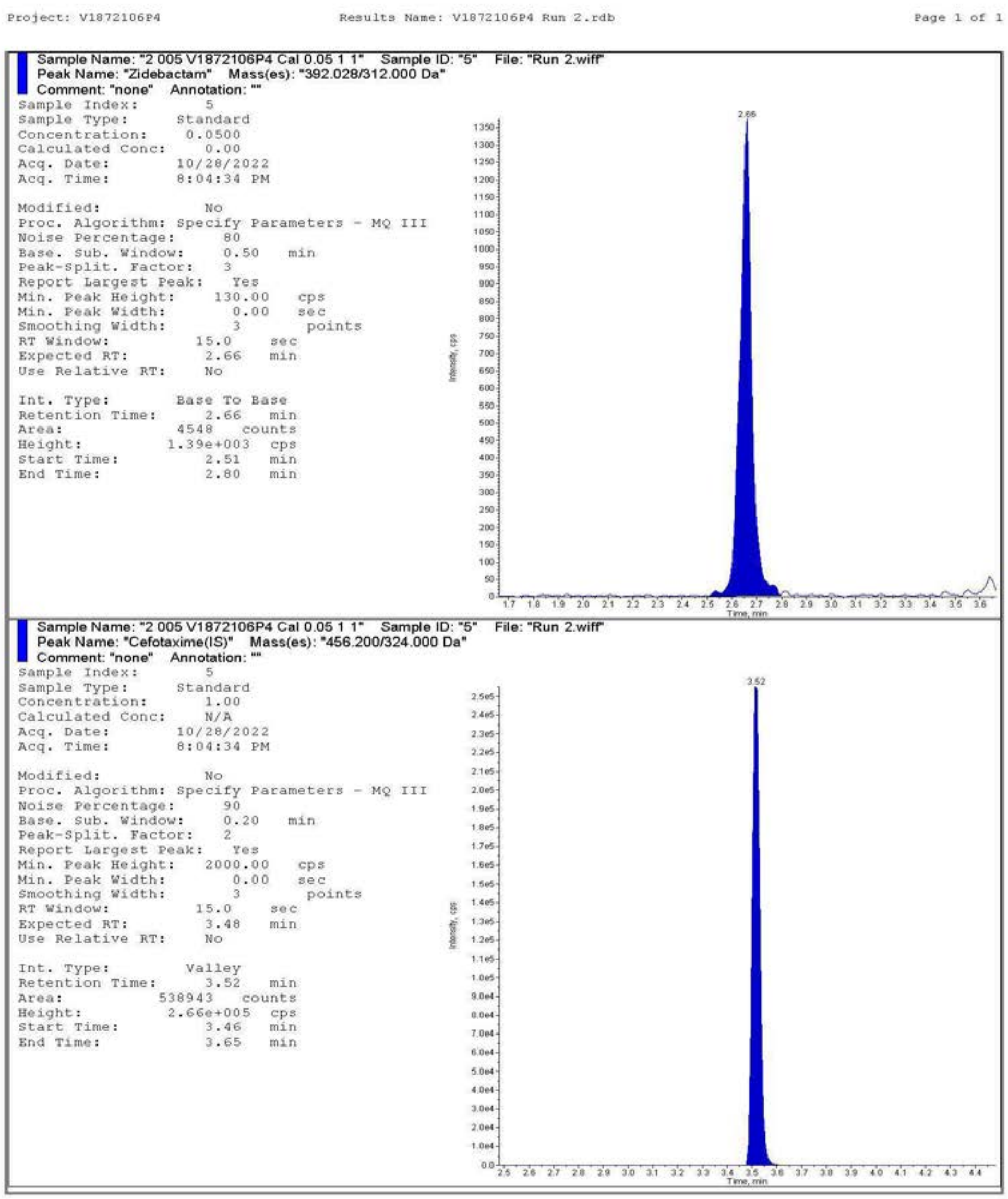

Figure 5: Typical Ion Chromatogram Calibration Standard, Zidebactam = 20.0 mcg/mL

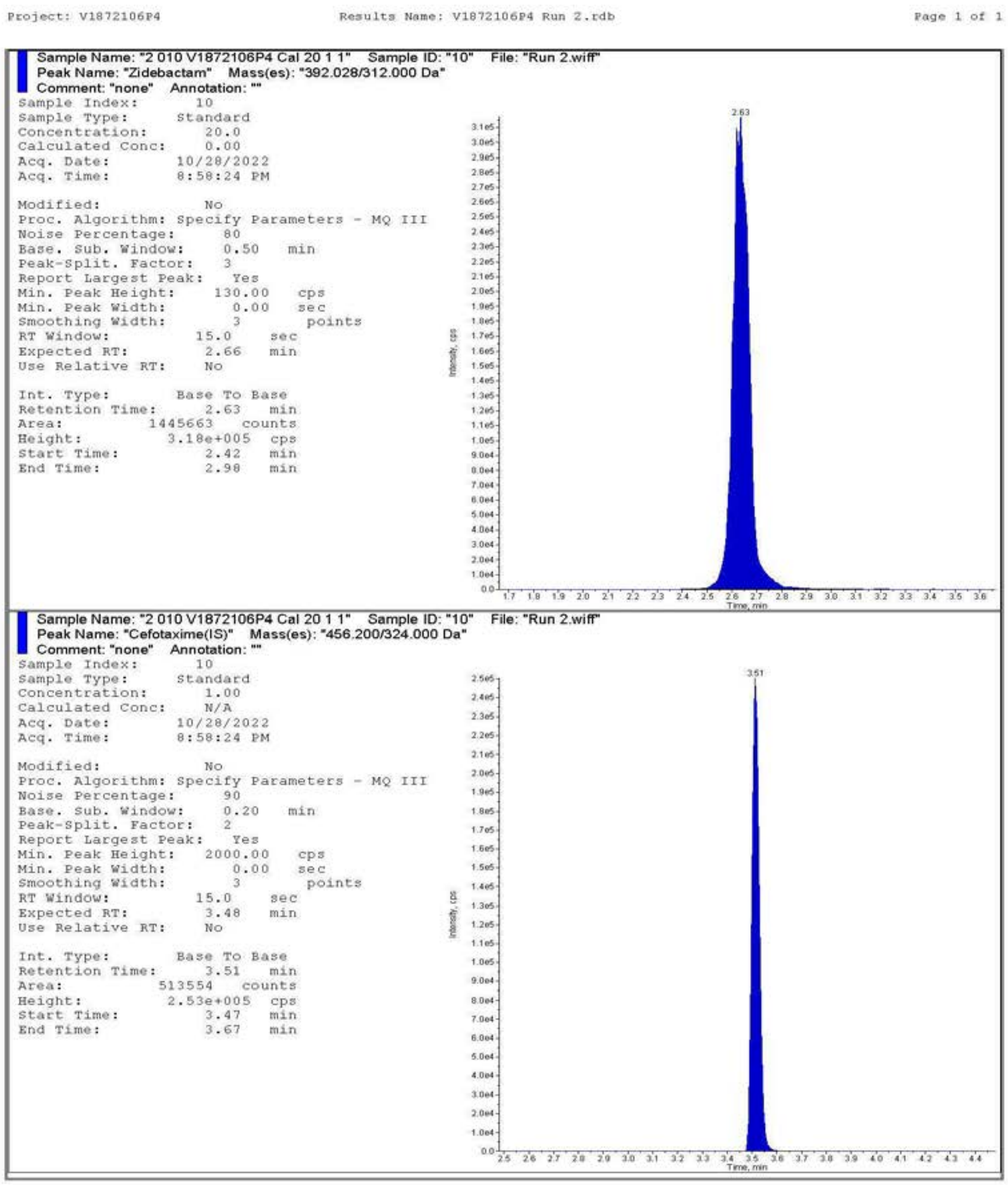

Figure 6: Typical Ion Chromatogram ULOQ Calibration Standard, Zidebactam = 50.0 mcg/mL

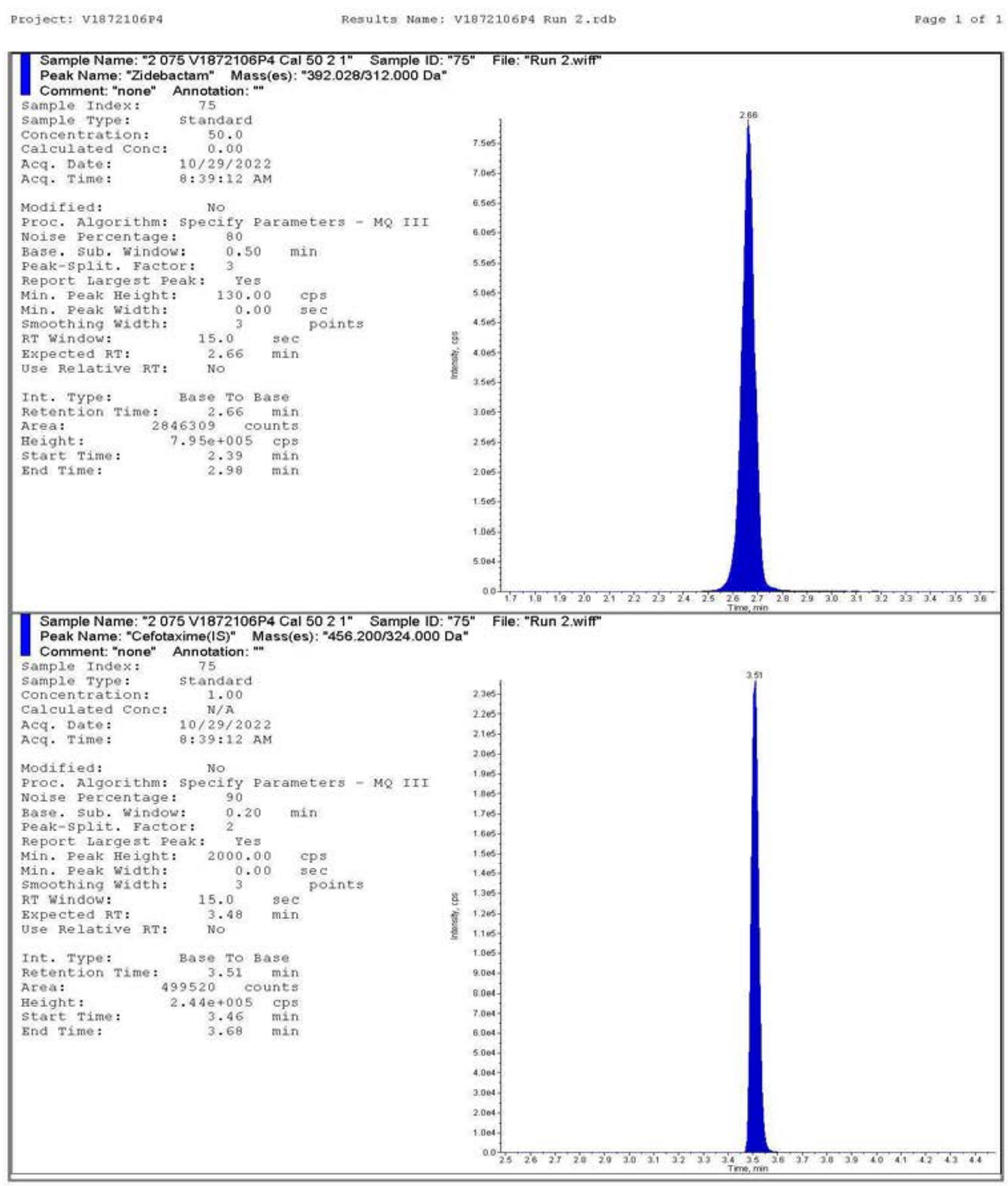

Figure 7: Typical Ion Chromatogram Cross-Talk Evaluation High QC No IS

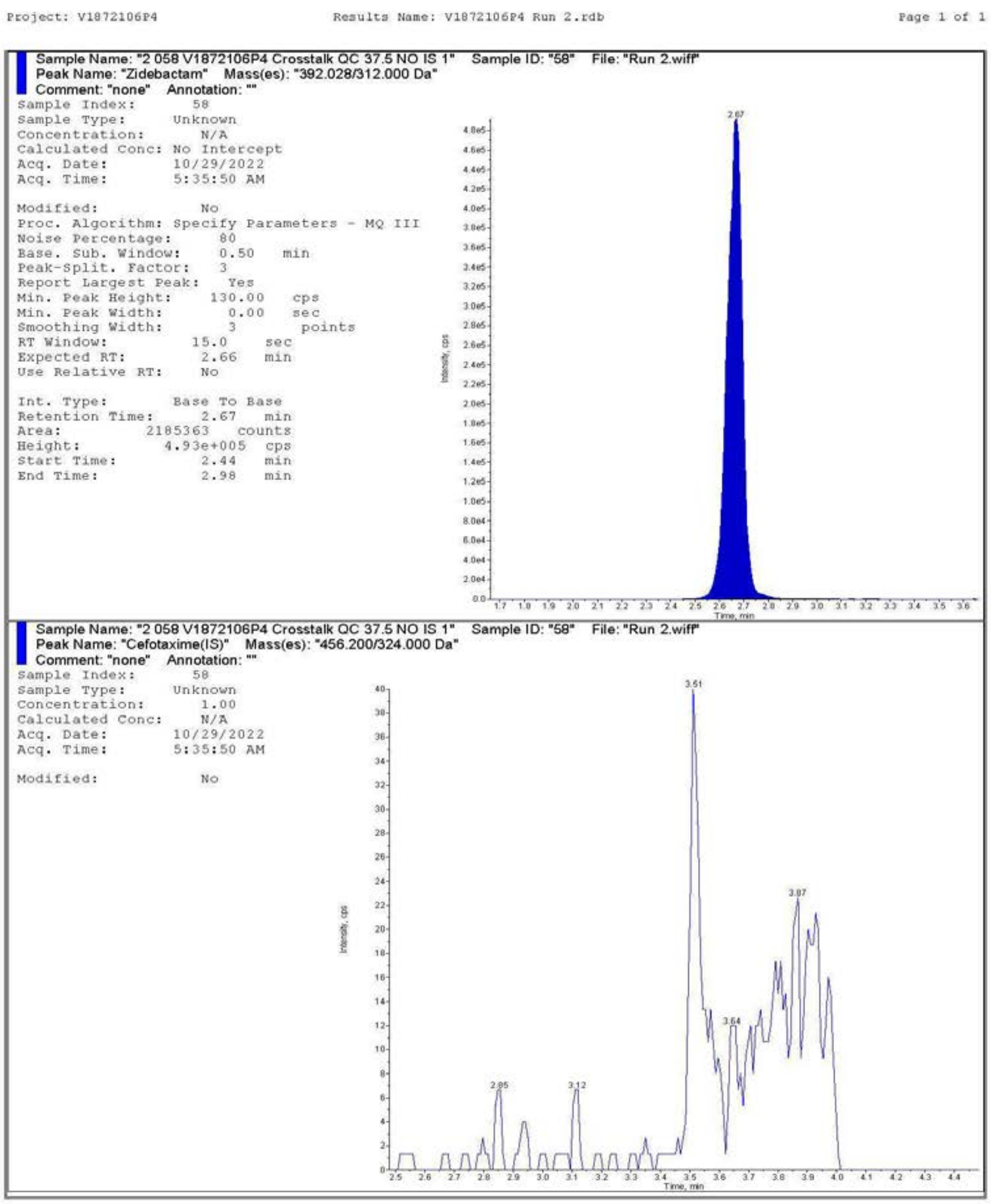

Title: **LC-MS/MS Analysis of Free Zidebactam in Treated Human Plasma Ultrafiltrate (Sodium Heparin, 50 mcL, 0.0500 – 50.0 mcg/mL; Prior to MES Treatment)**
Validation Number: V1872106P4

1. GENERAL INFORMATION

1.1. Client Name: Dynport Vaccine Company

1.2. Reference: Validation V1872106P4

2. TABLE OF CONTENTS

1. GENERAL INFORMATION 1
1.1. Client Name: Dynport Vaccine Company 1
1.2. Reference: Validation V1872106P4 1
2. TABLE OF CONTENTS 1
3. SAMPLE REQUIREMENTS 3
4. METHOD SUMMARY 3
5. SPECIAL HANDLING OF BIOLOGICAL SAMPLES 3
6. DATA COLLECTION AND ANALYSIS SOFTWARE 3
7. INSTRUMENTS, EQUIPMENT, AND CONDITIONS 3
7.1. LC and MS Conditions 4
Table 1: HPLC System 4
7.1.1. Gradient Table 5
7.1.2. Autosampler Settings 5
7.1.3. Mass Spectrometer System 5
8. REAGENTS, STANDARDS, AND SUPPLIES 6
Table 2: Reagents, Standards, and Supplies 7
9. SAFETY 7
10. PROCEDURE 8
10.1. Preparation of Analyte Stock Solutions and Dilutions 8

CONFIDENTIAL

Serial Number: 1b7b4052-15d8-48b3-8e5f-a2b9525d9398 accessed by Anika Pippin (PIPPINA) on Mon, 28 Nov 2022 23:04:06 GMT

10.1.1. Zidebactam Primary Solution, 4.00 mg/mL (Prepare on wet ice) 8
10.1.2. Working Internal Standard Solution, ~50 mcg/mL Cefotaxime 8
10.2. Preparation of Other Reagents 8
10.2.1. Mobile Phase A: 10 mM Ammonium Acetate in Deionized Water 8
10.2.2. Mobile Phase B: 95/5 Acetonitrile / 10mM Ammonium Acetate 8
10.2.3. Rinse: 90/10 Acetone/Deionized Water 9
10.2.4. 1 N Sodium Hydroxide (NaOH) in Deionized Water 9
10.2.5. MES (2-(N-Morpholino) Ethanesulphonic Acid Hydrate) Buffer pH 6.4, ~0.1M....9
10.2.6. 5/95 Acetonitrile/Deionized Water 9
10.3. Preparation of Standards and QC Samples 9
10.3.1. Preparation of Standard and QC Spiking Solutions on Wet Ice 9
10.3.2. Treated Human Plasma Ultrafiltrate (Ultrafiltrate : MES Buffer 1:1) 9
10.3.3. Preparation of Standards and QC Samples in Human Plasma Ultrafiltrate....10
10.3.4. Ultrafiltration of Human Plasma Samples using Centrifugal Filters 10
10.4. Extraction Procedure 11
10.5. Dilutions 11
10.6. Calculations 12
11. REFERENCE CHROMATOGRAMS 12
Figure 1: Blank with IS 12
Figure 2: Carryover Blank with IS 13
Figure 3: LLOQ – Zidebactam 0.050 mcg/mL 13
12. REVISION HISTORY 14
Table 3: Revision History 14

CONFIDENTIAL

Serial Number: 1b7b4052-15d8-48b3-8e5f-a2b9525d9398 accessed by Anika Pippin (PIPPINA) on Mon, 28 Nov 2022 23:04:06 GMT

3. SAMPLE REQUIREMENTS

Ultrafiltered human sodium heparin plasma samples (treated with 1:1 MES Buffer, pH 6.4, ~0.1M) minimum volume 50 mcL each, for determination of free zidebactam concentration.

Refer to the relevant study protocol for a description of expected samples.

4. METHOD SUMMARY

The method involves 96-well protein precipitation followed by chromatographic separation and tandem MS detection using the Sciex 5000 LC/MS/MS system. The concentration range is 0.0500 – 50.0 mcg/mL in human plasma ultrafiltrate only. Since unknown samples are treated with the MES buffer after collection at a 1:1 ratio, the calibration standard and QC concentrations are based on the ultrafiltrate volume only. Unknown samples do not need to be adjusted for the dilution with MES buffer.

5. SPECIAL HANDLING OF BIOLOGICAL SAMPLES

The method requires no special handling. Please ensure all "Universal Precautions" are taken.

Primaries, spiking solutions, calibration standards and QCs should be prepared on wet ice, and each prepared level should be aliquoted immediately and aliquots should be flash frozen in a dry-ice/acetone bath for ~1 minute and stored in the freezer immediately after flash freezing.

6. DATA COLLECTION AND ANALYSIS SOFTWARE

Description	Version
Watson LIMS™ (Thermo Fisher Scientific)	7.5 or higher
Analyst® (AB Sciex)	1.7.2 or higher

7. INSTRUMENTS, EQUIPMENT, AND CONDITIONS

Equivalent equipment can be substituted, except where noted in Section 7.1. Minor modifications of conditions are permitted to optimize chromatography, except where noted in

CONFIDENTIAL

Serial Number: 1b7b4052-15d8-48b3-8e5f-a2b9525d9398 accessed by Anika Pippin (PIPPINA) on Mon, 28 Nov 2022 23:04:06 GMT

Section 7.1. Refer to SOP-00073 for allowable minor modifications that do not require validation.

7.1. LC and MS Conditions

Table 1: HPLC System

**System Controller**	**Shimadzu Controller, CBM20A**
Pump	Shimadzu LC-30AD
Autosampler	Shimadzu SIL-30AC
Column Oven	Shimadzu CTO-30A
MS Interface	ESI
Analytical Column	Fortis Technology Ltd. Fortis C18 4.6 x 50mm, 5 µ
Column Guard Cartridge	Security Guard Cartridge AQ C18, 4.0 x 2.0 mm
Column Temperature (set at)	35°C
Mobile Phase A	10 mM Ammonium Acetate in Deionized Water
Mobile Phase B	95/5 Acetonitrile / 10 mM Ammonium Acetate
Needle Wash Solvent (R0, R3)	90/10 Acetone /Deionized Water
Total Run Time (approximate)	7.9 min
Injection Volume (µl)	1-10 mcL
Flow Rate	1.0 mL/min
Pressure, kgf/cm2 (approximate)	~250
Approximate Retention Time (min.)	Zidebactam: 1.80
	Cefotaxime: 3.50

CONFIDENTIAL

Serial Number: 1b7b4052-15d8-48b3-8e5f-a2b9525d9398 accessed by Anika Pippin (PIPPINA) on Mon, 28 Nov 2022 23:04:06 GMT

7.1.1. Gradient Table

Time Program

Time	Module	Events	Parameter
1.00	Controller	Event 1*	
1.50	Pumps	Pump B Conc.	2
2.50	Pumps	Pump B Conc.	16
3.80	Pumps	Pump B Conc.	100
4.00	Controller	Event 2*	
5.80	Pumps	Pump B Conc.	100
6.00	Pumps	Pump B Conc.	2
7.00	System Controller	Stop	

* Mobile phase flow is directed to the mass spectrometer from 1.00 min (Event 1) to 4.00 min (Event 2). The rest of the gradient time, the mobile phase flow goes to waste.

7.1.2. Autosampler Settings

Temperature:	Set at 4°C
Rinse Type:	External
Rinse Mode:	Before and after aspiration
Rinse Dip Time:	2 sec
Rinse Method:	Rinse Pump Then Port
Rinse Time:	2 sec
Rinsing Speed:	35 µL/sec
Rinsing Volume:	800 µL

7.1.3. Mass Spectrometer System

Mass Spectrometer	Sciex 5000
Source Type:	Turbo spray
Scan Type:	MRM (MRM)
Polarity:	Positive
Resolution Q1:	Unit
Resolution Q3:	Unit

CONFIDENTIAL

Serial Number: 1b7b4052-15d8-48b3-8e5f-a2b9525d9398 accessed by Anika Pippin (PIPPINA) on Mon, 28 Nov 2022 23:04:06 GMT

All the *below* parameters in this section may be adjusted to optimize the instrument conditions.

Zidebactam:

Q1 Mass (Da)	Q3 Mass (Da)	Dwell (msec)
392.028	312.000	250.00
DP	66.00	
CE	21.00	
CXP	16.00	

Cefotaxime (IS):

Q1 Mass (Da)	Q3 Mass (Da)	Dwell (msec)
456.200	324.000	250.00
DP	50.00	
CE	32.00	
CXP	10.00	

Parameter Table:

CAD:	12.00
CUR:	40.00
GS1:	50.00
GS2:	60.00
IS:	5500
TEM:	600
EP	12

8. REAGENTS, STANDARDS, AND SUPPLIES

Reagents from alternate supplier and equivalent grade can be substituted.

CONFIDENTIAL

Serial Number: 1b7b4052-15d8-48b3-8e5f-a2b9525d9398 accessed by Anika Pippin (PIPPINA) on Mon, 28 Nov 2022 23:04:06 GMT

Table 2: Reagents, Standards, and Supplies

**Chemical / Grade**	**Manufacturer/Supplier**
Zidebactam	Performa
Cefotaxime Sodium	Sigma Aldrich
MES ([2-(N-Morpholino) ethane sulphonic acid hydrate]) buffer	Sigma
Acetonitrile, HPLC grade	Honeywell
Formic Acid, 98+%	EMD Millipore
Deionized Water	Millipore
Ammonium Acetate ≥ 99.9%	Sigma
Methanol	Honeywell
Acetone	Honeywell
Ultrafiltered Human Sodium Heparin Plasma	BioIVT
Centrifugal Filters	Merck Millipore, Lot: 0000158890
Advantage SiliGuard Coated 2 mL 96 well Polypropylene plate: Square Top, Round Bottom	Analytical Sales and Services, Catalog # 27P687GC
Purple, pre-slit, square well, soft silicone/PTFE Cap Mats	Analytical Sales & Services, Inc., Catalog# 965085

9. SAFETY

Always observe safe laboratory practices. Consult the appropriate SDS before handling any chemical if you are unfamiliar with the proper safety precautions.

CONFIDENTIAL

Serial Number: 1b7b4052-15d8-48b3-8e5f-a2b9525d9398 accessed by Anika Pippin (PIPPINA) on Mon, 28 Nov 2022 23:04:06 GMT

10. PROCEDURE

10.1. Preparation of Analyte Stock Solutions and Dilutions

The quantities of analyte solution prepared may be scaled up or down proportionally as needed. Alternate dilution schemes can be used to achieve the appropriate final concentrations of standards, QCs, working internal standard, and system verification solution (SVS). For assignment of expiration dates to analyte stocks and their dilutions, refer to the latest version of the validation report.

10.1.1. Zidebactam Primary Solution, 4.00 mg/mL (Prepare on wet ice)

Check drying conditions prior to use. Weigh approximately 40.0 mg of zidebactam reference standard (correcting for purity and correction factor if necessary) in a polypropylene container and add 10.0 mL of deionized water. Mix well and sonicate if necessary. Store solution at -20°C in a polypropylene container.

10.1.2. Working Internal Standard Solution, ~50 mcg/mL Cefotaxime

Check drying conditions prior to use. Weigh 10.0 mg of cefotaxime reference standard into a glass container. Add 200 mL of deionized water (do not correct for conversion factor and purity). Mix well and sonicate if necessary. Store at or below -20 °C in a glass container.

10.2. Preparation of Other Reagents

Prepared quantities of all reagents may be scaled up or down as necessary. Volumes provided are examples. For assignment of expiration dates, refer to SOP-00094.

10.2.1. Mobile Phase A: 10 mM Ammonium Acetate in Deionized Water

Weigh ~3.08 g of ammonium acetate (F.W. 77.08) and transfer to a 4-L volumetric flask. Dilute to volume with deionized water. Mix well and store at room temperature.

10.2.2. Mobile Phase B: 95/5 Acetonitrile / 10mM Ammonium Acetate

Combine 3800 mL of acetonitrile with 200 mL of 10 mM ammonium acetate in a 4-L flask. Mix well and store at room temperature.

CONFIDENTIAL

Serial Number: 1b7b4052-15d8-48b3-8e5f-a2b9525d9398 accessed by Anika Pippin (PIPPINA) on Mon, 28 Nov 2022 23:04:06 GMT

10.2.3. Rinse: 90/10 Acetone/Deionized Water

Combine 900 mL of acetone and 100 mL of deionized water in a 1-L flask. Mix well and store at room temperature.

10.2.4. 1 N Sodium Hydroxide (NaOH) in Deionized Water

Weight ~ 4.00 g of sodium hydroxide (F.W. 40.00) and gradually transfer it to a 100-mL volumetric flask containing ~50 mL of deionized water. Mix to dissolve and wait until it reaches room temperature. Dilute to volume with deionized water and mix well. Store in a polypropylene container at room temperature.

10.2.5. MES (2-(N-Morpholino) Ethanesulphonic Acid Hydrate) Buffer pH 6.4, ~0.1M

Weigh ~ 4000 mg of MES hydrate (F.W. 195.24) and transfer to a 200-mL volumetric flask containing ~150 mL of deionized water. Dissolve and adjust the pH to 6.4 with 1N NaOH. Dilute to volume with deionized water. Mix well and store refrigerated.

10.2.6. 5/95 Acetonitrile/Deionized Water

Combine 50 mL of acetonitrile and 950 mL of deionized water in a 1-L flask. Mix well and store at room temperature.

10.3. Preparation of Standards and QC Samples

10.3.1. Preparation of Standard and QC Spiking Solutions on Wet Ice

Starting from the zidebactam primary solution (4.00 mg/mL) prepare a series of zidebactam spiking solutions in deionized water on wet ice. The following concentrations are recommended but can be altered as necessary: For standards: 500, 400, 200, 100, 50.0, 10.0, 1.00, and 0.500 mcg/mL. For QC spiking solutions: 375, 250, and 1.50 mcg/mL, and 0.500 mcg/mL for the LLOQ QC when needed. Store solutions in polypropylene at -20°C.

10.3.2. Treated Human Plasma Ultrafiltrate (Ultrafiltrate : MES Buffer 1:1)

Add 200 mL of chilled sodium heparin human plasma ultrafiltrate to a polypropylene container containing 200 mL of chilled 0.1M MES Buffer, pH 6.4. Mix well and store at -20°C.

It is recommended to prepare the treated human plasma ultrafiltrate fresh prior to usage.

CONFIDENTIAL

Serial Number: 1b7b4052-15d8-48b3-8e5f-a2b9525d9398 accessed by Anika Pippin (PIPPINA) on Mon, 28 Nov 2022 23:04:06 GMT

10.3.3. Preparation of Standards and QC Samples in Human Plasma Ultrafiltrate

Use treated sodium heparin human plasma ultrafiltrate that has been diluted 1:1 with MES buffer, screened for the lack of interference at the retention times of zidebactam and cefotaxime (IS). Dilute each spiking solution by a factor of 20 into treated human plasma ultrafiltrate on wet ice and mix well. For example, combine 0.500 mL of spiking solution and 9.5 mL of treated ultrafiltrate. Since unknown samples are treated with the MES buffer after collection at a 1:1 ratio, the calibration standard and QC concentrations are based on the ultrafiltrate volume only. Unknown samples do not need to be adjusted for the dilution with MES buffer. The prepared concentrations in ultrafiltrate only, not accounting for MES buffer, will be as follows: For standards: 50.0, 40.0, 20.0, 10.0, 5.00, 1.00, 0.100, and 0.0500 mcg/mL. For QCs: 37.5, 25.0, and 0.150 mcg/mL, with 0.0500 mcg/mL for the LLOQ QCs when needed. Other concentrations may be prepared, provided they meet the requirements of SOP-00035 and provided they remain within the range of 0.0500 to 50.0 mcg/mL. Store solutions in polypropylene at -70°C or -20°C.

10.3.4. Ultrafiltration of Human Plasma Samples using Centrifugal Filters

1. Thaw human plasma sample on wet ice. Once sample is thawed, vortex each sample for ~ 20 seconds.
2. Add ~1 mL of sample into a centrifugal filter. Hold the tube at ~45° with pipette tip touching the tube wall and add sample slowly with even flow to avoid air locking.
3. Cap the filter. The caps should extend no more than 3-4 mm the top. Further compression can cause prefiltration, which could introduce analytical error.
4. Centrifuge sample at 2000 x g for 30 minutes, this will yield ~150 mcL of filtrate.
5. Use the resulting plasma filtrate samples for the extraction procedure in Section 10.4.

CONFIDENTIAL

Serial Number: 1b7b4052-15d8-48b3-8e5f-a2b9525d9398 accessed by Anika Pippin (PIPPINA) on Mon, 28 Nov 2022 23:04:06 GMT

10.4. Extraction Procedure

1. Thaw treated human plasma ultrafiltrate samples on wet ice. Once samples are thawed, either vortex the batch as a whole for ~5 minutes followed by vortexing each for 10 seconds, or vortex each sample for ~ 20 seconds each.
2. Pipet 50 mcL of MES Buffer, pH 6.4 into each well in a 1-mL 96-well plate.
3. Pipet 50 mcL of ultrafiltered human plasma sample or blank to the corresponding wells of the 1-mL 96-well plate.
4. Add 50 mcL of working IS (50 mcg/mL cefotaxime in deionized water) to all applicable wells. For **Control Blanks** add 50 mcL of deionized water instead.
5. Seal the plate with an adhesive pad (or equivalent) and vortex at 1300 rpm for 5 minutes using a plate shaker.
6. Add 200 mcL of acetonitrile to all wells. Seal the plate with an adhesive pad (or equivalent) and vortex for 10 minutes at 1200 rpm.
7. Centrifuge the plate for 10 minutes at a setting of 4000 rpm (ambient conditions).
8. Transfer 200 mcL of the supernatant to a new 1-mL 96-well plate using a multichannel pipette (or equivalent).
9. Evaporate the sample using a 96-well SPE dryer at 37 °C at 60-80 psi. This generally takes around 60 minutes.
10. Reconstitute the plate with 150 mcL of 5/95 acetonitrile/deionized water in each well. Seal the plate with a cap mat.
11. Vortex the final plate at 1300 rpm for 5 minutes using a plate shaker.
12. Refrigerate prior to LC-MS injection according to the conditions outlined in Section 7.

10.5. Dilutions

2x = 25 mcL of sample + 25 mcL of blank treated human plasma ultrafiltrate

10x = 20 mcL of sample + 180 mcL of blank treated human plasma ultrafiltrate, mix well and use 50 mcL for analysis

CONFIDENTIAL

Serial Number: 1b7b4052-15d8-48b3-8e5f-a2b9525d9398 accessed by Anika Pippin (PIPPINA) on Mon, 28 Nov 2022 23:04:06 GMT

10.6. Calculations

Quadratic regression is performed with a 1/x2 weighting factor.

Equation: y = ax2 + bx + c

Where y = analyte/internal standard area ratio

x = concentration of analyte (mcg/mL)

a, b, c = Coefficients of regression

Results were obtained by solving for the analyte concentration (x) as shown:

$$x = \frac{-b \pm \sqrt{b^2 - 4ac}}{2a}$$

11. REFERENCE CHROMATOGRAMS

Figure 1: Blank with IS

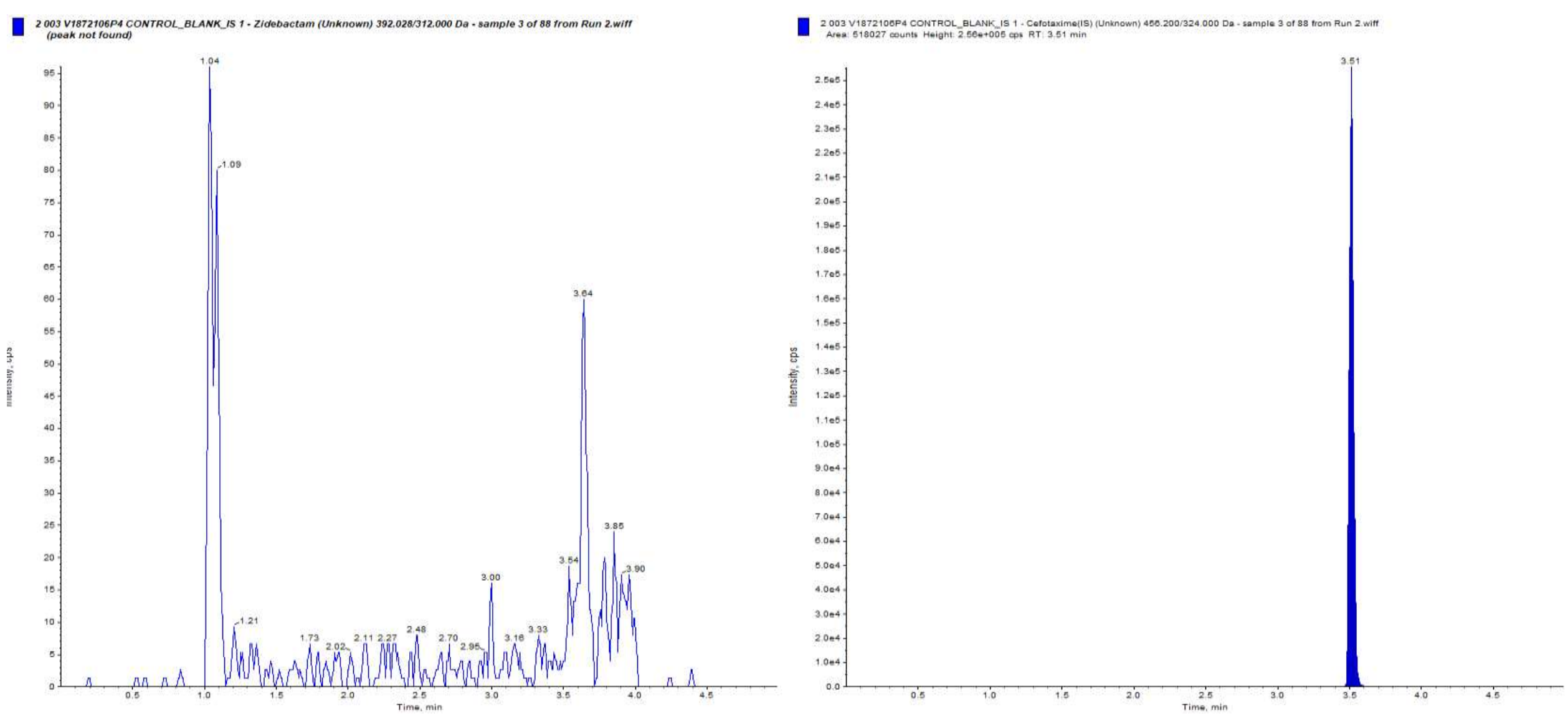

CONFIDENTIAL

Serial Number: 1b7b4052-15d8-48b3-8e5f-a2b9525d9398 accessed by Anika Pippin (PIPPINA) on Mon, 28 Nov 2022 23:04:06 GMT

Figure 2: Carryover Blank with IS

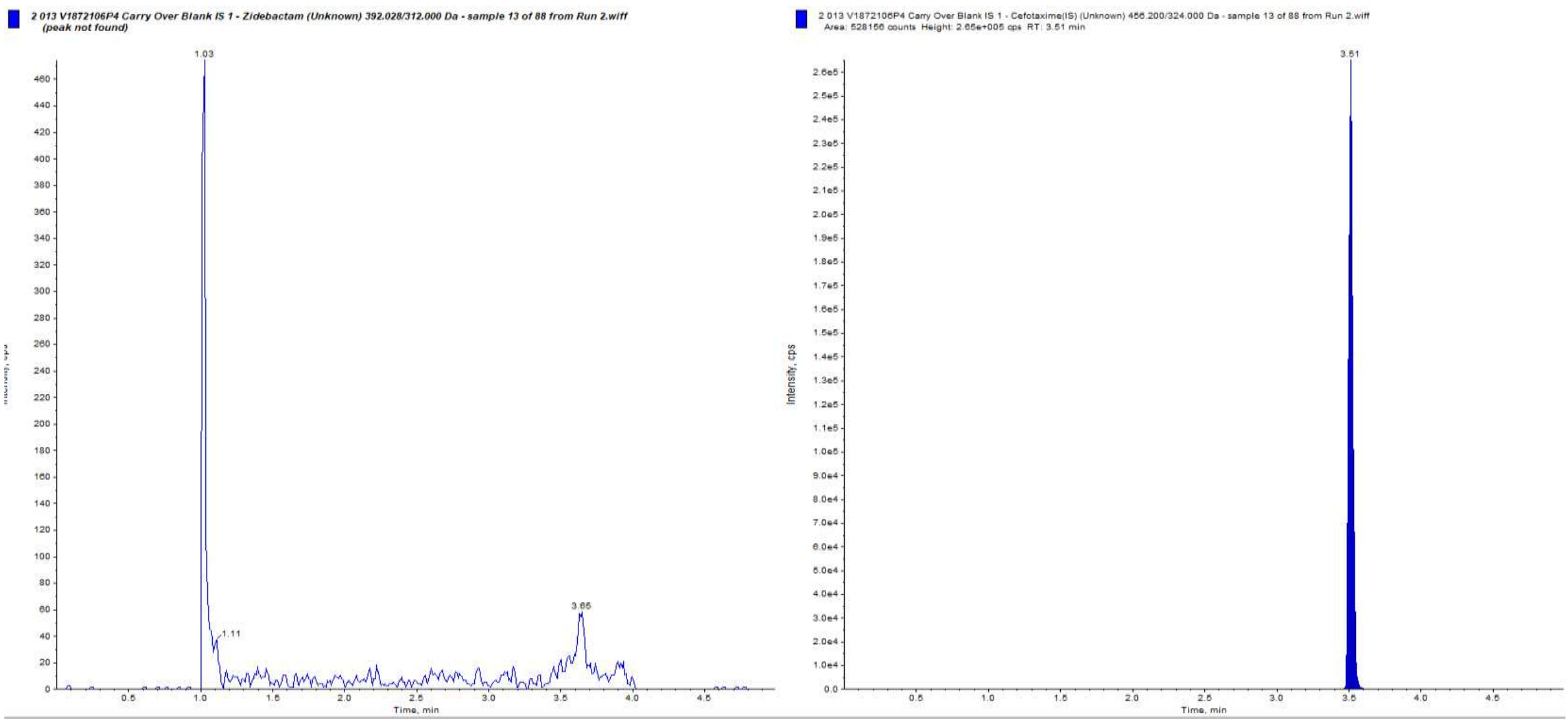

Figure 3: LLOQ – Zidebactam 0.050 mcg/mL

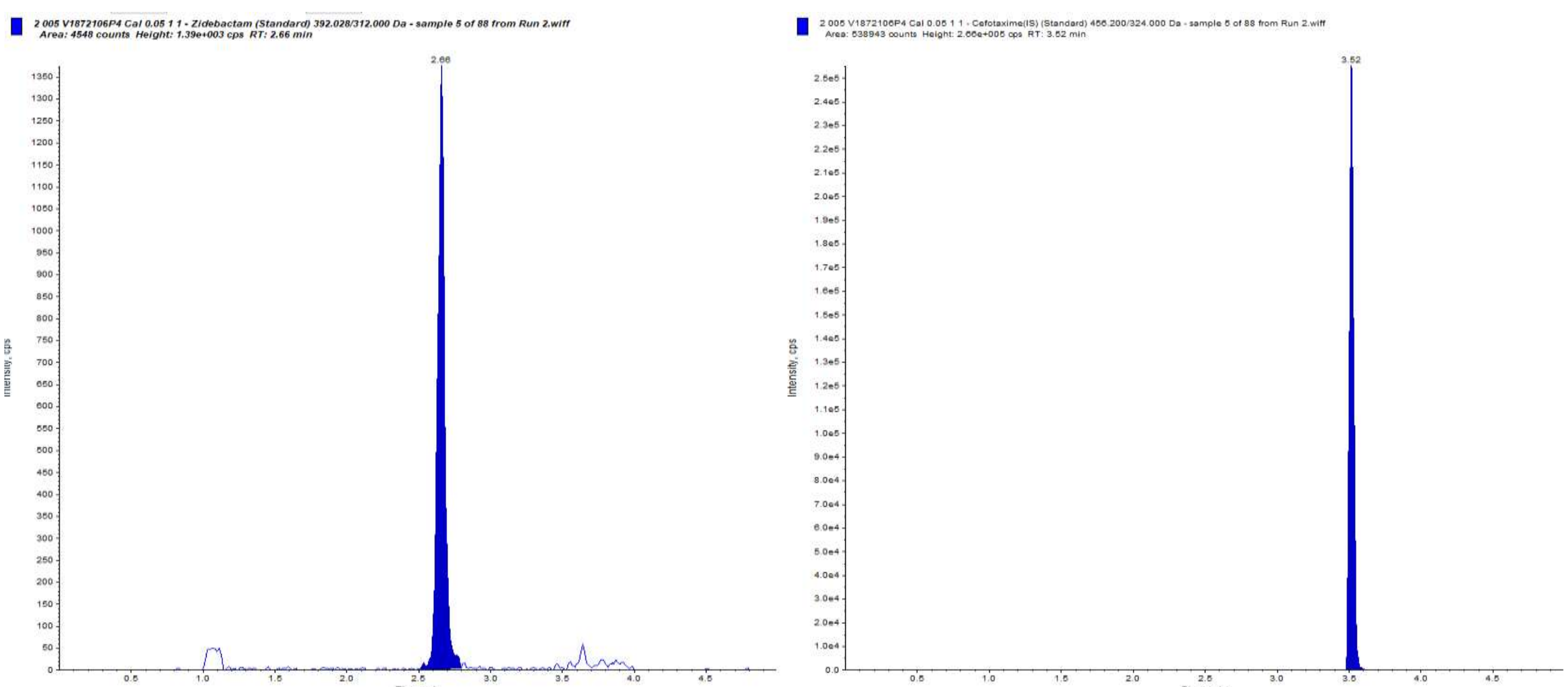

CONFIDENTIAL

Serial Number: 1b7b4052-15d8-48b3-8e5f-a2b9525d9398 accessed by Anika Pippin (PIPPINA) on Mon, 28 Nov 2022 23:04:06 GMT

12. REVISION HISTORY

Table 3: Revision History

Revision	Effective Date	Section	Revision Summary
00	See Header	NA	New Procedure

CONFIDENTIAL

Serial Number: 1b7b4052-15d8-48b3-8e5f-a2b9525d9398 accessed by Anika Pippin (PIPPINA) on Mon, 28 Nov 2022 23:04:06 GMT

TP-80217 [00]: V1872106P4 - Free Zidebactam in Treated Human Plasma Ultrafiltrate (50 mcL, 0.0500 - 50 mcg/mL)

All blank fields are considered as data not available.

All Signature (Date and Time) shown in this report are in GMT.

**Type**	TP	**Status**	CURRENT	
**Effective Date**	25-Nov-2022	**Periodic Review Date**	11-Apr-2050	
**Owner**	PARMENTIERB	**Department Name**	Pharma LC-MS/MS	Pharma LC-MS/MS

Description

Full Title: LC-MS/MS Analysis of Free Zidebactam in Treated Human Plasma Ultrafiltrate (Sodium Heparin, 50 mcL, 0.0500 – 50.0 mcg/mL; Prior to MES Treatment)

Revision Notes (5)

Task Name	Action	Comment	Signature
Check In	Document Profile Preparation	Created outside of the system.	NEVINSC<br>21-Nov-2022 22:04:27 [GMT]
Check Out	Document Finalization	TOC correction needed.	NEVINSC<br>21-Nov-2022 22:14:28 [GMT]
Check In	Document Finalization	TOC corrected.	NEVINSC<br>21-Nov-2022 22:15:05 [GMT]
Check Out	Document Finalization	Update Section 10.6 - Calculations	NEVINSC<br>23-Nov-2022 22:08:16 [GMT]
Check In	Document Finalization	Updated Section 10.6 - Calculations	NEVINSC<br>23-Nov-2022 22:08:51 [GMT]

Serial Number: 1b7b4052-15d8-48b3-8e5f-a2b9525d9398 accessed by Anika Pippin (PIPPINA) on Mon, 28 Nov 2022 23:04:06 GMT.

Approval Information

Note to Approver

Reviewer: Bryan Parmentier, MLT (ASCP)
Management: Marsha Luna, B.S.
PI: Anika Pippin, B.S.

Approval Decision	Comment	Signature
Approve		PARMENTIERB<br>23-Nov-2022 22:21:27 [GMT]
Approve		LUNAM<br>23-Nov-2022 22:35:17 [GMT]
Approve		PIPPINA<br>26-Nov-2022 1:08:38 [GMT]

Serial Number: 1b7b4052-15d8-48b3-8e5f-a2b9525d9398 accessed by Anika Pippin (PIPPINA) on Mon, 28 Nov 2022 23:04:06 GMT.

WOCKHARDT LIFE WINS

ANALYTICAL RESEARCH

CERTIFICATE OF ANALYSIS FOR REFERENCE STANDARD

**Name**	:	**Zidebactam (WCK 5107)**			
Molecular Formula	:	$C_{13}H_{21}N_5O_7S.2H_2O$	Batch Number	:	WK1/WP-0060/016
Molecular Weight	:	427.45	Laboratory Note Book Reference	:	WA-2004-038
Date of Analysis	:	30/03/2022	Month of Manufacturing	:	May' 2019
Valid Up To	:	29/03/2023	Reference Standard Number	:	RS/2022-013
Effective Date	:	13/04/2022	Page Number	:	Page 1 of 4

Sr. No.	Tests	Specifications	Results
1	Description	White to pale yellow powder	White powder
2	Identification (By IR, Mass & NMR Spectroscopy)	Spectral data should conform to the structure	Spectral data conforms the structure
3	Solubility	Freely soluble in water, Insoluble in dichloromethane	Freely soluble in water, Insoluble in dichloromethane
4	Constituted solution (10% w/v solution in WFI) Completeness of solution	The solid dissolves completely, leaving no visible residue as un-dissolved matter	Complies
	Clarity of solution	The constituted solution is not significantly less clear than an equal volume of the WFI contained in a similar vessel and examined similarly	Complies
5	pH of solution	Between 5.0 to 7.0	6.0
6	Absorbance of solution (1.25 % w/v in water at 420 nm)	Not more than 0.2 AU	0.01 AU
7	Water content (By KF Titration)	Not more than 12.0 % w/w	8.7 % w/w
8	Specific optical rotation (On anhydrous basis)	Between –28° to -40°	-36°
9	Heavy metals	Not more than 20 µg/g	Less than 20 µg/g
10	Palladium content (By ICP-OES)	Not more than 10 µg/g	BLQ (LOQ 2.5 µg/g)*

	Prepared by	Checked by	Approved by
Name :	Mr. Sitaram Ubale	Mr. Rajendra Chavan	Mr Subhash Chandra Shukla
Department :	AVL	AVL	CRDQ
Sign :	[signature]	[signature]	[signature]
Date :	12/04/2022	12/04/2022	13/04/2022

Format No. : AVL-045-F-02-00

Ranked # 1 In Healthcare
Best Companies to Work for 2015 India

ANALYTICAL RESEARCH

CERTIFICATE OF ANALYSIS FOR REFERENCE STANDARD

**Name**	:	**Zidebactam (WCK 5107)**			
Molecular Formula	:	$C_{13}H_{21}N_5O_7S.2H_2O$	Batch Number	:	WK1/WP-0060/016
Molecular Weight	:	427.45	Laboratory Note Book Reference	:	WA-2004-038
Date of Analysis	:	30/03/2022	Month of Manufacturing	:	May' 2019
Valid Up To	:	29/03/2023	Reference Standard Number	:	RS/2022-013
Effective Date	:	23/04/2022	Page Number	:	Page 2 of 4

Sr. No.	Tests	Specifications	Results
11	Related substances (By HPLC) :		
	i. Impurity -1	Not more than 2.00 % w/w	0.28 % w/w
	ii. Impurity -2	Not more than 1.00 % w/w	Not detected
	iii. Impurity -3	Not more than 1.00 % w/w	0.10 % w/w
	iv. Impurity -4	Not more than 0.10 % w/w	Not detected
	v. Specified impurity at RRT 2.16-2.28 (MW 782)	Not more than 0.50 % w/w	0.03 % w/w
	vi. Any unspecified impurity	Not more than 0.15 % w/w	BDL
	vii. Total impurities	Not more than 4.00 % w/w	0.41 % w/w
12	Residual solvents (By GC) :		
	Group A		
	i. Isopropyl alcohol	Not more than 5000 µg/g	Not detected
	ii. Dichloromethane	Not more than 600 µg/g	Not detected
	iii. Methanol	Not more than 3000 µg/g	1762 µg/g
	iv. Ethyl acetate	Not more than 5000 µg/g	Not detected
	v. Cyclohexane	Not more than 3880 µg/g	Not detected
	vi. Acetone	Not more than 5000 µg/g	Not detected
	vii. Methyl tertiary butyl ether	Not more than 5000 µg/g	Not detected
	viii. Toluene	Not more than 890 µg/g	Not detected
	ix. Ethanol	Not more than 5000 µg/g	2990 µg/g
	Group B		
	i. Triethylamine	Not more than 250 µg/g	Not detected
	ii. Pyridine	Not more than 200 µg/g	Not detected

	Prepared by	Checked by	Approved by
Name :	Mr. Sitaram Ubale	Mr. Rajendra Chavan	Mr Subhash Chandra Shukla
Department :	AVL	AVL	CRDQ
Sign :	[signature]	[signature]	[signature]
Date :	12/04/2022	12/04/2022	13/04/2022

Format No. : AVL-045-F-02-00

Ranked # 1 in Healthcare
Great Place to Work
Best Companies to Work for 2015 India

ANALYTICAL RESEARCH

CERTIFICATE OF ANALYSIS FOR REFERENCE STANDARD

Name	:	**Zidebactam (WCK 5107)**			
Molecular Formula	:	$C_{13}H_{21}N_5O_7S.2H_2O$	Batch Number	:	WK1/WP-0060/016
Molecular Weight	:	427.45	Laboratory Note Book Reference	:	WA-2004-038
Date of Analysis	:	30/03/2022	Month of Manufacturing	:	May' 2019
Valid Up To	:	29/03/2023	Reference Standard Number	:	RS/2022-013
Effective Date	:	23/04/2022	Page Number	:	Page 3 of 4

Sr. No.	Tests	Specifications	Results
13	Sodium content (By ICP-OES)	Not more than 0.5 % w/w	BLQ (LOQ 0.25 % w/w)*
14	Sulfate content (By Ion chromatography)	Not more than 1.0 % w/w	BLQ (LOQ 0.626 % w/w)
15	Mono-methyl sulphate content (By Ion chromatography)	Not more than 0.15% w/w	Not detected
16	Polymorphism (By X-ray Diffraction)	The diffractogram should exhibit characteristic 2 theta values at 13.8, 15.6, 20.3 and 24.3 (± 0.2°)	The diffractogram exhibits characteristic 2 theta values at 13.8, 15.6, 20.3 & 24.3
17	Potency (on as is basis)	Not applicable	90.4 %

BLQ = Below limit of quantification
BDL = Below disregard limit. Disregard limit 0.05 %
LOQ = Limit of quantification

* LOQ Values are based on analytical method verification report (Report nos. MVR/VP17053 and MVR/VP17054) of Analytical Solutions (CRO).

Specification Reference Number: **ZDB-DSS-112-05**

Remark: Potency is calculated on the basis of Total impurities, Water content, Residual solvents, Sulphate, Mono-methyl sulphate, Sodium and Palladium content.

Storage Condition: Store at 2°C to 8°C temperature.

	Prepared by	Checked by	Approved by
Name :	Mr. Sitaram Ubale	Mr. Rajendra Chavan	Mr Subhash Chandra Shukla
Department :	AVL	AVL	CRDQ
Sign :	[signature]	[signature]	[signature]
Date :	12/04/2022	12/04/2022	13/04/2022

Format No. : AVL-045-F-02-00

Ranked # 1 In Healthcare
GREAT PLACE TO WORK Best Companies to Work for 2015 India

ANALYTICAL RESEARCH

CERTIFICATE OF ANALYSIS FOR REFERENCE STANDARD

**Name**	:	**Zidebactam (WCK 5107)**			
Molecular Formula	:	$C_{13}H_{21}N_5O_7S.2H_2O$	Batch Number	:	WK1/WP-0060/016
Molecular Weight	:	427.45	Laboratory Note Book Reference	:	WA-2004-038
Date of Analysis	:	30/03/2022	Month of Manufacturing	:	May' 2019
Valid Up To	:	29/03/2023	Reference Standard Number	:	RS/2022-013
Effective Date	:	23/04/2022	Page Number	:	Page 4 of 4

Chemical Names:

i. **WCK 5107**: (2S,5R)-7-Oxo-6-sulphoxy-2-[N'-((R)-piperidin-3-carbonyl)-hydrazinocarbonyl]-1,6-diaza-bicyclo[3.2.1]octane dehydrate

ii. **Impurity-1**: (2S,5R)-5-sulphooxyamino-2-[N'-((R)-piperidin-3-carbonyl)-hydrazinocarbonyl]-piperidine

iii. **Impurity-2**: (2S,5R)-7-Oxo-6-hydroxy-2-[N'-((R)-piperidin-3-carbonyl)-hydrazinocarbonyl]-1,6-diaza-bicyclo[3.2.1]octane

iv. **Impurity-3**: (2S,5R)-7-Oxo-6-sulphooxy-2-[N'-((S)-piperidin-3-carbonyl)-hydrazinocarbonyl]-1,6-diaza-bicyclo[3.2.1]octane

v. **Impurity-4**: (2S,5R)-7-Oxo-6-sulphooxy-2-[N'-((R)-1-tert-butoxycarbonyl-piperidin-3-yl)-carbonyl)-hydrazinocarbonyl] -1,6-diaza-bicyclo[3.2.1]octane

	Prepared by	Checked by	Approved by
Name :	Mr. Sitaram Ubale	Mr. Rajendra Chavan	Mr Subhash Chandra Shukla
Department :	AVL	AVL	CRDQ
Sign :	[signature]	[signature]	[signature]
Date :	12/04/2022	12/04/2022	13/04/2022

Format No. : AVL-045-F-02-00

Ranked # 1 in Healthcare
Best Companies to Work for 2015 India

Sigma-Aldrich.

3050 Spruce Street, Saint Louis, MO 63103, USA
Website: www.sigma-aldrich.com
Email USA: techserv@sial.com
Outside USA: eurtechserv@sial.com

Certificate of Analysis

**Product Name :**	**Cefotaxime sodium salt suitable for plant cell culture, BioReagent, powder**
**Product Number :**	**C7039-500MG**
**Batch Number :**	**0000146942**
Source Batch :	157206
CAS Number :	64485-93-4
Storage Temperature :	Cooler/Refrigerated
Molecular Formula :	$C_{16}H_{16}N_5NaO_7S_2$
Formula Weight :	477.45
Recommended Retest Date :	Sep 2023
Quality Release Date :	02 Jan 2022

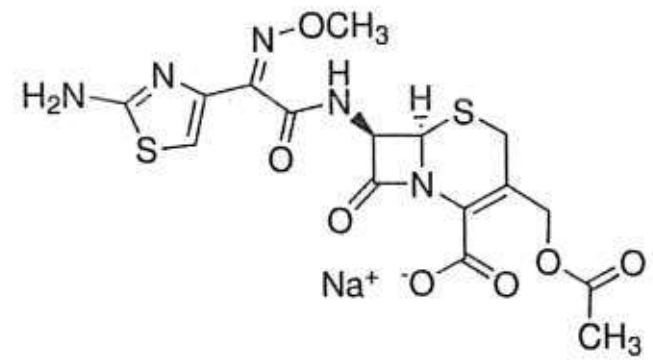

Test	Specification	Result
Appearance (Form)	Powder or Crystals	Powder
Appearance (Color)<br>Off-white to pale yellow	Conforms	Conforms
Solubility<br>10% in water	Clear	Clear
Infrared Spectrum	Conforms to Structure	Conforms
Total Impurity	≤ 3.0 %	1.7 %
Specific Rotation<br>[a]20/D	58 - 64 °	61 °
Assay (Dry Basis)	916 - 964 µg/mg	955 µg/mg
Loss on Drying	≤ 3.0 %	2.4 %
Plant Cell Culture Test	Pass	Pass

Anna Bendersky, Quality Control Manager
Quallity Control
Israel
IL

Sigma-Aldrich warrants, that at the time of the quality release or subsequent retest date this product conformed to the information contained in this publication. The current Specification sheet may be available at Sigma-Aldrich.com. For further inquiries, please contact Technical Service. Purchase must determine the suitability of the product for its particular use. See reverse side of website or packing slip for additional terms and conditions of sale

Version Number: 01 Doc: 1074711

The branding on the header and/or footer of this document may temporarily not visually match the product purchased as we transition our branding. However, all of the information in the document regarding the product remains unchanged and matches the product ordered.
For further information please contact mlsbranding@sial.com

Certificate of Analysis

Inhibitors • Screening Libraries • Proteins

Ertapenem sodium

Cat. No.:	HY-13625
CAS No.:	153773-82-1
Batch No.:	180017
Chemical Name:	1-Azabicyclo[3.2.0]hept-2-ene-2-carboxylic acid, 3-[[(3S,5S)-5-[[(3-carboxyphenyl)amino]carbonyl]-3-pyrrolidinyl]thio]-6-[(1R)-1-hydroxyethyl]-4-methyl-7-oxo-, sodium salt (1:1), (4R,5S,6S)-

PHYSICAL AND CHEMICAL PROPERTIES

Molecular Formula:	$C_{22}H_{24}N_3NaO_7S$
Molecular Weight:	**497.50**
Storage:	4°C, sealed storage, away from moisture<br>* In solvent : -80°C, 6 months; -20°C, 1 month (sealed storage, away from moisture)

Chemical Structure:

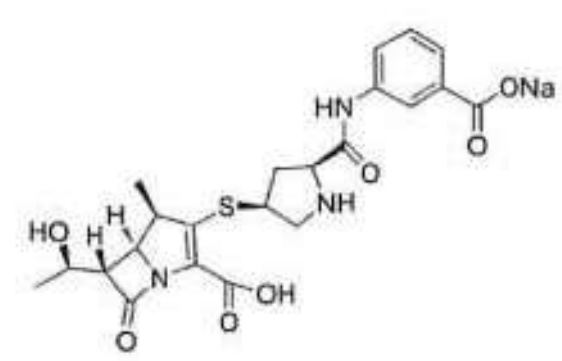

ANALYTICAL DATA

Appearance:	White to light yellow (Solid)
^{1}H NMR Spectrum:	Consistent with structure
LCMS:	Consistent with structure
Purity (LCMS):	**98.86%**
Test date:	2022/08/12
Retest date:	2024/08/11
Conclusion:	The product has been tested and complies with the given specifications.

Name: Lingli Ma
Designation: QA Manager
Date: 6/27/2022

Caution: Product has not been fully validated for medical applications. For research use only.

Tel: 609-228-6898 Fax: 609-228-5909 E-mail: tech@MedChemExpress.com
Address: 1 Deer Park Dr, Suite Q, Monmouth Junction, NJ 08852, USA
