# Supplementary material for: A Phase I, randomized, double-blind, placebo-controlled, dose escalation study to evaluate the safety, tolerability, and pharmacokinetics of intravenous ertapenem and zidebactam in combination (WCK 6777) in healthy adult subjects
Source: Antimicrob Agents Chemother. 2026 Sep 29;70(11):e00390-26. doi: 10.1128/aac.00390-26 (PMC13731237; doi:10.1128/aac.00390-26)

1. TITLE: THE DETERMINATION OF ERTAPENEM IN TREATED HUMAN URINE BY LC-MS/MS

BIOANALYTICAL METHOD VALIDATION REPORT

Bioanalytical Project Number: V1872106U3

Sponsor: DynPort Vaccine Company, LLC
110 Thomas Johnson Drive
Suite 200
Frederick, MD 21702

Bioanalytical Laboratory: KCAS Bio
10830 S Clay Blair Boulevard
Olathe, KS 66061

Original Report Issue Date: February 2023

Amendment 1 Issue Date: September 2024

2. SYNOPSIS

This report presents the validation data for an LC-MS/MS method for the determination of ertapenem in treated human urine. The assay was analyzed in the range of 10.0 - 4000 mcg/mL and is for use on treated human urine samples obtained during clinical trials.

AMENDMENT OUTLINE FORM

1. Amendment Number 1	September 2024. This amendment was created to report:<br>• Long-term matrix stability at -70 °C for up to 101 days (with zidebactam) and 194 days (without zidebactam) for ertapenem in treated human urine.<br>• Platform change (API5000 to API6500)

3. TABLE OF CONTENTS FOR THE BIOANALYTICAL METHOD VALIDATION REPORT

1. TITLE: THE DETERMINATION OF ERTAPENEM IN TREATED HUMAN URINE BY LC-MS/MS 1
2. SYNOPSIS 2
3. TABLE OF CONTENTS FOR THE BIOANALYTICAL METHOD VALIDATION REPORT 3
4. LIST OF TABLES 4
5. SIGNATURE PAGE 5
6. QUALITY ASSURANCE STATEMENT 6
7. STUDY COMPLIANCE STATEMENT 7
8. LIST OF ABBREVIATIONS AND DEFINITIONS OF TERMS 8
9. AMENDMENT 10
10. EXPERIMENTAL DESIGN AND RESULTS 10
10.1. Reference Standards 10
10.2. Calibration Standards and QC Samples Preparation 10
10.3. Run Acceptance 11
10.3.1. Standards 11
10.3.2. QCs 11
10.3.3. Blanks 11
10.4. Carryover 11
10.5. Long-Term Matrix Stability at -70 °C 12
10.6. Platform Change (API5000 to API6500) 12
10.7. SOP Deviations and Investigations 13
11. CONCLUSION 13
12. PERTINENT SOPS 13
13. ARCHIVES 13
14. REFERENCES 13
15. TABLES 14

4. LIST OF TABLES

Table 1: Abbreviations and Codes 8
Table 2: Mass Spec Transitions 14
Table 3: Reference Standards 14
Table 4: Calibration Standards and QC Samples Preparation 15
Table 5: Matrix Description 18
Table 6: Summary of Results and Conclusions 19
Table 7: Summary of Validation Run Objectives and Outcome 22
Table 8: Carryover 23
Table 9: Standards and QCs of Non-A&P Runs 24
Table 10: Standard and QC of Failed Runs 26
Table 11: Long-Term Stability at -70 °C 28
Table 12: Platform Change (API5000 to API6500) 31

5. SIGNATURE PAGE

Bioanalytical Project Number: V1872106U3

Amendment 1 Issue Date: Date of PI Signature

APPROVED

Signed by:
Anika Pippin
Signer Name: Anika Pippin
Signing Reason: I approve this document
Signing Time: 06-Sep-2024 | 16:14 CDT
BE3AA1198E40459E8A7E3C615D981D82

Anika Pippin, B.S.
Principal Investigator
08Nov2022 to 30Jun2024
22Jul2024 to Present

Yu-Hui Ann Fu, M.S.
Principal Investigator
01Jul2024 to 19Jul2024

REVIEWED

Signed by:
Marsha Luna
Signer Name: Marsha Luna
Signing Reason: I have reviewed this document
Signing Time: 06-Sep-2024 | 15:24 CDT
0D4CD5E742334ED98F5FE515F0F46E9B

Marsha Luna, B.S.
Management

6. QUALITY ASSURANCE STATEMENT

This Bioanalytical Method Validation Report has been found to describe accurately the methods and SOPs used and to accurately reflect the raw data collected. The Quality Assurance Unit initiated audits on this study and reported the findings to the Principal Investigator (PI) and Management as follows:

		Date(s) Sent to:	
**Inspection Date(s)**	**Inspection Phase**	**PI**	**Management**
23Feb2023	Data Audit	23Feb2023	07Mar2023
26Jun2023	Data Audit	26Jun2023	16Aug2023
27Jun2023	Draft Report Audit	27Jun2023	11Aug2023
19Feb2024 and 20Feb2024	Data Audit	20Feb2024	21Feb2024
27Feb2024	Data Audit	27Feb2024	08Mar2024
20May2024	Data Audit	20May2024	20May2024
13Aug2024	Data Audit	13Aug2024	13Aug2024
05Sep2024	Draft Report Audit	05Sep2024	06Sep2024
06Sep2024	Final Report Review	06Sep2024	06Sep2024

REVIEWED

Signed by:

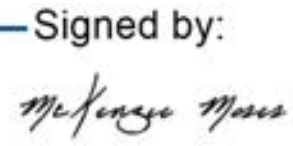

Signer Name: McKenzie Moses
Signing Reason: I have reviewed this document
Signing Time: 06-Sep-2024 | 15:23 CDT
EC84DBFDD7D44D238BCE4363EEA6D90A

McKenzie Moses, B.S.
Quality Assurance Unit

7. STUDY COMPLIANCE STATEMENT

I, the undersigned, hereby declare that this study was conducted in compliance with the relevant bioanalytical aspects of FDA guidelines, GLP documentation and KCAS Bio (KCAS) procedures and that the work was performed by me, or under my direction, and the report reflects a true and accurate record of the results obtained.

The acceptance criteria are based on the FDA Guidance for Industry, Bioanalytical Method Validation (2018). [1]

APPROVED

Signed by:

Anika Pippin

Signer Name: Anika Pippin
Signing Reason: I approve this document
Signing Time: 06-Sep-2024 | 16:14 CDT
BE3AA1198E40459E8A7E3C615D981D82

____

Anika Pippin, B.S.
Principal Investigator
08Nov2022 to 30Jun2024
22Jul2024 to Present

Yu-Hui Ann Fu, M.S.
Principal Investigator
01Jul2024 to 19Jul2024

8. LIST OF ABBREVIATIONS AND DEFINITIONS OF TERMS

Table 1: Abbreviations and Codes

Abbreviation	Definition
<	Concentration less than the lowest acceptable calibration standard
>	Concentration exceeded the upper limit of calibration range (> ULOQ)
A	Amendment
AQL	Above Quantitation Limit
%Bias	Percentage measurement valid
Batch	Is the same as Run
BQL	Below Quantifiable Limit
Cal	Calibration standard
Conc.	Concentration
Conv.	Conversion
%CV	Coefficient of Variation
dev	Deviation
diff	Difference
EMA	European Medicines Agency
FDA	Food and Drug Administration
GLP	Good Laboratory Practice
HPLC	High Performance Liquid Chromatography
ID	Identification
IS	Internal Standard
ISR	Incurred Sample Reanalysis
K_2EDTA or K_3EDTA	Dipotassium or Tripotassium Ethylenediaminetetraacetic Acid
KCAS	KCAS Bio
LC-MS/MS	Liquid Chromatography Tandem Mass Spectrometry
LLOQ	Lower Limit of Quantitation
mcg or µg or ug	Microgram
mcL or µL or uL	Microliter

Table 1: Abbreviations and Codes (Continued)

Abbreviation	Definition
ME	Matrix Effect
MHLW	Ministry of Health, Labour and Welfare of Japan
mL	Milliliter
MS	Mass Spectrometry
n	number
N.C.	Not Calculated
ND	No difference reported as ISR will be counted
ng	nanogram
n.report	No Data available to report
N/A or NA	Not Applicable
N.R.	Not Reported
P&A	Precision and Accuracy
p.a.r.	Peak Area Ratio
PI	Principal Investigator
QC	Quality Control Sample
QCs	Quality Control Samples
OECD	Organization for Economic Co-operation and Development
RT	Room Temperature
sd or SD	Standard deviation
Seq	Sequence
SOP (s)	Standard Operating Procedure (s)
temp	Temperature
TP	Test procedure
UHPLC	Ultra High Performance Liquid Chromatography
ULOQ	Upper Limit of Quantitation
Watson LIMS 7.5	Watson Laboratory Information Management System, Version 7.5, Service Pack 1
WIS	Working Internal Standard

This is a basic list. Some of the abbreviations may not have been used in this report.

9. AMENDMENT

This amendment was created to report long-term matrix stability at -70 °C for up to 101 days (with zidebactam), 194 days (without zidebactam) for ertapenem in treated human urine and platform change (API5000 to API6500).

The validation procedure was designed to meet or exceed the requirements of FDA Guidance for Industry on Bioanalytical Methods Validation, May 2018. The software programs used were Microsoft Office Excel 365, acquisition and processing software Analyst 1.7.2, and data management software Watson LIMS 7.5.

10. EXPERIMENTAL DESIGN AND RESULTS

In this method, ertapenem and internal standard, ertapenem-d_4, were released from human urine using 96-well protein precipitation. After extraction, the sample was chromatographed using reversed phase HPLC with a Fortis Technology Ltd. Fortis C18 analytical column. Ertapenem and ertapenem-d_4 (IS) were detected by monitoring the precursor and product ions described in Table 2 using an Applied Biosystems Sciex API 5000 or API 6500 LC-MS/MS. The quantification was based on setting a calibration graph using the internal standard method based on bioanalytical test procedure TP80182, entitled “LC-MS/MS Analysis of Ertapenem in Treated Human Urine (50 mcL, 10.0 - 4000 mcg/mL; Prior to MES Treatment)”. The test procedure can be found in Annex 1 (V1872106U3).

The human urine was treated with 0.1M 2-(N-Morpholino) ethane sulphonic acid hydrate (MES) buffer, pH 6.4 at a 1:1 ratio. The validation assay range is calculated based on urine volume only to mimic unknown study samples and no concentration adjustment is needed during sample analysis since the unknown samples are treated in the same manner. The assay range is 10.0 to 4000 mcg/mL in urine.

A summary of the validation results is provided in Table 6. A summary describing the objective(s) and outcome of each validation run attempted is provided in Table 7.

The method was validated within the analytical range of 10.0 - 4000 mcg/mL in human urine. No development work was performed after the method was validated.

10.1. Reference Standards

Reference standards used during this validation are presented in Table 3.

10.2. Calibration Standards and QC Samples Preparation

Calibration standards and quality control pools were prepared by spiking appropriate levels of ertapenem into human urine. Primary reference standards will be prepared in at least duplicate, and they need to compare within 5% of each other. Standards and QCs were prepared and stored

frozen at KCAS at -70 °C. Standards, QCs, and the critical solutions used during this validation are described in Table 4, and matrix used is described in Table 5.

10.3. Run Acceptance

For a run containing standards and QCs to be considered acceptable, the following criteria must be met:

10.3.1. Standards

For each run, the calculated concentrations of at least 75% of the calibration standards were required to interpolate within ± 15% from nominal with a minimum of six calibration standard levels, except for the lower limit of quantitation (LLOQ) for which it was required to be within 20%.

10.3.2. QCs

In order for QC sample results to be accepted for each run, at least half of QC samples at each concentration level and two-thirds of the total number of all QC samples are required to interpolate within ± 15% from nominal value.

- QCs were analyzed in replicates of at least n=2, but a greater number of replicates could have been used as well.
- For validation runs, the sensitivity QC is not considered part of run acceptance.

10.3.3. Blanks

At least three of the four blank matrix samples processed were required to be free from interference. To be deemed interference-free, the maximum allowable response (peak area for blanks without internal standard, or peak area ratio for blanks containing internal standard) at the retention time of analyte in a blank sample must be ≤ 20% of the corresponding mean response for the acceptable LLOQ standards in the run.

In addition, at least one of the two blanks processed without internal standard is required to be free from interference at the retention time of the internal standard. To be deemed interference-free, the maximum allowable response must be ≤ 5% of the average internal standard response for the acceptable LLOQ standard.

10.4. Carryover

Carryover is evaluated within each run. It consists of measuring the response for each analyte and if it exceeds 20.0% of the response of LLOQ standard or other sample reflecting the lower sensitivity limit required, a carryover assessment is completed, and affected samples are rejected. The internal standard carryover evaluation is assessed within each run and if it exceeds 5.0% of the average internal standard response of acceptable standards and QCs, impact is evaluated within the run. Data are presented in Table 8.

10.5. Long-Term Matrix Stability at -70 °C

To evaluate ertapenem in treated human urine with and without the presence of zidebactam during sample storage, aliquots of the high and low concentration QC pools were stored frozen at -70 °C nominally for 74 days (low QC without zidebactam), 79 days (high QC without zidebactam and high and low QCs with zidebactam), 101 days (high and low QCs with zidebactam), and 194 days (high and low QCs without zidebactam) prior to being analyzed, along with freshly spiked standards with QCs for which stability has been proven. To be deemed stable, the mean %Bias at each level must be within ± 15% with %CV ≤ 15%. In addition, run acceptance must be met as outlined in Section 10.3.

Data is presented in Table 11.

Ertapenem in treated human urine (without zidebactam) -70 °C 79 days LTS low QC in V1872106U3 run 16 failed %Bias criteria at -16.3%. The high QC concentration does not show the same low bias indicating it is not likely a stability related issue. Stability was run at both concentrations for the 194-day timepoint in run 2 for confirmation with passing results at both concentrations. Therefore, ertapenem in treated human urine without the presence of zidebactam was proven to be stable at -70 °C over a period of 194 days in polypropylene tubes.

Ertapenem in treated human urine (with zidebactam) stability in V1872106U3A1 runs 5 (185 days) and 6 (234 days) -70 °C Comed stability failed %Bias high at both QC levels. Ertapenem in treated human urine with the presence of zidebactam was proven to be stable at -70 °C over a period of 101 days in polypropylene tubes in run 8.

10.6. Platform Change (API5000 to API6500)

Platform change was assessed by injecting run 4 on a different MS platform (Applied Biosystems API6500).

The maximum allowable percent deviation from nominal for each standard was ± 15%, except at the LLOQ, where ± 20% was accepted. Any standard deviating by more than these limits was required to be excluded from the regression line. For the calibration curve to be accepted, at least 75% of the initial number of standards was required to remain in the curve. In addition, for accuracy and precision (A&P) runs, the criteria (within ± 15% or ± 20% for LLOQ) should also be fulfilled for at least 50% of the calibration standards tested per concentration level. In addition, run acceptance must be met as outlined in Section 10.3.

The accuracy at each concentration level should be within ± 15% of the nominal concentration, except at the LLOQ, where it should be within ± 20%. The precision (%CV) of the concentrations determined at each level should not exceed 15%, except at the LLOQ, where it should not exceed 20%. Along with the sensitivity QC having signal to noise of at least 5, to be considered an acceptable platform to be used.

Data are presented in Table 12 for the precision/accuracy portion of this experiment. All criteria are met.

10.7. SOP Deviations and Investigations

No SOP deviations occurred during this study. No investigations were conducted during this study.

11. CONCLUSION

Ertapenem in treated human urine without the presence of zidebactam was proven to be stable at -70 °C over a period of 194 days in polypropylene tubes. Ertapenem in treated human urine with the presence of zidebactam was proven to be stable at -70 °C over a period of 101 days in polypropylene tubes. Platform change (API5000 vs API6500) has been proven.

12. PERTINENT SOPS

The following SOPs were followed during the conduct of this study:

SOP-00073 [04-07] - Bioanalytical Method Validation - Chromatographic Assays

SOP-00035 [02-03] - Procedures, Reporting, and Acceptance Criteria for Bioanalytical Chromatographic Projects

13. ARCHIVES

All raw data, including paper and electronic files associated with this study will remain archived at a location specified by KCAS, 10830 S Clay Blair Boulevard, Olathe, Kansas 66061 for a period of at least 10 years, after which the client may be contacted to arrange for continued storage of the files, or disposition according to an agreed procedure. KCAS will maintain an electronic (scanned/true) copy of the method validation report that can be viewed and printed on site. The study plan, documentation, raw data, and final report are indexed under Bioanalytical Project Number V1872106U3A1 and V1872106U3.

14. REFERENCES

1. Guidance for Industry: Bioanalytical Method Validation. U.S. Department of Health and Human Services. Food and Drug Administration. Center for Drug Evaluation and Research. Center for Veterinary Medicine. May 2018.

15. TABLES

Table 2: Mass Spec Transitions

Analyte	Transition
Ertapenem	m/z 476.047→431.9
Ertapenem-d_4 (IS)	m/z 480.2→436.3

Table 3: Reference Standards

Analyte	Supplier	Lot/Batch Number	Purity	Expiration Date or Retest Date	Conversion Factor	Storage Temperature
Ertapenem Sodium	MedChemExpress	180017	98.9%	Retest 11Aug2024	1.046	2-8 °C
		229333	98.43%	Retest 29Sep2025	1.046	2-8 °C
		361923	98.35%	Retest 03Mar2027	1.046	-80 °C
Ertapenem-d_4 Sodium Salt (IS)	AlsaChim	DH-ALS-20-002-P2	*	Expires 29Apr2023****	*	-20 °C
			**	Retest 26Oct2023****	**	-20 °C***
		CAY-ALS-23-025-P9	*	Expires 24Mar2024****	*	-20 °C
Zidebactam	Wockhardt	WK1/WP-0060/016	90.4%	Expires 29Mar2023****	1.000	2-8 °C
				Expires 01Mar2024****		

Certificates of analysis for the above reference standards are presented in Annex 2 (V1872106U3).
*Used as is with 1.000 conversion and 100% purity as IS only.
**Used as is with no correction for sodium or purity of 83.6%
***Desiccate- protect from moisture
****Used prior to expiration.

Table 4: Calibration Standards and QC Samples Preparation

<table>
<tr><th>Critical Solutions</th><th>Lot # for Standards (Prep Date)</th><th>Lot # for QCs (Prep Date)</th><th>Standards Lot (Prep Date)</th><th>QCS Lot (Prep Date)</th><th>Runs used</th></tr>
<tr><td colspan="3">Treated Human Urine V1872106U3-5-1</td><td rowspan="2">NA</td><td rowspan="2">V1872106U3-6-1
Set A
(17Nov2022)</td><td rowspan="2">V1872106U3 run 1 6
(LTS, high QC only)</td></tr>
<tr><td>MES buffer, pH 6.4, ~0.1M, frozen (-20°C) in polypropylene</td><td>NA</td><td>V1872106U3-2-1
(15Nov2022)</td></tr>
<tr><td colspan="3">Treated Human Urine V1872106U3-5-1</td><td rowspan="4">NA</td><td rowspan="4">Comed QC
V1872106U3-7-1
Set A
(17Nov2022)</td><td rowspan="4">V1872106U3 run 1 6
(LTS)</td></tr>
<tr><td>Deionized water frozen (-20°C) in glass</td><td>NA</td><td>V1872106U2-31-1
(20Sep2022)</td></tr>
<tr><td>MES buffer, pH 6.4, ~0.1M, frozen (-20°C) in polypropylene</td><td>NA</td><td>V1872106U3-2-1
(15Nov2022)</td></tr>
<tr><td>Treated 1:1 Urine:MES buffer, pH 6.4, ~0.1M, frozen (-70°C) in polypropylene</td><td>NA</td><td>V1872106U3-18-1
(17Nov2022)</td></tr>
<tr><td colspan="3">Treated Human Urine V1872106U3-31-1</td><td rowspan="2">NA</td><td rowspan="2">V1872106U3-32-1
Set C
(22Nov2022)</td><td rowspan="2">V1872106U3 run 1 6
(LTS, low QC only)</td></tr>
<tr><td>MES buffer, pH 6.4, ~0.1M, frozen (-20°C) in polypropylene</td><td>NA</td><td>V1872106U3-2-1
(15Nov2022)</td></tr>
<tr><td colspan="3">Treated Human Urine V1872106U3-31-1</td><td rowspan="2">NA</td><td rowspan="2">V1872106U3-39-1
Set D
(23Nov2022)</td><td rowspan="2">V1872106U3A1 ru n 2
(LTS)</td></tr>
<tr><td>MES buffer, pH 6.4, ~0.1M, frozen (-20°C) in polypropylene</td><td>NA</td><td>V1872106U3-25-1
(22Nov2022)</td></tr>
<tr><td colspan="3">Treated Human Urine V1872106U3-62-2</td><td rowspan="2">V1872106U3-62 -1
Set L
(04Feb2023)</td><td rowspan="2">V1872106U3-63-1
Set L
(04Feb2023)</td><td rowspan="2">V1872106U3 run 1 6</td></tr>
<tr><td>MES buffer, pH 6.4, ~0.1M, frozen (-20°C) in polypropylene</td><td>V1872106P5-1 09-1
(01Feb2023)</td><td>V1872106P5-109-1
(01Feb2023)</td></tr>
<tr><td colspan="3">Treated Human Urine V1872106U3A1-19-1</td><td rowspan="3">V1872106U3A1 -20-1
Set B
(05Jun2023)</td><td rowspan="3">V1872106U3A1-2 1-1
Set B
(05Jun2023)</td><td rowspan="3">V1872106U3A1 run 2, 3</td></tr>
<tr><td>MES buffer, pH 6.4, ~0.1M, frozen (-20°C) in polypropylene</td><td>V1872106U3A 1-14-1
(01Jun2023)</td><td>V1872106U3A1-14- 1
(01Jun2023)</td></tr>
<tr><td>Treated 1:1 Urine:MES buffer, pH 6.4, ~0.1M, frozen (-70°C) in polypropylene</td><td>V1872106U3A 1-19-2
(05Jun2023)</td><td>V1872106U3A1-19- 3
(05Jun2023)</td></tr>
</table>

Table 4: Calibration Standards and QC Samples Preparation (Continued)

Critical Solutions	Lot # for Standards (Prep Date)	Lot # for QCs (Prep Date)	Standards Lot (Prep Date)	QCS Lot (Prep Date)	Runs used
Treated Human Urine V1872106U3A1-19-1			NA	Comed QC V1872106U3A1-29-1 Set B (13Jun2023)	V1872106U3 A1 run 3, 5 (LTS), 6 (LTS
Deionized water frozen (-20°C) in glass	NA	B1872106U2-1-1 (19May2023)			
MES buffer, pH 6.4, ~0.1M, frozen (-20°C) in polypropylene	NA	V1872106U3A1-14-1 (01Jun2023)			
Treated 1:1 Urine:MES buffer, pH 6.4, ~0.1M, frozen (-70°C) in polypropylene	NA	V1872106U3A1-19-3 (05Jun2023)			
Treated Human Urine V1872106U3A1-10-1			V1872106U3A 1-11-1 Set L (23May2023)	V1872106U3A1-12 -1 Set L (23May2023)	V1872106U3 A1 run 4
MES buffer, pH 6.4, ~0.1M, frozen (-20°C) in glass	V1872106U3A1-4-1 (22May2023)				
Treated 1:1 Urine:MES buffer, pH 6.4, ~0.1M, frozen (-70°C) in polypropylene	V1872106U3A1-10-2 (23May2023)				
Treated Human Urine V1872106U3A1-32-1			V1872106U3A 1-37-1 Set C (15Dec2023)	V1872106U3A1-38-1 Set C (15Dec2023)	V1872106U3 A1 run 5
MES buffer, pH 6.4, ~0.1M, frozen (-20°C) in polypropylene	V1872106U3A1-30-1 (15Dec2023)				
Treated 1:1 Urine:MES buffer, pH 6.4, ~0.1M, frozen (-70°C) in polypropylene	V1872106U3A1-32-2 (15Dec2023)				
Treated Human Urine B1872106U2-17-2			V1872106U3A 1-43-1 Set D (02Feb2024)	V1872106U3A1-44-1 Set D (02Feb2024)	V1872106U3 A1 run 6
MES buffer, pH 6.4, ~0.1M, frozen (-20°C) used fresh	V1872106U3A1-39-1 (02Feb2024)				
Treated 1:1 Urine:MES buffer, pH 6.4, ~0.1M, frozen (-70°C) in polypropylene	V1872106U3A1-42-1 (02Feb2024)				

Table 4: Calibration Standards and QC Samples Preparation (Continued)

<table>
<tr><th>Critical Solutions</th><th>Lot # for Standards
(Prep Date)</th><th>Lot # for QCs
(Prep Date)</th><th>Standards Lot
(Prep Date)</th><th>QCS Lot
(Prep Date)</th><th>Runs used</th></tr>
<tr><td colspan="3">Treated Human Urine V1872106U3A1-50-1 (In-House)</td><td rowspan="3">V1872106U3A1-51-1
Set E
(15Mar2024)</td><td rowspan="3">V1872106U3A1-52-1
Set E
(15Mar2024)</td><td rowspan="4">V1872106U3A1
run 7
Comed also in V1872106U3A1 run 8</td></tr>
<tr><td>MES buffer, pH 6.4, ~0.1M, frozen (-20°C) in polypropylene</td><td colspan="2">V1872106U3A1-46-1
(14Mar2024)</td></tr>
<tr><td>Treated 1:1 Urine:MES buffer, pH 6.4, ~0.1M, frozen (-70°C) in polypropylene</td><td colspan="2">V1872106U3A1-50-2
(15Mar2024)</td></tr>
<tr><td>In addition, for Comed only - deionized water (-20°C) in glass</td><td>N/A</td><td>V1872106U3A1-54-1
(15Mar2024)</td><td>N/A</td><td>V1872106U3A1-53-1
Set E
(15Mar2024)</td></tr>
<tr><td colspan="3">Treated Human Urine B1872106U2-17-2</td><td rowspan="3">V1872106U3A1-59-1
Set F
(24Jun2024)</td><td rowspan="3">V1872106U3A1-60-1
Set F
(24Jun2024)</td><td rowspan="3">V1872106U3A1
run 8</td></tr>
<tr><td>MES buffer, pH 6.4, ~0.1M – used fresh</td><td colspan="2">V1872106U3A1-55-1
(24Jun2024)</td></tr>
<tr><td>Treated 1:1 Urine:MES buffer, pH 6.4, ~0.1M, frozen (-70°C) in polypropylene</td><td colspan="2">V1872106U3A1-59-2
(24Jun2024)</td></tr>
</table>

Table 5: Matrix Description

Matrix	Source Lot #	Date Received
Treated Human Urine V1872106U3-5-1	BioChemed BC41992HU-9	Oct2022
Treated Human Urine V1872106U3-31-1	BioChemed BC41992HU-10	Oct2022
Treated Human Urine V1872106U3-62-2	BioChemed BC41992HU-13	Oct2022
Treated Human Urine V1872106U3A1-19-1 and V1872106U3A1-10-1	BioChemed BC44737HU-4	May2023
Treated Human Urine V1872106U3A1-32-1	BioChemed BC41992HU-4	Oct2022
Treated Human Urine B1872106U2-17-2	BioChemed BC41992HU-1	Oct2022
V1872106U3A1-50-1 (In-House)	In-house – 15Mar2024-U01	Mar2024
B1872106U2-17-2	BioChemed – BC41992HU-1	Aug2022

Table 6: Summary of Results and Conclusions

Methodology – TP80182	Method Performance – LC-MS/MS	Hyperlink – N/A	Comments
Matrix	Urine		
Anticoagulant	N/A		
Assay Volume	50 mcL		
**Selectivity**			
Analyte	Ertapenem		V1872106U3
Internal Standard	Ertapenem-d_4		V1872106U3
Matrix Effect (6 spiked matrix lots)	Complies		V1872106U3
Comed Interference	Complies		V1872106U3
Carryover	Assessed with every run with standards and QCs	Summary tables Table 8<br><br>Report text Section 10.4	
**Standard Summary**			
Regression Weighting	Linear $1/x^2$		V1872106U3
Bias at LLOQ	-1.5%		V1872106U3
Bias above LLOQ	-3.8 to 4.3%		V1872106U3
Bioanalytical Range	10.0 to 4000 mcg/mL		V1872106U3
**Precision**			
Intra-assay	3.6 to 7.7% (LLOQ);<br>1.2 to 5.1% (above LLOQ)		V1872106U3
Inter-assay	6.9% (LLOQ);<br>2.2 to 3.5% (above LLOQ)		V1872106U3
**Accuracy**			
Intra-assay	-4.5 to 5.0% (LLOQ);<br>-2.7 to 4.7% (above LLOQ)		V1872106U3
Inter-assay	-0.4% (LLOQ);<br>0.3 to 4.0% (above LLOQ)		V1872106U3
**Large Run Size**	Determined to be 122 samples.		V1872106U3
**Dilution**			

Table 6: Summary of Results and Conclusions (Continued)

Methodology – TP80182	Method Performance – LC-MS/MS	Hyperlink – N/A	Comments
Dilution factor: 2x	Dilution QC: 3000 mcg/mL<br>Precision: 1.3%<br>Accuracy: -2.7%		V1872106U3
Dilution factor: 5x (run 5) - failed	Dilution QC: 16000 mcg/mL<br>Precision: 27.5%<br>Accuracy: -18.1%		V1872106U3
Dilution factor: 5x (run 8)	Dilution QC: 16000 mcg/mL<br>Precision: 3.3%<br>Accuracy: 3.8%		V1872106U3
**Adsorption**	No significant lost after 3 transfers in either polypropylene or high density polyethylene		V1872106U3
**Stability**			
Matrix (in polypropylene tubes)			
Freeze/Thaw (-20 °C/wet ice) with and without the presence of zidebactam	Demonstrated for 3 cycles		V1872106U3
Freeze/Thaw (-70 °C/wet ice) with and without the presence of zidebactam	Demonstrated for 3 cycles		V1872106U3
Bench-top stability in wet ice with and without the presence of zidebactam	Demonstrated for 24 hours		V1872106U3
Refrigeration with and without the presence of zidebactam	Demonstrated for 19 hours		V1872106U3
Long-term (-20 °C)	Demonstrated instability for 34 days low and high QCs with the presence of zidebactam<br><br>Demonstrated instability for 29 days (low QC) and 34 days (high QC) without the presence of zidebactam		V1872106U3
Long-term (-70 °C)	Demonstrated for 101 days with the presence of zidebactam.<br>Demonstrated instability for 185 and 234 days with the presence of zidebactam<br><br>Demonstrated for 194 days without the presence of zidebactam	Summary tables Table 11<br><br>Report text Section 10.5	

Table 6: Summary of Results and Conclusions (Continued)

**Methodology – TP80182**	**Method Performance – LC-MS/MS**	**Hyperlink – N/A**	**Comments**
Processed Matrix polypropylene			
Refrigeration	Demonstrated for 5 days 21 hours		V1872106U3
**Individual Sample Reinjection**	Reproducible		V1872106U3
**Whole Run Reinjection Integrity**	Reproducible<br>Duration 5 days 9 hours		V1872106U3
**Column Ruggedness**	Reproducible		V1872106U3
**Platform Change (API5000 vs API6500)**	Reproducible	Summary tables<br>Table 12<br><br>Report text<br>Section 10.6	
**Extraction Recovery**			
Ertapenem	Overall 94.8%		V1872106U3
Ertapenem-d_4 (IS)	Overall 105.2%		V1872106U3
**Solution Stability**			
Solution, Primary polypropylene			
Ertapenem in MES buffer (RT)	Stable 6.75 hours		V1872106P5
Ertapenem in MES buffer (-20 °C)	Stable 7 days		V1872106P5
Ertapenem-d_4 (IS) in MES buffer (RT)	Taken to be same as ertapenem		
Ertapenem-d_4 (IS) in MES buffer (-20 °C)	Taken to be same as ertapenem		

Table 7: Summary of Validation Run Objectives and Outcome

Run ID	Instrument ID	Extraction Date	Assay Date	Run Objective(s)	Accepted or Rejected	Comment
V1872106U3						
14	LC/MS-25	25Jan2023	30Jan2023	Long-term matrix stability with and without zidebactam	Rejected	a
15	LC/MS-24	02Feb2023	02Feb2023	Long-term matrix stability with and without zidebactam	Rejected	QCs failed %Bias criteria. LTS QCs not assessed because of run failure
16	LC/MS-22	04Feb2023	04Feb2023	Long-term matrix stability with and without zidebactam	Accepted	-70 °C LTS low failed %Bias criteria at -16.3%
V1872106U3A1						
2	LC/MS-22	05Jun2023	06Jun2023	Long-term matrix stability without zidebactam, comed QC Qualification	Accepted	b
3	LC/MS-22	13Jun2023	13Jun2023	Comed QC Qualification	Accepted	
4	LC/MS-31	22Aug2023	26Aug2023	Platform change to API6500 system	Accepted	
5	LC/MS-31	15Dec2023	19Dec2023	Long-term matrix stability with zidebactam	Accepted	c
6	LC/MS-22	02Feb2024	05Feb2024	Long-term matrix stability with zidebactam	Accepted	d
7	LC/MS-22	15Mar2024	15Mar2024	Comed QC Qualification	Accepted	
8	LC/MS-22	24Jun2024	24Jun2024	Long-term matrix stability with zidebactam	Accepted	

V1872106U3 A1 Run 1 was used for Cal/QC qualification and will not be reported.
Runs 1-13 were reported in V1872106U3

[a] Run QCs failed %Bias criteria. No assessment of the LTS QCs could be made. Run was reprepared for analysis. The calibration curve was bending significantly which contributed to the failure. Reinjection of the run did not help.

[b] The acceptance criteria were not met at the low QCs for the comed QCs due to a preparation error as the low QC was ~5 times higher than the nominal concentration. Data is not reported.

[c] LTS runs ~25% higher than cals and QCs at both levels. Experiment was repeated to confirm high bias does not show instability and Time 0 for the LTS samples passed well.

[d] Stability QCs showed >15% bias which matched the previous experiment.

Table 8: Carryover

Run ID	Result (Extracted Blank after High Standard)	Carryover Results for Internal Standard
V1872106U3		
16	Seq 13 and Seq 56 are <20.0% of the LLOQ.	Seq 4 and Seq 47 are < 5.0% internal standard peak of acceptable standards.
V1872106U3A1		
2	Seq 13 and Seq 57 are <20.0% of the LLOQ.	Seq 4 and Seq 48 are < 5.0% internal standard peak of acceptable standards.
3	Seq 13 and Seq 44 are <20.0% of the LLOQ.	Seq 4 and Seq 35 are < 5.0% internal standard peak of acceptable standards.
4	Seq 13 and Seq 50 are <20.0% of the LLOQ.	Seq 4 and Seq 41 are < 5.0% internal standard peak of acceptable standards.
5	Seq 13 and Seq 44 are <20.0% of the LLOQ.	Seq 4 and Seq 35 are < 5.0% internal standard peak of acceptable standards.
6	Seq 13 and Seq 44 are <20.0% of the LLOQ.	Seq 4 and Seq 35 are < 5.0% internal standard peak of acceptable standards.
7	Seq 13 and Seq 44 are <20.0% of the LLOQ.	Seq 4 and Seq 35 are < 5.0% internal standard peak of acceptable standards.
8	Seq 13 and Seq 44 are <20.0% of the LLOQ.	Seq 4 and Seq 35 are < 5.0% internal standard peak of acceptable standards.

Note: Calibration Standards and QCs are arranged in a way to mitigate carryover in all validation runs.

Table 9: Standards and QCs of Non-A&P Runs

Analytical Performance: Back-Calculated Concentrations (µg/mL) of Ertapenem Calibration Standard in (Human) (Urine) in (Study V1872106U3A1)

Assay Date	Run ID	Cal 10 10.0 µg/mL	Cal 20 20.0 µg/mL	Cal 160 160 µg/mL	Cal 400 400 µg/mL	Cal 800 800 µg/mL	Cal 1600 1600 µg/mL	Cal 3200 3200 µg/mL	Cal 4000 4000 µg/mL
06-Jun-2023	2	9.72	20.4	166	395	799	1550	3040	3730
		9.98	20.5	175	426	827	1610	3040	3870
13-Jun-2023	3	9.55	19.8	166	412	816	1520	3020	3640
		10.4	20.1	173	423	809	1630	3120	3890
19-Dec-2023	5	9.39	20.8	173	411	828	1610	3150	3860
		10.1	20.6	169	416	802	1420	3020	3770
05-Feb-2024	6	9.35	20.9	167	403	823	1590	3130	3850
		10.3	20.3	163	406	803	1610	3100	3780
15-Mar-2024	7	9.82	20.2	165	413	822	1620	3040	3770
		9.86	20.8	165	414	809	1580	3120	3760
24-Jun-2024	8	10.3	21.8	158	402	781	1570	3030	3700
		9.16	20.1	168	407	829	1650	3260	3890
Mean		9.83	20.5	167	411	812	1580	3090	3790
S.D.		0.407	0.521	4.74	8.78	14.6	61.8	71.9	80.2
%CV		4.1	2.5	2.8	2.1	1.8	3.9	2.3	2.1
%Bias		-1.7	2.5	4.4	2.8	1.5	-1.3	-3.4	-5.3
n		12	12	12	12	12	12	12	12

Calibration Curve Parameters for Ertapenem Calibration Standards in Human Urine (Study V1872106U3A1)

Run Date	Run ID	Slope	Intercept	R-Squared	LLOQ	ULOQ
06-Jun-2023	2	0.00568	0.00385	0.9976	10.0	4000
13-Jun-2023	3	0.00560	0.00597	0.9973	10.0	4000
19-Dec-2023	5	0.00228	0.00211	0.9966	10.0	4000
05-Feb-2024	6	0.00236	0.00209	0.9986	10.0	4000
15-Mar-2024	7	0.00391	0.00333	0.9985	10.0	4000
24-Jun-2024	8	0.00892	0.01468	0.9973	10.0	4000

Table 9: Standards and QCs of Non-A&P Runs (Continued)

Human Urine Ertapenem Concentrations in Quality Controls for 6 separate batch runs (Study V1872106U3A1)

Run Date	Run ID	QCS 30 30.0 µg/mL	%Bias	QCS 2000 2000 µg/mL	%Bias	QCS 3000 3000 µg/mL	%Bias
06-Jun-2023	2	31.5	5.0	2000	0.0	2930	-2.3
		32.6	8.7	2060	3.0	3010	0.3
Intrarun Mean		32.1		2030		2970	
Intrarun %Bias		7.0		1.5		-1.0	
13-Jun-2023	3	32.0	6.7	2050	2.5	3050	1.7
		32.5	8.3	2090	4.5	2940	-2.0
Intrarun Mean		32.3		2070		3000	
Intrarun %Bias		7.7		3.5		0.0	
19-Dec-2023	5	30.2	0.7	1970	-1.5	3010	0.3
		30.3	1.0	1970	-1.5	2800	-6.7
Intrarun Mean		30.3		1970		2910	
Intrarun %Bias		1.0		-1.5		-3.0	
05-Feb-2024	6	31.9	6.3	2080	4.0	3040	1.3
		32.1	7.0	2080	4.0	3080	2.7
Intrarun Mean		32.0		2080		3060	
Intrarun %Bias		6.7		4.0		2.0	
15-Mar-2024	7	30.6	2.0	1970	-1.5	2920	-2.7
		30.3	1.0	1980	-1.0	2920	-2.7
Intrarun Mean		30.5		1980		2920	
Intrarun %Bias		1.7		-1.0		-2.7	
24-Jun-2024	8	30.5	1.7	1960	-2.0	2840	-5.3
		27.7	-7.7	1980	-1.0	2920	-2.7
Intrarun Mean		29.1		1970		2880	
Intrarun %Bias		-3.0		-1.5		-4.0	

Criteria: Run acceptance criteria needs to be met – see Section 10.3.
Results: Acceptance criteria were met.

Table 10: Standard and QC of Failed Runs

V1872106U3:

Analytical Performance: Back-Calculated Concentrations (µg/mL) of Ertapenem Calibration Standard in (Human) (Urine) in (Study V1872106U3) (Rejected Runs)

Assay Date	Run ID	Cal 10 10.0 µg/mL	Cal 20 20.0 µg/mL	Cal 160 160 µg/mL	Cal 400 400 µg/mL	Cal 800 800 µg/mL	Cal 1600 1600 µg/mL	Cal 3200 3200 µg/mL	Cal 4000 4000 µg/mL
30-Jan-2023	14	9.70	*24.0	179	449	893	1740	2780	*3310
		10.1	20.5	163	391	787	1510	2860	3470
02-Feb-2023	15	9.84	19.5	174	407	790	1480	2780	*2860
		10.1	20.4	175	409	873	1620	2900	*2670
Mean		9.94	20.1	173	414	836	1590	2830	3470
S.D.		0.199	0.551	6.85	24.7	55.2	118	60.0	
%CV		2.0	2.7	4.0	6.0	6.6	7.4	2.1	
%Bias		-0.6	0.5	8.1	3.5	4.5	-0.6	-11.6	-13.3
n		4	3	4	4	4	4	4	1
Reason Deactivated									
* Excluded due to being outside acceptance criteria									

Calibration Curve Parameters for Ertapenem Calibration Standards in Human Urine (Study V1872106U3) (Rejected Runs)

Run Date	Run ID	Slope	Intercept	R-Squared	LLOQ	ULOQ
30-Jan-2023	14	0.00802	-0.00256	0.9904	10.0	4000
02-Feb-2023	15	0.00840	0.00829	0.9941	10.0	3200

Table 10: Standard and QC of Failed Runs (Continued)

Human Urine Ertapenem Concentrations in Quality Controls for 2 separate batch runs (Study V1872106U3) (Rejected Runs)

Run Date	Run ID	QCS 10 10.0 µg/mL	%Bias	QCS 30 30.0 µg/mL	%Bias	QCS 2000 2000 µg/mL	%Bias	QCS 3000 3000 µg/mL	%Bias
30-Jan-2023	14	9.66	-3.4	##24.1	-19.7	##1540	-23.0	##2230	-25.7
		8.68	-13.2	##24.8	-17.3	##1530	-23.5	##2260	-24.7
		8.69	-13.1	##24.5	-18.3	##1530	-23.5	##2430	-19.0
		8.38	-16.2	##24.7	-17.7	##1530	-23.5	##2150	-28.3
		9.47	-5.3	25.8	-14.0	##1520	-24.0	##2230	-25.7
		9.07	-9.3	##25.3	-15.7	1900	-5.0	##2210	-26.3
Intrarun Mean		8.99		24.9		1590		2250	
Intrarun SD		0.499		0.602		151		94.7	
Intrarun %CV		5.6		2.4		9.5		4.2	
Intrarun %Bias		-10.1		-17.0		-20.5		-25.0	
n		6		6		6		6	
02-Feb-2023	15	#7.41	-25.9	##23.1	-23.0	##1600	-20.0	##2290	-23.7
		#6.83	-31.7	##20.6	-31.3	##1540	-23.0	##2190	-27.0
		#6.51	-34.9	##21.0	-30.0	##1430	-28.5	##2240	-25.3
		#7.30	-27.0	##24.2	-19.3	##1590	-20.5	##2290	-23.7
		#6.53	-34.7	26.7	-11.0	##1520	-24.0	##2100	-30.0
		#7.30	-27.0	##24.1	-19.7	##1560	-22.0	##2230	-25.7
Intrarun Mean		6.98		23.3		1540		2220	
Intrarun SD		0.409		2.26		61.6		71.5	
Intrarun %CV		5.9		9.7		4.0		3.2	
Intrarun %Bias		-30.2		-22.3		-23.0		-26.0	
n		6		6		6		6	
# > 20%Bias									
## > 15%Bias									

Criteria: Run acceptance criteria needs to be met – see Section 10.3.
Results: See Table 7 for run conclusions.

Table 11: Long-Term Stability at -70 °C

V1872106U3:

Analytical Performance: Back-Calculated Concentrations (µg/mL) of Ertapenem Calibration Standard in (Human) (Urine) in (Study V1872106U3)

Assay Date	Run ID	Cal 10 10.0 µg/mL	Cal 20 20.0 µg/mL	Cal 160 160 µg/mL	Cal 400 400 µg/mL	Cal 800 800 µg/mL	Cal 1600 1600 µg/mL	Cal 3200 3200 µg/mL	Cal 4000 4000 µg/mL
04-Feb-2023	16	10.5	19.6	173	403	818	1500	3070	3780
		9.28	21.0	165	433	860	1620	3020	3630
Mean		9.89	20.3	169	418	839	1560	3050	3710
%Bias		-1.1	1.5	5.6	4.5	4.9	-2.5	-4.7	-7.3

Calibration Curve Parameters for Ertapenem Calibration Standards in Human Urine (Study V1872106U3)

Run Date	RunID	Slope	Intercept	R-Squared	LLOQ	ULOQ
04-Feb-2023	16	0.00294	0.00626	0.9957	10.0	4000

Analytical Performance of Ertapenem Quality Control Samples in Human Urine (Study V1872106U3)

Run Date	Run ID	QCS 30 30.0 µg/mL	%Bias	QCS 2000 2000 µg/mL	%Bias	QCS 3000 3000 µg/mL	%Bias
04-Feb-2023	16	32.5	8.3	2140	7.0	2990	-0.3
		33.1	10.3	2080	4.0	2960	-1.3
Mean		32.8		2110		2980	
%Bias		9.3		5.5		-0.7	

V1872106U3:

With and without presence of zidebactam

Run Date	Run ID	Comed1 LTS -70 low 30.0 µg/mL	%Bias	LTS -70 low 30.0 µg/mL	%Bias	Comed1 LTS -70 high 3000 µg/mL	%Bias	LTS -70 high 3000 µg/mL	%Bias
04-Feb-2023	16	30.3	1.0	25.7	-14.3	2750	-8.3	2780	-7.3
(79 days)		30.4	1.3	#23.3	-22.3	2860	-4.7	2910	-3.0
		31.1	3.7	26.6	-11.3	2720	-9.3	2870	-4.3
		28.6	-4.7	#25.1	-16.3	2780	-7.3	2850	-5.0
		30.8	2.7	#24.4	-18.7	2930	-2.3	2850	-5.0
		29.5	-1.7	#25.3	-15.7	2750	-8.3	2950	-1.7
Mean		30.1		25.1		2800		2870	
S.D.		0.920		1.13		80.4		58.1	
%CV		3.1		4.5		2.9		2.0	
%Bias		0.3		-16.3		-6.7		-4.3	
n		6		6		6		6	
# > 15%Bias									

Table 11: Long-Term Stability at -70 °C (Continued)

V1872106U3A1:

Time 0 for Comed QC (Presence of Zidebactam)

Run Date	Run ID	Comed QC Low 30.0 µg/mL	%Bias	Comed QC High 3000 µg/mL	%Bias
13-Jun-2023	3	32.9	9.7	2830	-5.7
		28.5	-5.0	2770	-7.7
		30.6	2.0	2960	-1.3
		27.2	-9.3	2790	-7.0
		27.6	-8.0	2740	-8.7
		28.6	-4.7	2740	-8.7
Intrarun Mean		29.2		2810	
Intrarun SD		2.15		83.1	
Intrarun %CV		7.4		3.0	
Intrarun %Bias		-2.7		-6.3	
n		6		6	
15-Mar-2024	7	31.4	4.7	2970	-1.0
		30.9	3.0	2940	-2.0
		30.9	3.0	2940	-2.0
		30.3	1.0	3020	0.7
		30.7	2.3	2960	-1.3
		30.0	0.0	2960	-1.3
Intrarun Mean		30.7		2970	
Intrarun SD		0.494		29.5	
Intrarun %CV		1.6		1.0	
Intrarun %Bias		2.3		-1.0	
n		6		6	

Criteria: See Section 10.5
Results: The acceptance criteria were met.

Without presence of zidebactam

Run Date	Run ID	LTS -70 low 30.0 µg/mL	%Bias	LTS -70 high 3000 µg/mL	%Bias
06-Jun-2023	2	29.7	-1.0	2830	-5.7
(194 days)		28.3	-5.7	2820	-6.0
		29.8	-0.7	2790	-7.0
		29.5	-1.7	2850	-5.0
		30.6	2.0	2860	-4.7
		29.7	-1.0	2910	-3.0
Mean		29.6		2840	
S.D.		0.743		40.8	
%CV		2.5		1.4	
%Bias		-1.3		-5.3	
n		6		6	

Table 11: Long-Term Stability at -70 °C (Continued)

With presence of zidebactam

Run Date	Run ID	Comed1 LTS -70 low 30.0 µg/mL	%Bias	Comed1 LTS -70 high 3000 µg/mL	%Bias
19-Dec-2023	5	#38.1	27.0	3320	10.7
(185 days)		#35.9	19.7	#3770	25.7
		#37.9	26.3	#3760	25.3
		#38.7	29.0	#3770	25.7
		#38.8	29.3	#3630	21.0
		#36.6	22.0	#3810	27.0
Intrarun Mean		37.7		3680	
Intrarun SD		1.17		185	
Intrarun %CV		3.1		5.0	
Intrarun %Bias		25.7		22.7	
n		6		6	
05-Feb-2024	6	#39.7	32.3	#3990	33.0
(234 days)		#41.5	38.3	#3990	33.0
		#41.5	38.3	#3950	31.7
		#38.9	29.7	#4020	34.0
		#40.9	36.3	#4000	33.3
		#41.5	38.3	#3980	32.7
Intrarun Mean		40.7		3990	
Intrarun SD		1.11		23.2	
Intrarun %CV		2.7		0.6	
Intrarun %Bias		35.7		33.0	
n		6		6	
24-Jun-2024	8	30.4	1.3	2910	-3.0
(101 days)		31.7	5.7	2940	-2.0
		33.5	11.7	3120	4.0
		32.2	7.3	2920	-2.7
		33.8	12.7	3110	3.7
		32.3	7.7	3170	5.7
Intrarun Mean		32.3		3030	
Intrarun SD		1.24		117	
Intrarun %CV		3.8		3.9	
Intrarun %Bias		7.7		1.0	
n		6		6	
# > 15%Bias					

Criteria: See Section 10.5.

Results: Ertapenem is stable in treated human urine without presence of zidebactam for up to 194 days upon storage in polypropylene tube at -70 °C. Ertapenem is stable in treated human urine with presence of zidebactam for up to 101 days upon storage in polypropylene tube at -70 °C. V1872106U3 run 16 -70 °C LTS without zidebactam low QC failed %Bias criteria at -16.3%. The high QC meets criteria and it does not appear to be stability related as the high QC does not show the same trend. This is confirmed since the 194-day stability passes all criteria in run 2. V1872106U3A1 runs 5 and 6 -70 °C LTS failed %Bias high at both levels.

Table 12: Platform Change (API5000 to API6500)

Analytical Performance: Back-Calculated Concentrations (µg/mL) of Ertapenem Calibration Standard in (Human) (Urine) in (Study V1872106U3A1)

Assay Date	Run ID	Cal 10 10.0 µg/mL	Cal 20 20.0 µg/mL	Cal 160 160 µg/mL	Cal 400 400 µg/mL	Cal 800 800 µg/mL	Cal 1600 1600 µg/mL	Cal 3200 3200 µg/mL	Cal 4000 4000 µg/mL
26-Aug-2023	4	10.7	17.1	154	432	775	1770	3010	3940
		9.41	22.5	166	366	787	1730	3050	*3160
Mean		10.1	19.8	160	399	781	1750	3030	3940
%Bias		1.0	-1.0	0.0	-0.3	-2.4	9.4	-5.3	-1.5
Reason Deactivated									
* Excluded due to being outside acceptance criteria									

Calibration Curve Parameters for Ertapenem Calibration Standards in Human Urine (Study V1872106U3A1)

Run Date	Run ID	Slope	Intercept	R-Squared	LLOQ	ULOQ
26-Aug-2023	4	0.00602	0.00600	0.9922	10.0	4000

Analytical Performance of Ertapenem Quality Control Samples in Human Urine (Study V1872106U3A1)

Run Date	Run ID	QCS 10 10.0 µg/mL	%Bias	QCS 30 30.0 µg/mL	%Bias	QCS 2000 2000 µg/mL	%Bias	QCS 3000 3000 µg/mL	%Bias
26-Aug-2023	4	9.01	-9.9	32.9	9.7	1980	-1.0	2650	-11.7
		9.35	-6.5	31.1	3.7	1860	-7.0	3020	0.7
		9.89	-1.1	28.5	-5.0	2070	3.5	3060	2.0
		10.2	2.0	33.9	13.0	2150	7.5	2770	-7.7
		8.43	-15.7	26.4	-12.0	1960	-2.0	3260	8.7
		9.91	-0.9	29.9	-0.3	#1620	-19.0	2560	-14.7
Mean		9.47		30.5		1940		2890	
S.D.		0.665		2.79		185		270	
%CV		7.0		9.1		9.5		9.3	
%Bias		-5.3		1.7		-3.0		-3.7	
n		6		6		6		6	
# > 15%Bias									

Note: Run 4 was injected on a different MS platform (Applied Biosystems API6500).

Criteria: Data were required to meet the accuracy and precision criteria outlined in Section 10.6 to be the considered as reproducible/rugged method.

Results: The acceptance criteria were met.

1. TITLE: THE DETERMINATION OF ERTAPENEM IN TREATED HUMAN URINE BY LC-MS/MS

BIOANALYTICAL METHOD VALIDATION REPORT

Bioanalytical Project Number:	V1872106U3
Sponsor:	DynPort Vaccine Company, LLC<br>110 Thomas Johnson Drive<br>Suite 200<br>Frederick, MD 21702
Bioanalytical Laboratory:	KCAS Bioanalytical & Biomarker Services.<br>10830 S Clay Blair Boulevard<br>Olathe, KS 66061
Issue Date:	February 2023

2. SYNOPSIS

This report presents the validation data for an LC-MS/MS method for the determination of ertapenem in treated human urine. The assay was analyzed in the range of 10.0 - 4000 mcg/mL and is for use on treated human urine samples obtained during clinical trials.

3. TABLE OF CONTENTS FOR THE BIOANALYTICAL METHOD VALIDATION REPORT

1. TITLE: THE DETERMINATION OF ERTAPENEM IN TREATED HUMAN URINE BY LC-MS/MS 1
2. SYNOPSIS 2
3. TABLE OF CONTENTS FOR THE BIOANALYTICAL METHOD VALIDATION REPORT 3
4. LIST OF TABLES 4
5. LIST OF FIGURES 6
6. SIGNATURE PAGE 7
7. QUALITY ASSURANCE STATEMENT 8
8. STUDY COMPLIANCE STATEMENT 9
9. LIST OF ABBREVIATIONS AND DEFINITIONS OF TERMS 10
10. INTRODUCTION 12
11. EXPERIMENTAL DESIGN AND RESULTS 12
11.1. Reference Standards 12
11.2. Calibration Standards and QC Samples Preparation 12
11.3. Selectivity 13
11.3.1. Blank Evaluation 13
11.3.2. Matrix Effect 13
11.3.3. Comed Interference Evaluation 14
11.3.4. Carryover 14
11.4. Linearity and Analytical Range 14
11.5. Precision and Accuracy 15
11.6. Sensitivity 15
11.7. Run Size 15
11.8. Precision and Accuracy of Dilution 16
11.9. Analyte Adsorption to Container Test 16
11.10. Freeze/Thaw Stability 16
11.11. Bench-Top Stability in Wet Ice 17

11.12. Refrigeration Stability (Treated and Untreated) 17
11.13. Long-Term Matrix Stability at -20 °C and -70 °C 17
11.14. Processed Sample Stability when Refrigerated 18
11.15. Individual Sample Reinjection Reproducibility 18
11.16. Whole Run Reinjection Reproducibility 18
11.17. Column Ruggedness 19
11.18. Extraction Recovery 19
11.19. Solution Stabilities 19
11.20. Calculations 20
11.21. SOP Deviations and Investigations 20
12. CONCLUSION 21
13. PERTINENT SOPS 21
14. ARCHIVES 21
15. REFERENCES 21
16. TABLES 22
17. FIGURES 53

4. LIST OF TABLES

Table 1: Abbreviations and Codes 10
Table 2: Deviations 20
Table 3: Mass Spec Transitions 22
Table 4: Reference Standards 22
Table 5: Calibration Standards and QC Samples Preparation 23
Table 6: Matrix Description 23
Table 7: Summary of Results and Conclusions 24
Table 8: Summary of Validation Run Objectives and Outcome 28
Table 9: Carryover 30
Table 10: Selectivity - Blank Assessment 30
Table 11: Matrix Effect – Low Concentration 31

Table 12: Matrix Effect – High Concentration 32
Table 13: Comed Interference Evaluation 33
Table 14: Summary of Standard Curves 34
Table 15: Precision and Accuracy (Intra-Assay) 35
Table 16: Precision and Accuracy (Inter-Assay) 36
Table 17: Standard and QC of Failed Runs 37
Table 18: Standards and QCs of Non-P&A Runs 38
Table 19: Precision and Accuracy of Sample Dilution 40
Table 20: Analyte Adsorption Container Test 41
Table 21: Freeze-Thaw Stability, (Frozen At -20 °C and -70 °C Thawed in Ice-Water Bath) 42
Table 22: Bench-Top Stability in Wet Ice 43
Table 23: Refrigeration Stability 44
Table 24: Long-Term Stability at -20 °C and -70 °C 45
Table 25: Refrigerated Processed Sample Stability 46
Table 26: Individual Sample Reinjection Reproducibility 47
Table 27: Whole Run Reinjection Reproducibility and Column Ruggedness 48
Table 28: Extraction Recovery – Ertapenem 49
Table 29: Extraction Recovery – Ertapenem-d_4 50
Table 30: Stability of Ertapenem Primary Solution in MES Buffer at Room Temperature 51
Table 31: Stability of Ertapenem Primary Solution in MES Buffer, ~0.1M, pH 6.4 at -20 °C 52

5. LIST OF FIGURES

Figure 1: Typical Calibration Curve 53
Figure 2: Typical Ion Chromatogram Blank Human Urine Without Internal Standard 54
Figure 3: Typical Ion Chromatogram Blank Human Urine With Internal Standard 55
Figure 4: Typical Ion Chromatogram LLOQ Calibration Standard, Ertapenem = 10.0 mcg/mL 56
Figure 5: Typical Ion Chromatogram Calibration Standard, Ertapenem = 1600 mcg/mL 57
Figure 6: Typical Ion Chromatogram ULOQ Calibration Standard, Ertapenem = 4000 mcg/mL 58
Figure 7: Typical Ion Chromatogram Cross-Talk Evaluation High QC No IS 59

Annex 1 .... Test Procedure
Annex 2 .... Certificate(s) of Analysis

6. SIGNATURE PAGE

Bioanalytical Project Number: V1872106U3

Issue Date: February 2023

APPROVED

DocuSigned by:

Anika Pippin

Signer Name: Anika Pippin
Signing Reason: I approve this document
Signing Time: 21-Feb-2023 | 13:01 CST
BE3AA1198E40459E8A7E3C615D981D82

Anika Pippin, B.S.
Principal Investigator

REVIEWED

DocuSigned by:

Marsha Luna

Signer Name: Marsha Luna
Signing Reason: I have reviewed this document
Signing Time: 21-Feb-2023 | 12:50 CST
C9CC738561AF4CC1B375ED013DD1E4BD

Marsha Luna, B.S.
Management

7. QUALITY ASSURANCE STATEMENT

This Bioanalytical Method Validation Report has been found to describe accurately the methods and SOPs used and to accurately reflect the raw data collected. The Quality Assurance Unit initiated audits on this study and reported the findings to the Principal Investigator (PI) and Management as follows:

		Date(s) Sent to:	
**Inspection Date(s)**	**Inspection Phase**	**PI**	**Management**
07Dec2022 & 08Dec2022	Data Audit	08Dec2022	29Dec2022
20Dec2022	Data Audit	20Dec2022	29Dec2022
20Dec2022 & 21Dec2022	Data Audit	21Dec2022	09Jan2023
05Jan2023	Draft Report Review	05Jan2023	26Jan2023
12Jan2023	Data Audit	12Jan2023	12Jan2023
12Jan2023	Data Audit	12Jan2023	27Jan2023
15Feb2023 and 16Feb2023	Data Audit	16Feb2023	21Feb2023
16Feb2023	Data Audit	16Feb2023	21Feb2023
20Feb2023	Updated Draft Report Review	20Feb2023	20Feb2023
21Feb2023	Final Report Review	21Feb2023	21Feb2023

REVIEWED

DocuSigned by:

Monica Pirez

Signer Name: Monica Pirez
Signing Reason: I have reviewed this document
Signing Time: 21-Feb-2023 | 12:49 CST
518486CF3F7A41039BB7811A9B22618B

Monica Pirez, B.S.
Quality Assurance Unit

8. STUDY COMPLIANCE STATEMENT

I, the undersigned, hereby declare that this study was conducted in compliance with the relevant bioanalytical aspects of FDA guidelines, GLP documentation and reporting GLP regulations 21CFR Part 58 and KCAS Bioanalytical & Biomarker Services (KCAS) procedures and that the work was performed by me, or under my direction, and the report reflects a true and accurate record of the results obtained.

The acceptance criteria are based on the FDA Guidance for Industry, Bioanalytical Method Validation (2018). [1]

APPROVED

DocuSigned by:

Anika Pippin

Signer Name: Anika Pippin
Signing Reason: I approve this document
Signing Time: 21-Feb-2023 | 13:01 CST
BE3AA1198E40459E8A7E3C615D981D82

Anika Pippin, B.S.
Principal Investigator

9. LIST OF ABBREVIATIONS AND DEFINITIONS OF TERMS

Table 1: Abbreviations and Codes

Abbreviation	Definition
<	Concentration less than the lowest acceptable calibration standard
>	Concentration exceeded the upper limit of calibration range (> ULOQ)
A	Amendment
AQL	Above Quantitation Limit
%Bias	Percentage measurement valid
Batch	Is the same as Run
BQL	Below Quantifiable Limit
Cal	Calibration standard
Conc.	Concentration
Conv.	Conversion
%CV	Coefficient of Variation
dev	Deviation
diff	Difference
EMA	European Medicines Agency
FDA	Food and Drug Administration
GLP	Good Laboratory Practice
HPLC	High Performance Liquid Chromatography
ID	Identification
IS	Internal Standard
ISR	Incurred Sample Reanalysis
K_2EDTA or K_3EDTA	Dipotassium or Tripotassium Ethylenediaminetetraacetic Acid
KCAS	KCAS Bioanalytical & Biomarker Services
LC-MS/MS	Liquid Chromatography Tandem Mass Spectrometry
LLOQ	Lower Limit of Quantitation
mcg or µg or ug	Microgram
mcL or µL or uL	Microliter

Table 1: Abbreviations and Codes (Continued)

Abbreviation	Definition
ME	Matrix Effect
MF	Matrix Factor
MHLW	Ministry of Health, Labour and Welfare of Japan
mL	Milliliter
MS	Mass Spectrometry
n	number
N.C.	Not Calculated
ND	No difference reported as ISR will be counted
ng	nanogram
n.report	No Data available to report
N/A or NA	Not Applicable
N.R.	Not Reported
P&A	Precision and Accuracy
p.a.r.	Peak Area Ratio
PI	Principal Investigator
QC	Quality Control Sample
QCs	Quality Control Samples
OECD	Organization for Economic Co-operation and Development
RT	Room Temperature
sd or SD	Standard deviation
Seq	Sequence
SOP (s)	Standard Operating Procedure (s)
temp	Temperature
TP	Test procedure
UHPLC	Ultra High Performance Liquid Chromatography
ULOQ	Upper Limit of Quantitation
WIS	Working Internal Standard

This is a basic list. Some of the abbreviations may not have been used in this report.

10. INTRODUCTION

The objective of this study was to perform a full validation of a bioanalytical method for the determination of ertapenem in MES (0.1M [2-(N-Morpholino) ethane sulphonic acid hydrate, pH 6.4])-treated human urine (at a 1:1 ratio). This validation report describes the selectivity, sensitivity, linearity, accuracy, precision, stability and recovery experiments based on an established bioanalytical test procedure.

The validation procedure was designed to meet or exceed the requirements of FDA Guidance for Industry on Bioanalytical Methods Validation, May 2018. The software programs used were Microsoft Office Excel 365, acquisition and processing software Analyst 1.7.2, and data management software Watson LIMS 7.5.

11. EXPERIMENTAL DESIGN AND RESULTS

In this method, ertapenem and internal standard, ertapenem-d_4, were released from human urine using 96 well protein precipitation. After extraction, the sample was chromatographed using reversed phase HPLC with a Fortis Technology Ltd. Fortis C18 analytical column. Ertapenem and ertapenem-d_4 (IS) were detected by monitoring the precursor and product ions described in Table 3 using an Applied Biosystems Sciex API 5000 LC-MS/MS. The quantification was based on setting a calibration graph using the internal standard method based on bioanalytical test procedure TP80182, entitled “LC-MS/MS Analysis of Ertapenem in Treated Human Urine (50 mcL, 10.0 - 4000 mcg/mL; Prior to MES Treatment)”. The test procedure can be found in Annex 1.

The human urine was treated with 0.1M 2-(N-Morpholino) ethane sulphonic acid hydrate (MES) buffer, pH 6.4 at a 1:1 ratio. The validation assay range is calculated based on urine volume only to mimic unknown study samples and no concentration adjustment is needed during sample analysis since the unknown samples are treated in the same manner. The assay range is 10.0 to 4000 mcg/mL in urine.

A summary of the validation results is provided in Table 7. A summary describing the objective(s) and outcome of each validation run attempted is provided in Table 8.

The method was validated within the analytical range of 10.0 - 4000 mcg/mL in human urine. No development work was performed after the method was validated.

11.1. Reference Standards

Reference standards used during this validation are presented in Table 4.

11.2. Calibration Standards and QC Samples Preparation

Calibration standards and quality control pools were prepared by spiking appropriate levels of ertapenem into human urine. Primary reference standards will be prepared in at least duplicate,

and they need to compare within 5% of each other. Standards and QCs were prepared and stored frozen at KCAS at -70 °C. Stability QCs were also stored at -20 °C and -70 °C. Standards, QCs, and the critical solutions used during this validation are described in Table 5, and matrix used is described in Table 6.

11.3. Selectivity

Selectivity is the ability of the bioanalytical method to measure and differentiate ertapenem in the presence of components that may be expected to be present. These could include metabolites, impurities, degradants, or matrix components. The selectivity of this method was achieved by a sample clean-up procedure using protein precipitation followed by chromatography and specific detection of the precursor and product ions of each analyte using tandem mass spectrometry (MS/MS).

11.3.1. Blank Evaluation

A minimum of six different sources of treated human urine were screened. To be deemed acceptable, the peak response observed must be ≤ 20.0% of the mean response of the lower limit of quantitation (LLOQ) and ≤ 5.0% of the mean response of the internal standard. If any lots fail, further evaluation is needed.

All matrices lots were found to be free of any significant interference at the retention times of the analytes of interest. Data is presented in Table 10. Typical chromatograms for blank urine without and with internal standard are presented in Figure 2 and Figure 3, respectively. Cross talk was also evaluated by extracting the high QC with no internal standard. There are no apparent effects of cross talk, and a typical chromatogram is present in Figure 7.

11.3.2. Matrix Effect

Matrix effect in MS applications is defined as inter-matrix variation in signal due to co-eluting matrix substances which cause enhancement or suppression of the detector response for the measured analyte.

To evaluate matrix effect using matrix factor, ertapenem and ertapenem-d_4 (IS) were spiked in four replicates into six different lots of extracted human urine. Ertapenem and ertapenem-d_4 (IS) were also spiked in four replicates into a proper extracted solvent or neat solution for comparison.

For ertapenem and ertapenem-d_4 (IS), the matrix factor (MF) was calculated for each lot of human urine using the following equation:

$$MF = \frac{\text{Peak Response in Presence of Matrix Substances}}{\text{Mean Peak Response in Absence of Matrix}}$$

The IS normalized MF was calculated using the following equation:

$$\text{IS Normalized MF} = \frac{MF_{Analyte}}{MF_{IS}}$$

The evaluation was conducted for Ertapenem at a high (close to ULOQ) and a low concentration (maximum of three times the LLOQ) and then the MF and IS Normalized MF were calculated for each lot of matrix. To be deemed acceptable for matrix effect test, the %CV for IS Normalized MF must be ≤ 15.0% over all six lots.

Matrix effect data are presented in Table 11 to Table 12. The matrix effect results meet acceptance criteria.

11.3.3. Comed Interference Evaluation

Assay selectivity was confirmed by spiking quality control pools with 3000 mcg/mL (based on urine volume only) of zidebactam. These QC pools, containing low and high levels of ertapenem, were analyzed in replicates in six in a single run. To be considered acceptable, the mean %Bias at each level was also required to be within ± 15.0% and the %CV was required to be ≤ 15.0%.

Run 3 interference results cannot be reported, because the run failed QC acceptance. However, the interference QCs compared to the passing calibration curve. Evaluation was repeated in run 10. The high QC level meets criteria; however, the low QC level shows a >15% bias. This appears to be an extraction issue and not a prep issue since all the criteria for the comed pool in the freeze/thaw and long-term stability assessment met criteria. The low comed QC met all criteria in run 11 where both the low and high comed QC met all acceptance criteria for 34 days at -70°C storage. Since the interference QC assessment does not need to be done from a fresh preparation, the run 11 data is used to confirm that the presence of zidebactam does not affect the analysis of ertapenem. The results are presented in Table 13.

11.3.4. Carryover

Carryover is evaluated within each run. It consists of measuring the response for each analyte and if it exceeds 20.0% of the response of LLOQ standard or other sample reflecting the lower sensitivity limit required, a carryover assessment is completed, and affected samples are rejected. The internal standard carryover evaluation is assessed within each run and if it exceeds 5.0% of the average internal standard response of acceptable standards and QCs, impact is evaluated within the run. Data are presented in Table 9.

11.4. Linearity and Analytical Range

The calibration curve was constructed with eight different, non-zero standard concentrations ranging from 10.0 - 4000 mcg/mL in treated human urine (equivalent to 5.00 - 2000 mcg/mL in untreated human urine). Calibration standards were processed in duplicate and the calibration curve was evaluated using least squares linear regression with weighting $1/x^2$. The maximum allowable percent deviation from nominal for each standard was ± 15.0%, except at the LLOQ,

where ± 20.0% was accepted. Any standard deviating by more than these limits was required to be excluded from the regression line. For the calibration curve to be accepted, at least 75% of the initial number of standards was required to remain in the curve.

The calibration curve was found to be linear. The data are presented in Table 14. A typical calibration curve is presented graphically in Figure 1. Typical chromatograms illustrating blank human urine standards spiked with ertapenem at the LLOQ, mid, and high concentrations are presented in Figure 4 to Figure 6, respectively.

11.5. Precision and Accuracy

The precision of an assay is defined as the closeness in which replicate analyses agree and is reported as percent coefficient of variation (%CV). The accuracy of a method is defined as the closeness of a determined value to its true (nominal) value and is reported as %Bias. Inter-assay (between runs) precision & accuracy and intra-assay (within runs) precision & accuracy were assessed in three runs by analyzing six (6) replicates each of QCs fortified at four separate concentrations (LLOQ, low, mid, and high). For inter- and intra-assay, the mean %Bias at each QC level was required to be within ±15.0% from its respective nominal concentration, except at the LLOQ, where ± 20.0% was accepted. The %CV at each QC level was required to be ≤ 15.0%, except at the LLOQ, where ≤ 20.0% was accepted.

The intra-assay and inter-assay precision and accuracy data for this method met the required criteria and are presented in Table 15 (intra-assay precision and accuracy) and Table 16 (inter-assay precision and accuracy).

Run 3 LLOQ, low, and mid QC's failed criteria, therefore, the acceptance criteria were not met. Failed run is reported in Table 17.

Standards and QCs of non-P&A runs are reported in Table 18.

11.6. Sensitivity

The sensitivity of the method was determined in three runs by analyzing six (6) replicates of QCs at the lower limit of quantitation (LLOQ) which were equal in concentration to the lowest calibration standard. The observed response at the LLOQ was required to be at least 5 times the response of noise in the human urine blank, in addition to the precision and accuracy requirements outlined in the previous section.

The LLOQ for this method was determined to be 10.0 mcg/mL, and the response at the LLOQ was at least 5 times the response of noise in the human matrix blank. Data are presented in Table 15 (intra-assay precision and accuracy) and Table 16 (inter-assay precision and accuracy).

11.7. Run Size

In order to evaluate the largest size run that can be used for study sample analysis, a run was processed with a set of 8 calibration standards and a minimum of 3 QC samples, processed in

duplicate. In addition, at least 3 matrix blanks (one with internal standard and two without internal standard) were processed with the run. Run 6 was used for this assessment, and additional experiments were also analyzed which made the final run size 122.

In order for the run to be acceptable, at least one QC at each concentration level and two-thirds of the total number of QC samples were required to interpolate within ± 15.0% from nominal.

The standard and QC summary of run 6 is presented in Table 14 and Table 15 and acceptance criteria was met.

11.8. Precision and Accuracy of Dilution

To determine the accuracy of dilution, the high concentration QC spiked with ertapenem (3000 mcg/mL) as well as an above-range QC (ARQC; 16000 mcg/mL) were evaluated. All samples were extracted in six replicates according to the test procedure. To be deemed acceptable, the mean %Bias at each level must be within ± 15.0% with %CV ≤ 15.0%. The replicates were prepared as follows:

- Dilution factor 2x (high QC) = 25 mcL samples + 25 mcL blank treated human urine
- Dilution factor 5x (ARQC) = 10 mcL samples + 40 mcL blank treated human urine

Dilution precision and accuracy, using dilution factors of 2x and 5x, were proven to be valid. Data are presented in Table 19.

11.9. Analyte Adsorption to Container Test

Adsorption of ertapenem in treated and untreated human urine when stored in polyethylene, polypropylene, or HDPE containers was evaluated to ensure that no significant amounts of ertapenem are lost during sample collection. A low concentration QC sample in untreated human urine was aliquoted into each container type tested and stored in each container type on wet ice prior to being transferred to the next container of the same type. A total of four times of transferring in 40 minutes was tested. After each 10-minute interval, the sample were vortexed and pipetted into a polypropylene tube for analysis and the appropriate amount of MES buffer was added. The rest is transferred into a clean container of the same type. Upon completion, the samples were extracted. The %Change is calculated with each timepoint comparing to the 0-hour data (same lot of QC aliquoted into a micro-centrifuge tube at the beginning of the test). The %Change between 0-hour and each transfer be within ± 15.0% to be considered not significant. There appears to be no significant adsorption effect in any type of container tested. Data are provided in Table 20.

11.10. Freeze/Thaw Stability

Ertapenem stability in treated human urine with and without the presence of zidebactam during sequential freeze/thaw cycles (frozen separately at -20 °C and -70 °C and thawed in wet ice) was evaluated. Aliquots of high and low concentration QC samples (with and without the presence

of zidebactam) were frozen for at least 12 hours before the initial thawing. They were re-frozen 2 more times for at least 12 hours each time and were thawed prior to assay along with a freshly prepared set of QCs and calibration standards. To be deemed stable, the mean %Bias at each level must be within ± 15.0% with %CV ≤ 15.0%.

Ertapenem in treated human urine (with and without the presence of zidebactam) were proven stable over 3 cycles of freeze (-20 °C and -70 °C) and thaw (wet ice). Data are provided in Table 21.

11.11. Bench-Top Stability in Wet Ice

To evaluate ertapenem in treated human urine with and without the presence of zidebactam stability during sample processing in wet ice, aliquots of the high and low concentration QC pools (with and without the presence of zidebactam) were allowed to thaw in wet ice for 6 hours and 24 hours, prior to analysis along with a freshly prepared set of QCs and calibration standards. To be deemed stable, the mean %Bias at each level must be within ± 15.0% with %CV ≤ 15.0%.

Ertapenem in treated human urine was proven stable in an ice-water bath under normal laboratory lighting over a period of 24 hours. Data are provided in Table 22.

11.12. Refrigeration Stability (Treated and Untreated)

To evaluate ertapenem in treated and untreated human urine stability during sample processing at refrigerated temperatures, aliquots of the high and low concentration QC pools (treated and untreated) were allowed to thaw at refrigerated temperatures for 19 hours, prior to analysis along with a freshly prepared set of QCs and calibration standards. To be deemed stable, the mean %Bias at each level must be within ± 15.0% with %CV ≤ 15.0%.

Run 5 treated refrigerated stability failed at the low concentration and was repeated in run 8. Ertapenem in treated and untreated human urine was proven stable at refrigerated temperatures over a period of 19 hours. Data are provided in Table 23.

11.13. Long-Term Matrix Stability at -20 °C and -70 °C

To evaluate ertapenem in treated human urine with and without the presence of zidebactam during sample storage, aliquots of the high and low concentration QC pools were stored frozen at -20 °C and -70 °C nominally for 29 days (low QC without zidebactam), 34 days (high QC without zidebactam and high and low QCs with zidebactam), and 57 days (high QC without the presence of zidebactam only) prior to being analyzed, along with freshly prepared standards and QC samples. To be deemed stable, the mean %Bias at each level must be within ± 15.0% with %CV ≤ 15.0%.

Ertapenem in treated human urine without the presence of zidebactam was proven to be stable at -70 °C over a period of 57 days (high QCs run 13) and 29 days (low QCs, run 11) in

polypropylene tubes. In run 11, the high QC level without zidebactam shows responses equal to the low QC level for 4 out of the 6 aliquots due to a pipetting error in the run extraction. The two aliquots pipetted correctly are within ±15% bias. The high QC extraction was repeated in run 13 as confirmation for the high QC only. Reported stability is 29 days. Ertapenem in treated human urine with the presence of zidebactam was proven to be stable at -70 °C over a period of 34 days in polypropylene tubes.

Ertapenem in treated human urine with and without the presence of zidebactam was not demonstrated to be stable at -20 °C over a period of 29 days and 34 days due to %Bias outside of - 15.0%. Unknown samples should be stored at -70°C. Data is provided in Table 24.

Re-extraction of the LTS high QC was attempted in run 12, but the Cals and QCs were prepared 10x lower due to a calculation error and the run was rejected. Data is presented in Table 17.

11.14. Processed Sample Stability when Refrigerated

To evaluate ertapenem stability in processed samples at refrigerated temperatures, high and low concentration QC pools were processed, placed into polypropylene 96 well plates, and stored at refrigerated temperatures before being chromatographed with samples in a subsequent run. To be deemed stable, the mean %Bias at each level must be within ± 15.0% with %CV ≤ 15.0%.

Processed ertapenem samples were proven stable when refrigerated stored in polypropylene 96 well plates over a period of 5 days 21 hours. Data are provided in Table 25.

11.15. Individual Sample Reinjection Reproducibility

To evaluate individual sample reinjection reproducibility, low and high QC extracts (6 each) injected during the run were reinjected at the end of the run, within the established stability time window of processed samples in the refrigerator. To be deemed reproducible, at least four out of six QCs at each level must interpolate with dev[%] within ± 15.0% from nominal, and the mean %Bias at each level must be within ± 15.0% with %CV ≤ 15.0%.

Run 3 individual sample reinjection reproducibility could not be evaluated due to analytical QC failure and was repeated in run 8. Reinjection of individual samples can be made with adequate precision and accuracy. Data are provided in Table 26.

11.16. Whole Run Reinjection Reproducibility

To evaluate whole run reinjection reproducibility, run 6 was reinjected as run 9 on the same MS platform using a different chromatographic column from the same manufacturer, with the same specification as the original within 5 days 9 hours following the original injection. To be deemed acceptable, the reinjected run was required to meet the acceptance criteria outlined in Sections 11.4, 11.5, and 11.6.

It has been demonstrated that entire runs can be reinjected with adequate accuracy and precision. Data are presented in Table 27.

11.17. Column Ruggedness

Column ruggedness was assessed by reinjecting run 6 as run 9 on the same MS platform (Applied Biosystems API5000) using a different chromatographic column from the same manufacturer, with the same specification as the original.

Data were required to meet the precision and accuracy criteria outlined in Sections 11.4, 11.5, and 11.6 to be considered as reproducible/rugged method. Data are presented in Table 27. It has been demonstrated that ruggedness has been met.

11.18. Extraction Recovery

To evaluate the extraction recovery, the low, mid, and high extracted QC samples were compared to extracted human urine blanks to which an equal amount of ertapenem at each level, assuming 100% recovery, was added in six replicates.

To evaluate the extraction recovery of ertapenem-d_4 (IS), ertapenem was added only for tracking during analysis. Ertapenem-d_4 (IS) for the control was added after extraction, while the same amount of ertapenem-d_4 (IS) for the extracted QC was added before extraction.

$$\text{\% Recovery} = \frac{\text{Response ratio of "Extracted QC"}}{\text{Average Response Ratio of Control}} \times 100$$

The mean recovery of ertapenem was determined to be consistent, precise and reproducible at 94.8%. It was found that recovery solutions were calculated based on reported concentrations in plasma instead of actual concentrations in treated matrix (which are half of the reported concentrations). A correction factor of 0.5 is applied to the areas of the post extraction spike samples. In addition, there was a prep error in the mid and low QC preps, resulting in concentrations 25% lower than the nominal concentration. A 1.25 correction factor is applied to the extracted low and mid QC.

The recovery of ertapenem-d_4 (IS) was determined to be consistent, precise and reproducible at 105.2%. Data are presented in Table 28 to Table 29.

11.19. Solution Stabilities

The stability of various solutions of ertapenem in various solvents used during the sample analysis following storage at various temperatures was determined. Ertapenem-d_4 (IS) is a deuterated analog, so its stability at room temperature is taken as equivalent to ertapenem.

Data showing the ertapenem(s), solvent composition, storage term, and temperature are presented in Table 30 and Table 31.

Solution stabilities previously conducted in other studies.

11.20. Calculations

The peak areas of ertapenem and ertapenem-d_4 were integrated by Analyst 1.7.2 (Applied Biosystems; Foster City, CA, USA). The data results were imported electronically into Watson LIMS 7.5 for the calculation of ertapenem concentrations and statistical analysis. The calibration curves were obtained by a weighted (1/concentration^2) least squares linear regression. After the best fit from data, the concentrations (mcg/mL) were calculated based on the measured peak area ratios of ertapenem/ertapenem-d_4 using the following equation:

$$\text{Response} = \text{Slope} * \text{Conc} + \text{Intercept}$$

$$\text{Mean Value } (\overline{X}) = \frac{\sum_{i=1}^{n} \text{individual data (X)}}{\text{number of data points (n)}}$$

$$\text{Standard Deviation (SD)} = \sqrt{\frac{\sum_{i=1}^{n}\left[\text{individual data (X)-Mean Value } (\overline{X})\right]^2}{\text{n-1}}}$$

$$\text{Coefficient of Variation } (\%\text{CV}) = \left[\frac{\text{Standard Deviation (SD)}}{\text{Mean Value } (\overline{X})}\right] \times 100$$

$$\%\text{Bias} = \left(\frac{\text{Calculated Mean of the Observed Values or Observed Value-Nominal Value}}{\text{Nominal Value}}\right) \times 100$$

All calibration standards and QCs concentrations were reported to three significant figures, and study samples will be reported to three significant figures.

11.21. SOP Deviations and Investigations

One SOP deviation occurred during this study. No investigations were conducted during this study.

Table 2: Deviations

Deviation #	Description	Impact
DEV-2022-076	For run 7 the data was inadvertently imported into Watson prior to chromatogram approval. No changes were made to the chromatograms after import and data was only imported once.	There is no impact.

12. CONCLUSION

Validation results obtained in this study indicate that a robust and reliable method has been established for the measurement of ertapenem in treated human urine by LC-MS/MS in the concentration range of 10.0 - 4000 mcg/mL. The generally recommended acceptance criteria, FDA Guidance for Industry on Bioanalytical Methods Validation, May 2018, in terms of precision, accuracy, selectivity including matrix effect, recovery and all types of stability have been met. Thus, this validated method is deemed suitable for determination of ertapenem in human urine for unknown samples.

13. PERTINENT SOPS

The following SOPs were followed during the conduct of this study:

SOP-00073 [02-03] - Bioanalytical Method Validation - Chromatographic Assays

SOP-00035 [00-02] - Procedures, Reporting, and Acceptance Criteria for Bioanalytical Chromatographic Projects

14. ARCHIVES

All raw data, including paper and electronic files associated with this study will remain archived at a location specified by KCAS, 10830 S Clay Blair Boulevard, Olathe, Kansas 66061 for a period of at least 10 years, after which the client may be contacted to arrange for continued storage of the files, or disposition according to an agreed procedure. KCAS will maintain an electronic (scanned/true) copy of the method validation report that can be viewed and printed on site. The study plan, documentation, raw data, and final report are indexed under Bioanalytical Project Number V1872106U3.

15. REFERENCES

1. Guidance for Industry: Bioanalytical Method Validation. U.S. Department of Health and Human Services. Food and Drug Administration. Center for Drug Evaluation and Research. Center for Veterinary Medicine. May 2018.

16. TABLES

Table 3: Mass Spec Transitions

Analyte	Transition
Ertapenem	m/z 476.047→431.9
Ertapenem-d_4 (IS)	m/z 480.2→436.3

Table 4: Reference Standards

Analyte	Supplier	Lot/Batch Number	Purity	Expiration Date or Retest Date	Conversion Factor
Ertapenem Sodium	MedChemExpress	180017	98.9%	Retest 11 Aug 2024	1.046
Ertapenem-d_4 Sodium Salt (IS)	AlsaChim	DH-ALS-20-002-P2	*	Expire 29 Apr 2023	*
Zidebactam	Wockhardt	WK1/WP-0060/016	90.4%	Retest 29 Mar 2023	1.000

Certificates of analysis for the above reference standards are presented in Annex 2.
*Used as is with 1.000 conversion and 100% purity as IS only.

Table 5: Calibration Standards and QC Samples Preparation

Critical Solutions	Lot # for Standards (Prep Date)	Lot # for QCs (Prep Date)	Standards Lot # (Prep Date)	QCS Lot # (Prep Date)	Runs used
Treated Human Urine V1872106U3-5-1			V1872106U3-5-2 Set A (17Nov2022)	V1872106U3-6-1 Set A (17Nov2022)	3 QCs only: 2, 4, 11 (LTS), 13 (LTS)
MES buffer, pH 6.4, ~0.1M, frozen (-20°C) in polypropylene	V1872106U3-1-1 (15Nov2022)	V1872106U3-2-1 (15Nov2022)			
Treated Human Urine V1872106U3-5-1			NA	V1872106U3-7-1 Set A (17Nov2022)	Comed QCs: 3, 10, 11 (LTS)
Deionized water frozen (-20°C) in glass	NA	V1872106U2-31-1 (20Sep2022)			
MES buffer, pH 6.4, ~0.1M, frozen (-20°C) in polypropylene	NA	V1872106U3-2-1 (15Nov2022)			
Treated 1:1 Urine:MES buffer, pH 6.4, ~0.1M, frozen (-70°C) in polypropylene	NA	V1872106U3-18-1 (17Nov2022)			
Treated Human Urine V1872106U3-5-1			V1872106U3-22-1 Set B (21Nov2022)	V1872106U3-23-1 Set B (21Nov2022)	5
MES buffer, pH 6.4, ~0.1M, frozen (-20°C) in polypropylene	V1872106U3-1-1 (15Nov2022)	V1872106U3-1-1 (15Nov2022)			
Treated Human Urine V1872106U3-31-1			V1872106U3-33-1 Set C (22Nov2022)	V1872106U3-32-1 Set C (22Nov2022)	6, QCs only: 11 (LTS)
MES buffer, pH 6.4, ~0.1M, frozen (-20°C) in polypropylene	V1872106U3-2-1 (15Nov2022)	V1872106U3-2-1 (15Nov2022)			
Treated Human Urine V1872106U3-31-1			V1872106U3-38-1 Set D (23Nov2022)	V1872106U3-39-1 Set D (23Nov2022)	7
MES buffer, pH 6.4, ~0.1M, frozen (-20°C) in polypropylene	V1872106U3-25-1 (22Nov2022)	V1872106U3-25-1 (22Nov2022)			
Treated Human Urine V1872106U3-31-1			V1872106U3-40-1 Set E (28Nov2022)	V1872106U3-41-1 Set E (28Nov2022)	8, 10
MES buffer, pH 6.4, ~0.1M, frozen (-20°C) in polypropylene	V1872106U3-24-1 (22Nov2022)	V1872106U3-24-1 (22Nov2022)			
Treated Human Urine V1872106U3-31-1			V1872106U3-49-1 Set F (21Dec2022)	V1872106U3-50-1 Set F (21Dec2022)	11
MES buffer, pH 6.4, ~0.1M, frozen (-20°C) in polypropylene	V1872106U3-42-1 (20Dec2022)	V1872106U3-43-1 (20Dec2022)			
Treated Human Urine V1872106U3-31-1			V1872106U3-53-1 Set H (13Jan2022)	V1872106U3-54-1 Set H (13Jan2022)	13
MES buffer, pH 6.4, ~0.1M, frozen (-20°C) in polypropylene	V1872106P5-84-1 (12Jan2022)	V1872106P5-85-1 (12Jan2022)			

Table 6: Matrix Description

Matrix	Source Lot #	Date Received
Treated Human Urine V1872106U3-5-1	BioChemed BC41992HU-9	Aug 2022
Treated Human Urine V1872106U3-31-1	BioChemed BC41992HU-10	Aug 2022

Table 7: Summary of Results and Conclusions

Methodology – TP80182	Method Performance – LC-MS/MS	Hyperlink – N/A	Comments
Matrix	Urine		
Anticoagulant	N/A		
Assay Volume	50 mcL		
**Selectivity**			
Analyte	Ertapenem	Summary tables<br>Table 10<br><br>Report text<br>Section 11.3.1	
Internal Standard	Ertapenem-d4	Summary tables<br>Table 10<br><br>Report text<br>Section 11.3.1	
Matrix Effect<br>(6 spiked matrix lots)	Complies	Summary tables<br>Table 11 and Table 12<br><br>Report text<br>Section 11.3.2	
Comed Interference	Complies	Summary tables<br>Table 13<br><br>Report text<br>Section 11.3.3	
Carryover	Assessed with every run with standards and QCs	Summary tables<br>Table 9<br><br>Report text<br>Section 11.3.4	
**Linearity**			
Weighting	$1/x^2$	Report text<br>Section 11.4	
Bias at LLOQ	-1.5%	Summary tables<br>Table 14<br><br>Report text<br>Section 11.4	
Bias above LLOQ	-3.8 to 4.3%	Summary tables<br>Table 14<br><br>Report text<br>Section 11.4	

Table 7: Summary of Results and Conclusions (Continued)

**Methodology – TP80182**	**Method Performance – LC-MS/MS**	**Hyperlink – N/A**	**Comments**
Bioanalytical Range	Complies<br>10.0 to 4000 mcg/mL	Summary tables<br>Table 14<br><br>Report text<br>Section 11.4	
**Precision**			
Intra-assay	3.6 to 7.7% (LLOQ);<br>1.2 to 5.1% (above LLOQ)	Summary tables<br>Table 15<br><br>Report text<br>Section 11.5	
Inter-assay	6.9% (LLOQ);<br>2.2 to 3.5% (above LLOQ)	Summary tables<br>Table 16<br><br>Report text<br>Section 11.5	
**Accuracy**			
Intra-assay	-4.5 to 5.0% (LLOQ);<br>-2.7 to 4.7% (above LLOQ)	Summary tables<br>Table 15<br><br>Report text<br>Section 11.5	
Inter-assay	-0.4% (LLOQ);<br>0.3 to 4.0% (above LLOQ)	Summary tables<br>Table 16<br><br>Report text<br>Section 11.5	
**Large Run Size**	Determined to be 122 samples.	Summary tables<br>Table 14 and Table 15<br><br>Report text<br>Section 11.7	
**Dilution**			
Dilution factor: 2x	Dilution QC: 3000 mcg/mL<br>Precision: 1.3%<br>Accuracy: -2.7%	Summary tables<br>Table 19<br><br>Report text<br>Section 11.8	
Dilution factor: 5x (run 5)	Dilution QC: 16000 mcg/mL<br>Precision: 27.5%<br>Accuracy: -18.1%	Summary tables<br>Table 19<br><br>Report text<br>Section 11.8	
Dilution factor: 5x (run 8)	Dilution QC: 16000 mcg/mL<br>Precision: 3.3%<br>Accuracy: 3.8%	Summary tables<br>Table 19<br><br>Report text<br>Section 11.8	

Table 7: Summary of Results and Conclusions (Continued)

Methodology – TP80182	Method Performance – LC-MS/MS	Hyperlink – N/A	Comments
**Adsorption**	No significant lost after 3 transfers in either polypropylene or high density polyethylene	Summary tables Table 20<br><br>Report text Section 11.9	
**Stability**			
Matrix (in polypropylene tubes)			
Freeze/Thaw (-20 °C/wet ice) with and without the presence of zidebactam	Demonstrated for 3 cycles	Summary tables Table 21<br><br>Report text Section 11.10	
Freeze/Thaw (-70 °C/wet ice) with and without the presence of zidebactam	Demonstrated for 3 cycles	Summary tables Table 21<br><br>Report text Section 11.10	
Bench-top stability in wet ice with and without the presence of zidebactam	Demonstrated for 24 hours	Summary tables Table 22<br><br>Report text Section 11.11	
Refrigeration with and without the presence of zidebactam	Demonstrated for 19 hours	Summary tables Table 23<br><br>Report text Section 11.12	
Long-term (-20 °C)	Demonstrated instability for 34 days low and high QCs with the presence of zidebactam<br><br>Demonstrated instability for 29 days (low QC) and 34 days (high QC) without the presence of zidebactam	Summary tables Table 24<br><br>Report text Section 11.13	
Long-term (-70 °C)	Demonstrated for 57 days (high QCs) without the presence of zidebactam (will be reported as 29 days)<br><br>Demonstrated for 34 days low and high QCs with the presence of zidebactam<br><br>Demonstrated for 29 days (low QCs) without the presence of zidebactam	Summary tables Table 24<br><br>Report text Section 11.13	
Processed Matrix polypropylene			
Refrigeration	Demonstrated for 5 days 21 hours	Summary tables Table 25<br><br>Report text Section 11.14	

Table 7: Summary of Results and Conclusions (Continued)

**Methodology – TP80182**	**Method Performance – LC-MS/MS**	**Hyperlink – N/A**	**Comments**
**Individual Sample Reinjection**	Reproducible	Summary tables<br>Table 26<br><br>Report text<br>Section 11.15	
**Whole Run Reinjection Integrity**	Reproducible<br>Duration 5 days 9 hours	Summary tables<br>Table 27<br><br>Report text<br>Section 11.16	
**Column Ruggedness**	Reproducible	Summary tables<br>Table 27<br><br>Report text<br>Section 11.17	
**Extraction Recovery**			
Ertapenem	Overall 94.8%	Summary tables<br>Table 28<br><br>Report text<br>Section 11.18	
Ertapenem-d_4 (IS)	Overall 105.2%	Summary tables<br>Table 29<br><br>Report text<br>Section 11.18	
**Solution Stability**			
Solution, Primary polypropylene			
Ertapenem in MES buffer (RT)	Stable 6.75 hours	Summary tables<br>Table 30	V1872106P5
Ertapenem in MES buffer (-20 °C)	Stable 7 days	Summary tables<br>Table 31	V1872106P5
Ertapenem-d_4 (IS) in MES buffer (RT)	Taken to be same as ertapenem	SOP 00073	
Ertapenem-d_4 (IS) in MES buffer (-20 °C)	1 year stability can be assigned and evaluated thereafter in yearly increments	SOP 00073	

Table 8: Summary of Validation Run Objectives and Outcome

Run ID	Instrument ID	Extraction Date	Assay Date	Run Objective(s)	Accepted or Rejected	Comment
2	LC/MS-22	17-Nov-2022	18-Nov-2022	Matrix effect and recovery	Accepted	[a]
3	LC/MS-39	17-Nov-2022	17-Nov-2022	Precision and accuracy, IS cross-talk, selectivity, individual sample reinjection reproducibility, interference QC qualification, and extraction of processed stability samples	Rejected	[b]
4	LC/MS-22	17-Nov-2022	18-Nov-2022	Adsorption test	Accepted	
5	LC/MS-22	21-Nov-2022	21-Nov-2022	Precision and accuracy, dilution integrity, and treated and untreated refrigerated stability	Accepted	[c]
6	LC/MS-22	22-Nov-2022	23-Nov-2022	Precision and accuracy, freeze/thaw stability without zidebactam (high QCs only), freeze/thaw stability with zidebactam (high and low QCs), ice-water bath stability without zidebactam (high QCs only), ice-water bath stability with zidebactam (high and low QCs), and large run size	Accepted	
7	LC/MS-22	23-Nov-2022	23-Nov-2022	Precision and accuracy, IS cross-talk, selectivity	Accepted	IS cross-talk showed 6.1% cross-talk to IS and was repeated in run 10.
8	LC/MS-22	28-Nov-2022	28-Nov-2022	Precision and accuracy, dilution integrity (5x only), treated refrigerated stability (low QC only), freeze/thaw stability (low QC only), ice-water bath stability (low QC only), and injection of refrigerated processed stability samples	Accepted	
9	LC/MS-22	22-Nov-2022	28-Nov-2022	Whole run reinjection reproducibility and column ruggedness	Accepted	
10	LC/MS-39	07-Dec-2022	07-Dec-2022	Comed interference and IS cross-talk	Accepted	[d]
11	LC/MS-39	21 Dec 2022	21 Dec 2022	Long-term matrix stability with and without zidebactam (high and low QCs), Comed QC Interference	Accepted	
12	LC/MS-39	10 Jan 2023	11 Jan 2023	Long-term matrix stability without zidebactam (high QCs)	Rejected	[e]

Table 8: Summary of Validation Run Objectives and Outcome (Continued)

Run ID	Instrument ID	Extraction Date	Assay Date	Run Objective(s)	Accepted or Rejected	Comment
13	LC/MS-39	13 Jan 2023	16 Jan 2023	Long-term matrix stability without zidebactam (high QCs)	Accepted	

Run 1 was used for a primary comparison and will not be reported.

[a]Recovery %CV was within 15%, however since it is known that the mid and low QCs were prepared incorrectly based on run 3 results, recovery was repeated for the analyte only.

[b]LLOQ, Low, and Mid QC's failed criteria. The interference QCs and individual sample reinjection reproducibility could not be evaluated in this run and were repeated. This appears to be due to a preparation error for the Mid QC which trickled down to the low and LLOQ QCs (made by serial dilution).

[c]Run failed precision and accuracy criteria but passes run acceptance criteria. Dilution integrity passed for 2x but failed for 5x. Treated refrigerated stability failed at the low concentration but passed at the high concentration.

[d] The high QC level meets criteria, however, the low QC level shows a >15% bias. This appears to be an extraction issue and not a prep issue since all acceptance criteria for comed freeze-thaw and benchtop stability are met for the low level in run 8. The low comed QC met all criteria in run 11 where both the low and high comed QC met all acceptance criteria for 34 days at -70°C storage. Since the interference QC assessment does not need to be done from a fresh preparation, the run 11 data is used to confirm that the presence of zidebactam does not affect the analysis of ertapenem.

[e] The fresh cals/QCs were prepared incorrectly due to a calculation error such that the nominal and actual concentrations differ by 10x. The run was rejected. This run was repeated in run 13.

Table 9: Carryover

Run ID	Result (Extracted Blank after High Standard)	Carryover Results for Internal Standard
5	Seq 13 and Seq 86 are <20.0% of the LLOQ.	Seq 4 and Seq 77 are < 5.0% internal standard peak of acceptable standards.
6	Seq 13 and Seq 122 are <20.0% of the LLOQ.	Seq 4 and Seq 113 are < 5.0% internal standard peak of acceptable standards.
7	Seq 13 and Seq 64 are <20.0% of the LLOQ.	Seq 4 and Seq 55 are < 5.0% internal standard peak of acceptable standards.
8	Seq 13 and Seq 86 are <20.0% of the LLOQ.	Seq 4 and Seq 77 are < 5.0% internal standard peak of acceptable standards.
9	Seq 13 and Seq 50 are <20.0% of the LLOQ.	Seq 4 and Seq 41 are < 5.0% internal standard peak of acceptable standards.
10	Seq 13 was 50.3% of the LLOQ and Seq 58 was 37.5% of the LLOQ	Seq 4 and Seq 49 are < 5.0% internal standard peak of acceptable standards.
11	Seq 13 was 50.6% of the LLOQ and Seq 98 was 59.8% of the LLOQ	Seq 4 and Seq 98 are < 5.0% internal standard peak of acceptable standards.
13	Seq 13 and Seq 50 are <20.0% of the LLOQ.	Seq 4 and Seq 41 are < 5.0% internal standard peak of acceptable standards.

Note: Calibration Standards and QCs are arranged in a way to mitigate carryover in all validation runs.

Table 10: Selectivity - Blank Assessment

Run 7 Selectivity Blank Screen

Blank ID with or without IS	Blank Lot Number	Peak Area of Analyte	Peak Area of Internal Standard	Percent of LLOQ Peak Area	Percent of LLOQ Peak Area Ratio	Percent of IS in Blanks without IS
Blank 1 NO IS	V1872106U3-21-1	0	0	0.0	NA	0.0
Blank 2 NO IS	V1872106U3-21-2	0	0	0.0	NA	0.0
Blank 3 NO IS	V1872106U3-21-3	0	0	0.0	NA	0.0
Blank 4 NO IS	V1872106U3-21-4	0	0	0.0	NA	0.0
Blank 5 NO IS	V1872106U3-21-5	0	0	0.0	NA	0.0
Blank 6 NO IS	V1872106U3-21-6	0	0	0.0	NA	0.0
Blank 1	V1872106U3-21-1	0	195602	0.0	0.0	NA
Blank 2	V1872106U3-21-2	0	197109	0.0	0.0	NA
Blank 3	V1872106U3-21-3	0	196357	0.0	0.0	NA
Blank 4	V1872106U3-21-4	0	184955	0.0	0.0	NA
Blank 5	V1872106U3-21-5	0	194435	0.0	0.0	NA
Blank 6	V1872106U3-21-6	0	198686	0.0	0.0	NA

Note: Average IS response are from all of the accepted standards and QCs

Criteria: A minimum of six different sources of treated human urine were screened. To be deemed acceptable, the peak response observed must be ≤ 20.0% of the mean response of the lower limit of quantitation (LLOQ) and ≤ 5.0% of the mean response of the internal standard. If any lots fail, further evaluation is needed.

Results: All matrix lots were found to be free of any significant interference at the retention times of the analytes of interest.

Table 11: Matrix Effect – Low Concentration

Run Number:	2								
Concentration:	30.0 mcg/mL								
**Sample ID**	**Drug in neat/matrix Area**	**Average Drug in Neat**	**Individual MF analyte**	**Average MF analyte**	**IS in neat/ matrix Area**	**Average IS in neat Area**	**Individual IS MF**	**Average IS MF**	**Average IS Normalized MF**
Neat	17954	18578			605484	615076			
	18553				614857				
	19156				610531				
	18650				629430				
ME1	19288		1.0382	1.0143	627448		1.0201	1.0084	1.0058
	18363		0.9884		609659		0.9912		
	18524		0.9971		626532		1.0186		
	19198		1.0334		617452		1.0039		
ME2	19553		1.0525	1.0148	629959		1.0242	1.0103	1.0045
	18692		1.0061		617387		1.0038		
	18138		0.9763		614154		0.9985		
	19031		1.0244		624039		1.0146		
ME3	18741		1.0088	1.0210	595210		0.9677	0.9963	1.0247
	17624		0.9486		598699		0.9734		
	19775		1.0644		622941		1.0128		
	19732		1.0621		634467		1.0315		
ME4	19380		1.0432	1.0296	620658		1.0091	1.0083	1.0211
	18036		0.9708		603432		0.9811		
	19091		1.0276		636447		1.0347		
	20007		1.0769		620186		1.0083		
ME5	19141		1.0303	1.0383	620681		1.0091	1.0162	1.0217
	19086		1.0273		624008		1.0145		
	19591		1.0545		629937		1.0242		
	19341		1.0411		625623		1.0171		
ME6	19334		1.0407	1.0456	623240		1.0133	1.0156	1.0295
	19173		1.0320		618565		1.0057		
	19105		1.0284		623977		1.0145		
	20087		1.0812		632919		1.0290		
**Mean**									1.0179
**N**									6
**SD**									0.0103
**%CV**									1.0

neat = in absence of matrix, matrix = in presence of extracted matrix
Criteria: %CV of Average IS Normalized MF among 6 lots of matrix must be ≤ 15.0%.
Meets Acceptance Criteria: YES MF=matrix factor

$$\mathrm{MF} = \frac{\text{Peak Response in Presence of Matrix Substances}}{\text{Mean Peak Response in Absence of Matrix}}$$

$$\text{IS Normalized MF} = \frac{\mathrm{MF}_{Analyte}}{\mathrm{MF}_{IS}}$$

Table 12: Matrix Effect – High Concentration

Run Number:	2								
Concentration:	3000 mcg/mL								
**Sample ID**	**Drug in neat/matrix Area**	**Average Drug in Neat**	**Individual MF analyte**	**Average MF analyte**	**IS in neat/ matrix Area**	**Average IS in neat Area**	**Individual IS MF**	**Average IS MF**	**Average IS Normalized MF**
Neat	1983251	1978326			628748	627599			
	2040838				638899				
	1966469				627202				
	1922745				615548				
ME1	1998221		1.0101	0.9970	632942		1.0085	1.0086	0.9885
	1934033		0.9776		639324		1.0187		
	1957718		0.9896		631653		1.0065		
	1999480		1.0107		628070		1.0008		
ME2	1964224		0.9929	0.9996	621359		0.9901	0.9928	1.0068
	2001910		1.0119		631916		1.0069		
	2012014		1.0170		634319		1.0107		
	1931834		0.9765		604746		0.9636		
ME3	1896106		0.9584	0.9862	603158		0.9611	0.9892	0.9970
	1966823		0.9942		633563		1.0095		
	2026338		1.0243		633313		1.0091		
	1914941		0.9680		613149		0.9770		
ME4	1963145		0.9923	1.0089	624708		0.9954	1.0029	1.0059
	2024364		1.0233		641260		1.0218		
	2009963		1.0160		634874		1.0116		
	1986123		1.0039		616912		0.9830		
ME5	1965821		0.9937	0.9904	631351		1.0060	0.9878	1.0027
	1971597		0.9966		618044		0.9848		
	1956452		0.9889		615441		0.9806		
	1943611		0.9825		614829		0.9797		
ME6	1982114		1.0019	1.0055	623332		0.9932	0.9956	1.0099
	1998912		1.0104		631587		1.0064		
	1972860		0.9972		616596		0.9825		
	2002826		1.0124		627878		1.0004		
**Mean**									1.0018
**N**									6
**SD**									0.00786
**%CV**									0.8

neat = in absence of matrix, matrix = in presence of extracted matrix
Criteria: %CV of Average IS Normalized MF among 6 lots of matrix must be ≤ 15.0%.
Meets Acceptance Criteria: YES MF=matrix factor

$$MF = \frac{\text{Peak Response in Presence of Matrix Substances}}{\text{Mean Peak Response in Absence of Matrix}}$$

$$\text{IS Normalized MF} = \frac{MF_{Analyte}}{MF_{IS}}$$

Table 13: Comed Interference Evaluation

Run Date	Curve Number	Comed1 low 30.0 µg/mL	%Bias	Comed1 high 3000 µg/mL	%Bias
07-Dec-2022	10	#37.0	23.3	3120	4.0
		#36.4	21.3	3100	3.3
		#35.7	19.0	3230	7.7
		#36.0	20.0	3140	4.7
		#36.5	21.7	3230	7.7
		#37.5	25.0	3170	5.7
Mean		36.5		3170	
S.D.		0.655		55.4	
%CV		1.8		1.7	
%Bias		21.7		5.7	
n		6		6	
# > 15%Bias					

With presence of zidebactam

Run Date	Curve Number	Comed1 LTS -70 low 30.0 µg/mL	%Bias	Comed1 LTS -70 high 3000 µg/mL	%Bias
21-Dec-2022	11	32.9	9.7	2940	-2.0
		33.8	12.7	2780	-7.3
		32.6	8.7	2870	-4.3
		32.0	6.7	2980	-0.7
		##35.7	19.0	2910	-3.0
		33.1	10.3	2760	-8.0
Intrarun Mean		33.4		2870	
Intrarun SD		1.29		88.0	
Intrarun %CV		3.9		3.1	
Intrarun %Bias		11.3		-4.3	
n		6		6	
## > 15%Bias					

Criteria: To be considered acceptable, the mean %Bias at each level was also required to be within ± 15.0% and the %CV was required to be ≤ 15.0%.

Results: Run 3 interference results cannot be reported, because the run failed QC acceptance. However, the interference QCs compared to the passing calibration curve. Evaluation was repeated in run 10. The high QC level meets criteria; however, the low QC level shows a >15% bias. This appears to be an extraction issue and not a prep issue since all the criteria for the comed pool in the freeze/thaw and long-term stability assessment met criteria. The low comed QC met all criteria in run 11 where both the low and high comed QC met all acceptance criteria for 34 days at -70°C storage. Since the interference QC assessment does not need to be done from a fresh preparation, the run 11 data is used to confirm that the presence of zidebactam does not affect the analysis of ertapenem.

Table 14: Summary of Standard Curves

Analytical Performance: Back-Calculated Concentrations (µg/mL) of Ertapenem Calibration Standard in (Human) (Urine) in (Study V1872106U3)

Assay Date	Analytical Run Number	Cal 10 10.0 µg/mL	Cal 20 20.0 µg/mL	Cal 160 160 µg/mL	Cal 400 400 µg/mL	Cal 800 800 µg/mL	Cal 1600 1600 µg/mL	Cal 3200 3200 µg/mL	Cal 4000 4000 µg/mL
23-Nov-2022	6	9.96	20.4	164	422	862	1630	3050	3810
		9.61	21.2	157	412	801	1510	3060	3850
23-Nov-2022	7	9.46	20.8	167	416	814	1580	3130	3880
		10.3	19.9	170	416	816	1470	3020	3870
28-Nov-2022	8	9.84	20.0	159	407	810	1550	3170	3890
		9.90	20.8	168	427	817	1560	3090	3790
Mean		9.85	20.5	164	417	820	1550	3090	3850
S.D.		0.292	0.508	5.19	7.09	21.4	55.5	55.4	40.2
%CV		3.0	2.5	3.2	1.7	2.6	3.6	1.8	1.0
%Bias		-1.5	2.5	2.5	4.3	2.5	-3.1	-3.4	-3.8
n		6	6	6	6	6	6	6	6

Calibration Curve Parameters for Ertapenem Calibration Standards in Human Urine (Study V1872106U3)

Run Date	Curve Number	Slope	Intercept	R-Squared	LLOQ	ULOQ
23-Nov-2022	6	0.00286	0.00262	0.9976	10.0	4000
23-Nov-2022	7	0.00323	0.00308	0.9977	10.0	4000
28-Nov-2022	8	0.00285	0.00045	0.9986	10.0	4000

See Table 8 for runs not used for precision/accuracy evaluation.

Table 15: Precision and Accuracy (Intra-Assay)

Human Urine Ertapenem Concentrations in Quality Controls for 3 separate batch runs (Study V1872106U3)

Run Date	Curve Number	QCS 10 10.0 µg/mL	%Bias	QCS 30 30.0 µg/mL	%Bias	QCS 2000 2000 µg/mL	%Bias	QCS 3000 3000 µg/mL	%Bias
23-Nov-2022	6	10.3	3.0	30.5	1.7	2050	2.5	3000	0.0
		9.13	-8.7	31.1	3.7	2090	4.5	3140	4.7
		9.24	-7.6	30.4	1.3	2090	4.5	2980	-0.7
		10.4	4.0	31.2	4.0	1980	-1.0	3020	0.7
		9.03	-9.7	32.0	6.7	1970	-1.5	3160	5.3
		9.22	-7.8	32.2	7.3	2110	5.5	3060	2.0
Intrarun Mean		9.55		31.2		2050		3060	
Intrarun SD		0.622		0.745		60.1		74.8	
Intrarun %CV		6.5		2.4		2.9		2.4	
Intrarun %Bias		-4.5		4.0		2.5		2.0	
n		6		6		6		6	
23-Nov-2022	7	10.2	2.0	29.7	-1.0	1990	-0.5	2870	-4.3
		9.21	-7.9	29.6	-1.3	1990	-0.5	2850	-5.0
		9.92	-0.8	31.0	3.3	2000	0.0	2920	-2.7
		11.1	11.0	33.2	10.7	2030	1.5	2890	-3.7
		8.98	-10.2	33.1	10.3	2090	4.5	2980	-0.7
		9.81	-1.9	31.9	6.3	1980	-1.0	3000	0.0
Intrarun Mean		9.87		31.4		2010		2920	
Intrarun SD		0.756		1.59		41.3		60.5	
Intrarun %CV		7.7		5.1		2.1		2.1	
Intrarun %Bias		-1.3		4.7		0.5		-2.7	
n		6		6		6		6	
28-Nov-2022	8	10.4	4.0	32.1	7.0	2030	1.5	3090	3.0
		10.5	5.0	30.3	1.0	2030	1.5	3040	1.3
		10.2	2.0	30.1	0.3	2080	4.0	3090	3.0
		10.0	0.0	30.1	0.3	2030	1.5	2960	-1.3
		10.5	5.0	31.3	4.3	2020	1.0	3020	0.7
		11.1	11.0	31.5	5.0	2070	3.5	3030	1.0
Intrarun Mean		10.5		30.9		2040		3040	
Intrarun SD		0.373		0.849		25.0		48.8	
Intrarun %CV		3.6		2.7		1.2		1.6	
Intrarun %Bias		5.0		3.0		2.0		1.3	
n		6		6		6		6	

Criteria: At each level, the %CV was required to be ≤ 15.0%, except at the LLOQ level, where ≤ 20.0% was accepted. The mean %Bias at each level was also required to be within ± 15.0%, except at the LLOQ, where ± 20.0% was accepted.

Results: The acceptance criteria were met. Run size (n=122) was completed in run 6 and met acceptance criteria.

Table 16: Precision and Accuracy (Inter-Assay)

Human Urine Ertapenem Concentrations in Quality Controls for 3 separate batch runs (Study V1872106U3)

Run Date	Curve Number	QCS 10 10.0 µg/mL	%Bias	QCS 30 30.0 µg/mL	%Bias	QCS 2000 2000 µg/mL	%Bias	QCS 3000 3000 µg/mL	%Bias
23-Nov-2022	6	10.3	3.0	30.5	1.7	2050	2.5	3000	0.0
		9.13	-8.7	31.1	3.7	2090	4.5	3140	4.7
		9.24	-7.6	30.4	1.3	2090	4.5	2980	-0.7
		10.4	4.0	31.2	4.0	1980	-1.0	3020	0.7
		9.03	-9.7	32.0	6.7	1970	-1.5	3160	5.3
		9.22	-7.8	32.2	7.3	2110	5.5	3060	2.0
23-Nov-2022	7	10.2	2.0	29.7	-1.0	1990	-0.5	2870	-4.3
		9.21	-7.9	29.6	-1.3	1990	-0.5	2850	-5.0
		9.92	-0.8	31.0	3.3	2000	0.0	2920	-2.7
		11.1	11.0	33.2	10.7	2030	1.5	2890	-3.7
		8.98	-10.2	33.1	10.3	2090	4.5	2980	-0.7
		9.81	-1.9	31.9	6.3	1980	-1.0	3000	0.0
28-Nov-2022	8	10.4	4.0	32.1	7.0	2030	1.5	3090	3.0
		10.5	5.0	30.3	1.0	2030	1.5	3040	1.3
		10.2	2.0	30.1	0.3	2080	4.0	3090	3.0
		10.0	0.0	30.1	0.3	2030	1.5	2960	-1.3
		10.5	5.0	31.3	4.3	2020	1.0	3020	0.7
		11.1	11.0	31.5	5.0	2070	3.5	3030	1.0
Mean Concentration Found (µg/mL)		9.96		31.2		2040		3010	
Inter-run SD		0.685		1.08		44.8		86.8	
Inter-run %CV		6.9		3.5		2.2		2.9	
Inter-run %Bias		-0.4		4.0		2.0		0.3	
n		18		18		18		18	

Criteria: At each level, the %CV was required to be ≤ 15.0%, except at the LLOQ level, where ≤ 20.0% was accepted. In addition, the mean %Bias at each level was required to be within ± 15.0% from nominal value, except at the LLOQ, where ± 20.0% was accepted.

Results: The acceptance criteria were met.

Table 17: Standard and QC of Failed Runs

Analytical Performance: Back-Calculated Concentrations (µg/mL) of Ertapenem Calibration Standard in (Human) (Urine) in (Study V1872106U3) (Rejected Runs)

Assay Date	Analytical Run Number	Cal 10 10.0 µg/mL	Cal 20 20.0 µg/mL	Cal 160 160 µg/mL	Cal 400 400 µg/mL	Cal 800 800 µg/mL	Cal 1600 1600 µg/mL	Cal 3200 3200 µg/mL	Cal 4000 4000 µg/mL
17-Nov-2022	3	9.52	21.2	166	387	798	1600	3150	3790
		9.82	21.3	164	392	811	1610	3210	3940
11-Jan-2023	12	8.17	20.1	163	399	791	1570	3100	3870
		11.2	22.5	164	406	797	1590	3160	3980
Mean		9.68	21.3	164	396	799	1590	3160	3900
S.D.		1.24	0.981	1.26	8.29	8.42	17.1	45.1	83.5
%CV		12.8	4.6	0.8	2.1	1.1	1.1	1.4	2.1
%Bias		-3.2	6.5	2.5	-1.0	-0.1	-0.6	-1.3	-2.5
n		4	4	4	4	4	4	4	4

Calibration Curve Parameters for Ertapenem Calibration Standards in Human Urine (Study V1872106U3) (Rejected Runs)

Run Date	Curve Number	Slope	Intercept	R-Squared	LLOQ	ULOQ
17-Nov-2022	3	0.00077	0.00094	0.9985	10.0	4000
11-Jan-2023	12	0.00076	0.00075	0.9945	10.0	4000

Analytical Performance of Ertapenem Quality Control Samples in Human Urine (Study V1872106U3) (Rejected Runs)

Run Date	Curve Number	QCS 10 10.0 µg/mL	%Bias	QCS 30 30.0 µg/mL	%Bias	QCS 2000 2000 µg/mL	%Bias	QCS 3000 3000 µg/mL	%Bias
17-Nov-2022	3	#6.89	-31.1	##22.8	-24.0	##1580	-21.0	2960	-1.3
		#7.24	-27.6	##23.2	-22.7	##1580	-21.0	3080	2.7
		#7.70	-23.0	##23.6	-21.3	##1570	-21.5	3030	1.0
		8.56	-14.4	##23.6	-21.3	##1600	-20.0	3050	1.7
		#7.50	-25.0	##22.6	-24.7	##1570	-21.5	2900	-3.3
		#7.36	-26.4	##23.2	-22.7	##1630	-18.5	3040	1.3
Intrarun Mean		7.54		23.2		1590		3010	
Intrarun SD		0.568		0.408		23.2		66.9	
Intrarun %CV		7.5		1.8		1.5		2.2	
Intrarun %Bias		-24.6		-22.7		-20.5		0.3	
n		6		6		6		6	
11-Jan-2023	12			28.9	-3.7	1970	-1.5	2860	-4.7
				29.9	-0.3	1970	-1.5	2890	-3.7
				29.7	-1.0	2000	0.0	2970	-1.0
				31.2	4.0	1980	-1.0	2830	-5.7
				29.5	-1.7	1990	-0.5	2950	-1.7
				32.7	9.0	2010	0.5	2990	-0.3
Intrarun Mean				30.3		1990		2920	
Intrarun SD				1.39		16.3		64.4	
Intrarun %CV				4.6		0.8		2.2	
Intrarun %Bias				1.0		-0.5		-2.7	
n				6		6		6	
# > 20%Bias									
## > 15%Bias									

Criteria: For each run, the calculated concentrations of at least 75% of the calibration standards are required to interpolate within ± 15.0% from nominal with a minimum of six calibration standard levels, except for the LLOQ for which it was required to be within ± 20.0%. In order for the run to be acceptable, at least one QC at each concentration level and two-thirds of the total number of QC samples were required to interpolate within ± 15.0% from nominal.

Results: Run 3 LLOQ, low, and mid QC's failed criteria, therefore, the acceptance criteria were not met. Run 12 the fresh cals/QCs were prepared incorrectly due to a calculation error such that the nominal and actual concentrations differ by 10x. The run was rejected.

Table 18: Standards and QCs of Non-P&A Runs

Analytical Performance: Back-Calculated Concentrations (µg/mL) of Ertapenem Calibration Standard in (Human) (Urine) in (Study V1872106U3)

Assay Date	Analytical Run Number	Cal 10 10.0 µg/mL	Cal 20 20.0 µg/mL	Cal 160 160 µg/mL	Cal 400 400 µg/mL	Cal 800 800 µg/mL	Cal 1600 1600 µg/mL	Cal 3200 3200 µg/mL	Cal 4000 4000 µg/mL
21-Nov-2022	5	10.0	19.6	158	388	810	1560	3030	3870
		9.70	21.3	166	420	818	1610	3230	3950
07-Dec-2022	10	9.45	20.3	162	388	823	1550	3280	3830
		10.3	20.6	164	402	817	1600	3160	3920
21-Dec-2022	11	10.0	19.5	160	394	832	1570	2990	3760
		9.75	21.2	169	422	807	1640	3100	3960
16-Jan-2023	13	9.97	20.8	169	417	825	1600	3080	3750
		9.74	20.1	165	410	831	1570	3050	3710
Mean		9.86	20.4	164	405	820	1590	3120	3840
S.D.		0.258	0.676	3.98	14.1	9.10	30.1	101	96.5
%CV		2.6	3.3	2.4	3.5	1.1	1.9	3.2	2.5
%Bias		-1.4	2.0	2.5	1.3	2.5	-0.6	-2.5	-4.0
n		8	8	8	8	8	8	8	8

Calibration Curve Parameters for Ertapenem Calibration Standards in Human Urine (Study V1872106U3)

Run Date	Curve Number	Slope	Intercept	R-Squared	LLOQ	ULOQ
21-Nov-2022	5	0.00057	0.00035	0.9986	10.0	4000
07-Dec-2022	10	0.00558	0.00193	0.9990	10.0	4000
21-Dec-2022	11	0.00606	0.00403	0.9981	10.0	4000
16-Jan-2023	13	0.00590	0.01157	0.9979	10.0	4000

Table 18: Standards and QCs of Non-P&A Runs (Continued)

Analytical Performance of Ertapenem Quality Control Samples in Human Urine (Study V1872106U3)

Run Date	Curve Number	QCS 10 10.0 µg/mL	%Bias	QCS 30 30.0 µg/mL	%Bias	QCS 2000 2000 µg/mL	%Bias	QCS 3000 3000 µg/mL	%Bias
21-Nov-2022	5	9.91	-0.9	29.3	-2.3	2020	1.0	2980	-0.7
		9.77	-2.3	29.1	-3.0	1960	-2.0	2890	-3.7
		9.45	-5.5	30.6	2.0	2040	2.0	3020	0.7
		11.6	16.0	31.5	5.0	2040	2.0	3080	2.7
		#13.2	32.0	31.6	5.3	2040	2.0	3090	3.0
		#6.88	-31.2	31.0	3.3	2030	1.5	3040	1.3
Intrarun Mean		10.1		30.5		2020		3020	
Intrarun SD		2.13		1.08		31.3		73.9	
Intrarun %CV		21.1		3.5		1.5		2.4	
Intrarun %Bias		1.0		1.7		1.0		0.7	
n		6		6		6		6	
07-Dec-2022	10			31.1	3.7	1990	-0.5	2980	-0.7
				31.4	4.7	2030	1.5	2930	-2.3
				30.8	2.7	2030	1.5	2970	-1.0
				32.0	6.7	2000	0.0	3060	2.0
				31.2	4.0	1960	-2.0	3010	0.3
				31.1	3.7	2060	3.0	3010	0.3
Intrarun Mean				31.3		2010		2990	
Intrarun SD				0.408		35.4		44.1	
Intrarun %CV				1.3		1.8		1.5	
Intrarun %Bias				4.3		0.5		-0.3	
n				6		6		6	
21-Dec-2022	11	10.6	6.0	27.6	-8.0	1970	-1.5	2850	-5.0
		9.63	-3.7	26.1	-13.0	2050	2.5	2970	-1.0
		10.3	3.0	29.4	-2.0	1980	-1.0	2980	-0.7
		10.1	1.0	30.5	1.7	2010	0.5	2980	-0.7
		9.35	-6.5	31.9	6.3	2030	1.5	2910	-3.0
		9.21	-7.9	30.7	2.3	2040	2.0	3010	0.3
Intrarun Mean		9.87		29.4		2010		2950	
Intrarun SD		0.554		2.16		32.7		59.0	
Intrarun %CV		5.6		7.3		1.6		2.0	
Intrarun %Bias		-1.3		-2.0		0.5		-1.7	
n		6		6		6		6	
16-Jan-2023	13			30.8	2.7	2030	1.5	2910	-3.0
				30.4	1.3	2060	3.0	2940	-2.0
				31.4	4.7	2030	1.5	2970	-1.0
				30.2	0.7	2020	1.0	2970	-1.0
				31.3	4.3	2030	1.5	2870	-4.3
				31.0	3.3	2040	2.0	2960	-1.3
Intrarun Mean				30.9		2040		2940	
Intrarun SD				0.481		13.8		39.8	
Intrarun %CV				1.6		0.7		1.4	
Intrarun %Bias				3.0		2.0		-2.0	
n				6		6		6	
# > 20%Bias									

Criteria: For each run, the calculated concentrations of at least 75% of the calibration standards are required to interpolate within ± 15.0% from nominal with a minimum of six calibration standard levels, except for the LLOQ for which it was required to be within ± 20.0%. In order for the run to be acceptable, at least one QC at each concentration level and two-thirds of the total number of QC samples were required to interpolate within ± 15.0% from nominal.

Results: Acceptance criteria were met in this run for batch acceptance, but not for precision and accuracy since the LLOQ %CV > 20%.

Table 19: Precision and Accuracy of Sample Dilution

Run Date	Curve Number	Dil QC 3000 2x 3000 µg/mL	%Bias	Dil QC 16,000 5x 16000 µg/mL	%Bias
21-Nov-2022	5	2990	-0.3	##7100	-55.6
		2930	-2.3	13900	-13.1
		2880	-4.0	15900	-0.6
		2890	-3.7	##10800	-32.5
		2920	-2.7	16900	5.6
		2920	-2.7	13900	-13.1
Mean		2920		13100	
S.D.		38.7		3600	
%CV		1.3		27.5	
%Bias		-2.7		-18.1	
n		6		6	
## > 15%Bias					

Run Date	Curve Number	Dil QC 16,000 5x 16000 µg/mL	%Bias
28-Nov-2022	8	16500	3.1
		15900	-0.6
		16400	2.5
		16300	1.9
		17500	9.4
		16800	5.0
Mean		16600	
SD		543	
%CV		3.3	
%Bias		3.8	
n		6	

Sample dilution as follows:

2x = 25 mcL samples + 25 mcL blank treated human urine
5x = 10 mcL samples + 40 mcL blank treated human urine

Criteria: To be deemed acceptable, the mean %Bias at each level must be within ± 15.0% with %CV ≤ 15.0%.
Results: In run 5 5x dilution did not meet criteria for both, %bias and %CV, and was repeated in run 8. The acceptance criteria were met.

Table 20: Analyte Adsorption Container Test

Run: 4
Adsorption Test of Ertapenem in Untreated Human Urine in Polypropylene (PP) and High-Density Polyethylene (HDPE)

Analyte	Sample Name	Sequence Number	Analyte Peak Area	IS Peak Area	Peak Area Ratio		Transfer 3	Transfer 2	Transfer 1	Time 0
Ertapenem	4 003 V1872106U3 Adsorption T0 1 1	3	19293	604049	0.0319395	Material	PP	PP	PP	N/A
30.0 mcg/mL	4 004 V1872106U3 Adsorption T0 2 1	4	18850	596376	0.0316076	Duration	~30 min on wet ice	~20 min on wet ice	~10 min on wet ice	Comparison
	4 005 V1872106U3 Adsorption T0 3 1	5	18395	602361	0.0305382					
	4 006 V1872106U3 Adsorption PP T1 1 1	6	18840	594404	0.0316956	Avg	0.0317295	0.0317001	0.0335044	0.0313617
	4 007 V1872106U3 Adsorption PP T1 2 1	7	20224	589295	0.0343190	Std Dev	0.000353	0.0011177	0.0015690	0.0007323
	4 008 V1872106U3 Adsorption PP T1 3 1	8	20416	591793	0.0344985	%CV	1.1	3.5	4.7	2.3
	4 009 V1872106U3 Adsorption PP T2 1 1	9	19138	581930	0.0328871	%Change	1.2	1.1	6.8	N/A
	4 010 V1872106U3 Adsorption PP T2 2 1	10	18668	608716	0.0306678					
	4 011 V1872106U3 Adsorption PP T2 3 1	11	18409	583573	0.0315453					
	4 012 V1872106U3 Adsorption PP T3 1 1	12	19119	608605	0.0314145					
	4 013 V1872106U3 Adsorption PP T3 2 1	13	19013	592116	0.0321103					
	4 014 V1872106U3 Adsorption PP T3 3 1	14	18992	599805	0.0316636					
	4 015 V1872106U3 Adsorption HDPE T1 1 1	15	17589	594818	0.0295704	Material	HDPE	HDPE	HDPE	N/A
	4 016 V1872106U3 Adsorption HDPE T1 2 1	16	16726	620157	0.0269706	Duration	~30 min on wet ice	~20 min on wet ice	~10 min on wet ice	Comparison
	4 017 V1872106U3 Adsorption HDPE T1 3 1	17	18431	639088	0.0288395					
	4 018 V1872106U3 Adsorption HDPE T2 1 1	18	18633	590805	0.0315383	Avg	0.0304153	0.0313679	0.0284602	0.0313617
	4 019 V1872106U3 Adsorption HDPE T2 2 1	19	18585	598175	0.0310695	Std Dev	0.0006886	0.0002592974	0.001341	0.0007323
	4 020 V1872106U3 Adsorption HDPE T2 3 1	20	18680	593093	0.0314959	%CV	2.3	0.8	4.7	2.3
	4 021 V1872106U3 Adsorption HDPE T3 1 1	21	18108	605308	0.0299153	%Change	-3.0	0.0	-9.3	N/A
	4 022 V1872106U3 Adsorption HDPE T3 2 1	22	18414	611157	0.0301297					
	4 023 V1872106U3 Adsorption HDPE T3 3 1	23	18775	601750	0.0312007					

$$\%\text{Change} = \frac{\text{mean peak area ratio of transferred samples} - \text{mean peak area ratio of T0}}{\text{mean peak area ratio of T0}}$$

Criteria: To be deemed acceptable, the %Change for each transfer must be within ± 15.0% with %CV ≤ 15.0%.
Results: The acceptance criteria were met for all transfers and both container types. No significant adsorption was observed..

Table 21: Freeze-Thaw Stability, (Frozen At -20 °C and -70 °C Thawed in Ice-Water Bath)

Without presence of zidebactam

Run Date	Curve Number	F/T -20/RT low 30.0 µg/mL	%Bias	F/T -70/RT low 30.0 µg/mL	%Bias	F/T -20/RT high 3000 µg/mL	%Bias	F/T -70/RT high 3000 µg/mL	%Bias
23-Nov-2022	6					2770	-7.7	3170	5.7
						#2540	-15.3	3090	3.0
						2650	-11.7	2960	-1.3
						2700	-10.0	3160	5.3
						2730	-9.0	#2280	-24.0
						2750	-8.3	2720	-9.3
Intrarun Mean						2690		2900	
Intrarun SD						84.6		346	
Intrarun %CV						3.1		11.9	
Intrarun %Bias						-10.3		-3.3	
n						6		6	
28-Nov-2022	8	31.0	3.3	32.5	8.3				
		29.5	-1.7	31.2	4.0				
		30.3	1.0	30.4	1.3				
		31.1	3.7	33.1	10.3				
		#36.8	22.7	32.3	7.7				
		31.0	3.3	32.3	7.7				
Intrarun Mean		31.6		32.0					
Intrarun SD		2.61		0.983					
Intrarun %CV		8.3		3.1					
Intrarun %Bias		5.3		6.7					
n		6		6					
# > 15%Bias									

With presence of zidebactam

Run Date	Curve Number	Comed1 F/T -20 low 30.0 µg/mL	%Bias	Comed1 F/T -70 low 30.0 µg/mL	%Bias	Comed1 F/T -20 high 3000 µg/mL	%Bias	Comed1 F/T -70 high 3000 µg/mL	%Bias
23-Nov-2022	6	31.7	5.7	33.6	12.0	2570	-14.3	3040	1.3
		30.3	1.0	#35.0	16.7	2650	-11.7	3210	7.0
		32.2	7.3	34.5	15.0	2670	-11.0	3110	3.7
		30.1	0.3	34.2	14.0	2610	-13.0	2940	-2.0
		30.2	0.7	34.5	15.0	2620	-12.7	3080	2.7
		32.0	6.7	#34.8	16.0	2680	-10.7	3040	1.3
Mean		31.1		34.4		2630		3070	
SD		0.983		0.493		41.3		89.4	
%CV		3.2		1.4		1.6		2.9	
%Bias		3.7		14.7		-12.3		2.3	
n		6		6		6		6	
# > 15%Bias									

Criteria: To be deemed stable, the mean %Bias at each level must be within ± 15.0% with %CV ≤ 15.0%.

Results: Ertapenem is stable in treated human urine with and without presence zidebactam for at least 3 cycles when frozen at -20 °C and -70 °C and thawed in wet ice.

Table 22: Bench-Top Stability in Wet Ice

Without presence of zidebactam

Run Date	Curve Number	BT 24hr low 30.0 µg/mL	%Bias	BT 6hr low 30.0 µg/mL	%Bias	BT 24hr high 3000 µg/mL	%Bias	BT 6hr high 3000 µg/mL	%Bias
23-Nov-2022	6					2920	-2.7	2950	-1.7
						2900	-3.3	3050	1.7
						2830	-5.7	3050	1.7
						2890	-3.7	2970	-1.0
						#1370*	-54.3*	#1770*	-41.0*
**With Outlier**						2720	-9.3	3060	2.0
Intrarun Mean						2610		2810	
Intrarun SD						609		511	
Intrarun %CV						23.3		18.2	
Intrarun %Bias						-13.0		-6.3	
n						6		6	
**Without Outlier**									
Intrarun Mean						2850		3020	
Intrarun SD						81.1		51.8	
Intrarun %CV						2.8		1.7	
Intrarun %Bias						-5.0		0.7	
n						5		5	
28-Nov-2022	8	31.6	5.3	31.8	6.0				
		32.1	7.0	33.0	10.0				
		31.5	5.0	34.1	13.7				
		32.5	8.3	31.6	5.3				
		30.8	2.7	33.1	10.3				
		32.0	6.7	33.7	12.3				
Intrarun Mean		31.8		32.9					
Intrarun SD		0.589		1.00					
Intrarun %CV		1.9		3.0					
Intrarun %Bias		6.0		9.7					
n		6		6					

Reason Deactivated
> 15%Bias
*statistical outlier (t-test, p<0.01)

With presence of zidebactam

Run Date	Curve Number	Comed1 BT 24hr low 30.0 µg/mL	%Bias	Comed1 BT 6hr low 30.0 µg/mL	%Bias	Comed1 BT 24hr high 3000 µg/mL	%Bias	Comed1 BT 6hr high 3000 µg/mL	%Bias
23-Nov-2022	6	33.5	11.7	34.0	13.3	2610	-13.0	2660	-11.3
		31.5	5.0	31.2	4.0	2670	-11.0	3000	0.0
		30.3	1.0	34.4	14.7	2590	-13.7	2980	-0.7
		31.7	5.7	33.2	10.7	3170	5.7	2840	-5.3
		32.2	7.3	34.5	15.0	3300	10.0	2940	-2.0
		32.3	7.7	33.9	13.0	2940	-2.0	2720	-9.3
Mean		31.9		33.5		2880		2860	
SD		1.06		1.23		305		142	
%CV		3.3		3.7		10.6		5.0	
%Bias		6.3		11.7		-4.0		-4.7	
n		6		6		6		6	

Criteria: To be deemed stable, the mean %Bias at each level must be within ± 15.0% with %CV ≤ 15.0%.
Results: Ertapenem is stable in treated human urine with and without presence of zidebactam for at least 24 hours in wet ice.

Table 23: Refrigeration Stability

Run Date	Curve Number	Treated Refr Low 30.0 µg/mL	%Bias	Untreated Refr Low 30.0 µg/mL	%Bias	Treated Refr High 3000 µg/mL	%Bias	Untreated Refr High 3000 µg/mL	%Bias
21-Nov-2022	5	26.3	-12.3	28.3	-5.7	2810	-6.3	2820	-6.0
		25.7	-14.3	29.2	-2.7	2760	-8.0	2810	-6.3
		##37.0	23.3	30.5	1.7	2680	-10.7	2770	-7.7
		*75.4		29.0	-3.3	2680	-10.7	2900	-3.3
		27.4	-8.7	28.5	-5.0	2730	-9.0	2810	-6.3
		26.7	-11.0	29.5	-1.7	2660	-11.3	2940	-2.0
With outlier									
Mean		36.4							
S.D.		19.6							
%CV		53.8							
%Bias		21.3							
n		6							
Without outlier									
Mean		28.6		29.2		2720		2840	
S.D.		4.73		0.789		57.6		64.3	
%CV		16.5		2.7		2.1		2.3	
%Bias		-4.7		-2.7		-9.3		-5.3	
n		5		6		6		6	
28-Nov-2022	8	30.3	1.0						
		33.0	10.0						
		32.4	8.0						
		31.4	4.7						
		31.2	4.0						
		31.7	5.7						
Mean		31.7							
S.D.		0.946							
%CV		3.0							
%Bias		5.7							
n		6							
Reason Deactivated									
* Statistical Outlier (t-test, p<0.01)									
## > 15%Bias									

Criteria: To be deemed stable, the mean %Bias at each level must be within ± 15.0% with %CV ≤ 15.0%.

Results: Run 5 treated refrigerated stability failed at the low concentration and was repeated in run 8. Ertapenem is stable in treated and untreated human urine for at least 19.0 hours at refrigerated temperatures.

Table 24: Long-Term Stability at -20 °C and -70 °C

Without presence of zidebactam

Run Date	Curve Number	LTS -20 low 30.0 µg/mL	%Bias	LTS -70 low 30.0 µg/mL	%Bias	LTS -20 high 3000 µg/mL	%Bias	LTS -70 high 3000 µg/mL	%Bias
21-Dec-2022	11	##20.9	-30.3	29.1	-3.0	##1330	-55.7	##27.6	-99.1
		##22.1	-26.3	##25.3	-15.7	##1390	-53.7	##27.1	-99.1
		##22.7	-24.3	27.3	-9.0	##1380	-54.0	##26.3	-99.1
		##22.6	-24.7	28.4	-5.3	##1440	-52.0	##25.6	-99.1
		##23.5	-21.7	29.2	-2.7	##1640	-45.3	2760	-8.0
		##21.5	-28.3	28.4	-5.3	##1670	-44.3	2790	-7.0
Intrarun Mean		22.2		28.0		1480		943	
Intrarun SD		0.926		1.47		144		1420	
Intrarun %CV		4.2		5.3		9.7		150.6	
Intrarun %Bias		-26.0		-6.7		-50.7		-68.6	
n		6		6		6		6	
16-Jan-2023	13							2880	-4.0
								2780	-7.3
								2770	-7.7
								2790	-7.0
								2760	-8.0
								2730	-9.0
Intrarun Mean								2790	
Intrarun SD								50.9	
Intrarun %CV								1.8	
Intrarun %Bias								-7.0	
n								6	
## > 15%Bias									

With presence of zidebactam

Run Date	Curve Number	Comed1 LTS -20 low 30.0 µg/mL	%Bias	Comed1 LTS -70 low 30.0 µg/mL	%Bias	Comed1 LTS -20 high 3000 µg/mL	%Bias	Comed1 LTS -70 high 3000 µg/mL	%Bias
21-Dec-2022	11	##21.3	-29.0	32.9	9.7	##1410	-53.0	2940	-2.0
		##21.8	-27.3	33.8	12.7	##1410	-53.0	2780	-7.3
		##22.7	-24.3	32.6	8.7	##1470	-51.0	2870	-4.3
		##23.6	-21.3	32.0	6.7	##1490	-50.3	2980	-0.7
		##23.8	-20.7	##35.7	19.0	##1400	-53.3	2910	-3.0
		##22.5	-25.0	33.1	10.3	##1440	-52.0	2760	-8.0
Intrarun Mean		22.6		33.4		1440		2870	
Intrarun SD		0.979		1.29		36.7		88.0	
Intrarun %CV		4.3		3.9		2.5		3.1	
Intrarun %Bias		-24.7		11.3		-52.0		-4.3	
n		6		6		6		6	
## > 15%Bias									

Criteria: To be deemed stable, the mean %Bias at each level must be within ± 15.0% with %CV ≤ 15.0%.

Results: Ertapenem is stable in treated human urine without presence of zidebactam for, up to 29 days upon storage in polypropylene tube at -70 °C. Ertapenem is stable in treated human urine with presence of zidebactam for up to 34 days upon storage in polypropylene tube at -70 °C. Ertapenem in treated human urine with and without the presence of zidebactam was not demonstrated to be stable at -20 °C over a period of 29 days and 34 days due to %Bias being outside of -15.0%. In run 12 -70 °C high QCs were prepared incorrectly and were repeated in run 13. It appears that the low QC was extracted in place of the high QC for four of the aliquots.

Table 25: Refrigerated Processed Sample Stability

Run Date	Curve Number	Processed Refr low 30.0 µg/mL	%Bias	Processed Refr high 3000 µg/mL	%Bias
28-Nov-2022	8	32.5	8.3	3060	2.0
		34.3	14.3	3200	6.7
		33.0	10.0	3170	5.7
		31.6	5.3	3180	6.0
		33.7	12.3	#2210	-26.3
		#35.5	18.3	*-0.157	
Mean		33.4		2960	
SD		1.38		425	
%CV		4.1		14.4	
%Bias		11.3		-1.3	
n		6		5	
Reason Deactivated					
* Lost during extraction					
# > 15%Bias					

Samples were processed with run 6, and then stored for 5 days 21 hours in polypropylene 96 well plates at refrigerated temperatures prior to injection with run 8.

Criteria: To be deemed stable, the mean %Bias at each level must be within ± 15.0% with %CV ≤ 15.0%.
Results: The acceptance criteria were met.

Table 26: Individual Sample Reinjection Reproducibility

Run Date	Curve Number	Indiv Reinj low 30.0 µg/mL	%Bias	Indiv Reinj high 3000 µg/mL	%Bias
28-Nov-2022	8	29.0	-3.3	2850	-5.0
		29.7	-1.0	2930	-2.3
		31.2	4.0	2890	-3.7
		31.9	6.3	2930	-2.3
		30.2	0.7	2940	-2.0
		29.3	-2.3	2960	-1.3
Mean		30.2		2920	
SD		1.13		39.8	
%CV		3.7		1.4	
%Bias		0.7		-2.7	
n		6		6	

Low and high QC extracts (6 each) were injected during the run and then reinjected at the end of the same run.

Criteria: To be deemed reproducible, at least four out of six QCs at each level must interpolate with dev[%] within ± 15.0% from nominal, and the mean %Bias at each level must be within ± 15.0% with %CV ≤ 15.0%.

Results: Run 3 individual sample reinjection reproducibility could not be evaluated due to analytical QC failure and was repeated in run 8. The acceptance criteria for run 8 were met.

Table 27: Whole Run Reinjection Reproducibility and Column Ruggedness

Analytical Performance: Back-Calculated Concentrations (µg/mL) of Ertapenem Calibration Standard in (Human) (Urine) in (Study V1872106U3)

Assay Date	Analytical Run Number	Cal 10 10.0 µg/mL	Cal 20 20.0 µg/mL	Cal 160 160 µg/mL	Cal 400 400 µg/mL	Cal 800 800 µg/mL	Cal 1600 1600 µg/mL	Cal 3200 3200 µg/mL	Cal 4000 4000 µg/mL
28-Nov-2022	9	10.1	19.1	159	412	823	1530	2990	3890
		9.99	20.3	168	433	808	1580	3200	3920
Mean		10.0	19.7	164	423	816	1560	3100	3910
%Bias		0.0	-1.5	2.5	5.8	2.0	-2.5	-3.1	-2.3

Calibration Curve Parameters for Ertapenem Calibration Standards in Human Urine (Study V1872106U3)

Run Date	Curve Number	Slope	Intercept	R-Squared	LLOQ	ULOQ
28-Nov-2022	9	0.00306	0.00317	0.9982	10.0	4000

Analytical Performance of Ertapenem Quality Control Samples in Human Urine (Study V1872106U3)

Run Date	Curve Number	QCS 10 10.0 µg/mL	%Bias	QCS 30 30.0 µg/mL	%Bias	QCS 2000 2000 µg/mL	%Bias	QCS 3000 3000 µg/mL	%Bias
28-Nov-2022	9	9.58	-4.2	31.1	3.7	2050	2.5	3020	0.7
		9.01	-9.9	30.6	2.0	2100	5.0	3140	4.7
		9.70	-3.0	29.6	-1.3	2060	3.0	3080	2.7
		10.3	3.0	31.2	4.0	2110	5.5	3180	6.0
		9.16	-8.4	33.2	10.7	2100	5.0	3250	8.3
		9.92	-0.8	31.2	4.0	2240	12.0	3140	4.7
Mean		9.61		31.2		2110		3140	
S.D.		0.478		1.18		68.1		79.4	
%CV		5.0		3.8		3.2		2.5	
%Bias		-3.9		4.0		5.5		4.7	
n		6		6		6		6	

Note: Run 9 is a reinjection of run 6 on a different analytical column of the same manufacturer, dimensions, and type, using same MS platform (Applied Biosystems API5000) – reinjected within 5 days 9 hours

Criteria: At each level, the %CV was required to be ≤ 15.0% from the nominal value, except at the LLOQ level, where ≤ 20.0% was accepted. The mean %Bias at each level was also required to be within ± 15.0%, except at the LLOQ, where ± 20.0% was accepted.

Results: The acceptance criteria were met.

Table 28: Extraction Recovery – Ertapenem

Run: 2

Control (Post-extraction Spike)					Extracted QCs					
QC Conc. *mcg/mL*	Drug Peak Area	Drug Peak Area (corrected)	IS Peak Area	Peak Area Ratio	QC Conc. *mcg/mL*	Drug Peak Area	Drug Peak Area (corrected)	IS Peak Area	Peak Area Ratio	Extraction Recovery %
Low QC					Low QC					
30.000	19442	9721	615805	0.0158	30.000	7958	9948	663850	0.0150	96.4
	19141	9571	627084	0.0153		7391	9239	638542	0.0145	93.1
	19463	9732	617824	0.0158		7239	9049	639172	0.0142	91.1
	19199	9600	620936	0.0155		7649	9561	633458	0.0151	97.1
	19336	9668	623088	0.0155		7271	9089	629515	0.0144	92.9
	18616	9308	601909	0.0155		7587	9484	631856	0.0150	96.6
			Average	0.0155					Average	94.5
Mid QC					Mid QC					
2000.00	1294615	647308	605018	1.0699	2000.00	475147	593934	619326	0.9590	91.7
	1292296	646148	626847	1.0308		477908	597385	622053	0.9603	91.9
	1283051	641526	621350	1.0325		475547	594434	617797	0.9622	92.1
	1316338	658169	630087	1.0446		485677	607096	617682	0.9829	94.0
	1285216	642608	605405	1.0615		479923	599904	605857	0.9902	94.7
	1290166	645083	624807	1.0325		471952	589940	586355	1.0061	96.3
			Average	1.0453					Average	93.4
High QC					High QC					
3000.0	1955601	977801	634258	1.5416	3000.0	909129		606306	1.4995	98.0
	1920200	960100	624536	1.5373		914019		625739	1.4607	95.4
	1955290	977645	635926	1.5374		910431		620213	1.4679	95.9
	1864816	932408	615098	1.5159		935015		624073	1.4982	97.9
	1971920	985960	646487	1.5251		919319		621013	1.4804	96.7
	1887467	943734	618021	1.5270		911357		623598	1.4614	95.5
			Average	1.5307					Average	96.6
										======
									n:	18
									Overall Mean Recovery:	**94.8**
									Overall Standard Deviation:	**2.2**
									Overall % CV:	**2.4**

Note: It was found that recovery solutions were calculated based on reported concentrations in plasma instead of actual concentrations in treated matrix (which are half of the reported concentrations). A correction factor of 0.5 is applied to the areas of the post extraction spike samples. In addition, there was a prep error in the mid and low QC preps, resulting in concentrations 25% lower than the nominal concentration. A 1.25 correction factor is applied to the extracted low and mid QC.

$$\%\ \text{Recovery} = \frac{\text{Response Ratio of "Extracted QC"}}{\text{Average Response Ratio of Control}} \times 100$$

Table 29: Extraction Recovery – Ertapenem-d_4

Run: 2

Control				Extracted QCs				
WIS Conc. *500 mcg/mL*	IS Peak Area	Drug Peak Area	Peak Area Ratio	WIS Conc. *500 mcg/mL*	IS Peak Area	Drug Peak Area	Peak Area Ratio	Extraction Recovery %
	594604	487971	1.2185		619326	475147	1.3034	107.1
	587354	478819	1.2267		622053	477908	1.3016	106.9
	577952	486681	1.1875		617797	475547	1.2991	106.7
	598793	495058	1.2095		617682	485677	1.2718	104.5
	586442	478172	1.2264		605857	479923	1.2624	103.7
	583050	472039	1.2352		586355	471952	1.2424	102.1
		Average	1.2173					======
							n:	6
							Mean Recovery:	**105.2**
							Standard Deviation:	**2.1**
							%CV:	**2.0**

$$\% \text{ Recovery} = \frac{\text{Response Ratio of "Extracted QC"}}{\text{Average Response Ratio of Control}} \times 100$$

Table 30: Stability of Ertapenem Primary Solution in MES Buffer at Room Temperature

V1872106P5 Run: 4
Container: Polypropylene

Primary Solution - Solution Stability at Room Temperature									
Concentration: 8.00 mg/mL									
Stability Data (Room Temperature)					Comparison Data (4°C)				
Lot #:	V1872106P5-14-1				Lot #:	V1872106P5-14-2			
Prepared:	3-Nov-2022				Prepared:	3-Nov-2022			
Sample ID	Seq #	Drug Peak Area	IS Peak Area	Peak Area Ratio	Sample ID	Seq #	Drug Peak Area	IS Peak Area	Peak Area Ratio
Primary BT-1	7	5620573	88122	63.7817	Primary Refr-1	5	5960585	91974	64.8073
Primary BT-1	13	6621884	95052	69.6659	Primary Refr-1	11	5865724	92488	63.4215
Primary BT-1	19	7062212	113045	62.4726	Primary Refr-1	17	6074087	95377	63.6850
Primary BT-2	8	5736744	88649	64.7130	Primary Refr-2	6	4583100	70979	64.5698
Primary BT-2	14	6152635	92769	66.3221	Primary Refr-2	12	5209699	84342	61.7687
Primary BT-2	20	7002169	106053	66.0252	Primary Refr-2	18	7361633	107453	68.5103
			Mean	65.4968				Mean	64.4604
			n	6				n	6
			SD	2.4917				SD	2.2565
			%CV	3.8				%CV	3.5
%Change =	1.6								
Duration:	6.75	Hours							

$$\%\text{Change} = \frac{\text{mean response of stored solutions} - \text{mean response of comparison solutions}}{\text{mean response of comparison solutions}} \times 100$$

Criteria: To be deemed stable, % Change upon storage for analyte must be within ±15.0% and %CV must be within ≤15.0%.
Meets Criteria: YES

Table 31: Stability of Ertapenem Primary Solution in MES Buffer, ~0.1M, pH 6.4 at -20 °C

V1872106P5 Run: 10
Container: Polypropylene

Concentration: 8.00 mg/mL									
Stability Data (Stored -20°C)					Comparison Data (Fresh)				
Lot #:	V1872106P5-2-1				Lot #:	V1872106P5-38-1			
Prepared:	3-Nov-2022				Prepared:	10-Nov-2022			
Sample ID	Seq #	Drug Peak Area	IS Peak Area	Peak Area Ratio	Sample ID	Seq #	Drug Peak Area	IS Peak Area	Peak Area Ratio
Primary stored-1	4	5817264	30968	187.8476	Primary fresh-1	3	5482273	32017	171.2301
Primary stored-1	8	5954724	31229	190.6793	Primary fresh-1	7	6302709	32203	195.7181
Primary stored-1	12	5658237	34334	164.7998	Primary fresh-1	11	6405482	36041	177.7276
Primary stored-2	6	5097485	30613	166.5137	Primary fresh-2	5	6041908	32145	187.9579
Primary stored-2	10	4919923	28241	174.2121	Primary fresh-2	9	5971914	32011	186.5582
Primary stored-2	14	5479678	31178	175.7546	Primary fresh-2	13	6214568	31401	197.9099
			Mean	176.6345				Mean	186.1836
			n	6				n	6
			SD	10.6969				SD	10.2646
			%CV	6.1				%CV	5.5
%Change =	-5.1								
Duration:	7	Days							

$$\%\text{Change} = \frac{\text{mean response of stored solutions} - \text{mean response of comparison solutions}}{\text{mean response of comparison solutions}} \times 100$$

Criteria: To be deemed stable, % Change upon storage for analyte must be within ±15.0% and %CV must be within ≤15.0%.
Meets Criteria: YES

17. FIGURES

Figure 1: Typical Calibration Curve

Analytical Run 6 Run Acceptance Parameters for Ertapenem (µg/mL) (Study V1872106U3)

Regression Type	LINEAR
Weighting Factor	1/X**2
Slope	0.002862988422712780
Intercept	0.002624502091028920
R-Squared	0.997642704774169
LLOQ	10
ULOQ	4000
Mean Std %Diff	3.6
S.D. Std %Diff	2.1
Mean Std Response	0.002916
S.D. Std Response	0.000154

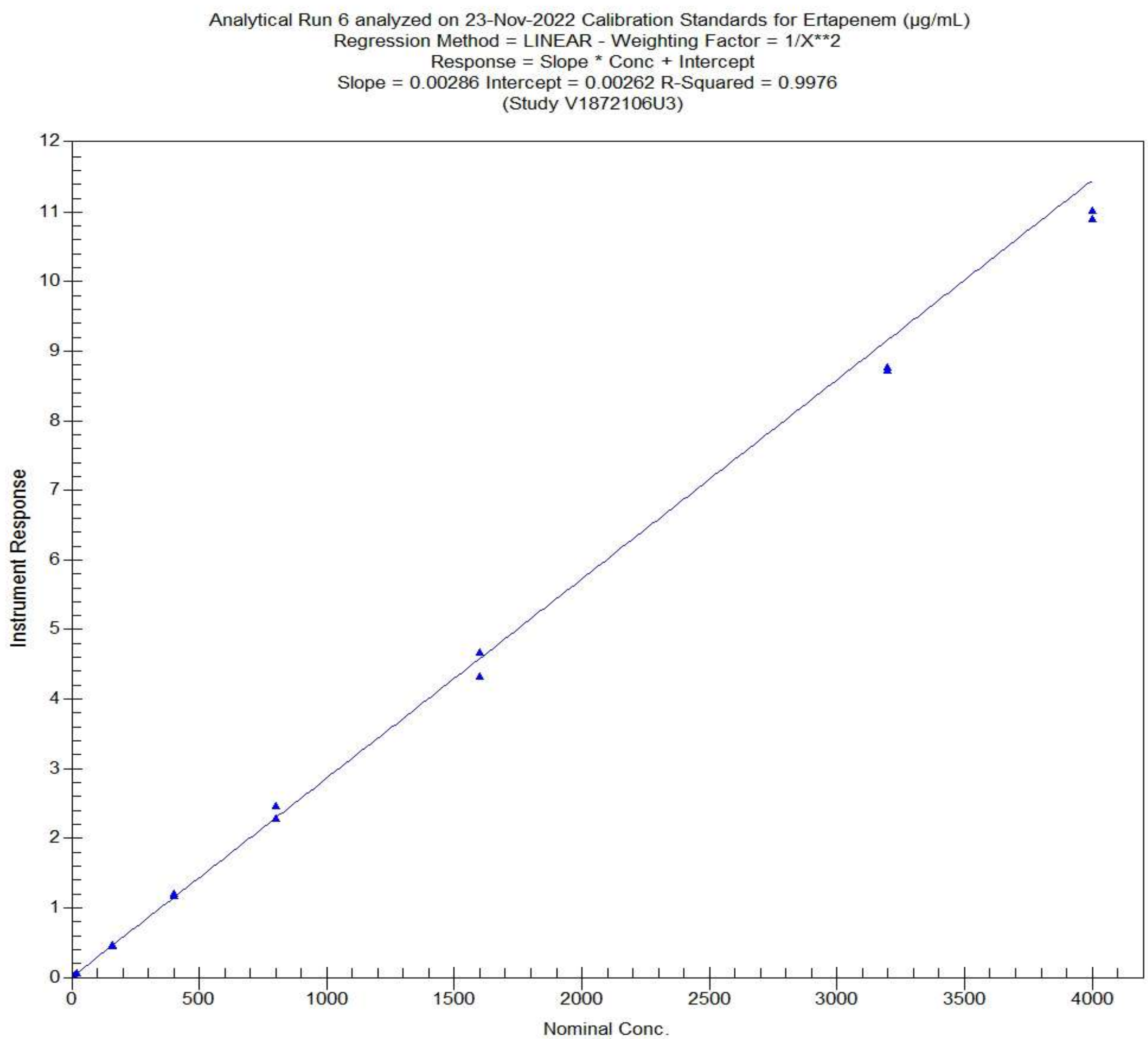

Figure 2: Typical Ion Chromatogram Blank Human Urine Without Internal Standard

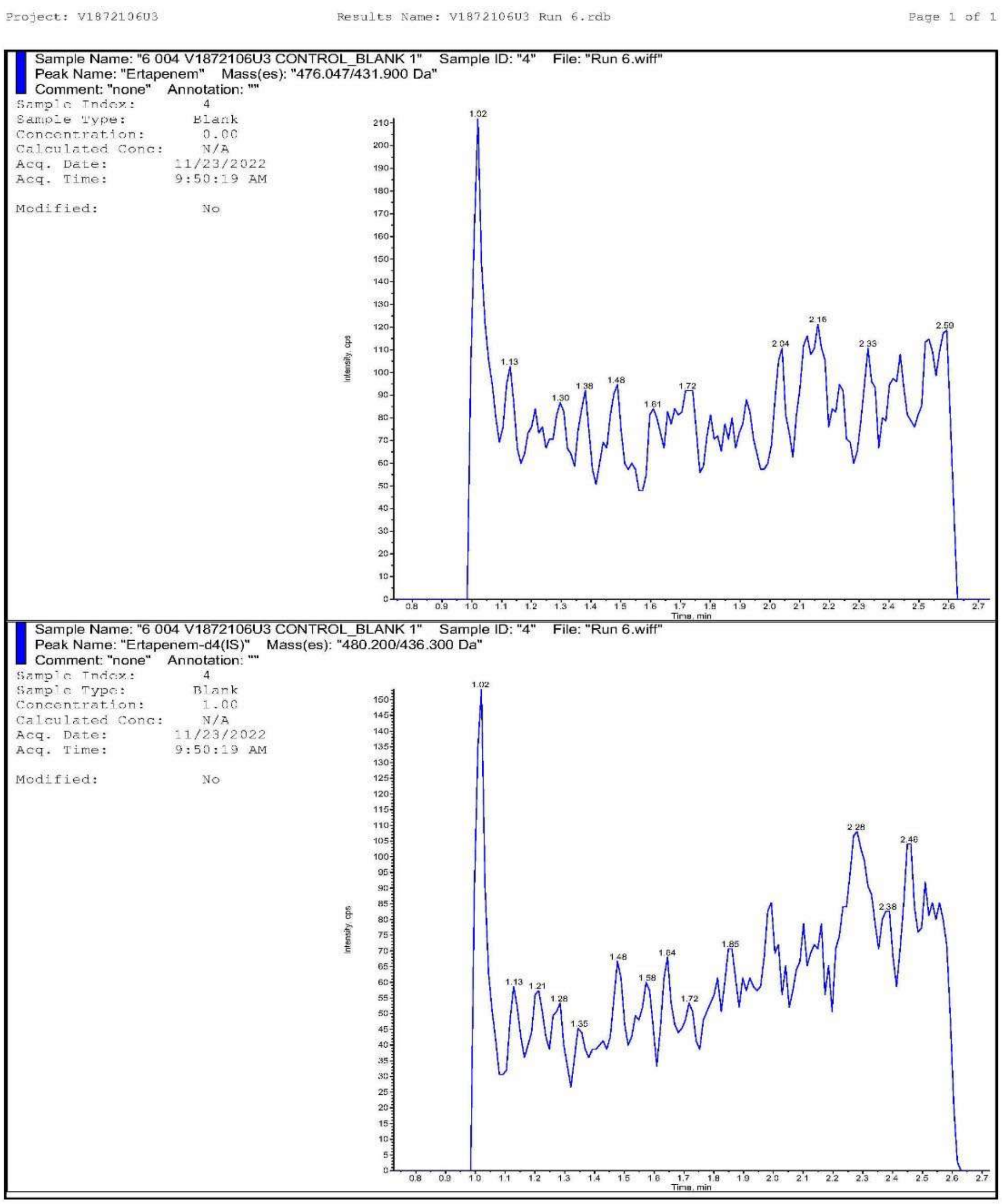

Figure 3: Typical Ion Chromatogram Blank Human Urine With Internal Standard

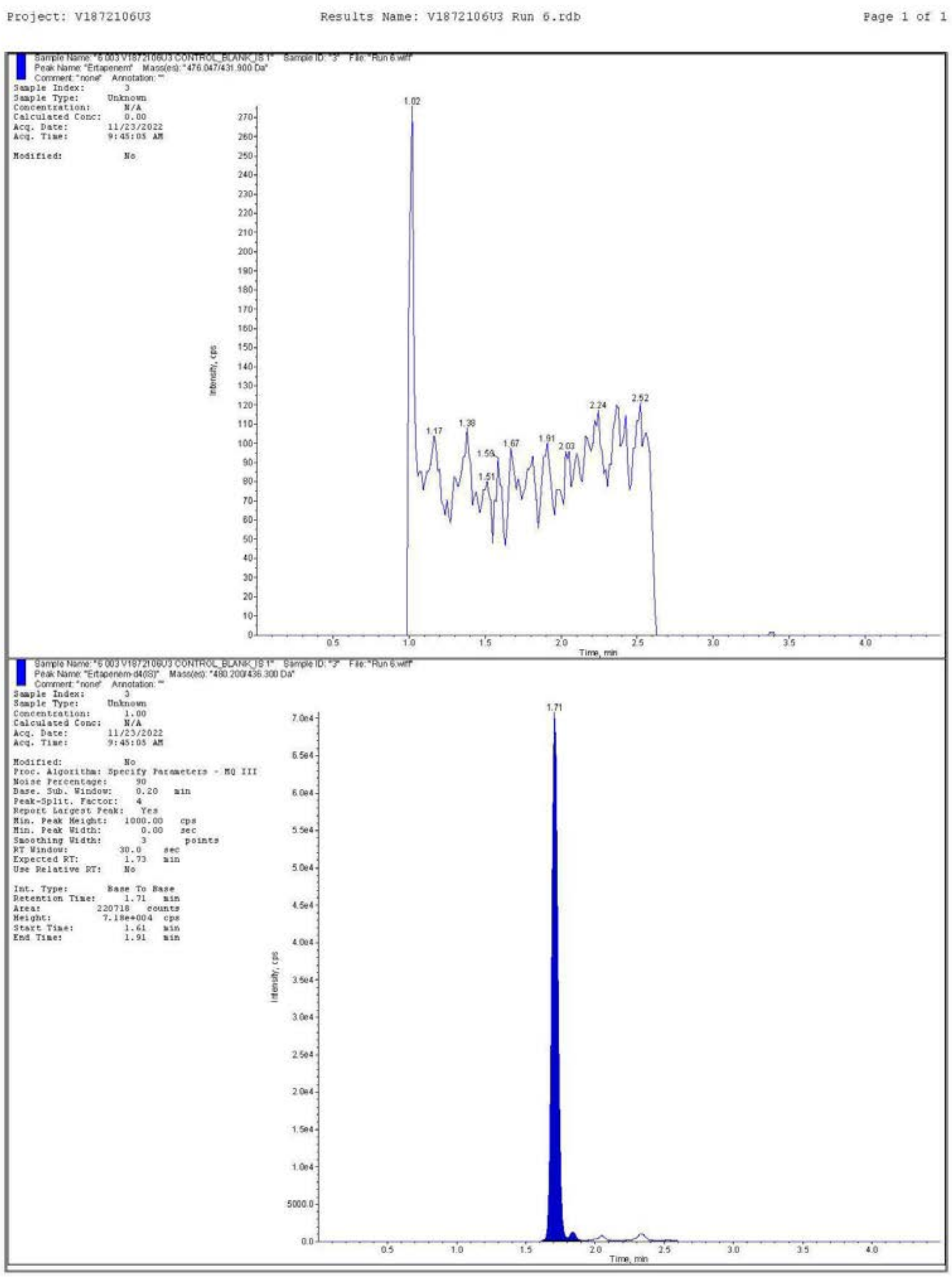

Figure 4: Typical Ion Chromatogram LLOQ Calibration Standard, Ertapenem = 10.0 mcg/mL

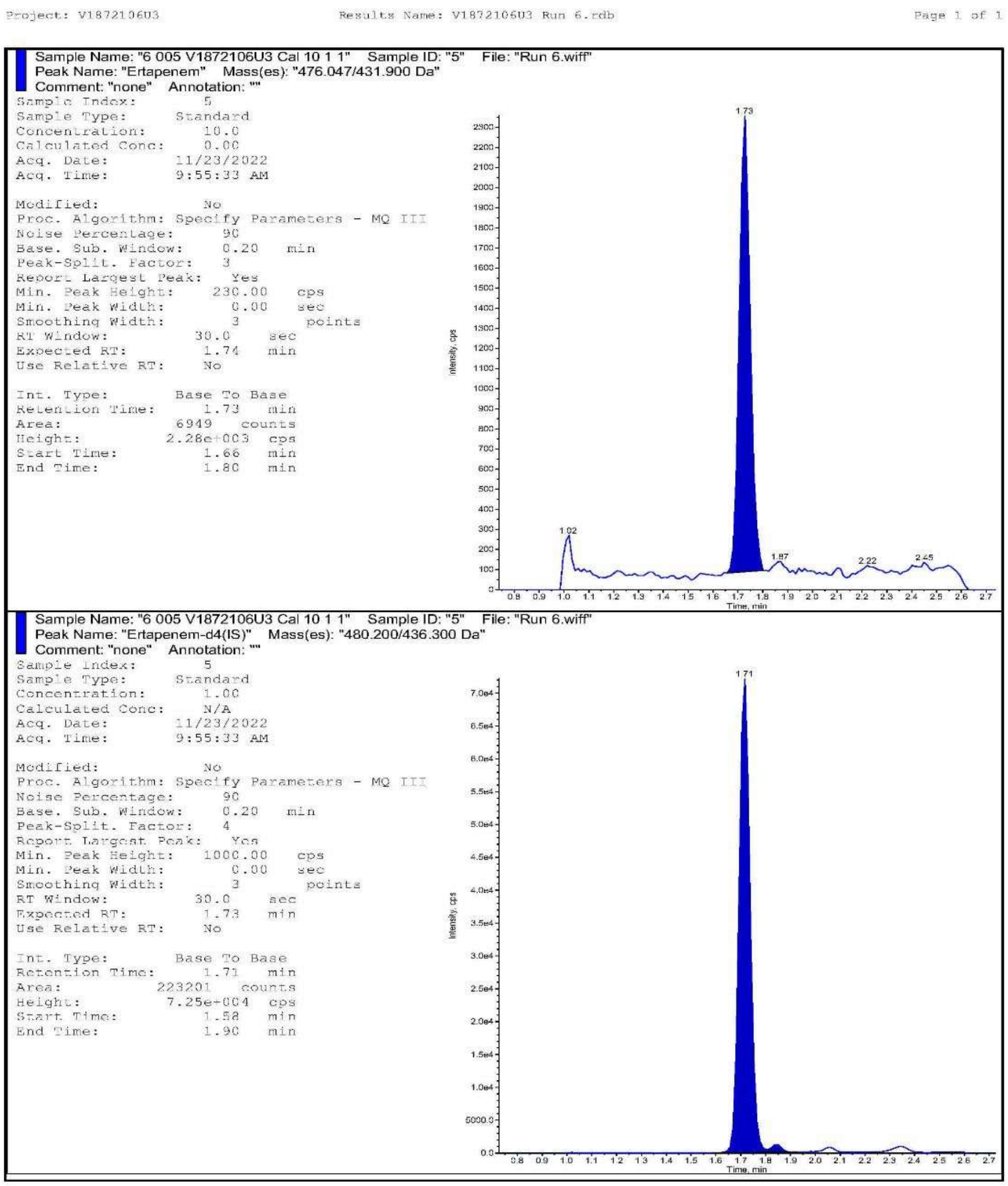

Figure 5: Typical Ion Chromatogram Calibration Standard, Ertapenem = 1600 mcg/mL

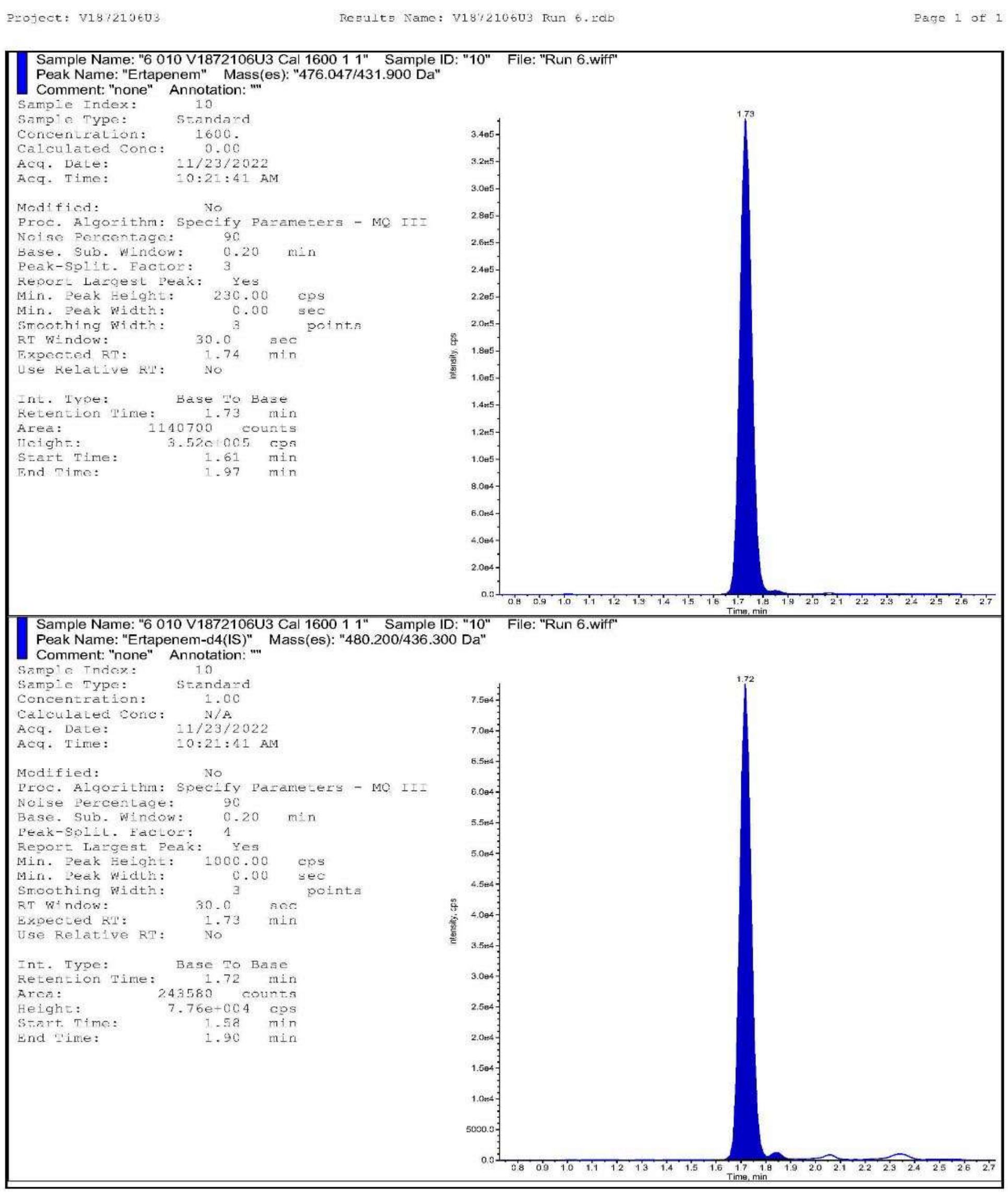

Figure 6: Typical Ion Chromatogram ULOQ Calibration Standard, Ertapenem = 4000 mcg/mL

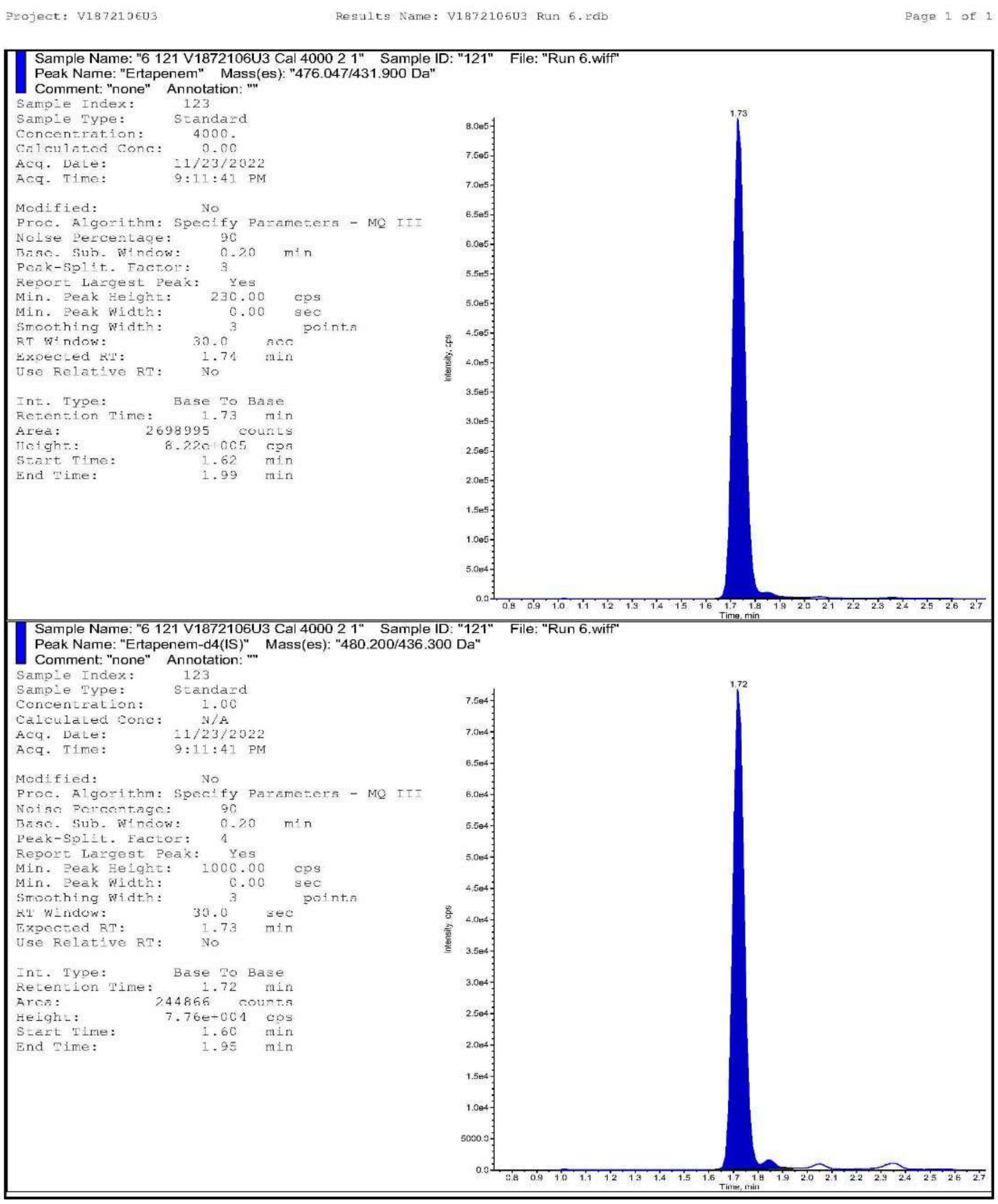

Figure 7: Typical Ion Chromatogram Cross-Talk Evaluation High QC No IS

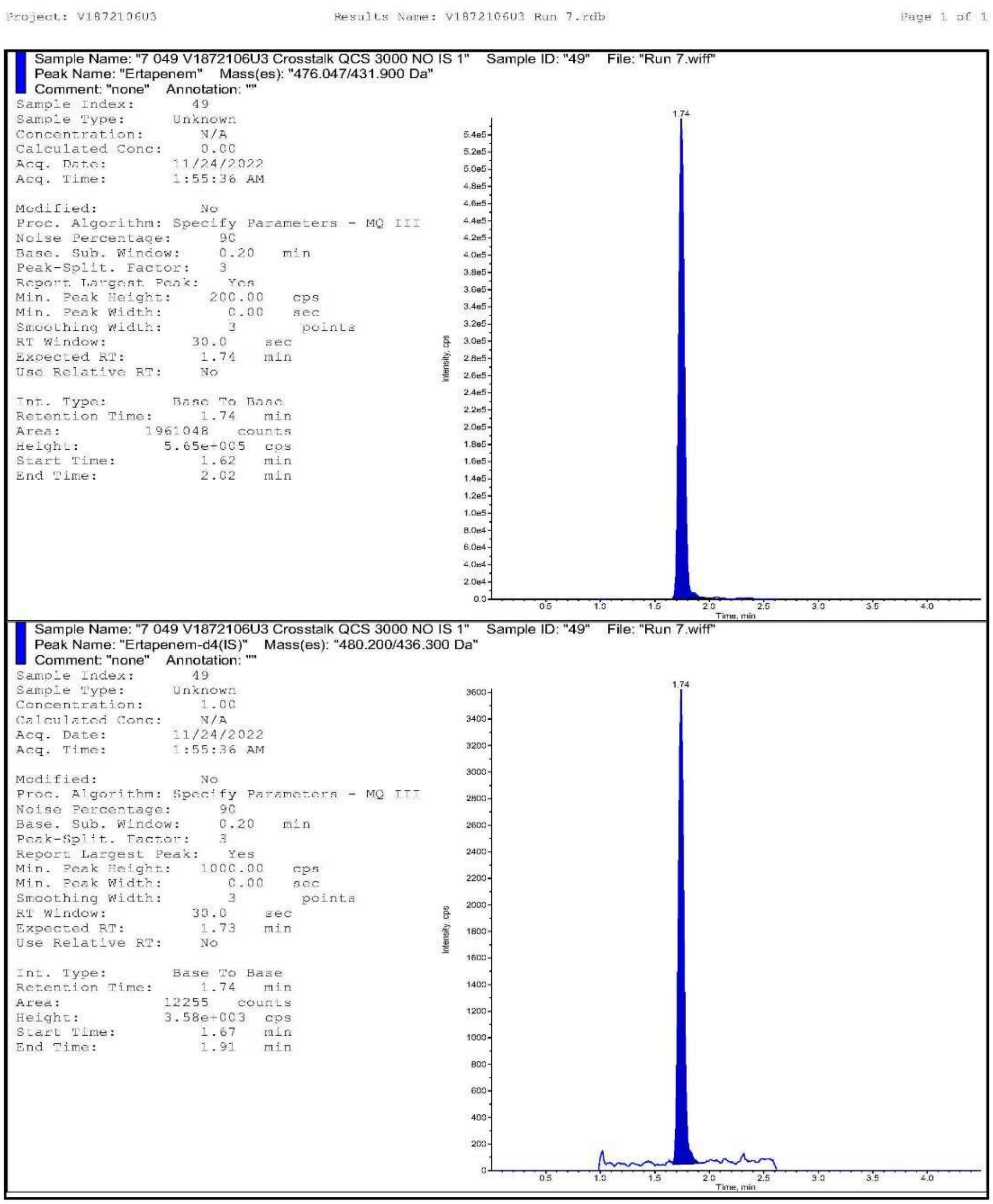

1. GENERAL INFORMATION

1.1. Client Name: Dynport Vaccine Company

1.2. Reference: Validation V1872106U3

2. TABLE OF CONTENTS

1. GENERAL INFORMATION 1
1.1. Client Name: Dynport Vaccine Company 1
1.2. Reference: Validation V1872106U3 1
2. TABLE OF CONTENTS 1
3. SAMPLE REQUIREMENTS 3
4. METHOD SUMMARY 3
5. SPECIAL HANDLING OF BIOLOGICAL SAMPLES 3
6. DATA COLLECTION AND ANALYSIS SOFTWARE 3
7. INSTRUMENTS, EQUIPMENT, AND CONDITIONS 4
7.1. LC and MS Conditions 4
Table 1: HPLC System 4
7.1.1. Gradient Table 5
7.1.2. Autosampler Settings 5
7.1.3. Mass Spectrometer System 5
8. REAGENTS, STANDARDS, AND SUPPLIES 7
Table 2: Reagents, Standards, and Supplies 7
9. SAFETY 7
10. PROCEDURE 8
10.1. Preparation of Analyte Stock Solutions and Dilutions 8

CONFIDENTIAL

Serial Number: f8e32a97-97cb-462d-913f-49b17ebec9a4 accessed by Trey Vinke (VINKET) on Thu, 12 Jan 2023 21:25:01 GMT.

10.1.1. Ertapenem Primary Solution, 80.0 mg/mL (Prepare on wet ice) 8
10.1.2. Working Internal Standard, ~100 mcg/mL Ertapenem-d_4 (Prepare on wet ice)....8
10.1.3. Working Internal Standard, ~500 mcg/mL Ertapenem-d_4 (Prepare on wet ice)....8
10.2. Preparation of Other Reagents....9
10.2.1. Mobile Phase A: 0.5% Formic Acid in Deionized Water 9
10.2.2. Mobile Phase B: 90/10 Acetone / Deionized Water....9
10.2.3. Rinse: 90/10 Acetone/Deionized Water 9
10.2.4. 1 N Sodium Hydroxide (NaOH) in Deionized Water 9
10.2.5. MES (2-(N-Morpholino) Ethanesulphonic Acid Hydrate) Buffer pH 6.4, ~0.1M....9
10.2.6. 80/20 Acetonitrile/Deionized Water....9
10.2.7. 5/95 Acetonitrile/Deionized Water....10
10.3. Preparation of Standards and QC Samples....10
10.3.1. Treated Human Urine (Human Urine: MES Buffer 1:1)....10
10.3.2. Ertapenem Secondary Solution, 8000 mcg/mL in Treated Human Urine (Reported as 16000 mcg/mL; Prepare on wet ice)....10
10.3.3. Preparation of Standards and QC Samples in Treated Human Urine (Prepare on wet ice) 10
10.4. Extraction Procedure 11
10.5. Dilutions 11
10.6. Calculations 12
11. REFERENCE CHROMATOGRAMS 12
Figure 1: Blank without IS 12
Figure 2: Blank with IS 13
Figure 3: Carryover Blank with IS....13
Figure 4: LLOQ – Ertapenem 10.0 mcg/mL....14
12. REVISION HISTORY 14

CONFIDENTIAL

Serial Number: f8e32a97-97cb-462d-913f-49b17ebec9a4 accessed by Trey Vinke (VINKET) on Thu, 12 Jan 2023 21:25:01 GMT.

Table 3: Revision History 14

3. SAMPLE REQUIREMENTS

Human urine samples (treated with 1:1 MES Buffer pH 6.4, ~0.1M), minimum volume 50 mcL each, for determination of ertapenem concentration.

Refer to the relevant study protocol for a description of expected samples.

4. METHOD SUMMARY

The method involves 96-well protein precipitation followed by chromatographic separation and tandem MS detection using the Sciex 5000 LC/MS/MS system. The concentration range is 10.0 - 4000 mcg/mL in human urine only. Since unknown samples are treated with the MES buffer after collection at a 1:1 ratio, the calibration standard and QC concentrations are based on the urine volume only. Unknown samples do not need to be adjusted for the dilution with MES buffer.

5. SPECIAL HANDLING OF BIOLOGICAL SAMPLES

The method requires no special handling. Please ensure all "Universal Precautions" are taken.

- Primaries, spiking solutions, calibration standards, and QCs should be prepared on wet ice. Each prepared level should be aliquoted immediately and aliquots should be flash frozen in a dry-ice/acetone bath for ~1 minute and stored in the freezer immediately after flash freezing.

6. DATA COLLECTION AND ANALYSIS SOFTWARE

**Description**	**Version**
Watson LIMS™ (Thermo Fisher Scientific)	7.5 or higher
Analyst® (AB Sciex)	1.7.2 or higher

CONFIDENTIAL

Serial Number: f8e32a97-97cb-462d-913f-49b17ebec9a4 accessed by Trey Vinke (VINKET) on Thu, 12 Jan 2023 21:25:01 GMT.

7. INSTRUMENTS, EQUIPMENT, AND CONDITIONS

Equivalent equipment can be substituted, except where noted in Section 7.1. Minor modifications of conditions are permitted to optimize chromatography, except where noted in Section 7.1. Refer to SOP-00073 for allowable minor modifications that do not require validation.

7.1. LC and MS Conditions

Table 1: HPLC System

Parameter	Parameter Value
System Controller	Shimadzu Controller, CBM20A
Pump	Shimadzu LC-30AD
Autosampler	Shimadzu SIL-30AC
Column Oven	Shimadzu CTO-30A
MS Interface	ESI
Analytical Column	Fortis Technology Ltd. Fortis C18 4.6 x 50mm, 5 µ
Column Guard Cartridge	Security Guard Cartridge AQ C18, 4.0 x 2.0 mm
Column Temperature (set at)	35°C
Mobile Phase A	0.5% Formic acid in deionized water
Mobile Phase B	90/10 Acetone/deionized water
Needle Wash Solvent (R0, R3)	90/10 Acetone/deionized water (or other acceptable solution)
Total Run Time (approximate)	4.5 min
Injection Volume (µl)	0.1-5.0
Flow Rate	1.0 mL/min
Pressure, kgf/cm2 (approximate)	~100
Approximate Retention Time (min.)	Ertapenem: 1.66
	Ertapenem-d4: 1.66

CONFIDENTIAL

Serial Number: f8e32a97-97cb-462d-913f-49b17ebec9a4 accessed by Trey Vinke (VINKET) on Thu, 12 Jan 2023 21:25:01 GMT.

7.1.1. Gradient Table

Time Program

Time	Module	Events	Parameter
0.00	Pumps	Pump B Conc.	18
0.50	Pumps	Pump B Conc.	18
1.00	Controller	Event 1	
2.40	Pumps	Pump B Conc.	45
2.50	Pumps	Pump B Conc.	100
2.60	Controller	Event 2	
3.50	Pumps	Pump B Conc.	100
3.60	Pumps	Pump B Conc.	18
4.20	System Controller	Stop	

7.1.2. Autosampler Settings

Temperature: Set at 4°C

Rinse Type: External

Rinse Mode: Before and after aspiration

Rinse Dip Time: 0 sec

Rinse Method: Rinse Pump Then Port

Rinse Time: 2 sec

Rinsing Speed: 35 µL/sec

Rinsing Volume: 500 µL

7.1.3. Mass Spectrometer System

Mass Spectrometer Sciex 5000 or 6500 (Low Mass Mode)

Source Type: Turbo spray

Scan Type: MRM (MRM)

Polarity: Positive

Resolution Q1: Unit

Resolution Q3: Unit

CONFIDENTIAL

Serial Number: f8e32a97-97cb-462d-913f-49b17ebec9a4 accessed by Trey Vinke (VINKET) on Thu, 12 Jan 2023 21:25:01 GMT.

All the below parameters in this section may be adjusted to optimize the instrument conditions.

Ertapenem:

Q1 Mass (Da)	Q3 Mass (Da)	Dwell (msec)
476.047	431.9	250.00
DP	56.00	
CE	13.00	
CXP	13.00	

Ertapenem-d_4 (IS):

Q1 Mass (Da)	Q3 Mass (Da)	Dwell (msec)
480.2	436.3	250.00
DP	56.00	
CE	13.00	
CXP	13.00	

Dummy Ions (to counter-act potential crosstalk):

Q1 Mass (Da)	Q3 Mass (Da)	Dwell (msec)
250	200	100
254	204	100

Parameter Table

CAD: 12.00
CUR: 40.00
GS1: 50.00
GS2: 60.00
IS: 5500
TEM: 600
EP 12

CONFIDENTIAL

Serial Number: f8e32a97-97cb-462d-913f-49b17ebec9a4 accessed by Trey Vinke (VINKET) on Thu, 12 Jan 2023 21:25:01 GMT.

8. REAGENTS, STANDARDS, AND SUPPLIES

Reagents from alternate supplier and equivalent grade can be substituted.

Table 2: Reagents, Standards, and Supplies

Chemical / Grade	Manufacturer/Supplier
Ertapenem	Performa
Ertapenem-d4	Sigma Aldrich
MES ([2-(N-Morpholino) ethane sulphonic acid hydrate]) buffer	Sigma
Acetonitrile, HPLC grade	Honeywell
Formic Acid, 98+%	EMD Millipore
Deionized Water	Millipore
Ammonium Acetate ≥ 99.9%	Sigma
Methanol	Honeywell
Acetone	Honeywell
Human Urine	In house
Advantage SiliGuard Coated 2 mL 96 well Polypropylene plate: Square Top, Round Bottom	Analytical Sales and Services, Catalog # 27P687GC
Purple, pre-slit, square well, soft silicone/PTFE Cap Mats	Analytical Sales & Services, Inc., Catalog# 965085

9. SAFETY

Always observe safe laboratory practices. Consult the appropriate SDS before handling any chemical if you are unfamiliar with the proper safety precautions.

CONFIDENTIAL

Serial Number: f8e32a97-97cb-462d-913f-49b17ebec9a4 accessed by Trey Vinke (VINKET) on Thu, 12 Jan 2023 21:25:01 GMT.

10. PROCEDURE

10.1. Preparation of Analyte Stock Solutions and Dilutions

The quantities of analyte solution prepared may be scaled up or down proportionally as needed. Alternate dilution schemes can be used to achieve the appropriate final concentrations of standards, QCs, working internal standard, and system verification solution (SVS). For assignment of expiration dates to analyte stocks and their dilutions, refer to the latest version of the validation report.

10.1.1. Ertapenem Primary Solution, 80.0 mg/mL (Prepare on wet ice)

Check drying conditions prior to use. Weigh approximately 400 mg of ertapenem reference standard (correcting for purity and correction factor if necessary) into a polypropylene container and add 5.0 mL of MES buffer, pH 6.4, ~0.1M. Mix well and sonicate if necessary. Store solution at -20°C in a polypropylene container.

10.1.2. Working Internal Standard, ~100 mcg/mL Ertapenem-d_4 (Prepare on wet ice)

For 1 mg size reference material bottles, quantitatively transfer using the following procedure: Transfer 1-2 mL of MES buffer, pH 6.4, ~0.1M directly into the reference material bottle containing 1.0 mg of ertapenem-d4, replace cap, and mix well. Transfer the whole volume from the bottle to a 15-mL polypropylene tube (with graduation). Thoroughly rinse the transfer pipette and bottle two more times with MES buffer, pH 6.4, ~0.1M into the same 15-mL polypropylene tube. Dilute to the 10-mL graduation line with more MES buffer, pH 6.4, ~0.1M. Mix well and sonicate if necessary. Store at or below -20°C in polypropylene.

OR

Check drying conditions prior to use. Weigh 5.0 mg of ertapenem-d_4 reference standard into a polypropylene container. Add 50 mL of MES buffer, pH 6.4, ~0.1M (do not correct for conversion factor and purity). Mix well and sonicate if necessary. **Aliquot** (~10 mL aliquots) and store at or below -20 °C in polypropylene containers.

The following WIS concentration is validated as well:

10.1.3. Working Internal Standard, ~500 mcg/mL Ertapenem-d_4 (Prepare on wet ice)

Check drying conditions prior to use. Weigh 25.0 mg of ertapenem-d_4 reference standard into a polypropylene container. Add 50 mL of MES buffer, pH 6.4, ~0.1M (do not correct for

CONFIDENTIAL

Serial Number: f8e32a97-97cb-462d-913f-49b17ebec9a4 accessed by Trey Vinke (VINKET) on Thu, 12 Jan 2023 21:25:01 GMT.

conversion factor and purity). Mix well and sonicate if necessary. **Aliquot** (~10 mL aliquots) and store at or below -20 °C in polypropylene containers.

10.2. Preparation of Other Reagents

Prepared quantities of all reagents may be scaled up or down as necessary. Volumes provided are examples. For assignment of expiration dates, refer to SOP-00094.

10.2.1. Mobile Phase A: 0.5% Formic Acid in Deionized Water

Add 20 mL of formic acid to a 4-L volumetric flask containing ~3800 mL of deionized water. Dilute to volume with deionized water. Mix well and store at room temperature.

10.2.2. Mobile Phase B: 90/10 Acetone / Deionized Water

Combine 3600 mL of acetone with 400 mL of deionized water in a 4-L flask. Mix well and store at room temperature.

10.2.3. Rinse: 90/10 Acetone/Deionized Water

Combine 3600 mL of acetone and 400 mL of deionized water in a 1-L flask. Mix well and store at room temperature.

10.2.4. 1 N Sodium Hydroxide (NaOH) in Deionized Water

Weight ~ 4.00 g of sodium hydroxide (F.W. 40.00) and gradually transfer it to a 100-mL volumetric flask containing ~50 mL of deionized water. Mix to dissolve and wait until it reaches room temperature. Dilute to volume with deionized water and mix well. Store in a polypropylene container at room temperature.

10.2.5. MES (2-(N-Morpholino) Ethanesulphonic Acid Hydrate) Buffer pH 6.4, ~0.1M

Weigh ~ 4000 mg of MES hydrate (F.W. 195.24) and transfer to a 200-mL volumetric flask containing ~150 mL of deionized water. Dissolve and adjust the pH to 6.4 with 1N NaOH. Dilute to volume with deionized water. Mix well and store refrigerated.

10.2.6. 80/20 Acetonitrile/Deionized Water

Combine 800 mL of acetonitrile and 200 mL of deionized water in a 1-L flask. Mix well and stored at room temperature.

CONFIDENTIAL

Serial Number: f8e32a97-97cb-462d-913f-49b17ebec9a4 accessed by Trey Vinke (VINKET) on Thu, 12 Jan 2023 21:25:01 GMT.

10.2.7. 5/95 Acetonitrile/Deionized Water

Combine 50 mL of acetonitrile and 950 mL of deionized water in a 1-L flask. Mix well and store at room temperature.

10.3. Preparation of Standards and QC Samples

10.3.1. Treated Human Urine (Human Urine: MES Buffer 1:1)

Add 200 mL of chilled human urine to a polypropylene container containing 200 mL of chilled 0.1M MES Buffer, pH 6.4. Mix well and store at -20°C.

It is recommended to prepare the treated human urine fresh prior to usage.

10.3.2. Ertapenem Secondary Solution, 8000 mcg/mL in Treated Human Urine (Reported as 16000 mcg/mL; Prepare on wet ice)

Transfer 1.0 mL of ertapenem primary solution (80.0 mg/mL) to a polypropylene tube containing 9.0 mL of **chilled** treated human urine on wet ice. Mix well, aliquot, and store at -70 °C or -20 °C.

10.3.3. Preparation of Standards and QC Samples in Treated Human Urine (Prepare on wet ice)

Use treated human urine that has been diluted 1:1 with MES buffer, screened for the lack of interference at the retention times of ertapenem and ertapenem-d_4 (IS). Starting from the ertapenem secondary in treated human urine (8000 mcg/mL), prepare a series of calibration standards and QCs with treated human urine on wet ice. Since unknown samples are treated with the MES buffer after collection at a 1:1 ratio, the calibration standard and QC concentrations are based on the urine volume only. Unknown samples do not need to be adjusted for the dilution with MES buffer. The prepared concentrations in urine only, not accounting for MES buffer, will be as follows: For standards: 4000, 3200, 1600, 800, 400, 160, 20.0, and 10.0 mcg/mL. For QCs: 3000, 2000, and 30.0 mcg/mL, and 10.0 mcg/mL for the LLOQ QC when needed. Other concentrations may be prepared, provided they meet the requirements of SOP-00035 and provided they remain within the range of 10.0 to 4000 mcg/mL. Store solutions in polypropylene at -70°C or -20°C.

CONFIDENTIAL

Serial Number: f8e32a97-97cb-462d-913f-49b17ebec9a4 accessed by Trey Vinke (VINKET) on Thu, 12 Jan 2023 21:25:01 GMT.

10.4. Extraction Procedure

1. Thaw human urine samples on wet ice. Once samples are thawed, vortex each sample for ~10 seconds immediately prior to pipetting.
2. Pipet 50 mcL of human urine sample or blank to the corresponding wells of a 2-mL 96-well plate on wet ice.
3. Add 50 mcL of **COLD** working IS (500 mcg/mL ertapenem-d_4 in MES buffer, pH 6.4, ~0.1M) to all applicable wells. For **Control Blanks** add 50 mcL of MES buffer, pH 6.4, ~0.1M instead.
4. Seal the plate with an adhesive pad (or equivalent) and vortex at 1300 rpm for 3 minutes using a plate shaker.
5. Add 250 mcL of acetonitrile to all wells. Seal the plate with an adhesive pad (or equivalent) and vortex for 5 minutes at 1200 rpm.
6. Centrifuge the plate for 15 minutes at a setting of 4000 rpm (at ambient conditions).
7. Transfer 50 mcL of supernatant volume to a new 2-mL 96-well plate using a multichannel pipette (or equivalent).
8. Add 300 mcL of 80/20 acetonitrile/deionized water to all wells. Seal the plate with an adhesive pad (or equivalent).
9. Vortex the plate at 1200 rpm for 5 minutes.
10. Transfer 100 mcL of the mixture into a new 2-mL 96-well plate (final plate), using a multichannel pipette (or equivalent).
11. Add 300 mcL of 5/95 acetonitrile/deionized water to each well. Seal the plate with a cap mat.
12. Vortex the final plate at 1200 rpm for 5 minutes.
13. Refrigerate prior to LC-MS injection according to the conditions outlined in Section 7.

10.5. Dilutions

2x = 25 mcL sample + 25 mcL blank treated human urine

5x = 10 mcL sample + 40 mcL of blank treated human urine

CONFIDENTIAL

Serial Number: f8e32a97-97cb-462d-913f-49b17ebec9a4 accessed by Trey Vinke (VINKET) on Thu, 12 Jan 2023 21:25:01 GMT.

10.6. Calculations

Linear regression is performed with a $1/x^2$ weighting factor.

Equation: Response = Slope * Conc + Intercept

11. REFERENCE CHROMATOGRAMS

Figure 1: Blank without IS

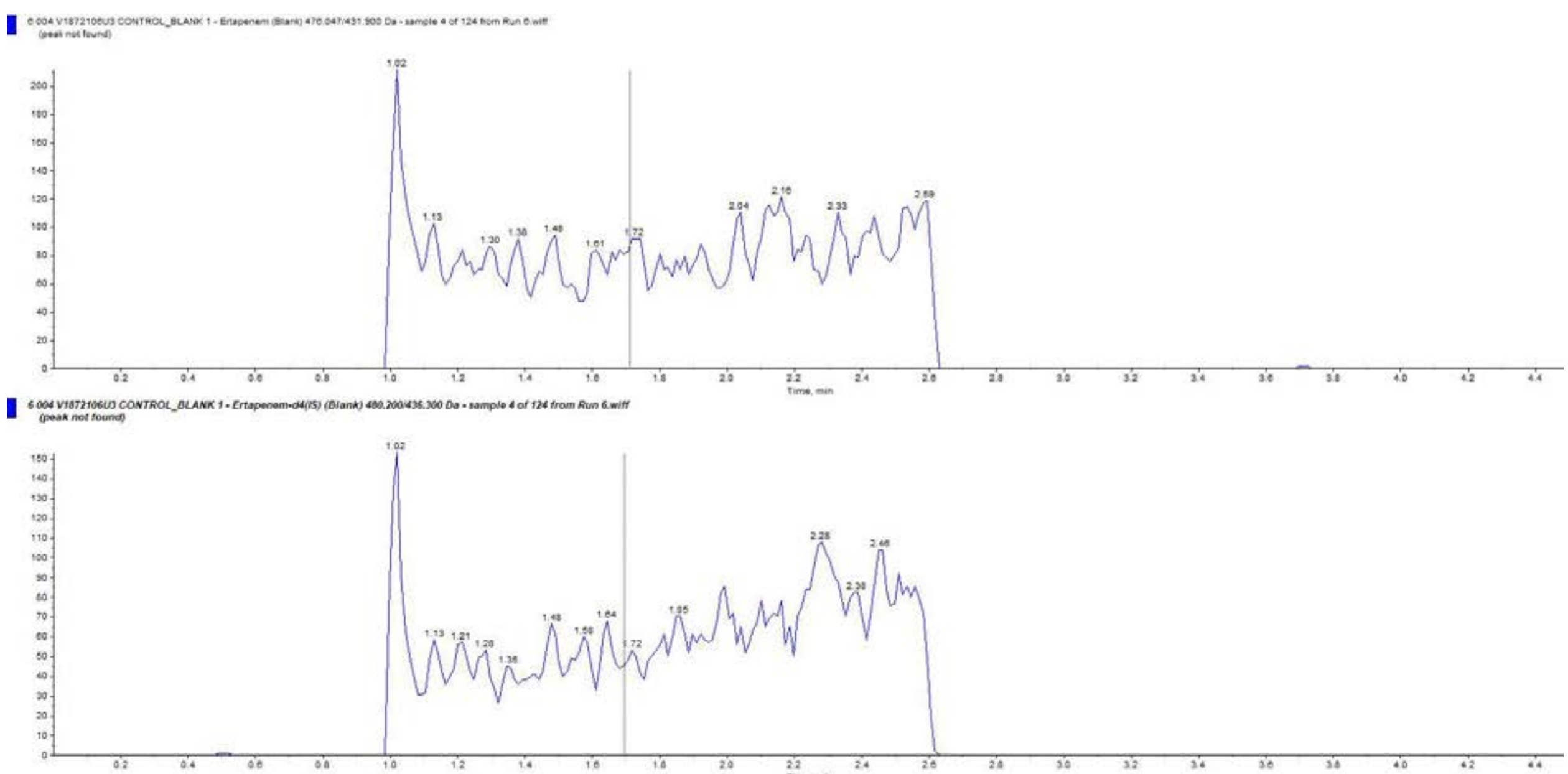

CONFIDENTIAL

Serial Number: f8e32a97-97cb-462d-913f-49b17ebec9a4 accessed by Trey Vinke (VINKET) on Thu, 12 Jan 2023 21:25:01 GMT.

Figure 2: Blank with IS

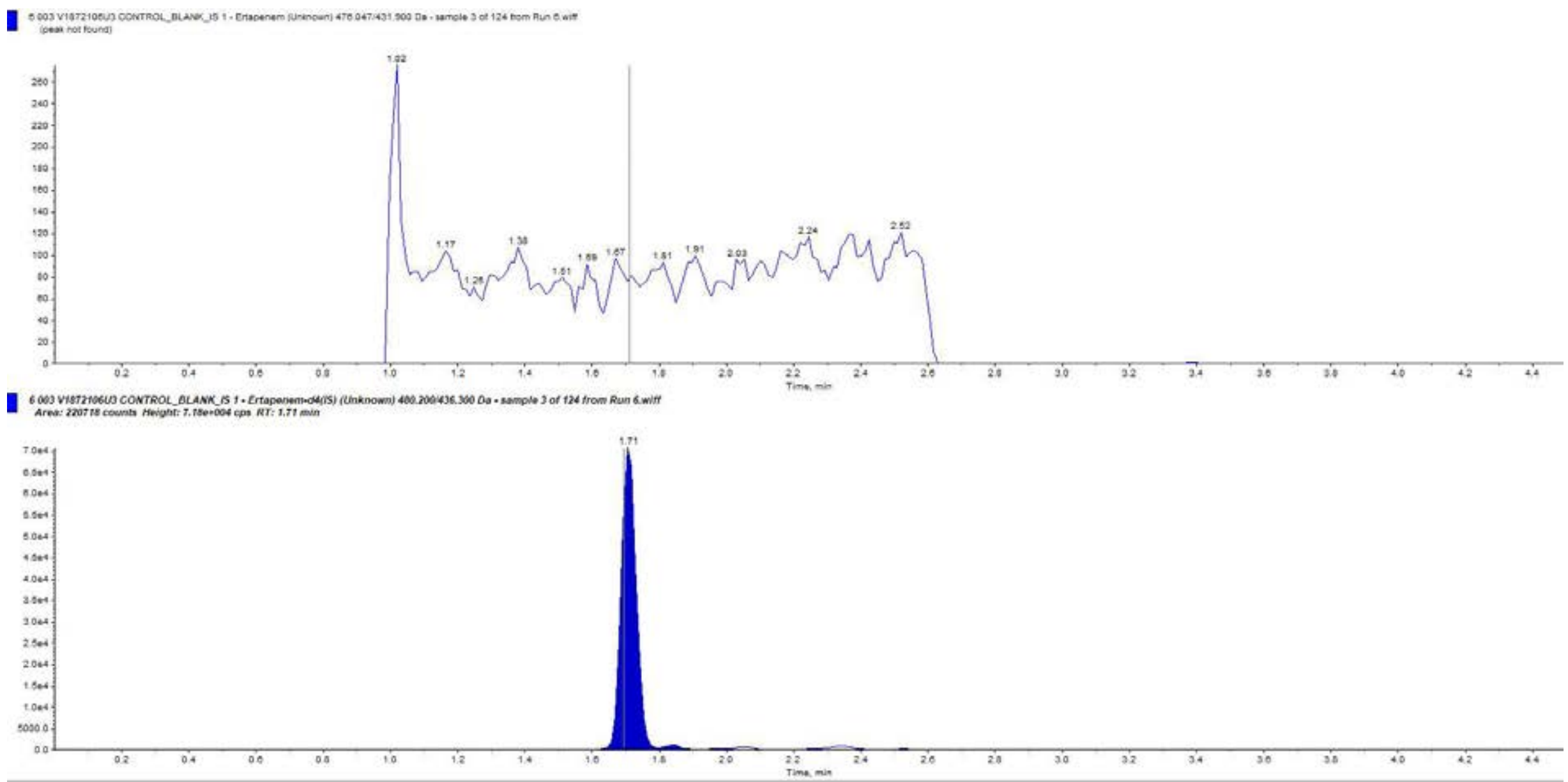

Figure 3: Carryover Blank with IS

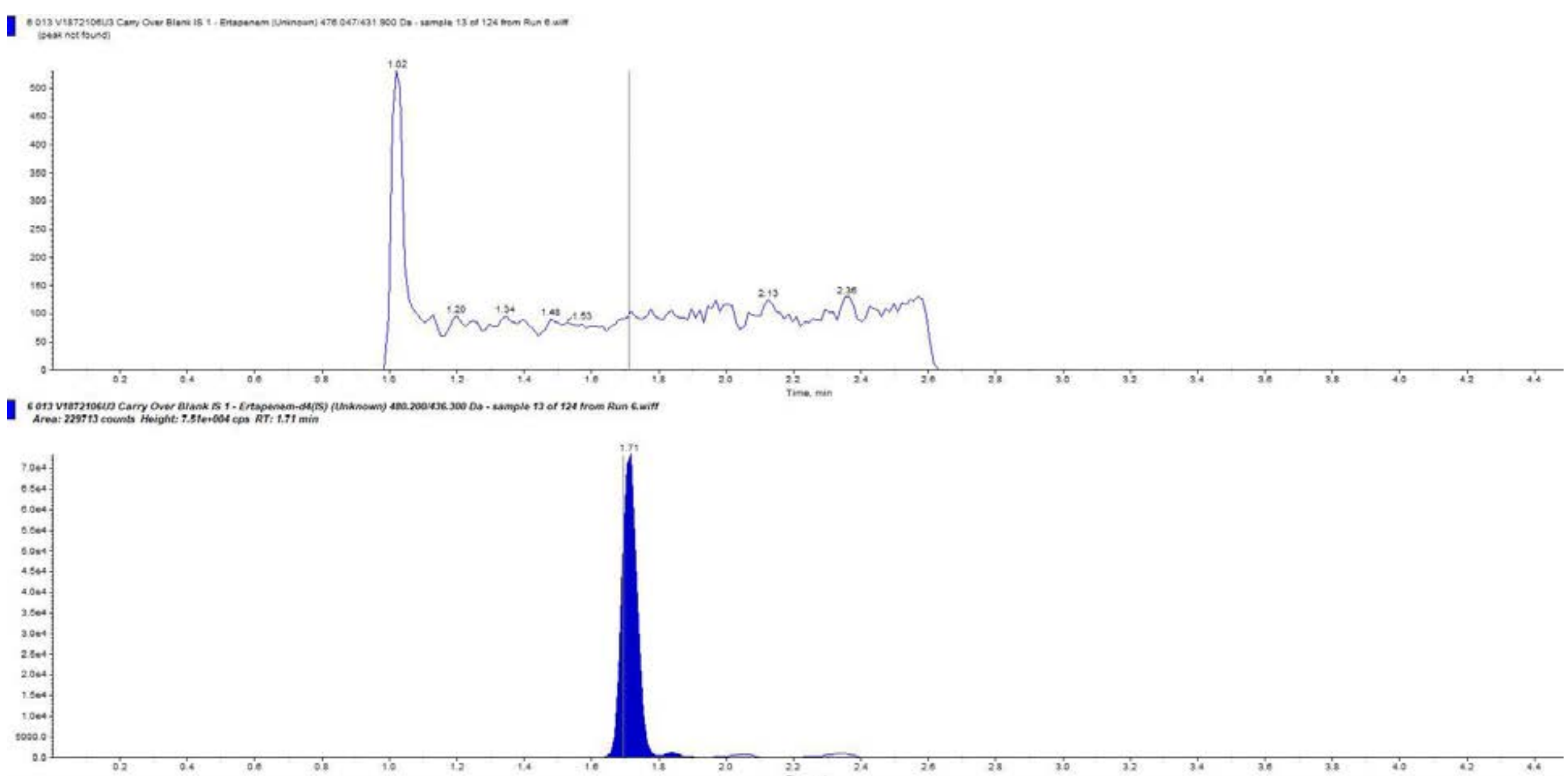

CONFIDENTIAL

Serial Number: f8e32a97-97cb-462d-913f-49b17ebec9a4 accessed by Trey Vinke (VINKET) on Thu, 12 Jan 2023 21:25:01 GMT.

Figure 4: LLOQ – Ertapenem 10.0 mcg/mL

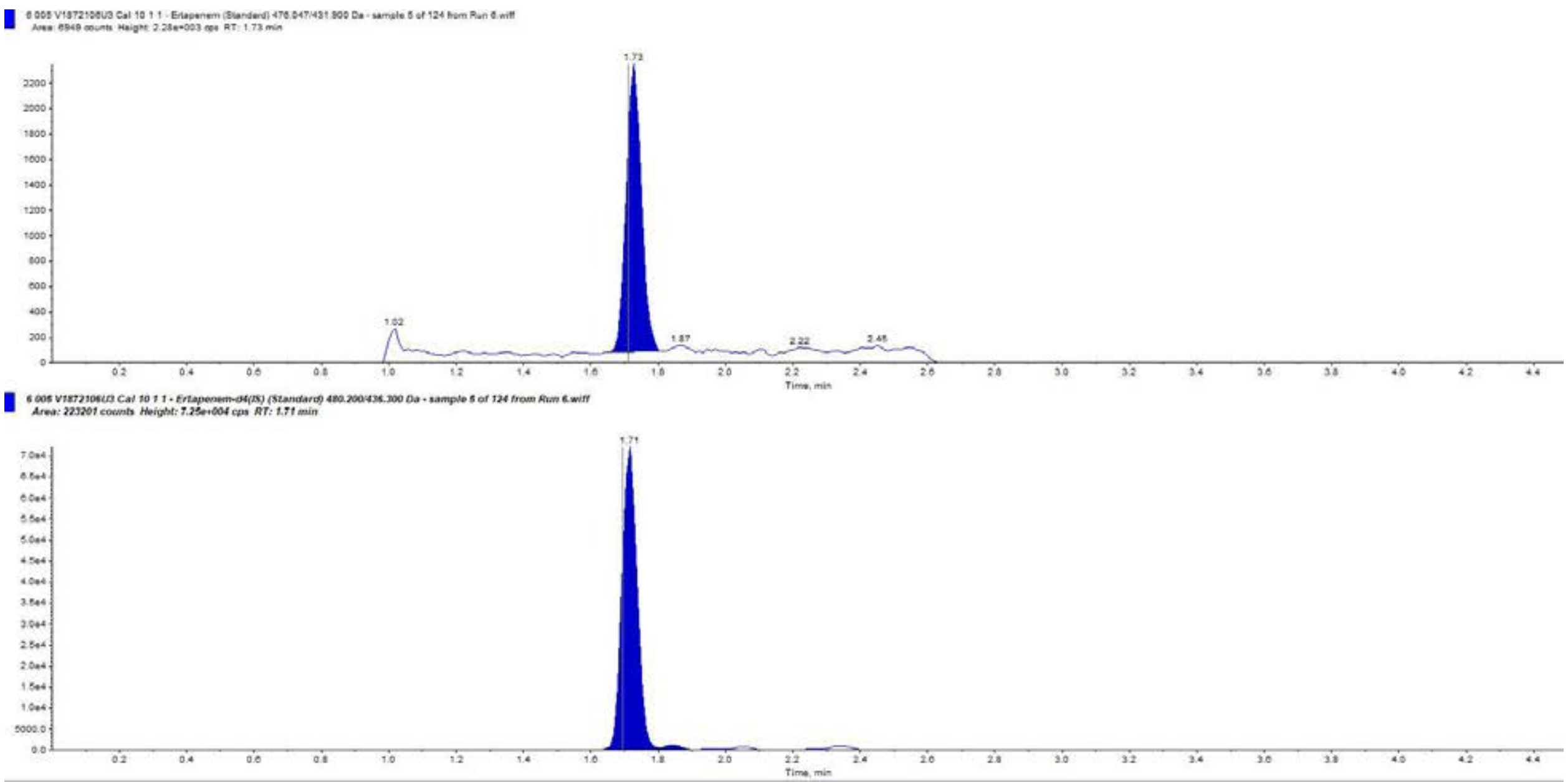

12. REVISION HISTORY

Table 3: Revision History

Revision	Effective Date	Section	Revision Summary
00	See Header	NA	New Procedure

CONFIDENTIAL

Serial Number: f8e32a97-97cb-462d-913f-49b17ebec9a4 accessed by Trey Vinke (VINKET) on Thu, 12 Jan 2023 21:25:01 GMT.

TP-80182 [00]: V1872106U1 - Ertapenem in Human Urine (50 mcL, 5.00 -2,000 mcg/mL)

All blank fields are considered as data not available. All Signature (Date and Time) shown in this report are in GMT.

**Type**	TP	**Status**	CURRENT	
**Effective Date**	06-Jan-2023	**Periodic Review Date**		
**Owner**	PIPPINA	**Department Name**	Pharma LC-MS/MS	Pharma LC-MS/MS
**Description**	Full Title: LC-MS/MS Analysis of Ertapenem in Treated Human Urine (50 mcL, 10.0 - 4000 mcg/mL; Prior to MES Treatment)			

Revision Notes (1)

Task Name	Action	Comment	Signature
Check In	Document Profile Preparation	Created outside of the system.	NEVINSC<br>05-Jan-2023 19:14:20 [GMT]

Serial Number: f8e32a97-97cb-462d-913f-49b17ebec9a4 accessed by Trey Vinke (VINKET) on Thu, 12 Jan 2023 21:25:01 GMT.

Approval Information

Note to Approver

Reviewer: Bryan Parmentier, MLT (ASCP)
Management: Marsha Luna, B.S.
Author & PI: Anika Pippin, B.S.

Approval Decision	Comment	Signature
Approve		PARMENTIERB<br>05-Jan-2023 19:57:23 [GMT]
Approve		LUNAM<br>05-Jan-2023 20:07:45 [GMT]
Approve		PIPPINA<br>06-Jan-2023 14:45:21 [GMT]

Serial Number: f8e32a97-97cb-462d-913f-49b17ebec9a4 accessed by Trey Vinke (VINKET) on Thu, 12 Jan 2023 21:25:01 GMT.

MedChemExpress

Certificate of Analysis

Inhibitors • Screening Libraries • Proteins

Ertapenem sodium

Cat. No.:	HY-13625
CAS No.:	153773-82-1
Batch No.:	180017
Chemical Name:	1-Azabicyclo[3.2.0]hept-2-ene-2-carboxylic acid, 3-[[(3S,5S)-5-[[(3-carboxyphenyl)amino]carbonyl]-3-pyrrolidinyl]thio]-6-[(1R)-1-hydroxyethyl]-4-methyl-7-oxo-, sodium salt (1:1), (4R,5S,6S)-

PHYSICAL AND CHEMICAL PROPERTIES

Molecular Formula:	$C_{22}H_{24}N_3NaO_7S$
Molecular Weight:	**497.50**
Storage:	4°C, sealed storage, away from moisture<br>* In solvent : -80°C, 6 months; -20°C, 1 month (sealed storage, away from moisture)

Chemical Structure:

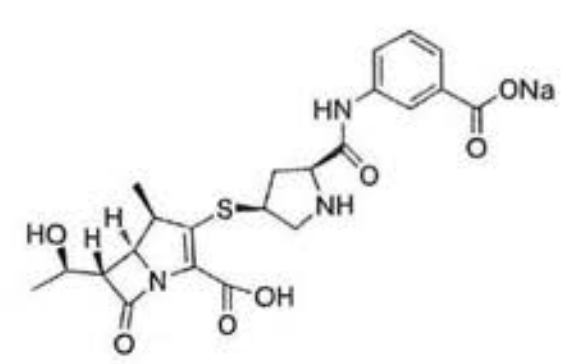

ANALYTICAL DATA

Appearance:	White to light yellow (Solid)
^{1}H NMR Spectrum:	Consistent with structure
LCMS:	Consistent with structure
Purity (LCMS):	**98.86%**
Test date:	2022/08/12
Retest date:	2024/08/11
Conclusion:	The product has been tested and complies with the given specifications.

Name: Lingli Ma
Designation: QA Manager
Date: 6/27/2022

Caution: Product has not been fully validated for medical applications. For research use only.

Tel: 609-228-6898 Fax: 609-228-5909 E-mail: tech@MedChemExpress.com
Address: 1 Deer Park Dr, Suite Q, Monmouth Junction, NJ 08852, USA

MedChemExpress

Product Data Sheet

Ertapenem sodium

Cat. No.:	HY-13625
CAS No.:	153773-82-1
Molecular Formula:	$C_{22}H_{24}N_3NaO_7S$
Molecular Weight:	497.5
Target:	Bacterial; Antibiotic
Pathway:	Anti-infection
Storage:	4°C, sealed storage, away from moisture<br>* In solvent : -80°C, 6 months; -20°C, 1 month (sealed storage, away from moisture)

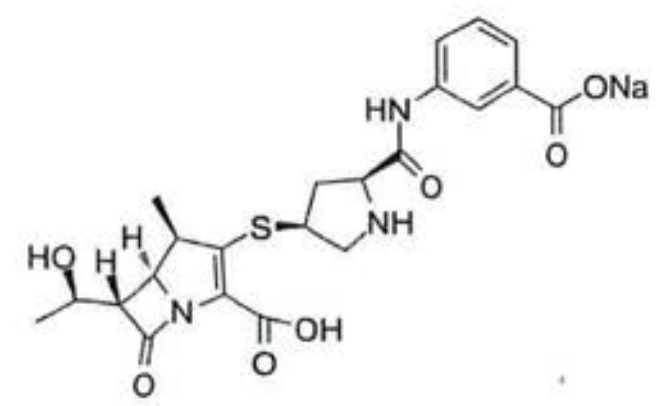

SOLVENT & SOLUBILITY

In Vitro

H_2O : 50 mg/mL (100.50 mM; Need ultrasonic)

	Solvent / Concentration / Mass	1 mg	5 mg	10 mg
Preparing Stock Solutions	1 mM	2.0101 mL	10.0503 mL	20.1005 mL
	5 mM	0.4020 mL	2.0101 mL	4.0201 mL
	10 mM	0.2010 mL	1.0050 mL	2.0101 mL

Please refer to the solubility information to select the appropriate solvent.

BIOLOGICAL ACTIVITY

Description

Ertapenem sodium (L-749345) is a broad spectrum and long acting β-lactam antibiotic. Ertapenem sodium has a broad-spectrum anti-anaerobic activity against a variety of anaerobes with a mode MIC of 0.12 μg/mL. Ertapenem sodium can be used for the research of severe infections caused by bacteria in the skin, lungs, stomach, pelvis, and urinary tract[1][2].

In Vitro

Ertapenem sodium (0-100 μg/mL approximately, 48 h) is active against 99.1% of all anaerobes with a mode MIC of 0.12 μg/mL and MIC_{90} of 1 μg/mL, and MIC's ≥8 μg/mL for B.fragilis and B.vulgatus species, respectively[1].

MCE has not independently confirmed the accuracy of these methods. They are for reference only.

Cell Viability Assay[1]

Cell Line:	B. fragilis (ATCC 25285), B. thetaiotaomicron (ATCC 29741), and Eubacterium lentum (ATCC 43055)
Concentration:	0-100 μg/mL approximately
Incubation Time:	48 h
Result:	Inhibited 99.1% of all isolate with a mode MIC of 0.12 μg/mL and MIC_{90} of 1 μg/mL, and

98.8% of the isolates were susceptible among the B. fragilis group.

In Vivo

Ertapenem sodium (Subcutaneous injection, 0-10 mg/kg, 0-120 h after infection, S. aureus thigh tissue infection model) shows > 3 log_{10} CFU reduction of organism at 10 mg/kg, and maintains the activity with 3.3 and 4.4 log_{10} CFU eliminated at 2 mg/kg[2].

Ertapenem sodium (Subcutaneous injection, 4h after infection, systemic infection model) is active against all gram-positive organisms, and is also active against gram-negative organisms tested with ED_{50}s of <0.25 mg/kg/dose[2].

MCE has not independently confirmed the accuracy of these methods. They are for reference only.

Animal Model:	S. aureus thigh tissue infection model (DBA/2 mice)[2]
Dosage:	0.5,1, 2, 5, 10 mg/kg (given at 2, 6, 10, 24, 48, 72, 96, 120 h)
Administration:	Subcutaneous injection (0.5 mL after infection)
Result:	Displayed > 3 log_{10} CFU reduction of organism compared to non-antibiotic-treated controls at 10 mg/kg.<br>Maintained the activity with 3.3 and 4.4 log_{10} CFU eliminated at 2 mg/kg.

Animal Model:	Systemic infection model (DBA/2 female mice, viral antibody-free CD-1 female mice)[2]
Dosage:	0-3 mg/kg approximately
Administration:	Subcutaneous injection (0.5 mL, begin immediately and 4 h after infection)
Result:	Showed activity against all gram-positive organisms, and also ram-negative organisms tested with ED_{50}s of <0.25 mg/kg/dose.

Animal Model:	CD-1 mice, rats[2]
Dosage:	10 mg/kg approximately
Administration:	Intraperitoneal injection (pharmacokinetic assay)
Result:	Exhibited an $AUC_{0-\infty}$ ranging from 1.8-21.82 µg•hr/mL in tissue in mice following a 10-mg/kg i.p. dose.<br>Exhibited slow clearance rate with a $t_{1/2\beta}$ of 3.2 h, Clp of 0.47 mL/min/kg, AUC_{0-8} of 284.15 µg•hr/mL.

CUSTOMER VALIDATION

- Nat Commun. 2022 Mar 2;13(1):1116.
- Nat Commun. 2021 Jul 22;12(1):4461.
- J Antimicrob Chemother. 2020 Jul 1;75(7):1850-1858.
- Biomed Res Int. 2018 Jul 2;2018:3579832.

See more customer validations on www.MedChemExpress.com

REFERENCES

[1]. Kenneth E Aldridge. Ertapenem (MK-0826), a new carbapenem: comparative in vitro activity against clinically significant anaerobes. Diagn Microbiol Infect Dis. 2002 Oct;44(2):181-6.

[2]. C J Gill, et al. In vivo activity and pharmacokinetic evaluation of a novel long-acting carbapenem antibiotic, MK-826 (L-749,345). Antimicrob Agents Chemother. 1998 Aug;42(8):1996-2001.

Caution: Product has not been fully validated for medical applications. For research use only.

Tel: 609-228-6898 Fax: 609-228-5909 E-mail: tech@MedChemExpress.com

Address: 1 Deer Park Dr, Suite Q, Monmouth Junction, NJ 08852, USA

SAMPLE INFORMATION

CAS NO.:	153773-82-1	Acquired By:	LY
Sample infromation:		Date Acquired:	8/12/2022 2:04:10 PM CST
Vial:	2:A,3	Acq. Method Set:	1_POS_2MIN
Injection #:	1	Date Processed:	8/12/2022 2:36:50 PM CST,
Injection Volume:	0.30 ul	Processing Method:	PDA_01, MS_scan
Run Time:	2.8 Minutes	Channel Name:	PDA Ch1 214nm@4.8nm, MS TIC,
Sample Set Name:	20220812	Column Name:	A-RP-985

Auto-Scaled Chromatogram

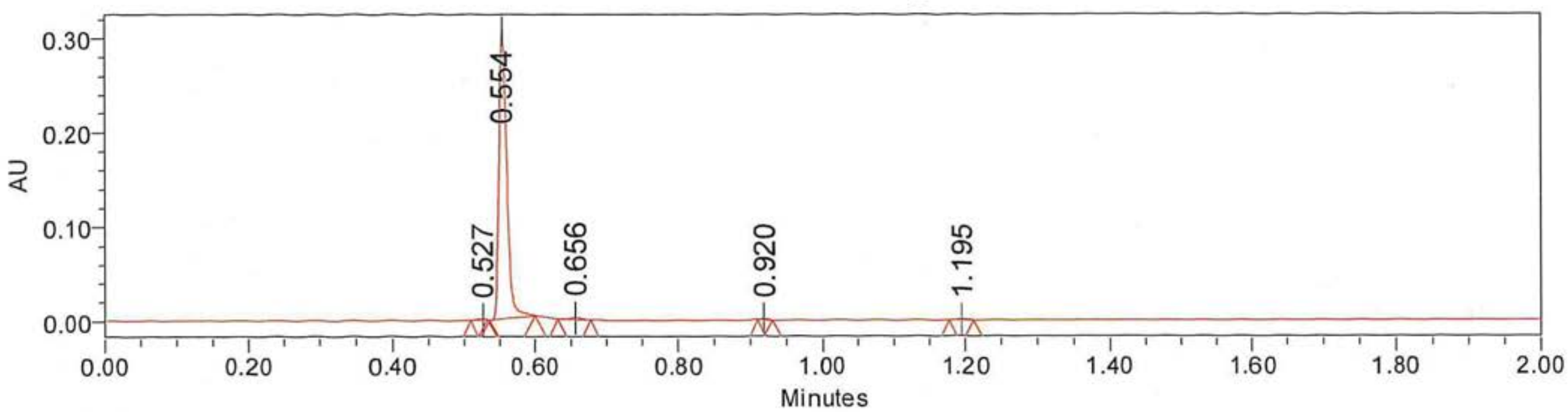

Processed Channel Descr. PDA Ch2 254nm@4.8nm

Auto-Scaled Chromatogram

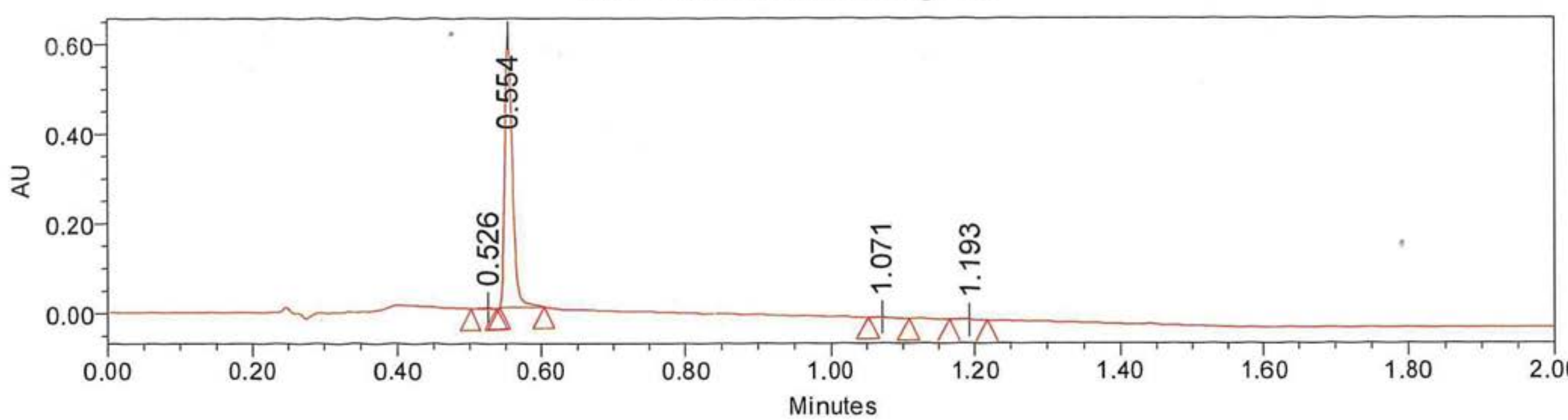

Processed Channel Descr. PDA Ch1 214nm@4.8nm

Auto-Scaled Chromatogram

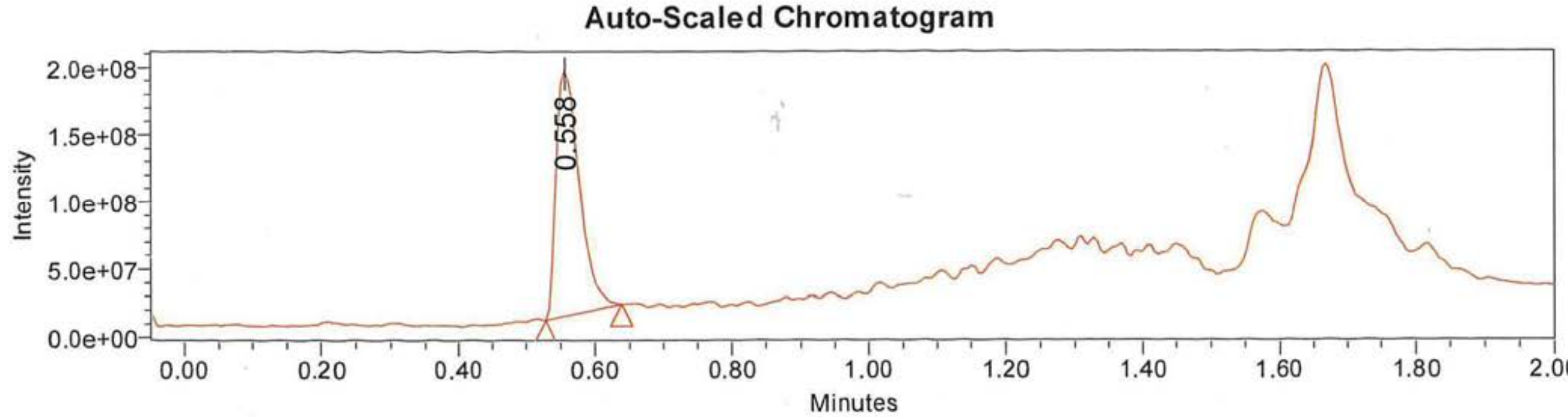

Processed Channel Descr. ACQ-SQD2 1: MS Scan MS TIC, Smoothed by 9 point Savitzky-Golay Filter., Time offset by -0.053 mins. (1: 150.00-1000.00 ES+, Centroid, CV=30)

Peak Results
Channel Name: PDA Ch2 254nm@4.8nm

	RT	Width (sec)	Area	Height	% Area	Base Peak (Combined) (m/z)	Channel Name
1	0.527	1.400	477	640	0.20	153.83	PDA Ch2 254nm@4.8nm
2	0.554	3.800	237134	308413	98.19	151.96	PDA Ch2 254nm@4.8nm
3	0.656	2.650	2446	2436	1.01	151.06	PDA Ch2 254nm@4.8nm
4	0.920	1.200	632	1080	0.26	152.07	PDA Ch2 254nm@4.8nm
5	1.195	2.050	823	941	0.34	152.07	PDA Ch2 254nm@4.8nm

Peak Results
Channel Name: PDA Ch1 214nm@4.8nm

	RT	Width (sec)	Area	Height	% Area	Base Peak (Combined) (m/z)	Channel Name
1	0.526	2.150	1796	2101	0.38	153.84	PDA Ch1 214nm@4.8nm
2	0.554	3.700	471358	613577	98.86	151.97	PDA Ch1 214nm@4.8nm
3	1.071	3.350	1629	1281	0.34	151.03	PDA Ch1 214nm@4.8nm
4	1.193	3.150	1997	1243	0.42	152.07	PDA Ch1 214nm@4.8nm

Peak Results
Channel Name: MS TIC

	RT	Width (sec)	Area	Height	% Area	Base Peak (Combined) (m/z)	Channel Name
1	0.558	6.673	431532509	182380390	100.00	476.14	MS TIC

Match Plot

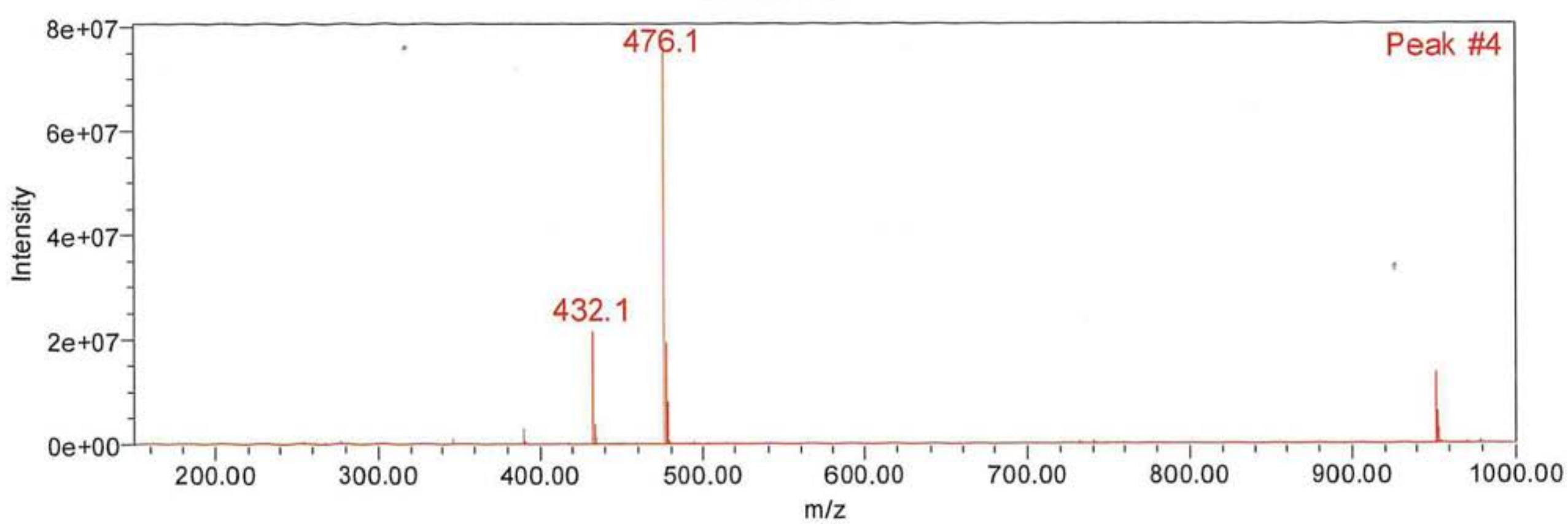

Retention Time 0.558 Channel Name MS TIC

CAS NO.: 153773-82-1, D2O

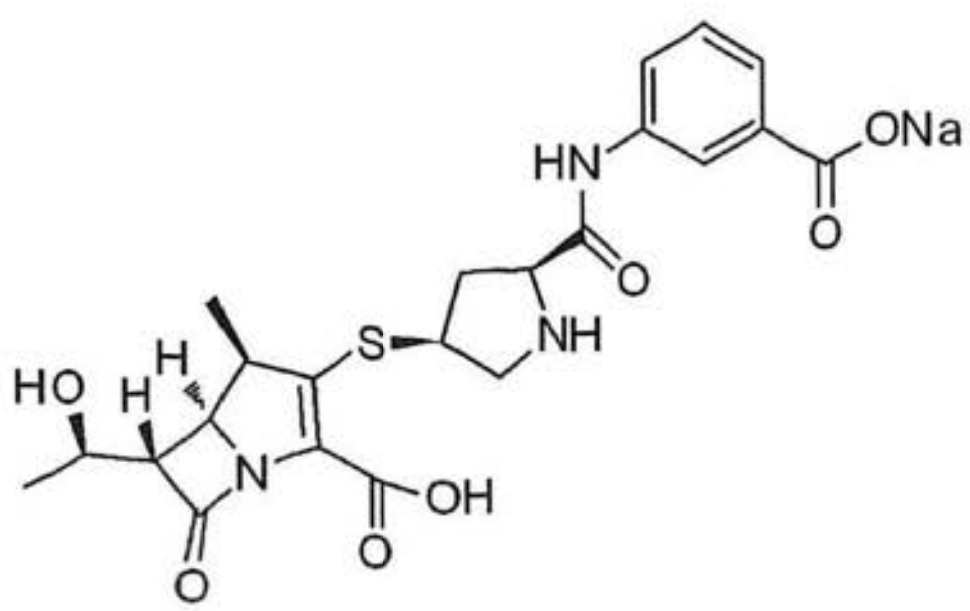

Chemical Formula: $C_{22}H_{24}N_3NaO_7S$
Exact Mass: 497.12

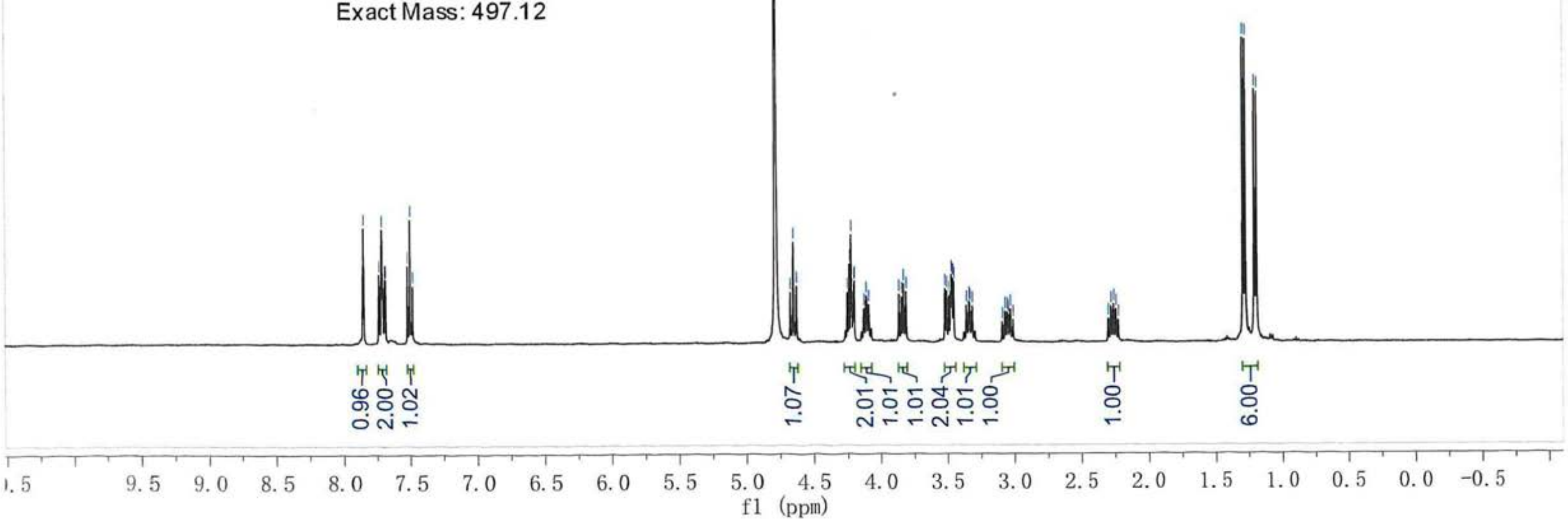

	Parameter	Value
1	Title	
2	Origin	Bruker BioSpin GmbH
3	Owner	nmrsu
4	Spectrometer	Avance
5	Author	
6	Solvent	D2O
7	Temperature	298.2
8	Pulse Sequence	zg30
9	Experiment	1D
10	Number of Scans	8
11	Receiver Gain	101
12	Relaxation Delay	1.0000
13	Pulse Width	8.0000
14	Acquisition Time	3.9977
15	Acquisition Date	2022-08-10T13:49:06
16	Modification Date	2022-08-10T13:53:00
17	Spectrometer Frequency	400.13
18	Nucleus	1H

CERTIFICATE OF ANALYSIS

ALSACHIM
a Shimadzu Group Company

**Product Name:**	[$^{2}H_{4}$]-Ertapenem sodium salt
**Molecular Formula:**	$C_{22}H_{24}N_{3}O_{7}S$. Na
**Labeled Molecular Formula:**	$C_{22}H_{20}D_{4}N_{3}O_{7}S$. Na
**Molecular Weight:**	501.52 g/mol
**Structure:**	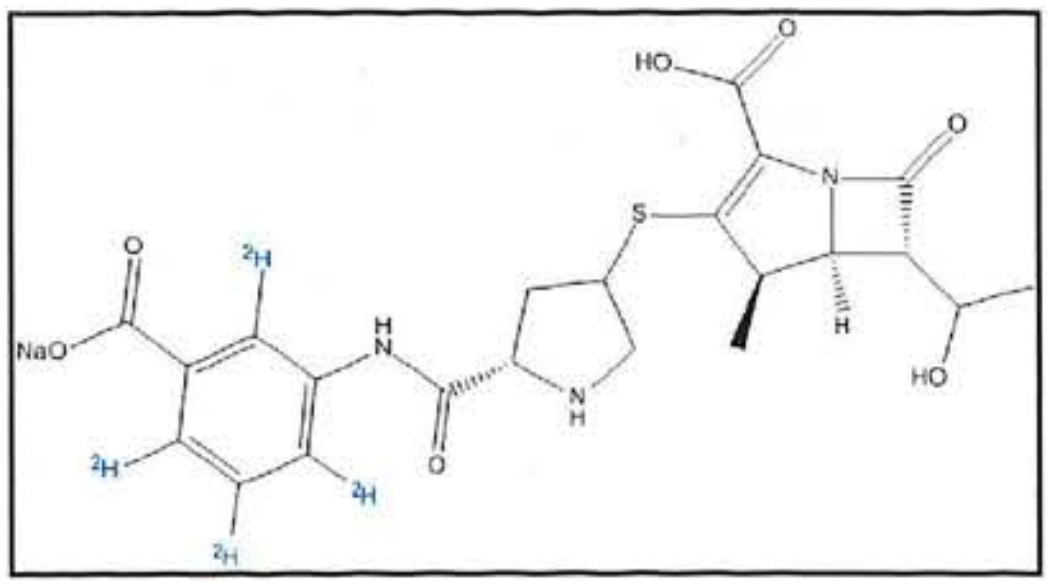

**Batch Number:**	DH-ALS-20-002-P2	
**Product Number:**	C3376	
**CAS Number:**	153773-82-1 unlabeled	
**Storage:**	For a prolonged period, keep at -20°C (±5°C) in a closed container, away from light and moisture	
**Stability:**	Stable (12 months) (*)	
**Extended expiring date:**	Saturday April 29, 2023	
**Solvent solubility:**	Soluble in Water or Acetonitrile/Water (1/1)	
	**Specification:**	**Results:**
**Appearance:**	Pale grey to white solid	Off white solid
**Chemical Purity (HPLC):**	≥ 80.0%	89,4%
**Isotopic Enrichment:**	≥ 98.0% ^{2}H	99.0% ^{2}H
**Unlabeled (M+0) Content:**	Unlabeled (M+0) content < 0.5%	<0.5%
**Mass Spectrometry:**	Conform to structure	Conforms
**Manufacturing date:**	Tuesday January 7, 2020	
**Reanalysis date:**	Friday April 29, 2022	
**Note:**	For research and development use only, not for use in humans.	

Quality Review & Approval

Francis ROESSLINGER, Quality Manager
Date : Wednesday, May 18, 2022

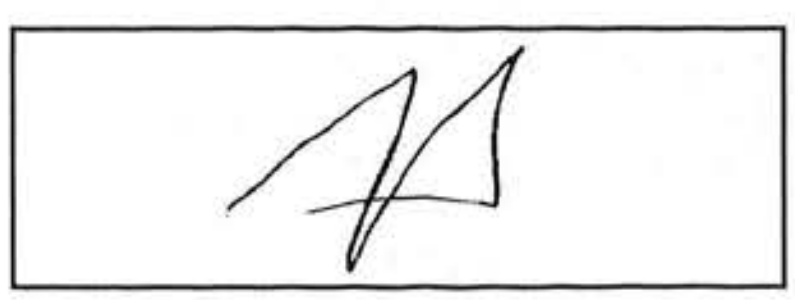

RECEIVED
SEP 21 REC'D
By

ALSACHIM
a Shimadzu Group Company

Analysis Report

<Equipment Information>

LCMS Shimadzu

Alsachim Reference: AN-LC01 + AN-MS01

Pump : LC-40D

Degassing Unit : DGU-405

Auto Sampler : SIL-40C

Diode Array Detector : SPD-M40

Column Oven : CTO-40S

Valve Unit : FCV-20AH2

Liquid Chromatograph-Mass Spectrometer : LCMS-2020

Column : Accucore C18 50*4.6 2.6um PN: 17126-054630 SN: 20333623 LOT: 20512

<Sample Information>

Sample Name : DH-ALS-20-002-P2
Data Filename : DH-ALS-20-002-P2_002.lcd
Method Filename : HP-C18-AF-20MIN.lcm
Batch Filename : 2022-APR-29-3.lcb
Vial # : 2-20
Injection Volume : 0,5 uL
Date Acquired : 29/04/2022 14:31:49
Date Processed : 02/05/2022 08:59:47
Solvent : Water
Concentration : 1 mg/ml
Operator : CD

<Peak Table >

PDA Ch1 295nm

Peak#	Ret. Time	Area	Height	Area%
1	1,861	2224	1305	0,544
2	2,364	16348	3642	4,001
3	2,684	365589	157196	89,478
4	2,940	5546	2826	1,357
5	3,046	2444	1326	0,598
6	3,202	596	403	0,146
7	3,540	15028	2285	3,678
8	3,776	806	433	0,197
Total		408582	169417	100,000

160, Rue Tobias Stimmer 67400 Illkirch

HPLC method and UV Instrument Parameters

<<Pump>>

Mode	: Binary gradient
Pump A	: LC-40D X3
Pump B	: LC-40D X3
PressMax	: 400 bar
PressMin	: 0 bar
Pump A Valve Model	: Switching Valve
Pump B Valve Model	: Switching Valve
Gradient Program:	

Pump A + B

#	Time(min) / Flow(mL/min) / B.Conc(%) / B.Curve
1	0,00/ 1,2000/ 1,0/ 0
2	9,00/ / 70,0/
3	15,00/ / 99,0/
4	19,00/ / 99,0/
5	19,10/ / 1,0/

Pump A Mobile Phase Settings : Water + 0.1% formic acid 0,45 /GPa
Pump B Mobile Phase Settings : Acetonitrile + 0.1% formic acid 1,20 /GPa

<<LC Time Program>>

Time	Module	Command	Value	Comment
20,00	Controller	Stop		

<<PDA>>

Lamp	: D2 & W
Start Wavelength	: 190 nm
End Wavelength	: 800 nm

<HPLC-UV Chromatogram >

Chromatogram

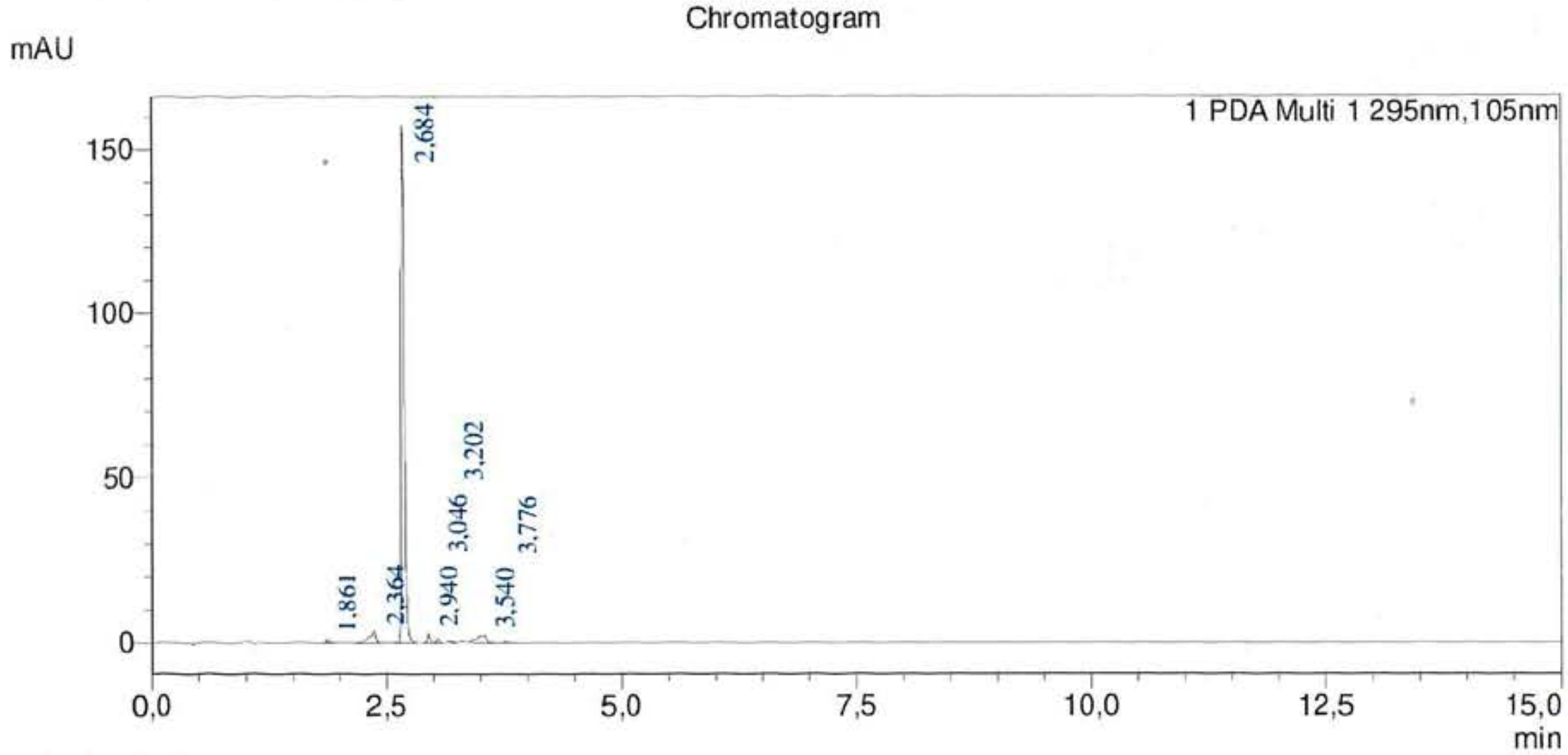

<UV Spectrum >

UV Spectrum

Peak#	: 3
Retention Time	: 2,684 min
Spectrum Operation	: None

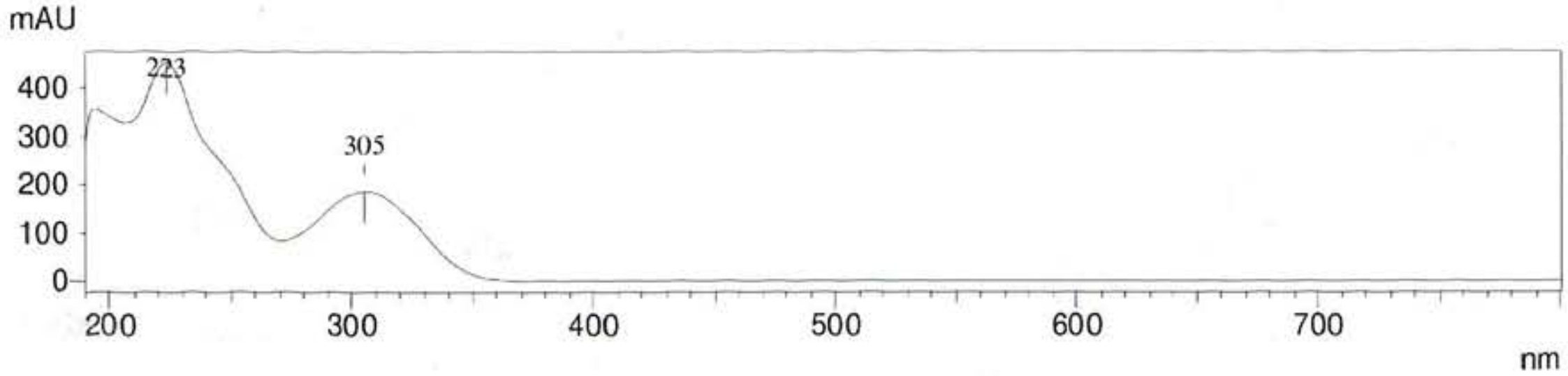

MS Instrument Parameter

<<MS Parameter>>	
Initial Valve Position	:0
--Segment 1 Event 1--	
Start Time	:0,00 min
End Time	:20,00 min
Acquisition Mode	:Scan
Polarity	:Positive
Event Time	:1,00 sec
Detector Voltage	:+1,10 kV
Threshold	:0
Start m/z	:100,00
End m/z	:1100,00
Scan Speed	:1072 u/sec
Interface Volt.	:Use the Data in the Tuning File
DL Volt.	:Use the Data in the Tuning File
Qarray DC Voltage	:Use the Data in the Tuning File
Qarray DC Voltage	:Use the Data in the Tuning File
--Segment 1 Event 2--	
Start Time	:0,00 min
End Time	:20,00 min
Acquisition Mode	:Scan
Polarity	:Negative
Event Time	:1,00 sec
Detector Voltage	:+1,10 kV
Threshold	:0
Start m/z	:100,00
End m/z	:1100,00
Scan Speed	:1072 u/sec
Interface Volt.	:Use the Data in the Tuning File
DL Volt.	:Use the Data in the Tuning File
Qarray DC Voltage	:Use the Data in the Tuning File
Qarray DC Voltage	:Use the Data in the Tuning File
<<Interface>>	
Interface	:DUIS
DL Temperature	:300 C
Nebulizing Gas Flow	:1,50 L/min
Heat Block	:400 C
Drying Gas	:On<br>18,00 L/min

Segment	Start Time	End Time	Ionization Mode
1	0,00	20,00	ESI&APCI

<HPLC-MS Chromatogram>

MS Chromatogram

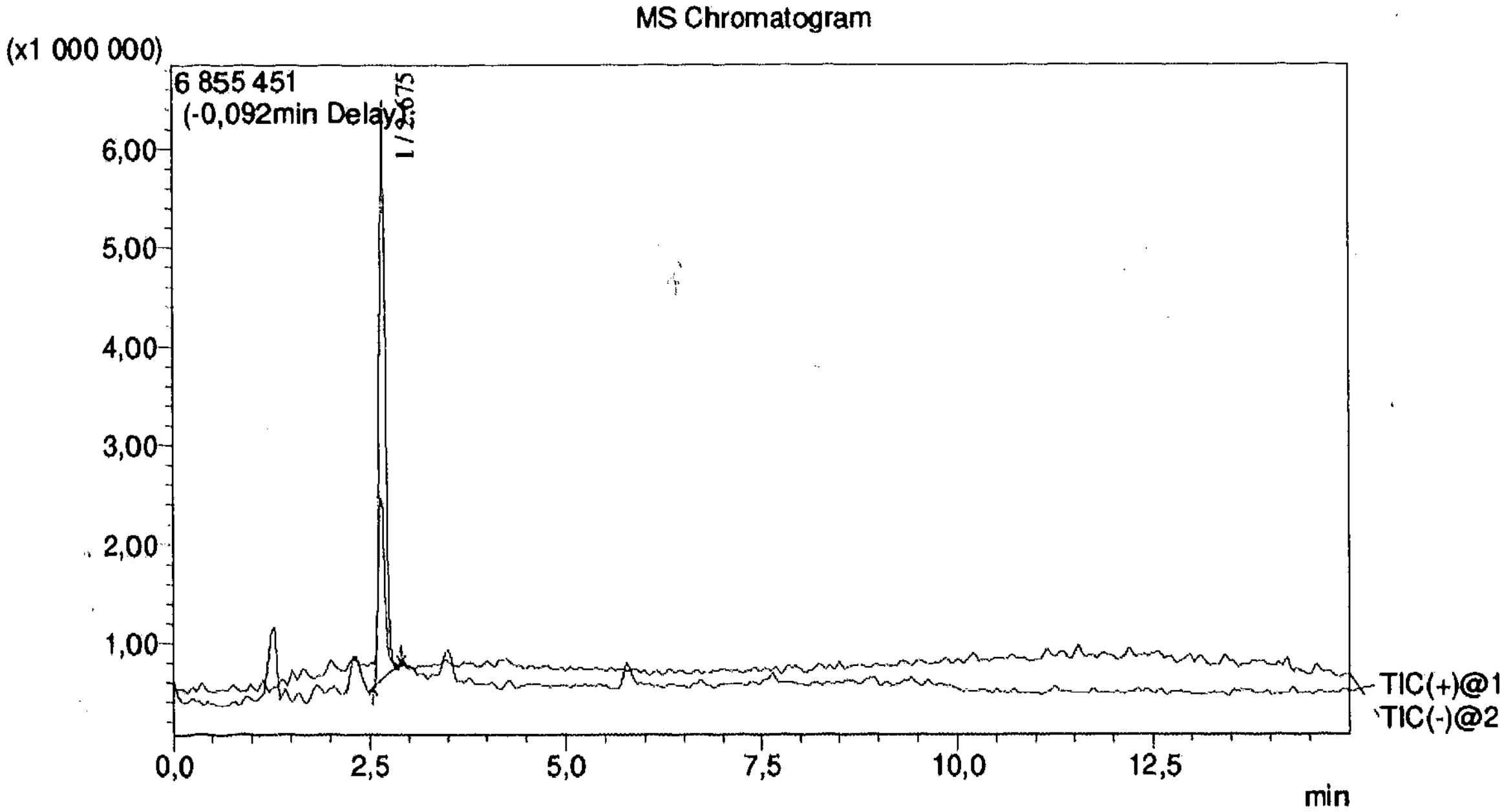

< Mass spectrum>

MS Spectrum

Peak#:1 R.Time:2,675(Scan#:167)
MassPeaks:660
Spectrum Mode:Averaged 2,641-2,708(165-169)
BG Mode:Calc Segment 1 - Event 1

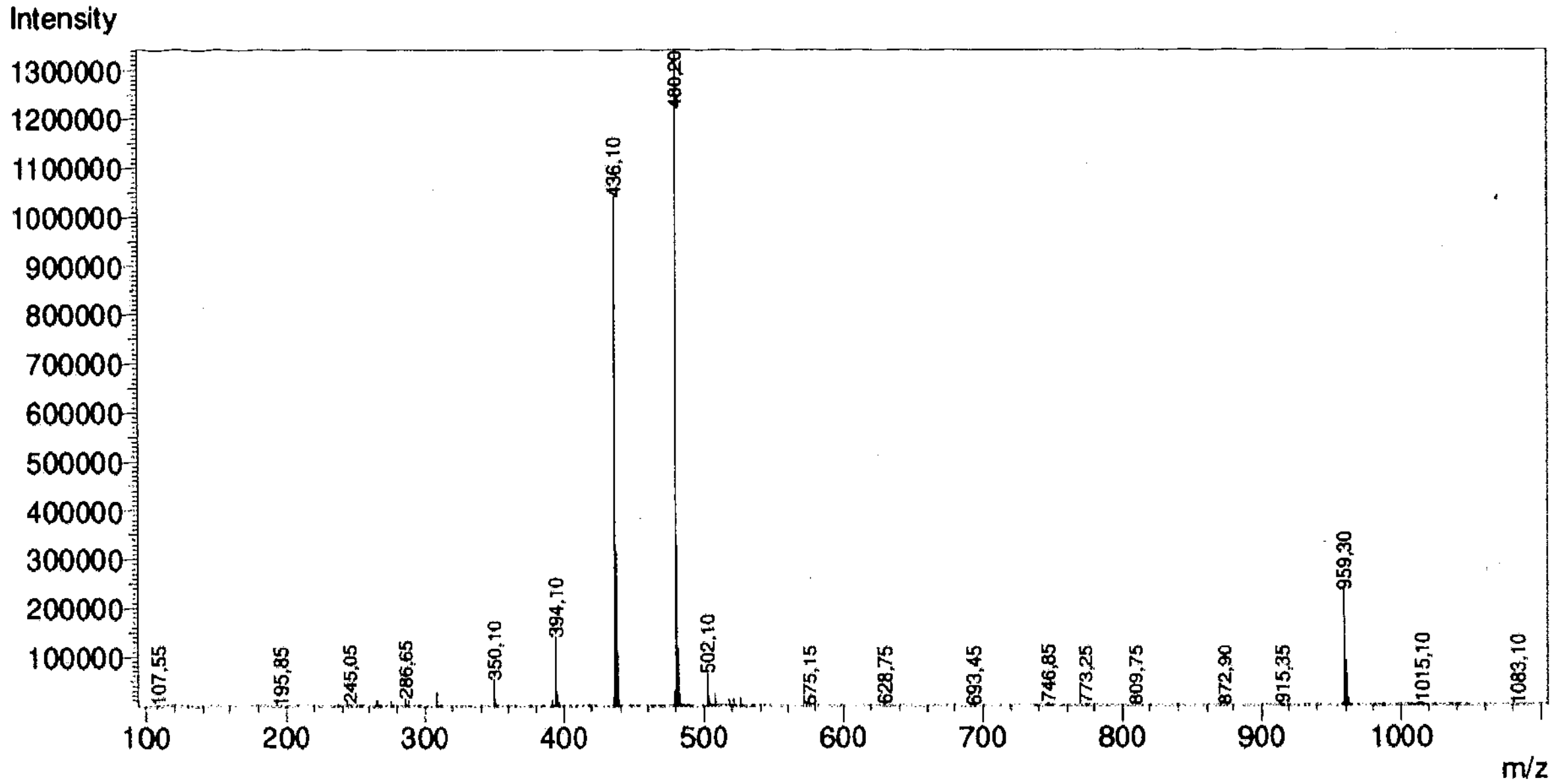

< Mass Profile Spectrum>

MS Profile Spectrum

Peak#:1 R.Time:2,675(Scan#:167)
MassPeaks:660
Spectrum Mode:Averaged 2,641-2,708(165-169)
BG Mode:Calc Segment 1 - Event 1

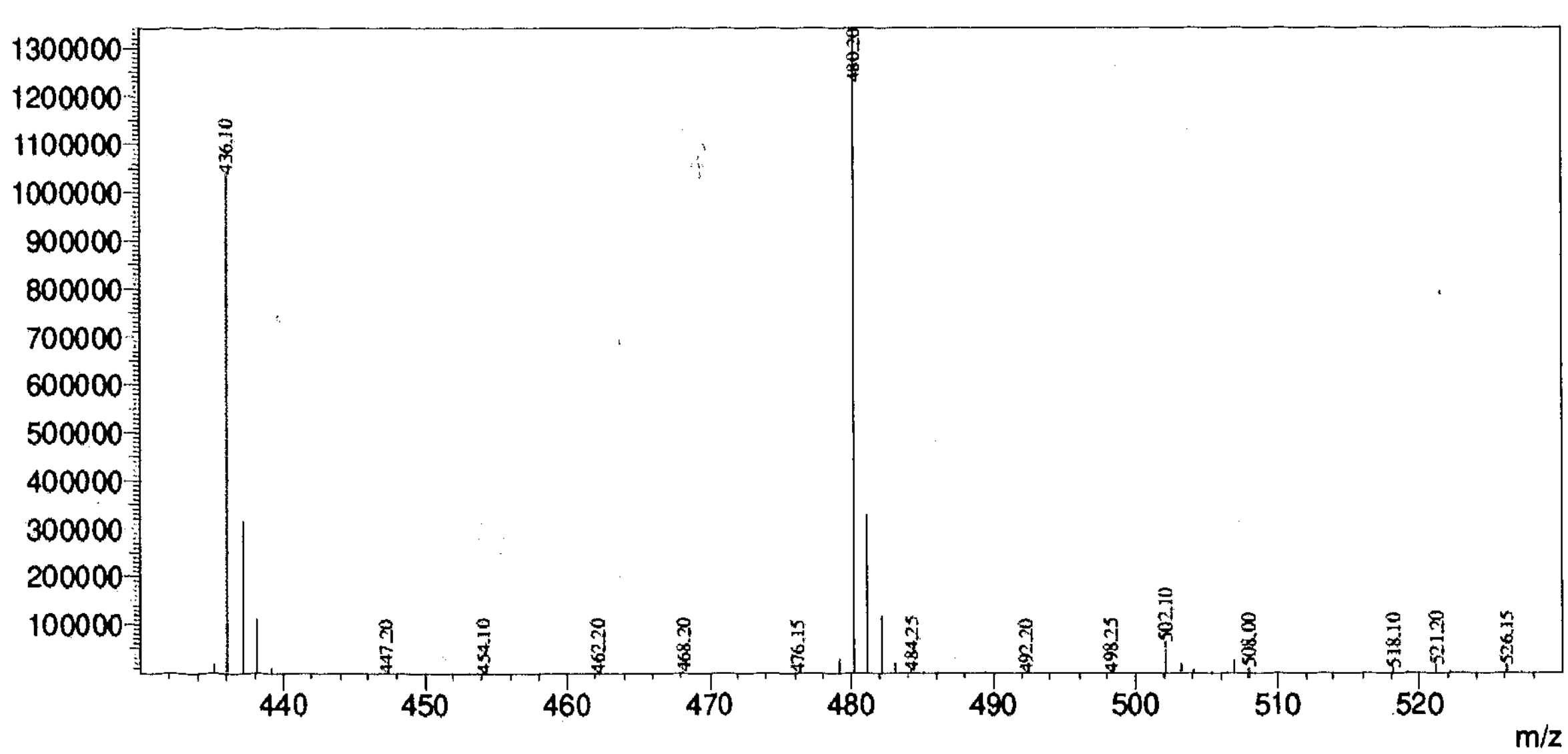

Supplement: Supplementary file 5 [file aac.00390-26-s0005.pdf]
