# Supplementary material for: A Phase I, randomized, double-blind, placebo-controlled, dose escalation study to evaluate the safety, tolerability, and pharmacokinetics of intravenous ertapenem and zidebactam in combination (WCK 6777) in healthy adult subjects
Source: Antimicrob Agents Chemother. 2026 Sep 29;70(11):e00390-26. doi: 10.1128/aac.00390-26 (PMC13731237; doi:10.1128/aac.00390-26)
Supplement: Supplementary file 6 [file aac.00390-26-s0006.pdf]

1. TITLE: THE DETERMINATION OF ZIDEBACTAM IN TREATED HUMAN URINE BY LC-MS/MS

BIOANALYTICAL METHOD VALIDATION REPORT

Bioanalytical Project Number:	V1872106U2
Sponsor:	DynPort Vaccine Company, LLC<br>110 Thomas Johnson Drive<br>Suite 200<br>Frederick, MD 21702
Bioanalytical Laboratory:	KCAS Bioanalytical & Biomarker Services.<br>10830 S Clay Blair Boulevard<br>Olathe, KS 66061
Issue Date:	November 2022

2. SYNOPSIS

This report presents the validation data for an LC-MS/MS method for the determination of zidebactam in human urine. Calibration standards and QCs are prepared in 1:1 human urine: 0.1M [2-(N-Morpholino) ethanesulphonic acid hydrate] buffer, pH 6.4 (MES buffer) and unknown samples are treated with MES buffer at the same ratio. The validation assay range is calculated based on urine volume only to mimic unknown study samples and no concentration adjustment is needed during sample analysis. The assay range is 10.0 to 4000 mcg/mL in human urine and is for use on human urine samples obtained during clinical trials.

3. TABLE OF CONTENTS FOR THE BIOANALYTICAL METHOD VALIDATION REPORT

1. TITLE: THE DETERMINATION OF ZIDEBACTAM IN TREATED HUMAN URINE BY LC-MS/MS 1
2. SYNOPSIS 2
3. TABLE OF CONTENTS FOR THE BIOANALYTICAL METHOD VALIDATION REPORT 3
4. LIST OF TABLES 4
5. LIST OF FIGURES 6
6. SIGNATURE PAGE 7
7. QUALITY ASSURANCE STATEMENT 8
8. STUDY COMPLIANCE STATEMENT 9
9. LIST OF ABBREVIATIONS AND DEFINITIONS OF TERMS 10
10. INTRODUCTION 12
11. EXPERIMENTAL DESIGN AND RESULTS 12
11.1. Reference Standards 12
11.2. Calibration Standards and QC Samples Preparation 12
11.3. Selectivity 13
11.3.1. Blank Evaluation 13
11.3.2. Matrix Effect 13
11.3.3. Interference Evaluation 14
11.3.4. Carryover 14
11.4. Linearity and Analytical Range 14
11.5. Precision and Accuracy 14
11.6. Sensitivity 15
11.7. Run Size 15
11.8. Precision and Accuracy of Dilution 15
11.9. Analyte Adsorption to Container Test 16
11.10. Freeze/Thaw Stability 16
11.11. Bench-Top Stability in Wet Ice 16

11.12. Refrigeration Stability 17
11.13. Long-Term Matrix Stability at -20 °C and -70 °C 17
11.14. Processed Sample Stability when Refrigerated 17
11.15. Individual Sample Reinjection Reproducibility 17
11.16. Whole Run Reinjection Reproducibility 18
11.17. Column Ruggedness 18
11.18. Extraction Recovery 18
11.19. Solution Stabilities 19
11.20. Calculations 19
11.21. SOP Deviations and Investigations 19
12. CONCLUSION 20
13. PERTINENT SOPS 20
14. ARCHIVES 20
15. REFERENCES 20
16. TABLES 22
17. FIGURES 59

4. LIST OF TABLES

Table 1: Abbreviations and Codes 10
Table 2: Deviations 20
Table 3: Mass Spec Transitions 22
Table 4: Reference Standards 22
Table 5: Calibration Standards and QC Samples Preparation 22
Table 6: Matrix Description 23
Table 7: Summary of Results and Conclusions 24
Table 8: Summary of Validation Run Objectives and Outcome 28
Table 9: Carryover 29
Table 10: Selectivity - Blank Assessment 30
Table 11: Matrix Effect – Low Concentration 31

Table 12: Matrix Effect – High Concentration 32
Table 13: Interference Evaluation 33
Table 14: Summary of Standard Curves 34
Table 15: Precision and Accuracy (Intra-Assay) 35
Table 16: Precision and Accuracy (Inter-Assay) 36
Table 17: Standard and QC of Failed Runs 37
Table 18: Standards and QCs of Non-P&A Runs 38
Table 19: Precision and Accuracy of Sample Dilution 39
Table 20: Analyte Adsorption Container Test 40
Table 21: Freeze-Thaw Stability, (Frozen At -20 °C and -70 °C Thawed in Wet Ice) 41
Table 22: Bench-Top Stability in Wet Ice 42
Table 23: Refrigerated Stability (Treated and Untreated) 43
Table 24: Long-Term Stability at -20 °C and -70 °C 44
Table 25: Refrigerated Processed Sample Stability 45
Table 26: Individual Sample Reinjection Reproducibility 46
Table 27: Whole Run Reinjection Reproducibility and Column Ruggedness 47
Table 28: Extraction Recovery – Zidebactam 48
Table 29: Extraction Recovery – Cefotaxime 49
Table 30: Stability of Zidebactam Primary Solution in Deionized Water at Room Temperature 50
Table 31: Stability of Zidebactam Primary Solution in Deionized Water at -20 °C 51
Table 32: Stability of Zidebactam Spiking Solution in Deionized Water at Room Temperature 53
Table 33: Stability of Zidebactam Spiking Solution in Deionized Water at -20 °C 55
Table 34: Stability of Cefotaxime Working Internal Standard Solution in Deionized Water at Room Temperature 58

5. LIST OF FIGURES

Figure 1: Typical Calibration Curve 59
Figure 2: Typical Ion Chromatogram Blank Human Urine Without Internal Standard 60
Figure 3: Typical Ion Chromatogram Blank Human Urine with Internal Standard 61
Figure 4: Typical Ion Chromatogram LLOQ Calibration Standard, Zidebactam = 10.0 mcg/mL 62
Figure 5: Typical Ion Chromatogram Calibration Standard, Zidebactam = 1600 mcg/mL 63
Figure 6: Typical Ion Chromatogram ULOQ Calibration Standard, Zidebactam = 4000 mcg/mL 64

Annex 1 .... Test Procedure
Annex 2 .... Certificate(s) of Analysis
Annex 3 .... Interference Testing Memo

6. SIGNATURE PAGE

Bioanalytical Project Number: V1872106U2

Issue Date: November 2022

APPROVED

____

Anika Pippin, B.S.
Principal Investigator

REVIEWED

____

Marsha Luna, B.S.
Management

7. QUALITY ASSURANCE STATEMENT

This Bioanalytical Method Validation Report has been found to describe accurately the methods and SOPs used and to accurately reflect the raw data collected. The Quality Assurance Unit initiated audits on this study and reported the findings to the Principal Investigator (PI) and Management as follows:

		**Date(s) Sent to:**	
**Inspection Date(s)**	**Inspection Phase**	**PI**	**Management**
06Sep2022	Data Audit	06Sep2022	
07Sep2022	Draft Report Review	07Sep2022	29Sep2022
	Updated Draft Report Review		
	Final Report Review		

REVIEWED

Laura Garrett, B.S.
Quality Assurance Unit

8. STUDY COMPLIANCE STATEMENT

I, the undersigned, hereby declare that this study was conducted in compliance with the relevant bioanalytical aspects of FDA guidelines, GLP documentation and reporting GLP regulations 21CFR Part 58 and KCAS Bioanalytical & Biomarker Services (KCAS) procedures and that the work was performed by me, or under my direction, and the report reflects a true and accurate record of the results obtained.

The acceptance criteria are based on the FDA Guidance for Industry, Bioanalytical Method Validation (2018). [1]

APPROVED

____

Anika Pippin, B.S.
Principal Investigator

9. LIST OF ABBREVIATIONS AND DEFINITIONS OF TERMS

Table 1: Abbreviations and Codes

Abbreviation	Definition
<	Concentration less than the lowest acceptable calibration standard
>	Concentration exceeded the upper limit of calibration range (> ULOQ)
A	Amendment
AQL	Above Quantitation Limit
%Bias	Percentage measurement valid
Batch	Is the same as Run
BQL	Below Quantifiable Limit
Cal	Calibration standard
Conc.	Concentration
Conv.	Conversion
%CV	Coefficient of Variation
dev	Deviation
diff	Difference
EMA	European Medicines Agency
FDA	Food and Drug Administration
GLP	Good Laboratory Practice
HPLC	High Performance Liquid Chromatography
ID	Identification
IS	Internal Standard
ISR	Incurred Sample Reanalysis
K_2EDTA or K_3EDTA	Dipotassium or Tripotassium Ethylenediaminetetraacetic Acid
KCAS	KCAS Bioanalytical & Biomarker Services
LC-MS/MS	Liquid Chromatography Tandem Mass Spectrometry
LLOQ	Lower Limit of Quantitation
mcg or μg or ug	Microgram
mcL or μL or uL	Microliter
ME	Matrix Effect

Table 1: Abbreviations and Codes (Continued)

Abbreviation	Definition
MES buffer	0.1M [2-(N-Morpholino) ethanesulphonic acid hydrate] buffer, pH 6.4
MF	Matrix Factor
MHLW	Ministry of Health, Labour and Welfare of Japan
mL	Milliliter
MS	Mass Spectrometry
n	Number
N.C.	Not Calculated
ND	No difference reported as ISR will be counted
ng	Nanogram
n.report	No Data available to report
N/A or NA	Not Applicable
N.R.	Not Reported
P&A	Precision and Accuracy
p.a.r.	Peak Area Ratio
PI	Principal Investigator
QC	Quality Control Sample
QCs	Quality Control Samples
OECD	Organization for Economic Co-operation and Development
RT	Room Temperature
sd or SD	Standard deviation
Seq	Sequence
SOP (s)	Standard Operating Procedure (s)
temp	Temperature
TP	Test procedure
UHPLC	Ultra High Performance Liquid Chromatography
ULOQ	Upper Limit of Quantitation
WIS	Working Internal Standard

This is a basic list. Some of the abbreviations may not have been used in this report.

10. INTRODUCTION

The objective of this study was to fully validate a bioanalytical method for the determination of zidebactam in treated human urine to support the Phase 1 clinical trial studying the safety, tolerability and pharmacokinetics of intravenous ertapenem in combination with zidebactam (WCK 6777) in health adult subjects. The human urine is treated with 0.1M 2-(N-Morpholino) ethane sulphonic acid hydrate (MES) buffer, pH 6.4 at a 1:1 ratio. This validation report describes the selectivity, sensitivity, linearity, accuracy, precision, stability and recovery experiments based on an established bioanalytical test procedure.

The validation procedure was designed to meet or exceed the requirements of FDA Guidance for Industry on Bioanalytical Methods Validation, May 2018. The software programs used were Microsoft Office Excel 365, acquisition and processing software Analyst 1.7.2, and data management software Watson LIMS 7.5.

11. EXPERIMENTAL DESIGN AND RESULTS

In this method, zidebactam and internal standards, cefotaxime, were released from treated human urine using protein precipitation. After dilution, the diluent was chromatographed using reversed phase HPLC with a Fortis C18 analytical column. Zidebactam and cefotaxime (IS) were detected by monitoring the precursor and product ions described in Table 3 using an Applied Biosystems Sciex API 5000 LC-MS/MS. The quantification was based on setting a calibration graph using the internal standard method based on bioanalytical test procedure TP80187, entitled “LC-MS/MS Analysis of Zidebactam in Treated Human Urine (50 mcL, 10.0 - 4000 mcg/mL in Urine only)”. The test procedure can be found in Annex 1. A decision was made to only test interference of ertapenem, a memo of this decision can be found in Annex 3.

A summary of the validation results is provided in Table 7. A summary describing the objective(s) and outcome of each validation run attempted is provided in Table 8.

The method was validated within the analytical range of 10.0 - 4000 mcg/mL in human urine. The validation assay range is calculated based on urine volume only to mimic unknown study samples and no concentration adjustment is needed during sample analysis. No development work was performed after the method was validated.

11.1. Reference Standards

Reference standards used during this validation are presented in Table 4.

11.2. Calibration Standards and QC Samples Preparation

Calibration standards and quality control pools were prepared by spiking appropriate levels of zidebactam into human urine treated with MES buffer at a 1:1 ratio. Standards and QCs were prepared and stored frozen at KCAS at -70 °C. Stability QCs were also stored at -20 °C.

Standards, QCs, and the critical solutions used during this validation are described in Table 5, and matrix used is described in Table 6.

11.3. Selectivity

Selectivity is the ability of the bioanalytical method to measure and differentiate zidebactam in the presence of components that may be expected to be present. These could include metabolites, impurities, degradants, or matrix components. The selectivity of this method was achieved by a sample clean-up procedure using dilution followed by chromatography and specific detection of the precursor and product ions of each analyte using tandem mass spectrometry (MS/MS).

11.3.1. Blank Evaluation

A minimum of six different sources of urine were screened. To be deemed acceptable, the peak response observed must be ≤ 20.0% of the mean response of the lower limit of quantitation (LLOQ) and ≤ 5.0% of the mean response of the internal standard. If any lots fail, further evaluation is needed.

All matrices lots were found to be free of any significant interference at the retention times of the analytes of interest. Data is presented in Table 10. Typical chromatograms for blank urine without and with internal standard are presented in Figure 2 and Figure 3, respectively. .

11.3.2. Matrix Effect

Matrix effect in MS applications is defined as inter-matrix variation in signal due to co-eluting matrix substances which cause enhancement or suppression of the detector response for the measured analyte.

To evaluate matrix effect using matrix factor, zidebactam and cefotaxime (IS) were spiked in four replicates into six different lots of diluted human urine. Zidebactam and cefotaxime (IS) were also spiked in four replicates into a proper extracted solvent or neat solution for comparison.

For zidebactam and cefotaxime (IS), the matrix factor (MF) was calculated for each lot of human urine using the following equation:

$$\text{MF}= \frac{\text{Peak Response in Presence of Matrix Substances}}{\text{Mean Peak Response in Absence of Matrix}}$$

The IS normalized MF was calculated using the following equation:

$$\text{IS Normalized MF}= \frac{\text{MF}_{\text{Analyte}}}{\text{MF}_{\text{IS}}}$$

The evaluation was conducted for zidebactam at a high (close to ULOQ) and a low concentration (maximum of three times the LLOQ) and then the MF and IS Normalized MF were calculated

for each lot of matrix. To be deemed acceptable for matrix effect test, the %CV for IS Normalized MF must be ≤ 15.0% over all six lots.

Matrix effect data are presented in Table 11 to Table 12. The matrix effect results meet acceptance criteria.

11.3.3. Interference Evaluation

Assay selectivity was confirmed by spiking quality control pools with 3000 mcg/mL (based on urine volume only) of ertapenem. These QC pools, containing low and high level of zidebactam, were analyzed in six replicates in a single run. To be considered acceptable, the mean %Bias at each level was required to be within ± 15.0% and the %CV was required to be ≤ 15.0%.

The results presented in Table 13, indicate the presence of 3000 mcg/mL (based on urine volume only) of ertapenem does not affect the accuracy or precision of the data.

11.3.4. Carryover

Carryover is evaluated within each run. It consists of measuring the response for each analyte and if it exceeds 20.0% of the response of LLOQ standard or other sample reflecting the lower sensitivity limit required, a carryover assessment is completed, and affected samples are rejected. The internal standard carryover evaluation is assessed within each run and if it exceeds 5.0% of the average internal standard response of acceptable standards and QCs, impact is evaluated within the run. Data are presented in Table 9.

11.4. Linearity and Analytical Range

The calibration curve was constructed with eight different, non-zero standard concentrations ranging from 10.0 - 4000 mcg/mL in human urine. Calibration standards were processed in duplicate and the calibration curve was evaluated using least squares linear regression with weighting $1/x^2$. The maximum allowable percent deviation from nominal for each standard was ± 15.0%, except at the LLOQ, where ± 20.0% was accepted. Any standard deviating by more than these limits was required to be excluded from the regression line. For the calibration curve to be accepted, at least 75% of the initial number of standards was required to remain in the curve.

The calibration curve was found to be linear. The data are presented in Table 14. A typical calibration curve is presented graphically in Figure 1. Typical chromatograms illustrating blank human urine standards spiked with zidebactam at the LLOQ, mid, and high concentrations are presented in Figure 4 to Figure 6, respectively.

11.5. Precision and Accuracy

The precision of an assay is defined as the closeness in which replicate analyses agree and is reported as percent coefficient of variation (%CV). The accuracy of a method is defined as the

closeness of a determined value to its true (nominal) value and is reported as %Bias. At least one precision and accuracy run was performed using standards prepared from one primary stock and the QCs were prepared from a separate primary stock. Inter-assay (between runs) precision and accuracy and intra-assay (within runs) precision and accuracy were assessed in three runs by analyzing six replicates each of QCs fortified at four separate concentrations (LLOQ, low, mid, and high). For inter- and intra-assay, the mean %Bias at each QC level was required to be within ±15.0% from its respective nominal concentration, except at the LLOQ, where ± 20.0% was accepted. The %CV at each QC level was required to be ≤ 15.0%, except at the LLOQ, where ≤ 20.0% was accepted.

The intra-assay and inter-assay precision and accuracy data for this method met the required criteria and are presented in Table 15 (intra-assay precision and accuracy) and Table 16 (inter-assay precision and accuracy).

Standards and QCs of non-P&A runs are reported in Table 18.

11.6. Sensitivity

The sensitivity of the method was determined in three runs by analyzing six replicates of QCs at the lower limit of quantitation (LLOQ) which were equal in concentration to the lowest calibration standard. The observed response at the LLOQ was required to be at least 5 times the response of noise in the human urine blank, in addition to the precision and accuracy requirements outlined in the previous section.

The LLOQ for this method was determined to be 10.0 mcg/mL, and the response at the LLOQ was at least 5 times the response of noise in the human matrix blank. Data are presented in Table 15 (intra-assay precision and accuracy) and Table 16 (inter-assay precision and accuracy).

11.7. Run Size

In order to evaluate the largest size run that can be used for study sample analysis, a run was processed with a set of 8 calibration standards and a minimum of 3 QC samples, processed in duplicate. In addition, at least 3 matrix blanks (one with internal standard and two without internal standard) were processed with the run. Run 6 was used for this assessment, and additional experiments were also analyzed which made the final run size 164.

In order for the run to be acceptable, at least one QC at each concentration level and two-thirds of the total number of QC samples were required to interpolate within ± 15.0% from nominal.

The standard and QC summary of run 6 is presented in Table 14 and Table 15 and acceptance criteria was met.

11.8. Precision and Accuracy of Dilution

To determine the accuracy of dilution, the high concentration QC spiked with zidebactam (3000 mcg/mL) as well as an above-range QC (ARQC; 16000 mcg/mL) were evaluated. All samples

were extracted in six replicates according to the test procedure. To be deemed acceptable, the mean %Bias at each level must be within ± 15.0% with %CV ≤ 15.0%. The replicates were prepared as follows:

- Dilution factor 2x (high QC) = 25 mcL sample + 25 mcL blank treated human urine
- Dilution factor 5x (ARQC) = 10 mcL sample + 40 mcL blank treated human urine

Dilution precision and accuracy, using dilution factors of 2x and 5x, were proven to be valid. Data are presented in Table 19.

11.9. Analyte Adsorption to Container Test

Adsorption of zidebactam in urine to various container types was assessed by using low and high concentration QC samples (treated and untreated urine) aliquotted into 2 different container types (high density polyethylene (HDPE) and polypropylene) and stored in each container at room temperature prior to being transferred to the next container of the same type. Upon completion, the samples were extracted. The untreated urine stability samples were treated with MES buffer at a 1:1 ratio prior to extraction. They were assayed along with a qualified set of QCs and calibration standards. To be deemed stable, the mean %Bias at each level must be within ± 15.0% with %CV ≤ 15.0%. There appears to be no significant adsorption effect in any type of container tested. Data are provided in Table 20.

11.10. Freeze/Thaw Stability

Zidebactam stability in human urine with and without the presence of interference[2] during sequential freeze/thaw cycles (frozen separately at -20 °C and -70 °C and thawed unassisted in wet ice) was evaluated. Aliquots of high and low concentration QC samples were frozen for at least 12 hours before the initial thawing. They were re-frozen 3 more times for at least 12 hours each time and were thawed prior to assay along with a freshly prepared set of QCs and calibration standards. To be deemed stable, the mean %Bias at each level must be within ± 15.0% with %CV ≤ 15.0%.

Zidebactam in human urine with and without the presence of interference[2] was proven stable over 4 cycles of freeze (-20 °C and -70 °C) and thaw (wet ice). Data are provided in Table 21.

11.11. Bench-Top Stability in Wet Ice

To evaluate zidebactam in human urine with and without the presence of interference[2] stability during sample processing on wet ice, aliquots of the high and low concentration QC pools were allowed to thaw at ambient conditions for 6 hours and 24 hours , prior to analysis along with a freshly prepared set of QCs and calibration standards. To be deemed stable, the mean %Bias at each level must be within ± 15.0% with %CV ≤ 15.0%.

Zidebactam in human urine with and without the presence of interference[2] was proven stable on wet ice under normal laboratory lighting over a period of 24 hours. Data are provided in Table 22.

11.12. Refrigeration Stability

To evaluate zidebactam in human urine stability (treated and untreated) during sample processing at refrigerated temperatures, aliquots of the high and low concentration QC pools (treated and untreated) were stored at refrigerated temperatures for 18.75 hours, prior to analysis along with a qualified set of QCs and calibration standards. To be deemed stable, the mean %Bias at each level must be within ± 15.0% with %CV ≤ 15.0%.

Zidebactam in human urine (treated and untreated) was proven stable at refrigerated temperatures over a period of 18.75 hours. Data are provided in Table 23.

11.13. Long-Term Matrix Stability at -20 °C and -70 °C

To evaluate zidebactam in human urine with and without the presence of interference[2] stability during sample storage, aliquots of the high and low concentration QC pools were stored frozen at -20 °C and -70 °C nominally for 70 days prior to being analyzed, along with freshly prepared standards and QC samples. To be deemed stable, the mean %Bias at each level must be within ± 15.0% with %CV ≤ 15.0%.

Run 13 did not meet acceptance criteria, therefore, long-term stability could not be evaluated and was repeated in run 15. Zidebactam in human urine with and without the presence of interference[2] was proven to be stable at -20 °C and -70 °C over a period of 70 days in polypropylene tubes. Data are provided in Table 24.

11.14. Processed Sample Stability when Refrigerated

To evaluate zidebactam stability in processed samples at refrigerated temperatures, high and low concentration QC pools were processed, placed into polypropylene 96-well plates, and stored at refrigerated temperatures before being chromatographed with a subsequent run. To be deemed stable, the mean %Bias at each level must be within ± 15.0% with %CV ≤ 15.0%.

In run 6 refrigerated processed sample stability failed at both levels for 5 days and 21 hours. Refrigerated processed sample stability was repeated in run 16 and met acceptance criteria for 3 days 23 hours. Data are provided in Table 25.

11.15. Individual Sample Reinjection Reproducibility

To evaluate individual sample reinjection reproducibility, low and high QC extracts (6 each) injected during the run were reinjected at the end of the run, within the established stability time window of processed samples in the refrigerator. To be deemed reproducible, at least four out of

six QCs at each level must interpolate with dev [%] within ± 15.0% from nominal, and the mean %Bias at each level must be within ± 15.0% with %CV ≤ 15.0%.

Reinjection of individual samples can be made with adequate precision and accuracy. Data are provided in Table 26.

In run 2 low QCs did not meet acceptance criteria for individual reinjection and were repeated in run 6. The acceptance criteria were met in run 6.

11.16. Whole Run Reinjection Reproducibility

To evaluate whole run reinjection reproducibility, run 6 was reinjected as run 11 on the same MS platform using a different chromatographic column from the same manufacturer, with the same specification as the original within 20 days following the original injection. To be deemed acceptable, the reinjected run was required to meet the acceptance criteria outlined in Sections 11.4, 11.5, and 11.6.

It has been demonstrated that entire runs can be reinjected with adequate accuracy and precision. Data are presented in Table 27.

11.17. Column Ruggedness

Column ruggedness was assessed by reinjecting run 6 as run 11 on the same MS platform (Applied Biosystems API 5000) using a different chromatographic column from the same manufacturer, with the same specification as the original.

Data were required to meet the precision and accuracy criteria outlined in Sections 11.4, 11.5, and 11.6 to be considered as reproducible/rugged method. Column ruggedness met acceptance criteria. Data are presented in Table 27.

11.18. Extraction Recovery

To evaluate the extraction recovery, the low, mid, and high extracted QC samples were compared to extracted human urine blanks to which an equal amount of zidebactam at each level, assuming 100% recovery, was added in six replicates.

To evaluate the extraction recovery of cefotaxime (IS), zidebactam was added only for tracking during analysis. Cefotaxime (IS) for the control was added after extraction, while the same amount of cefotaxime (IS) for the extracted QC was added before extraction.

$$\% \text{ Recovery} = \frac{\text{Response ratio of "Extracted QC"}}{\text{Average Response Ratio of Control}} \times 100$$

The mean recovery of zidebactam was determined to be consistent, precise and reproducible at 53.1%.

The recovery of cefotaxime (IS) was determined to be consistent, precise and reproducible at 110.9%. Data are presented in Table 28 to Table 29.

11.19. Solution Stabilities

The stability of various solutions of zidebactam in various solvents used during the sample analysis following storage at various temperatures was determined.

Data showing the zidebactam(s), solvent composition, storage term, and temperature are presented in Table 30 to Table 33.

11.20. Calculations

The peak areas of zidebactam and cefotaxime were integrated by Analyst 1.7.2 (Applied Biosystems; Foster City, CA, USA). The data results were imported electronically into Watson LIMS 7.5 for the calculation of zidebactam concentrations and statistical analysis. The calibration curves were obtained by a weighted (1/concentration^2) least squares linear regression. After the best fit from data, the concentrations (mcg/mL) were calculated based on the measured peak area ratios of zidebactam/cefotaxime using the following equation:

Response = Slope * Conc + Intercept

$$\text{Mean Value }(\overline{X})=\frac{\sum_{i=1}^{n}\text{individual data }(X)}{\text{number of data points }(n)}$$

$$\text{Standard Deviation (SD)}=\sqrt{\frac{\sum_{i=1}^{n}\left[\text{individual data }(X)\text{-Mean Value }(\overline{X})\right]^2}{n-1}}$$

$$\text{Coefficient of Variation }(\%CV)=\left[\frac{\text{Standard Deviation (SD)}}{\text{Mean Value }(\overline{X})}\right]\times 100$$

$$\%\text{Bias}=\left(\frac{\text{Calculated Mean of the Observed Values or Observed Value-Nominal Value}}{\text{Nominal Value}}\right)\times 100$$

All calibration standards and QCs concentrations were reported to three significant figures, and study samples will be reported to three significant figures.

11.21. SOP Deviations and Investigations

One SOP deviation occurred. No investigations were conducted during this study.

Table 2: Deviations

Deviation #	Description	Impact
DEV-2022-0120	Solution pages 21-30 were inadvertently printed twice, and pages 21-28 were used for different preparations. All 8 have different preparation dates and solution types and data can be easily separated.	There is no impact.

12. CONCLUSION

Validation results obtained in this study indicate that a robust and reliable method has been established for the measurement of zidebactam in human urine by LC-MS/MS in the concentration range of 10.0 - 4000 mcg/mL. The generally recommended acceptance criteria, FDA Guidance for Industry on Bioanalytical Methods Validation, May 2018, in terms of precision, accuracy, selectivity including matrix effect, recovery and all types of stability have been met. Thus, this validated method is deemed suitable for determination of zidebactam in human urine for unknown samples.

13. PERTINENT SOPS

The following SOPs were followed during the conduct of this study:

SOP-00073 [02] - Bioanalytical Method Validation - Chromatographic Assays

SOP-00035 [00] - Procedures, Reporting, and Acceptance Criteria for Bioanalytical Chromatographic Projects

14. ARCHIVES

All raw data, including paper and electronic files associated with this study will remain archived at a location specified by KCAS, 10830 S Clay Blair Boulevard, Olathe, Kansas 66061 for a period of at least 10 years, after which the client may be contacted to arrange for continued storage of the files, or disposition according to an agreed procedure. KCAS will maintain an electronic (scanned/true) copy of the method validation report that can be viewed and printed on site. The study plan, documentation, raw data, and final report are indexed under Bioanalytical Project Number V1872106U2.

15. REFERENCES

1. Guidance for Industry: Bioanalytical Method Validation. U.S. Department of Health and Human Services. Food and Drug Administration. Center for Drug Evaluation and Research. Center for Veterinary Medicine. May 2018.

2. Interference: QCs in the presence of 3000 mcg/mL (based on urine volume only) of ertapenem.

16. TABLES

Table 3: Mass Spec Transitions

Analyte	Transition
Zidebactam	m/z 392.028→312.000
Cefotaxime (IS)	m/z 456.200→324.000

Table 4: Reference Standards

Analyte	Supplier	Lot/Batch Number	Purity	Expiration Date or Retest Date	Conversion Factor
Zidebactam	Performa	WK1/WP-00160/016	90.4%	Expire 29 Mar 2023	1.000
Cefotaxime Sodium Salt (IS)	Sigma-Aldrich	0000146942	95.9%	Retest 30 Sep 2023	1.048
Ertapenem	MedChem Express	155576	98.9%	Retest 20 Jun 2024	1.046

Certificates of analysis for the above reference standards are presented in Annex 2.

Table 5: Calibration Standards and QC Samples Preparation

Critical Solutions	Lot # for Standards (Prep Date)	Lot # for QCs (Prep Date)	Standards Lot (Prep Date)	QCS Lot (Prep Date)	Runs used
Treated Human Urine	In House V1872106U2-17-1		V1872106U2-10-1 Set A (22 Jul 2022)	V1872106U2-11-1 Set A (22 Jul 2022)	2 QCs only: 4, 5, 13 (LTS), 15 (LTS)
Deionized water stored at -20 °C in glass	V1872106U2-1-1 (21 Jul 2022)	V1872106U2-2-1 (21 Jul 2022			
Deionized water stored at -20 °C in polypropylene	V1872106U2-3-1 (21 Jul 2022)	V1872106U2-4-1 (21 Jul 2022)			
Treated Human Urine	In House V1872106U2-21-2		V1872106U2-21-1 Set B (25 Jul 2022)	N/A	4
Deionized water stored at -20 °C in glass	V1872106U2-1-1 (21 Jul 2022)	N/A			
Deionized water stored at -20 °C in polypropylene	V1872106U2-3-1 (21 Jul 2022)	N/A			
Treated Human Urine	In House V1872106U2-21-2		V1872106U2-23-1 Set C (28 Jul 2022)	V1872106U2-24-1 Set B (28 Jul 2022)	6, 7, 10
Deionized water stored at -20 °C in glass	V1872106U2-1-1 (21 Jul 2022)	V1872106U2-2-1 (21 Jul 2022			
Deionized water stored at -20 °C in polypropylene	V1872106U2-3-1 (21 Jul 2022)	V1872106U2-4-1 (21 Jul 2022)			
Treated Human Urine	In House V1872106U2-17-1		NA	V1872106U2-12-1 Set A (22 Jul 2022)	2, 13 (LTS), 15 (LTS) (Interference QCs)
Ertapenem in deionized water stored at -20 °C in glass	NA	V1872106U1-2-1 (21 Jul 2022)			
Deionized water stored at -20 °C in glass		V1872106U2-2-1 (21 Jul 2022			
Deionized water stored at -20 °C in polypropylene	NA	V1872106U2-4-1 (21 Jul 2022)			
Treated Human Urine	In House V1872106U2-21-2		V1872106U2-39-1 Set D (21 Sep 2022)	V1872106U2-40-1 Set C (21 Sep 2022)	13
Deionized water stored at -20 °C in glass	V1872106U2-31-1 (20 Sep 2022)	V1872106U2-32-1 (20 Sep 2022)			
Deionized water stored at -20 °C in polypropylene	V1872106U2-33-1 (20 Sep 2022)	V1872106U2-34-1 (20 Sep 2022)			
Treated Human Urine	In House V1872106U2-43-2		V1872106U2-43-1 Set E (30 Sep 2022)	V1872106U2-44-1 Set D (30 Sep 2022)	15, 16
Deionized water stored at -20 °C in glass	V1872106U2-31-1 (20 Sep 2022)	V1872106U2-32-1 (20 Sep 2022)			
Deionized water stored at -20 °C in polypropylene	V1872106U2-33-1 (20 Sep 2022)	V1872106U2-34-1 (20 Sep 2022) V1872106U2-41-1 (26 Sep 2022)			

Table 6: Matrix Description

Matrix	Source Lot #	Date Collected
Treated Human Urine V1872106U2-17-1	In house Lot 05-06-22U01	06 May 2022
Treated Human Urine V1872106U2-21-2	In house Lot 072522U01	25 Jul 2022
Treated Human Urine V1872106U2-43-2	BioChemed Lot BC41992HU-12	Aug 2022

Table 7: Summary of Results and Conclusions

Methodology – TP80187	Method Performance – LC-MS/MS	Hyperlink – N/A	Comments
Matrix	Urine		
Anticoagulant	N/A		
Assay Volume	50 mcL		
**Selectivity**			
Analyte	Zidebactam	Summary tables<br>Table 10<br><br>Report text<br>Section 11.3.1	
Internal Standard	Cefotaxime	Summary tables<br>Table 10<br><br>Report text<br>Section 11.3.1	
Matrix Effect<br>(6 spiked matrix lots)	Complies	Summary tables<br>Table 11,Table 12<br><br>Report text<br>Section 11.3.2	
Interference[2]	Complies	Summary tables<br>Table 13<br><br>Report text<br>Section 11.3.3	
Carryover	Assessed with every run with standards and QCs	Summary tables<br>Table 9<br><br>Report text<br>Section 11.3.4	
**Linearity**			
Weighting	$1/x^2$	Report text<br>Section 11.4	
Bias at LLOQ	1.0%	Summary tables<br>Table 14<br><br>Report text<br>Section 11.4	
Bias above LLOQ	-2.0 to 1.6%	Summary tables<br>Table 14<br><br>Report text<br>Section 11.4	
Bioanalytical Range	Complies<br>10.0 - 4000 mcg/mL	Summary tables<br>Table 14<br><br>Report text<br>Section 11.4	

Table 7: Summary of Results and Conclusions (Continued)

Methodology – TP80187	Method Performance – LC-MS/MS	Hyperlink – N/A	Comments
**Precision**			
Intra-assay	2.6 to 3.8% (LLOQ);<br>1.5 to 4.3% (above LLOQ)	Summary tables<br>Table 15<br><br>Report text<br>Section 11.5	
Inter-assay	3.5% (LLOQ);<br>2.5 to 5.0% (above LLOQ)	Summary tables<br>Table 16<br><br>Report text<br>Section 11.5	
**Accuracy**			
Intra-assay	-0.4 to 3.0% (LLOQ);<br>-7.0 to 4.3% (above LLOQ)	Summary tables<br>Table 15<br><br>Report text<br>Section 11.5	
Inter-assay	1.0% (LLOQ);<br>-2.0 to 3.3% (above LLOQ)	Summary tables<br>Table 16<br><br>Report text<br>Section 11.5	
**Large Run Size**	Determined to be 164 samples.	Summary tables<br>Table 14 and Table 15<br><br>Report text<br>Section 11.7	
**Dilution**			
Dilution factor: 2x	Dilution QC: 3000 µg/mL<br>Precision: 1.9%<br>Accuracy: 3.3%	Summary tables<br>Table 19<br><br>Report text<br>Section 11.8	
Dilution factor: 5x	Dilution QC: 16000 µg/mL<br>Precision: 12.0%<br>Accuracy: -5.0%	Summary tables<br>Table 19<br><br>Report text<br>Section 11.8	
**Adsorption**	No significant loss after 24 hours in either polypropylene or high density polyethylene	Summary tables<br>Table 20<br><br>Report text<br>Section 11.9	

Table 7: Summary of Results and Conclusions (Continued)

Methodology – TP80187	Method Performance – LC-MS/MS	Hyperlink – N/A	Comments
**Stability**			
Matrix (in polypropylene tubes)			
Freeze/Thaw (-20 °C/wet ice) with and without the presence of interference[2]	Demonstrated for 4 cycles	Summary tables<br>Table 21<br><br>Report text<br>Section 11.10	
Freeze/Thaw (-70 °C/wet ice) with and without the presence of interference[2]	Demonstrated for 4 cycles	Summary tables<br>Table 21<br><br>Report text<br>Section 11.10	
Bench-top (wet ice) with and without the presence of interference[2]	Demonstrated for 24 hours	Summary tables<br>Table 22<br><br>Report text<br>Section 11.11	
Refrigerated Stability (Treated and Untreated)	Demonstrated for 18.75 hours	Summary tables<br>Table 23<br><br>Report text<br>Section 11.12	
Long-term (-20 °C)	Demonstrated for 70 days	Summary tables<br>Table 24<br><br>Report text<br>Section 11.13	
Long-term (-70 °C)	Demonstrated for 70 days	Summary tables<br>Table 24<br><br>Report text<br>Section 11.13	
Processed Matrix polypropylene			
Refrigeration	Demonstrated for 3 days 23 hours	Summary tables<br>Table 25<br><br>Report text<br>Section 11.14	
**Individual Sample Reinjection**	Reproducible	Summary tables<br>Table 26<br><br>Report text<br>Section 11.15	
**Whole Run Reinjection Integrity**	Reproducible<br>Duration 20 days	Summary tables<br>Table 27<br><br>Report text<br>Section 11.16	

Table 7: Summary of Results and Conclusions (Continued)

**Methodology – TP80187**	**Method Performance – LC-MS/MS**	**Hyperlink – N/A**	**Comments**
**Column Ruggedness**	Reproducible	Summary tables<br>Table 27<br><br>Report text<br>Section 11.17	
**Extraction Recovery**			
Zidebactam	Overall 53.1%	Summary tables<br>Table 28<br><br>Report text<br>Section 11.18	
Cefotaxime (IS)	Overall 110.9%	Summary tables<br>Table 29<br><br>Report text<br>Section 11.18	
**Solution Stability**			
Solution, Primary glass			
Zidebactam in deionized water (RT)	Stable 17.75 hours	Summary tables<br>Table 30	
Zidebactam in deionized water (-20 °C)	Stable 25 days<br>Stable 61 days	Summary tables<br>Table 31	
Solution, Working (polypropylene)			
Zidebactam Spike in deionized water (RT)	Stable 17.75 hours	Summary tables<br>Table 32	
Zidebactam Spike in deionized water (-20 °C)	Stable 25 days<br>Stable 61 days for the high concentration, but not the low spike	Summary tables<br>Table 33	
Cefotaxime (IS) in deionized water in glass (RT)	Stable 15.5 hours	Summary tables<br>Table 34	

Table 8: Summary of Validation Run Objectives and Outcome

Run ID	Instrument ID	Extraction Date	Assay Date	Run Objective(s)	Accepted or Rejected	Comment
2	LC/MS-39	22 Jul 2022	23 Jul 2022	Precision and accuracy, IS cross-talk, selectivity, individual sample reinjection reproducibility, interference QCs, and processed sample stability extraction	Accepted	There is a -18.7 %Bias for the individual sample reinjection low samples. This was repeated in run 6
3	LC/MS-39	22 Jul 2022	23 Jul 2022	Spike and primary bench-top solution stability	Accepted	
4	LC/MS-39	25 Jul 2022	25 Jul 2022	Precision and accuracy and dilution integrity	Accepted	
5	LC/MS-39	27 Jul 2022	28 Jul 2022	Matrix factor and recovery	Accepted	
6	LC/MS-39	28 Jul 2022	29 Jul 2022	Precision and accuracy, freeze/thaw stability (comed/interference and regular QCs), bench-top stability in wet ice (comed/interference and regular QCs), individual sample reinjection reproducibility (low QC only), processed sample stability injection, and large run size	Accepted	Refrigerated processed sample stability failed at both low and high. Whole run reinjection duration will be used.
7	LC/MS-39	11 Aug 2022	11 Aug 2022	Treated and untreated refrigerated stability	Accepted	
9	LC/MS-39	15 Aug 2022	16 Aug 2022	Spike and primary long-term solution stability	Accepted	
10	LC/MS-39	17 Aug 2022	17 Aug 2022	Adsorption	Accepted	
11	LC/MS-39	28-Jul-2022	18-Aug-2022	Whole run reinjection reproducibility and column ruggedness	Accepted	
12	LC/MS-22	20 Sep 2022	21 Sep 2022	Primary check, linearity check, and primary and spiking long-term solution stability	Accepted	
13	LC/MS-22	21 Sep 2022	21 Sep 2022	Long-term matrix stability (comed/interference and regular QCs) and processed stability extraction	Rejected	Both QC 30.0 replicates do not meet acceptance criteria, so long-term stability cannot be evaluated
15	LC/MS-22	30 Sep 2022	03 Oct 2022	Long-term matrix stability (comed/interference and regular QCs) and processed stability extraction	Accepted	
16	LC/MS-24	04 Oct 2022	05 Oct 2022	Processed sample stability	Accepted	

Runs 1, 8, and 14 were used for a spike linearity check and primary comparison and are not reported.

Table 9: Carryover

Run ID	Result (Extracted Blank after High Standard)	Carryover Results for Internal Standard
2	Seq 13 and Seq 76 are <20.0% of the LLOQ.	Seq 4 and Seq 67 are < 5.0% internal standard peak of acceptable standards.
4	Seq 13 and Seq 62 are <20.0% of the LLOQ.	Seq 4 and Seq 53 are < 5.0% internal standard peak of acceptable standards.
6	Seq 13 and Seq 146 are <20.0% of the LLOQ.	Seq 4 and Seq 137 are < 5.0% internal standard peak of acceptable standards.
7	Seq 13 and Seq 56 are <20.0% of the LLOQ.	Seq 4 and Seq 47 are < 5.0% internal standard peak of acceptable standards.
11	Seq 13 and Seq 50 are <20.0% of the LLOQ.	Seq 4 and Seq 41 are < 5.0% internal standard peak of acceptable standards.
15	Seq 13 and Seq 80 are <20.0% of the LLOQ.	Seq 4 and Seq 71 are < 5.0% internal standard peak of acceptable standards.
16	Seq 13 and Seq 32 are <20.0% of the LLOQ.	Seq 4 and Seq 23 are < 5.0% internal standard peak of acceptable standards.

Note: Calibration Standards and QCs are arranged in a way to mitigate carryover in all validation runs.

Table 10: Selectivity - Blank Assessment

Run 2 Selectivity Blank Screen

Blank ID with or without IS	Blank Lot Number	Peak Area of Analyte	Peak Area of Internal Standard	Percent of LLOQ Peak Area	Percent of LLOQ Peak Area Ratio	Percent of IS in Blanks without IS
Blank 1 NO IS	V1872106U2-17-2	0	0	0.0	NA	0.0
Blank 2 NO IS	V1872106U2-17-3	0	0	0.0	NA	0.0
Blank 3 NO IS	V1872106U2-17-4	0	0	0.0	NA	0.0
Blank 4 NO IS	V1872106U2-17-5	0	0	0.0	NA	0.0
Blank 5 NO IS	V1872106U2-17-6	0	0	0.0	NA	0.0
Blank 6 NO IS	V1872106U2-17-7	0	0	0.0	NA	0.0
Blank 1	V1872106U2-17-2	0	68762	0.0	0.0	NA
Blank 2	V1872106U2-17-3	0	67620	0.0	0.0	NA
Blank 3	V1872106U2-17-4	0	68352	0.0	0.0	NA
Blank 4	V1872106U2-17-5	0	68853	0.0	0.0	NA
Blank 5	V1872106U2-17-6	0	67229	0.0	0.0	NA
Blank 6	V1872106U2-17-7	0	68516	0.0	0.0	NA

Note: Average IS response are from all of the accepted standards and QCs

Criteria: A minimum of six different sources of urine were screened. To be deemed acceptable, the peak response observed must be ≤ 20.0% of the mean response of the lower limit of quantitation (LLOQ) and ≤ 5.0% of the mean response of the internal standard. If any lots fail, further evaluation is needed.

Results: All matrix lots were found to be free of any significant interference at the retention times of the analytes of interest.

Table 11: Matrix Effect – Low Concentration

Run Number:	5								
Concentration:	30.0 mcg/mL								
**Sample ID**	**Drug in neat/matrix Area**	**Average Drug in Neat**	**Individual MF analyte**	**Average MF analyte**	**IS in neat/ matrix Area**	**Average IS in neat Area**	**Individual IS MF**	**Average IS MF**	**Average IS Normalized MF**
Neat	38643	37810			32283	31630			
	37710				31264				
	37574				31791				
	37312				31180				
ME1	38458		1.0171	1.0215	31631		1.0000	0.9939	1.0278
	39419		1.0426		32161		1.0168		
	38038		1.0060		30776		0.9730		
	38573		1.0202		31178		0.9857		
ME2	39805		1.0528	1.0297	32104		1.0150	1.0108	1.0187
	38389		1.0153		31485		0.9954		
	38005		1.0052		32368		1.0233		
	39532		1.0456		31931		1.0095		
ME3	37628		0.9952	1.0146	31569		0.9981	1.0093	1.0053
	38783		1.0257		32205		1.0182		
	38942		1.0299		31751		1.0038		
	38089		1.0074		32164		1.0169		
ME4	38299		1.0129	1.0143	31853		1.0071	0.9979	1.0164
	38046		1.0062		32122		1.0156		
	39083		1.0337		31220		0.9871		
	37977		1.0044		31060		0.9820		
ME5	39182		1.0363	1.0301	31935		1.0097	1.0115	1.0184
	38875		1.0282		32333		1.0222		
	39337		1.0404		31976		1.0110		
	38396		1.0155		31729		1.0031		
ME6	38290		1.0127	1.0221	32351		1.0228	1.0117	1.0103
	38731		1.0244		31935		1.0097		
	38474		1.0176		31321		0.9902		
	39081		1.0336		32386		1.0239		
**Mean**									1.0161
**N**									6
**SD**									0.0077
**%CV**									0.8

neat = in absence of matrix, matrix = in presence of extracted matrix
Criteria: %CV of Average IS Normalized MF among 6 lots of matrix must be ≤ 15.0%.
Meets Acceptance Criteria: YES MF=matrix factor

$$MF = \frac{\text{Peak Response in Presence of Matrix Substances}}{\text{Mean Peak Response in Absence of Matrix}}$$

$$\text{IS Normalized MF} = \frac{MF_{Analyte}}{MF_{IS}}$$

Table 12: Matrix Effect – High Concentration

Sample ID	Drug in neat/matrix Area	Average Drug in Neat	Individual MF analyte	Average MF analyte	IS in neat/ matrix Area	Average IS in neat Area	Individual IS MF	Average IS MF	Average IS Normalized MF
Run Number:	5								
Concentration:	3000 mcg/mL								
Neat	1599	2226			32359	32106			
	937				32335				
	3281				32257				
	3085				31472				
ME1	3584884		1610.8218	1597.8351	32157		1.0016	1.0019	1594.7430
	3554502		1597.1701		32426		1.0100		
	3549257		1594.8133		32354		1.0077		
	3535285		1588.5352		31735		0.9885		
ME2	3567196		1602.8740	1585.6254	32293		1.0058	0.9946	1594.1658
	3527822		1585.1818		31926		0.9944		
	3528839		1585.6387		32400		1.0092		
	3491380		1568.8070		31116		0.9692		
ME3	3554296		1597.0775	1573.3915	31425		0.9788	0.9894	1590.1945
	3514140		1579.0339		31880		0.9930		
	3410820		1532.6084		31335		0.9760		
	3527075		1584.8461		32426		1.0100		
ME4	3522182		1582.6475	1600.0491	31241		0.9731	0.9788	1634.7105
	3525204		1584.0054		31339		0.9761		
	3584241		1610.5329		31761		0.9893		
	3612010		1623.0106		31359		0.9767		
ME5	3563469		1601.1993	1590.9490	32256		1.0047	0.9965	1596.5309
	3523913		1583.4253		31920		0.9942		
	3570642		1604.4224		32274		1.0052		
	3504604		1574.7490		31524		0.9819		
ME6	3556654		1598.1370	1585.4378	32269		1.0051	0.9938	1595.4010
	3509266		1576.8439		31247		0.9733		
	3527780		1585.1629		32053		0.9984		
	3519867		1581.6073		32052		0.9983		
**Mean**									1600.9576
**N**									6
**SD**									16.6753
**%CV**									1.0

neat = in absence of matrix, matrix = in presence of extracted matrix
Criteria: %CV of Average IS Normalized MF among 6 lots of matrix must be ≤ 15.0%.
Meets Acceptance Criteria: YES MF=matrix factor

$$\text{MF} = \frac{\text{Peak Response in Presence of Matrix Substances}}{\text{Mean Peak Response in Absence of Matrix}}$$

$$\text{IS Normalized MF} = \frac{\text{MF}_{Analyte}}{\text{MF}_{IS}}$$

Table 13: Interference Evaluation

Run Date	Curve Number	Comed1[2] low 30.0 µg/mL	%Bias	Comed1[2] high 3000 µg/mL	%Bias
23-Jul-2022	2	28.2	-6.0	3070	2.3
		28.3	-5.7	3060	2.0
		28.8	-4.0	3170	5.7
		27.6	-8.0	3060	2.0
		29.7	-1.0	3120	4.0
		26.4	-12.0	3080	2.7
Mean		28.2		3090	
SD		1.11		43.7	
%CV		3.9		1.4	
%Bias		-6.0		3.0	
n		6		6	

Criteria: To be considered acceptable, the mean %Bias at each level was also required to be within ± 15.0% and the %CV was required to be ≤ 15.0%.

Results: The acceptance criteria were met.

Table 14: Summary of Standard Curves

Analytical Performance: Back-Calculated Concentrations (µg/mL) of Zidebactam Calibration Standard in (Human) (Urine) in (Study V1872106U2)

Assay Date	Analytical Run Number	Cal 10.0 10.0 µg/mL	Cal 20.0 20.0 µg/mL	Cal 160 160 µg/mL	Cal 400 400 µg/mL	Cal 800 800 µg/mL	Cal 1600 1600 µg/mL	Cal 3200 3200 µg/mL	Cal 4000 4000 µg/mL
23-Jul-2022	2	10.9	19.6	157	416	867	1670	3410	4200
		9.53	18.9	143	366	748	1610	3270	3990
25-Jul-2022	4	9.99	20.0	159	381	818	1630	3170	3940
		9.92	20.3	164	389	799	1640	3190	4030
29-Jul-2022	6	11.0	21.4	168	424	843	1640	3430	4250
		8.96	18.8	150	374	733	1540	3020	3910
Mean		10.1	19.8	157	392	801	1620	3250	4050
S.D.		0.788	0.969	9.15	23.4	52.6	44.5	156	140
%CV		7.8	4.9	5.8	6.0	6.6	2.7	4.8	3.5
%Bias		1.0	-1.0	-1.9	-2.0	0.1	1.3	1.6	1.3
n		6	6	6	6	6	6	6	6

Calibration Curve Parameters for Zidebactam Calibration Standards in Human Urine (Study V1872106U2)

Run Date	Curve Number	Slope	Intercept	R-Squared	LLOQ	ULOQ
23-Jul-2022	2	0.01388	-0.01947	0.9954	10.0	4000
25-Jul-2022	4	0.01583	-0.00307	0.9995	10.0	4000
29-Jul-2022	6	0.01639	-0.00665	0.9943	10.0	4000

See Table 8 for runs not used for precision/accuracy evaluation.

Table 15: Precision and Accuracy (Intra-Assay)

Human Urine Zidebactam Concentrations in Quality Controls for 3 separate batch runs (Study V1872106U2)

Run Date	Curve Number	QC 10.0 10.0 µg/mL	%Bias	QC 30.0 30.0 µg/mL	%Bias	QC 1200 1200 µg/mL	%Bias	QC 3000 3000 µg/mL	%Bias
23-Jul-2022	2	10.3	3.0	27.5	-8.3	1130	-5.8	3200	6.7
		10.4	4.0	27.1	-9.7	1140	-5.0	3080	2.7
		10.6	6.0	27.8	-7.3	1130	-5.8	3060	2.0
		9.56	-4.4	28.1	-6.3	1210	0.8	3140	4.7
		10.1	1.0	28.7	-4.3	1230	2.5	2960	-1.3
		9.88	-1.2	27.9	-7.0	1170	-2.5	3070	2.3
Intrarun Mean		10.1		27.9		1170		3090	
Intrarun SD		0.377		0.543		43.1		80.9	
Intrarun %CV		3.7		1.9		3.7		2.6	
Intrarun %Bias		1.0		-7.0		-2.5		3.0	
n		6		6		6		6	
25-Jul-2022	4	10.1	1.0	29.2	-2.7	1190	-0.8	3040	1.3
		9.89	-1.1	28.8	-4.0	1230	2.5	3090	3.0
		9.58	-4.2	30.0	0.0	1210	0.8	3090	3.0
		9.79	-2.1	29.7	-1.0	1180	-1.7	3100	3.3
		10.1	1.0	28.8	-4.0	1270	5.8	3130	4.3
		10.3	3.0	31.1	3.7	1210	0.8	3000	0.0
Intrarun Mean		9.96		29.6		1220		3080	
Intrarun SD		0.258		0.879		32.1		46.8	
Intrarun %CV		2.6		3.0		2.6		1.5	
Intrarun %Bias		-0.4		-1.3		1.7		2.7	
n		6		6		6		6	
29-Jul-2022	6	10.4	4.0	30.8	2.7	1240	3.3	3160	5.3
		10.3	3.0	32.4	8.0	1200	0.0	3250	8.3
		10.2	2.0	31.2	4.0	1280	6.7	3200	6.7
		10.0	0.0	29.7	-1.0	1160	-3.3	3050	1.7
		10.9	9.0	29.0	-3.3	1190	-0.8	3120	4.0
		9.73	-2.7	30.9	3.0	1140	-5.0	2980	-0.7
Intrarun Mean		10.3		30.7		1200		3130	
Intrarun SD		0.396		1.19		51.5		99.1	
Intrarun %CV		3.8		3.9		4.3		3.2	
Intrarun %Bias		3.0		2.3		0.0		4.3	
n		6		6		6		6	

Criteria: At each level, the %CV was required to be ≤ 15.0%, except at the LLOQ level, where ≤ 20.0% was accepted. The mean %Bias at each level was also required to be within ± 15.0%, except at the LLOQ, where ± 20.0% was accepted.

Results: The acceptance criteria were met. Run size (n=164) was completed in run 6 and met acceptance criteria.

Table 16: Precision and Accuracy (Inter-Assay)

Human Urine Zidebactam Concentrations in Quality Controls for 3 separate batch runs (Study V1872106U2)

Run Date	Curve Number	QC 10.0 10.0 µg/mL	%Bias	QC 30.0 30.0 µg/mL	%Bias	QC 1200 1200 µg/mL	%Bias	QC 3000 3000 µg/mL	%Bias
23-Jul-2022	2	10.3	3.0	27.5	-8.3	1130	-5.8	3200	6.7
		10.4	4.0	27.1	-9.7	1140	-5.0	3080	2.7
		10.6	6.0	27.8	-7.3	1130	-5.8	3060	2.0
		9.56	-4.4	28.1	-6.3	1210	0.8	3140	4.7
		10.1	1.0	28.7	-4.3	1230	2.5	2960	-1.3
		9.88	-1.2	27.9	-7.0	1170	-2.5	3070	2.3
25-Jul-2022	4	10.1	1.0	29.2	-2.7	1190	-0.8	3040	1.3
		9.89	-1.1	28.8	-4.0	1230	2.5	3090	3.0
		9.58	-4.2	30.0	0.0	1210	0.8	3090	3.0
		9.79	-2.1	29.7	-1.0	1180	-1.7	3100	3.3
		10.1	1.0	28.8	-4.0	1270	5.8	3130	4.3
		10.3	3.0	31.1	3.7	1210	0.8	3000	0.0
29-Jul-2022	6	10.4	4.0	30.8	2.7	1240	3.3	3160	5.3
		10.3	3.0	32.4	8.0	1200	0.0	3250	8.3
		10.2	2.0	31.2	4.0	1280	6.7	3200	6.7
		10.0	0.0	29.7	-1.0	1160	-3.3	3050	1.7
		10.9	9.0	29.0	-3.3	1190	-0.8	3120	4.0
		9.73	-2.7	30.9	3.0	1140	-5.0	2980	-0.7
Mean Concentration Found (µg/mL)		10.1		29.4		1200		3100	
Inter-run SD		0.351		1.47		45.1		77.4	
Inter-run %CV		3.5		5.0		3.8		2.5	
Inter-run %Bias		1.0		-2.0		0.0		3.3	
n		18		18		18		18	

Criteria: At each level, the %CV was required to be ≤ 15.0%, except at the LLOQ level, where ≤ 20.0% was accepted. In addition, the mean %Bias at each level was required to be within ± 15.0% from nominal value, except at the LLOQ, where ± 20.0% was accepted.

Results:The acceptance criteria were met.

Table 17: Standard and QC of Failed Runs

Analytical Performance: Back-Calculated Concentrations (µg/mL) of Zidebactam Calibration Standard in (Human) (Urine) in (Study V1872106U2) (Rejected Runs)

Assay Date	Analytical Run Number	Cal 10.0 10.0 µg/mL	Cal 20.0 20.0 µg/mL	Cal 160 160 µg/mL	Cal 400 400 µg/mL	Cal 800 800 µg/mL	Cal 1600 1600 µg/mL	Cal 3200 3200 µg/mL	Cal 4000 4000 µg/mL
21-Sep-2022	13	10.9	19.4	165	*525	*1250	1560	3390	4240
		9.09	20.7	157	404	799	1540	2970	3980
Mean		10.0	20.1	161	404	799	1550	3180	4110
%Bias		0.0	0.5	0.6	1.0	-0.1	-3.1	-0.6	2.8
Reason Deactivated									
* Excluded due to being outside acceptance criteria									

Calibration Curve Parameters for Zidebactam Calibration Standards in Human Urine (Study V1872106U2) (Rejected Runs)

Run Date	Curve Number	Slope	Intercept	R-Squared	LLOQ	ULOQ
21-Sep-2022	13	0.04857	0.03059	0.9966	10.0	4000

Run Date	Curve Number	QC 30.0 30.0 µg/mL	%Bias	QC 1200 1200 µg/mL	%Bias	QC 3000 3000 µg/mL	%Bias
21-Sep-2022	13	#34.7	15.7	1320	10.0	3090	3.0
		#38.1	27.0	1290	7.5	3170	5.7
Mean		36.4		1310		3130	
%Bias		21.3		9.2		4.3	
# > 15%Bias							

Criteria: For each run, the calculated concentrations of at least 75% of the calibration standards are required to interpolate within ± 15.0% from nominal with a minimum of six calibration standard levels, except for the LLOQ for which it was required to be within ± 20.0%. In order for the run to be acceptable, at least one QC at each concentration level and two-thirds of the total number of QC samples were required to interpolate within ± 15.0% from nominal.

Results: The acceptance criteria were not met.

Table 18: Standards and QCs of Non-P&A Runs

Analytical Performance: Back-Calculated Concentrations (µg/mL) of Zidebactam Calibration Standard in (Human) (Urine) in (Study V1872106U2)

Assay Date	Analytical Run Number	Cal 10.0 10.0 µg/mL	Cal 20.0 20.0 µg/mL	Cal 160 160 µg/mL	Cal 400 400 µg/mL	Cal 800 800 µg/mL	Cal 1600 1600 µg/mL	Cal 3200 3200 µg/mL	Cal 4000 4000 µg/mL
11-Aug-2022	7	9.69	20.6	164	426	820	1590	3140	3940
		10.1	20.2	156	399	805	1570	3120	3890
03-Oct-2022	15	9.94	20.1	153	406	852	1610	3230	3810
		9.91	20.6	156	402	850	1610	3110	3810
05-Oct-2022	16	10.0	20.3	160	423	857	1660	3250	3940
		10.0	19.7	144	402	817	1690	2940	3710
Mean		9.94	20.3	156	410	834	1620	3130	3850
S.D.		0.139	0.339	6.80	11.7	22.1	44.9	111	90.1
%CV		1.4	1.7	4.4	2.9	2.6	2.8	3.5	2.3
%Bias		-0.6	1.5	-2.5	2.5	4.3	1.3	-2.2	-3.8
n		6	6	6	6	6	6	6	6

Calibration Curve Parameters for Zidebactam Calibration Standards in Human Urine (Study V1872106U2)

Run Date	Curve Number	Slope	Intercept	R-Squared	LLOQ	ULOQ
11-Aug-2022	7	0.01756	-0.00888	0.9991	10.0	4000
03-Oct-2022	15	0.03822	-0.00985	0.9985	10.0	4000
05-Oct-2022	16	0.01562	0.00638	0.9970	10.0	4000

Human Urine Zidebactam Concentrations in Quality Controls for 3 separate batch runs (Study V1872106U2)

Run Date	Curve Number	QC 30.0 30.0 µg/mL	%Bias	QC 1200 1200 µg/mL	%Bias	QC 3000 3000 µg/mL	%Bias
11-Aug-2022	7	31.7	5.7	1180	-1.7	3060	2.0
		31.0	3.3	1110	-7.5	3070	2.3
Intrarun Mean		31.4		1150		3070	
Intrarun %Bias		4.7		-4.2		2.3	
03-Oct-2022	15	34.0	13.3	1280	6.7	3150	5.0
		31.8	6.0	1240	3.3	3110	3.7
Intrarun Mean		32.9		1260		3130	
Intrarun %Bias		9.7		5.0		4.3	
05-Oct-2022	16	33.7	12.3	1300	8.3	3140	4.7
		#34.6	15.3	1310	9.2	3000	0.0
Intrarun Mean		34.2		1310		3070	
Intrarun %Bias		14.0		9.2		2.3	
# > 15%Bias							

Criteria: For each run, the calculated concentrations of at least 75% of the calibration standards are required to interpolate within ± 15.0% from nominal with a minimum of six calibration standard levels, except for the LLOQ for which it was required to be within ± 20.0%.
In order for the run to be acceptable, at least one QC at each concentration level and two-thirds of the total number of QC samples were required to interpolate within ± 15.0% from nominal.

Results: Acceptance criteria were met.

Table 19: Precision and Accuracy of Sample Dilution

Dilution Factor		2x		5x	
**Run Date**	**Curve Number**	**Dil QC 3000 3000 µg/mL**	**%Bias**	**Dil QC 16000 16000 µg/mL**	**%Bias**
25-Jul-2022	4	3110	3.7	14900	-6.9
		3110	3.7	#13000	-18.8
		3190	6.3	14700	-8.1
		3050	1.7	17500	9.4
		3020	0.7	17300	8.1
		3100	3.3	13900	-13.1
Mean		3100		15200	
SD		58.5		1820	
%CV		1.9		12.0	
%Bias		3.3		-5.0	
n		6		6	
# > 15%Bias					

Sample dilution as follows:

2x =25 mcL sample + 25 mcL blank treated human urine
5x =10 mcL sample + 40 mcL blank treated human urine

Criteria: To be deemed acceptable, the mean %Bias at each level must be within ± 15.0% with %CV ≤ 15.0%.
Results: The acceptance criteria were met.

Table 20: Analyte Adsorption Container Test

Analytical Performance: Back-Calculated Concentrations (µg/mL) of Zidebactam Calibration Standard in Human Urine (Study V1872106U2)

Assay Date	Analytical Run Number	Cal 10.0 10.0 µg/mL	Cal 20.0 20.0 µg/mL	Cal 160 160 µg/mL	Cal 400 400 µg/mL	Cal 800 800 µg/mL	Cal 1600 1600 µg/mL	Cal 3200 3200 µg/mL	Cal 4000 4000 µg/mL
17-Aug-2022	10	10.4	20.2	158	421	826	1630	3280	4090
		9.59	*16.6	150	399	785	1580	3130	3870
Mean		10.0	20.2	154	410	806	1610	3210	3980
%Bias		0.0	1.0	-3.8	2.5	0.8	0.6	0.3	-0.5
Reason Deactivated									
* Outside Acceptance Criteria									

Calibration Curve Parameters for Zidebactam Calibration Standards in Human Urine (Study V1872106U2)

Run Date	Curve Number	Slope	Intercept	R-Squared	LLOQ	ULOQ
17-Aug-2022	10	0.01646	-0.00724	0.9987	10.0	4000

Analytical Performance of Zidebactam Quality Control Samples in Human Urine (Study V1872106U2)

Run Date	Curve Number	QC 30.0 30.0 µg/mL	%Bias	QC 1200 1200 µg/mL	%Bias	QC 3000 3000 µg/mL	%Bias
17-Aug-2022	10	30.0	0.0	1200	0.0	3110	3.7
		29.5	-1.7	1160	-3.3	3020	0.7
Mean		29.8		1180		3070	
%Bias		-0.7		-1.7		2.3	

Run Date	Curve Number	Ads. HDPE Low Treat 30.0 µg/mL	%Bias	Ads. HDPE Low Urine 30.0 µg/mL	%Bias	Ads. HDPE High Treat 3000 µg/mL	%Bias	Ads. HDPE High Urine 3000 µg/mL	%Bias
17-Aug-2022	10	30.7	2.3	27.2	-9.3	2860	-4.7	3010	0.3
		30.2	0.7	28.8	-4.0	2880	-4.0	2950	-1.7
		29.6	-1.3	29.0	-3.3	2850	-5.0	3010	0.3
		29.7	-1.0	28.7	-4.3	2870	-4.3	2940	-2.0
		30.2	0.7	28.5	-5.0	2850	-5.0	2910	-3.0
		29.1	-3.0	28.3	-5.7	2890	-3.7	2880	-4.0
Mean		29.9		28.4		2870		2950	
S.D.		0.564		0.643		16.3		52.5	
%CV		1.9		2.3		0.6		1.8	
%Bias		-0.3		-5.3		-4.3		-1.7	
n		6		6		6		6	

Run Date	Curve Number	Ads. Poly Low Treat 30.0 µg/mL	%Bias	Ads. Poly Low Urine 30.0 µg/mL	%Bias	Ads. Poly High Treat 3000 µg/mL	%Bias	Ads. Poly High Urine 3000 µg/mL	%Bias
17-Aug-2022	10	30.4	1.3	29.3	-2.3	2940	-2.0	2900	-3.3
		30.6	2.0	29.0	-3.3	2950	-1.7	2980	-0.7
		28.8	-4.0	29.9	-0.3	2970	-1.0	2840	-5.3
		29.5	-1.7	29.0	-3.3	2900	-3.3	2870	-4.3
		28.4	-5.3	29.6	-1.3	2910	-3.0	2950	-1.7
		30.2	0.7	29.0	-3.3	2880	-4.0	2950	-1.7
Mean		29.7		29.3		2930		2920	
S.D.		0.903		0.379		33.9		53.9	
%CV		3.0		1.3		1.2		1.8	
%Bias		-1.0		-2.3		-2.3		-2.7	
n		6		6		6		6	

Criteria: To be deemed stable, the mean %Bias at each level must be within ± 15.0% with %CV ≤ 15.0%.
Results: No significant adsorption is seen for zidebactam in human urine (treated and untreated) in high density polyethylene or polypropylene.

Table 21: Freeze-Thaw Stability, (Frozen At -20 °C and -70 °C Thawed in Wet Ice)

Without Interference[2]

Run Date	Curve Number	F/T -20/RT low 30.0 µg/mL	%Bias	F/T -70/RT low 30.0 µg/mL	%Bias	F/T -20/RT high 3000 µg/mL	%Bias	F/T -70/RT high 3000 µg/mL	%Bias
29-Jul-2022	6	33.3	11.0	32.3	7.7	3100	3.3	3160	5.3
		31.5	5.0	32.1	7.0	3200	6.7	3110	3.7
		31.5	5.0	32.3	7.7	3230	7.7	3250	8.3
		29.2	-2.7	29.7	-1.0	3070	2.3	3010	0.3
		30.4	1.3	30.0	0.0	3100	3.3	3000	0.0
		29.5	-1.7	30.4	1.3	3030	1.0	2970	-1.0
Mean		30.9		31.1		3120		3080	
SD		1.52		1.23		77.3		109	
%CV		4.9		4.0		2.5		3.5	
%Bias		3.0		3.7		4.0		2.7	
n		6		6		6		6	

With Interference[2]

Run Date	Curve Number	Comed1 F/T - 20 low 30.0 µg/mL	%Bias	Comed1 F/T - 70 low 30.0 µg/mL	%Bias	Comed1 F/T - 20 high 3000 µg/mL	%Bias	Comed1 F/T - 70 high 3000 µg/mL	%Bias
29-Jul-2022	6	31.8	6.0	30.8	2.7	3240	8.0	3260	8.7
		32.7	9.0	31.7	5.7	3140	4.7	3160	5.3
		32.2	7.3	31.7	5.7	3290	9.7	3050	1.7
		31.1	3.7	30.2	0.7	2970	-1.0	3030	1.0
		30.2	0.7	29.8	-0.7	3080	2.7	3040	1.3
		30.2	0.7	30.0	0.0	3150	5.0	3090	3.0
Mean		31.4		30.7		3150		3110	
SD		1.04		0.844		114		89.6	
%CV		3.3		2.7		3.6		2.9	
%Bias		4.7		2.3		5.0		3.7	
n		6		6		6		6	

Criteria: To be deemed stable, the mean %Bias at each level must be within ± 15.0% with %CV ≤ 15.0%.

Results: Zidebactam is stable in human urine with and without the presence of interference[2] for at least 4 cycles when frozen at -20 °C and -70 °C and thawed in wet ice.

Table 22: Bench-Top Stability in Wet Ice

Without Interference[2]

Run Date	Curve Number	BT 24hr low 30.0 µg/mL	%Bias	BT 6hr low 30.0 µg/mL	%Bias	BT 24hr high 3000 µg/mL	%Bias	BT 6hr high 3000 µg/mL	%Bias
29-Jul-2022	6	31.1	3.7	32.2	7.3	3110	3.7	2960	-1.3
		31.1	3.7	32.3	7.7	3150	5.0	3240	8.0
		32.0	6.7	31.8	6.0	3160	5.3	3360	12.0
		29.0	-3.3	29.2	-2.7	2930	-2.3	2990	-0.3
		27.5	-8.3	29.0	-3.3	2990	-0.3	3000	0.0
		27.8	-7.3	29.6	-1.3	2970	-1.0	3030	1.0
Mean		29.8		30.7		3050		3100	
SD		1.90		1.57		100		164	
%CV		6.4		5.1		3.3		5.3	
%Bias		-0.7		2.3		1.7		3.3	
n		6		6		6		6	

With Interference[2]

Run Date	Curve Number	Comed1 BT 24hr low 30.0 µg/mL	%Bias	Comed1 BT 6hr low 30.0 µg/mL	%Bias	Comed1 BT 24hr high 3000 µg/mL	%Bias	Comed1 BT 6hr high 3000 µg/mL	%Bias
29-Jul-2022	6	31.6	5.3	32.2	7.3	3170	5.7	3180	6.0
		30.8	2.7	32.8	9.3	3200	6.7	3290	9.7
		32.1	7.0	33.2	10.7	3130	4.3	3040	1.3
		29.2	-2.7	28.0	-6.7	2990	-0.3	3150	5.0
		30.1	0.3	31.1	3.7	3030	1.0	2950	-1.7
		30.0	0.0	30.1	0.3	2950	-1.7	2980	-0.7
Mean		30.6		31.2		3080		3100	
SD		1.08		1.95		102		131	
%CV		3.5		6.3		3.3		4.2	
%Bias		2.0		4.0		2.7		3.3	
n		6		6		6		6	

Criteria: To be deemed stable, the mean %Bias at each level must be within ± 15.0% with %CV ≤ 15.0%.
Results: Zidebactam is stable in human urine with and without the presence of interference[2] for at least 24 hours in wet ice.

Table 23: Refrigerated Stability (Treated and Untreated)

Run Date	Curve Number	Treated Refr. Low 30.0 µg/mL	%Bias	Untreated Refr. Low 30.0 µg/mL	%Bias	Treated Refr. High 3000 µg/mL	%Bias	Untreated Refr. High 3000 µg/mL	%Bias
11-Aug-2022	7	30.4	1.3	30.4	1.3	3000	0.0	2990	-0.3
		30.3	1.0	29.8	-0.7	2920	-2.7	2940	-2.0
		29.9	-0.3	30.7	2.3	2960	-1.3	2960	-1.3
		30.1	0.3	29.9	-0.3	2990	-0.3	3000	0.0
		29.0	-3.3	30.4	1.3	2870	-4.3	3100	3.3
		30.3	1.0	30.5	1.7	2940	-2.0	3060	2.0
Mean		30.0		30.3		2950		3010	
S.D.		0.522		0.354		48.0		60.8	
%CV		1.7		1.2		1.6		2.0	
%Bias		0.0		1.0		-1.7		0.3	
n		6		6		6		6	

Criteria: To be deemed stable, the mean %Bias at each level must be within ± 15.0% with %CV ≤ 15.0%.

Results: The acceptance criteria were met for both untreated and treated QCs. Zidebactam in treated and untreated urine is stable for up to 18.75 hours when stored refrigerated in polypropylene.

Table 24: Long-Term Stability at -20 °C and -70 °C

Without Interference[2]

Run Date	Curve Number	LTS -20 low 30.0 µg/mL	%Bias	LTS -70 low 30.0 µg/mL	%Bias	LTS -20 high 3000 µg/mL	%Bias	LTS -70 high 3000 µg/mL	%Bias
03-Oct-2022	15	27.4	-8.7	32.5	8.3	2600	-13.3	3070	2.3
		28.8	-4.0	32.3	7.7	2940	-2.0	3240	8.0
		27.6	-8.0	33.3	11.0	2930	-2.3	3140	4.7
		27.4	-8.7	33.9	13.0	2780	-7.3	2730	-9.0
		31.7	5.7	32.6	8.7	2760	-8.0	3100	3.3
		31.6	5.3	32.7	9.0	2860	-4.7	3110	3.7
Mean		29.1		32.9		2810		3070	
SD		2.06		0.601		128		174	
%CV		7.1		1.8		4.6		5.7	
%Bias		-3.0		9.7		-6.3		2.3	
n		6		6		6		6	
# > 15%Bias									

With Interference[2]

Run Date	Curve Number	LTS -20 low Ertap 30.0 µg/mL	%Bias	LTS -70 low Ertap 30.0 µg/mL	%Bias	LTS -20 high Ertap 3000 µg/mL	%Bias	LTS -70 high Ertap 3000 µg/mL	%Bias
03-Oct-2022	15	29.6	-1.3	33.4	11.3	2710	-9.7	2970	-1.0
		27.5	-8.3	#34.6	15.3	2860	-4.7	3060	2.0
		28.7	-4.3	#35.4	18.0	2840	-5.3	3340	11.3
		27.0	-10.0	33.6	12.0	2850	-5.0	3050	1.7
		28.3	-5.7	33.4	11.3	2730	-9.0	3310	10.3
		30.5	1.7	33.3	11.0	2870	-4.3	3110	3.7
Mean		28.6		34.0		2810		3140	
SD		1.30		0.857		70.7		150	
%CV		4.5		2.5		2.5		4.8	
%Bias		-4.7		13.3		-6.3		4.7	
n		6		6		6		6	
# > 15%Bias									

Criteria: To be deemed stable, the mean %Bias at each level must be within ± 15.0% with %CV ≤ 15.0%.

Results: Zidebactam is stable in human urine with and without the presence of interference[2] for up to 70 days upon storage in polypropylene tubes at -20 °C and -70 °C.

Table 25: Refrigerated Processed Sample Stability

Run Date	Curve Number	Processed Refr low 30.0 µg/mL	%Bias	Processed Refr high 3000 µg/mL	%Bias
29-Jul-2022	6	#18.2	-39.3	#2010	-33.0
		#19.6	-34.7	#1990	-33.7
		#19.6	-34.7	#2010	-33.0
		#18.6	-38.0	#2030	-32.3
		#18.5	-38.3	#2080	-30.7
		#18.7	-37.7	#2120	-29.3
Mean		18.9		2040	
SD		0.592		49.8	
%CV		3.1		2.4	
%Bias		-37.0		-32.0	
n		6		6	
# > 15%Bias					

Processed sample stability QCs were processed with run 2 and then stored for 5 days 21 hours in polypropylene 96-well plates at refrigerated temperatures prior to injection in run 6.

Run Date	Curve Number	Processed Refr low 30.0 µg/mL	%Bias	Processed Refr high 3000 µg/mL	%Bias
05-Oct-2022	16	28.4	-5.3	#2520	-16.0
		27.6	-8.0	2620	-12.7
		25.9	-13.7	#2500	-16.7
		26.3	-12.3	2580	-14.0
		26.7	-11.0	#2530	-15.7
		26.7	-11.0	2590	-13.7
Mean		26.9		2560	
SD		0.914		46.8	
%CV		3.4		1.8	
%Bias		-10.3		-14.7	
n		6		6	
# > 15%Bias					

Processed sample stability QCs were processed with run 15 and then stored for 3 days 23 hours in polypropylene 96-well plates at refrigerated temperatures prior to injection in run 16.

Criteria: To be deemed stable, the mean %Bias at each level must be within ± 15.0% with %CV ≤ 15.0%.

Results: The acceptance criteria were not met in run 6. Processed sample stability failed at both levels for 5 days and 21 hours. Processed sample stability was repeated in run 16 and met acceptance criteria for 3 days 23 hours.

Table 26: Individual Sample Reinjection Reproducibility

Run Date	Curve Number	Indiv Reinj low 30.0 µg/mL	%Bias	Indiv Reinj high 3000 µg/mL	%Bias
23-Jul-2022	2	#24.8	-17.3	3050	1.7
		#24.6	-18.0	3040	1.3
		26.2	-12.7	3100	3.3
		#24.5	-18.3	2950	-1.7
		#24.0	-20.0	2950	-1.7
		#22.0	-26.7	3080	2.7
Intrarun Mean		24.4		3030	
Intrarun SD		1.37		64.3	
Intrarun %CV		5.6		2.1	
Intrarun %Bias		-18.7		1.0	
n		6		6	
29-Jul-2022	6	26.3	-12.3		
		28.3	-5.7		
		27.3	-9.0		
		28.1	-6.3		
		28.2	-6.0		
		28.0	-6.7		
Intrarun Mean		27.7			
Intrarun SD		0.772			
Intrarun %CV		2.8			
Intrarun %Bias		-7.7			
n		6			
# > 15%Bias					

Low and high QC extracts (6 each) were injected during the run and then reinjected at the end of the same run.

Criteria: To be deemed reproducible, at least four out of six QCs at each level must interpolate with dev [%] within ± 15.0% from nominal, and the mean %Bias at each level must be within ± 15.0% with %CV ≤ 15.0%.

Results: Run 2 low QCs did not meet acceptance criteria and were repeated in run 6. The acceptance criteria were met.

Table 27: Whole Run Reinjection Reproducibility and Column Ruggedness

Analytical Performance: Back-Calculated Concentrations (µg/mL) of Zidebactam Calibration Standard in Human Urine (Study V1872106U2)

Assay Date	Analytical Run Number	Cal 10.0 10.0 µg/mL	Cal 20.0 20.0 µg/mL	Cal 160 160 µg/mL	Cal 400 400 µg/mL	Cal 800 800 µg/mL	Cal 1600 1600 µg/mL	Cal 3200 3200 µg/mL	Cal 4000 4000 µg/mL
18-Aug-2022	11	9.03	19.5	157	404	797	1530	3130	4090
		10.9	20.9	165	403	786	1650	3180	4000
Mean		9.97	20.2	161	404	792	1590	3160	4050
%Bias		-0.3	1.0	0.6	1.0	-1.0	-0.6	-1.3	1.3

Calibration Curve Parameters for Zidebactam Calibration Standards in Human Urine (Study V1872106U2)

Run Date	Curve Number	Slope	Intercept	R-Squared	LLOQ	ULOQ
18-Aug-2022	11	0.00768	-0.00058	0.9979	10.0	4000

Analytical Performance of Zidebactam Quality Control Samples in Human Urine (Study V1872106U2)

Run Date	Curve Number	QC 10.0 10.0 µg/mL	%Bias	QC 30.0 30.0 µg/mL	%Bias	QC 1200 1200 µg/mL	%Bias	QC 3000 3000 µg/mL	%Bias
18-Aug-2022	11	9.82	-1.8	28.9	-3.7	1180	-1.7	2990	-0.3
		10.0	0.0	28.4	-5.3	1170	-2.5	3120	4.0
		10.0	0.0	31.2	4.0	1220	1.7	3090	3.0
		9.21	-7.9	30.7	2.3	1180	-1.7	3060	2.0
		10.7	7.0	30.2	0.7	1200	0.0	3080	2.7
		9.79	-2.1	30.9	3.0	1180	-1.7	3040	1.3
Mean		9.92		30.1		1190		3060	
S.D.		0.480		1.14		18.3		45.0	
%CV		4.8		3.8		1.5		1.5	
%Bias		-0.8		0.3		-0.8		2.0	
n		6		6		6		6	

Note: Run 11 is a reinjection of run 6 on a different analytical column of the same manufacturer, dimensions, and type, using same MS platform (Applied Biosystems API 5000) – reinjected within 20 days

Criteria: At each level, the %CV was required to be ≤ 15.0% from the nominal value, except at the LLOQ level, where ≤ 20.0% was accepted. The mean %Bias at each level was also required to be within ± 15.0%, except at the LLOQ, where ± 20.0% was accepted.

Results: The acceptance criteria were met.

Table 28: Extraction Recovery – Zidebactam

Run: 5

Control (Post Extraction Spike)					Extracted QCs				
QC Conc. mcg/mL	Drug Peak Area	Drug Peak Area (corrected)	IS Peak Area	Peak Area Ratio	QC Conc. mcg/mL	Drug Peak Area	IS Peak Area	Peak Area Ratio	Extraction Recovery %
Low QC					Low QC				
30.0	36245	18123	36233	0.5002	30.0	18855	35051	0.5379	108.2
30.0	36303	18152	36685	0.4948	30.0	18689	36632	0.5102	102.6
30.0	36441	18221	36679	0.4968	30.0	18748	36313	0.5163	103.9
30.0	36458	18229	36129	0.5046	30.0	18299	35964	0.5088	102.3
30.0	33723	16862	34823	0.4842	30.0	19339	36347	0.5321	107.0
30.0	36219	18110	36051	0.5023	30.0	19361	37271	0.5195	104.5
			Average	0.4971				Average	104.8
Mid QC					Mid QC				
1200	1437418	718709	37233	19.3030	1200	735113	36335	20.2315	102.2
1200	1418718	709359	35602	19.9247	1200	758454	35595	21.3079	107.7
1200	1423530	711765	35878	19.8385	1200	755867	36289	20.8291	105.3
1200	1456435	728218	36947	19.7098	1200	767272	35566	21.5732	109.0
1200	1422910	711455	35546	20.0151	1200	762640	35617	21.4122	108.2
1200	1413928	706964	35453	19.9409	1200	763022	36443	20.9374	105.8
			Average	19.7887				Average	106.4
High QC					High QC				
3000	3584226	1792113	36815	48.6789	3000	1886091	36335	51.9084	106.7
3000	3561680	1780840	36820	48.3661	3000	1867088	36112	51.7027	106.3
3000	3454126	1727063	34736	49.7197	3000	1880658	35963	52.2942	107.5
3000	3603081	1801541	36835	48.9084	3000	1814483	35518	51.0863	105.0
3000	3503416	1751708	35857	48.8526	3000	1830181	35003	52.2864	107.5
3000	3487630	1743815	36796	47.3914	3000	1927273	35860	53.7444	110.5
			Average	48.6528				Average	107.2
								n:	18
								Overall Mean Recovery:	**106.1**
								Overall Standard Deviation:	**2.4**
								Overall % CV:	**2.2**

$$\% \text{ Recovery} = \frac{\text{Response Ratio of "Extracted QC"}}{\text{Average Response Ratio of Control}} \times 100$$

Table 29: Extraction Recovery – Cefotaxime

Run: 5

Control				Extracted QCs				
WIS Conc. *mcg/mL*	IS Peak Area	Drug Peak Area	Peak Area Ratio	WIS Conc. *mcg/mL*	IS Peak Area	Drug Peak Area	Peak Area Ratio	Extraction Recovery %
500	32179	762787	0.042186	500	36335	735113	0.049428	115.3
500	32154	769314	0.041796	500	35595	758454	0.046931	109.5
500	31884	756587	0.042142	500	36289	755867	0.048010	112.0
500	32557	753983	0.043180	500	35566	767272	0.046354	108.2
500	31247	734942	0.042516	500	35617	762640	0.046702	109.0
500	32463	716483	0.045309	500	36443	763022	0.047761	111.4
		Average	0.042855					=====
							n:	6
							Mean Recovery:	**110.9**
							Standard Deviation:	**2.6**
							%CV:	**2.4**

$$\% \text{ Recovery} = \frac{\text{Response Ratio of "Extracted QC"}}{\text{Average Response Ratio of Control}} \times 100$$

Table 30: Stability of Zidebactam Primary Solution in Deionized Water at Room Temperature

Run: 3
Container: Glass

Analyte	Sample Name	Sequence Number	Analyte Peak Area	IS Peak Area	Peak Area Ratio		Bench Top	Refrigerated	-20 °C Reference
Zidebactam 160 mg/mL	3 040 V1872106U2 Primary -20°C-1 1 1	40	5873361	70659	83.1226	Lot	V1872106U2-7-1 compared to Refr	V1872106U2-7-2 Compared to -20°C	V1872106U2-7-3
	3 041 V1872106U2 Primary -20°C-1 2 1	41	5955067	72984	81.5941				
	3 042 V1872106U2 Primary -20°C-1 3 1	42	6037661	72006	83.8494				
	3 043 V1872106U2 Primary -20°C-2 1 1	43	6159684	73349	83.9778				
	3 044 V1872106U2 Primary -20°C-2 2 1	44	6042317	70926	85.1918				
	3 045 V1872106U2 Primary -20°C-2 3 1	45	6229043	73072	85.2453	Avg	82.8448	87.6236	83.8302
	3 046 V1872106U2 Primary Refr-1 1 1	46	6399570	70861	90.3116	Std Dev	2.236	1.673	1.370
	3 047 V1872106U2 Primary Refr-1 2 1	47	6360185	73071	87.0412	%CV	2.7	1.9	1.6
	3 048 V1872106U2 Primary Refr-1 3 1	48	6146003	71623	85.8105	%Change	-5.5	4.5	N/A
	3 049 V1872106U2 Primary Refr-2 1 1	49	6284414	71539	87.8460				
	3 050 V1872106U2 Primary Refr-2 2 1	50	6330916	71469	88.5827				
	3 051 V1872106U2 Primary Refr-2 3 1	51	6223524	72241	86.1495	Duration	17.75 hours		
	3 052 V1872106U2 Primary BT-1 1 1	52	5820122	72929	79.8053				
	3 053 V1872106U2 Primary BT-1 2 1	53	5878923	71780	81.9020				
	3 054 V1872106U2 Primary BT-1 3 1	54	5916822	71690	82.5334				
	3 055 V1872106U2 Primary BT-2 1 1	55	6102441	70418	86.6602				
	3 056 V1872106U2 Primary BT-2 2 1	56	6029362	72377	83.3049				
	3 057 V1872106U2 Primary BT-2 3 1	57	6075182	73316	82.8630				

$$\%\text{Change} = \frac{\text{mean response of stored solutions} - \text{mean response of comparison solutions}}{\text{mean response of comparison solutions}} \times 100$$

Criteria: To be deemed stable, % Change upon storage for analyte must be within ±15.0% and %CV must be within ≤15.0%.
Meets Criteria: YES

Table 31: Stability of Zidebactam Primary Solution in Deionized Water at -20 °C

Run: 9
Container: Glass

Concentration: 160 mg/mL									
Stability Data (Stored)					Comparison Data (Fresh)				
Lot #:	V1872106U2-2-1				Lot #:	V1872106U2-28-1			
Prepared:	21-Jul-2022				Prepared:	15-Aug-2022			
Sample ID	Seq #	Drug Peak Area	IS Peak Area	Peak Area Ratio	Sample ID	Seq #	Drug Peak Area	IS Peak Area	Peak Area Ratio
9 034 V1872106U2 Primary stored-1 1 1	34	4889962	53131	92.0359	9 028 V1872106U2 Primary fresh-1 1 1	28	4929982	53808	91.6217
9 035 V1872106U2 Primary stored-1 2 1	35	4930025	55191	89.3266	9 029 V1872106U2 Primary fresh-1 2 1	29	4982471	54565	91.3126
9 036 V1872106U2 Primary stored-1 3 1	36	4936729	54750	90.1686	9 030 V1872106U2 Primary fresh-1 3 1	30	4928925	52976	93.0407
9 037 V1872106U2 Primary stored-2 1 1	37	4912770	55707	88.1895	9 031 V1872106U2 Primary fresh-2 1 1	31	5019924	54404	92.2712
9 038 V1872106U2 Primary stored-2 2 1	38	4956650	56101	88.3523	9 032 V1872106U2 Primary fresh-2 2 1	32	4992694	54774	91.1508
9 039 V1872106U2 Primary stored-2 3 1	39	4975100	55073	90.3365	9 033 V1872106U2 Primary fresh-2 3 1	33	4959224	54632	90.7751
			Mean	89.7349				Mean	91.6954
			n	6				n	6
			SD	1.4361				SD	0.8291
			%CV	1.6				%CV	0.9
%Change =	-2.1								
Duration:	25	Days							

$$\%\text{Change} = \frac{\text{mean response of stored solutions} - \text{mean response of comparison solutions}}{\text{mean response of comparison solutions}} \times 100$$

Criteria: To be deemed stable, % Change upon storage for analyte must be within ±15.0% and %CV must be within ≤15.0%.
Meets Criteria: YES

Table 31: Stability of Zidebactam Primary Solution in Deionized Water at -20 °C (Continued)

Run: 12
Container: Glass

Analyte	Sample Name	Sequence Number	Analyte Peak Area	IS Peak Area	Peak Area Ratio		Stored	Fresh
Zidebactam 160 mg/mL	12 057 V1872106U2 Primary fresh-1 1 1	57	6395288	43481	147.0824	Lot	V1872106U2-2-1	V1872106U2-31-1
	12 058 V1872106U2 Primary fresh-1 2 1	58	6510560	43940	148.1693	Prep Date	21-Jul-22	20-Sep-22
	12 059 V1872106U2 Primary fresh-1 3 1	59	6638070	45716	145.2023			
	12 060 V1872106U2 Primary fresh-2 1 1	60	6591301	44820	147.0616			
	12 061 V1872106U2 Primary fresh-2 2 1	61	6519385	45470	143.3777			
	12 062 V1872106U2 Primary fresh-2 3 1	62	6416012	45079	142.3282	Avg	153.1481	145.5369
	12 063 V1872106U2 Primary stored-1 1 1	63	7142353	44327	161.1287	Std Dev	7.50048	2.31139
	12 064 V1872106U2 Primary stored-1 2 1	64	7211902	46450	155.2616	%CV	4.9	1.6
	12 065 V1872106U2 Primary stored-1 3 1	65	7203453	45510	158.2829	%Change	5.2	
	12 066 V1872106U2 Primary stored-2 1 1	66	6547190	46825	139.8225			
	12 067 V1872106U2 Primary stored-2 2 1	67	6945315	45065	154.1177			
	12 068 V1872106U2 Primary stored-2 3 1	68	6751274	44926	150.2754	Duration	61	days

$$\%\text{Change} = \frac{\text{mean response of stored solutions} - \text{mean response of comparison solutions}}{\text{mean response of comparison solutions}} \times 100$$

Criteria: To be deemed stable, % Change upon storage for analyte must be within ±15.0% and %CV must be within ≤15.0%.
Meets Criteria: YES

Table 32: Stability of Zidebactam Spiking Solution in Deionized Water at Room Temperature

Run: 3
Container: Polypropylene

Analyte	Sample Name	Sequence Number	Analyte Peak Area	IS Peak Area	Peak Area Ratio		Bench Top	Refrigerated	-20 °C Reference
Zidebactam 300 mcg/mL	3 003 V1872106U2 Spike low -20°C-1 1 1	3	48648	71445	0.6809	Lot	V18721062U2-7-4 compared to Refr	V18721062U2-7-5 Compared to -20°C	V18721062U2-7-6
	3 004 V1872106U2 Spike low -20°C-1 2 1	4	47266	71530	0.6608				
	3 005 V1872106U2 Spike low -20°C-1 3 1	5	48604	71728	0.6776				
	3 006 V1872106U2 Spike low -20°C-2 1 1	6	46856	71893	0.6517				
	3 007 V1872106U2 Spike low -20°C-2 2 1	7	47254	72325	0.6534				
	3 008 V1872106U2 Spike low -20°C-2 3 1	8	47607	69761	0.6824	Avg	0.6592	0.6617	0.6678
	3 009 V1872106U2 Spike low Refr-1 1 1	9	46091	71404	0.6455	Std Dev	0.00742	0.01150	0.01413
	3 010 V1872106U2 Spike low Refr-1 2 1	10	47706	70985	0.6721	%CV	1.1	1.7	2.1
	3 011 V1872106U2 Spike low Refr-1 3 1	11	46231	70901	0.6521	%Change	-0.4	-0.9	N/A
	3 012 V1872106U2 Spike low Refr-2 1 1	12	47641	72348	0.6585				
	3 013 V1872106U2 Spike low Refr-2 2 1	13	48275	71681	0.6735				
	3 014 V1872106U2 Spike low Refr-2 3 1	14	47749	71398	0.6688	Duration	17.75 hours		
	3 015 V1872106U2 Spike low RT-1 1 1	15	46353	70274	0.6596				
	3 016 V1872106U2 Spike low RT-1 2 1	16	46100	71065	0.6487				
	3 017 V1872106U2 Spike low RT-1 3 1	17	46824	70742	0.6619				
	3 018 V1872106U2 Spike low RT-2 1 1	18	47068	70552	0.6671				
	3 019 V1872106U2 Spike low RT-2 2 1	19	46743	71676	0.6521				
	3 020 V1872106U2 Spike low RT-2 3 1	20	47764	71727	0.6659				

Criteria: To be deemed stable, % Change upon storage for analyte must be within ±15.0% and %CV must be within ≤15.0%.
Meets Criteria: YES

Table 32: Stability of Zidebactam Spiking Solution in Deionized Water at Room Temperature (Continued)

Run: 3
Container: Polypropylene

Analyte	Sample Name	Sequence Number	Analyte Peak Area	IS Peak Area	Peak Area Ratio		Bench Top	Refrigerated	-20 °C Reference
Zidebactam 30,000 mcg/mL	3 021 V1872106U2 Spike high -20°C-1 1 1	21	4572243	70263	65.0733	Lot	V18721062U2-8-1 compared to Refr	V18721062U2-8-2 Compared to -20°C	V18721062U2-8-3
	3 022 V1872106U2 Spike high -20°C-1 2 1	22	4491468	71708	62.6355				
	3 023 V1872106U2 Spike high -20°C-1 3 1	23	4518595	71162	63.4973				
	3 024 V1872106U2 Spike high -20°C-2 1 1	24	4459474	71160	62.6683				
	3 025 V1872106U2 Spike high -20°C-2 2 1	25	4567708	71493	63.8903				
	3 026 V1872106U2 Spike high -20°C-2 3 1	26	4443614	71038	62.5526	Avg	64.4876	64.0624	63.3862
	3 027 V1872106U2 Spike high Refr-1 1 1	27	4384160	71276	61.5096	Std Dev	0.65091	2.68461	0.98858
	3 028 V1872106U2 Spike high Refr-1 2 1	28	4294992	70371	61.0336	%CV	1.0	4.2	1.6
	3 029 V1872106U2 Spike high Refr-1 3 1	29	4491832	71960	62.4212	%Change	0.7	1.1	N/A
	3 030 V1872106U2 Spike high Refr-2 1 1	30	4673188	70197	66.5725				
	3 031 V1872106U2 Spike high Refr-2 2 1	31	4760824	71302	66.7699				
	3 032 V1872106U2 Spike high Refr-2 3 1	32	4733414	71645	66.0676	Duration	17.75 hours		
	3 033 V1872106U2 Spike high RT-1 1 1	33	4528619	70459	64.2731				
	3 034 V1872106U2 Spike high RT-1 2 1	34	4641357	73083	63.5080				
	3 035 V1872106U2 Spike high RT-1 3 1	35	4610917	71399	64.5796				
	3 036 V1872106U2 Spike high RT-2 1 1	36	4619486	71895	64.2532				
	3 037 V1872106U2 Spike high RT-2 2 1	37	4659281	71204	65.4357				
	3 038 V1872106U2 Spike high RT-2 3 1	38	4668585	71962	64.8757				

$$\%\text{Change} = \frac{\text{mean response of stored solutions} - \text{mean response of comparison solutions}}{\text{mean response of comparison solutions}} \times 100$$

Criteria: To be deemed stable, % Change upon storage for analyte must be within ±15.0% and %CV must be within ≤15.0%.
Meets Criteria: YES

Table 33: Stability of Zidebactam Spiking Solution in Deionized Water at -20 °C

Run: 9
Container: Polypropylene

Concentration: 300 mcg/mL									
Stability Data (Stored)					Comparison Data (Fresh)				
Lot #:	V1872106U2-4-1				Lot #:	V1872106U2-22-1			
Prepared:	21-Jul-2022				Prepared:	15-Aug-2022			
Sample ID	Seq #	Drug Peak Area	IS Peak Area	Peak Area Ratio	Sample ID	Seq #	Drug Peak Area	IS Peak Area	Peak Area Ratio
9 009 V1872106U2 Spike low stored-1 1 1	9	38263	53582	0.7141	9 003 V1872106U2 Spike low fresh-1 1 1	3	40256	54025	0.7451
9 010 V1872106U2 Spike low stored-1 2 1	10	38757	54465	0.7116	9 004 V1872106U2 Spike low fresh-1 2 1	4	39981	54394	0.7350
9 011 V1872106U2 Spike low stored-1 3 1	11	38892	54006	0.7201	9 005 V1872106U2 Spike low fresh-1 3 1	5	39548	53018	0.7459
9 012 V1872106U2 Spike low stored-2 1 1	12	38202	53365	0.7159	9 006 V1872106U2 Spike low fresh-2 1 1	6	40384	52849	0.7641
9 013 V1872106U2 Spike low stored-2 2 1	13	39876	54343	0.7338	9 007 V1872106U2 Spike low fresh-2 2 1	7	40030	54425	0.7355
9 014 V1872106U2 Spike low stored-2 3 1	14	37889	54140	0.6998	9 008 V1872106U2 Spike low fresh-2 3 1	8	39846	54579	0.7301
			Mean	0.7159				Mean	0.7426
			n	6				n	6
			SD	0.0111				SD	0.0122
			%CV	1.6				%CV	1.6
%Change =	-3.6								
Duration:	25	Days							

$$\%\text{Change} = \frac{\text{mean response of stored solutions} - \text{mean response of comparison solutions}}{\text{mean response of comparison solutions}} \times 100$$

Criteria: To be deemed stable, % Change upon storage for analyte must be within ±15.0% and %CV must be within ≤15.0%.
Meets Criteria: YES

Table 33: Stability of Zidebactam Spiking Solution in Deionized Water at -20 °C (Continued)

Run: 9
Container: Polypropylene

Concentration: 30,000 mcg/mL									
Stability Data (Stored)					Comparison Data (Fresh)				
Lot #:	V1872106U2-4-1				Lot #:	V1872106U2-22-1			
Prepared:	21-Jul-2022				Prepared:	15-Aug-2022			
Sample ID	Seq #	Drug Peak Area	IS Peak Area	Peak Area Ratio	Sample ID	Seq #	Drug Peak Area	IS Peak Area	Peak Area Ratio
9 021 V1872106U2 Spike high stored-1 1 1	21	3782495	54413	69.5145	9 015 V1872106U2 Spike high fresh-1 1 1	15	3759255	53829	69.8370
9 022 V1872106U2 Spike high stored-1 2 1	22	3741223	54289	68.9131	9 016 V1872106U2 Spike high fresh-1 2 1	16	3793491	54048	70.1874
9 023 V1872106U2 Spike high stored-1 3 1	23	3741273	54767	68.3125	9 017 V1872106U2 Spike high fresh-1 3 1	17	3762946	55443	67.8705
9 024 V1872106U2 Spike high stored-2 1 1	24	3519263	54803	64.2166	9 018 V1872106U2 Spike high fresh-2 1 1	18	3813334	55022	69.3056
9 025 V1872106U2 Spike high stored-2 2 1	25	3627002	54256	66.8498	9 019 V1872106U2 Spike high fresh-2 2 1	19	3775447	54443	69.3468
9 026 V1872106U2 Spike high stored-2 3 1	26	3591727	54910	65.4112	9 020 V1872106U2 Spike high fresh-2 3 1	20	3809358	53956	70.6012
			Mean	67.2030				Mean	69.5248
			n	6				n	6
			SD	2.0858				SD	0.9499
			%CV	3.1				%CV	1.4
%Change =	-3.3								
Duration:	25	Days							

$$\%\text{Change} = \frac{\text{mean response of stored solutions} - \text{mean response of comparison solutions}}{\text{mean response of comparison solutions}} \times 100$$

Criteria: To be deemed stable, % Change upon storage for analyte must be within ±15.0% and %CV must be within ≤15.0%.
Meets Criteria: YES

Table 33: Stability of Zidebactam Spiking Solution in Deionized Water at -20 °C (Continued)

Run: 12
Container: Polypropylene

Analyte	Sample Name	Sequence Number	Analyte Peak Area	IS Peak Area	Peak Area Ratio		Stored	Fresh
Zidebactam 30.0 mcg/mL	12 031 V1872106U2 Spike low fresh-1 1 1	31	61273	41966	1.4601	Lot	V1872106U2-4-1	V1872106U2-34-1
	12 032 V1872106U2 Spike low fresh-1 2 1	32	65893	43994	1.4978	Prep Date	21-Jul-22	20-Sep-22
	12 033 V1872106U2 Spike low fresh-1 3 1	33	65670	44788	1.4662			
	12 034 V1872106U2 Spike low fresh-2 1 1	34	62602	44627	1.4028			
	12 035 V1872106U2 Spike low fresh-2 2 1	35	61173	44881	1.3630			
	12 036 V1872106U2 Spike low fresh-2 3 1	36	63454	44837	1.4152	Avg	1.2110	1.4342
	12 037 V1872106U2 Spike low stored-1 1 1	37	56861	44926	1.2657	Std Dev	0.05267	0.04930
	12 038 V1872106U2 Spike low stored-1 2 1	38	57797	44849	1.2887	%CV	4.3	3.4
	12 039 V1872106U2 Spike low stored-1 3 1	39	55195	46147	1.1961	%Change	-15.6	
	12 040 V1872106U2 Spike low stored-2 1 1	40	52701	44774	1.1770			
	12 041 V1872106U2 Spike low stored-2 2 1	41	53169	45499	1.1686			
	12 042 V1872106U2 Spike low stored-2 3 1	42	52331	44722	1.1701	Duration	61	days
Zidebactam 3,000 mcg/mL	12 044 V1872106U2 Spike high fresh-1 1 1	44	5070025	44066	115.0553	Lot	V1872106U2-4-1	V1872106U2-34-1
	12 045 V1872106U2 Spike high fresh-1 2 1	45	5297563	44976	117.7864	Prep Date	21-Jul-22	20-Sep-22
	12 046 V1872106U2 Spike high fresh-1 3 1	46	5549046	42693	129.9755			
	12 047 V1872106U2 Spike high fresh-2 1 1	47	5610729	43504	128.9704			
	12 048 V1872106U2 Spike high fresh-2 2 1	48	4763342	43658	109.1058			
	12 049 V1872106U2 Spike high fresh-2 3 1	49	5160410	43236	119.3545	Avg	111.6635	120.0413
	12 050 V1872106U2 Spike high stored-1 1 1	50	5001460	45790	109.2260	Std Dev	6.65019	8.10456
	12 051 V1872106U2 Spike high stored-1 2 1	51	4952186	44738	110.6931	%CV	6.0	6.8
	12 052 V1872106U2 Spike high stored-1 3 1	52	5187790	44557	116.4304	%Change	-7.0	
	12 053 V1872106U2 Spike high stored-2 1 1	53	4618099	45003	102.6176			
	12 054 V1872106U2 Spike high stored-2 2 1	54	4955409	45389	109.1764			
	12 055 V1872106U2 Spike high stored-2 3 1	55	5328551	43735	121.8372	Duration	61	days

$$\%\text{Change} = \frac{\text{mean response of stored solutions} - \text{mean response of comparison solutions}}{\text{mean response of comparison solutions}} \times 100$$

Criteria: To be deemed stable, % Change upon storage for analyte must be within ±15.0% and %CV must be within ≤15.0%.
Meets Criteria: YES for the high concentration, but not at the low concentration. Spikes should only be used for the 25 days established previously.

Table 34: Stability of Cefotaxime Working Internal Standard Solution in Deionized Water at Room Temperature

Run: V1872106U1 Run 3
Container: Glass

Analyte	Sample Name	Sequence Number	Analyte Peak Area	IS Peak Area	Peak Area Ratio		Bench Top	Refrigerated	-20 °C Reference
Cefotaxime	3 059 V1872106U1 WIS -20°C-1 1 1	59	1313192	485422	2.7053	Lot	V1872106U1-11-1	V1872106U1-11-2	V1872106U1-11-3
~500 mcg/mL	3 060 V1872106U1 WIS -20°C-1 3 1	60	1339652	522363	2.5646		compared to Refr	Compared to -20°C	
	3 061 V1872106U1 WIS -20°C-1 2 1	61	1340502	550053	2.4370				
	3 062 V1872106U1 WIS -20°C-2 2 1	62	1336674	522445	2.5585				
	3 063 V1872106U1 WIS -20°C-2 3 1	63	1341412	560588	2.3929				
	3 064 V1872106U1 WIS -20°C-2 1 1	64	1343379	587756	2.2856	Avg	2.5534	2.7884	2.4906
	3 065 V1872106U1 WIS Refr-1 1 1	65	1335207	450429	2.9643	Std Dev	0.129	0.232	0.149
	3 066 V1872106U1 WIS Refr-1 3 1	66	1348441	467684	2.8832	%CV	5.0	8.3	6.0
	3 067 V1872106U1 WIS Refr-1 2 1	67	1335092	554007	2.4099	%Change	-8.4	12.0	N/A
	3 068 V1872106U1 WIS Refr-2 2 1	68	1321946	453246	2.9166				
	3 069 V1872106U1 WIS Refr-2 3 1	69	1326727	447448	2.9651				
	3 070 V1872106U1 WIS Refr-2 1 1	70	1335122	515294	2.5910	Duration	15.5 hours		
	3 071 V1872106U1 WIS RT-1 1 1	71	1336567	547268	2.4423				
	3 072 V1872106U1 WIS RT-1 2 1	72	1341696	504955	2.6571				
	3 073 V1872106U1 WIS RT-1 3 1	73	1329061	557140	2.3855				
	3 074 V1872106U1 WIS RT-2 3 1	74	1340202	529252	2.5323				
	3 075 V1872106U1 WIS RT-2 2 1	75	1319308	483530	2.7285				
	3 076 V1872106U1 WIS RT-2 1 1	76	1347636	523394	2.5748				

$$\%\text{Change} = \frac{\text{mean response of stored solutions} - \text{mean response of comparison solutions}}{\text{mean response of comparison solutions}} \times 100$$

Criteria: To be deemed stable, % Change upon storage for analyte must be within ±20.0% and %CV must be within ≤15.0%.
Meets Criteria: YES

17. FIGURES

Figure 1: Typical Calibration Curve

Analytical Run 2 Run Acceptance Parameters for Zidebactam (µg/mL) (Study V1872106U2)

Regression Type	LINEAR
Weighting Factor	1/X**2
Slope	0.013884720909235
Intercept	-0.019467987546366200
R-Squared	0.995354791822719
LLOQ	10
ULOQ	4000
Mean Std %Diff	5.0
S.D. Std %Diff	3.1
Mean Std Response	0.013493
S.D. Std Response	0.001087

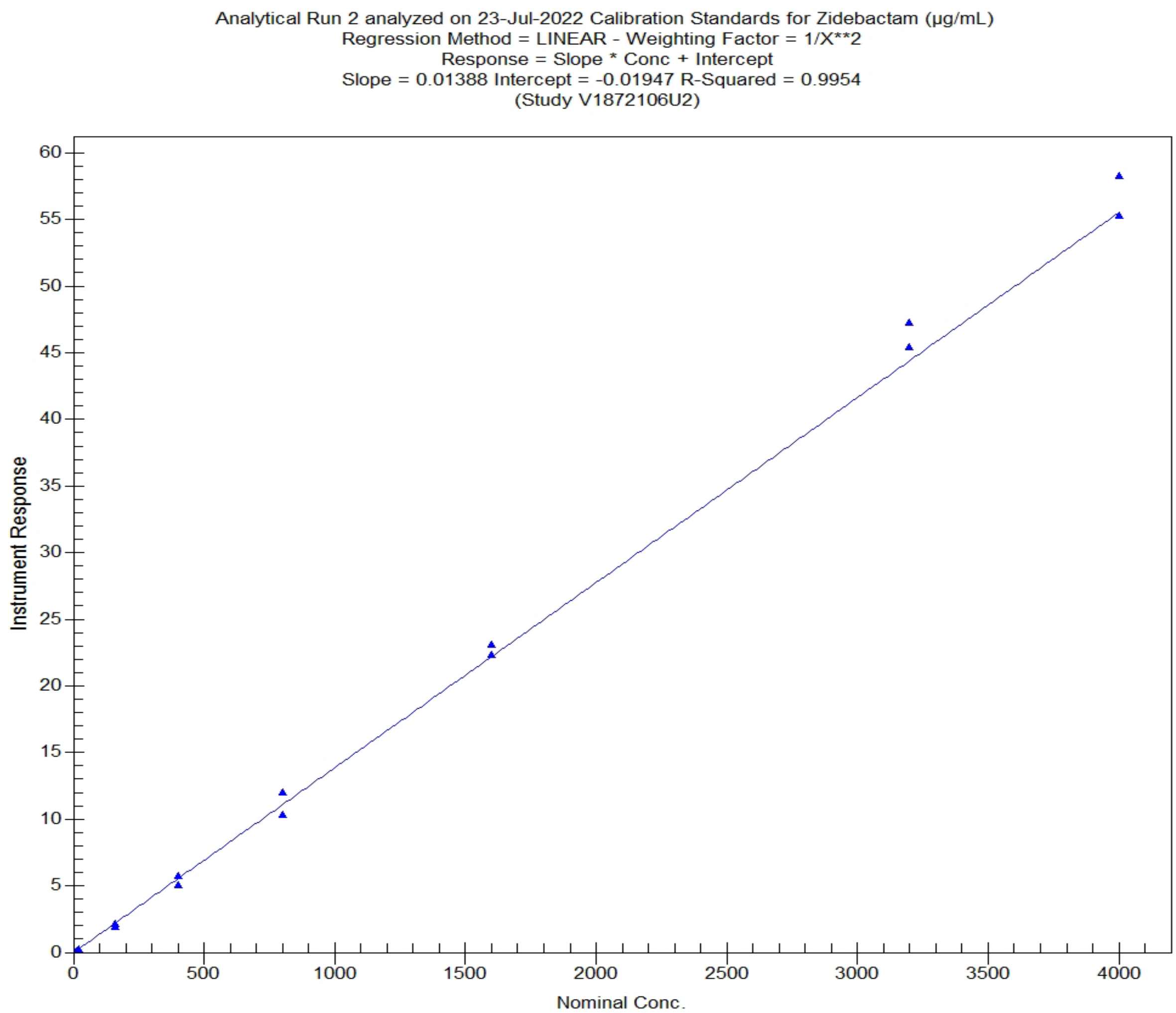

Figure 2: Typical Ion Chromatogram Blank Human Urine Without Internal Standard

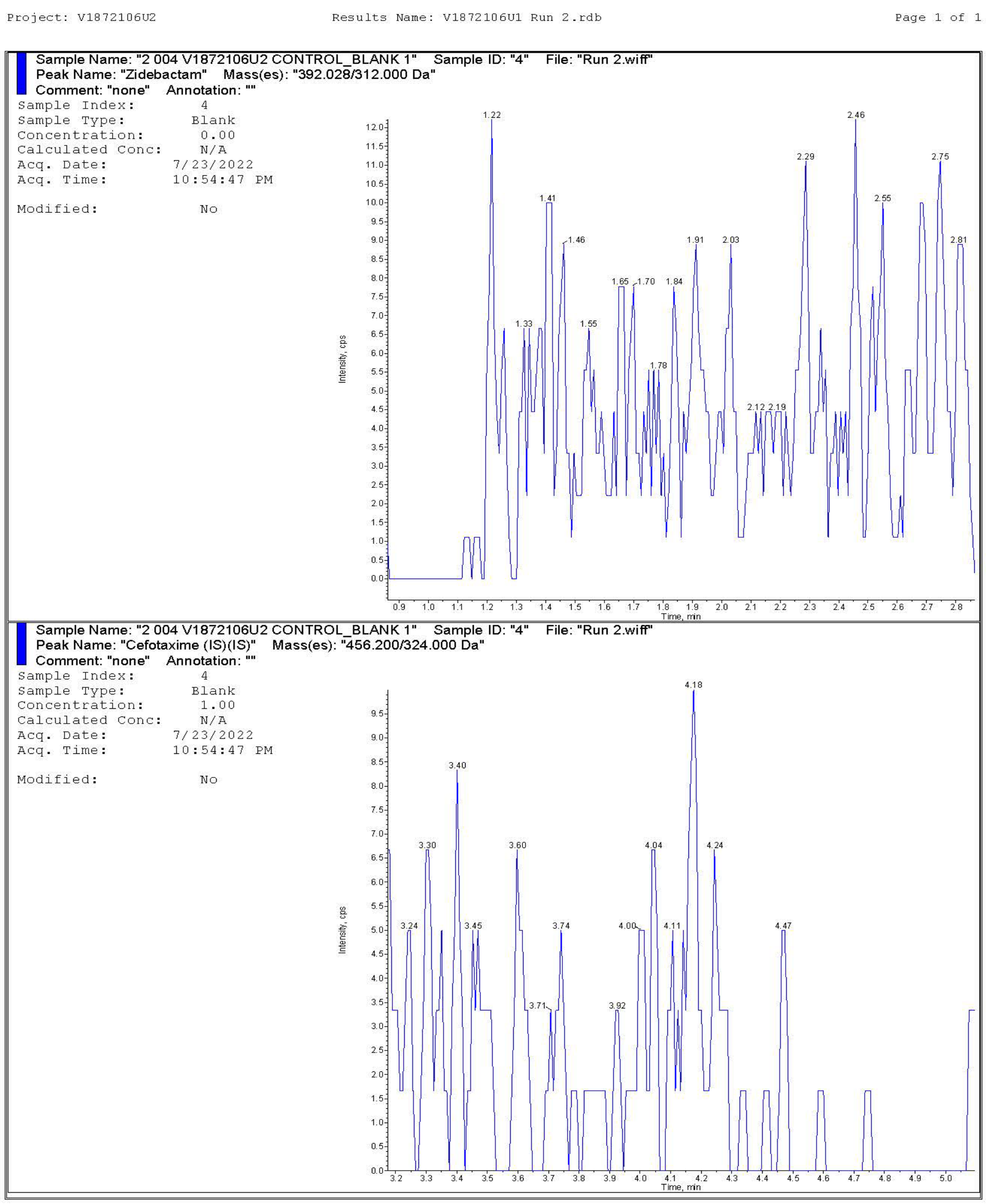

Figure 3: Typical Ion Chromatogram Blank Human Urine with Internal Standard

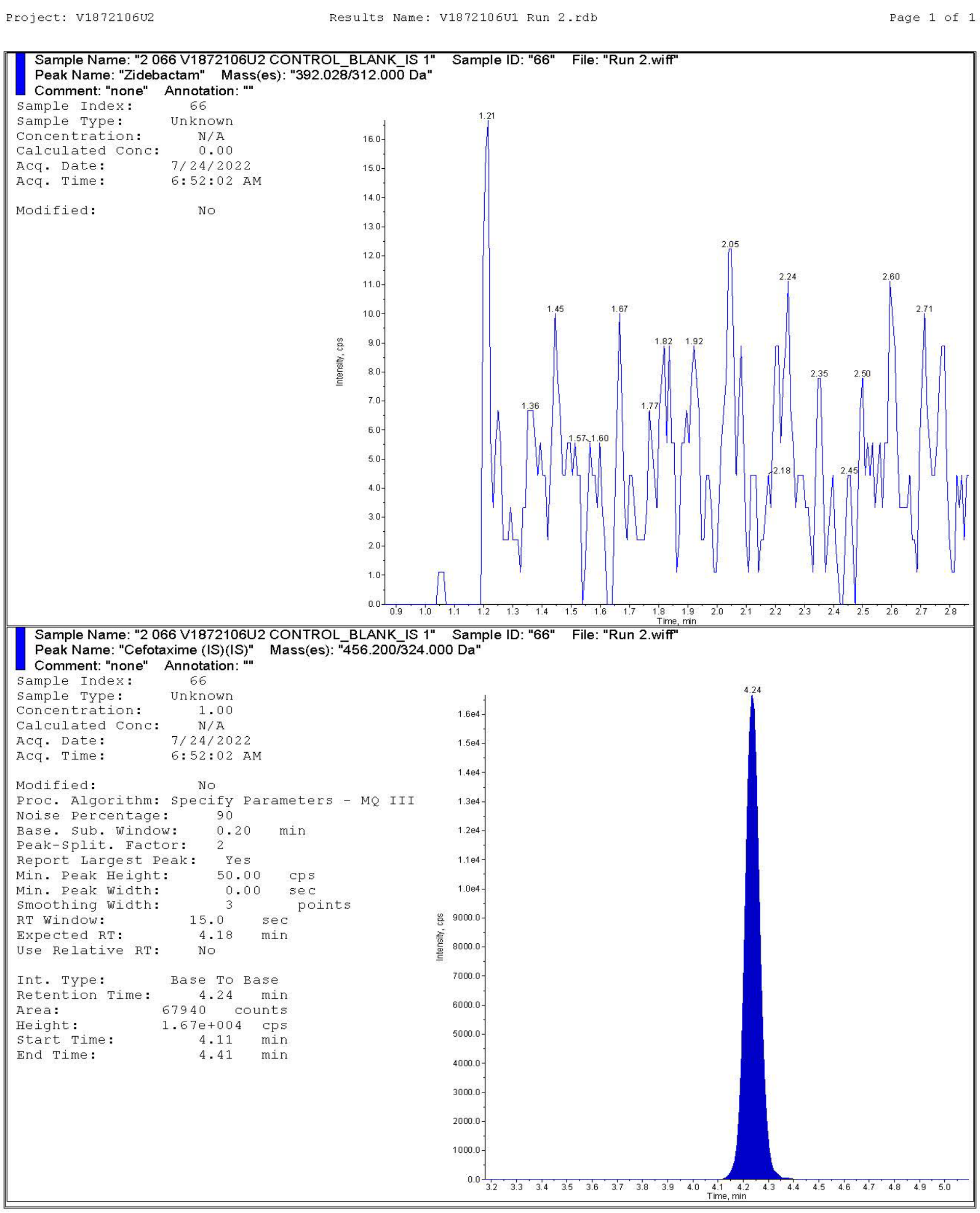

Figure 4: Typical Ion Chromatogram LLOQ Calibration Standard, Zidebactam = 10.0 mcg/mL

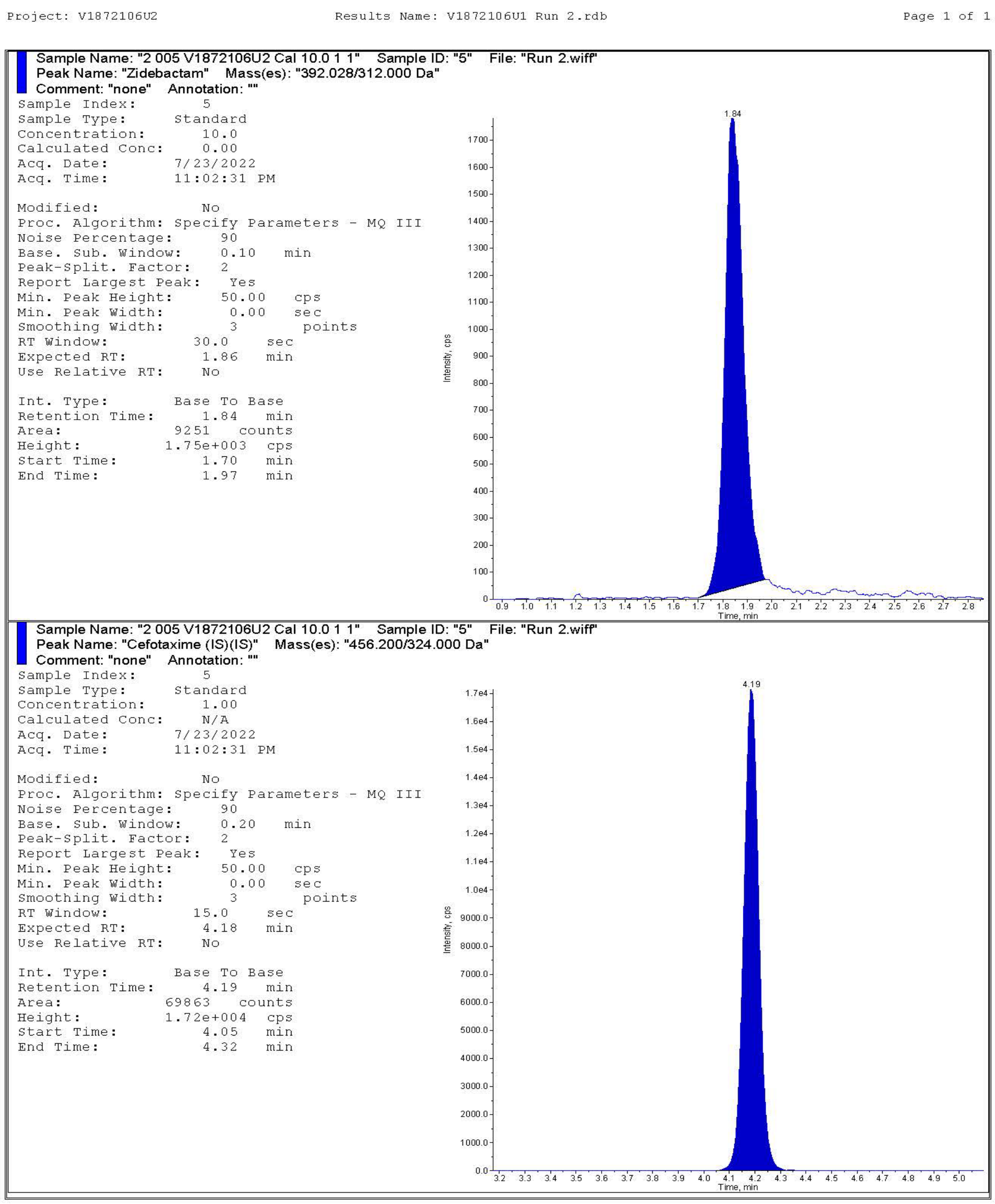

Figure 5: Typical Ion Chromatogram Calibration Standard, Zidebactam = 1600 mcg/mL

Project: V1872106U2 Results Name: V1872106U1 Run 2.rdb Page 1 of 1

Sample Name: "2 010 V1872106U2 Cal 1600 1 1" Sample ID: "10" File: "Run 2.wiff"
Peak Name: "Zidebactam" Mass(es): "392.028/312.000 Da"
Comment: "none" Annotation: ""

```
Sample Index:        10
Sample Type:         Standard
Concentration:       1600.
Calculated Conc:     0.00
Acq. Date:           7/23/2022
Acq. Time:           11:41:01 PM

Modified:            No
Proc. Algorithm: Specify Parameters - MQ III
Noise Percentage:    90
Base. Sub. Window:   0.10    min
Peak-Split. Factor:  2
Report Largest Peak: Yes
Min. Peak Height:    50.00   cps
Min. Peak Width:     0.00    sec
Smoothing Width:     3       points
RT Window:           30.0    sec
Expected RT:         1.86    min
Use Relative RT:     No

Int. Type:           Base To Base
Retention Time:      1.89    min
Area:                1635512 counts
Height:              3.29e+005 cps
Start Time:          1.73    min
End Time:            2.13    min
```

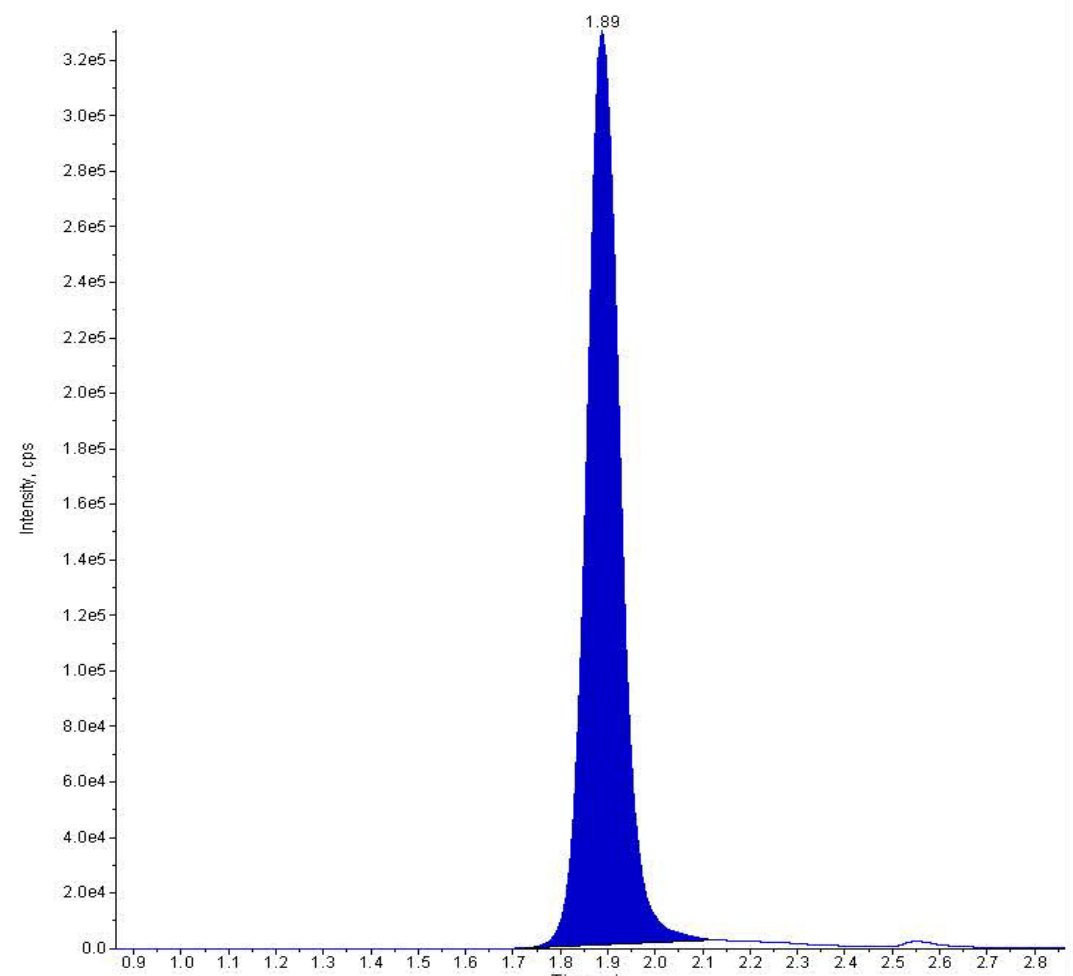

Sample Name: "2 010 V1872106U2 Cal 1600 1 1" Sample ID: "10" File: "Run 2.wiff"
Peak Name: "Cefotaxime (IS)(IS)" Mass(es): "456.200/324.000 Da"
Comment: "none" Annotation: ""

```
Sample Index:        10
Sample Type:         Standard
Concentration:       1.00
Calculated Conc:     N/A
Acq. Date:           7/23/2022
Acq. Time:           11:41:01 PM

Modified:            No
Proc. Algorithm: Specify Parameters - MQ III
Noise Percentage:    90
Base. Sub. Window:   0.20    min
Peak-Split. Factor:  2
Report Largest Peak: Yes
Min. Peak Height:    50.00   cps
Min. Peak Width:     0.00    sec
Smoothing Width:     3       points
RT Window:           15.0    sec
Expected RT:         4.18    min
Use Relative RT:     No

Int. Type:           Base To Base
Retention Time:      4.20    min
Area:                70718   counts
Height:              1.79e+004 cps
Start Time:          4.06    min
End Time:            4.34    min
```

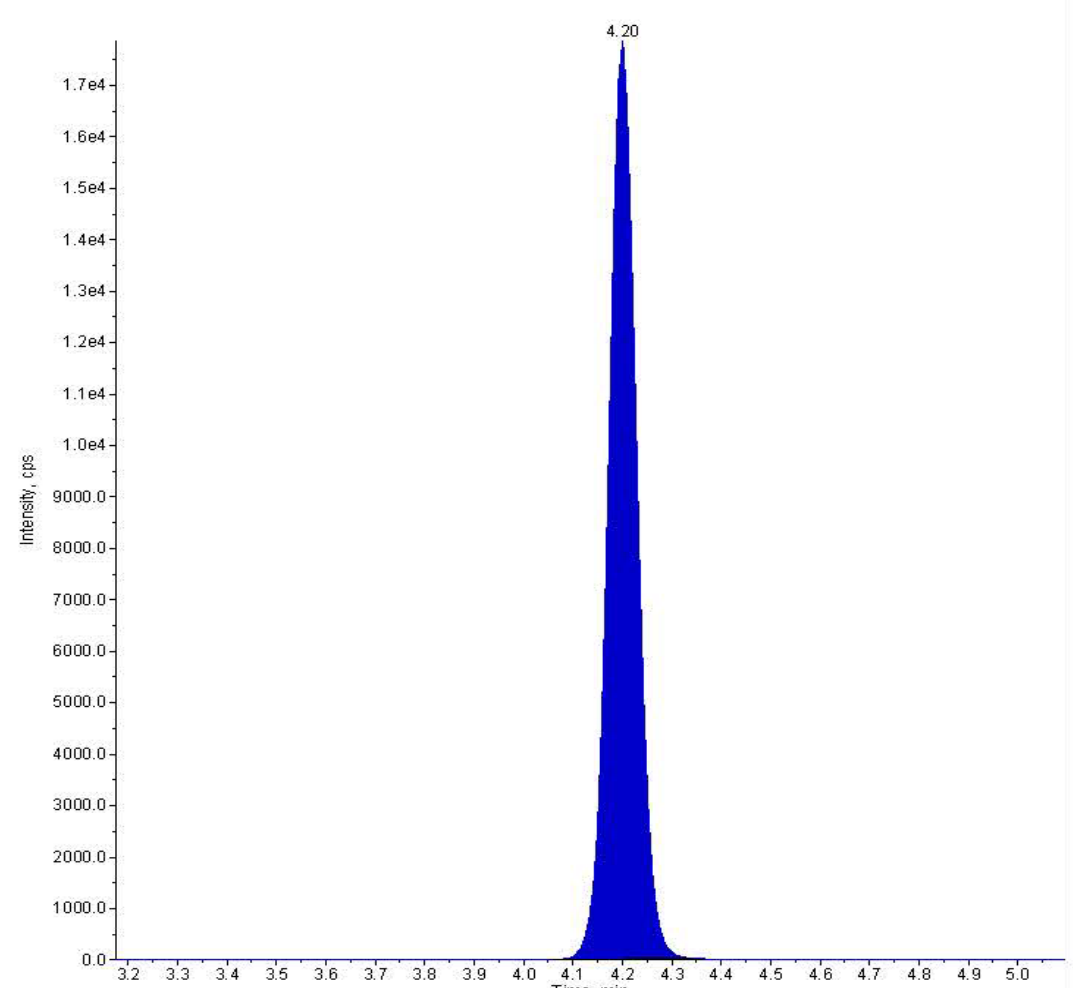

Figure 6: Typical Ion Chromatogram ULOQ Calibration Standard, Zidebactam = 4000 mcg/mL

Project: V1872106U2 Results Name: V1872106U1 Run 2.rdb Page 1 of 1

Sample Name: "2 075 V1872106U2 Cal 4000 2 1" Sample ID: "75" File: "Run 2.wiff"
Peak Name: "Zidebactam" Mass(es): "392.028/312.000 Da"
Comment: "none" Annotation: ""

```
Sample Index:        75
Sample Type:         Standard
Concentration:       4000.
Calculated Conc:     0.00
Acq. Date:           7/24/2022
Acq. Time:           8:01:25 AM

Modified:            No
Proc. Algorithm: Specify Parameters - MQ III
Noise Percentage:    90
Base. Sub. Window:   0.10   min
Peak-Split. Factor:  2
Report Largest Peak: Yes
Min. Peak Height:    50.00  cps
Min. Peak Width:     0.00   sec
Smoothing Width:     3      points
RT Window:           30.0   sec
Expected RT:         1.86   min
Use Relative RT:     No

Int. Type:           Base To Base
Retention Time:      1.95   min
Area:                3789070  counts
Height:              8.23e+005  cps
Start Time:          1.78   min
End Time:            2.22   min
```

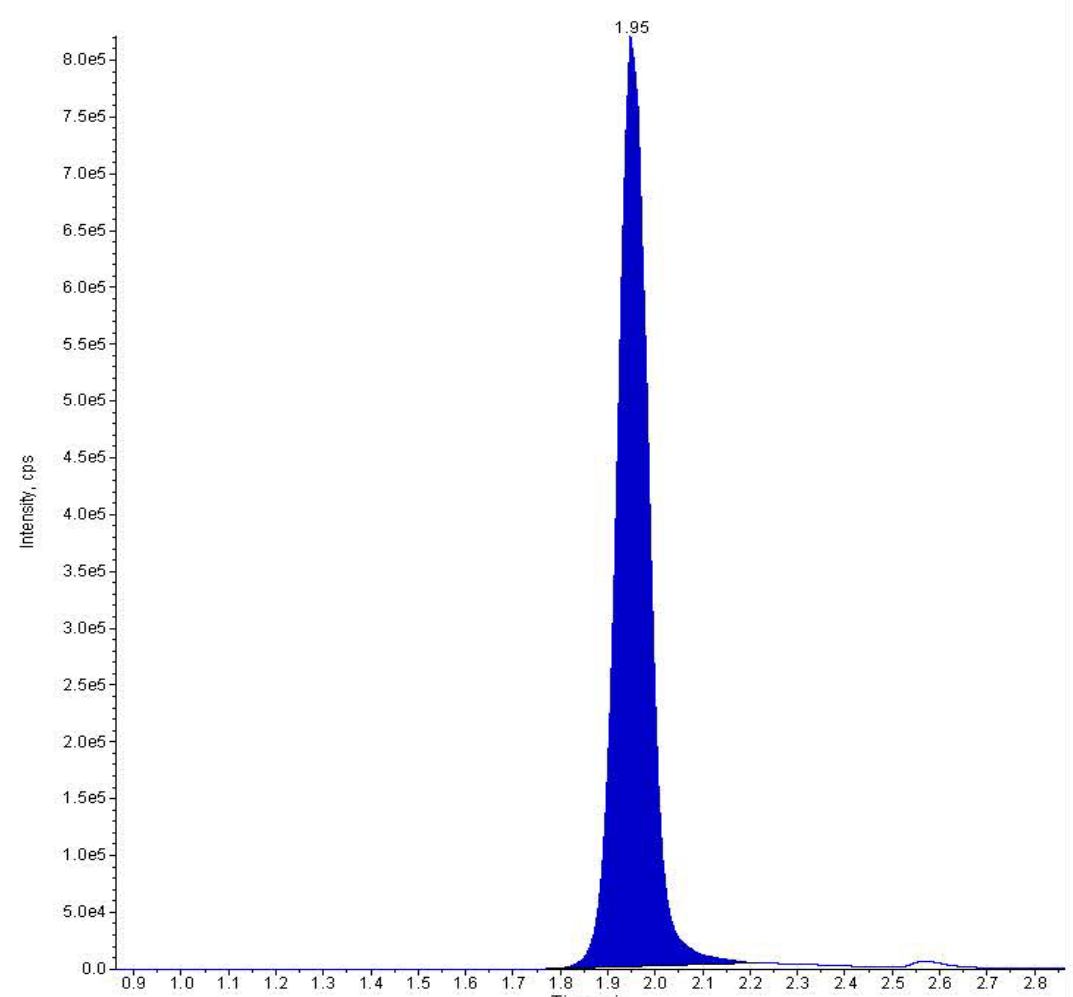

Sample Name: "2 075 V1872106U2 Cal 4000 2 1" Sample ID: "75" File: "Run 2.wiff"
Peak Name: "Cefotaxime (IS)(IS)" Mass(es): "456.200/324.000 Da"
Comment: "none" Annotation: ""

```
Sample Index:        75
Sample Type:         Standard
Concentration:       1.00
Calculated Conc:     N/A
Acq. Date:           7/24/2022
Acq. Time:           8:01:25 AM

Modified:            No
Proc. Algorithm: Specify Parameters - MQ III
Noise Percentage:    90
Base. Sub. Window:   0.20   min
Peak-Split. Factor:  2
Report Largest Peak: Yes
Min. Peak Height:    50.00  cps
Min. Peak Width:     0.00   sec
Smoothing Width:     3      points
RT Window:           15.0   sec
Expected RT:         4.18   min
Use Relative RT:     No

Int. Type:           Base To Base
Retention Time:      4.23   min
Area:                68500  counts
Height:              1.66e+004  cps
Start Time:          4.12   min
End Time:            4.39   min
```

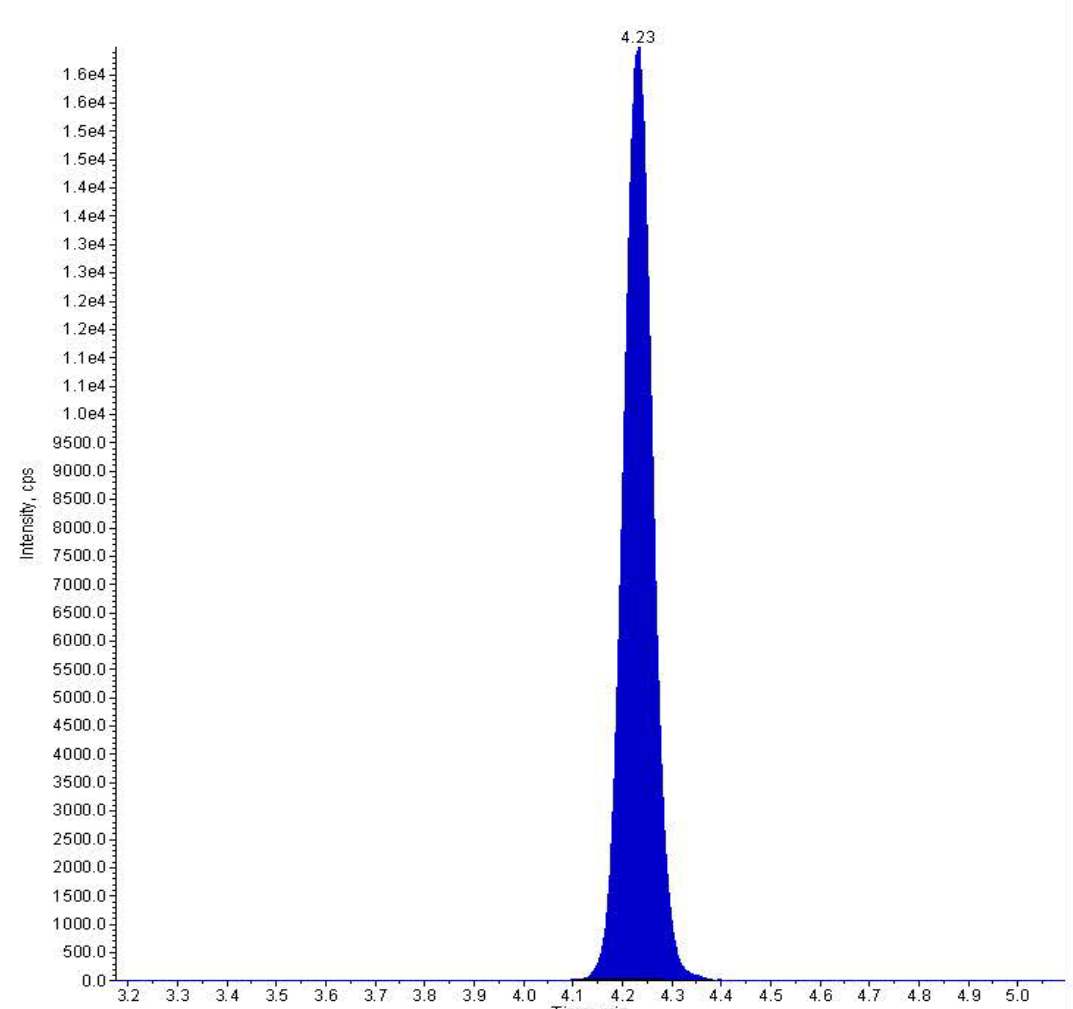

Title: **LC-MS/MS Analysis of Zidebactam in Treated Human Urine (50 mcL, 10.0 - 4000 mcg/mL in Urine only)**
Validation Number: V1872106U2

1. GENERAL INFORMATION

1.1. Client Name: Dynport Vaccine Company

1.2. Reference: Validation V1872106U2

2. TABLE OF CONTENTS

1. GENERAL INFORMATION 1
1.1. Client Name: Dynport Vaccine Company 1
1.2. Reference: Validation V1872106U2 1
2. TABLE OF CONTENTS 1
3. SAMPLE REQUIREMENTS 3
4. METHOD SUMMARY 3
5. SPECIAL HANDLING OF BIOLOGICAL SAMPLES 3
6. DATA COLLECTION AND ANALYSIS SOFTWARE 3
7. INSTRUMENTS, EQUIPMENT, AND CONDITIONS 3
7.1. LC and MS Conditions 4
Table 1: HPLC System 4
7.1.1. Gradient Table 5
7.1.2. Autosampler Settings 5
7.1.3. Mass Spectrometer System 5
8. REAGENTS, STANDARDS, AND SUPPLIES 7
Table 2: Reagents, Standards, and Supplies 7
9. SAFETY 7
10. PROCEDURE 8
10.1. Preparation of Analyte Stock Solutions and Dilutions 8

CONFIDENTIAL

Serial Number: 909413cc-045a-4ce7-a36e-9ddc32d52f79 accessed by Anika Pippin (PIPPINA) on Tue, 29 Nov 2022 00:21:08 GMT.

10.1.1. Zidebactam Primary Solution, 160 mg/mL 8
10.1.2. Working Internal Standard Solution, ~500 mcg/mL Cefotaxime 8
10.2. Preparation of Other Reagents 9
10.2.1. Mobile Phase A: 10 mM Ammonium Acetate in Deionized Water 9
10.2.2. Mobile Phase B: 95/5 Acetonitrile / 10mM Ammonium Acetate 9
10.2.3. Rinse: 90/10 Acetone/Deionized Water 9
10.2.4. 1 N Sodium Hydroxide (NaOH) in Deionized Water 9
10.2.5. MES (2-(N-Morpholino) Ethanesulphonic Acid Hydrate) Buffer pH 6.4, ~0.1M....9
10.2.6. 0.1% Formic Acid in Deionized Water 9
10.3. Preparation of Standards and QC Samples 10
10.3.1. Preparation of Standard and QC Spiking Solutions 10
10.3.1. Treated Human Urine (Human Urine: MES Buffer 1:1) 10
10.3.2. Preparation of Standards and QC Samples in Treated Human Urine 10
10.4. Extraction Procedure 11
10.5. Dilutions 11
10.6. Calculations 12
11. REFERENCE CHROMATOGRAMS 12
Figure 1: Blank without IS 12
Figure 2: Blank with IS 13
Figure 3: Carryover Blank with IS 13
Figure 4: LLOQ – Zidebactam 10.0 mcg/mL 14
12. REVISION HISTORY 14
Table 3: Revision History 14

CONFIDENTIAL

Serial Number: 909413cc-045a-4ce7-a36e-9ddc32d52f79 accessed by Anika Pippin (PIPPINA) on Tue, 29 Nov 2022 00:21:08 GMT.

3. SAMPLE REQUIREMENTS

Human urine samples (treated with 1:1 MES Buffer pH 6.4, ~0.1M), minimum volume 50 mcL each, for determination of zidebactam concentration.

Refer to the relevant study protocol for a description of expected samples.

4. METHOD SUMMARY

The method involves 96-well protein precipitation followed by chromatographic separation and tandem MS detection using the Sciex 5000 LC/MS/MS system. The concentration range is 10.0 - 4000 mcg/mL in human urine only. Since unknown samples are treated with the MES buffer after collection at a 1:1 ratio, the calibration standard and QC concentrations are based on the urine volume only. Unknown samples do not need to be adjusted for the dilution with MES buffer.

5. SPECIAL HANDLING OF BIOLOGICAL SAMPLES

The method requires no special handling. Please ensure all "Universal Precautions" are taken.

6. DATA COLLECTION AND ANALYSIS SOFTWARE

**Description**	**Version**
Watson LIMS™ (Thermo Fisher Scientific)	7.5 or higher
Analyst® (AB Sciex)	1.7.2 or higher

7. INSTRUMENTS, EQUIPMENT, AND CONDITIONS

Equivalent equipment can be substituted, except where noted in Section 7.1. Minor modifications of conditions are permitted to optimize chromatography, except where noted in Section 7.1. Refer to SOP-00073 for allowable minor modifications that do not require validation.

CONFIDENTIAL

Serial Number: 909413cc-045a-4ce7-a36e-9ddc32d52f79 accessed by Anika Pippin (PIPPINA) on Tue, 29 Nov 2022 00:21:08 GMT.

7.1. LC and MS Conditions

Table 1: HPLC System

**System Controller**	**Shimadzu Controller, CBM20A**
Pump	Shimadzu LC-30AD
Autosampler	Shimadzu SIL-30AC
Column Oven	Shimadzu CTO-30A
MS Interface	ESI
Analytical Column	Fortis Technology Ltd. Fortis C18 4.6 x 50mm, 5 µ
Column Guard Cartridge	Security Guard Cartridge AQ C18, 4.0 x 2.0 mm
Column Temperature (set at)	35°C
Mobile Phase A	10 mM Ammonium Acetate in Deionized Water
Mobile Phase B	95/5 Acetonitrile / 10 mM Ammonium Acetate
Needle Wash Solvent (R0, R3)	90/10 Acetone /Deionized Water
Total Run Time (approximate)	7.9 min
Injection Volume (µl)	3-10 mcL
Flow Rate	1.0 mL/min
Pressure, kgf/cm2 (approximate)	~250
Approximate Retention Time (min.)	Zidebactam: 1.70
	Cefotaxime: 4.00

CONFIDENTIAL

Serial Number: 909413cc-045a-4ce7-a36e-9ddc32d52f79 accessed by Anika Pippin (PIPPINA) on Tue, 29 Nov 2022 00:21:08 GMT.

7.1.1. Gradient Table

Time Program

Time	Module	Events	Parameter
1.20	Pumps	Pump B Conc.	2
1.20	Controller	Event 1*	
2.80	Pumps	Pump B Conc.	16
3.80	Pumps	Pump B Conc.	16
4.50	Pumps	Pump B Conc.	100
4.50	Controller	Event 2*	
5.80	Pumps	Pump B Conc.	100
6.00	Pumps	Pump B Conc.	2
7.00	System Controller	Stop	

* Mobile phase flow is directed to the mass spectrometer from 1.20 min (Event 1) to 4.50 min (Event 2). The rest of the gradient time, the mobile phase flow goes to waste.

7.1.2. Autosampler Settings

Temperature: Set at 4°C
Rinse Type: External
Rinse Mode: Before and after aspiration
Rinse Dip Time: 0 sec
Rinse Method: Rinse Pump Then Port
Rinse Time: 2 sec
Rinsing Speed: 35 µL/sec
Rinsing Volume: 500 µL

7.1.3. Mass Spectrometer System

Mass Spectrometer Sciex 5000
Source Type: Turbo spray
Scan Type: MRM (MRM)
Polarity: Positive
Resolution Q1: Unit
Resolution Q3: Unit

CONFIDENTIAL

Serial Number: 909413cc-045a-4ce7-a36e-9ddc32d52f79 accessed by Anika Pippin (PIPPINA) on Tue, 29 Nov 2022 00:21:08 GMT.

All the below parameters in this section may be adjusted to optimize the instrument conditions.

Zidebactam:

Q1 Mass (Da)	Q3 Mass (Da)	Dwell (msec)
392.028	312.000	300.00
DP	66.00	
CE	21.00	
CXP	16.00	

Cefotaxime (IS):

Q1 Mass (Da)	Q3 Mass (Da)	Dwell (msec)
456.200	324.000	200.00
DP	50.00	
CE	33.00	
CXP	10.00	

Parameter Table:

CAD: 12.00

CUR: 40.00

GS1: 50.00

GS2: 60.00

IS: 5500

TEM: 600

EP 12

CONFIDENTIAL

Serial Number: 909413cc-045a-4ce7-a36e-9ddc32d52f79 accessed by Anika Pippin (PIPPINA) on Tue, 29 Nov 2022 00:21:08 GMT.

8. REAGENTS, STANDARDS, AND SUPPLIES

Reagents from alternate supplier and equivalent grade can be substituted.

Table 2: Reagents, Standards, and Supplies

Chemical / Grade	Manufacturer/Supplier
Zidebactam	Performa
Cefotaxime Sodium	Sigma Aldrich
MES ([2-(N-Morpholino) ethane sulphonic acid hydrate]) buffer	Sigma
Acetonitrile, HPLC grade	Honeywell
Formic Acid, 98+%	EMD Millipore
Deionized Water	Millipore
Ammonium Acetate ≥ 99.9%	Sigma
Methanol	Honeywell
Acetone	Honeywell
Human Urine	In house
Advantage SiliGuard Coated 2 mL 96 well Polypropylene plate: Square Top, Round Bottom	Analytical Sales and Services, Catalog # 27P687GC
Purple, pre-slit, square well, soft silicone/PTFE Cap Mats	Analytical Sales & Services, Inc., Catalog# 965085

9. SAFETY

Always observe safe laboratory practices. Consult the appropriate SDS before handling any chemical if you are unfamiliar with the proper safety precautions.

CONFIDENTIAL

Serial Number: 909413cc-045a-4ce7-a36e-9ddc32d52f79 accessed by Anika Pippin (PIPPINA) on Tue, 29 Nov 2022 00:21:08 GMT.

10. PROCEDURE

10.1. Preparation of Analyte Stock Solutions and Dilutions

The quantities of analyte solution prepared may be scaled up or down proportionally as needed. Alternate dilution schemes can be used to achieve the appropriate final concentrations of standards, QCs, working internal standard, and system verification solution (SVS). For assignment of expiration dates to analyte stocks and their dilutions, refer to the latest version of the validation report.

10.1.1. Zidebactam Primary Solution, 160 mg/mL

Check drying conditions prior to use. Weigh approximately 800 mg of zidebactam reference standard (correcting for purity and correction factor if necessary) in a glass container and add 5.0 mL of deionized water. Mix well and sonicate if necessary. Store solution at -20°C in a glass container.

10.1.2. Working Internal Standard Solution, ~500 mcg/mL Cefotaxime

Check drying conditions prior to use. Weigh 100 mg of cefotaxime reference standard. Add 200 mL of deionized water (do not correct for conversion factor and purity). Mix well and sonicate if necessary. Store at or below -20 °C in a glass container.

CONFIDENTIAL

Serial Number: 909413cc-045a-4ce7-a36e-9ddc32d52f79 accessed by Anika Pippin (PIPPINA) on Tue, 29 Nov 2022 00:21:08 GMT.

10.2. Preparation of Other Reagents

Prepared quantities of all reagents may be scaled up or down as necessary. Volumes provided are examples. For assignment of expiration dates, refer to SOP-00094.

10.2.1. Mobile Phase A: 10 mM Ammonium Acetate in Deionized Water

Weigh ~3.08 g of ammonium acetate (F.W. 77.08) and transfer to a 4-L volumetric flask. Dilute to volume with deionized water. Mix well and store at room temperature.

10.2.2. Mobile Phase B: 95/5 Acetonitrile / 10mM Ammonium Acetate

Combine 3800 mL of acetonitrile with 200 mL of 10 mM ammonium acetate in a 4-L flask. Mix well and store at room temperature.

10.2.3. Rinse: 90/10 Acetone/Deionized Water

Combine 900 mL of acetone and 100 mL of deionized water in a 1-L flask. Mix well and store at room temperature.

10.2.4. 1 N Sodium Hydroxide (NaOH) in Deionized Water

Weight ~ 4.00 g of sodium hydroxide (F.W. 40.00) and gradually transfer it to a 100-mL volumetric flask containing ~50 mL of deionized water. Mix to dissolve and wait until it reaches room temperature. Dilute to volume with deionized water and mix well. Store in a polypropylene container at room temperature.

10.2.5. MES (2-(N-Morpholino) Ethanesulphonic Acid Hydrate) Buffer pH 6.4, ~0.1M

Weigh ~ 4000 mg of MES hydrate (F.W. 195.24) and transfer to a 200-mL volumetric flask containing ~150 mL of deionized water. Dissolve and adjust the pH to 6.4 with 1N NaOH. Dilute to volume with deionized water. Mix well and store refrigerated.

10.2.6. 0.1% Formic Acid in Deionized Water

Combine 800 mL of acetonitrile and 200 mL of deionized water in a 1-L flask. Mix well and store at room temperature.

CONFIDENTIAL

Serial Number: 909413cc-045a-4ce7-a36e-9ddc32d52f79 accessed by Anika Pippin (PIPPINA) on Tue, 29 Nov 2022 00:21:08 GMT.

10.3. Preparation of Standards and QC Samples

10.3.1. Preparation of Standard and QC Spiking Solutions

Starting from the zidebactam primary solution (160 mg/mL) prepare a series of zidebactam spiking solutions in deionized water on wet ice. The following concentrations are recommended but can be altered as necessary: For standards: 40000, 32000, 16000, 8000, 4000, 1600, 200, 100 mcg/mL. For QC spiking solutions: 30000, 12000, 300 mcg/mL, and 100 mcg/mL for the LLOQ QC when needed. Store solutions in polypropylene at -20°C.

10.3.1. Treated Human Urine (Human Urine: MES Buffer 1:1)

Add 200 mL of chilled human urine to a polypropylene container containing 200 mL of chilled 0.1M MES Buffer, pH 6.4. Mix well and store at -20°C.

10.3.2. Preparation of Standards and QC Samples in Treated Human Urine

Use treated human urine that has been diluted 1:1 with MES buffer screened for the lack of interference at the retention times of zidebactam and cefotaxime (IS). Dilute each spiking solution by a factor of 20 into treated human urine on wet ice and mix well. For example, combine 0.500 mL of spiking solution and 9.5 mL of treated human urine. Since unknown samples are treated with the MES buffer after collection at a 1:1 ratio, the calibration standard and QC concentrations are based on the urine volume only. Unknown samples do not need to be adjusted for the dilution with MES buffer. The prepared concentrations in urine only, not accounting for MES buffer, will be as follows: For standards: 4000, 3200, 1600, 800, 400, 160, 20.0, and 10.0 mcg/mL. For QCs: 3000, 1200, and 30.0 mcg/mL, and 10.0 mcg/mL for the LLOQ QC when needed. Other concentrations may be prepared, provided they meet the requirements of SOP-00035 and provided they remain within the range of 10.0 to 4000 mcg/mL. Store solutions in polypropylene at -70°C or -20°C.

CONFIDENTIAL

Serial Number: 909413cc-045a-4ce7-a36e-9ddc32d52f79 accessed by Anika Pippin (PIPPINA) on Tue, 29 Nov 2022 00:21:08 GMT.

10.4. Extraction Procedure

1. Thaw human urine samples on wet ice. Once samples are thawed, either vortex the batch as a whole for ~5 minutes followed by vortexing each for 10 seconds, or vortex each sample for ~ 20 seconds each.
2. Pipet 50 mcL of human urine sample or blank to the corresponding wells of a 2-mL 96-well plate.
3. Add 50 mcL of working IS (500 mcg/mL cefotaxime in deionized water) to all applicable wells. For **Control Blanks** add 50 mcL of deionized water instead.
4. Seal the plate with and adhesive pad (or equivalent) and vortex at 1300 rpm for 3 minutes using a plate shaker.
5. Add 250 mcL of acetonitrile to all wells. Seal the plate with an adhesive pad (or equivalent) and vortex for 5 minutes at 1300 rpm.
6. Centrifuge the plate for 15 minutes at a setting of 4000 rpm (at ambient conditions).
7. Transfer 50 mcL of supernatant volume to a new 2-mL 96-well plate using a multichannel pipette (or equivalent).
8. Add 300 mcL of 0.1% formic acid in deionized water to all wells. Seal the plate with an adhesive pad (or equivalent).
9. Vortex the plate at 1200 rpm for 5 minutes.
10. Transfer 100 mcL of the mixture into a new 2-mL 96-well plate (final plate), using a multichannel pipette (or equivalent).
11. Add 300 mcL of 0.1% formic acid in deionized water to each well. Seal the plate with a cap mat.
12. Vortex the final plate at 1200 rpm for 5 minutes.
13. Refrigerate prior to LC-MS injection according to the conditions outlined in Section 7.

10.5. Dilutions

2x = 25 mcL sample + 25 mcL blank treated human urine

5x = 10 mcL sample + 40 mcL of blank treated human urine

CONFIDENTIAL

Serial Number: 909413cc-045a-4ce7-a36e-9ddc32d52f79 accessed by Anika Pippin (PIPPINA) on Tue, 29 Nov 2022 00:21:08 GMT.

10.6. Calculations

Linear regression is performed with a $1/x^2$ weighting factor.

Equation: Response = Slope * Conc + Intercept

11. REFERENCE CHROMATOGRAMS

Figure 1: Blank without IS

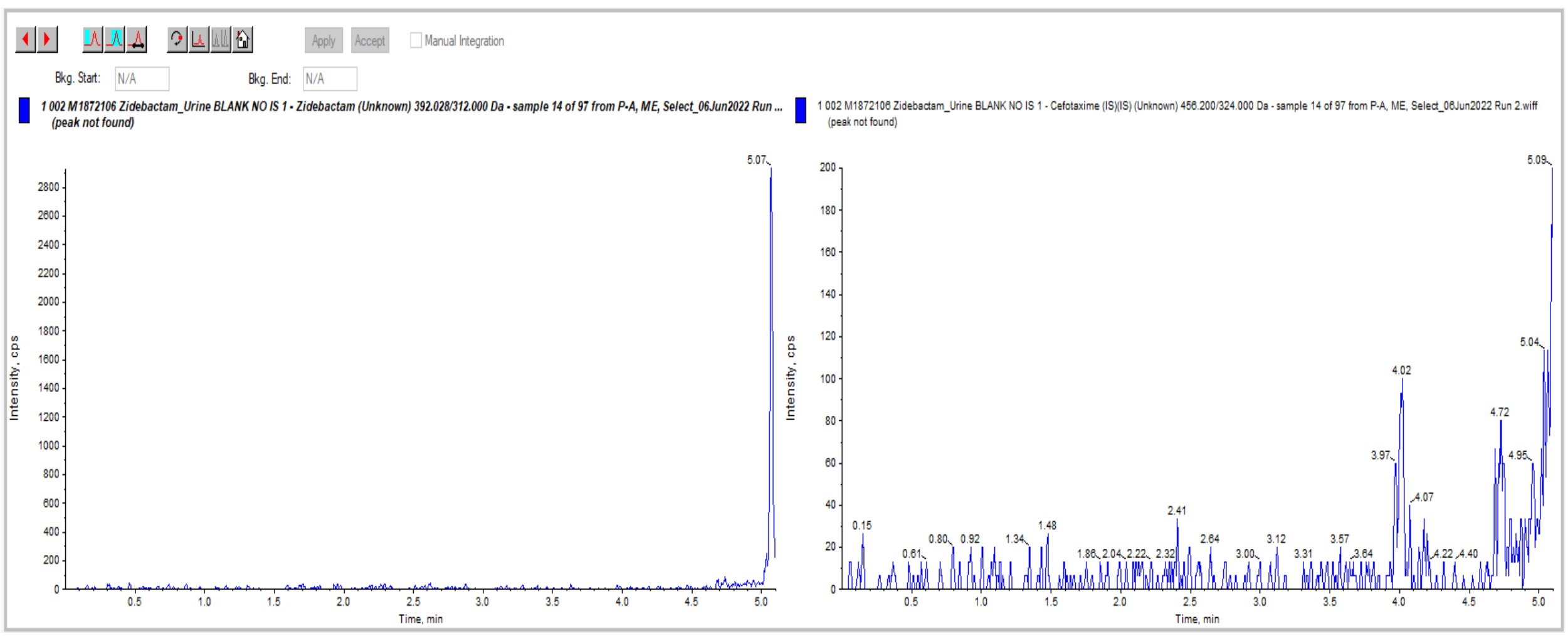

CONFIDENTIAL

Serial Number: 909413cc-045a-4ce7-a36e-9ddc32d52f79 accessed by Anika Pippin (PIPPINA) on Tue, 29 Nov 2022 00:21:08 GMT.

Figure 2: Blank with IS

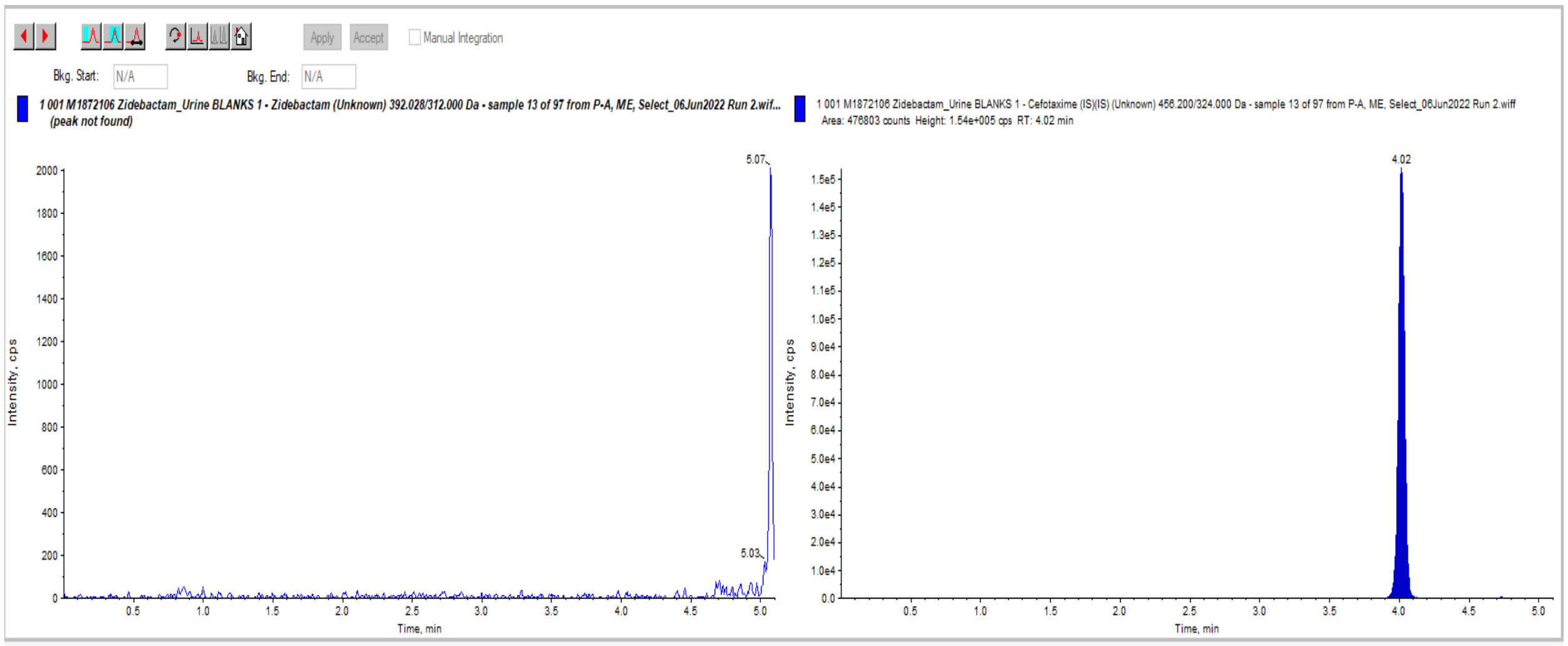

Figure 3: Carryover Blank with IS

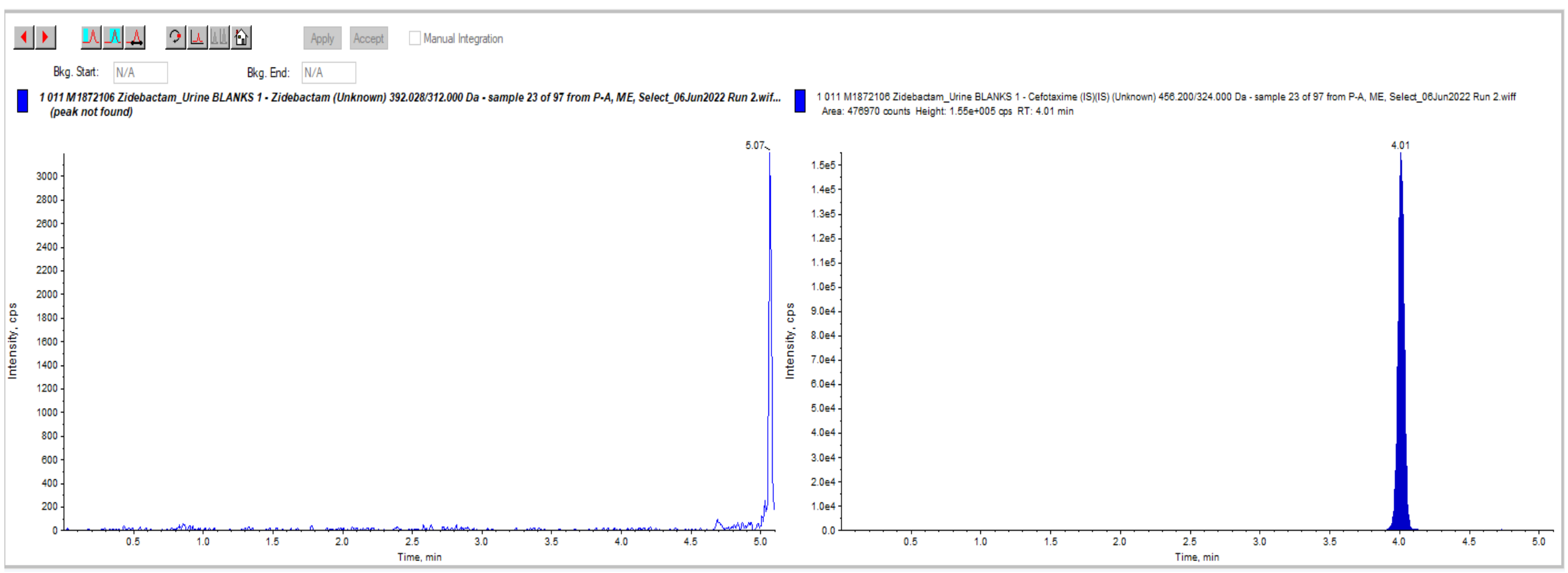

CONFIDENTIAL

Serial Number: 909413cc-045a-4ce7-a36e-9ddc32d52f79 accessed by Anika Pippin (PIPPINA) on Tue, 29 Nov 2022 00:21:08 GMT.

Figure 4: LLOQ – Zidebactam 10.0 mcg/mL

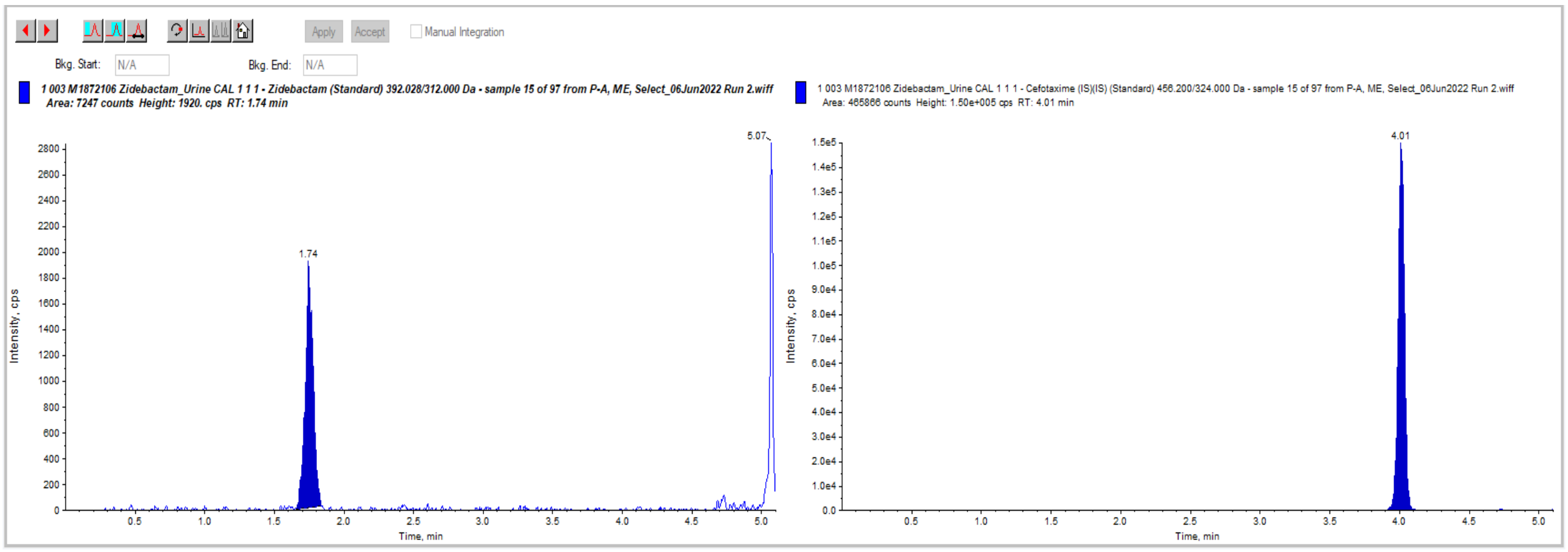

12. REVISION HISTORY

Table 3: Revision History

Revision	Effective Date	Section	Revision Summary
00	See Header	NA	New Procedure

CONFIDENTIAL

Serial Number: 909413cc-045a-4ce7-a36e-9ddc32d52f79 accessed by Anika Pippin (PIPPINA) on Tue, 29 Nov 2022 00:21:08 GMT.

TP-80187 [00]: V1872106U2 - Zidebactam in Treated Human Urine (50 mcL, 10.0 - 4000 mcg/mL)

All blank fields are considered as data not available.　　All Signature (Date and Time) shown in this report are in GMT.

**Type**	TP	**Status**	CURRENT	
**Effective Date**	28-Nov-2022	**Periodic Review Date**		
**Owner**	PARMENTIERB	**Department Name**	Pharma LC-MS/MS	Pharma LC-MS/MS
**Description**	Full Title: LC-MS/MS Analysis of Zidebactam in Treated Human Urine (50 mcL, 10.0 - 4000 mcg/mL in Urine only)			

Revision Notes (1)

Task Name	Action	Comment	Signature
Check In	Document Profile Preparation	Created outside of the system.	NEVINSC<br>23-Nov-2022 22:53:50 [GMT]

Serial Number: 909413cc-045a-4ce7-a36e-9ddc32d52f79 accessed by Anika Pippin (PIPPINA) on Tue, 29 Nov 2022 00:21:08 GMT.

Approval Information

Note to Approver

Reviewer: Bryan Parmentier, MLT (ASCP)
Managment: Marsha Luna, B.S.
PI: Anika Pippin, B.S.

Approval Decision	Comment	Signature
Approve		LUNAM<br>28-Nov-2022 1:23:38 [GMT]
Approve		PIPPINA<br>28-Nov-2022 16:28:04 [GMT]
Approve		PARMENTIERB<br>23-Nov-2022 23:07:13 [GMT]

Serial Number: 909413cc-045a-4ce7-a36e-9ddc32d52f79 accessed by Anika Pippin (PIPPINA) on Tue, 29 Nov 2022 00:21:08 GMT.

WOCKHARDT LIFE WINS

ANALYTICAL RESEARCH

CERTIFICATE OF ANALYSIS FOR REFERENCE STANDARD

**Name**	:	**Zidebactam (WCK 5107)**			
Molecular Formula	:	$C_{13}H_{21}N_5O_7S.2H_2O$	Batch Number	:	WK1/WP-0060/016
Molecular Weight	:	427.45	Laboratory Note Book Reference	:	WA-2004-038
Date of Analysis	:	30/03/2022	Month of Manufacturing	:	May' 2019
Valid Up To	:	29/03/2023	Reference Standard Number	:	RS/2022-013
Effective Date	:	23/04/2022	Page Number	:	Page 1 of 4

Sr. No.	Tests	Specifications	Results
1	Description	White to pale yellow powder	White powder
2	Identification (By IR, Mass & NMR Spectroscopy)	Spectral data should conform to the structure	Spectral data conforms the structure
3	Solubility	Freely soluble in water, Insoluble in dichloromethane	Freely soluble in water, Insoluble in dichloromethane
4	Constituted solution (10% w/v solution in WFI) Completeness of solution	The solid dissolves completely, leaving no visible residue as un-dissolved matter	Complies
	Clarity of solution	The constituted solution is not significantly less clear than an equal volume of the WFI contained in a similar vessel and examined similarly	Complies
5	pH of solution	Between 5.0 to 7.0	6.0
6	Absorbance of solution (1.25 % w/v in water at 420 nm)	Not more than 0.2 AU	0.01 AU
7	Water content (By KF Titration)	Not more than 12.0 % w/w	8.7 % w/w
8	Specific optical rotation (On anhydrous basis)	Between –28° to -40°	-36°
9	Heavy metals	Not more than 20 μg/g	Less than 20 μg/g
10	Palladium content (By ICP-OES)	Not more than 10 μg/g	BLQ (LOQ 2.5 μg/g)*

	Prepared by	Checked by	Approved by
Name :	Mr. Sitaram Ubale	Mr. Rajendra Chavan	Mr Subhash Chandra Shukla
Department :	AVL	AVL	CRDQ
Sign :	[signature]	[signature]	[signature]
Date :	12/04/2022	12/04/2022	13/04/2022

Format No. : AVL-045-F-02-00

Ranked # 1 In Healthcare Great Place to Work Best Companies to Work for 2015 India

ANALYTICAL RESEARCH

CERTIFICATE OF ANALYSIS FOR REFERENCE STANDARD

Name	:	**Zidebactam (WCK 5107)**			
Molecular Formula	:	$C_{13}H_{21}N_5O_7S.2H_2O$	Batch Number	:	WK1/WP-0060/016
Molecular Weight	:	427.45	Laboratory Note Book Reference	:	WA-2004-038
Date of Analysis	:	30/03/2022	Month of Manufacturing	:	May' 2019
Valid Up To	:	29/03/2023	Reference Standard Number	:	RS/2022-013
Effective Date	:	23/04/2022	Page Number	:	Page 2 of 4

Sr. No.	Tests	Specifications	Results
11	Related substances (By HPLC) :		
	i. Impurity -1	Not more than 2.00 % w/w	0.28 % w/w
	ii. Impurity -2	Not more than 1.00 % w/w	Not detected
	iii. Impurity -3	Not more than 1.00 % w/w	0.10 % w/w
	iv. Impurity -4	Not more than 0.10 % w/w	Not detected
	v. Specified impurity at RRT 2.16-2.28 (MW 782)	Not more than 0.50 % w/w	0.03 % w/w
	vi. Any unspecified impurity	Not more than 0.15 % w/w	BDL
	vii. Total impurities	Not more than 4.00 % w/w	0.41 % w/w
12	Residual solvents (By GC) :		
	Group A		
	i. Isopropyl alcohol	Not more than 5000 µg/g	Not detected
	ii. Dichloromethane	Not more than 600 µg/g	Not detected
	iii. Methanol	Not more than 3000 µg/g	1762 µg/g
	iv. Ethyl acetate	Not more than 5000 µg/g	Not detected
	v. Cyclohexane	Not more than 3880 µg/g	Not detected
	vi. Acetone	Not more than 5000 µg/g	Not detected
	vii. Methyl tertiary butyl ether	Not more than 5000 µg/g	Not detected
	viii. Toluene	Not more than 890 µg/g	Not detected
	ix. Ethanol	Not more than 5000 µg/g	2990 µg/g
	Group B		
	i. Triethylamine	Not more than 250 µg/g	Not detected
	ii. Pyridine	Not more than 200 µg/g	Not detected

	Prepared by	Checked by	Approved by
Name :	Mr. Sitaram Ubale	Mr. Rajendra Chavan	Mr Subhash Chandra Shukla
Department :	AVL	AVL	CRDQ
Sign :	[signature]	[signature]	[signature]
Date :	12/04/2022	12/04/2022	13/04/2022

Format No. : AVL-045-F-02-00

Ranked # 1 In Healthcare
Great Place to Work
Best Companies to Work for 2015 India

ANALYTICAL RESEARCH

CERTIFICATE OF ANALYSIS FOR REFERENCE STANDARD

**Name**	:	**Zidebactam (WCK 5107)**			
Molecular Formula	:	$C_{13}H_{21}N_5O_7S.2H_2O$	Batch Number	:	WK1/WP-0060/016
Molecular Weight	:	427.45	Laboratory Note Book Reference	:	WA-2004-038
Date of Analysis	:	30/03/2022	Month of Manufacturing	:	May' 2019
Valid Up To	:	29/03/2023	Reference Standard Number	:	RS/2022-013
Effective Date	:	23/04/2022	Page Number	:	Page 3 of 4

Sr. No.	Tests	Specifications	Results
13	Sodium content (By ICP-OES)	Not more than 0.5 % w/w	BLQ (LOQ 0.25 % w/w)*
14	Sulfate content (By Ion chromatography)	Not more than 1.0 % w/w	BLQ (LOQ 0.626 % w/w)
15	Mono-methyl sulphate content (By Ion chromatography)	Not more than 0.15% w/w	Not detected
16	Polymorphism (By X-ray Diffraction)	The diffractogram should exhibit characteristic 2 theta values at 13.8, 15.6, 20.3 and 24.3 (± 0.2°)	The diffractogram exhibits characteristic 2 theta values at 13.8, 15.6, 20.3 & 24.3
17	Potency (on as is basis)	Not applicable	90.4 %

BLQ = Below limit of quantification
BDL = Below disregard limit. Disregard limit 0.05 %
LOQ = Limit of quantification

* LOQ Values are based on analytical method verification report (Report nos. MVR/VP17053 and MVR/VP17054) of Analytical Solutions (CRO).

Specification Reference Number: **ZDB-DSS-112-05**

Remark: Potency is calculated on the basis of Total impurities, Water content, Residual solvents, Sulphate, Mono-methyl sulphate, Sodium and Palladium content.

Storage Condition: Store at 2°C to 8°C temperature.

	Prepared by	Checked by	Approved by
Name :	Mr. Sitaram Ubale	Mr. Rajendra Chavan	Mr Subhash Chandra Shukla
Department :	AVL	AVL	CRDQ
Sign :	[signature]	[signature]	[signature]
Date :	12/04/2022	12/04/2022	13/04/2022

Format No. : AVL-045-F-02-00

Ranked # 1 In Healthcare
GREAT PLACE TO WORK Best Companies to Work for 2015 India

ANALYTICAL RESEARCH

CERTIFICATE OF ANALYSIS FOR REFERENCE STANDARD

**Name**	**:**	**Zidebactam (WCK 5107)**			
Molecular Formula	:	$C_{13}H_{21}N_5O_7S.2H_2O$	Batch Number	:	WK1/WP-0060/016
Molecular Weight	:	427.45	Laboratory Note Book Reference	:	WA-2004-038
Date of Analysis	:	30/03/2022	Month of Manufacturing	:	May' 2019
Valid Up To	:	29/03/2023	Reference Standard Number	:	RS/2022-013
Effective Date	:	23/04/2022	Page Number	:	Page 4 of 4

Chemical Names:

i. **WCK 5107**: (2S,5R)-7-Oxo-6-sulphoxy-2-[N'-((R)-piperidin-3-carbonyl)-hydrazinocarbonyl]-1,6-diaza-bicyclo[3.2.1]octane dehydrate

ii. **Impurity-1**: (2S,5R)-5-sulphooxyamino-2-[N'-((R)-piperidin-3-carbonyl)-hydrazinocarbonyl]-piperidine

iii. **Impurity-2**: (2S,5R)-7-Oxo-6-hydroxy-2-[N'-((R)-piperidin-3-carbonyl)-hydrazinocarbonyl]-1,6-diaza-bicyclo[3.2.1]octane

iv. **Impurity-3**: (2S,5R)-7-Oxo-6-sulphooxy-2-[N'-((S)-piperidin-3-carbonyl)-hydrazinocarbonyl]-1,6-diaza-bicyclo[3.2.1]octane

v. **Impurity-4**: (2S,5R)-7-Oxo-6-sulphooxy-2-[N'-((R)-1-tert-butoxycarbonyl-piperidin-3-yl)-carbonyl)-hydrazinocarbonyl] -1,6-diaza-bicyclo[3.2.1]octane

	Prepared by	Checked by	Approved by
Name :	Mr. Sitaram Ubale	Mr. Rajendra Chavan	Mr Subhash Chandra Shukla
Department :	AVL	AVL	CRDQ
Sign :	[signature]	[signature]	[signature]
Date :	12/04/2022	12/04/2022	13/04/2022

Format No. : AVL-045-F-02-00

Ranked # 1 in Healthcare Best Companies to Work for 2015 India

3050 Spruce Street, Saint Louis, MO 63103, USA
Website: www.sigma-aldrich.com
Email USA: techserv@sial.com
Outside USA: eurtechserv@sial.com

Certificate of Analysis

**Product Name :**	**Cefotaxime sodium salt suitable for plant cell culture, BioReagent, powder**
**Product Number :**	**C7039-500MG**
**Batch Number :**	**0000146942**
Source Batch :	157206
CAS Number :	64485-93-4
Storage Temperature :	Cooler/Refrigerated
Molecular Formula :	$C_{16}H_{16}N_5NaO_7S_2$
Formula Weight :	477.45
Recommended Retest Date :	Sep 2023
Quality Release Date :	02 Jan 2022

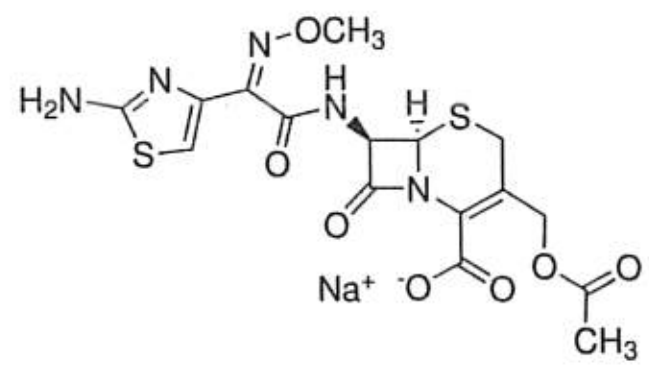

Test	Specification	Result
Appearance (Form)	Powder or Crystals	Powder
Appearance (Color)<br>Off-white to pale yellow	Conforms	Conforms
Solubility<br>10% in water	Clear	Clear
Infrared Spectrum	Conforms to Structure	Conforms
Total Impurity	≤ 3.0 %	1.7 %
Specific Rotation<br>[a]20/D	58 - 64 °	61 °
Assay (Dry Basis)	916 - 964 µg/mg	955 µg/mg
Loss on Drying	≤ 3.0 %	2.4 %
Plant Cell Culture Test	Pass	Pass

Anna Bendersky, Quality Control Manager
Quallity Control
Israel
IL

Sigma-Aldrich warrants, that at the time of the quality release or subsequent retest date this product conformed to the information contained in this publication. The current Specification sheet may be available at Sigma-Aldrich.com. For further inquiries, please contact Technical Service. Purchase must determine the suitability of the product for its particular use. See reverse side of website or packing slip for additional terms and conditions of sale

The branding on the header and/or footer of this document may temporarily not visually match the product purchased as we transition our branding. However, all of the information in the document regarding the product remains unchanged and matches the product ordered.
For further information please contact mlsbranding@sial.com

Record of Decision – Interference Testing
Study 21-0013: Zidebactam

Memo Date: November 10, 2022

RE: Interference Testing of Clinically Authorized Medications for Study 21-0013 (Zidebactam)

For this clinical study KCAS is developing several analytical LC-MS/MS assays for the detection and quantitation of zidebactam in plasma and urine and falls under the Bioanalytical Method Validation, FDA Guidance for Industry (May 2018) which states:

> *Potential interfering substances in a biological matrix include endogenous matrix components such as metabolites, decomposition products – and from the actual study – concomitant medications and other xenobiotics. If a stabilizer or enzyme inhibitor is used during sample collection, the sponsor should evaluate the potential for interference on the quantitation of the analyte. Sponsors should make a scientific judgment about the need to assess these (and any other) potential interferences during method development.*

The clinical trial has stringent screening and exclusion criteria but allows all volunteers to use acetaminophen (paracetamol) for temporary pain and headaches and allows women to continue using commercially available birth control.

An industry paper quoted de Zwart. M Lausecker *et al*, Co-medication and interference testing in bioanalysis: a European Bioanalysis Forum recommendation *Bioanalysis* 8(19), 2065-2070 (2016) contains a decision flow chart (Figure 1) that based on whether co-medication is scheduled or unscheduled and potential interference based on physical properties. The Figure 1 chart indicates that:

1. These inferring substances may not be considered scheduled co-medication because definition within the paper of "drugs which dose, time and route of administration are defined in the clinical study protocol", however the CDC estimates that ~ 25% of women aged, age 18-49 years old use hormone containing contraceptive, so it is prudent to expect ~12.5% of clinical samples to contain one or more of the contraceptives listed below when adjusting for study enrollment of both genders.
2. Therefore, following the "Scheduled co-medication" decision tree the interference testing is not required because the significant differences in the molecular weights of the test analyte and the ConMeds (see table below).

Zidebactam MASS (MW) vs CONMEDS (source: www.ChemSpider.com)

DRUG/Analyte	Formula	Avg. Mass	Monoisotopic Mass
Zidebactam	C13H21N5O7S	391.400 Da	391.116180 Da
Ertapenem	C22H25N3O7S	475.55 Da	475.142327 Da
Urea	CH4N20	60.0553 Da	60.055 Da
Ethinylestradiol	C20H24O2	296.403 Da	296.177643 Da
Levonorgestrel	C21H28O2	312.446 Da	312.208923 Da
Etonogestrel	C22H28O2	324.457 Da	324.208923 Da
Norethindrone	C20H26O2	298.419 Da	298.193268 Da
Drospirenone	C24H30O3	366.493 Da	366.219482 Da

These items are controlled by the U.S. Government and authorized for export only to the country of ultimate destination for use by the ultimate consignee or end-user(s) herein identified. They may not be resold, transferred, or otherwise disposed of, to any other country or to any person other than the authorized ultimate consignee or end-user(s), either in their original form or after being incorporated into other items, without first obtaining approval from the U.S. Government or as otherwise authorized by U.S. law and regulations.

Paracetamol	C8H9NO2	151.163 Da	151.063324 Da

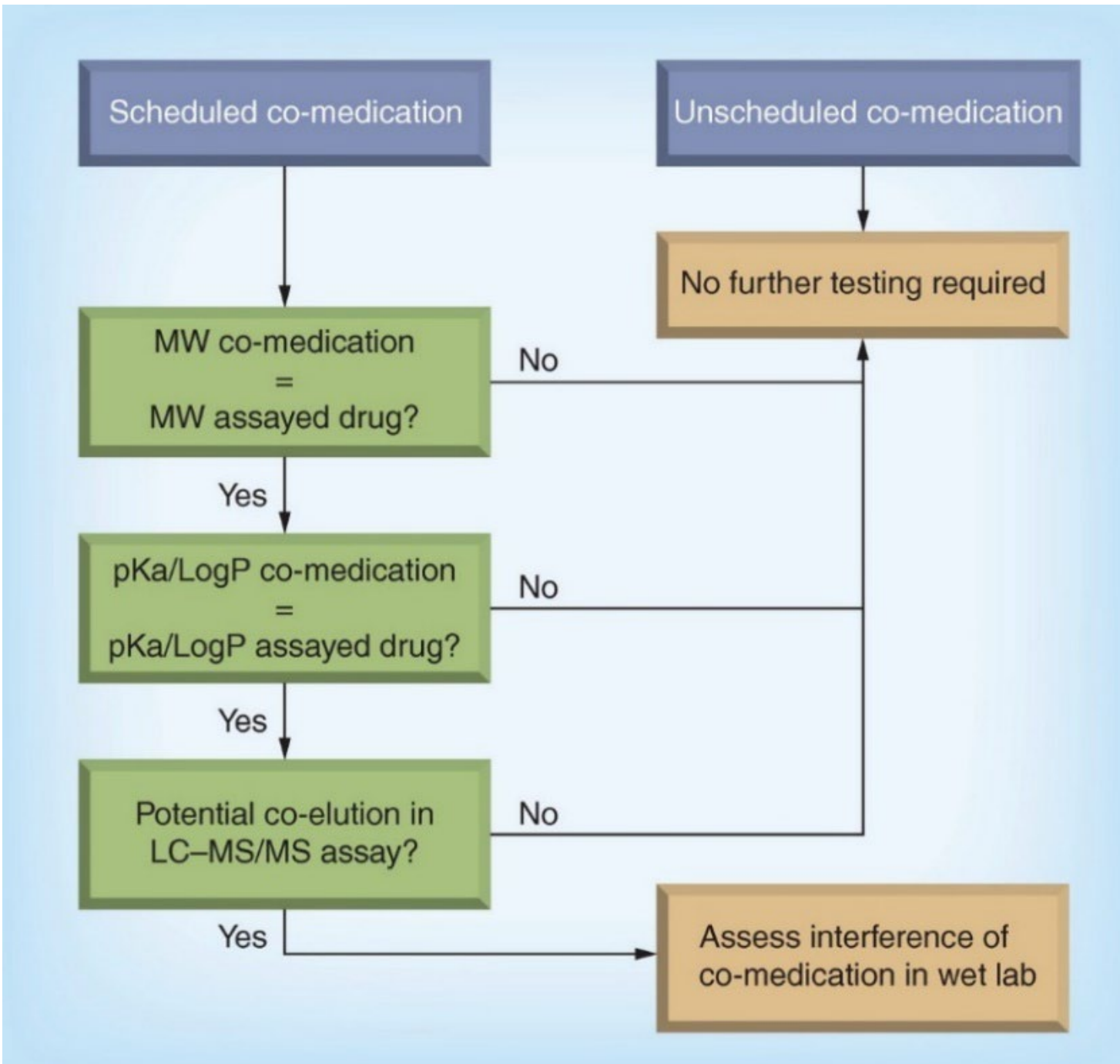

Figure 1. Recommendation for scheduled and unscheduled co-medication testing for LC–MS/MS-based assays.

Therefore, based on both sides of the decision tree in Figure 1, further interference testing other than the evaluation of the planned co-administered drug, ertapenem, is not required.

Decision issued by:

____

Matthew E. Hinz, M.S. Sr. Manager
DVC LLC, A GDIT Company

These items are controlled by the U.S. Government and authorized for export only to the country of ultimate destination for use by the ultimate consignee or end-user(s) herein identified. They may not be resold, transferred, or otherwise disposed of, to any other country or to any person other than the authorized ultimate consignee or end-user(s), either in their original form or after being incorporated into other items, without first obtaining approval from the U.S. Government or as otherwise authorized by U.S. law and regulations.
