# Supplementary material for: A Phase I, randomized, double-blind, placebo-controlled, dose escalation study to evaluate the safety, tolerability, and pharmacokinetics of intravenous ertapenem and zidebactam in combination (WCK 6777) in healthy adult subjects
Source: Antimicrob Agents Chemother. 2026 Sep 29;70(11):e00390-26. doi: 10.1128/aac.00390-26 (PMC13731237; doi:10.1128/aac.00390-26)
Supplement: Supplementary file 7 [file aac.00390-26-s0007.pdf]

Supplemental Appendix Legends

Supplemental Appendix A. Bioanalytical Method Validation Report for the Determination of Total Ertapenem in Treated Human Plasma, with Sodium Heparin as Anticoagulant, by LC-MS/MS.

Supplemental Appendix B. Bioanalytical Method Validation Report for the Determination of Free Ertapenem in Treated Human Plasma Ultrafiltrate, with Sodium Heparin as Anticoagulant, by LC-MS/MS.

Supplemental Appendix C. Bioanalytical Method Validation Report for the Determination of Total Zidebactam in Treated Human Plasma, with Sodium Heparin as Anticoagulant, by LC-MS/MS.

Supplemental Appendix D. Bioanalytical Method Validation Report for the Determination of Free Zidebactam in Treated Human Plasma Ultrafiltrate, with Sodium Heparin as Anticoagulant, by LC-MS/MS.

Supplemental Appendix E. Bioanalytical Method Validation Report for the Determination of Ertapenem in Treated Human Urine by LC-MS/MS.

Supplemental Appendix F. Bioanalytical Method Validation Report for the Determination of Zidebactam in Treated Human Urine by LC-MS/MS.
