# Supplementary material for: A Phase I, randomized, double-blind, placebo-controlled, dose escalation study to evaluate the safety, tolerability, and pharmacokinetics of intravenous ertapenem and zidebactam in combination (WCK 6777) in healthy adult subjects
Source: Antimicrob Agents Chemother. 2026 Sep 29;70(11):e00390-26. doi: 10.1128/aac.00390-26 (PMC13731237; doi:10.1128/aac.00390-26)
Supplement: Supplementary file 8 [file aac.00390-26-s0008.docx]

**Supplemental Table 1.** Summary of Participants with Pre-Existing Medical Conditions by MedDRA System Organ Class and Treatment Group

| **MedDRA System Organ Class** | **WCK 6777 2 g (N=6)** | **ERT 2 g (N=6)** | **ZID 2 g (N=6)** | **WCK 6777 4 g (N=6)** | **ERT 3 g (N=6)** | **ZID 3 g (N=6)** | **WCK 6777 6 g (N=6)** | **Placebo (N=10)** | **All Participants (N=52)** |
| --- | --- | --- | --- | --- | --- | --- | --- | --- | --- |
| **Any SOC** | 3 (50) | 3 (50) | 3 (50) | 5 (83) | 1 (17) | 4 (67) | 4 (67) | 4 (40) | 27 (52) |
| Blood and lymphatic system disorders | 1 (17) | 0 | 0 | 0 | 0 | 0 | 2 (33) | 0 | 3 (6) |
| Cardiac disorders | 0 | 0 | 0 | 0 | 0 | 0 | 1 (17) | 0 | 1 (2) |
| Congenital, familial and genetic disorders | 0 | 0 | 0 | 1 (17) | 0 | 0 | 0 | 0 | 1 (2) |
| Gastrointestinal disorders | 0 | 0 | 2 (33) | 0 | 0 | 1 (17) | 0 | 1 (10) | 4 (8) |
| Infections and infestations | 0 | 2 (33) | 1 (17) | 1 (17) | 0 | 3 (50) | 1 (17) | 0 | 8 (15) |
| Injury, poisoning and procedural complications | 1 (17) | 1 (17) | 0 | 0 | 0 | 1 (17) | 0 | 1 (10) | 4 (8) |
| Investigations | 0 | 0 | 0 | 0 | 0 | 1 (17) | 0 | 0 | 1 (2) |
| Nervous system disorders | 0 | 0 | 0 | 1 (17) | 0 | 1 (17) | 0 | 0 | 2 (4) |
| Renal and urinary disorders | 0 | 1 (17) | 0 | 0 | 0 | 0 | 0 | 0 | 1 (2) |
| Reproductive system and breast disorders | 1 (17) | 0 | 1 (17) | 2 (33) | 1 (17) | 0 | 2 (33) | 2 (20) | 9 (17) |
| Respiratory, thoracic and mediastinal disorders | 1 (17) | 1 (17) | 0 | 0 | 0 | 1 (17) | 0 | 1 (10) | 4 (8) |
| Skin and subcutaneous tissue disorders | 0 | 0 | 1 (17) | 1 (17) | 0 | 1 (17) | 0 | 0 | 3 (6) |
| Surgical and medical procedures | 1 (17) | 3 (50) | 1 (17) | 2 (33) | 0 | 3 (50) | 1 (17) | 3 (30) | 14 (27) |

Notes: N = number of participants in the Safety Population. n = number of participants reporting medical history within the specified MedDRA System Organ Class. Each participant is counted once per SOC, regardless of the number of reported conditions.

**Supplemental Table 2.** Adverse Events Occurring in 5% of Participants in Any Treatment Groups by MedDRA System Organ Class and Preferred Term, and Treatment Group - Safety Population

| **MedDRA System Organ Class / Preferred Term** | **WCK 6777 2 g (N=6)** | **ERT 2 g (N=6)** | **ZID 2 g (N=6)** | **WCK 6777 4 g (N=6)** | **ERT 3 g (N=6)** | **ZID 3 g (N=6)** | **WCK 6777 6 g (N=6)** | **Placebo (N=10)** | **All Participants (N=52)** |
| --- | --- | --- | --- | --- | --- | --- | --- | --- | --- |
| **Any SOC (any PT)** | 6 (100) [14] | 6 (100) [17] | 5 (83) [15] | 5 (83) [9] | 4 (67) [10] | 2 (33) [4] | 4 (67) [9] | 9 (90) [22] | 41 (79) [100] |
| **Blood and lymphatic system disorders** | 0 | 1 (17) [1] | 1 (17) [1] | 0 | 0 | 0 | 0 | 1 (10) [1] | 4 (8) [4] |
| Anaemia | 0 | 1 (17) [1] | 1 (17) [1] | 0 | 0 | 0 | 0 | 1 (10) [1] | 3 (6) [3] |
| Leukopenia | 0 | 0 | 0 | 0 | 0 | 0 | 1 (17) [1] | 0 | 1 (17) [1] |
| **Gastrointestinal disorders** | 2 (33) [3] | 2 (33) [2] | 0 | 1 (17) [1] | 1 (17) [1] | 0 | 1 (17) [1] | 1 (10) [1] | 8 (15) [9] |
| Diarrhoea | 0 | 2 (33) [2] | 0 | 1 (17) [1] | 0 | 0 | 1 (17) [1] | 0 | 4 (8) [4] |
| Nausea | 2 (33) [2] | 0 | 0 | 0 | 1 (17) [1] | 0 | 0 | 1 (10) [1] | 4 (8) [4] |
| **General disorders and administration-site conditions** | 3 (50) [6] | 5 (83) [11] | 5 (83) [8] | 4 (67) [6] | 4 (67) [6] | 2 (33) [3] | 3 (50) [6] | 6 (60) [14] | 32 (62) [60] |
| Infusion-site erythema | 0 | 2 (33) [5] | 3 (50) [3] | 1 (17) [1] | 1 (17) [1] | 1 (17) [1] | 2 (33) [2] | 1 (10) [1] | 11 (21) [14] |
| Infusion-site extravasation | 1 (17) [1] | 0 | 0 | 2 (33) [2] | 2 (33) [2] | 0 | 1 (17) [1] | 3 (30) [4] | 9 (17) [10] |
| Infusion-site haemorrhage | 0 | 0 | 0 | 1 (17) [1] | 0 | 0 | 0 | 3 (30) [5] | 4 (8) [6] |
| Infusion-site pain | 1 (17) [1] | 2 (33) [4] | 1 (17) [1] | 0 | 0 | 0 | 0 | 0 | 4 (8) [6] |
| Infusion-site phlebitis | 0 | 1 (17) [1] | 0 | 1 (17) [1] | 0 | 0 | 0 | 1 (10) [1] | 3 (6) [3] |
| Infusion-site swelling | 0 | 1 (17) [1] | 1 (17) [1] | 0 | 0 | 0 | 1 (17) [1] | 2 (20) [2] | 5 (10) [5] |
| Injection-site haemorrhage | 0 | 0 | 0 | 0 | 1 (17) [1] | 0 | 2 (33) [2] | 0 | 3 (6) [3] |
| **Investigations** | 1 (17) [1] | 0 | 1 (17) [1] | 1 (17) [1] | 1 (17) [2] | 0 | 1 (17) [1] | 3 (30) [3] | 8 (15) [9] |
| ECG QT prolonged | 0 | 0 | 0 | 1 (17) [1] | 1 (17) [1] | 0 | 0 | 0 | 2 (4) [2] |
| Heart rate decreased | 1 (17) [1] | 0 | 0 | 0 | 1 (17) [1] | 0 | 1 (17) [1] | 0 | 3 (6) [3] |
| **Nervous system disorders** | 1 (17) [1] | 1 (17) [1] | 1 (17) [1] | 0 | 1 (17) [1] | 0 | 0 | 2 (20) [2] | 6 (12) [6] |
| Headache | 1 (17) [1] | 1 (17) [1] | 1 (17) [1] | 0 | 1 (17) [1] | 0 | 0 | 2 (20) [2] | 6 (12) [6] |
| **All other SOCs†** | ≤1 (≤17) | ≤1 (≤17) | ≤1 (≤17) | ≤1 (≤17) | ≤1 (≤17) | ≤1 (≤17) | ≤1 (≤17) | ≤2 (≤20) | ≤2 (≤4) |

Values are n (%) [number of events]

†Includes blood and lymphatic system disorders, gastrointestinal disorders, general disorders, infections and infestations, injury/procedural complications, investigations, musculoskeletal, renal and urinary, respiratory, and skin disorders, each reported in ≤2 participants overall.

**Supplemental Table 3.** Moderate Adverse Events (Safety Population)

| **Treatment Group** | **Adverse Event (Verbatim)** | **Associated with Dose Number** | **No. of Days Post Associated Dose (Duration, days)** | **Severity** | **Relationship to Study Treatment** | **If Not Related, Alternative Etiology** | **Action Taken with Study Treatment** | **Participant Discontinued Due to AE** | **Outcome** | **MedDRA System Organ Class** | **MedDRA High Level Group Term** | **MedDRA Preferred Term** |
| --- | --- | --- | --- | --- | --- | --- | --- | --- | --- | --- | --- | --- |
| WCK 6777 2 g | Decreased heart rate | 1 | 0 (5) | Moderate | Related | — | Dose not changed | N | Recovered/Resolved | Investigations | Cardiac and vascular investigations (excl enzyme tests) | Heart rate decreased |
| ERT 2 g | Diarrhea | 2 | 1 (5) | Moderate | Related | — | Dose not changed | N | Recovered/Resolved | Gastrointestinal disorders | Gastrointestinal motility and defaecation conditions | Diarrhoea |
| ERT 2 g | Infusion site swelling | 2 | 0 (3) | Moderate | Related | — | Dose not changed | N | Recovered/Resolved | General disorders and administration site conditions | Administration site reactions | Infusion site swelling |
| WCK 6777 4 g | QTcF interval prolongation | 7 | 4 (Ongoing) | Moderate | Related | — | Not applicable | N | Not Recovered/Not Resolved (lost to follow-up; certified letter sent 28Aug2023; no return) | Investigations | Cardiac and vascular investigations (excl enzyme tests) | Electrocardiogram QT prolonged |
| ERT 3 g | Venipuncture arm pain left antecubital | 4 | 0 (1) | Moderate | Not related | Study procedure: venipuncture | Dose not changed | N | Recovered/Resolved | General disorders and administration site conditions | Administration site reactions | Vessel puncture site pain |
| ERT 3 g | QTcF interval prolonged | 7 | 1 (1) | Moderate | Related | — | Not applicable | N | Recovered/Resolved | Investigations | Cardiac and vascular investigations (excl enzyme tests) | Electrocardiogram QT prolonged |
| Placebo | Headache | 1 | 1(1) | Moderatre | Related | — | Dose not change | N | Recovered/Resolved | Nervous system disorders | Headaches | Headache |
